# *MAPKAPK2*, a potential dynamic network biomarker of α-synuclein prior to its aggregation in PD patients

**DOI:** 10.1038/s41531-023-00479-z

**Published:** 2023-03-16

**Authors:** Zhenggang Zhong, Jiabao Li, Jiayuan Zhong, Yilin Huang, Jiaqi Hu, Piao Zhang, Baowen Zhang, Yabin Jin, Wei Luo, Rui Liu, Yuhu Zhang, Fei Ling

**Affiliations:** 1 Guangdong Key Laboratory of Fermentation and Enzyme Engineering, School of Biology and Biological Engineering, South China University of Technology, Guangzhou, Guangdong China; 2 School of Mathematics, South China University of Technology, Guangzhou, Guangdong China; 3 Department of Neurology, Guangdong Neuroscience Institute, Guangdong Provincial People’s Hospital, Guangdong Academy of Medical Sciences, Guangzhou, China; 4 The First People’s Hospital of Foshan, Sun Yat-sen University, Foshan, China

**Keywords:** Parkinson's disease, Sequencing, Computational biology and bioinformatics

## Abstract

One of the important pathological features of Parkinson’s disease (PD) is the pathological aggregation of α-synuclein (α-Syn) in the substantia nigra. Preventing the aggregation of α-Syn has become a potential strategy for treating PD. However, the molecular mechanism of α-Syn aggregation is unclear. In this study, using the dynamic network biomarker (DNB) method, we first identified the critical time point when α-Syn undergoes pathological aggregation based on a SH-SY5Y cell model and found that DNB genes encode transcription factors that regulated target genes that were differentially expressed. Interestingly, we found that these DNB genes and their neighbouring genes were significantly enriched in the cellular senescence pathway and thus proposed that the DNB genes *HSF1* and *MAPKAPK2* regulate the expression of the neighbouring gene *SERPINE1*. Notably, in Gene Expression Omnibus (GEO) data obtained from substantia nigra, prefrontal cortex and peripheral blood samples, the expression level of *MAPKAPK2* was significantly higher in PD patients than in healthy people, suggesting that *MAPKAPK2* has potential as an early diagnostic biomarker of diseases related to pathological aggregation of α-Syn, such as PD. These findings provide new insights into the mechanisms underlying the pathological aggregation of α-Syn.

## Introduction

Parkinson’s disease is the second most common neurodegenerative disease after Alzheimer’s disease and is characterized by a high prevalence and disability rate. The clinical symptoms of Parkinson’s disease include motor symptoms such as gait disorders and nonmotor symptoms such as cognitive disorders^1,2^. Therefore, PD patients have difficulty living independently, which places a heavy burden on patients and their families. PD is usually accompanied by neurodegenerative pathological changes before clinical symptoms appear^3^. The early diagnosis and clinical management of PD is difficult, as the majority of neurons in a patient’s brain die sequentially before clinical features become apparent. The important pathological features of PD are the progressive loss of dopaminergic neurons in the substantia nigra and pathological aggregation of α-synuclein, which is the main component of Lewy bodies^4^. Although the aetiology of PD is not well understood, the pathological aggregation of α-Syn is known to be an important step in the pathogenesis of PD.

α-Syn is encoded by *SNCA* in presynaptic terminals and plays a role in regulating neurotransmitter release, synaptic function and plasticity^5^. Recent research has suggested that physiological α-Syn is a helical tetramer that resists aggregation. In this stage, α-Syn does not induce neurotoxicity^6^. Excessive accumulation of α-Syn, such as that caused by stimulation with inducing drugs such as rotenone or MPTP, can lead to pathological α-Syn aggregation^7,8^. Under pathological conditions, α-Syn is converted from a tetramer to a monomer, and monomeric α-Syn readily aggregates and transforms into misfolded β-sheet oligomers, which is indicative of pathological aggregation^5^. Pathologically aggregated α-Syn is usually phosphorylated at serine 129^9^. Pathologically aggregated α-Syn induces neurotoxicity and inhibits ubiquitin‒proteasome system activity and blocks the autophagic lysosomal pathway, two important mechanisms for the repair or removal of abnormal proteins in cells^10,11^. Once pathological aggregation of α-Syn is formed, the ubiquitin-proteasome system and the autophagic lysosomal pathway are inhibited, leading to difficulties in clearing the abnormal protein, which in turn leads to difficulties in degrading the pathological aggregation of α-Syn. Pathological oligomeric α-Syn accumulated in large quantities forms α-Syn fibrils and Lewy bodies, which induce neurotoxicity, similar to α-Syn oligomers, leading to mitochondrial abnormalities, abnormal endoplasmic reticulum–Golgi trafficking and inhibition of the autophagy–lysosomal pathway, causing the death of dopaminergic neurons and manifesting as Parkinson’s disease^5^. Pathological aggregation of α-Syn is therefore an important step in the pathogenesis of PD. Prevention of pathological aggregation of α-Syn has become a potential strategy for the mitigation and prevention of PD^12,13^. Levin et al. found that the oligomeric modulator anle138b inhibited α-Syn oligomer formation in vitro, and anle138b treatment slowed the progression of PD in an A30P α-Syn transgenic mouse model^14^. Therefore, to provide clues for PD intervention and diagnosis, we searched for key genes affecting the pathological aggregation of α-Syn and biomarkers for the early diagnosis of diseases associated with the pathological aggregation of α-Syn.

The DNB method is an approach used for mathematically modelling gene expression networks on the basis of a temporally expressed sequence that can identify biomarkers for the early detection of the prepathological α-Syn aggregation^15,16^. In PD patients, the formation of pathologically aggregated α-Syn impairs the function of the ubiquitin‒proteasome system and the autophagy–lysosomal pathway, resulting in a reduced rate of pathologically aggregated α-Syn degradation^10,11^. Several studies have suggested that pathological α-Syn aggregates propagate between cells, thereby further promoting α-Syn aggregation in other neurons in a ‘prion-like’ manner^17–20^. These aforementioned studies illustrated that the transition from a normal to a pathologically aggregated state of α-Syn is a drastic change that is difficult to reverse. To quantify this process, we applied the DNB method to predict the critical point before pathological aggregation of α-Syn. The DNB method is based on the theory that disease progresses through three states, namely, the normal state, the predisease state and the disease state. The predisease state is an unstable critical state in which the normal state is changing into the disease state. At this time, gene expression levels and gene network structures change dramatically. DNB genes are at the core of these gene networks. The DNB method has been used in studies in several fields for research into, for example, colorectal cancer metastasis, the epithelial–mesenchymal transformation and breast cancer^21–23^. Compared to traditional molecular biomarkers that are used to detect disease states on the basis of their differential molecular levels measured at a single time point, the DNB method integrates temporal information, and this case was chosen for its superiority in identifying the critical time before prepathological aggregation of the α-Syn state (the tipping point just before the dramatic transition from the physiological tetramer state to the pathological state of α-Syn aggregation). The DNB method revealed the key genes with changed expression before pathological aggregation of α-Syn and biomarkers useful for early diagnosis of diseases associated with the pathological aggregation of α-Syn, contributing to the study of the mechanisms underlying the pathological aggregation of α-Syn.

In this study, to identify the key genes affecting the pathological aggregation of α-Syn, we constructed a cell model of α-Syn pathological aggregation and used the DNB method to predict the critical time point immediately before α-Syn undergoes pathological aggregation. Combining multiple biochemical analysis methods based on dynamic changes in key gene expression levels, regulatory networks and functional enrichment of DNB genes, we investigated the effect of DNB gene expression on the pathological aggregation of α-Syn. Combining our experimental results with clinical data, we found that the *MAPKAPK2* gene in peripheral blood is a potential biomarker for early diagnosis of PD because its expressed was changed immediately before pathological aggregation of α-Syn. Finally, we found that the DNB genes *HSF1* and *MAPKAPK2* regulate the expression of their neighbouring gene *SERPINE1*; all three of these genes were thus identified as possible key genes with changed expression before pathological aggregation of α-Syn, and we propose a molecular mechanism that possibly explains this outcome.

## Results

### Construction of a cell model of pathological α-Syn aggregation

**Fig. 1:**
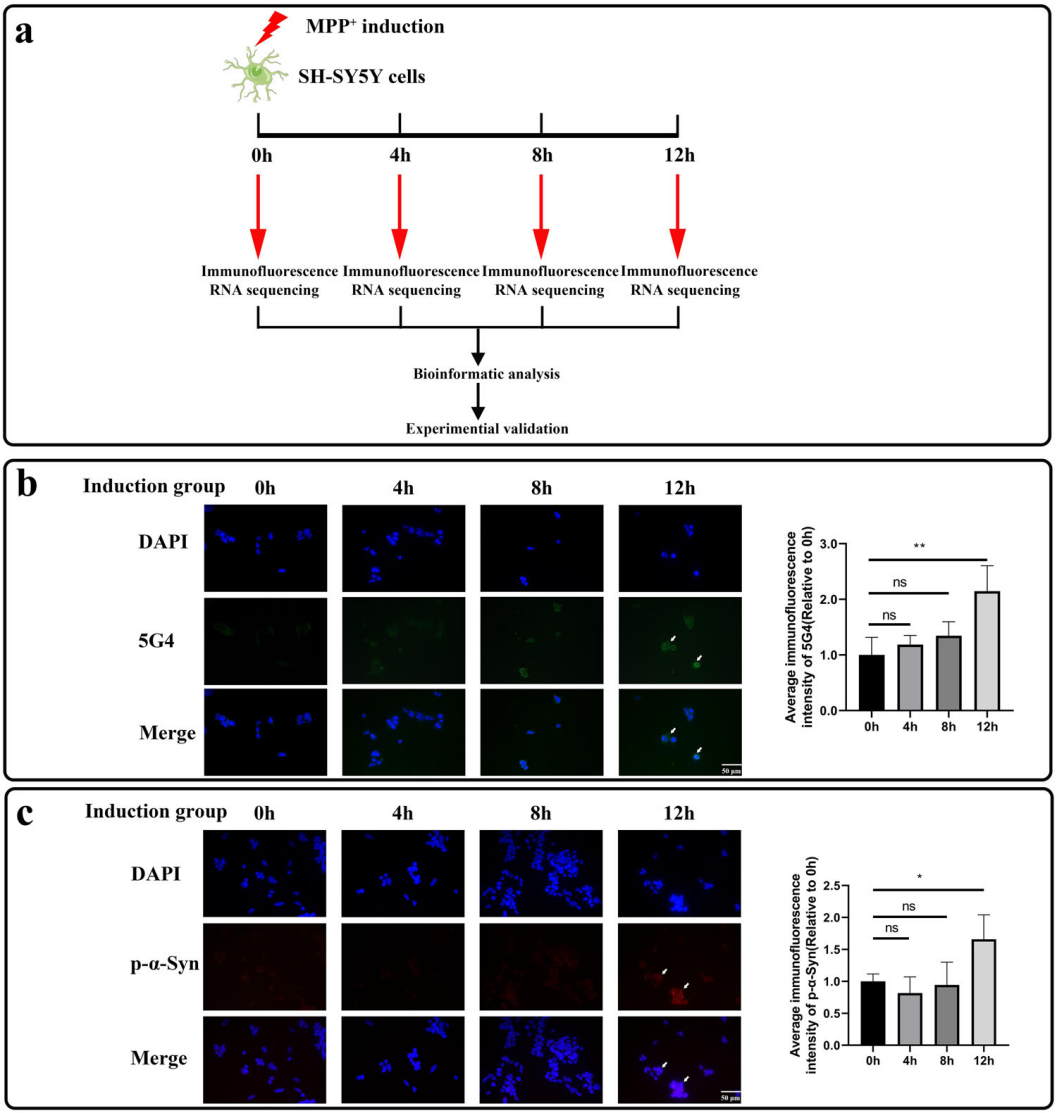
Construction of a cell model of pathological α-Syn aggregation. **a** Overview of the experimental design in this study. **b**, **c** Results of immunofluorescence staining for the 5G4 antibody (**b**) and anti-p-α-Syn antibody (**c**) in the induction group. Blue fluorescence represents 4′,6-diamidino-2-phenylindole (DAPI)-stained nuclei, green fluorescence represents pathological aggregation of α-syn, and red fluorescence represents p-α-Syn. The average immunofluorescence intensities of these two antibodies were normalized using the average immunofluorescence intensities of DAPI. Immunofluorescence experiments showed that the α-Syn pathological aggregation cell model was successfully constructed, while α-Syn pathological aggregation appeared 12 h after induction. *n* = 4. ns: no significant difference. *p < 0.05. **p < 0.01. The data are expressed as the means ± SEMs. Figure 1a was partly generated by adapting Servier Medical Art pictures provided by Servier, licensed under a Creative Commons Attribution 3.0 unported license.

To investigate the pathological aggregation of α-Syn, we constructed a cell model of pathological aggregation of α-Syn using MPP^+^ induction while setting up a control group for comparison. To verify that the α-Syn pathological aggregation cell model had been successfully constructed, immunofluorescence staining of cells 0 h, 4 h, 8 h and 12 h after induction was performed using both a 5G4 antibody and an anti-p-α-Syn antibody (Fig. 1a).

In immunofluorescence experiments with the 5G4 antibody, the relative mean immunofluorescence intensities 4 h and 8 h after induction were not significantly different from those 0 h after induction, while the relative mean immunofluorescence intensity 12 h after induction was significantly higher than that 0 h after induction (Fig. 1b). The relative mean immunofluorescence intensities at each time point in the control group were not significantly different. Comparisons performed at the same time points revealed that only the difference found at 12 h in the induction group and 12 h in the control group was significant (Supplementary Fig. 2a, b). Similar results were observed in the experiments with the p-α-syn antibody (Fig. 1c, Supplementary Fig. 2c, d). We therefore concluded that the cell model of pathological aggregation of α-Syn had been successfully constructed, and the appearance of pathological aggregation of α-Syn was observed 12 h after induction.

### DNB genes are transcription factors that regulate expression of target genes that were differentially expressed

To determine the critical time points before pathological aggregation of α-Syn, we collected samples 0 h, 4 h, 8 h and 12 h after induction and performed transcriptome sequencing. The four time points were chosen to cover the entire process from the cellular transition between the normal state to the pathological α-Syn aggregation state.

**Fig. 2:**
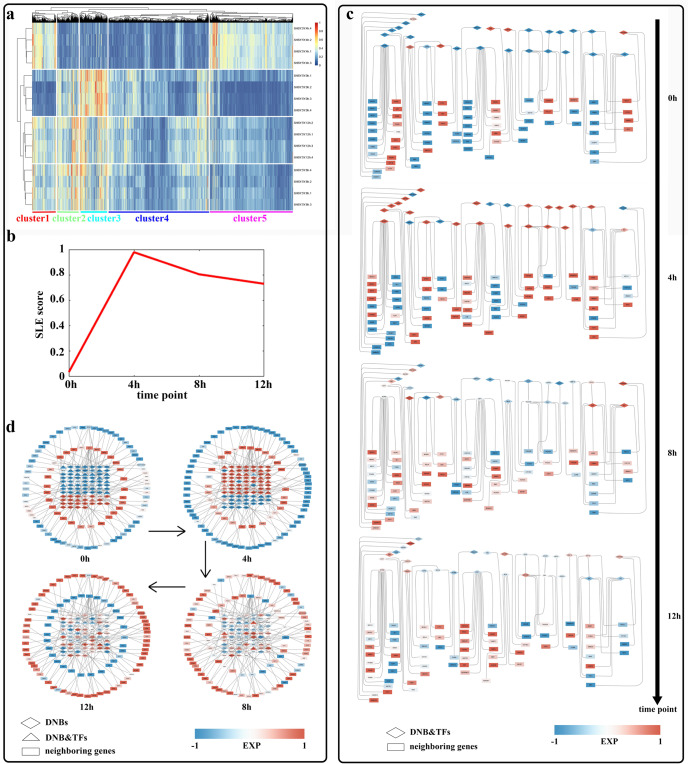
Detection of the critical time point before pathological aggregation of α-Syn and analysis of DNB genes. **a** Heatmap showing DEG gene expression profiles. Hierarchical clustering showed that the gene expression profile 4 h after induction differed from that at other time points. **b** DNB analysis showed that the single-sample network entropy peaked 4 h after induction. **c** Regulatory networks revealed that DNB-related transcription factors regulated neighbouring gene expression. The diamonds represent DNB-related transcription factors located upstream of the regulatory network. Rectangles represent neighbouring genes located downstream of the regulatory network. The shades of colour indicate high and low gene rankings. **d** DNB genes regulate the differential expression of neighbouring genes. We selected DEGs in Clusters 4, 5 and 7 in the soft clustering analysis of neighbouring genes, as well as their corresponding DNB genes, and drew a network graph of the changes in the expression levels of these genes. Triangles and diamonds indicate DNB genes with and without transcription factor function located in the centre of the network. Rectangles represent neighbouring genes located at the periphery of the network. The shades of colour indicate high and low gene ranking.

The transcriptome expression profile at a certain time point reflects the state of the sample in that instant. To characterize the specific state at each time point during the progressive pathological aggregation of α-Syn, we identified 6150 DEGs via multiple comparisons with FDR adjustment (*p* < 0.05, Supplementary Table 1). After hierarchical clustering of the DEGs, we found that the samples at each time point were clustered into one class, implying good repeatability of parallel sample clustering (Fig. 2a). In addition, the samples assessed at 0 h, 8 h, and 12 h are first clustered into one class, and the samples assessed at 4 h clustered into only one class. On the other hand, hierarchical clustering led to the clustering of all differentially expressed genes into 5 clusters. For Cluster 2 and Cluster 3, the expression levels at 0 h, 8 h, and 12 h were similar, with genes all highly expressed relative to their expression assessed at 4 h. For Cluster 5, compared with the expression level at 0 h, which was the control level, the expression level at 4 h was significantly higher, and the expression level at 8 h and 12 h was only slightly higher. These findings indicated that the expression profile at 4 h was quite different from that at the other three time points. We performed a KEGG pathway enrichment analysis with the genes in these three clusters and found that the genes in Cluster 2 and Cluster 3 were enriched in pathways such as the neuroactive ligand‒receptor interaction pathway and cell adhesion molecule pathway (Supplementary Fig. 3a). The genes in Cluster 5 were enriched in pathways such as the basic transcription factor pathway and nucleocytoplasmic transport pathway (Supplementary Fig. 3b). These findings indicated that compared with those in the cells assessed 0 h, 8 h and 12 h after induction, more transcription factors entered the nucleus to regulate transcription 4 h after induction. Four hours after induction, cell adhesion ability and neural activity were inhibited, which deviated from the normal state of nerve cells to a certain extent, suggesting that a critical transition may take place 4 h after induction.

We then used the DNB method and identified 4 h after induction as the critical time point before pathological aggregation of α-Syn, with a strong signal indicating the critical state before pathological aggregation of α-Syn composed of a significant change in single-sample landscape entropy (SLE) 4 h after induction. (Fig. 2b). Moreover, we identified the corresponding DNB members, which were composed of 453 genes. DNB genes are core genes in some gene networks, and their expression fluctuates dramatically at critical time points. A soft clustering analysis showed that the majority of the identified DNB genes were expressed at the highest or lowest levels compared to that of all the other time points 4 h after induction (Supplementary Fig. 4). This finding indicated that the expression levels of most DNB genes had markedly changed at the critical time point before pathological aggregation, which corroborated the conclusion that 4 h after induction was the critical time point before pathological aggregation of α-Syn. In Supplementary Fig. 4, the DNB genes of DNB-cluster 1 and DNB-cluster 2 were expressed at the highest levels at 4 h after induction, compared with other time points. And the DNB genes of DNB-cluster 4 were expressed at the lowest levels at 4 h after induction. We performed a KEGG pathway enrichment analysis with the DNB genes in these three DNB-clusters and found that the genes in DNB-cluster 1 and DNB-cluster 2 were enriched in pathways such as the nucleocytoplasmic transport pathway (Supplementary Fig. 5a). This finding indicated the frequent material transport between the nucleus and cytoplasm at 4 h after induction, which echoed the results in Supplementary Fig. 3b. The genes in DNB-cluster 4 were enriched in pathways such as the biosynthesis of amino acids pathway and metabolism pathways of various amino acids (Supplementary Fig. 5b). These findings indicated that the amino acid biosynthesis and metabolism of cells were inhibited 4 h after induction, and the cells were not in normal growth state, suggesting that a critical transition may occur 4 h after induction.

To investigate the regulatory role played by DNB genes, we used the STRING database and retrieved 2418 genes that neighboured DNB genes (Supplementary Table 2). A PPI analysis revealed that the 100 most highly ranked genes in the topological analysis were located at the core of the PPI network, suggesting that they may exhibit a relatively important biological function in the pathological aggregation of α-Syn (Supplementary Fig. 6). To investigate how DNB genes regulate neighbouring genes in depth, we identified 28 differentially expressed DNB genes that had been identified with transcription factor function and 75 differentially expressed downstream neighbouring genes. We then mapped DNB-related transcription factor regulatory networks, which revealed that DNB genes, which encoded transcription factors (TFs), regulated neighbouring genes that were differentially expressed before and after induction and that DNB gene products also interacted with each other (Fig. 2c). Interestingly, we also found that certain DNB genes were expressed at low levels at 0 h and highly expressed at 4 h; in contrast, other genes were highly expressed at 0 h and expressed at low levels at 4 h, and their expression levels underwent a reversal between 0 h and 4 h. However, the expression levels of the neighbouring genes regulated by these DNB transcription factor genes underwent a reversal in expression between 0 h and 12 h (Fig. 2d, Supplementary Fig. 7). In conclusion, we found that the critical time point before the pathological aggregation of α-Syn was 4 h after induction and that DNB genes with transcription factor function regulated the differential expression of their target genes before and after induction, thus establishing a connection between DNB genes and DEGs.

### DNB genes and neighbouring genes were enriched in the cellular senescence pathway

**Fig. 3:**
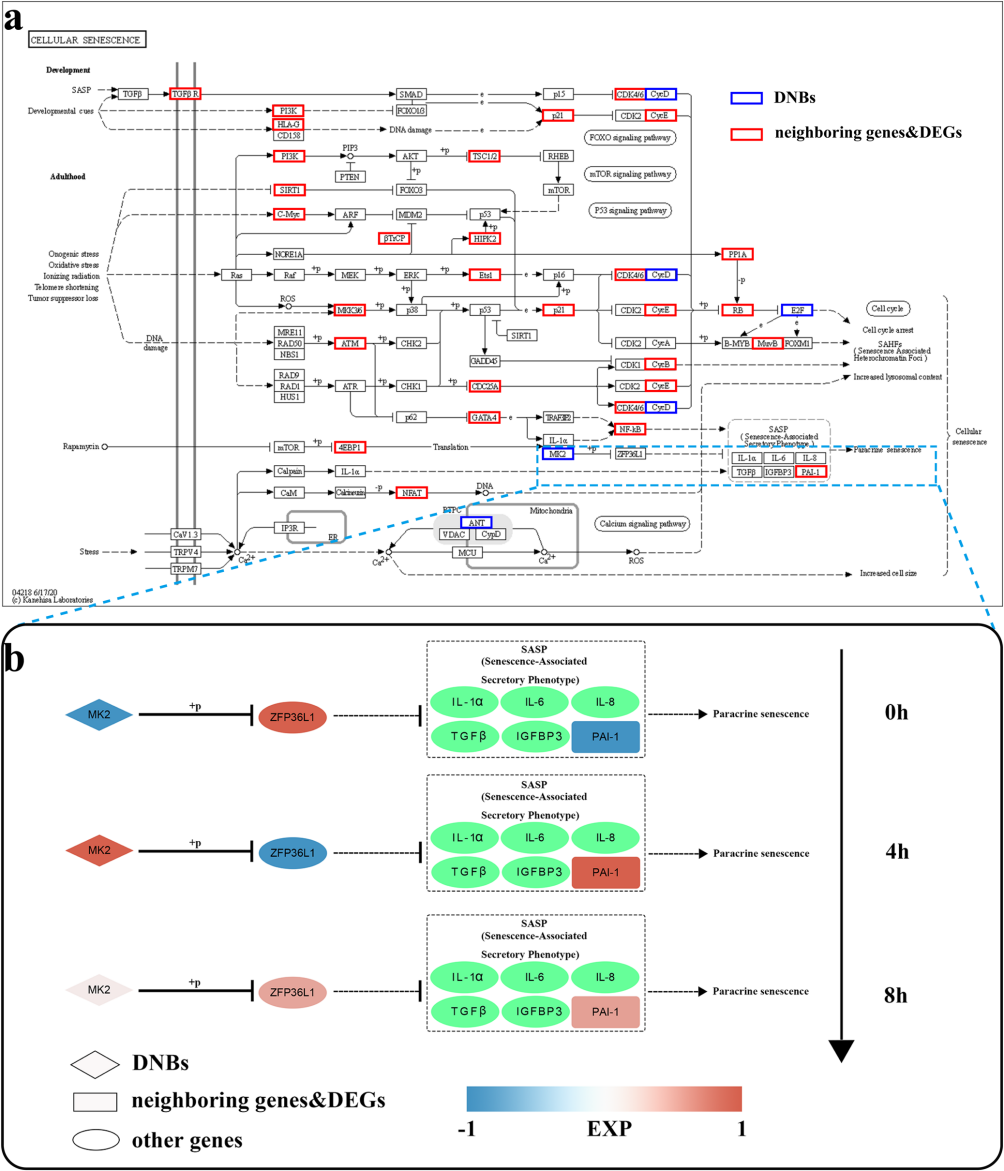
The DNB gene *MAPKAPK2* regulates neighbouring gene *SERPINE1* expression in the cellular senescence pathway. **a** Multiple DNB genes and neighbouring genes were enriched in the cellular senescence pathway. Blue borders indicate DNB genes, and red borders indicate differentially expressed neighbouring genes. The light blue dashed box represents a portion of the pathway studied in detail. **b** *MAPKAPK2* regulated *SERPINE1* expression and activated the cellular senescence pathway. Diamonds represent DNB genes, rectangles represent differentially expressed neighbouring genes, and ovals represent other genes that are neither DNB genes nor neighbouring genes. The colour indicates the expression level of a gene, and the green background indicates that the expression levels of genes that are not shown.

After studying the expression levels and regulatory networks of the DNB genes and their neighbouring genes, we focused on the pathways through which these genes influenced the pathological aggregation of α-Syn. A KEGG pathway enrichment analysis revealed that these genes were significantly enriched in pathways such as the Parkinson’s disease pathway, pathways of neurodegeneration-multiple diseases and the cellular senescence pathway (Supplementary Fig. 8). We annotated the DNB genes and the differentially expressed neighbouring genes involved in these pathways and found that, in part of the cellular senescence pathway, a DNB gene was located upstream adjacent to a neighbouring gene; therefore, we selected this pathway for in-depth study (Fig. 3).

At the critical time point before the aggregation of α-Syn, the expression of the DNB gene *MAPKAPK2* in the cellular senescence pathway was upregulated, which increased the expression level of the downstream differentially expressed neighbouring gene *SERPINE1* via regulation of the expression of the zinc finger protein ZFP36L1. Eight hours after induction, that is, after the critical time point, the expression levels of *MAPKAPK2* and *SERPINE1* decreased but were still higher than those at 0 h. The product of the *SERPINE1* gene is plasminogen activator inhibitor-1 (PAI-1), one of the components of the senescence-associated secretory phenotype (SASP), whose upregulation leads to the activation of paracrine senescence, promoting cellular senescence and impairing autophagy–lysosomal activity^24,25^. The substrate of PAI-1, plasminogen activator, regulates the production of plasmin, which degrades both normal α-Syn and pathological α-Syn^26^. Thus, at the critical time point before the pathological aggregation of α-Syn, upregulation of the DNB gene *MAPKAPK2* caused upregulation of the differentially expressed neighbouring gene *SERPINE1*, which promoted pathological aggregation of α-Syn by affecting the cellular senescence pathway, impeding plasmin production and impairing autophagy‒lysosome pathway activity.

### *MAPKAPK2* is significantly highly expressed in the brain tissue and peripheral blood of PD patients

**Fig. 4:**
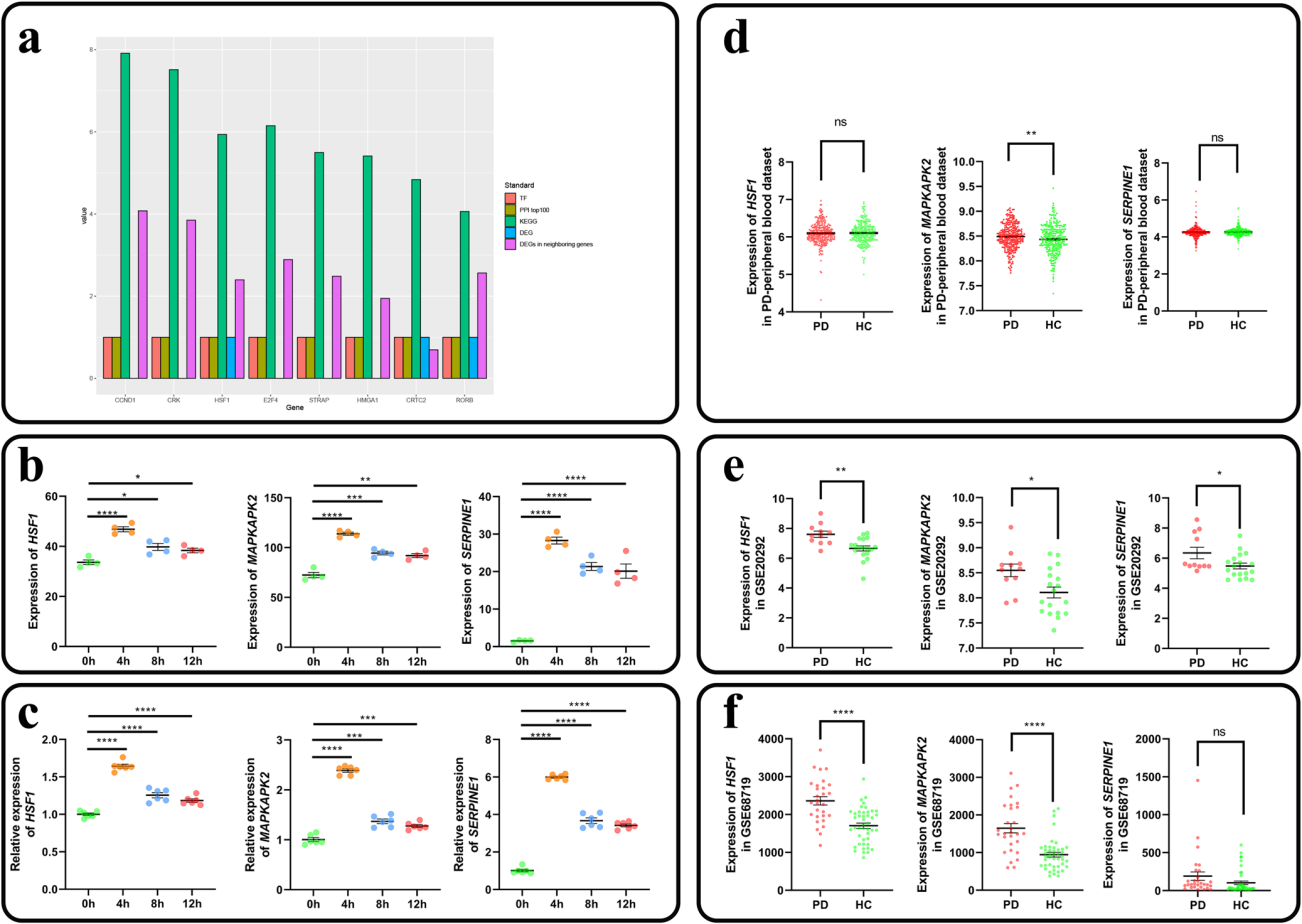
Identification of DNB core genes and mRNA expression of *HSF1*, *MAPKAPK2* and *SERPINE1*. **a** Screening of DNB core genes. TF, transcription factor. PPI top100, the 100 genes with the highest topological analysis score in the PPI network. KEGG, the sum of the number of DNB genes and their neighbouring genes involved in significant pathways shown after natural logarithm processing. DEG, differentially expressed gene. DEGs in neighbouring genes, the number of differentially expressed genes in neighbouring genes after natural logarithm processing. **b**, **c** Dynamic changes in the expression levels of *HSF1*, *MAPKAPK2* and *SERPINE1* as determined with sequencing data (**b**, *n* = 4) and qPCR experiment (**c**, *n* = 6). **d**, **e** and **f** Expression levels of *HSF1*, *MAPKAPK2* and *SERPINE1* in PD patients and healthy controls in PD-related GEO data obtained from peripheral blood (**d**), the substantia nigra (**e**) and the prefrontal cortex (**f**) sources. ns: no significant difference. *p < 0.05. **p < 0.01. ***p < 0.001. ****p < 0.0001. The data are expressed as the means ± SEMs. PD: PD patients. HC: healthy controls.

To identify potential key genes leading to pathological aggregation of α-Syn, we comprehensively ranked DNB genes based on five priority criteria (see the screening protocol for DNB core genes in the Materials and methods section) and chose to identify eight genes, including *CCND1*, *CRK* and *HSF1*, as DNB core genes (Fig. 4a, Supplementary Table 3). Among these genes, we found that the *SERPINE1* gene neighboured the DNB core gene *HSF1* (Supplementary Fig. 9). Using the JASPAR database, we found that the transcription factor HSF1 binds three sites upstream of *SERPINE1* (Supplementary Table 4). Furthermore, it has been previously shown that in vascular endothelial cells, *HSF1* positively regulated PAI-1 expression levels^27,28^. Therefore, *HSF1* potentially regulates the expression level of *SERPINE1*. Considering the results of the aforementioned pathway studies, we suggest that *HSF1* and *SERPINE1*, as well as *MAPKAPK2*, may play important roles in the pathological aggregation of α-Syn.

To identify the relationship between these three genes and pathological aggregation, we found that all three *HSF1*, *SERPINE1*, and *MAPKAPK2* genes were expressed at significantly higher levels in both the prepathological aggregation state and the pathological aggregation state than in the normal state. This result was corroborated by qPCR experiments with these genes. Therefore, these three genes may be potential biomarkers before the pathological aggregation of α-Syn.

To identify the relevance of these three genes to α-Syn pathological aggregation-related diseases, we collected PD-related GEO data and measured the expression levels of these three genes (Supplementary Table 6). The results showed that the expression levels of all three genes in were significantly higher in the substantia nigra in PD patients than in HCs (Fig. 4e). In the prefrontal cortex, only the expression levels of *HSF1* and *MAPKAPK2* were significantly higher in PD patients than in HCs (Fig. 4f). The analysis of PD-peripheral blood dataset integrating four GSE datasets showed that in peripheral blood, only the expression level of *MAPKAPK2* was significantly higher in PD patients than in HCs (Fig. 4d, Supplementary Figs. 10 and 11). The independent analysis of four peripheral blood datasets showed that only in GSE99039 dataset, the expression level of *MAPKAPK2* was significantly higher in PD patients than in HCs (Supplementary Fig. 12).

Similarly, we performed analyses of DLB and multiple stem atrophy (MSA), two neurodegenerative diseases associated with the pathological aggregation of α-Syn, and found that in DLB-related data obtained from the prefrontal cortex, the expression level of *MAPKAPK2* was significantly higher in DLB patients than in HCs, while the expression levels of the other two genes did not differ significantly between DLB patients and HCs; in MSA-related data obtained from cerebellar white matter, the expression levels of the three genes also did not differ significantly between MSA patients and HCs (Supplementary Fig. 13).

Hence, qPCR experiments and GEO clinical data corroborated the correlation between the expression levels of the DNB core gene *HSF1* and the DNB gene *MAPKAPK2* and the neighbouring gene *SERPINE1*, indicating that these three genes may be potential biomarkers indicating the pathological preaggregation of α-Syn and that *MAPKAPK2* in peripheral blood may serve as a potential biomarker for early PD diagnosis, which may be rendered before the pathological aggregation of α-Syn.

## Discussion

One of the important pathological features of Parkinson’s disease is the aggregation of α-Syn, which is the main component of Lewy bodies, in the substantia nigra. Pathologically aggregated α-Syn induces neurotoxicity and can lead to the death of dopaminergic neurons, leading to Parkinson’s disease. Therefore, prevention of the pathological aggregation of α-Syn is critical. To explore potential key genes affecting the pathological aggregation of α-Syn and to identify potential biomarkers for the early diagnosis of α-Syn pathological aggregation-related diseases, we used MPP^+^ for induction, constructed a cell model of pathological aggregation of α-Syn, applied DNB analysis based to a gene expression network model, and identified 453 DNB genes and 4 h post-induction as the critical time point before pathological aggregation of α-Syn. Furthermore, we found that DNB genes enriched in the cellular senescence pathway affected the pathological aggregation of α-Syn. Finally, we identified *HSF1* as a core DNB gene and found that *HSF1* and the DNB gene *MAPKAPK2* may regulate the neighbouring gene *SERPINE1*, with all three potential biomarkers of the pathological preaggregation of α-Syn, and combined with clinical data, we identified *MAPKAPK2* in peripheral blood as a potential biomarker for the early PD diagnosis based on pathological pre-aggregation of α-Syn.

We used MPP^+^ to induce SH-SY5Y cells and construct a cell model of pathological aggregation of α-Syn. MPP^+^ is commonly used to induce Parkinson’s cell models. MPP^+^ acts on the mitochondrial respiratory chain enzyme complex I in dopaminergic neurons, blocking respiratory chain electron transmission, leading to disruption in energy metabolism and a series of oxidative stress injuries, as well as impairing dopamine transporter function. MPP^+^ causes a local increase in glutamate, which indirectly leads to impaired mitochondrial function and accelerates dopamine oxidative metabolism, increasing the production of reactive products such as peroxides and causing oxidative damage to dopamine neurons. Lin et al. treated human SH-SY5Y cells with low doses of MPP^+^ and found a sustained increase in α-Syn monomer levels from 0 h to 72 h after administration, which indicated that induction of low doses of MPP^+^ led to the development of pathological aggregation of α-Syn^29^. To verify that the cell model for the pathological aggregation of α-Syn was successfully constructed, we performed cellular immunofluorescence with two different antibodies, namely, the 5G4 antibody and an anti-p-α-Syn antibody. The 5G4 antibody is a monoclonal antibody that specifically binds to pathologically aggregated α-Syn^30^. In 2019, Qiao et al. performed cellular immunofluorescence experiments using the 5G4 antibody and demonstrated that methamphetamine induction increased the aggregation of pathological α-Syn in SH-SY5Y cells^31^. The anti-p-α-Syn antibody specifically binds to α-Syn phosphorylated at serine 129. This phosphorylation modification is found in PD patients but not healthy people. Moreover, this phosphorylation modification has frequently been found pathological α-Syn^32–34^. In 2021, Zhang et al. performed immunofluorescence staining with sural nerve samples obtained from PD patients and healthy individuals using an anti-p-α-Syn antibody. Intense and bright anti-p-α-Syn antibody staining was observed in samples obtained from PD patients, whereas no p-α-Syn antibody staining was observed in samples obtained from healthy individuals^35^. Finally, according to the immunofluorescence staining results obtained with these two antibodies, we successfully constructed a cell model of α-Syn pathological aggregation, and at the same time, we found the appearance of α-Syn pathological aggregation 12 h after induction.

Combining multiple bioinformatics analysis methods, we found that the upregulated expression of the DNB gene *MAPKAPK2* caused the upregulated expression of the differentially expressed neighbouring gene *SERPINE1*, which blocked the production of plasmin and impaired the activity of the autophagy–lysosomal pathway by affecting the cellular senescence pathway. In addition, we found that one of the genes neighbouring the DNB core gene *HSF1* was *SERPINE1*, and the transcription factor HSF1 was found to bind three sites upstream of the *SERPINE1* gene. Zhou et al. found that *HSF1* positively regulated the expression level of PAI-1 in endothelial cells; hence, *HSF1* theoretically can regulate the expression of *SERPINE1*^27,28^. In this study, *MAPKAPK2* and *HSF1* are DNB genes. DNB gene are at the core of the networks in which the expression levels of members and the network structures change dramatically under the predisease state, and can distinguish between the normal state and the predisease state. DNB genes are obtained from time sequence transcriptome data analysis, which have dynamic characteristics. DEGs are static results based on the comparison of two groups of gene expression data, which can only distinguish between normal state and disease state. In a word, DNB genes and DEGs are two different concepts. Although *MAPKAPK2* and *HSF1* in this study are both DNB genes and DEGs, some DNB genes are not DEGs, while *SERPINE1* gene is not a DNB gene. There may also be a regulatory relationship between DNB genes and DEGs. For example, in this study, the DNB gene *HSF1* regulates the differential expression gene *SERPINE1*.

The product of the *MAPKAPK2* gene is MAPK-activated protein kinase 2 (MK2). In 2008, Tobias et al.‘s in vitro culture experiments showed that *MAPKAPK2*-deficient mouse dopaminergic neurons were more resistant to neurotoxicity than wild-type neurons, and they suggested that eliminating MK2 expression can prevent neurodegeneration^36^. The product of the SERPINE1 gene is PAI-1, one of the components of the SASP. Plasmin is a serine protease derived from inactive plasminogen, which is activated by tissue plasminogen activator (tPA) or urokinase plasminogen activator (uPA). Plasmin plays a central role in fibrinolysis by dissolving insoluble fibrin, rendering it into soluble fibrin degradation products^37^. Plasmin activity is regulated by the activities of tPA and uPA and by inhibitors of tPA and uPA, including PAI-1. In 2012, Park et al. conducted in vitro experiments and found that plasmin degraded normal α-Syn and pathological α-Syn^26^. In addition, some studies have shown that the plasma PAI-1 level in PD patients was significantly higher than that in healthy people, and the cognitive function of PD patients was negatively correlated with plasma PAI-1 level^38,39^. In the cellular senescence pathway, in the critical state before the pathological aggregation of α-Syn, the upregulation of the DNB gene *MAPKAPK2* led to ZFP36L1 phosphorylation, inhibiting its activity. ZFP36L1 is involved in posttranscriptional regulation. It binds to the ARE (AU-rich element) sequence in the 3’-UTR of the target mRNA through a tandem zinc finger structure, thereby promoting the deadenylation and decapping of the target mRNA. This mRNA modulation leads to the degradation of the polyadenylic acid tail structure of the target mRNA, which in turn leads to the degradation of the target mRNA and plays a role in posttranscriptional regulation^40^. ZFP36L1 regulated the SASP by reducing the mRNA expression of SASP components, thereby inhibiting cell senescence. However, in the critical state, ZFP36L1 activity was inhibited, and therefore, the expression of the neighbouring gene *SERPINE1* was upregulated, PAI-1 expression was upregulated, plasminogen activator activity was decreased, the level of plasmin was decreased, and α-Syn started to accumulate. Excessive accumulation of α-Syn led to its pathological aggregation. In addition, upregulation of PAI-1 expression triggered paracrine senescence, which promoted cellular senescence and disrupted autophagy‒lysosome activity, which is an important pathway by which cells degrade toxic oligomeric α-Syn^24,25^. Meanwhile, we found that multiple DNB genes (*CCND1*, *E2F4*) and differentially expressed neighbouring genes (*ATM*, *CDKN1A*, *CDK6*, *CCNE1*) were involved in the ATM/p53/p21/Rb pathway in the cellular senescence pathway. The ATM/p53/p21/Rb pathway is associated with cell cycle arrest, and we hypothesized that this pathway may influence the pathological aggregation of α-Syn and be involved in the development of aging-related diseases. This remains in-depth experiments to further confirm.

**Fig. 5:**
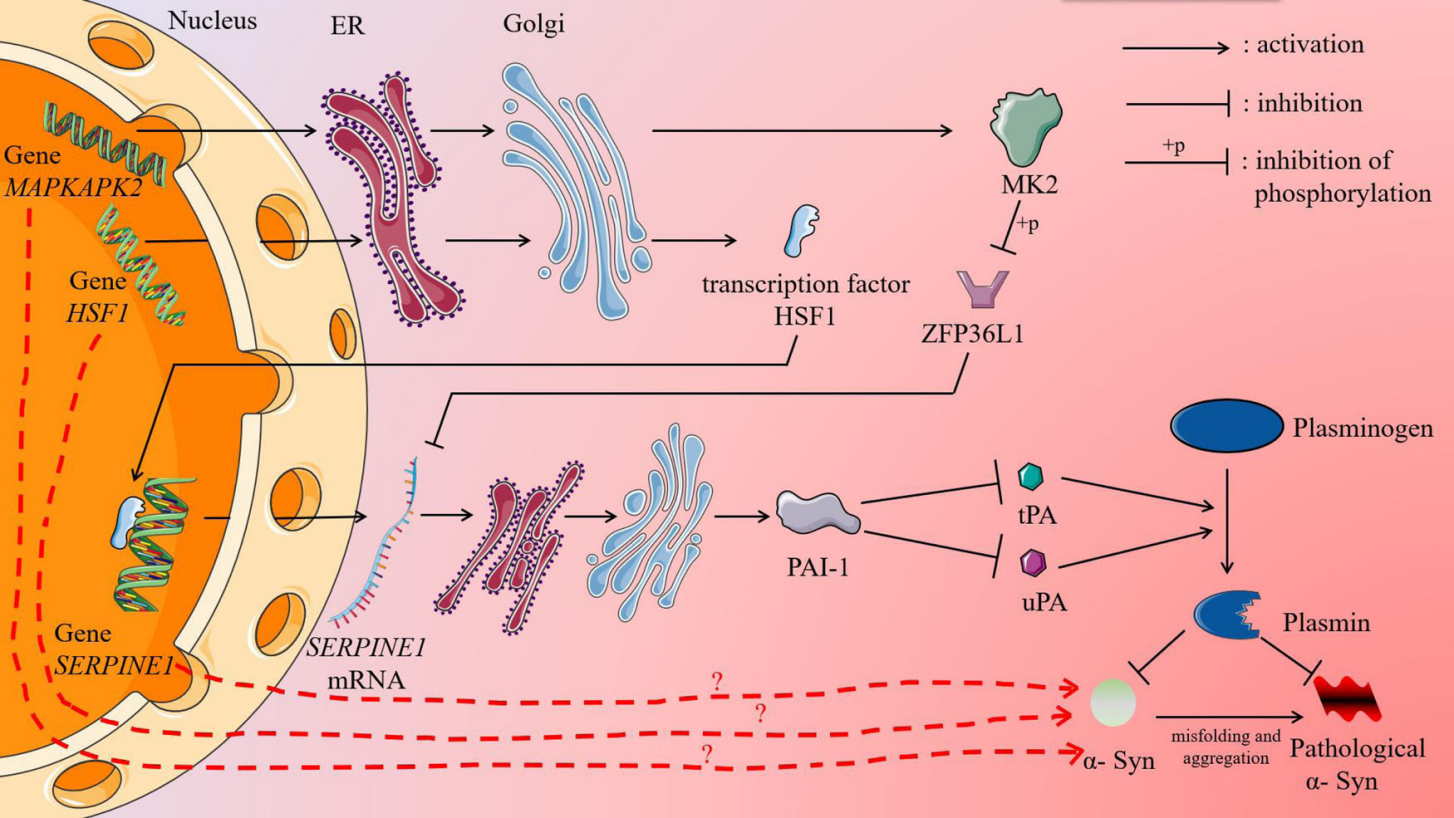
Graphical abstract showing *HSF1* and *MAPKAPK2* regulating *SERPINE1* expression and ultimately promoting the pathological aggregation of α-Syn. In the prepathological aggregation state, the expression of the DNB genes *HSF1* and *MAPKAPK2* is upregulated, upregulating neighbouring gene *SERPINE1* at the transcriptional and posttranscriptional levels, respectively, resulting in high expression of its product PAI-1, which inhibits plasminogen activator activity and thus reducing plasmin production, preventing α-Syn degradation, and leading to the accumulation and aggregation of α-Syn. When the accumulation reaches a certain level, excess normal α-Syn accumulates, leading to pathological aggregation; that is, α-Syn enters a state of pathological aggregation. The figure was partly generated by adapting Servier Medical Art pictures provided by Servier, licensed under a Creative Commons Attribution 3.0 unported license.

The *HSF1* gene encodes heat shock protein transcription factor 1 (HSF1). Normally, this transcription factor is regulated by an inhibitory complex and remains in a latent state, and under stress, HSF1 is transiently activated and triggers heat shock protein (HSP) expression in response to various forms of physiological and environmental stress. Xu et al. constructed a mutant model of SH-SY5Y cells with the mutation of *HSF1* to *HSF1*( + ), which resulted in enhanced expression of *HSF1* in the absence of stress, and found that the mutation increased the expression of HSP70 and reduced total α-Syn levels and the toxicity induced by α-Syn in SH-SY5Y cells^41^. Although upregulation of *HSF1* expression was found to reduce the toxicity induced α-Syn in this study, no studies related to α-Syn pathological aggregation were conducted. Both sequencing data and qPCR experimental validation showed that the expression levels of *HSF1*, *MAPKAPK2* and *SERPINE1* were significantly or extremely significantly upregulated at 4 h and 12 h, the prepathological aggregation state and the pathological aggregation state, respectively, with 0 h used as the control. Notably, although the expression levels of *HSF1* and *MAPKAPK2* were significantly different between 0 h and 12 h, according to the sequencing data, these two genes were not among the DEGs identified in the differential expression analysis of performed between 0 h and 12 h (Supplementary Table 1). This finding suggests that traditional molecular biomarker methods for distinguishing normal and disease states cannot identify these two genes and that they can be identified only via the DNB method. Sequencing data and qPCR experiments as well as analysis of PD-related GEO data also demonstrated that the expression levels of the DNB core gene *HSF1* and the DNB gene *MAPKAPK2* correlated with the level of the neighbouring gene *SERPINE1* and that these three genes may be potential biomarkers of the pathological pre-aggregation of α-Syn, while revealing that *MAPKAPK2* in peripheral blood may be a potential biomarker for an early PD diagnosis rendered before the pathological aggregation of α-Syn. In the PD-peripheral blood dataset (with 305 PD patients and 283 HCs), which integrated four datasets, and the GSE99039 dataset (with 205 PD patients and 233 HCs), the expression level of *MAPKAPK2* was significantly higher in PD patients than in HCs. However, in the other datasets, including GSE6613, GSE72267 and the GSE100054 dataset, the expression level of *MAPKAPK2* did not differ significantly between PD patients and HCs, which were probably caused by the small sample size of these datasets (Fig. 4d and Supplementary Fig. 12). Finally, we proposed a hypothetical molecular mechanism through which the DNB genes *HSF1* and *MAPKAPK2* regulate the neighbouring gene *SERPINE1* at the transcriptional level and posttranscriptional level, respectively, in the prepathological aggregation state; this mechanism led to the upregulation of PAI-1 expression, causing the accumulation and aggregation of α-Syn, which was not degraded, and ultimately promoting pathological aggregation of α-Syn (Fig. 5). Although experimental or theoretical support for each step of the mechanism, the expression levels of these three genes have not yet been altered to determine the effects of their changed expression on α-Syn aggregation; therefore, the possible molecular mechanism remains to be experimentally validated.

There are some limitations to this study. First, we used a cell model for our studies, which, although human in origin, was differed significantly from the course of PD pathology in humans. Second, the analyses of the genes in this study was performed at the theoretical level, and no subsequent causal experiments were performed to support the conclusions obtained from these analyses. Therefore, we will subsequently validate the findings in a PD mouse model and collect time-series physical examination cohort population data to verify the difference between *MAPKAPK2* expression in PD patients and HCs, and we will also perform experiments involving gene editing to determine whether the proposed molecular mechanisms is valid.

In conclusion, by constructing a cell model of α-Syn pathological aggregation and using the DNB method, we detected that 4 h after induction is the critical time point before pathological aggregation of α-Syn. The DNB gene promoted the pathological aggregation of α-Syn through the cellular senescence pathway, hindering the production of plasmin and inhibiting the activity of the autophagy–lysosomal pathway. Importantly, we found that the *MAPKAPK2* expression level in peripheral blood is a potential biomarker for early PD diagnosis, which can be rendered before pathological aggregation of α-Syn. Finally, we proposed that the DNB genes *HSF1* and *MAPKAPK2* regulated the expression of the neighbouring gene *SERPINE1*, indicating that all three genes are potential key genes that are involved in the transition to the pathological aggregation of α-Syn.

## Methods

### α-Syn pathological aggregation cell model

Human neuroblastoma cells (SH-SY5Y cells) were purchased from Procell Life Science & Technology Co., Ltd. SH-SY5Y cells were cultured in MEM/F12 (Gibco) containing 10% foetal bovine serum and 1% penicillin/streptomycin, hereafter referred to marked as SH-SY5Y medium. The cells were maintained at 37 °C in an atmosphere of 5% carbon dioxide and 95% humidity. The induction medium consisted of SH-SY5Y medium supplemented with MPP^+^(D048, Sigma‒Aldrich) at a concentration of 5 μM. To establish a α-Syn pathological aggregation cell model, we first cultured SH-SY5Y cells on cell culture dishes or coverslips with SH-SY5Y medium for 24 h. Next, we aspirated the original SH-SY5Y medium, washed the cells with PBS, and added an equal volume of induction medium to obtain the induced treatment group. The control group was obtained in a similar way but the SH-SY5Y medium was replaced with fresh SH-SY5Y medium, not induction medium.

### Immunostaining analysis

We first cultured SH-SY5Y cells with SH-SY5Y medium on coverslips with a poly-D-lysine coating and then added induction medium as described. The cells were fixed with 4% paraformaldehyde for 30 min at each induction time points (0 h, 4 h, 8 h, and 12 h, *n* = 4) and were then washed twice with PBS and blocked with 3% BSA for 30 min at room temperature. To assess the degree of pathological α-syn aggregation, we incubated the fixed cells overnight at 4 °C with a 5G4 antibody (1:400, Merck) or an anti-p-α-syn antibody (1:500, Abcam). The samples were then washed with PBS and incubated at room temperature with fluorescein isothiocyanate (FITC)-conjugated secondary antibody (Jackson Immunology Laboratories, Inc.) or cyanidin 3 (CY3)-conjugated secondary antibody (Jackson Immunology Laboratories, Inc.) for 50 min. Then, the samples were incubated for 10 min at room temperature, protected from light and treated with 4′,6-diamidino-2-phenylindole dihydrochloride (DAPI). Fluorescence images were acquired with a confocal microscope (Zeiss Confocal LSM 710) after the coverslips were mounted. All images were processed with Zeiss Zen software. The average immunofluorescence intensity of the antibody in selected areas was measured with ImageJ software.

### RNA extraction and RNA sequencing

SH-SY5Y cells were treated with induction medium and collected at the various time points after induction (0 h, 4 h, 8 h, 12 h). The collected cells were immediately lysed with TRIzol (Beyotime), and total RNA was prepared using an RNeasy Plus Mini Kit (Qiagen) per the manufacturer’s instructions. A portion of the total RNA in the cell samples was used for RNA-seq. The total RNA in each sample was quantified and qualified with an Agilent 2100 Bioanalyzer (Agilent Technologies) and NanoDrop spectrophotometer (Thermo Fisher Scientific Inc.). RNA-seq libraries were prepared with an R8.VAHTS mRNA-seq V3 Library Prep Kit for Illumina (NR611-01, Vazyme) per the manufacturer’s instructions. High-throughput sequencing was performed using an Illumina NovaSeq 6000 (Novo Gold Bioinformatics, Ltd.). The amount of data per sample was 6 G in four parallel samples per time point. The concentration of the other portion of the total RNA was measured with a NanoDrop spectrophotometer and quickly reverse transcribed into cDNA using HiScript II Q Select RT SuperMix in a qPCR kit (R232-01, Vazyme). The cDNA was stored at −20 °C for subsequent use in qPCR experiments. Six parallel experiments were established per time point.

### DEG identification

DESeq2 (version 1.30.1) was used to identify differentially expressed genes (DEGs) between different stages. Genes in which differences in expression were associated with a p.adj value <0.05 and a fold change > 0.3 were identified as DEGs^42^.

### Clustering

We used hierarchical clustering to cluster the expression profiles of DEGs at different time points and determine the general expression of DEGs at each time point. We simultaneously used a more noise-robust soft clustering method (R package: Mfuzz) to cluster DNB gene expression profiles and first-order neighbouring gene (hereafter referred to as neighbouring genes) expression profiles according to time trends. The clustering hyperparameters were set to 4 and 9^16^.

### KEGG pathway enrichment analysis

To gain insight into the biological functions of DEGs and DNB genes in the cells and their regulatory relationships with other genes, we used Kobas (version 3.0) and the KEGG Pathway database to perform KEGG pathway enrichment analysis and subsequent in-depth studies into DEGs and DNB genes and their neighbouring genes. The KEGG pathways were identified on the basis of a p.adj value < 0.05 indicating a significantly enriched pathways in this study.

### Theoretical basis of the DNB method

The DNB method can be used to characterized a cell state before it undergoes a critical transition from the normal state into the pathological state, which in this case is the state of α-Syn^43^. In the critical transition state, network gene expression undergoes dramatic fluctuations, and DNB biomolecules are at the core of these networks. DNB biomolecules were recognized when the following three statistical conditions were satisfied:

1. The SD_in_ of the genes in the DNB group increased markedly, where SD_in_ represents the standard deviation or coefficient of variation;
2. The PCC_in_ of genes in the DNB group increased sharply, where PCC_in_ represents the Pearson’s correlation coefficient; and
3. The PCC_out_ declines rapidly, where PCC_out_ represents the Pearson’s correlation coefficient between any member in the DNB group and any other non-DNB biomolecule;

The three statistical conditions re necessary conditions for phase transition in biological systems. A quantitative analysis of the variables in the networks that undergo dramatic fluctuations may indicate early warning signals of critical transitions in the system.

### Single-sample landscape entropy (SLE) algorithm

The SLE is a specific algorithm based on DNB method theory^44^. It is used to explore dynamic differences between normal and predisease states and for identifying local network-based entropy, producing an SLE score that characterizes the statistical perturbations attributed by each treatment group sample to a given set of control group samples. Specifically, the SLE requires that a number of control group samples are first defined, and then, the following steps are performed:

[step 1] Use the STRING database to map genes to protein‒protein interaction (PPI) networks (or other template networks) to form the global network N^G^.

[step 2] Extract each local network from the global network N^G^ such that each local network N^X^ (X = 1, 2, 3,…, K) is centred on the gene g^*x*^. Suppose that there are M first-order neighbouring genes of gene g^*x*^ in the g^*x*^-local network, that is, g^1*x*^, g^2*x*^, g^3*x*^, …, g^*Mx*^. If there are K genes in the global network N^G^, then there is a total of K local networks.

[step 3] For each local network N^X^ (X = 1, 2, 3,…, K) at time point *t*, based on *n* control samples {*s*_1_(*t*), *s*_2_(*t*), …, *s*_*n*_(*t*)}, calculate the local network entropy *H*^*n*^(*x, t*); i.e.,

1 $$H^n(x,t) = - \frac{1}{{\log M}}\mathop {\sum }\limits_{i = 1}^M p_i^n(t)logp_i^n(t)$$

with

2 $$p_i^n(t) = \frac{{|PCC^n({{{\mathrm{g}}}}_i^x(t),{{{\mathrm{g}}}}^x(t))|}}{{\mathop {\sum }\nolimits_{j = 1}^M |PCC^n({{{\mathrm{g}}}}_j^x(t),{{{\mathrm{g}}}}^x(t))|}}$$

where $PCC^n({{{\mathrm{g}}}}_i^x(t),{{{\mathrm{g}}}}^{{{\boldsymbol{x}}}}(t))$ represents Pearson’s correlation coefficient for the central gene g^*x*^ and a neighbouring gene ${{{\mathrm{g}}}}_i^x$ based on n control samples. In Eq. (1), the superscript *x* indicates that the local network is centred at g^*x*^, the subscript *n* denotes the number of samples and the constant *M* represents the number of neighbouring genes in the local network N^X^. In Eq. (2), The symbols g^*x*^(*t*) and ${{{\mathrm{g}}}}_i^x(t)$ represent the expression of genes g^*x*^ and ${{{\mathrm{g}}}}_i^x$ at time point *t*, respectively.

[step 4] The newly added sample *s*_*case*_(*t*), which is a treatment group individual, is mixed with *n* control group samples. Based on *n* + 1 mixed samples {*s*_1_(*t*), *s*_2_(*t*), …, *s*_*n*_(*t*), *s*_*case*_(*t*)}, calculate the local network entropy *H*^*n*+1^(*x,t*); i.e.,

3 $$H^{n + 1}(x,t) = - \frac{1}{{{{{\mathrm{log}}}}M}}\mathop {\sum }\limits_{i = 1}^M p_i^{n + 1}(t)logp_i^{n + 1}(t)$$

In Eq. (3), the definition of $p_i^{n + 1}$ is similar to that in Eq. (2), but in Eq. (3) the correlation $PCC^{n + 1}({{{\mathrm{g}}}}_{{{\mathrm{i}}}}^x(t),{{{\mathrm{g}}}}^x(t))$ is based on *n* + 1 mixed samples.

[step 5] Calculate the differential entropy ∆*H*^*n*^(*x, t*) between *H*^*n*^(*x, t*) and *H*^*n*+1^(*x,t*); i.e.,

4 $$\Delta H^n\left( {x,t} \right) = \Delta SD(x,t)|H^{n + 1}(x,t) - H^n(x,t)|$$

with

5 $$\Delta SD(x,t) = |SD^{n + 1}(x,t) - SD^n(x,t)|$$

where *SD*^*n*^(*x, t*) and *SD*^*n*+1^(*x, t*) are the standard deviations of the expression of the centre gene g^*x*^ based on *n* control samples {*s*_1_(*t*), *s*_2_(*t*), …, *s*_*n*_(*t*)} and *n* + 1 mixed samples {*s*_1_(*t*), *s*_2_(*t*), …, *s*_*n*_(*t*), *s*_*case*_(*t*)}, respectively. The differential entropy ∆*H*^*n*^(*x, t*) between *H*^*n*^(*x, t*) and *H*^*n*+1^(*x, t*) represents differences caused by the newly added sample *s*_*case*_(*t*) from the treatment group. In other words, the local entropy *H*^*n*^(*x, t*) based on *n* control samples {*s*_1_(*t*), *s*_2_(*t*), …, *s*_*n*_(*t*)}, *H*^*n*+1^(*x, t*) is compared with that based on *n* + 1 mixed samples {*s*_1_(*t*), *s*_2_(*t*), …, *s*_*n*_(*t*), *s*_*case*_(*t*)}, which indicates the perturbation caused by the addition of single sample *s*_*case*_(*t*) to local network N^X^. In addition, to account for gene expression fluctuations, the differential standard deviation ∆*SD*(*x, t*) is regarded as the weight coefficient.

[step 6] Calculate the weighted sum of Δ*H*(*x*) for all local networks; i.e.,

6 $$\Delta H\left( t \right) = \frac{1}{K}\mathop {\sum}\limits_{x = 1}^K {\Delta H} \left( {x,t} \right)$$

where constant K is the number of all genes. In Eq. (6), Δ*H*(*x*) indicates the overall effect caused by the addition of the treatment group sample *s*_*case*_(*t*) and is therefore referred to as the global SLE score, hereafter the SLE score, of the global network N^G^. Similarly, ∆*H*^*n*^(*x, t*) in Eq. (4) is the local SLE score of the local network N^X^, which is centred on gene g^*x*^.

When the system approaches the vicinity of the critical point, the DNB biomolecules exhibit significant collective fluctuations. In a local network with DNB biomolecules represented as nodes, Pearson’s correlation coefficients $PCC^{n + 1}({{{\mathrm{g}}}}_{{{\mathrm{i}}}}^x(t),{{{\mathrm{g}}}}^x(t))$ becomes more similar or are equalized when the system is in a critical state, resulting in an increase in the local SLE score ∆*H*(*x*) in Eq. (4). In addition, ∆*SD*(*x, t*) in Eq. (6) increases accordingly, which contributes to the increase in the global SLE score Δ*H*(*t*). Therefore, the SLE score can provide an early warning signal of an impending critical state transition. When the global SLE score Δ*H*(*t*) reaches a peak value at a certain time point, the time point is considered to be indicative of the critical state (Supplementary Fig. 1).

### PPI network analysis

PPI network analysis was performed by importing the DNB gene list into the STRING database (version 11.0). We used Cytoscape software (version 3.7.1) to export the adjacency matrix for visualization and applied the CytoHubba plugin to perform a topological analysis, in which the node genes were ranked on the basis of their properties in the network, and then, the 100 genes with the highest rankings were visualized^21^.

### Transcription factor annotation

AnimalTFDB (v3.0) is a database of 125,135 TFs and 80,060 transcription cofactors that are classified and annotated at the genome-wide level for 97 species, with various functions, such as transcription, and prediction of transcription factor-binding sites. In this study, we used human the TF database (HumanTFDB), an independent web interface, to annotate DNB genes to facilitate the analysis of the network regulatory relationships involving DNB genes^45^.

### Confirmation of DNB core genes

The criteria we used to identify DNB core genes were: (1) TF annotation of DNB genes, enabling selection of genes with a transcription factor identity; (2) the 100 DNB mostly highly ranked genes in the PPI analysis were selected; (3) KEGG functional enrichment analysis of DNB genes and their neighbouring genes in which each gene was given an attribute; then, the number of genes enriched in a significant KEGG pathway was determined, and DNB genes with the most DNB gene attributes and all their neighbouring genes were selected; (4) differentially expressed genes; and (5) DNB genes with a higher number of DEG-identity genes among their neighbouring genes were selected.

### Identification of transcription factor-binding sites

The UCSC database contains genome assembly and annotation data for a large number of vertebrates and model organisms^46^. In this study, *SERPINE1* in the hg38 version of the human reference genome was searched in the UCSC database. This gene was located on the chr7:101,127,104-101,139,247 in the genome. The sequence from the start site to 2000 bp upstream of this gene, chr7:101,125,104-101,127,103, was searched and downloaded. The JASPAR database is an open source database of transcription factor-binding site information that is reported in the form of position frequency matrices and TF flexibility models based on recorded DNA-binding preference information for transcription factors in six different groups of organisms, and this database can be used to predict the binding regions of transcription factors to sequences^47^. In this study, we searched the JASPAR database for the transcription factor HSF1 and identified the binding site of this transcription factor in the 2000 bp sequence upstream of *SERPINE1*.

### Quantitative PCR (qPCR)

The *HSF1*, *MAPKAPK2* and *SERPINE1* primer pairs are listed in Supplementary Table 5. The β-actin gene was used as the reference gene for qPCR analysis. Reagents for qPCR were obtained from Takara Biotechnology (DRR096A; Dalian, China). Relative expression was calculated using the following formula: relative expression = 2^−ΔΔCt^; the relative expression was normalized based on the expression level of the samples 0 h after induction^48^.

### The GEO database

We searched a blood microarray dataset in the GEO database using the keywords “PD”, “blood”, and “Homo sapiens” and ultimately selected the GSE6613, GSE72267, GSE99039 and GSE100054 databases, which included information on 305 PD patients and 283 healthy controls (HCs) in total. Detailed information on the datasets is provided in Supplementary Table 6. We downloaded the raw data and platform information of these datasets and then annotated the probe ID after preprocessing the raw data. The common genes were merged into four expression matrices, and the batch effect among them was removed. The raw data of these datasets were processed through the affy package to read the.cel file and RMA algorithm for background correction and data normalization. Then, we normalized four gene expression matrices, and the interbatch difference was removed using the remove batch effect function of the limma package. The boxplots and two-dimensional PCA plots before and after removing the batch effect are shown in the Supplementary Figures. After normalization, the median expression values of the samples from the four datasets were at the same level, and the PCA plot showed that the difference among them was decreased, indicating that the merged expression matrix was appropriate for use in further analysis.

We then collected two PD transcriptome datasets consisting of brain sample data in the GEO database: the GSE20292 dataset (with 11 PD patients and 18 HCs) and the GSE68719 (with 29 PD patients and 44 HCs). The GSE20292 dataset samples, sequenced using the Affymetrix Human Genome U133 Array platform, had been obtained from the substantia nigra in the brain. The GSE68719 dataset samples, sequenced using the Illumina HiSeq 2000 platform, were obtained postmortem from the prefrontal cortex area (BA9). Furthermore, we collected two transcriptome datasets with information on other α-Syn-associated diseases in the GEO database: the GSE150696 dataset (with 12 dementia with Lewy bodies (DLB) patients and 9 HCs) and the GSE199258 dataset (with 19 multiple system atrophy patients and 19 HCs). The GSE150696 dataset samples, sequenced using the Affymetrix Human Transcriptome Array 2.0 platform, were obtained postmortem from the prefrontal cortex area (BA9). The GSE199258 dataset samples, sequenced using the Illumina HiSeq 2500 platform, were obtained from cerebellar white matter.

### Statistical analysis

The number of parallel experiments is shown in the corresponding figure note. The data are depicted as means ± SEMs and were analysed using an unpaired Student’s *t*-test. A *P*-value <0.05 was defined as statistically significant. All graphs and statistical calculations were performed using GraphPad Prism (Version 8.3.0) and R (version 4.0.4).

### Reporting summary

Further information on research design is available in the Nature Research Reporting Summary linked to this article.

## Supplementary information

SUPPLEMENTAL MATERIAL

Reporting Summary

## Data Availability

The RNA-seq data were deposited in the NCBI Sequence Read Archive (SRA) database (https://www.ncbi.nlm.nih.gov/sra/) under the BioProject accession PRJNA859664. Publicly available datasets were analysed in this study. These data can be found here: https://www.ncbi.nlm.nih.gov/geo/query/acc.cgi?acc=GSE20292; https://www.ncbi.nlm.nih.gov/geo/query/acc.cgi?acc=GSE68719; https://www.ncbi.nlm.nih.gov/geo/query/acc.cgi?acc=GSE6613; https://www.ncbi.nlm.nih.gov/geo/query/acc.cgi?acc=GSE72267; https://www.ncbi.nlm.nih.gov/geo/query/acc.cgi?acc=GSE99039; https://www.ncbi.nlm.nih.gov/geo/query/acc.cgi?acc=GSE100054; https://www.ncbi.nlm.nih.gov/geo/query/acc.cgi?acc=GSE150696; and https://www.ncbi.nlm.nih.gov/geo/query/acccgi?acc=GSE199258.

## Code availability

The custom R code used to generate results that are reported in this study are available from the corresponding author upon reasonable request.
