# Supplementary material for: MAPKAPK2, a potential dynamic network biomarker of α-synuclein prior to its aggregation in PD patients
Source: NPJ Parkinsons Dis. 2023 Mar 16;9:41. doi: 10.1038/s41531-023-00479-z (PMC10020541; doi:10.1038/s41531-023-00479-z)
Supplement: Supplementary file 1 — SUPPLEMENTAL MATERIAL [file 41531_2023_479_MOESM1_ESM.pdf]

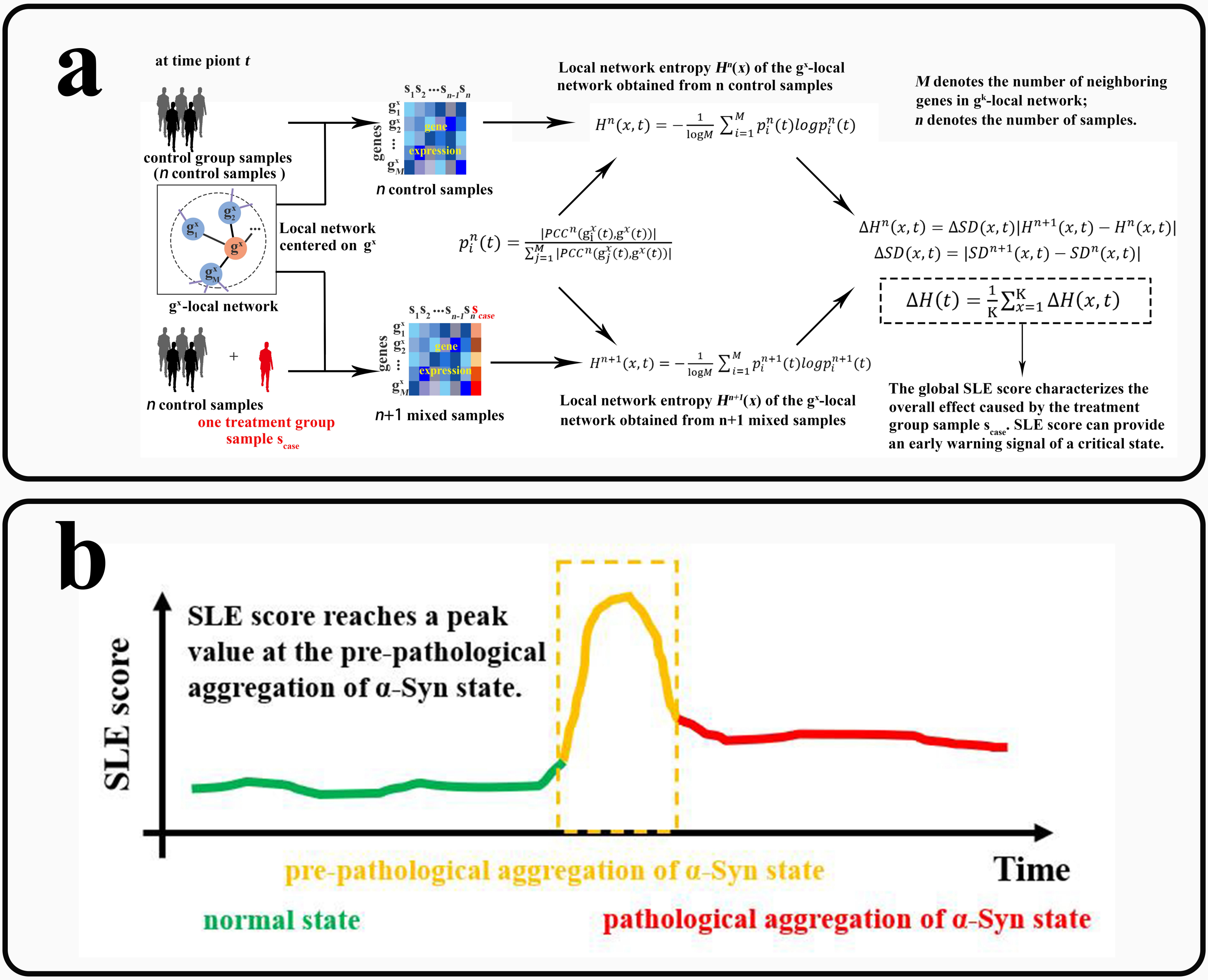
a
at time piont t
control group samples
(n control samples)
Local network centered on g^x
g^x-local network
n control samples
one treatment group sample s_{case}
genes
gene
expression
n control samples
n+1 mixed samples
Local network entropy $H^n(x)$ of the g^x-local network obtained from n control samples
$H^n(x,t) = -\frac{1}{\log M}\sum_{i=1}^{M} p_i^n(t)\log p_i^n(t)$
$p_i^n(t) = \frac{|PCC^n(g_i^x(t),g^x(t))|}{\sum_{j=1}^{M}|PCC^n(g_j^x(t),g^x(t))|}$
$H^{n+1}(x,t) = -\frac{1}{\log M}\sum_{i=1}^{M} p_i^{n+1}(t)\log p_i^{n+1}(t)$
Local network entropy $H^{n+1}(x)$ of the g^x-local network obtained from n+1 mixed samples
M denotes the number of neighboring genes in g^k-local network;
n denotes the number of samples.
$\Delta H^n(x,t) = \Delta SD(x,t)|H^{n+1}(x,t) - H^n(x,t)|$
$\Delta SD(x,t) = |SD^{n+1}(x,t) - SD^n(x,t)|$
$\Delta H(t) = \frac{1}{K}\sum_{x=1}^{K}\Delta H(x,t)$
The global SLE score characterizes the overall effect caused by the treatment group sample s_{case}. SLE score can provide an early warning signal of a critical state.
b
SLE score
SLE score reaches a peak value at the pre-pathological aggregation of α-Syn state.
Time
pre-pathological aggregation of α-Syn state
normal state
pathological aggregation of α-Syn state

Supplementary Figure 1 Details of the SLE algorithm and its application to the study of pre-pathological aggregation of α-Syn. (a) Single-sample landscape entropy (SLE) algorithm. (b) A spike of the SLE score curve exists in the pre- pathological aggregation of α-Syn state.

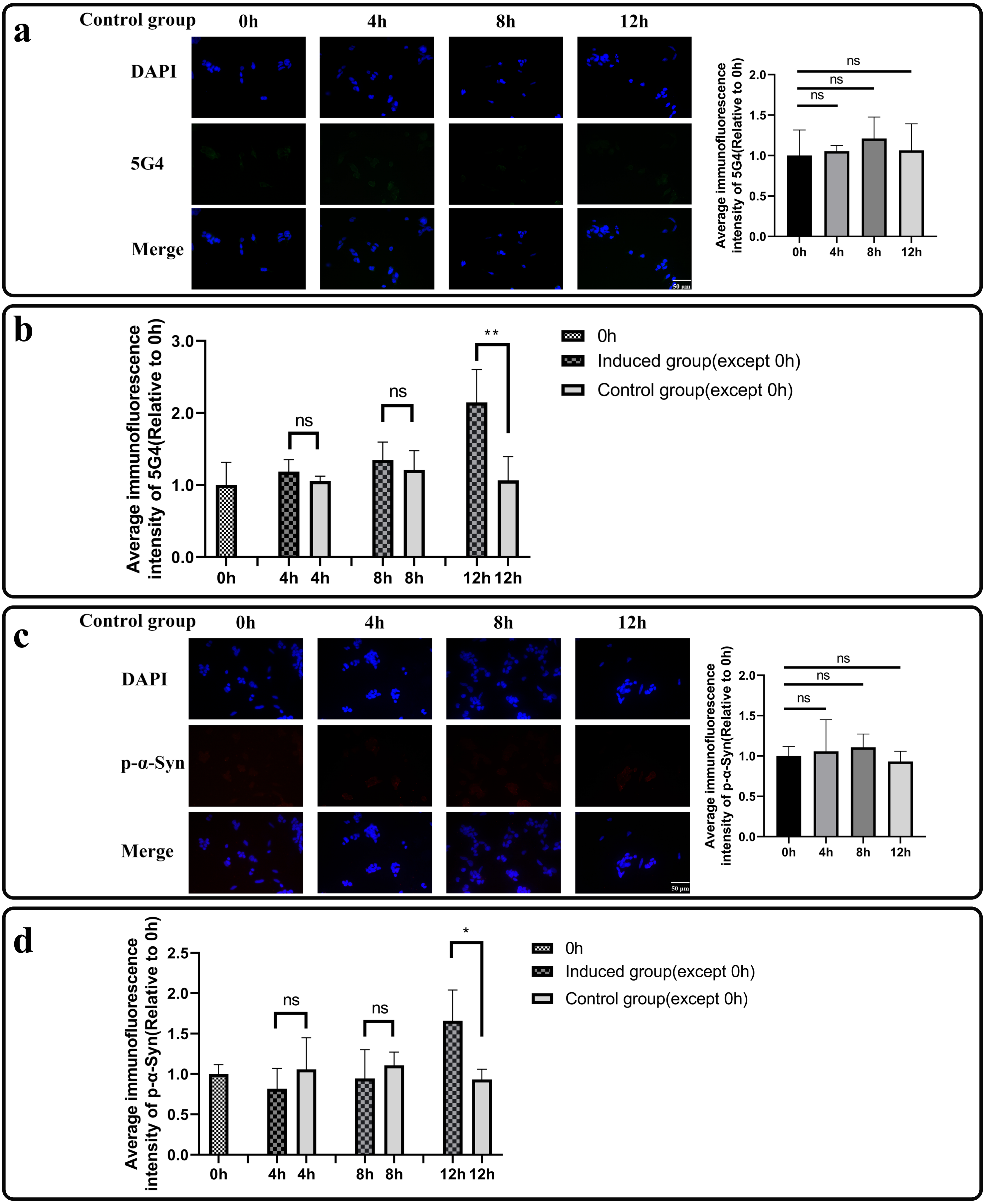
a
Control group
0h
4h
8h
12h
DAPI
5G4
Merge
50 μm
Average immunofluorescence intensity of 5G4(Relative to 0h)
ns
ns
ns
2.0
1.5
1.0
0.5
0.0
0h
4h
8h
12h
b
Average immunofluorescence intensity of 5G4(Relative to 0h)
3.0
2.0
1.0
0.0
ns
ns
**
0h
4h
4h
8h
8h
12h
12h
0h
Induced group(except 0h)
Control group(except 0h)
c
Control group
0h
4h
8h
12h
DAPI
p-α-Syn
Merge
50 μm
Average immunofluorescence intensity of p-α-Syn(Relative to 0h)
ns
ns
ns
2.0
1.5
1.0
0.5
0.0
0h
4h
8h
12h
d
Average immunofluorescence intensity of p-α-Syn(Relative to 0h)
2.5
2.0
1.5
1.0
0.5
0.0
ns
ns
*
0h
4h
4h
8h
8h
12h
12h
0h
Induced group(except 0h)
Control group(except 0h)

Supplementary Figure 2 Immunofluorescence experiments in the control group and comparative analysis of experimental results between the control and induction groups. (a) and (c) Results of immunofluorescence staining for 5G4 antibody (a) and p-α-Syn antibody (c) in the control group. Blue fluorescence represents 4′,6-diamidino-2-phenylindole (DAPI)-stained nuclei, green fluorescence represents pathological aggregation of α-syn, and red fluorescence represents p-α-Syn. Immunofluorescence experiments showed that pathological aggregation of α-Syn was consistently absent in the control group. n=4. (b) and (d) Analysis of the relative mean immunofluorescence intensities of the induction and control groups at the same time points in immunofluorescence experiments with 5G4 antibody (b) and p-α-Syn antibody (d). In both antibody experiments, there was only a significant difference between 12h in the induction group and 12h in the control group. ns: no significant difference. *: $p < 0.05$. Data are expressed as means ± SEMs.

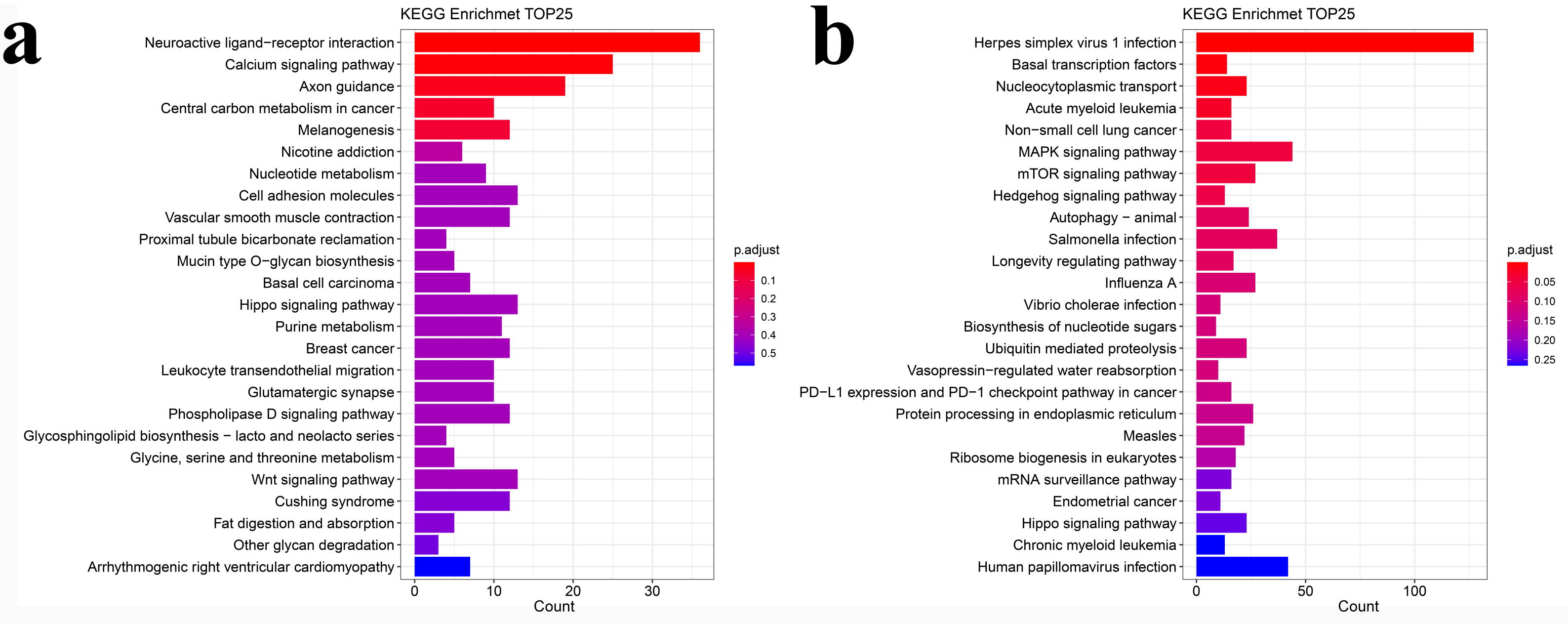
a
KEGG Enrichmet TOP25
Neuroactive ligand−receptor interaction
Calcium signaling pathway
Axon guidance
Central carbon metabolism in cancer
Melanogenesis
Nicotine addiction
Nucleotide metabolism
Cell adhesion molecules
Vascular smooth muscle contraction
Proximal tubule bicarbonate reclamation
Mucin type O−glycan biosynthesis
Basal cell carcinoma
Hippo signaling pathway
Purine metabolism
Breast cancer
Leukocyte transendothelial migration
Glutamatergic synapse
Phospholipase D signaling pathway
Glycosphingolipid biosynthesis − lacto and neolacto series
Glycine, serine and threonine metabolism
Wnt signaling pathway
Cushing syndrome
Fat digestion and absorption
Other glycan degradation
Arrhythmogenic right ventricular cardiomyopathy
0
10
20
30
Count
p.adjust
0.1
0.2
0.3
0.4
0.5
b
KEGG Enrichmet TOP25
Herpes simplex virus 1 infection
Basal transcription factors
Nucleocytoplasmic transport
Acute myeloid leukemia
Non−small cell lung cancer
MAPK signaling pathway
mTOR signaling pathway
Hedgehog signaling pathway
Autophagy − animal
Salmonella infection
Longevity regulating pathway
Influenza A
Vibrio cholerae infection
Biosynthesis of nucleotide sugars
Ubiquitin mediated proteolysis
Vasopressin−regulated water reabsorption
PD−L1 expression and PD−1 checkpoint pathway in cancer
Protein processing in endoplasmic reticulum
Measles
Ribosome biogenesis in eukaryotes
mRNA surveillance pathway
Endometrial cancer
Hippo signaling pathway
Chronic myeloid leukemia
Human papillomavirus infection
0
50
100
Count
p.adjust
0.05
0.10
0.15
0.20
0.25

Supplementary Figure 3 KEGG pathway enrichment analysis of genes in cluster2 and 3 (a) as well as cluster5 (b) in hierarchical clustering of DEGs.

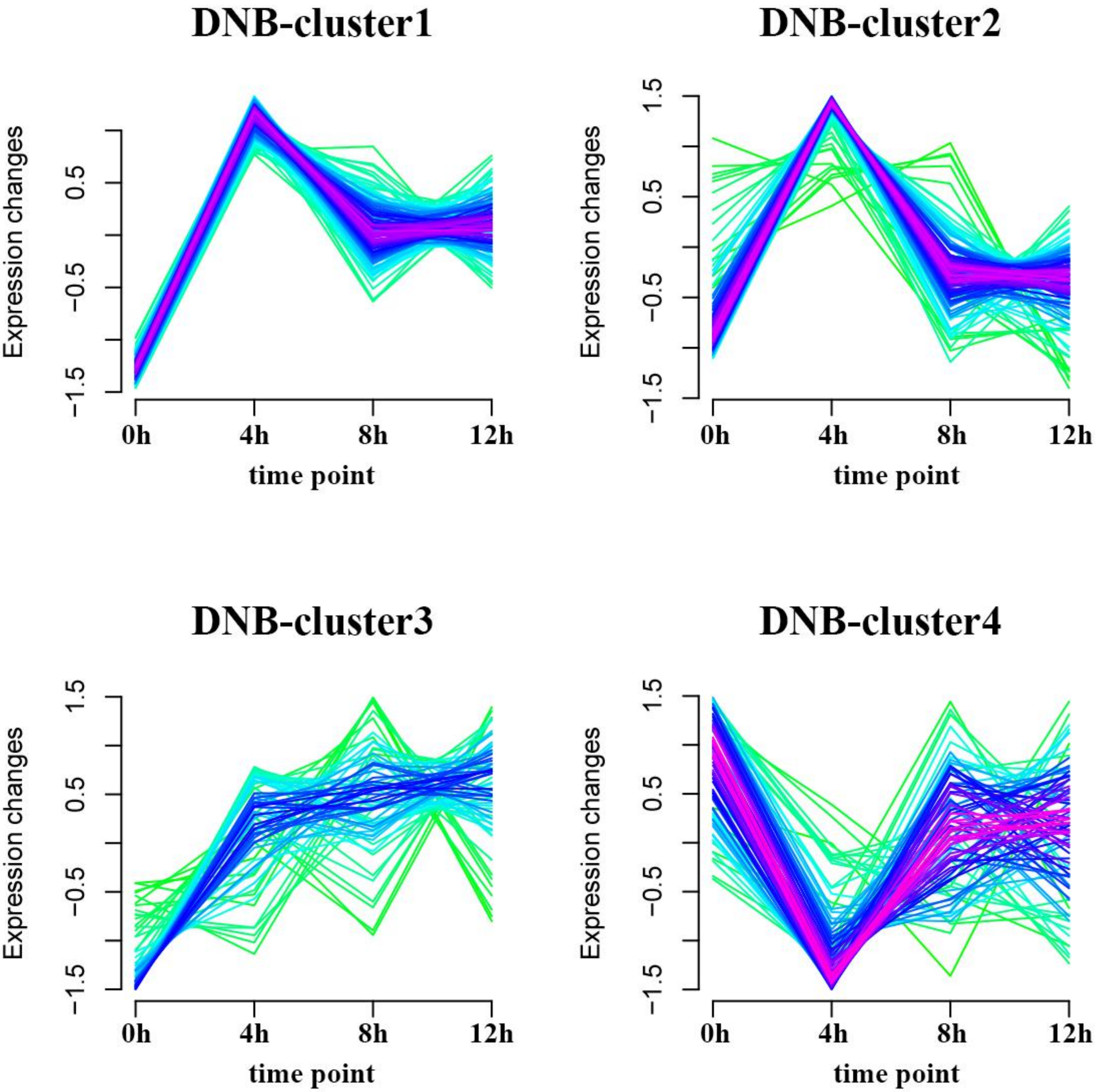
DNB-cluster1
DNB-cluster2
DNB-cluster3
DNB-cluster4
Expression changes
−1.5
−0.5
0.5
1.5
0h
4h
8h
12h
time point

Supplementary Figure 4 Soft clustering analysis of DNB genes showed that the DNB genes in cluster 1, 2 and 4 were expressed at the highest or lowest levels of all time points at 4h after induction.

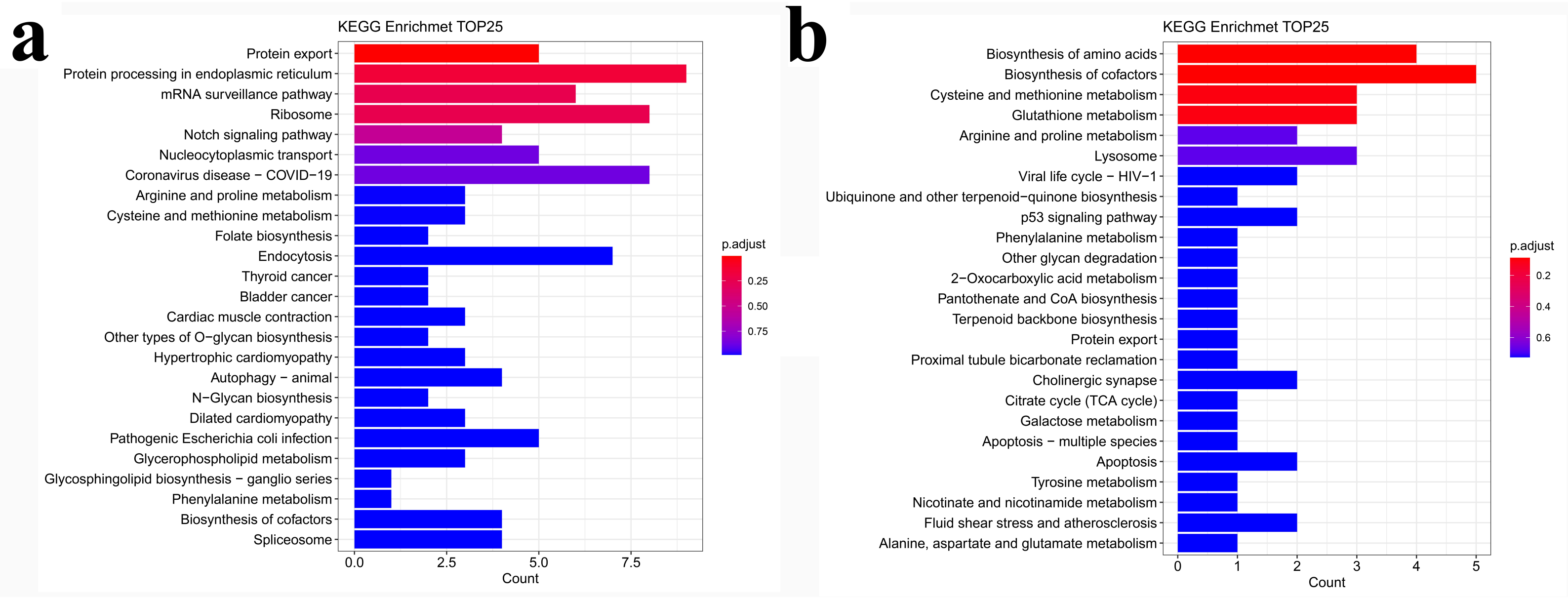
a
KEGG Enrichmet TOP25
Protein export
Protein processing in endoplasmic reticulum
mRNA surveillance pathway
Ribosome
Notch signaling pathway
Nucleocytoplasmic transport
Coronavirus disease − COVID−19
Arginine and proline metabolism
Cysteine and methionine metabolism
Folate biosynthesis
Endocytosis
Thyroid cancer
Bladder cancer
Cardiac muscle contraction
Other types of O−glycan biosynthesis
Hypertrophic cardiomyopathy
Autophagy − animal
N−Glycan biosynthesis
Dilated cardiomyopathy
Pathogenic Escherichia coli infection
Glycerophospholipid metabolism
Glycosphingolipid biosynthesis − ganglio series
Phenylalanine metabolism
Biosynthesis of cofactors
Spliceosome
0.0
2.5
5.0
7.5
Count
p.adjust
0.25
0.50
0.75
b
KEGG Enrichmet TOP25
Biosynthesis of amino acids
Biosynthesis of cofactors
Cysteine and methionine metabolism
Glutathione metabolism
Arginine and proline metabolism
Lysosome
Viral life cycle − HIV−1
Ubiquinone and other terpenoid−quinone biosynthesis
p53 signaling pathway
Phenylalanine metabolism
Other glycan degradation
2−Oxocarboxylic acid metabolism
Pantothenate and CoA biosynthesis
Terpenoid backbone biosynthesis
Protein export
Proximal tubule bicarbonate reclamation
Cholinergic synapse
Citrate cycle (TCA cycle)
Galactose metabolism
Apoptosis − multiple species
Apoptosis
Tyrosine metabolism
Nicotinate and nicotinamide metabolism
Fluid shear stress and atherosclerosis
Alanine, aspartate and glutamate metabolism
0
1
2
3
4
5
Count
p.adjust
0.2
0.4
0.6

Supplementary Figure 5 KEGG pathway enrichment analysis of genes in DNB-cluster 1 and DNB-cluster 2 (a) as well as DNB-cluster4 (b) in soft clustering of DNB genes.

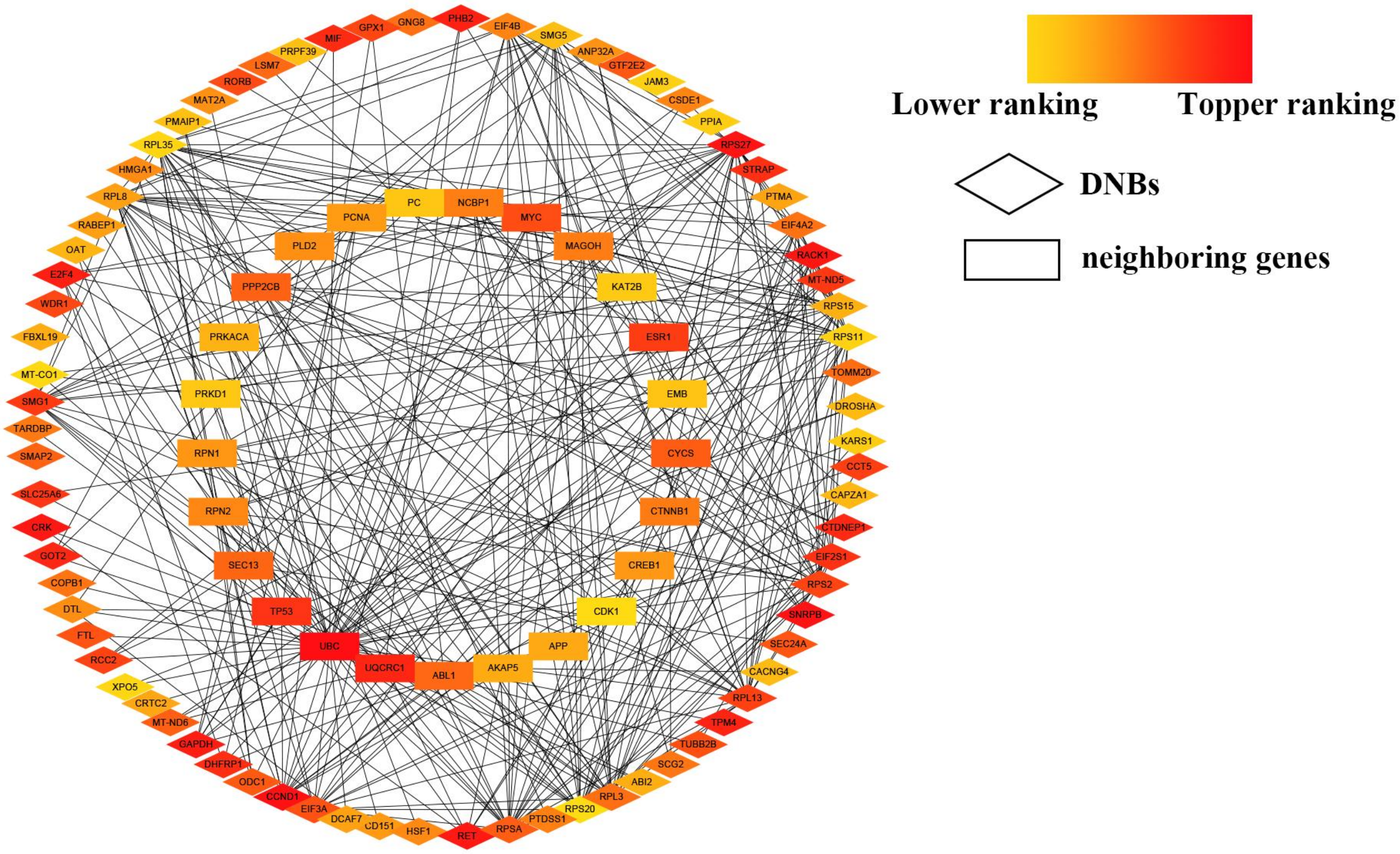
Lower ranking
Topper ranking
DNBs
neighboring genes
PHB2
GNG8
GPX1
MIF
PRPF39
LSM7
RORB
MAT2A
PMAIP1
RPL35
HMGA1
RPL8
RABEP1
OAT
E2F4
WDR1
FBXL19
MT-CO1
SMG1
TARDBP
SMAP2
SLC25A6
CRK
GOT2
COPB1
DTL
FTL
RCC2
XPO5
CRTC2
MT-ND6
GAPDH
DHFRP1
ODC1
CCND1
EIF3A
DCAF7
CD151
HSF1
RET
RPSA
PTDSS1
RPS20
RPL3
ABI2
SCG2
TUBB2B
TPM4
RPL13
CACNG4
SEC24A
SNRPB
RPS2
EIF2S1
CTDNEP1
CAPZA1
CCT5
KARS1
DROSHA
TOMM20
RPS11
RPS15
MT-ND5
RACK1
EIF4A2
PTMA
STRAP
RPS27
PPIA
CSDE1
JAM3
GTF2E2
ANP32A
SMG5
EIF4B
PC
NCBP1
PCNA
MYC
PLD2
MAGOH
PPP2CB
KAT2B
PRKACA
ESR1
PRKD1
EMB
RPN1
CYCS
RPN2
CTNNB1
SEC13
CREB1
TP53
CDK1
UBC
APP
UQCRC1
ABL1
AKAP5

Supplementary Figure 6 PPI analysis showed that genes scoring in the top 100 for topological analysis were at the core of the PPI network. Diamonds represent DNB genes and rectangles represent neighboring genes. The shades of color indicate high and low gene rank.

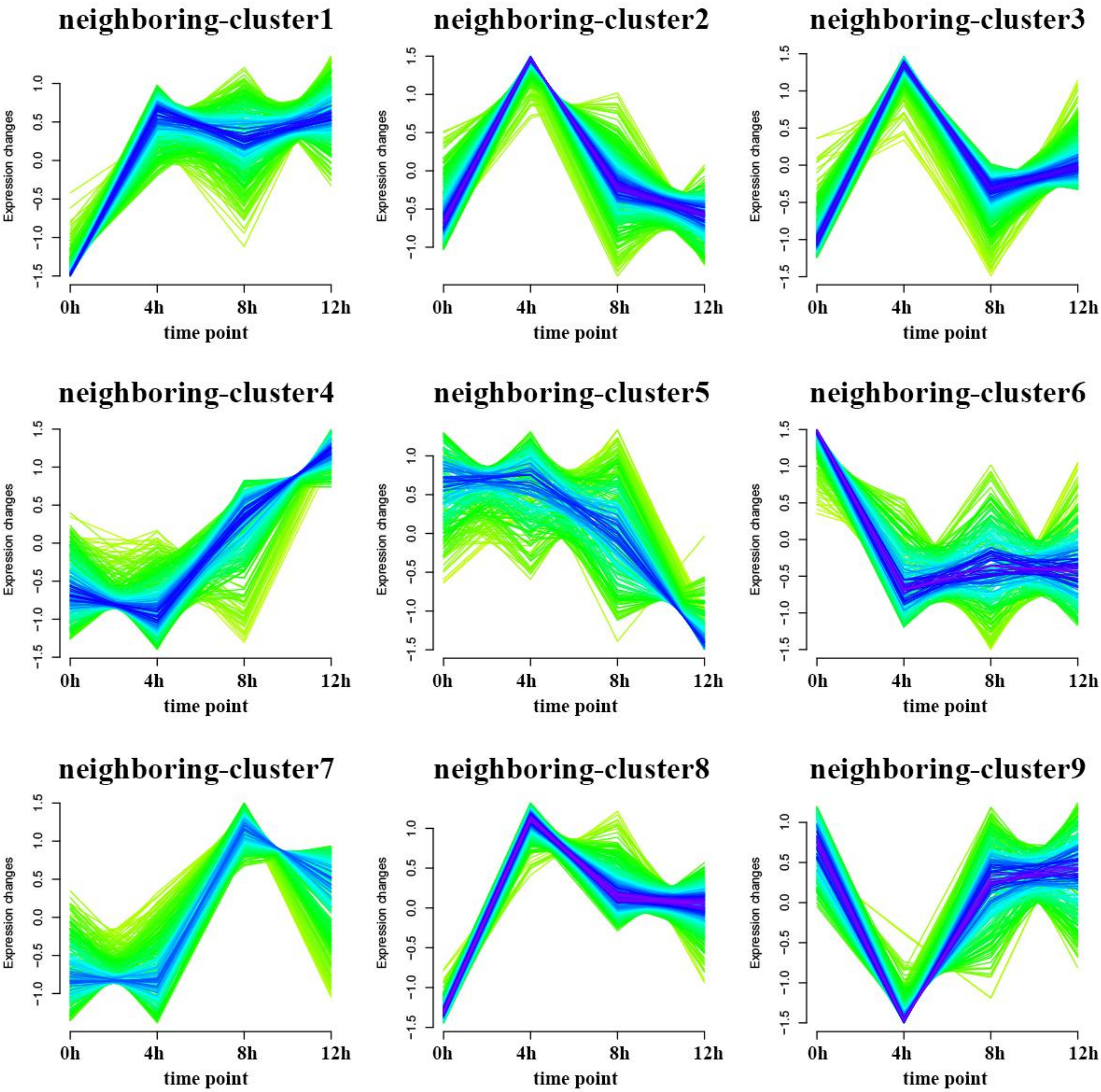

neighboring-cluster1
neighboring-cluster2
neighboring-cluster3
neighboring-cluster4
neighboring-cluster5
neighboring-cluster6
neighboring-cluster7
neighboring-cluster8
neighboring-cluster9
Expression changes
time point
0h
4h
8h
12h

Supplementary Figure 7 Soft clustering analysis of neighboring genes showed that the expression levels of neighboring genes in cluster 4, 5 and 7 did not change significantly between 0h and 4h, but changed sharply after 4h.

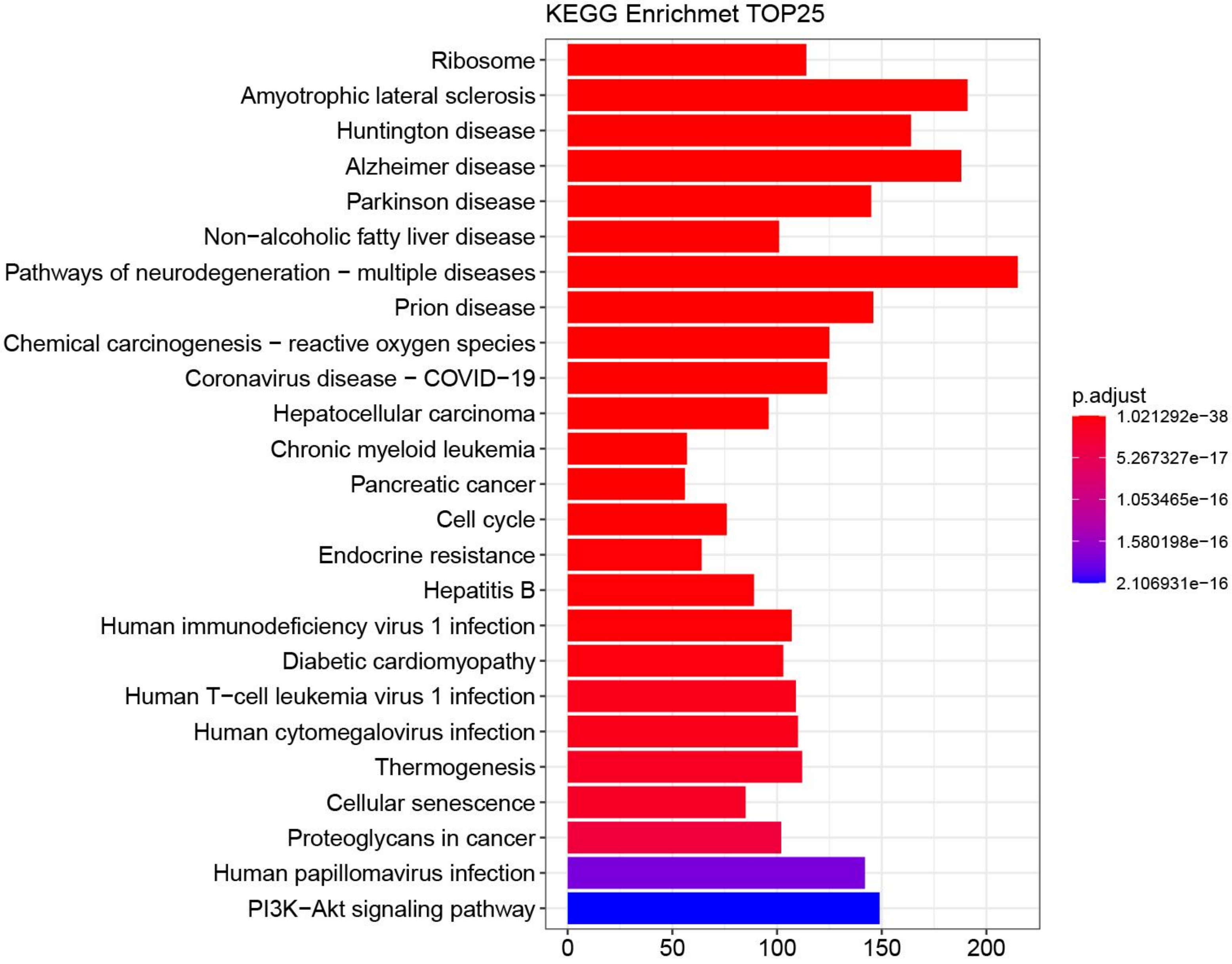

KEGG Enrichmet TOP25
Ribosome
Amyotrophic lateral sclerosis
Huntington disease
Alzheimer disease
Parkinson disease
Non−alcoholic fatty liver disease
Pathways of neurodegeneration − multiple diseases
Prion disease
Chemical carcinogenesis − reactive oxygen species
Coronavirus disease − COVID−19
Hepatocellular carcinoma
Chronic myeloid leukemia
Pancreatic cancer
Cell cycle
Endocrine resistance
Hepatitis B
Human immunodeficiency virus 1 infection
Diabetic cardiomyopathy
Human T−cell leukemia virus 1 infection
Human cytomegalovirus infection
Thermogenesis
Cellular senescence
Proteoglycans in cancer
Human papillomavirus infection
PI3K−Akt signaling pathway
0
50
100
150
200
p.adjust
1.021292e−38
5.267327e−17
1.053465e−16
1.580198e−16
2.106931e−16

Supplementary Figure 8 KEGG pathway enrichment analysis of DNB genes and neighboring genes.

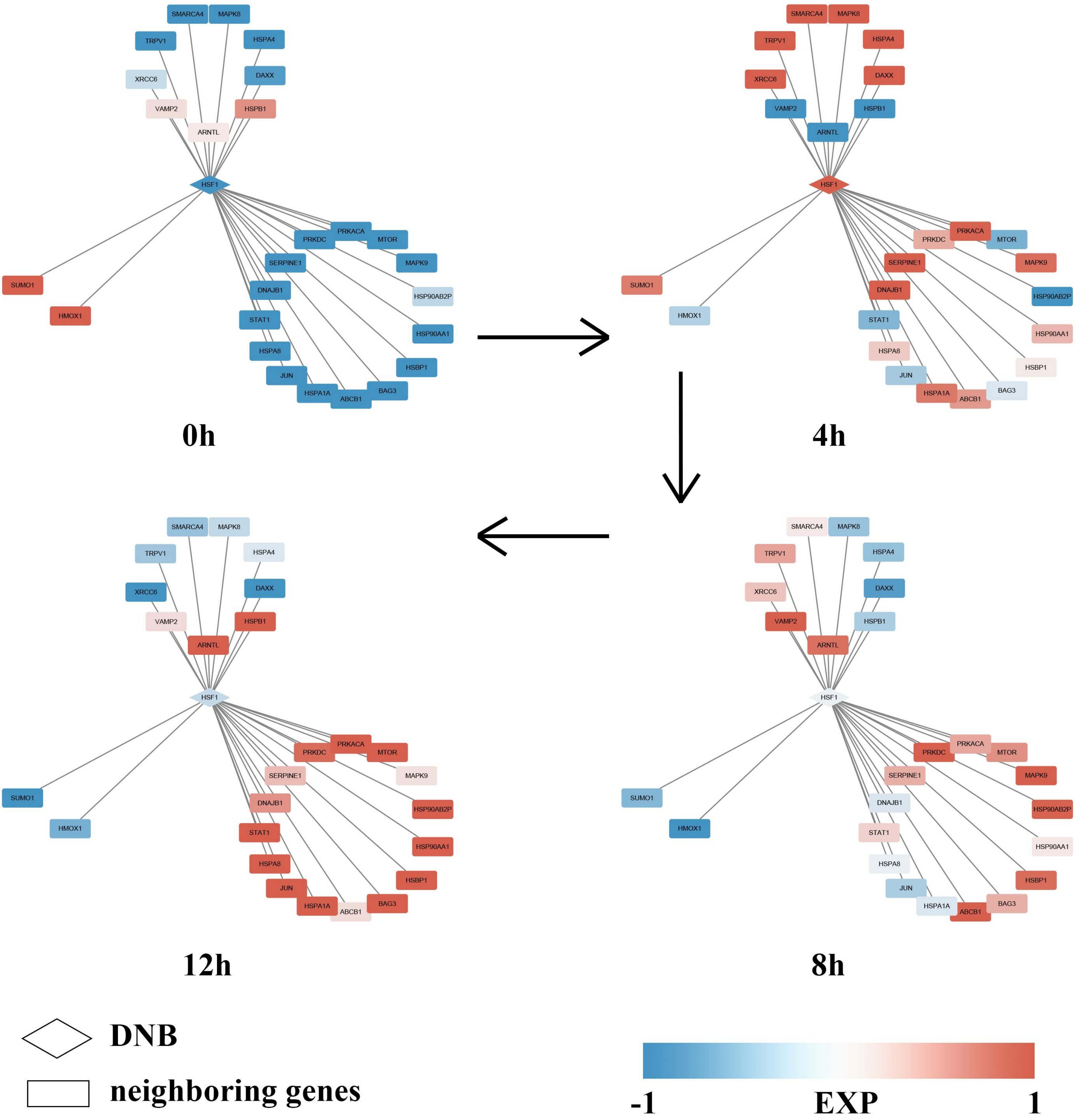

SMARCA4
MAPK8
TRPV1
HSPA4
XRCC6
DAXX
VAMP2
HSPB1
ARNTL
HSF1
PRKACA
PRKDC
MTOR
SERPINE1
MAPK9
SUMO1
DNAJB1
HSP90AB2P
HMOX1
STAT1
HSP90AA1
HSPA8
HSBP1
JUN
BAG3
HSPA1A
ABCB1
0h
4h
12h
8h
DNB
neighboring genes
-1
EXP
1

Supplementary Figure 9 Network graph of the changes in the expression levels of *HSF1* and its neighboring genes. One of the neighboring genes of DNB core gene *HSF1* was *SERPINE1*.

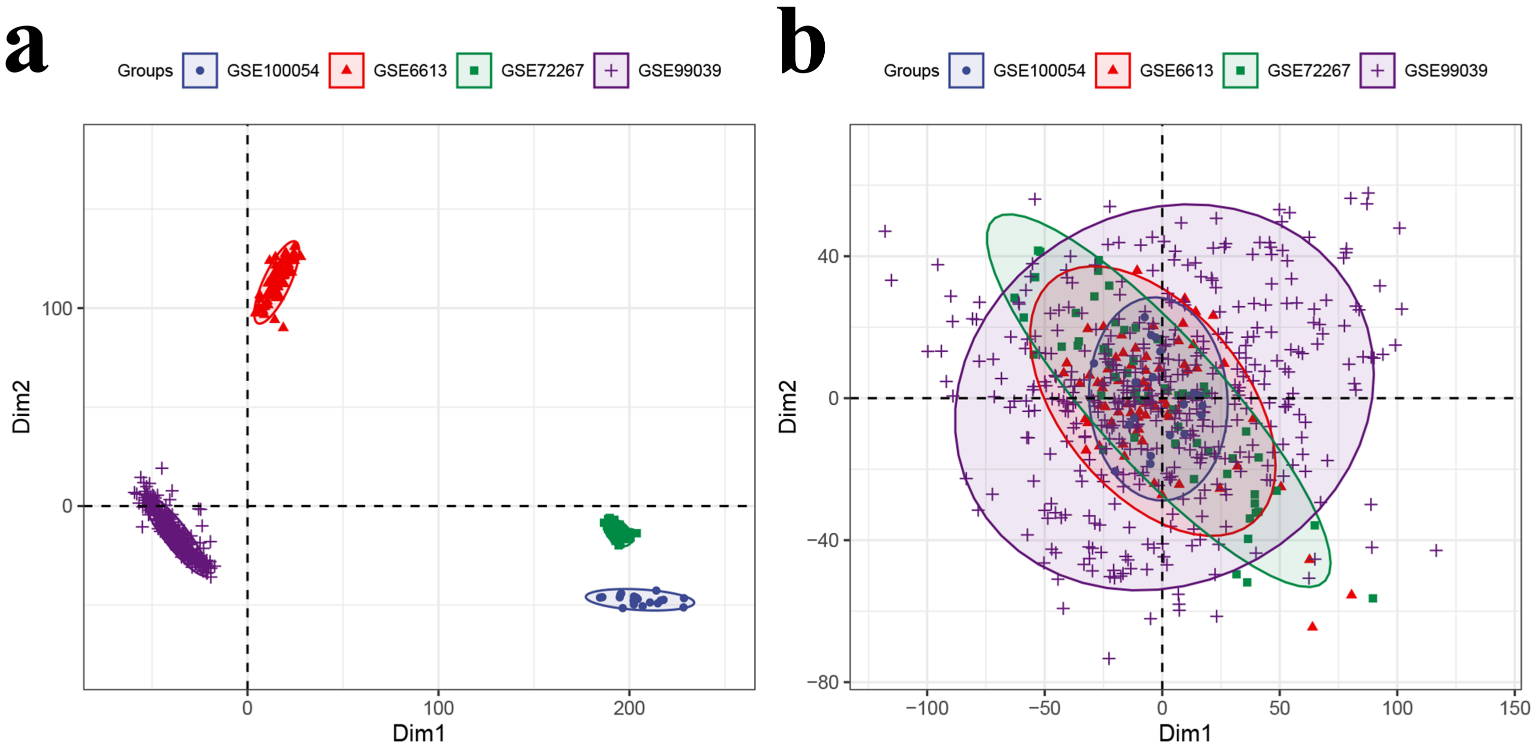
a
Groups GSE100054 GSE6613 GSE72267 GSE99039
Dim2
Dim1
100
0
0
100
200
b
Groups GSE100054 GSE6613 GSE72267 GSE99039
Dim2
Dim1
40
0
−40
−80
−100
−50
0
50
100
150

Supplementary Figure 10 Principal component analysis plots before (a) and after (b) removing batch effects.

a

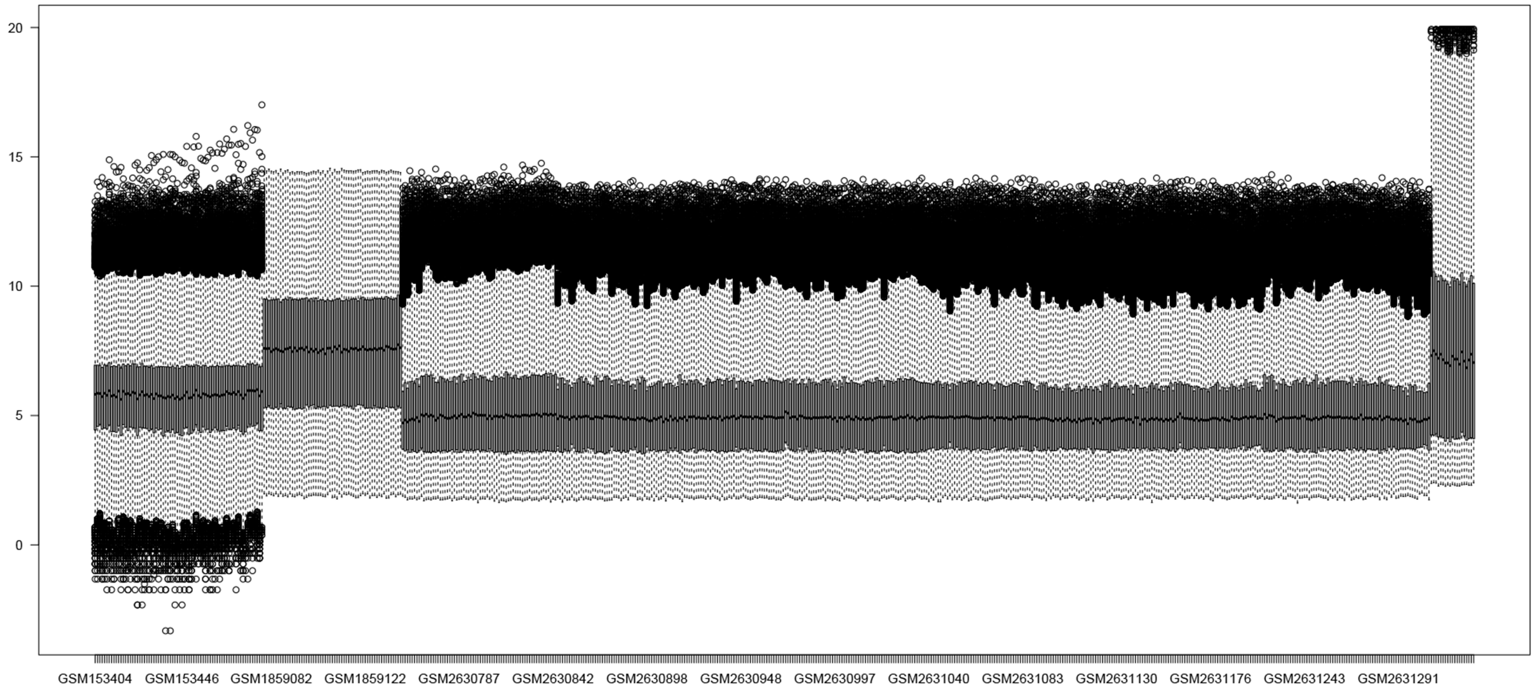
20
15
10
5
0
GSM153404
GSM153446
GSM1859082
GSM1859122
GSM2630787
GSM2630842
GSM2630898
GSM2630948
GSM2630997
GSM2631040
GSM2631083
GSM2631130
GSM2631176
GSM2631243
GSM2631291

b

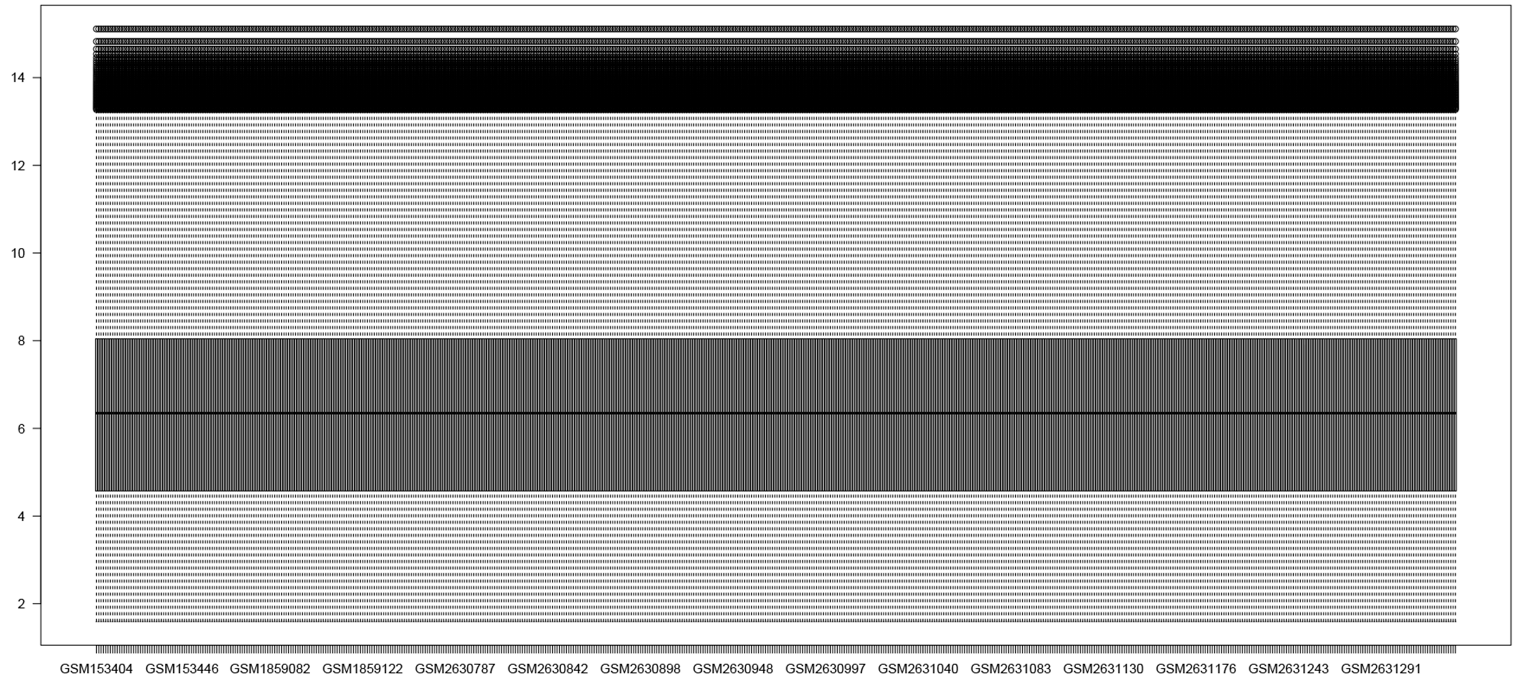
14
12
10
8
6
4
2
GSM153404
GSM153446
GSM1859082
GSM1859122
GSM2630787
GSM2630842
GSM2630898
GSM2630948
GSM2630997
GSM2631040
GSM2631083
GSM2631130
GSM2631176
GSM2631243
GSM2631291

Supplementary Figure 11 Boxplots before (a) and after (b) removing batch effects.

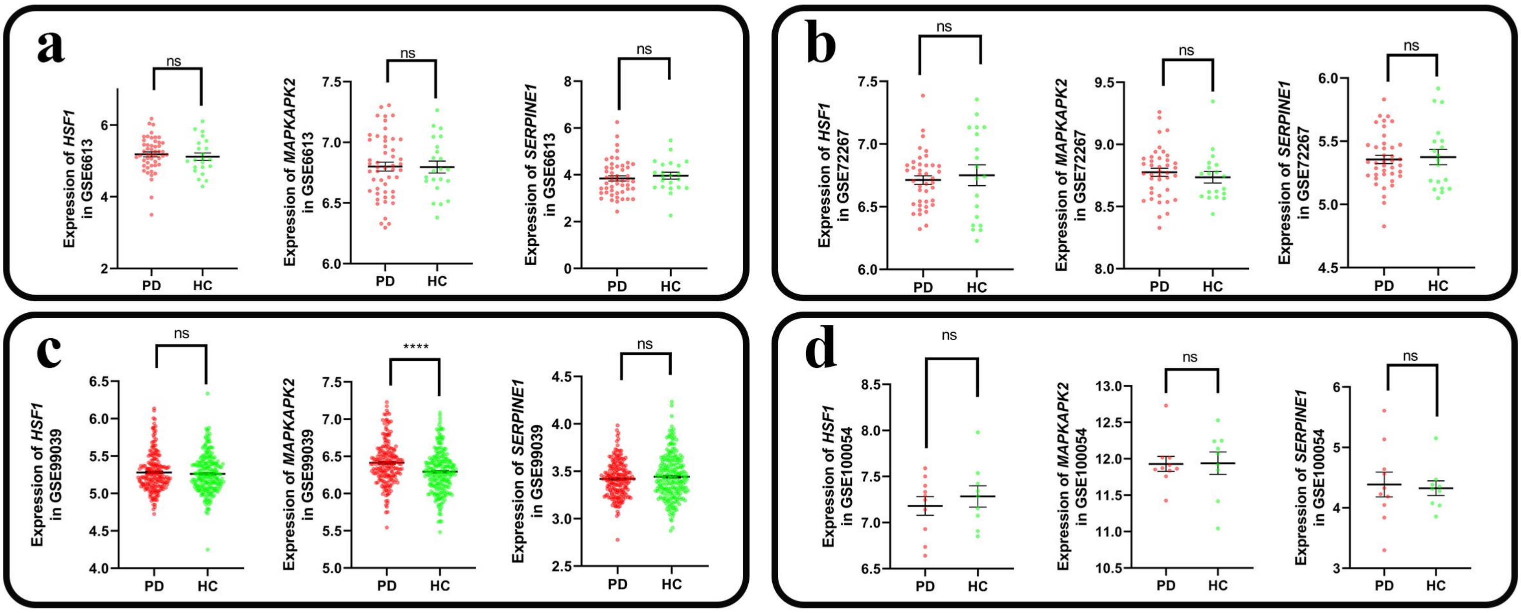
a
ns
Expression of HSF1 in GSE6613
PD
HC
ns
Expression of MAPKAPK2 in GSE6613
PD
HC
ns
Expression of SERPINE1 in GSE6613
PD
HC
b
ns
Expression of HSF1 in GSE72267
PD
HC
ns
Expression of MAPKAPK2 in GSE72267
PD
HC
ns
Expression of SERPINE1 in GSE72267
PD
HC
c
ns
Expression of HSF1 in GSE99039
PD
HC
****
Expression of MAPKAPK2 in GSE99039
PD
HC
ns
Expression of SERPINE1 in GSE99039
PD
HC
d
ns
Expression of HSF1 in GSE100054
PD
HC
ns
Expression of MAPKAPK2 in GSE100054
PD
HC
ns
Expression of SERPINE1 in GSE100054
PD
HC

Supplementary Figure 12 Expression levels of *HSF1*, *MAPKAPK2* and *SERPINE1* in PD patients and healthy controls in GSE6613 (a), GSE72267 (b), GSE99039 (c) and GSE100054 (d) obtained from peripheral blood. ns: no significant difference. ****: $p < 0.0001$. The data are expressed as the means ± SEMs. PD: PD patients. HC: healthy controls.

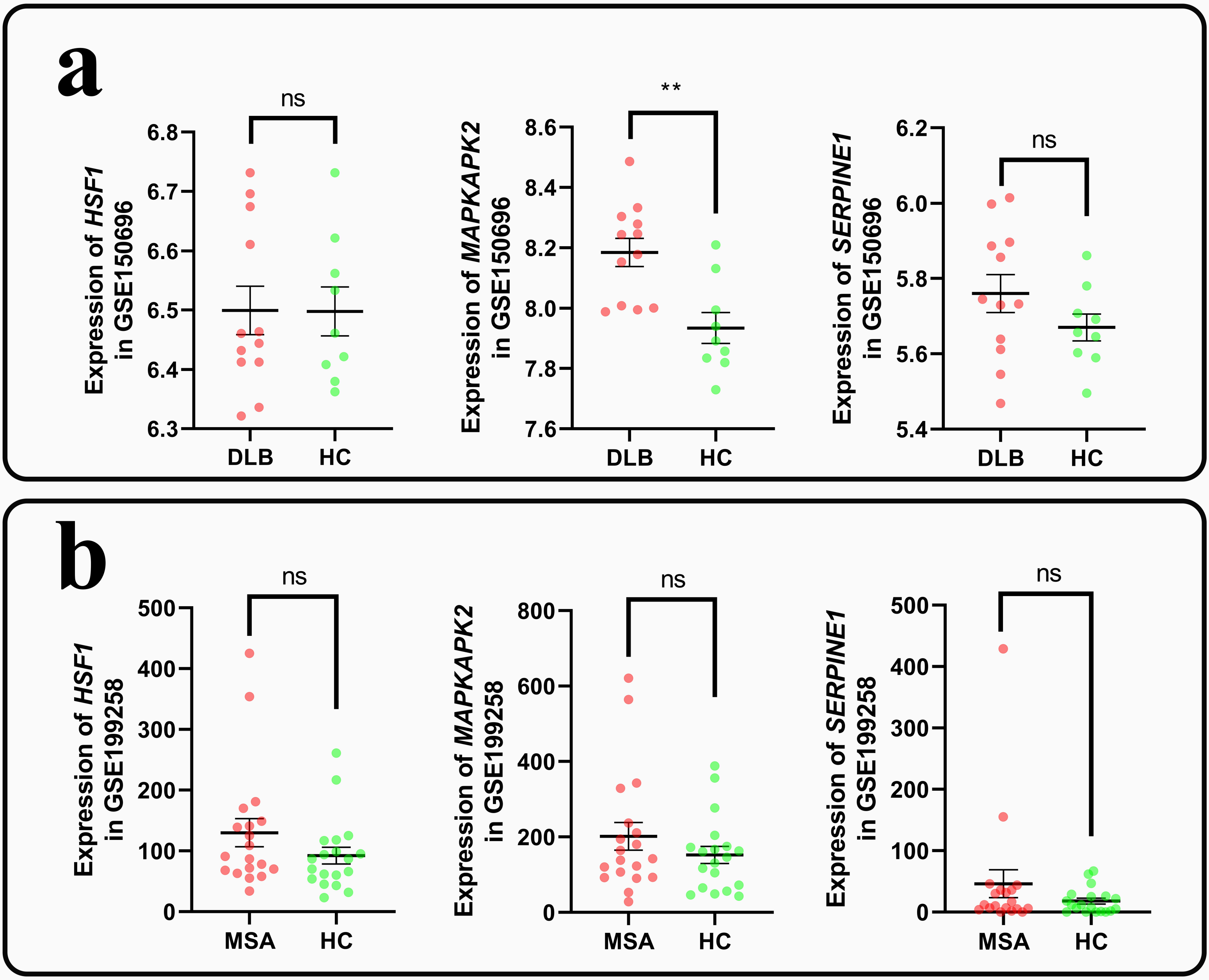
a
b
ns
**
Expression of HSF1 in GSE150696
Expression of MAPKAPK2 in GSE150696
Expression of SERPINE1 in GSE150696
DLB
HC
Expression of HSF1 in GSE199258
Expression of MAPKAPK2 in GSE199258
Expression of SERPINE1 in GSE199258
MSA
6.8
6.7
6.6
6.5
6.4
6.3
8.6
8.4
8.2
8.0
7.8
7.6
6.2
6.0
5.8
5.6
5.4
500
400
300
200
100
0
800
600

Supplementary Figure 12 Expression levels of *HSF1*, *MAPKAPK2* and *SERPINE1* in patients and healthy controls in DLB-related GEO data obtained from the prefrontal cortex (a) and in MSA-related GEO data obtained from cerebellar white matter (b). ns : no significant difference. **: $p < 0.01$. The data are expressed as the means ± SEMs. DLB: DLB patients. MSA: MSA patients. HC: healthy controls.

Supplementary Table 1. The list of differentially expressed genes.

GENE ID	GeneSymbol	(0-4)		(0-8)		(0-12)		(4-8)		(4-12)		(8-12)		hierarchical clustering	Is_DNB	(0-4)	(0-8)	(0-12)	(4-8)	(4-12)	(8-12)
		log2FoldChange	padj	log2FoldChange	padj	log2FoldChange	padj	log2FoldChange	padj	log2FoldChange	padj	log2FoldChange	padj								
ENSG00000000460	C1orf112	0.585148616	3.31E-13	0.304509029	0.001145	0.337448481	0.000238	-0.274236921	0.000939	-0.232956735	0.006615	0.035868981	0.860105	C1	-	Y	Y	Y	-	-	-
ENSG00000001167	NFYA	0.411023532	1.08E-09	0.257955177	0.004575	0.482078429	7.65E-13	-0.146344575	0.033335	0.085351534	0.140937	0.226539216	0.00412	C4	-	Y	-	Y	-	-	-
ENSG00000001460	STPG1	-0.342674955	0.018458	-0.136028297	0.499442	0.130730647	0.453822	0.21264843	0.168208	0.487756969	0.000111	0.270331731	0.121839	C4	-	Y	-	-	-	Y	-
ENSG00000001461	NIPAL3	-0.233374778	0.058433	0.083728401	0.634883	0.14207907	0.307779	0.323504711	0.007494	0.389964236	0.000481	0.061353728	0.796431	C4	-	-	-	-	Y	Y	-
ENSG00000001617	SEMA3F	-0.889062161	0.000292	-0.512912079	0.085975	-0.209394972	0.532132	0.381289323	0.223705	0.693937261	0.01074	0.306590735	0.503233	C4	-	Y	-	-	-	Y	-
ENSG00000001629	ANKIB1	0.44145837	4.79E-16	0.306941517	1.62E-06	0.309685711	5.57E-07	-0.128100623	0.007827	-0.117060862	0.013934	0.005621787	0.969121	C1	-	Y	Y	Y	-	-	-
ENSG00000001631	KRIT1	0.268526781	0.009835	-0.111655885	0.388301	-0.026651055	0.859694	-0.373984974	0.000151	-0.280786049	0.004189	0.087819591	0.612037	C5	Y	-	-	-	Y	-	-
ENSG00000002016	RAD52	0.61498541	1.39E-05	0.198670707	0.399221	0.736332542	4.54E-07	-0.409878183	0.003444	0.136090159	0.312307	0.540508842	0.000799	C1	-	Y	-	Y	Y	-	Y
ENSG00000003147	ICA1	-0.601766044	4.13E-32	-0.407183843	1.40E-12	-0.463862554	5.99E-17	0.20145453	6.95E-06	0.15274142	0.000798	-0.05404444	0.522024	C4	-	Y	Y	Y	-	-	-
ENSG00000003393	ALS2	0.319629945	0.000101	0.069360328	0.617346	0.196187284	0.030899	-0.243537005	0.001118	-0.108787153	0.123812	0.129269111	0.232391	C5	-	Y	-	-	-	-	-
ENSG00000003402	CFLAR	-0.449591961	0.001107	-0.259185027	0.092805	-0.183830124	0.25214	0.197032147	0.220213	0.280208212	0.064165	0.078279788	0.777627	C4	-	Y	-	-	-	-	-
ENSG00000003509	NDUFAF7	0.53959151	5.73E-15	0.204617927	0.020392	0.217365141	0.009843	-0.328776176	6.68E-07	-0.307480656	3.51E-06	0.015855766	0.934123	C5	-	Y	-	-	Y	Y	-
ENSG00000003987	MTMR7	-0.331728365	0.024945	-0.214280459	0.20573	0.0186096	0.931707	0.12359791	0.459412	0.364362538	0.004883	0.236542992	0.168713	C4	-	Y	-	-	-	Y	-
ENSG00000003989	SLC7A2	-0.339459893	0.032878	0.00130342	0.996961	-0.052868977	0.800268	0.348354244	0.06814	0.30101565	0.056298	-0.050808985	0.90074	C4	-	Y	-	-	-	-	-
ENSG00000004139	SARM1	0.32909611	1.84E-06	0.179015232	0.048277	0.120008633	0.148958	-0.143770712	0.085632	-0.194390412	0.006792	-0.056116183	0.716749	C5	-	Y	-	-	-	-	-
ENSG00000004766	VPS50	0.598031565	6.82E-19	0.095814427	0.38746	0.16754124	0.075652	-0.496001143	2.45E-17	-0.415639143	2.42E-12	0.074818152	0.562859	C5	-	Y	-	-	Y	Y	-
ENSG00000004799	PDK4	-0.599093794	0.265382	0.422531488	0.495177	0.465410644	0.365969	1.026279426	0.040533	1.078299359	0.018828	0.045032685	NA	C2	-	-	-	-	Y	Y	-
ENSG00000004864	SLC25A13	-0.3785087	1.09E-17	-0.110473124	0.042583	-0.121731423	0.021897	0.27450524	3.47E-10	0.271495673	4.21E-10	-0.008318724	0.946273	C4	-	Y	-	-	-	-	-
ENSG00000004948	CALCR	2.959045792	0.032851	0.979670607	NA	0.723188687	NA	-1.956619629	0.127093	-2.217124848	0.101015	-0.253868926	NA	C5	-	Y	-	-	-	-	-
ENSG00000004975	DVL2	0.304769387	1.11E-05	0.200420095	0.012374	0.26583626	0.000978	-0.097927665	0.234759	-0.024289781	0.819126	0.068374175	0.641855	C1	-	Y	-	-	-	-	-
ENSG00000005108	THSD7A	-0.449855375	9.00E-07	0.196027102	0.162362	0.210883638	0.051318	0.652135734	1.19E-15	0.675330002	4.34E-30	0.017853495	0.942494	C4	-	Y	-	-	Y	Y	-
ENSG00000005156	LIG3	0.428378626	6.36E-09	0.165551336	0.122348	0.164829755	0.108248	-0.256030345	0.000148	-0.249416085	0.000184	0.001916441	0.99358	C5	-	Y	-	-	-	-	-
ENSG00000005175	RPAP3	0.407109034	7.33E-11	0.062386074	0.556337	0.079357743	0.396236	-0.338084886	5.77E-09	-0.313142587	5.11E-08	0.019659131	0.905147	C5	-	Y	-	-	Y	Y	-
ENSG00000005189	REXO5	-0.514532801	2.67E-08	-0.080548141	0.64769	-0.159984071	0.149859	0.440602911	0.000122	0.369302587	0.000128	-0.076462756	0.739147	C4	-	Y	-	-	Y	Y	-
ENSG00000005206	SPPL2B	-0.226444213	0.01187	0.141037831	0.141675	0.018089663	0.887384	0.373655136	1.06E-05	0.259380518	0.002742	-0.120071723	0.32736	C4	-	-	-	-	Y	-	-
ENSG00000005379	TSPOAP1	-0.607157647	2.00E-10	-0.231694645	0.054198	-0.063831032	0.612681	0.381631662	0.000462	0.557949719	9.92E-10	0.170421077	0.241546	C4	-	Y	-	-	Y	Y	-
ENSG00000005436	GCFC2	-0.429796337	0.000566	-0.096636998	0.596138	-0.016221483	0.933967	0.339372779	0.009201	0.428249219	0.000345	0.082811094	0.715621	C4	-	Y	-	-	Y	Y	-
ENSG00000005448	WDR54	-0.236356102	0.008218	0.099269633	0.38746	0.064439617	0.614328	0.342331905	8.07E-06	0.315640799	9.38E-05	-0.031835266	0.866037	C4	-	-	-	-	Y	Y	-
ENSG00000005801	ZNF195	-0.413674	4.29E-16	-0.273607157	0.000117	-0.280652756	2.89E-06	0.146728803	0.021896	0.147853417	0.007278	-0.004238587	0.982152	C4	-	Y	-	-	-	-	-
ENSG00000005810	MYCBP2	0.374445419	0.000423	0.336211261	0.001411	0.35460112	0.001074	-0.03208691	0.789293	-0.005524757	0.977267	0.021390753	0.924306	C1	-	Y	Y	Y	-	-	-
ENSG00000005884	ITGA3	0.33563057	0.008974	0.477816951	0.000431	0.585992005	1.11E-06	0.14884781	0.313803	0.265296105	0.032445	0.111433046	0.610411	C4	-	Y	Y	Y	-	-	-
ENSG00000005961	ITGA2B	-1.106638059	3.01E-05	-0.752035886	0.018364	-0.322670419	0.400682	0.361769705	0.316051	0.798872097	0.012544	0.430064128	NA	C4	-	Y	Y	-	-	Y	-
ENSG00000006015	REX1BD	-0.471913534	1.05E-05	-0.168695576	0.224378	-0.20220215	0.098077	0.309890165	0.005844	0.284779394	0.009146	-0.02997558	0.910067	C4	-	Y	-	-	Y	-	-
ENSG00000006016	CRLF1	0.565601729	0.013401	0.752474623	0.005252	0.572717694	0.019392	0.193363391	0.495422	0.021775856	0.944369	-0.177751295	0.686328	C1	-	Y	Y	Y	-	-	-
ENSG00000006025	OSBPL7	0.489548665	4.40E-10	0.248435699	0.007731	0.306178442	0.000228	-0.234794302	0.002844	-0.168833925	0.032797	0.060488619	0.680501	C1	-	Y	-	Y	-	-	-
ENSG00000006047	YBX2	-0.558598166	1.79E-09	-0.155214896	0.167484	0.001472945	0.9925	0.409810373	1.20E-05	0.574890276	1.80E-11	0.159494717	0.176759	C4	-	Y	-	-	Y	Y	-
ENSG00000006071	ABCC8	-0.957572543	0.000166	-0.60363587	0.030109	-0.7136532	0.006752	0.358783166	0.282101	0.258106679	0.465769	-0.106859253	NA	C4	-	Y	Y	Y	-	-	-
ENSG00000006210	CX3CL1	-0.327901354	0.000946	0.026965641	0.87984	-0.063017662	0.7004	0.361369863	0.000162	0.279479508	0.009526	-0.087105724	0.670867	C4	-	Y	-	-	Y	-	-
ENSG00000006282	SPATA20	-0.388482833	4.32E-07	-0.096437499	0.416501	-0.175279166	0.044311	0.298616581	0.000366	0.228118389	0.003018	-0.07566139	0.611606	C4	-	Y	-	-	-	-	-
ENSG00000006283	CACNA1G	-1.22430184	2.00E-11	-0.314665307	0.106031	-0.236765755	0.237889	0.916536214	4.25E-06	1.001776186	4.76E-07	0.08051164	0.836208	C2	-	Y	-	-	Y	Y	-
ENSG00000006327	TNFRSF12A	1.886974367	4.20E-29	1.241122769	1.05E-07	1.537062822	8.40E-17	-0.641720487	0.000263	-0.33499101	0.030086	0.301060541	0.302106	C1	Y	Y	Y	Y	Y	Y	-
ENSG00000006377	DLX6	-0.662355067	8.58E-07	-0.290823909	0.046352	-0.348705283	0.017203	0.378470309	0.005246	0.328702439	0.02404	-0.054938855	0.849728	C2	-	Y	-	Y	Y	Y	-
ENSG00000006432	MAP3K9	0.632031282	3.30E-10	0.310773127	0.013608	0.51278803	2.84E-06	-0.315908143	0.000146	-0.104647999	0.269632	0.205743927	0.085831	C1	-	Y	Y	Y	Y	-	-
ENSG00000006453	BAIAP2L1	-0.354069014	0.004189	-0.048359704	0.806916	-0.172484227	0.225941	0.312147628	0.013205	0.196216423	0.146936	-0.121104973	0.553292	C4	-	Y	-	-	Y	-	-
ENSG00000006459	KDM7A	0.635465706	1.39E-16	0.152841855	0.167042	0.23002237	0.016339	-0.476605722	3.49E-15	-0.390789955	5.26E-11	0.080300504	0.52139	C5	-	Y	-	-	Y	Y	-
ENSG00000006468	ETV1	-1.160436654	2.16E-17	-0.108856215	0.575324	-0.033629844	0.894487	1.058467429	7.51E-14	1.141351423	2.97E-13	0.07741632	0.823297	C3	-	Y	-	-	Y	Y	-
ENSG00000006576	PHTF2	0.46383702	4.06E-15	0.181909253	0.015819	0.089100303	0.28261	-0.275565773	1.49E-06	-0.360115287	1.55E-11	-0.089850299	0.377059	C5	-	Y	-	-	-	Y	-
ENSG00000006695	COX10	0.318307531	0.002575	0.175705337	0.173911	0.024412385	0.897011	-0.136134151	0.233393	-0.27905942	0.011397	-0.148375373	0.39019	C5	-	Y	-	-	-	-	-
ENSG00000006704	GTF2IRD1	0.403263987	3.36E-09	0.181725773	0.015482	0.052159201	0.638246	-0.214990658	0.001016	-0.336487325	1.02E-06	-0.126787251	0.223973	C5	-	Y	-	-	-	Y	-
ENSG00000006756	ARSD	-0.469271581	0.000157	0.00105288	0.996745	-0.198344814	0.172572	0.476512148	7.70E-05	0.285470683	0.029783	-0.196306587	0.262262	C4	-	Y	-	-	Y	-	-
ENSG00000006757	PNPLA4	-1.070915635	0.000368	-0.544547581	0.126073	-0.710768415	0.024323	0.533368682	0.168919	0.373962095	0.35983	-0.164220445	NA	C3	-	Y	-	Y	-	-	-
ENSG00000007047	MARK4	0.324239586	3.23E-06	0.097078493	0.311782	0.14944207	0.10265	-0.22070579	0.002278	-0.159909341	0.04608	0.055180307	0.724653	C5	Y	Y	-	-	-	-	-
ENSG00000007171	NOS2	-1.154616205	8.23E-05	-0.465048428	0.163689	-0.650884242	0.029765	0.698015151	0.052566	0.518160736	0.177368	-0.183664632	NA	C3	-	Y	-	Y	-	-	-
ENSG00000007237	GAS7	-0.362672477	0.045666	-0.032361496	0.91022	0.006317925	0.981635	0.338064627	0.094194	0.383658757	0.044754	0.041324253	0.927911	C4	-	Y	-	-	-	Y	-
ENSG00000007255	TRAPPC6A	-0.456970675	0.005053	-0.41854871	0.018702	-0.524855062	0.007005	0.045918908	0.858016	-0.053040955	0.853506	-0.105106421	0.806285	C4	-	Y	Y	Y	-	-	-
ENSG00000007376	RPUSD1	0.362262769	0.006506	0.143257129	0.471621	0.068782261	0.732153	-0.212635231	0.066049	-0.278529108	0.006797	-0.071301323	0.755306	C5	-	Y	-	-	-	-	-
ENSG00000007392	LUC7L	0.419758673	9.52E-24	0.048480915	0.52222	0.080153375	0.188518	-0.364671352	4.10E-19	-0.324834752	1.24E-16	0.034484901	0.7275	C5	Y	Y	-	-	Y	Y	-
ENSG00000007402	CACNA2D2	-0.701320744	1.71E-48	-0.252895735	1.17E-06	-0.117454235	0.052661	0.454854107	7.68E-23	0.598465954	3.23E-38	0.13829022	0.030215	C4	-	Y	-	-	Y	Y	-
ENSG00000007541	PIGQ	-0.377827288	3.26E-07	-0.026596207	0.846885	-0.012816534	0.916162	0.357696008	9.59E-06	0.379838663	2.13E-07	0.016698911	0.936471	C4	-	Y	-	-	Y	Y	-
ENSG00000007944	MYLIP	-0.445166604	0.018313	0.045143153	0.888768	-0.113666697	0.693136	0.496918444	0.019808	0.345870902	0.148983	-0.156198454	0.714139	C2	-	Y	-	-	Y	-	-
ENSG00000007968	E2F2	-0.512017289	3.68E-17	-0.399094401	4.73E-07	-0.529809041	1.02E-11	0.119375039	0.172512	-0.003221974	0.97744	-0.126925959	0.407851	C4	-	Y	Y	Y	-	-	-
ENSG00000008128	CDK11A	0.281908658	0.003262	-0.030557005	0.864763	-0.188223226	0.170697	-0.305800237	0.001048	-0.45510086	6.26E-06	-0.15462352	0.393067	C5	-	-	-	-	Y	Y	-
ENSG00000008277	ADAM22	-0.139641671	0.057433	0.173841359	0.055593	0.212107914	0.003391	0.319777743	1.44E-08	0.366226295	7.74E-18	0.041257037	0.732055	C4	-	-	-	-	Y	Y	-

ENSG00000008283	CYB561	-0.439975244	5.73E-24	-0.249628982	1.22E-07	-0.329806513	9.10E-12	0.197093095	2.31E-08	0.124932486	0.001276	-0.077375913	0.168713	C4	-	Y	-	Y	-	-	-
ENSG00000008382	MPND	-0.493846044	2.97E-06	-0.157914357	0.275702	-0.183058144	0.16217	0.342447215	0.001382	0.325638992	0.002317	-0.022223093	0.937456	C4	-	Y	-	-	Y	Y	-
ENSG00000008405	CRY1	0.651542603	1.23E-13	0.029662744	0.882309	0.072997171	0.603298	-0.615204632	3.01E-10	-0.563727901	4.66E-11	0.045914248	0.860105	C5	-	Y	-	-	Y	Y	-
ENSG00000008438	PGLYRP1	1.276270844	0.130038	1.59464032	NA	1.716323164	0.033622	0.32459936	0.700909	0.453512217	0.565215	0.1227893	NA	C1	Y	-	-	Y	-	-	-
ENSG00000008441	NFIX	-0.419297015	9.29E-07	-0.078102136	0.549224	-0.09633623	0.381611	0.347533352	4.16E-05	0.337432101	2.63E-05	-0.015207672	0.944732	C4	-	Y	-	-	Y	Y	-
ENSG00000008710	PKD1	0.386857699	1.88E-11	0.193091153	0.005728	0.190863187	0.001725	-0.187233215	0.000182	-0.181619024	3.72E-05	0.000249294	0.998172	C5	-	Y	-	-	-	-	-
ENSG00000008853	RHOBTB2	0.300166384	3.17E-05	0.191426004	0.026967	0.147196614	0.085921	-0.102304647	0.205147	-0.138232322	0.066974	-0.041498643	0.785782	C5	-	Y	-	-	-	-	-
ENSG00000008869	HEATR5B	0.642947144	5.01E-24	0.358939718	2.43E-05	0.395438495	6.65E-09	-0.277867877	1.61E-05	-0.232898723	7.00E-06	0.039645318	0.785917	C5	-	Y	Y	Y	-	-	-
ENSG00000009724	MASP2	-0.211293356	0.434734	0.529487559	0.016075	0.054801817	0.897011	0.747168699	0.000468	0.281205456	0.377372	-0.471311535	0.166653	C1	-	-	Y	-	Y	-	-
ENSG00000010030	ETV7	-3.822553576	0.009061	-1.186705603	NA	-1.334343208	NA	2.641118687	NA	2.498925829	NA	-0.14674605	NA	C3	-	Y	-	-	-	-	-
ENSG00000010072	SPRTN	0.300216004	0.017729	-0.095396607	0.612745	0.067750877	0.715619	-0.390023573	1.73E-05	-0.21818544	0.026554	0.166432837	0.248878	C5	-	Y	-	-	Y	-	-
ENSG00000010219	DYRK4	-0.482921867	0.002434	-0.138443701	0.567268	-0.19705238	0.301709	0.350206609	0.060408	0.300262404	0.091469	-0.055828122	0.892177	C4	-	Y	-	-	-	-	-
ENSG00000010278	CD9	-0.48383725	0.017098	0.019602609	0.955691	-0.178895284	0.47969	0.509425219	0.004905	0.319672266	0.109429	-0.19465171	0.500085	C4	-	Y	-	-	Y	-	-
ENSG00000010295	IFFO1	0.463029087	6.30E-07	0.150936276	0.263794	0.314820403	0.002037	-0.30559692	0.0016	-0.13346765	0.185559	0.166744836	0.240869	C5	-	Y	-	Y	Y	-	-
ENSG00000010318	PHF7	-0.201121944	0.278217	0.213065611	0.355861	0.267588414	0.215327	0.421923833	0.029512	0.483382375	0.011779	0.055592328	0.912643	C4	-	-	-	-	Y	Y	-
ENSG00000010319	SEMA3G	-1.696800935	6.27E-06	-1.092263847	0.004285	-0.980501228	0.012484	0.612367746	0.262486	0.730439835	0.175816	0.113316645	NA	C3	-	Y	Y	Y	-	-	-
ENSG00000010361	FUZ	-0.377902708	1.14E-05	-0.019161751	0.906526	-0.062209373	0.617995	0.364949574	2.12E-06	0.330570131	3.69E-06	-0.039567198	0.817323	C4	-	Y	-	-	Y	Y	-
ENSG00000010404	IDS	0.283368822	0.000602	0.30586335	0.000358	0.201715718	0.02826	0.029124359	0.805038	-0.067081313	0.520116	-0.101317039	0.477664	C1	-	-	Y	-	-	-	-
ENSG00000010438	PRSS3	0.262995483	0.006066	0.095728268	0.516409	-0.178226889	0.153125	-0.160929811	0.144532	-0.426602178	1.91E-05	-0.270812188	0.062532	C5	-	-	-	-	-	Y	-
ENSG00000010539	ZNF200	-0.149732463	0.212778	-0.347146762	0.001408	-0.303982614	0.008708	-0.191171801	0.111912	-0.139674508	0.285117	0.046062814	0.860105	C4	-	-	Y	Y	-	-	-
ENSG00000010626	LRRC23	-0.489151814	0.006086	-0.127395892	0.558172	0.007118724	0.976641	0.369129215	0.039539	0.511787221	0.001403	0.13782131	0.561462	C2	-	Y	-	-	Y	Y	-
ENSG00000010818	HIVEP2	0.224236976	0.012359	0.243924564	0.03926	0.301237552	0.001062	0.026120502	0.843971	0.091398865	0.32301	0.06037638	0.755132	C4	-	-	-	Y	-	-	-
ENSG00000011007	ELOA	0.183991457	0.000177	-0.166042771	0.012237	-0.039342051	0.689633	-0.343643286	1.11E-12	-0.209006023	0.000157	0.129483293	0.182771	C5	-	-	-	-	Y	-	-
ENSG00000011021	CLCN6	0.351790595	9.42E-08	0.318484594	5.74E-06	0.299911371	0.00012	-0.026948276	0.775735	-0.037329379	0.705337	-0.015433375	0.939246	C1	-	Y	Y	-	-	-	-
ENSG00000011105	TSPAN9	-0.33177895	5.86E-05	-0.269390734	0.010342	-0.275839459	0.002229	0.068754454	0.585401	0.070683223	0.532627	-0.003271495	0.991385	C4	-	Y	-	-	-	-	-
ENSG00000011132	APBA3	0.567218125	3.59E-05	0.253125924	0.145639	0.343214276	0.019289	-0.307241604	0.021304	-0.208797817	0.11556	0.092791005	0.690042	C5	-	Y	-	Y	Y	-	-
ENSG00000011198	ABHD5	0.60881129	1.10E-18	0.197021443	0.099693	0.215153848	0.029348	-0.405267572	2.38E-06	-0.379053806	4.96E-07	0.021131001	0.939246	C5	Y	Y	-	-	Y	Y	-
ENSG00000011243	AKAP8L	0.314410191	2.63E-05	0.109479142	0.229187	0.075296734	0.423515	-0.198455502	0.006571	-0.224397839	0.001496	-0.031491811	0.833038	C5	Y	Y	-	-	-	-	-
ENSG00000011260	UTP18	0.306012466	1.15E-07	0.005656749	0.963184	-0.010185453	0.92667	-0.29372254	2.08E-07	-0.301507834	6.31E-08	-0.012999309	0.939872	C5	-	Y	-	-	-	Y	-
ENSG00000011332	DPF1	0.491365927	2.99E-06	0.243248904	0.11416	0.261655285	0.085324	-0.241071868	0.010317	-0.2155921	0.036119	0.020891565	0.947649	C5	-	Y	-	-	-	-	-
ENSG00000011347	SYT7	-0.329694548	1.06E-05	-0.27551765	0.001346	-0.028036166	0.854383	0.060712122	0.556184	0.316360923	0.000521	0.250411836	0.045396	C4	Y	Y	-	-	-	Y	-
ENSG00000011405	PIK3C2A	0.486016341	1.49E-07	0.177535667	0.161346	0.275992397	0.011401	-0.301872321	8.82E-08	-0.195585804	0.000799	0.100918917	0.338012	C1	-	Y	-	-	Y	-	-
ENSG00000011422	PLAUR	-0.233439722	0.043887	-0.343956254	0.005364	-0.357585513	0.005747	-0.103917054	0.435707	-0.109397311	0.433966	-0.010719293	0.974616	C4	-	-	Y	Y	-	-	-
ENSG00000011454	RABGAP1	0.458055717	4.95E-18	0.103547576	0.166533	0.19886428	0.004977	-0.348253926	2.39E-12	-0.244308435	1.23E-05	0.098204347	0.306699	C5	-	Y	-	-	Y	-	-
ENSG00000011566	MAP4K3	0.345596579	1.99E-09	0.177760137	0.020676	0.096796971	0.224006	-0.161395339	0.00652	-0.234007014	1.37E-05	-0.078063105	0.46204	C5	-	Y	-	-	-	-	-
ENSG00000011638	TMEM159	-0.436081321	0.10339	0.0921515	0.831722	0.103151275	0.787408	0.535280862	0.066314	0.5540771	0.04921	0.01322176	0.986496	C4	-	-	-	-	-	Y	-
ENSG00000011677	GABRA3	0.139335987	0.434636	0.322216988	0.045908	0.240533779	0.158958	0.18964915	0.255859	0.116083693	0.531984	-0.078649835	0.787764	C4	-	-	Y	-	-	-	-
ENSG00000013016	EHD3	0.329614097	0.000435	0.276043049	0.010438	0.25611308	0.02546	-0.046938465	0.694225	-0.058990104	0.633364	-0.017166964	0.947133	C5	-	Y	-	-	-	-	-
ENSG00000013293	SLC7A14	0.041682006	0.761268	0.37184465	0.001055	0.311926175	0.026662	0.336520023	0.000275	0.284899311	0.13677	-0.058253824	0.893051	C4	-	-	Y	Y	Y	-	-
ENSG00000013297	CLDN11	-1.086308277	0.331114	0.090352175	NA	0.845095115	0.328133	1.184263007	0.313405	1.94836585	0.022042	0.758995272	NA	C2	-	-	-	-	-	Y	-
ENSG00000013441	CLK1	0.313730977	0.000433	-0.380742069	0.000263	-0.420820016	0.060289	-0.688069262	3.67E-18	-0.720121265	0.000117	-0.03777175	0.943834	C5	-	Y	Y	-	Y	Y	-
ENSG00000013523	ANGEL1	0.091989536	0.436574	0.221123739	0.066585	0.303229086	0.00334	0.135566759	0.234374	0.225831833	0.02177	0.085050258	0.636953	C4	-	-	-	Y	-	-	-
ENSG00000013561	RNF14	0.440721121	2.08E-08	0.144842471	0.135846	0.252929758	0.002167	-0.289479075	0.000136	-0.173108709	0.0285	0.111066826	0.333442	C5	Y	Y	-	-	-	-	-
ENSG00000013563	DNASE1L1	-0.581579714	0.000405	-0.105580753	0.657458	-0.200456954	0.294842	0.482940654	0.007368	0.396023744	0.030542	-0.091931854	0.785782	C2	-	Y	-	-	Y	Y	-
ENSG00000013573	DDX11	0.206593587	0.000142	-0.043931508	0.627026	-0.148062446	0.028236	-0.24402955	9.40E-06	-0.339995669	2.85E-10	-0.101146465	0.292248	C5	-	-	-	-	-	Y	-
ENSG00000013583	HEBP1	-0.625324388	0.005086	-0.410577913	0.163449	-0.060485515	0.855866	0.222002233	0.383883	0.580899764	0.001454	0.352275254	0.217624	C4	-	Y	-	-	-	Y	-
ENSG00000013619	MAMLD1	3.303917193	0.002821	2.788667181	NA	2.704458523	NA	-0.502642124	0.593051	-0.585430717	0.519961	-0.087631928	NA	C5	-	Y	-	-	-	-	-
ENSG00000014164	ZC3H3	0.3729583	0.000598	0.12517437	0.436591	0.108273872	0.504031	-0.241217839	0.034075	-0.249981549	0.031019	-0.013939107	0.966758	C5	-	Y	-	-	-	-	-
ENSG00000015133	CCDC88C	-0.333597663	0.004301	-0.123863801	0.422498	-0.107137125	0.47592	0.215811547	0.110474	0.241222327	0.067743	0.019771805	0.95032	C4	-	Y	-	-	-	-	-
ENSG00000015153	YAF2	0.375674132	4.16E-05	0.053613023	0.72862	0.047469922	0.750727	-0.315815199	0.000233	-0.313353737	0.000317	-0.003002368	0.991437	C5	-	Y	-	-	Y	Y	-
ENSG00000015520	NPC1L1	3.414024194	0.024026	0.55716596	NA	1.580551874	NA	-2.822841405	NA	-1.819151043	0.132453	1.011612885	NA	C5	-	Y	-	-	-	-	-
ENSG00000015592	STMN4	0.51134245	1.25E-10	0.07643752	0.574114	0.117812204	0.289122	-0.427929666	1.45E-09	-0.378825441	1.93E-08	0.043792606	0.797535	C5	-	Y	-	-	Y	Y	-
ENSG00000016391	CHDH	-0.941272553	0.006674	-0.740696163	0.059068	-0.689184588	0.072241	0.208116465	0.698252	0.267378857	0.60555	0.054278296	NA	C4	-	Y	-	-	-	-	-
ENSG00000016402	IL20RA	-1.377283765	5.70E-11	-0.722386119	0.0014	-0.710438617	0.000701	0.660069051	0.013617	0.681684891	0.007989	0.015392441	0.981547	C3	-	Y	Y	Y	Y	Y	-
ENSG00000017797	RALBP1	0.404051151	6.43E-11	0.226243821	0.001051	0.161915184	0.01963	-0.171477526	0.003922	-0.227620911	3.81E-05	-0.061542789	0.541178	C5	-	Y	-	-	-	-	-
ENSG00000018408	WWTR1	1.160471052	0.006504	1.036078972	0.039716	0.606896902	0.29796	-0.117562161	0.808195	-0.537817591	0.187739	-0.425567871	NA	C1	-	Y	Y	-	-	-	-
ENSG00000018607	ZNF806	-0.652647586	0.549846	0.589211059	NA	1.135312557	0.149618	1.24689184	0.195082	1.801717109	0.024332	0.546864002	NA	C2	-	-	-	-	-	Y	-
ENSG00000018869	ZNF582	-0.254444312	0.252024	-0.542051256	0.018706	-0.715134353	0.001091	-0.282465212	0.267857	-0.446483828	0.064479	-0.168963639	0.720545	C5	-	-	Y	Y	-	-	-
ENSG00000019485	PRDM11	0.11352822	0.369573	-0.201309666	0.212389	-0.092551109	0.569363	-0.307987376	0.005344	-0.191605688	0.070512	0.111276086	0.607328	C5	-	-	-	-	Y	-	-
ENSG00000019505	SYT13	-0.875040222	0.000819	-0.858581413	0.000191	-0.255624177	0.309887	0.023550816	0.959704	0.634019827	0.028406	0.605678114	0.047284	C3	-	Y	Y	-	-	Y	Y
ENSG00000019549	SNAI2	0.280048907	0.003362	0.415878271	1.70E-05	0.099795557	0.41418	0.142106956	0.174023	-0.165469227	0.105761	-0.313320344	0.004047	C4	-	-	Y	-	-	-	Y
ENSG00000019991	HGF	0.578506815	0.351319	-0.981857411	0.196949	-0.578445688	0.449409	-1.553745273	0.008169	-1.141915934	0.04792	0.406905993	NA	C5	-	-	-	-	Y	Y	-
ENSG00000019995	ZRANB1	0.348025533	0.000425	0.043999593	0.793352	0.033934185	0.821186	-0.297783104	0.001286	-0.299531881	0.000556	-0.007087665	0.97796	C5	-	Y	-	-	-	-	-
ENSG00000020181	ADGRA2	-0.271003199	0.000266	0.150285277	0.095559	0.090260374	0.33927	0.427896392	3.38E-09	0.375840249	7.96E-08	-0.057117203	0.693425	C4	-	-	-	-	Y	Y	-
ENSG00000021355	SERPINB1	-0.877532175	2.64E-07	-0.262963887	0.358901	-0.181243174	0.374238	0.622750732	0.010779	0.710923829	0.000109	0.084803431	0.870563	C4	-	Y	-	-	Y	Y	-
ENSG00000021574	SPAST	0.327880635	1.35E-08	0.16688489	0.032356	0.089805498	0.320057	-0.15453794	0.005431	-0.223295574	6.66E-05	-0.074159871	0.518573	C5	-	Y	-	-	-	-	-
ENSG00000021645	NRXN3	0.145926166	0.80527	1.391967652	0.000418	1.147027434	0.002428	1.253251744	0.000871	1.015142967	0.006611	-0.23979219	NA	C2	-	-	Y	Y	Y	Y	-

ENSG00000021762	OSBPL5	-0.65500569	0.011221	-0.235693898	0.487488	-0.171072318	0.609516	0.425580925	0.165567	0.498493802	0.089163	0.067525328	0.918762	C2	-	Y	-	-	-	-	-
ENSG00000022976	ZNF839	-0.408663481	0.000454	-0.070026639	0.705598	-0.22234045	0.11042	0.345136685	0.005879	0.201025529	0.150925	-0.149372416	0.472349	C4	-	Y	-	-	Y	-	-
ENSG00000023041	ZDHHC6	0.360272289	5.83E-07	0.103510291	0.313135	0.112560873	0.263966	-0.250355243	6.62E-05	-0.232807178	0.000452	0.012104978	0.948558	C5	-	Y	-	-	-	-	-
ENSG00000023171	GRAMD1B	-0.665297153	5.83E-08	-0.380619272	0.00592	-0.036066571	0.853227	0.291444808	0.043772	0.643850156	2.80E-07	0.347376302	0.031355	C4	-	Y	Y	-	-	Y	Y
ENSG00000023287	RB1CC1	0.592613832	1.38E-18	0.241358717	0.010365	0.225260163	0.002412	-0.344864116	3.44E-07	-0.352771808	5.14E-11	-0.013482331	0.943834	C5	Y	Y	-	-	Y	Y	-
ENSG00000023330	ALAS1	0.673843986	1.52E-14	0.198109485	0.094338	0.413248443	1.50E-05	-0.469287317	6.74E-08	-0.246071822	0.004976	0.217949938	0.075964	C5	-	Y	-	Y	Y	-	-
ENSG00000023445	BIRC3	3.240827802	4.13E-07	1.889782496	NA	1.630202549	NA	-1.355464734	0.009155	-1.598197234	0.001615	-0.252012358	NA	C5	-	Y	-	-	Y	Y	-
ENSG00000023572	GLRX2	-0.031503158	0.811906	-0.470451806	2.56E-05	-0.333053272	0.005868	-0.43197693	5.30E-06	-0.287133158	0.005144	0.140394665	0.472014	C5	-	-	Y	Y	Y	-	-
ENSG00000023608	SNAPC1	0.490139788	3.85E-07	-0.007564463	0.968866	0.235893712	0.040417	-0.491397704	5.03E-07	-0.239530939	0.023548	0.246459695	0.075524	C5	-	Y	-	-	Y	-	-
ENSG00000023697	DERA	-0.536125941	5.39E-14	-0.241585618	0.003005	-0.168203753	0.040653	0.301271414	0.000163	0.382665488	3.81E-07	0.076330199	0.582472	C4	-	Y	-	-	Y	Y	-
ENSG00000023839	ABCC2	1.286042202	0.002037	0.651247123	0.291572	0.20630342	0.7618	-0.628782935	0.187733	-1.0650152	0.010156	-0.442770073	NA	C5	-	Y	-	-	-	Y	-
ENSG00000023909	GCLM	0.170480271	0.169234	-0.270709222	0.060013	-0.172367655	0.202295	-0.435215673	0.000614	-0.3280005	0.005755	0.101811326	0.688161	C5	-	-	-	-	Y	Y	-
ENSG00000024048	UBR2	0.580861323	3.90E-17	0.377297902	3.07E-06	0.356543422	5.53E-06	-0.197073864	0.002593	-0.209735123	0.000928	-0.017992488	0.921602	C1	-	Y	Y	Y	-	-	-
ENSG00000024422	EHD2	-1.169590388	0.001027	-0.159189856	0.752157	-0.372486865	0.38308	1.015789609	0.012701	0.811094993	0.069633	-0.210354086	NA	C3	-	Y	-	-	Y	-	-
ENSG00000025039	RRAGD	0.39040216	0.001911	0.211615596	0.11579	0.235562883	0.076703	-0.172306547	0.204054	-0.140294982	0.32636	0.026668272	0.928789	C5	-	Y	-	-	-	-	-
ENSG00000025423	HSD17B6	-0.537684362	0.188532	0.11215784	0.835377	0.192458769	0.654742	0.656029583	0.098892	0.744838832	0.040393	0.083379765	NA	C3	-	-	-	-	-	Y	-
ENSG00000025800	KPNA6	0.421614734	2.22E-10	0.186128175	0.030621	0.234124986	0.001399	-0.229366695	4.81E-05	-0.173124791	0.000768	0.051052292	0.63742	C1	-	Y	-	-	-	-	-
ENSG00000026297	RNASET2	-0.422162515	0.016013	0.128518728	0.534215	0.227379237	0.142474	0.558402661	0.001582	0.664304631	3.37E-05	0.101999573	0.709041	C4	-	Y	-	-	Y	Y	-
ENSG00000026508	CD44	1.304650816	6.43E-09	1.666768064	9.94E-13	1.397844032	3.08E-06	0.36894904	0.077936	0.110120056	0.743566	-0.264657718	0.563612	C1	-	Y	Y	Y	-	-	-
ENSG00000026652	AGPAT4	0.184862933	0.053918	0.187246393	0.103763	0.332764474	0.000324	0.00839255	0.947402	0.162263851	0.042356	0.148714948	0.199145	C4	-	-	-	Y	-	-	-
ENSG00000026950	BTN3A1	-0.234263343	0.025109	-0.112885108	0.456308	0.125699574	0.318249	0.127657565	0.290916	0.374676312	5.16E-05	0.241790631	0.070541	C4	-	-	-	-	-	Y	-
ENSG00000027001	MIPEP	-0.248463244	0.146735	0.083963946	0.736128	0.096875908	0.673568	0.338231443	0.063919	0.360075481	0.047585	0.016301142	0.972936	C4	-	-	-	-	-	Y	-
ENSG00000027075	PRKCH	-1.272666414	2.27E-11	-0.784954083	3.16E-05	-0.447554269	0.021491	0.494210035	0.0424	0.839913868	0.000115	0.340330059	0.247997	C3	-	Y	Y	Y	Y	Y	-
ENSG00000027644	INSRR	-1.234285051	1.20E-59	-0.337280231	0.001098	-0.255406754	0.017075	0.903777867	2.25E-21	0.993450747	2.00E-26	0.083719064	0.709041	C2	-	Y	Y	-	Y	Y	-
ENSG00000027697	IFNGR1	0.233359848	0.001454	-0.126683036	0.213988	-0.043726766	0.714988	-0.353643179	6.88E-07	-0.262621312	0.000356	0.085769101	0.551064	C5	-	-	-	-	Y	-	-
ENSG00000027847	B4GALT7	-0.371319257	0.018093	-0.244143435	0.227916	-0.208253507	0.269002	0.133643667	0.456887	0.17852724	0.264893	0.039135593	0.915306	C4	-	Y	-	-	-	-	-
ENSG00000028839	TBPL1	0.312675074	1.79E-05	0.073677847	0.491847	0.069639487	0.515956	-0.232507357	0.001855	-0.228317221	0.002671	-0.001130405	0.996048	C5	-	Y	-	-	-	-	-
ENSG00000029363	BCLAF1	0.108740369	0.035781	-0.006873828	0.949349	-0.209783154	3.38E-05	-0.109014309	0.006702	-0.303976397	1.95E-26	-0.200311106	7.11E-06	C5	-	-	-	-	-	Y	-
ENSG00000029725	RABEP1	0.386260561	2.05E-10	0.248279809	0.00015	0.193940154	0.002949	-0.131576792	0.02934	-0.177769077	0.00152	-0.051632761	0.612037	C5	Y	Y	-	-	-	-	-
ENSG00000029993	HMGB3	-0.246262374	2.58E-07	-0.077136695	0.258679	-0.327215171	1.16E-09	0.175779343	2.01E-05	-0.066140594	0.185005	-0.247239846	3.74E-07	C4	-	-	-	Y	-	-	-
ENSG00000030066	NUP160	0.461800251	2.30E-22	0.144102151	0.036775	0.104630272	0.070877	-0.31115054	4.89E-10	-0.342498066	1.25E-16	-0.036751236	0.737617	C5	-	Y	-	-	Y	Y	-
ENSG00000030110	BAK1	0.366047956	0.001845	-0.091148351	0.641242	-0.016338927	0.940062	-0.450841921	0.000192	-0.367572814	0.002295	0.077829607	0.777799	C5	-	Y	-	-	Y	Y	-
ENSG00000031081	ARHGAP31	0.197472188	0.00932	-0.129169272	0.19848	-0.064569591	0.545151	-0.319753958	1.78E-09	-0.247798101	1.61E-06	0.066775775	0.527252	C5	-	-	-	-	Y	-	-
ENSG00000031691	CENPQ	0.213308481	0.050375	-0.164889268	0.25374	0.004167248	0.981251	-0.371844215	0.000951	-0.194410211	0.079882	0.172047613	0.32098	C5	-	-	-	-	Y	-	-
ENSG00000032219	ARID4A	0.582323189	3.27E-16	0.276309389	0.001408	0.356015949	6.27E-06	-0.299763949	2.05E-06	-0.211757845	0.000833	0.082664261	0.470464	C5	-	Y	-	Y	-	-	-
ENSG00000032389	EIPR1	-0.646332983	1.28E-18	-0.083050677	0.363563	-0.168242579	0.022487	0.569792781	2.76E-15	0.49300948	4.28E-12	-0.082266883	0.45687	C2	-	Y	-	-	Y	Y	-
ENSG00000033011	ALG1	-0.199309806	0.265296	-0.207439961	0.320588	-0.355385646	0.026412	-0.001806979	0.993684	-0.140858199	0.460819	-0.144926782	0.615724	C4	-	-	-	Y	-	-	-
ENSG00000033030	ZCCHC8	0.423590346	9.10E-13	0.117593914	0.215542	0.16143574	0.029754	-0.299246898	4.37E-06	-0.247456371	3.12E-05	0.046508047	0.752303	C5	Y	Y	-	-	-	-	-
ENSG00000033100	CHPF2	0.546894604	2.22E-23	0.141462525	0.078472	0.067200816	0.421039	-0.398774574	3.64E-13	-0.464926188	4.45E-20	-0.071582661	0.517529	C5	-	Y	-	-	Y	Y	-
ENSG00000033122	LRRC7	-0.446415696	0.101792	0.369068225	0.16678	-0.134199096	0.698518	0.821991997	0.000631	0.327161689	0.287493	-0.500511528	0.094848	C2	-	-	-	-	Y	-	-
ENSG00000033178	UBA6	0.425997055	5.92E-12	0.049644867	0.649698	0.21944228	0.005777	-0.370512689	2.34E-13	-0.191755218	0.000664	0.172900227	0.034505	C5	-	Y	-	-	Y	-	-
ENSG00000033327	GAB2	0.372995735	5.85E-10	0.157481776	0.054405	-0.004050167	0.971887	-0.209338899	7.16E-06	-0.362694928	2.03E-23	-0.158570486	0.008404	C5	-	Y	-	-	-	Y	-
ENSG00000033627	ATP6V0A1	0.48458532	5.21E-17	0.184902036	0.014725	0.161749373	0.010337	-0.293165599	3.60E-07	-0.308218377	2.22E-10	-0.020436464	0.887295	C5	-	Y	-	-	-	Y	-
ENSG00000033867	SLC4A7	0.376346392	3.89E-07	0.100519098	0.330971	0.164792438	0.079324	-0.269456266	2.35E-05	-0.196874051	0.004505	0.067134647	0.612037	C5	-	Y	-	-	-	-	-
ENSG00000034152	MAP2K3	0.310871893	0.047826	0.176886625	0.435026	0.300636148	0.077584	-0.128016115	0.475698	0.004628003	0.979446	0.127120579	0.635413	C1	-	Y	-	-	-	-	-
ENSG00000034533	ASTE1	0.017254014	0.930613	-0.448496024	0.003615	-0.212629804	0.188679	-0.459532792	0.001554	-0.215199748	0.163014	0.239050482	0.307349	C5	-	-	Y	-	Y	-	-
ENSG00000035403	VCL	0.997077597	1.40E-57	0.308830531	0.000178	0.288411617	0.000253	-0.6820235	2.52E-31	-0.693946765	1.36E-35	-0.017482503	0.928882	C5	-	Y	Y	-	Y	Y	-
ENSG00000035664	DAPK2	-0.657872017	0.064583	-1.042838656	0.014161	-0.601598486	0.15743	-0.376536433	0.495085	0.071284458	0.908458	0.441436155	NA	C3	-	-	Y	-	-	-	-
ENSG00000035687	ADSS2	0.33819479	1.31E-08	0.139772925	0.069034	0.104414228	0.168872	-0.191857844	9.56E-05	-0.219083674	2.92E-06	-0.032699677	0.757257	C5	-	Y	-	-	-	-	-
ENSG00000036448	MYOM2	-0.842804273	0.174611	-0.33394055	0.679083	0.359740017	0.579013	0.516817543	0.472014	1.218370034	0.024438	0.697528905	NA	C4	-	-	-	-	-	Y	-
ENSG00000036549	ZZZ3	0.289121291	0.000157	-0.025144864	0.861545	0.075572361	0.45559	-0.307395648	6.01E-07	-0.199247198	0.000528	0.102563387	0.287144	C5	Y	-	-	-	Y	-	-
ENSG00000036565	SLC18A1	-0.267471366	0.0003	0.184480679	0.088833	0.209149352	0.012263	0.458480224	1.23E-07	0.491399937	1.77E-11	0.027434367	0.905466	C4	-	-	-	-	Y	Y	-
ENSG00000037280	FLT4	-2.321603208	0.002732	-1.456318996	NA	-1.474430416	0.058263	0.875245756	0.492592	0.867595169	0.522631	-0.013439204	NA	C3	-	Y	-	-	-	-	-
ENSG00000037757	MRI1	0.38813632	8.67E-06	0.105830799	0.439631	0.16978829	0.114481	-0.275682696	0.00305	-0.2037287	0.024324	0.066559678	0.723061	C5	-	Y	-	-	-	-	-
ENSG00000038002	AGA	-0.742732108	3.62E-09	-0.184799292	0.274353	-0.440448989	0.001558	0.564745686	9.03E-05	0.316745989	0.04966	-0.253422812	0.240869	C2	-	Y	-	Y	Y	Y	-
ENSG00000038210	PI4K2B	-0.447372861	0.002559	-0.167680252	0.391054	-0.389424779	0.022241	0.28612592	0.080187	0.072765247	0.744963	-0.218854034	0.38344	C4	-	Y	-	Y	-	-	-
ENSG00000038219	BOD1L1	0.375673551	7.74E-06	0.137086192	0.232585	0.146967836	0.145394	-0.232566657	4.24E-05	-0.214548237	1.68E-05	0.012883418	0.933407	C1	-	Y	-	-	-	-	-
ENSG00000038358	EDC4	0.504347568	3.14E-15	0.128520665	0.106516	0.133578188	0.078831	-0.36939283	1.96E-10	-0.35616609	4.78E-10	0.008018975	0.960084	C5	-	Y	-	-	Y	Y	-
ENSG00000038427	VCAN	-0.667947782	3.56E-05	1.023266998	2.22E-11	1.224922592	1.96E-06	1.697643053	4.61E-32	1.908003862	5.14E-14	0.202540679	0.709413	C2	-	Y	Y	Y	Y	Y	-
ENSG00000039068	CDH1	-2.072512495	0.00243	-1.023140747	0.140961	-0.55100687	0.379301	1.067566203	0.310819	1.535716776	0.062238	0.472150487	NA	C3	-	Y	-	-	-	-	-
ENSG00000039319	ZFYVE16	0.174794212	0.029803	0.243850774	0.018346	0.307132746	0.000123	0.075556195	0.333502	0.147013778	0.002757	0.066042645	0.599766	C4	-	-	-	Y	-	-	-
ENSG00000040531	CTNS	0.4196608	0.00023	0.289967676	0.032616	0.207261442	0.15743	-0.123040876	0.34069	-0.197932957	0.11072	-0.080065862	0.73505	C1	-	Y	-	-	-	-	-
ENSG00000040608	RTN4R	-0.748524588	0.009167	-0.214892581	0.562723	-0.015478622	0.974932	0.538998107	0.104132	0.748329247	0.022928	0.203860441	0.731303	C4	-	Y	-	-	-	Y	-
ENSG00000041515	MYO16	0.176933169	0.211074	-0.135050494	0.534466	-0.050756161	0.846022	-0.305593175	0.048954	-0.213360566	0.235686	0.089283045	0.829491	C5	-	-	-	-	Y	-	-
ENSG00000041880	PARP3	-0.429116426	0.002423	-0.495095122	0.002223	-0.246193382	0.123534	-0.05930304	0.778248	0.197798455	0.205562	0.25234841	0.234689	C4	-	Y	Y	-	-	-	-
ENSG00000041982	TNC	0.722517816	1.90E-07	1.336454331	5.84E-17	1.173441809	1.40E-15	0.619676885	2.98E-06	0.466119226	0.000106	-0.159681486	0.546192	C1	-	Y	Y	Y	Y	Y	-
ENSG00000042286	AIFM2	-0.537424649	0.01607	-0.324753219	0.210746	-0.430144697	0.057278	0.218777491	0.454505	0.122042089	0.69836	-0.10242234	0.835773	C3	-	Y	-	-	-	-	-

ENSG00000042493	CAPG	-2.225496777	0.029509	-0.166437258	NA	-0.174812815	0.884094	2.063611344	0.065952	2.068020036	0.043902	-0.005807622	NA	C4	-	Y	-	-	-	Y	-
ENSG00000043143	JADE2	0.151688381	0.296033	0.181287252	0.312938	0.432027941	0.000978	0.036317992	0.851171	0.295027932	0.021745	0.253622843	0.189606	C4	-	-	-	Y	-	-	-
ENSG00000044090	CUL7	-0.176385718	0.012261	0.07999659	0.363823	0.198060523	0.008904	0.262927274	7.92E-05	0.389130487	4.62E-09	0.120901864	0.226717	C4	-	-	-	-	-	Y	-
ENSG00000044459	CNTLN	-0.433065987	5.46E-06	-0.03697101	0.820136	-0.071835476	0.586378	0.402577058	3.97E-05	0.375772985	8.93E-05	-0.031852929	0.891955	C4	-	Y	-	-	Y	Y	-
ENSG00000046604	DSG2	2.323267407	0.01826	1.001015502	NA	1.265631011	NA	-1.313496609	0.166987	-1.041685186	0.267018	0.265861484	NA	C5	-	Y	-	-	-	-	-
ENSG00000046647	GEMIN8	-0.162316991	0.079958	-0.014870554	0.926551	0.140007126	0.161484	0.153755384	0.123115	0.317062156	0.000169	0.157768965	0.215908	C4	-	-	-	-	-	Y	-
ENSG00000046774	MAGEC2	-0.845853122	0.009387	-0.699679823	0.059217	-0.523356341	0.219205	0.152841539	0.773282	0.336831238	0.50419	0.177951372	NA	C3	-	Y	-	-	-	-	-
ENSG00000047578	KIAA0556	0.469087382	2.37E-09	0.251793172	0.009462	0.273797495	0.003553	-0.210870387	0.00857	-0.180616758	0.030405	0.024812536	0.906331	C1	-	Y	-	-	-	-	-
ENSG00000047579	DTNBP1	-0.305907299	0.154405	0.182337539	0.485639	-0.025285247	0.937664	0.494675041	0.010657	0.295003331	0.175631	-0.20481632	0.50724	C4	-	-	-	-	Y	-	-
ENSG00000047597	XK	-2.047756438	0.000166	-0.772499975	0.181027	-0.525110531	0.434188	1.279761624	0.071709	1.536226202	0.034859	0.25104427	NA	C3	-	Y	-	-	-	Y	-
ENSG00000047644	WWC3	0.491204746	1.39E-07	0.343052721	0.00011	0.354420229	3.68E-05	-0.141661738	0.146902	-0.122157424	0.213291	0.014376858	0.943834	C5	-	Y	Y	Y	-	-	-
ENSG00000047648	ARHGAP6	-0.488202471	0.006781	0.044277409	0.87795	0.072934414	0.814565	0.539259841	0.00284	0.575741656	0.006493	0.030163366	0.955616	C4	-	Y	-	-	Y	Y	-
ENSG00000047932	GOPC	0.547428511	1.40E-15	0.195648187	0.040459	0.302747813	6.40E-05	-0.344777479	1.53E-10	-0.230230061	9.76E-07	0.109155156	0.212719	C5	Y	Y	-	Y	Y	-	-
ENSG00000048028	USP28	0.764538388	3.27E-21	0.282382708	0.005728	0.590014646	1.96E-12	-0.475773637	9.18E-13	-0.160002808	0.021639	0.310270736	8.46E-05	C1	Y	Y	-	Y	Y	-	Y
ENSG00000048342	CC2D2A	-0.77910319	1.24E-07	-0.416243105	0.017008	-0.273920785	0.100186	0.369916296	0.038836	0.519808503	0.001059	0.145318828	0.61262	C4	-	Y	Y	-	Y	Y	-
ENSG00000048405	ZNF800	0.373924117	1.80E-06	0.027160615	0.871871	0.16119663	0.131274	-0.340205085	3.18E-05	-0.197919431	0.0147	0.136834618	0.364794	C5	-	Y	-	-	Y	-	-
ENSG00000048471	SNX29	-0.719360661	1.04E-10	-0.101175912	0.534989	-0.016676167	0.929166	0.624888412	8.23E-08	0.717125546	1.17E-10	0.087381532	0.690721	C4	-	Y	-	-	Y	Y	-
ENSG00000048540	LMO3	-0.790250835	4.93E-21	-0.136573508	0.293364	-0.061581921	0.680606	0.659983931	6.19E-13	0.743565051	8.41E-16	0.077291014	0.71782	C2	-	Y	-	-	Y	Y	-
ENSG00000048707	VPS13D	0.405925154	0.001044	0.332142602	0.019847	0.406584613	0.001397	-0.067819792	0.55633	0.01491959	0.906446	0.077444178	0.641552	C1	-	Y	Y	Y	-	-	-
ENSG00000048828	FAM120A	0.300657163	7.49E-07	0.187422307	0.008025	0.227144916	0.00086	-0.106823605	0.019496	-0.058790344	0.274196	0.042664724	0.629175	C5	-	Y	-	-	-	-	-
ENSG00000049089	COL9A2	-0.813337772	3.54E-05	-0.943699115	1.03E-05	-0.899416262	8.45E-05	-0.123566362	0.685588	-0.071253412	0.839149	0.046785101	0.943834	C4	-	Y	Y	Y	-	-	-
ENSG00000049130	KITLG	-0.441069311	4.83E-05	0.082728005	0.618995	-0.12064201	0.408797	0.529991054	2.41E-07	0.334985701	0.002096	-0.200248971	0.19315	C4	-	Y	-	-	Y	Y	-
ENSG00000049192	ADAMTS6	-0.949710762	0.075198	0.47041015	0.391054	0.435628178	0.449409	1.42800148	0.002716	1.399678819	0.005719	-0.032517134	NA	C2	-	-	-	-	Y	Y	-
ENSG00000049239	H6PD	-0.5684986	4.68E-18	-0.037927028	0.747933	-0.192452601	0.015429	0.537048666	1.40E-15	0.390756701	1.36E-08	-0.151656056	0.140447	C4	-	Y	-	-	Y	Y	-
ENSG00000049246	PER3	-0.258833548	0.023443	-0.091885592	0.612113	0.08388703	0.664768	0.172584005	0.145529	0.357617498	0.002645	0.178054878	0.40972	C4	-	-	-	-	-	Y	-
ENSG00000049449	RCN1	-0.440685541	2.26E-09	-0.473998457	3.73E-08	-0.353560271	2.14E-06	-0.026772289	0.81656	0.102042296	0.19911	0.123165976	0.312116	C4	-	Y	Y	Y	-	-	-
ENSG00000049541	RFC2	-0.331212515	4.29E-07	-0.207039092	0.012655	-0.360190465	1.29E-05	0.130853397	0.06882	-0.014396134	0.895788	-0.150078866	0.209127	C4	-	Y	-	Y	-	-	-
ENSG00000049769	PPP1R3F	0.269698838	0.109009	0.376709506	0.036011	0.249478912	0.192818	0.11383422	0.511493	-0.005334314	0.977988	-0.124511821	0.635328	C4	-	-	Y	-	-	-	-
ENSG00000050165	DKK3	-0.437167267	4.36E-09	-0.162524612	0.11328	-0.056429105	0.592292	0.281104099	0.000565	0.395675712	5.63E-09	0.109577895	0.377059	C4	-	Y	-	-	-	Y	-
ENSG00000050344	NFE2L3	-0.555249525	1.36E-11	-0.030837168	0.840382	-0.058067808	0.617652	0.530909155	4.00E-10	0.511748394	2.79E-11	-0.024332665	0.90989	C2	-	Y	-	-	Y	Y	-
ENSG00000050405	LIMA1	0.633006759	1.10E-26	0.19852765	0.011702	0.037802526	0.744821	-0.428467233	3.97E-15	-0.580269699	1.78E-23	-0.157736441	0.107082	C5	Y	Y	-	-	Y	Y	-
ENSG00000050438	SLC4A8	-0.434789324	1.15E-13	0.043109458	0.739325	0.026303962	0.832967	0.484360857	5.76E-13	0.475995543	7.94E-14	-0.014161018	0.951343	C4	-	Y	-	-	Y	Y	-
ENSG00000050628	PTGER3	-0.887074772	0.029475	-0.167425051	0.719363	-0.362010106	0.354042	0.727433366	0.090496	0.540273701	0.241508	-0.191572885	NA	C2	-	Y	-	-	-	-	-
ENSG00000050748	MAPK9	0.335382502	2.37E-07	0.369027216	1.44E-07	0.167385208	0.047888	0.040023327	0.639606	-0.153134132	0.038173	-0.198704545	0.029626	C1	-	Y	Y	-	-	-	-
ENSG00000050820	BCAR1	0.302201822	0.000238	0.280319394	0.001505	0.15975195	0.142519	-0.015486856	0.890834	-0.128138798	0.17532	-0.117919382	0.39019	C1	-	Y	-	-	-	-	-
ENSG00000051108	HERPUD1	-0.456473454	1.00E-07	-0.23211657	0.097962	-0.021117127	0.886821	0.230657718	0.069082	0.449980671	4.57E-07	0.214283232	0.218587	C4	-	Y	-	-	-	Y	-
ENSG00000051180	RAD51	-0.161992286	0.068895	-0.225796073	0.048277	-0.387352434	6.45E-06	-0.057732965	0.660693	-0.210600109	0.024676	-0.158257245	0.326298	C4	-	-	-	Y	-	-	-
ENSG00000051341	POLQ	0.392053122	4.90E-06	0.177976863	0.094879	0.125164039	0.250618	-0.207717459	0.014085	-0.252675361	0.001619	-0.050179879	0.770023	C5	-	Y	-	-	-	-	-
ENSG00000051382	PIK3CB	0.409598912	0.000475	0.191422077	0.136531	0.217893952	0.059968	-0.211959648	0.092335	-0.177168307	0.1549	0.029289969	0.908872	C5	-	Y	-	-	-	-	-
ENSG00000051523	CYBA	-0.485574982	4.19E-05	-0.310459843	0.024019	-0.32450659	0.019343	0.1816376	0.129373	0.176063789	0.163506	-0.010803307	0.972653	C4	-	Y	Y	Y	-	-	-
ENSG00000051620	HEBP2	-0.630481693	0.001542	-0.357542108	0.197512	-0.563606598	0.012204	0.278925734	0.340452	0.081379435	0.808325	-0.20305275	0.67669	C4	-	Y	-	Y	-	-	-
ENSG00000052126	PLEKHA5	-0.269445117	0.039397	0.08826358	0.621257	0.048060699	0.797215	0.364291962	0.004132	0.332147607	0.01081	-0.03733239	0.900802	C4	-	-	-	-	Y	Y	-
ENSG00000052795	FNIP2	0.327606449	0.000127	0.259023248	0.020771	0.129708474	0.257866	-0.062070573	0.548951	-0.183710515	0.018684	-0.126648061	0.362143	C1	-	Y	-	-	-	-	-
ENSG00000052841	TTC17	0.404134064	1.11E-10	0.186370704	0.016453	0.209654636	0.004837	-0.211439966	2.42E-06	-0.179745643	0.000123	0.026280954	0.816093	C5	-	Y	-	-	-	-	-
ENSG00000053371	AKR7A2	-0.488269507	9.12E-06	-0.149148526	0.329044	0.023145152	0.895628	0.345563999	0.004041	0.526087667	5.20E-07	0.175736512	0.284837	C4	-	Y	-	-	Y	Y	-
ENSG00000053438	NNAT	-0.302142406	4.04E-05	-0.356782148	0.000131	-0.388258791	7.86E-06	-0.047599886	0.587537	-0.071450891	0.354002	-0.02873029	0.8863	C4	-	Y	Y	Y	-	-	-
ENSG00000053524	MCF2L2	0.387805302	0.195491	0.657839999	0.01082	0.687684692	0.006689	0.27596927	0.345485	0.314414162	0.273814	0.032719819	0.955888	C1	-	-	Y	Y	-	-	-
ENSG00000054277	OPN3	0.413965015	0.003839	0.215875697	0.213514	0.170248836	0.331172	-0.191954916	0.206797	-0.229290309	0.117541	-0.042675853	0.897245	C5	-	Y	-	-	-	-	-
ENSG00000054282	SDCCAG8	-0.288906744	0.002608	0.008821217	0.961535	0.001853007	0.991357	0.304302034	0.001118	0.305738399	0.000959	-0.003676477	0.988432	C4	-	-	-	-	Y	Y	-
ENSG00000054392	HHAT	-0.577535814	2.09E-06	0.015309909	0.950203	-0.246065279	0.081055	0.598855911	5.81E-07	0.345785015	0.004894	-0.258233853	0.128594	C4	-	Y	-	-	Y	Y	-
ENSG00000054598	FOXC1	-0.573931181	2.35E-17	0.146881416	0.191261	-0.115676667	0.343692	0.727288088	4.66E-19	0.473031323	8.27E-08	-0.260525071	0.065186	C4	-	Y	-	-	Y	Y	-
ENSG00000054611	TBC1D22A	-0.379781521	0.000713	-0.113498853	0.478498	-0.166574431	0.222245	0.272742189	0.019401	0.228233628	0.054624	-0.049932143	0.841204	C4	-	Y	-	-	-	-	-
ENSG00000054690	PLEKHH1	-0.298566307	0.038352	0.044133456	0.835278	0.106621852	0.516405	0.349223684	0.010966	0.419765515	0.001144	0.065706622	0.790494	C4	-	-	-	-	Y	Y	-
ENSG00000054967	RELT	0.543633535	1.24E-07	0.103160551	0.627696	0.143629425	0.32909	-0.433578103	0.000772	-0.385383633	0.00028	0.043348065	0.900498	C5	-	Y	-	-	Y	Y	-
ENSG00000054983	GALC	-0.316097655	0.00027	0.083867704	0.52874	0.05126804	0.690525	0.406654936	1.61E-05	0.382058504	2.18E-05	-0.029840202	0.90074	C4	-	Y	-	-	Y	Y	-
ENSG00000055118	KCNH2	-0.150565864	0.0026	0.328274683	3.19E-09	0.164594518	0.002295	0.485402488	4.62E-29	0.330044058	2.59E-18	-0.160670867	0.00601	C4	-	-	Y	-	Y	Y	-
ENSG00000055147	FAM114A2	-0.421880303	6.95E-06	-0.144222411	0.173443	-0.100552069	0.373335	0.284554051	0.005537	0.336101443	0.000779	0.046524055	0.815904	C4	-	Y	-	-	-	Y	-
ENSG00000055332	EIF2AK2	0.366758779	3.60E-07	0.158977984	0.087501	0.219696413	0.004625	-0.201420138	0.000498	-0.132685571	0.012164	0.06348219	0.512115	C5	-	Y	-	-	-	-	-
ENSG00000055609	KMT2C	0.406111913	0.013931	0.1709139	0.459448	0.197711627	0.280858	-0.228674882	0.127835	-0.194167366	0.111696	0.02950067	0.928793	C5	-	Y	-	-	-	-	-
ENSG00000056586	RC3H2	0.540059131	4.04E-13	0.126895576	0.263206	0.200578519	0.025273	-0.406788956	1.88E-13	-0.324981825	7.35E-12	0.076453856	0.479268	C5	-	Y	-	-	Y	Y	-
ENSG00000056972	TRAF3IP2	-0.901891514	0.006236	0.083793432	0.875571	-0.122009865	0.779664	0.993110045	0.003089	0.793847335	0.015167	-0.202042994	0.732446	C2	-	Y	-	-	Y	Y	-
ENSG00000056998	GYG2	-0.638649275	0.000522	-0.237248317	0.312905	-0.365465607	0.094213	0.407649631	0.058475	0.287798548	0.236819	-0.125070471	0.766388	C4	-	Y	-	-	-	-	-
ENSG00000057019	DCBLD2	0.445763628	1.58E-16	0.371008107	2.65E-07	0.200644586	0.008097	-0.068392195	0.350263	-0.230192919	8.45E-05	-0.16756635	0.09071	C1	-	Y	Y	-	-	-	-
ENSG00000057704	TMCC3	-1.639947182	0.032603	-1.526913404	NA	-0.207831528	0.841087	0.109120335	0.945222	1.448268057	0.118861	1.331010332	NA	C3	-	Y	-	-	-	-	-
ENSG00000058063	ATP11B	0.595117114	4.04E-13	0.40815022	1.84E-05	0.395659804	5.75E-05	-0.181250672	0.007468	-0.184600162	0.01138	-0.009158887	0.96696	C5	-	Y	Y	Y	-	-	-
ENSG00000058091	CDK14	0.244460105	0.001266	-0.192608346	0.038174	-0.02788463	0.851318	-0.430672747	1.56E-10	-0.257903779	0.001444	0.16767871	0.189322	C5	-	-	-	-	Y	-	-
ENSG00000058272	PPP1R12A	0.382733603	3.03E-07	0.117758725	0.309042	0.131147745	0.17191	-0.259053391	1.83E-05	-0.23713882	4.91E-06	0.016570776	0.925299	C5	-	Y	-	-	-	-	-

ENSG00000058404	CAMK2B	-0.650450406	3.21E-08	-0.522723251	0.000157	-0.42162744	0.004169	0.134215193	0.445421	0.243664932	0.136745	0.104057966	0.743693	C4	-	Y	Y	Y	-	-	-
ENSG00000058600	POLR3E	0.422204841	2.02E-12	0.238897295	0.00108	0.172996107	0.016154	-0.176885333	0.006226	-0.23449834	8.06E-05	-0.062979996	0.593651	C5	-	Y	-	-	-	-	-
ENSG00000058668	ATP2B4	-0.943576281	1.65E-17	-0.274587692	0.032752	0.058867048	0.727842	0.67484628	7.24E-13	1.016779437	5.75E-28	0.336554325	0.002438	C4	-	Y	-	-	Y	Y	Y
ENSG00000058673	ZC3H11A	0.380434591	0.0095	0.097060649	0.710865	0.119056276	0.576421	-0.278061836	0.075634	-0.246706288	0.090975	0.02546642	0.951069	C5	-	Y	-	-	-	-	-
ENSG00000058729	RIOK2	0.319372306	0.001595	0.025097809	0.905266	0.127898498	0.356249	-0.288361126	0.00793	-0.177166259	0.085192	0.105950081	0.6074	C5	-	Y	-	-	-	-	-
ENSG00000058866	DGKG	-3.80405706	0.01465	-0.299978434	NA	-0.638271546	NA	3.49040876	NA	3.172221585	NA	-0.327521452	NA	C4	-	Y	-	-	-	-	-
ENSG00000059145	UNKL	0.592354732	3.29E-11	0.207832268	0.083321	0.20644119	0.089432	-0.378289853	1.09E-05	-0.371498319	3.61E-05	0.001429486	0.996048	C5	-	Y	-	-	Y	Y	-
ENSG00000059377	TBXAS1	-0.836129486	0.002046	-0.159436554	0.67936	-0.100426409	0.775106	0.681091662	0.031203	0.750142717	0.009912	0.061938458	0.927911	C3	-	Y	-	-	Y	Y	-
ENSG00000059588	TARBP1	-0.722089898	7.32E-20	-0.092060531	0.28246	-0.149744462	0.03466	0.636434449	2.01E-18	0.586806676	6.49E-17	-0.054769581	0.603402	C4	-	Y	-	-	Y	Y	-
ENSG00000059728	MXD1	0.506934169	7.18E-07	-0.070337237	0.732574	0.12935985	0.405126	-0.570431112	1.26E-07	-0.362806221	0.000421	0.202415276	0.284721	C5	-	Y	-	-	Y	Y	-
ENSG00000059769	DNAJC25	0.338148666	0.003663	-0.103843208	0.620636	-0.036497488	0.864713	-0.435720065	0.001006	-0.35968388	0.005112	0.070046117	0.836385	C5	-	Y	-	-	Y	Y	-
ENSG00000059915	PSD	0.032767182	0.768563	0.30824815	0.000216	0.289963209	0.007521	0.281897751	0.00046	0.271699099	0.005964	-0.01567354	0.95277	C4	-	-	Y	-	-	-	-
ENSG00000060237	WNK1	0.226860861	0.003663	0.34457354	3.85E-05	0.246240404	0.002478	0.124213159	0.078751	0.034004325	0.669142	-0.095478419	0.379545	C1	-	-	Y	-	-	-	-
ENSG00000060566	CREB3L3	-2.570571575	0.006504	-0.869985072	NA	-1.448361104	NA	1.722418802	0.177019	1.132195437	0.450088	-0.586476262	NA	C3	-	Y	-	-	-	-	-
ENSG00000060642	PIGV	-0.575348863	3.17E-05	-0.025116065	0.914982	0.21107927	0.157955	0.556916842	5.60E-05	0.801236702	2.30E-10	0.239693875	0.168996	C4	-	Y	-	-	Y	Y	-
ENSG00000060656	PTPRU	-0.458991728	1.10E-06	-0.398496927	0.00034	-0.127493382	0.317057	0.06719556	0.606069	0.346183499	0.000235	0.273725219	0.03543	C4	-	Y	Y	-	-	Y	-
ENSG00000060718	COL11A1	-0.307341944	0.476927	0.187219811	0.71647	0.349976518	0.388881	0.501074757	0.181679	0.67228427	0.048774	0.166440585	NA	C2	-	-	-	-	-	Y	-
ENSG00000060971	ACAA1	-0.115873942	0.366502	-0.322493257	0.016684	-0.125816013	0.382458	-0.1999904	0.118931	0.004981454	0.973662	0.19942035	0.258544	C4	-	-	Y	-	-	-	-
ENSG00000060982	BCAT1	-0.525839441	1.74E-26	-0.28134033	1.79E-05	-0.154096142	0.007131	0.251178752	4.75E-07	0.386417507	5.56E-25	0.129874714	0.060579	C4	Y	Y	-	-	-	Y	-
ENSG00000061337	LZTS1	-1.05488132	4.27E-09	-0.529178686	0.011423	-0.438924723	0.024098	0.531718003	0.013259	0.630465211	0.001484	0.09298851	0.83038	C3	-	Y	Y	Y	Y	Y	-
ENSG00000061656	SPAG4	0.372064692	0.009484	0.456366048	0.002455	0.401808467	0.009434	0.090796799	0.571905	0.044653555	0.808048	-0.051714524	0.862969	C1	-	Y	Y	Y	-	-	-
ENSG00000061676	NCKAP1	0.304265823	2.97E-09	0.223258594	0.000103	0.125992643	0.048357	-0.074751892	0.094349	-0.163780409	6.75E-05	-0.09436778	0.13614	C5	-	Y	-	-	-	-	-
ENSG00000061794	MRPS35	0.246106228	8.17E-05	-0.093061045	0.317755	-0.084495993	0.365089	-0.332782553	4.05E-08	-0.315622488	3.09E-07	0.011547122	0.951492	C5	-	-	-	-	Y	Y	-
ENSG00000061938	TNK2	0.072330037	0.450942	0.306487254	0.001195	0.340741893	6.01E-05	0.240582037	0.00209	0.283145784	8.33E-05	0.037147072	0.840067	C4	-	-	Y	Y	-	-	-
ENSG00000061987	MON2	0.458933649	9.81E-07	0.161845687	0.196882	0.369768143	0.000193	-0.291014551	6.98E-05	-0.07465437	0.381933	0.210966518	0.024557	C1	Y	Y	-	Y	-	-	-
ENSG00000062282	DGAT2	-1.575493515	0.001333	-1.206862758	0.010097	-0.704184914	0.16217	0.374777021	0.640615	0.885504648	0.186833	0.506002194	NA	C3	-	Y	Y	-	-	-	-
ENSG00000062725	APPBP2	0.26058681	0.000448	-0.093074964	0.432035	0.128054765	0.170469	-0.347066343	6.24E-10	-0.117946375	0.025458	0.223761851	0.003556	C5	-	-	-	-	Y	-	-
ENSG00000063015	SEZ6	-0.317585395	0.001492	-0.545637158	1.07E-07	-0.402000299	0.002312	-0.221279283	0.060697	-0.069960536	0.68002	0.145326391	0.541217	C4	-	Y	Y	Y	-	-	-
ENSG00000063169	BICRA	0.311667591	0.007643	0.191445205	0.208293	0.13463057	0.397818	-0.113897124	0.393747	-0.162274462	0.197234	-0.05384516	0.841305	C5	-	Y	-	-	-	-	-
ENSG00000063241	ISOC2	-0.433427662	0.000131	-0.31354874	0.029506	-0.127549742	0.440594	0.126120182	0.32248	0.320819721	0.002375	0.189260569	0.291851	C4	-	Y	Y	-	-	Y	-
ENSG00000063244	U2AF2	0.264044214	5.68E-11	-0.106171647	0.101684	-0.084346409	0.166776	-0.363740855	2.30E-20	-0.333785287	2.61E-22	0.024752304	0.825762	C5	-	-	-	-	Y	Y	-
ENSG00000063438	AHRR	1.332397676	0.016063	0.34444813	NA	-0.525926201	NA	-0.986652551	0.10649	-1.840571783	0.004099	-0.871588295	NA	C5	-	Y	-	-	-	Y	-
ENSG00000063601	MTMR1	0.316305235	2.22E-06	0.033844773	0.819271	0.083454372	0.420939	-0.276046951	0.000612	-0.218262763	0.002991	0.052615326	0.785782	C5	-	Y	-	-	-	-	-
ENSG00000063761	ADCK1	-0.531054788	0.013993	-0.160220317	0.64799	-0.272407036	0.286824	0.376736822	0.145014	0.272854049	0.264308	-0.109988744	0.83038	C4	-	Y	-	-	-	-	-
ENSG00000064102	INTS13	0.531317216	6.30E-10	0.168818661	0.153956	0.380840987	0.000149	-0.356211115	2.70E-05	-0.135539078	0.171418	0.214919463	0.095679	C5	-	Y	-	Y	Y	-	-
ENSG00000064199	SPA17	-0.4817031	1.13E-05	-0.033301062	0.861093	-0.177781809	0.147796	0.455268556	8.68E-05	0.318555251	0.005926	-0.141320294	0.43015	C4	-	Y	-	-	Y	Y	-
ENSG00000064201	TSPAN32	-1.430686154	0.00017	-0.284532188	0.580558	-0.282111252	0.538088	1.153039234	0.009283	1.162964245	0.006493	0.004973661	NA	C3	-	Y	-	-	Y	Y	-
ENSG00000064225	ST3GAL6	0.32688292	3.53E-10	0.344526763	3.85E-06	0.258541903	0.001518	0.024015323	0.786157	-0.05338223	0.517235	-0.083451282	0.572189	C4	-	Y	Y	-	-	-	-
ENSG00000064300	NGFR	-0.227552483	0.203098	-0.542274823	0.002034	-0.445863016	0.00576	-0.309063719	0.114936	-0.20364774	0.294347	0.099427796	0.784089	C5	-	-	Y	Y	-	-	-
ENSG00000064313	TAF2	0.325808311	2.62E-06	-0.002311551	0.989977	-0.080190256	0.415713	-0.321394582	3.98E-06	-0.391507193	6.80E-13	-0.075346118	0.591008	C5	-	Y	-	-	Y	Y	-
ENSG00000064393	HIPK2	0.579942945	1.43E-12	0.183759136	0.100629	0.274090344	0.004185	-0.389734842	1.04E-10	-0.291423735	2.82E-07	0.092969826	0.421345	C5	-	Y	-	-	Y	-	-
ENSG00000064490	RFXANK	-0.384169864	0.000157	-0.261022866	0.04597	-0.149854195	0.262673	0.129679376	0.263232	0.249403498	0.01082	0.114175619	0.524775	C4	-	Y	-	-	-	-	-
ENSG00000064607	SUGP2	0.254557421	1.31E-07	0.339409801	3.41E-12	0.341414412	2.55E-11	0.091384636	0.027296	0.101515277	0.020982	0.004791983	0.967582	C4	-	-	Y	Y	-	-	-
ENSG00000064651	SLC12A2	0.440413608	3.93E-08	0.076093691	0.687775	0.073543558	0.601315	-0.357433723	0.000867	-0.351793802	4.35E-05	0.000131875	0.99962	C5	-	Y	-	-	Y	Y	-
ENSG00000064666	CNN2	1.049168938	4.18E-23	0.229176861	0.176038	0.262421616	0.068221	-0.813965177	1.71E-15	-0.772055113	1.09E-16	0.036743567	0.904354	C5	Y	Y	-	-	Y	Y	-
ENSG00000064687	ABCA7	0.318709146	0.156749	0.584695917	0.007303	0.809649948	6.05E-05	0.2729623	0.147678	0.505998359	0.003412	0.227820285	0.40086	C4	-	-	Y	Y	-	Y	-
ENSG00000064703	DDX20	0.080567286	0.289145	-0.341450771	1.97E-05	-0.160611125	0.042076	-0.41542728	3.42E-09	-0.226401716	0.000791	0.183657519	0.080981	C5	-	-	Y	-	Y	-	-
ENSG00000064999	ANKS1A	0.490623488	8.95E-19	0.205709735	0.008618	0.243797797	0.00109	-0.278272293	2.28E-06	-0.232033944	0.000111	0.040978483	0.790777	C5	-	Y	-	-	-	-	-
ENSG00000065029	ZNF76	-0.534393301	1.54E-07	-0.12039165	0.369816	-0.158011756	0.189582	0.420617066	3.26E-05	0.391394393	0.000138	-0.034523873	0.882986	C4	-	Y	-	-	Y	Y	-
ENSG00000065243	PKN2	0.531707979	4.30E-10	0.146906358	0.154011	0.234038692	0.009714	-0.378569168	7.87E-08	-0.283338526	8.03E-05	0.089542313	0.423104	C5	-	Y	-	-	Y	-	-
ENSG00000065413	ANKRD44	0.235489058	0.001213	-0.101082903	0.363401	-0.020304855	0.865662	-0.330138274	2.13E-08	-0.241697576	5.73E-07	0.083487144	0.437736	C5	-	-	-	-	Y	-	-
ENSG00000065457	ADAT1	-0.370676525	1.53E-06	0.053216981	0.644573	-0.074296915	0.443372	0.430294351	1.07E-10	0.310921769	2.02E-06	-0.124513294	0.168972	C4	-	Y	-	-	Y	Y	-
ENSG00000065485	PDIA5	-0.537409097	0.001612	0.001443363	0.996745	-0.323452684	0.07653	0.54533976	0.002994	0.228524533	0.236386	-0.321686253	0.19895	C4	-	Y	-	-	Y	-	-
ENSG00000065526	SPEN	0.335939347	8.53E-05	0.200112198	0.066877	0.157915389	0.123014	-0.129605241	0.049646	-0.163680324	0.002678	-0.039327088	0.768922	C1	-	Y	-	-	-	-	-
ENSG00000065534	MYLK	-0.529653193	0.000129	-0.221562288	0.397458	-0.190029575	0.360296	0.315526745	0.160024	0.354416359	0.054779	0.032363402	0.956814	C2	-	Y	-	-	-	-	-
ENSG00000065600	PACC1	0.524822447	2.43E-18	0.147327968	0.080777	0.229166229	0.015894	-0.371088989	4.02E-10	-0.281003405	0.000138	0.084722944	0.572708	C5	-	Y	-	-	Y	-	-
ENSG00000065613	SLK	0.365141143	1.12E-05	0.170068502	0.143907	0.111409269	0.379471	-0.188517657	0.013716	-0.238788836	0.001856	-0.055645808	0.758828	C5	-	Y	-	-	-	-	-
ENSG00000065665	SEC61A2	-0.244881233	0.008071	0.053210334	0.711724	0.008637049	0.956889	0.304117447	3.80E-05	0.268252572	0.000366	-0.041189523	0.796431	C4	-	-	-	-	Y	-	-
ENSG00000065675	PRKCQ	-0.587352441	0.010194	-0.232911632	0.489392	-0.124083741	0.715469	0.360394303	0.229094	0.478155426	0.079323	0.113578857	0.853204	C4	-	Y	-	-	-	-	-
ENSG00000065833	ME1	0.180004657	0.023064	-0.228186471	0.030863	-0.3070046	0.000584	-0.401324234	1.05E-06	-0.472273631	4.13E-10	-0.075763668	0.67686	C5	-	-	-	Y	Y	Y	-
ENSG00000065883	CDK13	0.329552212	1.15E-08	0.089336453	0.268407	0.08626195	0.234189	-0.23377256	2.79E-05	-0.228799278	1.25E-05	-0.000432741	0.997573	C5	Y	Y	-	-	-	-	-
ENSG00000065911	MTHFD2	-0.600567526	9.84E-37	-0.495183693	3.85E-11	-0.40777855	2.62E-10	0.11218599	0.124205	0.207295187	0.000158	0.090524028	0.510095	C4	-	Y	Y	Y	-	-	-
ENSG00000065970	FOXJ2	0.542472828	7.82E-08	0.300742808	0.012506	0.28012583	0.013115	-0.235743525	0.010559	-0.24807755	0.004986	-0.017762185	0.940867	C1	-	Y	Y	-	-	-	-
ENSG00000065989	PDE4A	-0.72895271	0.010061	-0.166286159	0.707693	0.077124619	0.83963	0.568690582	0.07454	0.820988622	0.001491	0.245507314	0.601092	C4	-	Y	-	-	-	Y	-
ENSG00000066027	PPP2R5A	0.650804504	5.93E-08	0.28773807	0.049953	0.500826087	9.85E-05	-0.35682032	0.00266	-0.13533972	0.318943	0.216234581	0.210562	C5	-	Y	-	Y	Y	-	-
ENSG00000066084	DIP2B	0.511322979	7.97E-13	0.163862302	0.080384	0.094421465	0.347561	-0.341323559	1.65E-09	-0.402377139	1.06E-13	-0.066402896	0.551046	C5	-	Y	-	-	Y	Y	-
ENSG00000066136	NFYC	-0.38885992	7.95E-07	-0.101432309	0.379819	-0.157411872	0.111455	0.293799824	0.000239	0.246364937	0.00217	-0.052770371	0.755132	C4	-	Y	-	-	-	-	-

ENSG00000066248	NGEF	1.085029652	0.359021	-1.525474375	NA	0.501914557	NA	-2.576449363	0.028482	-0.564053275	0.591085	2.015203736	NA	C5	-	-	-	-	Y	-	-
ENSG00000066379	ZNRD1	0.487669035	0.000127	0.054239329	0.81375	0.069694194	0.729709	-0.427142916	0.000804	-0.403290632	0.001517	0.018173255	0.959572	C5	-	Y	-	-	Y	Y	-
ENSG00000066382	MPPED2	-0.669957562	0.000103	-0.383511836	0.05104	0.066524175	0.786507	0.292438285	0.135273	0.750836372	3.22E-06	0.45265813	0.020684	C4	-	Y	-	-	-	Y	Y
ENSG00000066422	ZBTB11	0.285138827	8.74E-05	-0.214037372	0.04882	0.079304723	0.434998	-0.492678073	1.37E-10	-0.191402042	0.003955	0.296222563	0.002862	C5	-	-	-	-	Y	-	-
ENSG00000066427	ATXN3	0.126572721	0.20936	-0.193204429	0.091587	-0.02926313	0.843948	-0.313334642	0.000595	-0.141125915	0.153711	0.166891745	0.228015	C4	-	-	-	-	Y	-	-
ENSG00000066468	FGFR2	-1.620750738	0.002792	-0.429284717	0.496752	-0.484252984	0.4099	1.204242978	0.075534	1.150617863	0.092683	-0.055978137	NA	C3	-	Y	-	-	-	-	-
ENSG00000066651	TRMT11	0.216336908	0.020652	0.060902872	0.731976	-0.117570498	0.354502	-0.14888757	0.19419	-0.319426228	0.000378	-0.175628727	0.307349	C5	-	-	-	-	-	Y	-
ENSG00000066735	KIF26A	0.322259017	5.07E-08	0.272705561	8.57E-05	0.082477671	0.251374	-0.042783679	0.534208	-0.224771911	2.90E-07	-0.187577372	0.001605	C5	-	Y	-	-	-	-	-
ENSG00000066739	ATG2B	0.391889195	3.11E-05	0.215662122	0.04882	0.185672138	0.060305	-0.170147428	0.034103	-0.191896278	0.005378	-0.026478584	0.861463	C5	-	Y	-	-	-	-	-
ENSG00000066777	ARFGEF1	0.563780566	1.19E-14	0.08747198	0.463171	0.204364155	0.021565	-0.469891711	8.52E-17	-0.344849295	1.27E-10	0.119661215	0.19895	C5	-	Y	-	-	Y	Y	-
ENSG00000066827	ZFAT	0.341475548	0.071476	0.365617329	0.049986	0.179442693	0.428048	0.030753565	0.896627	-0.147522735	0.477586	-0.183551431	0.488454	C1	-	-	Y	-	-	-	-
ENSG00000066855	MTFR1	0.396107644	3.37E-05	0.090302595	0.562049	0.093586966	0.522556	-0.299239564	0.002923	-0.288006468	0.004389	0.006266141	0.982455	C5	-	Y	-	-	-	-	-
ENSG00000066923	STAG3	0.691349501	2.68E-11	0.324706933	0.007528	0.570144677	8.05E-07	-0.360604199	9.97E-05	-0.106989829	0.362629	0.248144964	0.065114	C1	-	Y	Y	Y	Y	-	-
ENSG00000066933	MYO9A	0.568628608	1.03E-06	0.302847498	0.043936	0.412813048	0.000445	-0.259690772	0.009352	-0.141573803	0.097581	0.113050382	0.460404	C1	-	Y	Y	Y	-	-	-
ENSG00000067082	KLF6	0.486894943	9.15E-11	0.893957354	1.25E-22	0.480234834	2.24E-07	0.413623227	1.87E-07	0.008532847	0.941288	-0.410810514	9.07E-05	C4	-	Y	Y	Y	Y	-	Y
ENSG00000067113	PLPP1	-0.608932669	0.001344	-0.245807231	0.297516	-0.400297621	0.047336	0.37026231	0.10203	0.223349392	0.367367	-0.151776668	0.682049	C2	-	Y	-	Y	-	-	-
ENSG00000067167	TRAM1	0.497016848	6.84E-18	0.01442862	0.905874	0.134144194	0.118791	-0.4762015	1.69E-15	-0.348275385	3.09E-08	0.122604512	0.286084	C5	-	Y	-	-	Y	Y	-
ENSG00000067369	TP53BP1	0.393447464	3.63E-13	0.249441359	0.000366	0.261905759	3.67E-05	-0.13772594	0.004249	-0.116921239	0.007062	0.015409754	0.910372	C1	-	Y	-	-	-	-	-
ENSG00000067601	PMS2P4	-0.650947267	0.003838	-0.075767105	0.826819	-0.240743118	0.400814	0.581800821	0.007119	0.425465853	0.082691	-0.161639169	0.67888	C4	-	Y	-	-	Y	-	-
ENSG00000067900	ROCK1	0.489155646	2.33E-13	0.322861079	0.000179	0.194343683	0.01682	-0.159893076	0.010822	-0.280233201	2.64E-08	-0.12567868	0.17701	C1	-	Y	Y	-	-	-	-
ENSG00000067955	CBFB	0.362095691	1.52E-12	0.108176753	0.196014	-0.033724011	0.699906	-0.247627694	6.45E-06	-0.381106419	1.76E-16	-0.138965763	0.099224	C5	-	Y	-	-	-	Y	-
ENSG00000068001	HYAL2	-0.321708714	0.000747	-0.06392981	0.688983	-0.246095664	0.028108	0.264644164	0.003557	0.090517092	0.398735	-0.179496207	0.189051	C4	-	Y	-	-	-	-	-
ENSG00000068024	HDAC4	0.309115523	8.48E-05	0.056927345	0.741101	0.147827771	0.139933	-0.245619407	0.01995	-0.146645784	0.095964	0.093797127	0.639479	C5	-	Y	-	-	-	-	-
ENSG00000068078	FGFR3	-0.330907762	0.031041	-0.510507894	0.004085	-0.204019226	0.269471	-0.172191111	0.405546	0.141988463	0.453663	0.308916954	0.215908	C4	-	Y	Y	-	-	-	-
ENSG00000068097	HEATR6	0.176135094	0.002799	-0.137086229	0.071026	-0.086599118	0.303086	-0.306799415	5.28E-08	-0.24816024	1.79E-05	0.053332931	0.674578	C5	-	-	-	-	Y	-	-
ENSG00000068366	ACSL4	0.41493107	3.57E-09	0.273297332	0.005932	0.239036858	0.005042	-0.135296344	0.112293	-0.16113786	0.022615	-0.031386234	0.878258	C1	-	Y	-	-	-	-	-
ENSG00000068784	SRBD1	-0.798200421	4.29E-20	-0.121608405	0.313631	-0.143609545	0.169688	0.683074588	1.37E-16	0.669285879	1.33E-17	-0.019014959	0.931126	C4	-	Y	-	-	Y	Y	-
ENSG00000068878	PSME4	0.386288884	1.81E-11	0.11307863	0.176589	-0.043268824	0.646524	-0.266534122	9.96E-08	-0.414929718	4.89E-19	-0.153715007	0.040472	C5	-	Y	-	-	-	Y	-
ENSG00000068885	IFT80	-0.313805783	5.86E-05	-0.024242021	0.854325	-0.017717997	0.889326	0.295998492	9.51E-05	0.310752619	3.91E-05	0.00946192	0.961151	C4	-	Y	-	-	-	Y	-
ENSG00000068903	SIRT2	0.316430931	0.002042	0.307656669	0.001929	0.391388661	1.59E-05	-0.002404737	0.986223	0.089683797	0.436699	0.086648757	0.573048	C5	-	Y	Y	Y	-	-	-
ENSG00000068971	PPP2R5B	0.72268823	3.27E-21	0.413164321	1.04E-05	0.44694396	4.26E-07	-0.303299883	8.43E-05	-0.261077207	0.000606	0.036780062	0.849099	C5	-	Y	Y	Y	Y	-	-
ENSG00000069188	SDK2	0.413379127	0.427608	1.291544357	0.001587	1.350310236	0.000827	0.884905185	0.013726	0.952748741	0.008797	0.062047473	NA	C2	-	-	Y	Y	Y	Y	-
ENSG00000069345	DNAJA2	0.321250527	2.02E-09	0.023601722	0.810992	0.092898951	0.255641	-0.291270456	2.09E-08	-0.213900443	0.000237	0.072244851	0.519014	C5	-	Y	-	-	-	-	-
ENSG00000069482	GAL	-0.013413056	0.949117	-0.343069541	0.037719	-0.414801318	0.007006	-0.322978274	0.015549	-0.38668587	0.002592	-0.068643508	0.838445	C5	-	-	Y	Y	Y	Y	-
ENSG00000069509	FUNDC1	-0.391130293	0.000475	-0.204655488	0.128914	-0.332486773	0.006852	0.192904376	0.116955	0.073334842	0.62828	-0.124939631	0.524775	C4	-	Y	-	Y	-	-	-
ENSG00000069535	MAOB	-0.796702185	1.78E-08	-0.20226242	0.288445	-0.228359403	0.220309	0.601442093	0.00024	0.582903384	0.000647	-0.02386073	0.95884	C4	-	Y	-	-	Y	Y	-
ENSG00000069667	RORA	-0.247482217	0.049408	0.213276095	0.189627	-0.014133855	0.956427	0.46763959	0.000414	0.248435904	0.127562	-0.22584426	0.375095	C2	-	-	-	-	Y	-	-
ENSG00000069869	NEDD4	0.490485212	4.27E-06	0.043071099	0.829709	0.140390437	0.360963	-0.441304248	1.49E-05	-0.33551091	0.001444	0.100312098	0.630392	C5	-	Y	-	-	Y	Y	-
ENSG00000069943	PIGB	-0.388147245	0.084963	0.11020244	0.739311	0.090988211	0.764042	0.50487171	0.022747	0.493872354	0.02177	-0.016231474	0.977492	C4	-	-	-	-	Y	Y	-
ENSG00000070061	ELP1	0.353365285	2.98E-10	0.238590331	0.000264	0.225573165	0.000257	-0.108129206	0.026694	-0.113303242	0.013462	-0.010535276	0.933843	C5	-	Y	-	-	-	-	-
ENSG00000070269	TMEM260	-0.2376502	0.037243	0.086826557	0.605228	0.005544709	0.976291	0.331012021	0.006276	0.258029331	0.025847	-0.078317652	0.73031	C4	-	-	-	-	Y	-	-
ENSG00000070367	EXOC5	0.448119159	9.17E-23	0.120068959	0.136387	0.139521405	0.015105	-0.321297606	1.40E-10	-0.294000436	6.58E-15	0.022087862	0.87281	C5	-	Y	-	-	Y	-	-
ENSG00000070371	CLTCL1	0.009949671	0.967332	0.144146507	0.521807	0.404267391	0.009259	0.14044513	0.45747	0.408658654	0.00443	0.263641356	0.213671	C4	-	-	-	Y	-	Y	-
ENSG00000070388	FGF22	-0.634668357	0.324731	0.174414115	0.827392	0.481868953	0.437964	0.818035829	0.146854	1.132552301	0.029666	0.312433322	NA	C2	-	-	-	-	-	Y	-
ENSG00000070404	FSTL3	0.171879355	0.281266	0.502020286	0.000575	0.535977148	0.000171	0.336687459	0.017583	0.37884583	0.007106	0.036899932	0.914963	C4	-	-	Y	Y	Y	Y	-
ENSG00000070444	MNT	-0.809808241	3.36E-18	-0.317479925	0.001858	-0.325209579	0.001255	0.49877275	1.61E-08	0.498973273	2.34E-08	-0.005089317	0.982475	C4	-	Y	Y	Y	Y	Y	-
ENSG00000070495	JMJD6	0.81068461	6.32E-38	0.253182256	0.003498	0.478204752	7.58E-12	-0.550891001	8.16E-17	-0.317940783	3.58E-07	0.22782561	0.015467	C5	-	Y	-	Y	Y	Y	-
ENSG00000070610	GBA2	0.366731799	1.76E-12	0.102104149	0.198056	0.154066828	0.019425	-0.257834331	4.65E-07	-0.198071821	4.27E-05	0.054718984	0.609969	C5	-	Y	-	-	-	-	-
ENSG00000070669	ASNS	-0.322084335	0.014555	-0.245804143	0.199493	-0.126642039	0.450439	0.081800756	0.718385	0.210480624	0.1939	0.123569099	0.70884	C4	-	Y	-	-	-	-	-
ENSG00000070770	CSNK2A2	0.352938915	2.80E-05	0.062839804	0.664564	0.111180017	0.32909	-0.283580135	0.001085	-0.227346645	0.006637	0.051021749	0.793199	C5	-	Y	-	-	-	-	-
ENSG00000070778	PTPN21	0.416007751	2.69E-10	0.238387465	0.005932	0.212935521	0.007421	-0.171619259	0.006988	-0.188752149	0.000822	-0.022383187	0.893051	C5	-	Y	-	-	-	-	-
ENSG00000070814	TCOF1	0.313805905	3.06E-16	0.121282646	0.033313	0.082046429	0.154703	-0.185923836	5.07E-06	-0.217200599	1.74E-08	-0.036389446	0.709413	C5	-	Y	-	-	-	-	-
ENSG00000070882	OSBPL3	0.558362714	1.45E-05	0.122360585	0.657458	0.111347296	0.583054	-0.42861095	0.008751	-0.432011449	0.00082	-0.008443718	0.986496	C5	-	Y	-	-	Y	Y	-
ENSG00000070886	EPHA8	-0.735680323	0.000226	-0.294822573	0.236365	-0.127337619	0.652339	0.447214404	0.049712	0.622980922	0.003977	0.170385029	0.655393	C4	-	Y	-	-	Y	Y	-
ENSG00000070915	SLC12A3	-1.269600615	0.032645	0.195100868	0.785122	0.220818602	0.753549	1.469481417	0.004819	1.503921316	0.006526	0.028148382	NA	C3	-	Y	-	-	Y	Y	-
ENSG00000070961	ATP2B1	0.312823166	8.95E-06	0.288284079	0.000458	0.240022608	0.004162	-0.018418228	0.826941	-0.058311348	0.415702	-0.045262908	0.727041	C4	-	Y	-	-	-	-	-
ENSG00000071073	MGAT4A	0.632438477	9.01E-08	0.322153167	0.024215	0.330120842	0.027863	-0.304122681	0.020936	-0.287646137	0.039963	0.011472012	0.977318	C5	-	Y	Y	Y	Y	-	-
ENSG00000071127	WDR1	0.842477998	1.78E-78	0.304285858	7.64E-07	0.271386691	3.90E-07	-0.531978798	1.01E-29	-0.556190097	6.10E-44	-0.029795385	0.805117	C5	Y	Y	Y	-	Y	Y	-
ENSG00000071189	SNX13	0.318475358	2.84E-05	0.266543635	0.005247	0.185939271	0.041043	-0.044979498	0.596411	-0.118173823	0.074803	-0.078465265	0.52139	C5	-	Y	-	-	-	-	-
ENSG00000071205	ARHGAP10	-0.558080451	0.007968	-0.357205109	0.105259	-0.492627652	0.015875	0.206673562	0.463822	0.079908805	0.807671	-0.132245297	0.757663	C3	-	Y	-	Y	-	-	-
ENSG00000071242	RPS6KA2	-0.594480608	2.53E-22	-0.030749975	0.777818	0.023379684	0.823781	0.570101469	6.36E-21	0.632510752	2.29E-26	0.057081729	0.638566	C2	-	Y	-	-	Y	Y	-
ENSG00000071243	ING3	-0.157825637	0.06319	-0.199282012	0.057265	-0.469209207	5.53E-09	-0.034833461	0.771592	-0.296635846	0.000198	-0.267165129	0.01558	C5	-	-	-	Y	-	-	-
ENSG00000071246	VASH1	0.122827164	0.178798	0.110409651	0.33902	0.316733454	5.02E-05	-0.005866145	0.963906	0.208583998	0.007972	0.209307016	0.046118	C4	-	-	-	Y	-	-	-
ENSG00000071282	LMCD1	-0.023354413	0.898908	0.299103369	0.025252	0.195040936	0.215039	0.328715687	0.005707	0.232751404	0.091587	-0.101111922	0.662988	C4	-	-	-	-	Y	-	-
ENSG00000071564	TCF3	0.238168106	9.40E-08	-0.000312656	0.997464	-0.079677217	0.227984	-0.231929741	1.08E-07	-0.303196191	9.39E-15	-0.076520599	0.36959	C5	-	-	-	-	-	Y	-
ENSG00000071575	TRIB2	0.45669847	2.77E-13	0.302429465	9.55E-06	0.167739966	0.04484	-0.147796302	0.032301	-0.27415674	7.75E-05	-0.131864172	0.219232	C5	-	Y	Y	-	-	-	-
ENSG00000072134	EPN2	0.600838413	2.43E-18	0.257987472	0.003763	0.313003914	0.000325	-0.336452331	3.52E-07	-0.273350628	8.53E-05	0.057818806	0.715621	C5	-	Y	-	Y	Y	-	-

ENSG00000072135	PTPN18	-0.273281941	0.002711	0.11666855	0.404484	0.090818812	0.485976	0.396605384	8.73E-05	0.378816178	6.75E-05	-0.02338849	0.935372	C4	-	-	-	-	Y	Y	-
ENSG00000072182	ASIC4	-0.670646858	3.31E-08	-0.461159364	0.002878	-0.196435266	0.190781	0.216067779	0.146216	0.488936756	1.74E-05	0.267255803	0.148991	C4	-	Y	Y	-	-	Y	-
ENSG00000072195	SPEG	-0.182016602	0.024345	0.036701078	0.774478	0.189373844	0.020612	0.225324024	0.001744	0.386059012	2.18E-09	0.155219059	0.092592	C4	-	-	-	-	-	Y	-
ENSG00000072210	ALDH3A2	-0.388890391	3.19E-10	-0.105137921	0.216671	-0.019806369	0.841691	0.290209369	9.38E-06	0.383741995	1.26E-10	0.088082571	0.39673	C4	-	Y	-	-	-	Y	-
ENSG00000072274	TFRC	-0.505633286	3.07E-18	0.10618203	0.189138	0.378241296	1.81E-10	0.618231472	4.22E-51	0.898475275	3.86E-126	0.274832211	9.34E-09	C4	-	Y	-	Y	Y	Y	-
ENSG00000072310	SREBF1	-0.768477918	4.12E-14	-0.416452449	0.000656	-0.324834628	0.002175	0.35844436	0.001858	0.458554871	2.75E-06	0.094269884	0.634587	C4	-	Y	Y	Y	Y	Y	-
ENSG00000072364	AFF4	0.361283661	1.04E-05	0.143788753	0.198625	0.177409042	0.073262	-0.211376244	0.000206	-0.169461199	0.001909	0.036582142	0.781564	C5	-	Y	-	-	-	-	-
ENSG00000072401	UBE2D1	0.31163229	7.02E-06	-0.052764617	0.651968	0.068287457	0.531017	-0.358061941	2.04E-07	-0.228666523	0.001815	0.123950035	0.303482	C5	-	Y	-	-	Y	-	-
ENSG00000072422	RHOBTB1	-0.6634245	1.70E-14	-0.259500876	0.007298	-0.148626729	0.12922	0.410395927	7.34E-06	0.529557911	5.62E-10	0.113925915	0.407412	C4	-	Y	-	-	Y	Y	-
ENSG00000072518	MARK2	0.411855655	1.33E-09	0.230254856	0.009355	0.300855791	5.86E-05	-0.175107347	0.00986	-0.096523908	0.157452	0.073162875	0.549271	C5	-	Y	-	Y	-	-	-
ENSG00000072571	HMMR	-0.531984154	6.32E-16	-0.028284723	0.820576	-0.146179317	0.148727	0.509968997	4.05E-16	0.40067271	6.31E-08	-0.115178954	0.393747	C4	-	Y	-	-	Y	Y	-
ENSG00000072682	P4HA2	-0.108940254	0.48974	0.327273894	0.068181	0.561461058	1.57E-05	0.442217331	0.001744	0.685623602	9.95E-10	0.237726746	0.25635	C4	-	-	-	Y	Y	Y	-
ENSG00000072736	NFATC3	0.391112956	3.76E-06	0.325058132	0.005809	0.305405144	0.000952	-0.060164022	0.582928	-0.071350714	0.358351	-0.016395694	0.944407	C1	-	Y	Y	Y	-	-	-
ENSG00000072756	TRNT1	0.522656296	7.51E-09	0.105523778	0.41347	0.264744893	0.006078	-0.41074413	2.17E-06	-0.243277498	0.004749	0.162139345	0.186664	C5	-	Y	-	-	Y	-	-
ENSG00000072803	FBXW11	0.403255981	1.13E-14	0.253401579	1.19E-05	0.197391126	0.000369	-0.14337292	0.003341	-0.191313192	1.47E-05	-0.053349437	0.511403	C5	-	Y	-	-	-	-	-
ENSG00000072840	EVC	-0.420417621	0.003169	-0.015783876	0.952296	0.083223967	0.674503	0.410291922	0.000889	0.517762749	1.08E-05	0.102272726	0.6433	C4	-	Y	-	-	Y	Y	-
ENSG00000072952	IRAG1	-0.523146266	0.000756	-0.099175645	0.669056	-0.237835653	0.202861	0.430296374	0.005949	0.299816302	0.080017	-0.135590465	0.621168	C4	-	Y	-	-	Y	-	-
ENSG00000073008	PVR	0.498230501	9.28E-14	0.291811484	0.000119	0.358933676	1.27E-06	-0.200117875	0.002769	-0.124818075	0.085281	0.06995087	0.572524	C5	-	Y	-	Y	-	-	-
ENSG00000073060	SCARB1	-0.168334717	0.056564	0.127002573	0.219667	0.245245819	0.004399	0.301708296	0.000171	0.428223043	1.82E-08	0.121109039	0.316749	C4	-	-	-	-	Y	Y	-
ENSG00000073067	CYP2W1	-0.61940095	0.408097	0.512153974	0.499063	0.816337349	0.15743	1.14059929	0.10179	1.449607878	0.017152	0.307280242	NA	C3	-	-	-	-	-	Y	-
ENSG00000073146	MOV10L1	-1.6413502	0.016723	-0.056318325	0.954846	0.592233421	0.296854	1.59377386	0.038883	2.248474856	0.000347	0.652347488	NA	C2	-	Y	-	-	Y	Y	-
ENSG00000073150	PANX2	-0.250507598	0.32929	-0.485136254	0.08	-0.59823378	0.01804	-0.228755901	0.4214	-0.333620589	0.207389	-0.110378407	0.843071	C4	-	-	-	Y	-	-	-
ENSG00000073350	LLGL2	-0.257965871	0.361996	-0.016561473	0.968802	0.266308305	0.331603	0.249397352	0.397124	0.538383134	0.028483	0.285621215	0.435383	C4	-	-	-	-	-	Y	-
ENSG00000073417	PDE8A	-0.1944291	0.020664	0.041919668	0.776034	0.103790818	0.404746	0.242778949	0.007146	0.312972276	0.000605	0.064243955	0.756774	C4	-	-	-	-	-	Y	-
ENSG00000073464	CLCN4	-0.579132644	2.66E-12	-0.017271728	0.903779	0.065298676	0.577244	0.568676051	9.42E-11	0.65903963	3.61E-14	0.085237028	0.592369	C2	-	Y	-	-	Y	Y	-
ENSG00000073536	NLE1	0.233714867	0.021928	-0.082779907	0.546021	-0.058998977	0.679185	-0.310076363	0.000991	-0.277863685	0.004156	0.026637569	0.910675	C5	-	-	-	-	Y	-	-
ENSG00000073605	GSDMB	0.677080664	1.95E-06	0.36169944	0.033462	0.317969513	0.07139	-0.308938517	0.034733	-0.345134356	0.020095	-0.04108315	0.913257	C5	-	Y	Y	-	Y	Y	-
ENSG00000073711	PPP2R3A	-0.284306001	0.038588	-0.361984054	0.005765	-0.132017621	0.407946	-0.071609884	0.666777	0.166539519	0.248561	0.232820266	0.158463	C4	-	-	Y	-	-	-	-
ENSG00000073712	FERMT2	0.447021686	1.54E-20	0.256306981	0.000103	0.207156769	0.000539	-0.184491217	0.000103	-0.225058884	1.30E-08	-0.046067495	0.653289	C1	-	Y	-	-	-	-	-
ENSG00000073910	FRY	-0.425211747	1.07E-05	0.024869085	0.888768	0.172227428	0.213229	0.45630205	2.66E-07	0.61198179	3.96E-10	0.150063505	0.387475	C4	-	Y	-	-	Y	Y	-
ENSG00000073921	PICALM	0.2784793	2.98E-09	0.360485379	2.25E-10	0.226372052	2.47E-05	0.088481355	0.116037	-0.037383229	0.514352	-0.131177669	0.069496	C4	-	-	Y	-	-	-	-
ENSG00000074047	GLI2	-1.291345203	3.06E-05	-0.708801135	0.027636	-0.421113959	0.203609	0.588549692	0.161578	0.884992664	0.017393	0.290426484	NA	C3	-	Y	Y	-	-	Y	-
ENSG00000074181	NOTCH3	-0.68264696	1.13E-13	-0.48529452	1.42E-06	-0.226332892	0.026713	0.204065787	0.076083	0.470907782	6.37E-07	0.261754342	0.044614	C4	-	Y	Y	-	-	Y	-
ENSG00000074211	PPP2R2C	-0.143324729	0.182664	-0.423517997	0.000818	-0.14483279	0.321839	-0.274064786	0.022959	0.012769418	0.938728	0.281646603	0.128594	C4	-	-	Y	-	-	-	-
ENSG00000074266	EED	0.245579495	0.003794	-0.120480959	0.330977	-0.108484845	0.316226	-0.359503973	5.15E-05	-0.339460363	2.61E-05	0.014916436	0.952524	C5	-	-	-	-	Y	Y	-
ENSG00000074370	ATP2A3	-1.059069733	5.11E-08	-0.908832198	0.000188	-0.186337041	0.444186	0.159194668	0.659105	0.887370408	3.50E-05	0.724819632	0.018074	C3	-	Y	Y	-	-	Y	Y
ENSG00000074416	MGLL	-1.038391274	0.002629	-0.091757298	0.858333	0.129393548	0.790231	0.953153253	0.006087	1.182897443	0.000793	0.224084078	NA	C4	-	Y	-	-	Y	Y	-
ENSG00000074527	NTN4	-1.369948225	0.010444	-1.319863661	0.011181	-1.309503828	0.009187	0.058087001	0.954876	0.076604703	0.93793	0.013386368	NA	C3	-	Y	Y	Y	-	-	-
ENSG00000074582	BCS1L	0.185732627	0.018268	-0.140983698	0.222912	-0.060163184	0.576052	-0.320302238	0.000185	-0.231257291	0.002317	0.083507978	0.608319	C5	-	-	-	-	Y	-	-
ENSG00000074590	NUAK1	0.823339588	0.000226	1.463392468	9.23E-12	1.416714214	1.36E-11	0.646805501	0.001801	0.608263269	0.002831	-0.043679985	0.935133	C1	-	Y	Y	Y	Y	Y	-
ENSG00000074603	DPP8	0.202347925	0.002266	-0.077296668	0.446284	-0.146890658	0.089024	-0.273087573	9.40E-06	-0.334544324	6.30E-08	-0.066752614	0.622141	C5	-	-	-	-	-	Y	-
ENSG00000074621	SLC24A1	-0.789995929	2.11E-09	-0.294724516	0.052061	-0.358424327	0.008699	0.501940376	0.000835	0.445979288	0.002824	-0.06094268	0.84587	C4	-	Y	-	Y	Y	Y	-
ENSG00000074695	LMAN1	0.34125904	1.57E-09	0.149434923	0.037908	0.147506969	0.042261	-0.185541907	1.56E-05	-0.179149902	8.82E-05	0.000992254	0.994924	C5	-	Y	-	-	-	-	-
ENSG00000074755	ZZEF1	0.405098244	1.73E-09	0.320493377	0.000172	0.188979476	0.009683	-0.078033426	0.387524	-0.201391829	0.001308	-0.12865831	0.244326	C1	-	Y	Y	-	-	-	-
ENSG00000074771	NOX3	-0.77955068	0.079788	-0.092494463	0.882738	0.629168432	0.088509	0.69610869	0.133872	1.424778204	0.000106	0.726387976	NA	C4	-	-	-	-	-	Y	-
ENSG00000074935	TUBE1	0.365542325	9.13E-05	0.096440988	0.473795	0.487716359	6.05E-08	-0.262870305	0.003321	0.136667634	0.144817	0.394134067	1.37E-05	C4	-	Y	-	Y	-	-	Y
ENSG00000075035	WSCD2	-0.757395012	0.002865	-0.285118624	0.44167	0.034459147	0.933346	0.475017043	0.172293	0.806179134	0.004976	0.324834665	0.53385	C4	-	Y	-	-	-	Y	-
ENSG00000075043	KCNQ2	-0.248408036	1.88E-06	-0.021043314	0.829709	0.059534734	0.41952	0.233684275	2.04E-07	0.322787146	2.59E-15	0.083791633	0.223973	C4	-	-	-	-	-	Y	-
ENSG00000075131	TIPIN	-0.325335806	0.001059	-0.265202481	0.03889	-0.34169868	0.001057	0.066157678	0.66516	-0.001657605	0.99075	-0.073683068	0.758087	C4	-	Y	-	Y	-	-	-
ENSG00000075213	SEMA3A	-0.64778242	0.458838	1.290629316	0.017342	0.944064901	0.09954	1.945436121	0.001469	1.60611127	0.013331	-0.342812471	NA	C2	-	-	Y	-	Y	Y	-
ENSG00000075239	ACAT1	-0.346598264	1.30E-07	0.031824288	0.800991	-0.055096835	0.609794	0.384775383	4.38E-09	0.306420742	3.61E-06	-0.08395057	0.524775	C4	-	Y	-	-	Y	Y	-
ENSG00000075275	CELSR1	0.860681119	0.006501	0.154277178	0.815832	0.278401089	0.617526	-0.69970344	0.064849	-0.568646003	0.139562	0.128445223	NA	C1	-	Y	-	-	-	-	-
ENSG00000075292	ZNF638	0.424973717	1.47E-15	0.172268767	0.011867	0.258812725	1.84E-05	-0.246033964	3.19E-08	-0.151520227	0.000778	0.089193881	0.236659	C5	-	Y	-	-	-	-	-
ENSG00000075407	ZNF37A	0.536346639	9.08E-15	0.205983816	0.032024	0.237972766	0.007571	-0.323477408	1.26E-07	-0.283683342	3.46E-06	0.034600962	0.840067	C5	-	Y	-	-	Y	-	-
ENSG00000075426	FOSL2	0.16191262	0.143649	-0.445888281	0.000682	-0.053372209	0.752845	-0.601518268	2.93E-07	-0.200776461	0.096194	0.396257072	0.019202	C5	-	-	Y	-	Y	-	Y
ENSG00000075429	CACNG5	0.033607091	0.883732	-0.622523495	0.001469	-0.230054791	0.263004	-0.650420424	0.000412	-0.249044998	0.194879	0.395929428	0.154045	C5	-	-	Y	-	Y	-	-
ENSG00000075624	ACTB	0.420637916	1.78E-23	0.145191902	0.013509	0.060976189	0.356499	-0.268897166	1.30E-14	-0.34490041	2.30E-27	-0.081280874	0.213226	C5	-	Y	-	-	-	Y	-
ENSG00000075643	MOCOS	-2.33896641	0.01018	-0.825306864	NA	-0.38262394	0.667387	1.518105578	0.184404	1.972629137	0.047637	0.445492426	NA	C3	-	Y	-	-	-	Y	-
ENSG00000075702	WDR62	-0.115751002	0.18426	-0.13722486	0.153902	-0.369757858	8.50E-06	-0.014915131	0.892138	-0.239288041	0.002806	-0.229665954	0.019868	C5	-	-	-	Y	-	-	-
ENSG00000075711	DLG1	0.10882489	0.156056	0.330398352	4.52E-05	0.222505815	0.008133	0.227652535	0.000223	0.12851124	0.052473	-0.104907503	0.361959	C4	-	-	Y	-	-	-	-
ENSG00000075826	SEC31B	0.434084637	0.000164	0.220399785	0.136648	0.29315028	0.028602	-0.20710404	0.088403	-0.126060708	0.33747	0.075653207	0.744484	C5	-	Y	-	-	-	-	-
ENSG00000075856	SART3	0.318879147	4.21E-12	0.023281418	0.808345	-0.011538215	0.902366	-0.288963347	2.71E-09	-0.315775558	1.71E-11	-0.031905413	0.800737	C5	Y	Y	-	-	-	Y	-
ENSG00000075975	MKRN2	0.3359911	2.71E-06	0.159171054	0.08571	0.083752474	0.418591	-0.170529742	0.023189	-0.23766161	0.001015	-0.07249698	0.599096	C5	-	Y	-	-	-	-	-
ENSG00000076043	REXO2	0.212664437	0.059748	-0.121162081	0.446733	0.309223355	0.014488	-0.327427229	0.004604	0.111440043	0.414431	0.433000728	0.002223	C4	-	-	-	Y	Y	-	Y
ENSG00000076053	RBM7	0.175716575	0.074016	-0.227068046	0.056902	-0.020795843	0.903485	-0.39646491	3.97E-05	-0.181886601	0.087789	0.209156889	0.173511	C5	-	-	-	-	Y	-	-
ENSG00000076067	RBMS2	0.32692596	7.67E-05	0.255130055	0.022882	0.379025835	6.17E-06	-0.065669414	0.574344	0.066736231	0.484685	0.127209727	0.39019	C5	-	Y	-	Y	-	-	-
ENSG00000076108	BAZ2A	0.550910033	3.10E-22	0.295325865	8.65E-06	0.409209856	4.73E-12	-0.249366781	1.93E-08	-0.12716498	0.003146	0.116890223	0.069496	C5	-	Y	-	Y	-	-	-

ENSG00000076201	PTPN23	0.360126784	6.61E-09	0.109971217	0.220519	0.098484684	0.254342	-0.243659901	2.58E-05	-0.247162004	1.61E-05	-0.0088298	0.959571	C5	Y	Y	-	-	-	-	-
ENSG00000076242	MLH1	-0.552719838	3.13E-20	-0.035782144	0.770011	-0.114702679	0.113073	0.523261232	1.02E-14	0.452694583	9.34E-15	-0.076075496	0.544229	C4	-	Y	-	-	Y	Y	-
ENSG00000076248	UNG	-0.285789847	2.19E-07	-0.307538243	5.38E-06	-0.411499146	6.02E-09	-0.01523518	0.864551	-0.111281206	0.130399	-0.100786273	0.43015	C4	-	-	Y	Y	-	-	-
ENSG00000076321	KLHL20	0.342781918	9.55E-07	-0.043942356	0.721155	-0.051975808	0.658522	-0.380490597	5.24E-09	-0.380445495	1.25E-08	-0.005230737	0.980847	C5	-	Y	-	-	Y	Y	-
ENSG00000076344	RGS11	-0.536606026	0.000229	-0.465327747	0.002618	-0.415413811	0.005982	0.077962632	0.722687	0.135765479	0.490329	0.052658949	0.888164	C4	-	Y	Y	Y	-	-	-
ENSG00000076356	PLXNA2	0.352031246	2.12E-08	0.412736352	8.68E-08	0.317224149	1.24E-05	0.067121106	0.23464	-0.020337109	0.716291	-0.092740305	0.310907	C1	-	Y	Y	Y	-	-	-
ENSG00000076554	TPD52	-0.448085088	2.83E-08	-0.079602505	0.546611	-0.031733654	0.820184	0.374896202	3.68E-05	0.431201503	8.62E-07	0.050691947	0.807892	C4	-	Y	-	-	Y	Y	-
ENSG00000076555	ACACB	-0.752400552	2.08E-14	-0.17468952	0.227183	0.102126712	0.403564	0.584659748	5.53E-07	0.869090502	8.53E-21	0.279625543	0.056295	C4	-	Y	-	-	Y	Y	-
ENSG00000076650	GPATCH1	0.509596362	6.38E-07	0.108353851	0.504456	-0.033728361	0.86346	-0.394902135	0.000147	-0.528835831	9.09E-07	-0.139306622	0.498637	C5	-	Y	-	-	Y	Y	-
ENSG00000076685	NT5C2	0.325240199	6.15E-05	0.134718592	0.187104	0.148784897	0.111455	-0.184073329	0.015343	-0.162067051	0.029619	0.016672157	0.930354	C5	-	Y	-	-	-	-	-
ENSG00000076826	CAMSAP3	0.022832041	0.893586	0.117854589	0.541102	0.30157683	0.022798	0.101549074	0.49968	0.293305828	0.008721	0.186634398	0.343792	C4	-	-	-	Y	-	-	-
ENSG00000076864	RAP1GAP	-0.600891899	5.23E-11	-0.08998781	0.520797	-0.032542222	0.820366	0.51723458	2.39E-07	0.582931439	4.09E-10	0.060283953	0.766891	C4	-	Y	-	-	Y	Y	-
ENSG00000077092	RARB	-0.282517173	0.402143	0.249961701	0.511709	0.466928127	0.106084	0.539220156	0.071362	0.76475001	0.00407	0.220306224	0.616109	C4	-	-	-	-	-	Y	-
ENSG00000077157	PPP1R12B	0.753594866	7.38E-15	0.276252632	0.030492	0.319373344	0.009227	-0.472287715	5.28E-10	-0.420155753	7.54E-08	0.046396201	0.823431	C5	-	Y	-	Y	Y	Y	-
ENSG00000077238	IL4R	0.755658671	0.033182	1.262104124	0.000301	1.068602417	0.002744	0.513077766	0.118814	0.327784396	0.365265	-0.190100862	NA	C1	-	Y	Y	Y	-	-	-
ENSG00000077264	PAK3	0.453919642	0.00055	0.07621177	0.715433	0.36250611	0.010439	-0.37114758	0.000169	-0.07733099	0.535969	0.288291539	0.019343	C1	-	Y	-	Y	Y	-	-
ENSG00000077279	DCX	-0.228377392	0.015095	0.075879842	0.810992	0.157531309	0.496132	0.311078822	0.095624	0.400501332	0.003748	0.084154016	0.862969	C4	-	-	-	-	-	Y	-
ENSG00000077327	SPAG6	-0.517873074	0.033018	-0.215889287	0.498544	-0.307488466	0.345344	0.308602583	0.294646	0.224909621	0.515854	-0.088680553	0.894887	C3	-	Y	-	-	-	-	-
ENSG00000077454	LRCH4	0.412534024	0.000245	0.168484309	0.258175	0.205679137	0.113343	-0.237839069	0.047424	-0.192268992	0.107498	0.039797431	0.88523	C5	-	Y	-	-	-	-	-
ENSG00000077522	ACTN2	-0.958309391	0.000207	-0.494245452	0.067638	-0.512564931	0.048603	0.46902412	0.131602	0.45987895	0.142182	-0.015566002	0.982152	C3	-	Y	-	Y	-	-	-
ENSG00000077585	GPR137B	0.469648487	0.00054	-0.129090456	0.564993	0.003165806	0.990492	-0.592140846	1.94E-06	-0.452143006	0.000248	0.134531003	0.591991	C5	-	Y	-	-	Y	Y	-
ENSG00000078018	MAP2	-0.334996126	1.91E-08	-0.132223045	0.149614	-0.063266352	0.47969	0.209098508	9.39E-05	0.286316523	5.76E-12	0.071823128	0.494927	C4	-	Y	-	-	-	-	-
ENSG00000078114	NEBL	-0.887731093	0.000951	-0.371082919	0.246271	-0.213685666	0.485825	0.525068848	0.112846	0.688149108	0.017206	0.160105395	0.762025	C4	-	Y	-	-	-	Y	-
ENSG00000078142	PIK3C3	0.398303592	4.46E-12	0.205689928	0.003532	0.142624999	0.046764	-0.18639042	0.000143	-0.240995822	2.56E-07	-0.059985713	0.503233	C5	-	Y	-	-	-	-	-
ENSG00000078237	TIGAR	0.142527459	0.53562	-0.397735236	0.093479	-0.355758053	0.116552	-0.534303479	0.009283	-0.484115081	0.017462	0.044855894	0.936933	C5	-	-	-	-	Y	Y	-
ENSG00000078269	SYNJ2	0.922080109	2.04E-16	0.135916565	0.584322	0.204569428	0.203446	-0.779440074	2.68E-08	-0.702761934	3.15E-10	0.071209811	0.850198	C5	-	Y	-	-	Y	Y	-
ENSG00000078319	PMS2P1	-0.436081092	6.02E-09	-0.124528697	0.201575	-0.071000142	0.498477	0.318103401	5.41E-05	0.379933023	8.42E-07	0.056538682	0.70884	C4	-	Y	-	-	Y	Y	-
ENSG00000078487	ZCWPW1	-0.542543259	0.023017	0.260279999	0.370454	0.299743013	0.226162	0.809211281	0.000356	0.857507968	6.14E-05	0.042369105	0.937321	C4	-	Y	-	-	Y	Y	-
ENSG00000078549	ADCYAP1R1	-0.477123879	1.03E-13	0.029938134	0.802833	-0.040441355	0.698746	0.51384263	5.95E-33	0.45074885	4.34E-25	-0.068101412	0.445982	C4	-	Y	-	-	Y	Y	-
ENSG00000078725	BRINP1	-0.461833907	0.035843	-0.357824368	0.192593	-0.381039197	0.222748	0.110297623	0.726637	0.095632135	0.796647	-0.021620696	0.978036	C4	-	Y	-	-	-	-	-
ENSG00000078747	ITCH	0.437803654	1.05E-07	0.212305046	0.034037	0.128278494	0.219362	-0.219282759	0.000706	-0.295174187	1.55E-06	-0.081299406	0.455397	C5	-	Y	-	-	-	-	-
ENSG00000078900	TP73	0.297148694	0.002397	-0.020407116	0.90903	0.002810214	0.989463	-0.310954407	0.003093	-0.280042758	0.021423	0.025238337	0.941626	C5	-	-	-	-	Y	-	-
ENSG00000078902	TOLLIP	0.424189771	4.22E-08	0.070650105	0.592943	0.12833263	0.207598	-0.34674269	1.10E-05	-0.281110379	0.000183	0.060388634	0.714581	C5	-	Y	-	-	Y	-	-
ENSG00000078967	UBE2D4	-0.35946779	2.37E-11	-0.110713802	0.156973	-0.099342621	0.191372	0.255137437	1.72E-05	0.274955331	2.33E-06	0.014215135	0.935372	C4	-	Y	-	-	-	-	-
ENSG00000079102	RUNX1T1	-0.765272248	1.02E-05	-0.835767843	7.08E-07	-0.743864888	6.13E-06	-0.063279252	0.82311	0.035906151	0.902802	0.094544522	0.814705	C4	-	Y	Y	Y	-	-	-
ENSG00000079134	THOC1	0.346818284	1.17E-06	-0.102598797	0.36128	0.070995831	0.507722	-0.44265849	6.00E-12	-0.261619297	2.48E-05	0.176018838	0.075964	C5	-	Y	-	-	Y	-	-
ENSG00000079308	TNS1	-0.324636641	3.03E-06	0.085209657	0.380765	0.195934327	0.01388	0.415786681	9.13E-17	0.535178239	1.06E-25	0.11389579	0.148305	C4	-	Y	-	-	Y	Y	-
ENSG00000079335	CDC14A	0.586985311	0.000255	0.378758307	0.136208	0.394911878	0.038195	-0.200798572	0.371952	-0.177085161	0.333989	0.018760082	0.972936	C5	-	Y	-	Y	-	-	-
ENSG00000079337	RAPGEF3	-1.025745168	0.012693	-0.065989848	0.903779	-0.164206722	0.709377	0.96699367	0.02761	0.876072937	0.042711	-0.095426741	NA	C3	-	Y	-	-	Y	Y	-
ENSG00000079435	LIPE	0.327108654	0.03707	0.201893297	0.337597	0.249693617	0.196307	-0.118716531	0.54518	-0.062505423	0.770363	0.050526583	0.901808	C5	-	Y	-	-	-	-	-
ENSG00000079459	FDFT1	0.204047909	0.000647	0.370074933	5.61E-08	0.399572127	6.29E-10	0.172672391	0.005773	0.210066827	0.000332	0.0324717	0.833878	C4	-	-	Y	Y	-	-	-
ENSG00000079482	OPHN1	-0.174762885	0.402803	-0.055563603	0.861649	0.244834858	0.308441	0.125095251	0.578346	0.434394488	0.020767	0.302942345	0.294821	C4	-	-	-	-	-	Y	-
ENSG00000079689	SCGN	-1.265428134	2.40E-11	-0.39663148	0.077071	-0.361480326	0.075434	0.877180805	6.57E-05	0.918750493	8.41E-06	0.037660218	0.943834	C3	-	Y	-	-	Y	Y	-
ENSG00000079739	PGM1	-0.142110295	0.054662	0.154304591	0.016491	0.192210706	0.001	0.302967934	1.48E-06	0.349063492	4.53E-09	0.040859201	0.68278	C4	-	-	-	-	Y	Y	-
ENSG00000079841	RIMS1	0.305710938	0.02887	0.143530225	0.505939	0.02185103	0.930673	-0.155662274	0.376047	-0.269114643	0.083834	-0.118715593	0.701775	C5	-	Y	-	-	-	-	-
ENSG00000079999	KEAP1	-0.297272304	3.48E-05	-0.244968197	0.004279	-0.332754831	7.65E-06	0.058722299	0.488516	-0.020546433	0.820169	-0.084595977	0.457415	C4	-	-	-	Y	-	-	-
ENSG00000080031	PTPRH	-0.548014603	0.009772	-0.028400441	0.942228	0.218551388	0.369464	0.52677483	0.023308	0.78099991	6.09E-05	0.250881051	0.460887	C4	-	Y	-	-	Y	Y	-
ENSG00000080189	SLC35C2	-0.33104564	2.41E-07	-0.023358866	0.83409	-0.111462742	0.160616	0.31421066	1.01E-06	0.234416951	0.000462	-0.085226992	0.440789	C4	-	Y	-	-	Y	-	-
ENSG00000080224	EPHA6	-0.393975598	0.209365	0.21256231	0.589561	0.167612564	0.647834	0.614798993	0.048716	0.575768433	0.056696	-0.042866714	0.95277	C2	-	-	-	-	Y	-	-
ENSG00000080298	RFX3	0.440250561	7.95E-07	-0.110297504	0.456448	0.137287857	0.256928	-0.543959832	2.77E-09	-0.288524142	0.000829	0.250482401	0.072162	C5	-	Y	-	-	Y	-	-
ENSG00000080371	RAB21	0.317500396	1.40E-06	-0.052968341	0.727089	0.031284165	0.774976	-0.3637713	1.13E-05	-0.27160973	1.16E-05	0.087020703	0.596074	C5	Y	Y	-	-	Y	-	-
ENSG00000080608	PUM3	0.291341	1.71E-08	0.014004719	0.898607	-0.085476936	0.26875	-0.270531639	4.20E-08	-0.362170947	3.80E-14	-0.09670845	0.288265	C5	-	-	-	-	-	Y	-
ENSG00000080644	CHRNA3	-0.310492496	1.16E-15	-0.237436776	5.93E-08	-0.377787811	9.02E-11	0.079516143	0.06309	-0.052851191	0.399801	-0.137444939	0.078336	C4	Y	Y	-	Y	-	-	-
ENSG00000080802	CNOT4	0.415811065	7.69E-08	-0.081874463	0.541251	0.171803396	0.097221	-0.491612326	1.39E-11	-0.229816816	0.002064	0.25659092	0.016098	C5	-	Y	-	-	Y	-	-
ENSG00000080815	PSEN1	0.301576265	5.96E-07	0.101877574	0.282098	0.05313694	0.5467	-0.193495646	0.002076	-0.233984634	7.66E-06	-0.045743407	0.723061	C5	-	Y	-	-	-	-	-
ENSG00000080819	CPOX	0.681641716	1.14E-18	0.203088948	0.057569	0.447394517	3.30E-07	-0.472434321	4.51E-10	-0.219530411	0.004776	0.247438391	0.018109	C5	-	Y	-	Y	Y	-	-
ENSG00000080822	CLDND1	0.373811086	8.84E-18	0.153794204	0.006421	0.167798514	0.001677	-0.21352564	1.36E-07	-0.191420343	1.98E-06	0.01679527	0.88523	C5	-	Y	-	-	-	-	-
ENSG00000080839	RBL1	-0.413617309	5.49E-07	-0.373516888	0.000334	-0.42101788	2.53E-06	0.04655898	0.71904	0.007196787	0.955884	-0.04469437	0.846667	C4	-	Y	Y	Y	-	-	-
ENSG00000080845	DLGAP4	0.33635402	7.76E-13	0.14675514	0.027286	0.141963989	0.026514	-0.183302995	0.000389	-0.179615986	0.000401	-0.001959033	0.991437	C5	-	Y	-	-	-	-	-
ENSG00000080947	CROCCP3	0.395955616	0.009951	0.480270082	0.002113	0.570926076	6.52E-05	0.090468747	0.610122	0.188880986	0.203571	0.093919899	0.714139	C1	-	Y	Y	Y	-	-	-
ENSG00000081014	AP4E1	0.175360252	0.047473	0.031018904	0.845831	-0.18351691	0.051576	-0.137820479	0.179461	-0.344150126	2.48E-05	-0.211782278	0.089643	C5	-	-	-	-	-	Y	-
ENSG00000081019	RSBN1	0.42523597	1.56E-07	-0.153960222	0.192519	0.153238559	0.136675	-0.572672754	1.34E-11	-0.257327849	0.001821	0.309972853	0.006118	C5	-	Y	-	-	Y	-	Y
ENSG00000081052	COL4A4	1.136423179	0.214725	-1.814422406	NA	0.082538298	NA	-2.939454507	0.016112	-1.037532731	0.302453	1.899818837	NA	C5	-	-	-	-	Y	-	-
ENSG00000081087	OSTM1	0.544403155	4.84E-11	0.200625524	0.132551	0.208900267	0.047072	-0.337117653	0.000304	-0.320751915	5.22E-05	0.010900031	0.96897	C5	Y	Y	-	-	Y	Y	-
ENSG00000081177	EXD2	0.305754008	3.94E-06	0.164503561	0.036806	0.154913735	0.064735	-0.134797133	0.053592	-0.135971934	0.068285	-0.006601537	0.973458	C5	-	Y	-	-	-	-	-
ENSG00000081181	ARG2	0.366912575	0.023014	-0.076517854	0.771517	0.088500807	0.710743	-0.437308932	0.005515	-0.264151909	0.117187	0.168018003	0.561947	C5	-	Y	-	-	Y	-	-
ENSG00000081189	MEF2C	-1.05637233	5.98E-06	-0.360375203	0.182307	-0.201549573	0.482968	0.702824672	0.008013	0.869010316	0.0006	0.161558398	0.718114	C2	-	Y	-	-	Y	Y	-

ENSG00000081386	ZNF510	0.758757073	4.19E-17	0.116205248	0.397878	0.291304909	0.005769	-0.636512429	8.73E-19	-0.452996898	1.57E-10	0.178053318	0.104833	C5	-	Y	-	-	Y	Y	-
ENSG00000081665	ZNF506	0.197352306	0.030976	-0.167491211	0.178469	0.114942434	0.376451	-0.358696644	2.08E-05	-0.068046466	0.525309	0.285301694	0.020794	C5	-	-	-	-	Y	-	-
ENSG00000081721	DUSP12	-0.14713987	0.087099	-0.128926695	0.20001	-0.331676309	1.24E-05	0.024574964	0.843973	-0.169818364	0.055778	-0.199679579	0.076936	C4	-	-	-	Y	-	-	-
ENSG00000081842	PCDHA6	-2.427971938	0.007883	-0.409029805	NA	-0.814594179	0.429014	2.023284932	0.048221	1.629194672	0.199845	-0.399032744	NA	C3	-	Y	-	-	Y	-	-
ENSG00000082126	MPP4	-1.27385827	0.315089	1.209603834	NA	1.169115829	0.295344	2.494479973	0.010462	2.45599059	0.029433	-0.043392497	NA	C2	-	-	-	-	Y	Y	-
ENSG00000082146	STRADB	-0.530957265	2.89E-07	0.020811126	0.918164	0.005684246	0.974778	0.558365479	5.31E-07	0.551561708	3.09E-08	-0.011601531	0.968619	C2	-	Y	-	-	Y	Y	-
ENSG00000082269	FAM135A	0.586349729	1.54E-07	0.262321907	0.059714	0.392375229	0.001654	-0.317993098	0.001085	-0.179842304	0.088464	0.132702215	0.417301	C5	-	Y	-	Y	Y	-	-
ENSG00000082458	DLG3	0.586097776	9.28E-09	0.478627932	6.89E-05	0.409741205	0.001293	-0.101223754	0.402457	-0.162055753	0.164207	-0.066018209	0.7831	C1	-	Y	Y	Y	-	-	-
ENSG00000082497	SERTAD4	0.023438335	0.85261	-0.278642036	0.006516	-0.296962726	0.00193	-0.29564872	0.000748	-0.305630336	0.000337	-0.015426144	0.948722	C5	-	-	-	-	-	Y	-
ENSG00000082516	GEMIN5	0.380252582	2.94E-06	0.256021718	0.010998	0.304547612	0.00072	-0.118422949	0.11834	-0.061517357	0.43036	0.051749042	0.714139	C5	-	Y	-	Y	-	-	-
ENSG00000082684	SEMA5B	-1.686728596	5.85E-11	-0.787188215	0.002326	-0.583121896	0.006385	0.910719443	0.011488	1.119104753	0.000207	0.206491605	0.689251	C3	-	Y	Y	Y	Y	Y	-
ENSG00000082781	ITGB5	-0.659477281	0.030785	-0.082271272	0.866675	-0.068971125	0.878461	0.584172127	0.061242	0.605826894	0.045002	0.016902438	0.982152	C2	-	Y	-	-	-	Y	-
ENSG00000083097	DOP1A	0.70340227	1.92E-13	0.44174018	4.61E-05	0.525081795	1.51E-07	-0.254973196	0.003333	-0.163862114	0.068001	0.085501991	0.583262	C5	-	Y	Y	Y	-	-	-
ENSG00000083123	BCKDHB	-0.68172023	5.31E-15	-0.041385133	0.764429	-0.004388892	0.976293	0.647499272	3.51E-12	0.692036507	1.58E-14	0.039743463	0.843713	C2	-	Y	-	-	Y	Y	-
ENSG00000083168	KAT6A	0.367941764	4.58E-05	0.259520037	0.022095	0.196103632	0.071797	-0.102582497	0.162654	-0.157765008	0.008298	-0.060412687	0.656136	C5	-	Y	-	-	-	-	-
ENSG00000083223	TUT7	0.794745503	2.95E-09	0.529044026	0.001469	0.52986573	0.000349	-0.260627296	0.014703	-0.250504475	0.006498	0.004470517	0.988432	C1	-	Y	Y	Y	-	-	-
ENSG00000083312	TNPO1	0.309538211	4.70E-08	0.060237905	0.549224	0.015012293	0.893412	-0.242947567	8.82E-08	-0.279915341	4.17E-11	-0.042398819	0.714139	C5	-	Y	-	-	-	-	-
ENSG00000083444	PLOD1	-0.287872891	4.46E-05	0.049436248	0.660755	0.197720493	0.015773	0.343929751	2.73E-07	0.500524671	8.13E-15	0.15120582	0.131987	C4	-	-	-	-	Y	Y	-
ENSG00000083535	PIBF1	0.310111587	0.00016	0.209750643	0.030863	0.223596625	0.01558	-0.093913078	0.315675	-0.071723172	0.452723	0.016847712	0.936471	C5	-	Y	-	-	-	-	-
ENSG00000083720	OXCT1	-0.289819727	1.34E-06	0.009409873	0.933747	0.036701754	0.689094	0.305663339	8.42E-08	0.341142612	1.44E-09	0.030087528	0.818037	C4	-	-	-	-	Y	Y	-
ENSG00000083799	CYLD	0.345873807	0.000109	0.158337581	0.166179	0.209247721	0.023225	-0.181198615	0.037678	-0.122280827	0.142841	0.053154645	0.729387	C5	-	Y	-	-	-	-	-
ENSG00000083807	SLC27A5	-0.183925922	0.338165	0.163273404	0.486316	0.179467946	0.430952	0.353719598	0.021358	0.379222425	0.018541	0.019651515	0.96254	C4	-	-	-	-	Y	Y	-
ENSG00000083812	ZNF324	0.454792402	1.52E-05	-0.055883008	0.750255	0.088395843	0.554527	-0.504324209	5.92E-07	-0.351841808	0.000611	0.1471065	0.3891	C5	-	Y	-	-	Y	Y	-
ENSG00000083828	ZNF586	0.165759536	0.28619	0.304747188	0.043742	0.291784512	0.04484	0.145244474	0.326518	0.140427693	0.338493	-0.01029194	0.975773	C4	-	-	Y	-	-	-	-
ENSG00000083844	ZNF264	0.276311117	0.023142	-0.165381772	0.273993	-0.021731637	0.911181	-0.435210367	2.79E-06	-0.283604686	0.003428	0.145954952	0.32601	C5	-	-	-	-	Y	-	-
ENSG00000083857	FAT1	0.449630179	0.000192	0.380940997	0.012352	0.304796543	0.014491	-0.062685968	0.663907	-0.130797551	0.158632	-0.072627276	0.726932	C1	-	Y	Y	Y	-	-	-
ENSG00000083937	CHMP2B	0.702632628	1.37E-12	0.274587191	0.042056	0.219262096	0.117745	-0.421368718	2.41E-05	-0.468928906	4.00E-06	-0.052518483	0.841586	C5	-	Y	-	-	Y	Y	-
ENSG00000084070	SMAP2	0.330367533	1.67E-06	0.177548808	0.048842	0.227228308	0.006877	-0.146480445	0.040951	-0.088463901	0.253047	0.052708621	0.711022	C5	Y	Y	-	-	-	-	-
ENSG00000084073	ZMPSTE24	0.40026778	4.21E-12	0.042685593	0.696892	-0.038222006	0.668438	-0.351038502	1.05E-08	-0.423786386	2.10E-15	-0.078273911	0.494187	C5	-	Y	-	-	Y	Y	-
ENSG00000084112	SSH1	0.333170367	7.27E-06	0.344855113	1.37E-05	0.325445902	3.34E-05	0.018061769	0.826227	0.006664956	0.939319	-0.016800537	0.912643	C1	-	Y	Y	Y	-	-	-
ENSG00000084453	SLCO1A2	-0.945714989	1.79E-07	-0.361473778	0.085983	-0.466809256	0.017824	0.590766688	0.002493	0.493421369	0.015162	-0.102542302	0.794576	C3	-	Y	-	Y	Y	Y	-
ENSG00000084636	COL16A1	-0.671572236	0.000238	-0.466787125	0.026402	-0.231386224	0.315321	0.211088548	0.388003	0.455080688	0.02983	0.238320046	0.472014	C4	-	Y	Y	-	-	Y	-
ENSG00000084676	NCOA1	0.391799916	5.02E-08	0.282403323	0.001803	0.220684451	0.005868	-0.103232641	0.165098	-0.156653662	0.006292	-0.058587152	0.638566	C1	-	Y	-	-	-	-	-
ENSG00000084764	MAPRE3	0.440527264	6.84E-08	0.123059661	0.324234	0.341753453	0.000449	-0.310649855	1.32E-05	-0.084361906	0.325421	0.221194837	0.031665	C1	-	Y	-	Y	Y	-	-
ENSG00000084774	CAD	0.235035849	0.000128	0.365023586	4.48E-07	0.37438024	1.09E-08	0.136538618	0.022149	0.153617608	0.002875	0.012069054	0.943834	C4	-	-	Y	Y	-	-	-
ENSG00000085185	BCORL1	0.422216892	6.55E-08	0.069743333	0.590963	0.344063877	5.86E-05	-0.346369066	1.80E-06	-0.063442505	0.467595	0.27747352	0.002326	C5	-	Y	-	Y	Y	-	-
ENSG00000085224	ATRX	0.355567219	4.27E-06	0.10660104	0.338588	0.108646486	0.311575	-0.242647959	1.74E-09	-0.232488003	1.29E-07	0.004784962	0.974616	C1	-	Y	-	-	-	-	-
ENSG00000085274	MYNN	0.253570646	0.001771	-0.126736482	0.260099	-0.032259631	0.810283	-0.373910296	5.39E-07	-0.271253608	0.000326	0.097214865	0.488602	C5	-	-	-	-	Y	-	-
ENSG00000085415	SEH1L	0.127178441	0.028238	-0.200477539	0.00766	-0.180984888	0.005375	-0.321101459	1.97E-08	-0.293507856	1.01E-08	0.022233114	0.890821	C5	-	-	-	-	Y	-	-
ENSG00000085433	WDR47	0.315568423	0.000102	0.174005016	0.099486	0.301523797	0.000737	-0.135164664	0.069211	0.00042439	0.996017	0.130230992	0.21704	C1	-	Y	-	Y	-	-	-
ENSG00000085465	OVGP1	2.195464454	1.39E-06	0.889697694	NA	0.49805691	0.595304	-1.299116485	0.001716	-1.683229604	0.00016	-0.388913623	NA	C5	-	Y	-	-	Y	Y	-
ENSG00000085511	MAP3K4	0.494570965	4.22E-08	0.13557588	0.312905	0.116651384	0.341215	-0.352546035	1.80E-06	-0.363627582	5.89E-08	-0.016357037	0.937494	C5	-	Y	-	-	Y	Y	-
ENSG00000085552	IGSF9	0.310050222	1.34E-06	0.0855532	0.323294	0.221223666	0.000938	-0.21817155	0.000825	-0.074179074	0.316611	0.138481673	0.113187	C5	-	Y	-	-	-	-	-
ENSG00000085831	TTC39A	-0.865316815	0.000184	-0.44587718	0.145836	-0.743063108	0.002331	0.428793366	0.186155	0.1372806	0.7136	-0.295742834	NA	C4	-	Y	-	Y	-	-	-
ENSG00000085840	ORC1	-0.147612172	0.117801	-0.369447947	0.000248	-0.395172134	3.85E-06	-0.215474461	0.037388	-0.23285498	0.010849	-0.022717511	0.931565	C4	-	-	Y	Y	-	-	-
ENSG00000085871	MGST2	-0.598697164	0.008257	-0.234503913	0.441183	-0.257432072	0.380717	0.370708124	0.141586	0.356671571	0.174993	-0.020075062	0.975494	C4	-	Y	-	-	-	-	-
ENSG00000085978	ATG16L1	0.331997972	3.14E-06	-0.06742692	0.574502	0.046629556	0.695842	-0.392935771	1.30E-08	-0.270907926	0.000132	0.116829877	0.352857	C5	-	Y	-	-	Y	-	-
ENSG00000085982	USP40	-0.532080924	8.73E-13	-0.139251719	0.221051	-0.132292425	0.170469	0.399332047	9.23E-06	0.414394962	3.73E-07	0.009735965	0.970285	C4	-	Y	-	-	Y	Y	-
ENSG00000085999	RAD54L	0.485468078	2.59E-11	0.099697215	0.412613	0.025716716	0.876259	-0.379289882	4.15E-06	-0.445246214	6.00E-07	-0.07097489	0.749894	C5	-	Y	-	-	Y	Y	-
ENSG00000086015	MAST2	0.411694255	1.77E-09	0.248381105	0.000239	0.271901045	1.15E-05	-0.156874753	0.031151	-0.125165196	0.076039	0.026325021	0.853543	C5	-	Y	-	-	-	-	-
ENSG00000086061	DNAJA1	0.428499049	1.06E-26	0.118343016	0.069267	0.277144489	2.78E-08	-0.303526704	1.26E-14	-0.136888026	0.000174	0.161609478	0.009182	C5	-	Y	-	-	Y	-	-
ENSG00000086102	NFX1	0.243689433	0.00024	0.008806329	0.945256	-0.078196813	0.377258	-0.228419381	8.73E-05	-0.307274073	5.56E-08	-0.084357189	0.395152	C5	-	-	-	-	-	Y	-
ENSG00000086189	DIMT1	0.44769228	2.12E-06	0.239326224	0.055645	0.136591558	0.315069	-0.202077571	0.065572	-0.296229762	0.004353	-0.100031104	0.62517	C5	-	Y	-	-	-	-	-
ENSG00000086289	EPDR1	-0.285152829	0.000262	0.017180527	0.901729	-0.006302549	0.964694	0.308743941	7.71E-05	0.293597678	0.000224	-0.020600069	0.920886	C4	-	-	-	-	Y	-	-
ENSG00000086300	SNX10	-0.85044434	0.000258	-0.380607149	0.201577	-0.137087827	0.66724	0.477964155	0.116193	0.727696403	0.005469	0.245532394	0.598239	C4	-	Y	-	-	-	Y	-
ENSG00000086717	PPEF1	-0.585696648	3.65E-09	-0.050544064	0.740032	-0.093728092	0.427776	0.541064413	1.59E-07	0.50671628	3.11E-07	-0.040393321	0.853204	C4	-	Y	-	-	Y	Y	-
ENSG00000086991	NOX4	-0.957017723	0.001598	-0.148201036	0.776731	-0.043474199	0.916277	0.812321637	0.03723	0.928571462	0.002317	0.107049572	NA	C3	-	Y	-	-	Y	Y	-
ENSG00000087087	SRRT	0.505244023	2.27E-26	-0.206445454	0.000732	-0.256929059	2.00E-05	-0.704713241	1.20E-87	-0.747571253	4.14E-92	-0.047844997	0.587615	C5	-	Y	-	-	Y	Y	-
ENSG00000087088	BAX	-0.399212731	2.84E-07	-0.140960927	0.219667	-0.257034782	0.00658	0.264850169	7.07E-05	0.157046945	0.019403	-0.113173856	0.341131	C4	-	Y	-	-	-	-	-
ENSG00000087095	NLK	0.545702142	3.88E-08	0.162936383	0.38768	0.28593676	0.012644	-0.375905505	0.00188	-0.245401844	0.007377	0.12564056	0.573048	C5	-	Y	-	-	Y	-	-
ENSG00000087116	ADAMTS2	-0.123807048	0.115502	-0.127513519	0.163689	-0.361948415	6.95E-07	0.002738644	0.979209	-0.223447193	0.001907	-0.231483326	0.008722	C5	-	-	-	Y	-	-	-
ENSG00000087157	PGS1	0.327066478	1.99E-08	0.192946015	0.00737	0.079084669	0.40571	-0.127582629	0.046791	-0.233385463	0.000319	-0.110873112	0.311916	C5	-	Y	-	-	-	-	-
ENSG00000087258	GNAO1	-0.130511974	0.041914	0.167029168	0.01569	0.145904428	0.037683	0.303658297	6.30E-10	0.291125181	6.02E-09	-0.018059515	0.893051	C4	-	-	-	-	Y	-	-
ENSG00000087266	SH3BP2	-0.393507683	5.09E-10	-0.226860808	0.004104	-0.28108412	2.33E-05	0.173140382	0.014234	0.127096456	0.056433	-0.051361638	0.694813	C4	-	Y	-	-	-	-	-
ENSG00000087269	NOP14	0.299905146	1.45E-05	-0.062391754	0.603612	-0.034139996	0.798856	-0.35512147	5.44E-10	-0.319133041	5.62E-07	0.031109021	0.861463	C5	-	-	-	-	Y	Y	-
ENSG00000087338	GMCL1	0.487012153	1.75E-16	0.237790239	0.015343	0.185982804	0.0257	-0.242151694	0.000482	-0.286069794	5.62E-07	-0.049212445	0.778793	C5	-	Y	-	-	-	-	-
ENSG00000087494	PTHLH	1.853348165	0.019072	1.07527013	NA	0.599491597	NA	-0.769338285	0.344115	-1.237973729	0.144906	-0.470893513	NA	C5	-	Y	-	-	-	-	-

ENSG00000087502	ERGIC2	0.329554935	7.41E-05	-0.001853993	0.990996	-0.01946398	0.887912	-0.324979952	0.000117	-0.334315712	6.74E-05	-0.014745051	0.94815	C5	-	Y	-	-	Y	Y	-
ENSG00000087842	PIR	-0.704039686	3.58E-10	-0.616115284	6.42E-07	-0.462937252	8.00E-05	0.094566907	0.55633	0.256113859	0.042616	0.156579558	0.430659	C4	Y	Y	Y	Y	-	-	-
ENSG00000087884	AAMDC	-0.523350946	1.69E-05	-0.369214855	0.012563	-0.336253126	0.023497	0.161242218	0.343086	0.201996001	0.220712	0.034873538	0.930809	C4	-	Y	Y	Y	-	-	-
ENSG00000087903	RFX2	0.236827971	0.145974	0.412730197	0.009186	0.286777514	0.081591	0.182209339	0.230157	0.064672695	0.723468	-0.122663463	0.592783	C1	-	-	Y	-	-	-	-
ENSG00000088179	PTPN4	0.298162522	3.56E-05	-0.074236581	0.527745	0.075059085	0.463651	-0.365877128	7.94E-10	-0.208592976	0.000251	0.151902958	0.092833	C5	-	-	-	-	Y	-	-
ENSG00000088256	GNA11	0.302817025	1.03E-06	0.068706494	0.439649	0.066230959	0.455647	-0.227601897	3.56E-05	-0.22180512	6.76E-05	0.000538977	0.997282	C5	-	Y	-	-	-	-	-
ENSG00000088280	ASAP3	-0.559099775	7.01E-10	-0.191893406	0.113604	0.01169083	0.94236	0.37366763	0.000199	0.585660606	5.40E-11	0.206607028	0.128456	C4	-	Y	-	-	Y	Y	-
ENSG00000088305	DNMT3B	-0.732431768	1.52E-09	-0.206702703	0.117254	-0.131076476	0.393048	0.532158791	4.13E-05	0.616086708	4.49E-06	0.078507974	0.753864	C4	-	Y	-	-	Y	Y	-
ENSG00000088340	FER1L4	-0.117316737	0.652899	0.170168263	0.562354	0.468314626	0.052614	0.294384712	0.217696	0.600297655	0.007106	0.30055807	0.397037	C4	-	-	-	-	-	Y	-
ENSG00000088356	PDRG1	-0.070732349	0.542635	-0.390520397	0.000337	-0.348673057	0.002365	-0.313136302	0.005332	-0.2631198	0.026324	0.044379245	0.886213	C4	Y	-	Y	Y	Y	-	-
ENSG00000088451	TGDS	0.367619149	0.004924	-0.056291857	0.806916	0.232046551	0.155508	-0.41784808	0.002765	-0.120823755	0.471388	0.291408102	0.164126	C5	-	Y	-	-	Y	-	-
ENSG00000088543	C3orf18	-0.167694977	0.130038	0.044807744	0.776048	0.126461259	0.247233	0.219509116	0.046479	0.308808963	0.001306	0.084855813	0.610489	C4	-	-	-	-	-	Y	-
ENSG00000088756	ARHGAP28	-1.165873495	6.35E-12	-0.259192124	0.27449	-0.118486026	0.587818	0.911699335	8.29E-06	1.061731876	1.96E-09	0.1432081	0.689251	C3	-	Y	-	-	Y	Y	-
ENSG00000088812	ATRN	0.432099852	7.90E-15	0.300524036	2.15E-07	0.224156653	0.000987	-0.125264989	0.017084	-0.193151983	0.000557	-0.073443764	0.438309	C1	-	Y	Y	-	-	-	-
ENSG00000088876	ZNF343	0.457097936	4.79E-06	0.003386128	0.987462	0.253578978	0.035093	-0.447767221	9.77E-08	-0.188980452	0.042651	0.253112828	0.033267	C5	-	Y	-	-	Y	-	-
ENSG00000088882	CPXM1	-0.180874944	0.00549	-0.198793979	0.008415	-0.303320082	2.15E-05	-0.011489893	0.861978	-0.107762232	0.032638	-0.101558203	0.177884	C4	-	-	-	Y	-	-	-
ENSG00000089060	SLC8B1	-0.338723277	0.047903	-0.33646768	0.07228	-0.176333855	0.388881	0.007523839	0.97683	0.176951884	0.393914	0.163582089	0.599073	C4	-	Y	-	-	-	-	-
ENSG00000089063	TMEM230	-0.357113894	4.24E-12	0.026918503	0.78034	0.007123155	0.941852	0.390551322	1.73E-13	0.378997736	2.83E-14	-0.016911112	0.910067	C4	-	Y	-	-	Y	Y	-
ENSG00000089127	OAS1	-1.648020104	2.08E-27	-0.777087515	1.28E-07	-0.735034926	9.41E-06	0.877004139	2.91E-06	0.927203559	5.91E-06	0.044596319	0.930354	C3	-	Y	Y	Y	Y	Y	-
ENSG00000089169	RPH3A	-0.598641803	1.32E-40	-0.396397069	3.50E-16	-0.296458839	8.33E-11	0.208676895	4.37E-06	0.31670886	6.21E-16	0.10283433	0.092934	C4	-	Y	Y	-	-	Y	-
ENSG00000089195	TRMT6	-0.095999741	0.190499	-0.146590984	0.080384	-0.334653409	7.09E-07	-0.043954867	0.599393	-0.224019808	0.000283	-0.185360979	0.028794	C4	-	-	-	Y	-	-	-
ENSG00000089199	CHGB	-0.269775939	4.50E-10	-0.183301471	0.000839	-0.318603287	1.22E-11	0.092971987	0.065296	-0.034125643	0.547305	-0.132447605	0.042759	C4	-	-	-	Y	-	-	-
ENSG00000089248	ERP29	-0.346228852	5.64E-08	-0.305801426	7.53E-05	-0.335463001	9.65E-06	0.046419821	0.487634	0.025444159	0.729556	-0.02645785	0.855827	C4	-	Y	Y	Y	-	-	-
ENSG00000089250	NOS1	-1.016253384	0.000117	-0.183081306	0.618437	-0.194947184	0.561273	0.838821892	0.001591	0.835118587	0.00152	-0.009688185	0.988432	C3	-	Y	-	-	Y	Y	-
ENSG00000089280	FUS	-0.233883691	2.71E-08	-0.314315583	4.33E-11	-0.421115849	1.15E-23	-0.073794925	0.082583	-0.172458816	2.05E-07	-0.103983904	0.050337	C4	-	-	Y	Y	-	-	-
ENSG00000089505	CMTM1	1.007864594	2.14E-05	0.480764742	0.168699	0.823842815	0.003087	-0.519431066	0.024384	-0.16902858	0.513542	0.344719713	0.362352	C1	-	Y	-	Y	Y	-	-
ENSG00000089682	RBM41	0.464723583	5.57E-06	0.142599142	0.369441	0.185124211	0.123856	-0.315562264	0.00297	-0.265043582	0.00414	0.045184242	0.851487	C1	-	Y	-	-	Y	-	-
ENSG00000089723	OTUB2	-0.555879144	0.003977	-0.289515717	0.215031	-0.034093656	0.919757	0.272328016	0.231964	0.536136708	0.013648	0.258080031	0.45687	C4	-	Y	-	-	-	Y	-
ENSG00000089818	NECAP1	0.321165007	6.29E-06	0.11968718	0.243832	0.114738332	0.269468	-0.194860654	0.007822	-0.191715839	0.012548	-0.002037372	0.993273	C5	-	Y	-	-	-	-	-
ENSG00000089820	ARHGAP4	-0.527790552	2.89E-05	-0.228619168	0.138994	-0.188968395	0.223259	0.305781873	0.024427	0.353792722	0.008104	0.042498205	0.892368	C4	-	Y	-	-	Y	Y	-
ENSG00000089847	ANKRD24	-2.1909531	2.96E-05	-1.02715774	0.045653	-0.458636208	0.411447	1.169715907	0.099095	1.74745109	0.005123	0.57182595	NA	C3	-	Y	Y	-	-	Y	-
ENSG00000089902	RCOR1	0.392379065	1.97E-06	0.32718105	0.000458	0.159676263	0.129859	-0.059785973	0.442968	-0.218327479	0.000786	-0.164128927	0.078009	C1	-	Y	Y	-	-	-	-
ENSG00000089916	GPATCH2L	0.513552165	1.59E-16	0.318389519	4.61E-05	0.329556513	1.75E-06	-0.18883454	0.003303	-0.169341241	0.003105	0.014137302	0.937456	C1	-	Y	Y	Y	-	-	-
ENSG00000090013	BLVRB	-0.689318749	0.009853	-0.478287803	0.128735	-0.357392508	0.313492	0.218309807	0.570952	0.346594515	0.360214	0.122638631	NA	C2	-	Y	-	-	-	-	-
ENSG00000090061	CCNK	0.303790531	0.002206	0.068485165	0.708114	-0.011424297	0.953102	-0.228686282	0.028178	-0.300673461	0.001785	-0.077315724	0.73031	C5	-	Y	-	-	-	Y	-
ENSG00000090263	MRPS33	-0.324987013	4.22E-06	-0.054257382	0.630407	-0.137845277	0.144261	0.277194369	3.66E-05	0.201890444	0.006402	-0.080654588	0.529229	C4	-	Y	-	-	-	-	-
ENSG00000090339	ICAM1	2.312621036	0.034391	1.695189531	NA	1.939429129	NA	-0.601680521	0.587362	-0.360084864	0.734522	0.235407714	NA	C1	-	Y	-	-	-	-	-
ENSG00000090447	TFAP4	0.094914043	0.433762	-0.12350273	0.460167	-0.326489759	0.005266	-0.211928287	0.081049	-0.406597404	7.28E-05	-0.200124759	0.277567	C5	-	-	-	Y	-	Y	-
ENSG00000090470	PDCD7	0.49891581	4.90E-14	-0.250777105	0.008298	0.062854106	0.620402	-0.74346887	2.07E-26	-0.421654213	9.59E-09	0.316344122	0.005317	C5	-	Y	-	-	Y	Y	Y
ENSG00000090612	ZNF268	-0.341080937	2.60E-05	-0.148159788	0.170055	-0.110145996	0.307055	0.199403116	0.015761	0.245591637	0.001773	0.04076164	0.822512	C4	-	Y	-	-	-	-	-
ENSG00000090615	GOLGA3	0.515497707	2.06E-18	0.256009303	0.00019	0.278689291	4.89E-06	-0.253001545	8.29E-06	-0.222223625	2.76E-05	0.025314876	0.845887	C5	-	Y	-	-	-	-	-
ENSG00000090861	AARS1	-0.542941177	1.04E-28	-0.397809224	6.35E-11	0.013318392	0.904884	0.15164992	0.014425	0.570662206	1.30E-24	0.414330554	1.70E-08	C4	-	Y	Y	-	-	Y	Y
ENSG00000090924	PLEKHG2	0.33927862	1.32E-08	0.178134145	0.013529	0.203867345	0.000854	-0.154867788	0.008552	-0.120823987	0.024886	0.029056654	0.801162	C5	-	Y	-	-	-	-	-
ENSG00000090971	NAT14	-0.2114808	0.01744	0.059948503	0.641716	0.10934433	0.301694	0.278178207	6.12E-05	0.335677078	4.64E-07	0.052157299	0.67888	C4	-	-	-	-	-	Y	-
ENSG00000091039	OSBPL8	0.58898812	3.20E-16	0.260389345	0.010383	0.279043243	0.001587	-0.322184217	1.83E-08	-0.295245294	9.65E-10	0.021418591	0.904708	C5	-	Y	-	-	Y	-	-
ENSG00000091073	DTX2	0.877661937	5.03E-09	0.572560069	0.001238	0.634288165	0.00022	-0.298678141	0.053289	-0.228430965	0.16274	0.064830445	0.853543	C5	Y	Y	Y	Y	-	-	-
ENSG00000091136	LAMB1	-0.195067306	9.72E-05	0.145918959	0.054951	0.11987604	0.092623	0.34742708	2.35E-12	0.329611801	1.93E-13	-0.023282634	0.882605	C4	-	-	-	-	Y	Y	-
ENSG00000091157	WDR7	0.485573075	9.55E-07	0.171917141	0.224265	0.359405706	0.009488	-0.307371775	0.001286	-0.11221741	0.391266	0.190163062	0.304742	C5	-	Y	-	Y	Y	-	-
ENSG00000091409	ITGA6	2.145552758	9.28E-12	2.37820445	2.72E-13	2.118814663	1.02E-09	0.238287133	0.421244	-0.011266259	0.975119	-0.255257423	0.596726	C1	-	Y	Y	Y	-	-	-
ENSG00000091428	RAPGEF4	-0.649625854	1.33E-27	0.249056425	0.020303	0.202957235	0.032002	0.9055707	6.09E-27	0.867504678	1.25E-31	-0.04457062	0.905466	C2	Y	Y	-	-	Y	Y	-
ENSG00000091490	SEL1L3	-0.455579554	0.327027	0.5326964	0.221594	0.584729689	0.13275	0.995668021	0.002417	1.056045635	0.000715	0.055787843	0.936471	C2	-	-	-	-	Y	Y	-
ENSG00000091542	ALKBH5	0.308933166	3.73E-07	0.16807967	0.039493	0.173958988	0.020519	-0.134460038	0.058811	-0.12038707	0.074147	0.008826011	0.963713	C5	-	Y	-	-	-	-	-
ENSG00000091651	ORC6	-0.119675541	0.205115	-0.128957511	0.135079	-0.396561834	7.95E-07	-0.003291881	0.978531	-0.262131658	0.003732	-0.264349915	0.00743	C5	-	-	-	Y	-	-	-
ENSG00000091656	ZFHX4	0.035401174	0.859835	0.039302956	0.88773	0.321472671	0.041913	0.008822102	0.96112	0.300426162	0.002164	0.286179669	0.079744	C4	-	-	-	Y	-	Y	-
ENSG00000092051	JPH4	0.232706666	0.000178	0.198535082	0.001166	0.315389726	5.25E-09	-0.027578284	0.719826	0.097411581	0.091621	0.119951047	0.06086	C4	-	-	-	Y	-	-	-
ENSG00000092148	HECTD1	0.440964409	1.50E-09	0.138340316	0.203503	0.154981167	0.080132	-0.296198471	5.33E-07	-0.271530719	1.05E-08	0.019390266	0.904354	C5	-	Y	-	-	-	-	-
ENSG00000092200	RPGRIP1	-0.883999083	0.015275	-0.419728075	0.343124	-1.015854782	0.011595	0.469096429	0.322086	-0.116883259	0.858389	-0.591569079	NA	C3	-	Y	-	Y	-	-	-
ENSG00000092208	GEMIN2	0.332156252	0.047537	0.066521689	0.812864	0.118290131	0.627584	-0.259340544	0.14384	-0.199162733	0.29258	0.05462716	0.896839	C5	-	Y	-	-	-	-	-
ENSG00000092421	SEMA6A	-1.02454388	1.39E-09	0.157639227	0.592033	0.230936427	0.394466	1.189815691	3.11E-09	1.270434201	4.78E-10	0.070470726	0.944059	C4	-	Y	-	-	Y	Y	-
ENSG00000092439	TRPM7	0.301390309	5.26E-06	0.10703066	0.244195	0.115132186	0.209569	-0.188154296	0.000499	-0.171572615	0.003665	0.011100926	0.947733	C5	-	Y	-	-	-	-	-
ENSG00000092470	WDR76	0.162638576	0.057438	-0.113566777	0.250237	-0.225275099	0.013712	-0.269886739	0.000241	-0.373428519	9.61E-07	-0.108900136	0.395803	C5	-	-	-	-	-	Y	-
ENSG00000092529	CAPN3	-1.181259619	0.183978	0.737911701	0.36283	0.382809929	0.649571	1.933877143	0.016373	1.579420686	0.055416	-0.35118971	NA	C2	-	-	-	-	Y	-	-
ENSG00000092531	SNAP23	0.437587169	0.000319	0.260119521	0.118712	0.256899434	0.071656	-0.171666394	0.221759	-0.165907931	0.189574	0.000442672	0.998694	C5	-	Y	-	-	-	-	-
ENSG00000092621	PHGDH	-0.763892929	1.24E-46	-0.830964736	1.13E-07	-0.515713787	4.53E-09	-0.060825442	0.767792	0.262686442	0.000586	0.318735454	0.248878	C3	-	Y	Y	Y	-	-	-
ENSG00000092758	COL9A3	-1.151509372	0.001401	-0.644470869	0.197633	-0.305798139	0.493755	0.513752399	0.343946	0.86107467	0.036778	0.341938064	NA	C4	-	Y	-	-	-	Y	-
ENSG00000092847	AGO1	0.522723864	1.73E-11	0.196685974	0.071839	0.209712219	0.023835	-0.319927168	6.91E-08	-0.298962429	7.38E-10	0.015885684	0.927911	C5	-	Y	-	-	Y	-	-
ENSG00000092853	CLSPN	-0.104722004	0.122275	-0.248173105	0.005262	-0.351855211	1.68E-05	-0.136477084	0.055118	-0.23299233	0.000421	-0.100645958	0.501333	C4	-	-	-	Y	-	-	-

ENSG00000092929	UNC13D	-0.373663334	5.57E-11	-0.096272059	0.176398	-0.078932407	0.32206	0.283954088	1.56E-07	0.309374498	5.88E-08	0.020153582	0.887295	C4	-	Y	-	-	-	Y	-
ENSG00000092978	GPATCH2	0.346735364	4.83E-05	0.014924498	0.926556	0.068279757	0.593779	-0.325333696	4.79E-07	-0.264297602	8.33E-05	0.055677141	0.686296	C5	-	Y	-	-	Y	-	-
ENSG00000093072	ADA2	-0.022215338	0.975667	1.041313858	0.050022	0.768214813	0.25515	1.06981556	0.020192	0.805825437	0.16646	-0.270117961	NA	C2	-	-	-	-	Y	-	-
ENSG00000093217	XYLB	-0.18794264	0.236739	0.250022121	0.150121	0.355240956	0.015261	0.444118914	0.000692	0.55774231	4.63E-06	0.108419782	0.639479	C4	-	-	-	Y	Y	Y	-
ENSG00000094804	CDC6	-0.349842062	5.49E-11	-0.31372147	9.30E-06	-0.452263054	4.16E-06	0.042818107	0.607259	-0.088179862	0.406967	-0.134768811	0.43015	C4	-	Y	Y	Y	-	-	-
ENSG00000095015	MAP3K1	0.343073845	5.94E-08	0.230376441	0.00159	-0.073116911	0.389646	-0.106433425	0.047939	-0.401877281	3.46E-21	-0.300589782	7.00E-09	C5	-	Y	-	-	-	Y	Y
ENSG00000095066	HOOK2	-0.330597012	0.002383	0.038474248	0.818748	0.081966619	0.531301	0.375634087	0.000761	0.427383369	4.18E-05	0.046374991	0.835119	C4	-	Y	-	-	Y	Y	-
ENSG00000095209	TMEM38B	0.492201716	8.74E-06	0.270181026	0.104036	0.262342799	0.068514	-0.21573515	0.145997	-0.215353127	0.109145	-0.00507707	0.991193	C5	-	Y	-	-	-	-	-
ENSG00000095303	PTGS1	3.095030043	4.85E-25	2.276567228	1.49E-09	1.98907437	4.62E-07	-0.814309176	0.000357	-1.090077387	2.10E-06	-0.283034493	NA	C5	-	Y	Y	Y	Y	Y	-
ENSG00000095321	CRAT	-0.300176495	0.00289	0.122428842	0.442617	0.007848345	0.968445	0.429135069	0.000114	0.322740698	0.003781	-0.112104008	0.622141	C4	-	Y	-	-	Y	Y	-
ENSG00000095383	TBC1D2	0.846032428	0.015463	0.572021185	0.211112	0.768813556	0.036766	-0.266809158	0.51581	-0.061972325	0.890282	0.198018041	NA	C1	-	Y	-	Y	-	-	-
ENSG00000095539	SEMA4G	-0.317681544	0.000511	-0.037138472	0.820283	0.043789105	0.73819	0.28714561	0.00449	0.376174825	1.95E-05	0.084027293	0.630762	C4	-	Y	-	-	-	Y	-
ENSG00000095564	BTAF1	0.497304241	3.11E-12	0.189605763	0.057073	0.165383226	0.066743	-0.30111171	3.49E-07	-0.317694046	4.43E-10	-0.021683176	0.898672	C5	Y	Y	-	-	Y	Y	-
ENSG00000095574	IKZF5	0.381680304	4.01E-05	-0.02748254	0.868255	0.173631854	0.099439	-0.402781517	7.19E-06	-0.19356055	0.037199	0.203808844	0.089669	C5	-	Y	-	-	Y	-	-
ENSG00000095637	SORBS1	-0.34760227	0.000146	0.012417418	0.941118	0.135686974	0.23433	0.366244747	4.34E-07	0.497645228	1.20E-11	0.125964771	0.265132	C4	-	Y	-	-	Y	Y	-
ENSG00000095739	BAMBI	-0.119494999	0.211625	0.305720402	0.003322	0.063141619	0.664469	0.431636062	6.96E-09	0.198144461	0.019352	-0.239378946	0.046118	C4	-	-	Y	-	Y	-	-
ENSG00000095752	IL11	0.628538954	0.003898	0.048279687	0.908388	0.225485446	0.487638	-0.574288431	0.010819	-0.388483336	0.107199	0.180040175	0.702408	C5	-	Y	-	-	Y	-	-
ENSG00000095951	HIVEP1	0.160103151	0.197733	0.355427294	0.003205	0.169964887	0.263739	0.201636669	0.066804	0.024113861	0.884103	-0.18272207	0.300221	C1	-	-	Y	-	-	-	-
ENSG00000096060	FKBP5	-0.503271947	8.10E-14	-0.45936668	1.86E-09	-0.385766561	6.22E-08	0.050154317	0.640717	0.132234651	0.121942	0.076571542	0.609142	C4	-	Y	Y	Y	-	-	-
ENSG00000096063	SRPK1	0.576134573	1.05E-25	0.3033392	4.18E-06	0.213149154	0.001163	-0.266156333	1.26E-07	-0.34831053	3.81E-13	-0.087584869	0.31635	C5	Y	Y	Y	-	-	Y	-
ENSG00000096070	BRPF3	0.25646062	0.000302	-0.072588116	0.514639	-0.020854318	0.862059	-0.322458273	1.62E-07	-0.262971097	8.71E-06	0.054262861	0.674131	C5	-	-	-	-	Y	-	-
ENSG00000096092	TMEM14A	-0.544429374	8.51E-15	-0.30181793	4.61E-05	-0.310936874	1.58E-05	0.24908291	0.001091	0.24824423	0.001066	-0.006194068	0.975811	C4	-	Y	Y	Y	-	-	-
ENSG00000096654	ZNF184	0.108750209	0.448172	-0.500309809	0.000266	-0.048306122	0.807548	-0.602620404	5.43E-08	-0.142504509	0.269519	0.454712941	0.001465	C5	-	-	Y	-	Y	-	Y
ENSG00000096717	SIRT1	0.214637073	0.000149	-0.185672605	0.016979	-0.053795003	0.57461	-0.393638526	5.49E-11	-0.253713105	3.23E-05	0.134955198	0.212638	C5	-	-	-	-	Y	-	-
ENSG00000096746	HNRNPH3	-0.320985894	4.68E-18	-0.175472104	0.002923	-0.23623235	4.54E-06	0.152142329	0.001212	0.099639148	0.033115	-0.057797037	0.571585	C4	Y	Y	-	-	-	-	-
ENSG00000096872	IFT74	-0.132577116	0.339116	0.178918864	0.204902	0.107424443	0.476113	0.318277561	0.009407	0.254706521	0.044277	-0.068604679	0.768051	C4	-	-	-	-	Y	-	-
ENSG00000096968	JAK2	0.40118435	0.002627	0.161745225	0.357132	0.109996176	0.561907	-0.23250839	0.111247	-0.276315503	0.057614	-0.048954033	0.883781	C5	-	Y	-	-	-	-	-
ENSG00000099204	ABLIM1	-0.316832314	3.45E-07	-0.009008997	0.951347	0.02873793	0.785292	0.313824302	3.54E-08	0.360193768	8.98E-16	0.040855264	0.759725	C4	-	Y	-	-	Y	Y	-
ENSG00000099219	ERMP1	-0.353720074	0.015346	-0.012693956	0.962498	0.108114809	0.574529	0.347469212	0.021418	0.476538019	0.000644	0.123780694	0.635328	C4	-	Y	-	-	Y	Y	-
ENSG00000099250	NRP1	-0.6727771	0.002948	0.14883694	0.710174	0.059715815	0.87249	0.828525067	0.002169	0.746967684	0.002722	-0.087601296	0.897588	C2	-	Y	-	-	Y	Y	-
ENSG00000099251	HSD17B7P2	0.39227724	0.217888	-0.23881401	0.691295	0.662583789	0.041709	-0.624485523	0.125053	0.284732796	0.405311	0.904111284	NA	C1	-	-	-	Y	-	-	-
ENSG00000099256	PRTFDC1	-0.525276604	0.000385	-0.591175706	0.000546	-0.788433517	1.64E-06	-0.05957416	0.806921	-0.248700278	0.219464	-0.194977228	0.559898	C3	-	Y	Y	Y	-	-	-
ENSG00000099260	PALMD	-1.244112046	0.060345	-0.062790434	0.950272	0.307632505	0.657075	1.194555283	0.115769	1.565866979	0.016504	0.37265076	NA	C2	-	-	-	-	-	Y	-
ENSG00000099282	TSPAN15	-0.865116953	6.05E-10	-0.24210584	0.153488	-0.361715113	0.022172	0.628973248	6.16E-05	0.518372223	0.002036	-0.116346658	0.697586	C2	-	Y	-	Y	Y	Y	-
ENSG00000099290	WASHC2A	0.348212098	5.57E-08	0.079497945	0.395287	0.04442193	0.675519	-0.262501218	1.11E-05	-0.289098842	3.61E-06	-0.032165613	0.832353	C5	-	Y	-	-	-	-	-
ENSG00000099308	MAST3	0.392429775	2.21E-05	0.095245893	0.494978	0.315897196	0.003785	-0.290932241	0.001209	-0.06213958	0.602921	0.223461162	0.088061	C5	-	Y	-	Y	-	-	-
ENSG00000099330	OCEL1	-0.527150592	0.012378	-0.278747222	0.286654	-0.087570938	0.791882	0.254635458	0.24573	0.455541549	0.033437	0.195004123	0.586559	C4	-	Y	-	-	-	Y	-
ENSG00000099331	MYO9B	0.339241917	7.35E-12	0.116684263	0.086899	0.137434453	0.023776	-0.216019681	9.55E-06	-0.187175359	8.54E-05	0.023474859	0.849728	C5	-	Y	-	-	-	-	-
ENSG00000099338	CATSPERG	-0.364931239	0.239381	0.223533818	0.542655	0.251322722	0.451625	0.59443801	0.037483	0.630958724	0.025969	0.030432097	0.963632	C4	-	-	-	-	Y	Y	-
ENSG00000099365	STX1B	0.516194822	8.86E-06	0.588731476	4.99E-08	0.493959139	8.83E-06	0.078858452	0.561305	-0.007637242	0.960892	-0.092116418	0.604908	C1	-	Y	Y	Y	-	-	-
ENSG00000099617	EFNA2	0.423975882	0.000176	0.26396672	0.142711	0.302671224	0.028128	-0.154088863	0.317548	-0.106247065	0.426992	0.04173414	0.905466	C5	-	Y	-	Y	-	-	-
ENSG00000099622	CIRBP	-0.366753872	6.81E-14	-0.215578176	0.000453	-0.439604513	2.54E-14	0.157605628	7.04E-05	-0.057702813	0.242115	-0.220958288	4.64E-05	C4	-	Y	-	Y	-	-	-
ENSG00000099625	CBARP	0.6214321	1.87E-12	0.17489209	0.170076	0.288743506	0.006723	-0.439990728	2.12E-07	-0.317941191	0.00015	0.116669674	0.454595	C5	-	Y	-	-	Y	Y	-
ENSG00000099785	MARCHF2	-0.798165577	1.25E-06	-0.170659732	0.449112	-0.183728734	0.377433	0.634158148	8.41E-05	0.629724904	8.91E-05	-0.009836104	0.981656	C2	-	Y	-	-	Y	Y	-
ENSG00000099795	NDUFB7	-0.309321872	0.019799	-0.163356346	0.342106	-0.228941198	0.133954	0.153170245	0.130115	0.095604927	0.385898	-0.062890589	0.739067	C4	-	Y	-	-	-	-	-
ENSG00000099797	TECR	-0.319388677	1.65E-05	-0.149674112	0.175221	-0.089590289	0.402445	0.176520161	0.03181	0.244585409	0.000589	0.062865598	0.701775	C4	-	Y	-	-	-	-	-
ENSG00000099804	CDC34	0.338630934	1.13E-05	0.124669719	0.337673	0.090425216	0.412607	-0.206821563	0.012241	-0.233321386	0.000436	-0.031850018	0.879436	C5	-	Y	-	-	-	-	-
ENSG00000099810	MTAP	0.490575345	2.57E-12	0.118087754	0.215195	0.147668125	0.133228	-0.366131999	9.31E-12	-0.328229763	1.68E-07	0.032466755	0.835119	C5	-	Y	-	-	Y	Y	-
ENSG00000099814	CEP170B	0.306822235	0.000798	0.088708269	0.575725	0.199188894	0.02413	-0.211719427	0.062509	-0.092791895	0.347327	0.113081216	0.513349	C5	-	Y	-	-	-	-	-
ENSG00000099822	HCN2	-0.141833085	0.301276	0.205878959	0.104826	0.266027518	0.021724	0.354367011	0.001465	0.422739987	9.20E-05	0.063219758	0.749894	C4	-	-	-	-	Y	Y	-
ENSG00000099860	GADD45B	2.996253041	1.35E-05	3.04273434	0.000232	2.667041964	0.001037	0.050175352	0.946974	-0.312638937	0.602482	-0.37036684	NA	C1	-	Y	Y	Y	-	-	-
ENSG00000099866	MADCAM1	-1.043069026	0.235728	0.456154313	NA	0.572294549	0.511685	1.50939606	0.05759	1.629714136	0.040364	0.118392684	NA	C2	-	-	-	-	-	Y	-
ENSG00000099875	MKNK2	0.469219716	7.06E-09	0.057382667	0.687775	0.227095899	0.01707	-0.405473675	4.63E-09	-0.227176244	0.000985	0.172667003	0.088393	C5	-	Y	-	-	Y	-	-
ENSG00000099889	ARVCF	-0.341338389	2.06E-08	-0.149911009	0.04235	-0.092459824	0.225077	0.198155503	0.000981	0.263892507	2.66E-06	0.060283693	0.553113	C4	-	Y	-	-	-	-	-
ENSG00000099949	LZTR1	0.355226705	2.03E-06	0.063011538	0.669723	0.182584263	0.065019	-0.285503778	0.000975	-0.157847856	0.064306	0.12250808	0.448956	C5	-	Y	-	-	-	-	-
ENSG00000099953	MMP11	-0.956839308	4.98E-10	-0.365626862	0.028878	-0.428187376	0.008325	0.5978161	0.00011	0.54377734	0.00074	-0.059493877	0.857353	C3	-	Y	Y	Y	Y	Y	-
ENSG00000099957	P2RX6	-1.076861097	7.72E-12	-0.177121138	0.280905	-0.123372448	0.499889	0.907313326	7.81E-09	0.968224362	1.93E-09	0.05649689	0.853543	C3	-	Y	-	-	Y	Y	-
ENSG00000100027	YPEL1	-0.114134771	0.470639	0.196621966	0.194287	0.315998407	0.020348	0.317163151	0.008451	0.444852521	0.000169	0.122426927	0.522361	C4	-	-	-	Y	Y	Y	-
ENSG00000100038	TOP3B	0.582269261	0.039056	-0.024111766	0.967013	0.041781515	0.931764	-0.600762691	0.021625	-0.526215699	0.035698	0.068871207	NA	C5	-	Y	-	-	Y	Y	-
ENSG00000100065	CARD10	-0.498955706	0.012799	-0.465735228	0.056255	-0.492517823	0.032558	0.039914864	0.90451	0.020912589	0.949771	-0.024472514	0.97007	C4	-	Y	-	Y	-	-	-
ENSG00000100068	LRP5L	0.279888821	0.029429	0.461570669	0.000427	0.248867946	0.086767	0.188061792	0.167419	-0.016337864	0.930472	-0.209805277	0.254336	C4	-	-	Y	-	-	-	-
ENSG00000100075	SLC25A1	-0.293405937	7.00E-05	-0.118025597	0.288987	0.0022616	0.987816	0.181629808	0.01932	0.310338936	1.09E-05	0.123265895	0.341115	C4	-	-	-	-	-	Y	-
ENSG00000100084	HIRA	0.279016085	0.010849	-0.045116089	0.821616	-0.054629659	0.75225	-0.316660523	0.000807	-0.318710985	0.000449	-0.007365109	0.979342	C5	Y	-	-	-	Y	Y	-
ENSG00000100097	LGALS1	-0.372015027	1.52E-08	-0.263785287	0.006693	-0.252729715	0.006757	0.114495016	0.139812	0.134465616	0.059249	0.014006056	0.952524	C4	Y	Y	-	-	-	-	-
ENSG00000100106	TRIOBP	-0.237972453	0.009801	0.288560982	0.004905	0.220905323	0.035639	0.532598367	3.96E-12	0.474050836	1.30E-09	-0.06425381	0.697239	C4	-	-	-	-	Y	Y	-
ENSG00000100162	CENPM	-0.478652594	0.012881	-0.415551701	0.048668	-0.252530255	0.259567	0.069672152	0.786006	0.240956248	0.247284	0.165554554	0.606784	C4	-	Y	Y	-	-	-	-
ENSG00000100197	CYP2D6	-0.623621007	0.194152	0.358779761	0.444897	-0.059885724	0.922384	0.987484278	0.012442	0.577379028	0.219137	-0.417036566	NA	C3	-	-	-	-	Y	-	-

ENSG00000100234	TIMP3	1.076446268	9.71E-06	0.462396194	0.183392	0.313711035	0.383569	-0.607955112	0.018076	-0.747690918	0.002488	-0.145426089	0.804185	C5	-	Y	-	-	Y	Y	-
ENSG00000100239	PPP6R2	0.200401612	0.008748	0.331667493	4.98E-05	0.225603568	0.004688	0.138136031	0.056117	0.039856844	0.634787	-0.103676099	0.326298	C4	-	-	Y	-	-	-	-
ENSG00000100271	TTLL1	-0.505256901	0.00163	-0.075657308	0.762114	-0.061051512	0.795268	0.435517573	0.010211	0.458750873	0.006377	0.017337161	0.967582	C4	-	Y	-	-	Y	Y	-
ENSG00000100284	TOM1	-0.257809192	0.044134	0.051222142	0.799537	-0.210308106	0.166578	0.315541628	0.011791	0.062477555	0.720738	-0.258504573	0.139381	C4	-	-	-	-	Y	-	-
ENSG00000100292	HMOX1	-0.813433353	0.121944	-1.235488945	0.049986	-1.047338264	0.073507	-0.413865756	0.544782	-0.218164984	0.756559	0.190749183	NA	C4	-	-	Y	-	-	-	-
ENSG00000100297	MCM5	-0.274614041	3.40E-07	-0.141832914	0.044044	-0.416056893	2.45E-09	0.139387046	0.018687	-0.126912035	0.069968	-0.270979445	0.0012	C5	-	-	-	Y	-	-	-
ENSG00000100299	ARSA	-0.223003756	0.03708	0.036238364	0.857863	0.10278407	0.515353	0.265659672	0.023763	0.340079926	0.002528	0.069977108	0.795434	C4	-	-	-	-	-	Y	-
ENSG00000100336	APOL4	-1.817502406	0.000713	-0.968051501	0.082491	-1.25712768	0.026479	0.860930829	0.260326	0.576932661	0.519626	-0.289026712	NA	C3	-	Y	-	Y	-	-	-
ENSG00000100345	MYH9	0.64487226	4.34E-51	0.373778149	2.82E-17	0.258774629	5.53E-10	-0.264556311	5.24E-10	-0.37137814	2.77E-23	-0.112029456	0.037309	C5	-	Y	Y	-	-	Y	-
ENSG00000100350	FOXRED2	-0.450922954	1.79E-13	-0.187568451	0.006052	-0.216499804	0.000207	0.269918511	3.51E-05	0.24915878	3.48E-05	-0.026058403	0.847801	C4	-	Y	-	-	-	-	-
ENSG00000100354	TNRC6B	0.432875783	0.000635	0.138554909	0.342762	0.330322017	0.004789	-0.287293305	0.027183	-0.087935972	0.565072	0.194618356	0.222477	C5	-	Y	-	Y	-	-	-
ENSG00000100359	SGSM3	0.549671221	0.000126	0.329756584	0.04956	0.324981697	0.081237	-0.213204503	0.173268	-0.210176751	0.228275	-0.002483011	0.995987	C5	-	Y	Y	-	-	-	-
ENSG00000100372	SLC25A17	0.303827718	0.00113	0.073003372	0.659206	0.023972552	0.882474	-0.224259987	0.03873	-0.265127823	0.005941	-0.046091074	0.853558	C5	-	Y	-	-	-	-	-
ENSG00000100413	POLR3H	-0.325158224	0.00019	-0.215241417	0.046468	-0.252401448	0.013585	0.116313859	0.303481	0.087575731	0.456016	-0.034063768	0.891721	C4	-	Y	-	-	-	-	-
ENSG00000100416	TRMU	0.36269373	2.58E-10	0.024373212	0.845706	0.026474364	0.784467	-0.331812797	2.42E-08	-0.321312024	1.62E-10	0.004940642	0.978268	C5	-	Y	-	-	Y	Y	-
ENSG00000100418	DESI1	0.354297726	0.000339	0.339735371	0.001274	0.366658265	0.000345	-0.008043921	0.955704	0.027145072	0.841118	0.029838786	0.895364	C5	-	Y	Y	Y	-	-	-
ENSG00000100426	ZBED4	0.322565906	9.12E-07	0.177754563	0.012346	0.14832655	0.036748	-0.138370951	0.010644	-0.159663469	0.002432	-0.026753882	0.793199	C5	-	Y	-	-	-	-	-
ENSG00000100429	HDAC10	0.383100801	0.000483	0.2332367	0.069317	0.244720428	0.044555	-0.14375169	0.25692	-0.123626099	0.335821	0.014337012	0.960084	C5	-	Y	-	-	-	-	-
ENSG00000100439	ABHD4	0.738962439	2.71E-07	0.456755589	0.021149	0.556153702	0.000629	-0.275393892	0.106318	-0.168207682	0.309811	0.101567766	0.762735	C1	-	Y	Y	Y	-	-	-
ENSG00000100473	COCH	-0.424749908	0.001874	-0.395871391	0.011912	-0.348846181	0.019135	0.035030482	0.85371	0.090233577	0.579312	0.050048083	0.872221	C4	-	Y	Y	Y	-	-	-
ENSG00000100478	AP4S1	-0.580500569	0.000173	-0.203999069	0.335477	-0.205778688	0.271172	0.381995178	0.041748	0.389302284	0.029226	0.001124813	0.997573	C4	-	Y	-	-	Y	Y	-
ENSG00000100483	VCPKMT	-0.265498279	0.025173	-0.12075639	0.453614	-0.338333756	0.00795	0.150940673	0.25675	-0.058376862	0.717246	-0.214721668	0.218399	C4	-	-	-	Y	-	-	-
ENSG00000100485	SOS2	0.40653938	1.09E-09	0.22824074	0.00297	0.110955599	0.248358	-0.171972188	0.00449	-0.28090998	9.59E-06	-0.114330294	0.266414	C5	-	Y	-	-	-	-	-
ENSG00000100490	CDKL1	-0.530909093	0.003032	-0.006801638	0.983497	-0.203698394	0.330518	0.530237656	0.005971	0.342284746	0.087079	-0.193870438	0.520704	C4	-	Y	-	-	Y	-	-
ENSG00000100503	NIN	0.345258653	2.47E-08	0.154945957	0.094879	0.157111928	0.036625	-0.183921888	0.001665	-0.173588426	0.000151	0.005024404	0.977582	C5	-	Y	-	-	-	-	-
ENSG00000100522	GNPNAT1	0.396536826	6.21E-08	0.117614847	0.244704	0.231091001	0.011871	-0.272431635	2.12E-06	-0.151102735	0.025792	0.116129434	0.263542	C5	-	Y	-	-	-	-	-
ENSG00000100526	CDKN3	-0.472403658	3.96E-06	-0.042143251	0.797731	-0.088373505	0.542369	0.436548321	2.78E-05	0.398720018	0.000319	-0.043198787	0.860105	C4	-	Y	-	-	Y	Y	-
ENSG00000100554	ATP6V1D	0.432124364	3.10E-09	0.109878866	0.337305	0.156020091	0.09448	-0.315976771	2.04E-05	-0.261767272	0.000161	0.049118837	0.770649	C5	-	Y	-	-	Y	-	-
ENSG00000100564	PIGH	0.387446774	0.002243	0.163962945	0.3172	0.197747628	0.173767	-0.217383984	0.129542	-0.175122097	0.225745	0.036691307	0.910221	C5	-	Y	-	-	-	-	-
ENSG00000100577	GSTZ1	-0.445193301	0.000244	-0.308322982	0.014864	-0.230435206	0.082897	0.143637975	0.302628	0.229967566	0.075379	0.081279001	0.70497	C4	-	Y	Y	-	-	-	-
ENSG00000100578	KIAA0586	0.430630214	1.04E-07	0.329853107	0.000484	0.238594542	0.013111	-0.094203161	0.229542	-0.177271241	0.011439	-0.088460399	0.470531	C1	-	Y	Y	-	-	-	-
ENSG00000100580	TMED8	0.362075031	3.30E-07	0.166044849	0.096554	0.147639336	0.092829	-0.189437155	0.002699	-0.199779519	0.000195	-0.015656549	0.928882	C5	-	Y	-	-	-	-	-
ENSG00000100592	DAAM1	0.485356813	3.20E-12	0.246092806	0.010183	0.205954345	0.019764	-0.232724898	0.001855	-0.264972648	0.000108	-0.037400972	0.841305	C5	-	Y	-	-	-	-	-
ENSG00000100600	LGMN	-0.463764614	3.90E-06	-0.124781474	0.371881	-0.037208105	0.81629	0.346267067	0.003466	0.441149491	6.71E-05	0.090112131	0.681162	C4	-	Y	-	-	Y	Y	-
ENSG00000100601	ALKBH1	0.169848416	0.129736	-0.338556008	0.015506	-0.085436679	0.578579	-0.501906607	2.96E-05	-0.24074769	0.037156	0.256392286	0.168713	C5	-	-	Y	-	Y	-	-
ENSG00000100604	CHGA	-0.225200514	1.33E-05	-0.590618073	3.75E-05	-0.525325185	0.000141	-0.359214225	0.003566	-0.285498469	0.020719	0.068403119	0.860218	C5	-	-	Y	Y	Y	-	-
ENSG00000100632	ERH	-0.110208567	0.168516	-0.221267942	0.012278	-0.333988995	9.56E-06	-0.104244972	0.039307	-0.208880874	1.14E-07	-0.109929582	0.112694	C4	Y	-	-	Y	-	-	-
ENSG00000100647	SUSD6	0.415745176	0.001011	0.424911728	0.000561	0.235909493	0.077693	0.015486765	0.930368	-0.165262847	0.205247	-0.185906331	0.229419	C1	-	Y	Y	-	-	-	-
ENSG00000100650	SRSF5	-0.146548239	0.002499	-0.179374468	0.000518	-0.300839135	1.42E-09	-0.026335332	0.660679	-0.139623663	0.002321	-0.118582989	0.073029	C4	-	-	-	Y	-	-	-
ENSG00000100664	EIF5	0.320576156	1.17E-15	-0.012904971	0.906418	-0.182627434	0.000863	-0.32684715	6.65E-10	-0.488768648	2.85E-34	-0.166879608	0.046118	C5	Y	Y	-	-	Y	Y	-
ENSG00000100678	SLC8A3	-0.16334247	0.003917	0.277871378	0.000218	0.241207937	0.000207	0.447554676	2.17E-13	0.419375514	2.18E-15	-0.033895185	0.83368	C4	-	-	-	-	Y	Y	-
ENSG00000100711	ZFYVE21	0.418281082	0.002805	0.188162573	0.338588	0.396298228	0.0081	-0.223557864	0.163861	-0.007243253	0.969238	0.210874274	0.361437	C1	-	Y	-	Y	-	-	-
ENSG00000100731	PCNX1	0.340870993	0.00018	0.068176971	0.659188	0.048800421	0.71941	-0.266164857	0.001805	-0.27746859	0.000169	-0.016653733	0.943834	C5	-	Y	-	-	-	-	-
ENSG00000100744	GSKIP	0.242090069	0.002471	-0.097593621	0.401594	0.032261116	0.806552	-0.33322018	9.23E-06	-0.195479189	0.012634	0.132507171	0.2866	C5	-	-	-	-	Y	-	-
ENSG00000100749	VRK1	-0.429951397	7.68E-07	-0.213932231	0.037431	-0.430902786	2.06E-05	0.222316303	0.020799	0.013429149	0.930024	-0.214046854	0.137352	C4	Y	Y	-	Y	-	-	-
ENSG00000100767	PAPLN	-0.732703857	0.000263	-0.16923938	0.590963	-0.477232632	0.031611	0.570476618	0.013964	0.27052231	0.283495	-0.304871751	0.389811	C2	-	Y	-	Y	Y	-	-
ENSG00000100802	C14orf93	-0.544379736	2.30E-05	-0.188969932	0.263511	-0.228653682	0.126709	0.361804223	0.004613	0.330481414	0.008774	-0.036373066	0.905004	C2	-	Y	-	-	Y	Y	-
ENSG00000100813	ACIN1	0.335685521	1.27E-19	-0.071938647	0.167104	0.007382451	0.918021	-0.401076214	9.01E-38	-0.313749035	7.15E-24	0.082068399	0.116803	C5	-	Y	-	-	Y	Y	-
ENSG00000100815	TRIP11	0.348634173	0.000511	-0.01362483	0.948173	0.344305465	0.000874	-0.356068361	7.70E-05	0.010057334	0.932532	0.360925252	0.000508	C4	-	Y	-	Y	Y	-	Y
ENSG00000100852	ARHGAP5	0.410580251	0.000904	0.170842482	0.335984	0.173611901	0.266205	-0.234571535	0.002428	-0.223290106	0.000624	0.006104765	0.978055	C1	-	Y	-	-	-	-	-
ENSG00000100867	DHRS2	-0.584472398	0.007754	-1.292281303	1.65E-06	-0.931491883	0.000133	-0.699381454	0.021132	-0.331780795	0.275144	0.36279307	NA	C3	-	Y	Y	Y	Y	-	-
ENSG00000100889	PCK2	-0.95036669	2.99E-30	-0.499791081	2.40E-06	-0.157935934	0.128946	0.456998707	4.23E-06	0.807284704	1.64E-24	0.344740879	0.004047	C3	Y	Y	Y	-	Y	Y	Y
ENSG00000100897	DCAF11	-0.342476572	2.20E-06	-0.160947528	0.056383	-0.209213481	0.005579	0.188474024	0.021277	0.148072295	0.068115	-0.045300891	0.766813	C4	-	Y	-	-	-	-	-
ENSG00000100908	EMC9	-0.334979877	0.026397	-0.058150149	0.792109	-0.154178573	0.361404	0.284069887	0.084486	0.195750323	0.254733	-0.093412673	0.744989	C4	-	Y	-	-	-	-	-
ENSG00000100949	RABGGTA	-0.123906298	0.426275	0.164283093	0.313631	0.197176641	0.168091	0.294991214	0.018527	0.336303658	0.004687	0.035146538	0.899085	C4	-	-	-	-	-	Y	-
ENSG00000100968	NFATC4	-0.529455571	7.82E-05	-0.196777751	0.280291	-0.037600126	0.856889	0.339718325	0.030089	0.506839412	0.000212	0.162271753	0.501333	C4	-	Y	-	-	Y	Y	-
ENSG00000100979	PLTP	-0.318596106	9.34E-05	-0.156190353	0.192167	-0.140323692	0.28104	0.168945558	0.102536	0.193339417	0.075594	0.017750489	0.952948	C4	-	Y	-	-	-	-	-
ENSG00000100983	GSS	-0.323854923	9.11E-06	0.03101867	0.824312	-0.046831451	0.661851	0.361236444	2.77E-06	0.291703196	2.48E-05	-0.074650025	0.598842	C4	-	Y	-	-	Y	-	-
ENSG00000101040	ZMYND8	-0.646837271	2.20E-19	-0.206486443	0.022095	-0.030923401	0.783586	0.44660666	1.04E-09	0.630328584	9.57E-23	0.178612912	0.066506	C4	Y	Y	-	-	Y	Y	-
ENSG00000101104	PABPC1L	-0.186865837	0.023396	0.013036875	0.925589	0.136947718	0.1537	0.206341279	0.002715	0.33834556	1.00E-06	0.126614022	0.21454	C4	-	-	-	-	-	Y	-
ENSG00000101109	STK4	0.671661858	2.93E-25	0.410937953	8.55E-09	0.34097441	4.35E-06	-0.254068009	1.50E-05	-0.316231759	9.13E-08	-0.067388042	0.565527	C1	-	Y	Y	Y	-	Y	-
ENSG00000101115	SALL4	-0.752639765	0.004411	-0.525815919	0.054665	-0.502776579	0.061951	0.232761954	0.519899	0.265039217	0.450645	0.026274965	NA	C3	-	Y	-	-	-	-	-
ENSG00000101144	BMP7	-0.485798418	8.65E-06	-0.337617405	0.00766	-0.238581738	0.068225	0.154540344	0.259929	0.26204329	0.036904	0.102051018	0.644363	C4	-	Y	Y	-	-	-	-
ENSG00000101146	RAE1	-0.134990827	0.038816	-0.214890691	0.008415	-0.343607417	2.08E-06	-0.073735481	0.34616	-0.193848505	0.003028	-0.125821506	0.270286	C4	-	-	-	Y	-	-	-
ENSG00000101152	DNAJC5	0.328755325	6.24E-11	0.045207073	0.595156	-0.020910802	0.820284	-0.277180295	2.40E-08	-0.334936254	8.53E-12	-0.063181955	0.519048	C5	-	Y	-	-	-	Y	-
ENSG00000101181	MTG2	-0.060820866	0.555926	-0.303493618	0.000866	-0.17852721	0.054129	-0.236297718	0.009835	-0.103079855	0.298602	0.127930653	0.361691	C4	-	-	Y	-	-	-	-
ENSG00000101187	SLCO4A1	0.553187566	5.06E-09	0.474097236	6.14E-05	0.404920184	9.51E-05	-0.072478003	0.585886	-0.133551678	0.205285	-0.066435757	0.76649	C1	-	Y	Y	Y	-	-	-

ENSG00000101213	PTK6	-0.793570424	0.003854	-0.017332837	0.970003	-0.331311662	0.355573	0.783263447	0.008186	0.476965396	0.192094	-0.312286744	NA	C2	-	Y	-	-	Y	-	-
ENSG00000101216	GMEB2	-0.14554469	0.158211	-0.193318413	0.097198	-0.345583642	0.000902	-0.041461776	0.766385	-0.185322349	0.097589	-0.149063796	0.380618	C4	-	-	-	Y	-	-	-
ENSG00000101222	SPEF1	-1.084086079	0.088289	-1.460476927	0.040111	-0.923697183	0.142828	-0.372222336	0.735773	0.177562425	0.86241	0.543340313	NA	C3	-	-	Y	-	-	-	-
ENSG00000101236	RNF24	0.500810098	1.58E-08	0.323820632	0.002451	0.317311457	0.00101	-0.171086389	0.031892	-0.169287452	0.017572	-0.003288159	0.988432	C5	-	Y	Y	Y	-	-	-
ENSG00000101255	TRIB3	-0.234530008	0.049869	-0.311392069	0.031694	-0.220982047	0.130646	-0.070150367	0.656033	0.028156872	0.870197	0.093368966	0.715619	C4	-	-	Y	-	-	-	-
ENSG00000101280	ANGPT4	1.852126573	0.033516	1.060797864	NA	-0.146410907	NA	-0.78035998	0.370884	-1.988481771	0.04107	-1.203445903	NA	C5	-	Y	-	-	-	Y	-
ENSG00000101282	RSPO4	-0.549021178	0.069372	-0.363657067	0.26822	-0.627595011	0.0489	0.192236851	0.622408	-0.063013492	0.898421	-0.260907507	NA	C3	-	-	-	Y	-	-	-
ENSG00000101298	SNPH	-0.428658022	0.013838	0.147705772	0.504665	0.175614142	0.379957	0.582722878	0.00068	0.618990124	0.000248	0.030760956	0.941862	C4	-	Y	-	-	Y	Y	-
ENSG00000101311	FERMT1	-1.804771769	0.042601	-0.676777211	NA	-0.307660175	0.749091	1.131935881	0.338807	1.513054456	0.139504	0.373105873	NA	C3	-	Y	-	-	-	-	-
ENSG00000101331	CCM2L	-0.766357102	0.023443	-0.365193108	0.343679	-0.192648532	0.628087	0.405847614	0.336835	0.587970036	0.117139	0.175399995	NA	C2	-	Y	-	-	-	-	-
ENSG00000101333	PLCB4	-0.244268867	0.008363	-0.128502499	0.304168	0.078067506	0.520431	0.122130292	0.142212	0.337088539	2.46E-07	0.209572275	0.020798	C4	-	-	-	-	-	Y	-
ENSG00000101347	SAMHD1	-0.203779291	0.09889	0.049770388	0.781866	0.13176891	0.319274	0.260008497	0.023379	0.350075148	0.000804	0.085357133	0.647158	C4	-	-	-	-	-	Y	-
ENSG00000101353	MROH8	-0.82871233	0.004449	-0.254835615	0.470093	-0.117385388	0.747031	0.578095174	0.08041	0.726000313	0.017572	0.140192924	0.797818	C3	-	Y	-	-	-	Y	-
ENSG00000101363	MANBAL	-0.30692526	2.80E-05	-0.098996065	0.399429	-0.209285569	0.012571	0.214345034	0.009959	0.112449959	0.173792	-0.10736489	0.432943	C4	-	Y	-	-	-	-	-
ENSG00000101400	SNTA1	-0.352048207	0.008012	-0.170333772	0.33435	-0.0190747	0.935157	0.188333384	0.219845	0.348165126	0.014436	0.15400793	0.528129	C4	-	Y	-	-	-	Y	-
ENSG00000101412	E2F1	-0.207716478	0.007797	-0.16673801	0.121624	-0.375775747	0.000179	0.048508172	0.549703	-0.153545792	0.05302	-0.206160844	0.085692	C4	-	-	-	Y	-	-	-
ENSG00000101413	RPRD1B	-0.183618583	0.002846	-0.410251092	1.49E-09	-0.262840988	7.65E-05	-0.220152269	0.000933	-0.064628345	0.392898	0.150284802	0.1223	C4	-	-	Y	-	-	-	-
ENSG00000101417	PXMP4	-0.319897397	0.021731	-0.19778605	0.230772	0.044853426	0.820922	0.127971574	0.476836	0.37942871	0.007589	0.245827043	0.215908	C4	-	Y	-	-	-	Y	-
ENSG00000101439	CST3	-0.31412411	0.00201	-0.19508402	0.171534	-0.139340152	0.240587	0.125424448	0.351573	0.189750695	0.068106	0.05902986	0.800796	C4	-	Y	-	-	-	-	-
ENSG00000101464	PIGU	0.378033975	4.93E-05	0.242078974	0.048277	0.156534753	0.209489	-0.12935078	0.237782	-0.206760389	0.03645	-0.082752356	0.667281	C5	Y	Y	-	-	-	-	-
ENSG00000101489	CELF4	0.465663895	3.95E-12	0.106033259	0.344115	0.257049167	0.001813	-0.35296927	5.59E-07	-0.193745185	0.005332	0.153818269	0.186277	C5	-	Y	-	-	Y	-	-
ENSG00000101544	ADNP2	-0.281028235	3.72E-06	-0.310050261	3.73E-06	-0.240438258	1.66E-05	-0.022640164	0.820721	0.055380214	0.45613	0.072481757	0.521339	C4	-	-	Y	-	-	-	-
ENSG00000101596	SMCHD1	0.383838587	7.01E-08	0.254997724	0.002975	0.295370444	0.000126	-0.122389929	0.02218	-0.074134282	0.157587	0.042947278	0.676845	C5	-	Y	-	-	-	-	-
ENSG00000101605	MYOM1	-0.563450531	0.275888	0.637294375	0.266298	0.270992979	0.642866	1.207195645	0.018186	0.849142738	0.093317	-0.363151634	NA	C2	-	-	-	-	Y	-	-
ENSG00000101624	CEP76	0.357483912	0.0016	0.216256522	0.145468	0.174650648	0.232705	-0.134453338	0.276607	-0.168141991	0.153262	-0.039059793	0.887146	C1	-	Y	-	-	-	-	-
ENSG00000101745	ANKRD12	0.541212738	1.59E-07	0.289548493	0.02608	0.300636947	0.01307	-0.245373778	0.002148	-0.225924585	0.003396	0.013987361	0.951422	C1	-	Y	-	Y	-	-	-
ENSG00000101751	POLI	0.365019487	9.24E-07	0.208421736	0.010537	0.301611556	9.92E-05	-0.150272439	0.035734	-0.048803443	0.580209	0.096247632	0.366858	C5	-	Y	-	Y	-	-	-
ENSG00000101782	RIOK3	0.289012878	5.90E-06	-0.041240848	0.704217	0.095055306	0.219746	-0.323777729	1.39E-07	-0.179398785	0.002392	0.139030565	0.099642	C5	-	-	-	-	Y	-	-
ENSG00000101844	ATG4A	0.456494197	0.0016	0.199904861	0.311249	0.260341727	0.177686	-0.250192324	0.123554	-0.181545458	0.321494	0.063635575	0.869474	C5	-	Y	-	-	-	-	-
ENSG00000101868	POLA1	-0.340267482	0.000114	-0.047962462	0.726264	-0.017432429	0.907419	0.298705549	5.36E-05	0.336932922	8.14E-06	0.032930526	0.853851	C4	-	Y	-	-	-	Y	-
ENSG00000101888	NXT2	-0.400294226	0.00043	-0.221308511	0.056367	-0.354409301	0.001861	0.185320796	0.120505	0.060381036	0.69847	-0.130183494	0.45687	C4	-	Y	-	Y	-	-	-
ENSG00000101901	ALG13	-0.483721835	1.85E-10	-0.064564501	0.587506	-0.204011237	0.012049	0.425559234	1.17E-07	0.294309654	0.000187	-0.136567425	0.241546	C4	-	Y	-	-	Y	-	-
ENSG00000101986	ABCD1	-0.516931787	2.73E-07	-0.197683206	0.146661	-0.29647924	0.012779	0.326269365	0.007132	0.235118377	0.061382	-0.09654247	0.679753	C4	-	Y	-	-	Y	-	-
ENSG00000102024	PLS3	-0.63343229	1.82E-13	-0.181221366	0.078796	-0.241483622	0.009465	0.458921255	8.24E-07	0.406524615	1.25E-05	-0.057539262	0.75948	C2	-	Y	-	-	Y	Y	-
ENSG00000102053	ZC3H12B	0.279444487	0.075682	0.319274307	0.035018	0.329414793	0.020748	0.045996426	0.810158	0.064342227	0.720437	0.013364256	0.967582	C1	-	-	Y	Y	-	-	-
ENSG00000102098	SCML2	0.356322341	1.19E-08	0.246709683	0.002183	0.168181824	0.041637	-0.103022295	0.18108	-0.173359785	0.013286	-0.075555916	0.585849	C5	-	Y	-	-	-	-	-
ENSG00000102125	TAZ	-0.218612281	0.04677	0.147037955	0.273375	0.261078741	0.023324	0.372036394	4.37E-05	0.494978128	3.54E-08	0.117496378	0.438365	C4	-	-	-	-	Y	Y	-
ENSG00000102181	CD99L2	-0.628990844	1.58E-11	0.014071774	0.929305	-0.060879173	0.645521	0.649681437	1.26E-13	0.582975369	1.55E-10	-0.071914057	0.652484	C2	-	Y	-	-	Y	Y	-
ENSG00000102189	EEA1	0.910410083	8.45E-26	0.443434045	3.38E-05	0.597532465	6.35E-09	-0.460305244	1.61E-08	-0.298040838	0.000614	0.156915995	0.283801	C1	-	Y	Y	Y	Y	-	-
ENSG00000102230	PCYT1B	-0.355714055	0.000906	-0.200399202	0.158369	-0.128805984	0.3478	0.16219367	0.239723	0.241553791	0.041401	0.074226975	0.761898	C4	-	Y	-	-	-	-	-
ENSG00000102309	PIN4	-0.343676012	4.66E-05	-0.308085379	0.000103	-0.302760016	0.00014	0.042208291	0.708984	0.055839278	0.603013	0.008178551	0.967582	C4	-	Y	Y	Y	-	-	-
ENSG00000102385	DRP2	-0.422785991	0.023866	-0.045353905	0.883864	0.150263458	0.470748	0.38416022	0.071609	0.587646985	0.000839	0.198712804	0.50724	C4	-	Y	-	-	-	Y	-
ENSG00000102387	TAF7L	-0.901913433	0.021969	-0.642269253	0.131236	-0.664340854	0.138738	0.265767261	0.647032	0.252573228	0.691191	-0.018961829	NA	C3	-	Y	-	-	-	-	-
ENSG00000102390	PBDC1	-0.091016454	0.466765	-0.223621082	0.082907	-0.302907076	0.022132	-0.126106195	0.283721	-0.197443471	0.105692	-0.076120357	0.757566	C5	-	-	-	Y	-	-	-
ENSG00000102409	BEX4	-0.447464238	1.03E-16	-0.219513393	0.002914	-0.24786021	3.67E-05	0.2342667	6.01E-05	0.214389827	1.87E-05	-0.025218308	0.857835	C4	-	Y	-	-	-	-	-
ENSG00000102466	FGF14	-0.355890952	0.013987	-0.082995384	0.698859	-0.335357767	0.029953	0.27948145	0.044089	0.035684997	0.856353	-0.248950131	0.170135	C4	-	Y	-	Y	-	-	-
ENSG00000102531	FNDC3A	0.61495561	5.47E-25	0.207143573	0.008927	0.212762852	0.002481	-0.401792291	1.56E-19	-0.387554275	2.40E-23	0.008824716	0.948722	C5	-	Y	-	-	Y	Y	-
ENSG00000102580	DNAJC3	0.35353168	0.000718	0.078982925	0.694574	0.064594993	0.690591	-0.267566535	0.025509	-0.274371418	0.006603	-0.012043807	0.971963	C5	-	Y	-	-	-	-	-
ENSG00000102699	PARP4	-0.352340479	9.08E-06	0.009040206	0.95269	0.101333405	0.331369	0.367624186	5.31E-10	0.467978215	7.77E-15	0.094947456	0.334766	C4	-	Y	-	-	Y	Y	-
ENSG00000102710	SUPT20H	0.313016995	8.98E-07	0.096278525	0.313155	0.19715407	0.006177	-0.21101353	3.50E-05	-0.101422638	0.038741	0.104284337	0.173069	C5	-	Y	-	-	-	-	-
ENSG00000102738	MRPS31	0.495951815	6.01E-07	0.246011502	0.059564	0.219098195	0.086751	-0.243240208	0.011267	-0.262094318	0.005593	-0.024222799	0.927911	C5	Y	Y	-	-	-	-	-
ENSG00000102763	VWA8	-0.332555022	0.000305	0.159869123	0.221594	0.065214494	0.642866	0.498825062	7.81E-09	0.41254096	4.88E-07	-0.091672002	0.592783	C4	-	Y	-	-	Y	Y	-
ENSG00000102780	DGKH	0.925543127	5.82E-14	0.228367044	0.209006	0.371891294	0.005061	-0.69050485	9.91E-09	-0.539001863	1.79E-07	0.14647332	0.489479	C1	-	Y	-	Y	Y	Y	-
ENSG00000102796	DHRS12	-0.669505685	0.001889	0.0514462	0.87807	-0.253075198	0.376764	0.728014128	0.000637	0.431721582	0.104213	-0.301647559	0.389811	C2	-	Y	-	-	Y	-	-
ENSG00000102878	HSF4	-0.317032097	8.13E-05	-0.186001677	0.08859	-0.123303791	0.220344	0.137498448	0.155371	0.208660761	0.01069	0.065418495	0.700194	C4	-	Y	-	-	-	-	-
ENSG00000102879	CORO1A	0.435206746	0.000702	0.091576669	0.657412	0.259212893	0.083901	-0.337237501	0.012545	-0.161260042	0.272171	0.170523996	0.43492	C5	-	Y	-	-	Y	-	-
ENSG00000102893	PHKB	-0.562181105	5.08E-20	-0.251834446	0.000193	-0.111805056	0.16247	0.316664876	2.25E-08	0.465020324	5.42E-16	0.14294301	0.077504	C4	-	Y	-	-	Y	Y	-
ENSG00000102897	LYRM1	-0.483437196	1.62E-08	-0.146373208	0.191701	-0.202396295	0.060937	0.343635948	0.000181	0.295996237	0.002745	-0.053116643	0.797569	C4	-	Y	-	-	Y	-	-
ENSG00000102908	NFAT5	0.623496027	0.001079	0.335942767	0.273993	0.428464994	0.10075	-0.281138369	0.164776	-0.180702818	0.336819	0.095176073	0.852721	C1	-	Y	-	-	-	-	-
ENSG00000102910	LONP2	0.395926332	1.25E-08	0.110809204	0.234921	0.127707181	0.111737	-0.278610021	1.43E-05	-0.253581638	2.96E-05	0.019467458	0.901678	C5	-	Y	-	-	-	-	-
ENSG00000102921	N4BP1	0.383340301	9.75E-09	0.086387684	0.498738	0.259928961	0.000592	-0.289844972	6.94E-05	-0.108905135	0.091832	0.175833359	0.097949	C5	-	Y	-	-	-	-	-
ENSG00000102924	CBLN1	-0.503370042	0.007326	-0.322453775	0.13383	-0.223176554	0.285811	0.188253001	0.41948	0.29527194	0.144242	0.102905199	0.775391	C4	-	Y	-	-	-	-	-
ENSG00000103021	CCDC113	-0.59628632	0.000581	-0.042235678	0.88773	0.053183362	0.839518	0.560808095	0.002604	0.663924968	0.000172	0.098207518	0.788894	C4	-	Y	-	-	Y	Y	-
ENSG00000103024	NME3	-0.483937824	0.002177	-0.219868236	0.316274	-0.126814731	0.570957	0.27085341	0.067705	0.371911776	0.005303	0.09579143	0.736151	C4	-	Y	-	-	-	Y	-
ENSG00000103047	TANGO6	-0.213211543	0.075977	0.106390899	0.509312	0.075169472	0.653882	0.325606103	0.006904	0.303015309	0.015906	-0.028116963	0.930354	C4	-	-	-	-	Y	Y	-
ENSG00000103064	SLC7A6	0.39149389	5.57E-05	0.31667106	0.004285	0.272361094	0.008092	-0.068285087	0.5409	-0.10472299	0.269979	-0.04165377	0.826319	C4	-	Y	Y	-	-	-	-
ENSG00000103066	PLA2G15	0.482878776	0.006356	0.252420153	0.261332	0.230457187	0.270749	-0.224344882	0.251987	-0.238037479	0.20053	-0.019256242	0.96696	C1	-	Y	-	-	-	-	-

ENSG00000103126	AXIN1	0.422866221	4.80E-08	0.278288661	0.02308	0.27426451	0.000865	-0.13826346	0.224363	-0.133922219	0.106388	-0.001221273	0.997161	C5	-	Y	-	-	-	-	-
ENSG00000103145	HCFC1R1	-0.413202211	0.001824	-0.348462172	0.029976	-0.158641082	0.361867	0.071655598	0.721844	0.269407518	0.076227	0.192620873	0.433632	C4	-	Y	Y	-	-	-	-
ENSG00000103148	NPRL3	-0.257977325	0.001545	0.034714328	0.804645	0.142547017	0.15988	0.299238596	0.000386	0.415322542	4.01E-07	0.110618134	0.448376	C4	-	-	-	-	-	Y	-
ENSG00000103152	MPG	-0.302627681	0.047243	-0.032525624	0.897668	-0.104893419	0.569343	0.277366353	0.110945	0.212604311	0.205399	-0.069719473	0.835119	C4	-	Y	-	-	-	-	-
ENSG00000103154	NECAB2	0.089992477	0.707953	0.680449935	0.000127	0.422510566	0.02972	0.597088076	0.000414	0.347477811	0.066537	-0.254675261	0.306077	C4	-	-	Y	Y	Y	-	-
ENSG00000103174	NAGPA	0.045030966	0.821575	-0.018207218	0.95256	-0.282145823	0.088015	-0.056813054	0.798962	-0.312241188	0.041342	-0.261084199	0.28309	C4	-	-	-	-	-	Y	-
ENSG00000103187	COTL1	-0.708963729	1.87E-08	-0.371445672	0.017611	-0.348927437	0.019237	0.345332684	0.038992	0.374694669	0.019435	0.02355744	0.95765	C4	-	Y	Y	Y	Y	Y	-
ENSG00000103196	CRISPLD2	-2.141752098	6.19E-10	-0.807043241	0.036823	-1.188010254	0.000975	1.343858731	0.002927	0.968462068	0.052244	-0.379435964	NA	C3	-	Y	Y	Y	Y	-	-
ENSG00000103199	ZNF500	0.350696651	0.005855	0.113862821	0.605228	0.159334802	0.33849	-0.23000929	0.131331	-0.176350768	0.208408	0.047930874	0.892368	C5	-	Y	-	-	-	-	-
ENSG00000103226	NOMO3	0.479356168	2.44E-08	0.51270011	2.18E-07	0.42565523	4.62E-05	0.040014736	0.712805	-0.038910351	0.735898	-0.084190071	0.62104	C1	-	Y	Y	Y	-	-	-
ENSG00000103253	HAGHL	-0.423647583	3.13E-05	-0.035490225	0.868392	-0.08245214	0.593949	0.394523744	0.000391	0.356260866	0.000282	-0.043714521	0.876139	C4	-	Y	-	-	Y	Y	-
ENSG00000103257	SLC7A5	-0.90263454	1.53E-50	-0.664169597	8.51E-05	-0.293590347	0.005735	0.244649257	0.144532	0.623821386	2.21E-07	0.374198094	0.183357	C3	-	Y	Y	-	-	Y	-
ENSG00000103260	METRN	-0.251702775	0.072162	0.130077651	0.459312	0.08590267	0.640055	0.388228069	7.50E-05	0.353216232	0.000515	-0.040104718	0.853204	C4	-	-	-	-	Y	Y	-
ENSG00000103264	FBXO31	0.361414625	7.37E-05	0.008985735	0.968866	0.022792764	0.88896	-0.346149917	0.001694	-0.323824319	0.000314	0.016388064	0.958938	C5	-	Y	-	-	Y	Y	-
ENSG00000103326	CAPN15	0.350765615	1.92E-06	0.43274674	1.88E-08	0.321521662	3.27E-05	0.088573938	0.266418	-0.014622226	0.8832	-0.108649325	0.311916	C1	-	Y	Y	Y	-	-	-
ENSG00000103335	PIEZO1	-0.268582021	0.179507	0.320515245	0.061579	0.646853992	1.07E-05	0.595331725	0.00051	0.929740815	6.75E-09	0.329248072	0.07753	C4	-	-	-	Y	Y	Y	-
ENSG00000103353	UBFD1	0.318733252	2.95E-10	0.10114635	0.155314	0.159486284	0.010317	-0.211112517	1.77E-06	-0.144480504	0.001385	0.061232309	0.474909	C5	-	Y	-	-	-	-	-
ENSG00000103404	USP31	0.52859949	1.06E-07	0.142954763	0.346011	0.209849453	0.105167	-0.379305021	0.000174	-0.303988777	0.002512	0.069838338	0.758651	C5	-	Y	-	-	Y	Y	-
ENSG00000103423	DNAJA3	0.422771855	1.61E-12	0.095412627	0.300779	0.109351377	0.165834	-0.320930455	2.57E-07	-0.298667321	4.67E-07	0.016746548	0.926517	C5	-	Y	-	-	Y	-	-
ENSG00000103479	RBL2	0.41116311	1.15E-08	0.267566956	0.001791	0.355716359	4.34E-06	-0.137890383	0.010843	-0.04087674	0.525127	0.091424465	0.282485	C1	-	Y	-	Y	-	-	-
ENSG00000103485	QPRT	-0.439688009	5.68E-11	-0.297970258	3.34E-05	-0.184611347	0.023225	0.148202666	0.035939	0.270033434	6.69E-05	0.116405396	0.258544	C4	Y	Y	-	-	-	-	-
ENSG00000103489	XYLT1	0.218238129	0.04853	0.695486838	1.73E-10	0.585595693	7.62E-07	0.483463776	1.12E-06	0.382136732	0.000417	-0.107365933	0.619605	C4	-	-	Y	Y	Y	Y	-
ENSG00000103494	RPGRIP1L	0.425921115	9.29E-05	0.35347535	0.008959	0.554667121	7.54E-08	-0.066349404	0.607398	0.143100172	0.115229	0.204534577	0.118352	C4	-	Y	Y	Y	-	-	-
ENSG00000103496	STX4	0.481343799	2.72E-06	0.111827612	0.48403	0.153314118	0.244804	-0.362824884	0.000232	-0.312982526	0.000909	0.044291968	0.846395	C5	-	Y	-	-	Y	Y	-
ENSG00000103507	BCKDK	-0.22070912	0.00488	-0.068706603	0.561936	0.07207948	0.484561	0.158619541	0.049753	0.307585432	1.06E-05	0.14355748	0.173277	C4	-	-	-	-	-	Y	-
ENSG00000103528	SYT17	-0.319982223	0.045703	0.013187481	0.957456	-0.12313723	0.463657	0.341450694	0.04309	0.21132738	0.235148	-0.133755113	0.58438	C4	-	Y	-	-	Y	-	-
ENSG00000103540	CCP110	0.430270807	1.87E-15	0.200415812	0.011144	0.309101352	7.55E-08	-0.223060861	2.56E-05	-0.106599792	0.013925	0.111136257	0.168713	C4	-	Y	-	Y	-	-	-
ENSG00000103549	RNF40	0.395892488	1.35E-16	0.148111576	0.014444	0.122102415	0.124968	-0.241298283	3.00E-07	-0.259371094	5.25E-06	-0.023145483	0.88523	C5	-	Y	-	-	-	-	-
ENSG00000103647	CORO2B	0.821384301	0.072213	0.928331503	0.039507	0.362736547	0.495929	0.113521355	0.84745	-0.443589088	0.365147	-0.56248553	NA	C1	-	-	Y	-	-	-	-
ENSG00000103653	CSK	0.346833264	2.20E-09	0.108599142	0.197226	0.084407669	0.343372	-0.231719416	2.89E-06	-0.247686983	1.01E-06	-0.021297753	0.879183	C5	-	Y	-	-	-	-	-
ENSG00000103657	HERC1	0.679066504	3.85E-14	0.474663816	1.94E-06	0.498594203	4.29E-08	-0.198081611	0.015184	-0.166158977	0.02678	0.026756814	0.890117	C1	-	Y	Y	Y	-	-	-
ENSG00000103811	CTSH	-0.80309167	1.84E-05	-0.419060915	0.10001	-0.350609889	0.084781	0.391173801	0.12787	0.467096888	0.026425	0.070954432	0.896771	C4	-	Y	-	-	-	Y	-
ENSG00000103852	TTC23	-0.89166199	1.41E-07	-0.273462705	0.201505	0.006536346	0.982114	0.624687126	0.001654	0.912841418	9.71E-07	0.282861528	0.326733	C3	-	Y	-	-	Y	Y	-
ENSG00000103876	FAH	-0.649400481	8.38E-06	-0.231024643	0.288769	-0.271839557	0.128661	0.425543611	0.019645	0.392536743	0.020776	-0.038285841	0.934798	C4	-	Y	-	-	Y	Y	-
ENSG00000103888	CEMIP	0.633760983	0.051883	0.289953691	0.527031	0.766129985	0.012484	-0.33715564	0.350714	0.146497187	0.687403	0.480705291	0.25635	C1	-	-	-	Y	-	-	-
ENSG00000103942	HOMER2	0.261543828	0.011798	0.412378872	4.58E-05	0.312359119	0.001543	0.157303731	0.131604	0.065518027	0.570264	-0.096752384	0.523805	C1	-	-	Y	Y	-	-	-
ENSG00000103994	ZNF106	0.604957699	7.05E-14	0.402186901	6.78E-05	0.340404997	0.000107	-0.196372793	0.003656	-0.250301961	1.11E-06	-0.059164213	0.641935	C1	-	Y	Y	Y	-	-	-
ENSG00000103995	CEP152	0.439682765	2.38E-08	0.050230236	0.727711	0.230019405	0.021949	-0.383063726	5.39E-09	-0.195631891	0.005535	0.182380661	0.086217	C5	-	Y	-	-	Y	-	-
ENSG00000104044	OCA2	-1.100396789	0.001307	-0.941594789	0.013305	-0.843966436	0.024219	0.168381686	0.772697	0.270843924	0.61743	0.097245443	NA	C3	-	Y	Y	Y	-	-	-
ENSG00000104047	DTWD1	0.309899495	0.006441	0.128169339	0.444742	0.328224553	0.019375	-0.17469219	0.084299	0.033199741	0.818968	0.202828156	0.213345	C4	-	Y	-	Y	-	-	-
ENSG00000104064	GABPB1	-0.316042045	1.33E-05	-0.339262084	5.80E-05	-0.396706532	8.74E-07	-0.016915966	0.890765	-0.065879814	0.529799	-0.054431059	0.770023	C4	-	Y	Y	Y	-	-	-
ENSG00000104081	BMF	-0.558026651	4.88E-10	-0.226906727	0.065101	-0.165488461	0.110699	0.337758662	0.001294	0.407606723	2.85E-06	0.064797036	0.748723	C4	-	Y	-	-	Y	Y	-
ENSG00000104093	DMXL2	0.117182437	0.242058	0.284022211	0.022256	0.397212336	6.94E-05	0.172937468	0.049646	0.294644388	7.62E-06	0.116146833	0.472366	C4	-	-	-	Y	-	-	-
ENSG00000104129	DNAJC17	0.293811716	0.026932	-0.013329833	0.954421	0.025842694	0.895591	-0.300837057	0.029019	-0.253290012	0.058584	0.041976714	0.89024	C5	-	-	-	-	Y	-	-
ENSG00000104131	EIF3J	0.457218524	1.17E-18	0.10467433	0.116911	0.071928192	0.279846	-0.346056922	9.31E-12	-0.370559656	7.09E-15	-0.029848383	0.785648	C5	Y	Y	-	-	Y	Y	-
ENSG00000104133	SPG11	-0.24989143	0.000165	0.154592465	0.069945	0.187039863	0.025464	0.410635284	5.02E-12	0.451669018	2.39E-13	0.035310401	0.828373	C4	-	-	-	-	Y	Y	-
ENSG00000104218	CSPP1	0.347303704	0.0001	0.071699119	0.635507	0.186900525	0.083918	-0.268816592	0.002852	-0.145696234	0.118645	0.117653766	0.438524	C5	-	Y	-	-	-	-	-
ENSG00000104219	ZDHHC2	-0.254129731	0.179484	0.089129519	0.778256	0.198502722	0.356443	0.349228897	0.076961	0.467157993	0.003952	0.112755808	0.761549	C4	-	-	-	-	-	Y	-
ENSG00000104228	TRIM35	0.348672048	0.000173	0.042519107	0.805759	0.169865046	0.150099	-0.299571337	0.001038	-0.164426529	0.087452	0.129860388	0.412815	C5	-	Y	-	-	-	-	-
ENSG00000104312	RIPK2	0.613808901	2.99E-07	0.512731876	0.000207	0.478444619	0.000713	-0.094591125	0.541421	-0.120862294	0.424665	-0.031318249	0.928882	C1	-	Y	Y	Y	-	-	-
ENSG00000104313	EYA1	-0.320673316	0.003509	-0.295641646	0.01434	-0.311787857	0.00972	0.031100418	0.845148	0.023247261	0.891881	-0.013217705	0.967212	C4	-	Y	-	Y	-	-	-
ENSG00000104320	NBN	-0.450640729	3.05E-08	-0.285533839	0.001538	-0.229342141	0.01224	0.171505772	0.046903	0.235803875	0.004496	0.058794744	0.710389	C4	-	Y	-	-	-	-	-
ENSG00000104325	DECR1	-0.4034787	0.000332	-0.213707798	0.130032	-0.256531397	0.063975	0.196464344	0.110532	0.162031046	0.230852	-0.039723663	0.891721	C4	-	Y	-	-	-	-	-
ENSG00000104331	BPNT2	0.286107692	2.72E-07	0.027029136	0.82331	-0.0318325	0.723312	-0.252453744	2.88E-06	-0.303319047	1.02E-13	-0.056173638	0.606784	C5	-	-	-	-	-	Y	-
ENSG00000104332	SFRP1	-0.787842127	1.10E-26	-0.355246602	5.98E-06	-0.276299278	0.003826	0.439308175	6.39E-08	0.526302986	3.83E-09	0.081112493	0.644363	C4	Y	Y	Y	-	Y	Y	-
ENSG00000104343	UBE2W	0.188375191	0.047998	-0.210262507	0.072495	-0.202012564	0.083887	-0.39259599	1.29E-05	-0.375731197	4.00E-05	0.010852569	0.970509	C5	-	-	-	-	Y	Y	-
ENSG00000104356	POP1	0.480306676	2.32E-06	0.108943491	0.499263	0.151759977	0.268059	-0.36480912	7.07E-07	-0.31414866	1.43E-05	0.045296424	0.794576	C5	-	Y	-	-	Y	Y	-
ENSG00000104365	IKBKB	0.468861874	3.33E-06	0.022921831	0.916318	0.187432206	0.244193	-0.439656767	2.21E-05	-0.267330466	0.02218	0.167361942	0.453117	C5	-	Y	-	-	Y	-	-
ENSG00000104368	PLAT	1.131219087	6.78E-14	0.33512343	0.173443	-0.119140349	0.73494	-0.791473259	1.41E-07	-1.233647629	1.25E-12	-0.452279518	0.160902	C5	-	Y	-	-	Y	Y	-
ENSG00000104412	EMC2	-0.347133184	0.002498	-0.271132822	0.035409	-0.138575973	0.267028	0.082617867	0.592104	0.22298536	0.054102	0.135762528	0.445857	C4	-	Y	-	-	-	-	-
ENSG00000104419	NDRG1	-0.97042232	1.82E-17	-0.331800247	0.017951	-0.286829934	0.028214	0.645768634	5.84E-07	0.698670855	9.66E-09	0.047879498	0.876139	C4	-	Y	Y	-	Y	Y	-
ENSG00000104432	IL7	0.354111314	0.000658	0.282627494	0.037357	0.090201004	0.542042	-0.065336173	0.655211	-0.249381646	0.018549	-0.189361787	0.269591	C5	-	Y	-	-	-	-	-
ENSG00000104447	TRPS1	-0.941097645	0.000772	-0.202355773	0.677124	-0.254478879	0.473151	0.746366939	0.030983	0.701213403	0.017461	-0.049666879	0.952948	C2	-	Y	-	-	Y	Y	-
ENSG00000104497	SNX16	0.836402384	3.86E-09	0.149848707	0.45153	0.393970177	0.006914	-0.680620646	1.23E-06	-0.427879616	0.00204	0.246788823	0.200991	C5	-	Y	-	Y	Y	Y	-
ENSG00000104517	UBR5	0.339362692	1.26E-06	0.156296134	0.111904	0.155371215	0.040706	-0.176500368	0.021496	-0.169338064	0.005916	0.001932341	0.992942	C5	-	Y	-	-	-	-	-
ENSG00000104524	PYCR3	-0.920778197	1.13E-05	-0.224829819	0.336536	-0.075149361	0.771675	0.703723214	0.000946	0.8606967	1.20E-05	0.153328701	0.609835	C3	-	Y	-	-	Y	Y	-
ENSG00000104613	INTS10	0.347695981	1.51E-05	0.325763949	0.000585	0.209532422	0.057953	-0.015519441	0.889581	-0.123467963	0.184266	-0.113347503	0.461686	C1	-	Y	Y	-	-	-	-

ENSG00000104626	ERI1	0.327786169	1.11E-05	0.086811883	0.476179	0.093726705	0.367558	-0.234859682	0.002429	-0.219490998	0.001938	0.009996147	0.964368	C5	-	Y	-	-	-	-	-
ENSG00000104691	UBXN8	-0.079639325	0.553502	-0.178534799	0.20158	-0.486916673	3.48E-05	-0.092376201	0.509928	-0.392521971	0.000728	-0.305501765	0.049291	C5	-	-	-	Y	-	Y	Y
ENSG00000104722	NEFM	-0.865010657	1.29E-77	-0.588534343	1.99E-19	-0.409961084	1.03E-14	0.283205015	8.99E-07	0.469535603	1.45E-28	0.181363533	0.028245	C4	-	Y	Y	Y	-	Y	-
ENSG00000104728	ARHGEF10	0.911659758	0.002542	0.630319852	0.124856	0.628751695	0.121732	-0.273493006	0.406782	-0.268832256	0.428619	0.000684335	NA	C1	-	Y	-	-	-	-	-
ENSG00000104756	KCTD9	0.31664969	0.000602	0.00635165	0.974546	0.175708443	0.130489	-0.303897031	0.002492	-0.126129314	0.233816	0.172161949	0.284837	C5	-	Y	-	-	Y	-	-
ENSG00000104765	BNIP3L	-0.218164078	2.27E-06	0.12661568	0.064474	-0.052279131	0.529351	0.351241889	1.15E-11	0.180699682	0.001136	-0.176281171	0.035206	C4	Y	-	-	-	Y	-	-
ENSG00000104812	GYS1	-0.284070077	0.000249	0.22604	0.011951	0.261419157	0.001945	0.516623193	1.46E-11	0.560209342	6.54E-14	0.03823954	0.835119	C4	-	-	-	-	Y	Y	-
ENSG00000104825	NFKBIB	0.416480845	0.000372	-0.116260797	0.554731	-0.119502386	0.527886	-0.525721087	1.23E-07	-0.521356309	4.69E-07	-0.000679783	0.997918	C5	-	Y	-	-	Y	Y	-
ENSG00000104833	TUBB4A	-0.386140759	7.75E-07	-0.317507798	0.000377	-0.289263706	0.001733	0.074998351	0.451041	0.111462223	0.242113	0.031119104	0.883781	C4	-	Y	Y	-	-	-	-
ENSG00000104835	SARS2	-0.204638802	0.325009	-0.214094464	0.289322	-0.570919019	0.001198	-0.00401873	0.987917	-0.351023062	0.098286	-0.353142319	0.15234	C5	-	-	-	Y	-	-	-
ENSG00000104856	RELB	1.502738262	4.79E-07	0.645630831	0.136991	0.58237211	0.209293	-0.850042745	0.002445	-0.905972071	0.002528	-0.060532918	NA	C5	-	Y	-	-	Y	Y	-
ENSG00000104879	CKM	4.11332102	0.020968	2.401929778	NA	2.430435414	NA	-1.674995547	NA	-1.665199508	NA	0.018486921	NA	C5	-	Y	-	-	-	-	-
ENSG00000104883	PEX11G	-1.162567712	0.010684	-0.298270448	0.619115	-0.724951626	0.168117	0.869169776	0.077634	0.451053334	0.466428	-0.422893344	NA	C4	-	Y	-	-	-	-	-
ENSG00000104885	DOT1L	0.474253618	5.82E-19	0.268232836	1.76E-05	0.240252482	0.000364	-0.199733106	5.68E-05	-0.219675886	2.79E-05	-0.025213419	0.858052	C5	-	Y	-	-	-	-	-
ENSG00000104888	SLC17A7	-0.195990443	0.843577	0.277948728	NA	1.470861056	0.008906	0.482770394	0.6286	1.678519936	0.00454	1.199398726	NA	C3	-	-	-	Y	-	Y	-
ENSG00000104901	DKKL1	-1.500507306	0.007337	-1.194484775	0.071955	-1.151027327	0.045318	0.317975636	0.758046	0.363016512	0.696085	0.042342621	NA	C3	-	Y	-	Y	-	-	-
ENSG00000104936	DMPK	0.257458696	3.49E-05	-0.014162468	0.914982	-0.067724773	0.525244	-0.26506858	5.00E-05	-0.310293478	4.03E-06	-0.050606204	0.764023	C5	-	-	-	-	-	Y	-
ENSG00000104953	TLE6	-1.348590809	1.53E-12	-0.39559696	0.042915	-0.352793433	0.096321	0.959630716	6.95E-06	1.00989027	8.11E-06	0.045516835	0.930809	C3	-	Y	Y	-	Y	Y	-
ENSG00000104983	CCDC61	0.253586393	0.225157	-0.179835111	0.532259	0.153758697	0.578004	-0.426931437	0.034853	-0.084676122	0.745306	0.336501155	0.266237	C5	-	-	-	-	Y	-	-
ENSG00000104998	IL27RA	-0.034680032	0.880349	-0.416181826	0.035167	-0.243936069	0.224189	-0.375140248	0.026779	-0.19464572	0.281644	0.174916465	0.559415	C4	-	-	Y	-	Y	-	-
ENSG00000105011	ASF1B	-0.36423139	1.93E-05	-0.292828863	0.015258	-0.267791591	0.00708	0.077676434	0.565298	0.111190926	0.306446	0.028064735	0.925998	C4	-	Y	-	-	-	-	-
ENSG00000105048	TNNT1	-0.413417173	0.000156	-0.381641551	0.005876	-0.387959191	0.000963	0.037675711	0.807233	0.040728582	0.755148	-0.00239795	0.994356	C4	-	Y	Y	Y	-	-	-
ENSG00000105058	FAM32A	-0.189599454	0.002272	-0.153067347	0.050713	-0.304872003	3.55E-06	0.043428323	0.519274	-0.100394613	0.072882	-0.149174268	0.04285	C4	-	-	-	Y	-	-	-
ENSG00000105063	PPP6R1	0.537962366	1.25E-27	0.205966958	0.004184	0.110995645	0.114324	-0.325516597	1.80E-09	-0.412229538	4.50E-18	-0.092080945	0.370627	C5	-	Y	-	-	Y	Y	-
ENSG00000105072	C19orf44	0.74428128	7.41E-11	0.332393863	0.01991	0.439557423	0.001296	-0.40548092	0.000238	-0.289669297	0.014366	0.110305941	0.613179	C5	-	Y	Y	Y	Y	-	-
ENSG00000105085	MED26	-0.176770183	0.314486	-0.615570588	0.000656	-0.306549983	0.137901	-0.432266059	0.018888	-0.115332272	0.622035	0.312054589	0.310171	C5	-	-	Y	-	Y	-	-
ENSG00000105127	AKAP8	0.052953365	0.560793	-0.162619886	0.064061	-0.286980214	0.000613	-0.209065098	0.003197	-0.325202252	8.27E-06	-0.121453193	0.309024	C5	-	-	-	-	-	Y	-
ENSG00000105131	EPHX3	-1.568000624	0.246885	0.737404374	NA	-0.443695107	NA	2.315031879	0.027295	1.139281644	0.483929	-1.17901759	NA	C2	-	-	-	-	Y	-	-
ENSG00000105171	POP4	-0.122190186	0.238829	-0.145523119	0.273375	-0.330325347	0.001345	-0.016723712	0.914149	-0.19345055	0.072332	-0.182018745	0.27312	C4	-	-	-	Y	-	-	-
ENSG00000105173	CCNE1	-0.739119255	1.97E-12	-0.653009523	1.99E-08	-0.602223937	1.41E-10	0.091156292	0.608993	0.151627248	0.279071	0.053932396	0.852721	C4	-	Y	Y	Y	-	-	-
ENSG00000105186	ANKRD27	0.678175234	3.52E-14	0.37129814	0.000159	0.554543326	8.24E-10	-0.300522339	0.000292	-0.109134027	0.247495	0.185904077	0.091151	C5	Y	Y	Y	Y	Y	-	-
ENSG00000105219	CCNP	2.193592367	5.45E-05	1.08812667	NA	0.667489138	NA	-1.100462351	0.026659	-1.512995314	0.002897	-0.418242289	NA	C5	-	Y	-	-	Y	Y	-
ENSG00000105245	NUMBL	0.461152584	3.09E-07	0.171872326	0.249886	0.277337337	0.017969	-0.282980668	0.007119	-0.169208874	0.094591	0.108422173	0.604908	C5	-	Y	-	-	-	-	-
ENSG00000105248	YJU2	-0.525417631	4.69E-05	-0.241699914	0.131438	-0.432015472	0.001312	0.290095343	0.024541	0.108613798	0.464863	-0.186454039	0.301234	C4	-	Y	-	Y	-	-	-
ENSG00000105251	SHD	-0.104738259	0.056469	0.194642661	0.003162	0.069803528	0.294215	0.306021884	4.57E-12	0.189402328	1.62E-07	-0.12197877	0.053244	C4	-	-	-	-	Y	-	-
ENSG00000105278	ZFR2	-0.848998141	1.53E-06	-0.178614734	0.493938	-0.414818515	0.031038	0.677294059	0.001472	0.449201874	0.039477	-0.233653865	0.501149	C3	-	Y	-	Y	Y	Y	-
ENSG00000105287	PRKD2	0.550245698	1.29E-13	0.351522898	9.81E-05	0.428700967	3.14E-08	-0.192356619	0.015738	-0.106533962	0.176765	0.080353141	0.554495	C5	-	Y	Y	Y	-	-	-
ENSG00000105327	BBC3	-0.437158184	0.002212	0.003554593	0.988664	-0.023424147	0.931764	0.446815714	0.002373	0.428421182	0.017496	-0.023425869	0.958491	C4	-	Y	-	-	Y	Y	-
ENSG00000105339	DENND3	-0.416421937	0.002806	0.033135471	0.88773	0.214760361	0.153125	0.455750979	0.001337	0.645919078	7.07E-07	0.18425097	0.363559	C4	-	Y	-	-	Y	Y	-
ENSG00000105355	PLIN3	-0.435038502	5.53E-05	-0.22063949	0.097878	-0.093306599	0.520778	0.221192414	0.084919	0.356390955	0.001642	0.130229399	0.503233	C4	-	Y	-	-	-	Y	-
ENSG00000105379	ETFB	-0.572224033	1.06E-05	-0.13705051	0.479702	-0.194752451	0.234257	0.44103917	0.000238	0.392866157	0.000867	-0.053295976	0.843922	C4	-	Y	-	-	Y	Y	-
ENSG00000105447	GRWD1	0.338869258	4.01E-06	0.174908867	0.061652	0.076159404	0.455412	-0.157666922	0.053424	-0.248144814	0.00072	-0.095982896	0.474909	C5	-	Y	-	-	-	-	-
ENSG00000105464	GRIN2D	-0.58303308	0.04534	-0.238827347	0.563271	-0.557459181	0.09222	0.351667953	0.309967	0.040730035	0.928271	-0.315915057	NA	C2	-	Y	-	-	-	-	-
ENSG00000105479	CCDC114	-1.382021882	0.046155	0.420862097	0.583248	0.138015065	0.875441	1.809893846	0.001782	1.535199352	0.013937	-0.280910901	NA	C4	-	Y	-	-	Y	Y	-
ENSG00000105483	CARD8	0.474970192	5.69E-10	0.253351054	0.009483	0.424235362	2.78E-07	-0.215145633	0.013244	-0.036015014	0.739619	0.173700755	0.158078	C1	-	Y	-	Y	-	-	-
ENSG00000105497	ZNF175	0.237761199	0.036697	-0.163981732	0.334016	0.060634322	0.719005	-0.395096472	0.00087	-0.162302797	0.167292	0.227456903	0.214179	C5	-	-	-	-	Y	-	-
ENSG00000105516	DBP	-0.503730668	0.002316	-0.147601136	0.505594	0.056280302	0.817474	0.363111768	0.047184	0.574891433	0.000746	0.206712466	0.433095	C4	-	Y	-	-	Y	Y	-
ENSG00000105538	RASIP1	-1.26740236	0.000928	-0.038818245	0.952296	-0.281681468	0.547222	1.238607636	0.004676	0.999777053	0.028394	-0.242716408	NA	C3	-	Y	-	-	Y	Y	-
ENSG00000105552	BCAT2	-0.477470298	1.01E-06	-0.230440591	0.040637	-0.052265924	0.704881	0.253644243	0.01847	0.440082268	4.79E-06	0.181191746	0.179023	C4	-	Y	-	-	-	Y	-
ENSG00000105559	PLEKHA4	0.41757054	0.008644	0.100214019	0.712563	-0.17181146	0.445588	-0.310888959	0.049403	-0.574839743	6.88E-05	-0.269214736	0.270443	C5	-	Y	-	-	Y	Y	-
ENSG00000105612	DNASE2	-0.401727729	0.000648	-0.195097675	0.213888	-0.15877884	0.329064	0.213018015	0.12722	0.257820624	0.063955	0.039298751	0.906925	C4	-	Y	-	-	-	-	-
ENSG00000105613	MAST1	0.569417106	7.38E-20	0.262594267	0.00042	0.331307555	9.70E-06	-0.300162748	2.10E-07	-0.223088827	0.000383	0.071662423	0.544887	C5	-	Y	-	Y	Y	-	-
ENSG00000105619	TFPT	-0.299337454	0.014908	-0.352313734	0.008225	-0.308307814	0.015064	-0.046215152	0.78575	0.005867569	0.971336	0.047280153	0.862235	C4	-	-	Y	Y	-	-	-
ENSG00000105639	JAK3	-0.949124582	0.00024	-0.307153267	0.316274	-0.329350538	0.278116	0.649863008	0.028126	0.634373558	0.040364	-0.020141055	0.978047	C4	-	Y	-	-	Y	Y	-
ENSG00000105641	SLC5A5	-2.08492584	1.94E-06	-0.419034216	0.424188	-0.34285927	0.468701	1.678657935	0.001558	1.756602169	0.00043	0.076865372	NA	C3	-	Y	-	-	Y	Y	-
ENSG00000105649	RAB3A	0.034430586	0.838323	0.267943886	0.055697	0.368194732	0.00799	0.240081847	0.014825	0.349233305	0.000766	0.103626673	0.581355	C4	-	-	-	Y	-	Y	-
ENSG00000105664	COMP	1.344040813	0.033592	1.676962871	0.010291	1.784082747	0.006723	0.338840391	0.580882	0.456384548	0.454684	0.111065526	NA	C1	-	Y	Y	Y	-	-	-
ENSG00000105699	LSR	-0.331364673	0.596764	0.638524574	0.153935	0.13204499	0.83896	0.979148739	0.044781	0.477145112	0.436628	-0.50348033	NA	C2	-	-	-	-	Y	-	-
ENSG00000105705	SUGP1	0.043839335	0.641013	-0.263137809	0.002287	-0.214011203	0.008497	-0.300386621	7.10E-05	-0.243034442	0.000779	0.052008554	0.745813	C5	-	-	-	-	Y	-	-
ENSG00000105722	ERF	0.380380297	5.87E-08	0.275223441	0.000758	0.30035986	9.22E-05	-0.098776419	0.104064	-0.065074564	0.297338	0.028314252	0.816997	C5	Y	Y	-	Y	-	-	-
ENSG00000105732	ZNF574	0.073745439	0.518369	-0.391377075	0.000236	-0.137592397	0.3284	-0.458770943	2.11E-06	-0.19683791	0.087625	0.256857564	0.108102	C5	-	-	Y	-	Y	-	-
ENSG00000105737	GRIK5	-0.259300007	6.09E-05	0.003097845	0.980292	0.038328252	0.690252	0.268783763	1.48E-05	0.312410229	2.64E-07	0.038295161	0.766819	C4	-	-	-	-	-	Y	-
ENSG00000105738	SIPA1L3	0.493235227	1.95E-09	0.172590146	0.124856	0.098118474	0.41988	-0.314807741	1.24E-06	-0.380483944	4.31E-09	-0.071244743	0.59249	C5	-	Y	-	-	Y	Y	-
ENSG00000105778	AVL9	0.308340462	9.75E-07	0.212586924	0.006521	0.205990315	0.004189	-0.089596855	0.117621	-0.087667472	0.094228	-0.003551771	0.98207	C1	-	Y	-	-	-	-	-
ENSG00000105784	RUNDC3B	0.070152907	0.64477	-0.377588387	0.009688	-0.234257674	0.167051	-0.441403888	0.000639	-0.289530004	0.049684	0.146744183	0.599411	C4	-	-	Y	-	Y	-	-
ENSG00000105792	CFAP69	0.525093373	0.002432	0.04140794	0.903389	0.067078268	0.834072	-0.476906933	0.005297	-0.443420729	0.015105	0.028551347	0.958373	C5	-	Y	-	-	Y	Y	-
ENSG00000105793	GTPBP10	0.21178115	0.015818	-0.099634091	0.449137	0.014204943	0.931261	-0.305387142	0.000167	-0.182850129	0.037581	0.116701328	0.463858	C5	-	-	-	-	Y	-	-

ENSG00000105808	RASA4	0.070462839	0.735922	0.631905446	0.000222	0.547630543	0.001422	0.567722719	5.94E-06	0.492483943	0.000126	-0.080628897	0.780412	C4	-	-	Y	Y	Y	Y	-
ENSG00000105810	CDK6	-0.041629813	0.723627	-0.314565629	0.001382	-0.206314465	0.222245	-0.266492162	0.000362	-0.150040775	0.317656	0.110952752	0.633677	C4	-	-	Y	-	-	-	-
ENSG00000105821	DNAJC2	0.510468022	1.11E-20	0.100709426	0.18948	0.026632445	0.78875	-0.403453778	1.77E-17	-0.469195478	9.50E-22	-0.071192253	0.458985	C5	Y	Y	-	-	Y	Y	-
ENSG00000105825	TFPI2	1.030933224	8.37E-66	0.655542283	3.14E-20	0.405440307	2.11E-08	-0.368978017	1.16E-08	-0.610551776	3.05E-25	-0.247221289	0.00601	C1	-	Y	Y	Y	Y	Y	-
ENSG00000105829	BET1	0.453898702	2.11E-08	0.013496035	0.926116	0.088177378	0.390738	-0.434048854	1.03E-07	-0.350937441	1.21E-05	0.077558186	0.596447	C5	-	Y	-	-	Y	Y	-
ENSG00000105835	NAMPT	0.462825119	6.59E-14	0.26071182	0.00042	0.303911935	7.46E-05	-0.195436557	0.000202	-0.144254523	0.016176	0.046012008	0.711065	C1	-	Y	-	Y	-	-	-
ENSG00000105854	PON2	-0.36929974	1.15E-07	-0.149900414	0.097174	-0.21573928	0.00698	0.225832618	0.003845	0.16824353	0.035893	-0.063038783	0.674383	C4	-	Y	-	-	-	-	-
ENSG00000105865	DUS4L	-0.241969101	0.030352	0.197490866	0.139445	-0.058756811	0.712884	0.445773272	2.72E-05	0.197898528	0.096228	-0.25332932	0.082976	C4	-	-	-	-	Y	-	-
ENSG00000105877	DNAH11	1.513755398	0.046588	-0.011840866	NA	0.528685996	NA	-1.512187074	0.074054	-0.970196162	0.223219	0.543251541	NA	C5	-	Y	-	-	-	-	-
ENSG00000105879	CBLL1	0.202351536	0.001728	-0.260317639	0.000239	-0.104319026	0.219746	-0.456308802	4.10E-18	-0.292254251	1.01E-07	0.158848348	0.051017	C5	-	-	-	-	Y	-	-
ENSG00000105880	DLX5	-1.746368432	1.24E-15	-0.812529157	0.001408	-0.976922373	5.77E-06	0.944478867	0.002096	0.782935573	0.007812	-0.165825553	0.783424	C3	-	Y	Y	Y	Y	Y	-
ENSG00000105894	PTN	-0.41418865	1.28E-10	-0.051748866	0.69363	-0.119912987	0.271928	0.368927935	2.67E-07	0.309521087	3.81E-05	-0.065395606	0.718671	C4	-	Y	-	-	Y	Y	-
ENSG00000105926	MPP6	0.324950476	4.12E-06	0.220394157	0.031596	0.154964791	0.072683	-0.097760769	0.253751	-0.155468261	0.01552	-0.063080215	0.67888	C5	-	Y	-	-	-	-	-
ENSG00000105928	GSDME	-0.338577633	1.20E-08	-0.126592997	0.124541	-0.073032909	0.361389	0.218369567	0.000733	0.280193474	6.95E-07	0.056613057	0.635548	C4	-	Y	-	-	-	-	-
ENSG00000105948	TTC26	0.374673447	0.005249	0.015732792	0.952806	0.144641223	0.417954	-0.352583178	0.000279	-0.216202578	0.029433	0.131003004	0.440061	C5	-	Y	-	-	Y	-	-
ENSG00000105953	OGDH	-0.134673817	0.036047	0.147814472	0.035821	0.204773349	0.000603	0.288999792	1.69E-06	0.35419771	1.46E-10	0.059924668	0.567942	C4	-	-	-	-	-	Y	-
ENSG00000105976	MET	-1.070511959	0.000799	-0.260176355	0.495723	-0.22539163	0.520143	0.818714888	0.02367	0.85941615	0.012473	0.037661051	NA	C4	-	Y	-	-	Y	Y	-
ENSG00000105982	RNF32	-0.48901708	0.017088	0.061407813	0.841956	-0.006487123	0.985502	0.556390632	0.004335	0.497413997	0.021145	-0.064663525	0.888439	C2	-	Y	-	-	Y	Y	-
ENSG00000106003	LFNG	-0.554127054	3.54E-05	-0.282119658	0.061593	-0.334799795	0.017437	0.278369502	0.053821	0.234179072	0.114227	-0.050044124	0.864786	C4	-	Y	-	Y	-	-	-
ENSG00000106012	IQCE	0.300870747	3.84E-06	0.223067132	0.010051	0.002535307	0.98584	-0.071109074	0.365546	-0.284003276	2.00E-05	-0.217720735	0.029795	C5	-	Y	-	-	-	-	-
ENSG00000106013	ANKRD7	-1.003131098	0.011054	-0.393398927	0.389414	-0.142357092	0.769259	0.619482271	0.187451	0.87445472	0.034547	0.253837475	NA	C2	-	Y	-	-	-	Y	-
ENSG00000106025	TSPAN12	0.426940345	0.015116	-0.107908259	0.738348	0.194553846	0.418476	-0.528283693	0.007166	-0.217487535	0.284724	0.30526047	0.332752	C5	-	Y	-	-	Y	-	-
ENSG00000106034	CPED1	0.573801265	0.009847	0.454853864	0.069877	0.361345156	0.173147	-0.112495853	0.659657	-0.197582003	0.41357	-0.090676489	0.840063	C1	-	Y	-	-	-	-	-
ENSG00000106077	ABHD11	-0.859423365	1.21E-08	-0.146076214	0.521618	-0.274079872	0.108237	0.720282997	2.44E-05	0.600346223	0.000234	-0.124870614	0.694838	C2	-	Y	-	-	Y	Y	-
ENSG00000106080	FKBP14	0.61627006	7.72E-12	0.386096406	0.00227	0.512576267	8.58E-07	-0.224648809	0.015079	-0.088763392	0.324533	0.129851448	0.435383	C1	-	Y	Y	Y	-	-	-
ENSG00000106086	PLEKHA8	0.481702853	5.97E-10	0.135910301	0.219461	0.32225947	5.95E-05	-0.339413617	6.95E-08	-0.145248328	0.011175	0.188973655	0.016082	C5	-	Y	-	Y	Y	-	-
ENSG00000106105	GARS1	-0.669742837	1.02E-52	-0.539881698	4.38E-13	-0.306001939	4.19E-06	0.136519757	0.05009	0.378347771	6.06E-14	0.236998134	0.017167	C4	-	Y	Y	Y	-	Y	-
ENSG00000106133	NSUN5P2	0.136818617	0.498745	0.505545649	0.003932	0.224480134	0.263042	0.375220427	0.010392	0.102866658	0.577396	-0.277664029	0.151315	C1	-	-	Y	-	Y	-	-
ENSG00000106211	HSPB1	-0.368265828	5.06E-05	-0.260206551	0.009531	-0.07628974	0.528076	0.114827777	0.177158	0.306964925	1.24E-05	0.186697767	0.034211	C4	-	Y	-	-	-	Y	-
ENSG00000106236	NPTX2	-0.68184909	1.28E-29	-0.271268004	0.000168	-0.108133207	0.187095	0.41700696	4.39E-12	0.588456936	4.13E-25	0.166147834	0.047284	C4	-	Y	-	-	Y	Y	-
ENSG00000106266	SNX8	-0.397293717	5.83E-06	-0.328303669	0.000936	-0.270309806	0.010753	0.075532889	0.558126	0.141695058	0.233705	0.060699503	0.793199	C4	-	Y	Y	-	-	-	-
ENSG00000106268	NUDT1	-0.42964374	1.90E-05	-0.383532935	0.000483	-0.345212807	0.002796	0.053328543	0.644644	0.099166835	0.370058	0.040683173	0.849442	C4	-	Y	Y	Y	-	-	-
ENSG00000106299	WASL	0.514888643	2.70E-21	0.170439599	0.018364	0.176412202	0.005042	-0.338269614	1.37E-12	-0.323918471	8.34E-15	0.009078312	0.948042	C5	-	Y	-	-	Y	Y	-
ENSG00000106327	TFR2	-0.376473088	0.001083	-0.10312706	0.528215	-0.207924532	0.174373	0.279498607	0.032386	0.183231801	0.243362	-0.101644505	0.70249	C4	-	Y	-	-	-	-	-
ENSG00000106336	FBXO24	-0.907920225	0.029032	-0.160167763	0.776272	-0.700887062	0.098268	0.755082777	0.102006	0.221494608	0.720254	-0.538028649	NA	C3	-	Y	-	-	-	-	-
ENSG00000106346	USP42	0.230778386	0.000204	-0.165402246	0.040513	-0.01726666	0.876573	-0.389535112	6.83E-12	-0.233521813	3.67E-05	0.15080978	0.087538	C5	-	-	-	-	Y	-	-
ENSG00000106351	AGFG2	0.510451628	8.40E-05	0.068795812	0.783416	0.097368774	0.673281	-0.434687902	0.001488	-0.398229793	0.005916	0.031777912	0.942288	C5	Y	Y	-	-	Y	Y	-
ENSG00000106366	SERPINE1	4.109258068	6.77E-131	3.71919495	1.96E-94	3.607498142	3.12E-74	-0.382935681	0.000151	-0.48668747	4.06E-05	-0.108620245	0.68056	C1	-	Y	Y	Y	Y	Y	-
ENSG00000106367	AP1S1	-0.314052736	2.17E-06	-0.097015434	0.316006	-0.083585002	0.381877	0.223776585	6.98E-07	0.245434709	5.23E-08	0.016295229	0.899704	C4	-	Y	-	-	-	-	-
ENSG00000106392	C1GALT1	-0.314513026	1.98E-06	-0.232162854	0.001686	-0.098443623	0.251752	0.088696734	0.2405	0.230588364	0.000414	0.13648402	0.148991	C4	-	Y	-	-	-	-	-
ENSG00000106397	PLOD3	0.377159598	3.83E-13	0.196750219	0.003087	0.251426288	3.53E-05	-0.17362252	0.000442	-0.110804021	0.030661	0.057422945	0.552062	C5	-	Y	-	-	-	-	-
ENSG00000106399	RPA3	-0.338473332	9.49E-07	-0.298648995	0.000255	-0.353981976	1.14E-06	0.046206909	0.595667	-0.000731526	0.992789	-0.052240207	0.695357	C4	-	Y	-	Y	-	-	-
ENSG00000106477	CEP41	-0.475182378	2.87E-16	-0.193888446	0.012779	-0.185375961	0.005053	0.287730497	3.49E-06	0.304388354	2.43E-08	0.011364096	0.947133	C4	-	Y	-	-	-	Y	-
ENSG00000106479	ZNF862	0.261410211	0.004093	0.353631892	0.000239	0.406628334	7.63E-06	0.098523193	0.310369	0.159809559	0.067445	0.055921502	0.736482	C4	-	-	Y	Y	-	-	-
ENSG00000106524	ANKMY2	-0.08438252	0.6135	0.161828814	0.330182	0.349339896	0.007571	0.253021044	0.088726	0.448290606	0.000611	0.190217085	0.338201	C4	-	-	-	Y	-	Y	-
ENSG00000106591	MRPL32	-0.088554986	0.193127	-0.150029359	0.062539	-0.304305119	1.46E-06	-0.054980436	0.498602	-0.201051509	0.001044	-0.151525914	0.101265	C4	-	-	-	Y	-	-	-
ENSG00000106628	POLD2	-0.443389741	4.48E-13	-0.144943247	0.051813	-0.163631912	0.023365	0.305215134	1.61E-09	0.294607435	9.79E-09	-0.015947495	0.904853	C4	-	Y	-	-	Y	-	-
ENSG00000106633	GCK	-1.001003889	0.001234	-0.07535147	0.875468	-0.43555066	0.222245	0.933351706	0.003002	0.580799108	0.114196	-0.356934966	NA	C2	-	Y	-	-	Y	-	-
ENSG00000106665	CLIP2	0.301993024	1.24E-07	0.039349818	0.666785	0.171992343	0.005909	-0.256011456	7.27E-06	-0.115301071	0.052634	0.135590126	0.077493	C5	-	Y	-	-	-	-	-
ENSG00000106688	SLC1A1	0.310011973	0.051458	0.234526293	0.230508	0.407168149	0.0152	-0.069158153	0.734031	0.112063531	0.549214	0.175623536	0.493975	C4	-	-	-	Y	-	-	-
ENSG00000106771	TMEM245	0.32138653	6.03E-07	0.159120314	0.103751	0.07945316	0.385827	-0.155912448	0.025481	-0.227398158	3.16E-05	-0.076817294	0.542155	C5	-	Y	-	-	-	-	-
ENSG00000106785	TRIM14	-0.433006354	0.024484	-0.10366782	0.689634	-0.038061342	0.889326	0.336836255	0.094201	0.410068985	0.031856	0.069030714	0.853204	C4	-	Y	-	-	-	Y	-
ENSG00000106789	CORO2A	-0.35590381	0.013285	-0.060555632	0.804645	0.159743535	0.329079	0.301141877	0.031071	0.5303939	2.34E-06	0.224599392	0.19895	C4	-	Y	-	-	Y	Y	-
ENSG00000106804	C5	-0.763495842	7.58E-12	0.060005451	0.744865	0.123551711	0.353223	0.829731845	9.85E-15	0.90131977	6.45E-21	0.066820834	0.738896	C2	-	Y	-	-	Y	Y	-
ENSG00000106829	TLE4	-0.421465337	5.79E-13	-0.392542461	7.64E-07	-0.339954256	2.81E-07	0.03555527	0.721711	0.096281974	0.181641	0.05522829	0.727739	C4	-	Y	Y	Y	-	-	-
ENSG00000106852	LHX6	-2.161460126	0.00037	-0.473178495	0.498006	-0.516004119	0.451316	1.69449011	0.020123	1.656679079	0.029226	-0.041517254	NA	C3	-	Y	-	-	Y	Y	-
ENSG00000106853	PTGR1	-0.546174606	0.018645	-0.646353641	0.014178	-0.435894407	0.113197	-0.093579443	0.77515	0.125886337	0.691537	0.213540305	0.637807	C4	-	Y	Y	-	-	-	-
ENSG00000106948	AKNA	-0.849583631	3.04E-07	-0.805809411	9.18E-06	-0.433370186	0.035316	0.049633767	0.871489	0.431081648	0.057932	0.376843809	0.248547	C4	-	Y	Y	Y	-	-	-
ENSG00000106991	ENG	-0.651994989	2.48E-22	-0.030917305	0.776034	-0.130768323	0.073901	0.627842224	5.73E-21	0.536035931	9.07E-17	-0.096775473	0.331685	C4	-	Y	-	-	Y	Y	-
ENSG00000106992	AK1	-0.496559305	0.002627	-0.241140165	0.227207	-0.058777933	0.802993	0.262045225	0.149343	0.452768547	0.0048	0.185265138	0.456495	C4	-	Y	-	-	-	Y	-
ENSG00000106993	CDC37L1	0.485217481	2.70E-07	-0.253664452	0.042254	0.13892122	0.305959	-0.732680633	1.31E-16	-0.331649394	0.000354	0.395476803	0.000928	C5	-	Y	-	-	Y	Y	Y
ENSG00000107020	PLGRKT	-0.713220952	1.27E-09	-0.085363805	0.626781	-0.094068225	0.559018	0.634455779	5.88E-08	0.634116218	5.58E-08	-0.005426221	0.986496	C4	-	Y	-	-	Y	Y	-
ENSG00000107036	RIC1	0.53221452	5.28E-11	0.358369749	0.000239	0.32445315	0.000461	-0.167942049	0.018809	-0.193401117	0.003958	-0.030827541	0.854084	C1	-	Y	Y	Y	-	-	-
ENSG00000107140	TESK1	0.67264786	6.47E-17	0.334919733	0.001412	0.250485441	0.022272	-0.330978625	0.000126	-0.407660967	2.23E-06	-0.081303008	0.672522	C5	-	Y	Y	-	Y	Y	-
ENSG00000107147	KCNT1	-0.150147068	0.012536	-0.005420246	0.967389	0.13746892	0.076962	0.151007788	0.020448	0.302576044	1.86E-07	0.145693075	0.13587	C4	-	-	-	-	-	Y	-
ENSG00000107185	RGP1	0.406128804	1.27E-11	0.253496567	0.000186	0.305517694	7.98E-07	-0.146173886	0.0105	-0.085984033	0.145103	0.054830429	0.572698	C5	-	Y	-	Y	-	-	-
ENSG00000107263	RAPGEF1	0.255594419	0.000191	0.304865446	4.07E-05	0.167776834	0.016272	0.055808369	0.509928	-0.073232928	0.29131	-0.134289166	0.128656	C1	-	-	Y	-	-	-	-

ENSG00000107282	APBA1	-0.359234407	0.023475	0.076289885	0.755378	0.139843079	0.474811	0.441967503	0.004187	0.513732306	0.000419	0.066911601	0.838445	C4	-	Y	-	-	Y	Y	-
ENSG00000107290	SETX	0.520932881	2.31E-10	0.165384699	0.171757	0.188215543	0.075891	-0.349161912	9.73E-09	-0.318081302	6.71E-09	0.025659271	0.887679	C5	Y	Y	-	-	Y	Y	-
ENSG00000107338	SHB	-0.960993734	1.05E-16	-0.19832457	0.173348	-0.292050977	0.021565	0.769183407	3.83E-11	0.683880616	4.36E-09	-0.091008764	0.67544	C2	-	Y	-	-	Y	Y	-
ENSG00000107404	DVL1	0.517097358	9.10E-12	0.307752275	0.000327	0.288837505	8.64E-05	-0.202787538	0.009985	-0.21339277	0.001939	-0.016205419	0.931126	C5	-	Y	Y	-	-	-	-
ENSG00000107438	PDLIM1	-0.602206397	0.016352	-0.159613311	0.646601	-0.105673671	0.751709	0.448701017	0.113156	0.511471581	0.057793	0.056754444	0.927911	C4	-	Y	-	-	-	-	-
ENSG00000107443	CCNJ	0.107203045	0.137334	-0.266566063	0.001446	-0.301428498	3.24E-05	-0.367059354	1.06E-07	-0.394016172	4.25E-10	-0.031998414	0.861463	C4	-	-	-	Y	Y	Y	-
ENSG00000107518	ATRNL1	-0.293722385	0.001552	-0.006129879	0.973219	0.01349073	0.931755	0.293969901	0.001601	0.32180871	0.000185	0.022539967	0.927485	C4	-	-	-	-	-	Y	-
ENSG00000107537	PHYH	-0.480560885	2.21E-05	-0.204611994	0.189694	-0.116188951	0.478395	0.282700843	0.035347	0.379330676	0.002454	0.091155969	0.727246	C4	-	Y	-	-	-	Y	-
ENSG00000107551	RASSF4	-1.054366944	6.12E-09	-0.630865966	0.000868	-0.335178934	0.094569	0.430557977	0.068475	0.733931831	0.00054	0.298466743	0.331685	C3	-	Y	Y	-	-	Y	-
ENSG00000107554	DNMBP	0.382823056	0.002242	0.2475813	0.085975	0.375462647	0.004927	-0.129186887	0.32661	0.006810228	0.965562	0.130579103	0.498963	C5	-	Y	-	Y	-	-	-
ENSG00000107562	CXCL12	0.878853415	5.21E-08	1.56181904	3.17E-18	1.423348721	2.07E-16	0.689122381	2.30E-05	0.55985292	0.000399	-0.135735481	0.704067	C1	-	Y	Y	Y	Y	Y	-
ENSG00000107566	ERLIN1	0.342265165	1.82E-07	-0.003631365	0.980292	0.183337819	0.032054	-0.339439334	4.63E-07	-0.144239319	0.05204	0.189864114	0.063659	C5	-	Y	-	-	Y	-	-
ENSG00000107581	EIF3A	0.311860498	7.30E-13	0.106188801	0.062427	0.042105155	0.482085	-0.199221514	5.55E-09	-0.255178869	7.43E-19	-0.061323438	0.241262	C5	Y	Y	-	-	-	-	-
ENSG00000107611	CUBN	-0.72678598	0.319029	0.696930434	0.316006	0.277666232	0.742292	1.427713767	0.015053	1.017427759	0.131176	-0.417123018	NA	C2	-	-	-	-	Y	-	-
ENSG00000107614	TRDMT1	-0.155001334	0.327228	0.1839248	0.293205	0.042716324	0.852653	0.345260681	0.009256	0.212292515	0.174193	-0.138414548	0.563462	C4	-	-	-	-	Y	-	-
ENSG00000107669	ATE1	0.308064548	1.60E-07	0.280880551	5.18E-05	0.191813745	0.007006	-0.0208521	0.790756	-0.101544426	0.093009	-0.086162043	0.377464	C1	-	Y	-	-	-	-	-
ENSG00000107672	NSMCE4A	-0.196001288	0.007356	-0.115769851	0.231184	-0.321706547	3.55E-05	0.086647127	0.318826	-0.110915335	0.186652	-0.20301232	0.04336	C4	-	-	-	Y	-	-	-
ENSG00000107679	PLEKHA1	0.356499317	1.07E-07	0.262819691	0.002969	0.299791285	6.63E-05	-0.087412093	0.321506	-0.041915813	0.634405	0.039956654	0.818037	C5	-	Y	-	-	-	-	-
ENSG00000107731	UNC5B	0.188427576	8.22E-07	0.335657653	1.17E-15	0.265908256	2.76E-13	0.153715073	3.29E-05	0.092122145	0.003456	-0.066843689	0.227747	C4	Y	-	Y	-	-	-	-
ENSG00000107738	VSIR	-1.673168164	0.047303	-1.548549526	NA	-0.478515507	0.549446	0.139890341	0.934696	1.209182589	0.196284	1.072685198	NA	C3	-	Y	-	-	-	-	-
ENSG00000107742	SPOCK2	-1.326793443	7.44E-12	-0.273674691	0.24537	-0.43571564	0.043214	1.05968796	7.13E-08	0.905510315	1.75E-05	-0.15925928	0.654553	C3	-	Y	-	Y	Y	Y	-
ENSG00000107771	CCSER2	0.414894332	2.04E-09	0.36583797	9.55E-07	0.262038843	0.002042	-0.042683673	0.620103	-0.138117759	0.074987	-0.100852453	0.399832	C1	-	Y	Y	-	-	-	-
ENSG00000107789	MINPP1	0.387523368	2.17E-06	0.168207592	0.123313	0.212513001	0.046101	-0.212314096	0.001643	-0.159792867	0.036563	0.047273869	0.769676	C1	-	Y	-	-	-	-	-
ENSG00000107796	ACTA2	-0.16592965	0.332835	0.4594584	0.007222	0.43151739	0.014514	0.632160564	5.67E-05	0.612428518	0.000178	-0.025556774	0.956995	C4	-	-	Y	Y	Y	Y	-
ENSG00000107798	LIPA	-0.112748265	0.316619	0.112236791	0.379449	0.197429876	0.08644	0.231355221	0.024049	0.324802079	0.001558	0.088133556	0.635548	C4	-	-	-	-	-	Y	-
ENSG00000107821	KAZALD1	-1.312908826	6.81E-05	-0.348065531	0.390718	-0.340422532	0.360296	0.973456043	0.011812	0.986974007	0.008614	0.010044033	NA	C3	-	Y	-	-	Y	Y	-
ENSG00000107829	FBXW4	-0.447485136	4.35E-05	-0.139937576	0.327111	-0.288828821	0.027192	0.314471788	0.001135	0.17347211	0.142171	-0.146165851	0.366839	C4	-	Y	-	-	Y	-	-
ENSG00000107854	TNKS2	0.424532891	1.87E-08	0.04368279	0.744865	0.172916211	0.05356	-0.374358415	3.45E-12	-0.237223733	4.02E-06	0.131774092	0.092398	C5	-	Y	-	-	Y	-	-
ENSG00000107864	CPEB3	0.513859157	0.001553	0.338353767	0.120164	0.510490535	0.001954	-0.169817877	0.376333	0.010906028	0.957755	0.175983967	0.512021	C1	-	Y	-	Y	-	-	-
ENSG00000107882	SUFU	0.464168553	2.71E-07	0.401609144	1.41E-05	0.261572117	0.008114	-0.056315415	0.601194	-0.187948424	0.035236	-0.137133921	0.256859	C1	-	Y	Y	-	-	-	-
ENSG00000107890	ANKRD26	0.624444282	2.68E-12	0.333583241	0.003087	0.471624539	7.16E-06	-0.284826107	0.000411	-0.137797127	0.129617	0.141289827	0.316957	C5	-	Y	Y	Y	-	-	-
ENSG00000107897	ACBD5	0.508425145	5.52E-09	0.269417717	0.010377	0.334792921	0.000475	-0.233207019	0.000283	-0.1591414	0.014851	0.068719316	0.555009	C5	-	Y	-	Y	-	-	-
ENSG00000107929	LARP4B	0.266604694	3.35E-05	-0.050268243	0.590603	0.055258022	0.556751	-0.310447847	3.32E-07	-0.196936435	0.00227	0.108367163	0.282066	C5	-	-	-	-	Y	-	-
ENSG00000107938	EDRF1	0.366751744	1.14E-08	-0.054759486	0.648059	0.193071789	0.033127	-0.414993762	8.19E-12	-0.159297629	0.021941	0.250623414	0.009151	C5	-	Y	-	-	Y	-	-
ENSG00000107957	SH3PXD2A	0.271141635	0.00022	0.419324176	1.60E-07	0.506921649	3.22E-11	0.154741717	0.000129	0.249992626	4.77E-10	0.090147496	0.242952	C4	-	-	Y	Y	-	-	-
ENSG00000107960	STN1	-0.21830864	0.091585	0.107076277	0.543864	0.214167293	0.093516	0.331733179	0.004209	0.447058428	1.18E-05	0.109840378	0.557454	C4	-	-	-	-	Y	Y	-
ENSG00000107984	DKK1	0.460498258	1.31E-09	0.555139539	2.05E-08	0.384749282	2.04E-06	0.100646181	0.33954	-0.061114551	0.49762	-0.167094028	0.210317	C1	-	Y	Y	Y	-	-	-
ENSG00000108021	TASOR2	0.314843018	6.00E-05	0.132498636	0.22524	0.110919811	0.279341	-0.176094422	0.000238	-0.189511028	4.69E-06	-0.01874638	0.882397	C1	-	Y	-	-	-	-	-
ENSG00000108091	CCDC6	0.608748664	9.00E-27	0.270048411	0.003157	0.193683736	0.007182	-0.332376839	3.14E-06	-0.400267272	2.93E-13	-0.07355967	0.619035	C5	Y	Y	-	-	Y	Y	-
ENSG00000108175	ZMIZ1	-0.437414987	1.38E-18	-0.099552877	0.081505	-0.117864828	0.036063	0.344387392	5.21E-18	0.334108818	1.76E-16	-0.015567911	0.875615	C4	-	Y	-	-	Y	Y	-
ENSG00000108179	PPIF	-0.367483291	3.38E-12	-0.111418744	0.133287	-0.233840255	0.000278	0.262488362	1.10E-06	0.148242595	0.011489	-0.119531505	0.175445	C4	-	Y	-	-	-	-	-
ENSG00000108187	PBLD	-0.234904201	0.106572	-0.050822088	0.806662	-0.328278518	0.041659	0.190899722	0.220817	-0.07861687	0.707445	-0.275160949	0.190902	C4	-	-	-	Y	-	-	-
ENSG00000108219	TSPAN14	-0.321378912	8.65E-06	-0.21059339	0.013341	-0.174359848	0.053049	0.117261274	0.123412	0.162063675	0.033231	0.039325898	0.80132	C4	-	Y	-	-	-	-	-
ENSG00000108239	TBC1D12	0.671462313	2.27E-09	0.457827369	0.001485	0.387543402	0.003671	-0.206596576	0.055217	-0.26968662	0.00494	-0.068350468	0.760807	C1	-	Y	Y	Y	-	-	-
ENSG00000108256	NUFIP2	0.629586668	6.47E-17	-0.072703883	0.612745	0.259877668	0.003209	-0.695890749	3.32E-27	-0.355379032	3.82E-12	0.335340336	4.94E-05	C5	-	Y	-	-	Y	Y	Y
ENSG00000108262	GIT1	0.403152734	2.86E-16	0.355035646	1.62E-10	0.26610024	3.09E-06	-0.041462408	0.472739	-0.122338838	0.010903	-0.086177189	0.260143	C1	-	Y	Y	-	-	-	-
ENSG00000108370	RGS9	-0.734043688	9.96E-08	-0.15482769	0.45633	-0.21735651	0.235757	0.587254979	0.000544	0.531149728	0.00193	-0.061706043	0.885587	C4	-	Y	-	-	Y	Y	-
ENSG00000108379	WNT3	-0.903814487	3.16E-07	-0.510514791	0.008108	-0.655427057	0.000329	0.400547957	0.074718	0.262909635	0.286129	-0.142982646	0.721346	C3	-	Y	Y	Y	-	-	-
ENSG00000108387	SEPTIN4	-0.986463657	9.58E-07	-0.638863502	0.002971	0.093490362	0.737999	0.355135613	0.208991	1.094406196	5.91E-07	0.734856947	0.004545	C4	-	Y	Y	-	-	Y	Y
ENSG00000108395	TRIM37	0.427272643	3.92E-16	0.210942642	0.001305	0.229117992	0.000199	-0.209634336	2.67E-05	-0.183489958	0.000194	0.02088637	0.876139	C5	-	Y	-	-	-	-	-
ENSG00000108423	TUBD1	0.774937166	4.11E-07	0.24191487	0.297758	0.423524111	0.017716	-0.52645074	0.000673	-0.336583339	0.024531	0.184625787	0.498637	C5	-	Y	-	Y	Y	Y	-
ENSG00000108439	PNPO	-0.079693772	0.570848	-0.05810242	0.766668	0.268154379	0.019255	0.028324912	0.854037	0.362144026	7.94E-05	0.327732318	0.005676	C4	-	-	-	-	-	Y	Y
ENSG00000108443	RPS6KB1	0.457801201	1.97E-11	0.046809103	0.69883	-0.021021599	0.855271	-0.404381027	1.67E-11	-0.464176901	1.12E-17	-0.065271001	0.571036	C5	-	Y	-	-	Y	Y	-
ENSG00000108479	GALK1	-0.814936869	8.72E-10	-0.433819635	0.001788	-0.383462777	0.005691	0.38808305	0.017849	0.446157632	0.005327	0.052888196	0.880973	C4	Y	Y	Y	Y	Y	Y	-
ENSG00000108509	CAMTA2	0.559992738	7.05E-09	0.490387412	3.03E-06	0.462867187	1.10E-06	-0.063477955	0.63528	-0.082477798	0.484584	-0.024790971	0.924657	C1	-	Y	Y	Y	-	-	-
ENSG00000108510	MED13	0.398560976	0.002191	0.054585225	0.72939	0.092325249	0.431611	-0.337647297	1.96E-07	-0.291956139	7.92E-09	0.040408153	0.778535	C5	-	Y	-	-	Y	-	-
ENSG00000108515	ENO3	-0.379859794	0.002383	-0.017542633	0.934355	0.139312693	0.366668	0.36925213	0.002367	0.534312818	5.05E-06	0.160109311	0.381698	C4	-	Y	-	-	Y	Y	-
ENSG00000108528	SLC25A11	-0.25003153	0.001365	-0.020347171	0.886832	0.036442518	0.751709	0.236255437	0.001558	0.30143384	7.33E-06	0.059859493	0.653173	C4	-	-	-	-	-	Y	-
ENSG00000108551	RASD1	-1.748405281	0.001961	-0.082486651	0.918788	-0.803015575	0.20896	1.67647474	0.006608	0.961386795	0.23068	-0.720229709	NA	C2	-	Y	-	-	Y	-	-
ENSG00000108559	NUP88	0.334478542	1.80E-09	0.064923604	0.537302	0.004293534	0.96874	-0.262793961	1.91E-05	-0.315504554	8.20E-10	-0.057990337	0.650345	C5	-	Y	-	-	-	Y	-
ENSG00000108590	MED31	0.434618336	0.003161	0.084558616	0.741931	0.201308609	0.30528	-0.34385064	0.029605	-0.21856354	0.185868	0.119698551	0.697636	C5	-	Y	-	-	Y	-	-
ENSG00000108622	ICAM2	-1.176805486	0.01003	-0.540490001	0.308158	-0.250099622	0.646249	0.642230558	0.280901	0.941658986	0.068413	0.293604415	NA	C3	-	Y	-	-	-	-	-
ENSG00000108641	B9D1	-0.772185518	1.39E-07	0.004102689	0.987565	0.030900272	0.889552	0.783067282	1.09E-07	0.817812748	2.30E-08	0.029756176	0.933843	C2	-	Y	-	-	Y	Y	-
ENSG00000108654	DDX5	-0.223433895	9.17E-14	-0.16381958	0.000284	-0.317384143	1.02E-21	0.066115192	0.05839	-0.079254695	0.001903	-0.150704606	0.00033	C4	-	-	-	Y	-	-	-
ENSG00000108679	LGALS3BP	-0.568724559	7.97E-34	-0.19105673	0.022446	-0.188051389	0.001762	0.384169288	3.46E-10	0.395750838	7.71E-20	0.005851874	0.9772	C4	-	Y	-	-	Y	Y	-
ENSG00000108691	CCL2	2.850169668	3.58E-24	3.519897888	5.23E-38	2.805542031	4.76E-22	0.675788009	0.001298	-0.028947939	0.928271	-0.710740998	0.006837	C1	-	Y	Y	Y	Y	-	Y
ENSG00000108733	PEX12	-0.076952509	0.694746	-0.657019952	0.000529	-0.198126984	0.34238	-0.573562399	0.000807	-0.107027938	0.590827	0.461926187	0.057727	C5	-	-	Y	-	Y	-	-

ENSG00000108771	DHX58	-2.042594594	0.039606	-0.188738191	NA	-1.997771722	NA	1.871214492	0.156979	0.050713359	NA	-1.828350723	NA	C2	-	Y	-	-	-	-	-
ENSG00000108784	NAGLU	-0.297426316	0.040575	0.053392239	0.818642	-0.02184477	0.916451	0.357617474	0.015005	0.290602857	0.03232	-0.072316913	0.789446	C4	-	-	-	-	Y	-	-
ENSG00000108785	HSD17B1P1	0.315340751	0.198139	0.153481374	0.653839	0.547555016	0.030544	-0.155698886	0.599646	0.24686643	0.368676	0.396763381	0.267146	C4	-	-	-	Y	-	-	-
ENSG00000108788	MLX	0.499894592	8.18E-10	0.099539528	0.412613	0.171979581	0.076442	-0.393665718	1.71E-09	-0.313179129	6.67E-07	0.074857045	0.527709	C5	Y	Y	-	-	Y	Y	-
ENSG00000108797	CNTNAP1	-0.010442818	0.933417	0.108890037	0.322566	0.289498477	9.58E-05	0.125953613	0.184602	0.314537591	1.26E-05	0.183554248	0.082976	C4	-	-	-	-	-	Y	-
ENSG00000108799	EZH1	0.348803143	3.59E-06	0.280813154	0.000558	0.307055327	0.005174	-0.061602949	0.491472	-0.027419346	0.835882	0.028826577	0.906437	C1	-	Y	-	Y	-	-	-
ENSG00000108819	PPP1R9B	0.521364733	2.16E-30	0.228385643	2.72E-05	0.300339945	2.25E-08	-0.28645517	1.46E-11	-0.206326024	3.30E-06	0.074821004	0.349529	C5	-	Y	-	Y	-	-	-
ENSG00000108821	COL1A1	-1.062176006	0.000361	-0.290098302	0.431877	0.406858555	0.284116	0.77898164	0.009275	1.483857402	1.03E-06	0.699782677	0.073344	C4	-	Y	-	-	Y	Y	-
ENSG00000108830	RND2	-0.494896161	2.40E-05	-0.260978089	0.066224	-0.228687206	0.134558	0.240852291	0.022799	0.280735502	0.014047	0.034674124	0.899704	C4	-	Y	-	-	-	-	-
ENSG00000108839	ALOX12	-0.656658923	0.117423	0.270454311	0.587506	0.565565343	0.07578	0.933633756	0.030504	1.235941451	0.000416	0.298042907	NA	C3	-	-	-	-	Y	Y	-
ENSG00000108846	ABCC3	1.338863144	7.35E-11	0.921752133	0.000585	0.945992512	0.00102	-0.411628906	0.061557	-0.377102174	0.129309	0.026725468	0.970638	C1	-	Y	Y	Y	-	-	-
ENSG00000108854	SMURF2	0.335372393	1.85E-05	0.121057802	0.233884	0.093136481	0.315571	-0.207947644	0.005371	-0.228026489	0.0005	-0.02516388	0.877727	C5	-	Y	-	-	-	-	-
ENSG00000108861	DUSP3	0.332292617	2.57E-06	0.357933685	4.46E-06	0.268452547	0.000251	0.032227085	0.751993	-0.048985847	0.575959	-0.08624397	0.476378	C4	-	Y	Y	-	-	-	-
ENSG00000108924	HLF	-0.72284681	1.50E-05	-0.527952475	0.004183	-0.29261395	0.1537	0.201698262	0.37882	0.444689478	0.028233	0.237750502	0.454661	C4	-	Y	Y	-	-	Y	-
ENSG00000108947	EFNB3	0.366799499	5.35E-13	0.103490374	0.215891	0.196386144	0.002566	-0.257118263	6.14E-06	-0.155569343	0.004295	0.095837682	0.349512	C5	-	Y	-	-	-	-	-
ENSG00000108984	MAP2K6	-0.798761333	2.70E-16	-0.026575474	0.88136	0.149155879	0.271033	0.778609142	5.90E-19	0.962748744	5.67E-26	0.17866807	0.23125	C4	-	Y	-	-	Y	Y	-
ENSG00000109046	WSB1	-0.513245549	1.23E-25	-0.044207978	0.641263	-0.042289572	0.554075	0.475522475	1.20E-20	0.485607184	3.33E-35	0.005007818	0.974427	C2	-	Y	-	-	Y	Y	-
ENSG00000109062	SLC9A3R1	-0.671469887	4.66E-19	-0.246110298	0.001406	-0.049501867	0.633995	0.4321217	3.88E-08	0.636694027	9.96E-18	0.199562798	0.02897	C4	-	Y	-	-	Y	Y	-
ENSG00000109063	MYH3	-0.53166615	0.020785	-0.103397828	0.755703	0.07613677	0.811737	0.434565164	0.077527	0.622635971	0.006826	0.182296708	0.635328	C4	-	Y	-	-	-	Y	-
ENSG00000109066	TMEM104	0.489955169	1.30E-06	0.103767532	0.480226	0.289440027	0.012484	-0.379782512	4.91E-05	-0.186197674	0.072882	0.188264551	0.187391	C5	-	Y	-	-	Y	-	-
ENSG00000109079	TNFAIP1	0.358315418	2.01E-10	0.096039755	0.251333	0.136821391	0.066539	-0.255869718	1.25E-05	-0.206752848	0.000524	0.043703476	0.743614	C5	-	Y	-	-	-	-	-
ENSG00000109099	PMP22	-0.892225885	1.16E-10	-0.245643653	0.08571	-0.193094341	0.18462	0.653452734	9.23E-06	0.713638635	8.92E-07	0.055470332	0.848238	C2	-	Y	-	-	Y	Y	-
ENSG00000109103	UNC119	0.301261073	3.75E-05	0.166915618	0.130336	0.221146337	0.011594	-0.128011861	0.148895	-0.065486436	0.440423	0.057289483	0.739067	C5	-	Y	-	-	-	-	-
ENSG00000109107	ALDOC	-0.265202032	0.001871	0.085313507	0.582142	0.402231055	8.94E-06	0.357194105	0.000218	0.682552007	1.01E-19	0.31990205	0.009797	C4	-	-	-	Y	Y	Y	Y
ENSG00000109132	PHOX2B	-0.505225299	1.43E-29	-0.087849935	0.191679	-0.218460823	5.77E-06	0.423860659	7.35E-23	0.301541508	2.02E-15	-0.12768353	0.034948	C4	Y	Y	-	-	Y	Y	-
ENSG00000109171	SLAIN2	0.309698181	3.97E-09	0.132296252	0.082563	0.127234189	0.070868	-0.170864354	0.003754	-0.167689864	0.002583	-0.002154699	0.991365	C5	-	Y	-	-	-	-	-
ENSG00000109205	ODAM	-0.082535882	0.961762	2.553002986	1.02E-05	2.681878385	4.13E-06	2.63429881	1.54E-05	2.770474117	6.41E-06	0.133694171	NA	C2	-	-	Y	Y	Y	Y	-
ENSG00000109255	NMU	-0.258016086	0.062951	-0.227935816	0.224378	-0.300139207	0.030814	0.03720032	0.843543	-0.027024001	0.866726	-0.070031954	0.79766	C4	-	-	-	Y	-	-	-
ENSG00000109320	NFKB1	0.501095827	1.84E-05	0.301276481	0.038414	0.26790432	0.058957	-0.192493309	0.06112	-0.21818317	0.030086	-0.031218183	0.901588	C1	-	Y	Y	-	-	-	-
ENSG00000109323	MANBA	0.424338714	0.0004	0.435760266	0.001215	0.506349343	0.000125	0.018025041	0.915335	0.096779912	0.498282	0.073432601	0.767009	C1	-	Y	Y	Y	-	-	-
ENSG00000109339	MAPK10	-0.529778182	1.61E-08	-0.151915509	0.300449	-0.022615121	0.883564	0.384303573	0.000623	0.521948537	1.62E-08	0.131780781	0.469108	C4	-	Y	-	-	Y	Y	-
ENSG00000109445	ZNF330	0.6458248	1.38E-16	0.229494882	0.048635	0.379348367	6.94E-06	-0.410117606	2.24E-07	-0.2518211	0.000154	0.15285117	0.216395	C5	-	Y	-	Y	Y	-	-
ENSG00000109458	GAB1	0.21827051	0.034377	0.106079392	0.512143	0.399282929	0.000207	-0.106384611	0.280926	0.195879737	0.013157	0.296491311	0.008169	C4	-	-	-	Y	-	-	-
ENSG00000109466	KLHL2	0.38139045	0.000391	0.123932285	0.483306	0.174688464	0.214089	-0.250331631	0.018882	-0.192460066	0.06095	0.052716642	0.832105	C5	-	Y	-	-	-	-	-
ENSG00000109472	CPE	-0.397313948	1.60E-08	-0.220102973	0.019408	-0.321820499	6.88E-05	0.183713689	0.017002	0.090637027	0.259241	-0.098615546	0.463768	C4	-	Y	-	Y	-	-	-
ENSG00000109501	WFS1	-0.248863657	0.049377	0.091801241	0.568923	0.191007679	0.139474	0.347468142	0.004151	0.454513016	8.15E-05	0.102186006	0.605953	C4	-	-	-	-	Y	Y	-
ENSG00000109534	GAR1	-0.034257828	0.65663	-0.19620858	0.003684	-0.348818188	9.18E-08	-0.155477333	0.01251	-0.299987625	9.09E-07	-0.149672299	0.097949	C4	-	-	-	Y	-	-	-
ENSG00000109536	FRG1	0.180223058	0.086363	-0.020163983	0.915478	-0.142068768	0.270959	-0.194414961	0.036902	-0.307408913	0.00025	-0.118342653	0.446511	C5	-	-	-	-	-	Y	-
ENSG00000109572	CLCN3	0.393089751	7.63E-12	0.137307498	0.069765	0.120202521	0.092536	-0.249212709	1.16E-09	-0.25837578	4.12E-11	-0.014586675	0.899085	C5	-	Y	-	-	-	-	-
ENSG00000109586	GALNT7	0.302230747	1.85E-06	0.130933164	0.134103	0.155877989	0.051234	-0.16461592	0.01087	-0.131959564	0.044972	0.027608524	0.862235	C5	-	Y	-	-	-	-	-
ENSG00000109689	STIM2	0.302786474	0.000134	0.378943718	0.000312	0.156781858	0.124923	0.082483748	0.459529	-0.131523487	0.138942	-0.219218236	0.101427	C4	-	Y	Y	-	-	-	-
ENSG00000109743	BST1	-1.075626675	0.009211	-0.767570097	0.108839	-0.856818129	0.052517	0.313273953	0.627137	0.233400861	0.728222	-0.085460329	NA	C3	-	Y	-	-	-	-	-
ENSG00000109762	SNX25	0.565376026	5.53E-08	0.20641017	0.164599	0.259606474	0.057132	-0.352836633	0.000473	-0.291148041	0.004787	0.056367153	0.818037	C5	-	Y	-	-	Y	-	-
ENSG00000109814	UGDH	0.412380991	2.22E-09	0.072845341	0.590375	0.104794388	0.328354	-0.332521028	1.89E-05	-0.293080406	3.95E-05	0.034951161	0.873941	C5	-	Y	-	-	Y	-	-
ENSG00000109832	DDX25	-0.606929687	5.42E-05	-0.217860588	0.243363	-0.158432582	0.400245	0.395626267	0.013616	0.463569257	0.002867	0.062228943	0.848977	C4	-	Y	-	-	Y	Y	-
ENSG00000109911	ELP4	-0.317940184	0.034555	-0.164969667	0.404464	-0.199116913	0.255489	0.159397729	0.407046	0.1335763	0.486355	-0.031375702	0.93984	C4	-	Y	-	-	-	-	-
ENSG00000109920	FNBP4	0.346701763	1.18E-12	0.149126339	0.014178	0.138989429	0.019745	-0.191075131	6.34E-05	-0.193088776	4.25E-05	-0.007369806	0.958591	C5	-	Y	-	-	-	-	-
ENSG00000109929	SC5D	0.313466563	4.18E-06	0.022384434	0.841038	0.193142198	0.015582	-0.28476314	1.69E-05	-0.105661682	0.19292	0.173674279	0.064773	C5	-	Y	-	-	-	-	-
ENSG00000109971	HSPA8	0.643053964	1.04E-50	0.475033746	2.80E-17	0.91917284	9.43E-82	-0.161643048	0.000124	0.290574645	1.86E-18	0.446984276	2.45E-19	C1	-	Y	Y	Y	-	-	Y
ENSG00000110011	DNAJC4	-0.212055109	0.040615	0.117525529	0.417815	-0.012936905	0.948965	0.336035416	0.000565	0.214210369	0.065314	-0.127436743	0.524522	C4	-	-	-	-	Y	-	-
ENSG00000110013	SIAE	-0.417292045	0.001695	-0.065627979	0.782716	-0.026589427	0.907867	0.358986791	0.026359	0.40535549	0.007161	0.040691403	0.921908	C4	-	Y	-	-	Y	Y	-
ENSG00000110042	DTX4	-1.268636436	0.010356	-0.775399189	0.176645	-0.208146033	0.750966	0.501335148	0.487015	1.075468638	0.064044	0.56937799	NA	C3	-	Y	-	-	-	-	-
ENSG00000110046	ATG2A	0.373253136	0.000198	0.112894539	0.46712	0.16241555	0.217299	-0.254007932	0.019094	-0.1961218	0.076115	0.052414361	0.831267	C5	-	Y	-	-	-	-	-
ENSG00000110047	EHD1	0.568706641	1.54E-12	0.288565201	0.001934	0.398310049	2.70E-06	-0.273497238	0.000605	-0.155530749	0.063411	0.112549078	0.371283	C5	-	Y	-	Y	-	-	-
ENSG00000110057	UNC93B1	-0.489420037	0.010332	-0.165505582	0.506513	-0.083033529	0.743985	0.330591867	0.138254	0.421214837	0.041837	0.085457632	0.835119	C4	-	Y	-	-	-	Y	-
ENSG00000110060	PUS3	0.344886108	0.000225	-0.12269508	0.377414	0.044201557	0.772121	-0.461319868	5.41E-08	-0.28612811	0.000795	0.169846519	0.204806	C5	-	Y	-	-	Y	-	-
ENSG00000110066	KMT5B	-0.00706193	0.942579	-0.321145167	4.18E-05	-0.265330651	7.96E-05	-0.307420238	3.49E-06	-0.243602551	2.76E-05	0.058559183	0.685463	C4	-	-	Y	-	Y	-	-
ENSG00000110104	CCDC86	0.396970659	2.96E-06	0.062324289	0.670032	0.065547525	0.613549	-0.328056796	0.00011	-0.31678512	9.54E-05	0.005915106	0.981547	C5	-	Y	-	-	Y	Y	-
ENSG00000110171	TRIM3	0.304938172	0.001577	0.14775212	0.270121	0.151086987	0.174511	-0.150712966	0.177406	-0.138955744	0.170842	0.006041648	0.982152	C5	-	Y	-	-	-	-	-
ENSG00000110172	CHORDC1	1.228213436	5.44E-39	0.233507854	0.095831	0.750913272	2.02E-14	-0.987744617	8.08E-48	-0.463519361	1.20E-16	0.519143236	1.96E-11	C5	-	Y	-	Y	Y	Y	Y
ENSG00000110200	ANAPC15	-0.257971563	0.026011	-0.334051489	0.010454	-0.27914579	0.054167	-0.069565194	0.643915	-0.00639127	0.97048	0.057696163	0.845294	C4	-	-	Y	-	-	-	-
ENSG00000110315	RNF141	-0.362139617	3.67E-06	0.051107709	0.691312	-0.027711367	0.831744	0.41970108	9.49E-09	0.349018272	2.27E-06	-0.075966348	0.594474	C4	-	Y	-	-	Y	Y	-
ENSG00000110324	IL10RA	-1.29478162	0.024466	-0.789245551	0.166779	-0.436391232	0.483674	0.515099712	0.534464	0.873393264	0.233684	0.354728556	NA	C3	-	Y	-	-	-	-	-
ENSG00000110328	GALNT18	-0.792348568	0.014438	-0.191610002	0.675127	-0.013091854	0.979435	0.606538559	0.077612	0.794963777	0.012546	0.181138986	NA	C2	-	Y	-	-	-	Y	-
ENSG00000110330	BIRC2	0.409002734	4.29E-08	0.203001846	0.025762	0.338352282	0.000188	-0.1999773	0.001619	-0.055789344	0.533109	0.138424813	0.206154	C5	-	Y	-	Y	-	-	-
ENSG00000110395	CBL	0.712716275	8.98E-07	0.392977699	0.047109	0.406794772	0.013351	-0.313375777	0.010624	-0.291442386	5.05E-06	0.016842979	0.943834	C1	-	Y	Y	Y	Y	-	-
ENSG00000110422	HIPK3	0.392861198	1.46E-05	0.202768901	0.116429	0.142569265	0.264097	-0.183770863	0.037787	-0.235515136	0.003706	-0.057380726	0.775263	C1	-	Y	-	-	-	-	-

ENSG00000110427	KIAA1549L	-0.237687722	0.017423	0.100619457	0.435082	0.136235151	0.209485	0.34449548	8.88E-05	0.388254605	2.97E-06	0.038772182	0.834066	C4	-	-	-	-	Y	Y	-
ENSG00000110435	PDHX	0.305520288	0.00037	0.022938058	0.88354	-0.007965956	0.963885	-0.276180222	0.000795	-0.298878146	0.000843	-0.027924046	0.906344	C5	-	Y	-	-	-	-	-
ENSG00000110455	ACCS	-1.031435695	3.04E-17	-0.286901413	0.033948	-0.080605562	0.586254	0.75222676	2.13E-08	0.965489158	2.20E-16	0.209563399	0.207902	C3	-	Y	-	-	Y	Y	-
ENSG00000110492	MDK	-0.320459008	9.04E-08	-0.429973393	7.40E-09	-0.415874592	2.23E-10	-0.102797691	0.036902	-0.080372749	0.024392	0.017021148	0.899009	C4	Y	Y	Y	Y	-	-	-
ENSG00000110619	CARS1	-0.899206346	1.72E-50	-0.679877674	4.18E-09	-0.404410092	1.74E-07	0.224484851	0.205298	0.509409674	2.59E-14	0.280001174	0.293964	C3	-	Y	Y	Y	-	Y	-
ENSG00000110628	SLC22A18	-0.796572615	0.001469	-0.377550588	0.154597	-0.083726597	0.790316	0.429103203	0.17023	0.727401847	0.006579	0.295790652	0.442693	C2	-	Y	-	-	-	Y	-
ENSG00000110660	SLC35F2	0.344425804	4.98E-05	0.077525977	0.620331	0.120221305	0.319118	-0.260302093	0.009266	-0.209655179	0.025106	0.045729485	0.852721	C5	-	Y	-	-	-	-	-
ENSG00000110665	C11orf21	-2.660582057	0.000228	-0.8417119	0.170846	-0.624589263	0.369856	1.81715797	0.044043	2.047313223	0.024833	0.223473536	NA	C3	-	Y	-	-	Y	Y	-
ENSG00000110693	SOX6	-0.454176434	0.006152	-0.383649985	0.040449	-0.128165504	0.610142	0.076402044	0.763251	0.340851273	0.107107	0.25911177	0.412815	C4	-	Y	Y	-	-	-	-
ENSG00000110697	PITPNM1	0.526010862	1.19E-11	0.374185876	3.75E-05	0.499398959	9.28E-10	-0.145442751	0.109816	-0.011753854	0.920726	0.128015565	0.340602	C5	-	Y	Y	Y	-	-	-
ENSG00000110713	NUP98	0.333641206	2.53E-10	0.190501306	0.001112	0.102564398	0.085208	-0.13662808	0.010057	-0.216397181	3.86E-06	-0.08498578	0.229419	C5	-	Y	-	-	-	-	-
ENSG00000110721	CHKA	0.396416318	7.38E-07	0.144412187	0.240685	0.078361147	0.490157	-0.245748596	0.006941	-0.303377158	9.32E-05	-0.063189943	0.732055	C5	-	Y	-	-	-	Y	-
ENSG00000110768	GTF2H1	0.25265766	0.000217	-0.096479664	0.387559	0.004655525	0.972817	-0.342379809	9.64E-07	-0.233353376	0.000598	0.103850181	0.445982	C5	-	-	-	-	Y	-	-
ENSG00000110811	P3H3	0.441412708	6.06E-09	0.24510253	0.020864	0.257888142	0.002648	-0.190074576	0.031646	-0.168890991	0.029325	0.015626367	0.947028	C5	-	Y	-	-	-	-	-
ENSG00000110844	PRPF40B	-0.124072442	0.296033	0.124415418	0.415199	0.194111096	0.097298	0.25492101	0.03044	0.332790321	0.001333	0.072570782	0.746039	C4	-	-	-	-	-	Y	-
ENSG00000110880	CORO1C	0.734904968	3.16E-80	0.261106509	1.75E-08	0.178366873	0.000305	-0.467401908	1.09E-39	-0.54176871	6.56E-54	-0.079773578	0.204806	C5	-	Y	-	-	Y	Y	-
ENSG00000110881	ASIC1	-0.278493968	0.080697	-0.087118266	0.663617	0.041952322	0.83678	0.197752345	0.21603	0.335509815	0.017806	0.131429485	0.534484	C4	-	-	-	-	-	Y	-
ENSG00000110900	TSPAN11	-0.317749482	0.000876	-0.021560777	0.894609	-0.035858444	0.785292	0.302462889	0.001145	0.296435112	0.000578	-0.011321949	0.959612	C4	-	Y	-	-	Y	-	-
ENSG00000110925	CSRNP2	0.508897036	1.62E-19	0.09415883	0.258627	0.139058515	0.041544	-0.408187703	1.01E-15	-0.355182825	1.35E-13	0.04750873	0.653173	C5	-	Y	-	-	Y	Y	-
ENSG00000111011	RSRC2	0.525165246	1.85E-18	0.206302584	0.002448	0.182318754	0.008594	-0.312675459	6.52E-11	-0.328571169	6.21E-12	-0.021195206	0.860105	C5	-	Y	-	-	Y	Y	-
ENSG00000111057	KRT18	1.856450233	0.001178	0.964677603	0.161359	1.794804768	0.000973	-0.883357997	0.078994	-0.045086543	0.945499	0.832532692	NA	C5	-	Y	-	Y	-	-	-
ENSG00000111077	TNS2	-0.514421694	0.010251	-0.037812188	0.906521	0.07680937	0.779664	0.482757373	0.020927	0.605745003	0.00202	0.117443968	0.755669	C4	-	Y	-	-	Y	Y	-
ENSG00000111145	ELK3	0.136180174	0.182144	0.514164692	8.92E-07	0.404857503	0.000212	0.384482333	6.63E-05	0.283410641	0.004947	-0.106948034	0.601463	C4	-	-	Y	Y	Y	-	-
ENSG00000111186	WNT5B	-1.392078677	2.54E-08	-0.299144337	0.299562	-0.778833093	0.005383	1.099982964	5.79E-05	0.627703588	0.074978	-0.477386772	NA	C3	-	Y	-	Y	Y	-	-
ENSG00000111199	TRPV4	0.470825899	0.030548	0.926467409	9.00E-06	0.661335306	0.001762	0.462226027	0.01942	0.204940713	0.362696	-0.263020755	0.365708	C1	-	Y	Y	Y	Y	-	-
ENSG00000111224	PARP11	-0.50089672	0.000213	-0.558433641	9.20E-05	-0.500766228	0.000531	-0.051591378	0.803037	0.01460629	0.947889	0.060631674	0.861463	C4	-	Y	Y	Y	-	-	-
ENSG00000111249	CUX2	-0.189076507	0.000216	0.141206776	0.014128	0.05933892	0.364301	0.33670333	1.74E-14	0.263039901	9.09E-10	-0.079006885	0.258983	C4	-	-	-	-	Y	-	-
ENSG00000111266	DUSP16	0.101414162	0.175335	0.302822002	9.05E-05	0.303663992	9.97E-06	0.207937159	0.000697	0.216504321	9.78E-05	0.00330762	0.985624	C4	-	-	Y	Y	-	-	-
ENSG00000111271	ACAD10	-0.301112813	0.000457	-0.022374187	0.885779	0.008223638	0.956419	0.284663049	0.000187	0.323801869	7.67E-06	0.033825045	0.843922	C4	-	Y	-	-	-	Y	-
ENSG00000111300	NAA25	0.358279261	1.83E-06	-0.012545283	0.933335	-0.101689326	0.34689	-0.36434996	7.02E-10	-0.445220947	1.45E-13	-0.086371804	0.472014	C5	-	Y	-	-	Y	Y	-
ENSG00000111331	OAS3	-0.98707897	7.51E-29	-0.333292891	0.000419	-0.3279814	0.000613	0.659741942	9.98E-13	0.673516752	1.08E-12	0.00831647	0.975325	C4	-	Y	Y	Y	Y	Y	-
ENSG00000111341	MGP	0.349850319	0.1672	0.748988879	0.010266	0.796007579	0.000754	0.405342915	0.129636	0.461704394	0.038965	0.050532113	0.93752	C4	-	-	Y	Y	-	Y	-
ENSG00000111445	RFC5	0.236463983	0.000154	-0.117464057	0.227783	-0.036350751	0.798441	-0.346978847	1.97E-08	-0.258326357	0.000851	0.084576517	0.619543	C5	-	-	-	-	Y	-	-
ENSG00000111540	RAB5B	0.43342131	1.50E-14	0.157361774	0.033196	0.216744825	0.000545	-0.269362456	6.79E-07	-0.201950716	9.72E-05	0.06193134	0.536283	C5	-	Y	-	-	-	-	-
ENSG00000111641	NOP2	0.572209643	9.04E-07	0.160049698	0.383616	0.092023499	0.574396	-0.405465229	0.001458	-0.465330252	1.98E-05	-0.065765923	0.810219	C5	-	Y	-	-	Y	Y	-
ENSG00000111644	ACRBP	-0.472093526	0.217829	-0.052795091	0.928309	0.41376708	0.249053	0.424579823	0.334462	0.90030342	0.00826	0.469047601	NA	C4	-	-	-	-	-	Y	-
ENSG00000111647	UHRF1BP1L	0.500686512	5.26E-11	0.22157521	0.065915	0.140271316	0.210792	-0.272329297	0.002269	-0.345566414	6.51E-06	-0.078644509	0.682921	C5	-	Y	-	-	-	Y	-
ENSG00000111652	COPS7A	-0.371955192	7.35E-09	-0.002584117	0.983962	-0.056050417	0.530112	0.375840324	4.66E-09	0.330719686	2.07E-07	-0.050587712	0.677557	C4	-	Y	-	-	Y	Y	-
ENSG00000111664	GNB3	0.306379336	0.008218	0.174306938	0.280196	0.220825292	0.097855	-0.125717165	0.360913	-0.070719817	0.617116	0.049162489	0.85949	C5	-	Y	-	-	-	-	-
ENSG00000111696	NT5DC3	-0.067979622	0.744952	0.270987599	0.181075	0.214082132	0.332556	0.345641412	0.047616	0.296711714	0.118153	-0.053701347	0.905218	C4	-	-	-	-	Y	-	-
ENSG00000111707	SUDS3	0.53713638	3.57E-17	0.175723783	0.058348	0.298281719	1.04E-05	-0.354451753	1.02E-09	-0.224355252	3.98E-06	0.12464377	0.16245	C5	-	Y	-	-	Y	-	-
ENSG00000111727	HCFC2	0.59088969	0.000116	-0.118779502	0.642169	0.11526602	0.613825	-0.704732922	7.80E-09	-0.461862206	0.000116	0.237575849	0.21733	C5	-	Y	-	-	Y	Y	-
ENSG00000111785	RIC8B	0.505954132	1.13E-12	0.157547574	0.157542	0.078549045	0.485639	-0.341981698	8.67E-06	-0.412874822	2.77E-09	-0.07606959	0.63838	C5	-	Y	-	-	Y	Y	-
ENSG00000111790	FGFR1OP2	0.227260585	0.006487	-0.103713533	0.372454	0.126299946	0.196677	-0.324518548	1.68E-05	-0.086510096	0.312513	0.232484059	0.013285	C5	-	-	-	-	Y	-	-
ENSG00000111802	TDP2	0.291405969	4.40E-05	-0.187847211	0.027586	-0.010996001	0.931707	-0.472885704	1.06E-12	-0.287654518	2.97E-05	0.179706929	0.06417	C5	Y	-	-	-	Y	-	-
ENSG00000111816	FRK	-0.886889959	0.197908	-0.127945674	0.900527	0.687487422	0.236885	0.768035532	0.325543	1.58894116	0.004203	0.821349749	NA	C2	-	-	-	-	-	Y	-
ENSG00000111817	DSE	-0.160590715	0.337125	0.032526385	0.889599	0.175025338	0.298697	0.199331393	0.161971	0.350170047	0.006867	0.145687085	0.460975	C4	-	-	-	-	-	Y	-
ENSG00000111859	NEDD9	-0.035126004	0.880147	0.608355947	0.000229	0.604923259	4.61E-05	0.649271421	9.60E-07	0.653993967	8.51E-08	0.00014347	0.99962	C4	-	-	Y	Y	Y	Y	-
ENSG00000111860	CEP85L	0.171738264	0.229503	0.334648451	0.019597	0.150528916	0.386514	0.169008802	0.205735	-0.006498012	0.97013	-0.180954087	0.351149	C4	-	-	Y	-	-	-	-
ENSG00000111879	FAM184A	-0.25747555	0.011717	-0.30074795	0.007198	-0.194036162	0.077584	-0.037014062	0.798603	0.07799674	0.526009	0.109315411	0.526583	C4	-	-	Y	-	-	-	-
ENSG00000111880	RNGTT	0.413425861	1.99E-10	0.173795738	0.027004	0.09179564	0.32909	-0.233289872	0.00011	-0.306899235	7.61E-07	-0.079044806	0.485666	C5	-	Y	-	-	-	Y	-
ENSG00000111886	GABRR2	1.698290338	0.013194	0.741141998	NA	1.017743118	NA	-0.950504658	0.148247	-0.660495009	0.362771	0.281698116	NA	C5	-	Y	-	-	-	-	-
ENSG00000111912	NCOA7	0.596625022	1.36E-16	0.337769808	1.44E-05	0.204808562	0.018396	-0.252538988	1.23E-05	-0.377146506	1.88E-10	-0.130109573	0.136286	C5	-	Y	Y	-	-	Y	-
ENSG00000111913	RIPOR2	-1.716498688	NA	1.872236455	NA	-0.002685038	NA	3.603484835	0.028816	1.730019203	NA	-1.873427741	NA	C2	-	-	-	-	Y	-	-
ENSG00000111981	ULBP1	-2.481484945	3.22E-05	-1.527322579	0.035821	-1.091136151	0.045222	0.949574648	0.396915	1.407160252	0.08858	0.446374864	NA	C3	-	Y	Y	Y	-	-	-
ENSG00000112029	FBXO5	-0.144448755	0.01064	-0.333640365	5.16E-07	-0.319667068	1.30E-07	-0.182702968	0.004391	-0.16064023	0.007972	0.017115922	0.927911	C4	-	-	Y	Y	-	-	-
ENSG00000112033	PPARD	0.447234862	1.21E-09	0.125515314	0.215031	0.198612822	0.033533	-0.315262216	1.60E-05	-0.234307118	0.002599	0.075959289	0.61262	C5	-	Y	-	-	Y	-	-
ENSG00000112038	OPRM1	0.615265423	0.0003	0.316652259	0.140994	0.434293004	0.022191	-0.292006436	0.054375	-0.166483758	0.303097	0.120349833	0.651212	C1	-	Y	-	Y	-	-	-
ENSG00000112130	RNF8	0.331491379	0.000111	0.063590565	0.642996	0.108274057	0.391841	-0.261391622	0.002839	-0.208481671	0.029753	0.047486283	0.822807	C5	-	Y	-	-	-	-	-
ENSG00000112137	PHACTR1	0.44981249	7.47E-08	0.420164033	8.64E-06	0.265690507	0.009286	-0.023164817	0.843543	-0.169295224	0.063538	-0.151638602	0.248512	C1	-	Y	Y	-	-	-	-
ENSG00000112139	MDGA1	-0.478858358	0.01044	-0.189079104	0.59032	-0.445963497	0.055835	0.297406791	0.319284	0.047125173	0.890282	-0.256828588	0.610987	C2	-	Y	-	-	-	-	-
ENSG00000112144	CILK1	0.333831113	0.000357	0.0536819	0.751313	0.044146629	0.790316	-0.273855194	0.000605	-0.274795844	0.000914	-0.006568283	0.980847	C5	-	Y	-	-	-	-	-
ENSG00000112149	CD83	-0.589662133	0.00208	-0.397953508	0.078611	-0.332240313	0.166092	0.197564114	0.460026	0.2722155	0.300909	0.069784175	0.90074	C4	-	Y	-	-	-	-	-
ENSG00000112159	MDN1	0.659500658	1.28E-16	0.44995156	3.47E-08	0.417148515	2.91E-07	-0.203215175	0.000904	-0.227899675	0.000132	-0.029973327	0.814324	C1	-	Y	Y	Y	-	-	-
ENSG00000112186	CAP2	-0.410410142	0.138181	0.178911552	0.607497	0.409483701	0.109309	0.596538033	0.025433	0.834847638	0.000521	0.234078612	0.543603	C4	-	-	-	-	Y	Y	-
ENSG00000112200	ZNF451	0.249424744	0.000352	-0.17941865	0.081979	0.007884734	0.958462	-0.421902496	1.31E-10	-0.226602122	0.000952	0.190109167	0.107017	C5	-	-	-	-	Y	-	-
ENSG00000112210	RAB23	0.437997704	4.47E-05	0.100444463	0.598346	0.198421542	0.166328	-0.331024423	0.006547	-0.225019157	0.061406	0.101055093	0.683546	C5	Y	Y	-	-	Y	-	-

ENSG00000112245	PTP4A1	-3.956759155	0.012464	-0.886852275	NA	-1.729544694	NA	3.059638721	NA	2.249225693	NA	-0.822639154	NA	C3	-	Y	-	-	-	-	-
ENSG00000112280	COL9A1	-1.198203345	0.02357	-0.433858725	0.502001	-0.541968277	0.374209	0.770075221	0.231454	0.66995245	0.329164	-0.10550689	NA	C4	-	Y	-	-	-	-	-
ENSG00000112282	MED23	0.403381943	5.82E-07	0.004432319	0.979955	0.167859826	0.112235	-0.392903876	3.32E-10	-0.221567754	0.000612	0.166167155	0.099224	C5	-	Y	-	-	Y	-	-
ENSG00000112294	ALDH5A1	-0.387614976	1.63E-05	-0.2164357	0.039104	-0.189322048	0.057183	0.177778404	0.104966	0.212957093	0.037361	0.029829424	0.90074	C4	-	Y	-	-	-	-	-
ENSG00000112305	SMAP1	0.432490923	5.15E-05	0.359798926	0.002751	0.11337425	0.451316	-0.066426416	0.632609	-0.30446502	0.006118	-0.243542605	0.102519	C1	-	Y	Y	-	-	Y	-
ENSG00000112312	GMNN	-0.228198223	0.000637	-0.282856847	0.000341	-0.327627957	1.26E-05	-0.048255002	0.561305	-0.084877534	0.254053	-0.041918734	0.78435	C4	-	-	-	Y	-	-	-
ENSG00000112319	EYA4	-0.82350501	0.000762	-0.353303939	0.208475	-0.364580523	0.151718	0.473688446	0.113661	0.473610652	0.102087	-0.007626587	0.991437	C3	-	Y	-	-	-	-	-
ENSG00000112320	SOBP	0.076331365	0.645173	-0.084097795	0.694889	-0.268397216	0.113372	-0.154012902	0.30724	-0.330331181	0.019421	-0.181215177	0.460404	C5	-	-	-	-	-	Y	-
ENSG00000112357	PEX7	-0.826718876	6.48E-07	-0.177781782	0.478932	-0.366985634	0.071634	0.655216714	0.000566	0.474540974	0.018236	-0.185944406	0.601843	C3	-	Y	-	-	Y	Y	-
ENSG00000112367	FIG4	-0.328443133	0.000312	0.065981849	0.617188	-0.047654728	0.723006	0.401065268	1.00E-05	0.295487837	0.002139	-0.110810533	0.470464	C4	-	Y	-	-	Y	-	-
ENSG00000112394	SLC16A10	-0.091207897	0.759905	0.023512924	0.950203	0.389222957	0.072204	0.120423756	0.669133	0.494555485	0.018541	0.367990096	0.146968	C4	-	-	-	-	-	Y	-
ENSG00000112419	PHACTR2	-0.395057508	0.046781	-0.376535849	0.151392	-0.227984031	0.332291	0.025845158	0.944855	0.181602947	0.485357	0.150566352	0.755269	C2	-	Y	-	-	-	-	-
ENSG00000112425	EPM2A	-0.584793802	5.27E-07	-0.297870551	0.025125	-0.395155413	0.001513	0.293236466	0.018654	0.204187255	0.131262	-0.094372341	0.669912	C4	-	Y	-	Y	-	-	-
ENSG00000112531	QKI	0.447918169	4.24E-10	0.277055666	0.001838	0.110508444	0.314797	-0.164561903	0.003836	-0.322677931	2.47E-08	-0.163583425	0.077493	C5	-	Y	-	-	-	Y	-
ENSG00000112541	PDE10A	0.852304336	2.49E-11	0.48360583	0.001519	0.304572538	0.049933	-0.362411154	0.009835	-0.533133276	4.10E-05	-0.176124513	0.455865	C5	-	Y	Y	Y	Y	Y	-
ENSG00000112559	MDFI	-0.577222124	0.000138	-0.104566177	0.643043	-0.095399385	0.652806	0.47934442	0.000962	0.497198015	0.000475	0.012503342	0.974321	C4	-	Y	-	-	Y	Y	-
ENSG00000112576	CCND3	0.479565372	5.96E-08	0.094235791	0.579179	0.172792064	0.301274	-0.37880327	0.000271	-0.292008089	0.018754	0.082283536	0.787224	C5	-	Y	-	-	Y	-	-
ENSG00000112624	BICRAL	-0.305407325	0.000658	-0.083929928	0.549359	-0.040719158	0.746845	0.22797972	0.01872	0.279217076	0.000558	0.045951052	0.814705	C4	-	Y	-	-	-	-	-
ENSG00000112658	SRF	0.647247726	6.01E-25	0.142958021	0.209793	0.177418397	0.068663	-0.497566086	1.53E-10	-0.455417072	4.21E-10	0.038090986	0.874037	C5	-	Y	-	-	Y	Y	-
ENSG00000112667	DNPH1	-0.491178888	0.00398	-0.333140649	0.097711	-0.275916458	0.141183	0.164137986	0.314279	0.23080281	0.097581	0.061836898	0.824877	C4	-	Y	-	-	-	-	-
ENSG00000112679	DUSP22	0.404122721	0.001258	0.326015832	0.030629	0.394470103	0.005453	-0.071700842	0.669583	0.00529808	0.976632	0.071350136	0.801162	C5	-	Y	Y	Y	-	-	-
ENSG00000112699	GMDS	-0.580521438	0.00011	-0.059150959	0.81569	0.022764972	0.927338	0.528167216	0.001046	0.617968643	5.87E-05	0.084926044	0.793676	C4	-	Y	-	-	Y	Y	-
ENSG00000112715	VEGFA	-0.150536283	0.000285	0.091721555	0.066224	0.141949958	0.000935	0.248751162	1.89E-11	0.30719571	5.43E-19	0.053069108	0.395182	C4	-	-	-	-	-	Y	-
ENSG00000112739	PRPF4B	0.564059778	2.45E-21	0.105783426	0.235431	0.207314719	0.00218	-0.451743264	1.21E-24	-0.342333479	7.69E-20	0.104132125	0.125891	C5	-	Y	-	-	Y	Y	-
ENSG00000112763	BTN2A1	0.467217429	2.08E-08	0.072603505	0.627026	0.225300042	0.04	-0.387377346	5.09E-07	-0.227044206	0.004617	0.155319008	0.241505	C5	-	Y	-	-	Y	-	-
ENSG00000112769	LAMA4	-1.603896856	5.66E-10	-0.604530211	0.04868	-0.530948677	0.158602	1.009299435	0.003438	1.088141469	0.004828	0.073368395	NA	C3	-	Y	Y	-	Y	Y	-
ENSG00000112773	TENT5A	0.455393561	0.040807	-0.349053666	0.255292	-0.236644485	0.44496	-0.797191607	0.001015	-0.67709728	0.004742	0.113326546	0.856273	C5	-	Y	-	-	Y	Y	-
ENSG00000112855	HARS2	0.17775123	0.001157	-0.138499606	0.063707	-0.126255576	0.068704	-0.309590711	1.40E-08	-0.289090286	3.39E-08	0.015064163	0.928882	C5	-	-	-	-	Y	-	-
ENSG00000112902	SEMA5A	0.044508702	0.782631	-0.282776981	0.047233	-0.50188637	0.000468	-0.321052079	0.00329	-0.532669803	7.32E-06	-0.216523971	0.26805	C5	-	-	-	Y	Y	Y	-
ENSG00000112936	C7	-0.292694515	9.03E-18	0.109086995	0.273447	0.102802627	0.255763	0.40877579	2.91E-05	0.41023811	1.60E-05	-0.003789653	0.991966	C4	-	-	-	-	Y	Y	-
ENSG00000112941	TENT4A	0.343734764	1.73E-10	0.030890574	0.774441	0.087950906	0.221212	-0.306051151	1.97E-08	-0.240984029	9.16E-07	0.059729243	0.56513	C5	-	Y	-	-	Y	-	-
ENSG00000112972	HMGCS1	0.145873762	0.016564	0.341463091	0.006172	0.240498397	0.001185	0.200776544	0.260065	0.108947625	0.095485	-0.096930161	0.787224	C4	-	-	Y	-	-	-	-
ENSG00000112977	DAP	-0.298205991	1.35E-05	0.077858981	0.43984	-0.050272131	0.604905	0.382646337	7.78E-08	0.262660392	0.000168	-0.125148267	0.238004	C4	-	-	-	-	Y	-	-
ENSG00000112996	MRPS30	0.42327427	5.15E-07	-0.128172232	0.323106	0.027468288	0.845402	-0.545236829	3.88E-12	-0.381131993	7.28E-08	0.158814993	0.19895	C5	-	Y	-	-	Y	Y	-
ENSG00000113070	HBEGF	1.197970184	6.05E-16	0.747366921	3.15E-05	0.446898115	0.040253	-0.444474109	0.00238	-0.735856074	2.55E-06	-0.297517039	0.264599	C1	-	Y	Y	Y	Y	Y	-
ENSG00000113108	APBB3	0.095562336	0.498458	0.291130059	0.009241	0.457175544	1.59E-06	0.202234032	0.095736	0.376416785	0.000294	0.169028671	0.211699	C4	-	-	-	Y	-	Y	-
ENSG00000113140	SPARC	-0.323812143	8.42E-14	-0.139996505	0.018562	-0.180171574	0.004566	0.190164128	4.24E-05	0.158589162	0.002802	-0.037415371	0.757566	C4	-	Y	-	-	-	-	-
ENSG00000113163	CERT1	0.33707498	0.000103	0.267982529	0.002941	0.144832918	0.114921	-0.062789653	0.497179	-0.177947493	0.013768	-0.120769925	0.196376	C1	-	Y	-	-	-	-	-
ENSG00000113196	HAND1	-0.621532342	1.97E-20	-0.047447562	0.676162	-0.1956368	0.015849	0.580659456	1.52E-24	0.440721346	1.92E-14	-0.145258397	0.102737	C4	Y	Y	-	-	Y	Y	-
ENSG00000113212	PCDHB7	-0.982789958	0.021948	-0.231235945	0.686582	0.09591861	0.866204	0.757214801	0.116694	1.092729771	0.011964	0.329928024	NA	C2	-	Y	-	-	-	Y	-
ENSG00000113240	CLK4	0.49762882	1.10E-06	-0.335795359	0.032798	0.008998667	0.969138	-0.827181183	6.43E-15	-0.47475088	4.99E-06	0.347611531	0.063565	C5	-	Y	Y	-	Y	Y	-
ENSG00000113272	THG1L	-0.277258085	0.001768	-0.173454432	0.167077	-0.405099926	0.000145	0.11025079	0.318882	-0.112582227	0.318704	-0.228793852	0.128594	C4	-	-	-	Y	-	-	-
ENSG00000113296	THBS4	-0.524482439	0.038268	0.070898005	0.835278	-0.026728659	0.942501	0.601177783	0.011943	0.511762542	0.050845	-0.094542963	0.840232	C2	-	Y	-	-	Y	-	-
ENSG00000113319	RASGRF2	-0.037223364	0.912823	0.44138653	0.08859	0.302763291	0.256393	0.485309524	0.037384	0.354730898	0.14506	-0.135707188	0.754057	C4	-	-	-	-	Y	-	-
ENSG00000113361	CDH6	4.214672261	0.016483	2.250343674	NA	2.509919701	NA	-1.966948005	0.548726	-1.68600517	0.276766	0.253951976	NA	C5	-	Y	-	-	-	-	-
ENSG00000113368	LMNB1	-0.185977789	5.87E-05	-0.189221917	0.000419	-0.304553911	2.60E-09	0.003412232	0.954707	-0.103882556	0.011101	-0.11254792	0.040262	C4	-	-	-	Y	-	-	-
ENSG00000113369	ARRDC3	-0.097715206	0.135965	0.377407882	0.000239	0.175267841	0.097855	0.481708367	2.86E-08	0.288086877	0.000609	-0.199916974	0.472014	C4	-	-	Y	-	Y	-	-
ENSG00000113389	NPR3	3.797840337	0.03162	0.7411052	NA	2.883685881	NA	-3.047325967	NA	-0.900220716	0.568145	2.14274971	NA	C5	-	Y	-	-	-	-	-
ENSG00000113396	SLC27A6	-0.62220116	0.002673	0.087708848	0.783588	0.11655573	0.71424	0.716996316	0.000789	0.753608762	0.001174	0.030518155	0.95951	C4	-	Y	-	-	Y	Y	-
ENSG00000113441	LNPEP	0.316370167	0.003706	-0.115999976	0.57103	0.197493882	0.215454	-0.425593562	0.000774	-0.103665619	0.477188	0.316357266	0.125918	C5	-	Y	-	-	Y	-	-
ENSG00000113448	PDE4D	-0.387977515	0.00714	-0.098832436	0.65208	-0.231212472	0.216881	0.295269008	0.066507	0.171149545	0.367367	-0.128944658	0.674417	C4	-	Y	-	-	-	-	-
ENSG00000113460	BRIX1	0.030033274	0.715135	-0.189160612	0.031349	-0.32743397	7.14E-06	-0.212637971	0.000693	-0.342867852	3.71E-10	-0.135353901	0.200382	C5	-	-	-	Y	-	Y	-
ENSG00000113522	RAD50	0.267680876	0.117495	0.431784772	0.014335	0.290122827	0.113828	0.170266117	0.313337	0.036602999	0.86093	-0.138715048	0.597943	C1	-	-	Y	-	-	-	-
ENSG00000113578	FGF1	-1.294447193	0.00057	-0.593885023	0.14744	-0.5591901	0.156775	0.706347772	0.119916	0.75016516	0.095606	0.037535814	NA	C4	-	Y	-	-	-	-	-
ENSG00000113580	NR3C1	-0.461142601	0.100465	0.087208048	0.836899	0.251478203	0.395102	0.556328307	0.056925	0.727373046	0.004352	0.168106432	0.721721	C4	-	-	-	-	-	Y	-
ENSG00000113593	PPWD1	0.235726102	0.004872	-0.104850106	0.375386	-0.116486071	0.276204	-0.334221778	1.67E-05	-0.337583107	6.78E-06	-0.008738729	0.969793	C5	-	-	-	-	Y	Y	-
ENSG00000113595	TRIM23	0.500043093	1.41E-07	-0.273071551	0.048154	0.233566093	0.061211	-0.766913198	2.76E-15	-0.25175583	0.010106	0.509500942	5.09E-05	C5	Y	Y	-	-	Y	-	Y
ENSG00000113615	SEC24A	0.727923343	1.09E-19	0.215071969	0.140844	0.274293621	0.00722	-0.506050599	5.49E-08	-0.439076034	2.99E-10	0.061752941	0.790777	C5	Y	Y	-	-	Y	Y	-
ENSG00000113638	TTC33	0.590857701	1.65E-15	0.378187702	8.09E-05	0.451482197	1.18E-08	-0.206224016	0.012484	-0.12462135	0.104862	0.076183399	0.611286	C5	-	Y	Y	Y	-	-	-
ENSG00000113657	DPYSL3	-0.451689676	5.64E-06	-0.17801015	0.241243	-0.131879913	0.368299	0.280275832	0.028359	0.334554968	0.004702	0.048617444	0.879022	C4	-	Y	-	-	-	Y	-
ENSG00000113658	SMAD5	0.378556363	3.17E-07	0.121834631	0.274844	0.210063854	0.016536	-0.250364953	7.53E-06	-0.154178561	0.002244	0.090941323	0.366351	C5	-	Y	-	-	-	-	-
ENSG00000113721	PDGFRB	0.135827065	0.052874	0.31990625	7.35E-05	0.276054975	0.000218	0.1904978	0.012519	0.15500151	0.032845	-0.041232902	0.818037	C4	-	-	Y	-	-	-	-
ENSG00000113739	STC2	-1.239615572	4.69E-21	-0.039083307	0.857129	-0.129109029	0.447465	1.207690028	1.15E-27	1.125709025	9.04E-23	-0.087414297	0.661345	C3	-	Y	-	-	Y	Y	-
ENSG00000113742	CPEB4	0.361309406	1.22E-06	0.304263965	0.001146	0.230342833	0.004475	-0.050438534	0.586467	-0.116334674	0.073354	-0.071272667	0.587615	C1	-	Y	Y	-	-	-	-
ENSG00000113761	ZNF346	0.524845118	1.81E-06	0.20461464	0.10749	0.225227966	0.05684	-0.313592537	0.002287	-0.284671977	0.005137	0.023541037	0.923448	C5	-	Y	-	-	Y	-	-
ENSG00000113763	UNC5A	-0.490641382	2.37E-14	0.130883627	0.145543	0.104426012	0.234353	0.628100184	3.77E-22	0.610052724	1.50E-22	-0.023636074	0.898232	C2	-	Y	-	-	Y	Y	-
ENSG00000113790	EHHADH	-0.557882413	2.37E-05	-0.209455293	0.187946	-0.097843349	0.556138	0.354923605	0.01087	0.474399664	0.000177	0.114948459	0.593965	C4	-	Y	-	-	Y	Y	-

ENSG00000113805	CNTN3	-2.122108452	0.02257	0.052803493	0.967013	0.327039098	0.721104	2.180684054	0.009243	2.460814685	0.00144	0.283513727	NA	C3	-	Y	-	-	Y	Y	-
ENSG00000113812	ACTR8	0.364986581	1.78E-06	0.056505467	0.639367	0.159090288	0.098037	-0.302135987	2.51E-05	-0.191019897	0.014008	0.105667074	0.40175	C5	-	Y	-	-	Y	-	-
ENSG00000113838	TBCCD1	0.404553719	0.002949	0.009677315	0.972722	0.191972049	0.262298	-0.390217725	0.000693	-0.198463751	0.072261	0.186346392	0.315247	C1	-	Y	-	-	Y	-	-
ENSG00000113845	TIMMDC1	-0.521761533	2.27E-28	-0.158495048	0.01032	-0.107590382	0.102686	0.369840169	1.46E-13	0.428842481	5.73E-18	0.05386343	0.610211	C4	-	Y	-	-	Y	Y	-
ENSG00000113851	CRBN	0.312982378	8.11E-06	0.102049906	0.292833	0.039488681	0.727036	-0.20472077	0.002766	-0.258759048	0.00013	-0.05948486	0.658238	C5	-	Y	-	-	-	-	-
ENSG00000113971	NPHP3	0.336462581	0.016384	-0.059114153	0.837945	0.269723718	0.144018	-0.388921392	0.018888	-0.05146382	0.802094	0.331379605	0.200061	C5	-	Y	-	-	Y	-	-
ENSG00000114023	FAM162A	-0.303806859	4.00E-05	0.302882696	0.007314	0.180651139	0.067344	0.613227605	1.50E-12	0.499592083	2.22E-12	-0.119284094	0.489604	C4	-	Y	Y	-	Y	Y	-
ENSG00000114030	KPNA1	0.357018552	1.17E-10	0.096233202	0.240998	0.095793592	0.206105	-0.25445401	1.32E-06	-0.246520707	1.41E-06	0.002514759	0.98835	C5	-	Y	-	-	-	-	-
ENSG00000114107	CEP70	-0.319687792	0.001548	-0.107249182	0.560902	-0.055255615	0.695873	0.218844323	0.09422	0.279242234	0.00308	0.054988046	0.835773	C4	-	Y	-	-	-	-	-
ENSG00000114127	XRN1	0.652590605	6.51E-11	0.303341332	0.016124	0.293921514	0.004682	-0.342868463	8.96E-05	-0.344599034	8.51E-07	-0.006972549	0.976568	C1	-	Y	Y	-	Y	Y	-
ENSG00000114200	BCHE	-0.859342851	3.27E-14	-0.211405368	0.10126	-0.094969244	0.575261	0.65509401	1.26E-07	0.779113024	3.35E-09	0.118412835	0.613179	C4	-	Y	-	-	Y	Y	-
ENSG00000114251	WNT5A	-0.83325833	0.321655	0.161145762	NA	0.80870234	0.239493	1.003303382	0.268223	1.656023113	0.015979	0.651863379	NA	C2	-	-	-	-	-	Y	-
ENSG00000114268	PFKFB4	-0.068203013	0.738297	0.396418957	0.03633	0.36730512	0.025909	0.470829628	0.002745	0.450338876	0.001358	-0.025992541	0.949938	C4	-	-	Y	Y	Y	Y	-
ENSG00000114270	COL7A1	0.669102506	0.000859	0.533116868	0.036588	0.525769754	0.022152	-0.12924696	0.632151	-0.128266024	0.613033	-0.004871976	0.993919	C1	-	Y	Y	Y	-	-	-
ENSG00000114302	PRKAR2A	0.332496179	9.68E-06	0.283840868	0.001753	0.152547812	0.105502	-0.042821427	0.606069	-0.165456799	0.008733	-0.128021404	0.193422	C1	-	Y	-	-	-	-	-
ENSG00000114315	HES1	-0.058819693	0.896016	0.696194348	0.037701	0.367152982	0.30951	0.762117763	0.006449	0.441491057	0.142603	-0.325779181	0.454595	C2	-	-	Y	-	Y	-	-
ENSG00000114316	USP4	0.324673048	1.22E-09	0.111432006	0.132815	0.143319113	0.048486	-0.20679362	2.43E-05	-0.166891568	0.001823	0.034648063	0.778966	C5	-	Y	-	-	-	-	-
ENSG00000114331	ACAP2	0.423697338	1.36E-10	0.292755868	0.00104	0.280004827	0.000328	-0.124610217	0.111067	-0.128853991	0.056537	-0.009806685	0.962837	C1	-	Y	-	-	-	-	-
ENSG00000114395	CYB561D2	-0.431369706	0.010556	-0.147472853	0.488874	-0.194000867	0.31809	0.290680697	0.098448	0.252371667	0.167664	-0.043781985	0.906331	C4	-	Y	-	-	-	-	-
ENSG00000114423	CBLB	0.273829803	0.005477	-0.05449413	0.74261	0.162742544	0.182116	-0.321654985	2.45E-05	-0.096337698	0.286815	0.219816846	0.04232	C5	-	-	-	-	Y	-	-
ENSG00000114473	IQCG	-0.209943855	0.098734	0.065356439	0.739384	0.093029477	0.557224	0.2821574	0.035658	0.317665335	0.009496	0.030500607	0.926363	C4	-	-	-	-	-	Y	-
ENSG00000114648	KLHL18	0.497191164	1.63E-07	0.226324703	0.075702	0.206255778	0.050127	-0.264457628	0.009448	-0.276412008	0.001407	-0.017013026	0.947133	C1	-	Y	-	-	-	-	-
ENSG00000114670	NEK11	-1.05555771	0.000438	-0.028121583	0.957171	-0.088445763	0.859515	1.032279709	0.001076	0.98292964	0.005473	-0.054968043	0.949104	C3	-	Y	-	-	Y	Y	-
ENSG00000114735	HEMK1	-0.506584916	1.28E-13	0.104410821	0.241262	0.142816588	0.072953	0.617478415	9.44E-23	0.664191228	1.95E-26	0.041396802	0.752336	C2	-	Y	-	-	Y	Y	-
ENSG00000114738	MAPKAPK3	-0.343741425	0.002944	-0.049406303	0.792142	0.047247441	0.763831	0.300791497	0.020525	0.40577275	0.000426	0.099408068	0.641419	C4	-	Y	-	-	Y	Y	-
ENSG00000114742	WDR48	0.300520569	3.89E-09	0.028973246	0.798232	0.01285401	0.894487	-0.265227889	1.10E-06	-0.273154769	2.69E-10	-0.013110784	0.936382	C5	-	Y	-	-	-	-	-
ENSG00000114745	GORASP1	0.421810965	1.24E-08	0.154089821	0.150467	0.209874336	0.023835	-0.261252412	0.001197	-0.197488046	0.013844	0.058774062	0.745702	C5	-	Y	-	-	-	-	-
ENSG00000114779	ABHD14B	-0.722130568	3.27E-10	0.060212971	0.731342	0.079817513	0.630044	0.789272439	1.56E-11	0.816735293	1.38E-11	0.02233504	0.943467	C2	-	Y	-	-	Y	Y	-
ENSG00000114790	ARHGEF26	-0.583322988	0.012997	-0.181339773	0.568233	0.011208272	0.976394	0.408500427	0.139426	0.609221903	0.016956	0.195309634	0.653466	C4	-	Y	-	-	-	Y	-
ENSG00000114805	PLCH1	-0.718465779	0.037291	-0.763111117	0.055823	-0.113654701	0.801694	-0.039507435	0.949956	0.618948584	0.099442	0.652032268	NA	C4	-	Y	-	-	-	-	-
ENSG00000114812	VIPR1	-0.977669113	0.132605	-1.336809442	0.049986	-1.162840629	0.082964	-0.350165452	0.722016	-0.170942967	0.870631	0.175438159	NA	C3	-	-	Y	-	-	-	-
ENSG00000114841	DNAH1	0.636883657	1.21E-05	0.473862004	0.010908	0.309105721	0.09553	-0.156228826	0.330348	-0.31315335	0.023031	-0.162420502	0.518796	C1	-	Y	Y	-	-	Y	-
ENSG00000114853	ZBTB47	-0.472846455	0.001124	-0.193597148	0.320588	-0.199107299	0.232264	0.28530475	0.117074	0.288520606	0.087942	-0.002501416	0.995987	C4	-	Y	-	-	-	-	-
ENSG00000114857	NKTR	0.311896447	5.13E-05	0.188526428	0.064061	0.105797267	0.318653	-0.117045226	0.059246	-0.191520604	0.000277	-0.079874538	0.474909	C1	-	Y	-	-	-	-	-
ENSG00000114861	FOXP1	-0.232835298	0.011638	0.078168236	0.604345	-0.134286883	0.254181	0.317470857	0.002102	0.113280771	0.324767	-0.209349602	0.168713	C4	-	-	-	-	Y	-	-
ENSG00000114902	SPCS1	-0.327115913	3.85E-06	-0.128564818	0.170905	-0.197204446	0.015278	0.205442116	0.002337	0.144824479	0.03643	-0.065999482	0.594047	C4	-	Y	-	-	-	-	-
ENSG00000114904	NEK4	0.99201012	2.23E-20	0.299909919	0.042744	0.353981567	0.012077	-0.685088627	6.71E-20	-0.623824989	2.41E-15	0.056164574	0.787719	C5	-	Y	-	Y	Y	Y	-
ENSG00000114923	SLC4A3	0.109314771	0.265041	-0.162035911	0.176495	0.137885159	0.188113	-0.265078149	0.003795	0.043270581	0.697209	0.302776051	0.005539	C5	-	-	-	-	-	-	Y
ENSG00000114933	INO80D	0.234392485	0.146342	-0.141485106	0.487754	0.019251235	0.939542	-0.369250227	0.008911	-0.20014154	0.201484	0.163570515	0.501333	C5	-	-	-	-	Y	-	-
ENSG00000114948	ADAM23	-0.275053049	0.000385	0.146399702	0.133239	0.054572456	0.61232	0.427987163	2.05E-10	0.344285002	9.02E-08	-0.08897675	0.423104	C4	-	-	-	-	Y	Y	-
ENSG00000114978	MOB1A	0.208982235	0.005013	-0.118113592	0.233041	0.0479922	0.631596	-0.320832785	1.84E-07	-0.146609519	0.009225	0.16911501	0.027176	C5	-	-	-	-	Y	-	-
ENSG00000114988	LMAN2L	0.24579894	0.033021	0.254062678	0.054	0.316655486	0.011047	0.014513891	0.931259	0.085234281	0.540457	0.065374118	0.787696	C5	-	-	-	Y	-	-	-
ENSG00000115020	PIKFYVE	0.647553347	9.31E-17	0.319854527	0.000823	0.289277171	0.002136	-0.321484751	1.07E-06	-0.343565176	8.09E-08	-0.027593352	0.884209	C1	-	Y	Y	-	Y	Y	-
ENSG00000115084	SLC35F5	0.423325493	2.18E-10	0.117131906	0.223745	0.202015944	0.005554	-0.299624011	3.72E-05	-0.206619233	0.002139	0.087786582	0.474162	C5	-	Y	-	-	-	-	-
ENSG00000115107	STEAP3	-0.344960632	0.011478	-0.064552511	0.765825	-0.109690811	0.532132	0.286595557	0.062904	0.249974671	0.102313	-0.042166986	0.901698	C4	-	Y	-	-	-	-	-
ENSG00000115109	EPB41L5	0.321529055	1.64E-08	0.019488406	0.867222	0.156028435	0.018809	-0.29547317	1.44E-10	-0.151002801	0.000281	0.139148791	0.028724	C5	-	Y	-	-	-	-	-
ENSG00000115129	TP53I3	-0.731089121	1.07E-14	-0.327228059	0.004293	-0.312504232	0.007294	0.410637193	0.000107	0.433760983	6.12E-05	0.017720618	0.953134	C4	Y	Y	Y	Y	Y	Y	-
ENSG00000115194	SLC30A3	-0.507939735	0.002838	-0.498688595	0.006581	-0.277716139	0.178805	0.015789999	0.956789	0.245142644	0.274196	0.223937372	0.492723	C4	-	Y	Y	-	-	-	-
ENSG00000115211	EIF2B4	0.46124664	6.05E-16	0.030859747	0.786065	0.0401488	0.704881	-0.42371788	4.40E-14	-0.406531218	2.93E-12	0.012196907	0.948661	C5	-	Y	-	-	Y	Y	-
ENSG00000115255	REEP6	-0.412979657	0.035873	-0.330018424	0.253763	-0.192120337	0.459522	0.088033093	0.751219	0.235679267	0.223357	0.142235396	0.73559	C4	-	Y	-	-	-	-	-
ENSG00000115266	APC2	-0.127819767	0.010241	-0.053369381	0.422498	0.164763378	0.000612	0.080940754	0.129431	0.307234917	3.31E-13	0.220998522	9.76E-06	C4	-	-	-	-	-	Y	-
ENSG00000115271	GCA	-0.73660091	1.79E-12	-0.33289936	0.00563	-0.120051948	0.345158	0.411312214	0.001268	0.631357174	1.08E-08	0.215639032	0.182394	C4	-	Y	Y	-	Y	Y	-
ENSG00000115275	MOGS	0.310458432	9.14E-07	0.225496265	0.003588	0.199816031	0.015031	-0.078507059	0.284941	-0.095547517	0.199562	-0.022631618	0.898232	C5	Y	Y	-	-	-	-	-
ENSG00000115290	GRB14	-1.065790852	0.087021	0.670966794	0.10317	0.581901243	0.264355	1.746569244	0.000734	1.662468815	0.004688	-0.087097281	NA	C2	-	-	-	-	Y	Y	-
ENSG00000115307	AUP1	0.217864266	0.000404	0.018306884	0.874481	-0.157879804	0.038949	-0.193137012	0.000124	-0.361203825	2.95E-13	-0.173374479	0.013434	C5	-	-	-	-	-	Y	-
ENSG00000115317	HTRA2	0.334090128	2.45E-05	0.102594298	0.385383	0.065318671	0.640335	-0.224933954	0.00639	-0.253983836	0.004742	-0.034131366	0.884098	C5	-	Y	-	-	-	-	-
ENSG00000115363	EVA1A	1.080491793	0.000181	1.629386465	3.51E-09	1.325435596	4.20E-06	0.555619688	0.024639	0.260082487	0.37236	-0.301084059	0.428023	C1	-	Y	Y	Y	Y	-	-
ENSG00000115368	WDR75	0.302689241	1.16E-05	-0.081532046	0.427253	0.033187689	0.773497	-0.377769871	9.22E-16	-0.255121784	2.53E-07	0.117318519	0.128569	C5	-	Y	-	-	Y	-	-
ENSG00000115414	FN1	0.168560478	0.069027	0.945415911	7.02E-07	1.008249261	2.09E-09	0.783641316	5.62E-06	0.854348546	2.46E-08	0.065257427	0.904853	C4	-	-	Y	Y	Y	Y	-
ENSG00000115419	GLS	0.562420195	5.93E-27	0.085386034	0.348269	0.181211842	0.003553	-0.4705493	6.36E-21	-0.36649915	1.48E-19	0.098669623	0.250087	C5	-	Y	-	-	Y	Y	-
ENSG00000115421	PAPOLG	0.327561263	0.000261	-0.291155681	0.009637	0.01149916	0.944023	-0.611887116	1.92E-14	-0.301535449	0.000142	0.304990966	0.004434	C5	-	Y	-	-	Y	Y	Y
ENSG00000115425	PECR	-0.309538943	0.067488	-0.03955125	0.882192	0.060431384	0.809116	0.276351801	0.104018	0.384978495	0.022108	0.103144244	0.737836	C4	-	-	-	-	-	Y	-
ENSG00000115457	IGFBP2	-0.325168067	3.47E-06	-0.197385878	0.041103	-0.193786721	0.02439	0.134304771	0.033298	0.146305087	0.005629	0.006652095	0.97113	C4	-	Y	-	-	-	-	-
ENSG00000115459	ELMOD3	0.678057203	6.16E-09	0.324057673	0.039499	0.525515613	5.72E-05	-0.34779662	0.007331	-0.138125354	0.324648	0.204390268	0.322231	C5	-	Y	Y	Y	Y	-	-
ENSG00000115461	IGFBP5	-1.002030043	4.84E-69	-0.130904836	0.054502	-0.084653316	0.255642	0.877584846	2.98E-72	0.931927901	5.06E-83	0.049121384	0.617535	C2	-	Y	-	-	Y	Y	-
ENSG00000115464	USP34	0.530266692	0.000233	0.298177931	0.111537	0.332180186	0.064511	-0.225881587	0.000413	-0.18352955	0.004248	0.036888516	0.83368	C1	-	Y	-	-	-	-	-
ENSG00000115520	COQ10B	0.287635652	0.018458	-0.161608907	0.343678	0.042093436	0.831744	-0.442734108	0.000315	-0.230943203	0.075295	0.206632679	0.295679	C5	-	-	-	-	Y	-	-

ENSG00000115525	ST3GAL5	-0.121193166	0.446584	0.214882504	0.18896	0.252709849	0.07792	0.342671413	0.010375	0.388683953	0.002002	0.041189928	0.889834	C4	-	-	-	-	Y	Y	-
ENSG00000115548	KDM3A	0.189091319	0.011882	0.301733649	0.000898	0.117578585	0.247054	0.118685064	0.103171	-0.056755095	0.453372	-0.181188375	0.083858	C4	-	-	Y	-	-	-	-
ENSG00000115556	PLCD4	-0.421432373	5.82E-10	-0.02894673	0.808977	0.016947021	0.90072	0.399030198	4.84E-10	0.453389765	2.15E-10	0.048916013	0.752014	C4	-	Y	-	-	Y	Y	-
ENSG00000115568	ZNF142	0.400475619	1.12E-05	-0.008571232	0.962107	0.091779934	0.504031	-0.403115509	9.49E-09	-0.294590925	0.000159	0.103164019	0.46238	C5	-	Y	-	-	Y	-	-
ENSG00000115641	FHL2	0.942667693	1.26E-09	0.434202308	0.045895	0.165425475	0.514795	-0.50315603	0.001834	-0.762792347	6.82E-07	-0.265453718	0.352975	C5	-	Y	Y	-	Y	Y	-
ENSG00000115652	UXS1	0.293346661	0.003975	0.340581454	0.000834	0.10581799	0.421492	0.053513948	0.655981	-0.172812037	0.093317	-0.231813399	0.046184	C4	-	-	Y	-	-	-	-
ENSG00000115657	ABCB6	0.080182143	0.467093	-0.329628059	0.000979	-0.178963074	0.097855	-0.403475037	9.15E-06	-0.244285217	0.010767	0.153628879	0.295807	C4	-	-	Y	-	Y	-	-
ENSG00000115738	ID2	-0.024148588	0.730386	0.291501289	7.73E-05	-0.039397811	0.674401	0.322198547	2.42E-10	-0.000285905	0.996017	-0.327945577	1.67E-06	C4	Y	-	-	-	Y	-	Y
ENSG00000115756	HPCAL1	0.230934808	0.010284	0.032113583	0.835278	-0.152636953	0.143938	-0.191584639	0.004916	-0.368678731	1.02E-09	-0.182515921	0.029608	C5	-	-	-	-	-	Y	-
ENSG00000115758	ODC1	-0.49768275	2.34E-22	-0.26209161	0.000142	-0.27449806	3.65E-06	0.242224793	5.95E-08	0.237904545	3.70E-11	-0.009602638	0.943834	C4	Y	Y	-	-	-	-	-
ENSG00000115760	BIRC6	0.35636124	0.014723	0.17954959	0.402626	0.224825287	0.15988	-0.17032015	0.191205	-0.11734874	0.060601	0.048506614	0.765267	C5	-	Y	-	-	-	-	-
ENSG00000115762	PLEKHB2	0.365220935	1.57E-13	0.157565554	0.006629	0.191476074	0.002884	-0.20120284	2.04E-05	-0.159113021	0.003289	0.036819701	0.753107	C5	-	Y	-	-	-	-	-
ENSG00000115808	STRN	0.451895538	4.19E-10	0.278476321	0.00459	0.214754656	0.012786	-0.166694729	0.008835	-0.222548685	3.22E-06	-0.061199537	0.620799	C1	-	Y	-	-	-	-	-
ENSG00000115827	DCAF17	0.525164361	8.20E-13	0.006392502	0.969933	0.166048875	0.069849	-0.512467596	4.69E-14	-0.344577013	1.35E-08	0.162544634	0.1223	C5	-	Y	-	-	Y	Y	-
ENSG00000115839	RAB3GAP1	0.376566581	7.53E-08	0.156084783	0.054665	0.063974274	0.498547	-0.214080888	0.001558	-0.297929617	4.51E-06	-0.089270399	0.397429	C5	-	Y	-	-	-	-	-
ENSG00000115841	RMDN2	-0.402812226	0.00631	-0.13486003	0.460998	-0.39031014	0.005995	0.275282079	0.116102	0.026725596	0.911786	-0.253360776	0.251933	C3	-	Y	-	Y	-	-	-
ENSG00000115866	DARS1	-0.250495736	1.15E-05	0.082462535	0.289322	-0.02956587	0.743528	0.339296135	1.09E-11	0.23579567	4.68E-06	-0.108933587	0.149961	C4	-	-	-	-	Y	-	-
ENSG00000115884	SDC1	-0.390118025	2.49E-12	-0.055616373	0.565203	-0.170239766	0.024817	0.341117609	2.44E-11	0.234478131	1.74E-05	-0.111786065	0.238478	C4	-	Y	-	-	Y	-	-
ENSG00000115902	SLC1A4	-0.815461653	5.33E-17	-0.654625101	3.52E-10	-0.285757474	0.006694	0.167415397	0.167091	0.544625645	5.50E-09	0.371474558	0.001238	C3	-	Y	Y	-	-	Y	Y
ENSG00000115904	SOS1	0.494332679	1.05E-13	0.202756394	0.033462	0.28827694	0.000286	-0.284950213	4.54E-07	-0.191358782	0.000163	0.088250389	0.43492	C5	-	Y	-	-	-	-	-
ENSG00000115942	ORC2	0.400582472	3.77E-11	-0.066070388	0.59149	0.023258211	0.832334	-0.460190144	4.28E-12	-0.362670864	2.07E-11	0.092212044	0.483592	C5	-	Y	-	-	Y	Y	-
ENSG00000115963	RND3	0.018827707	0.836055	-0.380742046	4.35E-05	-0.208568527	0.009824	-0.392826719	6.19E-07	-0.212717023	0.002071	0.175202551	0.191951	C5	-	-	Y	-	Y	-	-
ENSG00000115966	ATF2	0.360040179	7.47E-08	0.051308217	0.654213	0.128695725	0.13599	-0.303037234	2.76E-10	-0.216740213	6.08E-06	0.080759504	0.356763	C5	-	Y	-	-	Y	-	-
ENSG00000115977	AAK1	0.419748699	1.62E-07	0.294374078	0.003358	0.299697399	0.000328	-0.118892736	0.168602	-0.105579437	0.140472	0.008156332	0.969121	C5	-	Y	-	-	-	-	-
ENSG00000115993	TRAK2	0.428946444	5.67E-09	0.376408304	9.44E-06	0.429929859	1.67E-08	-0.046671894	0.595504	0.015665146	0.864739	0.056930366	0.667843	C1	-	Y	Y	Y	-	-	-
ENSG00000115998	C2orf42	0.022543929	0.911586	-0.646516536	0.000228	-0.143559324	0.441446	-0.662974402	6.47E-05	-0.151483723	0.379781	0.505978017	0.020798	C5	-	-	Y	-	Y	-	Y
ENSG00000116016	EPAS1	-0.584708343	1.44E-23	-0.506990242	1.98E-18	-0.451234895	2.54E-14	0.084329676	0.287808	0.148219108	0.037792	0.058618174	0.633079	C4	-	Y	Y	Y	-	-	-
ENSG00000116017	ARID3A	0.401602838	3.36E-05	0.23028747	0.03937	0.345121357	0.001447	-0.165051619	0.111861	-0.042007173	0.757123	0.117641607	0.488316	C5	-	Y	-	Y	-	-	-
ENSG00000116031	CD207	-0.840283698	0.04234	0.196681807	0.703255	0.329657028	0.45559	1.04233571	0.007539	1.183856235	0.002142	0.13574989	NA	C2	-	Y	-	-	Y	Y	-
ENSG00000116044	NFE2L2	0.220072754	0.006674	-0.152942105	0.091535	0.068874272	0.534106	-0.366623892	2.00E-07	-0.136528947	0.093491	0.22469817	0.012564	C5	-	-	-	-	Y	-	-
ENSG00000116062	MSH6	-0.190334794	2.45E-08	-0.31213552	1.28E-10	-0.310190771	1.12E-12	-0.115114366	0.006479	-0.105328248	0.005872	0.004868855	0.972936	C4	-	-	Y	Y	-	-	-
ENSG00000116095	PLEKHA3	0.220195874	0.024982	-0.122407528	0.379041	0.079181777	0.59657	-0.336213776	0.000607	-0.126439646	0.275725	0.204449741	0.190825	C5	-	-	-	-	Y	-	-
ENSG00000116096	SPR	-0.318610786	0.034063	0.002452485	0.992467	-0.081942315	0.695368	0.327122429	0.03543	0.251389657	0.143879	-0.081335015	0.790777	C4	-	Y	-	-	Y	-	-
ENSG00000116133	DHCR24	0.312726623	8.56E-09	0.194016583	0.148279	0.37217879	0.000199	-0.113580053	0.535058	0.073927516	0.657367	0.182759198	0.607798	C1	-	Y	-	Y	-	-	-
ENSG00000116138	DNAJC16	0.453851671	2.42E-06	0.202128552	0.123043	0.288743553	0.007166	-0.245016528	0.007133	-0.150389176	0.093833	0.089018768	0.586559	C1	-	Y	-	-	-	-	-
ENSG00000116147	TNR	0.351778953	0.041257	0.195752687	0.168049	0.394375175	0.042093	-0.149940909	0.024329	0.056134954	0.513869	0.200963613	0.036324	C5	-	Y	-	Y	-	-	-
ENSG00000116151	MORN1	-0.723182527	7.06E-07	0.02129551	0.93442	0.077211602	0.70999	0.751239008	7.61E-07	0.814904121	4.00E-08	0.058789193	0.861463	C4	-	Y	-	-	Y	Y	-
ENSG00000116157	GPX7	-0.401172136	0.000863	-0.202783913	0.151785	-0.20673327	0.132867	0.205104728	0.139812	0.209324934	0.136571	-0.001068545	0.997282	C4	-	Y	-	-	-	-	-
ENSG00000116198	CEP104	1.160439974	5.62E-40	0.616118183	6.33E-09	0.57407138	7.67E-09	-0.537478306	6.43E-15	-0.571932322	5.18E-19	-0.039868649	0.826205	C1	-	Y	Y	Y	Y	Y	-
ENSG00000116205	TCEANC2	0.585990855	4.31E-12	0.379630924	5.20E-05	0.448675722	6.81E-07	-0.200048825	0.022899	-0.122989445	0.188698	0.071954729	0.656136	C5	-	Y	Y	Y	-	-	-
ENSG00000116254	CHD5	-0.1246837	0.161007	-0.2841175	0.000908	-0.358886325	5.22E-06	-0.153107409	0.081191	-0.219554631	0.007303	-0.072001322	0.631537	C4	-	-	-	Y	-	-	-
ENSG00000116266	STXBP3	0.36625445	2.05E-05	0.171535286	0.117941	0.147218177	0.166776	-0.18835792	0.050432	-0.204366934	0.028541	-0.021405422	0.930556	C5	-	Y	-	-	-	-	-
ENSG00000116273	PHF13	0.131464204	0.153921	-0.230735116	0.025125	-0.08526579	0.491645	-0.356108714	2.21E-07	-0.202472646	0.005953	0.148328791	0.200135	C5	-	-	-	-	Y	-	-
ENSG00000116285	ERRFI1	0.700645534	2.16E-15	0.13559018	0.346014	0.209707194	0.052307	-0.558319863	3.61E-09	-0.476100152	1.82E-08	0.076647358	0.701064	C5	-	Y	-	-	Y	Y	-
ENSG00000116299	ELAPOR1	-0.516519166	0.039357	-0.608584519	0.01938	-0.191285579	0.539555	-0.085649097	0.819861	0.340373061	0.246606	0.420524736	0.256323	C3	-	Y	Y	-	-	-	-
ENSG00000116353	MECR	-0.494391812	8.37E-06	-0.214702777	0.132148	-0.205185888	0.120841	0.286197683	0.021648	0.304133802	0.01144	0.012434823	0.96897	C4	-	Y	-	-	-	Y	-
ENSG00000116406	EDEM3	0.765590065	1.20E-28	0.454595265	1.41E-08	0.48112954	1.27E-10	-0.304374402	1.12E-09	-0.269939724	1.19E-08	0.029054576	0.821705	C5	Y	Y	Y	Y	Y	-	-
ENSG00000116497	S100PBP	0.409345855	8.78E-11	0.240516454	0.004653	0.439956775	5.31E-11	-0.162117109	0.019579	0.045257157	0.549994	0.202072931	0.018507	C4	-	Y	-	Y	-	-	-
ENSG00000116539	ASH1L	0.481148839	2.94E-08	0.420912966	1.17E-05	0.315478864	0.000516	-0.054209256	0.476836	-0.151596139	0.004387	-0.102395119	0.226073	C1	-	Y	Y	Y	-	-	-
ENSG00000116544	DLGAP3	0.309500096	0.005118	-0.090450982	0.615259	-0.047524336	0.798532	-0.39356372	0.000533	-0.342222392	0.003062	0.045881513	0.879183	C5	-	Y	-	-	Y	Y	-
ENSG00000116560	SFPQ	-0.365347994	8.94E-23	-0.132537658	0.00042	-0.259778131	2.19E-14	0.239302067	3.99E-13	0.120254715	0.000362	-0.124400016	0.000343	C4	Y	Y	-	-	-	-	-
ENSG00000116580	GON4L	0.41442067	1.77E-09	0.285429723	0.000526	0.196262606	0.028647	-0.122021472	0.010614	-0.203953506	7.00E-05	-0.086748748	0.37639	C1	-	Y	-	-	-	-	-
ENSG00000116584	ARHGEF2	0.303156603	1.09E-07	0.167840393	0.020864	0.24665195	0.000185	-0.128880818	0.012171	-0.041918598	0.498069	0.081586869	0.346307	C5	-	Y	-	-	-	-	-
ENSG00000116586	LAMTOR2	-0.40963027	9.37E-06	-0.325148321	0.007996	-0.249759958	0.022241	0.091162366	0.37332	0.174537246	0.017625	0.078315376	0.641821	C4	-	Y	Y	-	-	-	-
ENSG00000116604	MEF2D	0.343263404	1.11E-06	0.163469066	0.055097	0.216689358	0.00698	-0.173335567	0.008904	-0.111846784	0.122433	0.056092774	0.652484	C5	-	Y	-	-	-	-	-
ENSG00000116652	DLEU2L	-0.102737673	0.863036	0.88245253	0.028633	0.348787855	0.52121	0.991941212	0.01079	0.465910993	0.35279	-0.531194674	NA	C2	-	-	Y	-	Y	-	-
ENSG00000116661	FBXO2	-0.599511124	0.02427	-0.190691764	0.621954	-0.222570625	0.496922	0.416304186	0.183396	0.391775564	0.189401	-0.029124092	0.967582	C2	-	Y	-	-	-	-	-
ENSG00000116667	C1orf21	0.197523803	0.000455	0.457780262	2.75E-13	0.21319777	0.000604	0.266715654	1.44E-07	0.030251743	0.610072	-0.241747668	0.000154	C4	-	-	Y	-	-	-	-
ENSG00000116678	LEPR	0.66093865	5.79E-06	0.095206663	0.791722	0.171510566	0.491525	-0.560358973	0.003334	-0.474357701	0.002897	0.078370233	0.8863	C5	-	Y	-	-	Y	Y	-
ENSG00000116691	MIIP	0.39437317	0.004036	0.14401256	0.476799	0.24130661	0.123033	-0.243313941	0.034201	-0.137687626	0.231154	0.099892594	0.603061	C5	-	Y	-	-	-	-	-
ENSG00000116698	SMG7	0.307663058	3.54E-08	0.055947009	0.580028	0.05676019	0.487148	-0.245517288	4.84E-07	-0.236616368	4.96E-10	0.003813362	0.980179	C5	Y	Y	-	-	-	-	-
ENSG00000116701	NCF2	-1.333216126	0.52622	2.171346582	NA	2.188728611	NA	3.509175148	0.004395	3.533512359	0.004869	0.024876101	NA	C2	-	-	-	-	Y	Y	-
ENSG00000116704	SLC35D1	0.624598799	6.59E-07	0.040331652	0.87029	0.280327364	0.069292	-0.577649621	3.66E-10	-0.330491166	0.000283	0.241964146	0.080013	C5	Y	Y	-	-	Y	Y	-
ENSG00000116729	WLS	-0.55896042	8.53E-08	-0.400149395	0.012339	-0.337663555	0.009463	0.165216734	0.336094	0.235824713	0.077533	0.065847734	0.855213	C4	-	Y	Y	Y	-	-	-
ENSG00000116741	RGS2	-0.411807853	0.002749	-0.435637912	0.01293	-0.441064119	0.004626	-0.017831891	0.94317	-0.014536212	0.948391	-0.001808856	0.997282	C4	-	Y	Y	Y	-	-	-
ENSG00000116747	RO60	0.319115052	0.00142	-0.173532934	0.152394	-0.083854281	0.503408	-0.486708532	6.62E-11	-0.388770162	1.25E-08	0.09307285	0.411644	C5	-	Y	-	-	Y	Y	-
ENSG00000116761	CTH	-0.404573353	0.041639	-0.30208599	0.227796	-0.451416313	0.028891	0.108495274	0.694905	-0.032327739	0.913815	-0.146623371	0.715621	C4	-	Y	-	Y	-	-	-

ENSG00000116774	OLFML3	-1.230174343	5.61E-07	-0.388329473	0.220423	-0.759872204	0.021897	0.851360407	0.006794	0.483743734	0.236983	-0.375593858	0.541709	C3	-	Y	-	Y	Y	-	-
ENSG00000116791	CRYZ	-0.2784461	0.003816	-0.060565284	0.671182	0.083908649	0.453782	0.223807692	0.031472	0.377017502	2.24E-05	0.147171013	0.272541	C4	-	-	-	-	-	Y	-
ENSG00000116852	KIF21B	-1.023178002	7.29E-11	-0.403781052	0.048656	0.02923644	0.889326	0.629023402	0.004805	1.066780323	3.47E-11	0.434828227	0.075643	C4	-	Y	Y	-	Y	Y	-
ENSG00000116857	TMEM9	-0.38418377	5.01E-07	-0.168636069	0.090919	-0.179231118	0.046286	0.222870935	0.000269	0.220095765	0.000116	-0.008344544	0.959537	C4	Y	Y	-	-	-	-	-
ENSG00000116903	EXOC8	0.106271274	0.437782	-0.474604692	0.00159	-0.245201217	0.165131	-0.573689477	4.70E-07	-0.336821747	0.009044	0.232781554	0.34496	C5	-	-	Y	-	Y	Y	-
ENSG00000116922	C1orf109	0.332457253	3.04E-06	-0.092264768	0.444143	0.05385473	0.66083	-0.41836319	2.84E-08	-0.264031269	0.000474	0.149004452	0.271549	C5	-	Y	-	-	Y	-	-
ENSG00000116981	NT5C1A	0.18895436	0.28619	0.259606152	0.170116	0.364482883	0.032828	0.077524335	0.715646	0.190229183	0.295194	0.107471782	0.731303	C4	-	-	-	Y	-	-	-
ENSG00000116983	HPCAL4	-0.332080717	1.67E-07	0.04985037	0.601402	-0.005745915	0.960671	0.388351167	6.13E-11	0.34117195	3.52E-08	-0.052669253	0.654553	C4	-	Y	-	-	Y	Y	-
ENSG00000116985	BMP8B	-0.53514077	2.03E-08	-0.241637203	0.073944	-0.247459059	0.028236	0.29976946	0.014986	0.30253851	0.006485	-0.003060039	0.993273	C4	-	Y	-	-	-	Y	-
ENSG00000116990	MYCL	-0.700324848	0.013325	-0.419091929	0.196597	-0.303382852	0.367658	0.288445681	0.368898	0.412633843	0.17273	0.119062879	0.832105	C3	-	Y	-	-	-	-	-
ENSG00000117010	ZNF684	0.073781248	0.766313	-0.249992657	0.363072	0.274782689	0.169274	-0.316949787	0.149143	0.215837462	0.246414	0.528936398	0.018074	C1	-	-	-	-	-	-	Y
ENSG00000117016	RIMS3	-0.497524929	5.39E-23	-0.538145249	8.41E-25	-0.327021982	2.69E-09	-0.034285219	0.657051	0.185069442	0.001165	0.214078244	0.003172	C4	-	Y	Y	Y	-	-	-
ENSG00000117036	ETV3	0.236866461	0.003562	-0.331625412	0.000743	-0.135857055	0.275016	-0.562006424	5.30E-15	-0.358361342	7.92E-06	0.198566308	0.158813	C5	-	-	Y	-	Y	Y	-
ENSG00000117115	PADI2	-1.492460953	0.043885	-1.104373012	NA	-0.86518161	0.326779	0.385438451	0.771211	0.640037865	0.573327	0.244511546	NA	C4	-	Y	-	-	-	-	-
ENSG00000117143	UAP1	0.317826714	3.21E-07	0.201765617	0.015552	0.106945821	0.216524	-0.110094982	0.109688	-0.195943881	0.001014	-0.091531923	0.409181	C5	-	Y	-	-	-	-	-
ENSG00000117155	SSX2IP	0.409089399	1.78E-13	0.088551646	0.326985	0.033924197	0.727725	-0.313934396	4.06E-09	-0.360606127	9.90E-13	-0.051927473	0.666622	C5	Y	Y	-	-	Y	Y	-
ENSG00000117174	ZNHIT6	0.389643232	1.95E-06	-0.030054255	0.856621	0.049701823	0.723353	-0.413529455	9.96E-07	-0.325157823	0.000133	0.082593696	0.650708	C5	-	Y	-	-	Y	Y	-
ENSG00000117222	RBBP5	0.549490306	1.48E-09	0.105695391	0.457296	0.251274415	0.019408	-0.437393501	2.46E-13	-0.284133221	9.73E-07	0.147931982	0.108493	C5	-	Y	-	-	Y	-	-
ENSG00000117245	KIF17	-1.180033674	9.75E-05	-0.472724341	0.191651	-0.066538677	0.883585	0.71303861	0.02582	1.127481077	7.33E-05	0.410371121	0.316957	C4	-	Y	-	-	Y	Y	-
ENSG00000117262	GPR89A	0.135774585	0.453527	0.040848628	0.956089	-0.30377595	0.099132	-0.089615601	0.865017	-0.425557506	0.001263	-0.341577429	0.621168	C5	-	-	-	-	-	Y	-
ENSG00000117266	CDK18	-1.107658679	6.93E-17	-0.810796059	1.40E-06	-0.537561088	0.000104	0.30380604	0.137271	0.584537137	0.000132	0.275203057	0.316957	C3	-	Y	Y	Y	-	Y	-
ENSG00000117305	HMGCL	-1.193446332	1.12E-19	-0.464254195	0.001564	-0.281382123	0.058263	0.736541945	1.07E-06	0.927092795	4.69E-11	0.185734479	0.432096	C3	-	Y	Y	-	Y	Y	-
ENSG00000117360	PRPF3	0.607632566	2.09E-32	0.079396497	0.305729	0.087239855	0.191399	-0.521882448	5.91E-27	-0.50569296	8.74E-29	0.010830266	0.939872	C5	-	Y	-	-	Y	Y	-
ENSG00000117385	P3H1	0.362024972	0.000192	0.089567171	0.485832	0.248040542	0.006359	-0.265908573	0.004604	-0.098910808	0.330015	0.161100221	0.145561	C5	-	Y	-	-	-	-	-
ENSG00000117394	SLC2A1	0.211696967	0.00273	0.335157151	9.35E-07	-0.021977414	0.821548	0.130214764	0.072077	-0.218721087	0.000335	-0.35397332	9.34E-09	C4	-	-	Y	-	-	-	Y
ENSG00000117407	ARTN	-1.08168979	0.011612	0.286624838	0.56256	-0.171637633	0.791882	1.377678633	0.001206	0.9247338	0.094817	-0.458796931	NA	C2	-	Y	-	-	Y	-	-
ENSG00000117408	IPO13	0.453671816	1.28E-09	0.195662785	0.040315	0.306639678	0.000137	-0.251381148	0.000481	-0.132411376	0.075338	0.113466484	0.309634	C1	-	Y	-	Y	-	-	-
ENSG00000117448	AKR1A1	-0.386658021	1.89E-07	-0.331687567	7.86E-05	-0.22043761	0.010867	0.061723895	0.513012	0.181045978	0.018249	0.113920939	0.336188	C4	-	Y	Y	-	-	-	-
ENSG00000117461	PIK3R3	-0.305561568	9.88E-06	-0.375362526	1.40E-06	-0.258789061	0.00081	-0.063269907	0.497752	0.06137681	0.486347	0.119437983	0.316957	C4	-	Y	Y	-	-	-	-
ENSG00000117475	BLZF1	0.284390945	1.84E-05	-0.154459722	0.117291	0.074896111	0.458964	-0.432303791	4.03E-09	-0.194665008	0.006996	0.232125448	0.02897	C5	-	-	-	-	Y	-	-
ENSG00000117477	CCDC181	0.842076941	7.92E-13	0.247576163	0.156444	0.414063573	0.008679	-0.587769221	4.19E-07	-0.412493571	0.000928	0.169665802	0.474909	C5	-	Y	-	Y	Y	Y	-
ENSG00000117479	SLC19A2	0.390343985	2.76E-07	-0.053393114	0.71204	0.03309838	0.794736	-0.437231556	1.91E-07	-0.342555365	7.00E-06	0.08942277	0.586361	C5	-	Y	-	-	Y	Y	-
ENSG00000117480	FAAH	-0.382453223	0.005122	-0.499587553	0.001073	-0.256693688	0.095445	-0.11105296	0.569738	0.140524312	0.417299	0.245956785	0.274702	C4	-	Y	Y	-	-	-	-
ENSG00000117500	TMED5	0.571880003	5.09E-14	0.169695947	0.074852	0.369829671	2.48E-06	-0.395876566	2.34E-08	-0.187460422	0.010648	0.203038466	0.023349	C5	-	Y	-	Y	Y	-	-
ENSG00000117505	DR1	0.397178315	4.41E-17	0.084754106	0.21629	0.158984884	0.00621	-0.306133504	2.23E-12	-0.223532349	3.81E-07	0.077166375	0.315115	C5	Y	Y	-	-	Y	-	-
ENSG00000117533	VAMP4	0.385247792	1.60E-06	0.157428483	0.151182	0.293700461	0.001104	-0.221634639	0.008013	-0.076853176	0.417294	0.139385656	0.266197	C5	-	Y	-	-	-	-	-
ENSG00000117569	PTBP2	-0.249905544	0.000982	-0.209622067	0.007218	-0.334615089	2.00E-05	0.046870442	0.636309	-0.069942224	0.477119	-0.122137666	0.299852	C4	-	-	-	Y	-	-	-
ENSG00000117593	DARS2	-0.318135299	3.00E-07	-0.063796537	0.589256	-0.061972823	0.47969	0.260626651	0.000102	0.270580399	1.03E-07	0.004856195	0.980483	C4	-	Y	-	-	-	-	-
ENSG00000117595	IRF6	-0.498376443	1.25E-05	0.145899364	0.323928	0.170505343	0.217832	0.650547896	1.55E-08	0.683716411	3.97E-09	0.027595352	0.928793	C2	-	Y	-	-	Y	Y	-
ENSG00000117597	UTP25	0.256366988	0.000152	-0.107806035	0.296064	-0.008773703	0.945618	-0.357360579	6.37E-10	-0.250565669	6.06E-06	0.101583074	0.339412	C5	-	-	-	-	Y	-	-
ENSG00000117602	RCAN3	-0.434625457	0.000191	-0.332118286	0.021414	0.075200173	0.667272	0.108742936	0.493654	0.52442868	6.16E-06	0.410177265	0.007172	C4	-	Y	Y	-	-	Y	Y
ENSG00000117616	RSRP1	0.828597189	3.51E-19	0.335557447	5.53E-05	0.598531667	7.60E-12	-0.486235559	7.31E-09	-0.215153485	0.16497	0.265887907	0.006158	C5	-	Y	Y	Y	Y	-	-
ENSG00000117620	SLC35A3	-0.372618066	0.00098	-0.11614081	0.418752	-0.157267173	0.274016	0.263013549	0.022723	0.230074423	0.072928	-0.038307029	0.890117	C4	-	Y	-	-	-	-	-
ENSG00000117632	STMN1	-0.235758476	1.18E-07	-0.236049323	1.55E-05	-0.319798071	5.96E-10	0.006267898	0.887257	-0.069133984	0.023942	-0.080764437	0.126214	C4	-	-	-	Y	-	-	-
ENSG00000117691	NENF	-0.345858329	0.000476	-0.301706426	0.012455	-0.258946029	0.01965	0.051078266	0.65304	0.101987263	0.262889	0.0453319	0.809362	C4	-	Y	Y	-	-	-	-
ENSG00000117707	PROX1	-0.501737749	6.37E-20	-0.321770675	1.73E-05	-0.23770294	0.004405	0.186546943	0.00184	0.278677609	0.002855	0.086900955	0.529229	C4	-	Y	Y	-	-	-	-
ENSG00000117791	MTARC2	-0.173229467	0.31679	0.104798107	0.65208	0.291940841	0.106084	0.284428256	0.104388	0.479953944	0.003327	0.19026211	0.503347	C4	-	-	-	-	-	Y	-
ENSG00000117834	SLC5A9	-0.160020536	0.803976	0.556956494	0.312013	0.789222212	0.085921	0.722971529	0.123319	0.963648149	0.024476	0.23346884	NA	C2	-	-	-	-	-	Y	-
ENSG00000117971	CHRNB4	-0.787817392	2.19E-29	-0.356788842	1.77E-06	-0.502712739	1.00E-09	0.437515768	1.19E-09	0.299648249	0.000401	-0.142954386	0.252802	C4	-	Y	Y	Y	Y	-	-
ENSG00000118004	COLEC11	-0.450097743	6.02E-11	-0.129262811	0.337673	-0.133705074	0.218897	0.327246078	8.06E-05	0.331623649	5.94E-07	-0.001442335	0.996048	C4	-	Y	-	-	Y	Y	-
ENSG00000118007	STAG1	0.446482911	8.31E-08	0.194536583	0.073069	0.113884797	0.324728	-0.245128125	3.00E-05	-0.318179189	1.78E-08	-0.07830304	0.491895	C5	-	Y	-	-	-	Y	-
ENSG00000118058	KMT2A	0.524157628	0.001017	0.389301094	0.000168	0.447100815	4.08E-07	-0.128082357	0.476173	-0.062639145	0.734711	0.06077626	0.667843	C1	-	Y	Y	Y	-	-	-
ENSG00000118200	CAMSAP2	0.474294722	5.94E-12	0.320604503	9.20E-05	0.201381247	0.010865	-0.147207471	0.010075	-0.258350273	2.79E-08	-0.116550146	0.148991	C1	-	Y	Y	-	-	-	-
ENSG00000118257	NRP2	0.555248754	0.527934	1.696037367	0.006971	1.782593295	0.00235	1.14811146	0.029016	1.243869741	0.014287	0.091331043	NA	C2	-	-	Y	Y	Y	Y	-
ENSG00000118292	C1orf54	0.705260139	0.144329	-0.443389843	0.605511	0.004885687	0.995629	-1.142016033	0.024019	-0.687078442	0.169247	0.451983537	NA	C5	-	-	-	-	Y	-	-
ENSG00000118402	ELOVL4	0.417730288	0.013229	-0.011519759	0.970759	0.05418662	0.843281	-0.423275895	0.001349	-0.34907086	0.014617	0.068778745	0.827579	C1	-	Y	-	-	Y	Y	-
ENSG00000118412	CASP8AP2	0.123250025	0.177706	-0.288547822	0.002563	-0.08349781	0.463651	-0.405249741	3.10E-10	-0.192229556	0.003531	0.207656153	0.022197	C5	-	-	-	-	Y	-	-
ENSG00000118420	UBE3D	0.522105086	0.007775	0.514598666	0.021469	0.447179869	0.097026	-0.001108729	0.996345	-0.05962805	0.851945	-0.064249348	0.912195	C1	-	Y	Y	-	-	-	-
ENSG00000118496	FBXO30	0.409394581	2.17E-07	0.034359791	0.821415	0.069862305	0.552944	-0.368683267	6.09E-07	-0.325228009	2.14E-06	0.038222574	0.834038	C5	-	Y	-	-	Y	Y	-
ENSG00000118507	AKAP7	-0.993943576	0.015049	-0.479825287	0.305307	-0.599577627	0.143455	0.517918192	0.331217	0.409154249	0.445664	-0.115566487	NA	C3	-	Y	-	-	-	-	-
ENSG00000118508	RAB32	-0.266406708	0.13045	0.297923513	0.078435	-0.033112667	0.90072	0.571071717	3.33E-05	0.248083174	0.151452	-0.328652587	0.070088	C2	-	-	-	-	Y	-	-
ENSG00000118513	MYB	0.240279732	0.060202	-0.484159915	0.000725	-0.405312319	0.00972	-0.717959204	5.06E-09	-0.631049457	1.82E-06	0.082242462	0.806285	C5	-	-	Y	Y	Y	Y	-
ENSG00000118515	SGK1	0.263992665	1.09E-05	0.519290378	1.18E-16	0.323286755	1.22E-07	0.261690907	7.29E-07	0.073902553	0.180541	-0.193038626	0.004543	C4	-	-	Y	Y	-	-	-
ENSG00000118579	MED28	0.386122547	8.60E-11	0.184269307	0.021264	0.26498954	5.64E-05	-0.195324126	0.002066	-0.106550547	0.082437	0.083517404	0.444627	C5	-	Y	-	-	-	-	-
ENSG00000118600	RXYLT1	-0.124335906	0.421894	0.23249252	0.184766	0.237961094	0.100771	0.363394812	0.012701	0.377189465	0.00371	0.008285735	0.982359	C4	-	-	-	-	Y	Y	-
ENSG00000118620	ZNF430	0.085174346	0.497545	-0.305318078	0.010975	-0.163099734	0.218112	-0.384007592	0.000412	-0.23370998	0.042865	0.144991424	0.456746	C4	-	-	Y	-	Y	-	-
ENSG00000118655	DCLRE1B	0.106490254	0.282883	-0.216948495	0.059176	-0.118656451	0.394466	-0.316768315	0.000671	-0.210577272	0.04608	0.101858957	0.627073	C5	-	-	-	-	Y	-	-

ENSG00000118689	FOXO3	-0.278416956	7.95E-06	0.042323269	0.636549	0.068947539	0.358839	0.327052303	1.79E-11	0.361826254	1.91E-14	0.029302326	0.754779	C4	-	-	-	-	Y	Y	-
ENSG00000118733	OLFM3	-0.324017522	0.03718	-0.012223207	0.964845	-0.003440112	0.988763	0.31804302	0.025262	0.335280808	0.015933	0.011963769	0.973837	C4	-	Y	-	-	Y	Y	-
ENSG00000118762	PKD2	-0.314312889	1.64E-08	-0.103482992	0.165447	-0.208620943	0.002016	0.217236485	0.000238	0.120485367	0.077438	-0.102170691	0.303368	C4	-	Y	-	-	-	-	-
ENSG00000118849	RARRES1	-1.359685021	0.010194	-0.485255931	0.427814	-0.718900952	0.229111	0.87820965	0.187668	0.657237349	0.392659	-0.227726335	NA	C3	-	Y	-	-	-	-	-
ENSG00000118873	RAB3GAP2	0.487273929	3.22E-12	0.238974641	0.003791	0.226669871	0.004269	-0.241575347	3.07E-07	-0.245969708	1.27E-07	-0.009868625	0.942494	C5	Y	Y	-	-	-	-	-
ENSG00000118898	PPL	-1.097256777	0.043974	-0.686369378	0.258941	-0.468031189	0.399362	0.412777534	0.584318	0.643618229	0.303772	0.220746871	NA	C3	-	Y	-	-	-	-	-
ENSG00000118946	PCDH17	-0.469822088	3.51E-13	0.108930263	0.288463	0.054694708	0.66426	0.585229262	2.31E-19	0.539486585	6.04E-14	-0.051468108	0.778766	C4	-	Y	-	-	Y	Y	-
ENSG00000118960	HS1BP3	-0.319813318	0.001493	0.039237147	0.835311	0.159555232	0.137901	0.365966871	0.001504	0.494111814	8.53E-08	0.123595294	0.498731	C4	-	Y	-	-	Y	Y	-
ENSG00000118965	WDR35	-0.04929521	0.564534	0.247282017	0.001919	0.238350806	0.000905	0.303373585	5.91E-10	0.302252351	3.69E-12	-0.006504081	0.965685	C4	-	-	-	-	Y	Y	-
ENSG00000119138	KLF9	-0.789261161	4.95E-07	-0.19987945	0.320156	-0.178227294	0.405867	0.596653942	0.000593	0.625498983	0.000583	0.023756101	0.959572	C4	-	Y	-	-	Y	Y	-
ENSG00000119139	TJP2	0.522808192	0.000566	0.349902938	0.086481	0.282292967	0.13512	-0.166033921	0.371469	-0.225742879	0.171034	-0.065110887	0.862969	C5	-	Y	-	-	-	-	-
ENSG00000119227	PIGZ	-0.923158992	0.000918	-0.439172168	0.168673	-0.284570537	0.417785	0.4911714	0.136378	0.652700392	0.044024	0.156759064	NA	C4	-	Y	-	-	-	Y	-
ENSG00000119231	SENP5	0.503057651	8.77E-14	0.015090816	0.911609	0.014676559	0.907323	-0.481464328	2.22E-13	-0.473728756	4.66E-14	0.002384344	0.991437	C5	-	Y	-	-	Y	Y	-
ENSG00000119283	TRIM67	-0.46606499	0.002587	-0.31456907	0.138844	-0.222765463	0.227238	0.158430131	0.495154	0.25791935	0.162986	0.095067192	0.81377	C4	-	Y	-	-	-	-	-
ENSG00000119285	HEATR1	0.583452781	3.42E-12	0.178025661	0.121464	0.236691658	0.012491	-0.398998721	1.84E-14	-0.33266282	3.75E-16	0.061081323	0.506753	C5	-	Y	-	-	Y	Y	-
ENSG00000119328	ABITRAM	0.437646676	0.001891	0.013130033	0.962058	0.259550321	0.127303	-0.417299541	0.000484	-0.163648843	0.231677	0.248472164	0.160931	C5	-	Y	-	-	Y	-	-
ENSG00000119333	WDR34	-0.371085607	4.02E-06	-0.203625544	0.059623	-0.18619138	0.080275	0.174553649	0.017397	0.199830407	0.005488	0.020219479	0.926065	C4	-	Y	-	-	-	-	-
ENSG00000119402	FBXW2	0.353565535	9.18E-09	0.106722887	0.241262	0.181989375	0.006255	-0.240284859	1.26E-05	-0.157164308	0.000749	0.077794959	0.389254	C5	Y	Y	-	-	-	-	-
ENSG00000119403	PHF19	-0.185213907	0.013229	-0.175624285	0.025884	-0.32054409	3.11E-05	0.01616507	0.878697	-0.120634363	0.158572	-0.141947486	0.196321	C4	-	-	-	Y	-	-	-
ENSG00000119408	NEK6	-0.047027048	0.86189	0.037139032	0.900507	0.386668597	0.029063	0.090275393	0.698252	0.448073847	0.007303	0.351958772	0.067891	C4	-	-	-	Y	-	Y	-
ENSG00000119471	HSDL2	-0.416166431	5.02E-05	-0.030209123	0.875206	0.000648406	0.99652	0.392483493	0.000293	0.431372505	4.42E-06	0.034165088	0.891955	C4	-	Y	-	-	Y	Y	-
ENSG00000119508	NR4A3	0.479121808	0.005599	0.0356008	0.918164	0.03096821	0.922903	-0.436701675	0.019626	-0.433254653	0.018505	-0.001682812	0.997341	C5	-	Y	-	-	Y	Y	-
ENSG00000119514	GALNT12	-0.972606106	0.009102	-0.097344682	0.86258	-0.08698171	0.860914	0.88338917	0.034953	0.900697573	0.025002	0.01345374	NA	C3	-	Y	-	-	Y	Y	-
ENSG00000119522	DENND1A	-0.450284689	6.01E-07	0.05963794	0.601739	0.072704425	0.524939	0.516323921	3.64E-09	0.537540854	4.01E-09	0.015924461	0.943467	C2	-	Y	-	-	Y	Y	-
ENSG00000119523	ALG2	0.190492099	0.050191	-0.378111234	0.000578	-0.079445885	0.547638	-0.562308833	8.61E-09	-0.255223556	0.008556	0.301594373	0.02324	C5	-	-	Y	-	Y	-	Y
ENSG00000119547	ONECUT2	1.124403478	4.66E-07	0.258992236	0.411456	0.370861621	0.137595	-0.859034566	1.10E-06	-0.739246981	2.00E-06	0.114600607	0.588611	C5	-	Y	-	-	Y	Y	-
ENSG00000119574	ZBTB45	-0.039701127	0.796602	-0.320078514	0.01702	-0.206005261	0.183091	-0.274023721	0.031333	-0.151554945	0.316611	0.1170579	0.644427	C4	-	-	Y	-	-	-	-
ENSG00000119630	PGF	-1.29921477	3.90E-10	-0.318777182	0.208293	-0.463190522	0.040738	0.985254876	0.000106	0.850389507	0.001172	-0.140291134	0.781334	C3	-	Y	-	Y	Y	Y	-
ENSG00000119632	IFI27L2	-0.620477837	3.61E-05	-0.356361549	0.043742	-0.334779881	0.04852	0.271001875	0.070383	0.301193369	0.038965	0.024822225	0.943834	C4	-	Y	Y	Y	-	Y	-
ENSG00000119638	NEK9	-0.310775428	9.80E-10	-0.059382512	0.409798	-0.016463716	0.831744	0.257766544	1.23E-07	0.308958947	2.75E-12	0.045871034	0.592207	C4	-	Y	-	-	-	Y	-
ENSG00000119650	IFT43	-0.336638452	0.00404	0.015472516	0.941923	-0.143308781	0.374116	0.35854238	0.002555	0.208144612	0.137031	-0.15569313	0.455397	C4	-	Y	-	-	Y	-	-
ENSG00000119661	DNAL1	0.636700453	4.31E-13	0.224344568	0.1113	0.217376603	0.05397	-0.406354432	4.85E-06	-0.404673585	2.53E-08	-0.003875992	0.989481	C5	Y	Y	-	-	Y	Y	-
ENSG00000119669	IRF2BPL	-0.147791352	0.13535	0.371789162	9.62E-05	0.10086195	0.322049	0.52618046	4.21E-11	0.263823287	0.000278	-0.267322343	0.002326	C4	-	-	Y	-	Y	-	-
ENSG00000119673	ACOT2	-0.502573895	0.013915	-0.267557365	0.278679	-0.442383354	0.037688	0.242583396	0.231575	0.075526796	0.759343	-0.173278148	0.558058	C4	-	Y	-	Y	-	-	-
ENSG00000119681	LTBP2	-0.672753457	1.15E-07	0.004957684	0.985258	0.22126746	0.14561	0.683890546	1.52E-07	0.908663074	1.75E-14	0.219343007	0.271039	C4	-	Y	-	-	Y	Y	-
ENSG00000119688	ABCD4	0.412616858	4.82E-14	0.089945517	0.313112	0.205127674	0.005053	-0.316292867	5.68E-08	-0.192857427	0.001531	0.118055968	0.265533	C5	Y	Y	-	-	Y	-	-
ENSG00000119711	ALDH6A1	-0.329441707	0.000693	-0.018931756	0.918253	0.040088125	0.788438	0.316585006	0.000867	0.384388281	1.08E-05	0.062420447	0.740605	C4	-	Y	-	-	Y	Y	-
ENSG00000119714	GPR68	-0.945959633	6.64E-10	-0.267602566	0.17263	-0.690536346	0.000115	0.684077242	0.000144	0.269762434	0.246318	-0.419355456	0.122869	C3	-	Y	-	Y	Y	-	-
ENSG00000119725	ZNF410	0.30399653	0.056679	-0.077898539	0.770899	-0.133972857	0.553604	-0.375514902	0.016534	-0.423612152	0.006188	-0.053355817	0.894324	C5	-	-	-	-	Y	Y	-
ENSG00000119771	KLHL29	-0.340962604	0.001602	-0.315715661	0.033154	-0.131702013	0.367684	0.031689057	0.862019	0.2236524	0.06293	0.187045369	0.380196	C4	-	Y	Y	-	-	-	-
ENSG00000119777	TMEM214	0.34587807	6.74E-09	0.064921005	0.574925	0.100547807	0.260586	-0.274552082	7.19E-05	-0.230580566	0.000294	0.038438785	0.832353	C5	-	Y	-	-	-	-	-
ENSG00000119778	ATAD2B	0.686530192	4.62E-18	0.187648656	0.098817	0.349973623	0.000421	-0.492515038	1.94E-12	-0.321769927	1.10E-05	0.165166647	0.188673	C5	-	Y	-	Y	Y	Y	-
ENSG00000119782	FKBP1B	-0.520284473	7.61E-07	-0.250794114	0.047257	-0.201578023	0.124056	0.276639929	0.021349	0.334136659	0.00525	0.052070215	0.843749	C4	-	Y	-	-	-	Y	-
ENSG00000119812	FAM98A	0.477552953	2.24E-09	0.02957471	0.857678	0.135406775	0.205294	-0.441578956	1.96E-11	-0.327466525	7.93E-08	0.108634974	0.361968	C5	-	Y	-	-	Y	Y	-
ENSG00000119820	YIPF4	0.347538829	4.33E-08	0.060919852	0.608512	0.024225945	0.815596	-0.279834523	6.63E-06	-0.309039783	1.42E-10	-0.034413234	0.815291	C5	-	Y	-	-	-	Y	-
ENSG00000119844	AFTPH	0.424559429	6.59E-08	0.222087988	0.018557	0.365084105	8.60E-06	-0.196178133	0.006077	-0.045279322	0.603922	0.145646528	0.136166	C5	-	Y	-	Y	-	-	-
ENSG00000119866	BCL11A	-1.551602697	0.000785	-0.467270336	0.395566	-0.527638173	0.303548	1.089929507	0.037015	1.038271004	0.051869	-0.057220289	NA	C3	-	Y	-	-	Y	-	-
ENSG00000119878	CRIPT	0.188460819	0.00255	-0.124090132	0.209351	-0.016261247	0.879742	-0.306001962	1.64E-05	-0.190028206	0.002486	0.110675	0.375519	C5	-	-	-	-	Y	-	-
ENSG00000119888	EPCAM	-1.35156491	0.00825	-0.10842749	0.87984	-0.412987546	0.480686	1.251595071	0.013831	0.952887784	0.095262	-0.301541801	NA	C2	-	Y	-	-	Y	-	-
ENSG00000119899	SLC17A5	-0.021305937	0.933914	0.088475933	0.734122	0.345757037	0.053169	0.116119195	0.586355	0.381805959	0.023288	0.260237336	0.279479	C4	-	-	-	-	-	Y	-
ENSG00000119906	SLF2	0.192885408	0.028909	-0.128490575	0.351056	0.076976216	0.504909	-0.315007453	0.00014	-0.101220307	0.157524	0.20836309	0.072232	C5	-	-	-	-	Y	-	-
ENSG00000119915	ELOVL3	-1.308334905	0.009121	-1.153338405	0.032024	-0.945090047	0.063921	0.159056079	0.856225	0.378618643	0.617973	0.212529811	NA	C3	-	Y	Y	-	-	-	-
ENSG00000119927	GPAM	-0.440049347	3.01E-11	-0.18790121	0.082714	-0.045130031	0.689232	0.258923892	0.00375	0.409722899	4.56E-09	0.145410532	0.315425	C4	-	Y	-	-	-	Y	-
ENSG00000119929	CUTC	-0.383777877	0.005037	-0.084591128	0.629993	-0.198675019	0.150828	0.305390106	0.037485	0.199929382	0.205423	-0.111102752	0.614875	C4	-	Y	-	-	Y	-	-
ENSG00000119938	PPP1R3C	-0.636557706	0.009121	0.569010415	0.046686	0.004647777	0.991172	1.214347178	4.13E-06	0.656091288	0.016234	-0.563196041	0.126087	C2	-	Y	Y	-	Y	Y	-
ENSG00000119953	SMNDC1	0.186984994	0.013194	-0.03738154	0.77783	-0.175764921	0.053415	-0.218102761	0.002874	-0.347865592	5.70E-07	-0.135287052	0.244326	C5	-	-	-	-	-	Y	-
ENSG00000119965	C10orf88	0.468852989	2.99E-06	0.08966077	0.607926	0.147399819	0.289989	-0.373280709	0.000217	-0.306791613	0.001546	0.060922623	0.794993	C5	-	Y	-	-	Y	Y	-
ENSG00000119973	PRLHR	-0.94854496	0.032445	-0.596437068	0.21905	-0.673335545	0.14628	0.364075626	0.551776	0.290357451	0.653529	-0.076444677	NA	C3	-	Y	-	-	-	-	-
ENSG00000120049	KCNIP2	-0.067309336	0.768667	0.139080535	0.586699	0.288008307	0.135557	0.214105742	0.330511	0.370060275	0.048487	0.151138508	0.654742	C4	-	-	-	-	-	Y	-
ENSG00000120053	GOT1	-0.322680017	0.000479	-0.153951896	0.20466	-0.167819164	0.118142	0.175246067	0.08851	0.169716413	0.082166	-0.010815843	0.967143	C4	-	Y	-	-	-	-	-
ENSG00000120055	C10orf95	-2.0266202	0.208591	0.884636847	NA	0.81530934	NA	2.916370469	0.031151	2.858239169	0.041186	-0.067013759	NA	C2	-	-	-	-	Y	Y	-
ENSG00000120149	MSX2	-0.361290981	9.12E-06	0.182046822	0.050878	-0.108331611	0.314521	0.549793189	1.50E-13	0.267668037	0.001531	-0.287572601	0.001909	C4	-	Y	-	-	Y	-	-
ENSG00000120217	CD274	1.443839223	4.38E-07	1.163731208	0.00027	1.045740841	0.001703	-0.27312874	0.371225	-0.38391996	0.201454	-0.11559237	NA	C1	-	Y	Y	Y	-	-	-
ENSG00000120251	GRIA2	-0.089773274	0.447644	-0.315857067	0.023759	-0.048971254	0.767278	-0.218976176	0.046192	0.054715741	0.639267	0.269305543	0.087131	C5	-	-	Y	-	-	-	-
ENSG00000120253	NUP43	0.305453505	9.01E-06	0.161025791	0.043882	0.095565752	0.250929	-0.138074541	0.037883	-0.195416612	0.001408	-0.062734531	0.550996	C5	-	Y	-	-	-	-	-
ENSG00000120254	MTHFD1L	-0.01958028	0.842761	-0.369927848	0.000675	-0.165243178	0.068145	-0.343631658	0.000233	-0.131035925	0.104822	0.207836419	0.162306	C5	-	-	Y	-	Y	-	-
ENSG00000120306	CYSTM1	0.177193938	0.45584	0.202638284	0.449101	0.39810256	0.048035	0.032371221	0.9154	0.235450143	0.285826	0.198491953	0.550522	C4	-	-	-	Y	-	-	-

ENSG00000120324	PCDHB10	-0.333477675	0.018644	-0.172814824	0.272359	-0.16960775	0.248652	0.167967634	0.321105	0.17872641	0.268064	0.006142746	0.986496	C4	-	Y	-	-	-	-	-
ENSG00000120334	CENPL	0.128540662	0.309126	-0.206407051	0.169125	-0.140849919	0.328567	-0.328281209	0.006558	-0.254541948	0.026335	0.068440445	0.795275	C5	-	-	-	-	Y	-	-
ENSG00000120458	MSANTD2	0.182525909	0.057236	-0.181433981	0.179164	-0.01165901	0.943368	-0.356666402	7.77E-06	-0.179965771	0.01093	0.171909141	0.169629	C5	-	-	-	-	Y	-	-
ENSG00000120519	SLC10A7	0.435307316	0.012014	0.20026984	0.482919	0.174380865	0.536828	-0.22789652	0.250191	-0.246349605	0.215406	-0.022770394	0.968401	C1	-	Y	-	-	-	-	-
ENSG00000120526	NUDCD1	0.391444549	3.15E-10	0.210207226	0.005067	0.130811115	0.093454	-0.175002299	0.004572	-0.246012813	2.56E-05	-0.076435684	0.474909	C5	-	Y	-	-	-	-	-
ENSG00000120533	ENY2	-0.236883679	0.000103	-0.234829029	0.002727	-0.336308056	5.17E-07	0.009050557	0.917964	-0.084639615	0.165855	-0.098887705	0.319912	C4	-	-	-	Y	-	-	-
ENSG00000120549	KIAA1217	-2.157585892	1.79E-06	-0.748009701	0.115145	-0.226813936	0.640784	1.425246484	0.016645	1.944776661	6.97E-05	0.52112295	NA	C3	-	Y	-	-	Y	Y	-
ENSG00000120616	EPC1	-0.136065268	0.105773	-0.360339399	6.93E-07	-0.305470469	0.000102	-0.21806859	0.006752	-0.154687781	0.078806	0.057874409	0.709334	C4	-	-	Y	Y	-	-	-
ENSG00000120647	CCDC77	0.453856689	3.06E-07	0.096872255	0.52508	0.183000016	0.109272	-0.350861184	5.45E-05	-0.256565293	0.001303	0.089403712	0.606986	C5	-	Y	-	-	Y	-	-
ENSG00000120690	ELF1	0.477959726	2.12E-06	0.242597787	0.065827	0.229172283	0.060937	-0.228805593	0.049573	-0.234013759	0.034927	-0.010531873	0.973757	C5	Y	Y	-	-	-	-	-
ENSG00000120694	HSPH1	0.600138929	8.97E-44	0.231673874	0.000575	0.425295093	4.60E-17	-0.36176635	3.24E-17	-0.160366687	4.68E-06	0.196301353	0.002326	C5	-	Y	-	Y	Y	-	-
ENSG00000120696	KBTBD7	0.201857136	0.075848	-0.114555853	0.521807	0.090215263	0.608384	-0.309680428	0.006522	-0.097180854	0.471771	0.207721994	0.294907	C5	-	-	-	-	Y	-	-
ENSG00000120697	ALG5	-0.2817401	0.015941	-0.28907382	0.037606	-0.449216467	0.000194	-0.001207691	0.993358	-0.15261295	0.192325	-0.156788477	0.377672	C4	-	-	-	Y	-	-	-
ENSG00000120708	TGFBI	0.561722422	0.347292	1.11475346	0.04483	1.591206207	0.000319	0.558662089	0.272772	1.043118759	0.009728	0.476699285	NA	C2	-	-	Y	Y	-	Y	-
ENSG00000120725	SIL1	-1.005422787	9.80E-08	-0.36522154	0.048786	-0.317037774	0.068374	0.645578491	0.002893	0.702920376	0.000612	0.051104804	0.90377	C2	-	Y	Y	-	Y	Y	-
ENSG00000120756	PLS1	0.353944017	0.001719	0.357116118	0.006736	0.310847435	0.035061	0.009751525	0.954455	-0.029009877	0.869275	-0.04311626	0.891807	C1	-	Y	Y	Y	-	-	-
ENSG00000120784	ZFP30	0.303873338	6.24E-05	-0.081240491	0.566325	0.036909527	0.833141	-0.378389889	3.49E-06	-0.252169179	0.010991	0.12124295	0.554872	C5	-	Y	-	-	Y	-	-
ENSG00000120800	UTP20	0.484204362	1.25E-10	0.246885728	0.003789	0.111894619	0.221212	-0.230863424	0.000693	-0.357824916	8.65E-09	-0.132629998	0.14514	C5	-	Y	-	-	-	Y	-
ENSG00000120805	ARL1	0.310450224	6.58E-06	0.135070761	0.154075	0.080573006	0.493494	-0.169073818	0.024427	-0.215029007	0.008615	-0.05175651	0.777633	C5	-	Y	-	-	-	-	-
ENSG00000120860	WASHC3	-0.377409646	0.005951	-0.186277993	0.216518	0.081714722	0.61232	0.197574133	0.221934	0.473500963	0.000361	0.270775819	0.094643	C4	-	Y	-	-	-	Y	-
ENSG00000120868	APAF1	0.334596475	0.000401	0.184236845	0.167024	0.295284681	0.007747	-0.14453416	0.07085	-0.02456046	0.80317	0.114279171	0.405437	C4	-	Y	-	-	-	-	-
ENSG00000120875	DUSP4	0.702958638	5.03E-58	0.383927551	2.92E-15	0.347968039	6.62E-12	-0.312533619	1.18E-17	-0.340405055	3.13E-20	-0.033165994	0.709769	C5	-	Y	Y	Y	Y	Y	-
ENSG00000120885	CLU	-0.473554635	1.52E-06	-0.10856966	0.453058	-0.192254521	0.119457	0.371630107	0.001219	0.296012705	0.013754	-0.081305814	0.733616	C4	-	Y	-	-	Y	-	-
ENSG00000120896	SORBS3	-0.29652108	0.001884	-0.310168307	0.005588	-0.275884125	0.006032	-0.006710269	0.96112	0.035555871	0.752811	0.036623162	0.855827	C4	-	-	Y	-	-	-	-
ENSG00000120899	PTK2B	-1.09747984	6.23E-12	-0.218939128	0.218939	-0.266959826	0.180701	0.884301672	4.27E-07	0.844790003	1.88E-05	-0.044525358	0.925998	C3	-	Y	-	-	Y	Y	-
ENSG00000120903	CHRNA2	-2.862169534	0.028962	-1.694929639	NA	-3.95133942	NA	1.185930254	NA	-1.071446306	NA	-2.241650308	NA	C4	-	Y	-	-	-	-	-
ENSG00000120910	PPP3CC	0.326967838	0.015726	0.225168906	0.200252	0.172946074	0.329216	-0.095269688	0.556381	-0.139431031	0.355256	-0.049537532	0.879022	C5	-	Y	-	-	-	-	-
ENSG00000120913	PDLIM2	-0.570313296	0.006295	-0.211106486	0.46196	-0.379632937	0.112134	0.36613859	0.119772	0.206012296	0.428527	-0.1655664	0.686302	C4	-	Y	-	-	-	-	-
ENSG00000120915	EPHX2	-0.962661094	8.53E-17	-0.470857009	0.000276	-0.261290769	0.041009	0.49973865	0.000784	0.715815973	6.97E-08	0.21128144	0.317035	C4	-	Y	Y	-	Y	Y	-
ENSG00000120925	RNF170	0.263362496	0.005732	0.332374801	0.000222	0.383195351	1.62E-05	0.075397677	0.492459	0.134369233	0.181546	0.053659353	0.75144	C4	-	-	Y	Y	-	-	-
ENSG00000120948	TARDBP	0.338214124	1.09E-13	0.039911557	0.590293	-0.028347869	0.703679	-0.291943207	1.52E-15	-0.351930933	2.49E-23	-0.065333086	0.296205	C5	Y	Y	-	-	-	Y	-
ENSG00000121005	CRISPLD1	-0.683611286	4.82E-15	-0.200138575	0.06259	-0.300700747	0.002327	0.490255118	4.29E-07	0.397696434	7.48E-05	-0.097807349	0.587008	C2	-	Y	-	Y	Y	Y	-
ENSG00000121053	EPX	-0.714068158	0.211537	0.623791472	0.24907	0.275947772	0.677017	1.343237895	0.007645	1.004422216	0.078014	-0.344849793	NA	C2	-	-	-	-	Y	-	-
ENSG00000121060	TRIM25	-0.245615865	0.000185	-0.014747216	0.904748	0.106696439	0.22684	0.23722662	0.000208	0.367069336	6.90E-09	0.124343819	0.220878	C4	-	-	-	-	-	Y	-
ENSG00000121067	SPOP	0.316344319	1.31E-06	0.104317601	0.28815	0.114383514	0.150435	-0.205686724	0.003954	-0.187296285	0.003289	0.013074599	0.944407	C5	-	Y	-	-	-	-	-
ENSG00000121075	TBX4	-1.95529722	0.000236	-1.058826295	0.082963	-1.642836692	0.001882	0.8897495	0.322399	0.322386093	0.760309	-0.575013642	NA	C3	-	Y	-	Y	-	-	-
ENSG00000121274	TENT4B	0.465963559	2.53E-07	0.09042931	0.573795	0.198750576	0.076687	-0.369094687	1.74E-05	-0.252862849	0.00156	0.110845807	0.48776	C5	-	Y	-	-	Y	-	-
ENSG00000121281	ADCY7	-0.451164151	0.000819	-0.049643235	0.835278	0.156248986	0.308681	0.407915353	0.003686	0.621585045	4.93E-08	0.209296871	0.266197	C4	-	Y	-	-	Y	Y	-
ENSG00000121310	ECHDC2	-0.777600398	0.00011	-0.529485517	0.025225	-0.420137815	0.052955	0.253221525	0.3695	0.372358195	0.126953	0.112682014	0.816997	C4	-	Y	Y	-	-	-	-
ENSG00000121318	TAS2R10	1.144132754	0.035927	0.197257782	NA	1.146683515	0.054311	-0.939351998	0.126174	0.016813876	0.979158	0.950898002	NA	C1	-	Y	-	-	-	-	-
ENSG00000121350	PYROXD1	-0.616702444	6.79E-08	-0.132235156	0.333252	-0.314347926	0.006811	0.490281916	2.37E-05	0.316681135	0.013762	-0.179213801	0.273604	C3	-	Y	-	Y	Y	Y	-
ENSG00000121390	PSPC1	-0.629399907	2.30E-21	-0.078634198	0.385897	-0.274721654	0.000292	0.557099199	6.35E-18	0.369229114	3.44E-07	-0.19329653	0.035966	C4	-	Y	-	-	Y	Y	-
ENSG00000121417	ZNF211	0.188465077	0.100961	-0.12861879	0.461455	0.12484121	0.402445	-0.311362133	0.00675	-0.049153562	0.72498	0.256471052	0.126489	C5	-	-	-	-	Y	-	-
ENSG00000121440	PDZRN3	-0.923711432	8.95E-10	0.208700728	0.250082	0.024499364	0.916162	1.138772977	3.48E-14	0.96325708	1.34E-10	-0.181255691	0.433494	C3	-	Y	-	-	Y	Y	-
ENSG00000121454	LHX4	-0.329827885	0.045423	0.275118164	0.114479	0.312162021	0.046447	0.61111368	1.50E-05	0.656207448	1.49E-06	0.040445318	0.899704	C4	-	Y	-	Y	Y	Y	-
ENSG00000121577	POPDC2	-0.363877064	9.77E-05	-0.330989426	0.016634	-0.195908862	0.104102	0.039369328	0.824154	0.182694242	0.120534	0.138775941	0.553698	C4	-	Y	Y	-	-	-	-
ENSG00000121644	DESI2	0.33300031	4.18E-09	0.143312688	0.054198	0.143231303	0.031651	-0.182959677	6.84E-05	-0.175363744	3.12E-05	0.00226078	0.986675	C5	-	Y	-	-	-	-	-
ENSG00000121671	CRY2	0.175469423	0.169057	-0.22596449	0.14299	0.078869582	0.75097	-0.39512577	0.001117	-0.082363274	0.687996	0.307063212	0.21454	C5	-	-	-	-	Y	-	-
ENSG00000121716	PILRB	0.525942482	9.10E-05	0.226179709	0.219537	0.272958017	0.113001	-0.293170165	0.042931	-0.238087623	0.117899	0.049687333	0.89024	C5	-	Y	-	-	-	-	-
ENSG00000121753	ADGRB2	-0.674325344	3.87E-29	-0.055108896	0.568923	0.092850163	0.193731	0.625704231	7.76E-25	0.781946064	3.68E-47	0.150597061	0.057727	C4	-	Y	-	-	Y	Y	-
ENSG00000121774	KHDRBS1	-0.217968893	2.35E-10	-0.179641481	0.000544	-0.358318863	2.79E-19	0.044975567	0.325506	-0.125487474	9.71E-05	-0.175834316	0.000506	C4	-	-	-	Y	-	-	-
ENSG00000121851	POLR3GL	-0.48941784	5.28E-08	-0.199844375	0.083321	-0.221037626	0.070583	0.296084972	0.002658	0.283419035	0.00914	-0.018328018	0.949752	C4	-	Y	-	-	-	-	-
ENSG00000121879	PIK3CA	0.767002119	2.05E-27	0.319452715	0.001021	0.370442118	1.02E-05	-0.441423353	2.29E-13	-0.382275267	3.57E-13	0.05391559	0.705397	C5	-	Y	Y	Y	Y	Y	-
ENSG00000121892	PDS5A	0.395266191	7.35E-16	0.214421449	0.000973	0.219833679	0.000199	-0.174450757	5.69E-05	-0.160931074	3.90E-05	0.008251956	0.952524	C5	-	Y	-	-	-	-	-
ENSG00000121900	TMEM54	-0.513002881	0.009517	-0.429079721	0.061652	-0.378030916	0.130637	0.090341351	0.721921	0.150418366	0.556885	0.054223682	0.918762	C4	-	Y	-	-	-	-	-
ENSG00000121903	ZSCAN20	0.568987974	0.000312	0.141215169	0.536507	0.373601806	0.045567	-0.421634997	0.003705	-0.180720213	0.301906	0.235406931	0.309109	C5	-	Y	-	Y	Y	-	-
ENSG00000121904	CSMD2	-0.070984812	0.910124	0.848021045	0.044556	0.559458216	0.201752	0.926847747	0.021568	0.645688008	0.132532	-0.285301005	NA	C2	-	-	Y	-	Y	-	-
ENSG00000121931	LRIF1	0.258512145	0.003612	-0.235032156	0.039203	0.103186318	0.403153	-0.486672574	1.06E-10	-0.141088636	0.085077	0.340495765	0.000534	C5	-	-	-	-	Y	-	Y
ENSG00000121964	GTDC1	-0.299642406	0.005914	0.165273149	0.13888	-0.039425455	0.78708	0.471104026	3.70E-06	0.274891459	0.01418	-0.201776065	0.114345	C4	-	-	-	-	Y	-	-
ENSG00000122008	POLK	0.603409962	1.55E-14	0.25098987	0.012159	0.291130404	0.001362	-0.345853291	2.21E-07	-0.29767645	5.23E-06	0.042727129	0.782697	C5	-	Y	-	-	Y	-	-
ENSG00000122042	UBL3	0.422709147	2.32E-06	0.365261496	0.001753	0.216247536	0.075926	-0.051598188	0.659236	-0.191383901	0.038058	-0.145987192	0.377812	C1	-	Y	Y	-	-	-	-
ENSG00000122068	FYTTD1	0.415729044	1.65E-14	0.184816302	0.010923	0.058240698	0.45477	-0.224643973	3.32E-05	-0.342806822	7.06E-14	-0.123595157	0.118464	C5	-	Y	-	-	-	Y	-
ENSG00000122121	XPNPEP2	-0.221882693	0.456319	-0.591673321	0.092052	-0.714758116	0.023485	-0.361398528	0.32248	-0.477786526	0.156725	-0.124437989	NA	C5	-	-	-	Y	-	-	-
ENSG00000122335	SERAC1	0.479937915	5.18E-05	0.176878143	0.285642	0.329400191	0.009503	-0.296668908	0.018157	-0.136158783	0.299811	0.154974547	0.426914	C5	-	Y	-	Y	-	-	-
ENSG00000122359	ANXA11	-0.489880486	1.26E-09	-0.256810308	0.007327	-0.17675946	0.054584	0.240056087	0.012609	0.327774902	0.000129	0.082890631	0.611414	C4	-	Y	-	-	-	Y	-
ENSG00000122367	LDB3	-1.023331923	0.019535	-0.355149552	0.588681	-0.721361754	0.15759	0.676372256	0.255162	0.317149351	0.637056	-0.364886346	NA	C3	-	Y	-	-	-	-	-
ENSG00000122386	ZNF205	0.298680921	0.043644	-0.224690464	0.333593	0.145453715	0.449409	-0.517055208	0.001369	-0.138102285	0.389885	0.372993018	0.098285	C5	-	-	-	-	Y	-	-

ENSG00000122420	PTGFR	-1.5314293	0.001841	-1.421329749	0.008276	-0.551851749	0.289762	0.116451264	0.906169	0.994865032	0.109718	0.872746858	NA	C3	-	Y	Y	-	-	-	-
ENSG00000122435	TRMT13	0.459592033	1.16E-10	0.089329694	0.453707	0.21508901	0.010992	-0.364448	1.00E-09	-0.229988491	2.21E-05	0.129097336	0.170135	C5	-	Y	-	-	Y	-	-
ENSG00000122484	RPAP2	0.425098049	8.79E-05	0.178956853	0.29165	0.30410938	0.017606	-0.240420557	0.032003	-0.106626414	0.329897	0.128467342	0.521339	C5	-	Y	-	Y	-	-	-
ENSG00000122507	BBS9	-0.677043737	7.31E-10	-0.097854662	0.574502	-0.146932623	0.311966	0.585897573	2.38E-06	0.544822651	7.94E-06	-0.046210097	0.87983	C4	-	Y	-	-	Y	Y	-
ENSG00000122512	PMS2	0.309338939	2.51E-05	0.103437235	0.273241	0.219829676	0.003333	-0.19955729	0.003549	-0.074912114	0.329461	0.118907852	0.1995	C5	-	Y	-	-	-	-	-
ENSG00000122547	EEPD1	-0.856807012	3.37E-05	-0.416569885	0.053284	-0.069990508	0.794736	0.446528673	0.078404	0.801763921	0.000207	0.349605726	0.214048	C4	-	Y	-	-	-	Y	-
ENSG00000122550	KLHL7	0.42111272	1.49E-14	0.184740348	0.009458	0.226274091	0.000278	-0.229935142	5.51E-05	-0.180199462	0.001132	0.044278713	0.714719	C5	-	Y	-	-	-	-	-
ENSG00000122584	NXPH1	-0.88898991	0.035356	-1.118798219	0.019492	-0.976010219	0.043204	-0.22401726	0.719097	-0.072708048	0.916074	0.146461254	NA	C3	-	Y	Y	Y	-	-	-
ENSG00000122585	NPY	0.246474194	0.158021	0.482202856	0.014367	0.520816478	0.001398	0.243158503	0.134037	0.289347557	0.034215	0.040614704	0.912195	C1	-	-	Y	Y	-	-	-
ENSG00000122641	INHBA	0.695975955	0.033655	-0.058649269	0.933641	0.029002152	0.964273	-0.746351991	0.041217	-0.651993903	0.065506	0.090678271	NA	C5	-	Y	-	-	Y	-	-
ENSG00000122644	ARL4A	-0.229250025	0.159348	0.079766624	0.667307	-0.159357586	0.298061	0.315313572	0.048511	0.084530285	0.675784	-0.23624438	0.19895	C4	-	-	-	-	Y	-	-
ENSG00000122678	POLM	0.494247306	6.74E-08	0.295353045	0.010242	0.288411839	0.007481	-0.192219705	0.059689	-0.191021286	0.050904	-0.004069539	0.988432	C5	-	Y	-	-	-	-	-
ENSG00000122679	RAMP3	-1.134840233	0.011407	-0.756214406	0.131512	-0.746918186	0.116552	0.387268183	0.54077	0.403520788	0.515668	0.011919741	NA	C3	-	Y	-	-	-	-	-
ENSG00000122694	GLIPR2	-0.817167809	3.86E-13	-0.424202915	0.001116	-0.302730736	0.012484	0.399757582	0.004231	0.529312615	2.01E-05	0.124505854	0.591359	C4	-	Y	Y	Y	Y	Y	-
ENSG00000122735	DNAI1	-1.248673148	4.13E-05	-0.553978776	0.104821	-0.249159822	0.474061	0.702446377	0.05472	1.014293399	0.00131	0.307998409	NA	C4	-	Y	-	-	-	Y	-
ENSG00000122741	DCAF10	0.508258539	1.46E-11	0.259766492	0.003669	0.271759331	0.001445	-0.242334888	1.48E-05	-0.222014524	8.36E-05	0.014887547	0.925998	C1	-	Y	-	-	-	-	-
ENSG00000122756	CNTFR	-0.524095469	2.37E-24	-0.176107406	0.006508	-0.1527815	0.014448	0.354426331	7.31E-15	0.386318982	4.32E-19	0.026482055	0.800737	C4	-	Y	-	-	Y	Y	-
ENSG00000122778	KIAA1549	0.61463651	9.51E-24	0.485316275	6.44E-12	0.496577554	1.74E-13	-0.122744124	0.010115	-0.10351856	0.023927	0.013951449	0.922486	C1	-	Y	Y	Y	-	-	-
ENSG00000122882	ECD	0.21626982	0.005223	-0.042491875	0.742452	-0.116962647	0.224142	-0.252228782	0.000275	-0.318632208	1.47E-06	-0.071830796	0.582193	C5	Y	-	-	-	-	Y	-
ENSG00000122884	P4HA1	0.303585871	8.16E-05	0.551292888	2.50E-06	0.529530225	1.85E-09	0.254250676	0.021886	0.240976659	0.004343	-0.019309272	0.951002	C4	-	Y	Y	Y	-	-	-
ENSG00000122971	ACADS	-0.413606845	0.048545	-0.087586855	0.788471	-0.015891935	0.967124	0.332275205	0.169788	0.412602534	0.104823	0.075031756	0.89675	C4	-	Y	-	-	-	-	-
ENSG00000123066	MED13L	0.313115438	0.000135	0.330715882	0.000306	0.127169318	0.214277	0.023941198	0.7685	-0.171603516	0.002131	-0.200863986	0.004543	C1	-	Y	Y	-	-	-	-
ENSG00000123080	CDKN2C	-0.302387112	4.05E-07	-0.119817432	0.182302	-0.256358109	0.00012	0.189115971	0.004509	0.060956292	0.39963	-0.133700386	0.168713	C4	-	Y	-	-	-	-	-
ENSG00000123096	SSPN	-0.893583692	0.000572	-0.16119395	0.64799	-0.28306206	0.354202	0.737589677	0.009108	0.624967558	0.036453	-0.118707971	0.83038	C2	-	Y	-	-	Y	Y	-
ENSG00000123104	ITPR2	-0.414642858	0.059209	0.138767991	0.688851	0.380075868	0.122666	0.559123673	0.007924	0.809210314	1.84E-05	0.244427594	0.502302	C4	-	-	-	-	Y	Y	-
ENSG00000123119	NECAB1	-0.477153009	0.000827	0.024752261	0.921424	-0.069844346	0.743985	0.508514018	0.001184	0.422208344	0.009519	-0.091320031	0.778269	C4	-	Y	-	-	Y	Y	-
ENSG00000123131	PRDX4	-0.314789982	4.98E-05	-0.11719656	0.286993	-0.137817733	0.104878	0.204278386	0.026535	0.191792916	0.018568	-0.017588161	0.936471	C4	-	Y	-	-	-	-	-
ENSG00000123146	ADGRE5	-0.3394313	0.046151	-0.298997599	0.134698	-0.026514017	0.932358	0.047114402	0.848911	0.328078934	0.111987	0.274870971	0.368432	C4	-	Y	-	-	-	-	-
ENSG00000123191	ATP7B	0.336147537	0.005882	0.304720133	0.033914	0.397082305	0.003105	-0.024882561	0.881921	0.075657509	0.611671	0.095237644	0.679753	C4	-	Y	Y	Y	-	-	-
ENSG00000123200	ZC3H13	0.311192923	1.44E-07	0.110343335	0.167077	0.124225056	0.079919	-0.1946229	4.52E-07	-0.172618727	1.63E-06	0.016667451	0.860915	C5	-	Y	-	-	-	-	-
ENSG00000123240	OPTN	-0.282793125	0.012481	-0.024817655	0.908112	0.053756362	0.744302	0.264871611	0.049493	0.351302709	0.002458	0.081270833	0.75144	C4	-	-	-	-	-	Y	-
ENSG00000123352	SPATS2	0.388571734	2.04E-09	0.139138149	0.113388	0.160142715	0.048392	-0.242930203	3.21E-05	-0.213746553	0.00026	0.023796516	0.876139	C5	-	Y	-	-	-	-	-
ENSG00000123358	NR4A1	0.494059581	0.000202	0.287793985	0.091587	0.288751041	0.096573	-0.198505504	0.104055	-0.189967779	0.151996	0.003584628	0.992209	C5	-	Y	-	-	-	-	-
ENSG00000123415	SMUG1	-0.358245516	1.18E-07	-0.105584384	0.246341	-0.039403462	0.747019	0.259119053	0.000272	0.333474848	1.57E-05	0.068884305	0.649851	C4	-	Y	-	-	-	Y	-
ENSG00000123444	KBTBD4	-0.106961058	0.445577	-0.089149802	0.573795	-0.325863376	0.013886	0.024180113	0.888297	-0.204292027	0.15334	-0.233675566	0.180066	C5	-	-	-	Y	-	-	-
ENSG00000123453	SARDH	-0.970150056	7.91E-17	-0.326727922	0.012563	-0.4813283	7.08E-05	0.650493035	1.38E-06	0.503301164	0.000371	-0.152288096	0.503848	C2	-	Y	Y	Y	Y	Y	-
ENSG00000123454	DBH	0.212589057	1.68E-07	-0.174120543	0.022506	-0.218516794	0.007852	-0.380220093	3.62E-14	-0.416509654	4.81E-06	-0.041203649	0.880818	C5	-	-	-	-	Y	Y	-
ENSG00000123496	IL13RA2	-0.72991088	0.272017	0.511365844	0.496752	0.408074006	0.544463	1.24719651	0.028087	1.152736041	0.031856	-0.09915913	NA	C2	-	-	-	-	Y	Y	-
ENSG00000123545	NDUFAF4	0.363525227	6.45E-05	0.094008562	0.53651	0.044643843	0.756508	-0.262908567	0.002981	-0.304280388	0.000102	-0.046952306	0.815747	C5	-	Y	-	-	-	Y	-
ENSG00000123552	USP45	0.329634041	0.001119	0.314411456	0.004649	0.24871267	0.024945	-0.008837239	0.951805	-0.06645348	0.573521	-0.063018025	0.73505	C5	-	Y	Y	-	-	-	-
ENSG00000123575	FAM199X	0.419613016	1.18E-08	0.110224264	0.289233	0.238644342	0.000305	-0.30256479	0.0002	-0.166439462	0.020219	0.131453043	0.2208	C5	-	Y	-	-	Y	-	-
ENSG00000123595	RAB9A	0.423063727	0.008355	0.051410655	0.844145	0.173090358	0.369909	-0.365175817	0.0299	-0.234967365	0.178651	0.124672131	0.666588	C5	-	Y	-	-	Y	-	-
ENSG00000123600	METTL8	0.345490837	0.000799	0.226459209	0.073444	0.13667766	0.288752	-0.112542113	0.368285	-0.194185678	0.072568	-0.086760999	0.6558	C5	-	Y	-	-	-	-	-
ENSG00000123643	SLC36A1	0.301133981	0.002305	0.373655906	0.003259	0.34916068	0.00369	0.078866257	0.557547	0.062931449	0.636329	-0.021757287	0.944732	C4	-	Y	Y	Y	-	-	-
ENSG00000123836	PFKFB2	-0.202669205	0.118166	0.099546636	0.565922	-0.064076556	0.746845	0.309174617	0.018353	0.153390438	0.346718	-0.161866232	0.494132	C4	-	-	-	-	Y	-	-
ENSG00000123908	AGO2	0.488824994	0.000262	0.273549551	0.012884	0.255771077	0.0081	-0.208836325	0.078282	-0.218669798	0.000997	-0.015059074	0.936471	C5	-	Y	-	-	-	-	-
ENSG00000124126	PREX1	-0.489109644	0.000225	-0.254746087	0.077472	-0.111454464	0.491637	0.240869214	0.113914	0.392117815	0.004321	0.146079912	0.495473	C4	-	Y	-	-	-	Y	-
ENSG00000124145	SDC4	-0.428469823	0.007344	-0.034575708	0.890586	-0.047906273	0.834072	0.400605512	0.012477	0.395565641	0.013665	-0.010252954	0.979338	C4	-	Y	-	-	Y	Y	-
ENSG00000124151	NCOA3	0.384278643	0.000193	0.245143207	0.043537	0.201788523	0.11965	-0.132474458	0.152497	-0.168153383	0.081962	-0.040966683	0.843922	C1	-	Y	-	-	-	-	-
ENSG00000124160	NCOA5	-0.33119397	1.06E-07	-0.330892061	3.41E-06	-0.388322588	2.73E-10	0.006679422	0.952782	-0.042413164	0.622632	-0.054429401	0.696318	C4	-	Y	Y	Y	-	-	-
ENSG00000124171	PARD6B	0.422831727	0.040891	-0.136916057	0.700789	-0.360443236	0.207681	-0.553052382	0.013481	-0.768523567	0.000609	-0.219706051	0.647102	C5	-	Y	-	-	Y	Y	-
ENSG00000124191	TOX2	-0.78751192	1.14E-05	-0.237145358	0.360542	-0.055064484	0.840934	0.556693727	0.010543	0.747340972	7.73E-05	0.185268294	0.605894	C3	-	Y	-	-	Y	Y	-
ENSG00000124193	SRSF6	-0.193482777	3.32E-08	-0.175949978	0.001095	-0.333505494	2.40E-13	0.024162733	0.659659	-0.125434208	0.001468	-0.154648821	0.016726	C4	-	-	-	Y	-	-	-
ENSG00000124212	PTGIS	-1.20194708	1.05E-07	-0.604908752	0.004482	-0.41977611	0.060516	0.603119857	0.020839	0.796922418	0.00144	0.187901206	0.621746	C3	-	Y	Y	-	Y	Y	-
ENSG00000124214	STAU1	0.335841565	4.68E-18	0.099009018	0.076668	0.080914552	0.108828	-0.23021929	1.78E-09	-0.240188499	3.86E-12	-0.015354927	0.883392	C5	Y	Y	-	-	-	-	-
ENSG00000124228	DDX27	0.267579959	1.68E-07	0.021023623	0.840748	-0.060521357	0.473537	-0.240336093	5.35E-06	-0.313662379	1.85E-09	-0.078746341	0.470464	C5	-	-	-	-	-	Y	-
ENSG00000124243	BCAS4	-0.523809246	8.42E-05	-0.206794079	0.214508	-0.005636874	0.981068	0.323787112	0.033823	0.533148027	0.000212	0.203893626	0.355837	C4	-	Y	-	-	Y	Y	-
ENSG00000124260	MAGEA10	-1.279795264	0.000173	-0.880187451	0.019539	-1.081776243	0.003851	0.40845659	0.4234	0.212604487	0.723908	-0.200838321	NA	C3	-	Y	Y	Y	-	-	-
ENSG00000124275	MTRR	0.247053613	0.006111	-0.181503637	0.136225	0.069080758	0.608596	-0.421902742	3.65E-07	-0.163720902	0.063996	0.253053907	0.030437	C5	-	-	-	-	Y	-	-
ENSG00000124299	PEPD	-0.220191271	0.0018	0.115060808	0.186085	-0.073018672	0.414738	0.341719473	2.02E-09	0.162102325	0.006431	-0.184867651	0.00547	C4	-	-	-	-	Y	-	-
ENSG00000124302	CHST8	0.035918499	0.799176	-0.025332023	0.882656	-0.333865491	0.003034	-0.054641775	0.68692	-0.354968864	0.001005	-0.305587961	0.017235	C4	-	-	-	Y	-	Y	Y
ENSG00000124313	IQSEC2	0.491359392	0.007722	0.285822496	0.256146	0.488598235	0.012042	-0.199156966	0.330143	0.011695924	0.959992	0.204919469	0.480388	C1	-	Y	-	Y	-	-	-
ENSG00000124357	NAGK	0.323510113	0.000183	-0.066622498	0.636044	0.073123665	0.521675	-0.383713901	1.18E-05	-0.235913703	0.004094	0.142420984	0.282245	C5	Y	Y	-	-	Y	-	-
ENSG00000124374	PAIP2B	-0.168170233	0.351049	-0.062533225	0.818642	0.145432478	0.469156	0.11256514	0.604511	0.328196088	0.049454	0.210789362	0.431428	C4	-	-	-	-	-	Y	-
ENSG00000124380	SNRNP27	0.323427104	3.26E-05	0.003151832	0.986847	-0.032967169	0.821471	-0.312420565	3.71E-06	-0.341864669	1.20E-07	-0.033814451	0.861463	C5	-	Y	-	-	Y	Y	-
ENSG00000124383	MPHOSPH10	0.14908323	0.025909	-0.033402513	0.751381	-0.171794944	0.022941	-0.17610106	0.006397	-0.306392703	1.32E-06	-0.135467485	0.16145	C5	-	-	-	-	-	Y	-
ENSG00000124406	ATP8A1	-0.396976989	3.05E-09	-0.04014026	0.731636	0.12617415	0.1398	0.363088076	1.95E-14	0.53776551	3.97E-32	0.169284962	0.010298	C4	-	Y	-	-	Y	Y	-

ENSG00000124440	HIF3A	-0.594080137	0.009107	-0.335920797	0.210746	0.345947147	0.129811	0.265884665	0.363911	0.954674352	1.07E-05	0.684697855	0.006515	C4	-	Y	-	-	-	Y	Y
ENSG00000124496	TRERF1	-0.315355785	0.000378	0.015986526	0.92564	0.141371906	0.202456	0.337968279	0.000573	0.471529021	2.10E-07	0.128174753	0.434225	C4	-	Y	-	-	Y	Y	-
ENSG00000124507	PACSIN1	-1.708567433	0.001053	-0.46310199	0.439351	-0.727558935	0.154916	1.259268752	0.047551	0.995400279	0.140695	-0.264306908	NA	C3	-	Y	-	-	Y	-	-
ENSG00000124508	BTN2A2	-0.784650941	4.23E-11	-0.418298935	0.002183	-0.459566335	0.000496	0.372291155	0.009017	0.339824917	0.020633	-0.038161802	0.914963	C4	-	Y	Y	Y	Y	Y	-
ENSG00000124523	SIRT5	-0.302086802	0.020658	-0.010588598	0.965777	0.262359227	0.045036	0.298017799	0.031333	0.579333309	5.99E-07	0.276151786	0.089669	C4	-	Y	-	-	-	Y	-
ENSG00000124549	BTN2A3P	-0.931872923	0.005964	0.216283093	0.607926	-0.041344582	0.861024	1.155980459	0.000786	0.864927409	0.0196	-0.29494361	NA	C3	-	Y	-	-	Y	Y	-
ENSG00000124557	BTN1A1	-1.963035256	0.048042	-1.154393979	NA	-2.042479325	NA	0.808134452	NA	-0.057187914	NA	-0.871611114	NA	C3	-	Y	-	-	-	-	-
ENSG00000124570	SERPINB6	-0.386540282	2.73E-10	-0.191092319	0.020428	-0.126407141	0.086237	0.202008823	0.005844	0.274959156	6.97E-06	0.067830622	0.596074	C4	-	Y	-	-	-	-	-
ENSG00000124571	XPO5	0.458058508	5.20E-18	0.307183357	3.06E-07	0.209827341	0.00075	-0.144296801	0.002084	-0.233813839	9.86E-08	-0.094682445	0.1995	C1	Y	Y	Y	-	-	-	-
ENSG00000124574	ABCC10	0.298598341	0.002471	0.155251665	0.293205	0.318915043	0.003463	-0.136670728	0.25124	0.03478295	0.791248	0.166425646	0.317364	C4	-	-	-	Y	-	-	-
ENSG00000124587	PEX6	0.113823461	0.262546	0.142618252	0.227207	0.384986472	4.46E-05	0.035106365	0.758664	0.286001344	0.000426	0.245440505	0.019107	C4	-	-	-	Y	-	-	-
ENSG00000124588	NQO2	-0.403925194	0.001541	-0.257778163	0.08937	-0.256920547	0.077862	0.15260591	0.346884	0.161814866	0.313242	0.003811299	0.992111	C4	-	Y	-	-	-	-	-
ENSG00000124596	OARD1	-0.647490317	7.55E-19	-0.147163057	0.072481	-0.299589771	3.21E-05	0.506925192	7.90E-12	0.362614332	2.96E-06	-0.149540829	0.128594	C4	-	Y	-	-	Y	Y	-
ENSG00000124659	TBCC	-0.120773609	0.365185	-0.426546312	0.001571	-0.356514491	0.008447	-0.297968583	0.00557	-0.221020437	0.050198	0.072326457	0.768002	C5	-	-	Y	Y	-	-	-
ENSG00000124762	CDKN1A	-0.180859336	0.009686	0.227229168	0.009535	0.021821117	0.880649	0.415225132	7.22E-13	0.217241494	0.002426	-0.202365811	0.066193	C4	-	-	-	-	Y	-	-
ENSG00000124784	RIOK1	0.264799375	4.01E-06	-0.059766677	0.523089	-0.041418666	0.661258	-0.317961565	7.72E-09	-0.291527397	1.01E-07	0.021158779	0.89024	C5	-	-	-	-	Y	-	-
ENSG00000124785	NRN1	0.37062906	0.003845	0.186462369	0.273753	0.368442332	0.007294	-0.177769057	0.230055	0.01245538	0.947332	0.184940696	0.36625	C5	-	Y	-	Y	-	-	-
ENSG00000124802	EEF1E1	-0.099753556	0.453989	-0.071823605	0.69363	-0.336948412	0.006633	0.034994226	0.818129	-0.222166781	0.051372	-0.262464426	0.077977	C4	-	-	-	Y	-	-	-
ENSG00000124920	MYRF	-0.699076789	4.22E-07	-0.279760493	0.029182	-0.128716165	0.490365	0.427159091	0.005277	0.584821934	0.000353	0.152519845	0.554872	C4	-	Y	-	-	Y	Y	-
ENSG00000125046	SSUH2	-1.001658481	9.18E-12	-0.534441751	0.000254	-0.547007993	0.000112	0.473420145	0.007468	0.46954913	0.007879	-0.00952239	0.982152	C3	-	Y	Y	Y	Y	Y	-
ENSG00000125107	CNOT1	0.411458483	3.48E-07	0.274526665	0.005295	0.177826911	0.073243	-0.13095992	0.003975	-0.219192364	1.83E-08	-0.093665338	0.203996	C1	-	Y	-	-	-	-	-
ENSG00000125122	LRRC29	-1.201265358	0.00435	-0.36745937	0.440769	-0.368381751	0.379026	0.841442006	0.108016	0.848464667	0.085237	0.001918322	NA	C3	-	Y	-	-	-	-	-
ENSG00000125124	BBS2	0.213309475	0.011864	0.276794075	0.001698	0.37005058	3.50E-06	0.069845075	0.493951	0.171615628	0.041186	0.096160006	0.479368	C4	-	-	-	Y	-	-	-
ENSG00000125148	MT2A	-0.98373675	9.43E-05	-0.462285252	0.135352	-0.444690403	0.122986	0.528884407	0.07442	0.554297563	0.053369	0.020959436	0.977582	C3	-	Y	-	-	-	-	-
ENSG00000125149	C16orf70	0.302757118	0.004851	0.187700515	0.121829	0.284453091	0.006894	-0.108734797	0.365648	-0.003727073	0.977988	0.099615478	0.541217	C5	Y	Y	-	-	-	-	-
ENSG00000125170	DOK4	-0.143354432	0.002111	0.231271803	4.48E-06	0.145097899	0.005716	0.381321394	2.05E-32	0.303290732	2.79E-23	-0.083291876	0.095679	C4	-	-	-	-	Y	Y	-
ENSG00000125207	PIWIL1	-0.83750685	2.61E-15	-0.388296204	0.000523	-0.27559156	0.010593	0.45547424	2.67E-05	0.576318479	9.21E-09	0.115193658	0.487266	C4	-	Y	Y	-	Y	Y	-
ENSG00000125249	RAP2A	0.352324451	1.45E-08	0.156268639	0.074402	0.177338335	0.031449	-0.189503501	0.00136	-0.160177571	0.008391	0.023895537	0.888988	C5	Y	Y	-	-	-	-	-
ENSG00000125351	UPF3B	0.21716939	0.003272	-0.097272177	0.367695	-0.08962807	0.358467	-0.308192394	2.16E-05	-0.292297114	1.94E-05	0.010475367	0.960208	C5	-	-	-	-	Y	-	-
ENSG00000125352	RNF113A	0.606020906	1.41E-06	0.1736303	0.391125	0.219979468	0.231859	-0.425350496	0.000642	-0.37159634	0.003591	0.049013005	0.889237	C5	-	Y	-	-	Y	Y	-
ENSG00000125375	DMAC2L	-0.479659667	3.00E-05	0.14093371	0.346673	-0.18115186	0.173767	0.627333105	4.28E-08	0.313136211	0.011368	-0.319107342	0.023012	C4	-	Y	-	-	Y	Y	Y
ENSG00000125388	GRK4	0.511284491	0.000579	0.337418002	0.060161	0.275777316	0.12677	-0.167614964	0.365853	-0.220804893	0.207817	-0.058448228	0.880775	C5	-	Y	-	-	-	-	-
ENSG00000125398	SOX9	0.624057066	0.012721	0.637874736	0.023289	0.662512626	0.031243	0.020275581	0.954879	0.05270526	0.890163	0.027569462	0.969121	C1	-	Y	Y	Y	-	-	-
ENSG00000125434	SLC25A35	-0.529177962	0.013221	-0.002761789	0.994903	-0.12829413	0.668874	0.533767423	0.026917	0.415794754	0.088933	-0.122863727	0.801122	C4	-	Y	-	-	Y	-	-
ENSG00000125449	ARMC7	0.239444156	0.049766	-0.167826037	0.323589	0.121959091	0.438533	-0.400788634	0.001078	-0.102831131	0.457485	0.292578322	0.082316	C5	-	-	-	-	Y	-	-
ENSG00000125454	SLC25A19	0.691627795	1.26E-05	0.208848016	0.480128	0.30396983	0.175844	-0.475649805	0.004634	-0.373339424	0.01344	0.097492265	0.818037	C5	-	Y	-	-	Y	Y	-
ENSG00000125457	MIF4GD	-0.387552906	0.002484	-0.143546418	0.401902	0.019804358	0.926418	0.250590694	0.064488	0.422303402	0.000734	0.166395136	0.403164	C4	Y	Y	-	-	-	Y	-
ENSG00000125458	NT5C	-0.272204527	0.025321	-0.161631968	0.330266	-0.357647239	0.006474	0.116438149	0.319665	-0.070226007	0.56547	-0.192034328	0.196227	C4	-	-	-	Y	-	-	-
ENSG00000125482	TTF1	-0.149741605	0.030166	-0.129836002	0.020117	-0.347272201	3.20E-07	-0.053525293	0.573895	-0.182861915	0.00668	-0.13478122	0.248512	C4	-	-	-	Y	-	-	-
ENSG00000125485	DDX31	0.512373755	1.05E-07	0.069126159	0.689634	0.187682607	0.147796	-0.436608075	3.50E-08	-0.310346144	0.000113	0.121117949	0.426996	C5	-	Y	-	-	Y	Y	-
ENSG00000125505	MBOAT7	0.073185319	0.536899	0.167494694	0.215144	0.341173234	0.000163	0.10030509	0.371195	0.28256086	0.00017	0.177118622	0.157559	C4	-	-	-	Y	-	-	-
ENSG00000125510	OPRL1	-0.60793049	0.144842	0.255660612	0.604989	0.363093137	0.384663	0.868894615	0.031626	0.985623509	0.010494	0.110075771	NA	C2	-	-	-	-	Y	Y	-
ENSG00000125520	SLC2A4RG	-0.329566777	0.000184	-0.132821534	0.2618	-0.049272485	0.716988	0.202980584	0.012327	0.295403733	0.000139	0.08695047	0.532103	C4	-	Y	-	-	-	-	-
ENSG00000125531	FNDC11	-0.045923161	0.906893	0.128054882	0.76455	0.428711172	0.142588	0.179930449	0.542785	0.490262248	0.035018	0.305744928	0.373578	C1	-	-	-	-	-	Y	-
ENSG00000125534	PPDPF	-0.311613896	0.010769	-0.206256836	0.205492	-0.117338767	0.448446	0.11171328	0.302209	0.209689079	0.004352	0.093247376	0.537085	C4	-	Y	-	-	-	-	-
ENSG00000125551	PLGLB2	1.353529551	0.005722	0.735451412	0.321088	0.895231976	0.140948	-0.61584772	0.221165	-0.444200127	0.357606	0.166329486	NA	C1	-	Y	-	-	-	-	-
ENSG00000125611	CHCHD5	-0.560325736	8.49E-05	-0.135093966	0.482651	-0.213500459	0.235347	0.431962812	0.001317	0.362121884	0.015253	-0.075118393	0.790777	C4	-	Y	-	-	Y	Y	-
ENSG00000125618	PAX8	4.54239596	0.012733	3.840056542	NA	4.024801864	NA	-0.705699164	0.850862	-0.503576921	0.780454	0.193153441	NA	C5	-	Y	-	-	-	-	-
ENSG00000125629	INSIG2	0.449534155	1.23E-07	0.189454927	0.066501	0.361341097	2.00E-05	-0.253828518	0.002239	-0.073740928	0.432252	0.174956762	0.098285	C5	-	Y	-	Y	-	-	-
ENSG00000125630	POLR1B	0.485632684	5.12E-12	0.067511113	0.563915	0.261280973	0.001021	-0.411612399	3.66E-19	-0.21002155	1.89E-06	0.196328212	0.001204	C5	-	Y	-	-	Y	-	-
ENSG00000125633	CCDC93	0.758580519	4.26E-27	0.349271485	1.27E-05	0.44101551	9.03E-10	-0.402971877	2.36E-10	-0.303227013	4.71E-07	0.094541769	0.374258	C5	-	Y	Y	Y	Y	Y	-
ENSG00000125675	GRIA3	-0.358667847	0.006295	0.148957219	0.438232	0.326820933	0.036803	0.513739132	7.60E-05	0.700275359	3.26E-08	0.180208343	0.45687	C4	-	Y	-	Y	Y	Y	-
ENSG00000125676	THOC2	0.34317709	3.21E-07	0.176261306	0.04483	0.152489305	0.060513	-0.160400987	0.000573	-0.176202475	1.86E-05	-0.021181552	0.854084	C5	-	Y	-	-	-	-	-
ENSG00000125686	MED1	0.382777174	3.26E-07	0.146607376	0.178585	0.104187358	0.313492	-0.230210569	2.95E-05	-0.264304506	5.79E-09	-0.039361034	0.759011	C1	-	Y	-	-	-	-	-
ENSG00000125703	ATG4C	0.542237772	5.93E-06	0.420811783	0.009988	0.451748937	0.000574	-0.115061544	0.512186	-0.075588827	0.637531	0.033560418	0.930809	C1	-	Y	Y	Y	-	-	-
ENSG00000125733	TRIP10	0.453398904	3.95E-08	0.239276074	0.021799	0.456627577	6.61E-07	-0.207636156	0.016014	0.017952686	0.883576	0.220250898	0.054549	C1	-	Y	-	Y	-	-	-
ENSG00000125746	EML2	0.310243455	0.012799	0.333586166	0.040622	0.473857678	0.000194	0.030196295	0.863577	0.178720762	0.120421	0.143306596	0.503233	C4	-	Y	Y	Y	-	-	-
ENSG00000125753	VASP	0.681987009	8.94E-13	0.562376836	6.82E-07	0.581289427	1.17E-08	-0.11346751	0.326017	-0.085888656	0.432252	0.02220985	0.933843	C1	-	Y	Y	Y	-	-	-
ENSG00000125772	GPCPD1	-0.654012326	3.73E-07	-0.097454229	0.636533	-0.224139624	0.147796	0.56324134	2.50E-05	0.444745025	0.000798	-0.123808922	0.610727	C2	-	Y	-	-	Y	Y	-
ENSG00000125775	SDCBP2	0.787555684	0.033064	0.387581136	0.45557	-0.374994369	0.529225	-0.393700668	0.334598	-1.147842724	0.004435	-0.759284406	NA	C5	-	Y	-	-	-	Y	-
ENSG00000125804	FAM182A	-2.344823835	0.042673	-0.835895002	NA	-0.242754099	NA	1.512192793	NA	2.114194599	0.104553	0.595138984	NA	C3	-	Y	-	-	-	-	-
ENSG00000125812	GZF1	-0.043967617	0.714034	-0.307937953	0.002971	-0.11094272	0.335872	-0.257400272	0.009612	-0.052098759	0.664973	0.199926648	0.132933	C4	-	-	Y	-	-	-	-
ENSG00000125827	TMX4	-0.286461671	8.43E-07	0.130477752	0.134972	-0.247002099	0.000207	0.42345042	4.91E-11	0.054266493	0.481659	-0.374679893	8.12E-07	C4	-	-	-	-	Y	-	Y
ENSG00000125841	NRSN2	-0.630285149	1.78E-24	-0.152857258	0.055208	-0.22811733	0.002646	0.483818294	3.91E-14	0.416731781	4.40E-10	-0.072531254	0.577523	C4	-	Y	-	-	Y	Y	-
ENSG00000125844	RRBP1	-0.269640704	0.002152	-0.058954271	0.690582	0.082276207	0.476113	0.217259684	0.03054	0.366694057	1.72E-05	0.144303498	0.314217	C4	-	-	-	-	-	Y	-
ENSG00000125846	ZNF133	0.139888326	0.229879	-0.1699603	0.19249	0.041536201	0.80458	-0.303397825	0.003094	-0.08338447	0.514487	0.214520357	0.146675	C5	-	-	-	-	Y	-	-
ENSG00000125869	LAMP5	-1.313386961	5.89E-42	-0.438627021	0.000108	-0.733771064	1.31E-11	0.881696767	6.80E-15	0.594464718	1.05E-06	-0.29267398	0.085494	C3	-	Y	Y	Y	Y	Y	-

ENSG00000125895	TMEM74B	-0.785501832	0.002046	-0.165954099	0.590895	-0.112272743	0.712781	0.626492783	0.020923	0.687960887	0.008735	0.056765045	0.916086	C2	-	Y	-	-	Y	Y	-
ENSG00000125945	ZNF436	0.221187221	0.015238	-0.151857054	0.233025	0.023314249	0.886138	-0.366795823	6.43E-05	-0.183018234	0.060373	0.178040621	0.238504	C5	-	-	-	-	Y	-	-
ENSG00000125952	MAX	0.493859529	3.14E-12	0.093601196	0.313135	0.129019641	0.224006	-0.393579659	1.41E-10	-0.350177105	1.92E-06	0.038264897	0.825092	C5	-	Y	-	-	Y	Y	-
ENSG00000125962	ARMCX5	-0.539453605	5.48E-06	-0.160759829	0.293364	-0.497749818	0.000188	0.384558176	0.003834	0.056421769	0.780031	-0.33352618	0.069901	C4	-	Y	-	Y	Y	-	-
ENSG00000125967	NECAB3	-0.36275897	0.003405	-0.295123176	0.048842	-0.204904095	0.191226	0.073635277	0.625157	0.17312957	0.188139	0.093695521	0.693626	C4	-	Y	-	-	-	-	-
ENSG00000125968	ID1	0.289939309	3.41E-06	0.411821889	8.71E-07	0.03192264	0.811268	0.128720948	0.049122	-0.24309565	9.69E-05	-0.376868837	2.40E-05	C4	Y	-	Y	-	-	-	Y
ENSG00000125977	EIF2S2	-0.484368455	2.78E-16	-0.229830259	0.001429	-0.210748373	0.004124	0.261207764	1.50E-05	0.288282994	1.45E-06	0.022029241	0.897588	C4	-	Y	-	-	-	-	-
ENSG00000126001	CEP250	0.328464324	3.48E-10	0.196624086	0.00297	0.236633708	0.000119	-0.125514821	0.01932	-0.077019453	0.175816	0.042989315	0.691382	C5	-	Y	-	-	-	-	-
ENSG00000126003	PLAGL2	0.284439317	0.00016	-0.226830907	0.065146	-0.089793165	0.44628	-0.504606514	5.36E-08	-0.359486208	6.64E-06	0.139929748	0.454595	C5	-	-	-	-	Y	Y	-
ENSG00000126005	MMP24OS	-0.311440183	0.011266	-0.016287861	0.944119	-0.013690338	0.954105	0.301324186	0.010982	0.313231503	0.015081	0.005635761	0.988297	C4	-	Y	-	-	Y	Y	-
ENSG00000126016	AMOT	-0.676963686	1.56E-06	-0.280975229	0.149454	0.000439601	0.998531	0.403421625	0.027257	0.691911319	3.00E-06	0.283886336	0.240869	C4	-	Y	-	-	Y	Y	-
ENSG00000126070	AGO3	0.456815028	1.12E-06	0.027245501	0.877893	0.075538103	0.512856	-0.422892967	5.45E-06	-0.36655224	3.22E-06	0.051282936	0.780349	C5	-	Y	-	-	Y	Y	-
ENSG00000126088	UROD	-0.20908073	0.011715	-0.197024131	0.02852	-0.359032997	2.79E-05	0.01863128	0.865888	-0.13515132	0.132502	-0.159073547	0.153062	C4	-	-	-	Y	-	-	-
ENSG00000126091	ST3GAL3	-0.232124359	0.366015	0.311825841	0.179311	0.352308665	0.08368	0.550711086	0.010515	0.599042176	0.003031	0.043532396	0.923224	C1	-	-	-	-	Y	Y	-
ENSG00000126216	TUBGCP3	0.283056468	1.11E-06	0.094050495	0.255066	-0.035621226	0.741593	-0.182586763	0.000877	-0.30416523	2.45E-07	-0.126890404	0.182394	C5	-	-	-	-	-	Y	-
ENSG00000126218	F10	-0.489901234	0.000161	-0.23840448	0.135079	-0.165530258	0.337632	0.258122566	0.07291	0.33960723	0.016368	0.07594435	0.790777	C4	-	Y	-	-	-	Y	-
ENSG00000126226	PCID2	0.556612047	2.79E-19	0.178567461	0.033154	0.234740738	0.002138	-0.37207864	4.91E-11	-0.307418852	4.44E-08	0.05923446	0.623921	C5	-	Y	-	-	Y	Y	-
ENSG00000126249	PDCD2L	0.35417022	0.045448	0.148685402	0.602503	0.065217693	0.817017	-0.19911041	0.350714	-0.274562745	0.144906	-0.0806342	0.854265	C5	-	Y	-	-	-	-	-
ENSG00000126351	THRA	-0.273359283	0.000239	0.007402229	0.96165	0.027699555	0.813206	0.287047076	9.38E-05	0.315901432	3.24E-06	0.023468903	0.899407	C4	-	-	-	-	-	Y	-
ENSG00000126368	NR1D1	0.373217735	0.0458	0.511884533	0.006796	0.211863412	0.356249	0.145173142	0.458748	-0.146329795	0.470296	-0.29691267	0.188277	C4	-	Y	Y	-	-	-	-
ENSG00000126453	BCL2L12	-0.482083809	0.02338	-0.140794196	0.642823	-0.017210587	0.95568	0.349396148	0.144139	0.480058571	0.015187	0.127866955	0.732439	C4	-	Y	-	-	-	Y	-
ENSG00000126458	RRAS	-0.91335541	0.000497	-0.43820375	0.180683	-0.125636912	0.701809	0.483418713	0.156055	0.802355301	0.002723	0.316200436	0.472263	C4	-	Y	-	-	-	Y	-
ENSG00000126522	ASL	-0.365349979	0.00117	-0.040909241	0.824312	-0.001852802	0.992582	0.331099048	0.002597	0.378597828	0.000477	0.042137065	0.862235	C4	-	Y	-	-	Y	Y	-
ENSG00000126524	SBDS	0.551596172	3.74E-16	0.208830768	0.023864	0.231617982	0.005315	-0.336245394	2.15E-06	-0.305480847	6.05E-06	0.025621458	0.899699	C5	-	Y	-	-	Y	Y	-
ENSG00000126561	STAT5A	-0.524666099	0.302127	-0.079345423	0.909425	-0.995309337	0.049991	0.452390298	0.414833	-0.456984794	0.474484	-0.913348349	NA	C2	-	-	-	Y	-	-	-
ENSG00000126562	WNK4	-0.685012622	0.330063	0.173542812	0.846476	0.545576706	0.398206	0.865798003	0.177019	1.246093308	0.028437	0.375542482	NA	C2	-	-	-	-	-	Y	-
ENSG00000126603	GLIS2	0.362392595	0.017004	0.283657805	0.109167	0.278837596	0.08036	-0.072346482	0.706645	-0.068719106	0.709737	-0.002641021	0.994924	C1	-	Y	-	-	-	-	-
ENSG00000126653	NSRP1	-0.295655212	0.003857	-0.213914292	0.083825	-0.338156811	0.002244	0.088878975	0.419966	-0.027364262	0.836104	-0.121641647	0.437239	C4	-	-	-	Y	-	-	-
ENSG00000126705	AHDC1	0.502851701	4.12E-10	0.364052676	3.49E-05	0.341406153	7.02E-05	-0.132465465	0.122811	-0.146896561	0.075916	-0.019677818	0.923363	C5	-	Y	Y	Y	-	-	-
ENSG00000126709	IFI6	-0.597722812	0.000107	-0.487162672	0.013654	-0.740435959	9.73E-05	0.118738241	0.643915	-0.127787471	0.634872	-0.253913639	0.514573	C4	-	Y	Y	Y	-	-	-
ENSG00000126767	ELK1	0.31368114	0.000243	0.048913586	0.733489	0.055600221	0.711851	-0.258394609	0.001601	-0.243559322	0.007978	0.009314896	0.972936	C5	-	Y	-	-	-	-	-
ENSG00000126775	ATG14	-0.476203954	3.57E-09	-0.503833555	2.50E-09	-0.423250033	7.58E-07	-0.021378623	0.872199	0.067706665	0.560939	0.08353444	0.620631	C4	Y	Y	Y	Y	-	-	-
ENSG00000126803	HSPA2	-0.547232354	2.14E-05	-0.123005912	0.498828	0.1246517	0.465241	0.430864876	0.001456	0.686499072	6.36E-08	0.250562097	0.168713	C4	-	Y	-	-	Y	Y	-
ENSG00000126821	SGPP1	0.348551624	0.00046	0.187808318	0.098783	0.079329484	0.577244	-0.154397787	0.136541	-0.254659702	0.011594	-0.105622415	0.519467	C5	-	Y	-	-	-	-	-
ENSG00000126858	RHOT1	0.488270494	5.93E-16	0.451140805	2.44E-13	0.45147483	4.01E-12	-0.030678738	0.690724	-0.022085486	0.7928	0.003219109	0.984988	C1	-	Y	Y	Y	-	-	-
ENSG00000126870	WDR60	0.309451054	1.18E-05	0.239965176	0.003959	0.194376169	0.019764	-0.063112918	0.465865	-0.100402251	0.204708	-0.042787054	0.781334	C5	-	Y	-	-	-	-	-
ENSG00000126878	AIF1L	-0.144833196	0.200103	-0.422326822	5.75E-05	-0.247414885	0.030807	-0.270698601	0.017317	-0.087909525	0.519916	0.17765436	0.294117	C4	-	-	Y	-	-	-	-
ENSG00000126945	HNRNPH2	-0.222790405	1.99E-07	-0.14061108	0.007671	-0.323978292	7.46E-12	0.088656569	0.065952	-0.086423693	0.085531	-0.180458591	0.001574	C4	-	-	-	Y	-	-	-
ENSG00000127084	FGD3	-2.653774999	1.24E-06	-2.244004437	6.89E-05	-1.06197938	0.061961	0.4314681	0.717576	1.609055806	0.046995	1.177098088	NA	C3	-	Y	Y	-	-	Y	-
ENSG00000127324	TSPAN8	-0.529810288	0.027677	-0.299236081	0.457565	-0.403923605	0.168314	0.237876352	0.536651	0.14025029	0.698607	-0.104471519	0.896861	C4	-	Y	-	-	-	-	-
ENSG00000127328	RAB3IP	0.522245983	1.85E-07	0.283435625	0.090672	0.277327735	0.030929	-0.232008743	0.073547	-0.230206463	0.02467	-0.003459548	0.992964	C1	-	Y	-	-	-	-	-
ENSG00000127337	YEATS4	-0.318445964	5.12E-05	-0.224084983	0.024271	-0.34508703	0.000146	0.100815795	0.324194	-0.011715251	0.929631	-0.118012286	0.455002	C4	-	Y	-	Y	-	-	-
ENSG00000127364	TAS2R4	0.456591863	0.29553	0.143763381	0.820706	0.789470813	0.047342	-0.30518086	0.529875	0.347161904	0.418956	0.64902188	NA	C1	-	-	-	Y	-	-	-
ENSG00000127415	IDUA	-0.162077237	0.293848	0.189440487	0.2456	0.036432066	0.855316	0.358150084	0.006544	0.213590434	0.128158	-0.150107526	0.452541	C4	-	-	-	-	Y	-	-
ENSG00000127481	UBR4	0.469920901	0.000219	0.334814149	0.000655	0.272737391	0.003727	-0.12866119	0.017497	-0.183109928	4.85E-05	-0.059743316	0.482463	C5	-	Y	Y	-	-	-	-
ENSG00000127526	SLC35E1	0.314165419	4.59E-06	0.146036653	0.115307	0.071467631	0.474062	-0.162043669	0.008207	-0.22823972	5.04E-05	-0.071535481	0.513241	C5	-	Y	-	-	-	-	-
ENSG00000127527	EPS15L1	0.438598978	1.20E-10	0.281641093	0.000464	0.306232253	6.63E-05	-0.150416082	0.040732	-0.117564941	0.119667	0.027469412	0.87281	C5	-	Y	-	Y	-	-	-
ENSG00000127554	GFER	0.068884959	0.650452	-0.198784788	0.264443	-0.357766949	0.01076	-0.260467262	0.045044	-0.411815368	0.000369	-0.15637774	0.499198	C5	-	-	-	Y	-	Y	-
ENSG00000127585	FBXL16	0.364200624	7.31E-07	0.23611372	0.00544	0.266361172	0.003087	-0.121676046	0.131015	-0.083154534	0.367984	0.033174124	0.863643	C5	-	Y	-	-	-	-	-
ENSG00000127588	GNG13	0.203673303	0.644433	0.139964378	0.813462	-0.78287392	0.111103	-0.056706633	0.911461	-0.971333648	0.022039	-0.918828238	NA	C5	-	-	-	-	-	Y	-
ENSG00000127666	TICAM1	0.510981574	2.32E-05	0.201982086	0.229187	0.445990307	0.000793	-0.302596333	0.021319	-0.050185549	0.765899	0.24683296	0.182394	C4	-	Y	-	Y	Y	-	-
ENSG00000127804	METTL16	0.265897257	0.000344	0.087677504	0.412851	-0.08524352	0.400181	-0.171880901	0.021635	-0.336648944	9.62E-07	-0.170166293	0.089669	C5	-	-	-	-	-	Y	-
ENSG00000127870	RNF6	0.370037709	0.00175	-0.071406408	0.705577	0.202341803	0.121255	-0.435520888	2.24E-05	-0.153540573	0.158544	0.277028697	0.026763	C5	-	Y	-	-	Y	-	-
ENSG00000127903	ZNF835	0.330392243	0.20785	-0.357836066	0.301969	0.02832703	0.948703	-0.681808277	0.007795	-0.287403075	0.302706	0.389161797	NA	C5	-	-	-	-	Y	-	-
ENSG00000127914	AKAP9	0.417345995	2.32E-06	0.109502205	0.355875	0.209186827	0.046721	-0.301597559	1.03E-06	-0.193804273	0.07558	0.102483822	0.342511	C5	-	Y	-	-	Y	-	-
ENSG00000127920	GNG11	0.181950918	0.02453	0.199445157	0.044574	-0.14042703	0.173767	0.023460679	0.7685	-0.3071135	1.36E-08	-0.336222565	1.12E-06	C4	-	-	-	-	-	Y	Y
ENSG00000127952	STYXL1	-0.488846811	4.09E-08	-0.071517964	0.671159	-0.00251679	0.98776	0.42449786	0.000183	0.501176152	1.68E-07	0.07147597	0.762354	C4	-	Y	-	-	Y	Y	-
ENSG00000127954	STEAP4	-2.84159109	0.003751	-0.73857925	NA	-0.552779132	0.527562	2.096680171	0.099302	2.303228928	0.044482	0.191802971	NA	C3	-	Y	-	-	-	Y	-
ENSG00000127957	PMS2P3	0.286316015	0.106179	0.412953185	0.021483	0.240600598	0.215119	0.132908674	0.407636	-0.031229054	0.873677	-0.169424468	0.421024	C1	-	-	Y	-	-	-	-
ENSG00000127989	MTERF1	0.100332833	0.308525	-0.319659226	0.000151	-0.092070981	0.351956	-0.413930225	1.28E-06	-0.177742038	0.047005	0.230452796	0.02324	C5	-	-	Y	-	Y	-	-
ENSG00000127990	SGCE	0.338969451	1.25E-07	0.148827346	0.134343	0.07830046	0.423978	-0.183182401	0.009452	-0.245624799	7.28E-05	-0.067842787	0.629384	C5	-	Y	-	-	-	-	-
ENSG00000127993	RBM48	0.114534212	0.169267	-0.260401847	0.001964	-0.146810599	0.139081	-0.368644478	6.89E-07	-0.246772302	0.002589	0.116677	0.410425	C4	-	-	-	-	Y	-	-
ENSG00000128011	LRFN1	0.000480847	0.998238	0.15928316	0.314966	0.295169333	0.035316	0.165358343	0.239303	0.309328146	0.017666	0.138619318	0.525252	C4	-	-	-	-	-	Y	-
ENSG00000128016	ZFP36	0.7369813	0.001849	0.291867333	0.404484	0.271474793	0.455412	-0.438910524	0.079739	-0.450059874	0.090926	-0.017367371	0.981547	C5	-	Y	-	-	-	-	-
ENSG00000128039	SRD5A3	-0.62395784	4.29E-06	-0.267355266	0.082563	-0.254059675	0.205456	0.364170057	0.025014	0.385014914	0.048099	0.014726629	0.9772	C4	-	Y	-	-	Y	Y	-
ENSG00000128059	PPAT	0.372668287	6.77E-14	0.089456506	0.226416	0.157406168	0.009488	-0.27673429	8.73E-10	-0.200743145	4.90E-06	0.070706312	0.389775	C5	-	Y	-	-	-	-	-
ENSG00000128165	ADM2	-2.305235547	7.82E-14	-0.628663915	0.036883	-0.452161083	0.14499	1.679186318	5.62E-06	1.86734465	2.44E-07	0.180740011	NA	C3	-	Y	Y	-	Y	Y	-

ENSG00000128185	DGCR6L	-0.392756906	0.002448	-0.19679033	0.218156	-0.16214689	0.290565	0.202735603	0.116456	0.245539212	0.041339	0.036909736	0.8908	C4	-	Y	-	-	-	-	-
ENSG00000128191	DGCR8	-0.341119116	3.69E-10	-0.154335527	0.049953	-0.338558085	1.61E-07	0.193432368	0.002232	0.017155339	0.844817	-0.181280967	0.045236	C4	-	Y	-	Y	-	-	-
ENSG00000128203	ASPHD2	0.643583955	4.70E-06	0.437111157	0.008245	0.387123198	0.029794	-0.199915835	0.17662	-0.241885252	0.117992	-0.047186969	0.896094	C5	-	Y	Y	Y	-	-	-
ENSG00000128242	GAL3ST1	-1.269465564	0.0679	0.363472626	0.634883	0.346923646	0.633997	1.637279035	0.009087	1.629671937	0.009868	-0.014738537	NA	C3	-	-	-	-	Y	Y	-
ENSG00000128271	ADORA2A	1.109998329	0.000215	0.805505551	0.031349	0.672327988	0.086982	-0.299016442	0.34565	-0.423307926	0.174893	-0.129913404	NA	C1	-	Y	Y	-	-	-	-
ENSG00000128272	ATF4	-0.579267031	1.03E-18	-0.071918639	0.59149	0.013333615	0.9107	0.51395257	2.29E-13	0.607556538	2.31E-35	0.088021242	0.517627	C4	-	Y	-	-	Y	Y	-
ENSG00000128294	TPST2	0.299635959	0.001536	0.636768845	5.04E-10	0.598746286	3.81E-11	0.34355522	8.28E-05	0.314090996	4.07E-05	-0.034508679	0.868903	C4	-	-	Y	Y	Y	Y	-
ENSG00000128311	TST	-0.511840839	0.003045	-0.163044575	0.462525	-0.156503711	0.421651	0.357257297	0.074919	0.370400496	0.046696	0.009566865	0.982422	C4	-	Y	-	-	-	Y	-
ENSG00000128335	APOL2	-0.805359752	3.19E-05	-0.542768209	0.018706	-0.123125493	0.672455	0.269716736	0.341536	0.696716826	0.002586	0.421172028	0.220784	C4	-	Y	Y	-	-	Y	-
ENSG00000128342	LIF	2.052308267	5.83E-08	0.378491757	0.638155	0.272117694	0.740979	-1.662466936	3.38E-05	-1.763463448	1.72E-05	-0.101317251	NA	C5	-	Y	-	-	Y	Y	-
ENSG00000128394	APOBEC3F	-0.751669036	0.08905	-0.063350122	0.920097	0.303662338	0.52495	0.694945347	0.114258	1.070384652	0.00767	0.370132712	NA	C4	-	-	-	-	-	Y	-
ENSG00000128482	RNF112	-0.343918628	4.55E-06	-0.209574687	0.02802	-0.1574386	0.085606	0.141138255	0.101861	0.201355758	0.009291	0.05480651	0.73559	C4	-	Y	-	-	-	-	-
ENSG00000128487	SPECC1	-0.556401597	7.81E-08	-0.30300263	0.01528	-0.353105052	0.002135	0.259871313	0.025617	0.217976633	0.065655	-0.047400829	0.853543	C4	-	Y	Y	Y	-	-	-
ENSG00000128510	CPA4	-0.601983467	0.378928	-0.067326567	0.939204	0.780483425	0.123607	0.544185957	0.481324	1.397216581	0.013768	0.850317538	NA	C2	-	-	-	-	-	Y	-
ENSG00000128536	CDHR3	-0.340437172	0.1113	0.037335376	0.915404	0.207459093	0.366291	0.38403967	0.082363	0.562969783	0.002679	0.17213839	0.606986	C4	-	-	-	-	-	Y	-
ENSG00000128564	VGF	1.126622167	2.26E-114	0.66845401	3.41E-25	0.527806997	1.25E-20	-0.451460987	1.39E-30	-0.583856988	1.43E-118	-0.137769494	0.030105	C1	-	Y	Y	Y	Y	Y	-
ENSG00000128578	STRIP2	-0.468384226	0.000159	-0.277722262	0.073202	-0.216579918	0.13322	0.197515741	0.223889	0.26647497	0.063624	0.063864503	0.836681	C4	-	Y	-	-	-	-	-
ENSG00000128585	MKLN1	0.585302495	0.000762	0.254585751	0.303022	0.288600743	0.153125	-0.32561448	7.62E-06	-0.282715414	3.54E-06	0.037781374	0.821705	C1	-	Y	-	-	Y	-	-
ENSG00000128590	DNAJB9	0.036547374	0.750442	-0.315873395	0.002679	-0.216316202	0.060937	-0.346116451	0.000313	-0.238120693	0.021906	0.103142967	0.621746	C5	-	-	Y	-	Y	-	-
ENSG00000128591	FLNC	1.117105501	1.11E-29	0.140722064	0.450479	0.130234712	0.421651	-0.970844693	3.14E-20	-0.972010918	2.10E-24	-0.007618338	0.982455	C5	-	Y	-	-	Y	Y	-
ENSG00000128594	LRRC4	-0.489714373	0.001329	-0.670172495	0.000574	-0.938851295	1.89E-06	-0.174743855	0.469622	-0.434355128	0.050731	-0.262471124	0.538376	C3	-	Y	Y	Y	-	-	-
ENSG00000128596	CCDC136	0.201710414	0.047977	0.226971077	0.03197	0.332460655	0.001078	0.031056406	0.748358	0.145440685	0.074227	0.108916701	0.341115	C4	-	-	-	Y	-	-	-
ENSG00000128607	KLHDC10	0.451876008	5.01E-11	0.000436081	0.997464	0.185553019	0.022465	-0.444937037	8.48E-13	-0.251838164	1.60E-05	0.187799709	0.031239	C5	-	Y	-	-	Y	-	-
ENSG00000128617	OPN1SW	-3.53853999	0.040566	-1.305680582	NA	-1.900184367	NA	2.237181222	NA	1.663343976	NA	-0.586037918	NA	C3	-	Y	-	-	-	-	-
ENSG00000128652	HOXD3	-0.364467265	0.005842	-0.327201593	0.028491	-0.359200111	0.011112	0.043869034	0.801733	0.020114978	0.914738	-0.029447909	0.928882	C4	-	Y	Y	Y	-	-	-
ENSG00000128654	MTX2	-0.306876821	4.70E-06	-0.067039685	0.51006	-0.067940867	0.449286	0.246397144	0.000107	0.253690132	2.13E-05	0.001807024	0.991966	C4	Y	Y	-	-	-	-	-
ENSG00000128692	EIF2S2P4	-0.544711452	0.004316	-0.410018403	0.062404	-0.135387	0.606149	0.142235499	0.468405	0.424706936	0.007816	0.276860573	0.209127	C4	-	Y	-	-	-	Y	-
ENSG00000128709	HOXD9	-0.777552365	9.40E-15	-0.206739714	0.14461	-0.148167394	0.260586	0.57722098	6.34E-08	0.644436712	1.98E-11	0.061695966	0.794567	C2	-	Y	-	-	Y	Y	-
ENSG00000128710	HOXD10	-0.701301669	0.042484	-0.449571082	0.238895	0.065078798	0.89204	0.258385039	0.586683	0.780992935	0.035252	0.517290147	NA	C4	-	Y	-	-	-	Y	-
ENSG00000128791	TWSG1	0.278867412	0.002947	-0.088928383	0.543448	-0.151209835	0.278708	-0.36095996	0.000412	-0.415091145	0.000137	-0.060278566	0.824504	C5	-	-	-	-	Y	Y	-
ENSG00000128805	ARHGAP22	-0.967366223	1.90E-12	-0.453170895	0.01869	-0.352933151	0.016432	0.520530697	0.003754	0.62944943	6.23E-06	0.103216955	0.766411	C3	-	Y	Y	Y	Y	Y	-
ENSG00000128849	CGNL1	-1.484425095	0.045125	-1.316124195	0.065021	-1.007670041	0.136675	0.173425274	0.889511	0.490150381	0.638663	0.311172338	NA	C3	-	Y	-	-	-	-	-
ENSG00000128872	TMOD2	0.426293172	2.31E-08	0.217710396	0.026242	0.318123511	0.000248	-0.202257924	7.92E-05	-0.093566974	0.090587	0.103281911	0.244612	C5	-	Y	-	Y	-	-	-
ENSG00000128881	TTBK2	0.492207351	4.60E-08	0.34183434	0.003934	0.352541653	0.001403	-0.144005876	0.140889	-0.124892999	0.184711	0.013517928	0.96038	C1	-	Y	Y	Y	-	-	-
ENSG00000128923	MINDY2	0.336866353	0.015513	0.162738154	0.399285	0.259749259	0.107473	-0.169590672	0.083694	-0.06265077	0.584461	0.101232396	0.554373	C1	-	Y	-	-	-	-	-
ENSG00000128951	DUT	-0.329993652	2.35E-08	-0.179823348	0.038147	-0.291327707	1.55E-05	0.156873395	0.004676	0.053483188	0.29326	-0.108708343	0.217485	C4	-	Y	-	-	-	-	-
ENSG00000128965	CHAC1	-2.354803217	5.30E-19	0.164109272	0.806916	0.025102656	0.952756	2.527752004	1.33E-19	2.394658147	1.35E-22	-0.138994463	0.810219	C3	-	Y	-	-	Y	Y	-
ENSG00000128973	CLN6	-0.31703945	0.00052	-0.045962582	0.733688	-0.025542334	0.857403	0.277635445	0.003729	0.306102103	0.001468	0.023164912	0.923365	C4	-	Y	-	-	-	Y	-
ENSG00000129003	VPS13C	0.149485103	0.213777	0.253629032	0.247592	0.37791132	0.033519	0.110238187	0.25253	0.243011887	0.000456	0.127418757	0.397763	C4	-	-	-	Y	-	-	-
ENSG00000129009	ISLR	-0.416695772	0.00015	-0.15210847	0.254315	-0.320886874	0.005315	0.271256643	0.020927	0.11039003	0.432862	-0.165918219	0.319251	C4	-	Y	-	Y	-	-	-
ENSG00000129071	MBD4	0.309085531	0.000285	0.003110102	0.98614	0.092221058	0.435876	-0.299445914	0.000154	-0.202356751	0.012564	0.091858707	0.52517	C5	-	Y	-	-	-	-	-
ENSG00000129103	SUMF2	-0.3041262	3.93E-06	-0.031881369	0.81065	-0.071360647	0.483224	0.278647995	6.16E-05	0.247694068	0.000137	-0.036551311	0.837097	C4	-	Y	-	-	-	-	-
ENSG00000129173	E2F8	0.019402513	0.888636	-0.491660982	4.52E-05	-0.499605571	2.70E-06	-0.504438263	1.37E-06	-0.504659359	1.23E-07	-0.004901604	0.989082	C4	-	-	Y	Y	Y	Y	-
ENSG00000129187	DCTD	-0.332440679	0.000168	-0.001410197	0.993718	-0.091568664	0.506291	0.33762305	0.000172	0.25594075	0.016602	-0.087312178	0.646501	C2	-	Y	-	-	Y	-	-
ENSG00000129244	ATP1B2	0.67485137	0.000582	0.189315939	0.56679	0.264405619	0.378992	-0.478905385	0.030214	-0.396486027	0.089089	0.078973188	0.897862	C5	-	Y	-	-	Y	-	-
ENSG00000129245	FXR2	0.442994449	1.07E-14	0.017300058	0.878383	0.082783351	0.307737	-0.419147971	2.62E-14	-0.345600096	1.88E-10	0.068293394	0.53558	C5	Y	Y	-	-	Y	Y	-
ENSG00000129250	KIF1C	0.686299299	2.22E-14	0.399748448	0.000225	0.473508999	5.61E-06	-0.280132397	0.003352	-0.197936864	0.050731	0.07670159	0.70497	C1	-	Y	Y	Y	-	-	-
ENSG00000129292	PHF20L1	0.559477365	2.37E-16	0.213910321	0.009531	0.261603993	0.00025	-0.339001731	3.21E-08	-0.283220369	7.74E-07	0.050337002	0.674546	C5	-	Y	-	-	Y	-	-
ENSG00000129315	CCNT1	0.367591767	1.78E-08	-0.155137226	0.080384	0.084883253	0.312246	-0.516188618	1.44E-16	-0.268162501	2.32E-06	0.242605759	0.001886	C5	-	Y	-	-	Y	-	-
ENSG00000129353	SLC44A2	-0.402203104	5.83E-10	-0.207982944	0.00381	-0.173011369	0.014666	0.200954414	0.001356	0.244000699	4.27E-05	0.037555877	0.752547	C4	Y	Y	-	-	-	-	-
ENSG00000129422	MTUS1	-1.571101641	1.80E-06	-0.458243903	0.197092	-0.654439957	0.057891	1.122562962	0.00477	0.931169639	0.033945	-0.195503406	NA	C3	-	Y	-	-	Y	Y	-
ENSG00000129473	BCL2L2	0.463199097	1.04E-12	0.276538949	0.000789	0.149481776	0.077776	-0.179993202	0.011511	-0.299123521	3.35E-06	-0.124330936	0.263634	C5	Y	Y	-	-	-	-	-
ENSG00000129474	AJUBA	0.007833635	0.981454	0.1002494	0.778256	0.448727675	0.03923	0.099288495	0.768052	0.455616726	0.051315	0.351428281	0.248232	C4	-	-	-	Y	-	-	-
ENSG00000129493	HEATR5A	0.102396053	0.281956	-0.237484765	0.028478	-0.115376447	0.284218	-0.332875446	4.88E-06	-0.203523858	0.002101	0.124306827	0.311688	C5	-	-	-	-	Y	-	-
ENSG00000129518	EAPP	-0.258146937	0.005571	-0.161113493	0.156094	-0.309638868	0.001411	0.103491311	0.337658	-0.036686621	0.772615	-0.145621426	0.29094	C4	-	-	-	Y	-	-	-
ENSG00000129521	EGLN3	-0.543497103	9.68E-15	-0.124347085	0.207013	-0.299279417	0.003375	0.425680438	1.24E-08	0.259174489	0.006816	-0.172542831	0.228015	C4	-	Y	-	-	Y	-	-
ENSG00000129535	NRL	-1.065807782	0.01495	-0.283452277	0.626419	-0.319363475	0.508463	0.78825589	0.136445	0.761881036	0.12433	-0.032840252	NA	C3	-	Y	-	-	-	-	-
ENSG00000129566	TEP1	0.558154571	7.54E-05	0.314857871	0.052942	0.441740061	0.005068	-0.237708196	0.082386	-0.101863948	0.540457	0.130113437	0.582015	C5	-	Y	-	Y	-	-	-
ENSG00000129596	CDO1	-0.545014721	0.008848	-0.272319012	0.315684	-0.250033561	0.330886	0.279281736	0.30419	0.309711883	0.233075	0.025528465	0.967582	C4	-	Y	-	-	-	-	-
ENSG00000129657	SEC14L1	0.596540167	2.99E-24	0.359989692	1.44E-07	0.366735671	4.90E-09	-0.230390191	2.11E-06	-0.215441282	5.43E-07	0.009758704	0.944407	C5	-	Y	Y	Y	-	-	-
ENSG00000129667	RHBDF2	-0.616861807	3.00E-07	-0.029635286	0.886728	0.151977097	0.299333	0.593714323	5.03E-07	0.783666361	2.88E-12	0.185017031	0.270879	C4	-	Y	-	-	Y	Y	-
ENSG00000129675	ARHGEF6	-0.613326367	0.005027	0.194328175	0.494012	0.289565287	0.211233	0.814975253	0.000214	0.917243063	1.10E-05	0.09821768	0.821843	C4	-	Y	-	-	Y	Y	-
ENSG00000129691	ASH2L	-0.440286051	9.48E-09	-0.130405689	0.133147	-0.276030828	0.00074	0.316160029	6.27E-06	0.178692186	0.028839	-0.142846924	0.151336	C4	-	Y	-	-	Y	-	-
ENSG00000129696	TTI2	0.399178027	0.001331	-0.256106563	0.125766	-0.082417779	0.642931	-0.648748902	1.73E-06	-0.466881242	0.000196	0.176648649	0.45687	C5	-	Y	-	-	Y	Y	-
ENSG00000129757	CDKN1C	-0.659592483	4.68E-09	-0.172656544	0.256249	-0.084480449	0.598397	0.493250036	0.000124	0.590035151	1.27E-06	0.091043943	0.712971	C4	-	Y	-	-	Y	Y	-
ENSG00000129810	SGO1	0.194036426	0.047044	-0.133471732	0.384732	0.028174908	0.853646	-0.320804112	0.002123	-0.151318922	0.114583	0.1644052	0.322058	C4	-	-	-	-	Y	-	-
ENSG00000129925	PGAP6	0.30289866	2.35E-05	0.187966155	0.047109	0.276919594	0.00069	-0.108327622	0.163581	-0.011253935	0.910094	0.091718016	0.45687	C5	-	Y	-	-	-	-	-

ENSG00000129990	SYT5	0.463846291	0.000166	0.023411826	0.910114	-0.128152365	0.40571	-0.434412963	0.00031	-0.577272473	6.57E-07	-0.148908553	0.444757	C5	-	Y	-	-	Y	Y	-
ENSG00000129991	TNNI3	-1.348654578	1.13E-07	-0.891101471	0.00101	-0.772425938	0.004142	0.464500948	0.094765	0.592203315	0.026625	0.122388887	0.810986	C3	-	Y	Y	Y	-	Y	-
ENSG00000130005	GAMT	-0.44348045	4.84E-06	-0.326318572	0.004669	-0.251125841	0.022622	0.123524853	0.243982	0.207444726	0.021811	0.078450665	0.641821	C4	-	Y	Y	-	-	-	-
ENSG00000130021	PUDP	-0.173541214	0.205697	-0.362597192	0.009653	-0.190484248	0.183419	-0.183018014	0.220327	-0.002493651	0.988019	0.174907795	0.39874	C4	-	-	Y	-	-	-	-
ENSG00000130035	GALNT8	3.99120915	0.049265	NA	NA	3.416444526	NA	-3.956015202	NA	-0.553759859	0.759394	3.390415602	NA	C5	-	Y	-	-	-	-	-
ENSG00000130038	CRACR2A	-0.805843818	0.029967	-0.585674928	0.260773	-0.353948811	0.43122	0.230346408	0.714179	0.466060343	0.324164	0.231539456	NA	C3	-	Y	-	-	-	-	-
ENSG00000130066	SAT1	0.449954722	0.035776	-0.378275208	0.185359	-0.106145237	0.766752	-0.822307967	9.27E-05	-0.542147833	0.01636	0.274341344	0.521043	C5	-	Y	-	-	Y	Y	-
ENSG00000130119	GNL3L	0.351292189	3.22E-06	0.054122755	0.672253	0.083747853	0.461191	-0.291032057	5.51E-07	-0.25318516	4.04E-05	0.032420821	0.833312	C5	-	Y	-	-	-	-	-
ENSG00000130147	SH3BP4	-0.28698018	1.08E-06	-0.028468579	0.801244	0.006605122	0.950816	0.265069164	3.21E-05	0.308165398	6.25E-08	0.037839799	0.784312	C4	-	-	-	-	-	Y	-
ENSG00000130150	MOSPD2	0.169100115	0.105696	-0.171118468	0.214294	-0.039431844	0.816756	-0.333652667	0.001094	-0.194167547	0.076491	0.134752234	0.491829	C5	-	-	-	-	Y	-	-
ENSG00000130164	LDLR	-0.44749551	1.43E-06	-0.080529007	0.687227	0.054157496	0.715608	0.372848189	0.003615	0.516092059	1.43E-07	0.138006294	0.555221	C4	-	Y	-	-	Y	Y	-
ENSG00000130204	TOMM40	0.100066085	0.265049	0.005641289	0.972925	-0.229837693	0.013501	-0.087106274	0.26519	-0.315088357	1.37E-07	-0.232885358	0.009386	C4	-	-	-	-	-	Y	-
ENSG00000130224	LRCH2	0.362596675	3.09E-05	0.03798413	0.81065	0.14707011	0.177855	-0.318177221	0.000116	-0.200752085	0.016091	0.112100129	0.420342	C5	-	Y	-	-	Y	-	-
ENSG00000130226	DPP6	-0.410497476	2.40E-05	-0.277108391	0.035964	-0.303089589	0.025547	0.140021228	0.310081	0.122453086	0.406282	-0.024409268	0.947484	C4	-	Y	-	Y	-	-	-
ENSG00000130254	SAFB2	0.283510469	1.15E-07	-0.045613398	0.681878	-0.039195999	0.689278	-0.322418167	1.44E-07	-0.308224124	7.50E-08	0.009570746	0.96262	C5	-	-	-	-	Y	Y	-
ENSG00000130270	ATP8B3	-0.815833882	1.16E-06	-0.650830488	0.000418	-0.113629163	0.629426	0.171659322	0.443776	0.7175844	1.51E-05	0.540049865	0.006994	C4	-	Y	Y	-	-	Y	Y
ENSG00000130287	NCAN	-0.451171854	9.91E-08	-0.400655885	2.09E-05	-0.264580627	0.006028	0.056984941	0.613273	0.200904925	0.024864	0.138603817	0.308298	C4	-	Y	Y	-	-	-	-
ENSG00000130304	SLC27A1	-0.177279137	0.344008	0.010661883	0.970129	0.14536815	0.487148	0.194917645	0.215745	0.337673417	0.021448	0.137414186	0.552062	C4	-	-	-	-	-	Y	-
ENSG00000130305	NSUN5	0.138325362	0.045154	-0.033969591	0.783321	-0.193420918	0.019705	-0.165826983	0.008687	-0.317166578	6.41E-08	-0.15654858	0.09967	C5	-	-	-	-	-	Y	-
ENSG00000130313	PGLS	-0.303648747	0.000719	0.049977656	0.7331	-0.016531451	0.910735	0.360123175	4.10E-05	0.302028154	0.000437	-0.063746712	0.708729	C4	-	Y	-	-	Y	Y	-
ENSG00000130340	SNX9	0.404262846	8.96E-11	0.168438726	0.035405	0.210030556	0.010431	-0.229383877	0.000197	-0.179873706	0.008169	0.044419606	0.767009	C5	-	Y	-	-	-	-	-
ENSG00000130347	RTN4IP1	-0.490463537	0.007727	-0.156081281	0.55642	-0.439773621	0.030278	0.340083067	0.118931	0.065647259	0.820169	-0.280118883	0.384946	C4	-	Y	-	Y	-	-	-
ENSG00000130363	RSPH3	0.371896701	0.012473	0.061940073	0.822291	0.143710244	0.492389	-0.304311167	0.046192	-0.213249573	0.158457	0.085144957	0.796496	C5	-	Y	-	-	Y	-	-
ENSG00000130413	STK33	-1.01817147	1.99E-09	-0.281087904	0.197633	-0.07793938	0.734557	0.745400705	0.000283	0.954694487	1.07E-07	0.205478605	0.508688	C3	-	Y	-	-	Y	Y	-
ENSG00000130475	FCHO1	-0.252522182	0.167004	-0.144913965	0.520104	0.139721019	0.511406	0.11425425	0.609498	0.407153057	0.021629	0.287429952	0.23125	C4	-	-	-	-	-	Y	-
ENSG00000130477	UNC13A	0.333395639	5.94E-08	0.226721148	0.001808	0.255613296	0.000164	-0.100258882	0.034326	-0.063050159	0.205793	0.031848043	0.737453	C5	-	Y	-	-	-	-	-
ENSG00000130517	PGPEP1	0.37865136	0.00035	0.273746214	0.024256	0.264418568	0.022216	-0.098912455	0.463822	-0.099649206	0.440027	-0.006280033	0.982455	C1	-	Y	-	-	-	-	-
ENSG00000130518	IQCN	-0.766322473	8.65E-05	0.378557345	0.029469	0.348631418	0.038306	1.151211704	6.48E-10	1.129526026	7.38E-10	-0.027026636	0.947133	C2	-	Y	Y	Y	Y	Y	-
ENSG00000130520	LSM4	-0.345553727	1.35E-06	-0.25351462	0.003358	-0.217382871	0.015567	0.09918484	0.032942	0.143084786	0.003995	0.038817492	0.724011	C4	-	Y	-	-	-	-	-
ENSG00000130529	TRPM4	-0.701573506	4.22E-05	-0.168951321	0.442908	-0.043404689	0.86441	0.538633056	0.003167	0.672465355	0.000134	0.12834136	0.674815	C4	-	Y	-	-	Y	Y	-
ENSG00000130544	ZNF557	0.186895421	0.289533	-0.199510746	0.308168	0.006463643	0.980426	-0.38031127	0.004725	-0.166302438	0.27661	0.208458606	0.282748	C5	-	-	-	-	Y	-	-
ENSG00000130558	OLFM1	-0.429409238	1.37E-11	-0.2361066	0.001163	-0.27673709	6.60E-05	0.19975032	0.004081	0.167421274	0.018067	-0.037694274	0.796431	C4	-	Y	-	-	-	-	-
ENSG00000130590	SAMD10	0.52191955	7.57E-06	0.331916602	0.019121	0.272146303	0.049924	-0.183394342	0.184755	-0.235130982	0.067623	-0.057007637	0.84282	C5	-	Y	Y	-	-	-	-
ENSG00000130600	H19	0.7108774	0.018155	-0.304213924	0.551298	0.25229685	0.578608	-1.00834705	0.001416	-0.444146169	0.177368	0.559503108	NA	C5	-	Y	-	-	Y	-	-
ENSG00000130635	COL5A1	0.3526904	0.001257	0.643003096	1.51E-09	0.785718619	2.88E-16	0.296806076	0.005721	0.447670365	3.28E-06	0.145887328	0.348939	C4	-	Y	Y	Y	-	Y	-
ENSG00000130649	CYP2E1	2.344193226	0.000913	1.230895533	NA	0.981093455	NA	-1.095728404	0.109192	-1.34341044	0.044082	-0.248242841	NA	C5	-	Y	-	-	-	Y	-
ENSG00000130695	CEP85	0.332503324	1.65E-05	0.006301339	0.966449	0.095021939	0.307601	-0.319906445	1.12E-05	-0.222925542	0.001791	0.091268652	0.440789	C5	-	Y	-	-	Y	-	-
ENSG00000130699	TAF4	0.42921706	1.36E-08	0.128455278	0.29031	0.12734202	0.222197	-0.294511112	0.000301	-0.287338358	0.000126	0.001796466	0.994757	C5	-	Y	-	-	-	-	-
ENSG00000130702	LAMA5	-0.072176242	0.348224	0.057751031	0.505169	0.244832056	0.000107	0.136349032	0.021047	0.33169385	7.38E-10	0.190085098	0.001981	C4	-	-	-	-	-	Y	-
ENSG00000130706	ADRM1	0.177765597	0.003863	-0.038813418	0.726264	-0.171903647	0.023239	-0.210051325	0.000382	-0.334988987	7.31E-10	-0.130162948	0.166977	C5	-	-	-	-	-	Y	-
ENSG00000130714	POMT1	0.396664329	1.29E-06	0.26928452	0.009318	0.329383849	0.000353	-0.121134368	0.19938	-0.052522277	0.611157	0.06315031	0.714528	C5	Y	Y	-	Y	-	-	-
ENSG00000130724	CHMP2A	-0.323635056	8.54E-06	-0.191121165	0.044572	-0.159903781	0.060581	0.138913137	0.076961	0.17854961	0.006281	0.034491649	0.83368	C4	-	Y	-	-	-	-	-
ENSG00000130762	ARHGEF16	-0.655239319	0.010867	-0.585646963	0.06568	-0.295317504	0.34205	0.079433274	0.861861	0.374680108	0.251831	0.291486613	NA	C4	-	Y	-	-	-	-	-
ENSG00000130766	SESN2	-0.655507529	1.69E-05	-0.344104863	0.144229	-0.033285272	0.906917	0.31885715	0.090187	0.636516819	1.27E-05	0.313643791	0.265132	C4	-	Y	-	-	-	Y	-
ENSG00000130772	MED18	0.045498131	0.802554	-0.459970348	0.010538	-0.223665978	0.235449	-0.498649317	0.001955	-0.254571668	0.125563	0.239375452	0.424483	C5	-	-	Y	-	Y	-	-
ENSG00000130775	THEMIS2	-0.273112713	0.274329	-0.540551187	0.048342	0.077133809	0.819814	-0.260940707	0.368225	0.364340199	0.13334	0.620848538	0.039906	C4	-	-	Y	-	-	-	Y
ENSG00000130779	CLIP1	0.640430359	2.37E-24	0.409137521	2.39E-08	0.523835151	7.34E-15	-0.224608466	0.000409	-0.101917448	0.136947	0.117320185	0.235874	C5	-	Y	Y	Y	-	-	-
ENSG00000130810	PPAN	-0.244081647	0.038588	-0.238561272	0.114655	-0.366375362	0.001913	0.012452355	0.946557	-0.10736591	0.400366	-0.125072318	0.550899	C4	-	-	-	Y	-	-	-
ENSG00000130813	SHFL	-0.882186255	6.53E-06	-0.195361481	0.444897	-0.053436121	0.845308	0.693838132	0.000922	0.843035754	1.15E-05	0.145068133	0.667843	C3	-	Y	-	-	Y	Y	-
ENSG00000130827	PLXNA3	0.478897149	7.48E-17	0.279637247	3.07E-05	0.319358238	1.09E-06	-0.192819432	0.001649	-0.144783852	0.024205	0.042588077	0.755306	C5	-	Y	-	Y	-	-	-
ENSG00000130935	NOL11	0.284927692	5.80E-07	-0.08563725	0.309042	-0.004874669	0.964237	-0.364052616	1.46E-16	-0.275409536	9.48E-11	0.083307578	0.276801	C5	-	-	-	-	Y	-	-
ENSG00000130943	PKDREJ	-1.04084088	0.001349	-0.059461299	0.903779	0.121641293	0.766983	0.988552578	0.00296	1.176466899	0.00017	0.184418192	NA	C3	-	Y	-	-	Y	Y	-
ENSG00000131013	PPIL4	0.473284773	5.67E-09	-0.085409401	0.483041	0.055198113	0.674287	-0.552592946	2.47E-15	-0.403389011	5.05E-08	0.143672556	0.219596	C5	-	Y	-	-	Y	Y	-
ENSG00000131018	SYNE1	0.263561441	0.014163	0.283891208	0.03197	0.372139809	0.001304	0.025835083	0.833477	0.123174606	0.165524	0.091578464	0.598842	C1	-	-	-	Y	-	-	-
ENSG00000131019	ULBP3	-1.651544774	0.009616	-0.752599821	0.304836	-0.963797362	0.155895	0.903680882	0.285784	0.701415389	0.438944	-0.208256561	NA	C3	-	Y	-	-	-	-	-
ENSG00000131023	LATS1	0.308971573	6.47E-05	-0.100449752	0.444143	0.049539014	0.699381	-0.402673448	7.61E-11	-0.244734057	1.10E-05	0.152619946	0.160075	C5	-	Y	-	-	Y	-	-
ENSG00000131037	EPS8L1	-0.491165093	0.00235	-0.216979386	0.299201	-0.256943515	0.153459	0.28116375	0.135863	0.24908399	0.1758	-0.036966252	0.931289	C4	-	Y	-	-	-	-	-
ENSG00000131055	COX4I2	-0.544230139	0.262546	0.346784877	0.496752	0.494735474	0.257613	0.899430184	0.029878	1.054261304	0.007588	0.151935701	NA	C2	-	-	-	-	Y	Y	-
ENSG00000131069	ACSS2	-0.053304054	0.732233	0.191134348	0.148681	0.302619508	0.009441	0.251284107	0.021635	0.370763315	0.000284	0.114463442	0.482168	C4	-	-	-	Y	-	Y	-
ENSG00000131089	ARHGEF9	-0.261709934	0.012275	0.005934241	0.979271	0.14624845	0.199068	0.274510546	0.029259	0.422431572	1.05E-05	0.143283838	0.456495	C4	-	-	-	-	-	Y	-
ENSG00000131094	C1QL1	-0.578506568	0.000444	-0.600454543	0.002793	-0.441258093	0.048158	-0.016375421	0.950188	0.152593434	0.492466	0.161730318	0.674586	C4	-	Y	Y	Y	-	-	-
ENSG00000131127	ZNF141	0.314197373	0.001818	-0.027574236	0.885404	0.075477938	0.543513	-0.335268592	0.002227	-0.224256799	0.019028	0.105614139	0.568646	C5	-	Y	-	-	Y	-	-
ENSG00000131153	GINS2	-0.210937537	0.001059	-0.215189699	0.028509	-0.36791344	0.000151	0.002506562	0.982244	-0.142587435	0.140585	-0.148785328	0.394666	C4	-	-	-	Y	-	-	-
ENSG00000131171	SH3BGRL	-0.329532026	1.64E-06	-0.201260487	0.013901	-0.152490102	0.092451	0.134811601	0.076981	0.191967214	0.012878	0.051817077	0.735812	C4	-	Y	-	-	-	-	-
ENSG00000131187	F12	-0.330395539	0.040141	-0.182758609	0.419726	-0.20555352	0.279552	0.154841441	0.366661	0.140242382	0.369647	-0.02052897	0.958257	C4	-	Y	-	-	-	-	-
ENSG00000131351	HAUS8	-0.240991148	0.063185	-0.406516571	0.003683	-0.358706751	0.004564	-0.159615122	0.360312	-0.102889834	0.550774	0.051292958	0.8863	C5	-	-	Y	Y	-	-	-
ENSG00000131373	HACL1	-0.420810882	0.0003	-0.137294287	0.383989	-0.20087216	0.129811	0.290069179	0.027775	0.234675771	0.078014	-0.060599794	0.818037	C4	-	Y	-	-	-	-	-

ENSG00000131374	TBC1D5	-0.40596952	1.51E-10	0.012705163	0.924137	0.04952977	0.604426	0.424588789	5.72E-17	0.46998608	5.49E-27	0.039958294	0.723061	C4	-	Y	-	-	Y	Y	-
ENSG00000131375	CAPN7	0.325673598	7.76E-07	0.250629599	0.000533	0.179131633	0.020335	-0.068693091	0.360312	-0.13192761	0.052967	-0.068593973	0.544161	C5	-	Y	-	-	-	-	-
ENSG00000131386	GALNT15	3.408797532	0.005698	1.983540529	NA	2.498527596	NA	-1.408448805	0.197122	-0.896115115	0.333244	0.512060845	NA	C1	-	Y	-	-	-	-	-
ENSG00000131389	SLC6A6	0.303906391	0.000295	0.433978616	6.28E-06	0.110818351	0.314852	0.136701237	0.123319	-0.178855164	0.021811	-0.320770079	0.001098	C1	-	Y	Y	-	-	-	Y
ENSG00000131398	KCNC3	-0.188149879	0.038958	0.312478498	8.37E-05	0.186541327	0.054102	0.507207623	4.04E-11	0.389487415	6.65E-06	-0.122960741	0.316957	C4	-	-	Y	-	Y	Y	-
ENSG00000131437	KIF3A	0.620898286	6.78E-18	0.246865455	0.012352	0.298784885	7.44E-05	-0.367560911	1.81E-07	-0.307534215	5.09E-08	0.054740316	0.698702	C5	-	Y	-	-	Y	Y	-
ENSG00000131470	PSMC3IP	0.024604693	0.834297	-0.26735417	0.015504	-0.302469166	0.002686	-0.285039245	0.001118	-0.312505177	0.000197	-0.03231212	0.895842	C4	-	-	-	Y	-	Y	-
ENSG00000131475	VPS25	-0.351657107	7.00E-06	-0.172535463	0.164264	-0.214382663	0.014453	0.185527738	0.058908	0.152180259	0.052117	-0.038898773	0.857353	C4	-	Y	-	-	-	-	-
ENSG00000131477	RAMP2	-0.632331687	0.004259	-0.376619201	0.189767	-0.545908643	0.028837	0.263033912	0.375746	0.101774684	0.764105	-0.167558325	0.748723	C4	-	Y	-	Y	-	-	-
ENSG00000131504	DIAPH1	0.417301792	4.77E-17	0.221810518	0.000192	0.121927141	0.073721	-0.188883537	3.54E-05	-0.280786112	1.70E-09	-0.097149526	0.212738	C5	-	Y	-	-	-	-	-
ENSG00000131626	PPFIA1	0.313706782	4.95E-06	0.226606966	0.003091	0.146446353	0.075589	-0.080969889	0.219034	-0.153066306	0.010515	-0.077339473	0.43015	C5	-	Y	-	-	-	-	-
ENSG00000131669	NINJ1	-0.346158389	0.019441	-0.170672329	0.396645	0.094133281	0.658342	0.182190778	0.269594	0.454930244	0.001138	0.267489969	0.196227	C4	-	Y	-	-	-	Y	-
ENSG00000131697	NPHP4	-0.254871744	0.016776	0.074957851	0.640634	0.083657623	0.516258	0.336502289	0.002091	0.353527699	0.000274	0.011633487	0.966961	C4	-	-	-	-	Y	Y	-
ENSG00000131725	WDR44	0.354465804	0.000313	0.259444476	0.024271	0.209680115	0.066626	-0.088445779	0.454611	-0.129856402	0.231655	-0.047053519	0.828189	C5	Y	Y	-	-	-	-	-
ENSG00000131732	ZCCHC9	0.454389436	1.15E-06	0.004595181	0.980869	0.271341384	0.013939	-0.443407188	2.20E-06	-0.16839063	0.108218	0.269601882	0.037528	C5	-	Y	-	-	Y	-	-
ENSG00000131759	RARA	-0.465295253	2.02E-09	-0.025562992	0.838464	-0.23556653	0.00313	0.446388528	1.56E-10	0.244612813	0.001204	-0.206988516	0.010298	C4	-	Y	-	-	Y	-	-
ENSG00000131771	PPP1R1B	-0.452752497	0.489654	-1.321795738	0.022506	-0.702725002	0.174906	-0.845280898	0.2914	-0.233829313	0.777736	0.616591647	NA	C5	-	-	Y	-	-	-	-
ENSG00000131773	KHDRBS3	0.391467555	8.42E-14	0.097717809	0.285642	0.113673331	0.164793	-0.287003502	1.34E-06	-0.263096236	7.79E-06	0.019046606	0.923337	C5	-	Y	-	-	-	-	-
ENSG00000131781	FMO5	-0.396044422	0.113201	0.031392444	0.942228	0.253782751	0.342374	0.433334485	0.074134	0.664021611	0.000849	0.226564833	0.519467	C4	-	-	-	-	-	Y	-
ENSG00000131791	PRKAB2	0.332287081	8.69E-06	0.184023636	0.043011	0.331638857	5.42E-05	-0.142020812	0.058238	0.013891329	0.896464	0.150569751	0.151113	C1	Y	Y	-	Y	-	-	-
ENSG00000131797	CLUHP3	0.353308475	0.009874	0.091096595	0.691668	0.342439357	0.02208	-0.256127733	0.017046	0.002663487	0.982872	0.253478244	0.077229	C1	-	Y	-	Y	-	-	-
ENSG00000131845	ZNF304	0.010862134	0.942843	-0.332835525	0.003074	-0.266636201	0.027076	-0.337330796	0.001289	-0.263019261	0.019334	0.069370087	0.78381	C4	-	-	Y	-	Y	-	-
ENSG00000131848	ZSCAN5A	-0.485289014	0.005434	-0.381579948	0.07907	-0.587237492	0.004988	0.110166246	0.655103	-0.086968983	0.751204	-0.201577881	0.597603	C4	-	Y	-	Y	-	-	-
ENSG00000131876	SNRPA1	-0.132555586	0.138504	0.029656545	0.857129	-0.275701182	0.005219	0.168772518	0.081655	-0.128316764	0.196284	-0.302146444	0.015354	C4	-	-	-	-	-	-	Y
ENSG00000131951	LRRC9	-4.60414453	0.002863	-3.228574472	NA	-0.343698107	NA	1.366862873	NA	4.275461823	NA	2.891568375	NA	C3	-	Y	-	-	-	-	-
ENSG00000132002	DNAJB1	0.775013767	1.81E-35	0.341657763	9.98E-07	0.653233217	2.03E-22	-0.426984613	2.95E-14	-0.107172484	0.109604	0.314423854	5.51E-06	C5	-	Y	Y	Y	Y	-	Y
ENSG00000132003	ZSWIM4	0.372504215	0.000612	0.086737694	0.609922	0.204512868	0.113654	-0.279427826	0.014328	-0.153333781	0.207421	0.120684748	0.53878	C5	-	Y	-	-	-	-	-
ENSG00000132005	RFX1	0.481913083	1.29E-05	0.092189951	0.605157	0.153030615	0.385827	-0.383245544	0.000844	-0.313635106	0.017572	0.064624851	0.835119	C5	-	Y	-	-	Y	Y	-
ENSG00000132016	C19orf57	-0.255195636	0.063301	-0.00447944	0.986961	0.041979898	0.822569	0.257248952	0.105556	0.311895801	0.020297	0.049604885	0.880775	C4	-	-	-	-	-	Y	-
ENSG00000132024	CC2D1A	0.418297871	2.30E-09	0.033076743	0.8369	0.141094508	0.120748	-0.37865744	3.36E-06	-0.262471388	0.000138	0.110819142	0.463182	C5	-	Y	-	-	Y	-	-
ENSG00000132026	RTBDN	-0.5243106	0.005734	-0.509478975	0.042066	-0.393635706	0.079216	0.023347044	0.951054	0.145050476	0.614433	0.115132153	0.840232	C4	-	Y	Y	-	-	-	-
ENSG00000132031	MATN3	-1.084605187	1.72E-13	-0.311440786	0.115421	-0.132720627	0.54873	0.781424295	3.16E-05	0.966889836	4.49E-08	0.18010952	0.605098	C3	Y	Y	-	-	Y	Y	-
ENSG00000132109	TRIM21	-0.913718197	0.028278	-0.395627631	0.455182	-0.439643573	0.378502	0.527465888	0.324948	0.489179665	0.369421	-0.042824817	NA	C3	-	Y	-	-	-	-	-
ENSG00000132141	CCT6B	-1.113388305	0.053187	0.05128655	0.95269	-0.832667005	0.224536	1.169313427	0.049316	0.295414221	0.755063	-0.879146992	NA	C3	-	-	-	-	Y	-	-
ENSG00000132164	SLC6A11	-0.436638139	0.000857	-0.275874291	0.089784	-0.194460612	0.303548	0.166957929	0.331496	0.256881788	0.136236	0.084927868	0.809023	C4	-	Y	-	-	-	-	-
ENSG00000132199	ENOSF1	-0.590173495	8.84E-10	-0.127665576	0.385252	-0.400269477	0.000118	0.469085994	1.06E-05	0.20469441	0.068966	-0.269640858	0.063194	C4	-	Y	-	Y	Y	-	-
ENSG00000132207	SLX1A	-0.373241794	0.668525	-1.690848946	0.054979	-0.981660463	0.265098	-1.315255046	0.027397	-0.591391833	0.33311	0.717424657	NA	C4	-	-	-	-	Y	-	-
ENSG00000132275	RRP8	0.545528488	9.87E-07	0.145124802	0.357462	0.265124956	0.041913	-0.394313412	2.19E-05	-0.266056536	0.005543	0.122793359	0.442185	C5	-	Y	-	-	Y	-	-
ENSG00000132300	PTCD3	0.256436265	0.00015	-0.061844128	0.561613	-0.010282368	0.931707	-0.311875354	7.82E-10	-0.252073043	3.86E-07	0.054392856	0.587834	C5	-	-	-	-	Y	-	-
ENSG00000132326	PER2	0.111801557	0.542261	-0.593592382	0.001235	-0.080614897	0.729582	-0.698393087	4.47E-05	-0.177540734	0.34614	0.514975707	0.024926	C5	-	-	Y	-	Y	-	Y
ENSG00000132330	SCLY	0.301770892	0.026758	0.169639108	0.401225	0.147950831	0.404782	-0.125435645	0.460688	-0.139125797	0.358357	-0.019105491	0.960328	C5	-	Y	-	-	-	-	-
ENSG00000132359	RAP1GAP2	0.325955377	1.85E-15	0.371443237	1.66E-17	0.37099677	5.56E-19	0.051853238	0.212463	0.059665151	0.106928	0.002525944	0.9809	C4	-	Y	Y	Y	-	-	-
ENSG00000132361	CLUH	0.345532263	8.34E-12	0.04781591	0.588164	-0.026568687	0.781415	-0.291004255	3.48E-09	-0.357647139	1.10E-12	-0.071515771	0.495627	C5	-	Y	-	-	-	Y	-
ENSG00000132382	MYBBP1A	0.271377588	2.41E-06	0.084957888	0.303155	-0.048952887	0.497381	-0.180058927	0.003342	-0.305696485	1.12E-09	-0.131220782	0.097949	C5	-	-	-	-	-	Y	-
ENSG00000132386	SERPINF1	-0.574962284	3.26E-08	-0.475514985	7.66E-05	-0.32627432	0.010044	0.105727913	0.444874	0.263742348	0.027139	0.152180585	0.446036	C4	Y	Y	Y	Y	-	-	-
ENSG00000132394	EEFSEC	-0.366894489	0.005849	0.035977116	0.852661	-0.042835764	0.801694	0.409461089	0.000303	0.338973195	0.002673	-0.075744251	0.663015	C4	Y	Y	-	-	Y	Y	-
ENSG00000132406	TMEM128	-0.329985177	3.88E-05	-0.155665119	0.110035	-0.251706975	0.005811	0.180894554	0.04726	0.093098855	0.38005	-0.093249383	0.544229	C4	-	Y	-	-	-	-	-
ENSG00000132424	PNISR	0.305311929	1.91E-10	0.173577376	0.001792	0.249645219	1.75E-06	-0.125315813	0.000651	-0.041028998	0.354002	0.078879987	0.17252	C5	-	Y	-	-	-	-	-
ENSG00000132429	POPDC3	-0.459548465	3.57E-05	-0.251402603	0.053284	-0.204807804	0.146865	0.214798334	0.099111	0.269622559	0.042609	0.049417272	0.862969	C4	-	Y	-	-	-	-	-
ENSG00000132434	LANCL2	-0.191894132	0.030485	-0.13803984	0.169942	-0.44125352	4.66E-07	0.059942097	0.594138	-0.234612182	0.015243	-0.299994336	0.00601	C5	-	-	-	Y	-	-	-
ENSG00000132436	FIGNL1	0.281589576	1.31E-05	-0.08274603	0.460768	0.036721185	0.758775	-0.357251457	4.93E-09	-0.230707153	0.000202	0.122248016	0.315115	C5	-	-	-	-	Y	-	-
ENSG00000132437	DDC	-0.605918946	4.98E-35	-0.204358085	0.000697	-0.206713018	9.57E-06	0.408108708	2.16E-14	0.414026201	7.97E-21	0.000583822	0.997161	C4	-	Y	-	-	Y	Y	-
ENSG00000132466	ANKRD17	0.556233426	7.62E-15	0.365630476	1.17E-05	0.374983532	2.20E-06	-0.184726598	0.00018	-0.167082914	0.000328	0.012356745	0.933843	C5	-	Y	Y	Y	-	-	-
ENSG00000132475	H3-3B	-0.567686877	1.82E-33	-0.217168938	0.001353	-0.379149402	2.96E-09	0.35714606	3.73E-18	0.203504075	7.56E-06	-0.159039639	0.04026	C4	-	Y	-	Y	Y	-	-
ENSG00000132510	KDM6B	0.46866141	5.60E-14	0.212035968	0.004279	0.492528348	6.59E-15	-0.25028467	5.63E-05	0.038541805	0.630705	0.283472844	4.00E-05	C5	-	Y	-	Y	-	-	-
ENSG00000132535	DLG4	0.459990079	3.24E-11	0.328887896	1.56E-05	0.465778577	3.92E-11	-0.124835135	0.079961	0.020451463	0.824481	0.139987593	0.123495	C1	-	Y	Y	Y	-	-	-
ENSG00000132570	PCBD2	-0.590354405	2.97E-08	-0.2307937	0.142987	-0.128365742	0.412603	0.366324357	0.006267	0.476733398	6.70E-05	0.105101355	0.69361	C4	-	Y	-	-	Y	Y	-
ENSG00000132613	MTSS2	0.432668467	7.69E-08	0.169091832	0.135193	0.148717146	0.173705	-0.257026119	0.003719	-0.269308632	0.001689	-0.01741876	0.943834	C5	-	Y	-	-	-	-	-
ENSG00000132622	HSPA12B	-0.978831915	0.028016	-0.271455791	0.603247	-0.179799351	0.752591	0.712939744	0.139284	0.81200776	0.104896	0.09479165	NA	C2	-	Y	-	-	-	-	-
ENSG00000132623	ANKEF1	-0.634719307	1.30E-06	-0.12518373	0.506535	-0.014932857	0.944619	0.515758253	0.000301	0.63442854	2.01E-06	0.113167237	0.642618	C4	-	Y	-	-	Y	Y	-
ENSG00000132635	PCED1A	-0.390318338	5.43E-08	-0.08984895	0.342762	-0.122207482	0.181928	0.307137012	1.81E-06	0.282989532	4.69E-05	-0.029593058	0.848238	C4	-	Y	-	-	Y	-	-
ENSG00000132646	PCNA	-0.402398827	3.03E-17	-0.24813114	0.001711	-0.45791496	3.37E-12	0.161051374	0.005175	-0.040873841	0.520049	-0.20677279	0.024557	C4	-	Y	-	Y	-	-	-
ENSG00000132677	RHBG	-1.561300275	0.007129	-0.798687998	0.194786	-0.313428279	0.629723	0.766742855	0.335753	1.263867867	0.056314	0.489164536	NA	C3	-	Y	-	-	-	-	-
ENSG00000132680	KHDC4	0.488041103	4.28E-17	0.32600118	2.69E-06	0.250103045	0.000211	-0.155440221	0.015829	-0.223276087	6.69E-05	-0.07302319	0.53385	C5	-	Y	Y	-	-	-	-
ENSG00000132692	BCAN	-0.501856584	0.017906	-0.099161744	0.74858	-0.265303457	0.28497	0.408991407	0.050203	0.251345144	0.287611	-0.163302795	0.641821	C4	-	Y	-	-	-	-	-
ENSG00000132694	ARHGEF11	0.490165033	8.23E-23	0.294804863	1.62E-06	0.260611947	4.27E-05	-0.188502821	2.77E-05	-0.214865064	5.64E-06	-0.031480065	0.794576	C1	-	Y	-	-	-	-	-
ENSG00000132698	RAB25	-0.872469959	0.222837	0.112538967	0.90121	-1.583983999	0.031198	0.990420569	0.110343	-0.696686141	0.444434	-1.694766866	NA	C2	-	-	-	Y	-	-	-

ENSG00000132740	IGHMBP2	0.684330693	2.14E-12	0.362754551	0.003055	0.373208929	0.001837	-0.315351379	0.000411	-0.296989541	0.001014	0.013155257	0.961147	C5	-	Y	Y	Y	Y	-	-
ENSG00000132749	TESMIN	-0.586993583	0.000339	-0.28950408	0.16632	-0.103507662	0.65635	0.302653352	0.151864	0.498219724	0.007278	0.189453427	0.554873	C4	-	Y	-	-	-	Y	-
ENSG00000132763	MMACHC	0.33546707	0.006417	0.004958956	0.984188	0.258672147	0.062848	-0.324073599	0.008785	-0.062059067	0.686871	0.256738284	0.125018	C5	-	Y	-	-	Y	-	-
ENSG00000132773	TOE1	-0.294190879	0.00905	-0.203403345	0.114439	-0.485846995	1.80E-05	0.097274542	0.482937	-0.176959588	0.179276	-0.279564213	0.061412	C5	-	-	-	Y	-	-	-
ENSG00000132780	NASP	-0.189772943	3.44E-09	-0.234403331	1.18E-05	-0.362983105	1.29E-20	-0.038084602	0.46889	-0.158628014	8.74E-07	-0.125723405	0.059765	C4	-	-	-	Y	-	-	-
ENSG00000132781	MUTYH	-0.143377981	0.297441	-0.350613651	0.015845	-0.288873199	0.044404	-0.200729349	0.129048	-0.130377636	0.356311	0.064635556	0.818577	C4	-	-	Y	-	-	-	-
ENSG00000132792	CTNNBL1	0.337713331	8.80E-07	0.145913471	0.174041	0.059258477	0.605079	-0.184719871	0.015028	-0.263718566	9.97E-05	-0.083944226	0.568426	C5	-	Y	-	-	-	-	-
ENSG00000132801	ZSWIM3	0.292882229	0.161459	-0.619813014	0.010311	-0.038796495	0.931083	-0.906228905	1.03E-05	-0.317835935	0.262346	0.582134528	0.149786	C5	-	-	Y	-	Y	-	-
ENSG00000132821	VSTM2L	-0.794406757	7.21E-11	-0.341859618	0.01858	-0.377404751	0.003027	0.458187855	0.001955	0.431886358	0.002113	-0.032233444	0.930354	C3	-	Y	Y	Y	Y	Y	-
ENSG00000132823	OSER1	-0.063880532	0.588443	-0.354565199	0.000178	-0.265937326	0.012451	-0.284588125	0.004623	-0.187241765	0.094058	0.091995595	0.636064	C4	-	-	Y	-	-	-	-
ENSG00000132824	SERINC3	0.400504285	6.35E-12	0.118010887	0.168624	0.161143684	0.015878	-0.276101823	1.03E-05	-0.22458334	5.05E-05	0.046221262	0.717587	C5	-	Y	-	-	-	-	-
ENSG00000132846	ZBED3	-0.476841482	1.09E-07	-0.16859986	0.120688	-0.395157904	8.94E-05	0.314851557	0.001049	0.096294859	0.441637	-0.223824522	0.107677	C4	-	Y	-	Y	Y	-	-
ENSG00000132872	SYT4	-0.331629368	5.93E-13	-0.171033339	0.018706	-0.10237881	0.107601	0.167240026	0.000877	0.243578691	1.20E-10	0.071358434	0.472263	C4	-	Y	-	-	-	-	-
ENSG00000132879	FBXO44	-0.200488896	0.065387	0.025412137	0.892009	0.08734469	0.566983	0.232372141	0.032767	0.302963198	0.004444	0.064711278	0.780412	C4	-	-	-	-	-	Y	-
ENSG00000132906	CASP9	-0.191084629	0.225654	-0.193091737	0.30773	0.124555809	0.54669	0.005309774	0.981051	0.330623927	0.048636	0.319533075	0.166977	C4	-	-	-	-	-	Y	-
ENSG00000132932	ATP8A2	-2.070396787	0.349124	-0.353049049	NA	1.404077331	NA	1.7201982	NA	3.495170048	0.031716	1.757592851	NA	C3	-	-	-	-	-	Y	-
ENSG00000132952	USPL1	0.37880131	1.83E-07	-0.09242162	0.422424	0.160543005	0.057678	-0.464698037	7.37E-10	-0.203512281	0.004521	0.255925502	0.006642	C5	-	Y	-	-	Y	-	-
ENSG00000132958	TPTE2	4.264592969	0.003572	2.984878603	NA	3.313334304	NA	-1.248458011	0.335599	-0.941132093	0.395714	0.317221491	NA	C1	-	Y	-	-	-	-	-
ENSG00000132964	CDK8	0.422908146	1.07E-06	0.310713346	0.001538	0.173996194	0.094924	-0.105718492	0.299527	-0.234213989	0.007207	-0.13392301	0.325107	C1	-	Y	Y	-	-	-	-
ENSG00000132967	HMGB1P5	-0.199285541	0.000843	-0.163353443	0.02554	-0.317003338	5.77E-06	0.042346178	0.596411	-0.103145189	0.162744	-0.150600345	0.132933	C4	-	-	-	Y	-	-	-
ENSG00000133056	PIK3C2B	-0.295897466	0.000835	-0.149116092	0.232448	0.066716825	0.59497	0.152977471	0.123348	0.377260089	1.10E-06	0.219300563	0.060391	C4	-	-	-	-	-	Y	-
ENSG00000133059	DSTYK	0.536330045	9.10E-12	0.187114232	0.070583	0.287441485	0.001447	-0.342930899	2.16E-06	-0.234164372	0.001331	0.103353633	0.422915	C5	-	Y	-	-	Y	-	-
ENSG00000133065	SLC41A1	0.595723168	2.87E-13	0.223421119	0.033985	0.403943452	1.45E-06	-0.366674446	4.85E-08	-0.177347605	0.005039	0.184239452	0.032803	C5	-	Y	-	Y	Y	-	-
ENSG00000133069	TMCC2	0.04029795	0.793341	0.334948464	0.010097	0.405016845	0.000518	0.300852061	0.00605	0.37946688	0.000209	0.073301454	0.728646	C4	-	-	Y	Y	Y	Y	-
ENSG00000133083	DCLK1	-0.239859694	0.039068	-0.398124974	0.000682	-0.011627369	0.948064	-0.1520546	0.243755	0.24269211	0.027075	0.389077246	0.001083	C4	-	-	Y	-	-	-	Y
ENSG00000133103	COG6	0.483914026	9.24E-07	0.24252588	0.068216	0.284030385	0.017985	-0.235130303	0.023308	-0.185354848	0.075379	0.044294832	0.857353	C5	-	Y	-	-	-	-	-
ENSG00000133104	SPART	-0.486812961	5.99E-06	-0.281834626	0.014594	0.089149371	0.51793	0.211396017	0.08561	0.590588292	1.43E-08	0.373931947	0.00128	C4	-	Y	-	-	-	Y	Y
ENSG00000133119	RFC3	-0.165359914	0.004928	-0.091245428	0.166899	-0.392542359	2.45E-10	0.080709549	0.185911	-0.212546982	0.000496	-0.298456085	2.12E-06	C5	-	-	-	Y	-	-	-
ENSG00000133195	SLC39A11	-0.724800871	2.97E-11	-0.165887802	0.280196	-0.274477082	0.043965	0.56511446	2.38E-06	0.465219858	0.000254	-0.105542068	0.667135	C3	-	Y	-	-	Y	Y	-
ENSG00000133226	SRRM1	0.278659807	4.69E-06	-0.050662934	0.634426	-0.087072632	0.324004	-0.322608186	5.35E-14	-0.351454122	7.07E-17	-0.033900256	0.772475	C5	-	-	-	-	Y	Y	-
ENSG00000133275	CSNK1G2	0.333214732	3.02E-08	0.151816566	0.060655	0.135373298	0.060861	-0.174810545	0.007711	-0.183094686	0.002036	-0.013627773	0.939246	C5	-	Y	-	-	-	-	-
ENSG00000133302	SLF1	0.600293264	2.83E-15	0.148405852	0.195534	0.167432556	0.084662	-0.444696648	1.14E-11	-0.418398467	3.44E-12	0.020989324	0.910675	C5	-	Y	-	-	Y	Y	-
ENSG00000133398	MED10	-0.134122641	0.076575	-0.187394262	0.031458	-0.416700131	1.21E-07	-0.046738844	0.622383	-0.267498411	0.000401	-0.226272058	0.028257	C5	-	-	-	Y	-	-	-
ENSG00000133454	MYO18B	-0.932258692	0.000745	0.111871928	0.774924	0.052248035	0.893945	1.050646172	0.000178	0.999572992	0.000358	-0.056712909	0.928882	C3	-	Y	-	-	Y	Y	-
ENSG00000133460	SLC2A11	-0.604637026	1.30E-08	-0.090351992	0.604259	-0.096343034	0.506034	0.52048753	2.15E-06	0.523100309	1.84E-07	-0.002415843	0.994002	C4	-	Y	-	-	Y	Y	-
ENSG00000133466	C1QTNF6	-0.566400308	5.51E-09	-0.049248511	0.761747	0.020075616	0.892955	0.524021376	3.39E-07	0.601240637	5.01E-11	0.072744125	0.686456	C2	-	Y	-	-	Y	Y	-
ENSG00000133597	ADCK2	-0.291567362	0.054624	-0.046603506	0.840143	0.019217019	0.934509	0.251551157	0.076381	0.325609451	0.017812	0.068610289	0.794977	C4	-	-	-	-	-	Y	-
ENSG00000133612	AGAP3	0.333452321	1.42E-14	0.264155231	9.35E-07	0.106013303	0.076867	-0.063009115	0.178839	-0.212753954	1.35E-08	-0.155110882	0.005006	C5	-	Y	-	-	-	-	-
ENSG00000133619	KRBA1	0.311666571	0.000424	0.179679005	0.108243	0.171181929	0.15179	-0.125688417	0.193014	-0.125441305	0.233705	-0.005441421	0.982475	C5	-	Y	-	-	-	-	-
ENSG00000133641	C12orf29	0.302991799	4.24E-06	-0.076602531	0.478464	-0.037541095	0.754771	-0.373136194	4.55E-08	-0.325832211	4.39E-06	0.041956765	0.81713	C5	-	Y	-	-	Y	Y	-
ENSG00000133657	ATP13A3	0.535699152	4.81E-24	0.268347117	0.000191	0.152364074	0.020907	-0.260853803	1.29E-07	-0.368673113	3.31E-22	-0.113176919	0.152125	C5	-	Y	-	-	-	Y	-
ENSG00000133661	SFTPD	-2.816586797	0.002306	-0.988156972	NA	-1.242862471	0.125737	1.843859977	0.168208	1.590893081	0.228811	-0.253139791	NA	C3	-	Y	-	-	-	-	-
ENSG00000133687	TMTC1	-0.933513249	0.013752	-0.352534885	0.472078	-0.457203362	0.30528	0.590078035	0.237339	0.490732971	0.345153	-0.103947778	NA	C2	-	Y	-	-	-	-	-
ENSG00000133704	IPO8	0.340847869	5.98E-08	0.178766802	0.024215	0.181864147	0.011374	-0.155895499	0.002712	-0.144600403	0.002698	0.005940681	0.967582	C5	-	Y	-	-	-	-	-
ENSG00000133739	LRRCC1	-0.453695103	0.000468	0.019347751	0.92466	-0.001641838	0.992869	0.479131865	0.000521	0.466923227	0.000336	-0.018014297	0.953146	C2	-	Y	-	-	Y	Y	-
ENSG00000133740	E2F5	0.325723142	0.001469	0.223128368	0.098671	0.118819703	0.420666	-0.096328053	0.46308	-0.192267281	0.092928	-0.101395902	0.634616	C5	-	Y	-	-	-	-	-
ENSG00000133773	CCDC59	0.141610495	0.137983	-0.100871675	0.421024	-0.189237146	0.123014	-0.236232523	0.011816	-0.316248221	0.002089	-0.084910226	0.686381	C5	-	-	-	-	-	Y	-
ENSG00000133794	ARNTL	-0.393180143	2.19E-05	0.079051706	0.547218	0.077466749	0.620514	0.478978436	2.00E-07	0.485328429	6.55E-06	0.000755603	0.997573	C4	-	Y	-	-	Y	Y	-
ENSG00000133805	AMPD3	-1.13062743	2.15E-06	-0.605597613	0.009287	-0.496987517	0.027887	0.531447313	0.070114	0.648051479	0.018733	0.111451437	0.824504	C3	-	Y	Y	Y	-	Y	-
ENSG00000133816	MICAL2	0.680726655	0.014868	1.048725715	0.000119	1.172266785	4.94E-06	0.374593312	0.11999	0.507125772	0.024937	0.127367721	0.763472	C1	-	Y	Y	Y	-	Y	-
ENSG00000133858	ZFC3H1	0.730232994	2.53E-18	0.505686686	1.16E-07	0.599723771	2.35E-12	-0.218336182	0.006755	-0.116007055	0.14195	0.097034077	0.490512	C1	-	Y	Y	Y	-	-	-
ENSG00000133884	DPF2	0.35052263	5.11E-09	0.030494655	0.807118	0.186500063	0.009341	-0.313326046	1.46E-07	-0.149384759	0.007352	0.158720923	0.067891	C5	Y	Y	-	-	Y	-	-
ENSG00000133895	MEN1	0.379286521	6.83E-09	0.156181338	0.10126	0.236110142	0.00079	-0.216403954	0.002278	-0.128419894	0.040545	0.082593814	0.500833	C5	-	Y	-	-	-	-	-
ENSG00000133943	DGLUCY	-0.68855548	2.43E-07	-0.331103357	0.075184	-0.151145652	0.427686	0.365004109	0.05083	0.552043703	0.000589	0.181204056	0.572186	C4	-	Y	-	-	-	Y	-
ENSG00000133961	NUMB	0.532646952	2.08E-27	0.259159175	1.86E-05	0.334102936	1.55E-09	-0.267350289	1.03E-09	-0.183787353	1.75E-05	0.078062916	0.310184	C5	-	Y	-	Y	-	-	-
ENSG00000134013	LOXL2	0.256586592	0.158693	0.488258691	0.014884	0.505518724	0.00991	0.238449964	0.236001	0.263908211	0.183779	0.019235919	0.971024	C4	-	-	Y	Y	-	-	-
ENSG00000134072	CAMK1	-0.598274353	0.00011	-0.31684545	0.083536	-0.322059392	0.05699	0.289045417	0.143345	0.290835762	0.128938	-0.002937097	0.995721	C4	-	Y	-	-	-	-	-
ENSG00000134107	BHLHE40	1.923868323	0.024188	1.926071008	NA	1.269160773	NA	0.007035986	0.993083	-0.64114706	0.460979	-0.653802284	NA	C1	-	Y	-	-	-	-	-
ENSG00000134115	CNTN6	-0.427292874	0.511512	0.508874638	0.444895	0.921341707	0.07672	0.942229016	0.095165	1.362054341	0.006288	0.415300388	NA	C4	-	-	-	-	-	Y	-
ENSG00000134138	MEIS2	-0.562255642	4.49E-45	-0.250739318	1.38E-08	-0.304082838	1.18E-18	0.318161893	5.58E-14	0.272929893	1.22E-14	-0.050376073	0.460887	C4	-	Y	-	Y	Y	-	-
ENSG00000134146	DPH6	-0.330919501	0.032926	0.154740389	0.52944	-0.045250374	0.841293	0.49232332	0.003429	0.300268375	0.046718	-0.196514517	0.471971	C4	-	Y	-	-	Y	Y	-
ENSG00000134152	KATNBL1	0.665443859	4.55E-19	0.231152836	0.020342	0.333376175	5.73E-05	-0.42768359	4.21E-10	-0.317831375	6.89E-07	0.104497615	0.395182	C5	-	Y	-	Y	Y	Y	-
ENSG00000134153	EMC7	0.322997062	9.68E-06	-0.085017481	0.556681	-0.129410122	0.266905	-0.401882381	3.53E-06	-0.437879139	4.08E-08	-0.04168718	0.870117	C5	Y	Y	-	-	Y	Y	-
ENSG00000134186	PRPF38B	0.316267454	4.47E-10	0.027587581	0.798232	-0.036474405	0.695504	-0.281815418	7.32E-09	-0.338227588	3.21E-13	-0.06131461	0.586375	C5	-	Y	-	-	-	Y	-
ENSG00000134215	VAV3	-0.855684321	0.005381	-0.050954532	0.915404	0.135009586	0.729143	0.809909313	0.010197	1.004752818	0.000713	0.188333018	NA	C2	-	Y	-	-	Y	Y	-
ENSG00000134250	NOTCH2	-0.155621951	0.076418	0.360232569	0.000816	0.440122143	2.62E-06	0.521907186	2.09E-12	0.610495606	4.34E-21	0.08278598	0.627766	C4	-	-	Y	Y	Y	Y	-
ENSG00000134255	CEPT1	0.611668036	2.91E-12	0.246662897	0.042074	0.456327627	1.10E-05	-0.357994546	1.92E-05	-0.140604761	0.135626	0.212244319	0.107682	C1	-	Y	-	Y	Y	-	-

ENSG00000134278	SPIRE1	0.577418734	3.13E-14	0.196805	0.057569	0.196693735	0.045314	-0.374934022	3.37E-08	-0.366104484	3.69E-08	0.003050345	0.989495	C5	Y	Y	-	-	Y	Y	-
ENSG00000134318	ROCK2	0.491990953	5.08E-11	0.309508464	0.001791	0.202142827	0.038155	-0.175656367	0.008964	-0.275166916	2.18E-06	-0.104768897	0.404505	C1	-	Y	Y	-	-	-	-
ENSG00000134321	RSAD2	-1.374851685	0.01244	-1.107239735	0.071875	-0.678108885	0.311239	0.279531372	0.744983	0.711911942	0.325309	0.428047677	NA	C3	-	Y	-	-	-	-	-
ENSG00000134323	MYCN	-0.594084655	0.000229	-0.526809645	0.007143	-0.518351335	0.007859	0.073080445	0.774654	0.089974006	0.722947	0.012543977	0.982152	C4	-	Y	Y	Y	-	-	-
ENSG00000134324	LPIN1	0.36983278	4.76E-13	0.146707128	0.035471	0.235958393	0.000308	-0.216617302	2.58E-05	-0.11943679	0.037909	0.092178332	0.361022	C5	-	Y	-	-	-	-	-
ENSG00000134333	LDHA	-0.030116854	0.426058	0.319156588	4.41E-13	0.258214478	4.04E-08	0.355840456	6.51E-24	0.303171765	1.28E-17	-0.058174498	0.503233	C4	-	-	Y	-	Y	Y	-
ENSG00000134352	IL6ST	0.624873392	2.09E-17	0.3145416	0.00167	0.408550954	3.71E-07	-0.304772023	6.14E-07	-0.201878448	1.66E-05	0.097592909	0.364363	C5	-	Y	Y	Y	Y	-	-
ENSG00000134376	CRB1	-1.026094146	8.56E-06	-0.202930295	0.489506	0.134134293	0.645668	0.829746895	0.001268	1.174781122	9.97E-07	0.339860122	0.300956	C2	-	Y	-	-	Y	Y	-
ENSG00000134463	ECHDC3	-0.991973962	1.12E-06	-0.470555343	0.042506	-0.394101034	0.084347	0.528164225	0.015449	0.613109532	0.003874	0.080133869	0.858374	C4	-	Y	Y	-	Y	Y	-
ENSG00000134490	TMEM241	-0.302782906	0.039226	-0.282810665	0.106264	-0.058369632	0.775981	0.026663684	0.912737	0.259067009	0.117108	0.227010437	0.35774	C4	-	Y	-	-	-	-	-
ENSG00000134508	CABLES1	-0.532201622	0.000639	-0.262587475	0.139628	-0.115444649	0.557332	0.275441659	0.134869	0.431316589	0.009739	0.150075225	0.574359	C4	-	Y	-	-	-	Y	-
ENSG00000134516	DOCK2	-0.602942152	0.496225	1.266978849	0.045135	0.748462871	0.320631	1.876274616	0.001237	1.36638089	0.041352	-0.510779362	NA	C2	-	-	Y	-	Y	Y	-
ENSG00000134531	EMP1	2.791996166	0.001915	2.309246672	NA	2.216601052	NA	-0.474404866	0.594033	-0.55961254	0.473294	-0.093726606	NA	C1	-	Y	-	-	-	-	-
ENSG00000134533	RERG	-1.45265125	0.000785	-1.010423845	0.048524	-1.139246554	0.012484	0.447751157	0.533467	0.327831671	0.652738	-0.126020471	NA	C3	-	Y	Y	Y	-	-	-
ENSG00000134569	LRP4	-0.242657455	0.141755	-0.065337014	0.783416	0.147512345	0.404353	0.184285783	0.303567	0.40487785	0.005519	0.216147878	0.304421	C4	-	-	-	-	-	Y	-
ENSG00000134574	DDB2	-0.495994569	1.51E-07	-0.045227459	0.790519	-0.211099648	0.055375	0.457259699	1.93E-08	0.29956973	0.000197	-0.163136116	0.169071	C4	-	Y	-	-	Y	-	-
ENSG00000134575	ACP2	-0.362113205	0.000898	-0.128598512	0.400767	-0.133282527	0.388881	0.23992789	0.025516	0.243793385	0.033373	-0.001687175	0.996048	C4	-	Y	-	-	-	-	-
ENSG00000134668	SPOCD1	1.585417412	9.68E-05	1.466895148	0.00314	0.928995086	0.071735	-0.109698741	0.834298	-0.6413865	0.105999	-0.537238939	NA	C1	-	Y	Y	-	-	-	-
ENSG00000134684	YARS1	-0.641574366	5.47E-29	-0.603431388	4.08E-11	-0.174712085	0.078616	0.044722688	0.696309	0.481346101	3.17E-11	0.432965958	0.021249	C3	-	Y	Y	-	-	Y	Y
ENSG00000134697	GNL2	0.385792434	2.42E-14	0.041843484	0.631489	-0.01282216	0.889326	-0.337488383	1.30E-11	-0.384127537	1.85E-16	-0.051898916	0.613799	C5	Y	Y	-	-	Y	Y	-
ENSG00000134709	HOOK1	-0.226308938	0.151036	-0.323776163	0.052568	0.064682092	0.750559	-0.091187265	0.639708	0.305387651	0.0382	0.391374233	0.025064	C4	-	-	-	-	-	Y	Y
ENSG00000134716	CYP2J2	-0.847936398	0.019503	-0.727108324	0.073984	-0.216177392	0.653882	0.126579516	0.820134	0.645727156	0.115229	0.513671492	NA	C4	-	Y	-	-	-	-	-
ENSG00000134769	DTNA	0.436456647	4.02E-08	0.018030503	0.921329	0.129853964	0.286824	-0.411011029	3.31E-07	-0.292179287	0.000218	0.114580045	0.499042	C5	-	Y	-	-	Y	-	-
ENSG00000134802	SLC43A3	-0.319843101	0.349898	-0.766599827	0.038105	-0.799420584	0.043372	-0.439179929	0.286235	-0.464739224	0.296997	-0.032326054	NA	C3	-	-	Y	Y	-	-	-
ENSG00000134815	DHX34	0.762846644	1.33E-21	0.30907077	0.00338	0.413343935	2.75E-06	-0.447119997	2.22E-08	-0.334984963	4.98E-06	0.106951074	0.469789	C5	-	Y	Y	Y	Y	Y	-
ENSG00000134817	APLNR	-0.481299228	0.102548	0.412092185	0.148319	0.782416174	0.000546	0.899346347	0.000772	1.27808226	9.82E-08	0.372995757	0.245654	C4	-	-	-	Y	Y	Y	-
ENSG00000134851	TMEM165	0.29676798	3.00E-07	-0.006050858	0.961488	-0.022671609	0.831713	-0.296408016	1.11E-08	-0.304725018	9.96E-09	-0.013666498	0.931488	C5	-	-	-	-	-	Y	-
ENSG00000134853	PDGFRA	-0.766485552	2.19E-33	-0.651346726	2.30E-22	-0.743021219	6.55E-29	0.121686232	0.101797	0.038063179	0.679779	-0.088880617	0.469108	C4	-	Y	Y	Y	-	-	-
ENSG00000134864	GGACT	-1.587994715	2.07E-06	-0.437008389	0.247926	-0.517184684	0.134792	1.159512272	0.003419	1.084669755	0.00697	-0.078655804	NA	C3	-	Y	-	-	Y	Y	-
ENSG00000134871	COL4A2	-0.282481164	9.25E-07	0.090706371	0.302625	0.200625899	0.000787	0.379571147	3.62E-10	0.497744264	2.77E-23	0.113076055	0.203996	C4	-	-	-	-	Y	Y	-
ENSG00000134899	ERCC5	0.596864781	0.001983	0.172615837	0.519518	-0.041436536	0.899045	-0.418180425	0.02987	-0.623921104	0.000997	-0.211180074	0.511403	C5	-	Y	-	-	Y	Y	-
ENSG00000134900	TPP2	0.361256302	1.14E-09	0.102014063	0.2103	0.160407062	0.022622	-0.252902123	4.28E-08	-0.186396775	7.38E-05	0.061122761	0.482528	C5	-	Y	-	-	-	-	-
ENSG00000134901	POGLUT2	0.416053886	1.87E-08	-0.021811927	0.885023	0.086368656	0.404674	-0.431537333	4.63E-08	-0.314880255	2.70E-05	0.110993619	0.431247	C5	-	Y	-	-	Y	Y	-
ENSG00000134940	ACRV1	-0.570192977	0.216016	-0.564418683	0.297516	-1.088525807	0.035579	0.013255299	0.983834	-0.502989209	0.428358	-0.521810948	NA	C5	-	-	-	Y	-	-	-
ENSG00000134954	ETS1	-0.922290842	1.72E-33	-0.343877726	3.97E-05	-0.178325225	0.029198	0.584812318	1.31E-13	0.758420289	4.04E-27	0.168750934	0.096483	C4	-	Y	Y	-	Y	Y	-
ENSG00000134955	SLC37A2	1.109157695	0.005969	0.462244925	0.476179	0.217550569	0.761285	-0.642672384	0.070914	-0.878498934	0.013167	-0.24129546	NA	C5	-	Y	-	-	-	Y	-
ENSG00000134987	WDR36	0.426138617	5.02E-11	-0.001893682	0.991198	0.076144931	0.469343	-0.420798666	1.31E-12	-0.335294524	4.82E-10	0.080500854	0.547978	C5	-	Y	-	-	Y	Y	-
ENSG00000135002	RFK	0.495569866	2.18E-06	0.259471347	0.06309	0.312512524	0.013139	-0.228979886	0.031987	-0.16868622	0.119236	0.055425111	0.818037	C5	-	Y	-	Y	-	-	-
ENSG00000135045	C9orf40	0.441858808	0.000389	-0.025617711	0.916584	0.209845781	0.219002	-0.460716066	0.00023	-0.217292269	0.12327	0.238438517	0.247181	C5	-	Y	-	-	Y	-	-
ENSG00000135048	CEMIP2	1.843307548	0.026548	1.386253836	NA	1.472466843	NA	-0.446484597	0.562194	-0.357895113	0.663477	0.084329656	NA	C1	-	Y	-	-	-	-	-
ENSG00000135049	AGTPBP1	-0.108102556	0.171136	0.215757223	0.017795	0.133090663	0.150372	0.329999301	5.79E-06	0.255993614	0.00039	-0.079941977	0.591359	C4	-	-	-	-	Y	-	-
ENSG00000135069	PSAT1	-1.090520064	4.94E-80	-0.701987829	2.28E-16	-0.497114241	9.25E-09	0.395012048	1.19E-06	0.607948763	8.18E-16	0.209293762	0.376055	C3	Y	Y	Y	Y	Y	Y	-
ENSG00000135074	ADAM19	-0.177941563	0.269385	0.367791236	0.01838	0.600905518	1.91E-06	0.552635034	0.000146	0.793384985	5.62E-10	0.236178548	0.221658	C4	-	-	Y	Y	Y	Y	-
ENSG00000135093	USP30	0.483144581	1.50E-06	0.204067531	0.084243	0.235111124	0.038496	-0.272690782	0.006316	-0.233461994	0.023944	0.03391323	0.88523	C5	Y	Y	-	-	-	-	-
ENSG00000135097	MSI1	0.179251863	0.084232	-0.196212101	0.13494	-0.128478112	0.319828	-0.368911644	0.000211	-0.293231623	0.002216	0.070482932	0.749253	C4	-	-	-	-	Y	-	-
ENSG00000135124	P2RX4	-0.888584079	9.78E-09	-0.803755937	1.03E-06	-0.506956473	0.001874	0.090345055	0.726771	0.396356704	0.037203	0.300163958	0.262165	C4	-	Y	Y	Y	-	Y	-
ENSG00000135245	HILPDA	-0.199056768	0.043127	0.100076858	0.373921	-0.058419699	0.643597	0.305886466	0.001008	0.155343225	0.140501	-0.155606726	0.219285	C4	-	-	-	-	Y	-	-
ENSG00000135250	SRPK2	0.305901927	2.44E-08	0.182610517	0.002448	0.185836534	0.000987	-0.116858258	0.044781	-0.105349057	0.061921	0.006029626	0.967337	C5	-	Y	-	-	-	-	-
ENSG00000135299	ANKRD6	-0.898502914	1.63E-05	-0.620701274	0.017636	-0.269173411	0.272338	0.284244622	0.309436	0.644142277	0.001425	0.354521558	0.279479	C3	-	Y	Y	-	-	Y	-
ENSG00000135318	NT5E	0.035568255	0.953922	1.671788559	9.08E-07	1.495885369	7.52E-06	1.64364576	5.42E-07	1.474889076	6.92E-06	-0.172980039	0.79673	C2	-	-	Y	Y	Y	Y	-
ENSG00000135336	ORC3	0.312945301	3.01E-06	0.073256527	0.486605	0.088099249	0.343372	-0.233267466	0.000673	-0.210345752	0.001823	0.017618026	0.927911	C5	-	Y	-	-	-	-	-
ENSG00000135338	LCA5	0.57384589	2.69E-05	0.170590126	0.428121	0.529583298	0.00089	-0.398176353	0.001038	-0.03024276	0.862542	0.362296797	0.03266	C5	-	Y	-	Y	Y	-	Y
ENSG00000135341	MAP3K7	0.388913667	1.13E-12	0.16825197	0.03495	0.185697053	0.007757	-0.214662517	0.000126	-0.188609936	0.000174	0.020543062	0.898057	C5	-	Y	-	-	-	-	-
ENSG00000135372	NAT10	0.366905313	3.25E-08	0.095683011	0.224378	0.084587788	0.262286	-0.2648941	3.90E-05	-0.26751574	1.62E-05	-0.008272536	0.958938	C5	-	Y	-	-	-	-	-
ENSG00000135406	PRPH	-0.4995548	0.045958	0.007619368	0.98614	-0.202262583	0.509744	0.514437079	0.050806	0.312130372	0.288056	-0.207103174	0.630819	C2	-	Y	-	-	-	-	-
ENSG00000135423	GLS2	-1.703552704	0.028769	-1.301205404	NA	-0.920972397	0.242964	0.407461042	0.759634	0.793361544	0.474421	0.380737803	NA	C3	-	Y	-	-	-	-	-
ENSG00000135424	ITGA7	-0.437231578	0.005176	-0.302439452	0.078553	-0.213395244	0.180942	0.14002855	0.509742	0.238676032	0.176376	0.091962523	0.765768	C4	-	Y	-	-	-	-	-
ENSG00000135441	BLOC1S1	-0.411835223	0.000631	-0.209889362	0.170679	-0.261385402	0.061902	0.208608908	0.116955	0.165294476	0.233883	-0.048623998	0.863083	C4	-	Y	-	-	-	-	-
ENSG00000135472	FAIM2	-0.670650768	1.27E-08	-0.498276439	0.000103	-0.341939795	0.02329	0.17864328	0.210504	0.343320947	0.01538	0.159244162	0.500085	C4	-	Y	Y	Y	-	Y	-
ENSG00000135482	ZC3H10	0.141926518	0.265594	-0.231843954	0.105004	-0.018573681	0.925448	-0.367004199	0.000369	-0.146446655	0.205442	0.215473571	0.168156	C5	-	-	-	-	Y	-	-
ENSG00000135486	HNRNPA1	-0.090654875	0.010663	-0.146641278	0.013429	-0.307805221	2.23E-13	-0.049499088	0.292456	-0.202269815	6.49E-12	-0.158263009	0.006714	C4	-	-	-	Y	-	-	-
ENSG00000135541	AHI1	0.493092643	1.04E-11	0.370775888	1.23E-05	0.329786415	4.67E-05	-0.115742646	0.145119	-0.148638481	0.042549	-0.038243961	0.81521	C1	Y	Y	Y	Y	-	-	-
ENSG00000135622	SEMA4F	-0.287474322	0.002936	0.006234686	0.977466	0.085274945	0.531017	0.30022697	0.009718	0.387665061	5.69E-05	0.081855459	0.720648	C4	-	-	-	-	Y	Y	-
ENSG00000135655	USP15	0.337793981	0.000412	0.059269443	0.712241	0.046638176	0.767769	-0.272458267	2.33E-05	-0.277460797	3.69E-05	-0.010240541	0.959571	C5	-	Y	-	-	-	-	-
ENSG00000135686	KLHL36	0.118936326	0.398009	0.191264513	0.282644	0.483456517	2.73E-05	0.078671471	0.651824	0.379048839	0.000619	0.295085502	0.092206	C4	-	-	-	Y	-	Y	-
ENSG00000135698	MPHOSPH6	0.360454219	0.002678	0.16818092	0.303155	0.092841206	0.603567	-0.185876645	0.1543	-0.2529184	0.045002	-0.072473604	0.778766	C5	-	Y	-	-	-	-	-
ENSG00000135709	KIAA0513	0.50973492	0.003358	0.348878249	0.132815	0.638862327	0.000344	-0.154478993	0.470566	0.143800674	0.458453	0.29244804	0.259086	C1	-	Y	-	Y	-	-	-

ENSG00000135723	FHOD1	0.342495387	1.26E-07	0.249574305	0.000467	0.297840663	1.13E-05	-0.08652477	0.252495	-0.02992358	0.737053	0.051191116	0.681234	C5	Y	Y	-	-	-	-	-
ENSG00000135736	CCDC102A	-0.397563719	0.053835	-0.115207979	0.695334	0.125724042	0.659763	0.289085593	0.139506	0.538250057	0.003565	0.244158778	0.397673	C4	-	-	-	-	-	Y	-
ENSG00000135747	ZNF670-ZNF695	0.847757248	0.029857	0.38570244	0.573461	0.715050069	0.131342	-0.454282177	0.357597	-0.117284082	0.82112	0.330901885	NA	C1	-	Y	-	-	-	-	-
ENSG00000135750	KCNK1	0.089686604	0.465408	-0.297301611	0.020813	-0.071723246	0.710481	-0.380503087	0.000733	-0.14695538	0.31645	0.229288403	0.282245	C5	-	-	-	-	Y	-	-
ENSG00000135763	URB2	0.177242208	0.024714	-0.161256546	0.127663	-0.046737407	0.688781	-0.331523205	5.01E-07	-0.209646382	0.000601	0.116757278	0.309109	C5	-	-	-	-	Y	-	-
ENSG00000135766	EGLN1	0.298261562	0.090941	0.661255601	9.93E-06	0.367598185	0.051702	0.36961144	0.022809	0.084211125	0.720589	-0.290498373	0.210097	C4	-	-	Y	-	Y	-	-
ENSG00000135824	RGS8	-4.62916656	1.83E-07	-1.33489808	0.018755	-1.128787505	0.023535	3.282982828	0.004572	3.518226452	0.000886	0.218366174	NA	C3	-	Y	Y	Y	Y	Y	-
ENSG00000135835	KIAA1614	-1.078349127	2.08E-28	-0.307137235	0.00712	-0.20934848	0.047072	0.77710556	1.66E-12	0.8838113	1.80E-18	0.100596677	0.591978	C2	-	Y	Y	-	Y	Y	-
ENSG00000135837	CEP350	0.351674009	0.019429	0.338550848	0.002418	0.14459199	0.243358	-0.00713787	0.953788	-0.192759393	0.012498	-0.190835197	0.063565	C1	-	Y	Y	-	-	-	-
ENSG00000135838	NPL	-0.528670967	0.002144	-0.09757086	0.711114	0.023454595	0.927601	0.438499065	0.025481	0.56658429	0.001064	0.123984064	0.702892	C4	-	Y	-	-	Y	Y	-
ENSG00000135862	LAMC1	0.245052374	7.81E-05	0.330727112	0.00011	0.30315346	1.25E-05	0.091917548	0.195191	0.072669572	0.16274	-0.024667268	0.887295	C4	-	-	Y	Y	-	-	-
ENSG00000135900	MRPL44	0.169571806	0.039918	-0.150360895	0.157791	-0.067545282	0.602362	-0.31327572	6.43E-05	-0.222304959	0.009997	0.085928543	0.60749	C5	Y	-	-	-	Y	-	-
ENSG00000135905	DOCK10	-0.26078375	0.019642	-0.017595409	0.931868	0.148542102	0.207557	0.249685507	0.023058	0.42378553	7.74E-07	0.169061207	0.223973	C4	-	-	-	-	-	Y	-
ENSG00000135912	TTLL4	0.579581518	7.43E-17	0.347465426	3.32E-05	0.353476401	5.53E-06	-0.225767798	0.001006	-0.211533632	0.001214	0.0089375	0.963632	C1	-	Y	Y	Y	-	-	-
ENSG00000135913	USP37	0.268078052	0.022517	-0.09182466	0.613859	-0.029360277	0.880079	-0.352729265	1.62E-05	-0.283422453	0.000492	0.064063181	0.721501	C5	-	-	-	-	Y	-	-
ENSG00000135914	HTR2B	-0.859398379	0.034482	-0.037300817	0.953939	-0.078339152	0.886652	0.829225506	0.072431	0.795498237	0.077545	-0.038216263	NA	C2	-	Y	-	-	-	-	-
ENSG00000135932	CAB39	0.593737353	5.33E-32	0.200537928	0.002282	0.203067847	0.001185	-0.386701816	5.55E-15	-0.376050759	7.22E-15	0.005326895	0.974597	C5	-	Y	-	-	Y	Y	-
ENSG00000135953	MFSD9	0.436272959	3.47E-05	0.023061738	0.905874	0.338632101	0.008497	-0.406739115	0.000171	-0.0827267	0.571128	0.318594769	0.037757	C5	-	Y	-	Y	Y	-	Y
ENSG00000135968	GCC2	0.450873533	1.91E-09	0.112021087	0.325187	0.275058984	0.005812	-0.331988417	1.93E-08	-0.160855146	0.029115	0.165925791	0.132864	C5	-	Y	-	-	Y	-	-
ENSG00000135974	C2orf49	0.40281874	3.01E-06	0.014990599	0.922084	0.115149996	0.26606	-0.381493678	2.34E-06	-0.273215598	0.000606	0.103020562	0.426914	C5	Y	Y	-	-	Y	-	-
ENSG00000135976	ANKRD36	0.352088655	0.015366	0.364620585	0.050581	0.456994822	0.005228	0.019622199	0.928135	0.119819265	0.472943	0.094859933	0.768767	C4	-	Y	-	Y	-	-	-
ENSG00000136002	ARHGEF4	-0.457085027	0.000558	-0.261064306	0.152773	-0.270721064	0.127687	0.202937162	0.255901	0.200900785	0.2643	-0.007517047	0.988297	C4	-	Y	-	-	-	-	-
ENSG00000136010	ALDH1L2	-1.253581963	1.53E-29	-1.416685346	1.86E-29	-0.727007205	2.06E-07	-0.156896058	0.422065	0.541127963	0.000605	0.693009987	0.000664	C3	-	Y	Y	Y	-	Y	Y
ENSG00000136014	USP44	0.501253172	0.033318	0.147161258	0.69442	0.41211554	0.143248	-0.347378644	0.174027	-0.073770243	0.818968	0.267982579	0.518183	C1	-	Y	-	-	-	-	-
ENSG00000136021	SCYL2	0.470005233	4.85E-19	0.160336036	0.023335	0.236998814	6.19E-05	-0.303247067	4.10E-10	-0.218435948	1.52E-06	0.07947311	0.337572	C5	-	Y	-	-	Y	-	-
ENSG00000136048	DRAM1	-0.586525227	0.21732	0.342878598	0.513308	0.244198107	0.683215	0.936418118	0.016537	0.846843503	0.058086	-0.094987092	NA	C2	-	-	-	-	Y	-	-
ENSG00000136051	WASHC4	0.420285123	1.31E-11	0.291118164	7.89E-05	0.334440776	5.02E-07	-0.122824501	0.059836	-0.071111685	0.279815	0.046341801	0.705018	C1	-	Y	-	Y	-	-	-
ENSG00000136059	VILL	-0.504821283	0.135365	-0.324096593	0.39141	0.123528855	0.742981	0.187191695	0.677965	0.643047608	0.048117	0.450592026	NA	C4	-	-	-	-	-	Y	-
ENSG00000136098	NEK3	-0.237359737	0.019085	-0.057991219	0.714073	0.091731035	0.47789	0.186421567	0.113735	0.343809689	0.000693	0.152446243	0.358881	C4	-	-	-	-	-	Y	-
ENSG00000136104	RNASEH2B	-0.420538288	3.06E-10	-0.145634273	0.081544	-0.27353572	0.000258	0.281517513	9.55E-05	0.161741814	0.041199	-0.125050721	0.264463	C4	-	Y	-	-	-	-	-
ENSG00000136111	TBC1D4	-0.161767439	0.127898	0.080349223	0.584317	0.131850748	0.273338	0.248375149	0.010299	0.30806304	0.000698	0.054461494	0.7831	C4	-	-	-	-	-	Y	-
ENSG00000136114	THSD1	-1.025464691	0.035619	-0.564380804	0.323267	-0.182066922	0.776838	0.467263347	0.48328	0.858833804	0.130354	0.38560527	NA	C4	-	Y	-	-	-	-	-
ENSG00000136143	SUCLA2	0.355695202	5.79E-07	-0.013050354	0.925945	0.015198321	0.915775	-0.36219495	1.42E-07	-0.325655179	1.31E-05	0.031396234	0.874272	C5	-	Y	-	-	Y	Y	-
ENSG00000136153	LMO7	-0.082555529	0.58698	0.306901412	0.021452	0.182744452	0.223101	0.395759149	0.000436	0.280263915	0.022625	-0.120955339	0.551001	C4	-	-	Y	-	Y	-	-
ENSG00000136160	EDNRB	-3.717067542	0.021545	-0.870137853	NA	-0.510216362	NA	2.8677033	NA	3.221062049	NA	0.356591173	NA	C3	-	Y	-	-	-	-	-
ENSG00000136169	SETDB2	0.349304293	0.001824	0.006979672	0.9771	0.311592073	0.024726	-0.336158745	0.00454	-0.022919169	0.894661	0.307386814	0.082149	C5	-	Y	-	Y	Y	-	-
ENSG00000136205	TNS3	-0.614468529	0.083964	0.05419581	0.914475	-0.16547774	0.706878	0.675186675	0.037485	0.463118844	0.206638	-0.216146724	NA	C2	-	-	-	-	Y	-	-
ENSG00000136206	SPDYE1	-0.637119822	0.045154	0.171891053	0.685777	0.18168443	0.652339	0.81497849	0.00492	0.832931342	0.004976	0.013381997	0.985449	C4	-	Y	-	-	Y	Y	-
ENSG00000136247	ZDHHC4	-0.337855545	5.55E-07	-0.036029928	0.74428	-0.054759369	0.588287	0.308411288	4.91E-07	0.298144504	3.66E-06	-0.015698003	0.927369	C4	-	Y	-	-	Y	-	-
ENSG00000136261	BZW2	-0.423095901	1.38E-11	-0.098141179	0.276574	-0.054649556	0.555439	0.331317086	3.07E-08	0.383210027	4.38E-12	0.046595189	0.701126	C4	-	Y	-	-	Y	Y	-
ENSG00000136273	HUS1	0.37503019	4.88E-05	0.031688428	0.855217	0.146871621	0.202599	-0.337070733	0.000607	-0.213583016	0.030533	0.118037946	0.495893	C5	-	Y	-	-	Y	-	-
ENSG00000136367	ZFHX2	1.372398874	8.53E-22	0.950045658	1.60E-09	1.347100333	4.37E-21	-0.416196798	0.000101	-0.011252892	0.942839	0.39937856	0.001453	C1	-	Y	Y	Y	Y	-	Y
ENSG00000136379	ABHD17C	0.052395801	0.752274	-0.0648351	0.748161	-0.325358427	0.043263	-0.1112659	0.486544	-0.363602722	0.0147	-0.256543298	0.248512	C5	-	-	-	Y	-	Y	-
ENSG00000136383	ALPK3	0.912982987	0.028393	0.394394746	0.547292	0.461278133	0.43582	-0.513633607	0.212163	-0.435147993	0.304101	0.071643421	NA	C1	-	Y	-	-	-	-	-
ENSG00000136425	CIB2	-0.367209112	1.82E-05	-0.143496273	0.296064	-0.20902927	0.037565	0.2300202	0.021787	0.173298109	0.050065	-0.062322861	0.755306	C4	-	Y	-	-	-	-	-
ENSG00000136436	CALCOCO2	0.365203404	9.06E-06	0.170911188	0.122348	0.260490059	0.006019	-0.188083973	0.023811	-0.089944092	0.321759	0.092681759	0.513633	C5	-	Y	-	-	-	-	-
ENSG00000136444	RSAD1	0.353030437	4.09E-08	0.220861075	0.002751	0.209835948	0.007294	-0.125523686	0.04514	-0.128277808	0.054016	-0.008072689	0.962585	C5	-	Y	-	-	-	-	-
ENSG00000136449	MYCBPAP	-0.968687537	0.007095	-0.34023613	0.392098	0.263082397	0.395574	0.640576946	0.165164	1.246582612	0.000245	0.606319704	NA	C2	-	Y	-	-	-	Y	-
ENSG00000136450	SRSF1	0.283728829	7.45E-11	-0.014436155	0.877971	-0.102918848	0.033877	-0.291620335	3.24E-12	-0.372151953	4.01E-31	-0.085825516	0.183658	C5	-	-	-	-	-	Y	-
ENSG00000136478	TEX2	0.385775511	2.22E-09	0.344904734	5.18E-08	0.349230173	3.98E-09	-0.034397254	0.676842	-0.021846074	0.792665	0.007121361	0.962585	C5	-	Y	Y	Y	-	-	-
ENSG00000136531	SCN2A	-0.238421047	0.014314	0.1024934	0.521618	0.223973383	0.021897	0.347174633	0.000712	0.476941538	5.59E-10	0.12502349	0.445982	C4	-	-	-	-	Y	Y	-
ENSG00000136536	MARCHF7	0.304805839	9.55E-09	0.054164627	0.55582	0.082407564	0.286186	-0.244056176	1.64E-07	-0.20757629	7.11E-06	0.031083182	0.791991	C5	-	Y	-	-	-	-	-
ENSG00000136573	BLK	-0.407897612	1.71E-06	0.371764297	0.000144	0.418985841	2.85E-06	0.78629588	8.87E-19	0.841592155	1.18E-23	0.049683629	0.816997	C4	-	Y	Y	Y	Y	Y	-
ENSG00000136574	GATA4	-0.112142314	0.19435	0.229624616	0.010908	0.051502641	0.646606	0.348320379	1.30E-06	0.178649032	0.01416	-0.174919934	0.082976	C4	-	-	-	-	Y	-	-
ENSG00000136628	EPRS1	-0.435446549	3.13E-16	-0.182030108	0.00211	0.034924918	0.641644	0.259948396	3.48E-10	0.484865895	2.58E-40	0.219408019	2.46E-08	C4	-	Y	-	-	-	Y	-
ENSG00000136631	VPS45	0.333858171	6.19E-14	0.103530855	0.130032	0.140539002	0.021853	-0.223879232	6.98E-07	-0.178774509	6.78E-05	0.039843833	0.716838	C5	-	Y	-	-	-	-	-
ENSG00000136720	HS6ST1	-0.153500673	0.063826	-0.296638076	0.000229	-0.327254421	0.000935	-0.136471281	0.077734	-0.159226255	0.08721	-0.027429625	0.904354	C4	-	-	-	Y	-	-	-
ENSG00000136754	ABI1	0.581770608	7.21E-16	0.26352021	0.005628	0.301532475	0.000129	-0.311920999	4.19E-05	-0.265531638	0.000103	0.041055649	0.809775	C1	-	Y	-	Y	Y	-	-
ENSG00000136783	NIPSNAP3A	0.013317742	0.935166	0.058662075	0.749875	0.317628152	0.002496	0.051763913	0.737013	0.319007313	0.001204	0.262267667	0.066193	C4	-	-	-	Y	-	Y	-
ENSG00000136816	TOR1B	0.336745361	1.17E-06	0.147016632	0.121777	0.190824785	0.021949	-0.183341953	0.013591	-0.131216745	0.080682	0.046684429	0.762735	C5	-	Y	-	-	-	-	-
ENSG00000136828	RALGPS1	0.092680769	0.33343	0.302111204	0.000498	0.354340912	3.69E-05	0.215618595	0.004676	0.276304176	0.000294	0.055248533	0.718271	C4	-	-	Y	Y	-	-	-
ENSG00000136840	ST6GALNAC4	-0.406231397	0.003606	-0.030447855	0.905519	-0.012065386	0.959658	0.382363967	0.006189	0.409335966	0.001403	0.021200787	0.952524	C4	-	Y	-	-	Y	Y	-
ENSG00000136866	ZFP37	0.541931047	8.22E-08	-0.161141766	0.310061	0.209727507	0.127899	-0.696600052	9.91E-11	-0.317733158	0.004512	0.37398414	0.016082	C5	-	Y	-	-	Y	Y	Y
ENSG00000136870	ZNF189	0.174012846	0.05739	-0.20217384	0.104432	0.052177432	0.702431	-0.369501645	1.29E-05	-0.10757178	0.216221	0.256951229	0.031665	C5	-	-	-	-	Y	-	-
ENSG00000136895	GARNL3	-0.655812151	8.82E-14	0.214263343	0.028467	0.191910937	0.051232	0.877078681	6.29E-23	0.862466141	2.85E-22	-0.019715502	0.934123	C2	-	Y	-	-	Y	Y	-
ENSG00000136932	TRMO	-0.674300623	4.59E-06	-0.593114927	3.54E-05	-0.740559333	1.90E-06	0.087042035	0.709797	-0.05136727	0.851481	-0.143992793	0.669162	C3	-	Y	Y	Y	-	-	-
ENSG00000136936	XPA	-0.204918991	0.148253	0.065882314	0.747826	-0.374084899	0.006752	0.277926685	0.068171	-0.154659271	0.365654	-0.437669124	0.011968	C2	-	-	-	Y	-	-	Y

ENSG00000136937	NCBP1	0.322571054	7.16E-07	0.142960486	0.093642	0.136108413	0.084133	-0.172917055	0.000345	-0.172196043	0.000142	-0.00457798	0.97463	C5	-	Y	-	-	-	-	-
ENSG00000136938	ANP32B	-0.215655926	8.20E-05	-0.242169721	5.41E-05	-0.346258526	1.49E-09	-0.020079932	0.718452	-0.115865159	0.005275	-0.101074632	0.077504	C4	-	-	-	Y	-	-	-
ENSG00000136943	CTSV	-0.889628645	6.98E-18	-0.315690695	0.006584	-0.368553613	0.000417	0.580285086	1.89E-07	0.535524036	8.40E-07	-0.050022202	0.833466	C4	Y	Y	Y	Y	Y	Y	-
ENSG00000136997	MYC	-0.077133756	0.192707	-0.556998585	1.70E-19	-0.593790679	2.17E-17	-0.473321963	1.35E-17	-0.502157908	1.98E-16	-0.033681213	0.857353	C4	-	-	Y	Y	Y	Y	-
ENSG00000137033	IL33	1.539758626	0.270135	3.213487192	NA	3.143072052	0.085921	1.664650413	0.01932	1.620467025	0.009181	-0.045581035	NA	C2	-	-	-	-	Y	Y	-
ENSG00000137070	IL11RA	-0.353154243	0.000102	-0.080202571	0.574925	-0.142536819	0.228796	0.278898591	0.000117	0.225987429	0.002466	-0.058505113	0.693025	C4	-	Y	-	-	-	-	-
ENSG00000137094	DNAJB5	0.416513034	3.72E-07	0.072493017	0.61268	0.292392112	0.005716	-0.337512265	8.29E-05	-0.10977363	0.29042	0.222851828	0.10078	C1	-	Y	-	-	Y	-	-
ENSG00000137098	SPAG8	-0.500658976	0.280975	-0.057448869	0.935078	0.417197983	0.389531	0.451750226	0.409097	0.932983428	0.038379	0.477149065	NA	C4	-	-	-	-	-	Y	-
ENSG00000137103	TMEM8B	-0.401850353	0.002923	-0.005941037	0.977723	0.00014465	0.999263	0.402230223	0.001547	0.416639532	0.000888	0.009183475	0.97463	C4	-	Y	-	-	Y	Y	-
ENSG00000137124	ALDH1B1	-0.310262543	0.000234	-0.051820308	0.692114	-0.056339718	0.620599	0.264802841	0.002408	0.268726606	0.00115	-0.00179519	0.994002	C4	-	Y	-	-	-	-	-
ENSG00000137133	HINT2	-0.761967877	1.49E-06	-0.331012548	0.061762	-0.573889317	0.000168	0.437766924	0.00909	0.203331647	0.280342	-0.240284417	0.280913	C3	-	Y	-	Y	Y	-	-
ENSG00000137145	DENND4C	0.516318879	1.44E-06	0.314992762	0.016994	0.388929328	0.002873	-0.195375023	0.004458	-0.112817525	0.201794	0.07692562	0.635328	C5	-	Y	Y	Y	-	-	-
ENSG00000137171	KLC4	-0.21731311	0.045845	0.109429073	0.430047	0.178755121	0.144864	0.333196568	0.000859	0.410811234	4.60E-05	0.072149787	0.713445	C4	-	-	-	-	Y	Y	-
ENSG00000137204	SLC22A7	1.481306357	0.108898	0.452911297	NA	-1.030751807	NA	-1.024717237	0.307449	-2.491339633	0.024392	-1.491515787	NA	C5	-	-	-	-	-	Y	-
ENSG00000137210	TMEM14B	-0.384579699	5.12E-06	-0.088059808	0.466767	-0.229427375	0.019959	0.302925265	6.84E-06	0.170525529	0.026168	-0.137773876	0.165312	C4	-	Y	-	-	Y	-	-
ENSG00000137274	BPHL	-0.580727276	9.04E-07	-0.196514591	0.135079	-0.239753352	0.037977	0.391164974	0.000934	0.356134053	0.001775	-0.040268375	0.860457	C4	-	Y	-	-	Y	Y	-
ENSG00000137275	RIPK1	-0.22190818	0.006663	-0.351463866	1.85E-05	-0.323731644	9.69E-05	-0.123237993	0.195519	-0.087167738	0.393404	0.030638423	0.882986	C4	-	-	Y	Y	-	-	-
ENSG00000137338	PGBD1	0.315165477	0.001891	0.035369363	0.868255	0.121760283	0.413237	-0.272560111	0.013858	-0.178856837	0.09951	0.089246242	0.703663	C5	-	Y	-	-	-	-	-
ENSG00000137404	NRM	-0.506911431	4.52E-08	-0.195653581	0.084831	-0.138899517	0.218897	0.317999253	0.002195	0.382728578	8.14E-05	0.059663348	0.771446	C4	-	Y	-	-	Y	Y	-
ENSG00000137413	TAF8	0.382740848	7.85E-06	0.018990984	0.909425	0.031052451	0.833196	-0.357365524	4.68E-05	-0.336975371	0.000104	0.014862704	0.952948	C5	-	Y	-	-	Y	Y	-
ENSG00000137414	FAM8A1	0.539502776	3.41E-14	0.248994302	0.005559	0.290772095	0.000136	-0.28423345	5.37E-06	-0.234523093	2.11E-05	0.044574966	0.729425	C5	-	Y	-	-	-	-	-
ENSG00000137494	ANKRD42	0.711758969	5.98E-12	0.314414876	0.03145	0.404298921	0.003025	-0.390784112	0.000537	-0.292621025	0.013128	0.092845331	0.723061	C1	Y	Y	Y	Y	Y	-	-
ENSG00000137502	RAB30	0.357515596	0.000351	0.469575897	1.95E-05	0.433605447	0.000221	0.117735721	0.202673	0.090914786	0.399195	-0.032869477	0.892177	C1	-	Y	Y	Y	-	-	-
ENSG00000137507	LRRC32	-0.91182229	0.014835	-0.793274004	0.067499	-0.66924449	0.094957	0.127659189	0.843262	0.257738623	0.647495	0.126264089	NA	C3	-	Y	-	-	-	-	-
ENSG00000137522	RNF121	0.303094809	0.000633	0.157085772	0.147708	0.001830847	0.990999	-0.139598448	0.122264	-0.286762882	0.00035	-0.152348498	0.186525	C5	-	Y	-	-	-	-	-
ENSG00000137558	PI15	0.542200609	0.002085	0.19535032	0.489035	0.159485408	0.549054	-0.341023407	0.054938	-0.367875422	0.029531	-0.03259231	0.947133	C1	-	Y	-	-	-	Y	-
ENSG00000137601	NEK1	-0.366457398	4.53E-07	-0.218013124	0.011642	-0.200586221	0.010536	0.154725992	0.029878	0.180379927	0.005415	0.020206412	0.90173	C4	-	Y	-	-	-	-	-
ENSG00000137628	DDX60	-1.761715218	8.12E-05	-0.663861275	0.156818	-0.331300094	0.476489	1.110337598	0.072915	1.44172098	0.007285	0.329724645	NA	C3	-	Y	-	-	-	Y	-
ENSG00000137642	SORL1	-0.636356348	5.58E-09	-0.195482256	0.280865	0.087010692	0.638903	0.447003081	0.000445	0.738254449	1.13E-11	0.285035083	0.151978	C4	-	Y	-	-	Y	Y	-
ENSG00000137713	PPP2R1B	0.334373494	0.000685	0.133580173	0.32227	0.129804942	0.270982	-0.194328616	0.04959	-0.190213187	0.035247	-0.001037733	0.997161	C5	-	Y	-	-	-	-	-
ENSG00000137801	THBS1	0.687189953	0.008259	0.931015589	0.000333	0.647233213	0.026533	0.249975076	0.314478	-0.025254073	0.940379	-0.280560413	0.433031	C1	-	Y	Y	Y	-	-	-
ENSG00000137804	NUSAP1	-0.559474833	0.004708	-0.210504515	0.355875	-0.392518996	0.053977	0.35583768	0.123148	0.181377176	0.505308	-0.179439682	0.610325	C3	-	Y	-	-	-	-	-
ENSG00000137812	KNL1	0.425639481	8.31E-07	0.331855851	0.002013	0.311234304	0.000921	-0.087823553	0.2886	-0.099780837	0.125419	-0.017339594	0.928882	C1	-	Y	Y	Y	-	-	-
ENSG00000137814	HAUS2	0.180818111	0.029096	-0.138857998	0.262242	-0.142752854	0.167743	-0.313388528	0.00045	-0.308822173	0.000123	-0.000906675	0.997282	C5	-	-	-	-	Y	Y	-
ENSG00000137822	TUBGCP4	0.709615873	2.39E-22	0.231180322	0.017532	0.268722631	0.003686	-0.472003447	3.71E-16	-0.426886368	4.96E-13	0.039953992	0.795685	C5	-	Y	-	-	Y	Y	-
ENSG00000137824	RMDN3	0.310567813	0.001191	-0.069830386	0.692114	0.041248555	0.810812	-0.37313088	8.79E-05	-0.254723781	0.008211	0.113831238	0.559116	C5	-	Y	-	-	Y	-	-
ENSG00000137831	UACA	-0.37377101	0.001554	0.064272859	0.710865	0.045330307	0.810228	0.444171964	4.93E-05	0.433749504	0.00028	-0.015953347	0.958938	C4	-	Y	-	-	Y	Y	-
ENSG00000137834	SMAD6	-0.049191024	0.524454	0.167439112	0.045506	-0.137262103	0.096868	0.223083995	0.001047	-0.073552102	0.344787	-0.301955082	0.00049	C4	-	-	-	-	-	-	Y
ENSG00000137841	PLCB2	-1.513492523	9.78E-06	-0.707756677	0.063258	-0.820166345	0.007859	0.823452684	0.099979	0.707668539	0.133601	-0.116361965	NA	C3	-	Y	-	Y	-	-	-
ENSG00000137843	PAK6	-1.359514593	0.001268	-0.686023105	0.089439	-0.668653325	0.091764	0.677799258	0.204537	0.70412153	0.187148	0.020344549	NA	C3	-	Y	-	-	-	-	-
ENSG00000137872	SEMA6D	-0.57260128	4.19E-06	-0.204094651	0.166285	-0.143468676	0.301057	0.374466075	0.004952	0.444029717	0.000252	0.062943963	0.793949	C4	Y	Y	-	-	Y	Y	-
ENSG00000137936	BCAR3	-0.56420977	0.000785	-0.219067205	0.299175	0.034306443	0.891237	0.352613521	0.069576	0.613293472	0.000241	0.256458234	0.28885	C4	-	Y	-	-	-	Y	-
ENSG00000137941	TTLL7	0.460268358	6.05E-08	0.103072006	0.453711	0.450178466	6.74E-07	-0.351755304	1.64E-07	0.004387354	0.963649	0.350608147	8.49E-06	C4	-	Y	-	Y	Y	-	Y
ENSG00000137968	SLC44A5	-0.139832282	0.058301	0.18958763	0.026788	0.153385056	0.106569	0.335586013	4.42E-07	0.308022808	2.48E-05	-0.033602007	0.860105	C4	-	-	-	-	Y	Y	-
ENSG00000137992	DBT	0.509450834	1.95E-07	0.130390996	0.409995	0.364960418	0.000228	-0.372745759	0.000142	-0.130218442	0.148749	0.237277145	0.066506	C5	-	Y	-	Y	Y	-	-
ENSG00000138018	SELENOI	0.75917015	1.84E-35	0.339073951	9.87E-06	0.363326265	5.96E-07	-0.413524478	6.80E-19	-0.381319376	4.98E-17	0.02692883	0.845294	C5	-	Y	Y	Y	Y	Y	-
ENSG00000138028	CGREF1	-0.395413197	5.52E-06	-0.184019489	0.180612	-0.016960417	0.912995	0.217716257	0.069884	0.393175161	2.63E-05	0.169816466	0.341115	C4	-	Y	-	-	-	Y	-
ENSG00000138030	KHK	-0.173084541	0.305337	0.038067391	0.884578	0.249783088	0.139933	0.217507295	0.131114	0.43827285	0.000429	0.21533381	0.244738	C4	-	-	-	-	-	Y	-
ENSG00000138031	ADCY3	0.350296674	4.22E-06	0.072832678	0.51712	0.051295664	0.665549	-0.270938216	0.000486	-0.284290179	0.000328	-0.018743613	0.931318	C5	-	Y	-	-	-	-	-
ENSG00000138061	CYP1B1	-0.994033583	3.87E-05	-0.423902527	0.147923	-0.203769713	0.533493	0.575346556	0.050517	0.805151102	0.003766	0.223871696	0.647462	C4	-	Y	-	-	-	Y	-
ENSG00000138073	PREB	0.47670523	7.05E-14	0.127748197	0.20083	0.327514316	2.84E-05	-0.342294235	2.22E-07	-0.13457141	0.059794	0.202662426	0.046498	C5	-	Y	-	Y	Y	-	-
ENSG00000138085	ATRAID	-0.432441605	6.37E-11	-0.106377567	0.297532	-0.186317518	0.028833	0.332277007	5.60E-08	0.261025266	1.40E-05	-0.076724833	0.5353	C4	-	Y	-	-	Y	-	-
ENSG00000138131	LOXL4	-0.948652019	0.010982	-0.239229929	0.633362	-0.398419282	0.356709	0.718273571	0.096423	0.565489442	0.217644	-0.157315308	NA	C3	-	Y	-	-	-	-	-
ENSG00000138134	STAMBPL1	0.693026547	5.01E-10	0.306386857	0.0587	0.466002737	0.000224	-0.379730998	0.0013	-0.212529102	0.057603	0.162072582	0.425871	C1	-	Y	-	Y	Y	-	-
ENSG00000138138	ATAD1	0.339723391	7.96E-12	0.135919418	0.015845	0.084259783	0.156272	-0.197402095	1.38E-05	-0.240801099	2.60E-08	-0.048741632	0.535304	C5	-	Y	-	-	-	-	-
ENSG00000138162	TACC2	-0.319147279	0.013994	0.193912433	0.190504	0.156264747	0.302966	0.519623453	2.98E-06	0.490218466	1.84E-05	-0.034913364	0.897588	C4	-	Y	-	-	Y	Y	-
ENSG00000138166	DUSP5	1.41819165	1.55E-06	0.967502092	0.006421	0.511220212	0.241194	-0.444100853	0.112807	-0.893422232	0.00141	-0.454546127	NA	C5	-	Y	Y	-	-	Y	-
ENSG00000138185	ENTPD1	0.342134321	0.000208	0.088886466	0.598346	0.115648883	0.352744	-0.246202011	0.017583	-0.211643373	0.017051	0.02913736	0.912643	C5	-	Y	-	-	-	-	-
ENSG00000138231	DBR1	-0.107976111	0.197222	-0.251423772	0.002723	-0.331645189	0.000122	-0.137057226	0.102617	-0.208945868	0.01404	-0.077127684	0.62517	C4	-	-	-	Y	-	-	-
ENSG00000138246	DNAJC13	0.474738421	4.12E-12	0.271427172	0.003019	0.345087253	1.79E-05	-0.196383105	0.001964	-0.114839867	0.071728	0.076238974	0.551714	C1	-	Y	-	Y	-	-	-
ENSG00000138316	ADAMTS14	-1.152773534	4.81E-24	-0.180853698	0.265753	0.011297609	0.948703	0.979498033	3.70E-12	1.178856559	1.44E-24	0.19511523	0.323631	C3	Y	Y	-	-	Y	Y	-
ENSG00000138336	TET1	0.358900072	0.000574	0.106816269	0.531804	0.192188551	0.15123	-0.246455689	0.014302	-0.15225227	0.139389	0.088643278	0.655216	C5	-	Y	-	-	-	-	-
ENSG00000138375	SMARCAL1	0.21285289	0.028081	0.276204103	0.006277	0.395040793	8.11E-06	0.069973942	0.555981	0.196832683	0.036937	0.121883192	0.393067	C4	-	-	-	Y	-	-	-
ENSG00000138380	CARF	-0.666992771	4.81E-09	-0.070749928	0.675196	0.011458865	0.955402	0.603101346	7.31E-08	0.693092637	1.68E-09	0.084915231	0.698304	C2	-	Y	-	-	Y	Y	-
ENSG00000138398	PPIG	0.173254919	0.008686	-0.144223771	0.068554	-0.058553386	0.490157	-0.310852753	9.99E-11	-0.217433043	9.67E-07	0.0880008	0.221664	C5	-	-	-	-	Y	-	-
ENSG00000138399	FASTKD1	0.317830346	0.000106	0.136634607	0.280174	0.080751023	0.492162	-0.174641202	0.078611	-0.222243947	0.008965	-0.053157905	0.795685	C5	-	Y	-	-	-	-	-
ENSG00000138413	IDH1	-0.265286666	0.000293	-0.018997735	0.901469	0.145635326	0.081755	0.252928871	0.001038	0.425566937	1.05E-12	0.167161072	0.118985	C4	-	-	-	-	-	Y	-

ENSG00000138434	ITPRID2	0.652843759	4.85E-19	0.265587236	0.004487	0.283860593	0.002508	-0.380821666	1.03E-10	-0.354931167	1.18E-08	0.020878398	0.912409	C5	-	Y	-	-	Y	Y	-
ENSG00000138448	ITGAV	0.335286462	9.42E-07	0.350852492	4.78E-06	0.182999527	0.027891	0.021591625	0.785556	-0.137630543	0.018754	-0.164725147	0.040788	C4	-	Y	Y	-	-	-	-
ENSG00000138463	SLC49A4	0.504866947	0.001665	0.060784353	0.824312	0.161380074	0.476113	-0.438007271	0.007041	-0.329325329	0.058101	0.103349974	0.767513	C5	-	Y	-	-	Y	-	-
ENSG00000138495	COX17	-0.342276517	0.012068	-0.348062634	0.027865	-0.383724866	0.029732	0.000755336	0.996345	-0.026383966	0.903695	-0.032601281	0.936825	C4	-	Y	Y	Y	-	-	-
ENSG00000138496	PARP9	-1.242325047	7.60E-08	-0.702912837	0.002971	-0.441458364	0.065637	0.547233848	0.072077	0.815559644	0.002875	0.263887778	0.53984	C3	-	Y	Y	-	-	Y	-
ENSG00000138592	USP8	0.37395374	2.17E-06	0.130666184	0.246706	0.08722485	0.453822	-0.236710195	9.43E-05	-0.272021668	6.80E-06	-0.040785235	0.78381	C5	-	Y	-	-	-	-	-
ENSG00000138593	SECISBP2L	0.439546741	1.20E-13	0.252068943	0.000572	0.280818604	5.48E-05	-0.181475193	0.000877	-0.144007123	0.008841	0.031923833	0.805857	C5	-	Y	-	-	-	-	-
ENSG00000138594	TMOD3	0.390924659	7.27E-11	0.185983615	0.045902	0.219655313	0.001082	-0.198433488	0.00572	-0.15662104	0.004904	0.03659293	0.821705	C5	-	Y	-	-	-	-	-
ENSG00000138604	GLCE	0.382596934	0.000928	0.100020846	0.635256	0.297882752	0.023903	-0.276599467	0.031063	-0.070039538	0.607024	0.201121436	0.295679	C1	-	Y	-	-	-	-	-
ENSG00000138606	SHF	-0.537394634	1.14E-08	-0.373462142	0.000387	-0.258360567	0.0152	0.171035332	0.074604	0.294062682	0.000601	0.117620188	0.394811	C4	-	Y	Y	-	-	-	-
ENSG00000138613	APH1B	-1.001746692	3.06E-16	-0.396569235	0.023769	-0.272176935	0.072276	0.612561915	0.000258	0.744147683	1.75E-07	0.126117229	0.703663	C4	-	Y	Y	-	Y	Y	-
ENSG00000138617	PARP16	-0.108195815	0.529975	-0.321699796	0.056246	-0.32907535	0.038741	-0.206612624	0.236429	-0.206193646	0.227573	-0.004871861	0.991638	C4	-	-	-	Y	-	-	-
ENSG00000138621	PPCDC	-1.075645636	1.43E-09	-0.331673392	0.113316	-0.380402799	0.051234	0.75219378	0.000345	0.709941117	0.000768	-0.046648558	0.928789	C3	-	Y	-	-	Y	Y	-
ENSG00000138623	SEMA7A	0.624299407	0.00267	0.045968516	0.91022	0.066128534	0.858919	-0.571745792	0.008017	-0.543595712	0.012175	0.022936015	0.971963	C5	-	Y	-	-	Y	Y	-
ENSG00000138629	UBL7	-0.326383379	4.49E-09	-0.063455731	0.465869	-0.067611052	0.368492	0.269496317	5.00E-07	0.273641365	4.59E-08	-0.001363577	0.993273	C4	-	Y	-	-	-	-	-
ENSG00000138640	FAM13A	0.445418149	0.059924	0.515348359	0.045567	0.394937057	0.126633	0.077022232	0.805784	-0.035593828	0.914135	-0.11758183	0.804185	C1	-	-	Y	-	-	-	-
ENSG00000138642	HERC6	-1.109353139	5.67E-06	-0.907752506	8.98E-05	-0.413180013	0.108493	0.206930352	0.603318	0.710349308	0.02196	0.497699683	0.207156	C3	-	Y	Y	-	-	Y	-
ENSG00000138646	HERC5	-0.914709229	1.26E-06	-0.734837255	0.000596	-0.495721342	0.022789	0.185336867	0.527258	0.43328281	0.077191	0.242255358	0.553292	C3	-	Y	Y	Y	-	-	-
ENSG00000138675	FGF5	0.792152019	0.033424	1.283041216	0.000732	0.81759908	0.058548	0.496335888	0.156511	0.039358419	0.937583	-0.462461849	NA	C1	-	Y	Y	-	-	-	-
ENSG00000138685	FGF2	0.375070354	0.001069	0.095543048	0.564364	0.109756426	0.462614	-0.273165557	0.004598	-0.250838183	0.008938	0.016687792	0.944704	C5	-	Y	-	-	-	-	-
ENSG00000138688	KIAA1109	0.531576136	0.001758	0.362436979	0.001861	0.468231131	1.14E-06	-0.162322733	0.376186	-0.049159839	0.806614	0.109038624	0.42836	C1	-	Y	Y	Y	-	-	-
ENSG00000138696	BMPR1B	-0.725387952	0.007348	-0.630556356	0.07902	-0.927259126	0.000288	0.096474048	0.848923	-0.187417916	0.641745	-0.290955549	NA	C3	-	Y	-	Y	-	-	-
ENSG00000138698	RAP1GDS1	0.303078655	3.00E-07	0.206309575	0.003669	0.119777389	0.122767	-0.090317408	0.188966	-0.168451082	0.007189	-0.083550482	0.431008	C5	-	Y	-	-	-	-	-
ENSG00000138709	LARP1B	0.34083541	0.000355	0.05732147	0.713114	0.024751055	0.882257	-0.277116948	0.003834	-0.301629956	0.001729	-0.029777265	0.904354	C5	-	Y	-	-	-	Y	-
ENSG00000138750	NUP54	-0.263775326	3.20E-05	-0.330226003	5.76E-06	-0.303446455	5.80E-06	-0.059990936	0.501614	-0.024994877	0.790306	0.029745444	0.860105	C4	-	-	Y	Y	-	-	-
ENSG00000138759	FRAS1	-0.565653985	0.00054	-0.069110835	0.800337	-0.045303233	0.864256	0.502695376	0.002387	0.534788247	0.001129	0.026773276	0.951422	C2	-	Y	-	-	Y	Y	-
ENSG00000138767	CNOT6L	0.37083628	0.000603	0.062056497	0.727395	0.148907515	0.255763	-0.302547928	0.001442	-0.207508908	0.024476	0.089648451	0.594474	C5	-	Y	-	-	Y	-	-
ENSG00000138768	USO1	0.294863616	2.83E-06	0.192829463	0.004549	0.329100563	3.09E-07	-0.095773958	0.113661	0.048896924	0.479566	0.13929503	0.053627	C4	-	-	-	Y	-	-	-
ENSG00000138769	CDKL2	-0.513887904	0.062812	0.172793576	0.635496	0.426670212	0.106703	0.693164761	0.012211	0.95538006	0.000147	0.256802165	0.524775	C4	-	-	-	-	Y	Y	-
ENSG00000138778	CENPE	0.246127583	0.020525	0.230929876	0.09538	0.304841755	0.006746	-0.009547592	0.932862	0.073097617	0.276494	0.077244064	0.59034	C4	-	-	-	Y	-	-	-
ENSG00000138780	GSTCD	0.239985731	0.006802	0.301651324	0.000921	0.337316798	0.000195	0.068137363	0.518466	0.112076375	0.258037	0.038599991	0.840067	C4	-	-	Y	Y	-	-	-
ENSG00000138792	ENPEP	-1.403204849	0.000645	-1.078863531	0.014074	-0.205562055	0.666382	0.327553144	0.6286	1.212270521	0.005855	0.875925264	NA	C3	-	Y	Y	-	-	Y	-
ENSG00000138794	CASP6	-0.737395278	4.71E-06	-0.186024246	0.385874	-0.338458825	0.043182	0.558470544	0.002649	0.413359153	0.025879	-0.149786491	0.62517	C3	-	Y	-	Y	Y	Y	-
ENSG00000138795	LEF1	-0.210269761	0.071418	0.212667864	0.117441	0.068352068	0.704639	0.429483738	6.55E-05	0.293467805	0.01651	-0.141758889	0.500085	C4	-	-	-	-	Y	-	-
ENSG00000138802	SEC24B	0.568431414	5.21E-13	0.241543719	0.016645	0.29550719	0.001703	-0.320053205	1.73E-09	-0.25906051	2.63E-06	0.05600862	0.642574	C5	-	Y	-	-	Y	-	-
ENSG00000138821	SLC39A8	-0.300710443	0.053544	-0.083459828	0.739578	0.02719084	0.916162	0.224448439	0.24573	0.342581684	0.046758	0.112797906	0.748588	C4	-	-	-	-	-	Y	-
ENSG00000138823	MTTP	-2.824472617	0.0033	-1.747940348	NA	-0.288093257	0.760775	1.103306839	NA	2.552158255	0.018049	1.45961101	NA	C3	-	Y	-	-	-	Y	-
ENSG00000138834	MAPK8IP3	0.383910793	1.06E-15	0.146088185	0.054368	0.152851264	0.003702	-0.231205017	2.01E-05	-0.216399565	1.39E-07	0.009267938	0.952524	C5	-	Y	-	-	-	-	-
ENSG00000138835	RGS3	-0.269575467	0.177193	0.055567533	0.862544	0.152130976	0.553075	0.33213708	0.133073	0.436639115	0.037719	0.099429638	0.827579	C4	-	-	-	-	-	Y	-
ENSG00000139131	YARS2	0.430134661	0.000542	-0.003605381	0.990217	0.101225931	0.599274	-0.427353093	0.001498	-0.314166486	0.017167	0.107759477	0.701775	C5	-	Y	-	-	Y	Y	-
ENSG00000139132	FGD4	0.829795156	4.92E-13	0.395894127	0.016765	0.55893185	2.72E-05	-0.427120531	0.000197	-0.256263489	0.01626	0.165712936	0.423104	C1	-	Y	Y	Y	Y	-	-
ENSG00000139133	ALG10	0.392298092	0.026639	0.105473732	0.694574	0.201725824	0.365139	-0.280829749	0.116492	-0.175897933	0.362696	0.099552672	0.773338	C5	-	Y	-	-	-	-	-
ENSG00000139163	ETNK1	0.439202987	1.30E-14	0.046862848	0.699483	0.26452525	3.01E-05	-0.385573733	1.07E-09	-0.159777747	0.002748	0.220393583	0.008951	C5	-	Y	-	-	Y	-	-
ENSG00000139173	TMEM117	-0.406348158	0.006045	-0.173029967	0.439631	-0.141838243	0.472974	0.240029758	0.178461	0.279094435	0.076876	0.0341438	0.936471	C4	-	Y	-	-	-	-	-
ENSG00000139174	PRICKLE1	-0.255451774	0.034682	-0.40536007	0.003669	-0.29932714	0.038949	-0.143454677	0.289058	-0.029306433	0.864382	0.108928741	0.655393	C4	-	-	Y	-	-	-	-
ENSG00000139178	C1RL	-1.460366485	9.85E-14	-0.448645702	0.027586	-0.156138653	0.475043	1.018666971	7.05E-06	1.318937873	7.74E-11	0.29550032	0.279938	C3	-	Y	Y	-	Y	Y	-
ENSG00000139190	VAMP1	-0.171112916	0.264526	0.154456575	0.391125	0.076287363	0.698079	0.33196537	0.014802	0.262174305	0.067592	-0.075272661	0.78276	C4	-	-	-	-	Y	-	-
ENSG00000139192	TAPBPL	-1.25925295	0.01018	-0.415997032	0.506535	-0.367805361	0.532216	0.854125335	0.147092	0.907557837	0.112948	0.050626166	NA	C3	-	Y	-	-	-	-	-
ENSG00000139266	MARCHF9	0.261749798	0.01797	-0.125094952	0.413014	0.082300575	0.603298	-0.379750211	9.40E-06	-0.164273474	0.085489	0.210077118	0.087823	C5	-	-	-	-	Y	-	-
ENSG00000139278	GLIPR1	0.64844773	0.189489	1.162120068	0.011438	0.939533679	0.037917	0.520770667	0.170587	0.303762807	0.458029	-0.224593933	NA	C1	-	-	Y	Y	-	-	-
ENSG00000139289	PHLDA1	1.823726897	8.30E-07	1.255932375	0.002124	1.28808038	0.001923	-0.56161367	0.09465	-0.52059572	0.140924	0.035225299	NA	C1	-	Y	Y	Y	-	-	-
ENSG00000139292	LGR5	0.672994434	5.28E-51	0.086613913	0.341198	-0.067004765	0.273119	-0.579764514	9.49E-31	-0.725445478	2.77E-106	-0.151003005	0.053573	C5	-	Y	-	-	Y	Y	-
ENSG00000139324	TMTC3	0.491502004	9.09E-13	0.09084824	0.418923	0.220739498	0.011179	-0.394052226	2.72E-09	-0.255928619	0.00015	0.132697035	0.24742	C5	-	Y	-	-	Y	-	-
ENSG00000139329	LUM	-0.605638382	0.050492	-0.472128673	0.222142	-1.13927886	0.000621	0.142771129	0.766385	-0.518996652	0.215599	-0.668471098	NA	C3	-	-	-	Y	-	-	-
ENSG00000139344	AMDHD1	-1.05820761	0.015941	-0.609419766	0.298628	-0.278416199	0.624066	0.459050819	0.470487	0.794714104	0.119236	0.332151357	NA	C3	-	Y	-	-	-	-	-
ENSG00000139350	NEDD1	0.301082574	3.72E-05	0.030186509	0.821415	0.221468114	0.006874	-0.264304794	4.01E-05	-0.065079242	0.383643	0.1938564	0.019202	C5	-	Y	-	-	-	-	-
ENSG00000139352	ASCL1	-0.511101556	1.57E-18	-0.919507523	2.55E-23	-0.729823764	6.18E-17	-0.402225961	2.40E-05	-0.204305241	0.032132	0.194687876	0.51979	C5	-	Y	Y	Y	Y	-	-
ENSG00000139410	SDSL	-1.079499065	0.011896	-0.588600094	0.271422	-0.203013119	0.705378	0.498781713	0.362166	0.89257998	0.038254	0.38965409	NA	C4	-	Y	-	-	-	Y	-
ENSG00000139428	MMAB	-0.299963796	0.004396	0.030482033	0.861146	0.003597859	0.984469	0.337138224	0.000369	0.318265348	0.001081	-0.024418525	0.920668	C4	-	-	-	-	Y	Y	-
ENSG00000139436	GIT2	0.575942313	4.62E-08	0.32186525	0.009301	0.299402	0.011374	-0.247597246	0.00254	-0.262225299	0.001019	-0.020239961	0.925998	C1	-	Y	Y	-	-	-	-
ENSG00000139437	TCHP	0.314544768	5.30E-05	0.180331432	0.10712	0.164649794	0.08316	-0.127486096	0.174341	-0.135326075	0.097749	-0.013031056	0.957248	C5	-	Y	-	-	-	-	-
ENSG00000139438	FAM222A	0.144784231	0.041277	0.375034318	5.78E-08	0.300128981	5.44E-06	0.236488071	7.15E-07	0.169571718	9.74E-05	-0.072069884	0.366991	C4	-	-	Y	Y	-	-	-
ENSG00000139445	FOXN4	-0.535947532	1.33E-06	-0.12079298	0.438477	-0.083884487	0.584763	0.421664534	0.000289	0.466841058	3.33E-05	0.039999796	0.881786	C4	-	Y	-	-	Y	Y	-
ENSG00000139496	NUP58	0.445389494	4.55E-15	0.108675361	0.197402	0.145207478	0.045803	-0.329949439	1.83E-13	-0.285761569	3.09E-11	0.039013287	0.714139	C5	-	Y	-	-	Y	-	-
ENSG00000139505	MTMR6	0.45281073	1.31E-12	0.215318861	0.01921	0.091497169	0.356249	-0.230876236	0.000304	-0.346824062	5.40E-10	-0.121013969	0.282846	C5	Y	Y	-	-	-	Y	-
ENSG00000139514	SLC7A1	-0.356264461	2.00E-16	-0.211559837	0.000328	-0.10929069	0.091381	0.151269164	0.001586	0.261352288	6.75E-09	0.105152736	0.218342	C4	-	Y	-	-	-	-	-
ENSG00000139517	LNX2	0.604970676	4.95E-08	0.481442709	0.001122	0.394964455	0.002166	-0.117951956	0.407636	-0.195407425	0.084011	-0.082944481	0.739147	C1	-	Y	Y	Y	-	-	-

ENSG00000139537	CCDC65	0.415712774	0.013522	0.283364355	0.17758	0.162485855	0.465835	-0.126152521	0.539963	-0.238761414	0.188707	-0.117970715	0.714581	C1	-	Y	-	-	-	-	-
ENSG00000139567	ACVRL1	-1.18920733	6.95E-13	-0.675268497	0.000418	-0.352899589	0.05159	0.52130741	0.017329	0.851314128	3.62E-06	0.32537969	0.252322	C3	-	Y	Y	-	Y	Y	-
ENSG00000139597	N4BP2L1	0.519697145	1.14E-06	0.312828252	0.032638	0.429058106	0.000956	-0.200485413	0.119113	-0.076135005	0.605032	0.119234425	0.617535	C5	-	Y	Y	Y	-	-	-
ENSG00000139620	KANSL2	-0.154442563	0.061263	-0.267470749	0.009021	-0.30024103	0.000374	-0.106331232	0.323022	-0.130850273	0.149841	-0.029926395	0.899704	C4	-	-	-	Y	-	-	-
ENSG00000139629	GALNT6	-1.327958778	8.17E-13	-0.168375911	0.480699	-0.264490581	0.183908	1.165811914	4.22E-10	1.078488802	6.71E-09	-0.093469955	0.787224	C2	-	Y	-	-	Y	Y	-
ENSG00000139636	LMBR1L	0.410591198	1.30E-09	0.034398112	0.792952	0.1577616	0.102856	-0.369492705	1.11E-06	-0.237966607	0.003483	0.125513721	0.395152	C5	-	Y	-	-	Y	-	-
ENSG00000139645	ANKRD52	0.457971161	4.07E-17	0.334483963	3.73E-08	0.328946122	1.97E-08	-0.117273299	0.009155	-0.114495585	0.008002	-0.002563915	0.982584	C1	-	Y	Y	Y	-	-	-
ENSG00000139668	WDFY2	0.242582048	0.003698	0.370821311	2.43E-05	0.235462775	0.00797	0.134689911	0.099267	0.007514205	0.944369	-0.132540211	0.218201	C4	-	-	Y	-	-	-	-
ENSG00000139675	HNRNPA1L2	-0.10486886	0.44652	-0.237076392	0.094823	-0.4943208	0.000215	-0.125772184	0.378135	-0.374904958	0.004368	-0.254420089	0.18621	C5	-	-	-	Y	-	Y	-
ENSG00000139697	SBNO1	0.456955746	5.06E-09	0.14748785	0.207761	0.180404873	0.052517	-0.303096669	2.01E-05	-0.261974144	1.14E-05	0.035823305	0.83368	C5	-	Y	-	-	Y	-	-
ENSG00000139714	MORN3	-0.59414058	0.008042	-0.287596555	0.263511	-0.114167814	0.686622	0.314715209	0.235643	0.495324858	0.033945	0.1766577	0.63838	C2	-	Y	-	-	-	Y	-
ENSG00000139767	SRRM4	0.443289004	5.40E-06	0.3490808	0.003068	0.480911066	2.62E-06	-0.0872059	0.278864	0.052073208	0.508823	0.133892674	0.212738	C1	-	Y	Y	Y	-	-	-
ENSG00000139793	MBNL2	0.565118641	2.17E-08	0.187213343	0.224378	0.449814133	4.80E-05	-0.372356785	0.000148	-0.100693351	0.339317	0.266420185	0.041641	C1	-	Y	-	Y	Y	-	-
ENSG00000139826	ABHD13	0.449421115	6.20E-09	0.189820007	0.05915	0.174662501	0.080989	-0.253259885	0.000549	-0.259981932	0.000482	-0.012217768	0.95405	C1	-	Y	-	-	-	-	-
ENSG00000139835	GRTP1	-0.496617053	0.020638	-0.076782402	0.790519	0.050435552	0.847464	0.426403559	0.045517	0.562077354	0.003601	0.129799966	0.667135	C4	-	Y	-	-	Y	Y	-
ENSG00000139921	TMX1	0.312378396	5.81E-07	-0.046609306	0.695049	0.084555434	0.328794	-0.352273808	4.37E-08	-0.213095432	0.000438	0.133678554	0.188673	C5	-	Y	-	-	Y	-	-
ENSG00000139973	SYT16	0.00721394	0.970241	-0.460841066	0.006909	-0.017165355	0.935898	-0.461352223	0.000867	-0.009798448	0.951374	0.446188997	0.010286	C5	-	-	Y	-	Y	-	Y
ENSG00000139977	NAA30	0.509346171	4.22E-08	0.034659009	0.863999	0.325439151	0.000476	-0.468081688	7.00E-06	-0.169429884	0.061322	0.293275505	0.025267	C5	-	Y	-	Y	Y	-	-
ENSG00000139990	DCAF5	0.365498252	1.31E-08	0.192235758	0.011915	0.186820777	0.0102	-0.16707173	0.004299	-0.163927714	0.003639	-0.002118399	0.990166	C5	-	Y	-	-	-	-	-
ENSG00000140006	WDR89	0.193296197	0.039425	-0.209812421	0.038808	0.014694791	0.924182	-0.396593747	1.04E-05	-0.163898509	0.10811	0.22736529	0.069496	C5	-	-	-	-	Y	-	-
ENSG00000140025	EFCAB11	-0.469534883	0.003395	-0.255092145	0.191261	-0.211737239	0.247224	0.221437938	0.252276	0.272583796	0.124048	0.046646107	0.904354	C4	-	Y	-	-	-	-	-
ENSG00000140043	PTGR2	-0.452882538	6.45E-05	-0.103277093	0.554711	0.011656414	0.952438	0.356110842	0.004326	0.479369379	2.14E-05	0.117917237	0.57455	C4	-	Y	-	-	Y	Y	-
ENSG00000140044	JDP2	-1.507760155	4.88E-17	-0.614053615	0.000289	-0.468293498	0.006045	0.897931476	3.25E-05	1.053825031	4.68E-07	0.149295786	0.664301	C2	-	Y	Y	Y	Y	Y	-
ENSG00000140057	AK7	-0.958045093	1.32E-05	-0.257077694	0.351957	-0.392143614	0.100251	0.708133455	0.002299	0.581041177	0.01538	-0.131812164	0.755306	C2	-	Y	-	-	Y	Y	-
ENSG00000140104	CLBA1	-0.159695527	0.262546	0.077116639	0.691497	0.170403422	0.232753	0.243676098	0.092583	0.34485631	0.007081	0.096235505	0.686506	C4	-	-	-	-	-	Y	-
ENSG00000140105	WARS1	-0.327078069	5.95E-09	-0.293369498	6.00E-06	-0.21043106	0.017015	0.04003525	0.631848	0.131144695	0.116671	0.086426439	0.565651	C4	-	Y	-	-	-	-	-
ENSG00000140199	SLC12A6	0.012517163	0.930145	0.280656017	0.002922	0.307340935	0.000205	0.274449197	0.00112	0.309217496	5.07E-05	0.029939201	0.862774	C4	-	-	-	Y	-	Y	-
ENSG00000140262	TCF12	0.325965503	1.11E-07	0.175131274	0.02848	0.157370929	0.029246	-0.144892758	0.001239	-0.154136313	2.97E-05	-0.014600927	0.901698	C5	Y	Y	-	-	-	-	-
ENSG00000140263	SORD	-0.46160127	2.05E-13	-0.229611506	0.000957	-0.226309423	0.000324	0.23850171	0.000451	0.25001042	7.69E-05	0.006293071	0.971304	C4	-	Y	-	-	-	-	-
ENSG00000140265	ZSCAN29	0.363857802	3.20E-08	0.115269905	0.191807	0.139525092	0.072058	-0.242096331	1.49E-05	-0.209920531	9.30E-05	0.026823449	0.835119	C5	-	Y	-	-	-	-	-
ENSG00000140280	LYSMD2	0.45592806	0.000706	0.029941895	0.905191	-0.014245288	0.949421	-0.419621703	0.003834	-0.455390044	0.000514	-0.041494803	0.905466	C5	-	Y	-	-	Y	Y	-
ENSG00000140320	BAHD1	0.382196328	1.09E-08	0.006112124	0.965933	0.1576068	0.056004	-0.369597371	1.08E-07	-0.210021647	0.002729	0.154416919	0.144747	C5	-	Y	-	-	Y	-	-
ENSG00000140323	DISP2	0.325823617	0.001581	0.241390679	0.035226	0.466309709	1.40E-06	-0.078004252	0.525947	0.155114148	0.133149	0.227792492	0.055141	C4	-	Y	-	Y	-	-	-
ENSG00000140332	TLE3	-0.969731285	1.49E-63	-0.325997862	4.24E-07	-0.524361523	4.58E-19	0.650209636	7.15E-40	0.460300143	3.23E-21	-0.195132054	0.000854	C2	-	Y	Y	Y	Y	Y	-
ENSG00000140368	PSTPIP1	-0.980682267	0.011829	-0.450433942	0.374028	-0.131839665	0.803075	0.537491591	0.253562	0.862318145	0.025879	0.322778296	NA	C4	-	Y	-	-	-	Y	-
ENSG00000140386	SCAPER	-0.522708884	5.18E-07	-0.097444611	0.512012	-0.009687256	0.959156	0.431479058	3.17E-05	0.527556072	4.91E-07	0.090642604	0.641727	C4	-	Y	-	-	Y	Y	-
ENSG00000140398	NEIL1	-0.297693171	0.003788	-0.038990375	0.82641	0.043966473	0.770122	0.265305704	0.017681	0.356504402	0.000385	0.086203314	0.653426	C4	-	-	-	-	-	Y	-
ENSG00000140416	TPM1	-0.076327147	0.238835	0.424830037	2.78E-09	0.295017515	0.000521	0.507794704	2.13E-17	0.386504558	1.32E-08	-0.127147768	0.339554	C4	-	-	Y	-	Y	Y	-
ENSG00000140450	ARRDC4	-0.695830292	9.24E-07	-0.266328722	0.119897	-0.02205594	0.92138	0.435898215	0.005204	0.688461668	5.08E-07	0.247307	0.211726	C3	-	Y	-	-	Y	Y	-
ENSG00000140455	USP3	-0.090148491	0.325784	-0.204898011	0.030357	-0.310851671	0.000188	-0.10798698	0.217894	-0.205764595	0.007615	-0.103274666	0.420342	C4	-	-	-	Y	-	-	-
ENSG00000140463	BBS4	-0.604852299	4.41E-11	-0.139348517	0.300955	-0.213293248	0.079945	0.471960202	2.06E-06	0.406410117	9.80E-05	-0.071474634	0.754039	C4	-	Y	-	-	Y	Y	-
ENSG00000140464	PML	0.423454173	1.17E-05	0.226284025	0.099481	0.327905548	0.002598	-0.190929985	0.107637	-0.081029067	0.502818	0.10471672	0.608148	C5	-	Y	-	Y	-	-	-
ENSG00000140470	ADAMTS17	0.087083451	0.706363	-0.227736989	0.340936	-0.647738196	0.00268	-0.309339312	0.1449	-0.72030357	0.000669	-0.415836228	0.19786	C5	-	-	-	Y	-	Y	-
ENSG00000140471	LINS1	0.526502888	6.04E-05	-0.108677528	0.630482	0.192626386	0.255265	-0.628283995	5.94E-07	-0.319419761	0.009365	0.303368662	0.093219	C5	-	Y	-	-	Y	Y	-
ENSG00000140481	CCDC33	-0.603644966	7.53E-06	-0.242834102	0.182151	-0.300190988	0.045386	0.367436891	0.007258	0.317690036	0.013207	-0.055363369	0.851487	C4	-	Y	-	Y	Y	Y	-
ENSG00000140519	RHCG	-0.639522518	0.295292	0.129609079	0.875571	0.780666934	0.126645	0.775618757	0.168924	1.434148161	0.002041	0.655517387	NA	C2	-	-	-	-	-	Y	-
ENSG00000140526	ABHD2	-0.224027726	0.000379	0.120467754	0.149191	0.033663563	0.76237	0.350705702	1.42E-12	0.272320833	9.93E-07	-0.083971386	0.439449	C4	-	-	-	-	Y	-	-
ENSG00000140534	TICRR	0.305811914	0.000185	0.001987008	0.990665	0.181517247	0.082508	-0.297823608	1.54E-05	-0.110192873	0.204351	0.18224575	0.092384	C5	-	Y	-	-	-	-	-
ENSG00000140538	NTRK3	-0.400688598	0.040807	-0.0188666	0.954023	-0.004880316	0.989498	0.39023091	0.071272	0.410786726	0.093257	0.0142711	0.98207	C2	-	Y	-	-	-	-	-
ENSG00000140543	DET1	-0.924259587	5.90E-05	-0.160651538	0.628414	-0.336575031	0.202456	0.770389737	0.000874	0.602732921	0.011596	-0.173188759	0.66972	C2	-	Y	-	-	Y	Y	-
ENSG00000140564	FURIN	0.632330643	5.68E-11	0.59596575	2.89E-07	0.556104537	5.06E-07	-0.029892194	0.842344	-0.061183927	0.649687	-0.036992634	0.897084	C1	-	Y	Y	Y	-	-	-
ENSG00000140598	EFL1	0.489872366	1.69E-09	0.38449453	2.55E-05	0.268532934	0.004624	-0.098688829	0.231793	-0.206657843	0.005205	-0.11345209	0.334766	C1	-	Y	Y	-	-	-	-
ENSG00000140650	PMM2	-0.358828293	8.70E-06	-0.108229102	0.318979	-0.318301227	0.00016	0.25694337	0.001769	0.055121511	0.608579	-0.2073578	0.047918	C4	-	Y	-	Y	-	-	-
ENSG00000140682	TGFB1I1	0.745188936	2.35E-18	0.26222882	0.022332	0.238537321	0.027902	-0.476964501	2.76E-09	-0.491713534	1.35E-10	-0.020240054	0.933535	C5	-	Y	-	-	Y	Y	-
ENSG00000140691	ARMC5	-0.247071945	0.091556	-0.395188459	0.007826	-0.389339306	0.008029	-0.14147813	0.375332	-0.127312751	0.434151	0.008689556	0.981547	C4	-	-	Y	Y	-	-	-
ENSG00000140830	TXNL4B	0.105466107	0.351819	-0.351582811	0.001647	-0.116452902	0.377841	-0.450517531	1.92E-06	-0.206988208	0.039604	0.238027638	0.081881	C4	-	-	Y	-	Y	-	-
ENSG00000140839	CLEC18B	-1.409604957	0.063441	-0.131023745	0.899171	0.296552924	0.718076	1.289346775	0.130489	1.720768179	0.022705	0.430336991	NA	C3	-	-	-	-	-	Y	-
ENSG00000140853	NLRC5	-1.427465424	0.041571	-0.63912871	0.455182	-0.883608673	0.195024	0.807832592	0.414327	0.559904645	0.57286	-0.245208162	NA	C3	-	Y	-	-	-	-	-
ENSG00000140854	KATNB1	0.306275938	2.19E-05	0.209041241	0.024993	0.199719814	0.058867	-0.09073287	0.32246	-0.092156274	0.367984	-0.00584152	0.982359	C1	-	Y	-	-	-	-	-
ENSG00000140876	NUDT7	-1.470189835	0.000195	-0.350308496	0.399508	-0.32159833	0.474795	1.129659146	0.011682	1.163718281	0.013706	0.030115022	NA	C3	-	Y	-	-	Y	Y	-
ENSG00000140992	PDPK1	0.346252349	2.46E-05	0.172516908	0.073975	0.231939263	0.009375	-0.16731761	0.030617	-0.099700254	0.233728	0.0623621	0.650404	C5	-	Y	-	-	-	-	-
ENSG00000140993	TIGD7	-0.139993283	0.565925	0.447814817	0.06568	0.307509887	0.215311	0.593988825	0.005633	0.462405955	0.036216	-0.136540525	0.76232	C4	-	-	-	-	Y	Y	-
ENSG00000141012	GALNS	-0.337068311	0.015519	-0.06303875	0.769154	-0.045561411	0.81629	0.280888801	0.04097	0.30657049	0.016613	0.020843646	0.947733	C4	-	Y	-	-	-	Y	-
ENSG00000141013	GAS8	-0.112584597	0.297653	0.194730271	0.082563	0.164518241	0.130642	0.313785505	0.000732	0.291837348	0.00141	-0.027160409	0.904708	C4	-	-	-	-	Y	-	-
ENSG00000141027	NCOR1	0.544277221	1.44E-12	0.229226067	0.035167	0.280329688	0.000904	-0.308669068	2.87E-07	-0.249762597	1.20E-10	0.053850654	0.656136	C5	-	Y	-	-	Y	-	-
ENSG00000141034	GID4	0.597357871	1.10E-07	-0.075966833	0.712709	0.293126408	0.05087	-0.666450888	7.86E-12	-0.290444619	0.007057	0.371432636	0.010986	C5	-	Y	-	-	Y	-	Y
ENSG00000141040	ZNF287	0.060955042	0.665549	-0.528680813	4.93E-05	-0.038894152	0.830237	-0.583230012	4.17E-07	-0.085197906	0.534604	0.492512901	0.00049	C5	-	-	Y	-	Y	-	Y

ENSG00000141068	KSR1	0.50399198	9.74E-10	0.44638502	4.50E-07	0.468492372	3.75E-08	-0.051094544	0.641968	-0.020764288	0.863186	0.024953995	0.905466	C1	-	Y	Y	Y	-	-	-
ENSG00000141084	RANBP10	0.549941079	3.44E-16	0.306425838	0.000212	0.26515653	0.003105	-0.237241773	0.000909	-0.270139877	0.000313	-0.038495699	0.838766	C5	-	Y	Y	-	-	-	-
ENSG00000141098	GFOD2	0.510725848	3.47E-14	0.207523101	0.023065	0.221622249	0.011946	-0.296903103	2.66E-05	-0.274479869	0.000114	0.017006135	0.93752	C5	-	Y	-	-	-	-	-
ENSG00000141101	NOB1	0.303358316	9.77E-05	-0.052145387	0.746981	0.039476612	0.7697	-0.349216843	0.000135	-0.249035827	0.002426	0.094463262	0.61105	C5	-	Y	-	-	Y	-	-
ENSG00000141127	PRPSAP2	-0.315317667	9.02E-08	-0.026476469	0.790684	-0.131334039	0.081484	0.295305649	5.42E-07	0.198710433	0.002589	-0.101977695	0.299528	C4	-	Y	-	-	-	-	-
ENSG00000141198	TOM1L1	-0.449801139	0.000212	0.000558018	0.997664	-0.030148443	0.880649	0.456976316	0.000285	0.434259795	0.000549	-0.02807756	0.933843	C4	-	Y	-	-	Y	Y	-
ENSG00000141219	C17orf80	0.204181691	0.001748	-0.105802805	0.233884	0.074052804	0.412206	-0.303827786	2.21E-07	-0.115835123	0.068979	0.182745898	0.026628	C5	-	-	-	-	Y	-	-
ENSG00000141252	VPS53	0.303309531	1.26E-05	0.120062343	0.236091	0.139667756	0.144032	-0.176365348	0.00543	-0.149235042	0.024765	0.022225221	0.903232	C5	-	Y	-	-	-	-	-
ENSG00000141279	NPEPPS	0.441965262	3.94E-20	0.12769483	0.077338	0.223665235	3.48E-05	-0.307436752	3.08E-12	-0.203668965	6.83E-08	0.098437376	0.160699	C5	-	Y	-	-	Y	-	-
ENSG00000141298	SSH2	0.377420803	3.52E-07	0.253576925	0.005679	0.052603593	0.644161	-0.117361083	0.127494	-0.310381087	3.95E-07	-0.198205285	0.035715	C1	Y	Y	-	-	-	Y	-
ENSG00000141314	RHBDL3	0.103774797	0.672614	-0.311687567	0.266921	-0.355222144	0.17718	-0.409009301	0.046382	-0.445274513	0.031686	-0.040659636	0.946273	C5	-	-	-	-	Y	Y	-
ENSG00000141349	G6PC3	-0.317557098	7.07E-05	-0.00820916	0.957852	-0.06798952	0.595637	0.315796672	3.06E-06	0.264225919	0.000689	-0.056916114	0.716724	C4	-	Y	-	-	Y	-	-
ENSG00000141376	BCAS3	-0.167776011	0.142429	0.297319882	0.014416	0.136276127	0.333602	0.47142156	3.12E-06	0.318822961	0.003029	-0.158263006	0.370002	C4	-	-	-	-	Y	Y	-
ENSG00000141425	RPRD1A	0.322579151	8.24E-15	0.272113105	6.19E-09	0.213412431	2.76E-06	-0.044029988	0.330725	-0.094627552	0.010269	-0.055870347	0.38741	C5	-	Y	-	-	-	-	-
ENSG00000141441	GAREM1	0.784876035	4.02E-21	0.055292369	0.719994	-0.096974903	0.434037	-0.723293422	2.70E-27	-0.867928522	7.87E-47	-0.149580659	0.19895	C5	-	Y	-	-	Y	Y	-
ENSG00000141446	ESCO1	0.577311631	1.46E-12	0.091746721	0.48663	0.133943109	0.255655	-0.479448999	1.06E-10	-0.428625909	2.23E-08	0.045152047	0.816946	C5	-	Y	-	-	Y	Y	-
ENSG00000141447	OSBPL1A	-0.622644754	1.03E-13	0.08646808	0.459248	-0.010507882	0.939542	0.715936011	2.06E-16	0.626911474	1.37E-13	-0.09401763	0.519048	C2	-	Y	-	-	Y	Y	-
ENSG00000141452	RMC1	0.512530552	2.44E-08	0.090234737	0.604701	0.159824423	0.231569	-0.414981923	1.14E-05	-0.337932447	0.000157	0.072242874	0.75233	C5	-	Y	-	-	Y	Y	-
ENSG00000141458	NPC1	0.556708203	2.93E-12	0.321148971	0.005488	0.437015853	6.72E-07	-0.229358338	0.013091	-0.104894384	0.221774	0.118919898	0.450967	C5	-	Y	Y	Y	-	-	-
ENSG00000141503	MINK1	0.315213385	7.80E-06	0.229096638	0.000697	0.270548498	2.79E-05	-0.079368628	0.323833	-0.029781071	0.744603	0.044476811	0.705159	C5	-	Y	-	-	-	-	-
ENSG00000141504	SAT2	0.220051879	0.008638	0.039300015	0.801362	-0.11076219	0.355873	-0.17428344	0.043413	-0.315780929	8.42E-05	-0.147122851	0.286772	C5	Y	-	-	-	-	Y	-
ENSG00000141524	TMC6	-0.909427279	6.41E-08	-0.266600628	0.240193	-0.060198916	0.803102	0.650363589	0.000828	0.864399731	1.75E-07	0.210345933	0.467356	C2	-	Y	-	-	Y	Y	-
ENSG00000141526	SLC16A3	-0.547545008	0.046056	0.065958358	0.87984	-0.128695868	0.734172	0.620352264	0.037103	0.433947438	0.181221	-0.19162902	NA	C4	-	Y	-	-	Y	-	-
ENSG00000141540	TTYH2	-0.856754697	4.12E-12	-0.205275063	0.218226	-0.053144508	0.777679	0.658853056	4.41E-06	0.818544172	5.24E-10	0.154933303	0.512021	C2	-	Y	-	-	Y	Y	-
ENSG00000141542	RAB40B	0.212763892	0.030645	-0.123650715	0.374631	-0.063568987	0.680104	-0.329978123	0.000925	-0.261610406	0.011734	0.063131312	0.786386	C5	-	-	-	-	Y	-	-
ENSG00000141552	ANAPC11	-0.33047199	1.23E-05	-0.085740202	0.467417	-0.165536099	0.086038	0.251582133	3.70E-05	0.17982473	0.004693	-0.077086341	0.514285	C4	-	Y	-	-	-	-	-
ENSG00000141570	CBX8	0.269610053	0.000474	-0.147905585	0.225314	-0.100670066	0.399331	-0.410924501	1.14E-05	-0.355634844	5.90E-05	0.049894424	0.844772	C5	-	-	-	-	Y	Y	-
ENSG00000141582	CBX4	0.334789781	0.000568	0.100169885	0.546833	0.190199436	0.110508	-0.228390438	0.042589	-0.129836662	0.239728	0.093178015	0.654537	C5	-	Y	-	-	-	-	-
ENSG00000141639	MAPK4	0.134866538	0.543096	-0.0840023	0.813009	-0.315868957	0.204394	-0.213077005	0.36021	-0.436676538	0.030122	-0.22912196	0.562859	C5	-	-	-	-	-	Y	-
ENSG00000141642	ELAC1	-0.821613348	1.96E-08	-0.175829658	0.379507	-0.37881595	0.013132	0.652615821	0.00012	0.457485607	0.007596	-0.200197132	0.454595	C2	-	Y	-	Y	Y	Y	-
ENSG00000141644	MBD1	0.411475769	2.54E-16	0.03767599	0.710865	0.100042217	0.15661	-0.367022791	1.83E-14	-0.296787089	1.04E-11	0.0650513	0.52139	C5	-	Y	-	-	Y	-	-
ENSG00000141655	TNFRSF11A	-1.438612865	0.011954	-1.048857786	0.106994	-0.983551217	0.103461	0.39526472	0.669111	0.470101133	0.589158	0.069725483	NA	C3	-	Y	-	-	-	-	-
ENSG00000141664	ZCCHC2	0.83475193	9.19E-15	0.385784096	0.012563	0.660257094	7.94E-10	-0.443363689	0.000109	-0.160211699	0.114659	0.278444114	0.069441	C1	-	Y	Y	Y	Y	-	-
ENSG00000141665	FBXO15	-1.011494918	0.001361	-0.543878544	0.156444	-0.452623854	0.257916	0.469973016	0.299288	0.57254816	0.198351	0.097018027	NA	C3	-	Y	-	-	-	-	-
ENSG00000141682	PMAIP1	-0.707462685	1.22E-17	-0.264810971	0.025125	-0.248719274	0.022985	0.449072148	4.58E-06	0.4730462	4.05E-07	0.019360866	0.948722	C4	Y	Y	-	-	Y	Y	-
ENSG00000141699	RETREG3	0.392652964	4.86E-08	-0.017640139	0.90903	0.125016839	0.206692	-0.403831867	9.96E-08	-0.253056405	0.000489	0.1455094	0.252234	C5	-	Y	-	-	Y	-	-
ENSG00000141736	ERBB2	-0.796771251	2.62E-12	-0.133272196	0.419714	-0.293814901	0.020281	0.670153449	5.29E-08	0.517922108	2.46E-05	-0.157605692	0.431247	C2	-	Y	-	-	Y	Y	-
ENSG00000141741	MIEN1	-0.415566365	4.00E-05	-0.168930662	0.21696	-0.166346112	0.231243	0.253086514	0.008865	0.264179916	0.009637	0.00561683	0.983851	C4	-	Y	-	-	-	-	-
ENSG00000141744	PNMT	-0.676443328	0.016901	-0.683497672	0.034976	-0.841361694	0.004565	9.71E-06	0.99997	-0.149655256	0.709921	-0.154887902	NA	C4	-	Y	Y	Y	-	-	-
ENSG00000141750	STAC2	-0.354155747	0.026707	-0.351685737	0.064256	-0.078610332	0.722567	0.009182203	0.972305	0.290483127	0.105913	0.276021927	0.289382	C4	-	Y	-	-	-	-	-
ENSG00000141753	IGFBP4	-0.87312895	4.80E-43	-0.347334794	4.26E-06	-0.321407713	6.96E-06	0.53210762	2.72E-18	0.5666819	1.65E-23	0.029257514	0.846674	C4	-	Y	Y	Y	Y	Y	-
ENSG00000141933	TPGS1	-0.145458511	0.439334	-0.14169904	0.526503	-0.377443339	0.033151	0.010193514	0.962532	-0.216845652	0.198331	-0.232719922	0.295679	C4	-	-	-	Y	-	-	-
ENSG00000141934	PLPP2	-0.375931714	6.45E-05	-0.466288087	0.00026	-0.434287661	0.000164	-0.084010856	0.552896	-0.042947232	0.763158	0.034730071	0.915306	C4	-	Y	Y	Y	-	-	-
ENSG00000141956	PRDM15	0.351486555	0.000783	0.079085127	0.659277	-0.042781049	0.820184	-0.265783801	0.012903	-0.379989108	0.000291	-0.119106001	0.575626	C5	-	Y	-	-	-	Y	-
ENSG00000141959	PFKL	-0.270026542	6.68E-07	0.010255795	0.928959	0.079146711	0.262693	0.286871113	1.49E-06	0.364031419	9.64E-13	0.071933858	0.493975	C4	-	-	-	-	-	Y	-
ENSG00000141965	FEM1A	0.208536994	0.121905	-0.0850256	0.692114	-0.127553514	0.464921	-0.28657533	0.025285	-0.32173102	0.00699	-0.040154525	0.901599	C5	-	-	-	-	-	Y	-
ENSG00000141985	SH3GL1	0.316388073	3.06E-06	0.120099743	0.224378	0.040788622	0.717335	-0.189144324	0.000303	-0.260758374	7.08E-08	-0.076786105	0.420342	C5	-	Y	-	-	-	-	-
ENSG00000141994	DUS3L	-0.136675373	0.120178	-0.018620584	0.905874	-0.327109393	0.000117	0.124512517	0.215603	-0.175655942	0.048795	-0.305517556	0.004802	C4	-	-	-	Y	-	-	Y
ENSG00000142002	DPP9	0.306738663	7.07E-07	0.21470389	0.005091	0.161756443	0.043005	-0.085593144	0.266893	-0.130445218	0.066417	-0.050109931	0.731303	C5	-	Y	-	-	-	-	-
ENSG00000142065	ZFP14	0.525598192	3.89E-09	0.139595746	0.334998	0.363952589	0.002941	-0.379620736	2.30E-05	-0.147224668	0.174612	0.227133682	0.161667	C5	-	Y	-	Y	Y	-	-
ENSG00000142082	SIRT3	-0.128884193	0.34879	0.079504774	0.693814	0.169904499	0.232015	0.214596102	0.110644	0.313286346	0.00592	0.093040941	0.696695	C4	-	-	-	-	-	Y	-
ENSG00000142102	PGGHG	-0.65358256	0.00017	-0.583382873	0.004969	-0.336558933	0.095843	0.077543823	0.792886	0.331873911	0.125697	0.249163146	0.477128	C4	-	Y	Y	-	-	-	-
ENSG00000142166	IFNAR1	0.391799961	6.73E-07	-0.048911338	0.71224	0.160475443	0.073507	-0.434312195	7.84E-08	-0.216765394	0.006604	0.212216622	0.046498	C5	-	Y	-	-	Y	-	-
ENSG00000142168	SOD1	-0.371304246	1.30E-07	-0.260991727	0.000474	-0.396927094	1.37E-09	0.11692994	0.117624	-0.010786676	0.910876	-0.133114292	0.108149	C4	-	Y	-	Y	-	-	-
ENSG00000142173	COL6A2	-0.340651994	1.46E-08	-0.141631036	0.114687	-0.084434877	0.327816	0.20532542	0.0032	0.271185609	7.81E-06	0.060103183	0.664778	C4	-	Y	-	-	-	-	-
ENSG00000142235	LMTK3	0.274257772	0.031461	-0.075011206	0.749875	0.219865196	0.155895	-0.343090407	0.01813	-0.039970443	0.824736	0.298006538	0.158463	C5	-	-	-	-	Y	-	-
ENSG00000142279	WTIP	0.748277849	0.020779	0.180334377	0.751712	0.495429741	0.223671	-0.561426702	0.111912	-0.23750974	0.536023	0.318039146	NA	C5	-	Y	-	-	-	-	-
ENSG00000142408	CACNG8	-0.092239344	0.535545	0.32889564	0.003898	0.185688015	0.15001	0.427468367	2.03E-06	0.292689374	0.00308	-0.140314053	0.245456	C2	-	-	Y	-	Y	-	-
ENSG00000142444	TIMM29	0.19892802	0.193958	-0.13910002	0.503716	-0.065115446	0.775106	-0.331941256	0.01488	-0.249435497	0.086863	0.076971535	0.800796	C5	-	-	-	-	Y	-	-
ENSG00000142459	EVI5L	-0.315071596	0.007353	0.081409524	0.624527	0.043288639	0.817175	0.403714964	0.001003	0.373012048	0.004127	-0.035845169	0.910372	C4	-	Y	-	-	Y	Y	-
ENSG00000142512	SIGLEC10	-2.06944694	0.007183	1.063264909	0.04277	0.224751922	0.812664	3.138432418	1.27E-06	2.309602759	0.006075	-0.836619814	NA	C2	-	Y	Y	-	Y	Y	-
ENSG00000142544	CTU1	0.312869015	0.065166	-0.031898514	0.909416	-0.013622257	0.962528	-0.338312196	0.042825	-0.311816499	0.073064	0.021198751	0.961147	C5	-	-	-	-	Y	-	-
ENSG00000142552	RCN3	-0.619043528	6.70E-05	-0.515467142	0.001235	-0.522202976	0.000318	0.110352175	0.647061	0.111652983	0.625676	-0.003879237	0.993673	C4	-	Y	Y	Y	-	-	-
ENSG00000142606	MMEL1	-1.091456239	0.012733	-0.4263194	0.441461	-0.76988655	0.130275	0.672585903	0.18045	0.336254296	0.59303	-0.342039682	NA	C2	-	Y	-	-	-	-	-
ENSG00000142627	EPHA2	0.029598171	0.852061	0.208350367	0.120588	0.309040358	0.026698	0.185437079	0.097822	0.294540472	0.013863	0.103849154	0.62517	C4	-	-	-	Y	-	-	-
ENSG00000142632	ARHGEF19	-0.950402575	2.69E-10	-0.439214776	0.005572	-0.71803922	7.02E-07	0.517626733	0.001206	0.247524446	0.177368	-0.276155424	0.183357	C4	-	Y	Y	Y	Y	-	-
ENSG00000142675	CNKSR1	0.54521897	0.01073	-0.010645199	0.980318	0.074852083	0.837174	-0.549848053	0.010577	-0.456470959	0.041744	0.088081673	0.872255	C5	-	Y	-	-	Y	Y	-

ENSG00000142687	KIAA0319L	-0.304443177	3.27E-08	0.081913733	0.280229	0.101627996	0.112154	0.392839292	7.34E-14	0.420698258	6.42E-18	0.022562078	0.853543	C4	-	Y	-	-	Y	Y	-
ENSG00000142748	FCN3	-4.05655306	0.020487	-2.072797165	NA	-4.147983023	NA	2.023189791	NA	NA	NA	-2.102690634	NA	C3	-	Y	-	-	-	-	-
ENSG00000142765	SYTL1	-1.172004217	0.001824	-0.530955342	0.199622	-0.360320466	0.458493	0.645642056	0.165164	0.826183936	0.085973	0.175439036	NA	C3	-	Y	-	-	-	-	-
ENSG00000142794	NBPF3	0.367091831	0.001896	0.161434869	0.279963	0.174871224	0.187529	-0.199045815	0.145626	-0.177665713	0.181158	0.016233794	0.959571	C5	-	Y	-	-	-	-	-
ENSG00000142871	CCN1	1.566799261	4.77E-17	1.714005228	3.46E-23	1.592809613	2.05E-22	0.153800946	0.454366	0.040390963	0.859505	-0.117449541	0.674586	C1	-	Y	Y	Y	-	-	-
ENSG00000142875	PRKACB	0.400993896	6.82E-14	0.215841447	0.000696	0.156446134	0.020813	-0.178639174	0.000399	-0.229652175	4.49E-06	-0.056452376	0.56882	C5	-	Y	-	-	-	-	-
ENSG00000142892	PIGK	-0.233336151	0.010553	0.086137849	0.511709	-0.005848664	0.970288	0.325460552	8.96E-05	0.241908573	0.003568	-0.088882005	0.553292	C4	-	-	-	-	Y	-	-
ENSG00000142961	MOB3C	0.205015709	0.460401	-0.304292563	0.338284	0.171346275	0.582125	-0.502916272	0.042848	-0.019415856	0.951374	0.47893767	0.135408	C1	-	-	-	-	Y	-	-
ENSG00000143013	LMO4	-0.566916155	0.00033	-0.180545423	0.384732	-0.113388388	0.646035	0.392681092	0.016098	0.468176855	0.008436	0.069989342	0.85651	C4	-	Y	-	-	Y	Y	-
ENSG00000143033	MTF2	0.257457862	3.68E-05	-0.078516506	0.404416	0.062532562	0.492389	-0.329632726	7.21E-09	-0.180446713	0.001784	0.143931334	0.088607	C5	Y	-	-	-	Y	-	-
ENSG00000143061	IGSF3	-0.575056276	6.76E-22	-0.165299919	0.030621	-0.042864458	0.652135	0.415899632	1.33E-15	0.546560043	4.08E-29	0.125407493	0.118464	C4	-	Y	-	-	Y	Y	-
ENSG00000143093	STRIP1	0.375996126	1.26E-07	0.20084417	0.022343	0.114176359	0.263683	-0.168672351	0.014198	-0.247257456	0.000423	-0.083947506	0.529712	C1	-	Y	-	-	-	-	-
ENSG00000143110	C1orf162	0.368198739	0.428897	-0.65946224	0.315004	-0.305950462	0.666674	-1.02059433	0.044083	-0.659186019	0.213912	0.355621213	NA	C5	-	-	-	-	Y	-	-
ENSG00000143147	GPR161	0.425263295	6.89E-11	0.245955529	0.00084	0.360171249	6.07E-08	-0.172994994	0.001314	-0.050637374	0.419805	0.116934479	0.102615	C4	-	Y	-	Y	-	-	-
ENSG00000143157	POGK	0.432152705	6.85E-15	0.172603647	0.015718	0.176219658	0.007166	-0.253040807	4.91E-11	-0.241630336	6.59E-12	0.006155742	0.958938	C5	-	Y	-	-	-	-	-
ENSG00000143162	CREG1	-0.422867368	0.000921	-0.200214908	0.105399	-0.127099723	0.331812	0.229626743	0.079784	0.310669603	0.010742	0.076589386	0.668709	C4	-	Y	-	-	-	Y	-
ENSG00000143164	DCAF6	-0.278235395	0.001476	-0.094689653	0.429852	0.009799969	0.950337	0.18989428	0.027296	0.302522817	0.000331	0.107272459	0.448502	C4	-	-	-	-	-	Y	-
ENSG00000143303	RRNAD1	-0.304317363	0.004992	-0.057400224	0.705345	-0.056874329	0.698518	0.253497234	0.010597	0.262152582	0.009728	0.003272462	0.990185	C4	-	Y	-	-	-	-	-
ENSG00000143314	MRPL24	-0.505392446	4.90E-20	-0.099908235	0.210871	-0.136151224	0.058099	0.411916543	1.01E-15	0.384107584	7.93E-14	-0.033264335	0.787669	C4	Y	Y	-	-	Y	Y	-
ENSG00000143315	PIGM	0.307369058	0.002438	0.074362923	0.607705	0.201054234	0.073977	-0.226872941	0.001778	-0.092563315	0.275827	0.128901775	0.202929	C5	-	Y	-	-	-	-	-
ENSG00000143320	CRABP2	-0.708131804	6.60E-10	-0.349336774	0.018367	-0.447122333	0.000815	0.364551798	0.001233	0.276745505	0.01473	-0.093792365	0.68278	C4	-	Y	Y	Y	Y	-	-
ENSG00000143333	RGS16	-0.669622091	2.91E-09	0.190011968	0.156274	0.277052236	0.017379	0.866039063	2.11E-15	0.961419009	1.09E-19	0.090007378	0.644037	C4	-	Y	-	-	Y	Y	-
ENSG00000143337	TOR1AIP1	0.339551172	5.11E-08	0.02697796	0.85371	0.040565969	0.69096	-0.306185504	1.31E-05	-0.284219062	4.44E-07	0.01634981	0.938311	C5	-	Y	-	-	Y	-	-
ENSG00000143340	FAM163A	-0.51579217	4.26E-24	-0.262038175	0.001108	-0.290245574	2.87E-06	0.26003794	8.03E-05	0.240083472	3.37E-06	-0.025147019	0.889237	C4	-	Y	-	-	-	-	-
ENSG00000143353	LYPLAL1	-1.013489928	3.57E-17	-0.476020172	6.46E-05	-0.436998355	0.000974	0.543683118	8.98E-05	0.591471174	6.72E-05	0.042229848	0.901808	C3	-	Y	Y	Y	Y	Y	-
ENSG00000143363	PRUNE1	0.451748064	1.04E-11	0.267170486	0.000334	0.369035581	2.85E-08	-0.178211112	0.012219	-0.068075738	0.387749	0.104662058	0.310359	C5	-	Y	-	Y	-	-	-
ENSG00000143365	RORC	-0.891789636	0.006208	-0.344530159	0.370579	-0.633164782	0.097855	0.55439701	0.11953	0.273295737	0.559957	-0.286568821	NA	C3	-	Y	-	-	-	-	-
ENSG00000143375	CGN	-0.623422339	0.000164	-0.328240743	0.109656	-0.207003842	0.284218	0.303003197	0.160131	0.431160741	0.019641	0.124147818	0.723061	C4	-	Y	-	-	-	Y	-
ENSG00000143376	SNX27	0.643129807	1.67E-17	0.122238726	0.238895	0.304285487	0.000172	-0.514723249	3.42E-16	-0.324239961	2.41E-07	0.185000184	0.02897	C5	-	Y	-	Y	Y	Y	-
ENSG00000143382	ADAMTSL4	-0.937620491	0.000371	-0.167540497	0.663478	-0.394645449	0.191372	0.776883327	0.005792	0.557928922	0.064817	-0.224285593	0.638879	C3	-	Y	-	-	Y	-	-
ENSG00000143409	MINDY1	0.542247034	2.99E-08	0.417366532	0.00016	0.49625185	4.20E-06	-0.118324823	0.283791	-0.031290302	0.819287	0.081721897	0.659952	C1	-	Y	Y	Y	-	-	-
ENSG00000143437	ARNT	0.33581967	2.58E-07	0.234804229	0.002396	0.238971246	0.000188	-0.094497334	0.212719	-0.082142617	0.214685	0.007030481	0.967582	C4	-	Y	-	-	-	-	-
ENSG00000143442	POGZ	0.187388521	0.00024	-0.144626281	0.035492	0.022294068	0.789728	-0.325328011	4.56E-17	-0.150626516	7.77E-06	0.169492233	0.001098	C5	-	-	-	-	Y	-	-
ENSG00000143443	C1orf56	-0.470177626	0.000762	-0.068666571	0.762365	-0.121852979	0.507722	0.407737186	0.003621	0.362956112	0.009226	-0.050052304	0.873484	C4	-	Y	-	-	Y	Y	-
ENSG00000143457	GOLPH3L	0.533519528	1.61E-09	0.128629462	0.400593	0.42331551	9.25E-06	-0.397903125	2.05E-05	-0.095482887	0.307449	0.297302074	0.013285	C1	-	Y	-	Y	Y	-	-
ENSG00000143473	KCNH1	0.483799648	0.155501	0.177397635	0.7078	0.633018222	0.033762	-0.300270194	0.377511	0.163558108	0.623931	0.455259419	0.200382	C1	-	-	-	Y	-	-	-
ENSG00000143476	DTL	-0.316692015	8.95E-11	-0.353290618	1.02E-08	-0.403898048	1.47E-10	-0.029997374	0.68376	-0.07290129	0.269677	-0.047492356	0.735476	C4	Y	Y	Y	Y	-	-	-
ENSG00000143479	DYRK3	0.634235435	5.84E-20	0.245525661	0.012242	0.57469609	4.40E-12	-0.382431383	7.80E-08	-0.044552977	0.64082	0.332101575	0.000621	C1	-	Y	-	Y	Y	-	Y
ENSG00000143493	INTS7	0.227518787	6.30E-05	-0.06792565	0.451996	-0.156670493	0.043932	-0.28889745	3.84E-07	-0.369642091	3.73E-10	-0.085771736	0.472263	C5	-	-	-	-	-	Y	-
ENSG00000143498	TAF1A	0.569448438	9.88E-16	-0.037248918	0.796389	0.254933449	0.004315	-0.599952219	8.04E-18	-0.29997246	1.83E-05	0.29492042	0.002264	C5	-	Y	-	-	Y	-	-
ENSG00000143507	DUSP10	-0.345522621	0.027236	-0.278709065	0.140474	0.010762312	0.967124	0.073004323	0.737013	0.37086835	0.017916	0.292249871	0.183658	C4	-	Y	-	-	-	Y	-
ENSG00000143515	ATP8B2	0.688202067	1.34E-44	0.295740701	6.22E-07	0.399921372	1.40E-13	-0.385964614	1.74E-24	-0.273641677	2.20E-13	0.106887867	0.074897	C5	Y	Y	-	Y	Y	-	-
ENSG00000143578	CREB3L4	-0.583602369	2.95E-09	-0.210433878	0.07387	-0.240329271	0.022878	0.379415136	0.000416	0.358182048	0.000617	-0.027030541	0.915324	C2	-	Y	-	-	Y	Y	-
ENSG00000143590	EFNA3	0.627227446	5.05E-05	0.352164967	0.082095	0.769980867	1.46E-06	-0.269125692	0.104174	0.157355314	0.350299	0.421242667	0.026233	C1	-	Y	-	Y	-	-	Y
ENSG00000143595	AQP10	-1.47702796	4.18E-09	-0.196126632	0.570375	-0.321851859	0.238894	1.28888461	8.53E-07	1.170397353	4.80E-06	-0.121596007	0.801925	C2	Y	Y	-	-	Y	Y	-
ENSG00000143622	RIT1	0.310007728	7.73E-06	0.015046028	0.910114	0.092607457	0.34758	-0.288508096	6.43E-05	-0.202571166	0.007035	0.080491168	0.559895	C5	-	Y	-	-	-	-	-
ENSG00000143630	HCN3	0.454965776	1.34E-12	0.250407275	0.003481	0.366521113	4.76E-07	-0.197865029	0.00525	-0.073761491	0.330235	0.11893127	0.307619	C5	-	Y	-	Y	-	-	-
ENSG00000143633	C1orf131	0.192414725	0.046151	-0.022160552	0.890938	-0.163172906	0.118323	-0.208269921	0.033123	-0.340912977	0.000138	-0.138204521	0.333997	C5	-	-	-	-	-	Y	-
ENSG00000143643	TTC13	0.800635081	3.29E-30	0.392759774	1.59E-05	0.342070142	0.000134	-0.401031689	6.10E-08	-0.443917727	9.45E-10	-0.047701246	0.804185	C5	-	Y	Y	Y	Y	Y	-
ENSG00000143669	LYST	0.854153576	3.26E-14	0.477681296	0.000325	0.554756819	1.68E-05	-0.369951076	0.000794	-0.2848052	0.013128	0.079820371	0.738057	C1	-	Y	Y	Y	Y	-	-
ENSG00000143674	MAP3K21	-0.275736338	0.073555	-0.158797609	0.400641	0.316784373	0.030602	0.123522523	0.461858	0.607575528	9.49E-07	0.478562558	0.00049	C4	-	-	-	Y	-	Y	Y
ENSG00000143702	CEP170	0.353648486	1.01E-06	0.193989914	0.037502	0.178571929	0.035551	-0.153379692	0.001481	-0.160668816	4.74E-05	-0.012581321	0.927369	C1	-	Y	-	-	-	-	-
ENSG00000143756	FBXO28	0.347432845	1.28E-08	-0.123698239	0.151508	0.127528518	0.09623	-0.464724835	1.07E-15	-0.205288825	0.000347	0.254091233	0.000585	C5	-	Y	-	-	Y	-	-
ENSG00000143771	CNIH4	0.315292338	1.24E-06	-0.009671676	0.937941	0.120093837	0.131575	-0.318536676	7.16E-07	-0.180457964	0.006956	0.132663724	0.160243	C5	-	Y	-	-	Y	-	-
ENSG00000143786	CNIH3	-0.595153415	0.00419	-0.060986353	0.853719	-0.037417664	0.906724	0.540786281	0.009904	0.572541779	0.005881	0.026199812	0.95884	C4	-	Y	-	-	Y	Y	-
ENSG00000143793	C1orf35	0.193398647	0.023745	-0.141598772	0.182151	-0.123109084	0.307601	-0.328572197	1.20E-06	-0.30211994	9.29E-05	0.02122101	0.92351	C5	-	-	-	-	Y	Y	-
ENSG00000143819	EPHX1	-0.476985614	1.64E-05	-0.363087584	0.002741	-0.200577122	0.097669	0.121125687	0.437595	0.291238903	0.01952	0.165229452	0.381655	C4	-	Y	Y	-	-	-	-
ENSG00000143845	ETNK2	-0.313663458	0.007865	-0.099238027	0.529128	0.266985774	0.04174	0.221258166	0.071062	0.595393859	1.18E-07	0.368976596	0.006627	C4	-	Y	-	-	-	Y	Y
ENSG00000143847	PPFIA4	-0.659124427	1.71E-08	0.372419699	0.028147	0.444170558	0.001671	1.03812091	2.32E-15	1.118442472	2.06E-22	0.072077419	0.890117	C4	-	Y	Y	Y	Y	Y	-
ENSG00000143850	PLEKHA6	-0.33915885	3.57E-13	0.164189121	0.013477	0.128046963	0.066822	0.509923981	1.41E-31	0.481913551	3.09E-28	-0.033435789	0.796279	C4	-	Y	-	-	Y	Y	-
ENSG00000143858	SYT2	0.878306192	0.028045	0.24396687	0.746325	0.759987449	0.117099	-0.629532192	0.162866	-0.10395702	0.842967	0.519619701	NA	C1	-	Y	-	-	-	-	-
ENSG00000143878	RHOB	-0.401878035	1.38E-07	0.55578077	1.78E-09	0.260364815	0.004669	0.964201138	2.88E-37	0.677132266	3.32E-22	-0.292457421	0.007093	C2	-	Y	Y	-	Y	Y	-
ENSG00000143889	HNRNPLL	0.729462845	1.04E-50	0.241524213	0.00046	0.265304939	0.000133	-0.481906265	7.68E-23	-0.449197668	7.60E-19	0.026596576	0.867365	C5	-	Y	-	-	Y	Y	-
ENSG00000143919	CAMKMT	-1.030093412	5.71E-16	-0.627793918	2.01E-06	-0.380483493	0.004942	0.409602627	0.01005	0.66437388	4.52E-06	0.249997867	0.224947	C3	-	Y	Y	Y	Y	Y	-
ENSG00000143942	CHAC2	0.19175643	0.293712	-0.283295974	0.302002	-0.173714768	0.46772	-0.4679304	0.018412	-0.351117957	0.045489	0.112650557	0.809246	C5	-	-	-	-	Y	Y	-
ENSG00000143952	VPS54	0.397868965	8.98E-09	0.209500585	0.020795	0.147021481	0.12306	-0.181980951	0.010338	-0.236247679	0.000655	-0.059586572	0.693025	C5	Y	Y	-	-	-	-	-
ENSG00000143970	ASXL2	0.653153879	3.89E-12	0.283676907	0.013263	0.212431429	0.066828	-0.363571189	7.11E-07	-0.426303339	1.43E-09	-0.06805684	0.652347	C1	-	Y	-	-	Y	Y	-

ENSG00000143994	ABHD1	-0.939027956	0.000538	-0.600279808	0.072775	-0.161441824	0.676419	0.349441878	0.393599	0.792045374	0.014621	0.437796213	NA	C4	-	Y	-	-	-	Y	-
ENSG00000143995	MEIS1	-0.707154495	2.88E-18	-0.687580335	6.99E-17	-0.736827965	7.55E-18	0.026148059	0.837037	-0.014713652	0.915106	-0.046344508	0.819537	C4	-	Y	Y	Y	-	-	-
ENSG00000144026	ZNF514	0.318688233	0.000364	0.18631811	0.099486	0.46359984	8.44E-07	-0.12588458	0.198283	0.159858793	0.086236	0.28028097	0.009933	C4	-	Y	-	Y	-	-	-
ENSG00000144029	MRPS5	0.132161033	0.075874	-0.234147872	0.00544	0.062155655	0.562172	-0.359979719	2.24E-08	-0.055359068	0.500356	0.299269876	0.000255	C5	-	-	-	-	Y	-	-
ENSG00000144040	SFXN5	0.330650397	0.012945	0.285483116	0.073069	0.048862092	0.820922	-0.038741515	0.829763	-0.267298112	0.051571	-0.233964637	0.228015	C1	-	Y	-	-	-	-	-
ENSG00000144045	DQX1	0.379605298	1.16E-12	0.289748947	1.25E-05	0.035300839	0.697645	-0.083163782	0.151648	-0.329711758	2.66E-12	-0.251835697	5.70E-05	C5	-	Y	-	-	-	Y	-
ENSG00000144048	DUSP11	0.550175722	3.09E-08	0.134059033	0.377403	0.239848979	0.060937	-0.409648646	9.19E-05	-0.295677491	0.006385	0.108847194	0.604693	C5	-	Y	-	-	Y	-	-
ENSG00000144057	ST6GAL2	-0.105964442	0.475781	-0.303433991	0.033927	-0.290149228	0.067369	-0.190287281	0.220139	-0.169188198	0.326319	0.014755555	0.972653	C4	-	-	Y	-	-	-	-
ENSG00000144063	MALL	4.125320159	0.038764	3.038473204	NA	2.76440632	NA	-1.075834828	NA	-1.347268885	NA	-0.275658673	NA	C5	-	Y	-	-	-	-	-
ENSG00000144130	NT5DC4	-1.013702742	0.515593	1.205918054	NA	-0.028209067	NA	2.225812606	0.030945	0.997593745	0.517869	-1.226775891	NA	C2	-	-	-	-	Y	-	-
ENSG00000144134	RABL2A	0.207567101	0.129689	0.293311708	0.065146	0.349015477	0.010971	0.092415298	0.578346	0.156248781	0.267304	0.058445248	0.846395	C4	-	-	-	Y	-	-	-
ENSG00000144218	AFF3	-0.021370216	0.923663	-0.390194664	0.015854	-0.355652381	0.023098	-0.362890167	0.006916	-0.320403321	0.017267	0.037167275	0.909699	C4	-	-	Y	Y	Y	Y	-
ENSG00000144224	UBXN4	0.329605426	1.21E-09	0.170111248	0.014074	0.145048815	0.023719	-0.152750694	0.001114	-0.16986811	7.41E-05	-0.022534822	0.840887	C5	-	Y	-	-	-	-	-
ENSG00000144228	SPOPL	0.32563873	0.000159	0.080018732	0.56059	0.07956015	0.522263	-0.239454727	0.002668	-0.231445549	0.002728	0.002497499	0.991899	C5	-	Y	-	-	-	-	-
ENSG00000144283	PKP4	0.318224262	9.51E-07	0.339807149	7.64E-07	0.312951425	5.17E-05	0.027946815	0.74201	0.009579563	0.924577	-0.02399291	0.888164	C4	-	Y	Y	Y	-	-	-
ENSG00000144285	SCN1A	-1.692397356	0.016068	-1.422389841	NA	-0.982989441	0.163822	0.297065716	0.835838	0.724343366	0.460979	0.43197732	NA	C3	-	Y	-	-	-	-	-
ENSG00000144354	CDCA7	-0.247283084	1.24E-06	-0.198539424	0.020644	-0.380525562	2.83E-07	0.055483102	0.489377	-0.118794688	0.082764	-0.178808261	0.128137	C4	Y	-	-	Y	-	-	-
ENSG00000144357	UBR3	0.650800062	1.38E-17	0.174400976	0.105379	0.144887865	0.154594	-0.46957178	1.15E-13	-0.491166499	9.07E-17	-0.026977509	0.87848	C5	-	Y	-	-	Y	Y	-
ENSG00000144369	FAM171B	0.352888073	8.51E-09	0.169427875	0.031796	0.159211	0.032311	-0.176943091	0.008343	-0.178950643	0.005229	-0.007315661	0.968619	C5	-	Y	-	-	-	-	-
ENSG00000144407	PTH2R	-0.535704717	0.002196	0.044925335	0.868958	-0.101969064	0.646132	0.588715631	0.001279	0.448467927	0.015865	-0.144163858	0.632826	C2	-	Y	-	-	Y	Y	-
ENSG00000144445	KANSL1L	0.370018565	3.35E-05	0.022383757	0.892009	0.216543369	0.033153	-0.341309155	6.44E-05	-0.138912283	0.138151	0.196942636	0.094572	C5	-	Y	-	-	Y	-	-
ENSG00000144452	ABCA12	-0.396004501	3.55E-06	-0.204338	0.047161	-0.288858294	0.001304	0.198224321	0.010639	0.121226179	0.134744	-0.082531751	0.522661	C4	-	Y	-	-	-	-	-
ENSG00000144455	SUMF1	-0.419099791	0.000371	-0.138548998	0.429981	-0.111502859	0.436573	0.287567995	0.050328	0.322263688	0.009674	0.030085429	0.92968	C4	-	Y	-	-	-	Y	-
ENSG00000144468	RHBDD1	-0.431616295	6.69E-06	0.03342598	0.849334	0.166557566	0.135984	0.471343132	3.99E-06	0.612835079	5.91E-11	0.135895421	0.415965	C4	-	Y	-	-	Y	Y	-
ENSG00000144485	HES6	-0.425674065	0.000407	-0.16975866	0.338656	-0.296464735	0.038949	0.262227267	0.072536	0.144031578	0.357	-0.123615783	0.641855	C4	-	Y	-	-	-	-	-
ENSG00000144579	CTDSP1	-0.432530623	3.72E-07	-0.136345148	0.291339	-0.160478158	0.145351	0.302629494	0.000113	0.287262614	8.17E-05	-0.020970234	0.926363	C4	-	Y	-	-	Y	-	-
ENSG00000144583	MARCHF4	-0.294602198	0.005999	-0.103992088	0.551901	0.113292301	0.424568	0.196886562	0.146745	0.422656597	0.000113	0.220524466	0.219285	C4	-	-	-	-	-	Y	-
ENSG00000144591	GMPPA	-0.27402459	0.010001	-0.090083672	0.560746	0.012588799	0.939762	0.190627603	0.12742	0.301357877	0.004921	0.105548857	0.57838	C4	-	-	-	-	-	Y	-
ENSG00000144619	CNTN4	-0.455346456	1.27E-07	-0.019929556	0.903778	0.162750855	0.088955	0.442207772	7.61E-09	0.632275647	1.62E-22	0.184423001	0.058342	C4	-	Y	-	-	Y	Y	-
ENSG00000144635	DYNC1LI1	0.30405137	6.91E-08	0.00535045	0.967013	-0.056566624	0.535049	-0.292147825	9.61E-07	-0.34602656	1.44E-09	-0.058935956	0.647484	C5	-	Y	-	-	-	Y	-
ENSG00000144645	OSBPL10	-1.188359781	0.020342	0.098104507	0.894609	0.084845605	0.90461	1.293506903	0.019585	1.288040164	0.020338	-0.01090004	NA	C3	-	Y	-	-	Y	Y	-
ENSG00000144647	POMGNT2	0.309571385	0.001582	0.073220433	0.693573	0.084030292	0.597184	-0.229615735	0.044562	-0.210770507	0.054306	0.01366551	0.967582	C5	-	Y	-	-	-	-	-
ENSG00000144668	ITGA9	-0.354967844	3.60E-06	-0.020443543	0.903779	0.167842192	0.083918	0.3412241	0.000274	0.537507869	4.17E-12	0.190899653	0.183357	C4	-	Y	-	-	Y	Y	-
ENSG00000144674	GOLGA4	0.923406818	4.98E-28	0.325279291	0.003189	0.448999469	2.59E-06	-0.591225096	3.33E-27	-0.46018039	5.98E-21	0.1258683	0.196223	C5	-	Y	Y	Y	Y	Y	-
ENSG00000144712	CAND2	-0.391045743	0.003824	-0.2423303	0.162548	-0.219297197	0.182803	0.155068315	0.381988	0.186575289	0.267812	0.025836207	0.948722	C4	-	Y	-	-	-	-	-
ENSG00000144736	SHQ1	0.305747557	1.99E-05	0.044714619	0.717905	0.114814963	0.263179	-0.254630125	0.000661	-0.176444251	0.031046	0.073211946	0.63983	C5	Y	Y	-	-	-	-	-
ENSG00000144741	SLC25A26	-0.216147138	0.100044	-0.0261086	0.902513	-0.309430867	0.019745	0.196460174	0.134888	-0.078387411	0.616987	-0.28020703	0.072294	C4	-	-	-	Y	-	-	-
ENSG00000144746	ARL6IP5	-0.944110909	1.86E-17	-0.343317819	0.007045	-0.064662953	0.703437	0.607138381	6.16E-08	0.894364736	6.81E-17	0.2819269	0.051542	C4	-	Y	Y	-	Y	Y	-
ENSG00000144747	TMF1	0.664211022	6.61E-17	0.206067739	0.116795	0.361293963	6.46E-05	-0.451507346	1.13E-07	-0.288416992	2.37E-05	0.157933044	0.266575	C5	-	Y	-	Y	Y	-	-
ENSG00000144749	LRIG1	-1.115966169	0.000119	-0.37147423	0.304522	-0.835176134	0.008301	0.751360472	0.00848	0.295185974	0.420676	-0.461916041	NA	C4	-	Y	-	Y	Y	-	-
ENSG00000144824	PHLDB2	-0.021811031	0.944144	0.265694233	0.34216	0.478313402	0.017264	0.293876767	0.230181	0.514616105	0.007744	0.21568026	0.549271	C4	-	-	-	Y	-	Y	-
ENSG00000144840	RABL3	-0.406615294	6.83E-06	-0.161639345	0.20074	-0.347643468	0.000457	0.251437615	0.014373	0.073731735	0.549439	-0.183262585	0.215408	C4	-	Y	-	Y	-	-	-
ENSG00000144867	SRPRB	0.46863917	4.80E-11	-0.027769701	0.863828	-0.008811584	0.95141	-0.489538523	3.99E-10	-0.462787233	4.92E-11	0.02176952	0.928848	C5	-	Y	-	-	Y	Y	-
ENSG00000144868	TMEM108	-0.423102452	4.59E-06	0.030683735	0.836175	-0.04960722	0.747089	0.460157104	1.25E-07	0.388050149	0.000132	-0.077415347	0.676845	C4	-	Y	-	-	Y	Y	-
ENSG00000144909	OSBPL11	0.324597403	0.001646	0.131878019	0.358283	0.115446379	0.418476	-0.186462268	0.038275	-0.194681618	0.034152	-0.013663757	0.955888	C5	-	Y	-	-	-	-	-
ENSG00000144959	NCEH1	0.874464041	0.001775	0.414039585	0.264875	0.280464506	0.432598	-0.453717149	0.135863	-0.579810667	0.034018	-0.129672631	0.83126	C5	-	Y	-	-	-	Y	-
ENSG00000145002	FAM86B2	-1.442586608	0.031414	-0.738639301	0.256681	-0.53490473	0.399034	0.714829283	0.436941	0.923011005	0.264996	0.205124939	NA	C3	-	Y	-	-	-	-	-
ENSG00000145016	RUBCN	0.905836752	3.90E-15	0.667952278	8.92E-07	0.501538327	0.000172	-0.232062581	0.029501	-0.389784974	5.32E-05	-0.163248628	0.339047	C1	-	Y	Y	Y	-	Y	-
ENSG00000145022	TCTA	-0.361522194	0.001894	-0.097262755	0.532812	-0.155366077	0.223305	0.27037281	0.032815	0.220919719	0.084345	-0.055262355	0.818037	C4	-	Y	-	-	-	-	-
ENSG00000145041	DCAF1	0.343413391	7.95E-07	-0.011234459	0.935559	0.037451835	0.73136	-0.347889284	6.93E-10	-0.291439329	7.88E-08	0.050958392	0.659842	C5	Y	Y	-	-	Y	-	-
ENSG00000145198	VWA5B2	0.325536462	0.14686	-0.021392428	0.953741	-0.203449507	0.507878	-0.341652139	0.135023	-0.514309406	0.032827	-0.177435883	0.693425	C5	-	-	-	-	-	Y	-
ENSG00000145214	DGKQ	0.306396847	0.007681	0.316735058	0.041734	0.217195677	0.166483	0.016715119	0.930601	-0.074197613	0.645061	-0.097060442	0.73559	C4	-	Y	Y	-	-	-	-
ENSG00000145217	SLC26A1	-0.194093676	0.400543	-0.071238706	0.828383	0.261930952	0.218308	0.130044379	0.646405	0.470507509	0.020788	0.335753516	0.244738	C4	-	-	-	-	-	Y	-
ENSG00000145220	LYAR	0.454747413	4.92E-08	-0.026911937	0.87301	0.0685594	0.577244	-0.475017139	7.07E-08	-0.371385814	5.49E-06	0.098380425	0.547978	C5	Y	Y	-	-	Y	Y	-
ENSG00000145246	ATP10D	0.404775861	0.000266	0.093575946	0.608459	0.124420005	0.41247	-0.304800009	0.012839	-0.265697223	0.02459	0.033717368	0.913323	C5	-	Y	-	-	Y	-	-
ENSG00000145247	OCIAD2	-0.221250182	0.027729	-0.316155671	0.00609	-0.335882165	0.003467	-0.088165979	0.36696	-0.099913257	0.317096	-0.016921398	0.946273	C4	-	-	Y	Y	-	-	-
ENSG00000145248	SLC10A4	0.004167364	0.974293	-0.139299059	0.23671	-0.330611904	0.000211	-0.137332528	0.199489	-0.320000469	0.000385	-0.188374727	0.16309	C5	-	-	-	Y	-	Y	-
ENSG00000145284	SCD5	-0.46403163	0.000106	-0.382994572	0.008461	-0.116796319	0.470432	0.087068334	0.624763	0.361904604	0.004976	0.268908093	0.158463	C4	-	Y	Y	-	-	Y	-
ENSG00000145331	TRMT10A	-0.482287511	0.000984	0.048058806	0.866097	0.086065226	0.698563	0.537752978	0.002269	0.58301053	0.000272	0.040020438	0.933843	C2	-	Y	-	-	Y	Y	-
ENSG00000145335	SNCA	-0.698272034	1.68E-08	-0.231463744	0.161205	-0.098618977	0.523001	0.473467744	0.001172	0.614499712	4.59E-07	0.135807965	0.550996	C4	-	Y	-	-	Y	Y	-
ENSG00000145348	TBCK	0.167778916	0.090941	0.090930276	0.491255	0.383102836	1.76E-06	-0.07074246	0.562297	0.229833202	0.007572	0.294619291	0.003474	C4	-	-	-	Y	-	-	-
ENSG00000145362	ANK2	-0.490455909	0.00395	0.156555302	0.56254	0.025900089	0.884864	0.653376709	6.03E-06	0.530114476	1.64E-13	-0.127264073	0.416632	C4	-	Y	-	-	Y	Y	-
ENSG00000145365	TIFA	0.484737962	0.000121	0.07635684	0.726264	0.28516857	0.071383	-0.402029904	0.002683	-0.184953035	0.221605	0.211893552	0.339047	C1	-	Y	-	-	Y	-	-
ENSG00000145375	SPATA5	0.31552224	0.003492	-0.006952941	0.972405	0.046411583	0.771712	-0.315921868	0.002619	-0.25449704	0.013885	0.055932706	0.798659	C5	-	Y	-	-	Y	-	-
ENSG00000145388	METTL14	0.10974352	0.215727	-0.25699662	0.002054	-0.083494541	0.405413	-0.360361625	3.81E-06	-0.178405293	0.03107	0.176420909	0.097409	C5	Y	-	-	-	Y	-	-
ENSG00000145390	USP53	0.477217559	0.004855	0.238872964	0.264443	0.355556226	0.03938	-0.231925971	0.147159	-0.107149642	0.521132	0.119314065	0.62104	C1	-	Y	-	Y	-	-	-
ENSG00000145414	NAF1	0.360325712	0.000533	0.030544399	0.889599	0.078652898	0.680234	-0.322729193	0.004297	-0.266945255	0.027718	0.051457148	0.876139	C5	-	Y	-	-	Y	-	-

ENSG00000145416	MARCHF1	-0.246390927	0.145445	0.095512365	0.681826	-0.004945386	0.986237	0.348043375	0.018088	0.256504493	0.131573	-0.097258724	0.747544	C2	-	-	-	-	Y	-	-
ENSG00000145428	RNF175	-0.701526513	0.027947	-0.51844409	0.159976	0.003326641	0.994131	0.189787761	0.601352	0.719901458	0.004899	0.524792512	0.110304	C4	-	Y	-	-	-	Y	-
ENSG00000145439	CBR4	-0.523242029	1.12E-08	-0.205428674	0.036343	-0.062811033	0.61583	0.324362669	0.000371	0.475242116	8.33E-08	0.145658065	0.228015	C4	-	Y	-	-	Y	Y	-
ENSG00000145476	CYP4V2	-0.639334669	3.02E-12	0.094895468	0.467152	0.195695154	0.076828	0.741508625	1.29E-13	0.849644952	1.28E-17	0.102902485	0.58797	C2	-	Y	-	-	Y	Y	-
ENSG00000145569	OTULINL	-0.210505503	0.003891	0.132127047	0.113958	0.040515385	0.696696	0.34913472	3.46E-08	0.265575758	5.39E-05	-0.088853798	0.393067	C4	-	-	-	-	Y	-	-
ENSG00000145632	PLK2	0.076582382	0.646655	0.452983217	0.000189	0.140356851	0.419937	0.382850343	0.001755	0.07842591	0.665583	-0.309580014	0.057137	C4	-	-	Y	-	Y	-	-
ENSG00000145687	SSBP2	-0.387918922	6.69E-06	-0.180748572	0.059561	-0.062090279	0.562172	0.213359224	0.024393	0.340642003	5.07E-05	0.121393885	0.318784	C4	-	Y	-	-	-	Y	-
ENSG00000145700	ANKRD31	-1.365326452	0.002108	-0.783880743	0.099481	-0.791994661	0.087777	0.586178278	0.339565	0.586706518	0.34428	-0.005423114	NA	C3	-	Y	-	-	-	-	-
ENSG00000145703	IQGAP2	-0.731197561	0.003045	-0.094197439	0.815068	0.047092456	0.889552	0.646298494	0.030582	0.792967737	0.001614	0.144083089	0.77759	C3	-	Y	-	-	Y	Y	-
ENSG00000145723	GIN1	-0.209690274	0.139149	-0.371532935	0.021525	-0.434452368	0.00448	-0.155542855	0.375566	-0.210101927	0.207731	-0.059939097	0.876666	C4	-	-	Y	Y	-	-	-
ENSG00000145734	BDP1	0.550160044	3.85E-10	0.308915635	0.007298	0.270716943	0.013217	-0.234847018	0.000393	-0.264916957	1.09E-05	-0.03547532	0.84587	C1	-	Y	Y	-	-	-	-
ENSG00000145740	SLC30A5	0.44357122	2.57E-15	0.079838242	0.349458	0.152477178	0.024141	-0.357259348	7.69E-11	-0.276564325	1.84E-07	0.075335302	0.463768	C5	-	Y	-	-	Y	-	-
ENSG00000145779	TNFAIP8	-0.1349938	0.440159	-0.080923802	0.71918	0.181953707	0.297598	0.060876291	0.773752	0.33164705	0.031715	0.2658041	0.19895	C4	-	-	-	-	-	Y	-
ENSG00000145819	ARHGAP26	-0.127760064	0.338135	0.094799057	0.579179	0.170643708	0.213231	0.228594573	0.018034	0.312408787	0.000579	0.078338032	0.652484	C4	-	-	-	-	-	Y	-
ENSG00000145833	DDX46	0.550415339	1.70E-40	0.139259592	0.030863	-0.005803215	0.942501	-0.404489485	7.50E-23	-0.541563672	2.13E-63	-0.14244831	0.01827	C5	-	Y	-	-	Y	Y	-
ENSG00000145861	C1QTNF2	-2.00513462	0.048321	-1.659967766	NA	-1.286835799	NA	0.333833406	NA	0.733411383	0.636097	0.385459412	NA	C3	-	Y	-	-	-	-	-
ENSG00000145868	FBXO38	0.356143909	1.58E-06	-0.035514793	0.803716	0.052380595	0.658075	-0.384951353	8.49E-11	-0.289200295	4.78E-07	0.090352499	0.431351	C5	-	Y	-	-	Y	-	-
ENSG00000145882	PCYOX1L	-0.683832289	4.61E-18	-0.162451555	0.113958	-0.109040118	0.352405	0.527945085	5.33E-10	0.589596821	2.53E-11	0.056224136	0.776003	C4	-	Y	-	-	Y	Y	-
ENSG00000145911	N4BP3	-0.52790025	0.017552	-0.325186068	0.227516	-0.025779477	0.933967	0.212072182	0.50866	0.517039672	0.029226	0.301539437	0.410703	C4	-	Y	-	-	-	Y	-
ENSG00000145920	CPLX2	-0.300713907	9.56E-09	-0.152996337	0.016994	-0.186385858	0.014392	0.154394645	0.002716	0.128843164	0.046798	-0.03037076	0.841305	C4	-	Y	-	-	-	-	-
ENSG00000145979	TBC1D7	0.263127794	0.02978	0.335534016	0.011111	0.152654956	0.295993	0.078606815	0.604078	-0.09582347	0.506004	-0.180060302	0.317737	C4	-	-	Y	-	-	-	-
ENSG00000145982	FARS2	-0.703658278	4.16E-06	-0.388298315	0.016645	-0.370319684	0.018372	0.323156796	0.088935	0.347957855	0.060148	0.020389861	0.961428	C3	-	Y	Y	Y	-	-	-
ENSG00000145990	GFOD1	0.98232965	8.54E-07	0.588968199	0.016046	0.514674137	0.029519	-0.387157098	0.073031	-0.453060155	0.02796	-0.071439266	0.887295	C5	-	Y	Y	Y	-	Y	-
ENSG00000145996	CDKAL1	-0.342456108	7.25E-05	-0.213942624	0.049412	-0.279549751	0.005767	0.135166938	0.202519	0.077928459	0.503893	-0.062568499	0.757257	C4	-	Y	-	-	-	-	-
ENSG00000146006	LRRTM2	0.35185119	0.00418	0.314077376	0.042452	0.448046592	0.000803	-0.0315818	0.860608	0.110510085	0.444147	0.136858405	0.554373	C1	-	Y	Y	Y	-	-	-
ENSG00000146013	GFRA3	1.089289363	0.045259	0.436945735	NA	-0.324953425	0.764146	-0.648331689	0.337658	-1.402681147	0.022494	-0.759340794	NA	C5	-	Y	-	-	-	Y	-
ENSG00000146021	KLHL3	-0.818591173	1.58E-05	-0.338765043	0.07129	-0.110525212	0.662862	0.487391434	0.027775	0.722665651	0.000885	0.23045133	0.472053	C3	-	Y	-	-	Y	Y	-
ENSG00000146054	TRIM7	-0.802261446	0.115391	-0.726782516	0.257028	-1.312689218	0.04053	0.07778787	0.930573	-0.496914444	0.555657	-0.577682368	NA	C3	-	-	-	Y	-	-	-
ENSG00000146072	TNFRSF21	0.344812968	0.008967	-0.097445368	0.70426	0.044985584	0.831744	-0.436074876	0.007104	-0.285106638	0.044469	0.14553282	0.638347	C5	-	Y	-	-	Y	-	-
ENSG00000146094	DOK3	-0.834836703	6.63E-07	-0.317763041	0.108544	-0.31260213	0.124968	0.523595272	0.00454	0.536441348	0.005525	0.007697916	0.988432	C4	-	Y	-	-	Y	Y	-
ENSG00000146112	PPP1R18	0.383613729	1.68E-09	0.185853555	0.020165	0.326120641	2.62E-06	-0.191053751	0.000938	-0.042439679	0.550774	0.143098529	0.068843	C5	-	Y	-	Y	-	-	-
ENSG00000146151	HMGCLL1	-3.002792842	4.64E-05	0.179341423	0.834292	-0.147540924	0.849033	3.199267587	7.71E-05	2.869427937	0.000512	-0.329895806	NA	C3	-	Y	-	-	Y	Y	-
ENSG00000146216	TTBK1	0.591378515	2.49E-16	0.172466088	0.081213	0.274007876	0.001098	-0.412690402	7.85E-18	-0.302889049	9.96E-11	0.104335475	0.20546	C5	-	Y	-	-	Y	Y	-
ENSG00000146242	TPBG	0.405297505	0.014718	0.548649189	0.011467	0.508393331	0.012202	0.150009527	0.518709	0.11820125	0.602927	-0.038763808	0.943834	C1	-	Y	Y	Y	-	-	-
ENSG00000146247	PHIP	0.446121194	1.11E-08	0.252602836	0.011903	0.218821338	0.023795	-0.186925711	0.002132	-0.212688538	0.000209	-0.031119373	0.84742	C1	-	Y	-	-	-	-	-
ENSG00000146250	PRSS35	-1.332539826	3.60E-05	-0.943610163	0.007842	-1.091314595	0.001221	0.398176609	0.421342	0.255861525	0.636773	-0.147324756	NA	C3	-	Y	Y	Y	-	-	-
ENSG00000146281	PM20D2	0.385075551	1.81E-11	0.156388571	0.031406	0.240044012	9.59E-05	-0.222151047	8.87E-05	-0.130344395	0.022545	0.086376359	0.338628	C5	-	Y	-	-	-	-	-
ENSG00000146352	CLVS2	-0.665412751	3.06E-05	-0.374548743	0.042168	-0.007921545	0.97754	0.298169924	0.134458	0.672045226	0.000124	0.368777251	0.130663	C4	-	Y	Y	-	-	Y	-
ENSG00000146386	ABRACL	-0.466203077	0.023844	-0.109067405	0.745814	0.001298501	0.99652	0.363657659	0.162008	0.482185802	0.03795	0.112987419	0.824527	C4	-	Y	-	-	-	Y	-
ENSG00000146410	MTFR2	-0.221881525	0.222956	-0.358749566	0.111758	-0.461235804	0.009568	-0.131455642	0.598705	-0.224939813	0.267812	-0.099129218	0.827426	C4	-	-	-	Y	-	-	-
ENSG00000146411	SLC2A12	-2.123502939	0.014036	-0.939491913	NA	0.034992446	0.972913	1.191744751	0.323022	2.173658715	0.017206	0.97765892	NA	C2	-	Y	-	-	-	Y	-
ENSG00000146414	SHPRH	0.627369655	1.32E-09	0.449779294	0.000191	0.444806957	0.000115	-0.172005191	0.013262	-0.168611766	0.013768	-0.002009529	0.992545	C1	Y	Y	Y	Y	-	-	-
ENSG00000146416	AIG1	-0.367740137	8.84E-07	-0.173209235	0.047531	-0.071206025	0.458493	0.20128748	0.018273	0.311157449	4.39E-05	0.104976098	0.392995	C4	-	Y	-	-	-	Y	-
ENSG00000146426	TIAM2	-0.310711921	0.000586	-0.277022632	0.024639	-0.06699736	0.614841	0.040322181	0.799581	0.258378063	0.010355	0.213129418	0.197263	C4	-	Y	-	-	-	-	-
ENSG00000146463	ZMYM4	0.333463664	9.29E-07	0.180421053	0.046468	0.204929726	0.009002	-0.146649884	0.005282	-0.114207735	0.012473	0.02721415	0.827929	C5	-	Y	-	-	-	-	-
ENSG00000146469	VIP	-0.485072059	1.04E-09	-0.120527995	0.366101	-0.190812798	0.149608	0.371013254	7.62E-05	0.309462288	0.003808	-0.068293144	0.790777	C4	-	Y	-	-	Y	Y	-
ENSG00000146476	ARMT1	0.422687995	4.61E-05	0.088115158	0.589455	0.233569648	0.027153	-0.327349862	0.005859	-0.174398787	0.124742	0.148547483	0.40217	C5	-	Y	-	-	Y	-	-
ENSG00000146540	C7orf50	-0.402003148	7.38E-05	-0.170605733	0.201267	-0.20172378	0.095678	0.237785532	0.001021	0.215504958	0.002634	-0.027566685	0.872658	C4	-	Y	-	-	-	-	-
ENSG00000146587	RBAK	0.274183838	0.000599	-0.069515838	0.567473	-0.045098555	0.697502	-0.337480437	2.36E-08	-0.305175945	4.24E-08	0.027140811	0.847311	C5	-	-	-	-	Y	Y	-
ENSG00000146592	CREB5	0.57857801	2.57E-09	0.02899507	0.868255	0.005330293	0.972937	-0.543267344	2.64E-09	-0.559035505	2.95E-12	-0.021208183	0.926363	C5	-	Y	-	-	Y	Y	-
ENSG00000146670	CDCA5	-0.097371303	0.106624	-0.103146387	0.201505	-0.304226613	4.80E-06	0.000740959	0.992382	-0.192309632	0.001085	-0.198120903	0.019202	C4	-	-	-	Y	-	-	-
ENSG00000146674	IGFBP3	-0.194527728	0.012481	0.878172981	3.15E-33	0.60060096	7.27E-19	1.079251529	4.92E-59	0.809783203	1.75E-40	-0.274648476	0.000799	C2	-	-	Y	Y	Y	Y	-
ENSG00000146676	PURB	0.431273232	3.62E-07	0.038583774	0.809283	0.04099318	0.753891	-0.386152372	0.001396	-0.375768553	0.000415	0.005220387	0.982229	C5	Y	Y	-	-	Y	Y	-
ENSG00000146733	PSPH	-0.821202957	1.35E-27	-0.479018674	2.72E-08	-0.193885811	0.021932	0.348631568	6.16E-05	0.642225572	1.44E-18	0.288249562	0.002503	C3	-	Y	Y	-	Y	Y	-
ENSG00000146757	ZNF92	0.10310328	0.185203	-0.321646978	0.000103	-0.240282795	0.008972	-0.418122027	2.36E-10	-0.328878676	4.27E-06	0.084362077	0.586137	C4	-	-	Y	-	Y	Y	-
ENSG00000146826	MAP11	0.332787172	4.36E-05	0.163792717	0.176641	0.198842421	0.060937	-0.161961912	0.074428	-0.119341288	0.185283	0.037887299	0.862969	C5	-	Y	-	-	-	-	-
ENSG00000146833	TRIM4	0.510215744	2.18E-07	0.007880067	0.971342	0.232837235	0.061738	-0.496150862	2.44E-07	-0.26260959	0.007084	0.227986146	0.121977	C5	-	Y	-	-	Y	-	-
ENSG00000146834	MEPCE	-0.138027652	0.018043	-0.379794468	8.04E-11	-0.353277955	7.72E-10	-0.235280466	8.32E-05	-0.200591068	0.000652	0.029488227	0.838766	C4	-	-	Y	Y	-	-	-
ENSG00000146842	TMEM209	-0.349648937	6.26E-09	-0.231822378	0.000655	-0.296127151	2.70E-06	0.124270178	0.076247	0.068264714	0.36818	-0.061345321	0.601463	C4	-	Y	-	-	-	-	-
ENSG00000146856	AGBL3	0.032911471	0.912875	0.449136385	0.052102	0.101228198	0.737478	0.422928891	0.048987	0.083031488	0.772487	-0.344924259	0.244879	C4	-	-	-	-	Y	-	-
ENSG00000146858	ZC3HAV1L	0.527584252	5.44E-06	0.261802691	0.094255	0.157483288	0.384663	-0.260122336	0.005524	-0.355627552	0.000404	-0.101335581	0.636866	C1	-	Y	-	-	-	Y	-
ENSG00000146859	TMEM140	0.342826599	0.003137	0.263458111	0.092081	0.185380525	0.134063	-0.072863729	0.670011	-0.142659633	0.245975	-0.075405461	0.768872	C1	-	Y	-	-	-	-	-
ENSG00000146950	SHROOM2	-1.407866757	7.80E-06	-0.495333775	0.149108	-0.091058389	0.841652	0.921120969	0.01776	1.332707694	0.000294	0.406546748	NA	C2	-	Y	-	-	Y	Y	-
ENSG00000146963	LUC7L2	-0.402839163	1.24E-11	-0.194883227	0.012298	-0.286994016	2.28E-05	0.214679456	0.000364	0.13078614	0.033542	-0.089302233	0.395182	C4	-	Y	-	-	-	-	-
ENSG00000147003	CLTRN	-1.327937397	0.008202	-0.390475779	0.577021	-0.743729878	0.210782	0.942000291	0.147303	0.598466137	0.416542	-0.347886958	NA	C4	-	Y	-	-	-	-	-
ENSG00000147117	ZNF157	0.06957438	0.868023	-0.734395254	0.057445	-0.386417882	0.322082	-0.798548452	0.015585	-0.442046433	0.18593	0.350989014	NA	C5	-	-	-	-	Y	-	-
ENSG00000147118	ZNF182	0.385173225	6.38E-06	-0.155593839	0.298847	0.08391375	0.487885	-0.534240887	3.26E-07	-0.286553108	0.001181	0.24227271	0.120898	C5	-	Y	-	-	Y	-	-

ENSG00000147121	KRBOX4	-0.257233688	0.04234	-0.377387574	0.005824	-0.324195053	0.015603	-0.114031944	0.462741	-0.052355733	0.760446	0.055948079	0.851461	C4	-	-	Y	Y	-	-	-
ENSG00000147124	ZNF41	0.381377802	9.05E-05	0.06827239	0.709997	0.185865419	0.127097	-0.30649691	0.007555	-0.180775031	0.098006	0.1201709	0.567296	C5	-	Y	-	-	Y	-	-
ENSG00000147133	TAF1	0.327149627	5.26E-06	0.181142148	0.038132	0.129861627	0.144762	-0.139771979	0.039753	-0.182645308	0.003856	-0.048251781	0.716977	C5	-	Y	-	-	-	-	-
ENSG00000147155	EBP	-0.358075854	0.012359	-0.175697756	0.322825	-0.200223998	0.252857	0.189337976	0.176761	0.172576228	0.25373	-0.022129328	0.948042	C4	-	Y	-	-	-	-	-
ENSG00000147202	DIAPH2	-0.533938524	0.009261	-0.186494975	0.436249	-0.147590995	0.535571	0.35388921	0.107355	0.400789875	0.060703	0.041843052	0.927911	C4	-	Y	-	-	-	-	-
ENSG00000147394	ZNF185	-0.654434342	0.020975	-1.074099668	0.001229	-0.916662338	0.005715	-0.41329734	0.320029	-0.247196224	0.581574	0.161567904	NA	C3	-	Y	Y	Y	-	-	-
ENSG00000147400	CETN2	-0.321294173	0.000921	-0.183523009	0.189767	-0.178055594	0.118791	0.144146387	0.256462	0.158017359	0.140772	0.008521871	0.978268	C4	-	Y	-	-	-	-	-
ENSG00000147421	HMBOX1	0.375062043	0.001333	0.241765764	0.074166	0.346066166	0.000727	-0.12614968	0.54941	-0.014566524	0.919505	0.107312559	0.53558	C1	-	Y	-	Y	-	-	-
ENSG00000147437	GNRH1	0.135305946	0.739663	-0.723352288	0.064388	0.102770306	0.824282	-0.851947136	0.012209	-0.017730411	0.965042	0.829325406	NA	C5	-	-	-	-	Y	-	-
ENSG00000147454	SLC25A37	0.395556829	1.26E-10	0.308941459	1.90E-05	0.228976938	0.001238	-0.080483806	0.249253	-0.151890433	0.011451	-0.076776941	0.47034	C1	Y	Y	Y	-	-	-	-
ENSG00000147465	STAR	-1.359307977	4.02E-05	-0.923121269	0.061652	-0.787598291	0.030532	0.439777887	0.452992	0.587109826	0.176312	0.141190656	NA	C3	-	Y	-	Y	-	-	-
ENSG00000147475	ERLIN2	-0.39384705	4.68E-09	0.028105514	0.81065	-0.031236652	0.757153	0.428412307	5.89E-10	0.377361534	1.92E-08	-0.05641513	0.666588	C4	-	Y	-	-	Y	Y	-
ENSG00000147536	GINS4	-0.476971808	9.18E-06	-0.104626473	0.52298	-0.253707932	0.053784	0.378724786	0.001012	0.237690132	0.060064	-0.14628256	0.472616	C4	-	Y	-	-	Y	-	-
ENSG00000147548	NSD3	0.44465102	2.36E-11	0.2432329	0.002953	0.227908745	0.002412	-0.195002045	0.000541	-0.202186223	5.73E-05	-0.012533757	0.937494	C5	-	Y	-	-	-	-	-
ENSG00000147642	SYBU	-0.816521349	0.00908	-0.862229511	0.011455	-0.590619681	0.07377	-0.039233254	0.938843	0.239872238	0.546009	0.274878903	NA	C3	-	Y	Y	-	-	-	-
ENSG00000147654	EBAG9	-0.440681217	1.05E-06	-0.331493037	0.001367	-0.364752142	0.003834	0.115530271	0.338115	0.090604379	0.55152	-0.030491312	0.927911	C4	-	Y	Y	Y	-	-	-
ENSG00000147679	UTP23	0.185703108	0.014646	-0.139161491	0.20191	-0.159674534	0.09232	-0.318396321	3.93E-05	-0.330614204	5.65E-06	-0.017709417	0.940608	C5	-	-	-	-	Y	Y	-
ENSG00000147799	ARHGAP39	0.749108577	1.95E-12	0.452730107	0.000347	0.505551784	5.16E-06	-0.288927228	0.003568	-0.228457742	0.014471	0.054521484	0.78739	C5	-	Y	Y	Y	-	-	-
ENSG00000147804	SLC39A4	-0.379624095	0.004077	-0.029908653	0.908112	0.168417918	0.322519	0.356631692	0.023027	0.562812411	2.35E-05	0.20110091	0.420113	C4	-	Y	-	-	Y	Y	-
ENSG00000147862	NFIB	-0.301271138	1.03E-05	-0.277129648	0.000372	-0.244909661	0.001057	0.030597118	0.732309	0.070942145	0.32667	0.035031782	0.818106	C4	-	Y	-	-	-	-	-
ENSG00000147872	PLIN2	-0.349785053	2.14E-06	-0.17527622	0.049986	-0.280806515	0.001369	0.180975899	0.024674	0.083886862	0.39859	-0.102595292	0.463249	C4	-	Y	-	-	-	-	-
ENSG00000147905	ZCCHC7	0.408290363	2.42E-05	0.208662454	0.131038	0.199618636	0.081742	-0.194127376	0.042025	-0.193857544	0.015814	-0.005133192	0.982984	C5	-	Y	-	-	-	-	-
ENSG00000147955	SIGMAR1	-0.417625161	1.82E-09	-0.059363305	0.627932	-0.094583428	0.364469	0.364634447	6.99E-08	0.337689571	3.60E-07	-0.032278833	0.858374	C4	Y	Y	-	-	Y	Y	-
ENSG00000148053	NTRK2	-0.191564055	0.577813	0.800513533	0.004099	0.577045502	0.045798	0.997681486	4.04E-05	0.782595939	0.002003	-0.219892817	0.606986	C2	-	-	Y	Y	Y	Y	-
ENSG00000148057	IDNK	-0.902622783	0.000441	-0.353727063	0.294389	-0.630170469	0.01894	0.556268116	0.088156	0.287718034	0.413341	-0.27349312	NA	C3	-	Y	-	Y	-	-	-
ENSG00000148090	AUH	0.449207664	2.35E-05	0.388020016	0.001943	0.264913712	0.037621	-0.054843024	0.706772	-0.169530265	0.154125	-0.120230585	0.537085	C1	Y	Y	Y	-	-	-	-
ENSG00000148154	UGCG	0.573984625	1.95E-10	0.090300787	0.55642	0.192206075	0.09222	-0.477250042	1.83E-10	-0.367718058	2.50E-07	0.104329515	0.45687	C5	-	Y	-	-	Y	Y	-
ENSG00000148158	SNX30	0.588435273	5.55E-10	0.345527274	0.000388	0.16773397	0.143119	-0.236660942	0.01625	-0.405981445	1.97E-05	-0.174821216	0.191653	C5	-	Y	Y	-	-	Y	-
ENSG00000148175	STOM	-1.639226217	1.47E-09	-0.900706074	4.53E-05	-0.965226149	1.13E-05	0.746052669	0.033133	0.688147946	0.057125	-0.062256473	NA	C3	-	Y	Y	Y	Y	-	-
ENSG00000148180	GSN	-0.528932541	2.01E-05	-0.44325377	0.000602	-0.30328131	0.032655	0.092994899	0.619518	0.240394457	0.140437	0.142161875	0.577482	C4	-	Y	Y	Y	-	-	-
ENSG00000148200	NR6A1	0.204277884	0.27255	0.486516081	0.006693	0.172527618	0.398305	0.28815971	0.095525	-0.017279919	0.940225	-0.311583206	0.150527	C4	-	-	Y	-	-	-	-
ENSG00000148218	ALAD	0.373967853	7.39E-06	0.134915717	0.271586	0.187375069	0.075285	-0.232573109	0.007172	-0.171571303	0.051965	0.055369497	0.766547	C5	-	Y	-	-	-	-	-
ENSG00000148225	WDR31	-1.579922897	2.18E-12	-0.441931093	0.056734	-0.429253316	0.067711	1.144975555	2.15E-06	1.164720298	3.21E-06	0.015236636	0.979503	C3	-	Y	-	-	Y	Y	-
ENSG00000148229	POLE3	-0.10496478	0.06382	-0.191469888	0.001554	-0.380840639	4.07E-12	-0.079897326	0.157836	-0.261299466	1.17E-07	-0.186641274	0.004047	C4	-	-	-	Y	-	-	-
ENSG00000148290	SURF1	-0.347866069	3.53E-06	-0.130481958	0.214881	-0.125114874	0.195727	0.223886201	0.002099	0.237771517	0.00057	0.00842769	0.967287	C4	-	Y	-	-	-	-	-
ENSG00000148339	SLC25A25	0.248923968	0.004147	-0.093407332	0.434084	-0.029208187	0.827969	-0.335990274	4.72E-05	-0.263486316	0.001466	0.066926213	0.676618	C5	-	-	-	-	Y	-	-
ENSG00000148343	MIGA2	0.316776767	0.000496	0.149015766	0.198127	0.113151374	0.32547	-0.161113316	0.088164	-0.18869413	0.035529	-0.032909187	0.869517	C5	-	Y	-	-	-	-	-
ENSG00000148344	PTGES	-0.697256808	0.023671	-0.293646287	0.422498	-0.191931618	0.625912	0.408841879	0.234068	0.519423873	0.124652	0.104691068	NA	C4	-	Y	-	-	-	-	-
ENSG00000148357	HMCN2	-0.003529899	0.992789	-0.38548189	0.325187	-0.701110879	0.033039	-0.376214586	0.27878	-0.68384382	0.029433	-0.313057677	NA	C5	-	-	-	Y	-	Y	-
ENSG00000148384	INPP5E	0.314183044	0.000119	0.212257	0.021985	0.178214337	0.049078	-0.095605566	0.31278	-0.121301823	0.172573	-0.031271569	0.864366	C5	-	Y	-	-	-	-	-
ENSG00000148399	DPH7	0.245380125	0.005272	-0.019094125	0.909007	-0.0895697	0.475629	-0.257578868	0.000529	-0.320191295	6.53E-06	-0.067885314	0.658764	C5	-	-	-	-	-	Y	-
ENSG00000148400	NOTCH1	-0.642621784	0.010745	-0.096978753	0.802255	0.241262571	0.412206	0.552790831	0.048557	0.898522022	0.000362	0.340956051	0.364363	C4	-	Y	-	-	Y	Y	-
ENSG00000148408	CACNA1B	-0.330448297	7.47E-06	-0.132371814	0.327065	0.081999002	0.395238	0.20519311	0.065451	0.427187546	6.18E-09	0.217390695	0.126214	C4	-	Y	-	-	-	Y	-
ENSG00000148411	NACC2	-0.092859002	0.660322	0.278729742	0.141642	0.161720437	0.447418	0.378384016	0.02582	0.269234942	0.147466	-0.114176858	0.712857	C4	-	-	-	-	Y	-	-
ENSG00000148450	MSRB2	-0.444515158	5.02E-07	-0.110431336	0.408065	-0.071972718	0.617972	0.341355345	5.44E-05	0.387295205	8.32E-06	0.041217179	0.849728	C4	-	Y	-	-	Y	Y	-
ENSG00000148484	RSU1	0.631713754	3.19E-13	0.370814159	6.04E-05	0.215422747	0.036036	-0.25427967	0.002739	-0.401363555	1.03E-06	-0.152317466	0.209306	C5	-	Y	Y	-	-	Y	-
ENSG00000148516	ZEB1	0.344459012	6.95E-08	0.297336773	9.21E-05	0.316112552	2.56E-06	-0.040682925	0.617189	-0.013861039	0.867733	0.021621662	0.892177	C5	-	Y	-	Y	-	-	-
ENSG00000148600	CDHR1	-0.090989806	0.854182	-1.052608791	0.0228	-0.247419179	0.652806	-0.955244925	0.026376	-0.141684064	0.788871	0.807191167	NA	C5	-	-	Y	-	Y	-	-
ENSG00000148634	HERC4	0.44753629	1.47E-07	0.280704059	0.005787	0.374416235	1.09E-05	-0.160576164	0.042705	-0.058719729	0.477148	0.09648541	0.40755	C5	-	Y	-	Y	-	-	-
ENSG00000148655	LRMDA	-2.235046893	0.020158	-1.305994891	NA	-1.229608018	NA	0.932838341	NA	1.023970137	0.491256	0.08320994	NA	C3	-	Y	-	-	-	-	-
ENSG00000148677	ANKRD1	3.469158501	1.10E-19	3.365452821	3.50E-16	3.614052677	1.37E-21	-0.097851066	0.77284	0.160612545	0.572151	0.253499577	0.553167	C1	-	Y	Y	Y	-	-	-
ENSG00000148737	TCF7L2	-0.826992342	7.44E-06	-0.317608375	0.208754	-0.521979864	0.005609	0.517252952	0.033937	0.319210542	0.181808	-0.202519297	0.610987	C3	-	Y	-	Y	Y	-	-
ENSG00000148814	LRRC27	0.276615151	0.056438	0.403401222	0.013437	0.379860318	0.007442	0.132878043	0.454962	0.11794847	0.467915	-0.020788899	0.956831	C5	-	-	Y	Y	-	-	-
ENSG00000148835	TAF5	0.532466847	3.71E-09	0.075258952	0.640711	0.287963395	0.031109	-0.450795978	1.22E-06	-0.230016098	0.041817	0.215593285	0.220878	C5	-	Y	-	-	Y	-	-
ENSG00000148842	CNNM2	0.166869761	0.180092	0.391140036	2.52E-05	0.239409064	0.015878	0.230740551	0.027046	0.087135915	0.474753	-0.148658381	0.19895	C4	-	-	Y	-	-	-	-
ENSG00000148843	PDCD11	0.548740877	1.70E-27	0.212715653	0.001192	0.204384392	0.000371	-0.329531466	1.82E-17	-0.329887646	2.03E-24	-0.005644039	0.96197	C5	-	Y	-	-	Y	Y	-
ENSG00000148848	ADAM12	-0.492381345	4.13E-15	0.295297558	0.213331	0.14575179	0.15013	0.794894813	4.14E-06	0.652964635	3.08E-25	-0.147192009	0.712155	C4	-	Y	-	-	Y	Y	-
ENSG00000148925	BTBD10	0.393541028	1.86E-06	0.058772175	0.677124	0.216857216	0.031646	-0.328653179	4.12E-05	-0.161947969	0.061522	0.161229905	0.201741	C5	-	Y	-	-	Y	-	-
ENSG00000148926	ADM	-0.816598539	1.51E-12	-0.379611417	0.002735	-0.234376373	0.16247	0.443247059	0.001207	0.596950887	8.29E-05	0.14827006	0.594474	C3	-	Y	Y	-	Y	Y	-
ENSG00000148943	LIN7C	0.247598166	0.007193	-0.135440421	0.224378	-0.056756105	0.665521	-0.376662397	1.24E-05	-0.289548063	0.001195	0.081663491	0.613613	C5	-	-	-	-	Y	-	-
ENSG00000148950	IMMP1L	-0.43557406	0.000262	-0.157236328	0.344806	-0.349875998	0.008601	0.284862972	0.039539	0.100237357	0.542339	-0.189786812	0.368678	C4	-	Y	-	Y	-	-	-
ENSG00000149089	APIP	0.417454336	0.000941	0.090809764	0.61268	-0.016510589	0.943644	-0.320105495	0.003539	-0.41896489	0.000467	-0.104444302	0.632859	C5	Y	Y	-	-	Y	Y	-
ENSG00000149115	TNKS1BP1	-0.290241892	7.09E-06	-0.318908369	0.00024	-0.194473984	0.009734	-0.021853928	0.832843	0.110471388	0.090985	0.127096849	0.270185	C4	-	-	Y	-	-	-	-
ENSG00000149131	SERPING1	-0.452888019	0.032555	-0.652823025	0.008956	-0.602415817	0.00791	-0.191876055	0.544064	-0.134834236	0.66834	0.05112747	0.941862	C4	-	Y	Y	Y	-	-	-
ENSG00000149150	SLC43A1	0.352208049	0.045863	0.193174094	0.494584	0.174767314	0.439755	-0.152878331	0.51666	-0.162598462	0.406444	-0.014754576	0.978047	C5	-	Y	-	-	-	-	-
ENSG00000149177	PTPRJ	0.431474617	0.003598	0.034786119	0.905874	0.228692702	0.217297	-0.390508669	0.018201	-0.188119814	0.265434	0.196680162	0.484596	C5	-	Y	-	-	Y	-	-
ENSG00000149182	ARFGAP2	-0.465094685	4.95E-15	-0.147540251	0.049224	-0.171764896	0.016272	0.324110559	8.42E-08	0.30801744	4.47E-07	-0.021424382	0.891721	C4	-	Y	-	-	Y	Y	-

ENSG00000149212	SESN3	0.634427459	3.94E-14	0.050433866	0.794958	0.37355092	0.000451	-0.577387713	1.40E-09	-0.245872107	0.005836	0.325827135	0.023349	C5	-	Y	-	Y	Y	-	Y
ENSG00000149218	ENDOD1	0.429623041	0.009044	0.506289661	0.002199	0.590582555	0.000332	0.083066913	0.673356	0.175494057	0.329862	0.087288242	0.777633	C1	-	Y	Y	Y	-	-	-
ENSG00000149231	CCDC82	0.471925939	2.10E-18	0.027748757	0.793221	0.03725404	0.689034	-0.437715934	7.48E-19	-0.42005079	5.44E-18	0.012291439	0.939246	C5	-	Y	-	-	Y	Y	-
ENSG00000149256	TENM4	-0.420740243	3.84E-09	0.072824065	0.557624	0.170605782	0.05626	0.499782282	2.31E-21	0.605824847	1.81E-43	0.100677778	0.310589	C4	-	Y	-	-	Y	Y	-
ENSG00000149289	ZC3H12C	-0.304694623	0.027208	-0.130703895	0.484833	-0.306850249	0.048035	0.180227874	0.232979	0.012214564	0.951821	-0.173251022	0.45687	C4	-	Y	-	Y	-	-	-
ENSG00000149292	TTC12	-0.737661665	1.26E-10	-0.129636758	0.409995	-0.230604872	0.073575	0.614462727	7.20E-07	0.521670323	3.08E-05	-0.098090492	0.662255	C4	-	Y	-	-	Y	Y	-
ENSG00000149295	DRD2	-0.33761657	0.046709	-0.174158456	0.383294	-0.178790918	0.361471	0.169498198	0.361928	0.173358339	0.358474	-0.002059969	0.996237	C4	-	Y	-	-	-	-	-
ENSG00000149311	ATM	0.247721679	0.01641	0.302762053	0.006009	0.460754923	7.54E-06	0.061243495	0.565437	0.227413095	0.117139	0.160872699	0.220878	C4	-	-	Y	Y	-	-	-
ENSG00000149403	GRIK4	0.553688819	0.028373	-0.923072177	0.008067	-0.506568106	0.13932	-1.469917031	1.79E-07	-1.045679994	9.57E-05	0.420359071	NA	C5	-	Y	Y	-	Y	Y	-
ENSG00000149476	TKFC	-0.635939945	1.76E-18	-0.200702495	0.038255	-0.084415458	0.39882	0.441606465	9.72E-09	0.566472332	4.79E-17	0.119723817	0.316957	C4	-	Y	-	-	Y	Y	-
ENSG00000149489	ROM1	-0.416913642	0.049614	-0.083137427	0.800502	-0.04050448	0.901282	0.340811455	0.130533	0.391464192	0.071542	0.046016986	0.928882	C4	-	Y	-	-	-	-	-
ENSG00000149527	PLCH2	-0.879880095	1.99E-09	-0.19784963	0.292762	0.271160067	0.05699	0.689324204	3.50E-05	1.165928983	5.43E-17	0.471957916	0.004543	C4	-	Y	-	-	Y	Y	Y
ENSG00000149557	FEZ1	-0.784022485	3.83E-16	-0.586820756	3.27E-09	-0.433242725	4.26E-06	0.20357083	0.08724	0.365478061	0.000358	0.156494234	0.307935	C4	-	Y	Y	Y	-	Y	-
ENSG00000149577	SIDT2	0.108889732	0.371174	0.273591024	0.033782	0.350650137	0.002601	0.171253742	0.172113	0.256575116	0.024579	0.079553467	0.73559	C4	-	-	-	Y	-	-	-
ENSG00000149582	TMEM25	0.715548761	8.88E-14	0.244957003	0.058789	0.388385308	0.000755	-0.463975359	9.85E-07	-0.312624462	0.001403	0.146250148	0.395618	C5	-	Y	-	Y	Y	Y	-
ENSG00000149591	TAGLN	0.081831942	0.634225	0.944574917	3.37E-08	0.907654987	2.38E-10	0.869488178	7.83E-09	0.84149833	2.65E-11	-0.036734966	0.95765	C4	-	-	Y	Y	Y	Y	-
ENSG00000149633	KIAA1755	-0.88050903	0.010769	-0.479857456	0.238598	-0.709844334	0.054647	0.410506269	0.384178	0.185339127	0.736444	-0.229497771	NA	C3	-	Y	-	-	-	-	-
ENSG00000149634	SPATA25	-0.346965849	0.340285	0.187771562	0.669032	0.412413335	0.301224	0.540751097	0.105806	0.773752069	0.024776	0.228029101	NA	C4	-	-	-	-	-	Y	-
ENSG00000149639	SOGA1	0.305646525	5.68E-06	0.329100436	1.00E-05	0.282937181	4.74E-05	0.029657442	0.599842	-0.008315797	0.883539	-0.043259944	0.571938	C1	-	Y	Y	-	-	-	-
ENSG00000149646	CNBD2	-2.092835241	0.00155	-0.759113826	0.279963	-2.201202926	0.006167	1.334110388	0.1175	-0.101788548	0.950222	-1.437103617	NA	C3	-	Y	-	Y	-	-	-
ENSG00000149743	TRPT1	-0.175176928	0.139363	0.058876651	0.744683	0.143248833	0.307055	0.240416005	0.046982	0.333168884	0.003332	0.087436978	0.69654	C4	-	-	-	-	-	Y	-
ENSG00000149809	TM7SF2	-0.578465412	2.03E-07	-0.045891472	0.820136	0.10484466	0.557236	0.539303007	7.33E-06	0.698150053	1.46E-08	0.152876935	0.513633	C4	-	Y	-	-	Y	Y	-
ENSG00000149927	DOC2A	-0.703206858	2.27E-06	-0.423168998	0.02224	-0.090098782	0.695504	0.287603383	0.152522	0.627908237	0.000201	0.334658859	0.1995	C4	-	Y	Y	-	-	Y	-
ENSG00000149929	HIRIP3	-0.323032807	7.03E-07	-0.24218841	0.005488	-0.266900576	0.0015	0.088122887	0.247337	0.070692802	0.371662	-0.021885853	0.912643	C4	-	Y	-	-	-	-	-
ENSG00000149930	TAOK2	0.425525938	4.08E-14	0.072938894	0.425684	0.225970443	0.001493	-0.346234051	1.33E-10	-0.185170168	0.001308	0.155824038	0.07481	C5	-	Y	-	-	Y	-	-
ENSG00000150281	CTF1	-0.774171246	0.031046	-0.207766486	0.718523	0.13551081	0.767993	0.573959987	0.18077	0.924281131	0.004241	0.348077192	NA	C3	-	Y	-	-	-	Y	-
ENSG00000150316	CWC15	0.085695551	0.360625	-0.114191225	0.326091	-0.25156539	0.010797	-0.193152579	0.017181	-0.322477169	4.56E-05	-0.134415101	0.345476	C5	-	-	-	-	-	Y	-
ENSG00000150347	ARID5B	0.410506741	9.56E-05	0.394936582	0.006822	0.472851532	0.000158	-0.009656612	0.956052	0.077072955	0.555627	0.08087858	0.757566	C1	-	Y	Y	Y	-	-	-
ENSG00000150510	FAM124A	0.071277643	0.552383	0.303307361	0.002253	0.23452182	0.020866	0.238576916	0.008783	0.177910963	0.061185	-0.066023908	0.683402	C4	-	-	Y	-	-	-	-
ENSG00000150540	HNMT	-0.915167275	0.210998	0.980833363	0.187203	1.454135185	0.001492	1.907990219	0.006808	2.383241265	6.24E-06	0.479562498	NA	C2	-	-	-	Y	Y	Y	-
ENSG00000150593	PDCD4	-0.405384957	1.20E-12	-0.259821585	0.000824	-0.247097117	0.000114	0.151941785	0.039702	0.173050196	0.00494	0.015604728	0.935133	C4	-	Y	-	-	-	-	-
ENSG00000150627	WDR17	-0.103661539	0.344008	0.039007238	0.851576	0.183252593	0.126884	0.148933992	0.227318	0.301858828	0.000755	0.147109432	0.449228	C4	-	-	-	-	-	Y	-
ENSG00000150636	CCDC102B	-0.67355067	0.001991	-0.079706698	0.809963	0.023756638	0.943368	0.600652848	0.008583	0.712122384	0.001123	0.10640238	0.801162	C4	-	Y	-	-	Y	Y	-
ENSG00000150656	CNDP1	2.252341325	0.132747	-0.211967545	NA	-1.728159334	NA	-2.505012165	NA	-3.963013182	0.026557	-1.556425517	NA	C5	-	-	-	-	-	Y	-
ENSG00000150672	DLG2	-0.644413356	0.000126	-0.313708379	0.153273	-0.111109481	0.66083	0.337902217	0.110059	0.547942032	0.003753	0.204458953	0.551046	C4	-	Y	-	-	-	Y	-
ENSG00000150687	PRSS23	0.037713101	0.869095	0.905328159	4.06E-08	0.926407079	4.57E-10	0.874311113	2.63E-08	0.903594968	4.78E-10	0.023757232	0.957248	C4	-	-	Y	Y	Y	Y	-
ENSG00000150712	MTMR12	0.450610229	4.70E-06	0.230403659	0.04356	0.284862745	0.006752	-0.213931318	0.024674	-0.151351229	0.127491	0.057140431	0.751648	C5	-	Y	-	-	-	-	-
ENSG00000150867	PIP4K2A	0.350579704	0.00068	0.305626219	0.004889	0.162533946	0.149639	-0.038799749	0.78346	-0.173306473	0.106741	-0.140564558	0.339144	C1	-	Y	Y	-	-	-	-
ENSG00000150873	C2orf50	-2.797391075	0.005379	-0.990735686	NA	-0.585351083	0.587286	1.82516978	NA	2.225709474	0.08743	0.398939276	NA	C3	-	Y	-	-	-	-	-
ENSG00000150938	CRIM1	0.495585748	0.405241	1.868534897	1.33E-05	1.95300109	7.43E-06	1.380266627	0.000529	1.471762415	0.000315	0.087254187	NA	C2	-	-	Y	Y	Y	Y	-
ENSG00000150961	SEC24D	0.522671924	1.62E-19	0.26763118	0.000191	0.201149962	0.00528	-0.248206549	1.72E-06	-0.306634994	1.12E-09	-0.063791631	0.54249	C5	-	Y	-	-	-	Y	-
ENSG00000150990	DHX37	0.363191773	3.25E-06	0.208404737	0.063258	0.074993003	0.59038	-0.147755172	0.110532	-0.273634578	0.001799	-0.130110879	0.448101	C5	-	Y	-	-	-	-	-
ENSG00000150991	UBC	-0.741032859	6.43E-40	-0.259112265	0.000585	-0.280402549	1.04E-05	0.4882447	1.99E-29	0.475600098	1.60E-53	-0.017904785	0.878881	C4	-	Y	-	-	Y	Y	-
ENSG00000151006	PRSS53	-0.318292809	0.025619	0.063107658	0.748119	-0.058664192	0.769885	0.388065479	0.005103	0.27445839	0.078767	-0.118900559	0.613613	C2	-	Y	-	-	Y	-	-
ENSG00000151012	SLC7A11	-2.918683942	1.59E-46	-2.232275929	2.10E-05	-1.525432875	6.52E-14	0.695012069	0.145436	1.407838568	4.21E-06	0.713358454	0.47626	C3	-	Y	Y	Y	-	Y	-
ENSG00000151014	NOCT	0.431709106	0.016906	0.145815262	0.584419	0.125034885	0.684161	-0.279352006	0.156158	-0.292104036	0.197312	-0.018164453	0.975773	C5	-	Y	-	-	-	-	-
ENSG00000151090	THRB	-1.441229237	0.002487	-0.788816638	0.162357	-1.093941518	0.037055	0.662896511	0.323582	0.361349436	0.644364	-0.306509661	NA	C3	-	Y	-	Y	-	-	-
ENSG00000151092	NGLY1	-0.046523376	0.741697	0.07934133	0.615872	0.333144918	0.004547	0.132445038	0.242262	0.394588206	7.11E-05	0.25680052	0.049474	C4	-	-	-	Y	-	Y	-
ENSG00000151136	BTBD11	0.395266881	0.068487	-0.141003991	0.691295	-0.009063578	0.981108	-0.529625554	0.022044	-0.389519915	0.093944	0.135113384	0.785648	C5	-	-	-	-	Y	-	-
ENSG00000151148	UBE3B	-0.24169341	4.75E-05	0.022728973	0.826983	0.054725675	0.471315	0.270854541	7.02E-06	0.31110711	1.21E-08	0.034951209	0.777633	C4	-	-	-	-	-	Y	-
ENSG00000151150	ANK3	-0.480015317	0.037762	-0.205971831	0.480139	-0.330438487	0.174607	0.280552677	0.319053	0.164040632	0.589445	-0.121710141	0.796431	C2	-	Y	-	-	-	-	-
ENSG00000151151	IPMK	0.445297418	1.50E-08	0.099416899	0.462255	0.097845404	0.367511	-0.339606543	4.37E-05	-0.332829723	2.76E-06	0.001339651	0.996048	C5	-	Y	-	-	Y	Y	-
ENSG00000151164	RAD9B	-1.105414928	0.046709	-0.307879934	0.689634	-0.507363057	0.412321	0.802490703	0.250207	0.613762594	0.384993	-0.195001235	NA	C4	-	Y	-	-	-	-	-
ENSG00000151176	PLBD2	-0.343348633	5.43E-05	-0.080800833	0.543864	-0.038641623	0.760007	0.269071267	0.005249	0.319306929	0.000217	0.045013729	0.825277	C4	-	Y	-	-	-	Y	-
ENSG00000151208	DLG5	0.304368584	2.08E-05	0.302520599	8.65E-06	0.246839805	0.000462	0.004575326	0.956958	-0.043176032	0.541723	-0.05307252	0.542536	C4	-	Y	Y	-	-	-	-
ENSG00000151229	SLC2A13	0.377258015	5.43E-05	0.260685372	0.0203	0.219845196	0.051082	-0.110099254	0.278879	-0.142767748	0.144213	-0.038073764	0.855994	C1	-	Y	-	-	-	-	-
ENSG00000151233	GXYLT1	0.302567349	0.001151	0.051348942	0.786031	0.092431366	0.467179	-0.245167321	0.008577	-0.195766473	0.005407	0.044208461	0.838445	C5	-	Y	-	-	-	-	-
ENSG00000151239	TWF1	0.323374573	3.65E-09	0.229297837	0.000842	0.096686307	0.247569	-0.08787894	0.158561	-0.211715196	0.000216	-0.129662421	0.160902	C5	-	Y	-	-	-	-	-
ENSG00000151240	DIP2C	0.484617748	8.97E-11	0.069777626	0.549404	0.152054517	0.123998	-0.40864795	7.94E-12	-0.318204114	1.05E-06	0.084977535	0.474909	C5	-	Y	-	-	Y	Y	-
ENSG00000151304	SRFBP1	0.218353	0.110643	0.02825551	0.906434	-0.147885388	0.413354	-0.183943278	0.137247	-0.352082325	0.002045	-0.173556732	0.39019	C5	-	-	-	-	-	Y	-
ENSG00000151320	AKAP6	-0.058456108	0.612116	-0.30454676	0.013937	-0.074936846	0.596827	-0.239711683	0.006396	-0.001597804	0.987214	0.232465318	0.076449	C4	-	-	Y	-	-	-	-
ENSG00000151322	NPAS3	-0.38015885	0.828988	1.639800211	NA	1.212492487	NA	2.023837624	0.035592	1.609409701	0.183779	-0.420153821	NA	C2	-	-	-	-	Y	-	-
ENSG00000151353	TMEM18	-0.357810026	8.52E-08	-0.097485747	0.309682	-0.329493289	8.07E-06	0.266924676	3.43E-05	0.043277524	0.620808	-0.229084825	0.004557	C4	-	Y	-	Y	-	-	-
ENSG00000151388	ADAMTS12	-2.133457873	0.00387	-0.581642817	0.478192	-0.175269191	0.83496	1.556210203	0.100894	1.970399706	0.016787	0.408847595	NA	C3	-	Y	-	-	-	Y	-
ENSG00000151413	NUBPL	-0.390553976	0.004408	-0.109792534	0.554736	-0.270796165	0.094937	0.287400155	0.041702	0.134903991	0.446347	-0.157986264	0.500943	C4	-	Y	-	-	-	-	-
ENSG00000151414	NEK7	0.60971606	3.99E-18	0.33928603	0.000146	0.384140516	1.14E-06	-0.264034532	0.000291	-0.210778191	0.001933	0.047860372	0.766547	C5	-	Y	Y	Y	-	-	-
ENSG00000151470	C4orf33	-0.439201182	0.04563	-0.221483406	0.449137	-0.087887392	0.774426	0.224998702	0.437265	0.365858789	0.145702	0.135919813	0.775391	C4	-	Y	-	-	-	-	-

ENSG00000151474	FRMD4A	-0.779846542	3.79E-09	0.006079849	0.980345	-0.01205331	0.954105	0.792194605	2.59E-10	0.782138359	4.24E-12	-0.015190788	0.960084	C2	-	Y	-	-	Y	Y	-
ENSG00000151490	PTPRO	0.291980854	0.003163	0.31325216	0.008415	0.267363857	0.026006	0.027452224	0.843543	-0.009745253	0.947078	-0.042908398	0.862969	C4	-	-	Y	-	-	-	-
ENSG00000151503	NCAPD3	0.391408774	1.53E-14	0.287433945	4.35E-07	0.169881893	0.006661	-0.097753058	0.031171	-0.207113815	1.18E-06	-0.114716056	0.077493	C1	-	Y	-	-	-	-	-
ENSG00000151532	VTI1A	0.356796115	0.001423	0.282655688	0.018777	0.223861334	0.081384	-0.067729818	0.55554	-0.118535116	0.28256	-0.05611228	0.768872	C1	-	Y	-	-	-	-	-
ENSG00000151552	QDPR	-0.585796977	1.34E-08	-0.173207857	0.212291	-0.197818033	0.115602	0.419146249	3.81E-06	0.403100998	7.03E-06	-0.021460892	0.929329	C4	-	Y	-	-	Y	Y	-
ENSG00000151553	FAM160B1	0.401341947	2.03E-07	0.318561136	0.001331	0.236712914	0.027337	-0.077094585	0.401861	-0.149898105	0.084421	-0.0791124	0.652484	C1	-	Y	Y	-	-	-	-
ENSG00000151576	QTRT2	0.358005432	2.33E-05	-0.034025672	0.837244	0.065934159	0.62192	-0.384787195	3.73E-08	-0.277759968	5.76E-05	0.101965823	0.454661	C5	-	Y	-	-	Y	-	-
ENSG00000151611	MMAA	0.47234162	0.011789	0.230957123	0.379627	0.532020216	0.009146	-0.234999629	0.264189	0.074002395	0.76028	0.303853692	0.276801	C1	-	Y	-	Y	-	-	-
ENSG00000151617	EDNRA	-0.953812458	2.85E-12	0.099196497	0.615259	-0.274686311	0.109355	1.0599594	7.89E-14	0.693537461	2.43E-05	-0.371787003	0.067356	C2	-	Y	-	-	Y	Y	-
ENSG00000151623	NR3C2	-3.885485895	0.012177	-1.17316719	NA	-1.451536698	NA	2.747707926	NA	2.445961826	NA	-0.299468047	NA	C3	-	Y	-	-	-	-	-
ENSG00000151665	PIGF	-0.100573721	0.316408	-0.044742124	0.759944	0.300379107	0.000531	0.062176139	0.583435	0.41565815	9.43E-08	0.34838804	0.000189	C4	-	-	-	Y	-	Y	Y
ENSG00000151693	ASAP2	0.352846221	2.60E-05	0.366638291	0.000109	0.28143915	0.002295	0.019645879	0.813976	-0.057069356	0.390884	-0.082105683	0.41736	C1	-	Y	Y	-	-	-	-
ENSG00000151718	WWC2	0.423535354	0.00045	0.156659542	0.375298	0.119871505	0.501373	-0.260515565	0.011627	-0.289255264	0.00526	-0.034196976	0.90173	C1	-	Y	-	-	-	-	-
ENSG00000151725	CENPU	-0.813945163	3.43E-50	-0.323771804	2.89E-07	-0.550300522	2.98E-17	0.496885942	8.19E-25	0.278366013	1.02E-06	-0.223663966	0.001687	C4	-	Y	Y	Y	Y	-	-
ENSG00000151746	BICD1	0.489185555	5.25E-11	0.069583525	0.579738	0.130207923	0.198115	-0.412951921	7.82E-10	-0.344437311	2.33E-07	0.063256923	0.668504	C5	-	Y	-	-	Y	Y	-
ENSG00000151748	SAV1	0.178030525	0.036373	-0.131184558	0.241435	0.028258121	0.837479	-0.302762488	0.000255	-0.134927344	0.14195	0.162376019	0.200382	C5	-	-	-	-	Y	-	-
ENSG00000151773	CCDC122	-0.828520704	0.002063	-0.213650639	0.556046	-0.133289969	0.717043	0.621819833	0.027553	0.709870215	0.010613	0.08336875	0.891721	C4	-	Y	-	-	Y	Y	-
ENSG00000151778	SERP2	-0.270587364	0.167167	0.109216918	0.665485	-0.052889642	0.841919	0.38618118	0.024935	0.232599015	0.225745	-0.15950687	0.563457	C4	-	-	-	-	Y	-	-
ENSG00000151779	NBAS	-0.219703175	0.002575	0.029171712	0.818748	0.131051241	0.122359	0.255196679	0.000275	0.365270834	1.65E-08	0.104734456	0.342276	C4	-	-	-	-	-	Y	-
ENSG00000151835	SACS	0.419790858	0.002608	0.085739427	0.752289	0.225876631	0.050174	-0.32747632	0.028493	-0.179379656	0.166941	0.142826237	0.588611	C5	-	Y	-	-	Y	-	-
ENSG00000151849	CENPJ	0.681014585	2.35E-27	0.233577356	0.006893	0.270788745	0.000136	-0.440555518	3.16E-16	-0.395802738	1.20E-17	0.039359943	0.758618	C5	-	Y	-	-	Y	Y	-
ENSG00000151876	FBXO4	-0.733551656	0.007402	-0.102136672	0.801666	-0.22816456	0.484884	0.638561131	0.027802	0.520125372	0.084734	-0.122943742	0.823416	C2	-	Y	-	-	Y	-	-
ENSG00000151881	TMEM267	-0.614295466	9.85E-05	-0.328719672	0.12464	-0.189971218	0.389935	0.292226159	0.140363	0.439524614	0.013012	0.14109198	0.697586	C4	-	Y	-	-	-	Y	-
ENSG00000151883	PARP8	-0.31477049	8.34E-05	-0.202119544	0.034491	-0.127304269	0.163496	0.119549541	0.249102	0.202084895	0.018664	0.077801018	0.615445	C4	-	Y	-	-	-	-	-
ENSG00000151929	BAG3	0.207504244	0.429856	0.423048235	0.108325	0.544479838	0.017365	0.221998502	0.393983	0.351709563	0.124381	0.124091614	0.770632	C4	-	-	-	Y	-	-	-
ENSG00000151948	GLT1D1	-0.509757042	0.014772	-0.464969667	0.052542	-0.376701929	0.105174	0.04848347	0.891759	0.147824213	0.628872	0.093824132	0.869474	C3	-	Y	-	-	-	-	-
ENSG00000151967	SCHIP1	-0.161768219	0.072309	0.34721337	0.000123	0.171352266	0.058046	0.515653793	2.99E-09	0.347818456	3.46E-05	-0.17318936	0.168156	C4	-	-	Y	-	Y	Y	-
ENSG00000152061	RABGAP1L	-0.304270698	0.002503	0.194714746	0.098036	0.054257635	0.727807	0.505522473	2.79E-08	0.373397248	0.00019	-0.137486014	0.391651	C4	-	Y	-	-	Y	Y	-
ENSG00000152076	CCDC74B	-0.29843317	4.37E-05	0.062858749	0.61268	-0.151076254	0.060937	0.367804735	1.39E-06	0.162197788	0.025768	-0.211194412	0.029626	C4	-	-	-	-	Y	-	-
ENSG00000152092	ASTN1	-0.960078307	1.09E-07	-0.078181915	0.799772	0.254324481	0.119398	0.891399959	5.59E-05	1.229019213	7.23E-13	0.335331324	0.203996	C4	-	Y	-	-	Y	Y	-
ENSG00000152104	PTPN14	0.564994983	0.028377	0.690821331	0.025957	0.793531466	0.003914	0.131693701	0.675746	0.243285217	0.360404	0.105965426	0.853204	C1	-	Y	Y	Y	-	-	-
ENSG00000152133	GPATCH11	0.872787263	1.04E-34	0.270799882	0.003301	0.397846126	1.26E-06	-0.595247492	8.89E-24	-0.460494296	2.17E-15	0.129512989	0.204806	C5	-	Y	-	Y	Y	Y	-
ENSG00000152137	HSPB8	1.020086085	0.063855	1.729031589	0.000611	1.567967555	0.006398	0.716230701	0.105419	0.564464993	0.286209	-0.156698743	NA	C2	-	-	Y	Y	-	-	-
ENSG00000152193	OBI1	0.094660147	0.509678	-0.48464195	0.00089	-0.207293158	0.202399	-0.573679553	7.12E-08	-0.288000871	0.009277	0.280281139	0.127283	C5	-	-	Y	-	Y	-	-
ENSG00000152217	SETBP1	-0.026846356	0.934492	0.324798282	0.202606	0.032903588	0.926607	0.357714193	0.028269	0.073563398	0.747183	-0.289630507	0.216666	C1	-	-	-	-	Y	-	-
ENSG00000152219	ARL14EP	0.492281206	4.39E-11	-0.008442799	0.96165	0.269020701	0.002724	-0.494351679	2.46E-11	-0.20904785	0.004589	0.280147185	0.006348	C5	-	Y	-	-	Y	-	-
ENSG00000152223	EPG5	0.595917448	7.93E-06	0.346720895	0.016449	0.414144674	0.003087	-0.243485267	0.02436	-0.167480914	0.155456	0.070887918	0.712832	C5	-	Y	Y	Y	-	-	-
ENSG00000152242	C18orf25	0.301505925	5.07E-05	-0.096639546	0.38845	-0.003726168	0.979525	-0.391764101	2.02E-09	-0.290716549	1.37E-05	0.095654232	0.446345	C5	-	Y	-	-	Y	-	-
ENSG00000152256	PDK1	-0.223995024	0.003868	0.466849275	5.91E-06	0.231979277	0.04757	0.697438225	2.28E-16	0.47109816	1.12E-07	-0.233534823	0.401822	C4	-	-	Y	-	Y	Y	-
ENSG00000152332	UHMK1	0.372751841	1.08E-07	0.135931331	0.164341	0.091955534	0.344615	-0.230384427	1.58E-05	-0.266264092	3.77E-08	-0.041224779	0.728916	C5	-	Y	-	-	-	-	-
ENSG00000152348	ATG10	-0.780434845	9.57E-10	0.034754838	0.865306	0.035709324	0.855316	0.821756883	2.47E-11	0.830915427	4.82E-11	0.003976001	0.991269	C2	-	Y	-	-	Y	Y	-
ENSG00000152377	SPOCK1	0.294595337	0.28244	1.303967302	4.01E-10	1.548489561	2.49E-17	1.015923493	2.85E-07	1.268573373	8.92E-13	0.248947956	0.339314	C2	-	-	Y	Y	Y	Y	-
ENSG00000152380	FAM151B	0.834558492	0.003859	0.577607942	0.116086	0.260021271	0.54369	-0.25095093	0.453204	-0.56036727	0.059395	-0.314546138	NA	C1	-	Y	-	-	-	-	-
ENSG00000152402	GUCY1A2	0.761459221	0.026992	0.310742874	0.574502	0.252290641	0.640055	-0.443226721	0.228082	-0.494598258	0.170129	-0.056269134	NA	C5	-	Y	-	-	-	-	-
ENSG00000152404	CWF19L2	0.585559164	6.08E-06	0.209557467	0.257727	0.11478818	0.600977	-0.369421397	0.002995	-0.455855648	0.000591	-0.091750349	0.755306	C5	-	Y	-	-	Y	Y	-
ENSG00000152413	HOMER1	0.305058308	0.001599	0.175026381	0.145836	0.214078942	0.080975	-0.123715623	0.262757	-0.076081263	0.558735	0.04196592	0.863083	C5	-	Y	-	-	-	-	-
ENSG00000152422	XRCC4	-0.50482311	0.000103	0.18482173	0.234743	-0.03927134	0.83944	0.696714432	6.96E-08	0.480339172	0.000343	-0.221065745	0.218021	C2	-	Y	-	-	Y	Y	-
ENSG00000152433	ZNF547	0.158270578	0.574659	-0.454847238	0.225314	0.047536667	0.903095	-0.606298326	0.0426	-0.096587893	0.755687	0.505318731	0.245288	C5	-	-	-	-	Y	-	-
ENSG00000152454	ZNF256	-0.189608819	0.266987	0.368796633	0.00931	-0.203598598	0.241019	0.565298171	6.03E-05	0.000677759	0.997483	-0.56962545	0.000132	C4	-	-	Y	-	Y	-	Y
ENSG00000152457	DCLRE1C	0.434230537	1.56E-05	0.170400539	0.209705	0.260650749	0.02394	-0.257567302	0.008305	-0.159093962	0.119348	0.093117995	0.592369	C5	-	Y	-	-	-	-	-
ENSG00000152465	NMT2	-0.180183629	0.086456	0.033915252	0.837945	0.107184414	0.395822	0.220748923	0.033151	0.302174844	0.001933	0.076243417	0.679753	C4	-	-	-	-	-	Y	-
ENSG00000152467	ZSCAN1	-1.185758282	4.50E-05	-0.634453131	0.035281	-0.889504434	0.002053	0.559117475	0.127494	0.311516044	0.463559	-0.252362662	NA	C3	-	Y	Y	Y	-	-	-
ENSG00000152475	ZNF837	-1.007967857	0.002514	-0.738919542	0.036215	-0.495499484	0.144864	0.278689394	0.565075	0.527137482	0.190686	0.245938327	NA	C3	-	Y	Y	-	-	-	-
ENSG00000152484	USP12	0.457470623	1.40E-10	0.182808116	0.038628	0.175297342	0.043975	-0.268282274	1.74E-06	-0.268078413	2.26E-06	-0.005060762	0.977318	C5	-	Y	-	-	-	-	-
ENSG00000152503	TRIM36	-0.267132841	0.001394	-0.477471747	5.76E-09	-0.35587323	0.000182	-0.204081974	0.020704	-0.074214569	0.516005	0.124578471	0.423817	C4	-	-	Y	Y	-	-	-
ENSG00000152527	PLEKHH2	-0.859273461	0.000869	-0.598632493	0.028509	-0.271494713	0.328846	0.271329789	0.476633	0.602720116	0.042616	0.329133058	0.454595	C3	-	Y	Y	-	-	Y	-
ENSG00000152578	GRIA4	-0.453004962	0.008889	-0.155117594	0.498161	-0.116322965	0.630711	0.305198053	0.153427	0.351819184	0.096768	0.041271273	0.935871	C2	-	Y	-	-	-	-	-
ENSG00000152582	SPEF2	-0.113171754	0.719082	0.347138561	0.207326	0.615202027	0.006984	0.467291505	0.052648	0.743161702	0.000652	0.271355919	0.405196	C4	-	-	-	Y	-	Y	-
ENSG00000152620	NADK2	-0.395101524	5.83E-08	-0.031858019	0.825631	-0.046758041	0.675321	0.369647017	2.19E-05	0.363072986	3.85E-06	-0.012050989	0.959612	C4	-	Y	-	-	Y	Y	-
ENSG00000152672	CLEC4F	-1.048009386	0.000254	-0.286888151	0.41777	0.195400026	0.597027	0.769257452	0.021601	1.25801718	5.15E-05	0.484090669	NA	C4	-	Y	-	-	Y	Y	-
ENSG00000152782	PANK1	0.363559712	9.58E-06	0.243864671	0.027565	0.392489779	4.69E-06	-0.112752116	0.248719	0.043660648	0.665583	0.15082229	0.231619	C4	-	Y	-	Y	-	-	-
ENSG00000152795	HNRNPDL	0.014015229	0.798793	-0.1234929	0.009705	-0.360518512	5.21E-17	-0.130988016	5.67E-05	-0.359798459	1.04E-31	-0.234110738	7.84E-10	C5	-	-	-	Y	-	Y	-
ENSG00000152822	GRM1	-2.364455897	0.000677	-0.931136233	0.200326	-0.330086368	0.696772	1.436726487	0.094429	2.047532133	0.008404	0.604134951	NA	C3	-	Y	-	-	-	Y	-
ENSG00000152932	RAB3C	0.389618535	4.84E-06	0.280222231	0.002715	0.326056952	0.000235	-0.103256244	0.204999	-0.048930321	0.593612	0.049020999	0.729425	C1	-	Y	-	Y	-	-	-
ENSG00000152936	LMNTD1	-0.71747711	0.006329	0.143949964	0.685079	-0.206086915	0.509207	0.86907845	0.000731	0.526094898	0.063729	-0.346780392	0.325951	C2	-	Y	-	-	Y	-	-
ENSG00000152944	MED21	0.371327521	1.23E-06	0.140211452	0.180821	0.165352541	0.192292	-0.22479932	0.005038	-0.191072678	0.065475	0.027967636	0.916916	C5	-	Y	-	-	-	-	-
ENSG00000152990	ADGRA3	0.426385926	1.86E-12	0.151485767	0.081741	0.148943309	0.046398	-0.267970758	4.83E-07	-0.263119139	1.22E-08	-0.000246309	0.998367	C5	-	Y	-	-	-	-	-

ENSG00000153006	SREK1IP1	0.581921999	5.74E-15	0.043099916	0.785715	0.264181576	0.002043	-0.532227043	4.10E-10	-0.303140797	6.66E-05	0.223976588	0.072232	C5	-	Y	-	-	Y	Y	-
ENSG00000153015	CWC27	0.398200538	7.20E-05	0.061925424	0.749687	-0.002297121	0.991242	-0.329134922	0.000974	-0.385624878	3.23E-05	-0.061616726	0.796496	C5	-	Y	-	-	Y	Y	-
ENSG00000153048	CARHSP1	-0.38186364	4.15E-10	-0.273183843	5.89E-05	-0.358963851	6.77E-09	0.115475455	0.040732	0.037711105	0.562406	-0.083287957	0.275302	C4	-	Y	-	Y	-	-	-
ENSG00000153093	ACOXL	-1.547382184	0.014789	-0.409107628	0.584419	-0.683870743	0.312655	1.145806479	0.144532	0.87628403	0.319357	-0.273079713	NA	C3	-	Y	-	-	-	-	-
ENSG00000153094	BCL2L11	-0.372917458	2.30E-08	-0.06946472	0.479176	-0.239203993	0.002789	0.309985979	1.57E-06	0.148250463	0.054596	-0.166911894	0.083834	C4	-	Y	-	-	Y	-	-
ENSG00000153132	CLGN	-0.32539807	0.000123	-0.200312139	0.093254	-0.026623673	0.865728	0.131709058	0.264933	0.313481389	0.001311	0.175825359	0.305142	C4	-	Y	-	-	-	Y	-
ENSG00000153140	CETN3	-0.427937335	1.20E-09	-0.175944057	0.033477	-0.156108741	0.048048	0.258573385	0.000923	0.286529083	0.000118	0.022773832	0.90074	C4	-	Y	-	-	-	-	-
ENSG00000153162	BMP6	-0.918344265	0.689398	2.82566589	NA	2.067690109	NA	3.745508605	0.000188	2.996689761	0.004165	-0.747525703	NA	C2	-	-	-	-	Y	Y	-
ENSG00000153179	RASSF3	0.222395253	0.013734	0.449809384	6.62E-07	0.460193647	5.23E-07	0.234138652	0.011229	0.252696892	0.005825	0.013006386	0.959572	C4	-	-	Y	Y	-	-	-
ENSG00000153201	RANBP2	0.354552622	1.34E-06	-0.04996776	0.707648	0.091778084	0.38192	-0.397929459	5.33E-13	-0.248125269	2.83E-06	0.144416083	0.116803	C5	-	Y	-	-	Y	-	-
ENSG00000153207	AHCTF1	0.43679084	3.03E-11	0.111913458	0.261429	0.163759781	0.047403	-0.318618686	1.11E-10	-0.258448193	1.08E-08	0.054758544	0.609325	C5	-	Y	-	-	Y	-	-
ENSG00000153214	TMEM87B	0.891573357	2.05E-17	0.327143894	0.034507	0.299024863	0.026851	-0.558094761	3.82E-09	-0.577924079	1.52E-12	-0.025272646	0.93194	C1	-	Y	Y	-	Y	Y	-
ENSG00000153233	PTPRR	-0.64468118	1.85E-11	-0.24910562	0.026826	-0.054723716	0.688759	0.401890318	0.000101	0.604769216	1.05E-10	0.19708931	0.137108	C4	-	Y	-	-	Y	Y	-
ENSG00000153253	SCN3A	-0.217400033	0.037392	-0.154414339	0.255292	0.122849758	0.329625	0.069157833	0.560253	0.354848005	2.44E-05	0.280278937	0.014641	C4	-	-	-	-	-	Y	-
ENSG00000153303	FRMD1	-1.729328222	8.23E-05	0.739382106	0.047157	0.591436471	0.095441	2.476496706	1.01E-09	2.335249875	4.99E-09	-0.144315419	NA	C2	-	Y	Y	-	Y	Y	-
ENSG00000153339	TRAPPC8	0.457875642	8.60E-11	0.245584591	0.005327	0.196163742	0.025448	-0.205665666	0.002127	-0.246906439	0.000143	-0.046662249	0.751141	C5	-	Y	-	-	-	-	-
ENSG00000153363	LINC00467	-1.076372326	2.12E-16	-0.427577063	0.021149	-0.450266816	0.003286	0.65693517	0.000326	0.640859876	8.95E-05	-0.021854461	0.966961	C3	-	Y	Y	Y	Y	Y	-
ENSG00000153391	INO80C	-0.376597652	0.001861	-0.244797489	0.098532	-0.298528787	0.017773	0.138865403	0.383655	0.09285966	0.555674	-0.050696295	0.863411	C4	-	Y	-	-	-	-	-
ENSG00000153404	PLEKHG4B	-0.364503115	0.000491	-0.127053147	0.358675	-0.19177191	0.065199	0.243602599	0.039753	0.18739981	0.092436	-0.061894403	0.767316	C4	-	Y	-	-	-	-	-
ENSG00000153406	NMRAL1	-0.559040268	8.75E-09	-0.189944509	0.084613	-0.169401048	0.1336	0.375908265	1.74E-05	0.404532693	6.01E-06	0.023119239	0.912828	C4	-	Y	-	-	Y	Y	-
ENSG00000153443	UBALD1	0.364756361	0.000815	0.114026224	0.512298	0.091361777	0.625494	-0.243956015	0.025833	-0.258668829	0.027742	-0.019732178	0.952689	C5	-	Y	-	-	-	-	-
ENSG00000153487	ING1	0.274254601	0.008334	-0.072271122	0.647086	0.04885316	0.767376	-0.339805192	0.000215	-0.210799183	0.037909	0.123704966	0.456043	C5	-	-	-	-	Y	-	-
ENSG00000153531	ADPRHL1	-0.555963508	0.040584	-0.030742862	0.938897	0.095214444	0.757132	0.529437587	0.074822	0.665651052	0.01259	0.128921442	0.781815	C4	-	Y	-	-	-	Y	-
ENSG00000153551	CMTM7	-0.332625287	0.029666	-0.17403929	0.375174	-0.196559311	0.234255	0.165603387	0.368832	0.151025121	0.37666	-0.019276034	0.959902	C4	-	Y	-	-	-	-	-
ENSG00000153558	FBXL2	0.451914717	0.000106	0.334133179	0.062341	0.442607909	0.000451	-0.11146525	0.536667	0.005353841	0.973365	0.111701579	0.694101	C5	-	Y	-	Y	-	-	-
ENSG00000153563	CD8A	-0.848157408	0.000418	-0.248285934	0.409816	-0.232841247	0.427175	0.606294585	0.025856	0.630058537	0.020953	0.018522781	0.97796	C2	-	Y	-	-	Y	Y	-
ENSG00000153574	RPIA	0.698984599	2.10E-20	0.25859142	0.018684	0.177539959	0.085626	-0.43288788	1.38E-09	-0.506481728	2.75E-15	-0.078770722	0.607587	C5	-	Y	-	-	Y	Y	-
ENSG00000153721	CNKSR3	0.706961186	2.82E-07	0.164002427	0.430047	0.062457999	0.802841	-0.536678621	3.97E-05	-0.629844412	4.43E-06	-0.098596986	0.746074	C5	-	Y	-	-	Y	Y	-
ENSG00000153790	C7orf31	-0.474038606	0.025062	-0.374373983	0.118649	-0.172492723	0.494875	0.105530421	0.717576	0.315880347	0.165855	0.204542846	0.558058	C4	-	Y	-	-	-	-	-
ENSG00000153814	JAZF1	0.04708696	0.777047	-0.405905477	0.012655	-0.13677582	0.441191	-0.447163118	0.003074	-0.169272615	0.291102	0.27322061	0.261369	C5	-	-	Y	-	Y	-	-
ENSG00000153823	PID1	-0.145247402	0.931925	2.193569702	NA	2.238301529	0.009178	2.350258475	0.001499	2.403095012	0.00274	0.048777711	NA	C2	-	-	-	Y	Y	Y	-
ENSG00000153896	ZNF599	0.368543269	0.002797	0.097956035	0.644777	0.244620154	0.113651	-0.26480024	0.029536	-0.109858684	0.403303	0.149702779	0.491663	C5	-	Y	-	-	-	-	-
ENSG00000153922	CHD1	0.259755991	0.000375	-0.055930436	0.64226	0.018956035	0.889552	-0.309764265	5.15E-09	-0.226190117	9.76E-05	0.077928764	0.498637	C5	-	-	-	-	Y	-	-
ENSG00000153976	HS3ST3A1	3.517203095	0.044348	4.512473356	NA	4.521990028	NA	1.005159019	0.3695	1.022512894	0.320859	0.00614682	NA	C2	-	Y	-	-	-	-	-
ENSG00000154027	AK5	-0.431421911	0.003318	0.366617587	0.01293	0.477946787	8.34E-05	0.804464	4.45E-10	0.923781777	8.23E-16	0.114287205	0.559415	C4	-	Y	Y	Y	Y	Y	-
ENSG00000154040	CABYR	-0.298941478	0.090988	-0.159749014	0.45842	0.015809682	0.949258	0.14529056	0.486322	0.329400512	0.048469	0.178203974	0.472263	C4	-	-	-	-	-	Y	-
ENSG00000154059	IMPACT	-0.637459955	1.97E-17	-0.212543245	0.016254	-0.243002257	0.003937	0.431635306	1.84E-07	0.409023752	6.83E-07	-0.027792483	0.896094	C2	Y	Y	-	-	Y	Y	-
ENSG00000154079	SDHAF4	-0.383703879	0.090721	0.018572545	0.963692	0.082108873	0.795152	0.40861728	0.046301	0.481356226	0.010411	0.067026322	0.883392	C4	-	-	-	-	Y	Y	-
ENSG00000154124	OTULIN	0.754552652	2.46E-15	0.300984451	0.007643	0.480422049	6.03E-07	-0.447273072	1.04E-10	-0.259943033	0.000114	0.181801676	0.032865	C5	-	Y	Y	Y	Y	-	-
ENSG00000154127	UBASH3B	1.425085091	3.21E-05	1.772484926	2.91E-08	1.786879565	1.44E-08	0.354031534	0.267307	0.376777317	0.231488	0.017475219	0.979815	C1	-	Y	Y	Y	-	-	-
ENSG00000154134	ROBO3	-0.847047986	1.03E-16	-0.477597604	2.50E-05	-0.350487375	0.002427	0.375694067	0.001599	0.511346776	7.70E-06	0.129948682	0.528315	C4	-	Y	Y	Y	Y	Y	-
ENSG00000154146	NRGN	-0.408467276	0.013179	-0.387212531	0.058981	-0.308089611	0.127303	0.028881668	0.899466	0.115530341	0.551163	0.08157729	0.827929	C4	-	Y	-	-	-	-	-
ENSG00000154153	RETREG1	-0.853026868	9.48E-05	-0.370080347	0.120688	-0.063456603	0.83963	0.490959579	0.059246	0.804205626	0.000754	0.308834781	0.361968	C4	-	Y	-	-	-	Y	-
ENSG00000154188	ANGPT1	-0.329464189	0.10166	-0.291938525	0.223011	0.146920191	0.567966	0.043872502	0.88123	0.491083415	0.01459	0.441632581	0.092934	C4	-	-	-	-	-	Y	-
ENSG00000154217	PITPNC1	0.383577998	7.77E-08	-0.036465104	0.787399	-0.037490228	0.750058	-0.4135916	6.99E-09	-0.406417608	1.74E-09	0.001866939	0.993615	C5	-	Y	-	-	Y	Y	-
ENSG00000154222	CC2D1B	0.324992525	3.21E-05	0.079950416	0.531839	0.124804295	0.225077	-0.238393586	0.004635	-0.185403548	0.02449	0.047711985	0.795685	C5	-	Y	-	-	-	-	-
ENSG00000154229	PRKCA	0.34461013	6.99E-09	0.241992674	0.000698	0.261863926	2.29E-05	-0.096199999	0.028637	-0.068326717	0.047366	0.022661489	0.796431	C1	-	Y	-	-	-	-	-
ENSG00000154240	CEP112	-0.415504474	0.01834	-0.089030596	0.754761	0.06811315	0.732153	0.332919371	0.122494	0.498222916	0.001748	0.160208928	0.597195	C4	-	Y	-	-	-	Y	-
ENSG00000154265	ABCA5	-0.646592992	0.008224	-0.490759313	0.095368	0.009627827	0.9802	0.162047249	0.662811	0.670859218	0.009482	0.503415874	0.161374	C4	-	Y	-	-	-	Y	-
ENSG00000154305	MIA3	0.623149744	1.13E-25	0.32043127	2.13E-05	0.330261931	3.29E-06	-0.296229499	2.18E-11	-0.27809179	7.80E-11	0.012661177	0.933843	C5	-	Y	Y	Y	-	-	-
ENSG00000154309	DISP1	0.382343022	0.001989	0.349469426	0.016266	0.198015857	0.195952	-0.025905436	0.894351	-0.169412867	0.253468	-0.148950021	0.530813	C5	-	Y	Y	-	-	-	-
ENSG00000154310	TNIK	-0.667187966	1.34E-05	-0.12380591	0.606555	-0.024738222	0.92788	0.549274844	0.000362	0.657244039	1.21E-05	0.102081454	0.755306	C4	-	Y	-	-	Y	Y	-
ENSG00000154319	FAM167A	-0.182771499	6.65E-05	0.365233021	6.54E-10	0.239711696	5.67E-06	0.554695562	4.65E-27	0.437126941	3.08E-23	-0.12278269	0.16309	C4	-	-	Y	-	Y	Y	-
ENSG00000154328	NEIL2	0.027669385	0.865755	0.321164999	0.001938	0.340230766	0.000735	0.300162032	0.004215	0.32722118	0.001449	0.022251514	0.92351	C4	-	-	Y	Y	Y	Y	-
ENSG00000154429	CCSAP	0.35631795	1.26E-06	0.04182305	0.752426	0.149565983	0.10467	-0.308092598	1.10E-08	-0.192464434	0.000286	0.110407125	0.213226	C5	-	Y	-	-	Y	-	-
ENSG00000154511	DIPK1A	-0.32909796	0.014022	-0.12913159	0.483342	-0.155718828	0.321802	0.206071496	0.147497	0.187766467	0.171565	-0.023126379	0.943467	C4	-	Y	-	-	-	-	-
ENSG00000154548	SRSF12	0.401516832	1.51E-06	0.016264838	0.930574	0.061240107	0.678328	-0.378196184	1.23E-05	-0.325805747	0.000147	0.047906921	0.840101	C5	Y	Y	-	-	Y	Y	-
ENSG00000154556	SORBS2	-1.129120212	0.008248	-0.072627806	0.904799	-0.13870362	0.793768	1.064952151	0.008364	1.005636535	0.013363	-0.061025758	NA	C2	-	Y	-	-	Y	Y	-
ENSG00000154639	CXADR	-0.374894299	0.00108	-0.074871647	0.695164	0.082787722	0.617704	0.306429056	0.010633	0.472208858	1.17E-05	0.160507101	0.399549	C4	-	Y	-	-	Y	Y	-
ENSG00000154642	C21orf91	0.3074642	0.007135	-0.219645334	0.146849	0.015175818	0.939619	-0.520729426	1.70E-06	-0.277683793	0.011675	0.237579139	0.146465	C5	-	Y	-	-	Y	-	-
ENSG00000154721	JAM2	-0.452389267	0.000103	-0.368906644	0.045684	-0.110358824	0.518073	0.090558206	0.68376	0.356810849	0.009759	0.260607116	0.328007	C2	-	Y	Y	-	-	Y	-
ENSG00000154734	ADAMTS1	0.268099836	5.87E-05	-0.772721174	8.19E-19	-0.259526841	0.003422	-1.034225718	4.94E-46	-0.513511015	5.04E-15	0.516189304	5.80E-07	C5	-	-	Y	-	Y	Y	Y
ENSG00000154736	ADAMTS5	-0.497249702	0.083233	-0.898321147	0.003732	-0.687695532	0.015741	-0.397911016	0.322013	-0.175386764	0.67594	0.215946937	NA	C3	-	-	Y	Y	-	-	-
ENSG00000154743	TSEN2	-0.222413863	0.069918	0.14145863	0.330266	-0.128954267	0.363406	0.370383759	0.000954	0.108261891	0.427336	-0.267508111	0.052784	C4	-	-	-	-	Y	-	-
ENSG00000154814	OXNAD1	0.308421932	0.010676	0.088589591	0.640106	0.184944267	0.250183	-0.21355684	0.102029	-0.1087786	0.4726	0.099229827	0.693069	C5	-	Y	-	-	-	-	-
ENSG00000154822	PLCL2	-0.275665114	0.030084	0.074182373	0.699327	-0.102103589	0.574266	0.356359134	0.003921	0.188231129	0.197226	-0.173407257	0.409181	C4	-	-	-	-	Y	-	-
ENSG00000154832	CXXC1	0.276755916	1.17E-07	-0.06469777	0.494721	-0.017889621	0.861644	-0.334894013	2.05E-10	-0.280035984	1.28E-08	0.049712261	0.686381	C5	-	-	-	-	Y	-	-

ENSG00000154898	CCDC144CP	1.027001738	0.025786	1.014969105	0.069053	1.174448198	0.01772	-0.004442524	0.993684	0.162708769	0.764147	0.161278738	NA	C1	-	Y	-	Y	-	-	-
ENSG00000154920	EME1	-0.47839122	1.67E-13	-0.166827401	0.037707	-0.280387146	0.000382	0.317957137	4.40E-06	0.212633338	0.006506	-0.110412046	0.384946	C4	-	Y	-	-	Y	-	-
ENSG00000155011	DKK2	1.072745866	5.22E-26	0.989864398	5.22E-10	0.974771105	1.70E-07	-0.076864471	0.680185	-0.083539705	0.816341	-0.0161945	0.985471	C1	-	Y	Y	Y	-	-	-
ENSG00000155034	FBXL18	0.335188161	0.000431	-0.075483708	0.613163	0.157775908	0.196392	-0.404658647	1.48E-08	-0.163328923	0.043661	0.235968511	0.019596	C5	-	Y	-	-	Y	-	-
ENSG00000155052	CNTNAP5	0.752481231	0.043748	0.08872133	0.903657	0.098992101	0.87445	-0.659616002	0.089736	-0.64041032	0.074153	0.013728252	NA	C5	-	Y	-	-	-	-	-
ENSG00000155097	ATP6V1C1	0.388673231	1.44E-09	0.034496322	0.766493	0.082041685	0.401979	-0.347599989	8.28E-09	-0.291819455	6.37E-06	0.050475595	0.722416	C5	-	Y	-	-	Y	-	-
ENSG00000155100	OTUD6B	0.381749672	5.33E-06	0.011478442	0.949604	0.099718284	0.395289	-0.36341891	8.74E-07	-0.267613786	0.000153	0.090493901	0.521076	C5	-	Y	-	-	Y	-	-
ENSG00000155115	GTF3C6	-0.265074863	0.000633	-0.16255128	0.107537	-0.346283242	0.00012	0.108948962	0.267686	-0.066530703	0.549214	-0.180245177	0.185111	C4	-	-	-	Y	-	-	-
ENSG00000155158	TTC39B	-0.529882978	0.083063	0.005920454	0.991198	0.123742191	0.750453	0.543147947	0.088573	0.668363176	0.024069	0.121089388	NA	C2	-	-	-	-	-	Y	-
ENSG00000155254	MARVELD1	-0.58885285	3.26E-15	-0.201085378	0.038346	-0.296912912	0.000194	0.394287132	1.74E-06	0.306952088	8.65E-05	-0.092920041	0.52569	C2	-	Y	-	-	Y	Y	-
ENSG00000155256	ZFYVE27	0.122103583	0.10331	-0.1905524	0.027483	-0.113784063	0.2226	-0.3062387	8.89E-06	-0.221356638	0.001624	0.079632694	0.562859	C5	Y	-	-	-	Y	-	-
ENSG00000155275	TRMT44	0.482527992	0.000144	0.249061716	0.132148	0.292476623	0.078285	-0.226889454	0.076436	-0.176044218	0.222541	0.045994543	0.889019	C5	-	Y	-	-	-	-	-
ENSG00000155329	ZCCHC10	0.470457286	1.10E-06	-0.029973184	0.87835	0.031326407	0.851376	-0.493965732	1.47E-06	-0.424253037	1.40E-05	0.06407575	0.787224	C5	-	Y	-	-	Y	Y	-
ENSG00000155363	MOV10	-0.344954505	3.07E-06	-0.181083889	0.045567	-0.160135025	0.063168	0.170306423	0.056759	0.199473938	0.018259	0.023802829	0.90989	C4	-	Y	-	-	-	-	-
ENSG00000155366	RHOC	-0.390706529	6.76E-05	-0.204290792	0.192454	-0.100498355	0.466051	0.192548197	0.129754	0.305327852	0.001106	0.107207322	0.619322	C4	-	Y	-	-	-	Y	-
ENSG00000155495	MAGEC1	-0.377313074	0.042161	-0.256823511	0.207761	-0.110507757	0.631728	0.127119729	0.596411	0.28142179	0.172998	0.149166472	0.632826	C4	-	Y	-	-	-	-	-
ENSG00000155530	LRGUK	0.353825296	0.059782	-0.03281552	0.921424	-0.032209717	0.916277	-0.380295742	0.027595	-0.371761497	0.027776	0.003549853	0.994554	C5	-	-	-	-	Y	Y	-
ENSG00000155542	SETD9	-0.307424759	0.000901	-0.027776619	0.859739	-0.074166043	0.571454	0.285974536	0.002859	0.247923254	0.014463	-0.043535498	0.840887	C4	-	Y	-	-	-	-	-
ENSG00000155561	NUP205	0.411798758	4.90E-14	0.143503635	0.048348	0.058444335	0.458569	-0.261731875	2.78E-13	-0.338825415	4.53E-28	-0.082442515	0.146675	C5	-	Y	-	-	-	Y	-
ENSG00000155621	C9orf85	0.18394643	0.126864	-0.151972717	0.35651	0.129295008	0.378541	-0.329746271	0.004676	-0.040000376	0.787462	0.284161883	0.065195	C5	-	-	-	-	Y	-	-
ENSG00000155629	PIK3AP1	-0.658704364	0.002985	-0.438864487	0.150821	-0.873512462	0.001728	0.227218408	0.527609	-0.200929067	0.6106	-0.433747812	NA	C2	-	Y	-	Y	-	-	-
ENSG00000155657	TTN	-0.153648964	0.251762	0.095250399	0.599109	0.201527004	0.179476	0.254381394	0.015449	0.369841933	0.000386	0.109424761	0.591539	C4	-	-	-	-	-	Y	-
ENSG00000155666	KDM8	-0.14449349	0.555812	-0.537716337	0.027731	-0.125227103	0.687524	-0.386594169	0.120756	0.034042195	0.917894	0.415202129	0.237958	C4	-	-	Y	-	-	-	-
ENSG00000155744	FAM126B	0.490043961	0.00013	-0.060397912	0.800101	0.133069049	0.47239	-0.544382706	5.74E-09	-0.343220906	0.000226	0.195991994	0.212425	C5	-	Y	-	-	Y	Y	-
ENSG00000155849	ELMO1	0.271635147	8.21E-05	0.351742754	1.30E-06	0.270277538	0.000316	0.086740875	0.159576	0.013640627	0.869122	-0.078580018	0.397361	C4	-	-	Y	-	-	-	-
ENSG00000155858	LSM11	0.529183049	0.000106	0.213925155	0.187946	0.24440273	0.076137	-0.309009979	0.016864	-0.270396664	0.026346	0.033181707	0.901103	C1	-	Y	-	-	Y	-	-
ENSG00000155903	RASA2	0.488811008	6.16E-11	0.127532981	0.370318	0.136812238	0.19621	-0.354840954	5.75E-05	-0.33727684	1.23E-06	0.01236442	0.963619	C5	-	Y	-	-	Y	Y	-
ENSG00000155975	VPS37A	0.474169539	8.61E-06	0.172522195	0.292975	0.203571054	0.116985	-0.295405503	0.001983	-0.256066336	0.001458	0.034156424	0.885811	C5	-	Y	-	-	-	-	-
ENSG00000155980	KIF5A	0.472689291	1.43E-13	0.305465723	1.09E-06	0.450585352	2.34E-16	-0.16077363	0.010898	-0.007477791	0.930472	0.148302584	0.024557	C5	-	Y	Y	Y	-	-	-
ENSG00000156017	CARNMT1	0.296023497	0.077156	-0.09961845	0.660333	0.088641701	0.66418	-0.389944126	0.016972	-0.192593078	0.2643	0.190883636	0.411194	C5	-	-	-	-	Y	-	-
ENSG00000156026	MCU	0.659944848	1.49E-10	0.346792471	0.006622	0.312770473	0.008332	-0.306653166	0.007278	-0.332593452	0.00178	-0.031011311	0.914963	C5	-	Y	Y	Y	Y	Y	-
ENSG00000156030	MIDEAS	0.311245884	7.10E-05	0.393204859	6.17E-05	0.307280856	0.000137	0.088128286	0.391325	0.010762288	0.918856	-0.082721096	0.597603	C1	-	Y	Y	Y	-	-	-
ENSG00000156097	GPR61	-1.30049191	0.048916	0.075096607	0.944237	-0.623430628	0.417954	1.386217525	0.053604	0.691918026	0.417974	-0.69800779	NA	C2	-	Y	-	-	-	-	-
ENSG00000156103	MMP16	0.359140247	0.000175	-0.053092029	0.77457	0.063497755	0.667415	-0.406353837	0.00449	-0.281243962	0.027732	0.119171263	0.50724	C5	-	Y	-	-	Y	-	-
ENSG00000156113	KCNMA1	-0.781365828	0.010199	0.158055541	0.659188	-0.033612514	0.937257	0.946472824	0.000814	0.76228733	0.013093	-0.189016781	0.67488	C3	-	Y	-	-	Y	Y	-
ENSG00000156170	NDUFAF6	-0.43191714	2.65E-06	-0.255967644	0.01797	-0.430011476	0.000294	0.182324125	0.095221	0.016507665	0.924676	-0.171043263	0.371835	C4	-	Y	-	Y	-	-	-
ENSG00000156232	WHAMM	0.633818841	1.94E-05	0.126907219	0.658163	0.308240569	0.126406	-0.499825718	0.003422	-0.311149986	0.064298	0.184648499	0.587615	C5	-	Y	-	-	Y	-	-
ENSG00000156239	N6AMT1	0.486700396	1.38E-07	0.281987909	0.006377	0.542662488	1.88E-09	-0.198460875	0.020037	0.070697223	0.473644	0.263820746	0.005006	C4	-	Y	-	Y	-	-	-
ENSG00000156253	RWDD2B	-0.713085564	3.14E-14	-0.228856216	0.0592	-0.111314712	0.39371	0.491096637	1.21E-05	0.616462049	3.88E-09	0.120090517	0.553698	C4	-	Y	-	-	Y	Y	-
ENSG00000156256	USP16	-0.157436137	0.006731	-0.127819014	0.106506	-0.332803259	2.27E-08	0.036102425	0.670924	-0.160723061	0.008595	-0.201968369	0.015757	C4	-	-	-	Y	-	-	-
ENSG00000156273	BACH1	0.24007331	0.001668	0.020494469	0.88773	-0.095666552	0.365978	-0.213257561	0.009407	-0.321232093	2.92E-05	-0.113335417	0.423104	C5	-	-	-	-	-	Y	-
ENSG00000156345	CDK20	0.507856316	2.13E-05	0.166924548	0.310202	0.312581171	0.011356	-0.334724881	0.009916	-0.180666214	0.165434	0.148406749	0.460487	C5	Y	Y	-	Y	Y	-	-
ENSG00000156374	PCGF6	-0.177746312	0.118796	-0.269079007	0.065827	-0.474667816	1.01E-05	-0.085375627	0.609677	-0.282271563	0.018416	-0.202265038	0.337682	C4	-	-	-	Y	-	-	-
ENSG00000156381	ANKRD9	0.167514201	0.192272	0.221450016	0.108243	0.358339052	0.015457	0.060366072	0.691913	0.205235716	0.153976	0.139464244	0.558058	C4	-	-	-	Y	-	-	-
ENSG00000156398	SFXN2	-0.455707908	6.81E-06	0.02248949	0.903628	-0.065779734	0.66593	0.484829727	1.46E-06	0.40487512	8.56E-05	-0.08551002	0.658047	C4	Y	Y	-	-	Y	Y	-
ENSG00000156453	PCDH1	-0.256847265	0.032498	-0.010150836	0.965778	0.203953457	0.122666	0.253200155	0.049314	0.47535289	9.55E-06	0.217093756	0.210317	C4	-	-	-	-	-	Y	-
ENSG00000156469	MTERF3	0.260707737	0.029356	-0.436264549	0.000816	0.016152238	0.938556	-0.690537518	4.29E-09	-0.229819805	0.077714	0.455260205	0.003251	C5	-	-	Y	-	Y	-	Y
ENSG00000156471	PTDSS1	0.526124623	7.64E-18	0.244346893	0.002092	0.274681823	0.000196	-0.275281392	1.76E-05	-0.236813245	0.000183	0.033241781	0.844703	C5	Y	Y	-	-	-	-	-
ENSG00000156475	PPP2R2B	-0.740033441	0.001311	-0.100607744	0.767462	-0.180853818	0.557236	0.647082644	0.010203	0.573858696	0.034503	-0.077951689	0.892177	C2	-	Y	-	-	Y	Y	-
ENSG00000156521	TYSND1	-0.160654944	0.336458	0.203171794	0.258627	0.130092677	0.498359	0.370617638	0.012701	0.305578161	0.048826	-0.070115724	0.822625	C4	-	-	-	-	Y	Y	-
ENSG00000156531	PHF6	0.475607295	3.95E-12	0.146958076	0.140781	0.208772972	0.016585	-0.322436115	2.63E-10	-0.252135791	5.57E-07	0.064761202	0.571867	C5	-	Y	-	-	Y	-	-
ENSG00000156564	LRFN2	-0.700450239	3.34E-06	-0.027859883	0.915338	0.098326752	0.62878	0.679036321	1.69E-05	0.813510732	4.86E-08	0.128932991	0.638347	C4	-	Y	-	-	Y	Y	-
ENSG00000156587	UBE2L6	-0.479619375	4.86E-08	-0.256699906	0.009053	-0.196670786	0.036128	0.229753951	0.007056	0.298105738	0.000129	0.062566105	0.654553	C4	-	Y	-	-	-	-	-
ENSG00000156642	NPTN	0.38947659	2.26E-09	0.249946738	0.002758	0.180292794	0.037977	-0.133462082	0.055774	-0.19448105	0.003031	-0.066564194	0.620166	C5	-	Y	-	-	-	-	-
ENSG00000156671	SAMD8	0.466515117	2.34E-11	0.156463521	0.117283	0.169141098	0.093397	-0.303758706	1.65E-05	-0.282414232	0.000146	0.015569587	0.945376	C5	-	Y	-	-	Y	-	-
ENSG00000156687	UNC5D	0.419779187	0.027273	0.049537508	0.882309	0.106418351	0.707231	-0.364129983	0.064827	-0.29863566	0.144849	0.059767635	0.900554	C5	-	Y	-	-	-	-	-
ENSG00000156735	BAG4	0.412447155	3.49E-09	-0.174258643	0.095398	0.109546103	0.24973	-0.580130896	1.29E-13	-0.288283528	6.88E-05	0.286802828	0.006348	C5	-	Y	-	-	Y	-	-
ENSG00000156875	MFSD14A	0.400016244	0.002499	0.389316471	0.013935	0.303829855	0.045075	-0.004869745	0.979209	-0.08171605	0.60856	-0.082109901	0.758946	C1	-	Y	Y	Y	-	-	-
ENSG00000156958	GALK2	0.385251507	0.007423	-0.085737791	0.762551	-0.283641209	0.157955	-0.464162534	0.004644	-0.65419873	2.16E-05	-0.1943657	0.567669	C5	-	Y	-	-	Y	Y	-
ENSG00000156968	MPV17L	-0.622252915	2.83E-05	-0.453633259	0.003385	-0.400993363	0.036755	0.175240818	0.348912	0.235486225	0.262028	0.054642436	0.901698	C4	-	Y	Y	Y	-	-	-
ENSG00000156976	EIF4A2	0.291799098	4.23E-13	0.236770042	1.37E-06	0.335576614	1.21E-17	-0.048561695	0.280457	0.058471929	0.091123	0.101655061	0.044227	C5	Y	-	-	Y	-	-	-
ENSG00000157087	ATP2B2	-0.110767008	0.47574	0.237410594	0.106944	0.225423801	0.094712	0.354067247	0.002163	0.350389395	0.00154	-0.008949135	0.976274	C4	-	-	-	-	Y	Y	-
ENSG00000157106	SMG1	0.664852088	7.97E-05	0.268242509	0.172617	0.383892752	0.039443	-0.390167137	0.006333	-0.266601766	0.102786	0.118371469	0.632437	C1	Y	Y	-	Y	Y	-	-
ENSG00000157107	FCHO2	0.421579877	4.97E-06	0.233922576	0.068405	0.217472243	0.055209	-0.180910759	0.094436	-0.189394068	0.055409	-0.013951515	0.960328	C5	-	Y	-	-	-	-	-
ENSG00000157152	SYN2	-0.242237784	0.027236	0.052830498	0.731241	0.073438388	0.630323	0.301672964	0.005116	0.330216429	0.003936	0.023346158	0.933843	C4	-	-	-	-	Y	Y	-
ENSG00000157168	NRG1	-0.086360158	0.641739	0.404885494	0.002041	0.295046443	0.043068	0.498234463	0.000341	0.395899453	0.008299	-0.107046394	0.653289	C4	-	-	Y	-	Y	Y	-
ENSG00000157181	ODR4	0.604439935	1.62E-14	0.062974973	0.648059	0.210790015	0.027192	-0.535686063	3.06E-17	-0.379267729	4.68E-10	0.151055368	0.128236	C5	-	Y	-	-	Y	Y	-

ENSG00000157184	CPT2	-0.642398631	4.73E-10	-0.289931592	0.01082	-0.320563783	0.001091	0.359562529	0.00143	0.33688341	0.001297	-0.027198219	0.912643	C2	-	Y	-	Y	Y	Y	-
ENSG00000157193	LRP8	0.306956225	1.49E-05	0.2945451	9.61E-05	0.263030524	0.000278	-0.006002486	0.946084	-0.029584564	0.687512	-0.029001521	0.803932	C4	-	Y	-	-	-	-	-
ENSG00000157216	SSBP3	-0.33369368	8.48E-10	-0.033416156	0.723268	-0.117829565	0.086237	0.307099245	7.61E-11	0.230647445	1.47E-06	-0.081715157	0.311383	C4	-	Y	-	-	Y	-	-
ENSG00000157224	CLDN12	0.407198963	6.09E-09	0.022182952	0.871316	0.29381115	0.000267	-0.378810883	7.13E-17	-0.099298815	0.062104	0.274240922	5.37E-06	C5	Y	Y	-	-	Y	-	-
ENSG00000157326	DHRS4	-0.362366463	0.028106	-0.369785167	0.077552	-0.366032557	0.066573	-0.000860682	0.996968	0.011468621	0.962597	0.006494293	0.991365	C4	-	Y	-	-	-	-	-
ENSG00000157343	ARMC12	-0.839268899	0.003732	-0.2130624	0.603612	-0.111983846	0.734557	0.638647988	0.09363	0.741766882	0.0169	0.10252193	0.88523	C3	-	Y	-	-	-	Y	-
ENSG00000157349	DDX19B	-0.187661151	0.11208	-0.276383127	0.047257	-0.314487923	0.003214	-0.082735412	0.618755	-0.11217753	0.40298	-0.035226009	0.912066	C4	-	-	-	Y	-	-	-
ENSG00000157404	KIT	-1.826521316	2.67E-06	-2.165140502	9.35E-06	-1.440644255	0.000282	-0.346402619	0.682432	0.400242028	0.527983	0.737993125	NA	C3	-	Y	Y	Y	-	-	-
ENSG00000157426	AASDH	0.31554955	0.008339	0.089029891	0.614062	0.170885008	0.213229	-0.220314619	0.036896	-0.130133679	0.231363	0.085042194	0.632062	C1	-	Y	-	-	-	-	-
ENSG00000157429	ZNF19	-0.408590843	0.034298	-0.085817655	0.764744	-0.140271754	0.574266	0.329120193	0.114222	0.282806829	0.195668	-0.051759773	0.910998	C4	-	Y	-	-	-	-	-
ENSG00000157450	RNF111	0.410461265	7.78E-08	0.018712383	0.91022	0.164667197	0.127215	-0.385542271	5.81E-08	-0.231067045	0.001243	0.148911754	0.253304	C5	-	Y	-	-	Y	-	-
ENSG00000157456	CCNB2	-0.485467991	4.33E-13	-0.142619929	0.16614	-0.188816248	0.035911	0.349128669	1.56E-07	0.311532458	9.76E-07	-0.043040565	0.796431	C4	-	Y	-	-	Y	Y	-
ENSG00000157470	FAM81A	-0.536840709	0.109768	0.338202901	0.367695	-0.106919045	0.825992	0.882146965	0.005414	0.445019696	0.25871	-0.442601424	NA	C2	-	-	-	-	Y	-	-
ENSG00000157483	MYO1E	0.51349213	1.97E-05	0.078371159	0.75659	-0.022307503	0.930882	-0.428838662	0.00176	-0.521112825	4.57E-05	-0.097738398	0.773771	C5	-	Y	-	-	Y	Y	-
ENSG00000157500	APPL1	0.448786205	1.31E-11	0.202091705	0.024394	0.185332346	0.036648	-0.24018382	4.80E-06	-0.248615949	4.15E-06	-0.013929028	0.93752	C5	-	Y	-	-	-	-	-
ENSG00000157570	TSPAN18	-0.566205832	1.15E-28	-0.314352586	8.60E-10	-0.248129324	0.000109	0.258333034	2.00E-07	0.332602141	2.77E-09	0.069146518	0.500085	C4	-	Y	Y	-	-	Y	-
ENSG00000157617	C2CD2	0.230720061	0.083318	0.223752585	0.145639	0.385603519	0.002586	-0.000371685	0.998237	0.169920671	0.201585	0.165016857	0.375519	C4	-	-	-	Y	-	-	-
ENSG00000157637	SLC38A10	-0.590564474	1.03E-23	-0.009254666	0.93176	-0.047506128	0.562538	0.587858542	1.09E-33	0.557927847	2.15E-31	-0.035258642	0.702203	C4	-	Y	-	-	Y	Y	-
ENSG00000157654	PALM2AKAP2	0.130247715	0.487381	0.384061152	0.032411	0.378223478	0.027891	0.26075969	0.087529	0.262769272	0.083115	-0.003261197	0.993839	C1	-	-	Y	Y	-	-	-
ENSG00000157734	SNX22	-0.636663302	0.036683	-0.31578628	0.47586	-0.105127774	0.805643	0.327605054	0.386454	0.546956582	0.066968	0.212810705	NA	C4	-	Y	-	-	-	-	-
ENSG00000157741	UBN2	0.693059382	0.000305	0.1747015	0.56059	0.434901636	0.040998	-0.511859461	0.0037	-0.243740672	0.157866	0.262922979	0.313645	C1	-	Y	-	Y	Y	-	-
ENSG00000157764	BRAF	0.410041656	3.71E-07	0.064264168	0.61198	0.120253693	0.245208	-0.339391232	2.57E-06	-0.27518912	0.000141	0.058707115	0.694994	C5	-	Y	-	-	Y	-	-
ENSG00000157796	WDR19	0.376621449	3.66E-08	0.377015598	5.80E-07	0.338257653	6.46E-06	0.00683934	0.943815	-0.023882958	0.783609	-0.036120316	0.798004	C4	-	Y	Y	Y	-	-	-
ENSG00000157827	FMNL2	0.467545739	8.10E-14	0.039229399	0.697385	0.212084087	0.000905	-0.421922023	1.66E-15	-0.241073935	2.56E-06	0.175687842	0.004434	C5	-	Y	-	-	Y	-	-
ENSG00000157881	PANK4	0.489755297	1.27E-07	0.310989997	0.006322	0.194370046	0.088864	-0.171889753	0.084125	-0.280287961	0.001725	-0.114086314	0.473977	C5	Y	Y	Y	-	-	-	-
ENSG00000157911	PEX10	-0.665450267	1.77E-08	-0.126125081	0.51736	-0.251457628	0.094357	0.546662199	0.000204	0.428738108	0.003531	-0.123975646	0.67488	C4	-	Y	-	-	Y	Y	-
ENSG00000157927	RADIL	-0.403857185	2.84E-05	0.035328137	0.86888	-0.078775379	0.589322	0.445425577	0.000223	0.339876276	0.001449	-0.110862014	0.642319	C4	-	Y	-	-	Y	Y	-
ENSG00000157978	LDLRAP1	-0.387648227	0.278718	0.217845672	0.607646	0.529824588	0.079422	0.611646958	0.077262	0.931681187	0.001874	0.314737917	0.505914	C4	-	-	-	-	-	Y	-
ENSG00000157999	ANKRD61	0.679077004	0.043869	0.5557962	0.183943	0.734294372	0.026433	-0.117487246	0.775699	0.068445805	0.859505	0.182270437	NA	C1	-	Y	-	Y	-	-	-
ENSG00000158019	BABAM2	-0.509075277	8.98E-11	0.020698173	0.87984	-0.043615832	0.708924	0.536178012	1.23E-11	0.480233735	2.20E-09	-0.061439184	0.681747	C2	-	Y	-	-	Y	Y	-
ENSG00000158050	DUSP2	-1.266738822	2.95E-12	-0.385848021	0.047771	-0.404906316	0.031134	0.887412826	2.13E-06	0.877143177	3.83E-06	-0.016297922	0.971963	C2	-	Y	Y	Y	Y	Y	-
ENSG00000158089	GALNT14	-1.150508384	4.92E-05	-0.629012908	0.045895	-0.528387976	0.090073	0.528504083	0.123687	0.637257461	0.054445	0.103602048	NA	C3	-	Y	Y	-	-	-	-
ENSG00000158106	RHPN1	-0.200362621	0.14107	0.172055814	0.287741	0.293271057	0.024638	0.378625316	0.003416	0.508608979	2.25E-05	0.123977743	0.563782	C4	-	-	-	-	Y	Y	-
ENSG00000158158	CNNM4	-0.111153296	0.262091	-0.02702416	0.851475	0.216107295	0.018305	0.090559054	0.394721	0.341875862	5.70E-05	0.245992112	0.018109	C4	-	-	-	-	-	Y	-
ENSG00000158161	EYA3	0.749217156	7.29E-27	0.210419739	0.022883	0.287337176	0.004462	-0.532335141	1.19E-13	-0.447363655	4.16E-08	0.080342612	0.659832	C5	-	Y	-	-	Y	Y	-
ENSG00000158164	TMSB15A	-0.222796701	9.37E-05	-0.345389572	6.03E-08	-0.370442309	2.87E-08	-0.11621594	0.039396	-0.132958831	0.024447	-0.022163966	0.88523	C4	-	-	Y	Y	-	-	-
ENSG00000158220	ESYT3	-0.710080313	0.100026	-0.509588274	0.311218	0.148253278	0.774813	0.209029664	0.741196	0.872754608	0.044436	0.660690171	NA	C4	-	-	-	-	-	Y	-
ENSG00000158234	FAIM	-0.804600517	2.10E-07	-0.33134929	0.055097	-0.445471961	0.006417	0.479566717	0.007377	0.374197897	0.050665	-0.111051502	0.733222	C3	-	Y	-	Y	Y	-	-
ENSG00000158258	CLSTN2	0.365504436	0.139423	-0.059722869	0.887906	-0.368381506	0.166526	-0.421211718	0.145944	-0.718828839	0.003906	-0.303691502	0.514285	C5	-	-	-	-	-	Y	-
ENSG00000158286	RNF207	-0.429627943	6.34E-05	0.014890118	0.938308	0.065948016	0.647206	0.451600341	8.52E-05	0.510360706	2.23E-06	0.053961484	0.816611	C4	-	Y	-	-	Y	Y	-
ENSG00000158406	H4C8	-1.131412045	0.046983	-0.238115334	0.747692	-0.301458515	0.698079	0.901358846	0.123659	0.842917252	0.213476	-0.062324864	NA	C2	-	Y	-	-	-	-	-
ENSG00000158411	MITD1	0.553764196	7.37E-09	-0.018776835	0.92553	0.234856564	0.119457	-0.565930566	1.93E-09	-0.304510177	0.008739	0.256896202	0.155213	C5	-	Y	-	-	Y	Y	-
ENSG00000158417	EIF5B	0.322359454	3.38E-16	-0.003032623	0.978685	-0.053893848	0.343372	-0.318672388	1.15E-11	-0.361519453	1.29E-25	-0.048130802	0.62517	C5	Y	Y	-	-	Y	Y	-
ENSG00000158427	TMSB15B	-0.666352472	0.044248	-0.528840749	0.154597	-0.899889603	0.007857	0.142885032	0.752307	-0.218448918	0.628339	-0.367734448	NA	C4	-	Y	-	Y	-	-	-
ENSG00000158458	NRG2	0.116638436	0.496266	0.384092807	0.011467	0.307897352	0.051742	0.273948644	0.058379	0.205939926	0.188066	-0.073242843	0.799273	C4	-	-	Y	-	-	-	-
ENSG00000158467	AHCYL2	0.707498195	3.74E-23	0.278293925	0.001516	0.252025422	0.002327	-0.422646874	2.85E-09	-0.440727947	6.93E-11	-0.023469392	0.901588	C5	Y	Y	-	-	Y	Y	-
ENSG00000158480	SPATA2	0.360979461	0.002966	-0.241888778	0.123043	0.086934343	0.629358	-0.596332374	1.18E-07	-0.259950635	0.028624	0.331282454	0.034505	C5	-	Y	-	-	Y	-	Y
ENSG00000158483	FAM86C1P	-0.489926342	0.006865	-0.082246766	0.794958	-0.02526532	0.92263	0.414067437	0.064488	0.479031383	0.008006	0.059804865	0.899179	C4	-	Y	-	-	-	Y	-
ENSG00000158528	PPP1R9A	-0.190585772	0.050086	-0.024360098	0.885518	0.129217533	0.292871	0.172750408	0.083139	0.334626855	0.000208	0.156342931	0.301721	C4	-	-	-	-	-	Y	-
ENSG00000158552	ZFAND2B	0.381958163	0.001118	0.15164684	0.357077	0.234890748	0.101321	-0.223870863	0.079491	-0.132544943	0.340828	0.086135708	0.72593	C5	-	Y	-	-	-	-	-
ENSG00000158560	DYNC1I1	-0.724479566	0.006032	-0.218602714	0.562883	-0.093026409	0.799183	0.513385965	0.090708	0.646025602	0.015689	0.129066048	0.816997	C2	-	Y	-	-	-	Y	-
ENSG00000158636	EMSY	0.648950883	4.57E-19	0.467558171	4.17E-08	0.435632309	1.82E-06	-0.174594664	0.02422	-0.198546741	0.015509	-0.028945242	0.893738	C5	-	Y	Y	Y	-	-	-
ENSG00000158683	PKD1L1	-1.335895699	0.014974	-0.100197159	0.893798	0.191455081	0.757153	1.24455527	0.03245	1.542632321	0.003707	0.29467237	NA	C3	-	Y	-	-	Y	Y	-
ENSG00000158711	ELK4	0.285550936	0.003553	-0.034988736	0.855696	0.047243365	0.757153	-0.313762371	0.000969	-0.223737209	0.010419	0.084812317	0.658764	C5	-	-	-	-	Y	-	-
ENSG00000158715	SLC45A3	0.525845057	0.0076	0.46204537	0.055515	0.609160499	0.0032	-0.058325123	0.813396	0.098300677	0.650757	0.151302089	0.641855	C1	-	Y	-	Y	-	-	-
ENSG00000158716	DUSP23	-0.438074011	0.00873	-0.223185666	0.292906	-0.187805583	0.356627	0.222098642	0.175822	0.265374861	0.087689	0.037352108	0.915306	C4	-	Y	-	-	-	-	-
ENSG00000158748	HTR6	-0.901334179	4.98E-05	-0.322233534	0.23845	0.055413574	0.868348	0.586257005	0.007436	0.971994388	7.77E-07	0.379923521	0.148991	C4	-	Y	-	-	Y	Y	-
ENSG00000158773	USF1	0.375061988	2.63E-11	-0.01212828	0.92466	0.076112596	0.369856	-0.380795075	1.30E-09	-0.284319178	1.22E-06	0.09109847	0.456495	C5	-	Y	-	-	Y	-	-
ENSG00000158792	SPATA2L	0.565253701	0.003453	0.364207643	0.105059	0.399492526	0.039029	-0.194947511	0.361944	-0.151339691	0.45707	0.036590645	0.933407	C5	-	Y	-	Y	-	-	-
ENSG00000158815	FGF17	0.891041554	0.006747	0.373210398	0.519772	0.198133004	0.748438	-0.509494288	0.205939	-0.67885059	0.08223	-0.169607616	NA	C1	-	Y	-	-	-	-	-
ENSG00000158856	DMTN	-0.604444383	3.48E-14	-0.377947524	5.12E-05	-0.166394274	0.146814	0.233082111	0.020698	0.452662174	3.47E-06	0.213918605	0.156601	C4	-	Y	Y	-	-	Y	-
ENSG00000158859	ADAMTS4	-0.594961615	4.82E-05	-0.286754689	0.072325	-0.236256876	0.217475	0.315509776	0.072946	0.373440038	0.047887	0.05281228	0.901572	C3	-	Y	-	-	-	Y	-
ENSG00000158882	TOMM40L	0.334339945	0.000131	0.145167993	0.220519	0.076746816	0.559018	-0.182768753	0.05535	-0.242894696	0.008543	-0.065517457	0.724399	C5	-	Y	-	-	-	-	-
ENSG00000158887	MPZ	-0.99179141	0.00797	-0.515560143	0.327562	-0.374847263	0.389476	0.489091512	0.395966	0.63128745	0.177874	0.139388638	NA	C3	-	Y	-	-	-	-	-
ENSG00000159055	MIS18A	0.359515157	0.000234	0.054876418	0.711114	0.100601794	0.40371	-0.298307441	0.002106	-0.244150082	0.011544	0.048518592	0.801162	C5	-	Y	-	-	-	-	-
ENSG00000159079	CFAP298	0.269277958	0.007933	-0.225018299	0.095398	0.098264291	0.491412	-0.487167422	3.26E-07	-0.156266217	0.127425	0.325691588	0.009786	C5	-	-	-	-	Y	-	Y
ENSG00000159082	SYNJ1	0.811154848	6.82E-23	0.509294742	2.59E-08	0.428335023	2.00E-05	-0.295678496	0.000182	-0.367942744	9.99E-06	-0.077948857	0.646123	C1	-	Y	Y	Y	-	Y	-

ENSG00000159176	CSRP1	-0.422227096	8.79E-07	-0.111155019	0.373921	-0.294802668	0.000532	0.318057726	0.001249	0.142316105	0.148749	-0.180500522	0.173277	C4	-	Y	-	-	Y	-	-
ENSG00000159200	RCAN1	0.349608231	0.001599	0.171317815	0.260033	0.22654447	0.120543	-0.171932758	0.176683	-0.108536088	0.451507	0.057879679	0.839995	C5	-	Y	-	-	-	-	-
ENSG00000159208	CIART	-0.906051793	6.11E-05	-0.344730477	0.280905	-0.343000465	0.197805	0.570437929	0.06113	0.577567273	0.037283	0.002151811	0.997573	C3	-	Y	-	-	-	Y	-
ENSG00000159214	CCDC24	-1.019318867	9.07E-10	-0.185389259	0.360386	-0.131925502	0.541286	0.84030354	5.21E-07	0.902370227	1.31E-07	0.056307465	0.879022	C3	-	Y	-	-	Y	Y	-
ENSG00000159247	TUBBP5	-3.634551608	0.043884	-1.1525309	NA	-1.990183949	NA	2.49387617	NA	1.662988395	NA	-0.838833073	NA	C3	-	Y	-	-	-	-	-
ENSG00000159256	MORC3	0.269750277	0.000118	-0.102055063	0.287512	0.124124302	0.128711	-0.365700331	4.17E-09	-0.131135913	0.043592	0.229358032	0.002198	C5	-	-	-	-	Y	-	-
ENSG00000159259	CHAF1B	-0.452355472	1.79E-08	-0.289654745	0.009559	-0.336827382	0.000178	0.169543835	0.095389	0.13027757	0.154294	-0.044396596	0.841393	C4	-	Y	-	Y	-	-	-
ENSG00000159267	HLCS	-0.542041998	3.39E-07	0.040593229	0.803646	-0.142797538	0.282747	0.589303704	5.68E-09	0.413811962	0.00021	-0.180710404	0.235768	C2	-	Y	-	-	Y	Y	-
ENSG00000159307	SCUBE1	-0.565571427	1.07E-12	-0.162522497	0.092046	0.0252241	0.856889	0.409354262	5.68E-08	0.605207833	4.90E-15	0.190482682	0.077977	C4	-	Y	-	-	Y	Y	-
ENSG00000159346	ADIPOR1	0.38358441	5.12E-12	0.220414247	0.000755	0.224511282	0.000549	-0.156753767	0.005542	-0.144435383	0.01001	0.00707548	0.965719	C5	-	Y	-	-	-	-	-
ENSG00000159388	BTG2	-0.292652001	0.00922	0.088731795	0.566325	0.164532727	0.211611	0.387654568	0.000197	0.471628384	6.16E-06	0.07861883	0.694797	C4	-	-	-	-	Y	Y	-
ENSG00000159399	HK2	0.247417216	2.24E-06	0.631399473	7.61E-17	0.410003991	4.88E-07	0.390551439	1.26E-08	0.177526384	0.017181	-0.219642988	0.291851	C4	-	-	Y	Y	Y	-	-
ENSG00000159409	CELF3	0.419957622	5.79E-17	0.120769749	0.061257	0.170731041	0.008301	-0.292616182	5.74E-10	-0.234688936	3.29E-06	0.052912944	0.615724	C5	-	Y	-	-	-	-	-
ENSG00000159423	ALDH4A1	-0.241948895	0.125465	-0.072127342	0.758346	0.067623147	0.752276	0.176182991	0.28045	0.324474173	0.023679	0.142898833	0.552695	C4	-	-	-	-	-	Y	-
ENSG00000159433	STARD9	0.25332396	0.005877	0.243599464	0.018461	0.302291994	0.002204	-0.003125469	0.974391	0.0632564	0.450907	0.061078948	0.646006	C1	-	-	-	Y	-	-	-
ENSG00000159445	THEM4	-0.476431983	7.04E-08	-0.036430499	0.791552	-0.108005194	0.302456	0.446739778	1.67E-06	0.383196462	4.09E-05	-0.068711459	0.68278	C4	-	Y	-	-	Y	Y	-
ENSG00000159459	UBR1	0.488356023	3.87E-10	0.296587722	0.002737	0.349388425	6.27E-05	-0.185472466	0.007245	-0.124471234	0.060045	0.055631491	0.690042	C5	-	Y	-	Y	-	-	-
ENSG00000159556	ISL2	0.302439075	0.060422	-0.041626104	0.883357	0.129246092	0.57461	-0.337756911	0.043605	-0.158550331	0.406433	0.173546235	0.564344	C5	-	-	-	-	Y	-	-
ENSG00000159592	GPBP1L1	0.358877835	1.85E-09	0.066580551	0.462988	0.112578979	0.163043	-0.2860593	2.60E-08	-0.231739824	2.35E-05	0.048868012	0.661659	C5	-	Y	-	-	-	-	-
ENSG00000159640	ACE	-0.636026783	5.54E-08	-0.383960995	0.010156	-0.279471981	0.037977	0.258656081	0.088573	0.371274137	0.004183	0.107377573	0.681892	C4	-	Y	Y	-	-	Y	-
ENSG00000159658	EFCAB14	0.183189325	0.076864	-0.212977757	0.104974	0.042814963	0.779897	-0.389934219	2.46E-05	-0.126164498	0.185213	0.258707356	0.045236	C5	-	-	-	-	Y	-	-
ENSG00000159708	LRRC36	-1.24145044	0.012575	-0.586511476	0.320404	-0.178792793	0.783846	0.663040785	0.284219	1.077897311	0.042151	0.410646009	NA	C4	-	Y	-	-	-	Y	-
ENSG00000159713	TPPP3	-0.998263355	1.01E-11	-0.449803108	0.0072	-0.137523657	0.45559	0.555733999	0.001144	0.875769625	3.83E-09	0.315627303	0.12527	C3	-	Y	Y	-	Y	Y	-
ENSG00000159753	CARMIL2	0.146289327	0.175213	-0.202277943	0.106371	-0.175142384	0.168161	-0.341540977	6.88E-05	-0.306357795	0.000623	0.029870475	0.900692	C5	-	-	-	-	Y	Y	-
ENSG00000159792	PSKH1	-0.48019884	6.04E-09	-0.056036512	0.636996	-0.06667261	0.520778	0.430901408	2.22E-07	0.428236393	8.58E-08	-0.007577753	0.971304	C4	-	Y	-	-	Y	Y	-
ENSG00000159840	ZYX	1.137517894	4.13E-56	0.427896352	5.00E-06	0.322091156	0.000369	-0.703373086	2.36E-30	-0.80041202	1.75E-44	-0.102497719	0.396355	C5	-	Y	Y	Y	Y	Y	-
ENSG00000159873	CCDC117	0.754062026	1.71E-16	0.202372947	0.131645	0.595282239	1.96E-09	-0.544242174	3.56E-17	-0.144642897	0.029226	0.394487111	9.08E-07	C1	-	Y	-	Y	Y	-	Y
ENSG00000159882	ZNF230	0.206648748	0.154611	-0.466354355	0.011312	0.087878128	0.617883	-0.666924622	3.78E-05	-0.10401076	0.517465	0.556736419	0.003973	C5	-	-	Y	-	Y	-	Y
ENSG00000159899	NPR2	0.578485405	3.24E-06	-0.042729682	0.844725	0.222093877	0.197502	-0.615154131	1.02E-07	-0.342041469	0.011424	0.267541618	0.168713	C5	-	Y	-	-	Y	Y	-
ENSG00000159905	ZNF221	0.350518883	0.224581	0.315801105	0.35294	0.727369649	0.005382	-0.028957318	0.934046	0.391274453	0.102261	0.414558197	0.182122	C4	-	-	-	Y	-	-	-
ENSG00000159915	ZNF233	-0.137039026	0.4337	-0.395040802	0.021994	0.204586923	0.313839	-0.251525282	0.146258	0.35649637	0.03419	0.602584402	0.001856	C4	-	-	Y	-	-	Y	Y
ENSG00000160013	PTGIR	-0.530171003	0.127531	0.482237872	0.160852	0.490381581	0.082897	1.016968555	0.002332	1.035838334	0.000548	0.010884744	0.988517	C2	-	-	-	-	Y	Y	-
ENSG00000160014	CALM3	-0.2830096	5.56E-10	-0.110166766	0.096871	0.015427404	0.857403	0.179243835	1.04E-05	0.313272156	1.59E-19	0.128649597	0.029061	C4	-	-	-	-	-	Y	-
ENSG00000160049	DFFA	0.317314024	4.15E-08	0.133781491	0.069504	0.227961978	0.00031	-0.177007275	0.000125	-0.07471895	0.143543	0.096883597	0.1507	C5	-	Y	-	-	-	-	-
ENSG00000160058	BSDC1	0.433351871	2.39E-12	0.169812179	0.034843	0.16467119	0.038015	-0.257073654	1.21E-05	-0.254343056	1.57E-05	-0.002382164	0.991193	C5	-	Y	-	-	-	-	-
ENSG00000160062	ZBTB8A	-0.307115656	0.010656	-0.46688016	0.000637	-0.486728045	0.000125	-0.153193236	0.342035	-0.165022677	0.280895	-0.017217369	0.96555	C4	-	Y	Y	Y	-	-	-
ENSG00000160072	ATAD3B	0.335881065	2.27E-07	0.051537599	0.682833	-0.012392438	0.918869	-0.277935287	8.43E-05	-0.333524834	7.48E-08	-0.06116416	0.680246	C5	-	Y	-	-	-	Y	-
ENSG00000160097	FNDC5	-0.835098214	4.45E-05	-0.95026182	0.000108	-0.781827029	0.000215	-0.108573731	0.7685	0.067800258	0.843753	0.171035454	0.74831	C3	-	Y	Y	Y	-	-	-
ENSG00000160117	ANKLE1	0.45123292	6.23E-06	0.033580687	0.860577	0.19238672	0.149618	-0.411148852	0.000106	-0.244334586	0.032238	0.161635579	0.403617	C5	-	Y	-	-	Y	-	-
ENSG00000160145	KALRN	0.248227856	0.020638	0.239933878	0.061652	0.354786729	0.0009	-0.002246047	0.981699	0.120940793	0.056573	0.117845661	0.249889	C1	-	-	-	Y	-	-	-
ENSG00000160172	FAM86C2P	-0.811127357	7.29E-05	-0.294312619	0.254383	-0.311835884	0.19231	0.522635873	0.023516	0.514060606	0.02635	-0.01461387	0.979815	C4	-	Y	-	-	Y	Y	-
ENSG00000160179	ABCG1	-0.419887663	0.04775	-0.445458072	0.133828	-0.2492603	0.345344	-0.019199916	0.958286	0.185583803	0.457985	0.199265029	0.667843	C4	-	Y	-	-	-	-	-
ENSG00000160191	PDE9A	0.317503305	0.005223	0.013946509	0.952296	0.089141944	0.640651	-0.295865688	0.002091	-0.213401178	0.058424	0.077835432	0.738724	C5	-	Y	-	-	-	-	-
ENSG00000160193	WDR4	0.50335336	2.32E-05	0.21938062	0.160869	0.189783598	0.265277	-0.277762145	0.004441	-0.299446507	0.005945	-0.026990687	0.928789	C1	-	Y	-	-	-	-	-
ENSG00000160200	CBS	-0.633900599	0.000166	-0.485854125	0.015337	-0.41485872	0.294319	0.152408582	0.347733	0.233143932	0.246318	0.073238512	0.872221	C4	-	Y	Y	-	-	-	-
ENSG00000160201	U2AF1	0.018319009	0.949676	0.25363049	0.168386	-0.472543748	0.011594	0.241579457	0.221885	-0.476250326	0.020136	-0.723195022	9.06E-05	C2	-	-	-	Y	-	Y	Y
ENSG00000160208	RRP1B	0.421864291	1.27E-14	0.087996621	0.330977	0.001073987	0.992422	-0.326936389	1.48E-10	-0.406347426	2.35E-20	-0.084601437	0.36392	C5	-	Y	-	-	Y	Y	-
ENSG00000160214	RRP1	0.194647458	0.018925	-0.040231774	0.765087	-0.14727399	0.258117	-0.228402127	0.003575	-0.327102634	0.00057	-0.104427739	0.562859	C5	-	-	-	-	-	Y	-
ENSG00000160221	GATD3A	-0.340615752	0.011755	0.061119539	0.769037	0.021249387	0.924724	0.408158101	0.000485	0.376794031	0.002488	-0.03670474	0.899704	C4	-	Y	-	-	Y	Y	-
ENSG00000160233	LRRC3	-0.533260473	1.34E-07	-0.132055588	0.340714	0.057080359	0.682112	0.408106647	0.000378	0.605007565	1.96E-09	0.192193182	0.199209	C4	-	Y	-	-	Y	Y	-
ENSG00000160285	LSS	0.162982919	0.017169	0.206742699	0.018162	0.303821958	2.61E-06	0.050159732	0.596411	0.155510363	0.017148	0.099864988	0.410205	C4	-	-	-	Y	-	-	-
ENSG00000160298	C21orf58	-0.218697772	0.004817	0.116655223	0.278274	0.040240999	0.731151	0.341923716	1.73E-05	0.273438274	0.000334	-0.073475664	0.635328	C4	-	-	-	-	Y	-	-
ENSG00000160305	DIP2A	0.688779872	2.52E-16	0.401191244	2.27E-05	0.487541641	2.94E-08	-0.28102812	4.39E-06	-0.187239134	0.002698	0.088399353	0.390566	C5	-	Y	Y	Y	-	-	-
ENSG00000160307	S100B	-1.12417868	0.073045	-1.021368688	0.12207	-1.371673676	0.034192	0.123624541	0.911809	-0.236893248	0.834685	-0.364100718	NA	C3	-	-	-	Y	-	-	-
ENSG00000160323	ADAMTS13	-0.881626656	5.36E-22	-0.242887919	0.021994	-0.131920597	0.358839	0.644970063	7.66E-11	0.764759038	9.55E-12	0.114308229	0.583183	C4	-	Y	-	-	Y	Y	-
ENSG00000160325	CACFD1	-0.402434834	0.002372	-0.068626625	0.731342	-0.127079142	0.517444	0.340431369	0.007924	0.289854385	0.052442	-0.05553356	0.862177	C4	-	Y	-	-	Y	-	-
ENSG00000160326	SLC2A6	-0.304108758	0.055045	-0.074672652	0.754065	0.100317098	0.617704	0.236027334	0.176231	0.41906316	0.005598	0.177994636	0.464107	C4	-	-	-	-	-	Y	-
ENSG00000160345	C9orf116	-0.598478206	0.021376	-0.497213453	0.05994	-0.046797217	0.902366	0.107531767	0.776175	0.566579573	0.045255	0.453502726	0.199145	C4	-	Y	-	-	-	Y	-
ENSG00000160352	ZNF714	0.17486217	0.081236	-0.155853299	0.283383	-0.081002568	0.546322	-0.324446707	6.29E-05	-0.241528318	0.000175	0.077718324	0.639257	C5	-	-	-	-	Y	-	-
ENSG00000160392	C19orf47	0.324523964	0.006952	0.210673445	0.202839	0.068200993	0.73177	-0.107113059	0.402517	-0.242272062	0.03437	-0.140055098	0.50724	C1	-	Y	-	-	-	-	-
ENSG00000160439	RDH13	0.377975751	0.004664	0.308674827	0.056009	0.415947086	0.004308	-0.062910999	0.721711	0.05249726	0.767224	0.109990741	0.659889	C1	-	Y	-	Y	-	-	-
ENSG00000160446	ZDHHC12	-0.517035836	0.000387	-0.149888001	0.50405	-0.128931957	0.543319	0.373557182	0.033895	0.402898262	0.017267	0.024686901	0.958405	C4	-	Y	-	-	Y	Y	-
ENSG00000160447	PKN3	-0.287234832	0.012433	0.024732019	0.892304	0.022033671	0.907515	0.318613205	0.005325	0.323858669	0.007702	-7.73E-05	0.999689	C4	-	-	-	-	Y	Y	-
ENSG00000160551	TAOK1	0.438226981	0.000908	0.373960168	0.000378	0.3237117	0.030899	-0.058157609	0.480457	-0.100110477	0.117583	-0.047268864	0.749431	C1	-	Y	Y	Y	-	-	-
ENSG00000160563	MED27	0.431538012	0.000127	0.214967888	0.176645	0.168955857	0.278718	-0.209126429	0.062128	-0.247920967	0.019368	-0.043657157	0.872221	C5	-	Y	-	-	-	-	-
ENSG00000160570	DEDD2	0.503065792	7.81E-05	0.206826208	0.238111	0.518404804	0.000282	-0.289811828	0.013899	0.029977222	0.85889	0.314444189	0.044765	C1	-	Y	-	Y	-	-	Y
ENSG00000160584	SIK3	0.479064363	9.31E-13	0.191162281	0.012563	0.187351612	0.023568	-0.281542373	1.41E-05	-0.277285493	6.20E-05	-0.001027637	0.996048	C5	-	Y	-	-	-	-	-

ENSG00000160602	NEK8	-0.678279966	0.001409	-0.206663317	0.427814	-0.476031655	0.031193	0.477822276	0.046653	0.216898301	0.455347	-0.266272139	0.450835	C3	-	Y	-	Y	Y	-	-
ENSG00000160685	ZBTB7B	0.211703796	0.020801	0.177509169	0.167383	0.458501591	2.77E-07	-0.027900715	0.840008	0.26150289	0.001794	0.284279707	0.018512	C4	-	-	-	Y	-	-	-
ENSG00000160688	FLAD1	-0.219153122	0.002134	-0.296781278	0.000352	-0.327345807	5.02E-05	-0.070335945	0.33341	-0.093481457	0.184085	-0.028118117	0.862412	C4	-	-	-	Y	-	-	-
ENSG00000160703	NLRX1	-0.96814916	6.13E-14	-0.321509691	0.020712	-0.188332831	0.191372	0.653353218	5.18E-06	0.79464725	7.12E-09	0.136130245	0.540844	C2	-	Y	Y	-	Y	Y	-
ENSG00000160741	CRTC2	0.388171174	1.65E-10	0.087975204	0.351677	0.221600828	0.001128	-0.293638385	9.27E-06	-0.151956012	0.017758	0.136601555	0.163271	C5	Y	Y	-	-	-	-	-
ENSG00000160746	ANO10	-0.082758335	0.560037	0.136229024	0.385703	0.267863206	0.028565	0.225652586	0.08266	0.365331194	0.001408	0.134631455	0.500085	C4	-	-	-	-	-	Y	-
ENSG00000160767	FAM189B	0.215897534	0.002034	0.198900703	0.014509	0.303770759	0.00018	-0.010574092	0.911819	0.102418444	0.179437	0.107697934	0.347227	C4	-	-	-	Y	-	-	-
ENSG00000160781	PAQR6	-1.244002941	1.00E-30	0.023464519	0.899382	-0.227637196	0.159586	1.274231851	3.16E-36	1.031512793	2.35E-15	-0.248072691	0.182394	C3	-	Y	-	-	Y	Y	-
ENSG00000160783	PMF1	-0.228100445	0.044144	-0.100442216	0.562707	-0.399368559	0.000749	0.134322599	0.239208	-0.156179898	0.141864	-0.296023476	0.025025	C4	-	-	-	Y	-	-	-
ENSG00000160799	CCDC12	-0.317251048	0.000132	-0.357116627	0.000161	-0.484352742	7.55E-08	-0.03312048	0.776268	-0.152155455	0.103405	-0.124395473	0.389646	C4	-	Y	Y	Y	-	-	-
ENSG00000160801	PTH1R	-1.682572147	3.20E-06	-0.485123058	0.205133	-0.406045428	0.310854	1.205272812	0.002957	1.291017454	0.001815	0.081765938	NA	C2	-	Y	-	-	Y	Y	-
ENSG00000160808	MYL3	-2.628937857	0.000193	-0.36689813	0.64527	-0.093124191	0.924623	2.27493706	0.00554	2.551657758	0.002473	0.271522794	NA	C3	-	Y	-	-	Y	Y	-
ENSG00000160818	GPATCH4	0.165479705	0.004965	-0.006380111	0.96137	-0.182311205	0.010044	-0.16478708	0.002503	-0.333026058	1.04E-11	-0.173268952	0.024817	C5	-	-	-	-	-	Y	-
ENSG00000160867	FGFR4	-0.782469117	1.31E-05	-0.543209936	0.003087	-0.310176724	0.095581	0.246084619	0.247313	0.487633279	0.006409	0.23611901	0.337572	C4	-	Y	Y	-	-	Y	-
ENSG00000160883	HK3	-0.920195848	6.41E-05	-0.049009549	0.908741	-0.022638423	0.95385	0.87886467	0.001163	0.912669614	0.000238	0.028049711	0.968389	C2	-	Y	-	-	Y	Y	-
ENSG00000160888	IER2	0.371932463	7.43E-07	0.220304125	0.014554	0.320503528	0.000215	-0.145381934	0.060709	-0.036817198	0.714209	0.103156269	0.421518	C5	-	Y	-	Y	-	-	-
ENSG00000160917	CPSF4	-0.358066916	2.39E-07	-0.232923041	0.010188	-0.271977677	0.000894	0.132122855	0.043217	0.10056464	0.118324	-0.036542378	0.809023	C4	-	Y	-	-	-	-	-
ENSG00000161010	MRNIP	0.362711829	2.80E-06	0.310382256	0.00046	0.307417893	0.000339	-0.046200021	0.618936	-0.040463109	0.668831	0.000180271	0.999291	C5	-	Y	Y	Y	-	-	-
ENSG00000161021	MAML1	0.319371516	1.07E-07	0.09793817	0.248685	0.056746856	0.602371	-0.215140715	3.58E-05	-0.248421076	4.18E-05	-0.038451575	0.793512	C5	-	Y	-	-	-	-	-
ENSG00000161149	TUBA3FP	0.075633822	0.722375	0.39088077	0.030737	0.300160001	0.144018	0.322614368	0.014029	0.239241138	0.127558	-0.088334161	0.759011	C4	-	-	Y	-	Y	-	-
ENSG00000161179	YDJC	-0.530608223	5.10E-06	-0.172457083	0.291293	-0.417164296	0.000612	0.364568031	0.005922	0.128289711	0.390572	-0.241799903	0.183357	C4	-	Y	-	Y	Y	-	-
ENSG00000161202	DVL3	0.384702478	5.57E-18	0.159959264	0.006693	0.112613179	0.031169	-0.218418462	2.38E-07	-0.257531065	5.58E-13	-0.044508032	0.592688	C5	-	Y	-	-	-	-	-
ENSG00000161217	PCYT1A	0.464561341	1.45E-12	0.116180871	0.226885	0.127036334	0.147006	-0.342032491	1.95E-07	-0.322785335	3.15E-07	0.013969957	0.943025	C5	-	Y	-	-	Y	Y	-
ENSG00000161265	U2AF1L4	-0.170177578	0.290327	-0.096871798	0.709908	-0.355615088	0.041976	0.07936907	0.699812	-0.170462205	0.321082	-0.255853302	0.334959	C4	-	-	-	Y	-	-	-
ENSG00000161267	BDH1	-0.27120707	0.000437	0.090311046	0.445556	-0.099743641	0.330958	0.368210377	2.39E-06	0.18627398	0.020019	-0.187229751	0.090107	C4	-	-	-	-	Y	-	-
ENSG00000161328	LRRC56	-1.497866736	2.06E-08	-0.52469918	0.056326	-0.302108047	0.346169	0.97972757	0.00166	1.210100542	0.000111	0.225117253	0.661685	C3	-	Y	-	-	Y	Y	-
ENSG00000161509	GRIN2C	-0.277770275	0.396159	-0.423414711	0.23441	-0.775069019	0.01798	-0.13986163	0.737013	-0.4820186	0.187349	-0.347597922	NA	C4	-	-	-	Y	-	-	-
ENSG00000161513	FDXR	-0.426106099	2.07E-06	-0.155225727	0.223268	-0.19308577	0.076578	0.277230952	0.006339	0.247552779	0.011655	-0.034896898	0.889019	C4	-	Y	-	-	-	-	-
ENSG00000161544	CYGB	-0.120971037	0.077641	0.196210656	0.008346	-0.014254327	0.901217	0.32372069	1.55E-08	0.121431816	0.064479	-0.207644454	0.005582	C4	-	-	-	-	Y	-	-
ENSG00000161638	ITGA5	0.881960775	0.368122	1.39191024	NA	1.770201217	0.027719	0.512003369	0.539282	0.901693712	0.170933	0.387209543	NA	C2	-	-	-	Y	-	-	-
ENSG00000161642	ZNF385A	-0.680545351	8.11E-10	-0.013873001	0.945256	0.139342722	0.279346	0.67351475	6.66E-09	0.834526939	8.69E-15	0.156159386	0.363162	C2	Y	Y	-	-	Y	Y	-
ENSG00000161647	MPP3	-0.367766182	0.000182	-0.245178482	0.069796	-0.06866595	0.665438	0.129132355	0.328063	0.313418636	0.003469	0.1797362	0.343347	C4	-	Y	-	-	-	Y	-
ENSG00000161677	JOSD2	-0.396816793	0.015996	-0.051656806	0.847814	-0.126040657	0.578694	0.351461034	0.023096	0.286080357	0.087387	-0.070807166	0.833312	C4	-	Y	-	-	Y	-	-
ENSG00000161681	SHANK1	0.433581158	9.67E-07	0.208277948	0.087381	0.345103633	0.000212	-0.21884504	0.012211	-0.073832804	0.410281	0.139232374	0.286212	C5	-	Y	-	Y	-	-	-
ENSG00000161791	FMNL3	0.503832018	1.37E-08	0.207556055	0.07463	0.235329565	0.024215	-0.289246719	2.38E-05	-0.254132259	0.000114	0.029740629	0.860457	C5	-	Y	-	-	-	-	-
ENSG00000161813	LARP4	0.656921805	8.38E-16	0.068260253	0.627026	0.23353338	0.017203	-0.581701089	5.19E-32	-0.409271522	1.86E-18	0.167199326	0.023012	C5	-	Y	-	-	Y	Y	-
ENSG00000161835	TAMALIN	-0.696783633	0.005267	-0.143857057	0.684954	-0.174393646	0.599935	0.559797631	0.050666	0.537025203	0.071495	-0.028433678	0.967582	C3	-	Y	-	-	-	-	-
ENSG00000161904	LEMD2	0.495030201	1.36E-17	0.121176159	0.154011	0.140279629	0.071562	-0.367203465	6.03E-11	-0.340003851	7.16E-10	0.021901257	0.891955	C5	-	Y	-	-	Y	Y	-
ENSG00000161960	EIF4A1	-0.262957319	0.023376	0.222166506	0.089937	-0.072741285	0.6833	0.491366229	1.01E-05	0.20489909	0.136276	-0.291965239	0.089669	C4	-	-	-	-	Y	-	-
ENSG00000161980	POLR3K	-0.061934888	0.646969	-0.205034916	0.14759	-0.307380915	0.005769	-0.136625899	0.300662	-0.230630918	0.032646	-0.099491422	0.63838	C4	-	-	-	Y	-	-	-
ENSG00000162004	CCDC78	-0.24122325	0.073247	0.072283446	0.733489	-0.013792172	0.948703	0.319642644	0.011481	0.242046253	0.047119	-0.083006466	0.729263	C4	-	-	-	-	Y	-	-
ENSG00000162066	AMDHD2	-0.31201089	0.006112	-0.091230099	0.62132	-0.131418396	0.377222	0.227663925	0.068088	0.195450285	0.102902	-0.037656915	0.899023	C4	-	Y	-	-	-	-	-
ENSG00000162068	NTN3	-0.791111905	0.0018	0.009559245	0.985258	0.139481829	0.673281	0.807704211	0.007156	0.945629384	0.000244	0.132760783	0.822625	C4	-	Y	-	-	Y	Y	-
ENSG00000162086	ZNF75A	-0.480099045	6.07E-10	-0.104543956	0.469024	-0.118322045	0.260524	0.38199013	0.000213	0.376385888	1.46E-05	-0.010291974	0.972936	C4	-	Y	-	-	Y	Y	-
ENSG00000162105	SHANK2	-0.417721308	8.68E-06	-0.179759006	0.14236	0.015033115	0.928351	0.244217822	0.012002	0.447480907	5.51E-07	0.197755582	0.132933	C4	-	Y	-	-	-	Y	-
ENSG00000162144	CYB561A3	-0.306916379	0.004932	0.003942561	0.983497	-0.051926506	0.717324	0.31780771	0.004076	0.26990231	0.01418	-0.052802669	0.80132	C4	-	Y	-	-	Y	-	-
ENSG00000162148	PPP1R32	1.051999763	0.002289	0.658528488	0.242637	0.478304384	0.346575	-0.388428098	0.365554	-0.558573321	0.111158	-0.177873093	NA	C1	-	Y	-	-	-	-	-
ENSG00000162188	GNG3	-0.776499846	1.72E-05	-0.648220788	0.004487	-0.684333368	0.000378	0.136135885	0.654852	0.106998407	0.709855	-0.033835686	0.957248	C4	-	Y	Y	Y	-	-	-
ENSG00000162222	TTC9C	0.698739165	8.99E-14	0.287389485	0.016061	0.374502493	0.001131	-0.404960938	1.65E-05	-0.309769319	0.001822	0.089932258	0.649926	C5	-	Y	-	Y	Y	Y	-
ENSG00000162227	TAF6L	-0.238759995	0.036202	-0.18719999	0.192849	-0.365894128	0.003073	0.058772736	0.647061	-0.112589892	0.345377	-0.176529663	0.260273	C4	-	-	-	Y	-	-	-
ENSG00000162231	NXF1	0.475113253	7.54E-17	0.056311294	0.572247	0.068662707	0.497988	-0.41204808	3.15E-17	-0.39215154	1.46E-12	0.015112852	0.933843	C5	-	Y	-	-	Y	Y	-
ENSG00000162241	SLC25A45	-0.827716632	6.14E-06	-0.209869708	0.407344	-0.105370846	0.66083	0.62474005	0.001169	0.73702487	1.14E-05	0.108901057	0.75081	C4	-	Y	-	-	Y	Y	-
ENSG00000162298	SYVN1	0.467191699	9.18E-11	0.153649682	0.17088	0.130552543	0.108785	-0.307269846	0.000465	-0.321993733	4.78E-06	-0.020314322	0.930809	C5	-	Y	-	-	Y	Y	-
ENSG00000162300	ZFPL1	-0.330272466	0.036167	-0.14055804	0.555811	-0.15360382	0.493494	0.196153114	0.302525	0.191475012	0.32139	-0.01025544	0.982455	C4	-	Y	-	-	-	-	-
ENSG00000162302	RPS6KA4	0.466561358	0.000124	0.205024016	0.276995	0.360551188	0.011827	-0.254862038	0.063201	-0.091833889	0.528037	0.158323867	0.497876	C1	-	Y	-	Y	-	-	-
ENSG00000162337	LRP5	0.349200087	6.53E-10	0.268879895	3.79E-05	0.321295062	6.32E-08	-0.073773606	0.26857	-0.013244784	0.869531	0.055096681	0.604334	C5	-	Y	-	Y	-	-	-
ENSG00000162341	TPCN2	-0.28152206	0.035527	0.173785107	0.322857	-0.109897489	0.544773	0.461718389	0.000971	0.186299016	0.245905	-0.280466991	0.163987	C4	-	-	-	-	Y	-	-
ENSG00000162378	ZYG11B	0.481330563	2.76E-09	0.188689412	0.131693	0.217418849	0.030022	-0.285714426	9.95E-05	-0.249400213	3.49E-05	0.031047221	0.877754	C5	-	Y	-	-	-	-	-
ENSG00000162390	ACOT11	-1.076206616	4.41E-27	-0.27835982	0.02224	-0.17003895	0.12677	0.805065032	1.10E-11	0.920891075	2.82E-18	0.111127952	0.576984	C2	-	Y	-	-	Y	Y	-
ENSG00000162391	FAM151A	-1.532281193	0.000616	-0.368487832	0.448006	-0.913885291	0.034529	1.171319159	0.020426	0.633686239	0.316611	-0.542982697	NA	C3	-	Y	-	Y	Y	-	-
ENSG00000162396	PARS2	0.356582389	0.045052	-0.037327906	0.911609	0.241548873	0.305997	-0.387579365	0.041218	-0.100358473	0.664445	0.281530088	0.371123	C5	-	Y	-	-	Y	-	-
ENSG00000162402	USP24	0.479976889	7.63E-12	0.25738985	0.002878	0.264066346	0.00048	-0.216178406	0.000908	-0.20136778	0.000511	0.009434872	0.958587	C1	-	Y	-	-	-	-	-
ENSG00000162407	PLPP3	-1.207391511	8.34E-12	-1.07006253	8.99E-10	-1.055424478	3.92E-11	0.142953947	0.637925	0.166292092	0.550386	0.017602416	0.976804	C3	-	Y	Y	Y	-	-	-
ENSG00000162408	NOL9	-0.060327749	0.562844	-0.259796751	0.010521	-0.302478222	0.001053	-0.193032996	0.04498	-0.227635387	0.011991	-0.039833722	0.857353	C4	-	-	-	Y	-	-	-
ENSG00000162413	KLHL21	0.242637389	0.044005	-0.17310231	0.305307	-0.268466215	0.093385	-0.408961087	0.000525	-0.496803165	6.97E-05	-0.091793069	0.755306	C5	-	-	-	-	Y	Y	-
ENSG00000162426	SLC45A1	0.366205825	0.204043	0.399251942	0.182307	0.580050937	0.022961	0.040487418	0.914221	0.228133684	0.43183	0.184301063	0.668335	C1	-	-	-	Y	-	-	-
ENSG00000162433	AK4	-0.329407305	0.000585	0.089609717	0.414648	0.052973654	0.696123	0.425434923	1.26E-06	0.396954923	4.61E-05	-0.033728739	0.871903	C4	-	Y	-	-	Y	Y	-

ENSG00000162434	JAK1	0.195719926	0.001973	0.353946301	6.96E-09	0.332365766	5.73E-08	0.164600963	0.000923	0.151173499	0.002344	-0.018696736	0.876139	C4	-	-	Y	Y	-	-	-
ENSG00000162437	RAVER2	0.408323057	1.71E-09	0.223224662	0.007561	0.276895494	0.000249	-0.178962158	0.002416	-0.11679365	0.053853	0.056715973	0.61262	C1	-	Y	-	-	-	-	-
ENSG00000162461	SLC25A34	-0.555077323	0.017968	-0.234873511	0.47911	-0.166333993	0.61583	0.326700345	0.223355	0.403989686	0.115651	0.071384112	0.905184	C4	-	Y	-	-	-	-	-
ENSG00000162490	DRAXIN	-0.742233288	0.001331	-0.476819068	0.06856	-0.307608228	0.240991	0.27038184	0.365546	0.448897841	0.083606	0.171884811	0.705397	C4	-	Y	-	-	-	-	-
ENSG00000162522	KIAA1522	-0.423124026	0.000103	-0.116108862	0.415194	0.012206635	0.945858	0.313403796	0.005464	0.449968314	2.66E-05	0.131105212	0.450835	C4	-	Y	-	-	Y	Y	-
ENSG00000162542	TMCO4	-0.815563729	0.049347	-0.387722606	0.451649	-0.27823465	0.654742	0.434372459	0.386437	0.552004705	0.314505	0.112753168	NA	C4	-	Y	-	-	-	-	-
ENSG00000162551	ALPL	-0.35655494	0.247616	-0.943644368	0.004085	-0.856807404	0.005868	-0.580587311	0.062477	-0.485301303	0.116671	0.090169606	NA	C3	-	-	Y	Y	-	-	-
ENSG00000162572	SCNN1D	-0.734889223	0.000116	-0.085748022	0.775916	-0.21271126	0.333602	0.656994676	0.001431	0.537343812	0.007367	-0.123515082	0.73559	C4	-	Y	-	-	Y	Y	-
ENSG00000162576	MXRA8	-0.69974108	4.50E-07	0.053909371	0.814161	0.254529969	0.105073	0.76027021	5.02E-08	0.969228823	5.56E-13	0.203656631	0.330948	C4	-	Y	-	-	Y	Y	-
ENSG00000162591	MEGF6	-0.509284835	1.46E-09	-0.435396331	4.08E-06	-0.177233371	0.063308	0.079980548	0.531064	0.34679474	0.000153	0.261054028	0.031355	C4	-	Y	Y	-	-	Y	-
ENSG00000162592	CCDC27	-0.769442422	0.158083	0.476147875	0.379507	0.302673955	0.605903	1.251831556	0.008168	1.085654539	0.030893	-0.169952592	NA	C2	-	-	-	-	Y	Y	-
ENSG00000162599	NFIA	-1.725435764	2.17E-05	-0.370101619	0.435026	-0.30049118	0.580089	1.363069264	0.003197	1.440547122	0.00407	0.072292114	NA	C3	-	Y	-	-	Y	Y	-
ENSG00000162600	OMA1	0.56474215	0.001211	0.370680488	0.089313	0.648194835	0.000526	-0.187615537	0.356579	0.098609695	0.657382	0.280842524	0.286255	C4	-	Y	-	Y	-	-	-
ENSG00000162607	USP1	-0.13617359	0.014962	-0.196256194	0.013477	-0.335112454	1.98E-08	-0.053328356	0.459271	-0.184650748	0.00012	-0.136101726	0.148991	C4	-	-	-	Y	-	-	-
ENSG00000162613	FUBP1	0.283410979	9.58E-08	0.003827605	0.971287	-0.04877693	0.515299	-0.273358753	1.47E-12	-0.317608986	1.34E-18	-0.049530556	0.471971	C5	-	-	-	-	-	Y	-
ENSG00000162616	DNAJB4	0.47358792	3.38E-06	0.293313545	0.031796	0.547209351	9.66E-08	-0.173685075	0.16111	0.088207285	0.458559	0.256624925	0.077615	C4	-	Y	-	Y	-	-	-
ENSG00000162623	TYW3	0.203149922	0.011371	-0.194522821	0.040002	0.00433696	0.976467	-0.391363346	1.10E-07	-0.18431597	0.022107	0.201731179	0.061412	C5	-	-	-	-	Y	-	-
ENSG00000162650	ATXN7L2	0.06889289	0.653248	-0.186367043	0.22524	-0.258619164	0.064812	-0.248576049	0.02239	-0.312475155	0.003946	-0.069341407	0.754209	C5	-	-	-	-	-	Y	-
ENSG00000162669	HFM1	-0.419777746	0.008426	0.258712687	0.092052	0.011446968	0.961099	0.685508791	1.57E-06	0.445914477	0.004122	-0.244126396	0.175445	C2	-	Y	-	-	Y	Y	-
ENSG00000162687	KCNT2	-0.133928776	0.184379	-0.055250177	0.703255	0.31975964	0.001163	0.084832709	0.349397	0.468358226	3.15E-10	0.37797912	9.76E-06	C4	-	-	-	Y	-	Y	Y
ENSG00000162695	SLC30A7	0.334744313	0.000165	-0.034087236	0.826211	-0.108301022	0.456133	-0.362380545	4.64E-05	-0.42820222	2.07E-05	-0.071204354	0.754039	C5	-	Y	-	-	Y	Y	-
ENSG00000162702	ZNF281	0.147890009	0.081348	-0.231761306	0.036343	-0.043309938	0.748617	-0.372842109	8.03E-08	-0.177281874	0.007197	0.190962633	0.106348	C5	-	-	-	-	Y	-	-
ENSG00000162706	CADM3	-0.549750493	1.11E-08	-0.148885212	0.409995	-0.13189929	0.302844	0.408174193	0.003973	0.432423959	8.96E-05	0.018963277	0.959684	C4	-	Y	-	-	Y	Y	-
ENSG00000162728	KCNJ9	-0.253056717	0.013488	0.106747235	0.411083	0.130691982	0.232015	0.366326754	0.000403	0.398616388	3.37E-05	0.026911573	0.910221	C4	-	-	-	-	Y	Y	-
ENSG00000162733	DDR2	-0.94802184	2.89E-42	-0.277309297	0.000583	-0.02759305	0.797995	0.676970407	1.14E-36	0.934798161	1.03E-99	0.252711122	3.18E-06	C4	-	Y	-	-	Y	Y	-
ENSG00000162755	KLHDC9	-0.869890456	0.027171	-0.151673054	0.785828	0.029809897	0.959667	0.729046482	0.11582	0.91402114	0.036491	0.181810045	NA	C3	-	Y	-	-	-	Y	-
ENSG00000162769	FLVCR1	0.611892907	3.23E-17	0.207522161	0.027956	0.276529095	0.001091	-0.398129191	4.19E-12	-0.320897224	1.42E-08	0.071801536	0.537888	C5	-	Y	-	-	Y	Y	-
ENSG00000162772	ATF3	-1.554648748	2.43E-16	-0.077857575	0.818748	-0.200832595	0.378502	1.482554759	3.26E-12	1.369043025	9.06E-13	-0.119908651	0.775214	C3	-	Y	-	-	Y	Y	-
ENSG00000162804	SNED1	0.311480722	0.200205	-0.098858725	0.815279	-0.391270415	0.190541	-0.404541487	0.11025	-0.687540435	0.003298	-0.289440256	NA	C5	-	-	-	-	-	Y	-
ENSG00000162813	BPNT1	0.414018829	8.84E-09	0.244211112	0.00451	0.229379635	0.005807	-0.163389518	0.040733	-0.169907966	0.027705	-0.011905153	0.954624	C5	-	Y	-	-	-	-	-
ENSG00000162817	C1orf115	-1.530187722	8.15E-10	-0.907165009	0.000257	-0.676460839	0.012349	0.629933676	0.048753	0.868630264	0.005854	0.233394226	NA	C3	-	Y	Y	Y	Y	Y	-
ENSG00000162836	ACP6	0.109448541	0.509678	0.50662204	0.0014	0.595909394	9.86E-07	0.404603162	0.01027	0.501277529	8.38E-05	0.092439267	0.752547	C4	-	-	Y	Y	Y	Y	-
ENSG00000162849	KIF26B	-0.025059225	0.810319	0.357027523	6.00E-06	0.128215285	0.155895	0.388577089	1.98E-10	0.167837996	0.085178	-0.225974013	0.004576	C4	-	-	Y	-	Y	-	-
ENSG00000162851	TFB2M	-0.15188831	0.021095	-0.183825068	0.013437	-0.376850883	9.42E-09	-0.025478322	0.776621	-0.210217281	0.001188	-0.190065272	0.021956	C4	-	-	-	Y	-	-	-
ENSG00000162852	CNST	0.395556362	3.30E-13	0.183905856	0.011467	0.24842708	0.000144	-0.205181372	0.000506	-0.132223373	0.026314	0.067457688	0.553292	C5	-	Y	-	-	-	-	-
ENSG00000162869	PPP1R21	-0.080597286	0.602962	0.040663374	0.8498	0.242328555	0.172076	0.127380926	0.341918	0.338057908	0.015373	0.204218619	0.351557	C4	-	-	-	-	-	Y	-
ENSG00000162873	KLHDC8A	1.640152915	0.019536	1.033194319	NA	0.485094085	NA	-0.604468003	0.358108	-1.136880547	0.084768	-0.540992991	NA	C5	-	Y	-	-	-	-	-
ENSG00000162889	MAPKAPK2	0.545966948	1.50E-27	0.290143307	2.50E-06	0.229968147	0.00022	-0.249710822	1.82E-07	-0.301205327	1.14E-10	-0.057183067	0.574972	C1	Y	Y	-	-	-	Y	-
ENSG00000162894	FCMR	-1.001481892	5.12E-05	-0.364527464	0.271278	-0.259209681	0.360311	0.645079079	0.036038	0.75731464	0.003872	0.108708346	0.853543	C2	-	Y	-	-	Y	Y	-
ENSG00000162924	REL	0.978374455	0.020342	0.743141705	0.146007	0.995989533	0.028354	-0.232159385	0.118085	0.030588004	0.868043	0.257548596	0.182027	C1	-	Y	-	Y	-	-	-
ENSG00000162946	DISC1	-0.811983149	0.007635	0.037559479	0.940676	0.094556717	0.820572	0.857483042	0.007331	0.920395721	0.002713	0.059391154	0.936471	C4	-	Y	-	-	Y	Y	-
ENSG00000162959	MEMO1	0.48526389	0.000405	0.367289296	0.014074	0.297121356	0.053601	-0.112031112	0.520154	-0.173442626	0.282727	-0.06715204	0.82887	C5	-	Y	Y	-	-	-	-
ENSG00000162972	MAIP1	0.316911341	0.06406	-0.069782675	0.807046	-0.023434813	0.934033	-0.380217191	0.026162	-0.325634231	0.056091	0.049153843	0.909908	C5	-	-	-	-	Y	-	-
ENSG00000162976	SLC66A3	-0.91143227	0.000448	-0.378375287	0.330252	-0.302995314	0.413954	0.54143525	0.124453	0.623821598	0.058004	0.075214497	0.933843	C3	-	Y	-	-	-	-	-
ENSG00000162989	KCNJ3	-0.847706068	6.59E-05	-0.301752626	0.274229	-0.237514397	0.443182	0.551567228	0.008958	0.624872139	0.006966	0.067150982	0.905466	C4	-	Y	-	-	Y	Y	-
ENSG00000162998	FRZB	-1.444049807	0.00016	-0.955232494	0.025226	-0.833252463	0.040884	0.492896605	0.375771	0.625649357	0.225525	0.126294548	NA	C3	-	Y	Y	Y	-	-	-
ENSG00000163012	ZSWIM2	-4.492901145	0.001259	-1.349270259	NA	-0.548213795	NA	3.173685809	NA	3.957495279	0.014511	0.796185093	NA	C3	-	Y	-	-	-	Y	-
ENSG00000163017	ACTG2	1.620757505	0.007794	0.648862699	NA	0.577133787	NA	-0.964246809	0.139545	-1.028305027	0.117973	-0.068489194	NA	C1	-	Y	-	-	-	-	-
ENSG00000163026	WDCP	0.33667356	2.02E-07	-0.254189132	0.004285	0.0765421	0.426753	-0.584168716	1.28E-18	-0.245736212	7.51E-05	0.333498537	9.06E-05	C5	-	Y	-	-	Y	-	Y
ENSG00000163029	SMC6	0.304590131	5.47E-07	0.224709086	0.001711	0.11821445	0.105932	-0.073290963	0.229125	-0.172024204	0.000488	-0.104058404	0.182771	C5	-	Y	-	-	-	-	-
ENSG00000163032	VSNL1	-0.31074478	0.327356	0.21974549	0.558823	0.352876835	0.289098	0.536151618	0.072309	0.678420507	0.021432	0.136701448	0.817627	C4	-	-	-	-	-	Y	-
ENSG00000163040	CCDC74A	-0.315345833	0.004965	0.000834793	0.99697	-0.092503551	0.556719	0.323297789	0.000411	0.237885155	0.015947	-0.090880269	0.585849	C4	-	Y	-	-	Y	-	-
ENSG00000163053	SLC16A14	-0.812352983	1.03E-08	-0.364719996	0.015711	-0.182010136	0.26846	0.454587041	0.006804	0.644882015	4.23E-05	0.185415999	0.439885	C4	-	Y	Y	-	Y	Y	-
ENSG00000163082	SGPP2	-1.533949435	5.13E-10	-0.003976618	0.992169	-0.501298746	0.05699	1.535753959	7.46E-10	1.04675779	0.000416	-0.493750248	0.127958	C3	-	Y	-	-	Y	Y	-
ENSG00000163110	PDLIM5	0.48364621	1.47E-08	0.243440971	0.059137	0.269920262	0.009714	-0.233643989	0.026489	-0.198954009	0.031853	0.029297908	0.915306	C5	Y	Y	-	-	-	-	-
ENSG00000163155	LYSMD1	0.492931736	3.39E-11	-0.006491081	0.974746	0.204508613	0.02915	-0.493241612	1.91E-07	-0.273628871	0.000378	0.213937661	0.157559	C5	-	Y	-	-	Y	-	-
ENSG00000163161	ERCC3	0.369135048	1.76E-12	-0.014276734	0.893156	0.00308954	0.976641	-0.376775085	1.74E-18	-0.351186929	1.09E-16	0.020220522	0.862177	C5	-	Y	-	-	Y	Y	-
ENSG00000163191	S100A11	1.234327746	0.018626	1.520520531	0.003475	1.567876785	0.000833	0.292489432	0.592782	0.348016081	0.477188	0.053477689	NA	C1	-	Y	Y	Y	-	-	-
ENSG00000163291	PAQR3	0.307244682	3.80E-06	0.082745005	0.376047	0.132660729	0.122705	-0.218117715	0.000356	-0.159861454	0.016591	0.052858128	0.67488	C5	-	Y	-	-	-	-	-
ENSG00000163322	ABRAXAS1	-0.463221148	0.001063	-0.099286712	0.664591	0.058802143	0.790316	0.371307476	0.030506	0.536918944	0.000553	0.160331129	0.585621	C4	-	Y	-	-	Y	Y	-
ENSG00000163328	GPR155	-0.072059716	0.62414	0.128671659	0.456735	0.254465378	0.078888	0.207416333	0.136879	0.341343325	0.0087	0.12787684	0.604908	C4	-	-	-	-	-	Y	-
ENSG00000163344	PMVK	-0.47136823	3.72E-06	-0.285899527	0.022095	-0.256392405	0.029649	0.191696754	0.021798	0.23027988	0.002586	0.033124489	0.856647	C4	Y	Y	-	-	-	-	-
ENSG00000163347	CLDN1	5.185134477	0.0015	4.087084632	NA	4.321414087	NA	-1.07094247	0.468533	-0.84300249	0.541741	0.220544956	NA	C5	-	Y	-	-	-	-	-
ENSG00000163349	HIPK1	0.503016006	3.33E-10	0.110063323	0.377234	0.226877814	0.021245	-0.386509892	2.64E-14	-0.26153196	1.71E-07	0.119549673	0.183357	C5	-	Y	-	-	Y	-	-
ENSG00000163354	DCST2	-0.848850681	0.098113	-0.001971286	0.997613	0.282200663	0.631701	0.854397172	0.096	1.14577281	0.016281	0.287331497	NA	C3	-	-	-	-	-	Y	-
ENSG00000163357	DCST1	-1.059051003	0.000475	-0.578491211	0.133658	-0.93628636	0.006209	0.486784817	0.248857	0.137973468	0.793485	-0.354299745	NA	C3	-	Y	-	Y	-	-	-
ENSG00000163359	COL6A3	0.498469715	0.309269	1.857275351	1.87E-07	1.865551673	2.33E-08	1.364592319	5.43E-06	1.381514078	1.92E-06	0.011423493	0.98733	C2	-	-	Y	Y	Y	Y	-

ENSG00000163362	INAVA	-1.032450258	9.75E-06	-0.345750157	0.313631	-0.306441607	0.240576	0.692675383	0.022284	0.740609478	0.003031	0.041949489	0.952524	C3	-	Y	-	-	Y	Y	-
ENSG00000163364	LINC01116	-0.904925231	1.27E-24	-0.492463559	2.86E-07	-0.234643413	0.017365	0.419284612	4.95E-05	0.685127621	1.52E-13	0.260621491	0.03737	C4	-	Y	Y	-	Y	Y	-
ENSG00000163376	KBTBD8	0.234331667	0.217121	-0.541706919	0.015243	-0.141516777	0.589322	-0.768492867	7.02E-07	-0.3614134	0.020571	0.402211732	0.107189	C5	-	-	Y	-	Y	Y	-
ENSG00000163378	EOGT	-0.497635582	0.013087	-0.451724541	0.060762	-0.25717494	0.297208	0.052285187	0.862271	0.255001597	0.270833	0.197107307	0.6004	C4	-	Y	-	-	-	-	-
ENSG00000163382	NAXE	-0.337191888	1.45E-05	-0.165456257	0.157269	-0.063209924	0.594362	0.178088037	0.019187	0.288915423	3.80E-07	0.105580204	0.397037	C4	-	Y	-	-	-	-	-
ENSG00000163393	SLC22A15	-0.782124408	0.016352	-0.578261316	0.117356	-0.172425981	0.676857	0.212863687	0.658659	0.624404953	0.088298	0.408077334	NA	C3	-	Y	-	-	-	-	-
ENSG00000163406	SLC15A2	1.382620988	4.14E-05	0.763849255	0.106306	0.999689275	0.022227	-0.613399396	0.071594	-0.369385419	0.326319	0.238450526	NA	C1	-	Y	-	Y	-	-	-
ENSG00000163412	EIF4E3	-0.427032104	0.000685	-0.3308	0.019949	-0.399531898	0.003286	0.102421713	0.554392	0.042227737	0.83276	-0.06578604	0.830237	C4	-	Y	Y	Y	-	-	-
ENSG00000163428	LRRC58	0.359983031	2.41E-08	0.072997268	0.524479	0.052015546	0.647834	-0.280300027	2.20E-06	-0.29309904	4.52E-07	-0.018162504	0.927911	C5	-	Y	-	-	-	-	-
ENSG00000163430	FSTL1	-0.384972016	3.38E-13	-0.225070676	0.019656	-0.170745117	0.019235	0.166336523	0.053716	0.229171782	0.000229	0.056882235	0.755306	C4	-	Y	-	-	-	-	-
ENSG00000163462	TRIM46	0.473375314	1.18E-12	0.203608226	0.063363	0.342114962	3.03E-05	-0.262942829	0.001032	-0.116618045	0.120041	0.141262208	0.311688	C5	-	Y	-	Y	-	-	-
ENSG00000163472	TMEM79	0.511909254	4.53E-05	0.11139756	0.591301	0.211629135	0.198943	-0.394199261	0.002636	-0.285523254	0.032425	0.103193485	0.696695	C5	-	Y	-	-	Y	-	-
ENSG00000163482	STK36	0.375694583	5.88E-06	0.318060027	0.000959	0.3684334	8.50E-06	-0.050859501	0.59698	0.007060242	0.944438	0.052351767	0.721465	C4	-	Y	Y	Y	-	-	-
ENSG00000163485	ADORA1	-1.068911869	0.005385	-0.119254767	0.83409	0.295630453	0.478014	0.958062144	0.033513	1.378467205	0.000441	0.416615779	NA	C2	-	Y	-	-	Y	Y	-
ENSG00000163499	CRYBA2	-0.832228781	0.172113	-0.639408684	0.227833	-1.048019542	0.037001	0.200906885	0.798993	-0.199012993	0.813635	-0.405629038	NA	C3	-	-	-	Y	-	-	-
ENSG00000163507	CIP2A	0.529299293	1.60E-10	0.293050649	0.00208	0.245153751	0.00702	-0.229700809	0.000428	-0.26999457	7.14E-06	-0.045827364	0.711915	C5	Y	Y	-	-	-	-	-
ENSG00000163513	TGFBR2	1.007486774	0.000499	0.491143666	0.225817	0.67057864	0.082614	-0.5087921	0.08518	-0.32193142	0.343008	0.182382987	NA	C1	-	Y	-	-	-	-	-
ENSG00000163516	ANKZF1	0.509095939	1.33E-14	0.279525095	0.001383	0.242877426	0.005453	-0.223063535	0.004572	-0.251395799	0.000958	-0.034130257	0.871807	C5	-	Y	-	-	-	-	-
ENSG00000163520	FBLN2	-0.635909997	2.22E-08	-0.490607928	0.000716	-0.314515867	0.012262	0.152765444	0.382468	0.336085352	0.008695	0.178265191	0.462115	C4	-	Y	Y	Y	-	Y	-
ENSG00000163521	GLB1L	-0.874784473	5.14E-06	-0.164695318	0.541864	-0.317275482	0.179115	0.717506941	0.000945	0.572565766	0.015524	-0.15007431	0.72365	C2	-	Y	-	-	Y	Y	-
ENSG00000163531	NFASC	-0.436241308	9.34E-08	-0.096855274	0.470841	0.081554317	0.497988	0.345772954	0.000135	0.532488121	2.44E-11	0.181187055	0.188673	C4	-	Y	-	-	Y	Y	-
ENSG00000163536	SERPINI1	-0.51137602	0.028278	-0.332220569	0.192833	-0.196051156	0.492138	0.186716047	0.551211	0.329362978	0.254239	0.138113702	0.776881	C2	-	Y	-	-	-	-	-
ENSG00000163539	CLASP2	0.323614868	6.52E-08	0.127036753	0.149222	0.32676613	7.46E-08	-0.189949406	5.65E-05	0.017512736	0.715775	0.202168184	0.000216	C4	-	Y	-	Y	-	-	-
ENSG00000163558	PRKCI	0.362185553	1.30E-09	0.175112899	0.023069	0.195186362	0.004537	-0.180517899	0.001135	-0.152318857	0.003989	0.022765152	0.867365	C5	-	Y	-	-	-	-	-
ENSG00000163611	SPICE1	0.505398214	0.004063	0.325546011	0.151019	0.179326969	0.462473	-0.173162233	0.408332	-0.311310947	0.096228	-0.143364711	0.67544	C1	-	Y	-	-	-	-	-
ENSG00000163625	WDFY3	0.328314116	0.001866	0.061276354	0.703762	0.183458748	0.135058	-0.260769385	0.002468	-0.13041268	0.177446	0.125046615	0.363679	C5	-	Y	-	-	-	-	-
ENSG00000163629	PTPN13	-0.680588313	3.31E-12	-0.08206959	0.605228	0.011137538	0.948323	0.603747628	7.82E-14	0.706097579	6.95E-23	0.097358387	0.47626	C2	-	Y	-	-	Y	Y	-
ENSG00000163638	ADAMTS9	-0.360392538	1.30E-12	0.035838697	0.691668	0.02993074	0.721997	0.402457315	3.92E-24	0.404936255	9.39E-27	-0.002958667	0.982229	C4	-	Y	-	-	Y	Y	-
ENSG00000163644	PPM1K	0.901101719	4.36E-28	0.271285512	0.018856	0.399266096	9.00E-05	-0.623286097	8.19E-13	-0.486886317	1.44E-08	0.130923396	0.455002	C5	-	Y	-	Y	Y	Y	-
ENSG00000163659	TIPARP	0.648048744	2.67E-07	-0.120752271	0.576081	0.511425815	0.000107	-0.762216715	1.28E-08	-0.121689554	0.416005	0.635094697	1.67E-05	C5	-	Y	-	Y	Y	-	Y
ENSG00000163666	HESX1	-0.99358449	0.006748	-0.182064143	0.720969	-0.534364089	0.197226	0.818637413	0.046062	0.473815269	0.330015	-0.349432965	NA	C2	-	Y	-	-	Y	-	-
ENSG00000163673	DCLK3	-0.375300372	0.279277	-1.270871429	0.000622	-0.800522843	0.051847	-0.890598386	0.020704	-0.411713518	0.35817	0.477842108	NA	C5	-	-	Y	-	Y	-	-
ENSG00000163681	SLMAP	0.69992432	2.26E-31	0.363846877	4.06E-07	0.34598301	3.98E-06	-0.32975847	9.26E-09	-0.339359632	1.38E-08	-0.015005459	0.934528	C5	-	Y	Y	Y	Y	Y	-
ENSG00000163683	SMIM14	-0.452278099	1.36E-05	-0.077050542	0.648283	-0.05386129	0.810064	0.381499699	0.000202	0.413646551	0.001884	0.025858541	0.944754	C4	-	Y	-	-	Y	Y	-
ENSG00000163686	ABHD6	0.702793952	6.55E-05	0.333444345	0.190355	0.743635887	3.12E-05	-0.363645507	0.061308	0.055622295	0.802868	0.414790304	0.081075	C1	-	Y	-	Y	-	-	-
ENSG00000163689	CFAP20DC	-1.2074804	9.91E-11	-0.433738433	0.036588	-0.189790089	0.42373	0.781687507	0.000501	1.032181869	1.46E-06	0.245986242	0.47626	C3	-	Y	Y	-	Y	Y	-
ENSG00000163702	IL17RC	-0.503182242	0.002824	-0.322889012	0.113209	-0.365701809	0.07838	0.186862192	0.407456	0.152019062	0.543039	-0.040161027	0.939631	C4	-	Y	-	-	-	-	-
ENSG00000163703	CRELD1	0.367479728	0.11969	-0.079946122	0.843331	0.333486665	0.172903	-0.441729808	0.029676	-0.02050127	0.933067	0.416299097	0.092592	C4	-	-	-	-	Y	-	-
ENSG00000163728	TTC14	0.580940678	1.85E-12	0.343156587	0.000286	0.525935327	1.60E-09	-0.232034369	2.68E-05	-0.040571978	0.579144	0.186041212	0.012689	C1	-	Y	Y	Y	-	-	-
ENSG00000163754	GYG1	-0.227617812	0.260676	0.117261093	0.627026	0.024458056	0.929166	0.351491818	0.046985	0.26699478	0.15422	-0.089726314	0.77505	C2	-	-	-	-	Y	-	-
ENSG00000163781	TOPBP1	0.348907935	5.41E-17	0.145090034	0.009651	-0.016835149	0.836298	-0.197180382	6.84E-07	-0.351335668	3.21E-20	-0.159154581	0.00642	C5	-	Y	-	-	-	Y	-
ENSG00000163818	LZTFL1	0.358494791	0.00015	0.098462066	0.479463	0.01290906	0.939088	-0.253822274	0.003451	-0.331305972	8.24E-05	-0.082970212	0.60182	C5	-	Y	-	-	-	Y	-
ENSG00000163820	FYCO1	0.741818329	2.41E-07	0.812134445	2.90E-08	0.743523835	3.74E-07	0.075757105	0.628378	0.015519753	0.931747	-0.06549926	0.794801	C1	-	Y	Y	Y	-	-	-
ENSG00000163840	DTX3L	-0.500848756	0.005319	-0.32510243	0.187203	-0.023552886	0.937358	0.182163694	0.432626	0.491824721	0.004072	0.304244968	0.281696	C4	-	Y	-	-	-	Y	-
ENSG00000163848	ZNF148	0.301867761	1.03E-05	0.069736841	0.551813	0.095358048	0.383377	-0.225549143	0.001118	-0.191816538	0.009408	0.028467911	0.888439	C5	-	Y	-	-	-	-	-
ENSG00000163864	NMNAT3	-2.167769104	0.007973	-0.091055048	NA	-0.009718575	0.992816	2.081820219	0.02357	2.171512771	0.016604	0.08469358	NA	C3	-	Y	-	-	Y	Y	-
ENSG00000163872	YEATS2	0.530710147	3.35E-21	0.246395788	0.000382	0.421949107	2.26E-12	-0.277948768	6.92E-11	-0.094242717	0.032941	0.178372787	0.001124	C5	Y	Y	-	Y	-	-	-
ENSG00000163873	GRIK3	0.148983263	0.504747	-0.345891279	0.232418	-0.617093913	0.000771	-0.488044222	0.042212	-0.751914675	2.91E-05	-0.269000882	0.505914	C5	-	-	-	Y	Y	Y	-
ENSG00000163884	KLF15	0.650585574	3.95E-10	0.536147665	1.18E-05	0.455903187	0.000586	-0.108081366	0.404693	-0.180350263	0.154528	-0.077469205	0.761898	C5	-	Y	Y	Y	-	-	-
ENSG00000163909	HEYL	3.548131936	0.02901	2.920071578	NA	2.302661013	NA	-0.62043891	0.648362	-1.227294758	0.42292	-0.611781055	NA	C1	-	Y	-	-	-	-	-
ENSG00000163923	RPL39L	-0.548189042	2.42E-05	-0.181457723	0.304736	-0.316492452	0.03389	0.373580785	0.000916	0.247195347	0.040239	-0.131907613	0.487456	C4	-	Y	-	Y	Y	-	-
ENSG00000163935	SFMBT1	-0.123448312	0.35365	-0.320080486	0.023191	-0.231759544	0.113013	-0.190465591	0.187611	-0.093500416	0.570494	0.091540832	0.747793	C4	-	-	Y	-	-	-	-
ENSG00000163945	UVSSA	-0.331920349	0.004292	0.210449161	0.138393	0.276832573	0.038155	0.548341238	7.72E-12	0.623359021	1.98E-13	0.069440147	0.70884	C4	-	Y	-	-	Y	Y	-
ENSG00000163947	ARHGEF3	-0.872023281	0.001091	-0.18735987	0.586517	-0.163633503	0.639338	0.693661879	0.020935	0.722818597	0.018463	0.025248571	0.972098	C2	-	Y	-	-	Y	Y	-
ENSG00000163958	ZDHHC19	-0.612637511	0.159865	-1.026590931	0.019656	-0.57421233	0.18217	-0.405671891	0.487884	0.053362857	0.934204	0.454658411	NA	C5	-	-	Y	-	-	-	-
ENSG00000163960	UBXN7	0.415258136	4.53E-06	0.165383195	0.194287	0.232027816	0.179341	-0.243946392	0.000349	-0.168646933	0.016077	0.069726426	0.644363	C5	-	Y	-	-	-	-	-
ENSG00000163961	RNF168	0.149009233	0.061774	-0.227798772	0.016994	-0.15100556	0.087016	-0.370276174	3.40E-06	-0.285438893	9.29E-05	0.079777663	0.604693	C4	-	-	-	-	Y	-	-
ENSG00000163975	MELTF	-0.791482653	6.38E-15	-0.505700386	2.30E-06	-0.478680059	4.12E-06	0.292702995	0.015162	0.327594495	0.005033	0.029864197	0.915334	C4	-	Y	Y	Y	-	Y	-
ENSG00000163995	ABLIM2	-0.538359855	0.028098	-0.035961526	0.92564	-0.098203289	0.745689	0.507785396	0.063158	0.4549335	0.084534	-0.059235192	0.916086	C3	-	Y	-	-	-	-	-
ENSG00000164010	ERMAP	-0.272231442	0.062454	0.04735504	0.834847	0.023438334	0.92472	0.326196912	0.027031	0.310369957	0.054952	-0.021451923	0.957684	C4	-	-	-	-	Y	-	-
ENSG00000164031	DNAJB14	0.491807497	1.49E-14	0.151662708	0.065811	0.217857559	0.003321	-0.333873928	6.90E-10	-0.25927309	1.95E-06	0.069184739	0.496438	C5	-	Y	-	-	Y	-	-
ENSG00000164038	SLC9B2	0.366404605	0.000115	0.152098835	0.245658	0.200779383	0.076873	-0.207326553	0.00867	-0.150793613	0.060237	0.051024798	0.75233	C1	-	Y	-	-	-	-	-
ENSG00000164039	BDH2	-0.33473549	0.000125	-0.154246073	0.116585	-0.21718761	0.031796	0.186850943	0.051207	0.132220771	0.236321	-0.059932091	0.752014	C4	-	Y	-	-	-	-	-
ENSG00000164045	CDC25A	0.044880278	0.540841	-0.31423303	1.35E-05	-0.336790322	7.28E-06	-0.352601694	2.37E-08	-0.367187278	2.38E-08	-0.019112856	0.931063	C4	-	-	Y	Y	Y	Y	-
ENSG00000164054	SHISA5	-0.488825112	0.001545	-0.002614853	0.993784	-0.131532576	0.595099	0.492859784	0.003754	0.372118479	0.048593	-0.126802823	0.735812	C4	-	Y	-	-	Y	Y	-
ENSG00000164056	SPRY1	0.747517728	9.58E-11	0.624032141	9.12E-06	0.843698483	4.26E-11	-0.118001068	0.371941	0.110854805	0.38653	0.223025592	0.202599	C1	-	Y	Y	Y	-	-	-
ENSG00000164061	BSN	0.905330444	1.03E-27	0.495400864	4.36E-07	0.507081944	4.78E-08	-0.403575571	4.90E-10	-0.38405243	3.86E-10	0.014314284	0.943834	C5	-	Y	Y	Y	Y	Y	-

ENSG00000164062	APEH	-0.482819858	5.04E-18	-0.099396974	0.238819	-0.018109998	0.845894	0.389807458	4.22E-10	0.479473216	2.35E-18	0.084198975	0.432414	C4	-	Y	-	-	Y	Y	-
ENSG00000164066	INTU	0.454541092	0.000497	0.091076794	0.658356	0.314770922	0.021897	-0.357077129	0.006634	-0.125336119	0.375355	0.226543811	0.215908	C5	-	Y	-	Y	Y	-	-
ENSG00000164074	ABHD18	0.410038236	0.003114	0.168158288	0.381332	0.300241887	0.089022	-0.235482792	0.086175	-0.094628854	0.586818	0.135186731	0.596189	C5	-	Y	-	-	-	-	-
ENSG00000164076	CAMKV	0.33152292	6.66E-10	0.049360273	0.592943	0.019108963	0.836838	-0.275678934	9.96E-07	-0.297712739	7.96E-09	-0.027278405	0.84282	C5	-	Y	-	-	-	-	-
ENSG00000164077	MON1A	0.699152315	9.53E-10	0.113299705	0.592943	0.239736729	0.17069	-0.578837221	2.10E-06	-0.445211086	0.000532	0.129975394	0.650514	C5	-	Y	-	-	Y	Y	-
ENSG00000164081	TEX264	-0.358693837	0.001148	-0.134511365	0.401322	-0.116247157	0.441446	0.230714251	0.062658	0.25735913	0.029666	0.021109151	0.947028	C4	-	Y	-	-	-	-	-
ENSG00000164086	DUSP7	0.277287836	0.003805	-0.075526499	0.627026	0.277524526	0.014466	-0.346496892	0.000372	0.014781434	0.918457	0.35582785	0.005195	C5	-	-	-	-	Y	-	Y
ENSG00000164088	PPM1M	-0.39642985	0.000518	-0.257428689	0.085593	-0.053935694	0.750452	0.145557402	0.341861	0.357432401	0.002294	0.20660817	0.267893	C4	-	Y	-	-	-	Y	-
ENSG00000164096	C4orf3	-0.24538984	2.72E-05	-0.001339561	0.992169	0.057342402	0.514795	0.250579395	5.58E-06	0.317814397	8.80E-10	0.061637273	0.564344	C4	-	-	-	-	-	Y	-
ENSG00000164114	MAP9	0.428759646	3.01E-09	0.125088609	0.267916	0.264864649	0.00081	-0.296773103	1.53E-05	-0.149251386	0.016401	0.14201653	0.170193	C5	-	Y	-	-	-	-	-
ENSG00000164142	FAM160A1	-1.166756167	0.194491	-0.037527613	NA	0.471346439	0.603298	1.12988499	0.319897	1.654044722	0.04608	0.51071512	NA	C2	-	-	-	-	-	Y	-
ENSG00000164151	ICE1	0.410770732	1.93E-08	0.148238082	0.138303	0.150048749	0.113847	-0.256855664	1.51E-07	-0.246079612	7.72E-07	0.005140532	0.975293	C1	-	Y	-	-	-	-	-
ENSG00000164162	ANAPC10	0.347373015	0.028462	-0.0576206	0.866983	0.008062665	0.976852	-0.398447356	0.047089	-0.324293581	0.050848	0.068398187	0.889019	C1	-	Y	-	-	Y	-	-
ENSG00000164163	ABCE1	0.411984143	1.99E-19	0.107904788	0.081027	0.047157875	0.518827	-0.297506691	2.72E-12	-0.350334751	1.32E-16	-0.057969061	0.509778	C5	-	Y	-	-	-	Y	-
ENSG00000164164	OTUD4	0.627594721	4.47E-17	0.093213793	0.422305	0.381011793	1.58E-05	-0.528132193	4.19E-26	-0.231902935	8.42E-05	0.29068929	4.70E-05	C5	-	Y	-	Y	Y	-	-
ENSG00000164171	ITGA2	1.397591459	3.90E-33	1.599757593	3.15E-28	1.510309517	6.12E-30	0.208241692	0.141703	0.127765422	0.345301	-0.086620718	0.776003	C1	-	Y	Y	Y	-	-	-
ENSG00000164175	SLC45A2	-1.127907133	0.104175	-0.696743773	0.327097	-1.262419564	0.036903	0.427132071	0.664252	-0.119740353	0.915487	-0.559577628	NA	C3	-	-	-	Y	-	-	-
ENSG00000164180	TMEM161B	0.450745389	5.50E-11	0.123921016	0.204486	0.185210174	0.017689	-0.320503863	5.18E-07	-0.251020641	1.90E-05	0.064232207	0.58778	C5	-	Y	-	-	Y	-	-
ENSG00000164185	ZNF474	1.127619533	0.14304	0.293836726	NA	-0.702413962	0.514795	-0.825698433	0.325543	-1.818241567	0.037491	-0.99165576	NA	C5	-	-	-	-	-	Y	-
ENSG00000164187	LMBRD2	0.517816407	3.51E-08	0.421572732	0.00019	0.506340137	3.07E-07	-0.090239533	0.335426	0.003071804	0.975589	0.087820269	0.547217	C1	-	Y	Y	Y	-	-	-
ENSG00000164190	NIPBL	0.503683007	8.61E-11	0.226831376	0.017032	0.219495615	0.021412	-0.270470736	2.76E-05	-0.269525095	4.60E-05	-0.004505894	0.982359	C5	-	Y	-	-	-	-	-
ENSG00000164211	STARD4	-0.181863149	0.079579	0.412652932	0.002101	0.395525847	9.66E-05	0.600928309	1.19E-07	0.591977088	7.65E-11	-0.014180383	0.967212	C4	-	-	Y	Y	Y	Y	-
ENSG00000164237	CMBL	-0.514147427	2.73E-11	-0.128995157	0.168912	-0.120663207	0.17955	0.391424253	5.59E-07	0.408126277	1.30E-07	0.011096248	0.956289	C4	-	Y	-	-	Y	Y	-
ENSG00000164241	C5orf63	-0.442787438	0.004755	-0.045411916	0.848104	0.027155457	0.900362	0.403777242	0.019488	0.484486636	0.002119	0.075417413	0.804185	C4	-	Y	-	-	Y	Y	-
ENSG00000164251	F2RL1	0.998390836	0.013752	0.0149299	0.988017	0.288519881	0.665549	-0.976801711	0.031643	-0.694342737	0.113611	0.275909829	NA	C5	-	Y	-	-	Y	-	-
ENSG00000164265	SCGB3A2	-2.343321736	NA	-0.442355628	NA	2.427294142	NA	1.887732762	NA	4.79384621	0.005567	2.853320008	NA	C4	-	-	-	-	-	Y	-
ENSG00000164291	ARSK	0.69245042	2.41E-07	0.406520788	0.012741	0.627712763	9.62E-05	-0.279437854	0.053159	-0.049784215	0.808048	0.224268485	0.3464	C5	-	Y	Y	Y	-	-	-
ENSG00000164303	ENPP6	-0.885453156	0.01792	-0.366772685	0.335707	-0.541276768	0.104011	0.523377479	0.268067	0.359835405	0.468435	-0.170304691	NA	C3	-	Y	-	-	-	-	-
ENSG00000164306	PRIMPOL	-0.372124399	0.000312	0.054480558	0.743565	0.073964229	0.619783	0.432857669	1.02E-05	0.460516778	3.14E-06	0.022222616	0.933843	C4	-	Y	-	-	Y	Y	-
ENSG00000164307	ERAP1	-0.505302308	0.009519	0.047234604	0.881724	-0.049658125	0.882132	0.558608584	0.005632	0.47042729	0.040741	-0.093753263	0.843648	C4	-	Y	-	-	Y	Y	-
ENSG00000164308	ERAP2	-1.166467896	0.070245	-0.364809508	0.658388	0.174519109	0.829083	0.805821687	0.283835	1.354263014	0.033754	0.541626778	NA	C2	-	-	-	-	-	Y	-
ENSG00000164309	CMYA5	1.278126636	0.014835	0.325692456	0.737046	0.218728506	0.827812	-0.940379736	0.051133	-1.046037836	0.03844	-0.106187466	NA	C5	-	Y	-	-	-	Y	-
ENSG00000164323	CFAP97	0.343430269	3.28E-10	0.231372463	0.001008	0.185111407	0.00725	-0.105513954	0.057803	-0.143456904	0.004836	-0.043379953	0.709041	C5	-	Y	-	-	-	-	-
ENSG00000164327	RICTOR	0.580851358	1.56E-12	0.243989277	0.064291	0.218767402	0.107191	-0.330179634	0.000702	-0.346898353	0.000548	-0.022352171	0.947133	C5	-	Y	-	-	Y	Y	-
ENSG00000164330	EBF1	-0.903600637	0.020639	-0.750461792	0.101015	-0.85753645	0.038946	0.157256202	0.805815	0.059935021	0.928521	-0.104565826	NA	C3	-	Y	-	Y	-	-	-
ENSG00000164404	GDF9	-0.925498485	0.001722	-0.329458187	0.40316	-0.442779675	0.194674	0.603678097	0.097272	0.497590097	0.182043	-0.110669542	NA	C2	-	Y	-	-	-	-	-
ENSG00000164405	UQCRQ	-0.282258818	0.000734	-0.231733366	0.018346	-0.320677011	0.000792	0.057556563	0.348411	-0.023587732	0.769566	-0.086255334	0.379545	C4	-	-	-	Y	-	-	-
ENSG00000164406	LEAP2	-0.422322108	0.436985	-0.154459518	0.84977	0.486713836	0.395454	0.274443682	0.65206	0.923761593	0.040414	0.642804456	NA	C4	-	-	-	-	-	Y	-
ENSG00000164414	SLC35A1	0.551493299	7.35E-06	0.140936313	0.473672	0.272158445	0.112822	-0.403750738	0.000602	-0.26372858	0.047609	0.134429105	0.584338	C5	-	Y	-	-	Y	-	-
ENSG00000164506	STXBP5	0.532176187	1.21E-14	0.155516734	0.141257	0.288628078	0.000197	-0.37017977	4.58E-07	-0.22916113	0.000479	0.135998476	0.250087	C5	-	Y	-	-	Y	-	-
ENSG00000164535	DAGLB	0.440814517	1.25E-05	0.326810834	0.009537	0.284149024	0.025253	-0.107389847	0.395525	-0.142044204	0.243	-0.039662987	0.888469	C5	-	Y	Y	-	-	-	-
ENSG00000164548	TRA2A	0.469218063	1.71E-20	-0.041513918	0.650077	0.216856713	0.000213	-0.504298917	1.10E-41	-0.237902109	7.15E-11	0.261025787	1.75E-08	C5	Y	Y	-	-	Y	-	-
ENSG00000164574	GALNT10	-0.335204134	0.007082	0.31195612	0.015535	0.418815077	0.000891	0.653912906	2.28E-08	0.76892075	1.11E-10	0.109604709	0.619084	C4	-	Y	Y	Y	Y	Y	-
ENSG00000164576	SAP30L	0.649888449	1.52E-16	0.361398855	0.000166	0.448966874	2.48E-07	-0.281406082	8.87E-07	-0.186720572	0.000991	0.089518802	0.39321	C5	-	Y	Y	Y	-	-	-
ENSG00000164603	BMT2	0.529457043	8.74E-08	0.170769748	0.14744	0.238244138	0.022172	-0.352308287	0.000121	-0.276634301	0.002475	0.07060742	0.658238	C5	-	Y	-	-	Y	-	-
ENSG00000164604	GPR85	-0.537306235	0.021355	0.198734866	0.50319	0.312173963	0.220309	0.742785281	0.000297	0.864029235	2.07E-05	0.115711809	0.769799	C4	-	Y	-	-	Y	Y	-
ENSG00000164610	RP9	0.111093185	0.359812	-0.1886911	0.140783	-0.222241665	0.073527	-0.29341963	0.005658	-0.318716519	0.003238	-0.030664896	0.912709	C5	-	-	-	-	-	Y	-
ENSG00000164615	CAMLG	0.368186675	1.01E-05	0.165236866	0.124519	0.235448979	0.019745	-0.196476512	0.02342	-0.117587827	0.2237	0.073296304	0.661345	C5	-	Y	-	-	-	-	-
ENSG00000164619	BMPER	0.659022118	0.106733	0.871296819	0.040409	0.709098955	0.116421	0.219408019	0.639652	0.065257937	0.906639	-0.159926378	NA	C1	-	-	Y	-	-	-	-
ENSG00000164627	KIF6	-1.518570228	0.003247	0.18206635	0.7331	0.176015161	0.765177	1.708343234	0.000447	1.708624334	0.001317	-0.002745404	NA	C3	-	Y	-	-	Y	Y	-
ENSG00000164647	STEAP1	-1.499589144	0.003672	-0.44239713	0.48403	-0.280740034	0.645521	1.062464298	0.094567	1.232996765	0.036705	0.164528989	NA	C3	-	Y	-	-	-	Y	-
ENSG00000164649	CDCA7L	-0.305495584	0.002279	-0.267604791	0.01972	-0.174467837	0.122947	0.044210947	0.763251	0.145538674	0.203955	0.095992083	0.606622	C4	-	Y	-	-	-	-	-
ENSG00000164654	MIOS	0.265397981	1.80E-06	-0.149476084	0.076764	0.046865097	0.58228	-0.408045332	5.35E-14	-0.203860589	1.35E-05	0.19883848	0.008404	C5	-	-	-	-	Y	-	-
ENSG00000164659	ELAPOR2	-0.173222129	0.004627	0.148537661	0.045684	0.000924605	0.993365	0.327866221	8.09E-12	0.188789268	0.00014	-0.144496165	0.037309	C4	-	-	-	-	Y	-	-
ENSG00000164669	INTS4P1	1.119729823	0.006417	0.599998315	0.271586	0.584831437	0.312655	-0.513562149	0.157008	-0.520592032	0.197046	-0.012216239	NA	C5	-	Y	-	-	-	-	-
ENSG00000164683	HEY1	0.198388049	0.011308	-0.266499244	0.010537	-0.242653586	0.042913	-0.458577064	5.29E-08	-0.42642749	7.81E-06	0.026314	0.933843	C5	-	-	-	-	Y	Y	-
ENSG00000164687	FABP5	-0.679259362	0.009121	-0.751199731	0.006782	-0.748334823	0.002448	-0.067717465	0.874427	-0.053702933	0.897647	0.006876538	NA	C4	-	Y	Y	Y	-	-	-
ENSG00000164707	SLC13A4	0.279892097	0.104486	0.311629156	0.026547	0.661929636	7.58E-09	0.038470056	0.852695	0.396801094	0.003732	0.353450992	0.003375	C4	-	-	Y	Y	-	Y	Y
ENSG00000164776	PHKG1	0.946720531	0.000524	0.405030695	0.343769	0.572151934	0.09482	-0.536063058	0.079271	-0.360173162	0.228275	0.17052411	NA	C1	-	Y	-	-	-	-	-
ENSG00000164823	OSGIN2	0.341187116	0.000245	0.124167356	0.35555	5.20E-05	0.999623	-0.210583957	0.023732	-0.326430744	5.02E-05	-0.121107294	0.408204	C5	-	Y	-	-	-	Y	-
ENSG00000164830	OXR1	-0.360610785	0.032297	-0.176114197	0.519371	-0.272555562	0.190265	0.191154124	0.382468	0.102610044	0.64082	-0.093882968	0.83038	C4	-	Y	-	-	-	-	-
ENSG00000164845	FAM86FP	-1.572977737	0.003919	-0.257880066	0.710865	-0.223861638	0.735642	1.322553786	0.031893	1.363946149	0.027847	0.036984259	NA	C3	-	Y	-	-	Y	Y	-
ENSG00000164849	GPR146	-0.806300162	0.01165	-0.145975597	0.74858	-0.025812802	0.956427	0.667732174	0.057027	0.794874385	0.014551	0.123296943	NA	C4	-	Y	-	-	-	Y	-
ENSG00000164850	GPER1	-0.866542448	1.92E-11	-0.184974925	0.273632	-0.276212451	0.060456	0.688664694	7.22E-07	0.605207722	1.82E-05	-0.088375579	0.749641	C2	-	Y	-	-	Y	Y	-
ENSG00000164904	ALDH7A1	-0.305679701	1.53E-05	-0.086965308	0.372295	-0.032084816	0.777002	0.225036522	0.002783	0.288223714	9.78E-05	0.057776355	0.692958	C4	-	Y	-	-	-	-	-
ENSG00000164930	FZD6	-1.079241894	5.13E-13	-0.802061756	1.25E-05	-0.665983785	1.34E-05	0.283366455	0.21602	0.427996163	0.019416	0.139083761	0.714139	C4	-	Y	Y	Y	-	Y	-
ENSG00000164932	CTHRC1	-1.980039221	1.61E-06	-0.050785909	0.931203	-0.048983739	0.927601	1.936711621	8.97E-06	1.94519086	5.63E-06	0.004767028	NA	C3	-	Y	-	-	Y	Y	-

ENSG00000164941	INTS8	0.51612477	2.07E-09	0.22699166	0.030169	0.442270022	4.62E-07	-0.28270983	7.23E-05	-0.059673433	0.48531	0.217604659	0.01185	C5	-	Y	-	Y	-	-	-
ENSG00000164946	FREM1	-0.949382013	2.09E-08	-0.356982315	0.072122	-0.338251683	0.07092	0.600055659	0.002828	0.62587853	0.001392	0.021253875	0.96696	C4	-	Y	-	-	Y	Y	-
ENSG00000164949	GEM	0.203890702	0.025132	-0.231420617	0.045567	-0.107985252	0.38192	-0.428918876	3.43E-06	-0.297067997	0.001145	0.126247762	0.469108	C4	-	-	-	-	Y	-	-
ENSG00000164967	RPP25L	-0.983833638	5.94E-07	-0.32615878	0.249897	-0.328675571	0.143455	0.665116108	0.007398	0.670187942	0.002045	0.000280332	0.99962	C3	-	Y	-	-	Y	Y	-
ENSG00000164972	C9orf24	-0.770655408	0.15874	-0.462303574	0.607217	-1.165142444	0.04303	0.311064284	0.722913	-0.379140173	0.611671	-0.697049204	NA	C3	-	-	-	Y	-	-	-
ENSG00000164975	SNAPC3	0.462229578	2.70E-11	0.215477108	0.013925	0.247574938	0.000878	-0.24047051	0.000205	-0.200034064	0.000571	0.03507838	0.803913	C5	-	Y	-	-	-	-	-
ENSG00000164976	MYORG	-0.795153776	8.09E-14	-0.354214786	0.002699	-0.062258893	0.685185	0.447125773	0.000232	0.747580852	1.55E-11	0.294860615	0.037528	C4	-	Y	Y	-	Y	Y	-
ENSG00000164989	CCDC171	-0.630066512	0.007589	-0.139466429	0.732073	-0.229218641	0.453864	0.496762405	0.067367	0.414779041	0.095479	-0.086274981	0.88943	C2	-	Y	-	-	-	-	-
ENSG00000165029	ABCA1	-0.85882421	0.056889	-1.080630185	0.044484	-0.620072559	0.240713	-0.212462511	0.771592	0.254049799	0.698182	0.462069882	NA	C3	-	-	Y	-	-	-	-
ENSG00000165030	NFIL3	0.12112772	0.335388	-0.214525196	0.179447	-0.054274334	0.749067	-0.329417589	0.012925	-0.160725989	0.217778	0.163580641	0.473865	C5	-	-	-	-	Y	-	-
ENSG00000165046	LETM2	0.345469278	0.030366	-0.074726331	0.785122	0.063182084	0.815555	-0.413940797	0.007539	-0.268052663	0.119939	0.140528185	0.659205	C5	-	Y	-	-	Y	-	-
ENSG00000165060	FXN	-0.340806771	0.080763	-0.02995237	0.933448	0.055548796	0.841818	0.317323802	0.144891	0.410959232	0.026335	0.088040732	0.839023	C4	-	-	-	-	-	Y	-
ENSG00000165097	KDM1B	-0.033511047	0.797417	0.344286772	0.000103	0.33777124	0.000884	0.38444925	8.30E-06	0.385918439	5.90E-05	-0.003709594	0.988432	C4	-	-	Y	Y	Y	Y	-
ENSG00000165102	HGSNAT	-0.400507713	5.70E-09	-0.001552767	0.992528	-0.037933284	0.715877	0.405482284	6.70E-07	0.377340397	1.95E-08	-0.033280037	0.863083	C4	-	Y	-	-	Y	Y	-
ENSG00000165113	GKAP1	0.422312498	0.003266	0.236683599	0.229855	0.387062095	0.013853	-0.17925852	0.302481	-0.020452878	0.920658	0.153646302	0.555644	C5	-	Y	-	Y	-	-	-
ENSG00000165115	KIF27	0.319609503	0.04082	0.176509755	0.442596	0.561575167	0.000174	-0.136360315	0.455596	0.256700895	0.071495	0.387254194	0.034948	C4	-	Y	-	Y	-	-	Y
ENSG00000165124	SVEP1	0.641432138	0.162356	0.93011327	0.041838	0.105524507	0.901975	0.294050828	0.511723	-0.520123102	0.332685	-0.821580711	NA	C1	-	-	Y	-	-	-	-
ENSG00000165138	ANKS6	-0.421526445	8.38E-07	0.06398903	0.66931	0.049122673	0.721791	0.491878865	4.92E-08	0.48558372	1.55E-08	-0.011887108	0.964795	C4	-	Y	-	-	Y	Y	-
ENSG00000165169	DYNLT3	-0.599470792	7.48E-06	-0.077826451	0.718989	-0.126765258	0.485571	0.529037404	0.000549	0.48748657	0.00135	-0.046622468	0.90013	C2	-	Y	-	-	Y	Y	-
ENSG00000165171	METTL27	-0.548088552	0.189399	0.345547228	0.457765	0.449932521	0.213138	0.904038824	0.02668	1.013263913	0.004929	0.108107349	NA	C2	-	-	-	-	Y	Y	-
ENSG00000165175	MID1IP1	-0.457439043	1.74E-11	-0.143375419	0.094323	-0.200702724	0.003339	0.320157932	2.88E-05	0.271434167	0.000158	-0.054463905	0.701152	C4	-	Y	-	-	Y	-	-
ENSG00000165185	KIAA1958	0.550274982	3.71E-10	0.299444144	0.013267	0.295280819	0.005807	-0.244571363	0.008017	-0.240290636	0.002947	-0.001260486	0.997072	C1	-	Y	-	-	-	-	-
ENSG00000165195	PIGA	0.096220213	0.446937	-0.069099125	0.685225	-0.153010663	0.040223	-0.158592428	0.114037	-0.335254904	0.000294	-0.181995121	0.1995	C5	-	-	-	-	-	Y	-
ENSG00000165219	GAPVD1	0.502579098	3.16E-11	0.221036344	0.010219	0.141063472	0.091381	-0.275168912	4.66E-05	-0.347148547	1.08E-08	-0.077301664	0.460318	C5	-	Y	-	-	-	Y	-
ENSG00000165238	WNK2	-0.259184608	0.0003	0.096416119	0.261814	0.192680299	0.018759	0.361912794	3.45E-09	0.466597038	1.64E-12	0.099177871	0.346307	C4	-	-	-	-	Y	Y	-
ENSG00000165244	ZNF367	-0.482116604	5.17E-11	-0.355359395	1.41E-06	-0.664205493	4.07E-12	0.132948322	0.134037	-0.167521931	0.135506	-0.305241526	0.016726	C4	-	Y	Y	Y	-	-	Y
ENSG00000165272	AQP3	1.43216932	0.008059	0.197294628	NA	-0.609598287	NA	-1.225164222	0.018228	-2.027426726	0.000319	-0.804441201	NA	C5	-	Y	-	-	Y	Y	-
ENSG00000165282	PIGO	-0.131711093	0.186558	0.053946886	0.708556	0.181463676	0.070653	0.191949387	0.046513	0.327605848	0.000143	0.130277806	0.361959	C4	-	-	-	-	-	Y	-
ENSG00000165288	BRWD3	0.749802321	9.10E-15	0.286421292	0.012257	0.643979585	1.81E-09	-0.457095999	7.25E-09	-0.091070029	0.397009	0.360526714	0.000771	C1	-	Y	-	Y	Y	-	Y
ENSG00000165349	SLC7A3	-1.319722539	0.011639	-1.039711923	0.074629	-0.940785648	0.108729	0.284738323	0.744746	0.395042785	0.64246	0.104648636	NA	C3	-	Y	-	-	-	-	-
ENSG00000165355	FBXO33	0.45007136	0.021388	0.032650268	0.931301	0.298141051	0.215823	-0.411782909	0.010139	-0.138303229	0.434377	0.268321387	0.288454	C5	-	Y	-	-	Y	-	-
ENSG00000165424	ZCCHC24	-1.135590465	2.45E-15	-0.305452734	0.178585	0.032079887	0.873779	0.838269484	4.82E-05	1.182389538	6.67E-16	0.340057873	0.208345	C3	-	Y	-	-	Y	Y	-
ENSG00000165449	SLC16A9	-0.17301135	0.092004	-0.35993773	0.004383	-0.24335509	0.061046	-0.180327216	0.156674	-0.055664408	0.7156	0.120251608	0.621168	C4	-	-	Y	-	-	-	-
ENSG00000165475	CRYL1	-0.940265337	0.033516	-0.171105303	0.766568	-0.227936848	0.656832	0.775269458	0.081067	0.728197061	0.106498	-0.054568287	NA	C3	-	Y	-	-	-	-	-
ENSG00000165525	NEMF	0.646835239	9.92E-16	0.120915089	0.294389	0.232664059	0.020231	-0.519772665	1.81E-19	-0.40001479	9.92E-11	0.114370013	0.271752	C5	-	Y	-	-	Y	Y	-
ENSG00000165526	RPUSD4	0.187645705	0.009917	0.03033028	0.81569	-0.150059541	0.107191	-0.150944292	0.057318	-0.322994926	1.72E-05	-0.177404464	0.122593	C5	-	-	-	-	-	Y	-
ENSG00000165533	TTC8	-0.903415955	1.51E-82	-0.302067331	2.02E-10	-0.37980041	1.43E-09	0.607842862	4.34E-39	0.538378734	1.32E-20	-0.074991225	0.494052	C2	-	Y	Y	Y	Y	Y	-
ENSG00000165548	TMEM63C	-0.325772211	0.004965	-0.356567168	0.006041	-0.188868727	0.085378	-0.025555066	0.896778	0.151446422	0.247368	0.170433059	0.366665	C4	-	Y	Y	-	-	-	-
ENSG00000165568	AKR1E2	-0.682416853	2.70E-05	-0.136347961	0.56679	-0.009229516	0.972253	0.55261151	0.003245	0.687979822	4.78E-05	0.130140194	0.681892	C4	-	Y	-	-	Y	Y	-
ENSG00000165572	KBTBD6	0.288683763	0.000435	-0.19979195	0.083964	-0.0283196	0.814892	-0.48214074	1.89E-07	-0.3024267	0.000107	0.174191661	0.212425	C5	-	-	-	-	Y	Y	-
ENSG00000165606	DRGX	-0.618014849	0.008488	-0.602537306	0.020165	-1.143489091	1.41E-06	0.023313258	0.955913	-0.510242736	0.099911	-0.538860122	NA	C3	-	Y	Y	Y	-	-	-
ENSG00000165617	DACT1	0.3977868	0.59374	1.237309826	0.048348	1.597845382	0.002973	0.845284398	0.144207	1.214790053	0.016787	0.361766973	NA	C4	-	-	Y	Y	-	Y	-
ENSG00000165626	BEND7	0.47566317	1.29E-07	0.194897056	0.142823	0.417797478	2.02E-05	-0.274408264	0.006824	-0.043307312	0.720095	0.226037605	0.100722	C1	-	Y	-	Y	-	-	-
ENSG00000165632	TAF3	0.347871753	4.24E-07	0.151674888	0.201382	0.037975536	0.743654	-0.189345522	0.032461	-0.295188729	1.74E-05	-0.110941568	0.473188	C5	-	Y	-	-	-	-	-
ENSG00000165633	VSTM4	-0.982674544	8.96E-09	-0.409286385	0.02308	-0.293321055	0.136304	0.579877271	0.002053	0.703865542	0.000228	0.118720737	0.729425	C4	-	Y	Y	-	Y	Y	-
ENSG00000165650	PDZD8	0.486228043	1.39E-12	0.330337818	7.09E-05	0.301774349	8.27E-05	-0.149274483	0.028065	-0.170053614	0.005916	-0.026042972	0.872705	C1	-	Y	Y	Y	-	-	-
ENSG00000165655	ZNF503	-0.568513041	3.66E-09	-0.471259962	3.00E-05	-0.468691045	8.02E-06	0.10347897	0.462283	0.114584603	0.378346	0.005966674	0.985912	C4	-	Y	Y	Y	-	-	-
ENSG00000165661	QSOX2	0.367665051	1.60E-10	0.160665571	0.044911	0.142694785	0.023258	-0.200590485	0.002945	-0.210262029	0.000163	-0.01518471	0.93194	C5	-	Y	-	-	-	-	-
ENSG00000165675	ENOX2	-0.315149579	0.041611	0.244137808	0.208306	0.196507362	0.257227	0.565840657	2.37E-05	0.525793236	2.23E-05	-0.045727293	0.887679	C4	-	Y	-	-	Y	Y	-
ENSG00000165698	SPACA9	-0.812617746	0.000128	-0.271386276	0.300137	-0.30474388	0.177286	0.54890634	0.01932	0.523032572	0.019526	-0.029970638	0.955253	C2	-	Y	-	-	Y	Y	-
ENSG00000165699	TSC1	0.475458193	1.03E-10	0.146998317	0.154011	0.234949101	0.013857	-0.322812047	4.59E-09	-0.226276259	0.000242	0.09090331	0.446036	C5	-	Y	-	-	Y	-	-
ENSG00000165724	ZMYND19	0.204467033	0.030548	0.006632635	0.970129	-0.150104201	0.175966	-0.191085297	0.03245	-0.339921072	3.15E-05	-0.154162802	0.234689	C5	-	-	-	-	-	Y	-
ENSG00000165731	RET	-0.596113594	1.96E-52	0.043116993	0.606555	0.063980058	0.281278	0.645737621	8.08E-48	0.674838976	2.41E-79	0.023678903	0.847006	C2	Y	Y	-	-	Y	Y	-
ENSG00000165732	DDX21	0.440348713	6.68E-21	0.13909782	0.041869	0.040777603	0.56413	-0.294698671	8.21E-15	-0.385181556	1.17E-43	-0.09577959	0.117761	C5	-	Y	-	-	-	Y	-
ENSG00000165804	ZNF219	0.451620005	2.92E-06	0.315945673	0.009053	0.347899921	0.001671	-0.128889803	0.230903	-0.089347554	0.409641	0.034480593	0.887295	C1	-	Y	Y	Y	-	-	-
ENSG00000165806	CASP7	-0.441212374	0.001039	-0.270887562	0.07584	-0.121185433	0.465867	0.176974257	0.297385	0.334807595	0.020992	0.152542641	0.503412	C4	-	Y	-	-	-	Y	-
ENSG00000165813	CCDC186	0.472165078	2.53E-06	0.183694236	0.271547	0.281063786	0.012083	-0.281804912	0.01957	-0.176625741	0.081002	0.100024043	0.651289	C1	-	Y	-	-	-	-	-
ENSG00000165861	ZFYVE1	0.496076203	6.99E-07	0.214594429	0.077331	0.245496561	0.024945	-0.275138212	0.001318	-0.236028697	0.004792	0.033512762	0.860105	C5	-	Y	-	-	-	-	-
ENSG00000165912	PACSIN3	-0.467932305	0.00035	-0.359285013	0.015609	-0.269730431	0.067002	0.114697023	0.527035	0.213087278	0.177568	0.092492163	0.743991	C4	-	Y	Y	-	-	-	-
ENSG00000165959	CLMN	-0.913264701	2.88E-07	-0.663459721	0.00016	-0.353899436	0.07672	0.258328981	0.299591	0.574356382	0.008349	0.311104388	0.308223	C4	-	Y	Y	-	-	Y	-
ENSG00000165972	CCDC38	-1.172655675	0.004871	-0.675167097	0.153979	-0.381309555	0.434722	0.502767095	0.408146	0.805689989	0.128797	0.296648328	NA	C3	-	Y	-	-	-	-	-
ENSG00000165973	NELL1	0.377890959	0.40307	-0.612146923	0.315467	-1.178713172	0.033039	-0.984892138	0.014479	-1.538608377	0.000258	-0.563210236	NA	C5	-	-	-	Y	Y	Y	-
ENSG00000165995	CACNB2	-0.364514375	0.087811	0.262895469	0.256146	0.10875207	0.680131	0.635378207	0.002551	0.487700273	0.026124	-0.151978819	0.675496	C2	-	-	-	-	Y	Y	-
ENSG00000165996	HACD1	-0.305683886	0.031268	-0.180710494	0.315391	-0.143373636	0.480753	0.131676704	0.471619	0.177003205	0.348032	0.039827936	0.926363	C4	-	Y	-	-	-	-	-
ENSG00000165997	ARL5B	0.343815328	9.42E-08	-0.069538557	0.518723	-0.01539434	0.895628	-0.407170388	1.95E-14	-0.344850958	5.78E-13	0.057098828	0.609716	C5	-	Y	-	-	Y	Y	-
ENSG00000166004	CEP295	0.359887921	0.001679	0.180567245	0.279963	0.196442235	0.159813	-0.174663807	0.069032	-0.149201271	0.069968	0.019799747	0.938748	C5	-	Y	-	-	-	-	-
ENSG00000166012	TAF1D	0.48465518	8.56E-14	-0.056008995	0.610246	0.01128505	0.921589	-0.534141369	1.71E-19	-0.458978058	3.20E-18	0.069911351	0.541217	C5	-	Y	-	-	Y	Y	-

ENSG00000166025	AMOTL1	0.401850952	2.35E-08	0.291730452	0.000222	0.261286873	0.000749	-0.103638297	0.032164	-0.1264469	0.006493	-0.028165678	0.766411	C5	-	Y	-	-	-	-	-
ENSG00000166033	HTRA1	-0.45692316	0.053098	0.063405781	0.875001	-0.098932808	0.793768	0.527882771	0.038872	0.373159336	0.1976	-0.161194261	0.766088	C2	-	-	-	-	Y	-	-
ENSG00000166073	GPR176	-0.172066005	0.231881	0.177931031	0.297998	0.001781275	0.993892	0.357150599	0.014802	0.188421032	0.256936	-0.173813675	0.498637	C4	-	-	-	-	Y	-	-
ENSG00000166086	JAM3	0.706896644	1.30E-27	0.419427645	4.99E-08	0.361181175	8.50E-06	-0.280623766	4.58E-07	-0.330958199	1.64E-08	-0.055460944	0.681159	C1	Y	Y	Y	Y	-	Y	-
ENSG00000166106	ADAMTS15	2.546487382	0.007997	2.323815051	NA	2.728812567	NA	-0.225934871	0.831666	0.197896587	0.819775	0.421972039	NA	C1	-	Y	-	-	-	-	-
ENSG00000166123	GPT2	-0.560086502	4.50E-20	-0.473996841	1.99E-08	-0.312043683	0.000789	0.092552036	0.355064	0.262681677	0.002304	0.165630337	0.301335	C4	-	Y	Y	Y	-	-	-
ENSG00000166128	RAB8B	0.363317491	7.01E-06	0.257237047	0.009289	0.315183735	0.000351	-0.099543611	0.301406	-0.033468891	0.754189	0.060837412	0.715621	C4	-	Y	-	Y	-	-	-
ENSG00000166135	HIF1AN	0.30896176	2.17E-06	0.148345195	0.05799	0.140438989	0.078365	-0.154407799	0.009086	-0.154189192	0.010742	-0.005071866	0.9772	C5	-	Y	-	-	-	-	-
ENSG00000166147	FBN1	-0.355361847	0.00034	0.278290538	0.303763	0.284205118	0.021491	0.640678068	0.000596	0.654212081	9.10E-14	0.008077758	0.989969	C4	-	Y	-	-	Y	Y	-
ENSG00000166167	BTRC	0.395031941	5.56E-08	0.187448426	0.04362	0.158686396	0.081347	-0.201191765	0.001547	-0.221732482	0.000408	-0.026006777	0.869517	C5	-	Y	-	-	-	-	-
ENSG00000166170	BAG5	0.183243895	0.004486	-0.135892258	0.098314	-0.1396631	0.06146	-0.31270501	5.19E-08	-0.308305915	1.83E-08	-0.000928267	0.996048	C5	-	-	-	-	Y	Y	-
ENSG00000166173	LARP6	0.640293837	1.98E-07	0.221688607	0.185941	0.543259416	8.92E-05	-0.412154501	0.00046	-0.082356747	0.589445	0.324545679	0.043221	C1	-	Y	-	Y	Y	-	Y
ENSG00000166199	ALKBH3	-0.574392129	4.84E-07	-0.175616384	0.26238	-0.345905448	0.008329	0.40515519	0.001161	0.243540777	0.073967	-0.16726915	0.423817	C4	-	Y	-	Y	Y	-	-
ENSG00000166228	PCBD1	-0.387320738	3.97E-08	-0.179774205	0.028615	-0.122773517	0.184276	0.214062382	0.002824	0.279422452	0.000136	0.059970452	0.662979	C4	-	Y	-	-	-	-	-
ENSG00000166233	ARIH1	0.344082911	4.58E-07	0.182082718	0.026619	0.15065471	0.04777	-0.155569114	0.03211	-0.178777809	0.006358	-0.028541217	0.855465	C5	-	Y	-	-	-	-	-
ENSG00000166257	SCN3B	-0.164036819	0.017053	0.055066992	0.613081	0.219117212	0.00603	0.225371577	0.001214	0.397970565	1.96E-09	0.166705337	0.111448	C4	-	-	-	-	-	Y	-
ENSG00000166261	ZNF202	0.201348789	0.013471	-0.176082419	0.099481	-0.115853787	0.22636	-0.370956739	8.68E-06	-0.30250264	5.79E-05	0.062864026	0.714139	C5	-	-	-	-	Y	Y	-
ENSG00000166263	STXBP4	0.720298601	5.22E-11	0.326053385	0.017708	0.351398064	0.004506	-0.387248515	8.24E-05	-0.354356657	0.000138	0.027379305	0.915306	C5	-	Y	Y	Y	Y	Y	-
ENSG00000166292	TMEM100	-0.09529665	0.679099	-0.618700433	0.002906	0.028668945	0.921934	-0.517052912	0.008905	0.138333014	0.521132	0.650103217	0.003008	C5	-	-	Y	-	Y	-	Y
ENSG00000166295	ANAPC16	-0.534744602	1.58E-20	-0.109110353	0.138844	-0.088737156	0.254139	0.432205468	4.87E-16	0.46077642	2.71E-17	0.023240868	0.860105	C2	-	Y	-	-	Y	Y	-
ENSG00000166321	NUDT13	-0.573832061	0.000477	-0.063177101	0.835278	-0.200982979	0.317447	0.516961194	0.01231	0.387643189	0.047392	-0.134923026	0.728193	C4	-	Y	-	-	Y	Y	-
ENSG00000166347	CYB5A	-0.275343735	0.024122	-0.011113907	0.958156	0.036559715	0.832105	0.270757316	0.014373	0.326706256	0.001829	0.050228476	0.812182	C4	-	-	-	-	-	Y	-
ENSG00000166348	USP54	0.503032495	3.82E-10	0.319070258	0.000576	0.340027325	0.000196	-0.177727839	0.020796	-0.148477845	0.066417	0.023777368	0.902665	C5	-	Y	Y	Y	-	-	-
ENSG00000166352	IFTAP	-0.418178873	5.52E-05	-0.179225299	0.189694	-0.218225887	0.163292	0.24534857	0.04309	0.21443306	0.148749	-0.036663944	0.918762	C4	-	Y	-	-	-	-	-
ENSG00000166359	WDR88	-0.633072895	0.007445	-0.055339134	0.880513	0.005583368	0.988521	0.584168114	0.015294	0.653251496	0.008701	0.063650417	0.910372	C4	-	Y	-	-	Y	Y	-
ENSG00000166377	ATP9B	-0.331573185	0.013127	0.013581378	0.95872	-0.019733787	0.934956	0.351611804	0.011988	0.326574865	0.025197	-0.030987841	0.937216	C4	-	Y	-	-	Y	Y	-
ENSG00000166387	PPFIBP2	-0.601414711	1.03E-05	-0.304762631	0.038336	-0.187917977	0.191545	0.30420727	0.058876	0.428283756	0.002645	0.120059096	0.605336	C4	-	Y	Y	-	-	Y	-
ENSG00000166394	CYB5R2	-2.146961675	0.001265	-0.540111552	0.458949	-0.224030072	0.758271	1.620980649	0.047362	1.936523885	0.008357	0.317324045	NA	C3	-	Y	-	-	Y	Y	-
ENSG00000166398	GARRE1	0.323325569	0.00033	0.26114125	0.016049	0.269099154	0.00345	-0.05598542	0.576543	-0.039671599	0.659195	0.011207048	0.955888	C4	-	Y	-	-	-	-	-
ENSG00000166402	TUB	0.215808219	8.62E-06	0.383222641	7.37E-13	0.252362872	3.34E-05	0.173758398	7.13E-05	0.05099229	0.358351	-0.128109329	0.089669	C4	-	-	Y	-	-	-	-
ENSG00000166405	RIC3	0.43800677	3.48E-11	0.180623793	0.045229	0.273117646	0.001397	-0.250956291	0.000244	-0.15029072	0.049844	0.095374874	0.491439	C5	-	Y	-	-	-	-	-
ENSG00000166407	LMO1	-0.823931597	1.74E-32	-0.307677096	0.000131	-0.37129152	3.61E-07	0.522569324	7.68E-12	0.467383667	3.16E-10	-0.060671305	0.690413	C3	-	Y	Y	Y	Y	Y	-
ENSG00000166411	IDH3A	0.725416791	1.48E-16	0.195853914	0.172292	0.350067398	0.000843	-0.522711851	6.34E-08	-0.360842186	3.04E-05	0.156867828	0.363559	C5	-	Y	-	Y	Y	Y	-
ENSG00000166426	CRABP1	-0.397092455	0.001827	-0.174120786	0.315684	-0.151609715	0.344261	0.230069272	0.145264	0.260134739	0.076185	0.024840974	0.947081	C4	-	Y	-	-	-	-	-
ENSG00000166428	PLD4	-3.560564974	0.01931	-0.828336922	NA	-0.398962955	NA	2.733848074	NA	3.175246189	0.061857	0.433323594	NA	C3	-	Y	-	-	-	-	-
ENSG00000166432	ZMAT1	-1.192462369	1.50E-22	-0.465571127	0.000286	-0.133641378	0.46824	0.732983566	7.20E-11	1.073428639	3.40E-18	0.334814156	0.03989	C4	-	Y	Y	-	Y	Y	Y
ENSG00000166435	XRRA1	0.480264536	0.000375	0.19027017	0.33724	0.417966563	0.014666	-0.283494793	0.036667	-0.048265237	0.799537	0.230355145	0.315115	C5	-	Y	-	Y	-	-	-
ENSG00000166436	TRIM66	-1.065151994	2.52E-16	-0.029759332	0.892343	0.095282057	0.565842	1.043391804	4.44E-13	1.174937663	1.71E-18	0.127131943	0.604908	C3	-	Y	-	-	Y	Y	-
ENSG00000166439	RNF169	0.38673983	4.94E-05	0.21481615	0.066501	0.187711152	0.143434	-0.165647738	0.099001	-0.184349917	0.085481	-0.024314166	0.929748	C5	-	Y	-	-	-	-	-
ENSG00000166448	TMEM130	-0.749621383	5.65E-10	-0.147715793	0.365567	-0.006509345	0.972021	0.608768093	2.22E-05	0.757665356	9.84E-10	0.143780112	0.509107	C2	-	Y	-	-	Y	Y	-
ENSG00000166451	CENPN	-0.142604286	0.083233	-0.297725326	0.002325	-0.329222183	0.000342	-0.14850779	0.116456	-0.172243874	0.058959	-0.028090927	0.911454	C4	-	-	-	Y	-	-	-
ENSG00000166471	TMEM41B	0.182189558	0.036334	-0.207794508	0.022411	-0.144507847	0.118216	-0.383447223	7.76E-07	-0.312181934	3.98E-05	0.066136146	0.651289	C4	-	-	-	-	Y	Y	-
ENSG00000166483	WEE1	-0.230037121	3.81E-06	-0.359708927	1.96E-11	-0.304139779	3.90E-07	-0.123203113	0.021284	-0.059617957	0.35293	0.058477791	0.591978	C4	-	-	Y	Y	-	-	-
ENSG00000166508	MCM7	-0.161774269	0.000215	-0.236922293	0.00012	-0.447136096	2.63E-18	-0.068482825	0.171338	-0.270703338	1.38E-13	-0.207310058	0.001092	C4	-	-	-	Y	-	-	-
ENSG00000166532	RIMKLB	-0.435446534	1.45E-18	-0.224963327	0.000452	-0.219177313	0.000597	0.217016299	3.51E-05	0.231066539	1.24E-05	0.008655297	0.958938	C4	Y	Y	-	-	-	-	-
ENSG00000166548	TK2	-0.618361757	4.71E-06	-0.222759001	0.190355	-0.228106551	0.181283	0.402377166	0.009232	0.404827103	0.011964	-0.002958845	0.994924	C4	-	Y	-	-	Y	Y	-
ENSG00000166579	NDEL1	0.79364029	1.09E-19	0.407466226	0.000123	0.512344019	3.74E-07	-0.379879052	3.44E-06	-0.266918513	0.001586	0.107672987	0.494052	C5	-	Y	Y	Y	Y	-	-
ENSG00000166681	BEX3	-0.388283966	2.80E-11	-0.258727235	0.000268	-0.317265848	9.94E-07	0.13651997	0.002335	0.08610771	0.055513	-0.055845638	0.480654	C4	Y	Y	-	Y	-	-	-
ENSG00000166682	TMPRSS5	-1.209741676	0.033923	0.133111986	0.853228	-0.857837608	0.170469	1.349947032	0.015449	0.365335479	0.686912	-0.987987352	NA	C3	-	Y	-	-	Y	-	-
ENSG00000166710	B2M	-0.301490328	2.35E-07	-0.211537639	0.00331	-0.310429633	1.95E-06	0.096474309	0.112605	0.005996045	0.940842	-0.095950922	0.288265	C4	-	Y	-	Y	-	-	-
ENSG00000166803	PCLAF	-0.279509143	0.001146	-0.162612653	0.144148	-0.308973662	0.0014	0.123219149	0.255457	-0.014869972	0.916696	-0.143251456	0.362994	C4	-	-	-	Y	-	-	-
ENSG00000166813	KIF7	0.324118992	0.00311	0.053935417	0.757919	0.105369937	0.490548	-0.263659696	0.012807	-0.203994837	0.076557	0.054213174	0.819909	C5	-	Y	-	-	-	-	-
ENSG00000166822	TMEM170A	0.434403649	1.75E-09	0.063518572	0.612745	0.319409052	8.40E-05	-0.364890481	1.91E-07	-0.100386581	0.197567	0.259088492	0.004047	C5	-	Y	-	Y	Y	-	-
ENSG00000166825	ANPEP	-1.341631713	5.57E-10	-0.64016979	0.022173	-0.630154161	0.006939	0.710366052	0.019398	0.72599374	0.006998	0.0115808	0.988432	C3	-	Y	Y	Y	Y	Y	-
ENSG00000166831	RBPMS2	0.370943479	0.006198	0.145221049	0.477796	0.229794482	0.157124	-0.219288933	0.153011	-0.126329166	0.425222	0.087317348	0.763197	C5	-	Y	-	-	-	-	-
ENSG00000166833	NAV2	0.12716748	0.09213	0.330008716	0.00012	0.379784452	1.44E-06	0.209001925	4.61E-05	0.267180007	4.53E-09	0.052608967	0.67662	C4	-	-	Y	Y	-	-	-
ENSG00000166845	C18orf54	0.394042618	1.13E-05	0.076929427	0.608315	0.202805315	0.076938	-0.310427531	0.000324	-0.176584238	0.057208	0.128596052	0.410319	C5	-	Y	-	-	Y	-	-
ENSG00000166860	ZBTB39	0.260056376	0.000837	-0.059921765	0.573461	-0.055686247	0.662958	-0.313821213	4.86E-05	-0.301227026	0.000617	0.007650456	0.976274	C5	-	-	-	-	Y	Y	-
ENSG00000166886	NAB2	0.99890875	1.40E-52	0.303402947	0.000574	0.261024653	0.006084	-0.688739911	1.55E-26	-0.723342257	1.38E-26	-0.039334784	0.842595	C5	-	Y	Y	-	Y	Y	-
ENSG00000166889	PATL1	0.615172925	1.29E-16	0.07011288	0.570149	0.053590316	0.653882	-0.538684009	7.23E-24	-0.547138615	2.41E-25	-0.013843573	0.933535	C5	-	Y	-	-	Y	Y	-
ENSG00000166922	SCG5	-0.373096014	0.031309	0.008101562	0.978702	0.071186027	0.753392	0.38773463	0.029496	0.459302695	0.005903	0.066099773	0.851731	C4	-	Y	-	-	Y	Y	-
ENSG00000166923	GREM1	2.313098861	1.30E-118	2.20301335	5.00E-58	1.982547869	4.42E-47	-0.104013753	0.493975	-0.315198309	0.009696	-0.220213834	0.601463	C1	-	Y	Y	Y	-	Y	-
ENSG00000166924	NYAP1	0.572829828	3.46E-13	0.367399657	0.000418	0.54134858	2.93E-11	-0.199145765	0.035725	-0.016812016	0.88285	0.177459534	0.168635	C1	-	Y	Y	Y	-	-	-
ENSG00000166925	TSC22D4	0.751089105	1.15E-14	0.33935492	0.00851	0.513606536	3.27E-06	-0.404700127	3.38E-06	-0.222709403	0.01037	0.176813325	0.200021	C5	-	Y	Y	Y	Y	-	-
ENSG00000166946	CCNDBP1	-0.315549035	0.000112	-0.235850207	0.028491	-0.122658361	0.268938	0.086233837	0.468405	0.207720562	0.028539	0.116088589	0.503848	C4	-	Y	-	-	-	-	-
ENSG00000166963	MAP1A	0.257267391	8.64E-05	0.296390118	2.34E-05	0.329954128	2.71E-07	0.045549328	0.55304	0.087252278	0.167664	0.036417012	0.771677	C4	Y	-	-	Y	-	-	-
ENSG00000166986	MARS1	-0.273510801	9.17E-13	-0.375464956	7.04E-08	-0.179451572	0.010529	-0.095489355	0.161578	0.108504352	0.067863	0.199104182	0.053808	C4	-	-	Y	-	-	-	-

ENSG00000166987	MBD6	0.390404795	1.90E-05	0.113869672	0.453462	0.310372739	0.001997	-0.269971283	0.009043	-0.065037928	0.557439	0.199670981	0.169632	C5	-	Y	-	Y	-	-	-
ENSG00000166997	CNPY4	0.31309695	0.000199	-0.034174749	0.821576	0.114199625	0.353865	-0.340765154	9.55E-05	-0.184118915	0.062861	0.15114086	0.316758	C5	-	Y	-	-	Y	-	-
ENSG00000167011	NAT16	0.560662792	1.20E-09	0.463578916	3.83E-06	0.509352691	3.90E-07	-0.090622218	0.421101	-0.036617697	0.780979	0.048638216	0.818398	C1	-	Y	Y	Y	-	-	-
ENSG00000167037	SGSM1	0.182607387	0.024271	0.391349872	7.64E-07	0.506688302	6.05E-12	0.215174829	0.005024	0.338790489	1.55E-06	0.118394779	0.282479	C4	-	-	Y	Y	-	Y	-
ENSG00000167074	TEF	-0.79569222	1.00E-08	-0.182589612	0.381045	-0.394232488	0.012936	0.620766992	0.000291	0.416322693	0.016377	-0.209580269	0.472263	C2	-	Y	-	Y	Y	Y	-
ENSG00000167094	TTC16	-1.499874349	0.042845	-0.928375774	NA	-1.307850942	0.119266	0.577489823	0.566843	0.205773641	0.873237	-0.376615382	NA	C3	-	Y	-	-	-	-	-
ENSG00000167103	PIP5KL1	-0.171634946	0.430342	0.106387816	0.711017	0.160843957	0.530778	0.284853436	0.101028	0.347718257	0.0456	0.057469214	0.885268	C4	-	-	-	-	-	Y	-
ENSG00000167107	ACSF2	-0.89009269	4.81E-06	-0.432486166	0.055697	-0.243910208	0.269002	0.465725693	0.047761	0.661352442	0.001166	0.191659408	0.58778	C2	-	Y	-	-	Y	Y	-
ENSG00000167123	CERCAM	-0.190838608	0.239381	0.091246372	0.70965	0.317388538	0.028236	0.288802396	0.08266	0.52345431	4.24E-05	0.230012439	0.280913	C4	-	-	-	Y	-	Y	-
ENSG00000167130	DOLPP1	0.066724632	0.504216	-0.320316842	0.000599	-0.220388728	0.039483	-0.380499366	2.53E-06	-0.27234693	0.002418	0.103011527	0.549988	C4	-	-	Y	-	Y	-	-
ENSG00000167182	SP2	-0.506042813	1.12E-08	-0.390463187	2.01E-05	-0.584226132	1.86E-12	0.121767456	0.288526	-0.063377958	0.611521	-0.19076036	0.138485	C4	-	Y	Y	Y	-	-	-
ENSG00000167186	COQ7	-0.159127826	0.128478	0.136814217	0.255812	-0.052470727	0.710447	0.302376892	0.000298	0.121384272	0.222541	-0.186332427	0.097331	C4	-	-	-	-	Y	-	-
ENSG00000167196	FBXO22	0.388778695	9.78E-10	0.164207592	0.05418	0.179595598	0.025609	-0.217969182	0.000644	-0.194479898	0.002215	0.018193805	0.921144	C5	Y	Y	-	-	-	-	-
ENSG00000167202	TBC1D2B	0.353434215	0.001211	0.116639744	0.489506	0.188936525	0.157247	-0.230382033	0.01872	-0.15027874	0.128073	0.074589062	0.698058	C5	-	Y	-	-	-	-	-
ENSG00000167216	KATNAL2	-0.83462961	1.13E-06	-0.184448972	0.419778	-0.095185361	0.720853	0.656845884	0.000145	0.754557282	3.83E-05	0.092041397	0.811484	C4	-	Y	-	-	Y	Y	-
ENSG00000167232	ZNF91	0.228983263	0.040165	-0.081730689	0.61624	0.107818293	0.465529	-0.305429037	2.83E-05	-0.107238443	0.22324	0.192734616	0.077289	C5	-	-	-	-	Y	-	-
ENSG00000167257	RNF214	0.408158822	2.06E-08	0.143578724	0.24856	0.138092582	0.149618	-0.257878562	0.00238	-0.255346298	0.000222	-0.0027038	0.991899	C5	-	Y	-	-	-	-	-
ENSG00000167280	ENGASE	-0.114658845	0.128684	0.109047541	0.312275	0.320099401	1.76E-05	0.230092467	0.004343	0.449575118	2.68E-11	0.213811394	0.049867	C4	-	-	-	Y	-	Y	-
ENSG00000167283	ATP5MG	-0.224358357	0.000481	-0.157431674	0.053592	-0.305396145	2.87E-06	0.073550223	0.338231	-0.066312434	0.356455	-0.145214119	0.108554	C4	-	-	-	Y	-	-	-
ENSG00000167306	MYO5B	-1.079443863	0.01751	0.3260614	0.584301	0.240836808	0.707539	1.410470353	0.003789	1.334637936	0.010682	-0.079702348	NA	C2	-	Y	-	-	Y	Y	-
ENSG00000167315	ACAA2	-0.319425205	1.64E-09	-0.138794356	0.052208	-0.116517626	0.075926	0.18711039	0.000938	0.217581322	2.19E-05	0.025141984	0.850887	C4	-	Y	-	-	-	-	-
ENSG00000167323	STIM1	-0.318634441	0.000599	0.001992338	0.991198	0.110892705	0.336563	0.327132586	0.000486	0.444317367	7.94E-07	0.111930833	0.454595	C4	-	Y	-	-	Y	Y	-
ENSG00000167333	TRIM68	0.27458171	0.074153	-0.095032905	0.695488	0.091320288	0.677791	-0.363388208	0.026503	-0.168614614	0.347169	0.189227113	0.498731	C5	-	-	-	-	Y	-	-
ENSG00000167363	FN3K	-0.233330848	0.078471	0.135389534	0.427814	0.243644479	0.130048	0.375667846	0.004754	0.492155297	0.000426	0.110248731	0.688155	C4	-	-	-	-	Y	Y	-
ENSG00000167378	IRGQ	0.370347838	3.48E-09	0.066429162	0.471998	0.18912548	0.004289	-0.297553699	2.29E-06	-0.166651413	0.007596	0.125598245	0.15151	C5	-	Y	-	-	-	-	-
ENSG00000167384	ZNF180	-0.172356463	0.088488	-0.182338603	0.154506	-0.334601426	0.001295	-0.003174006	0.981058	-0.147683612	0.147504	-0.14985217	0.333965	C4	-	-	-	Y	-	-	-
ENSG00000167394	ZNF668	-0.309246598	0.028669	-0.208013845	0.219461	-0.322638458	0.028396	0.107523819	0.570419	0.001202705	0.995237	-0.111808293	0.690042	C4	-	Y	-	Y	-	-	-
ENSG00000167395	ZNF646	0.129835918	0.122315	-0.286936768	0.003684	-0.012280673	0.932347	-0.410412491	3.66E-08	-0.127668421	0.104088	0.277448754	0.008821	C5	-	-	-	-	Y	-	-
ENSG00000167414	GNG8	-0.734809838	8.50E-11	-0.38537942	0.005011	-0.230028455	0.100778	0.355277697	0.001032	0.520090375	1.33E-07	0.159507753	0.349796	C4	Y	Y	Y	-	Y	Y	-
ENSG00000167460	TPM4	0.637089635	1.18E-91	0.172601571	0.000108	0.031499952	0.603042	-0.457906735	1.73E-54	-0.591001667	3.29E-97	-0.138230506	0.004543	C5	Y	Y	-	-	Y	Y	-
ENSG00000167476	JSRP1	-0.592103232	0.015987	-0.043513804	0.909342	-0.26578942	0.377052	0.5560939	0.031671	0.341248003	0.258442	-0.219901779	0.610489	C4	-	Y	-	-	Y	-	-
ENSG00000167508	MVD	-0.084364057	0.537542	0.008476419	0.971342	0.22773263	0.101448	0.099590143	0.492708	0.326597552	0.003637	0.222257009	0.223973	C4	-	-	-	-	-	Y	-
ENSG00000167513	CDT1	-0.161308208	0.072367	-0.216262493	0.043024	-0.448936681	1.10E-06	-0.04826915	0.593901	-0.273013927	7.45E-05	-0.229946215	0.018555	C4	-	-	-	Y	-	-	-
ENSG00000167523	SPATA33	0.342814019	0.034366	0.195077793	0.383359	0.208309197	0.389295	-0.141234925	0.47641	-0.120202085	0.593163	0.016319034	0.976274	C5	-	Y	-	-	-	-	-
ENSG00000167524	RSKR	-0.219233642	0.075129	0.135697436	0.383294	0.09813038	0.4839	0.361565315	0.002328	0.331899187	0.002209	-0.0347082	0.896869	C4	-	-	-	-	Y	Y	-
ENSG00000167525	PROCA1	-1.14461155	2.67E-12	-0.20718243	0.256127	-0.34933378	0.039151	0.944979827	1.12E-07	0.809782272	2.03E-05	-0.139739722	0.643991	C3	-	Y	-	Y	Y	Y	-
ENSG00000167528	ZNF641	0.663285426	3.18E-11	0.204308162	0.181815	0.250099065	0.057105	-0.452785896	2.24E-06	-0.398496098	1.55E-05	0.048707916	0.845294	C1	-	Y	-	-	Y	Y	-
ENSG00000167548	KMT2D	0.598373875	6.98E-06	0.267590251	0.022256	0.365014656	0.000262	-0.324380861	4.27E-07	-0.219070856	0.000138	0.100000426	0.395618	C1	-	Y	-	Y	Y	-	-
ENSG00000167549	CORO6	-0.747066611	9.90E-22	-0.214563882	0.089781	-0.2067532	0.021708	0.539305678	4.15E-07	0.555045002	4.66E-11	0.010443903	0.972936	C4	-	Y	-	-	Y	Y	-
ENSG00000167550	RHEBL1	0.416670783	0.031388	0.350347173	0.141863	0.070527393	0.832466	-0.059952364	0.81656	-0.331875166	0.126434	-0.276998776	0.407972	C5	-	Y	-	-	-	-	-
ENSG00000167554	ZNF610	-0.210188561	0.218656	-0.584137892	0.001338	-0.019677424	0.941152	-0.367531553	0.045238	0.205578848	0.256409	0.567249284	0.00642	C4	-	-	Y	-	Y	-	Y
ENSG00000167555	ZNF528	-0.508023678	2.60E-06	-0.070334139	0.626781	-0.07611647	0.614841	0.444209606	4.25E-06	0.44678584	2.00E-05	-0.00263699	0.992234	C4	-	Y	-	-	Y	Y	-
ENSG00000167565	SERTAD3	-0.138181459	0.594241	-0.550050662	0.043028	-0.08347191	0.797727	-0.405408165	0.077455	0.068584773	0.793485	0.469282257	0.119511	C5	-	-	Y	-	-	-	-
ENSG00000167604	NFKBID	0.402563137	0.104063	-0.214562701	0.552141	0.074899931	0.856978	-0.610683188	0.012134	-0.313757662	0.265492	0.292204757	0.524522	C5	-	-	-	-	Y	-	-
ENSG00000167625	ZNF526	0.170446122	0.257723	-0.393471996	0.053064	-0.018026742	0.945888	-0.557874993	0.000506	-0.174182673	0.287208	0.378102142	0.144014	C5	-	-	-	-	Y	-	-
ENSG00000167632	TRAPPC9	-0.583646218	1.58E-09	0.00539375	0.976462	-0.02551614	0.871855	0.595323341	1.03E-10	0.572523347	9.16E-10	-0.028380312	0.902249	C4	-	Y	-	-	Y	Y	-
ENSG00000167641	PPP1R14A	-1.338250549	0.000635	-0.815788027	0.059728	-0.748542242	0.028609	0.540525718	0.382688	0.605756781	0.250388	0.065693155	NA	C3	-	Y	-	Y	-	-	-
ENSG00000167657	DAPK3	0.431711203	9.17E-07	0.23383171	0.07359	0.113632572	0.40571	-0.191285783	0.043131	-0.30361303	0.000204	-0.117360385	0.509778	C5	Y	Y	-	-	-	Y	-
ENSG00000167664	TMIGD2	-0.671045709	0.022313	-0.778000657	0.013012	-0.317011652	0.409313	-0.09991622	0.836697	0.369135268	0.36377	0.463347101	NA	C4	-	Y	Y	-	-	-	-
ENSG00000167671	UBXN6	-0.343714039	5.29E-08	-0.081946516	0.343678	-0.193343621	0.005897	0.268268797	2.46E-05	0.165147738	0.013996	-0.108404834	0.257932	C4	Y	Y	-	-	-	-	-
ENSG00000167676	PLIN4	-0.127908014	0.766312	0.701006627	0.021722	0.568425793	0.073067	0.836490211	0.005792	0.711202352	0.025985	-0.129413259	NA	C2	-	-	Y	-	Y	Y	-
ENSG00000167695	TLCD3A	0.307192426	0.000136	0.063791781	0.607509	0.024405673	0.88894	-0.236995568	0.005315	-0.268378317	0.008272	-0.036892857	0.887679	C5	-	Y	-	-	-	-	-
ENSG00000167700	MFSD3	-0.700317433	2.35E-07	-0.036646499	0.872207	0.057043233	0.771006	0.669989729	5.45E-06	0.772107711	6.25E-08	0.096541095	0.723283	C2	-	Y	-	-	Y	Y	-
ENSG00000167701	GPT	-0.558091073	0.05493	0.002122042	0.996745	0.350755789	0.264543	0.565887937	0.045006	0.923171664	0.000356	0.351515143	0.362143	C4	-	-	-	-	Y	Y	-
ENSG00000167703	SLC43A2	-0.62440706	4.38E-05	-0.576237549	0.001641	-0.259763183	0.113798	0.055398939	0.836968	0.379616317	0.022552	0.319635306	0.19895	C4	-	Y	Y	-	-	Y	-
ENSG00000167711	SERPINF2	1.354907607	0.011072	0.625294522	0.457296	0.982447429	0.132031	-0.721368176	0.148129	-0.357429482	0.487527	0.358627506	NA	C1	-	Y	-	-	-	-	-
ENSG00000167720	SRR	-0.486566513	2.93E-06	-0.19509425	0.146461	-0.256916683	0.039151	0.297697702	0.0133	0.244402922	0.054624	-0.058696128	0.824867	C4	-	Y	-	-	-	-	-
ENSG00000167723	TRPV3	-4.750496014	0.007292	-0.881281985	NA	-0.864022756	NA	3.886290608	NA	3.898731101	NA	0.0183375	NA	C3	-	Y	-	-	-	-	-
ENSG00000167740	CYB5D2	-0.945875827	4.12E-10	-0.175434702	0.384969	-0.537721265	0.001256	0.778105455	4.32E-06	0.422844544	0.03161	-0.360694873	0.137352	C3	-	Y	-	Y	Y	Y	-
ENSG00000167747	C19orf48	-0.268654734	0.017412	-0.205856889	0.146611	-0.393610911	0.001561	0.070007031	0.403449	-0.110069976	0.195052	-0.185243445	0.092318	C4	-	-	-	Y	-	-	-
ENSG00000167771	RCOR2	-0.467873825	4.04E-10	-0.089172895	0.440346	-0.046019606	0.716279	0.385212676	2.15E-07	0.436887693	8.11E-09	0.046127848	0.794803	C4	Y	Y	-	-	Y	Y	-
ENSG00000167772	ANGPTL4	-0.038350282	0.927892	0.200191254	0.642192	0.967168258	5.15E-05	0.244850694	0.481703	1.020022521	7.59E-06	0.77230647	0.00659	C4	-	-	-	Y	-	Y	Y
ENSG00000167863	ATP5PD	-0.352496895	1.38E-06	-0.253865138	0.010898	-0.273102342	0.001282	0.105034744	0.177248	0.094153115	0.15558	-0.016199719	0.935441	C4	-	Y	-	-	-	-	-
ENSG00000167874	TMEM88	-0.490792692	0.272974	-0.106459909	0.87507	0.352652628	0.427193	0.392251999	0.452303	0.85823647	0.02964	0.463338452	NA	C4	-	-	-	-	-	Y	-
ENSG00000167881	SRP68	0.364594697	3.34E-14	0.022531016	0.812869	0.016778678	0.83963	-0.335611076	6.19E-13	-0.33317605	4.52E-15	-0.002978984	0.982455	C5	Y	Y	-	-	Y	Y	-
ENSG00000167889	MGAT5B	-0.404321639	2.94E-06	-0.364078578	0.000746	-0.290453088	0.002173	0.046410222	0.74222	0.128585445	0.222244	0.076705455	0.707985	C4	-	Y	Y	-	-	-	-
ENSG00000167900	TK1	-0.482954944	1.72E-08	-0.231513477	0.029928	-0.289849447	0.003117	0.2589089	0.000217	0.207972525	0.003431	-0.056237051	0.686456	C4	-	Y	-	-	-	-	-

ENSG00000167920	TMEM99	-0.470688776	0.015333	-0.338185987	0.213362	-0.285851307	0.265445	0.138926323	0.648207	0.199558145	0.464833	0.054247518	0.93466	C4	-	Y	-	-	-	-	-
ENSG00000167930	FAM234A	-0.313728355	9.51E-09	-0.021777782	0.859906	-0.092460642	0.186072	0.298284795	8.55E-07	0.236131805	8.40E-07	-0.067349676	0.546192	C4	-	Y	-	-	-	-	-
ENSG00000167964	RAB26	-0.747802917	0.036243	0.025757728	0.967843	-0.087971998	0.856978	0.782479408	0.051668	0.675002063	0.076117	-0.110716478	NA	C3	-	Y	-	-	-	-	-
ENSG00000167965	MLST8	-0.323623611	5.64E-05	-0.116015718	0.322032	-0.123310803	0.234467	0.213765062	0.009654	0.215085743	0.005523	-0.003931893	0.986496	C4	-	Y	-	-	-	-	-
ENSG00000167969	ECI1	-0.384073306	5.02E-05	-0.229705631	0.061652	-0.222568597	0.051977	0.161519412	0.099289	0.176026779	0.05733	0.009423594	0.97113	C4	-	Y	-	-	-	-	-
ENSG00000167971	CASKIN1	0.569682761	6.86E-06	0.243946623	0.11328	0.503289588	4.99E-05	-0.319303245	0.006351	-0.051867993	0.728975	0.262204475	0.058766	C1	-	Y	-	Y	Y	-	-
ENSG00000167972	ABCA3	-0.326128527	3.51E-09	0.139091632	0.023971	0.151070286	0.009714	0.471799155	3.96E-21	0.491852914	1.18E-24	0.014954811	0.905466	C4	Y	Y	-	-	Y	Y	-
ENSG00000167981	ZNF597	0.1639425	0.350085	-0.336938483	0.083321	-0.099704738	0.688282	-0.494454907	0.001554	-0.248947541	0.159097	0.239935103	0.39874	C5	-	-	-	-	Y	-	-
ENSG00000167985	SDHAF2	-0.442643643	0.003383	-0.180620867	0.390024	-0.304519738	0.063434	0.267984803	0.123761	0.152630001	0.398238	-0.121386815	0.680347	C4	-	Y	-	-	-	-	-
ENSG00000167992	VWCE	-0.254577614	0.096052	0.001845454	0.994903	0.051281785	0.811018	0.262518283	0.118954	0.320776377	0.039616	0.052651505	0.88523	C4	-	-	-	-	-	Y	-
ENSG00000167994	RAB3IL1	-0.435209912	0.02601	-0.121623258	0.66362	-0.033642882	0.910789	0.319847099	0.115724	0.416102915	0.030793	0.090642764	0.81032	C4	-	Y	-	-	-	Y	-
ENSG00000167995	BEST1	-0.644709235	0.027236	-0.783099793	0.013437	-0.257851754	0.459993	-0.132219493	0.762231	0.402170025	0.222744	0.528446449	NA	C3	-	Y	Y	-	-	-	-
ENSG00000167996	FTH1	-0.280737764	3.20E-06	-0.269398216	1.73E-05	-0.398812821	2.31E-11	0.018070882	0.764021	-0.103121933	0.021875	-0.126456196	0.00828	C4	-	-	-	Y	-	-	-
ENSG00000168003	SLC3A2	-0.301332853	8.33E-05	-0.220793865	0.106506	-0.196900576	0.029034	0.085622795	0.67134	0.119158404	0.165993	0.028168817	0.948525	C4	-	Y	-	-	-	-	-
ENSG00000168005	SPINDOC	0.357693669	5.09E-09	0.039825462	0.720225	-0.007076751	0.952284	-0.3114609	3.32E-07	-0.350142085	6.45E-10	-0.044007259	0.749894	C5	-	Y	-	-	Y	Y	-
ENSG00000168010	ATG16L2	-0.300526332	0.001059	0.180705922	0.080871	0.203029147	0.088343	0.487776345	2.75E-09	0.518289959	4.63E-08	0.024979112	0.921602	C4	-	Y	-	-	Y	Y	-
ENSG00000168014	C2CD3	0.333359809	1.15E-07	0.217505752	0.0014	0.120266076	0.173634	-0.109432717	0.116816	-0.198546772	0.005465	-0.094369704	0.42666	C5	-	Y	-	-	-	-	-
ENSG00000168026	TTC21A	0.433690008	0.015317	0.353954124	0.061954	0.341230789	0.079807	-0.073593011	0.743895	-0.077711102	0.737053	-0.009875243	0.982152	C5	-	Y	-	-	-	-	-
ENSG00000168040	FADD	0.318913081	0.019387	-0.018503718	0.944012	0.078975231	0.710166	-0.330755993	0.024683	-0.225147716	0.153597	0.100121797	0.745523	C5	-	Y	-	-	Y	-	-
ENSG00000168061	SAC3D1	0.158956252	0.305621	0.012306083	0.965136	-0.226418642	0.29239	-0.139046451	0.336094	-0.371232287	0.012972	-0.235529844	0.347712	C5	-	-	-	-	-	Y	-
ENSG00000168066	SF1	-0.277971943	1.11E-10	-0.143091253	0.006584	-0.3088396	2.00E-11	0.141573161	0.000115	-0.016191779	0.753225	-0.163057749	0.000208	C4	-	-	-	Y	-	-	-
ENSG00000168071	CCDC88B	1.823068211	0.006128	0.656309075	NA	0.849219926	0.380172	-1.148181309	0.043406	-0.957245702	0.075094	0.188273493	NA	C5	-	Y	-	-	Y	-	-
ENSG00000168077	SCARA3	-0.894388819	2.61E-15	-0.607181417	1.69E-06	-0.650849564	3.68E-08	0.29392831	0.036338	0.25819919	0.063297	-0.04075391	0.900802	C4	-	Y	Y	Y	-	-	-
ENSG00000168124	OR1F1	-3.326208015	0.008689	-0.158574527	NA	0.077786283	0.949033	3.179051548	0.022622	3.417805156	0.010344	0.237751055	NA	C3	-	Y	-	-	Y	Y	-
ENSG00000168152	THAP9	-0.160451242	0.340399	-0.435936305	0.010438	-0.317062003	0.080665	-0.269117672	0.121698	-0.141999185	0.475107	0.12174197	0.719456	C4	-	-	Y	-	-	-	-
ENSG00000168172	HOOK3	0.496241803	1.84E-07	0.176126432	0.224378	0.268123422	0.028285	-0.313321518	0.000448	-0.213368746	0.016581	0.094630515	0.62517	C5	-	Y	-	-	Y	-	-
ENSG00000168209	DDIT4	-0.484889447	3.22E-06	0.42449014	6.05E-05	0.278589622	0.015939	0.915753663	1.08E-29	0.778961104	7.40E-20	-0.14209988	0.266197	C2	-	Y	Y	-	Y	Y	-
ENSG00000168228	ZCCHC4	-0.237703502	0.050268	-0.392315041	0.003807	-0.401568272	0.006815	-0.148185536	0.312321	-0.149299159	0.355647	-0.005595187	0.989907	C4	-	-	Y	Y	-	-	-
ENSG00000168255	POLR2J3	-0.542820567	0.029096	-0.093141878	0.797931	-0.157844549	0.627044	0.45613499	0.025758	0.400523036	0.070863	-0.060412082	0.892434	C2	-	Y	-	-	Y	-	-
ENSG00000168259	DNAJC7	0.230661175	4.15E-07	-0.085608959	0.187078	-0.173199594	0.007247	-0.309778814	1.56E-13	-0.389338976	4.59E-17	-0.084717503	0.335949	C5	-	-	-	-	Y	Y	-
ENSG00000168275	COA6	0.247523338	0.046346	-0.091377293	0.63475	-0.017912524	0.937854	-0.332767197	0.002593	-0.250857195	0.038058	0.07645365	0.766716	C5	Y	-	-	-	Y	-	-
ENSG00000168280	KIF5C	0.36473831	2.36E-13	0.057056101	0.505594	0.054513455	0.444981	-0.301004922	1.15E-16	-0.295700325	7.19E-24	-5.31E-05	0.99962	C5	-	Y	-	-	Y	-	-
ENSG00000168297	PXK	0.719248426	1.14E-08	0.657347172	1.17E-05	0.546406014	9.32E-05	-0.05555844	0.756881	-0.158213125	0.26415	-0.108261188	0.678254	C1	-	Y	Y	Y	-	-	-
ENSG00000168301	KCTD6	0.423570914	3.72E-05	-0.100024747	0.540461	0.125084119	0.390072	-0.517213238	9.25E-07	-0.283639845	0.010739	0.228116274	0.16309	C5	-	Y	-	-	Y	-	-
ENSG00000168310	IRF2	0.919581955	4.26E-10	0.603979104	0.000109	0.77787392	1.15E-07	-0.309313643	0.039232	-0.12720991	0.453883	0.177011164	0.43015	C1	-	Y	Y	Y	Y	-	-
ENSG00000168348	INSM2	-0.904238576	1.39E-26	-1.052145573	2.11E-38	-1.166516552	1.07E-41	-0.141476712	0.245737	-0.247333175	0.034259	-0.111159602	0.567996	C3	-	Y	Y	Y	-	-	-
ENSG00000168386	FILIP1L	-0.724225226	9.01E-06	-0.765788031	7.73E-05	-0.417074902	0.009042	-0.03377013	0.914791	0.321580767	0.086434	0.351760965	0.203883	C3	-	Y	Y	Y	-	-	-
ENSG00000168389	MFSD2A	0.306051635	0.020801	0.094699754	0.681995	-0.015381667	0.950401	-0.205261812	0.209533	-0.306843743	0.035727	-0.106260048	0.746484	C5	-	Y	-	-	-	Y	-
ENSG00000168393	DTYMK	-0.353946047	7.73E-06	-0.073356484	0.56679	-0.167398581	0.106738	0.287313052	2.74E-05	0.201576616	0.006359	-0.091104047	0.493975	C4	-	Y	-	-	-	-	-
ENSG00000168411	RFWD3	-0.252068112	5.97E-06	-0.155111103	0.042847	-0.303618921	2.61E-06	0.103626235	0.092066	-0.037149716	0.601121	-0.145713139	0.098285	C4	-	-	-	Y	-	-	-
ENSG00000168434	COG7	0.464593523	0.00081	0.275592861	0.098981	0.247359941	0.12882	-0.182211511	0.166934	-0.202317988	0.116633	-0.025389474	0.933843	C5	-	Y	-	-	-	-	-
ENSG00000168439	STIP1	0.328774542	1.32E-14	0.138021638	0.043537	0.242360993	6.59E-06	-0.184063592	6.68E-05	-0.071766053	0.111925	0.107216624	0.178624	C5	-	Y	-	-	-	-	-
ENSG00000168461	RAB31	-0.318865043	0.000122	-0.093065458	0.531839	-0.365437447	0.000124	0.232570131	0.035746	-0.031930504	0.816941	-0.27048153	0.075594	C4	-	Y	-	Y	-	-	-
ENSG00000168481	LGI3	-0.958447599	0.016149	-0.748529115	0.117254	-0.099821385	0.857403	0.21408458	0.720919	0.872613009	0.031376	0.649961176	NA	C4	-	Y	-	-	-	Y	-
ENSG00000168490	PHYHIP	-1.33307204	6.88E-06	-0.294061349	0.428121	-0.527576605	0.117295	1.046807511	0.000798	0.820528608	0.019109	-0.230782011	NA	C3	-	Y	-	-	Y	Y	-
ENSG00000168496	FEN1	-0.267062403	1.04E-07	-0.290167501	0.00011	-0.454556881	1.88E-10	-0.016312418	0.843894	-0.173018408	0.003789	-0.161325147	0.140447	C4	-	-	-	Y	-	-	-
ENSG00000168502	MTCL1	0.060964672	0.652785	0.26066545	0.020392	0.301427898	0.008699	0.206108389	0.046638	0.254897511	0.01688	0.043491017	0.848977	C4	-	-	-	Y	-	-	-
ENSG00000168522	FNTA	0.380243836	1.90E-09	-0.098541412	0.256011	0.018847297	0.872891	-0.472237742	2.62E-16	-0.346734522	1.34E-08	0.120279874	0.231807	C5	Y	Y	-	-	Y	Y	-
ENSG00000168528	SERINC2	-0.664759988	0.000173	-1.552419074	0.002505	-0.366952072	0.048836	0.118793869	0.59698	0.312503571	0.090693	0.189169765	0.479268	C4	-	Y	Y	Y	-	-	-
ENSG00000168539	CHRM1	-0.260565162	0.00882	0.124057937	0.414607	0.201525018	0.07997	0.391495917	7.27E-05	0.476840227	2.67E-08	0.080246305	0.691815	C4	-	-	-	-	Y	Y	-
ENSG00000168542	COL3A1	-0.542876586	0.007643	-0.652750404	0.000298	-0.081668391	0.779672	-0.099447593	0.757661	0.476072002	0.227985	0.57062595	0.053192	C4	-	Y	Y	-	-	-	-
ENSG00000168546	GFRA2	-0.160608465	0.737973	-0.996239968	0.033948	-0.848903378	0.066822	-0.82973887	0.072587	-0.673567777	0.155968	0.152038354	NA	C5	-	-	Y	-	-	-	-
ENSG00000168566	SNRNP48	0.639799061	7.87E-11	0.214598104	0.077278	0.348878393	0.001729	-0.419041896	3.00E-06	-0.276358225	0.003958	0.137350342	0.368678	C5	-	Y	-	Y	Y	-	-
ENSG00000168610	STAT3	0.424158071	2.36E-09	0.200696604	0.018706	0.213152827	0.007782	-0.217247886	0.001446	-0.196215118	0.003251	0.015738564	0.931126	C5	-	Y	-	-	-	-	-
ENSG00000168646	AXIN2	-0.170080788	0.143145	-0.308325483	0.025545	-0.2206396	0.087613	-0.131393989	0.327066	-0.035634364	0.810701	0.090473097	0.695748	C4	-	-	Y	-	-	-	-
ENSG00000168661	ZNF30	0.546624683	1.83E-06	0.02893362	0.902374	0.289642276	0.048534	-0.511399717	1.97E-05	-0.242105389	0.059548	0.263655459	0.160075	C5	-	Y	-	-	Y	-	-
ENSG00000168675	LDLRAD4	-0.696077743	0.016894	0.019819128	0.965469	-0.063699986	0.889326	0.725354284	0.014705	0.647690989	0.055416	-0.082599003	0.906331	C2	-	Y	-	-	Y	-	-
ENSG00000168676	KCTD19	-0.860388943	0.000544	-0.083273706	0.830693	0.178932421	0.557332	0.784532992	0.003103	1.054038262	1.54E-05	0.265168017	0.497614	C4	-	Y	-	-	Y	Y	-
ENSG00000168701	TMEM208	-0.226889957	0.056765	-0.136891985	0.379366	-0.311846859	0.013217	0.097163863	0.412483	-0.070048441	0.579429	-0.172644364	0.243664	C4	-	-	-	Y	-	-	-
ENSG00000168724	DNAJC21	0.334943665	4.88E-05	0.174736695	0.097711	0.250825527	0.00974	-0.153979256	0.026544	-0.069659481	0.395009	0.078909501	0.529229	C5	-	Y	-	-	-	-	-
ENSG00000168765	GSTM4	-0.295505121	0.029357	-0.139331889	0.374028	-0.007346168	0.972173	0.162659341	0.306498	0.30280621	0.034	0.134818122	0.528427	C4	-	-	-	-	-	Y	-
ENSG00000168769	TET2	0.3761215	1.14E-06	0.239750893	0.010935	0.219839748	0.025676	-0.130217187	0.09003	-0.141464851	0.080058	-0.016907357	0.937494	C1	-	Y	-	-	-	-	-
ENSG00000168772	CXXC4	0.395477874	1.13E-06	0.331490981	0.004946	0.518075057	1.36E-08	-0.05755874	0.663221	0.137527401	0.152405	0.189215083	0.233149	C5	-	Y	Y	Y	-	-	-
ENSG00000168802	CHTF8	-0.39616978	0.00949	-0.160129927	0.387497	-0.075777494	0.734557	0.243136197	0.170011	0.335141987	0.061552	0.086793079	0.796431	C4	-	Y	-	-	-	-	-
ENSG00000168803	ADAL	-0.421821606	4.03E-05	-0.177584155	0.181254	-0.076229411	0.634346	0.250643916	0.030843	0.360279953	0.001716	0.10392754	0.624409	C4	-	Y	-	-	-	Y	-
ENSG00000168806	LCMT2	-0.235667359	0.006929	-0.355288523	4.95E-05	-0.256521834	0.012948	-0.113434426	0.270006	-0.006349519	0.962597	0.102103711	0.558026	C4	-	-	Y	-	-	-	-
ENSG00000168807	SNTB2	0.351352505	8.91E-05	0.186616339	0.056596	0.10707174	0.308015	-0.158632022	0.054802	-0.230005193	0.002536	-0.076497641	0.521076	C5	-	Y	-	-	-	-	-

ENSG00000168813	ZNF507	0.32275064	0.000265	-0.048859262	0.750295	0.04522061	0.744302	-0.365940424	1.95E-09	-0.263332091	7.38E-06	0.097338823	0.364794	C5	-	Y	-	-	Y	-	-
ENSG00000168826	ZBTB49	0.209676111	0.163251	-0.250082688	0.180261	-0.047556156	0.829543	-0.453458143	0.001666	-0.242919993	0.098188	0.20506872	0.392995	C5	-	-	-	-	Y	-	-
ENSG00000168830	HTR1E	-0.367967987	0.198744	-0.963954773	0.000278	-0.906563001	0.000328	-0.588491046	0.072077	-0.522728667	0.10433	0.060740425	NA	C3	-	-	Y	Y	-	-	-
ENSG00000168843	FSTL5	-0.663564962	3.47E-08	0.062224587	0.750008	-0.040256602	0.826308	0.732457648	2.41E-09	0.637990628	9.82E-08	-0.099306172	0.654537	C2	-	Y	-	-	Y	Y	-
ENSG00000168874	ATOH8	-1.533393898	3.93E-09	-0.392698368	0.219461	-0.420757618	0.135058	1.145699978	5.27E-05	1.127126758	3.62E-05	-0.025825582	0.97084	C3	-	Y	-	-	Y	Y	-
ENSG00000168883	USP39	0.375695674	2.58E-11	0.064252474	0.567453	0.001783704	0.988521	-0.304812063	1.49E-06	-0.359190801	4.96E-10	-0.059463537	0.693024	C5	-	Y	-	-	Y	Y	-
ENSG00000168887	C2orf68	-0.260463267	6.96E-06	0.194918762	0.014683	0.045213827	0.620225	0.461633701	1.28E-13	0.320389464	1.78E-08	-0.146798649	0.132933	C4	-	-	-	-	Y	Y	-
ENSG00000168904	LRRC28	0.112434498	0.408212	0.124229256	0.461666	0.324523801	0.011124	0.018416688	0.917785	0.226979742	0.068101	0.20314351	0.265467	C4	-	-	-	Y	-	-	-
ENSG00000168906	MAT2A	-0.527374747	1.17E-39	-0.13140222	0.008526	-0.26148994	2.62E-11	0.402457205	1.22E-25	0.28058596	1.47E-15	-0.127268965	0.011856	C4	Y	Y	-	-	Y	-	-
ENSG00000168913	ENHO	-0.874911244	1.53E-05	-0.563924263	0.019121	-0.231704757	0.364938	0.318982071	0.241204	0.658220579	0.002601	0.334884523	0.341115	C3	-	Y	Y	-	-	Y	-
ENSG00000168916	ZNF608	-0.738682011	3.87E-12	-0.292308785	0.015474	-0.013393548	0.93983	0.453138741	0.000132	0.739929872	8.22E-12	0.281826097	0.042913	C4	-	Y	-	-	Y	Y	-
ENSG00000168917	SLC35G2	0.28026204	0.00375	-0.155375451	0.271557	-0.063143601	0.699124	-0.428878549	5.85E-05	-0.328755385	0.002677	0.094901184	0.693024	C5	-	-	-	-	Y	Y	-
ENSG00000168918	INPP5D	-0.98175454	0.001653	0.320042323	0.357958	0.184366495	0.671102	1.307722655	1.20E-05	1.181180042	0.000489	-0.132195701	NA	C3	-	Y	-	-	Y	Y	-
ENSG00000168924	LETM1	0.362558059	3.46E-13	0.106467352	0.187946	0.023664498	0.777679	-0.24947419	8.48E-06	-0.324166445	2.21E-12	-0.079982806	0.421345	C5	-	Y	-	-	-	Y	-
ENSG00000168936	TMEM129	-0.803207611	2.13E-14	0.006994225	0.973219	-0.14791118	0.249444	0.81648865	2.44E-18	0.670549485	3.51E-15	-0.150879254	0.27291	C2	-	Y	-	-	Y	Y	-
ENSG00000168938	PPIC	-0.505978344	0.109198	-0.022791337	0.970129	0.156942203	0.687284	0.489506486	0.205659	0.677510844	0.026168	0.182677243	NA	C2	-	-	-	-	-	Y	-
ENSG00000168970	JMJD7-PLA2G4B	-0.242239484	0.183205	0.088568564	0.729928	0.126747174	0.53779	0.337197459	0.052824	0.383458405	0.015689	0.040678899	0.914963	C4	-	-	-	-	-	Y	-
ENSG00000168994	PXDC1	0.895634959	0.014205	1.009566119	0.01259	0.81649923	0.046913	0.119816517	0.797108	-0.064819639	0.897647	-0.190234763	NA	C1	-	Y	Y	Y	-	-	-
ENSG00000169019	COMMD8	0.377658886	0.006871	0.045482048	0.881053	0.038328368	0.872826	-0.325672507	0.055418	-0.324609701	0.023945	-0.004476034	0.992964	C5	Y	Y	-	-	-	Y	-
ENSG00000169020	ATP5ME	-0.303718633	0.013663	-0.164191079	0.317315	-0.146198593	0.335737	0.145323752	0.129768	0.172264113	0.037346	0.02206167	0.918181	C4	-	Y	-	-	-	-	-
ENSG00000169032	MAP2K1	0.558028232	8.71E-13	0.41979375	3.97E-08	0.349735376	5.11E-05	-0.131573927	0.132176	-0.193749346	0.027218	-0.067348212	0.666351	C5	-	Y	Y	Y	-	-	-
ENSG00000169047	IRS1	0.352408007	0.023329	0.386245272	0.027366	0.389062521	0.020777	0.040170937	0.834298	0.05127068	0.785818	0.005513617	0.98852	C1	-	Y	Y	Y	-	-	-
ENSG00000169057	MECP2	0.433853792	2.91E-08	0.049291907	0.706915	0.221973734	0.017365	-0.378792215	6.20E-13	-0.197656339	0.000715	0.175704263	0.021709	C5	-	Y	-	-	Y	-	-
ENSG00000169064	ZBBX	-4.436754246	0.003827	-1.112472855	NA	-0.416876067	NA	3.322519582	NA	4.035492727	0.011143	0.697272448	NA	C3	-	Y	-	-	-	Y	-
ENSG00000169071	ROR2	-0.389110342	0.000133	-0.091026105	0.568923	-0.146329595	0.304788	0.304566607	0.00656	0.257465273	0.030289	-0.05237999	0.844772	C4	-	Y	-	-	Y	-	-
ENSG00000169087	HSPBAP1	0.301317351	0.02692	-0.073459718	0.751381	0.044537857	0.831744	-0.367959354	0.0057	-0.241940365	0.061406	0.12047913	0.61711	C5	-	Y	-	-	Y	-	-
ENSG00000169093	ASMTL	-0.383844713	0.000335	-0.205977782	0.118927	-0.210892907	0.09305	0.184276839	0.156538	0.187731978	0.148825	-0.001977614	0.995809	C4	-	Y	-	-	-	-	-
ENSG00000169105	CHST14	-0.318686097	0.0001	-0.085129266	0.489035	-0.167122185	0.132772	0.239865134	0.003175	0.166358994	0.075916	-0.079096366	0.635328	C4	-	Y	-	-	-	-	-
ENSG00000169118	CSNK1G1	0.546454648	9.28E-09	0.332510491	0.003621	0.293691997	0.007392	-0.208238919	0.005537	-0.23813382	0.000694	-0.035304054	0.839863	C1	-	Y	Y	-	-	-	-
ENSG00000169129	AFAP1L2	-1.108394187	0.013915	-0.222590423	0.701441	0.027291255	0.967124	0.896136624	0.079739	1.150164703	0.016813	0.251492311	NA	C3	-	Y	-	-	-	Y	-
ENSG00000169136	ATF5	-0.215818026	0.111078	-0.210105144	0.218031	-0.632601136	8.03E-06	0.012095584	0.951638	-0.402064465	0.003298	-0.419529836	0.018555	C5	-	-	-	Y	-	Y	Y
ENSG00000169189	NSMCE1	-0.486305197	2.29E-07	-0.209923027	0.074311	-0.09868267	0.435258	0.282769224	0.003498	0.402582464	4.62E-06	0.114756286	0.456844	C4	-	Y	-	-	-	Y	-
ENSG00000169193	CCDC126	0.525051479	5.95E-06	-0.17768203	0.274353	0.158363532	0.272525	-0.695937265	6.84E-09	-0.35193763	0.002728	0.33920536	0.027648	C5	-	Y	-	-	Y	Y	Y
ENSG00000169220	RGS14	-0.187498153	0.289445	-0.055627238	0.838464	0.383796662	0.01642	0.138379176	0.495274	0.586604999	5.40E-05	0.442017208	0.020794	C4	-	-	-	Y	-	Y	Y
ENSG00000169241	SLC50A1	-0.089258409	0.523266	0.027156742	0.88585	0.283118385	0.011133	0.123045453	0.319897	0.387311682	6.38E-05	0.259114012	0.026659	C4	-	-	-	-	-	Y	-
ENSG00000169242	EFNA1	0.463410496	6.78E-05	0.180019634	0.272236	0.48590847	0.00017	-0.27700801	0.021174	0.037168977	0.819301	0.308832594	0.047395	C4	-	Y	-	Y	-	-	Y
ENSG00000169249	ZRSR2	0.394145052	0.008548	-0.00669333	0.982189	0.049153203	0.85746	-0.393437833	0.000822	-0.330382818	0.015511	0.05823496	0.862969	C5	-	Y	-	-	Y	Y	-
ENSG00000169255	B3GALNT1	-0.810650553	0.000931	-0.559984709	0.046467	-0.439946954	0.134063	0.257396324	0.425712	0.385570834	0.210921	0.122881259	NA	C4	-	Y	Y	-	-	-	-
ENSG00000169299	PGM2	0.494748345	1.90E-09	0.344079996	0.000134	0.380813112	1.36E-05	-0.144136751	0.111021	-0.099022497	0.300405	0.03986337	0.828383	C5	-	Y	Y	Y	-	-	-
ENSG00000169330	MINAR1	1.044596462	1.33E-07	0.587400134	0.004747	0.680120463	0.000571	-0.451521371	0.018412	-0.350208038	0.075379	0.095344261	0.791574	C1	-	Y	Y	Y	Y	-	-
ENSG00000169359	SLC33A1	0.30198712	0.009498	-0.125025699	0.480494	0.136291478	0.369247	-0.420509526	0.000585	-0.150841245	0.254311	0.264152866	0.130566	C5	Y	Y	-	-	Y	-	-
ENSG00000169372	CRADD	-0.535587901	0.00055	-0.185360265	0.384948	-0.230494614	0.181517	0.357596891	0.046171	0.320287164	0.056148	-0.041659761	0.916333	C4	-	Y	-	-	Y	-	-
ENSG00000169379	ARL13B	0.358974752	0.000229	-0.148285125	0.312628	-0.102423446	0.48598	-0.500651606	4.35E-07	-0.447074436	4.72E-06	0.048736339	0.853204	C5	-	Y	-	-	Y	Y	-
ENSG00000169432	SCN9A	0.009564866	0.967332	0.721220216	1.25E-05	0.845733224	6.47E-09	0.718394908	2.68E-06	0.850942721	9.07E-10	0.127046816	0.686323	C4	-	-	Y	Y	Y	Y	-
ENSG00000169439	SDC2	-0.243702775	7.85E-06	-0.337721344	0.000149	-0.312029135	8.04E-08	-0.087397916	0.326518	-0.053658143	0.396878	0.028313246	0.887679	C4	-	-	Y	Y	-	-	-
ENSG00000169499	PLEKHA2	-0.492097882	0.001049	0.151479787	0.441237	0.19902572	0.378541	0.650116591	8.88E-06	0.705952899	5.18E-05	0.049729482	0.909803	C4	-	Y	-	-	Y	Y	-
ENSG00000169504	CLIC4	-0.388795033	1.72E-17	-0.350126523	1.60E-13	-0.209435495	0.000194	0.045108705	0.436126	0.193806489	6.75E-05	0.143642017	0.038496	C4	-	Y	Y	-	-	-	-
ENSG00000169598	DFFB	0.588362132	1.90E-05	0.105491214	0.677658	0.191076426	0.333858	-0.477947014	0.00011	-0.382332355	0.001267	0.089630624	0.752688	C1	-	Y	-	-	Y	Y	-
ENSG00000169599	NFU1	-0.516907887	3.08E-05	-0.195993201	0.166724	-0.30481554	0.012454	0.326958437	0.016417	0.226756006	0.111027	-0.105996763	0.620472	C2	-	Y	-	Y	Y	-	-
ENSG00000169609	C15orf40	0.224211487	0.012092	0.291825986	0.002455	0.336682703	0.000306	0.073962286	0.476209	0.127288346	0.182055	0.047821755	0.794088	C4	-	-	-	Y	-	-	-
ENSG00000169612	RAMAC	0.185587896	0.07122	-0.138148195	0.322157	-0.148955296	0.245366	-0.317189571	0.001349	-0.319634665	0.001085	-0.007936547	0.978268	C5	-	-	-	-	Y	Y	-
ENSG00000169629	RGPD8	0.788424921	0.031125	0.386430896	0.453301	0.694881826	0.087016	-0.395201298	0.34524	-0.078953637	0.873047	0.311576185	NA	C1	-	Y	-	-	-	-	-
ENSG00000169660	HEXD	-0.583508237	3.61E-12	-0.106623512	0.358588	-0.021655897	0.86565	0.483653176	3.00E-08	0.576655449	1.13E-13	0.088135069	0.527708	C2	-	Y	-	-	Y	Y	-
ENSG00000169696	ASPSCR1	-0.441486808	7.58E-07	-0.120898057	0.387995	-0.167528224	0.115412	0.327109836	0.000617	0.289144803	0.000681	-0.0438505	0.839789	C4	-	Y	-	-	Y	-	-
ENSG00000169733	RFNG	0.325500395	8.06E-06	0.099173836	0.388571	0.287337142	0.000253	-0.219680081	0.003443	-0.023212624	0.806085	0.19082476	0.053589	C5	Y	Y	-	-	-	-	-
ENSG00000169758	TMEM266	-0.497675005	0.178673	0.290188469	0.463684	0.232053036	0.539686	0.794762192	0.022174	0.744979172	0.029545	-0.055064602	NA	C3	-	-	-	-	Y	Y	-
ENSG00000169783	LINGO1	-0.85610848	1.92E-12	-0.519954755	0.000203	-0.30155763	0.040093	0.342564915	0.022899	0.569476553	3.68E-05	0.221562516	0.318871	C4	-	Y	Y	Y	Y	Y	-
ENSG00000169855	ROBO1	-0.252995017	0.000811	-0.053844253	0.664564	0.054894574	0.587664	0.205570908	0.014623	0.322508719	6.31E-06	0.111488782	0.367875	C4	-	-	-	-	-	Y	-
ENSG00000169891	REPS2	-1.102868399	0.01438	-0.253309622	0.667307	-0.018456048	0.976467	0.857069591	0.072587	1.098240749	0.010515	0.239379436	NA	C3	-	Y	-	-	-	Y	-
ENSG00000169902	TPST1	0.273902085	0.000231	0.078811962	0.487746	-0.063997071	0.613124	-0.188710883	0.011407	-0.323592422	2.96E-05	-0.140097526	0.271654	C5	-	-	-	-	-	Y	-
ENSG00000169908	TM4SF1	3.453834735	1.75E-07	2.946412451	NA	2.973880398	0.000182	-0.498015136	0.299864	-0.465960345	0.352252	0.028058559	NA	C1	-	Y	-	Y	-	-	-
ENSG00000169914	OTUD3	0.334554611	0.000138	-0.075310464	0.590375	-0.010296317	0.945454	-0.403398169	1.78E-06	-0.330195606	3.13E-05	0.067605736	0.684358	C5	-	Y	-	-	Y	Y	-
ENSG00000169926	KLF13	-0.350554455	1.97E-13	-0.068752358	0.281198	-0.092947272	0.139414	0.288168963	4.93E-10	0.272239734	2.28E-08	-0.021359506	0.860801	C4	-	Y	-	-	-	-	-
ENSG00000169964	TMEM42	-0.528227475	0.005016	0.105699836	0.666433	0.002119141	0.994307	0.640635598	0.000828	0.544857291	0.010575	-0.101057042	0.79857	C4	-	Y	-	-	Y	Y	-
ENSG00000169967	MAP3K2	0.385147044	1.97E-06	0.021582036	0.898673	0.076909681	0.521295	-0.357267836	1.20E-07	-0.293588584	5.52E-07	0.058274233	0.690042	C5	-	Y	-	-	Y	-	-
ENSG00000169972	PUSL1	0.405241778	0.004157	0.172295667	0.410015	0.20730482	0.279985	-0.226085948	0.145779	-0.183288821	0.258926	0.037864129	0.926182	C5	-	Y	-	-	-	-	-
ENSG00000169981	ZNF35	0.180566171	0.129588	-0.179922929	0.180497	-0.191812173	0.167051	-0.354363635	0.000549	-0.358039045	0.001133	-0.009138718	0.977318	C5	-	-	-	-	Y	Y	-

ENSG00000169989	TIGD4	0.584188437	0.045036	0.123553242	0.780391	0.170821274	0.665549	-0.455024788	0.138059	-0.39846144	0.203728	0.05023255	NA	C5	-	Y	-	-	-	-	-
ENSG00000170006	TMEM154	-2.67771407	0.019085	-0.476583637	NA	-1.00558249	NA	2.210395025	0.117304	1.687932419	0.275192	-0.526397021	NA	C3	-	Y	-	-	-	-	-
ENSG00000170011	MYRIP	-0.414015289	0.04929	-0.245324599	0.327883	-0.293064346	0.272338	0.174427052	0.518763	0.135227681	0.667499	-0.044401327	0.943025	C4	-	Y	-	-	-	-	-
ENSG00000170017	ALCAM	0.470939438	2.44E-26	0.3624571	8.26E-09	0.226649955	7.99E-06	-0.102035145	0.035734	-0.229946234	2.29E-16	-0.13304287	0.047395	C1	-	Y	Y	-	-	-	-
ENSG00000170043	TRAPPC1	-0.357170163	4.40E-05	-0.175306282	0.126073	-0.112493787	0.368299	0.188010137	0.005017	0.260268467	0.000138	0.066546709	0.616484	C4	-	Y	-	-	-	-	-
ENSG00000170085	SIMC1	0.313964209	5.53E-06	0.033232838	0.815524	0.150384917	0.095474	-0.273951106	0.000185	-0.148926094	0.041764	0.11982559	0.349194	C5	-	Y	-	-	-	-	-
ENSG00000170100	ZNF778	0.53728154	3.06E-12	0.071220563	0.59369	0.319567154	0.000204	-0.459554447	5.88E-13	-0.203021773	0.00106	0.251115193	0.002304	C5	-	Y	-	Y	Y	-	-
ENSG00000170113	NIPA1	0.608042106	3.73E-22	0.285483185	0.001115	0.299917083	0.000127	-0.316043362	1.20E-07	-0.293232954	4.83E-08	0.017339114	0.928676	C5	-	Y	-	-	Y	-	-
ENSG00000170145	SIK2	0.538985041	1.63E-05	0.430570999	0.005256	0.471953001	8.01E-05	-0.102415025	0.522922	-0.053046228	0.711864	0.045222429	0.875773	C1	-	Y	Y	Y	-	-	-
ENSG00000170166	HOXD4	-0.321724557	0.019568	-0.037503224	0.880257	0.113842431	0.572072	0.290980419	0.062372	0.450595054	0.002065	0.15375244	0.581795	C4	-	Y	-	-	-	Y	-
ENSG00000170191	NANP	0.168260834	0.240978	-0.459040188	0.004549	-0.104585491	0.56898	-0.620734301	3.15E-06	-0.258764546	0.047278	0.357142888	0.063565	C5	-	-	Y	-	Y	-	-
ENSG00000170234	PWWP2A	0.168745729	0.047493	-0.201998341	0.028917	0.055862273	0.645599	-0.364360203	4.07E-06	-0.098202381	0.310912	0.260650335	0.011856	C5	-	-	-	-	Y	-	-
ENSG00000170236	USP50	-0.177604238	0.75786	1.358555782	7.18E-05	0.635733983	0.1271	1.543438868	3.22E-06	0.82809986	0.035638	-0.717637059	NA	C2	-	-	Y	-	Y	Y	-
ENSG00000170260	ZNF212	-0.213581243	0.014847	-0.287012372	0.002853	-0.321889283	0.000369	-0.067032618	0.559752	-0.093352706	0.381007	-0.031951768	0.887695	C4	-	-	-	Y	-	-	-
ENSG00000170271	FAXDC2	-1.354539519	1.48E-12	-1.018964295	1.86E-06	-0.610302347	0.002229	0.344484411	0.275251	0.75876845	0.002039	0.410077202	0.27862	C3	-	Y	Y	Y	-	Y	-
ENSG00000170315	UBB	-0.378517914	1.22E-12	-0.347214991	2.78E-09	-0.277676405	2.29E-06	0.037884995	0.383568	0.115810088	0.000845	0.072657985	0.143111	C4	-	Y	Y	-	-	-	-
ENSG00000170322	NFRKB	0.389843295	1.05E-09	0.18565077	0.022936	0.23288601	0.00686	-0.197568842	0.000775	-0.142506003	0.041401	0.049996645	0.729391	C5	-	Y	-	-	-	-	-
ENSG00000170325	PRDM10	0.774733103	2.57E-16	0.15303121	0.296857	0.156634015	0.277383	-0.61572303	9.73E-14	-0.603397373	2.00E-12	0.006680968	0.981547	C5	Y	Y	-	-	Y	Y	-
ENSG00000170340	B3GNT2	0.27348848	0.00145	0.034406108	0.851475	0.324598117	0.00102	-0.231964886	0.012419	0.066267223	0.495006	0.293003437	0.014086	C4	-	-	-	Y	-	-	-
ENSG00000170345	FOS	0.867851861	0.02759	0.515391007	0.270762	0.612105572	0.135166	-0.346137636	0.409415	-0.240452652	0.572569	0.097833801	NA	C1	-	Y	-	-	-	-	-
ENSG00000170364	SETMAR	-0.448177071	1.72E-06	-0.466912758	6.79E-07	-0.580254967	5.40E-10	-0.012517834	0.936179	-0.117408773	0.337043	-0.11034465	0.53385	C4	-	Y	Y	Y	-	-	-
ENSG00000170365	SMAD1	-0.248477426	0.023899	-0.156736255	0.291731	-0.349281838	0.006417	0.098310924	0.486432	-0.085825651	0.569888	-0.190020197	0.328305	C4	-	-	-	Y	-	-	-
ENSG00000170382	LRRN2	-1.564000556	8.84E-07	-0.738837563	0.016994	-0.429723376	0.206328	0.833882925	0.044156	1.149042896	0.00332	0.310861366	NA	C3	-	Y	Y	-	Y	Y	-
ENSG00000170385	SLC30A1	-0.128590007	0.174897	-0.462817053	8.99E-08	-0.383565891	1.92E-06	-0.327704611	0.000151	-0.240591376	0.003422	0.082222972	0.594591	C4	-	-	Y	Y	Y	-	-
ENSG00000170396	ZNF804A	0.145658088	0.333296	-0.583704161	0.000494	-0.381345554	0.037454	-0.723376421	8.50E-07	-0.512903174	0.000999	0.204888774	0.541217	C5	-	-	Y	Y	Y	Y	-
ENSG00000170421	KRT8	4.335421942	0.002711	3.096300394	NA	4.87408461	0.000856	-1.2242145	0.581847	0.550963762	0.555305	1.76759624	NA	C5	-	Y	-	Y	-	-	-
ENSG00000170425	ADORA2B	-1.284113548	0.013605	-0.051390576	0.947899	-0.091308252	0.906352	1.241842766	0.021204	1.208097144	0.042166	-0.038166072	NA	C3	-	Y	-	-	Y	Y	-
ENSG00000170430	MGMT	-0.316457126	0.043673	-0.22386212	0.214182	-0.000287025	0.998959	0.099146335	0.634488	0.330967311	0.050193	0.226396461	0.345122	C4	-	Y	-	-	-	-	-
ENSG00000170458	CD14	1.092453912	0.029877	-0.172922349	0.87984	-0.145474926	0.88554	-1.258566497	0.00824	-1.220258018	0.005601	0.029757773	NA	C5	-	Y	-	-	Y	Y	-
ENSG00000170471	RALGAPB	0.405997201	9.24E-09	0.208142086	0.019055	0.207794375	0.00797	-0.191459115	0.000198	-0.183822692	1.41E-05	0.002396249	0.986496	C1	-	Y	-	-	-	-	-
ENSG00000170500	LONRF2	1.235804508	6.98E-49	0.407640526	0.000112	0.383159644	7.61E-05	-0.821380425	2.70E-33	-0.83841066	6.28E-41	-0.022402886	0.910446	C5	-	Y	Y	Y	Y	Y	-
ENSG00000170509	HSD17B13	1.771107849	0.00067	1.120025567	0.154506	0.975170136	0.167892	-0.644064229	0.2001	-0.780497661	0.078584	-0.142303972	NA	C1	-	Y	-	-	-	-	-
ENSG00000170558	CDH2	0.131269984	0.031576	0.381470314	1.95E-06	0.208989747	0.00729	0.2567508	0.000318	0.092611495	0.206318	-0.169782132	0.15061	C4	-	-	Y	-	-	-	-
ENSG00000170627	GTSF1	-1.275270759	0.043102	-0.639794344	0.346803	-0.339159482	0.595304	0.639914316	0.459058	0.951001849	0.177568	0.304301988	NA	C3	-	Y	-	-	-	-	-
ENSG00000170629	DPY19L2P2	-0.129978445	0.427729	0.138100041	0.495177	0.206938282	0.20023	0.274833102	0.040881	0.35133221	0.003847	0.070992365	0.788184	C4	-	-	-	-	-	Y	-
ENSG00000170631	ZNF16	-0.351561298	0.027432	-0.116810356	0.608346	-0.573182707	0.001154	0.241697174	0.120343	-0.207170923	0.258037	-0.453778733	0.019641	C2	-	Y	-	Y	-	-	Y
ENSG00000170633	RNF34	0.369135089	5.51E-07	0.146186406	0.16194	0.149344503	0.119457	-0.216510328	0.005383	-0.205157165	0.006246	0.006122934	0.978268	C5	-	Y	-	-	-	-	-
ENSG00000170638	TRABD	0.309086377	0.000124	0.229612814	0.045684	0.190285904	0.047888	-0.072838143	0.462741	-0.103964349	0.165661	-0.03649806	0.851487	C5	-	Y	-	-	-	-	-
ENSG00000170653	ATF7	0.450878278	4.88E-06	0.178965984	0.1556	0.278592778	0.016801	-0.266287957	0.001894	-0.158160862	0.101192	0.102636995	0.515302	C5	-	Y	-	-	-	-	-
ENSG00000170667	RASA4B	0.038658987	0.767064	0.535938919	9.84E-06	0.537502754	3.39E-09	0.503901858	3.82E-06	0.513287498	1.46E-09	0.004625232	0.988432	C4	-	-	Y	Y	Y	Y	-
ENSG00000170734	POLH	0.702925101	1.71E-14	0.525138423	6.80E-07	0.611088762	3.45E-10	-0.17192619	0.033782	-0.077638889	0.375398	0.08890876	0.524522	C1	-	Y	Y	Y	-	-	-
ENSG00000170743	SYT9	-1.042714033	0.032309	0.544659908	0.248685	0.385746248	0.418476	1.594668012	0.000462	1.442753613	0.001693	-0.15576708	NA	C3	-	Y	-	-	Y	Y	-
ENSG00000170759	KIF5B	0.302776653	1.82E-09	0.120607265	0.075497	0.126209585	0.040884	-0.175741124	1.03E-05	-0.162002782	1.10E-05	0.00843912	0.943834	C5	Y	Y	-	-	-	-	-
ENSG00000170775	GPR37	0.778886699	0.04934	0.10933722	0.875123	0.464355897	0.319118	-0.663305053	0.088851	-0.300626677	0.460979	0.35721745	NA	C1	-	Y	-	-	-	-	-
ENSG00000170791	CHCHD7	-0.329256701	0.121404	-0.009124088	0.980318	-0.59098791	0.009246	0.326879632	0.112413	-0.246841694	0.307449	-0.57893019	0.021149	C2	-	-	-	Y	-	-	Y
ENSG00000170802	FOXN2	0.43749657	5.66E-07	-0.056061213	0.72234	0.205610946	0.094231	-0.487553246	6.96E-12	-0.218127819	0.009342	0.264351794	0.02502	C5	-	Y	-	-	Y	-	-
ENSG00000170832	USP32	0.393438008	1.91E-08	0.341073354	1.45E-05	0.198495689	0.008412	-0.046193887	0.549789	-0.180698313	0.000614	-0.139617561	0.071683	C1	-	Y	Y	-	-	-	-
ENSG00000170836	PPM1D	0.014412724	0.898456	-0.353839643	2.60E-05	-0.078533843	0.482365	-0.361814353	9.93E-09	-0.078928374	0.316703	0.277841609	0.001344	C5	-	-	Y	-	Y	-	-
ENSG00000170873	MTSS1	-0.466317659	0.000758	-0.254308373	0.12186	-0.011110503	0.961295	0.218823129	0.177019	0.470139259	0.000625	0.246256527	0.201741	C4	-	Y	-	-	-	Y	-
ENSG00000170876	TMEM43	0.488587482	7.12E-22	0.123269588	0.150113	0.117798835	0.085995	-0.358508913	2.47E-11	-0.355883268	2.18E-15	-0.002841456	0.987605	C5	-	Y	-	-	Y	Y	-
ENSG00000170899	GSTA4	-0.315414493	3.72E-07	-0.188747815	0.026589	-0.180882691	0.023818	0.133173017	0.094626	0.149231954	0.044436	0.010788809	0.959572	C4	-	Y	-	-	-	-	-
ENSG00000170915	PAQR8	-0.406086798	5.51E-07	-0.072414465	0.482031	0.095078341	0.262634	0.340057979	7.81E-06	0.515928182	5.33E-14	0.170136669	0.031665	C4	-	Y	-	-	Y	Y	-
ENSG00000170917	NUDT6	-0.244005592	0.171532	0.087885286	0.717994	0.172248719	0.498936	0.338420262	0.044135	0.431500644	0.028073	0.087393078	0.833312	C4	-	-	-	-	Y	Y	-
ENSG00000170919	TPT1-AS1	0.546089934	1.36E-08	0.357644205	0.00256	0.544339518	5.94E-07	-0.181847021	0.076961	0.01310574	0.927156	0.189519026	0.204351	C5	-	Y	Y	Y	-	-	-
ENSG00000170946	DNAJC24	0.383366033	2.28E-05	0.156678375	0.218215	0.12711666	0.32206	-0.220050786	0.019151	-0.241936103	0.009415	-0.026894382	0.914971	C5	-	Y	-	-	-	-	-
ENSG00000170954	ZNF415	0.054947108	0.773559	-0.266120935	0.10579	0.004729857	0.983851	-0.314757978	0.035659	-0.035871857	0.856353	0.273723726	0.142473	C5	-	-	-	-	Y	-	-
ENSG00000170983	LINC00208	-1.177003322	0.369101	1.248723949	NA	0.622354919	0.557236	2.434687717	0.004663	1.8150759	0.056922	-0.616837913	NA	C2	-	-	-	-	Y	-	-
ENSG00000171004	HS6ST2	0.162936802	0.550843	-0.302643782	0.295558	-0.442775741	0.067079	-0.458666875	0.059547	-0.591498724	0.008486	-0.13694086	0.767513	C5	-	-	-	-	-	Y	-
ENSG00000171045	TSNARE1	-0.388948409	0.000899	-0.019574931	0.931615	0.070317207	0.666036	0.376605239	0.006981	0.474032594	8.53E-05	0.092839433	0.717029	C4	-	Y	-	-	Y	Y	-
ENSG00000171055	FEZ2	-0.382730397	8.96E-08	-0.110149083	0.223745	-0.13051906	0.137499	0.279150074	7.92E-05	0.267136425	0.000282	-0.017302626	0.927111	C4	-	Y	-	-	-	-	-
ENSG00000171067	C11orf24	0.378134391	5.38E-06	0.094416572	0.520771	0.217964061	0.054475	-0.277086034	0.002997	-0.145535354	0.142061	0.126551247	0.479268	C5	-	Y	-	-	-	-	-
ENSG00000171084	FAM86JP	-1.218692541	3.90E-07	-0.013333942	0.971957	-0.108248082	0.715469	1.21052283	2.36E-06	1.124917052	1.36E-05	-0.091864706	0.852721	C2	-	Y	-	-	Y	Y	-
ENSG00000171094	ALK	0.412233268	8.17E-15	-0.299388876	0.000279	-0.156619283	0.057068	-0.704736217	3.83E-36	-0.554594515	6.81E-27	0.145661542	0.213226	C5	-	Y	-	-	Y	Y	-
ENSG00000171097	KYAT1	-0.003619998	0.992011	0.68338407	0.018298	0.441320023	0.164544	0.69361754	0.008719	0.459235796	0.118366	-0.239884627	0.62178	C4	-	-	Y	-	Y	-	-
ENSG00000171100	MTM1	0.361607689	0.078442	0.580897698	0.0036	0.508413001	0.009342	0.225576768	0.23552	0.161793911	0.410395	-0.068688184	0.845294	C1	-	-	Y	Y	-	-	-
ENSG00000171150	SOCS5	0.47528401	5.22E-12	0.020555166	0.879551	0.215117454	0.013111	-0.448649308	3.68E-14	-0.245472715	0.000123	0.197669156	0.02502	C5	-	Y	-	-	Y	-	-
ENSG00000171160	MORN4	-0.248502067	0.004477	-0.024701439	0.865102	0.074301194	0.508479	0.230372206	0.004914	0.337672347	1.22E-05	0.101689746	0.410168	C4	-	-	-	-	-	Y	-

ENSG00000171163	ZNF692	0.452607237	1.10E-09	0.122958049	0.324289	0.329719261	0.000526	-0.323070425	6.92E-05	-0.108184928	0.236819	0.20966166	0.116234	C5	-	Y	-	Y	Y	-	-
ENSG00000171169	NAIF1	-0.166488561	0.35365	0.341193576	0.045902	0.088485712	0.683215	0.514148413	0.000344	0.269976334	0.091732	-0.249757621	0.21552	C4	-	-	Y	-	Y	-	-
ENSG00000171217	CLDN20	-0.088668894	0.914281	0.387899191	0.632038	1.173261928	0.017213	0.483412328	0.486029	1.276574744	0.008243	0.787436654	NA	C4	-	-	-	Y	-	Y	-
ENSG00000171295	ZNF440	0.339007743	0.00488	-0.034581351	0.87029	0.150675813	0.371507	-0.367262902	0.000745	-0.173991466	0.184266	0.188070944	0.315115	C5	-	Y	-	-	Y	-	-
ENSG00000171298	GAA	-0.519381691	4.17E-21	-0.078720058	0.323537	-0.110786608	0.11384	0.447173559	1.48E-15	0.423502276	1.99E-14	-0.029079854	0.829147	C2	-	Y	-	-	Y	Y	-
ENSG00000171310	CHST11	-0.91941764	4.86E-12	-0.067130849	0.792492	0.132119073	0.389476	0.85901421	3.26E-08	1.065964147	2.96E-19	0.202131768	0.375519	C4	-	Y	-	-	Y	Y	-
ENSG00000171314	PGAM1	-0.211261857	3.33E-06	0.164043926	0.009051	0.029322203	0.783545	0.381727114	1.32E-17	0.255463303	8.27E-06	-0.131862076	0.177551	C4	-	-	-	-	Y	-	-
ENSG00000171365	CLCN5	0.517001808	2.30E-14	0.274966326	0.003494	0.317118768	2.45E-05	-0.23573512	0.002232	-0.185275151	0.004639	0.045289084	0.787764	C5	-	Y	-	Y	-	-	-
ENSG00000171368	TPPP	0.01996165	0.928987	0.067197162	0.796865	0.379995484	0.013101	0.053769621	0.807219	0.374348489	0.009496	0.315692641	0.13614	C4	-	-	-	Y	-	Y	-
ENSG00000171388	APLN	-1.347371461	8.24E-06	-0.538874568	0.085975	-1.093764712	0.00069	0.811965401	0.034279	0.268945737	0.622257	-0.548818473	NA	C3	-	Y	-	Y	Y	-	-
ENSG00000171408	PDE7B	-0.516056693	4.47E-06	-0.226975659	0.102523	-0.17855401	0.244719	0.295746664	0.022492	0.352322011	0.008918	0.05087705	0.869474	C4	-	Y	-	-	-	Y	-
ENSG00000171428	NAT1	-0.498658763	0.358998	-0.539664459	0.382176	-1.170981545	0.038275	-0.034741356	0.963732	-0.656761033	0.325085	-0.62763523	NA	C3	-	-	-	Y	-	-	-
ENSG00000171435	KSR2	-0.669586789	0.021324	0.122789822	0.77154	-0.022849342	0.956889	0.799792797	0.00637	0.661157092	0.020013	-0.142452571	0.785782	C4	-	Y	-	-	Y	Y	-
ENSG00000171443	ZNF524	-0.269322343	0.403735	0.166255874	0.692056	0.255697132	0.480489	0.443178493	0.108019	0.540324742	0.045008	0.09153326	0.87848	C4	-	-	-	-	-	Y	-
ENSG00000171444	MCC	-0.646107809	1.74E-24	-0.278745408	0.000376	-0.206749358	0.0103	0.373610667	1.25E-09	0.453759718	5.15E-14	0.074775072	0.554373	C4	-	Y	-	-	Y	Y	-
ENSG00000171448	ZBTB26	0.309736712	1.03E-06	-0.037562835	0.768644	0.078480258	0.445581	-0.340727081	8.78E-07	-0.21640645	0.002558	0.118963807	0.367786	C5	-	Y	-	-	Y	-	-
ENSG00000171467	ZNF318	0.563895538	1.41E-19	0.345430652	3.20E-06	0.454935258	7.72E-11	-0.212160252	4.66E-05	-0.094273319	0.105591	0.112426305	0.194309	C5	-	Y	Y	Y	-	-	-
ENSG00000171469	ZNF561	0.321780615	0.000115	-0.008138994	0.963184	0.174182176	0.106383	-0.32341023	2.94E-07	-0.133441211	0.060574	0.184721619	0.070903	C5	-	Y	-	-	Y	-	-
ENSG00000171488	LRRC8C	0.477787375	1.20E-17	0.176790904	0.028597	0.187795778	0.007256	-0.294209006	3.58E-07	-0.275385344	1.59E-07	0.013657806	0.939083	C5	-	Y	-	-	-	-	-
ENSG00000171492	LRRC8D	0.10406567	0.291722	-0.319088012	0.001897	-0.14209935	0.185188	-0.416595363	6.78E-07	-0.231801879	0.005028	0.179680949	0.165108	C5	-	-	Y	-	Y	-	-
ENSG00000171497	PPID	0.504654702	7.72E-13	0.040346434	0.764094	0.250277561	0.003525	-0.457411386	2.08E-16	-0.239700852	2.54E-05	0.212434795	0.007893	C5	-	Y	-	-	Y	-	-
ENSG00000171503	ETFDH	-0.315846913	0.018586	-0.118237542	0.543448	0.001803393	0.993336	0.204055848	0.186149	0.332566806	0.011484	0.12291378	0.601463	C4	-	Y	-	-	-	Y	-
ENSG00000171517	LPAR3	0.085862609	0.790704	-0.32500074	0.344917	0.334357811	0.231729	-0.404427348	0.148295	0.263018586	0.314824	0.661787524	0.028794	C4	-	-	-	-	-	-	Y
ENSG00000171606	ZNF274	-0.524053358	7.27E-06	-0.119950853	0.354875	0.070162272	0.623669	0.410388099	0.00065	0.608837429	1.25E-07	0.192984634	0.176456	C4	-	Y	-	-	Y	Y	-
ENSG00000171608	PIK3CD	-0.712590315	2.55E-05	-0.15842198	0.601897	0.044387303	0.880649	0.562681115	0.014967	0.771885202	8.26E-05	0.203379412	0.625239	C4	-	Y	-	-	Y	Y	-
ENSG00000171617	ENC1	-0.44788844	1.55E-14	0.029109509	0.824312	-0.105671555	0.178881	0.483538633	1.27E-13	0.356978257	4.12E-11	-0.131945706	0.223973	C4	-	Y	-	-	Y	Y	-
ENSG00000171631	P2RY6	-1.418294134	3.02E-09	-0.856637014	0.000216	-0.829713593	0.000126	0.570042884	0.066221	0.603328395	0.041585	0.029619944	0.965116	C3	-	Y	Y	Y	-	Y	-
ENSG00000171649	ZIK1	-0.654308846	1.19E-09	0.032659971	0.870596	-0.105123012	0.458618	0.693100678	2.90E-09	0.563829294	5.58E-07	-0.134988578	0.49807	C4	-	Y	-	-	Y	Y	-
ENSG00000171680	PLEKHG5	-0.422989664	2.42E-05	-0.230274271	0.057655	-0.034118984	0.822569	0.199436735	0.059671	0.40356496	5.47E-06	0.198455849	0.115674	C4	-	Y	-	-	-	Y	-
ENSG00000171681	ATF7IP	-0.779496367	1.36E-21	-0.390521673	6.55E-05	-0.21522928	0.022241	0.395505164	2.88E-07	0.578554987	4.39E-19	0.177804279	0.098285	C4	Y	Y	Y	-	Y	Y	-
ENSG00000171714	ANO5	-0.312610311	0.0103	-0.053419534	0.763469	-0.047197621	0.779664	0.265426679	0.028044	0.279883055	0.021034	0.00914672	0.975811	C4	-	Y	-	-	-	-	-
ENSG00000171724	VAT1L	-0.793740664	1.58E-24	-0.156760409	0.13322	-0.026472701	0.837479	0.643693951	1.37E-14	0.781885449	2.80E-24	0.133281154	0.312772	C2	-	Y	-	-	Y	Y	-
ENSG00000171729	TMEM51	-0.359343432	6.39E-06	-0.100653115	0.372233	-0.26578651	0.005761	0.265343765	0.000413	0.10846179	0.246655	-0.162278751	0.158463	C4	-	Y	-	-	-	-	-
ENSG00000171757	LRRC34	-0.227107063	0.14723	0.131216656	0.5009	0.093628645	0.624065	0.364729156	0.014022	0.335202561	0.022677	-0.034594115	0.921602	C4	-	-	-	-	Y	Y	-
ENSG00000171763	SPATA5L1	0.254732832	0.066055	-0.128846439	0.543148	-0.051322716	0.791821	-0.377330139	0.011135	-0.291315402	0.029317	0.080113838	0.796283	C5	-	-	-	-	Y	-	-
ENSG00000171766	GATM	-0.953978775	9.85E-05	-0.509020875	0.059647	-0.10326953	0.747081	0.453385798	0.152584	0.865175011	0.000911	0.408216413	0.260457	C4	-	Y	-	-	-	Y	-
ENSG00000171791	BCL2	0.235920137	0.000103	0.32559896	2.42E-05	0.176292947	0.035392	0.095732101	0.109418	-0.044844975	0.505474	-0.146447321	0.132933	C4	-	-	Y	-	-	-	-
ENSG00000171798	KNDC1	-0.894488691	0.007123	0.070299264	0.888388	0.156681518	0.709062	0.971994133	0.002412	1.066637811	0.000677	0.089505501	NA	C4	-	Y	-	-	Y	Y	-
ENSG00000171806	METTL18	-0.016952246	0.927229	-0.184638109	0.282582	-0.375593898	0.00698	-0.160957918	0.276885	-0.343951008	0.007196	-0.187981308	0.392863	C5	-	-	-	Y	-	Y	-
ENSG00000171817	ZNF540	1.075493478	3.00E-05	0.831486149	0.004274	0.834212362	0.00373	-0.236965245	0.419561	-0.226333712	0.443862	0.005085871	0.994554	C1	-	Y	Y	Y	-	-	-
ENSG00000171823	FBXL14	0.427629322	0.000805	0.216915123	0.354045	0.363651124	0.056457	-0.204537054	0.269582	-0.049541961	0.819685	0.149906146	0.693305	C5	-	Y	-	-	-	-	-
ENSG00000171827	ZNF570	0.388841398	7.92E-05	-0.063025556	0.705764	0.091656827	0.577244	-0.445750474	1.42E-06	-0.282457839	0.008526	0.157532039	0.387643	C5	-	Y	-	-	Y	-	-
ENSG00000171843	MLLT3	-0.388647365	0.005506	0.054218323	0.773762	0.081526821	0.598268	0.449586242	0.001135	0.485017281	0.000197	0.030387192	0.913675	C4	-	Y	-	-	Y	Y	-
ENSG00000171848	RRM2	-0.317711876	4.19E-18	-0.252541539	2.44E-08	-0.395328512	6.85E-22	0.071712714	0.109395	-0.063005907	0.13376	-0.13986986	0.009149	C4	-	Y	-	Y	-	-	-
ENSG00000171914	TLN2	-0.284748501	1.15E-07	0.055336093	0.577891	-0.030839066	0.72443	0.346810249	5.75E-10	0.268659749	9.13E-08	-0.083592808	0.422397	C4	-	-	-	-	Y	-	-
ENSG00000171943	SRGAP2C	0.534375218	0.000241	0.225598451	0.323928	0.245698167	0.206409	-0.301762373	0.06639	-0.273902066	0.076039	0.022566928	0.959572	C5	-	Y	-	-	-	-	-
ENSG00000171951	SCG2	0.350023532	1.40E-14	0.447243605	3.23E-23	0.273687301	8.05E-09	0.103707049	0.023695	-0.061690138	0.204578	-0.170707954	0.001265	C1	Y	Y	Y	-	-	-	-
ENSG00000171992	SYNPO	-0.450046134	1.50E-17	-0.179813316	0.001438	-0.116550482	0.076137	0.276666725	1.30E-08	0.348007345	6.07E-12	0.066061755	0.46646	C4	-	Y	-	-	-	Y	-
ENSG00000172000	ZNF556	-0.207905006	0.46279	-0.797646342	0.007301	-0.535025806	0.071067	-0.583177946	0.03682	-0.312285391	0.294344	0.265289277	NA	C5	-	-	Y	-	Y	-	-
ENSG00000172007	RAB33B	0.254528258	0.013201	-0.351666682	0.003588	0.032182989	0.847888	-0.599929246	7.23E-09	-0.207572891	0.060772	0.386749181	0.004334	C5	-	-	Y	-	Y	-	Y
ENSG00000172020	GAP43	-0.591835837	4.61E-21	-0.309374391	2.16E-05	-0.260757672	5.48E-05	0.288957145	6.37E-05	0.34587985	6.42E-08	0.051597588	0.705077	C4	-	Y	Y	-	-	Y	-
ENSG00000172059	KLF11	-0.991541192	4.28E-25	-0.070907611	0.624536	-0.287058879	0.004119	0.927618674	1.88E-19	0.719206377	2.90E-12	-0.213317024	0.127054	C2	Y	Y	-	-	Y	Y	-
ENSG00000172071	EIF2AK3	0.631176814	1.83E-08	0.484078156	9.20E-06	0.514178334	2.19E-06	-0.141168992	0.247232	-0.10260845	0.425006	0.032742149	0.892177	C5	-	Y	Y	Y	-	-	-
ENSG00000172086	KRCC1	0.14832241	0.150918	-0.27054197	0.015461	0.085906902	0.487026	-0.412406042	1.89E-05	-0.047789307	0.696306	0.359550803	0.001148	C5	-	-	-	-	Y	-	Y
ENSG00000172159	FRMD3	-0.451520666	0.178895	0.634133521	0.023296	0.514510093	0.108402	1.091456843	8.75E-05	0.980831909	0.00161	-0.116240501	0.843457	C2	-	-	Y	-	Y	Y	-
ENSG00000172171	TEFM	0.187289927	0.195903	-0.239389379	0.176131	-0.121631473	0.56898	-0.420178781	0.003446	-0.293993287	0.069211	0.120841279	0.706832	C5	-	-	-	-	Y	-	-
ENSG00000172175	MALT1	0.701600627	1.54E-14	0.331058021	0.00101	0.282627868	0.006469	-0.364013051	8.21E-05	-0.404206447	1.22E-05	-0.045474417	0.829128	C5	-	Y	Y	-	Y	Y	-
ENSG00000172183	ISG20	-0.058015609	0.925741	-0.558303358	0.316103	-1.154250176	0.026514	-0.494246864	0.351051	-1.080704154	0.03886	-0.591692943	NA	C5	-	-	-	Y	-	Y	-
ENSG00000172197	MBOAT1	-0.243897042	0.081001	-0.133134179	0.534466	0.055166211	0.77448	0.117790142	0.542079	0.313978562	0.022268	0.191616967	0.425996	C4	-	-	-	-	-	Y	-
ENSG00000172201	ID4	0.559602711	0.003596	0.23939011	0.421612	0.038219895	0.91727	-0.311775436	0.095576	-0.505786269	0.004182	-0.198938696	0.571938	C5	-	Y	-	-	-	Y	-
ENSG00000172216	CEBPB	-0.400492473	0.100275	-0.196813365	0.524888	0.224346661	0.397377	0.210357413	0.465482	0.639843113	0.003841	0.424004863	0.148991	C4	-	-	-	-	-	Y	-
ENSG00000172232	AZU1	-1.49889471	0.02484	-0.187872811	0.83671	0.494497481	0.462134	1.322047112	0.063489	2.009230662	0.001031	0.687144998	NA	C3	-	Y	-	-	-	Y	-
ENSG00000172292	CERS6	0.387011791	2.04E-06	0.328645022	0.00039	0.313870106	0.000213	-0.051651908	0.518614	-0.058839756	0.393893	-0.012573994	0.941335	C4	-	Y	Y	Y	-	-	-
ENSG00000172315	TP53RK	0.387336942	1.34E-05	0.079949119	0.593386	0.171041307	0.141997	-0.300637568	0.001013	-0.201877173	0.030086	0.093882471	0.603242	C5	-	Y	-	-	Y	-	-
ENSG00000172318	B3GALT1	-0.60740459	0.007282	-0.204130983	0.603341	-0.517418487	0.041913	0.410065229	0.198314	0.104370302	0.764977	-0.31136771	0.540963	C2	-	Y	-	Y	-	-	-
ENSG00000172339	ALG14	-0.431915962	0.021186	-0.430017737	0.020979	-0.571833229	0.001361	0.007291435	0.97944	-0.124997469	0.64082	-0.138220571	0.710667	C5	-	Y	Y	Y	-	-	-
ENSG00000172340	SUCLG2	-0.570639337	1.85E-06	0.043655294	0.813676	-0.007796215	0.968288	0.62120205	3.65E-07	0.577369569	2.44E-06	-0.048632065	0.853543	C2	-	Y	-	-	Y	Y	-

ENSG00000172346	CSDC2	-0.500468353	0.04804	-0.185008491	0.59149	0.136902749	0.680799	0.322468094	0.28221	0.652235655	0.011264	0.324689582	0.402715	C4	-	Y	-	-	-	Y	-
ENSG00000172350	ABCG4	-0.839619023	0.00241	-0.445799965	0.148742	-0.261385439	0.43682	0.399827628	0.199454	0.592786032	0.043445	0.187269458	0.700053	C3	-	Y	-	-	-	Y	-
ENSG00000172375	C2CD2L	0.59283524	4.15E-14	0.217737087	0.044607	0.286359133	0.004625	-0.36865095	1.01E-05	-0.292019106	0.000623	0.071559773	0.705751	C5	-	Y	-	-	Y	-	-
ENSG00000172380	GNG12	-0.204271252	0.017241	0.094326386	0.458109	0.203504989	0.078326	0.304787591	0.000335	0.422461375	3.28E-06	0.111623153	0.535179	C4	-	-	-	-	Y	Y	-
ENSG00000172382	PRSS27	-0.622332898	0.004894	-0.168591591	0.619105	-0.424487136	0.096537	0.460623818	0.025189	0.213206291	0.348008	-0.252647764	0.457415	C4	-	Y	-	-	Y	-	-
ENSG00000172461	FUT9	0.326318668	0.015317	-0.0665467	0.787059	0.125557306	0.468364	-0.38628795	0.008217	-0.186364598	0.184255	0.194676879	0.416516	C5	-	Y	-	-	Y	-	-
ENSG00000172466	ZNF24	0.274119582	0.000339	-0.092271363	0.404416	-0.002101606	0.988521	-0.359835913	7.21E-14	-0.261990514	1.67E-06	0.092732911	0.368678	C5	-	-	-	-	Y	-	-
ENSG00000172469	MANEA	0.346476452	0.001078	0.049854797	0.786994	0.099822089	0.503556	-0.289994672	9.69E-05	-0.232270476	0.002426	0.052236384	0.752303	C5	-	Y	-	-	-	-	-
ENSG00000172476	RAB40A	-0.580660028	0.037791	0.041954393	0.925894	-0.076142994	0.852126	0.628938502	0.023946	0.518759284	0.078559	-0.115318887	0.840846	C4	-	Y	-	-	Y	-	-
ENSG00000172530	BANP	0.765277357	4.45E-12	0.484358991	0.000717	0.315510685	0.018192	-0.274294175	0.037487	-0.434939136	0.000109	-0.165934345	0.450967	C5	-	Y	Y	Y	-	Y	-
ENSG00000172548	NIPAL4	2.725072373	0.001213	2.91275646	NA	1.774562047	NA	0.199088757	0.778096	-0.933374473	0.190334	-1.135860817	NA	C1	-	Y	-	-	-	-	-
ENSG00000172640	OR10AD1	-0.917850319	0.590479	1.272735816	NA	0.422952734	NA	2.200674657	0.044781	1.356505077	0.358351	-0.84468849	NA	C2	-	-	-	-	Y	-	-
ENSG00000172661	WASHC2C	0.32796041	1.64E-08	0.090309289	0.278166	0.048058872	0.610052	-0.231261476	9.21E-05	-0.265069425	1.07E-05	-0.039362132	0.773975	C5	-	Y	-	-	-	-	-
ENSG00000172663	TMEM134	-0.172958973	0.316187	0.12738061	0.47662	0.132904966	0.416154	0.30699483	0.036768	0.320979204	0.024644	0.008338755	0.979338	C4	-	-	-	-	Y	Y	-
ENSG00000172667	ZMAT3	0.77267546	1.05E-26	0.375428902	1.19E-05	0.363572521	4.61E-06	-0.390831114	9.93E-09	-0.394660209	3.66E-10	-0.009088627	0.966351	C5	-	Y	Y	Y	Y	Y	-
ENSG00000172671	ZFAND4	-0.319677285	0.048254	-0.072255417	0.747826	-0.085656178	0.705388	0.253908086	0.13998	0.248442127	0.186277	-0.010793755	0.980189	C4	-	Y	-	-	-	-	-
ENSG00000172728	FUT10	0.714198017	7.38E-05	0.428930462	0.067074	0.553962145	0.006516	-0.279194987	0.168452	-0.145483529	0.494383	0.128581211	0.725726	C5	-	Y	-	Y	-	-	-
ENSG00000172731	LRRC20	-0.365932653	0.000292	0.080957242	0.485832	0.076950221	0.535802	0.453584795	3.47E-06	0.457430755	9.72E-06	-0.001186996	0.996684	C2	-	Y	-	-	Y	Y	-
ENSG00000172732	MUS81	0.394042555	2.57E-07	-0.006828591	0.962957	0.131568807	0.125584	-0.394365038	4.18E-07	-0.247651901	0.001175	0.141265534	0.199209	C5	-	Y	-	-	Y	-	-
ENSG00000172733	PURG	0.821955342	2.72E-13	0.463359043	0.003615	0.597938566	4.29E-06	-0.351614273	0.004259	-0.209459805	0.073877	0.136786448	0.562296	C1	-	Y	Y	Y	Y	-	-
ENSG00000172748	ZNF596	-0.527505238	9.21E-06	-0.392724265	0.002615	-0.273961422	0.02797	0.140624258	0.403485	0.268275361	0.055522	0.121747429	0.59793	C4	-	Y	Y	-	-	-	-
ENSG00000172789	HOXC5	-0.660797098	0.019082	-0.418856429	0.222142	-0.261141457	0.450726	0.246642012	0.517337	0.414495903	0.209951	0.161227918	NA	C4	-	Y	-	-	-	-	-
ENSG00000172794	RAB37	-1.605505527	0.000133	-0.814625583	0.05915	-0.808032563	0.04895	0.796868432	0.14542	0.812297985	0.13304	0.009365689	NA	C3	-	Y	-	Y	-	-	-
ENSG00000172795	DCP2	0.17267853	0.043708	-0.166760789	0.130089	0.05228171	0.618103	-0.332876693	9.07E-05	-0.105852285	0.185645	0.221818117	0.038554	C5	-	-	-	-	Y	-	-
ENSG00000172817	CYP7B1	-0.257132638	0.036979	-0.363948125	0.019061	-0.286809892	0.038198	-0.100282157	0.571988	-0.015027216	0.936576	0.080297297	0.796431	C4	-	-	Y	-	-	-	-
ENSG00000172819	RARG	-0.126729153	0.306684	-0.016855763	0.928309	0.194122562	0.079453	0.116713616	0.381196	0.335378522	0.001134	0.214035212	0.123283	C4	-	-	-	-	-	Y	-
ENSG00000172828	CES3	-1.570183666	0.000221	-0.599426936	0.222142	-0.544087834	0.207925	0.98282747	0.102469	1.040247896	0.061236	0.054192192	NA	C3	-	Y	-	-	-	-	-
ENSG00000172840	PDP2	0.592018338	6.04E-11	0.055076498	0.737248	0.17923957	0.104005	-0.531538124	6.52E-12	-0.398521552	2.97E-08	0.127735288	0.317056	C5	-	Y	-	-	Y	Y	-
ENSG00000172869	DMXL1	0.359039952	0.001275	0.315070082	0.019279	0.329019929	0.00795	-0.038397696	0.733646	-0.015526664	0.894048	0.017245845	0.943025	C1	-	Y	Y	Y	-	-	-
ENSG00000172878	METAP1D	-0.303052195	0.038355	0.007802209	0.971957	-0.16323451	0.280881	0.316583895	0.031893	0.154194325	0.364862	-0.168090793	0.39874	C4	-	Y	-	-	Y	-	-
ENSG00000172888	ZNF621	0.459094957	2.69E-11	0.111625153	0.217968	0.149553645	0.05484	-0.341247226	6.39E-08	-0.295006194	1.01E-06	0.041146462	0.744989	C5	-	Y	-	-	Y	-	-
ENSG00000172889	EGFL7	-0.646209949	2.22E-07	-0.486424058	0.001062	-0.318486832	0.046935	0.166731353	0.287645	0.342935341	0.016207	0.171062789	0.494052	C4	-	Y	Y	Y	-	Y	-
ENSG00000172893	DHCR7	0.318534982	0.000978	0.36146186	0.004959	0.397258278	4.05E-05	0.049642274	0.737254	0.093637109	0.374234	0.038389547	0.887146	C4	-	Y	Y	Y	-	-	-
ENSG00000172939	OXSR1	0.307298428	1.27E-08	0.200202827	0.001385	0.13475557	0.022132	-0.100608053	0.104174	-0.15792495	0.002203	-0.062394684	0.50724	C5	-	Y	-	-	-	-	-
ENSG00000172943	PHF8	0.470730757	5.44E-12	0.269642158	0.001107	0.289291165	0.000227	-0.194380911	0.003332	-0.16692534	0.012171	0.022214715	0.90013	C5	-	Y	-	-	-	-	-
ENSG00000172954	LCLAT1	0.232397022	0.00551	-0.080391039	0.588984	0.011054919	0.942501	-0.306106466	0.000124	-0.207208284	0.003449	0.09404621	0.549988	C5	-	-	-	-	Y	-	-
ENSG00000172965	MIR4435-2HG	0.249467438	0.515942	0.934001059	0.002425	0.334674676	0.46793	0.691082237	0.01285	0.100765011	0.827863	-0.595743352	NA	C2	-	-	Y	-	Y	-	-
ENSG00000172985	SH3RF3	-1.336982273	0.146918	0.531597903	0.586737	0.118079874	0.919757	1.875604101	0.028141	1.46869498	0.120096	-0.410241463	NA	C2	-	-	-	-	Y	-	-
ENSG00000173011	TADA2B	0.200351623	0.054124	-0.021729916	0.902503	-0.135999318	0.272338	-0.215650926	0.035419	-0.32189582	0.000809	-0.111509193	0.512947	C5	-	-	-	-	-	Y	-
ENSG00000173013	CCDC96	0.641533551	0.080766	-0.147066277	0.838404	-0.191775755	0.762108	-0.782995374	0.059415	-0.819269596	0.044218	-0.041895313	NA	C5	-	-	-	-	-	Y	-
ENSG00000173039	RELA	0.401268485	2.53E-09	0.236129938	0.001789	0.179416367	0.019135	-0.158569769	0.017602	-0.20715896	0.000999	-0.054019513	0.654537	C5	-	Y	-	-	-	-	-
ENSG00000173041	ZNF680	0.102600527	0.244797	-0.249429	0.005932	-0.09592389	0.419939	-0.345655153	8.77E-06	-0.184155444	0.042233	0.156476924	0.254025	C5	-	-	-	-	Y	-	-
ENSG00000173064	HECTD4	0.503882917	1.12E-08	0.259721405	0.017166	0.348098722	0.000324	-0.237859807	4.62E-06	-0.141354689	0.005954	0.091129163	0.300891	C5	-	Y	-	Y	-	-	-
ENSG00000173068	BNC2	-1.785904259	0.000132	-1.003752234	0.04771	-0.762944479	0.117745	0.789129324	0.250113	1.037269933	0.094728	0.243557728	NA	C3	-	Y	Y	-	-	-	-
ENSG00000173114	LRRN3	-0.2461709	1.45E-07	-0.437436558	2.12E-20	-0.48237028	1.58E-24	-0.184794419	4.66E-07	-0.221761211	5.76E-10	-0.042220245	0.565651	C4	-	-	Y	Y	-	-	-
ENSG00000173137	ADCK5	-0.423828693	0.010549	-0.268203727	0.210179	-0.070938222	0.835107	0.162177085	0.470487	0.368259313	0.126282	0.200640747	0.641821	C4	-	Y	-	-	-	-	-
ENSG00000173145	NOC3L	0.506203164	2.15E-19	0.213928476	0.002975	0.224107128	0.000815	-0.286069772	2.91E-07	-0.267585296	5.13E-07	0.013150306	0.937456	C5	-	Y	-	-	-	-	-
ENSG00000173166	RAPH1	0.500759632	0.04042	0.060427887	0.88773	0.296207684	0.311737	-0.433938014	0.028087	-0.190378721	0.369925	0.238008221	0.444627	C1	-	Y	-	-	Y	-	-
ENSG00000173175	ADCY5	-0.634290907	0.001835	-0.176087141	0.531804	0.101985842	0.71424	0.464883161	0.047184	0.750811066	0.000385	0.280686387	0.389191	C4	-	Y	-	-	Y	Y	-
ENSG00000173193	PARP14	-1.010755589	0.021181	-1.008057194	0.01528	-0.421236406	0.436624	0.008363438	0.989878	0.603183459	0.285739	0.589508544	NA	C4	-	Y	Y	-	-	-	-
ENSG00000173208	ABCD2	-0.193440672	0.019725	0.108478799	0.295673	0.018065461	0.896648	0.308257211	2.34E-05	0.226277081	0.004591	-0.087455347	0.515302	C4	-	-	-	-	Y	-	-
ENSG00000173209	AHSA2P	-0.299521716	3.21E-05	-0.063189042	0.534215	0.009022965	0.946366	0.243008352	0.000389	0.323512824	6.80E-06	0.07516085	0.553214	C4	-	-	-	-	-	Y	-
ENSG00000173210	ABLIM3	-1.211664014	0.005016	-0.378216714	0.480774	0.028202699	0.966764	0.840494062	0.093338	1.254910136	0.006062	0.409649826	NA	C3	-	Y	-	-	-	Y	-
ENSG00000173214	MFSD4B	0.743159727	1.04E-11	0.430775236	0.001809	0.592804816	4.36E-07	-0.306480006	0.006902	-0.135744332	0.239728	0.165379888	0.36932	C1	-	Y	Y	Y	Y	-	-
ENSG00000173218	VANGL1	0.332435417	0.036481	0.394971216	0.0215	0.453051561	0.004929	0.068976503	0.725659	0.135396935	0.43239	0.061005504	0.852021	C1	-	Y	Y	Y	-	-	-
ENSG00000173221	GLRX	-0.735931537	0.000309	-0.286216215	0.248675	-0.16123921	0.556834	0.457669258	0.057162	0.589699703	0.011467	0.127008753	0.777201	C3	-	Y	-	-	-	Y	-
ENSG00000173227	SYT12	1.331254899	0.047503	1.078716552	0.191679	0.338093005	0.75484	-0.24527284	0.733332	-0.97835603	0.114734	-0.737777352	NA	C5	-	Y	-	-	-	-	-
ENSG00000173230	GOLGB1	0.302482553	0.002004	0.081595751	0.619795	0.16111538	0.182572	-0.214302687	0.16194	-0.126925583	0.400465	0.082144375	0.791991	C5	-	Y	-	-	-	-	-
ENSG00000173258	ZNF483	0.139648824	0.36986	-0.324742386	0.030621	-0.064127242	0.685507	-0.45910689	0.001804	-0.189180551	0.179832	0.262986982	0.136166	C5	-	-	Y	-	Y	-	-
ENSG00000173273	TNKS	0.410845338	4.95E-08	0.153008108	0.115145	0.198456446	0.020348	-0.251563354	5.74E-05	-0.197881152	0.000934	0.048416498	0.701775	C5	-	Y	-	-	-	-	-
ENSG00000173275	ZNF449	0.057822022	0.775932	-0.415944369	0.032855	0.009227124	0.972173	-0.466418401	0.0018	-0.034588256	0.855255	0.426401616	0.021099	C5	-	-	Y	-	Y	-	Y
ENSG00000173276	ZBTB21	0.34553458	0.00627	-0.304323757	0.048361	0.085918919	0.652023	-0.644353488	1.26E-14	-0.2460621	0.006506	0.393014146	0.000657	C5	-	Y	Y	-	Y	-	Y
ENSG00000173281	PPP1R3B	0.182568989	0.252842	0.200542332	0.256996	0.415145279	0.004104	0.024170329	0.906368	0.247256904	0.093795	0.217359825	0.28183	C1	-	-	-	Y	-	-	-
ENSG00000173295	FAM86B3P	-0.477624972	0.007654	-0.004866146	0.987609	0.202216621	0.274918	0.478181304	0.011599	0.694459066	3.91E-05	0.209772843	0.41006	C4	-	Y	-	-	Y	Y	-
ENSG00000173327	MAP3K11	0.307314794	2.01E-05	0.231483977	0.015819	-0.025775077	0.834464	-0.069425041	0.48387	-0.318376847	9.28E-06	-0.254435949	0.013559	C5	-	Y	-	-	-	Y	-
ENSG00000173401	GLIPR1L1	-0.686869574	0.169985	0.25107413	0.687282	-0.150198156	0.822491	0.9422759	0.042812	0.550802958	0.318505	-0.398319652	NA	C2	-	-	-	-	Y	-	-
ENSG00000173442	EHBP1L1	-0.310669615	2.04E-05	-0.022554204	0.88773	-0.00786917	0.960096	0.294796324	0.001173	0.317801201	0.00027	0.016876296	0.951422	C4	-	Y	-	-	-	Y	-

ENSG00000173451	THAP2	1.140735531	1.18E-22	0.470909646	0.001533	1.070184017	6.81E-19	-0.664359644	1.39E-15	-0.056263576	0.595763	0.602650709	8.48E-11	C1	-	Y	Y	Y	Y	-	Y
ENSG00000173457	PPP1R14B	-0.345193094	8.79E-07	-0.107135457	0.300845	-0.262986485	0.000975	0.244610624	0.000889	0.097092186	0.236833	-0.152980829	0.163271	C4	Y	Y	-	-	-	-	-
ENSG00000173486	FKBP2	-0.489136862	0.023593	-0.299891101	0.261431	-0.272075048	0.296336	0.195213655	0.479937	0.231737404	0.383939	0.030845377	0.95884	C4	-	Y	-	-	-	-	-
ENSG00000173542	MOB1B	0.320312822	2.13E-06	0.051071533	0.677658	0.038999153	0.7254	-0.262816776	4.19E-06	-0.266642343	3.12E-07	-0.009248394	0.958938	C5	-	Y	-	-	-	-	-
ENSG00000173545	ZNF622	0.129989846	0.17176	-0.333894081	0.000726	-0.215189942	0.035087	-0.457386323	1.21E-08	-0.330635915	4.27E-05	0.121440382	0.412815	C4	-	-	Y	-	Y	Y	-
ENSG00000173546	CSPG4	0.852170481	0.004941	0.76224323	0.031662	1.217543218	3.44E-05	-0.083302189	0.829287	0.380191249	0.183902	0.458369608	NA	C1	-	Y	Y	Y	-	-	-
ENSG00000173548	SNX33	0.597353799	6.26E-12	0.358951294	0.000239	0.552858134	1.08E-08	-0.231893768	0.01437	-0.029892814	0.824262	0.19646652	0.160699	C5	-	Y	Y	Y	-	-	-
ENSG00000173557	FAM166C	-2.765882065	7.09E-06	-1.242102914	0.025585	-0.482343277	0.409146	1.532577743	0.079538	2.294716333	0.001533	0.75974536	NA	C3	-	Y	Y	-	-	Y	-
ENSG00000173559	NABP1	-0.338862295	0.317088	-0.15971167	0.753436	0.254928989	0.460532	0.186383997	0.67134	0.608822775	0.037796	0.417712456	NA	C4	-	-	-	-	-	Y	-
ENSG00000173588	CEP83	0.694315442	1.22E-08	0.429279926	0.002361	0.399099166	0.003932	-0.258452275	0.031358	-0.280607472	0.017793	-0.027721493	0.928882	C5	-	Y	Y	Y	-	-	-
ENSG00000173611	SCAI	0.554250653	2.20E-06	0.364876123	0.004556	0.355030751	0.008352	-0.183647818	0.099507	-0.18458965	0.120044	-0.006412763	0.982359	C5	-	Y	Y	Y	-	-	-
ENSG00000173638	SLC19A1	0.337241495	7.41E-05	0.170703501	0.211531	0.097167298	0.443286	-0.160311985	0.112688	-0.225329618	0.005954	-0.070471033	0.727185	C5	-	Y	-	-	-	-	-
ENSG00000173641	HSPB7	-0.614011183	0.017713	0.170764603	0.59149	-0.075026078	0.840345	0.791517587	0.000965	0.553504434	0.044286	-0.243007335	0.536705	C2	-	Y	-	-	Y	Y	-
ENSG00000173681	BCLAF3	-0.3090979	0.01399	-0.163316603	0.33902	-0.138967937	0.364938	0.152053207	0.278941	0.184249292	0.134885	0.027268634	0.930139	C4	-	Y	-	-	-	-	-
ENSG00000173706	HEG1	-0.233839455	0.265584	0.13647751	0.646266	0.350660323	0.07838	0.376357084	0.091942	0.599414643	0.001299	0.216848355	0.521076	C4	-	-	-	-	-	Y	-
ENSG00000173757	STAT5B	0.491040209	1.06E-15	0.175893477	0.026996	0.221204842	0.0021	-0.308421026	1.02E-09	-0.255316693	3.10E-07	0.04780398	0.6558	C5	-	Y	-	-	Y	-	-
ENSG00000173812	EIF1	-0.394678127	2.39E-16	-0.351885319	6.73E-07	-0.357085784	5.99E-11	0.049474358	0.443055	0.052325744	0.212994	-0.002409429	0.98852	C4	-	Y	Y	Y	-	-	-
ENSG00000173818	ENDOV	-0.215473766	0.050917	0.185484594	0.202171	-0.067556793	0.672056	0.406425673	1.48E-05	0.162807677	0.099847	-0.249448201	0.06493	C4	-	-	-	-	Y	-	-
ENSG00000173821	RNF213	-0.471210659	1.19E-07	0.149050139	0.506513	0.283573804	0.144139	0.625786308	1.18E-17	0.769338382	1.25E-12	0.137116827	0.632425	C4	-	Y	-	-	Y	Y	-
ENSG00000173826	KCNH6	-0.666647294	0.000277	-0.337467097	0.093992	-0.127643485	0.544463	0.33632065	0.137765	0.553717594	0.00335	0.212908638	0.456819	C4	-	Y	-	-	-	Y	-
ENSG00000173875	ZNF791	0.205249932	0.023718	-0.136234408	0.26492	0.050413397	0.761272	-0.334820716	4.39E-06	-0.140906408	0.160738	0.189375963	0.195857	C5	-	-	-	-	Y	-	-
ENSG00000173889	PHC3	0.324833592	0.010113	0.144304211	0.355733	0.073327627	0.687524	-0.174281187	0.165388	-0.236904666	0.057204	-0.068095938	0.781334	C5	-	Y	-	-	-	-	-
ENSG00000173918	C1QTNF1	-1.01727954	4.58E-13	-0.285499413	0.105004	-0.027395657	0.901566	0.738700262	2.07E-06	1.004696691	2.65E-13	0.261498044	0.19895	C4	-	Y	-	-	Y	Y	-
ENSG00000173930	SLCO4C1	-2.204361975	0.048305	-2.104235582	NA	-0.839439446	NA	0.084503344	NA	1.38448057	0.359141	1.280625903	NA	C3	-	Y	-	-	-	-	-
ENSG00000173988	LRRC63	0.023317637	0.978056	0.595379365	0.376047	1.20160959	0.009287	0.575598096	0.373461	1.191685453	0.013174	0.605426009	NA	C2	-	-	-	Y	-	Y	-
ENSG00000173991	TCAP	0.636884606	0.034482	0.104727355	0.878389	0.184465871	0.694497	-0.526448896	0.176342	-0.437520994	0.172747	0.081988806	NA	C5	-	Y	-	-	-	-	-
ENSG00000173992	CCS	-0.248730641	0.080193	-0.092990638	0.633883	0.086516748	0.653882	0.162501545	0.332533	0.34995789	0.01868	0.182237468	0.431008	C4	-	-	-	-	-	Y	-
ENSG00000174004	NRROS	0.094278215	0.94283	-1.477492565	NA	-2.730758921	NA	-1.56671318	0.21917	-2.811503242	0.049054	-1.251780001	NA	C5	-	-	-	-	-	Y	-
ENSG00000174013	FBXO45	0.305086082	8.97E-07	0.04310457	0.719994	0.084666518	0.378992	-0.255396595	7.82E-05	-0.20585996	0.001335	0.044407559	0.787696	C5	Y	Y	-	-	-	-	-
ENSG00000174028	FAM3C2	0.243671972	0.246363	-0.259997843	0.347323	-0.393139163	0.110529	-0.497288162	0.019585	-0.622014049	0.003254	-0.130870637	0.790777	C5	-	-	-	-	Y	Y	-
ENSG00000174032	SLC25A30	0.119948971	0.492874	-0.082139235	0.740032	0.315738962	0.044867	-0.19647671	0.181502	0.210843054	0.099546	0.402107187	0.008404	C4	-	-	-	Y	-	-	Y
ENSG00000174080	CTSF	-0.364837048	1.87E-06	-0.232944274	0.006738	-0.240355836	0.003932	0.138439277	0.094765	0.139360285	0.090045	-0.00435253	0.98241	C4	-	Y	-	-	-	-	-
ENSG00000174106	LEMD3	0.322972793	4.50E-05	0.129749866	0.21368	0.051593212	0.658868	-0.187106357	0.012998	-0.256882821	0.000291	-0.075169557	0.559515	C5	-	Y	-	-	-	-	-
ENSG00000174125	TLR1	-2.913935247	0.011526	-0.012709811	NA	-0.371160445	NA	2.909784877	0.011583	2.554316143	0.05188	-0.356358013	NA	C3	-	Y	-	-	Y	-	-
ENSG00000174130	TLR6	-3.772007837	0.008105	-1.447917411	NA	-0.301532772	NA	2.37185839	NA	3.479375038	0.031898	1.12994103	NA	C3	-	Y	-	-	-	Y	-
ENSG00000174132	FAM174A	0.401547336	0.000494	0.120102855	0.531355	0.131598247	0.388375	-0.274852931	0.032349	-0.255286612	0.026346	0.013860218	0.967582	C5	-	Y	-	-	-	-	-
ENSG00000174137	FAM53A	-0.94536188	0.007772	0.063173092	0.899541	0.191356568	0.645513	1.014690438	0.003244	1.152079496	0.000667	0.130645125	NA	C3	-	Y	-	-	Y	Y	-
ENSG00000174177	CTU2	0.403163245	0.002387	0.096050073	0.648717	0.036377838	0.874666	-0.300062394	0.01771	-0.351508682	0.00564	-0.056890924	0.848238	C5	Y	Y	-	-	Y	Y	-
ENSG00000174197	MGA	0.334267251	0.000336	0.083053211	0.521807	0.143529297	0.157247	-0.244952811	0.000869	-0.17653978	0.010788	0.063211761	0.582472	C5	-	Y	-	-	-	-	-
ENSG00000174233	ADCY6	0.485263198	2.15E-25	0.176162662	0.001919	0.309133905	2.11E-08	-0.302691162	1.46E-11	-0.161335811	0.00115	0.135870189	0.049275	C5	-	Y	-	Y	Y	-	-
ENSG00000174306	ZHX3	0.451884241	1.08E-05	0.448141048	5.42E-05	0.283733128	0.018422	0.002668359	0.981614	-0.153716453	0.094728	-0.161803051	0.192227	C1	-	Y	Y	-	-	-	-
ENSG00000174327	SLC16A13	0.603523368	0.10459	0.637459221	0.146814	0.953949411	0.004445	0.040750826	0.937982	0.365184253	0.300012	0.318260389	NA	C1	-	-	-	Y	-	-	-
ENSG00000174332	GLIS1	-2.191746301	0.043251	-1.0051036	NA	-0.125383985	NA	1.209207746	NA	2.0783514	0.084821	0.877986165	NA	C3	-	Y	-	-	-	-	-
ENSG00000174353	STAG3L3	0.335931485	0.025132	0.515625671	0.001345	0.517105849	0.000554	0.186177046	0.255875	0.196209402	0.206424	0.004506592	0.991437	C1	-	Y	Y	Y	-	-	-
ENSG00000174370	C11orf45	-2.918723813	0.002313	0.424752644	0.558494	-0.161253734	0.858514	3.352085002	0.000157	2.772330214	0.006656	-0.584213587	NA	C3	-	Y	-	-	Y	Y	-
ENSG00000174403	MIR1-1HG-AS1	-1.534808509	0.003621	-0.812758995	0.118927	-0.410666267	0.410159	0.718196184	0.348786	1.138084784	0.064074	0.406885296	NA	C3	-	Y	-	-	-	-	-
ENSG00000174448	STARD6	-2.410886304	0.101753	0.734131427	NA	-0.509432699	NA	3.160204238	0.019496	1.912587181	NA	-1.243183344	NA	C2	-	-	-	-	Y	-	-
ENSG00000174460	ZCCHC12	-0.196205564	0.217879	-0.284985919	0.161359	-0.326430725	0.041494	-0.082811021	0.714004	-0.115289845	0.536363	-0.03823992	0.933407	C5	-	-	-	Y	-	-	-
ENSG00000174469	CNTNAP2	0.32642534	2.79E-05	0.390140437	1.11E-06	0.48746362	6.49E-11	0.070235172	0.327954	0.17530039	0.003396	0.099575426	0.266197	C4	Y	Y	Y	Y	-	-	-
ENSG00000174482	LINGO2	-0.291923831	0.144428	0.127691595	0.624536	-0.08884563	0.730113	0.4256804	0.01996	0.217409004	0.292829	-0.214413934	0.437474	C4	-	-	-	-	Y	-	-
ENSG00000174483	BBS1	0.406235381	0.027796	0.388024334	0.109608	0.147937218	0.587026	-0.011936322	0.965807	-0.243767875	0.23849	-0.237164331	0.494052	C1	-	Y	-	-	-	-	-
ENSG00000174485	DENND4A	0.552714882	1.31E-10	0.287632001	0.012352	0.367264099	0.000711	-0.259074825	0.001224	-0.170975866	0.048363	0.082580286	0.638566	C5	-	Y	-	Y	-	-	-
ENSG00000174498	IGDCC3	-0.9496291	0.000378	-0.340220732	0.300845	-0.306255413	0.413488	0.616401451	0.045598	0.657875474	0.054779	0.035884342	NA	C2	-	Y	-	-	Y	-	-
ENSG00000174501	ANKRD36C	0.523867698	0.010137	0.496811786	0.014074	0.560452664	0.004792	-0.020488833	0.939835	0.051160978	0.843337	0.066921892	0.860457	C1	-	Y	Y	Y	-	-	-
ENSG00000174514	MFSD4A	-0.864617576	0.001839	-0.036083867	0.944237	-0.843377176	0.008301	0.832974513	0.011807	0.036196565	0.944757	-0.801430255	NA	C4	-	Y	-	Y	Y	-	-
ENSG00000174579	MSL2	0.15045755	0.049857	-0.225442954	0.029716	-0.053647956	0.627169	-0.36915217	3.54E-06	-0.189541917	0.008058	0.174497665	0.157664	C5	-	-	-	-	Y	-	-
ENSG00000174599	TRAM1L1	-0.428985065	0.008281	-0.022374617	0.934785	-0.263152881	0.199068	0.412734471	0.01367	0.180723017	0.40132	-0.237209566	0.39344	C4	-	Y	-	-	Y	-	-
ENSG00000174607	UGT8	-0.349995891	0.034167	-0.425442773	0.022109	-0.153967096	0.447108	-0.069482726	0.747918	0.210142788	0.197192	0.27504393	0.209911	C4	-	Y	Y	-	-	-	-
ENSG00000174640	SLCO2A1	-3.532094474	0.042486	-1.053745839	NA	-0.598427693	NA	2.497177972	NA	2.947566064	NA	0.454872153	NA	C4	-	Y	-	-	-	-	-
ENSG00000174669	SLC29A2	0.396850546	0.00061	0.197076925	0.196882	0.375418966	0.002418	-0.193473859	0.065901	-0.007117144	0.958771	0.1809483	0.204806	C1	-	Y	-	Y	-	-	-
ENSG00000174680	GRIK1-AS1	4.129149383	0.002412	3.032597589	NA	2.796267151	NA	-1.09493017	0.205735	-1.31664738	0.142607	-0.23080506	NA	C1	-	Y	-	-	-	-	-
ENSG00000174718	RESF1	0.268940477	0.004288	-0.105164957	0.446733	0.123349767	0.292264	-0.368114716	4.45E-06	-0.130991542	0.120501	0.231798172	0.026695	C5	-	-	-	-	Y	-	-
ENSG00000174721	FGFBP3	-0.712167274	3.45E-05	-0.346327882	0.082942	-0.386602169	0.044663	0.372389631	0.078994	0.340102414	0.12327	-0.037646728	0.940867	C2	-	Y	-	Y	-	-	-
ENSG00000174749	FAM241A	-0.437526512	0.219346	0.209120293	0.64799	0.317138098	0.439372	0.651112177	0.068987	0.76906415	0.030583	0.112388946	NA	C4	-	-	-	-	-	Y	-
ENSG00000174791	RIN1	0.554144464	0.000793	-0.048631923	0.885134	0.148129387	0.57461	-0.596369078	0.00035	-0.390798377	0.024053	0.199860583	0.548218	C5	-	Y	-	-	Y	Y	-
ENSG00000174804	FZD4	-1.228435382	1.41E-06	-0.023120148	0.95872	-0.075330002	0.85863	1.213136735	7.10E-06	1.167181981	9.25E-05	-0.051372314	0.943834	C3	-	Y	-	-	Y	Y	-
ENSG00000174807	CD248	-0.53419193	0.000112	-0.362717086	0.019917	-0.238877249	0.142226	0.17822353	0.326848	0.310103501	0.058296	0.126637236	0.646501	C4	-	Y	Y	-	-	-	-

ENSG00000174840	PDE12	0.315761276	5.57E-06	-0.111485311	0.324289	0.029773627	0.798973	-0.420745519	4.55E-10	-0.271698716	4.62E-06	0.144027871	0.186664	C5	-	Y	-	-	Y	-	-
ENSG00000174842	GLMN	0.340901131	0.000567	-0.07447403	0.690544	-0.041412982	0.808403	-0.408895272	0.000283	-0.367622103	0.000412	0.036089952	0.907605	C5	Y	Y	-	-	Y	Y	-
ENSG00000174886	NDUFA11	-0.301081555	0.012778	-0.188871436	0.198644	-0.344726492	0.006949	0.119678467	0.269052	-0.028226714	0.838477	-0.15344068	0.271039	C4	-	Y	-	Y	-	-	-
ENSG00000174915	PTDSS2	0.362338845	5.35E-06	0.201559541	0.06259	0.225815152	0.022132	-0.15429591	0.0999	-0.121667488	0.198427	0.027153777	0.908824	C5	-	Y	-	-	-	-	-
ENSG00000174917	MICOS13	-0.302112107	0.01552	-0.108781944	0.612585	0.009534698	0.965247	0.199481548	0.126669	0.326617216	0.000612	0.121928751	0.572524	C4	-	Y	-	-	-	Y	-
ENSG00000174943	KCTD13	0.360768979	1.74E-05	0.269054042	0.00807	0.347521946	0.000444	-0.085303591	0.396254	0.001307253	0.990963	0.081333066	0.6268	C5	-	Y	-	Y	-	-	-
ENSG00000174945	AMZ1	1.615642535	0.018211	1.571816793	NA	0.435504214	NA	-0.039815938	0.962145	-1.166299354	0.095663	-1.133554112	NA	C1	-	Y	-	-	-	-	-
ENSG00000174951	FUT1	-1.771758421	0.0059	-0.710110296	0.260814	-0.879283793	0.147796	1.056773399	0.235361	0.911099131	0.328078	-0.157741713	NA	C3	-	Y	-	-	-	-	-
ENSG00000174989	FBXW8	-0.187861933	0.048142	0.11039608	0.284685	-0.004998767	0.974932	0.304606605	9.65E-05	0.197210784	0.029502	-0.11262056	0.38344	C4	-	-	-	-	Y	-	-
ENSG00000175054	ATR	0.247375458	0.018205	0.209081499	0.069878	0.390669898	6.72E-05	-0.032431219	0.776143	0.157633902	0.064752	0.184910411	0.068615	C4	-	-	-	Y	-	-	-
ENSG00000175073	VCPIP1	0.256407093	0.001344	-0.442848741	1.30E-06	0.037257798	0.781415	-0.692828052	3.90E-17	-0.204298071	0.020776	0.482990798	1.22E-06	C5	-	-	Y	-	Y	-	Y
ENSG00000175087	PDIK1L	0.12543365	0.207068	-0.204072447	0.083536	-0.143841787	0.199272	-0.322757572	9.40E-05	-0.254795211	0.001202	0.062664131	0.721353	C5	-	-	-	-	Y	-	-
ENSG00000175093	SPSB4	-0.588451904	5.92E-07	-0.408177272	0.001105	-0.405977089	0.011463	0.186728118	0.211106	0.197394241	0.276348	0.005363747	0.991193	C4	-	Y	Y	Y	-	-	-
ENSG00000175104	TRAF6	0.309049243	0.007269	-0.273632978	0.056596	0.042018112	0.821161	-0.576634149	1.66E-09	-0.252797766	0.010613	0.318475711	0.014083	C5	-	Y	-	-	Y	-	Y
ENSG00000175105	ZNF654	0.492897421	7.99E-06	0.232763174	0.129004	0.307747523	0.034707	-0.253601792	0.025137	-0.170031695	0.180619	0.077954323	0.762928	C1	-	Y	-	Y	-	-	-
ENSG00000175106	TVP23C	-0.755546609	0.011892	0.032481424	0.949349	-0.259574248	0.562652	0.794839729	0.006798	0.510865424	0.179296	-0.290655026	NA	C2	-	Y	-	-	Y	-	-
ENSG00000175115	PACS1	0.155060055	0.012341	0.47663976	5.98E-15	0.426426861	5.70E-13	0.32811646	5.25E-09	0.286133496	7.86E-08	-0.047358856	0.693276	C4	-	-	Y	Y	Y	-	-
ENSG00000175147	TMEM51-AS1	-0.362539671	0.024433	0.101356398	0.704217	0.076649191	0.722706	0.470752395	0.00727	0.453852279	0.002634	-0.021506528	0.961034	C4	-	Y	-	-	Y	Y	-
ENSG00000175155	YPEL2	-0.182101213	0.050598	0.059055471	0.667601	0.391989631	1.02E-06	0.247824866	0.007678	0.588741525	6.27E-15	0.336078987	0.000345	C4	-	-	-	Y	-	Y	Y
ENSG00000175161	CADM2	-0.129016606	0.337726	0.261672649	0.059647	0.002127637	0.992277	0.39640147	0.000106	0.145653065	0.225313	-0.256257123	0.076062	C4	-	-	-	-	Y	-	-
ENSG00000175182	FAM131A	0.598529124	1.75E-13	0.369931528	5.77E-05	0.330830883	0.000529	-0.222195824	0.001199	-0.253385624	0.000335	-0.036499818	0.828811	C5	-	Y	Y	Y	-	-	-
ENSG00000175183	CSRP2	-0.384782369	6.71E-06	-0.170366593	0.136842	-0.119331945	0.273369	0.22088869	0.020925	0.280307947	0.001205	0.053951553	0.774633	C4	-	Y	-	-	-	-	-
ENSG00000175189	INHBC	-2.006316353	0.03355	-0.168338668	NA	-1.102276351	NA	1.842654289	0.070999	0.917536617	0.52525	-0.929898653	NA	C4	-	Y	-	-	-	-	-
ENSG00000175193	PARL	-0.351427924	0.007794	-0.422709841	0.004526	-0.404958024	0.002269	-0.064527897	0.732123	-0.038497744	0.834491	0.020458665	0.954338	C4	-	Y	Y	Y	-	-	-
ENSG00000175215	CTDSP2	0.347524067	4.15E-13	0.217910674	5.80E-05	0.353014073	5.55E-11	-0.123189637	0.014498	0.02004763	0.76964	0.137879057	0.05042	C5	-	Y	-	Y	-	-	-
ENSG00000175221	MED16	-0.579785434	7.51E-08	-0.086772235	0.49874	-0.123209208	0.229328	0.500199318	1.57E-06	0.47140489	1.53E-06	-0.032951199	0.86005	C4	-	Y	-	-	Y	Y	-
ENSG00000175274	TP53I11	-0.348480547	1.26E-05	-0.47252758	7.27E-07	-0.360438731	6.67E-05	-0.117606544	0.285272	0.002949421	0.980493	0.114721147	0.505914	C4	-	Y	Y	Y	-	-	-
ENSG00000175279	CENPS	-0.633560777	0.000773	0.017821638	0.960719	-0.111374471	0.681789	0.657738909	0.001402	0.536988823	0.011323	-0.126043002	0.759011	C4	-	Y	-	-	Y	Y	-
ENSG00000175305	CCNE2	-0.11106014	0.405325	-0.345519026	0.005372	-0.423764477	0.000633	-0.227617465	0.085888	-0.298069431	0.024476	-0.075716024	0.781564	C4	-	-	Y	Y	-	-	-
ENSG00000175309	PHYKPL	-0.277959678	0.004733	0.072533125	0.636533	0.237859129	0.019348	0.356659581	0.000215	0.530411489	3.28E-10	0.168591999	0.213226	C4	-	-	-	-	Y	Y	-
ENSG00000175322	ZNF519	0.463408799	1.05E-07	0.16954781	0.231308	0.244928842	0.022622	-0.287002213	0.00279	-0.203677022	0.016964	0.078127785	0.69007	C5	-	Y	-	-	-	-	-
ENSG00000175334	BANF1	-0.308472319	1.59E-05	-0.147074722	0.104826	-0.203280217	0.013139	0.167957701	0.000276	0.12026831	0.011554	-0.053021633	0.498767	C4	Y	Y	-	-	-	-	-
ENSG00000175344	CHRNA7	-0.539241206	2.64E-07	-0.283870113	0.026996	-0.29006624	0.013968	0.262156489	0.03733	0.263682798	0.028672	-0.003542694	0.991958	C4	-	Y	-	-	-	-	-
ENSG00000175352	NRIP3	-0.502744565	0.033326	-0.219197955	0.463416	-0.194933149	0.544337	0.28863326	0.323685	0.322551817	0.293479	0.028419003	0.967582	C4	-	Y	-	-	-	-	-
ENSG00000175354	PTPN2	0.270661493	0.0082	-0.08257578	0.572054	0.035510712	0.817735	-0.346886787	0.000492	-0.22060245	0.032337	0.121039981	0.462983	C5	-	-	-	-	Y	-	-
ENSG00000175376	EIF1AD	0.408155131	1.97E-08	-0.092871205	0.473535	0.123830573	0.300819	-0.494536845	5.32E-11	-0.269938075	0.000796	0.219627569	0.098273	C5	-	Y	-	-	Y	-	-
ENSG00000175395	ZNF25	-0.229479068	0.011649	0.092342356	0.433487	0.15959951	0.14185	0.328305921	2.58E-05	0.404037565	1.12E-06	0.07027324	0.649308	C4	-	-	-	-	Y	Y	-
ENSG00000175414	ARL10	0.469283077	1.93E-08	0.328271243	0.00083	0.393923011	8.83E-06	-0.135200995	0.025484	-0.061047866	0.320812	0.068856574	0.512341	C1	-	Y	Y	Y	-	-	-
ENSG00000175482	POLD4	-0.94242811	0.000704	-0.058352364	0.896241	-0.153175999	0.732153	0.891609205	0.001844	0.80461233	0.017119	-0.093358274	0.90074	C2	-	Y	-	-	Y	Y	-
ENSG00000175536	LIPT2	-0.675135933	0.037828	-0.04848898	0.916138	-0.329575373	0.313853	0.632588288	0.056459	0.360448432	0.326858	-0.278360647	NA	C3	-	Y	-	-	-	-	-
ENSG00000175567	UCP2	-0.717887584	1.59E-13	-0.43864353	1.61E-05	-0.309230151	0.015969	0.285816896	0.006603	0.423540775	0.000294	0.132601489	0.506753	C4	Y	Y	Y	Y	-	Y	-
ENSG00000175592	FOSL1	2.040444769	4.20E-06	1.288054432	0.020888	1.411440142	0.00892	-0.746910177	0.072314	-0.614514201	0.158572	0.126864745	NA	C1	-	Y	Y	Y	-	-	-
ENSG00000175595	ERCC4	0.2165703	0.122814	-0.257795846	0.126671	0.115996342	0.46152	-0.467943542	0.000376	-0.086271649	0.54592	0.376000627	0.014083	C4	-	-	-	-	Y	-	Y
ENSG00000175611	LINC00476	-0.500479904	0.005496	-0.111315327	0.667162	-0.012151738	0.968274	0.394180406	0.050432	0.502857642	0.010379	0.102555081	0.793199	C4	-	Y	-	-	-	Y	-
ENSG00000175643	RMI2	-0.344328425	3.92E-05	-0.069230124	0.588748	-0.361630103	0.000142	0.281183712	0.002275	-0.002606563	0.98379	-0.289117903	0.018503	C4	-	Y	-	Y	-	-	-
ENSG00000175691	ZNF77	-0.213686097	0.208897	-0.432271773	0.007981	-0.385860342	0.025985	-0.212196083	0.246354	-0.157367546	0.433446	0.049367769	0.904354	C4	-	-	Y	Y	-	-	-
ENSG00000175711	B3GNTL1	-0.348159627	0.00361	0.022039867	0.919114	-0.139401739	0.412321	0.376512094	0.004071	0.223556005	0.141483	-0.158228918	0.510867	C4	-	Y	-	-	Y	-	-
ENSG00000175727	MLXIP	0.393837682	4.03E-15	0.316550226	4.32E-07	0.237025431	9.86E-05	-0.070544741	0.241201	-0.141997488	0.006292	-0.076793011	0.431428	C1	-	Y	Y	-	-	-	-
ENSG00000175764	TTLL11	0.980699438	2.07E-10	0.473100023	0.016528	0.307790925	0.149476	-0.501245341	0.001532	-0.658665627	2.62E-05	-0.162405574	0.607555	C5	-	Y	Y	-	Y	Y	-
ENSG00000175782	SLC35E3	-0.263010102	0.001357	0.065621341	0.607217	-0.125285071	0.219362	0.335032728	8.94E-05	0.152383804	0.107611	-0.188050288	0.124344	C4	-	-	-	-	Y	-	-
ENSG00000175785	PRIMA1	-0.621308271	6.58E-16	-0.366043447	3.22E-06	-0.18241251	0.060586	0.261776201	0.00074	0.453454129	9.99E-09	0.186466918	0.086779	C4	-	Y	Y	-	-	Y	-
ENSG00000175792	RUVBL1	-0.32635232	6.32E-11	-0.107166713	0.139711	-0.121176117	0.051742	0.226060532	1.19E-06	0.220077004	3.44E-07	-0.011416248	0.932298	C4	-	Y	-	-	-	-	-
ENSG00000175854	SWI5	-0.443899007	0.003475	-0.255240914	0.161279	-0.108842583	0.599734	0.195050626	0.303157	0.349830322	0.039205	0.149362492	0.592416	C4	-	Y	-	-	-	Y	-
ENSG00000175868	CALCB	-0.680135104	0.000226	-0.406669464	0.094826	-0.177696691	0.502078	0.281305887	0.287645	0.51728663	0.021403	0.230576641	0.580084	C3	-	Y	-	-	-	Y	-
ENSG00000175879	HOXD8	-0.660053076	1.08E-11	-0.302366025	0.011912	0.108723476	0.378502	0.364486703	0.00143	0.783679539	2.01E-17	0.414221591	0.000457	C4	-	Y	Y	-	Y	Y	Y
ENSG00000175894	TSPEAR	-0.687011784	4.48E-08	0.230066585	0.246307	0.407292451	0.153007	0.924035316	1.30E-10	1.109477155	1.54E-06	0.177097695	0.761549	C4	-	Y	-	-	Y	Y	-
ENSG00000175899	A2M	1.897597658	0.029302	1.353772488	NA	0.947381142	NA	-0.534224464	0.584555	-0.937321243	0.302704	-0.404239881	NA	C5	-	Y	-	-	-	-	-
ENSG00000175928	LRRN1	-0.524239608	0.000111	-0.275991715	0.118232	-0.308507994	0.041677	0.254765155	0.096332	0.230441044	0.11278	-0.030118703	0.933843	C4	-	Y	-	Y	-	-	-
ENSG00000176014	TUBB6	0.165636041	0.287307	0.315154012	0.04882	0.063187869	0.762545	0.156488895	0.349397	-0.08765265	0.634593	-0.249219771	0.231953	C4	-	-	Y	-	-	-	-
ENSG00000176018	LYSMD3	0.534344686	4.70E-06	-0.137473967	0.477979	0.293878548	0.02116	-0.665800356	7.92E-09	-0.225778218	0.033616	0.435072931	0.001465	C5	-	Y	-	-	Y	-	Y
ENSG00000176054	RPL23P2	-0.803218309	0.109586	0.315915322	0.572697	0.092998532	0.903842	1.125042591	0.013566	0.911085116	0.105386	-0.219164562	NA	C2	-	-	-	-	Y	-	-
ENSG00000176092	CRYBG2	-0.771221418	0.03851	-0.68787186	0.232296	-0.004223228	0.993662	0.094634021	0.898407	0.782196341	0.039476	0.688189589	NA	C4	-	Y	-	-	-	Y	-
ENSG00000176095	IP6K1	0.306022767	1.53E-06	0.107153292	0.151865	0.082963469	0.287762	-0.192360091	0.002226	-0.208374829	0.000757	-0.02126675	0.883951	C5	-	Y	-	-	-	-	-
ENSG00000176102	CSTF3	-0.72443792	1.67E-25	-0.145342427	0.09405	-0.295447491	0.000103	0.585773197	1.85E-21	0.443723059	6.84E-12	-0.14749437	0.089669	C4	-	Y	-	-	Y	Y	-
ENSG00000176105	YES1	0.362888042	3.45E-06	0.215594762	0.035821	0.149571273	0.148712	-0.14068689	0.107701	-0.198629468	0.013266	-0.063226226	0.714139	C5	-	Y	-	-	-	-	-
ENSG00000176124	DLEU1	-0.00756978	0.974843	0.024218941	0.941382	0.298750899	0.113533	0.038968253	0.864661	0.320355533	0.036192	0.275802733	0.225609	C4	-	-	-	-	-	Y	-
ENSG00000176142	TMEM39A	0.395795926	3.90E-07	-0.242336859	0.027469	0.067185429	0.603164	-0.631705487	2.76E-15	-0.314020531	7.01E-05	0.312383114	0.006096	C5	-	Y	-	-	Y	Y	Y

ENSG00000176148	TCP11L1	0.233002616	0.084943	0.264372536	0.083957	0.333507937	0.016499	0.038224648	0.749421	0.1151792	0.247645	0.07147128	0.672522	C4	-	-	-	Y	-	-	-
ENSG00000176170	SPHK1	3.031380826	2.65E-07	2.897683916	3.73E-07	1.954899596	0.008594	-0.126887409	0.814417	-1.062146023	0.024978	-0.941360772	NA	C1	-	Y	Y	Y	-	Y	-
ENSG00000176171	BNIP3	-0.013929557	0.845559	0.482836492	5.02E-09	0.272331683	0.001023	0.504228133	1.29E-06	0.301167687	0.002894	-0.208534741	0.363094	C4	-	-	Y	-	Y	Y	-
ENSG00000176182	MYPOP	0.922380813	1.15E-06	0.584909097	0.014233	0.674309591	0.001794	-0.330778336	0.110733	-0.233366186	0.270369	0.091731154	0.838445	C1	-	Y	Y	Y	-	-	-
ENSG00000176208	ATAD5	0.199755122	0.031478	-0.078693805	0.562191	-0.117080407	0.406682	-0.271878695	8.99E-05	-0.302371639	0.000429	-0.035643947	0.87281	C5	-	-	-	-	-	Y	-
ENSG00000176209	SMIM19	-0.402024134	0.000692	-0.023948491	0.909007	-0.109975483	0.453822	0.384829555	0.000581	0.307115089	0.003684	-0.083493398	0.667843	C4	-	Y	-	-	Y	Y	-
ENSG00000176222	ZNF404	-0.519984011	0.048476	-0.082095518	0.845284	0.436520267	0.097952	0.446134045	0.144933	0.971092836	0.000111	0.520641865	0.134615	C4	-	Y	-	-	-	Y	-
ENSG00000176261	ZBTB8OS	-0.48970032	8.18E-08	-0.205232148	0.085704	-0.241699029	0.02807	0.29128926	0.002239	0.263079392	0.005861	-0.033563834	0.887679	C4	-	Y	-	-	-	-	-
ENSG00000176273	SLC35G1	-0.328361598	0.003881	-0.08499081	0.623981	-0.032095534	0.89204	0.249624932	0.059761	0.310951777	0.043387	0.055630034	0.880169	C4	-	Y	-	-	-	Y	-
ENSG00000176293	ZNF135	-1.082202312	2.60E-10	-0.41643529	0.048348	-0.30283666	0.09809	0.672991536	0.00238	0.794262238	3.95E-05	0.116548601	0.776314	C2	-	Y	Y	-	Y	Y	-
ENSG00000176371	ZSCAN2	-0.266681832	0.001525	-0.425176472	2.75E-06	-0.363487854	0.000129	-0.152138654	0.118166	-0.082245983	0.461537	0.064520218	0.739219	C4	-	-	Y	Y	-	-	-
ENSG00000176383	B3GNT4	-0.960289349	0.005142	-0.105150646	0.818246	-0.29274112	0.433032	0.864278433	0.021349	0.683610185	0.08938	-0.185183941	NA	C3	-	Y	-	-	Y	-	-
ENSG00000176386	CDC26	-0.143932547	0.38517	-0.418773296	0.015152	-0.312861736	0.045798	-0.268467997	0.132304	-0.154180358	0.384917	0.108769728	0.736482	C4	-	-	Y	Y	-	-	-
ENSG00000176390	CRLF3	0.323321695	0.000706	0.171288882	0.145509	0.198338355	0.048609	-0.145850969	0.193442	-0.110307978	0.314418	0.029903636	0.90013	C5	-	Y	-	-	-	-	-
ENSG00000176393	RNPEP	-0.573607966	8.35E-12	-0.327059787	0.000159	-0.216593806	0.016154	0.253142583	0.001431	0.372108214	6.38E-07	0.113913904	0.285272	C4	-	Y	Y	-	-	Y	-
ENSG00000176396	EID2	0.502605435	5.21E-05	0.155487376	0.381653	0.119397235	0.47239	-0.340601619	0.004627	-0.368310518	0.000895	-0.033008376	0.910372	C5	-	Y	-	-	Y	Y	-
ENSG00000176402	GJC3	-2.847867482	0.053395	0.305496465	NA	-0.718926507	NA	3.163272819	0.018265	2.144217093	NA	-1.018001525	NA	C2	-	-	-	-	Y	-	-
ENSG00000176407	KCMF1	0.358167408	4.08E-09	0.251917148	0.000456	0.105550579	0.202295	-0.099795747	0.166934	-0.238020517	0.000147	-0.143461024	0.130855	C5	-	Y	-	-	-	-	-
ENSG00000176422	SPRYD4	-0.276519251	0.101733	0.149159737	0.473513	-0.083198114	0.725344	0.432403237	0.001639	0.208712366	0.21772	-0.228969366	0.271549	C2	-	-	-	-	Y	-	-
ENSG00000176428	VPS37D	0.047152871	0.83232	0.131198751	0.642798	0.348745765	0.045838	0.091422036	0.72501	0.316354632	0.068712	0.220452434	0.495633	C4	-	-	-	Y	-	-	-
ENSG00000176438	SYNE3	-0.289548071	0.028276	-0.257877808	0.104501	0.020508483	0.925831	0.038274072	0.849477	0.324892167	0.02242	0.281274525	0.156541	C4	-	-	-	-	-	Y	-
ENSG00000176454	LPCAT4	0.627883295	4.41E-17	0.261314484	0.001857	0.382108824	1.90E-06	-0.36011794	9.23E-08	-0.231167127	0.001113	0.123547832	0.229419	C5	-	Y	-	Y	Y	-	-
ENSG00000176463	SLCO3A1	0.355570532	0.002251	0.171358289	0.274467	0.286068076	0.032153	-0.178346067	0.13054	-0.054938193	0.702274	0.117988627	0.543441	C4	-	Y	-	-	-	-	-
ENSG00000176476	SGF29	0.098523777	0.48969	-0.240433644	0.136387	-0.30754435	0.028285	-0.332167128	0.008619	-0.391343912	0.000942	-0.064150001	0.832105	C5	-	-	-	Y	Y	Y	-
ENSG00000176533	GNG7	-0.327553509	0.000487	-0.039240023	0.802917	0.068535525	0.627897	0.294848688	0.001134	0.410949129	6.57E-06	0.110680316	0.482463	C4	-	Y	-	-	-	Y	-
ENSG00000176623	RMDN1	-0.348266882	0.00035	0.087130213	0.525812	0.022671999	0.876259	0.442043256	7.07E-07	0.38567104	3.17E-06	-0.061045301	0.705661	C2	-	Y	-	-	Y	Y	-
ENSG00000176641	RNF152	-0.364231177	4.11E-08	0.091493109	0.444895	-0.042237669	0.777679	0.46199586	3.01E-10	0.336986895	8.85E-05	-0.131381226	0.446036	C2	-	Y	-	-	Y	Y	-
ENSG00000176681	LRRC37A	0.66930132	0.086465	0.377499125	0.480774	0.913831685	0.03092	-0.285865944	0.537018	0.258998814	0.577421	0.539832465	NA	C4	-	-	-	Y	-	-	-
ENSG00000176697	BDNF	-2.363958762	0.033717	-0.587515796	NA	-1.241975355	NA	1.776491971	0.174037	1.132368457	0.467893	-0.650927398	NA	C4	-	Y	-	-	-	-	-
ENSG00000176723	ZNF843	-0.41610109	0.332641	0.288299055	0.504543	0.31235824	0.415326	0.711650416	0.05654	0.742433366	0.037626	0.027842426	NA	C3	-	-	-	-	-	Y	-
ENSG00000176731	RBIS	-0.437943249	2.02E-08	-0.082119516	0.523667	-0.276844519	0.001558	0.362771091	3.41E-06	0.176015148	0.028499	-0.192243603	0.07753	C4	-	Y	-	-	Y	-	-
ENSG00000176732	PFN4	-1.077349106	0.002188	-0.413708659	0.322032	-0.526141815	0.210237	0.671054086	0.11448	0.56675708	0.23416	-0.109534441	NA	C3	-	Y	-	-	-	-	-
ENSG00000176761	ZNF285B	1.346752209	0.004207	0.316010939	0.728152	0.876000821	0.148377	-1.021959906	0.031547	-0.456193929	0.344828	0.5609586	NA	C5	-	Y	-	-	Y	-	-
ENSG00000176771	NCKAP5	-0.489757962	0.042264	-0.380570967	0.194798	0.139784358	0.672461	0.115407466	0.714842	0.643113635	0.004331	0.522578176	0.089669	C4	-	Y	-	-	-	Y	-
ENSG00000176783	RUFY1	0.442359559	2.17E-12	0.142944394	0.129339	0.181926015	0.022751	-0.292535757	7.86E-06	-0.245521657	9.09E-05	0.041612005	0.786386	C5	-	Y	-	-	-	-	-
ENSG00000176788	BASP1	-0.209508799	0.047589	-0.497983778	3.23E-07	-0.377608186	0.000227	-0.282042271	0.0077	-0.153196819	0.193474	0.123364494	0.482463	C4	-	-	Y	Y	-	-	-
ENSG00000176809	LRRC37A3	-0.693202497	1.46E-07	0.175231712	0.357917	-0.05067957	0.795159	0.875342986	4.10E-10	0.657300266	3.38E-07	-0.222770908	0.286262	C2	-	Y	-	-	Y	Y	-
ENSG00000176845	METRNL	0.589731269	0.00113	0.135077705	0.670832	0.21304798	0.40713	-0.448605528	0.021412	-0.361759343	0.05872	0.081375654	0.861463	C5	-	Y	-	-	Y	-	-
ENSG00000176853	FAM91A1	0.461944408	1.05E-18	0.180558593	0.01562	0.201331024	0.003979	-0.274777172	2.53E-06	-0.24582466	1.63E-05	0.023745845	0.888898	C5	-	Y	-	-	-	-	-
ENSG00000176884	GRIN1	-0.306452169	7.01E-05	-0.235690249	0.00338	-0.199246902	0.019764	0.077303824	0.437712	0.121998408	0.197343	0.039330961	0.824799	C4	-	Y	-	-	-	-	-
ENSG00000176887	SOX11	-0.205113079	0.000128	-0.135803064	0.126073	-0.379727567	1.34E-14	0.075917205	0.314352	-0.159717021	0.000444	-0.241130356	0.00137	C4	-	-	-	Y	-	-	-
ENSG00000176894	PXMP2	-0.585937348	6.53E-07	-0.298836578	0.038174	-0.218585093	0.108957	0.293542863	0.013815	0.382167861	0.000339	0.083436506	0.69007	C4	-	Y	-	-	-	Y	-
ENSG00000176907	TCIM	0.089340817	0.954004	0.131959727	NA	1.54910981	0.036648	0.052829427	0.96984	1.475282166	0.044556	1.431720687	NA	C2	-	-	-	Y	-	Y	-
ENSG00000176909	MAMSTR	-0.768828693	0.001658	-0.477681932	0.111537	-0.535938196	0.040498	0.298436001	0.364706	0.24748961	0.436646	-0.055382224	0.937456	C3	-	Y	-	Y	-	-	-
ENSG00000176912	TYMSOS	-0.738541683	0.039133	0.023134636	0.962058	-0.314845962	0.427904	0.768925074	0.03565	0.438265449	0.339061	-0.335756649	NA	C3	-	Y	-	-	Y	-	-
ENSG00000176915	ANKLE2	0.379326456	7.70E-13	0.068958841	0.389414	0.030414896	0.704677	-0.303947892	1.28E-08	-0.334260548	6.75E-12	-0.035851087	0.755306	C5	-	Y	-	-	Y	Y	-
ENSG00000176946	THAP4	0.325792667	3.55E-06	0.017036622	0.903779	0.031702336	0.840301	-0.302424464	4.57E-05	-0.27948828	0.002087	0.017301137	0.949104	C5	-	Y	-	-	Y	-	-
ENSG00000176974	SHMT1	-0.433436645	3.12E-06	0.067156046	0.631983	0.103923538	0.458493	0.50699635	1.27E-08	0.551968257	2.05E-08	0.039533648	0.868324	C4	-	Y	-	-	Y	Y	-
ENSG00000176986	SEC24C	0.326673566	2.12E-05	0.173851317	0.048277	0.183502073	0.037719	-0.146359397	0.025262	-0.128718065	0.066417	0.012249778	0.943834	C5	-	Y	-	-	-	-	-
ENSG00000177000	MTHFR	0.426983411	6.43E-09	0.224273126	0.013429	0.411765562	9.64E-08	-0.196194764	0.012499	-0.000514323	0.995691	0.190289425	0.057066	C5	-	Y	-	Y	-	-	-
ENSG00000177042	TMEM80	-0.345569745	0.00733	0.155905583	0.334458	0.083983775	0.642913	0.507997803	7.19E-05	0.44427584	0.000887	-0.069150734	0.799504	C4	-	Y	-	-	Y	Y	-
ENSG00000177051	FBXO46	0.60198392	2.50E-11	0.092977534	0.490676	0.207253571	0.056211	-0.502721272	1.97E-08	-0.380063474	3.12E-05	0.117153376	0.463182	C5	-	Y	-	-	Y	Y	-
ENSG00000177058	SLC38A9	0.48326338	6.37E-09	0.04849085	0.75575	0.169682206	0.122393	-0.428406825	7.49E-07	-0.298985055	0.000574	0.124102318	0.435604	C5	-	Y	-	-	Y	-	-
ENSG00000177098	SCN4B	-0.97743855	0.000272	-0.431526106	0.135103	-0.212644301	0.504735	0.552968775	0.046476	0.780137321	0.002735	0.221707338	0.571056	C4	-	Y	-	-	Y	Y	-
ENSG00000177105	RHOG	0.345717731	0.01134	0.233466486	0.15072	0.267937359	0.06767	-0.105699155	0.528864	-0.062968371	0.720075	0.037410747	0.9075	C5	-	Y	-	-	-	-	-
ENSG00000177106	EPS8L2	-0.862129613	0.219198	0.590877751	0.36128	0.073062749	0.932036	1.462306803	0.015294	0.949100876	0.170524	-0.514128231	NA	C2	-	-	-	-	Y	-	-
ENSG00000177125	ZBTB34	0.53929089	2.84E-12	-0.051580221	0.772213	0.201928155	0.034989	-0.583860363	3.26E-14	-0.323252372	6.13E-09	0.255731897	0.022614	C5	-	Y	-	-	Y	Y	-
ENSG00000177156	TALDO1	-0.346670476	4.12E-07	-0.127720416	0.14986	-0.093676623	0.32436	0.225495056	0.000257	0.267749149	1.84E-05	0.036870053	0.787034	C4	-	Y	-	-	-	-	-
ENSG00000177189	RPS6KA3	0.508537024	3.94E-09	0.204914832	0.063576	0.164580316	0.116498	-0.297186933	2.34E-05	-0.329719086	3.51E-07	-0.037866647	0.809023	C5	-	Y	-	-	-	Y	-
ENSG00000177192	PUS1	0.369142373	9.40E-05	0.038484801	0.841524	-0.043994154	0.787126	-0.323870042	0.001412	-0.398323148	1.92E-05	-0.079730627	0.716838	C5	-	Y	-	-	Y	Y	-
ENSG00000177272	KCNA3	0.54153053	0.003634	0.463708234	0.023412	0.245265643	0.331267	-0.07163669	0.77069	-0.281848619	0.188916	-0.215402073	0.506753	C1	-	Y	Y	-	-	-	-
ENSG00000177283	FZD8	-1.607309844	0.003794	-0.095926345	0.893863	-0.496410513	0.412286	1.516066355	0.010508	1.123553425	0.108944	-0.396965319	NA	C3	-	Y	-	-	Y	-	-
ENSG00000177303	CASKIN2	0.473714765	2.11E-09	0.177764652	0.089565	0.255943048	0.008175	-0.289489475	0.000316	-0.203164305	0.017306	0.08099438	0.610211	C5	-	Y	-	-	-	-	-
ENSG00000177335	C8orf31	-0.369664674	0.012853	0.099034539	0.654694	0.063639391	0.778577	0.475747167	0.002308	0.448118279	0.005184	-0.033100378	0.936471	C4	-	Y	-	-	Y	Y	-
ENSG00000177337	DLGAP1-AS1	-1.246936002	0.000741	0.219089084	0.638289	0.278329428	0.484974	1.47390436	5.64E-05	1.539329984	1.41E-05	0.062887345	NA	C3	-	Y	-	-	Y	Y	-
ENSG00000177352	CCDC71	-0.329328194	0.007242	-0.090564553	0.603161	-0.136661752	0.346875	0.24582993	0.018875	0.208204711	0.042617	-0.043096416	0.833312	C4	-	Y	-	-	-	-	-
ENSG00000177383	MAGEF1	-0.278506775	3.03E-07	-0.141342457	0.032024	-0.301062661	6.41E-08	0.14363572	0.005742	-0.007736094	0.916788	-0.156704584	0.008435	C4	-	-	-	Y	-	-	-

ENSG00000177409	SAMD9L	-4.277413985	0.004078	-1.615519579	NA	-0.857552139	NA	2.670860369	NA	3.429635538	NA	0.75362736	NA	C3	-	Y	-	-	-	-	-
ENSG00000177426	TGIF1	0.237678713	0.130216	-0.199360425	0.351429	0.260101695	0.16247	-0.430618134	0.007475	0.037272144	0.868043	0.462405327	0.029061	C5	-	-	-	-	Y	-	Y
ENSG00000177469	CAVIN1	1.529217202	2.70E-16	1.242940087	1.04E-10	1.143860988	1.17E-09	-0.279915462	0.165276	-0.370485571	0.04792	-0.095909844	0.796802	C1	-	Y	Y	Y	-	Y	-
ENSG00000177483	RBM44	1.551473752	4.30E-08	0.351969522	0.529987	0.398844426	0.408227	-1.198197273	6.78E-07	-1.140256607	7.22E-07	0.051460961	NA	C5	-	Y	-	-	Y	Y	-
ENSG00000177485	ZBTB33	0.354917118	5.75E-06	-0.141752159	0.219994	0.089815526	0.445796	-0.490111642	1.86E-13	-0.250545514	0.000204	0.234334884	0.018507	C5	-	Y	-	-	Y	-	-
ENSG00000177508	IRX3	-0.509809058	0.003659	-0.116669255	0.627683	0.057142105	0.810228	0.399726587	0.036592	0.581916972	0.000687	0.176773803	0.511632	C4	-	Y	-	-	Y	Y	-
ENSG00000177519	RPRM	-3.635890766	0.02653	-0.952086017	NA	-0.821218302	NA	2.70522136	NA	2.833071089	NA	0.130340927	NA	C3	-	Y	-	-	-	-	-
ENSG00000177548	RABEP2	0.571260867	1.07E-05	0.039566208	0.889599	0.025886169	0.918021	-0.525733494	0.000238	-0.530560964	4.54E-05	-0.01024227	0.98177	C5	Y	Y	-	-	Y	Y	-
ENSG00000177556	ATOX1	-0.451266182	0.001413	-0.37992393	0.011588	-0.232329615	0.124132	0.077900289	0.679217	0.233927432	0.115215	0.150446519	0.492498	C4	-	Y	Y	-	-	-	-
ENSG00000177565	TBL1XR1	-0.232958073	1.63E-05	-0.240594913	0.000619	-0.325157618	3.05E-09	-0.001031756	0.98958	-0.077609141	0.135198	-0.081914401	0.390501	C4	-	-	-	Y	-	-	-
ENSG00000177595	PIDD1	-0.170496923	0.068802	0.251151819	0.002033	0.146275028	0.103486	0.428204835	1.76E-08	0.331630929	2.68E-05	-0.102051152	0.349512	C4	-	-	-	-	Y	Y	-
ENSG00000177602	HASPIN	0.200661441	0.104835	-0.220626776	0.133568	-0.073139144	0.718076	-0.414882515	0.000558	-0.259344961	0.069972	0.15054167	0.550522	C5	-	-	-	-	Y	-	-
ENSG00000177606	JUN	0.071410396	0.559997	0.095578093	0.577756	0.45648302	0.000307	0.031211864	0.837376	0.399525033	0.000261	0.364426837	0.023349	C4	-	-	-	Y	-	Y	Y
ENSG00000177628	GBA	-0.733051829	1.88E-19	-0.302301993	0.003322	-0.397107818	3.27E-06	0.437028717	6.84E-07	0.350860795	1.43E-05	-0.091354509	0.561947	C2	-	Y	Y	Y	Y	Y	-
ENSG00000177674	AGTRAP	-0.427696725	0.015409	-0.043779716	0.87984	0.114200961	0.6048	0.390516795	0.0178	0.556976456	0.000153	0.161108926	0.50724	C4	-	Y	-	-	Y	Y	-
ENSG00000177685	CRACR2B	-0.499799379	0.102039	0.425936343	0.145098	0.52125706	0.097803	0.932645466	0.000401	1.035178891	0.000365	0.097255567	0.874624	C2	-	-	-	-	Y	Y	-
ENSG00000177692	DNAJC28	0.241737836	0.384702	-0.633604516	0.05917	-0.12590793	0.71675	-0.868422158	0.003134	-0.353064749	0.198442	0.51059119	NA	C5	-	-	-	-	Y	-	-
ENSG00000177700	POLR2L	-0.307962969	0.007931	-0.283576163	0.070866	-0.091161183	0.603298	0.030629356	0.842703	0.231783282	0.021898	0.195751176	0.271039	C4	-	Y	-	-	-	-	-
ENSG00000177707	NECTIN3	0.448610119	0.002772	0.309722711	0.115217	0.372474058	0.026219	-0.133399318	0.459082	-0.061967899	0.739009	0.066351956	0.846825	C1	-	Y	-	Y	-	-	-
ENSG00000177822	TENM3-AS1	0.185007322	0.505668	0.442387488	0.115421	0.570542711	0.025253	0.263545418	0.318882	0.399911289	0.097581	0.131020139	0.785782	C1	-	-	-	Y	-	-	-
ENSG00000177842	ZNF620	0.516635037	2.46E-05	-0.015799231	0.950782	0.177333654	0.289371	-0.525673846	1.58E-07	-0.325184953	0.001701	0.195357699	0.235768	C5	-	Y	-	-	Y	Y	-
ENSG00000177854	TMEM187	-0.76432721	0.020571	-0.02544257	0.959039	0.312646211	0.289273	0.74453193	0.028269	1.091778909	0.000194	0.340779775	0.379545	C3	-	Y	-	-	Y	Y	-
ENSG00000177868	SVBP	-0.457218118	0.002258	-0.041546227	0.883592	-0.207287144	0.209569	0.422159072	0.008996	0.265113449	0.064937	-0.162331027	0.526583	C2	-	Y	-	-	Y	-	-
ENSG00000177873	ZNF619	0.211606006	0.198425	-0.242597191	0.254471	0.109975069	0.639345	-0.447735061	0.004983	-0.086985708	0.670673	0.355392637	0.140447	C4	-	-	-	-	Y	-	-
ENSG00000177875	CCDC184	0.351386802	0.05216	0.297713481	0.196882	0.393825556	0.038979	-0.046100653	0.830817	0.057117813	0.764127	0.098144085	0.752745	C1	-	-	-	Y	-	-	-
ENSG00000177888	ZBTB41	0.889973251	3.69E-16	0.149185759	0.412851	0.614500322	3.36E-07	-0.736498626	1.14E-23	-0.26116307	0.000106	0.469607027	2.37E-07	C1	-	Y	-	Y	Y	-	Y
ENSG00000177943	MAMDC4	-0.556677006	3.32E-09	-0.310081176	0.004053	-0.103181651	0.397478	0.252754201	0.030582	0.468288407	4.19E-06	0.210021066	0.165857	C4	-	Y	Y	-	-	Y	-
ENSG00000177984	LCN15	-2.212793705	0.004418	-1.157435108	NA	-1.234804719	0.100945	1.060382894	0.353689	0.992723397	0.373185	-0.072953771	NA	C3	-	Y	-	-	-	-	-
ENSG00000177989	ODF3B	-0.40083059	0.003492	-0.415916664	0.011716	-0.199989128	0.247681	-0.008368862	0.970251	0.216031464	0.170631	0.218795649	0.366858	C4	-	Y	Y	-	-	-	-
ENSG00000177990	DPY19L2	-0.207713759	0.108875	0.17698372	0.248657	0.217504988	0.152504	0.391119487	0.000485	0.440289206	0.000209	0.043483213	0.883781	C4	-	-	-	-	Y	Y	-
ENSG00000178026	LRRC75B	0.131135778	0.406367	0.010112511	0.968802	0.311317012	0.032983	-0.114236515	0.513094	0.194942418	0.200629	0.304047284	0.097949	C4	-	-	-	Y	-	-	-
ENSG00000178031	ADAMTSL1	0.577710841	0.588357	0.611069187	NA	1.825006735	0.02527	0.039050529	0.974155	1.26313524	0.104536	1.213693224	NA	C4	-	-	-	Y	-	-	-
ENSG00000178038	ALS2CL	-1.089236438	0.004866	-0.428155384	0.369441	-0.426226094	0.339374	0.667839001	0.113865	0.677972648	0.102261	0.006242924	NA	C3	-	Y	-	-	-	-	-
ENSG00000178057	NDUFAF3	-0.312617917	0.003013	-0.089411373	0.525471	-0.070889954	0.639345	0.229969127	0.017584	0.256650169	0.011296	0.021503234	0.930556	C4	-	Y	-	-	-	-	-
ENSG00000178074	C2orf69	0.278279322	0.003764	-0.196011906	0.138694	0.067314818	0.684161	-0.467678546	2.64E-07	-0.196688031	0.061523	0.266152125	0.077504	C5	-	-	-	-	Y	-	-
ENSG00000178075	GRAMD1C	-0.976519925	0.000523	-0.818566094	0.046464	-0.01416635	0.979435	0.167302073	0.757661	0.97639104	0.004127	0.802271785	NA	C4	-	Y	Y	-	-	Y	-
ENSG00000178081	ULK4P3	-1.691257098	0.040577	0.011231107	0.992057	-1.350649132	0.119103	1.706200255	0.051881	0.352588768	0.828059	-1.356926844	NA	C3	-	Y	-	-	-	-	-
ENSG00000178082	TWF1P1	0.650495642	0.001189	0.313064951	0.246226	0.394789184	0.086735	-0.331323419	0.122767	-0.241358082	0.262895	0.085135156	0.847112	C1	-	Y	-	-	-	-	-
ENSG00000178104	PDE4DIP	0.367461454	1.40E-05	0.250571904	0.014729	0.373449856	8.01E-05	-0.110516598	0.265525	0.020771706	0.868835	0.125778215	0.388553	C1	-	Y	-	Y	-	-	-
ENSG00000178105	DDX10	0.718384578	1.63E-22	0.410968483	4.27E-06	0.273789789	0.002454	-0.300988488	7.79E-06	-0.430022273	1.65E-11	-0.134400376	0.218223	C5	-	Y	Y	-	Y	Y	-
ENSG00000178171	AMER3	-0.424088374	0.000977	-0.265736432	0.084674	-0.135232638	0.384335	0.165211804	0.336646	0.303559348	0.032238	0.133451481	0.583569	C4	-	Y	-	-	-	Y	-
ENSG00000178177	LCORL	0.46457217	6.37E-10	0.098258756	0.402936	0.095964864	0.365906	-0.360117188	6.77E-08	-0.354321773	1.22E-08	0.00060735	0.997522	C5	-	Y	-	-	Y	Y	-
ENSG00000178184	PARD6G	-0.446575097	0.000908	-0.234056538	0.148962	-0.268780998	0.071781	0.219487568	0.145995	0.192675869	0.204466	-0.031669909	0.927911	C4	-	Y	-	-	-	-	-
ENSG00000178187	ZNF454	0.625573871	0.012536	0.252180696	0.468643	0.600384764	0.020777	-0.367084601	0.161578	-0.010955439	0.971905	0.351015815	0.311688	C5	-	Y	-	Y	-	-	-
ENSG00000178188	SH2B1	0.344737279	5.94E-13	0.125294366	0.061825	0.13008545	0.060305	-0.213098983	1.56E-05	-0.200210491	0.000108	0.007593864	0.962837	C5	-	Y	-	-	-	-	-
ENSG00000178217	SH2D4B	2.670949685	0.005261	0.267889324	NA	-0.262749901	NA	-2.408934169	0.012442	-2.92341982	0.005945	-0.531431448	NA	C5	-	Y	-	-	Y	Y	-
ENSG00000178222	RNF212	-0.564239104	2.49E-05	-0.230820189	0.265969	-0.134433385	0.443727	0.339699438	0.063771	0.444521714	0.002645	0.099210533	0.777201	C4	-	Y	-	-	-	Y	-
ENSG00000178338	ZNF354B	0.529391381	2.77E-07	0.200123319	0.119477	0.327587759	0.003758	-0.322951832	0.002528	-0.187217345	0.100593	0.130353798	0.463249	C5	-	Y	-	Y	Y	-	-
ENSG00000178343	SHISA3	-1.160036947	0.016211	-0.501239624	0.412449	-0.538987256	0.326386	0.6625389	0.266233	0.635666243	0.272352	-0.034485486	NA	C3	-	Y	-	-	-	-	-
ENSG00000178385	PLEKHM3	0.352980863	8.80E-05	0.130521419	0.310061	0.065740905	0.619887	-0.216549838	0.012699	-0.272900466	0.000577	-0.061477348	0.714139	C5	-	Y	-	-	-	-	-
ENSG00000178409	BEND3	-0.643558822	2.54E-08	-0.511660187	0.000662	-0.542850291	1.82E-06	0.13823642	0.453164	0.115272071	0.45034	-0.028081189	0.943467	C4	-	Y	Y	Y	-	-	-
ENSG00000178425	NT5DC1	-0.629054075	5.22E-10	-0.122658811	0.376813	-0.13880122	0.254418	0.51323222	3.30E-06	0.504839311	1.98E-06	-0.013332921	0.9626	C2	-	Y	-	-	Y	Y	-
ENSG00000178445	GLDC	-0.608387258	1.65E-07	-0.389035709	0.003046	-0.15610403	0.284566	0.226027389	0.046022	0.466915637	2.15E-06	0.235391318	0.074964	C4	-	Y	Y	-	-	Y	-
ENSG00000178502	KLHL11	0.354193072	0.023278	-0.077764737	0.791234	0.181752004	0.34321	-0.427027487	0.00656	-0.158791452	0.287754	0.263589856	0.258449	C5	-	Y	-	-	Y	-	-
ENSG00000178537	SLC25A20	-0.324633551	0.002675	-0.183564388	0.141117	-0.106095722	0.402445	0.147775933	0.244494	0.233032741	0.03545	0.080474978	0.672522	C4	-	Y	-	-	-	-	-
ENSG00000178623	GPR35	0.907582194	0.009037	0.44207687	0.350185	0.708698258	0.059492	-0.458997857	0.238046	-0.184124772	0.661825	0.269467292	NA	C5	-	Y	-	-	-	-	-
ENSG00000178662	CSRNP3	0.725218533	1.66E-18	0.537528706	6.79E-07	0.480384728	5.22E-06	-0.181246557	0.064147	-0.22990747	0.010496	-0.055178328	0.88523	C1	-	Y	Y	Y	-	-	-
ENSG00000178665	ZNF713	0.444023991	0.000544	0.023863775	0.92564	0.089292344	0.670194	-0.414942952	7.11E-05	-0.340856939	0.001799	0.068516278	0.792104	C5	-	Y	-	-	Y	Y	-
ENSG00000178695	KCTD12	-1.027964791	1.06E-87	-0.337398997	4.12E-07	-0.437900802	2.23E-08	0.697027199	1.09E-33	0.604816261	8.54E-19	-0.098191875	0.496841	C2	-	Y	Y	Y	Y	Y	-
ENSG00000178700	DHFR2	-0.219355711	0.06318	-0.037286142	0.867222	0.140599677	0.285922	0.189046886	0.203089	0.374656564	0.000577	0.181273522	0.355391	C4	-	-	-	-	-	Y	-
ENSG00000178718	RPP25	-0.404491699	1.47E-07	-0.220180399	0.04235	-0.296383446	0.001281	0.190627028	0.041442	0.122979897	0.189436	-0.073286303	0.693689	C4	Y	Y	-	-	-	-	-
ENSG00000178752	ERFE	-0.98936307	0.013122	-0.669460741	0.101429	-0.573444454	0.124809	0.321104505	0.575139	0.429914416	0.396366	0.099445315	NA	C3	-	Y	-	-	-	-	-
ENSG00000178761	FAM219B	0.358154749	0.000639	0.140654389	0.340052	0.139650002	0.00238	-0.211178221	0.074578	-0.003707425	0.979181	0.201951445	0.206083	C5	-	Y	-	Y	-	-	-
ENSG00000178764	ZHX2	-0.992739364	0.004921	-1.046051072	0.007643	-0.715125039	0.077661	-0.047706802	0.94162	0.292672074	0.581444	0.335163542	NA	C3	-	Y	Y	-	-	-	-
ENSG00000178776	C5orf46	-1.233494531	0.112653	0.490654962	0.551813	0.363539398	0.654259	1.732140462	0.017725	1.610272765	0.029923	-0.123970465	NA	C2	-	-	-	-	Y	Y	-
ENSG00000178802	MPI	-0.214263249	0.027005	0.182727113	0.070878	0.223276772	0.029537	0.403358828	8.36E-07	0.452315743	1.70E-07	0.043687291	0.813799	C4	-	-	-	-	Y	Y	-
ENSG00000178803	ADORA2A-AS1	0.942272375	0.003532	0.782502	0.057569	0.968202317	0.006893	-0.152404237	0.714002	0.040773723	0.92495	0.187655822	NA	C1	-	Y	-	Y	-	-	-

ENSG00000178821	TMEM52	-0.920431035	0.006142	-0.449226251	0.274948	-0.172643868	0.703842	0.478906846	0.248992	0.76292214	0.039181	0.279394441	NA	C4	-	Y	-	-	-	Y	-
ENSG00000178878	APOLD1	-0.245013247	0.128773	0.251429987	0.194786	0.329294694	0.038155	0.502868405	0.000798	0.58943503	1.50E-05	0.08139302	0.787963	C4	-	-	-	Y	Y	Y	-
ENSG00000178904	DPY19L3	-0.309250545	1.94E-06	-0.106336933	0.271459	-0.150138117	0.098161	0.209485155	0.000281	0.173746844	0.006427	-0.041017137	0.788353	C4	-	Y	-	-	-	-	-
ENSG00000178917	ZNF852	0.829340052	5.45E-06	0.288212738	0.264553	0.683903632	0.000517	-0.536842367	0.000118	-0.130659158	0.420901	0.40065408	0.028034	C1	-	Y	-	Y	Y	-	Y
ENSG00000178947	SMIM10L2A	-0.996542083	1.72E-05	-0.297179066	0.288463	-0.114024466	0.720115	0.706036168	0.00851	0.897530424	0.000514	0.186157968	0.674608	C2	-	Y	-	-	Y	Y	-
ENSG00000178965	ERICH3	-1.178367866	2.88E-11	-0.559653875	0.000731	-0.737708996	2.76E-06	0.624670453	0.003772	0.455164373	0.047392	-0.175086964	0.592783	C3	-	Y	Y	Y	Y	Y	-
ENSG00000178971	CTC1	0.269808152	0.00091	0.279645714	0.003529	0.331143907	4.80E-05	0.016157145	0.900663	0.075880757	0.432898	0.054379787	0.767513	C5	Y	-	-	Y	-	-	-
ENSG00000179008	C14orf39	-1.366217914	1.46E-15	-0.389554135	0.027636	-0.562043796	0.000392	0.981697481	8.98E-07	0.818740082	5.85E-05	-0.169235188	0.593928	C3	-	Y	Y	Y	Y	Y	-
ENSG00000179021	C3orf38	0.275486543	0.003265	-0.212480655	0.098671	0.034063064	0.83678	-0.48149771	2.28E-06	-0.2268167	0.033115	0.250210135	0.123583	C5	-	-	-	-	Y	-	-
ENSG00000179029	TMEM107	-0.305242128	0.020814	-0.087506158	0.667307	0.120036004	0.501721	0.22481983	0.15357	0.440137987	0.00169	0.209581139	0.356344	C4	-	Y	-	-	-	Y	-
ENSG00000179041	RRS1	-0.03227967	0.736666	-0.353860137	9.61E-05	-0.396532558	3.36E-07	-0.314402216	2.56E-06	-0.3494099	1.33E-09	-0.040165449	0.807195	C5	-	-	Y	Y	Y	Y	-
ENSG00000179044	EXOC3L1	-0.738624608	0.006748	-0.238199151	0.547343	-0.18403997	0.570155	0.506994422	0.165828	0.569110151	0.068455	0.056593452	NA	C3	-	Y	-	-	-	-	-
ENSG00000179051	RCC2	0.213835785	1.73E-09	-0.081294523	0.151055	-0.142313287	0.008355	-0.288509199	1.82E-17	-0.341618096	2.91E-21	-0.058099701	0.495473	C5	Y	-	-	-	-	Y	-
ENSG00000179082	C9orf106	-2.610478476	0.162042	1.03910692	NA	0.397735099	NA	3.666048491	0.0195	3.025426135	0.08559	-0.637782841	NA	C2	-	-	-	-	Y	-	-
ENSG00000179094	PER1	0.295472748	0.002466	0.191729614	0.136648	0.352087101	0.001119	-0.097135017	0.393599	0.071247592	0.53974	0.163131907	0.282702	C1	-	-	-	Y	-	-	-
ENSG00000179104	TMTC2	-0.376829094	6.89E-06	-0.043213656	0.769606	-0.171912953	0.08414	0.339899535	4.60E-05	0.219212109	0.009764	-0.125876433	0.356926	C4	-	Y	-	-	Y	-	-
ENSG00000179119	SPTY2D1	0.235034329	0.009128	-0.168128451	0.151308	0.044171383	0.736288	-0.39681961	4.09E-06	-0.176380827	0.039316	0.215272037	0.072162	C5	-	-	-	-	Y	-	-
ENSG00000179134	SAMD4B	0.454118479	2.19E-20	0.240172947	4.13E-05	0.196751734	0.000207	-0.207549943	2.01E-05	-0.242695711	1.39E-08	-0.040429375	0.667459	C5	-	Y	-	-	-	-	-
ENSG00000179141	MTUS2-AS1	1.487275338	0.026123	0.02707042	NA	-0.173148074	NA	-1.437628366	0.050079	-1.640707541	0.026438	-0.192304762	NA	C5	-	Y	-	-	-	Y	-
ENSG00000179163	FUCA1	-0.228601192	0.130673	-0.145468308	0.489291	0.141289976	0.459721	0.089155899	0.627282	0.384765168	0.00525	0.289830301	0.15291	C4	Y	-	-	-	-	Y	-
ENSG00000179222	MAGED1	-0.31014637	3.26E-15	-0.141389043	0.002831	-0.182089486	1.45E-05	0.175369314	1.46E-06	0.142846643	3.57E-05	-0.037906705	0.571864	C4	-	Y	-	-	-	-	-
ENSG00000179240	GVQW3	0.518188916	9.96E-08	0.351855689	0.007002	0.587239981	5.54E-08	-0.160997187	0.119731	0.082708961	0.414526	0.238474053	0.09517	C5	-	Y	Y	Y	-	-	-
ENSG00000179284	DAND5	-1.033257023	3.80E-07	-0.634339491	0.004293	-0.36666187	0.093617	0.406762379	0.143844	0.681084912	0.004183	0.269937298	0.474909	C4	-	Y	Y	-	-	Y	-
ENSG00000179299	NSUN7	-0.657128148	0.000572	-0.140071499	0.604998	0.2193898	0.31809	0.523713887	0.002376	0.891992002	6.66E-09	0.363568648	0.060457	C4	-	Y	-	-	Y	Y	-
ENSG00000179331	RAB39A	0.536694082	2.84E-05	0.258433112	0.115374	0.401942105	0.004666	-0.272466877	0.002534	-0.120293357	0.233054	0.146811579	0.284608	C1	-	Y	-	Y	-	-	-
ENSG00000179335	CLK3	0.321990641	1.99E-08	0.020637813	0.857678	0.065468403	0.456453	-0.294883184	9.77E-08	-0.242023939	1.14E-05	0.04761924	0.701775	C5	-	Y	-	-	-	-	-
ENSG00000179348	GATA2	-0.162831733	0.000212	0.152933929	0.002253	-0.031992167	0.662092	0.322395503	1.26E-22	0.145732009	0.000147	-0.182014831	9.06E-05	C4	-	-	-	-	Y	-	-
ENSG00000179361	ARID3B	0.471481719	3.25E-08	0.119033036	0.348519	0.240159524	0.030602	-0.346024615	1.81E-06	-0.217085063	0.006998	0.123801564	0.362186	C5	-	Y	-	-	Y	-	-
ENSG00000179387	ELMOD2	-0.244986115	2.20E-06	-0.035910678	0.70052	0.058502506	0.429733	0.215626594	2.21E-05	0.318232219	1.61E-12	0.097289793	0.216666	C4	-	-	-	-	-	Y	-
ENSG00000179388	EGR3	2.590919643	4.31E-05	1.285444935	NA	0.096188762	NA	-1.299241034	0.016526	-2.483283836	9.68E-05	-1.187904129	NA	C5	-	Y	-	-	Y	Y	-
ENSG00000179403	VWA1	-0.611096736	0.00094	-0.285305987	0.211656	-0.158973277	0.532186	0.333001125	0.125799	0.467056781	0.025697	0.128984637	0.739574	C4	-	Y	-	-	-	Y	-
ENSG00000179406	LINC00174	-0.53479259	6.37E-06	0.006409103	0.9771	0.182072684	0.169688	0.548016568	9.84E-06	0.73152865	1.83E-10	0.178627952	0.311929	C2	-	Y	-	-	Y	Y	-
ENSG00000179431	FJX1	-0.49524198	0.043552	-0.262528842	0.418722	-0.386192146	0.143119	0.239761552	0.395143	0.124216179	0.67954	-0.121752605	0.79857	C4	-	Y	-	-	-	-	-
ENSG00000179454	KLHL28	0.364053315	0.002149	-0.237214554	0.109949	0.105266796	0.455412	-0.594929202	1.77E-07	-0.24454951	0.022267	0.345391592	0.007885	C5	-	Y	-	-	Y	-	Y
ENSG00000179455	MKRN3	0.368830404	0.014071	0.275080526	0.149051	0.296966448	0.14561	-0.088112268	0.622828	-0.057591693	0.787081	0.024268943	0.956575	C5	-	Y	-	-	-	-	-
ENSG00000179523	EIF3J-DT	-0.314189028	0.006972	0.011275929	0.962007	-0.002840721	0.988521	0.332245026	0.012241	0.326149633	0.007689	-0.011258724	0.974616	C4	-	Y	-	-	Y	Y	-
ENSG00000179532	DNHD1	-0.158877196	0.31785	0.268913103	0.08571	0.061535303	0.754269	0.434229535	0.001043	0.23503688	0.103414	-0.20398945	0.280913	C4	-	-	-	-	Y	-	-
ENSG00000179588	ZFPM1	-0.113623058	0.29713	0.317938037	0.000797	0.183349998	0.074742	0.438126737	5.75E-08	0.311832865	0.000194	-0.131486084	0.266197	C4	-	-	Y	-	Y	Y	-
ENSG00000179598	PLD6	-0.635250428	9.32E-08	-0.282372587	0.046468	-0.257172957	0.056583	0.358914908	0.007576	0.392779872	0.002606	0.028030268	0.933535	C4	-	Y	-	-	Y	Y	-
ENSG00000179603	GRM8	0.366696628	0.00116	-0.910819456	5.32E-11	-1.129016272	6.76E-10	-1.271039238	1.82E-24	-1.481752075	1.08E-20	-0.211771544	0.598842	C5	-	Y	Y	Y	Y	Y	-
ENSG00000179604	CDC42EP4	-0.186998382	0.021901	0.095384635	0.355875	0.196003512	0.036951	0.288970058	0.00018	0.397583445	4.87E-07	0.10325569	0.46346	C4	-	-	-	-	-	Y	-
ENSG00000179630	LACC1	-0.879102698	0.001883	-0.032715472	0.944514	-0.307310137	0.359011	0.852927064	0.00454	0.586313581	0.076917	-0.27196389	NA	C3	-	Y	-	-	Y	-	-
ENSG00000179698	WDR97	-0.656610361	0.048347	0.143848914	0.746325	-0.077843644	0.870718	0.807373218	0.00986	0.594225125	0.090889	-0.218640961	NA	C2	-	Y	-	-	Y	-	-
ENSG00000179761	PIPOX	-0.931497911	0.011853	-0.109323244	0.82058	-0.501787604	0.242469	0.82848128	0.037154	0.444712658	0.398238	-0.389720728	NA	C3	-	Y	-	-	Y	-	-
ENSG00000179820	MYADM	0.6994542	1.20E-27	0.218152079	0.019544	0.464798821	9.16E-10	-0.475302735	1.41E-12	-0.219959953	0.000901	0.249719236	0.009219	C5	-	Y	-	Y	Y	-	-
ENSG00000179832	MROH1	0.109889377	0.309624	0.261034084	0.023006	0.335171781	0.00042	0.157022558	0.120183	0.239916509	0.004901	0.077640218	0.644581	C4	-	-	-	Y	-	-	-
ENSG00000179841	AKAP5	1.114096372	7.70E-34	0.296805954	0.026996	0.235551186	0.126645	-0.810807501	1.26E-18	-0.863430895	2.40E-17	-0.058190536	0.845838	C5	-	Y	-	-	Y	Y	-
ENSG00000179889	PDXDC1	0.484642673	1.36E-18	0.261679032	0.000249	0.210242108	0.002312	-0.216063378	5.00E-05	-0.259564871	3.51E-07	-0.048822519	0.675496	C1	-	Y	-	-	-	-	-
ENSG00000179912	R3HDM2	-0.072567794	0.412983	0.225535557	0.013348	0.30676869	6.47E-05	0.304729332	1.49E-05	0.394041426	3.73E-10	0.084049987	0.500085	C4	-	-	-	Y	Y	Y	-
ENSG00000179915	NRXN1	-0.755943	7.25E-12	-0.205361932	0.195968	-0.06918858	0.751822	0.556547794	1.12E-06	0.701834101	7.28E-08	0.138091899	0.60677	C4	-	Y	-	-	Y	Y	-
ENSG00000179922	ZNF784	-0.606936063	0.000107	-0.327805899	0.088038	-0.66157505	0.000758	0.285366939	0.131673	-0.039895516	0.893317	-0.329806941	0.273604	C4	-	Y	-	Y	-	-	-
ENSG00000179941	BBS10	0.340809932	1.42E-05	-0.014553159	0.924137	0.058849835	0.656369	-0.348894066	4.86E-07	-0.267616394	0.000505	0.076149922	0.617535	C5	-	Y	-	-	Y	-	-
ENSG00000179978	NAIPP2	0.41211162	0.133205	0.107050272	0.81676	0.505326702	0.042694	-0.299057304	0.289475	0.107415418	0.676714	0.401340299	0.203306	C1	-	-	-	Y	-	-	-
ENSG00000180008	SOCS4	0.399738342	3.19E-10	-0.079894268	0.437187	0.057457523	0.560986	-0.47309493	5.50E-16	-0.327726319	4.96E-09	0.140072623	0.148255	C5	-	Y	-	-	Y	Y	-
ENSG00000180071	ANKRD18A	-1.421440977	0.000717	-0.558435956	0.280113	-0.605552361	0.205456	0.86783537	0.114594	0.830223328	0.132902	-0.042726104	NA	C4	-	Y	-	-	-	-	-
ENSG00000180155	LYNX1	-0.807651382	1.45E-08	-0.258569212	0.113367	-0.133914315	0.432323	0.555744934	0.00013	0.688558676	3.76E-07	0.128097953	0.564944	C2	-	Y	-	-	Y	Y	-
ENSG00000180182	MED14	0.31933081	1.52E-07	0.246055951	0.003209	0.257613677	0.000682	-0.066790817	0.344966	-0.046849647	0.487698	0.014424663	0.938937	C1	-	Y	-	-	-	-	-
ENSG00000180229	HERC2P3	0.589525237	1.28E-16	0.296483567	0.00083	0.52431417	8.69E-11	-0.286370185	2.54E-05	-0.050839478	0.568082	0.230420944	0.016147	C5	-	Y	-	Y	-	-	-
ENSG00000180287	PLD5	-0.415132619	5.09E-06	0.344116555	0.002098	0.220754113	0.031671	0.765853497	8.28E-15	0.650560984	5.58E-13	-0.120603461	0.501149	C4	-	Y	Y	-	Y	Y	-
ENSG00000180316	PNPLA1	2.725161235	0.024888	1.282325173	NA	1.670621459	NA	-1.423233263	0.256187	-1.035148525	0.366946	0.384977926	NA	C5	-	Y	-	-	-	-	-
ENSG00000180340	FZD2	-0.288099441	0.013229	-0.410212018	0.001261	-0.058817836	0.743778	-0.115283398	0.35278	0.24436671	0.025729	0.354196682	0.006994	C4	-	-	Y	-	-	-	Y
ENSG00000180357	ZNF609	0.507777599	9.57E-16	0.288500366	3.25E-05	0.266471292	0.000227	-0.212836598	7.25E-06	-0.226963454	5.72E-06	-0.019325124	0.883781	C1	-	Y	-	-	-	-	-
ENSG00000180370	PAK2	0.393339076	2.22E-15	0.148978758	0.035269	0.156951276	0.013945	-0.237924909	1.55E-08	-0.22167172	1.03E-08	0.010819235	0.937494	C5	-	Y	-	-	-	-	-
ENSG00000180376	CCDC66	0.432604176	1.66E-05	-0.012364681	0.95269	0.143401955	0.356443	-0.438841035	3.94E-06	-0.274709521	0.011899	0.158540489	0.401822	C5	-	Y	-	-	Y	-	-
ENSG00000180385	EMC3-AS1	0.220899663	0.23532	0.631839087	0.000263	0.216129997	0.322335	0.41734708	0.009975	0.01033299	0.964584	-0.412662387	0.049474	C4	-	-	Y	-	Y	-	Y
ENSG00000180425	C11orf71	-0.378296195	0.036462	-0.0749084	0.785122	-0.252254953	0.288448	0.310004164	0.104132	0.141008278	0.571615	-0.174465676	0.610617	C4	-	Y	-	-	-	-	-
ENSG00000180447	GAS1	-0.966003287	0.001508	-0.281461872	0.47586	-0.601514728	0.064027	0.694121934	0.046521	0.379238345	0.343649	-0.3186491	NA	C2	-	Y	-	-	Y	-	-

ENSG00000180448	ARHGAP45	-0.149054413	0.549699	-0.2711275	0.361753	-0.472406369	0.048374	-0.116131499	0.672078	-0.308216106	0.169553	-0.19758801	0.621168	C4	-	-	-	Y	-	-	-
ENSG00000180479	ZNF571	0.643177804	0.001413	-0.02088601	0.961488	0.396981172	0.078697	-0.657543031	0.000367	-0.232724212	0.214574	0.419590892	0.088061	C1	-	Y	-	-	Y	-	-
ENSG00000180537	RNF182	-0.308352322	0.000807	-0.082201932	0.535505	-0.076207957	0.549194	0.23257701	0.01103	0.246948551	0.006706	0.008947716	0.97007	C4	-	Y	-	-	-	-	-
ENSG00000180592	SKIDA1	-0.265578501	0.103171	0.040870548	0.877978	-0.056054318	0.81146	0.312761021	0.048954	0.224127335	0.180546	-0.093657534	0.755132	C4	-	-	-	-	Y	-	-
ENSG00000180616	SSTR2	-0.292531981	0.020475	-0.256117669	0.103179	-0.518252329	9.42E-05	0.044242753	0.830007	-0.210844527	0.194	-0.260577911	0.254683	C4	-	-	-	Y	-	-	-
ENSG00000180626	ZNF594	0.24321942	0.025056	-0.075706075	0.666433	0.263859263	0.029506	-0.313360314	0.000205	0.035106079	0.752365	0.342878065	0.001271	C4	-	-	-	-	Y	-	Y
ENSG00000180667	YOD1	0.430180162	7.44E-08	0.13883369	0.238895	0.016508572	0.911076	-0.284794872	0.001522	-0.399030269	1.12E-06	-0.11938176	0.454595	C5	-	Y	-	-	-	Y	-
ENSG00000180747	SMG1P3	0.548793965	0.037762	0.211251034	0.54517	0.384545076	0.207598	-0.331312237	0.189912	-0.149591416	0.622509	0.176141857	0.682272	C1	-	Y	-	-	-	-	-
ENSG00000180769	WDFY3-AS2	-0.945029728	0.009958	-0.410074971	0.297542	-0.237451838	0.609346	0.543849118	0.232652	0.722302433	0.113477	0.173688674	NA	C4	-	Y	-	-	-	-	-
ENSG00000180773	SLC36A4	0.447210653	4.16E-11	0.075495465	0.534223	0.062235924	0.542231	-0.364524319	6.46E-10	-0.370420425	9.47E-14	-0.011248712	0.949752	C5	-	Y	-	-	Y	Y	-
ENSG00000180787	ZFP3	0.287211312	0.003475	-0.278018509	0.016103	0.072422209	0.642866	-0.55868694	1.83E-10	-0.200540655	0.050141	0.353213805	0.004545	C5	-	-	-	-	Y	-	Y
ENSG00000180801	ARSJ	0.073881604	0.539218	0.24228886	0.032024	0.39129828	0.000143	0.174774487	0.102479	0.332220056	0.000748	0.15178191	0.332752	C4	-	-	-	Y	-	Y	-
ENSG00000180855	ZNF443	0.399765931	0.019781	-0.425454648	0.054501	-0.075325231	0.802779	-0.818954204	4.65E-08	-0.460795934	0.004641	0.352843825	0.19895	C5	-	Y	-	-	Y	Y	-
ENSG00000180875	GREM2	-1.264879071	9.19E-14	-0.769532088	4.95E-05	-0.885053974	2.68E-06	0.502724949	0.032687	0.394037662	0.123602	-0.115011991	0.824799	C3	Y	Y	Y	Y	Y	-	-
ENSG00000180881	CAPS2	-0.139077847	0.457267	0.152356322	0.445744	0.288982173	0.097144	0.297672336	0.047184	0.442651907	0.002825	0.139628404	0.563457	C4	-	-	-	-	-	Y	-
ENSG00000180884	ZNF792	0.358134088	0.000112	0.467628371	1.04E-06	0.332425776	0.00104	0.115950797	0.27104	-0.010815918	0.93768	-0.132129618	0.365135	C4	-	Y	Y	Y	-	-	-
ENSG00000180891	CUEDC1	-0.302327328	0.005913	-0.203628927	0.056841	-0.255749365	0.009081	0.105742992	0.420303	0.061482207	0.66283	-0.049093079	0.800737	C4	-	Y	-	-	-	-	-
ENSG00000180902	D2HGDH	-0.993521587	1.33E-13	-0.208153046	0.146019	-0.051024701	0.791428	0.792791717	4.23E-08	0.957317949	6.30E-11	0.159587336	0.472263	C3	-	Y	-	-	Y	Y	-
ENSG00000180914	OXTR	0.411594565	0.027267	0.01979143	0.96137	0.056289931	0.861901	-0.385821848	0.087056	-0.340578564	0.120912	0.040812826	0.947856	C5	-	Y	-	-	-	-	-
ENSG00000180917	CMTR2	0.456470414	4.41E-09	-0.064670261	0.647753	0.15739202	0.139933	-0.51472195	1.72E-11	-0.284781383	0.000224	0.224821926	0.062925	C5	-	Y	-	-	Y	-	-
ENSG00000180921	FAM83H	1.176275848	0.014868	0.365432678	0.659051	-0.29410827	0.768671	-0.806417163	0.08427	-1.45259619	0.005811	-0.656071131	NA	C5	-	Y	-	-	-	Y	-
ENSG00000180957	PITPNB	0.305027725	4.96E-08	-0.01501691	0.903778	-0.034656473	0.69887	-0.313105318	9.84E-11	-0.325168725	8.04E-17	-0.017362691	0.901678	C5	-	Y	-	-	Y	Y	-
ENSG00000180979	LRRC57	0.35921602	0.00042	-0.008396883	0.965147	0.004080545	0.981445	-0.361231636	0.000493	-0.340281322	0.001017	0.015409149	0.955616	C5	-	Y	-	-	Y	Y	-
ENSG00000181004	BBS12	0.4929163	0.021595	-0.276874464	0.414693	0.32003628	0.234145	-0.762294193	0.000472	-0.15821941	0.517235	0.598728934	0.044765	C5	-	Y	-	-	Y	-	Y
ENSG00000181007	ZFP82	0.1716626	0.192948	-0.22583311	0.060663	0.173633805	0.118197	-0.3908174	0.001039	0.016540699	0.919355	0.402472803	0.000223	C5	-	-	-	-	Y	-	Y
ENSG00000181019	NQO1	-0.498190856	1.61E-07	-0.556856006	3.57E-07	-0.728515199	4.54E-10	-0.052136903	0.721598	-0.215679244	0.090469	-0.168957973	0.430724	C4	Y	Y	Y	Y	-	-	-
ENSG00000181027	FKRP	-0.372962269	1.91E-06	-0.235102353	0.015845	-0.253897668	0.005083	0.144269447	0.11964	0.13401073	0.143999	-0.015825442	0.944754	C4	-	Y	-	-	-	-	-
ENSG00000181031	RPH3AL	-0.847303963	0.001242	-0.103159442	0.777158	-0.152320431	0.658342	0.750834855	0.00769	0.709824977	0.018251	-0.046471105	0.943834	C4	-	Y	-	-	Y	Y	-
ENSG00000181038	METTL23	-0.303284026	0.001454	0.011052212	0.949442	0.035765892	0.811018	0.321019625	0.000277	0.354094223	8.85E-05	0.027756965	0.899085	C4	-	Y	-	-	Y	Y	-
ENSG00000181072	CHRM2	0.011033979	0.935166	-0.38234648	0.000129	-0.594058977	2.83E-11	-0.386522378	5.08E-08	-0.590591784	2.35E-18	-0.209375457	0.053244	C4	-	-	Y	Y	Y	Y	-
ENSG00000181090	EHMT1	0.362168667	7.41E-14	0.102631902	0.14637	0.139172632	0.028226	-0.252980904	2.98E-07	-0.208390944	2.75E-05	0.039409224	0.731303	C5	-	Y	-	-	-	-	-
ENSG00000181215	C4orf50	-1.108627052	8.24E-06	0.193277902	0.558217	0.278970059	0.346575	1.307475172	1.63E-10	1.401178578	2.24E-11	0.088530811	0.841305	C3	-	Y	-	-	Y	Y	-
ENSG00000181240	SLC25A41	2.011289064	0.039248	1.203771901	NA	0.99107047	NA	-0.793185898	0.407046	-1.003034412	0.304909	-0.209834336	NA	C5	-	Y	-	-	-	-	-
ENSG00000181284	TMEM102	0.745016662	0.001217	0.610009648	0.019673	0.819099374	0.000125	-0.128472499	0.656226	0.089166695	0.74203	0.21287268	0.55087	C1	-	Y	Y	Y	-	-	-
ENSG00000181315	ZNF322	0.484292473	2.56E-07	0.01086432	0.960604	0.207878741	0.046354	-0.467200421	1.44E-06	-0.261943234	0.001242	0.200186734	0.152125	C5	-	Y	-	-	Y	-	-
ENSG00000181333	HEPHL1	-1.389657705	0.037036	-1.070508666	0.212273	-0.898986921	0.211851	0.32294221	0.759634	0.506426316	0.555457	0.173920799	NA	C4	-	Y	-	-	-	-	-
ENSG00000181381	DDX60L	-1.681007667	5.41E-06	-0.973604456	0.022322	-0.823430681	0.037683	0.71445886	0.208684	0.871008388	0.092067	0.150715725	NA	C3	-	Y	Y	Y	-	-	-
ENSG00000181472	ZBTB2	-0.063375705	0.557189	-0.196119568	0.068092	-0.602503738	2.24E-11	-0.126436765	0.2405	-0.524478991	1.19E-08	-0.40335245	0.00037	C5	-	-	-	Y	-	Y	Y
ENSG00000181513	ACBD4	-0.516787034	0.034754	0.080166825	0.852164	0.145026439	0.645457	0.603117997	0.017546	0.676548873	0.001939	0.068849241	0.904354	C4	-	Y	-	-	Y	Y	-
ENSG00000181523	SGSH	0.314590195	2.76E-05	0.165092112	0.139789	0.218633124	0.008369	-0.142746421	0.15023	-0.081286673	0.366041	0.056527801	0.760514	C5	-	Y	-	-	-	-	-
ENSG00000181544	FANCB	0.484284759	0.000176	0.119437865	0.592943	0.106700114	0.609246	-0.357933054	0.009452	-0.36269958	0.00703	-0.009963686	0.981123	C5	-	Y	-	-	Y	Y	-
ENSG00000181588	MEX3D	0.190401715	0.029449	-0.28025006	0.015494	-0.098012267	0.374727	-0.463771029	3.51E-07	-0.273736474	0.000296	0.185205846	0.196321	C5	-	-	-	-	Y	-	-
ENSG00000181619	GPR135	-0.15771389	0.446591	0.109636145	0.71008	0.190759996	0.351392	0.273388998	0.207961	0.36345627	0.039521	0.083587894	0.840063	C4	-	-	-	-	-	Y	-
ENSG00000181625	SLX1B	1.155860336	0.024664	0.322438785	0.707046	0.447656576	0.602485	-0.827037305	0.119606	-0.694626617	0.256877	0.129428134	NA	C5	-	Y	-	-	-	-	-
ENSG00000181649	PHLDA2	3.727017363	0.011603	2.723338454	NA	2.725722797	NA	-0.998762859	0.387017	-0.994438837	0.40187	0.002532056	NA	C5	-	Y	-	-	-	-	-
ENSG00000181652	ATG9B	-0.596118269	7.73E-06	0.175913049	0.317921	-0.129220647	0.460668	0.778913037	1.19E-08	0.481686214	0.000717	-0.302020872	0.097949	C4	-	Y	-	-	Y	Y	-
ENSG00000181790	ADGRB1	-0.381628563	0.000131	-0.186153298	0.183857	-0.153896127	0.217432	0.201570885	0.027589	0.242846635	0.001453	0.035973804	0.860105	C4	-	Y	-	-	-	-	-
ENSG00000181798	LINC00471	-0.260657503	0.657245	0.678135259	0.16089	0.536824038	0.267028	0.944591901	0.027537	0.811923223	0.066015	-0.140501109	NA	C2	-	-	-	-	Y	-	-
ENSG00000181826	RELL1	-0.613977542	0.004982	-0.665695517	0.005564	-0.704298828	0.001379	-0.043854577	0.909093	-0.07541508	0.832979	-0.036392522	0.957248	C4	-	Y	Y	Y	-	-	-
ENSG00000181827	RFX7	0.428293149	1.55E-11	0.341052014	3.14E-06	0.156417429	0.060937	-0.081109257	0.13819	-0.257143744	2.08E-07	-0.181577245	0.008987	C1	-	Y	Y	-	-	-	-
ENSG00000181852	RNF41	0.365458166	2.29E-10	0.209374056	0.005418	0.242033485	0.000539	-0.149396858	0.016223	-0.108564624	0.092416	0.03548161	0.803913	C5	Y	Y	-	-	-	-	-
ENSG00000181873	IBA57	-0.56717796	3.36E-07	-0.169282361	0.288595	-0.572131374	1.96E-06	0.403889218	0.001565	0.00963438	0.959169	-0.39962496	0.014264	C4	-	Y	-	Y	Y	-	Y
ENSG00000181904	C5orf24	0.343345018	1.25E-09	0.069502845	0.425769	0.115556778	0.122588	-0.267372883	2.98E-10	-0.213139189	1.48E-06	0.048847134	0.601463	C5	-	Y	-	-	-	-	-
ENSG00000181938	GINS3	-0.371461358	0.000292	-0.396233971	0.000467	-0.55620845	4.16E-09	-0.019442726	0.914322	-0.17002705	0.181033	-0.156645881	0.4142	C4	-	Y	Y	Y	-	-	-
ENSG00000182004	SNRPE	-0.247795885	0.000477	-0.220585985	0.005932	-0.354890091	2.61E-06	0.03392938	0.693359	-0.092230823	0.224024	-0.131548788	0.168713	C4	-	-	-	Y	-	-	-
ENSG00000182022	CHST15	-1.164707332	2.67E-08	-0.092299547	0.760829	-0.377556472	0.08284	1.079063903	2.69E-08	0.801654745	0.000108	-0.281942312	0.273604	C3	-	Y	-	-	Y	Y	-
ENSG00000182103	FAM181B	-1.171933457	7.56E-11	0.100868612	0.689265	-0.04247031	0.85929	1.281004755	2.71E-12	1.144217268	4.15E-11	-0.139627402	0.622626	C3	-	Y	-	-	Y	Y	-
ENSG00000182141	ZNF708	0.099926131	0.392373	-0.221066535	0.118483	-0.236379507	0.06767	-0.31445899	0.007277	-0.321544779	0.004378	-0.012862139	0.971648	C5	-	-	-	-	Y	Y	-
ENSG00000182154	MRPL41	-0.406342037	0.003659	-0.211863068	0.203172	-0.24038019	0.085574	0.201394352	0.163685	0.181057219	0.172371	-0.02592005	0.930809	C4	-	Y	-	-	-	-	-
ENSG00000182158	CREB3L2	0.551704778	9.44E-14	0.320098281	0.000232	0.277763752	0.006021	-0.225623024	9.13E-05	-0.259142975	0.000224	-0.039332402	0.822475	C5	-	Y	Y	-	-	-	-
ENSG00000182165	TP53TG1	-0.420551394	0.010946	-0.249637627	0.189767	-0.103413933	0.654287	0.177418123	0.37419	0.332083198	0.072934	0.149189907	0.615724	C4	-	Y	-	-	-	-	-
ENSG00000182173	TSEN54	0.554228042	8.30E-17	0.045069086	0.798038	0.149364494	0.09482	-0.502593494	2.53E-09	-0.390215542	9.44E-11	0.107037359	0.533078	C5	Y	Y	-	-	Y	Y	-
ENSG00000182175	RGMA	-0.784866234	0.038763	-0.394901219	0.413956	-0.195276111	0.704973	0.396231294	0.376333	0.604210561	0.140493	0.202020543	NA	C4	-	Y	-	-	-	-	-
ENSG00000182179	UBA7	1.797583187	5.14E-15	0.852056102	0.026008	0.743590402	0.02929	-0.935814942	0.000177	-1.038628404	3.08E-06	-0.106441922	NA	C5	-	Y	Y	Y	Y	Y	-
ENSG00000182195	LDOC1	-0.337379036	0.002738	-0.324374434	0.017662	-0.154030443	0.271498	0.019947486	0.916983	0.198146809	0.131392	0.173160533	0.397037	C4	-	Y	Y	-	-	-	-
ENSG00000182197	EXT1	0.328179631	0.000395	0.351807267	0.002193	0.159768261	0.216977	0.030556071	0.817705	-0.153599745	0.144149	-0.189218855	0.225175	C1	-	Y	Y	-	-	-	-
ENSG00000182199	SHMT2	-0.542334221	1.04E-28	-0.449802354	2.76E-08	-0.253699793	0.000564	0.099166233	0.223075	0.303129046	1.65E-07	0.1992832	0.087131	C4	-	Y	Y	-	-	Y	-

ENSG00000182247	UBE2E2	-0.319909743	0.006421	-0.283059848	0.046468	-0.142716362	0.324656	0.042929395	0.780109	0.19179562	0.080424	0.143518054	0.418076	C4	-	Y	-	-	-	-	-
ENSG00000182257	PRR34	-2.094836885	0.004947	-0.978387709	NA	-1.474845387	0.047867	1.122725696	0.326518	0.634949832	0.619472	-0.491416873	NA	C3	-	Y	-	Y	-	-	-
ENSG00000182263	FIGN	0.564851318	5.51E-07	0.288535493	0.035609	0.247267304	0.113013	-0.270312917	0.002048	-0.30312327	0.003301	-0.03839838	0.884001	C1	-	Y	-	-	-	Y	-
ENSG00000182307	C8orf33	0.086320328	0.165263	-0.070424917	0.453868	-0.230266703	0.000343	-0.150188172	0.016308	-0.301718257	1.39E-08	-0.156995172	0.077815	C5	-	-	-	-	-	Y	-
ENSG00000182308	DCAF4L1	1.473492167	0.000303	0.853640727	0.118208	0.716407376	0.23687	-0.615213254	0.137731	-0.740781135	0.093348	-0.134384537	NA	C1	-	Y	-	-	-	-	-
ENSG00000182310	SPACA6	0.363877505	0.114665	0.322164347	0.251333	0.648496933	0.006723	-0.035635083	0.909997	0.299086403	0.215521	0.32926535	0.333965	C4	-	-	-	Y	-	-	-
ENSG00000182318	ZSCAN22	0.51967577	0.00013	-0.222886298	0.308054	0.396219897	0.012083	-0.736053721	4.27E-07	-0.108734784	0.513542	0.621598407	0.00059	C5	-	Y	-	Y	Y	-	Y
ENSG00000182326	C1S	1.810884045	0.013831	1.030200681	NA	0.992923091	NA	-0.765463962	0.248684	-0.804171469	0.237596	-0.039597329	NA	C1	-	Y	-	-	-	-	-
ENSG00000182359	KBTBD3	-0.028123436	0.933115	-0.186190435	0.574502	0.503383458	0.027722	-0.151564011	0.598707	0.546478047	0.009166	0.69219729	0.006373	C4	-	-	-	Y	-	Y	Y
ENSG00000182362	YBEY	-0.335329808	0.045958	-0.012178183	0.967843	-0.103328084	0.649426	0.32924992	0.013244	0.247440175	0.078014	-0.086847162	0.723061	C4	-	Y	-	-	Y	-	-
ENSG00000182366	FAM87A	2.837695466	0.04999	2.206651036	NA	1.40078909	NA	-0.620558989	0.636281	-1.420826458	0.298933	-0.802391997	NA	C5	-	Y	-	-	-	-	-
ENSG00000182372	CLN8	0.984783688	3.08E-23	0.507413986	1.19E-05	0.653149661	3.29E-08	-0.470942056	1.81E-06	-0.317304438	0.003259	0.148561603	0.438693	C5	-	Y	Y	Y	Y	Y	-
ENSG00000182389	CACNB4	0.037870506	0.859722	0.172295286	0.518157	0.382506876	0.018706	0.140972844	0.527359	0.35929573	0.015309	0.213326105	0.474909	C4	-	-	-	Y	-	Y	-
ENSG00000182400	TRAPPC6B	0.373188756	0.049006	0.161974369	0.56256	0.165611432	0.558872	-0.204261577	0.2192	-0.193621299	0.300405	0.005887701	0.991278	C1	-	Y	-	-	-	-	-
ENSG00000182405	PGBD4	0.76304442	2.51E-12	0.159507175	0.361572	0.416365732	0.002362	-0.596917046	1.71E-07	-0.332064666	0.005592	0.259872224	0.16715	C5	-	Y	-	Y	Y	Y	-
ENSG00000182459	TEX19	-0.810123931	0.148287	-1.711082771	0.012092	-1.676133294	0.014137	-0.891793185	0.280901	-0.851313308	0.320162	0.031571817	NA	C3	-	-	Y	Y	-	-	-
ENSG00000182463	TSHZ2	0.405515913	0.037491	-0.231188572	0.430543	-0.379247852	0.117525	-0.630226587	0.002198	-0.77011998	9.39E-05	-0.145549268	0.755306	C5	-	Y	-	-	Y	Y	-
ENSG00000182472	CAPN12	-1.003132489	0.076368	-1.100298121	0.034569	-0.405878877	0.437803	-0.089974683	0.92408	0.61287435	0.338169	0.697688082	NA	C3	-	-	Y	-	-	-	-
ENSG00000182492	BGN	-1.485282941	0.270109	0.025491859	NA	0.824764054	NA	1.528730178	NA	2.329215613	0.02868	0.815628809	NA	C2	-	-	-	-	-	Y	-
ENSG00000182504	CEP97	0.323122997	0.001198	-0.061548493	0.744906	0.124120312	0.366291	-0.377938407	1.32E-05	-0.184509938	0.019416	0.188201482	0.186277	C5	-	Y	-	-	Y	-	-
ENSG00000182511	FES	-2.671908274	0.001549	-1.11933964	NA	-0.957241345	0.178881	1.575968785	0.183936	1.730494746	0.110804	0.161001787	NA	C3	-	Y	-	-	-	-	-
ENSG00000182518	FAM104B	-0.323304707	0.003396	-0.123843204	0.386432	-0.085972079	0.581782	0.20594853	0.063201	0.252160745	0.026381	0.04082867	0.869474	C4	-	Y	-	-	-	-	-
ENSG00000182580	EPHB3	-1.200229274	1.63E-15	-0.176794999	0.47493	-0.129787073	0.492698	1.030508724	9.53E-09	1.085653183	8.47E-14	0.050278809	0.905466	C3	-	Y	-	-	Y	Y	-
ENSG00000182628	SKA2	-0.413405868	3.69E-13	-0.068143049	0.43984	-0.288433284	1.82E-06	0.351786855	3.81E-12	0.139821524	0.007092	-0.21724924	0.000206	C4	-	Y	-	-	Y	-	-
ENSG00000182632	CCNYL2	-0.331294584	0.000313	-0.02922889	0.874481	0.009133544	0.958243	0.308906753	0.0054	0.355076994	0.000467	0.040462835	0.885412	C4	-	Y	-	-	Y	Y	-
ENSG00000182648	LINC01006	-0.017921452	0.936888	0.236358072	0.182307	0.335094409	0.01558	0.26090273	0.083339	0.367486784	0.003551	0.102113853	0.663858	C4	-	-	-	Y	-	Y	-
ENSG00000182667	NTM	-0.008582998	0.981764	1.083511837	6.79E-07	0.904563428	0.000248	1.098950493	5.42E-07	0.927928506	0.000172	-0.176177786	0.661659	C2	-	-	Y	Y	Y	Y	-
ENSG00000182685	BRICD5	0.186794744	0.321394	-0.043691632	0.88754	-0.141907589	0.537033	-0.223517726	0.171913	-0.313364546	0.032459	-0.095532376	0.755306	C5	-	-	-	-	-	Y	-
ENSG00000182700	IGIP	0.18875226	0.421872	-0.461857108	0.189253	0.003883524	0.991763	-0.642402775	0.012392	-0.170514335	0.431987	0.467916284	0.221914	C5	-	-	-	-	Y	-	-
ENSG00000182718	ANXA2	-0.094546257	0.539136	0.498265189	7.93E-05	0.418210612	0.000123	0.59997314	2.85E-07	0.527724052	2.74E-07	-0.076516004	0.723061	C4	-	-	Y	Y	Y	Y	-
ENSG00000182747	SLC35D3	-0.670259203	0.006775	-0.601492858	0.045684	-0.779929001	0.002753	0.074622939	0.854194	-0.094817489	0.804826	-0.174763402	NA	C4	-	Y	Y	Y	-	-	-
ENSG00000182752	PAPPA	0.342909933	0.191769	0.647361962	0.04627	0.59576071	0.016199	0.309320591	0.250876	0.2673634	0.185912	-0.046823756	0.940125	C1	-	-	Y	Y	-	-	-
ENSG00000182771	GRID1	0.315113441	0.007282	-0.217204421	0.349155	-0.615601188	2.01E-05	-0.526374226	0.000814	-0.915827776	5.77E-14	-0.396361615	0.135408	C5	-	Y	-	Y	Y	Y	-
ENSG00000182796	TMEM198B	-0.695837432	2.70E-20	-0.173091594	0.16114	-0.104883047	0.255918	0.529552318	5.18E-08	0.605468982	1.11E-16	0.071315375	0.705879	C2	-	Y	-	-	Y	Y	-
ENSG00000182827	ACBD3	0.327758198	2.69E-06	0.003663691	0.980292	0.04975671	0.639345	-0.317836826	2.30E-08	-0.263862225	6.44E-07	0.048815256	0.68056	C5	-	Y	-	-	Y	-	-
ENSG00000182841	RRP7BP	0.392003879	0.050548	0.327581459	0.158369	0.454209219	0.015754	-0.05880111	0.834259	0.077035915	0.757678	0.129210036	0.716724	C1	-	-	-	Y	-	-	-
ENSG00000182901	RGS7	0.639268983	0.003303	0.056092576	0.896919	0.384579646	0.163776	-0.577198193	0.01458	-0.24045201	0.344307	0.331636806	0.399036	C5	-	Y	-	-	Y	-	-
ENSG00000182912	TSPEAR-AS2	-0.655018397	4.11E-18	0.19923332	0.446508	0.450677229	0.023306	0.861412378	4.15E-06	1.1204512	6.18E-11	0.253369978	0.566662	C4	-	Y	-	Y	Y	Y	-
ENSG00000182957	SPATA13	-0.467156615	0.009005	0.063597878	0.80735	0.078428204	0.742981	0.53801408	0.00188	0.560189856	0.001329	0.017712319	0.967582	C2	-	Y	-	-	Y	Y	-
ENSG00000182983	ZNF662	-0.692500045	1.51E-08	-0.306392342	0.029976	-0.387498212	0.004417	0.392659283	0.004787	0.319698808	0.030753	-0.078455545	0.78435	C4	-	Y	Y	Y	Y	Y	-
ENSG00000182986	ZNF320	-0.068026142	0.428427	0.091336644	0.321794	0.219516104	0.003553	0.16582435	0.003323	0.302106748	2.61E-08	0.130936051	0.077977	C4	-	-	-	-	-	Y	-
ENSG00000183010	PYCR1	-0.532273931	4.85E-18	-0.449656968	1.77E-09	-0.190422073	0.029649	0.089008434	0.306959	0.356345291	6.29E-07	0.262369482	0.014086	C4	Y	Y	Y	-	-	Y	-
ENSG00000183011	NAA38	-0.379213986	6.11E-05	-0.269471865	0.01411	-0.192357351	0.125695	0.116565519	0.277687	0.201690469	0.058101	0.080058969	0.679753	C4	-	Y	-	-	-	-	-
ENSG00000183018	SPNS2	-0.662818818	0.028278	0.07505202	0.87807	-0.094285264	0.827004	0.744726808	0.023159	0.58352672	0.083664	-0.16646794	NA	C4	-	Y	-	-	Y	-	-
ENSG00000183023	SLC8A1	-0.621742287	0.006487	0.005287677	0.990205	0.043705446	0.901217	0.633099875	0.004208	0.679181149	0.001831	0.041252802	0.939246	C4	-	Y	-	-	Y	Y	-
ENSG00000183044	ABAT	-0.194289658	0.001861	0.16635753	0.039404	0.274071501	7.58E-06	0.366948414	2.44E-10	0.483130316	8.08E-25	0.11086654	0.209913	C4	-	-	-	-	Y	Y	-
ENSG00000183048	SLC25A10	-0.295454197	0.076018	0.214230563	0.249186	0.08256473	0.721104	0.516443472	0.000252	0.3929004	0.012706	-0.128903703	0.629977	C4	-	-	-	-	Y	Y	-
ENSG00000183077	AFMID	-0.333889823	0.000567	-0.045977764	0.793275	-0.040245568	0.793075	0.294633167	0.002609	0.308539534	0.000627	0.008650151	0.974616	C4	-	Y	-	-	-	Y	-
ENSG00000183087	GAS6	-0.344768034	3.74E-07	-0.14037885	0.120918	-0.111537106	0.30027	0.210784759	0.002507	0.248168819	0.001403	0.03171403	0.871807	C4	-	Y	-	-	-	-	-
ENSG00000183091	NEB	-0.498557327	0.078239	-0.076110254	0.874896	0.137062073	0.684161	0.428411454	0.205278	0.64965663	0.014196	0.215159276	0.667135	C2	-	-	-	-	-	Y	-
ENSG00000183092	BEGAIN	0.324694759	7.88E-09	0.061001104	0.621893	-0.003439175	0.977236	-0.256662553	7.41E-05	-0.313612451	5.52E-10	-0.061680052	0.67669	C5	-	Y	-	-	-	Y	-
ENSG00000183098	GPC6	0.466117095	2.71E-06	0.00324659	0.989499	0.066961032	0.693136	-0.455400328	1.21E-06	-0.384724872	1.75E-05	0.065996307	0.784312	C5	-	Y	-	-	Y	Y	-
ENSG00000183111	ARHGEF37	-0.80692597	2.80E-05	-0.387051517	0.051124	-0.413164431	0.028807	0.426968744	0.03917	0.408644815	0.049029	-0.022649586	0.959572	C3	-	Y	-	Y	Y	Y	-
ENSG00000183137	CEP57L1	0.420986882	0.000856	0.260350083	0.14236	0.339626287	0.012222	-0.154974632	0.289216	-0.066554626	0.630168	0.083393899	0.739574	C5	-	Y	-	Y	-	-	-
ENSG00000183153	GJD3	-1.604077748	0.002125	-0.656249582	0.235225	-0.858882295	0.076942	0.955722166	0.124125	0.759489894	0.236819	-0.200205252	NA	C3	-	Y	-	-	-	-	-
ENSG00000183160	TMEM119	-0.616118852	9.10E-21	-0.236403026	0.001579	-0.201274973	0.014509	0.38628951	1.52E-10	0.429610201	2.88E-11	0.038001544	0.791116	C4	-	Y	-	-	Y	Y	-
ENSG00000183186	C2CD4C	-0.463073355	0.004335	-0.559461939	0.007405	-0.15614604	0.485938	-0.089056958	0.744472	0.321852624	0.090798	0.405537517	0.153647	C4	-	Y	Y	-	-	-	-
ENSG00000183248	PRR36	0.293814283	0.001758	0.089131399	0.542872	0.313325747	0.002372	-0.197961749	0.041392	0.034260947	0.781811	0.226908382	0.070167	C4	-	-	-	Y	-	-	-
ENSG00000183250	LINC01547	-1.181657643	2.15E-12	-0.3904655	0.072362	-0.537978593	0.002194	0.799785008	0.000254	0.658369189	0.001533	-0.14603612	0.723061	C3	-	Y	-	Y	Y	Y	-
ENSG00000183307	TMEM121B	0.200017918	0.098569	-0.277420278	0.058973	-0.167760166	0.28585	-0.47082503	0.000106	-0.353133695	0.004222	0.112577787	0.665374	C5	-	-	-	-	Y	Y	-
ENSG00000183309	ZNF623	0.367188674	9.42E-07	-0.01852817	0.895662	0.155603678	0.110811	-0.379774297	3.19E-10	-0.196981809	0.003291	0.177202904	0.062906	C5	-	Y	-	-	Y	-	-
ENSG00000183317	EPHA10	-0.761364698	1.89E-06	-0.44994339	0.023727	-0.353495333	0.057442	0.317255856	0.068003	0.423041968	0.006062	0.10053077	0.757663	C4	-	Y	Y	-	-	Y	-
ENSG00000183340	JRKL	0.65247242	7.67E-15	0.002770931	0.989307	0.335959672	0.00048	-0.643314113	5.05E-13	-0.301729847	0.00044	0.336185763	0.003748	C5	-	Y	-	Y	Y	Y	Y
ENSG00000183346	CABCOCO1	-1.343452088	8.68E-06	-0.25294477	0.500713	-0.092396517	0.824554	1.099025032	0.001803	1.26499845	0.000211	0.162175607	NA	C2	-	Y	-	-	Y	Y	-
ENSG00000183386	FHL3	0.537521873	0.000433	0.116747367	0.612298	0.223878361	0.306438	-0.414728913	0.009259	-0.299193529	0.105676	0.110732778	0.757698	C5	-	Y	-	-	Y	-	-
ENSG00000183426	NPIPA1	0.119712618	0.26875	-0.101496671	0.343679	-0.334282434	0.003286	-0.214447798	0.028925	-0.439324138	6.29E-05	-0.230252506	0.120558	C5	-	-	-	Y	-	Y	-
ENSG00000183474	GTF2H2C	0.328556076	0.000717	0.128472862	0.349122	0.111595212	0.425805	-0.193541375	0.078028	-0.202224485	0.069035	-0.013947605	0.96197	C5	-	Y	-	-	-	-	-

ENSG00000183475	ASB7	0.327402706	0.000393	-0.197165168	0.1556	0.196356364	0.119103	-0.51804659	3.26E-07	-0.116422384	0.324604	0.396481545	0.005195	C5	-	Y	-	-	Y	-	Y
ENSG00000183530	PRR14L	0.349308552	5.54E-06	0.098131216	0.43393	0.174559565	0.06047	-0.244755489	0.001484	-0.160094641	0.02232	0.079301669	0.592085	C5	-	Y	-	-	-	-	-
ENSG00000183570	PCBP3	-0.345577773	2.52E-12	0.038464093	0.64357	0.053266589	0.526671	0.390576251	2.43E-16	0.413533046	2.75E-15	0.017741333	0.905184	C4	-	Y	-	-	Y	Y	-
ENSG00000183576	SETD3	0.375476117	1.08E-07	0.26220899	0.000484	0.146078029	0.072226	-0.106799553	0.135274	-0.214631754	0.000788	-0.113261253	0.21454	C5	-	Y	-	-	-	-	-
ENSG00000183617	MRPL54	-0.567686341	2.21E-07	-0.184705909	0.184399	-0.520720456	1.39E-05	0.389488622	0.000304	0.062141485	0.696119	-0.332838285	0.015577	C4	-	Y	-	Y	Y	-	Y
ENSG00000183647	ZNF530	-0.332154743	0.003486	-0.381340711	0.006348	-0.469253299	0.00011	-0.04266331	0.806082	-0.122391548	0.379716	-0.085099353	0.748723	C4	-	Y	Y	Y	-	-	-
ENSG00000183665	TRMT12	0.465457954	3.51E-06	0.106090154	0.512616	0.236045584	0.078912	-0.35293114	0.000934	-0.214928224	0.068868	0.133299579	0.525068	C5	-	Y	-	-	Y	-	-
ENSG00000183682	BMP8A	-0.360293379	0.10675	-0.161235779	0.607926	0.225983542	0.368437	0.206218999	0.458384	0.600849895	0.004521	0.39027268	0.19895	C4	-	-	-	-	-	Y	-
ENSG00000183684	ALYREF	-0.255269677	0.00305	-0.188118547	0.343679	-0.92016945	9.72E-09	0.072997918	0.762263	-0.650275724	0.000361	-0.728670814	0.003251	C5	-	-	-	Y	-	Y	Y
ENSG00000183718	TRIM52	0.183462841	0.078841	0.329550492	0.004936	0.221224708	0.05861	0.152478946	0.176132	0.052413062	0.685164	-0.105435405	0.580345	C4	-	-	Y	-	-	-	-
ENSG00000183722	LHFPL6	-0.868738373	0.007589	-0.440311588	0.230409	-0.513304663	0.13598	0.435819279	0.295175	0.3707029	0.390459	-0.070296628	NA	C3	-	Y	-	-	-	-	-
ENSG00000183735	TBK1	0.502328104	9.66E-13	0.087648006	0.499762	0.140931268	0.126337	-0.408001919	2.97E-08	-0.346842874	6.36E-08	0.055689147	0.744484	C5	-	Y	-	-	Y	Y	-
ENSG00000183762	KREMEN1	-0.74514276	3.62E-15	-0.151357739	0.193869	-0.070284025	0.600044	0.600475375	1.25E-09	0.689397839	2.44E-12	0.083811148	0.644363	C4	Y	Y	-	-	Y	Y	-
ENSG00000183778	B3GALT5	-1.420832888	0.00023	-0.50753464	0.181346	-0.256565106	0.513086	0.922528943	0.034174	1.177971406	0.002724	0.254144045	NA	C3	-	Y	-	-	Y	Y	-
ENSG00000183798	EMILIN3	-1.266672838	1.02E-13	-0.285040162	0.164341	-0.136286396	0.534454	0.987744973	8.69E-08	1.145303662	7.39E-11	0.151729765	0.620166	C2	-	Y	-	-	Y	Y	-
ENSG00000183808	RBM12B	0.384047163	1.22E-05	0.069491141	0.670838	0.286435993	0.0041	-0.308526917	5.04E-05	-0.082824759	0.255977	0.220200411	0.036531	C5	-	Y	-	-	Y	-	-
ENSG00000183814	LIN9	0.253193608	0.023039	-0.102522914	0.507256	-0.09464952	0.55626	-0.349492234	1.80E-06	-0.334146204	5.49E-05	0.010159282	0.967143	C5	-	-	-	-	Y	Y	-
ENSG00000183831	ANKRD45	-0.390870379	0.094627	-0.123178828	0.719099	0.10774155	0.706219	0.273953478	0.286616	0.512901117	0.012418	0.234818793	0.479466	C4	-	-	-	-	-	Y	-
ENSG00000183837	PNMA3	-1.001527968	2.60E-07	-0.64779166	0.000175	-0.638205331	0.000448	0.361283279	0.117274	0.378709061	0.11253	0.012245026	0.98105	C3	-	Y	Y	Y	-	-	-
ENSG00000183850	ZNF730	0.320983047	0.087999	-0.175496525	0.575652	0.259034844	0.293305	-0.489671406	0.017649	-0.046971169	0.862145	0.437538191	0.161345	C5	-	-	-	-	Y	-	-
ENSG00000183853	KIRREL1	-0.584572877	0.2533	0.13119146	0.864561	0.433050073	0.370554	0.722696419	0.178651	1.032373352	0.016139	0.305108133	NA	C2	-	-	-	-	-	Y	-
ENSG00000183864	TOB2	0.806315725	5.70E-14	-0.235599237	0.210973	0.632808586	3.25E-08	-1.037242863	1.54E-20	-0.159373056	0.116465	0.873064771	1.76E-12	C5	-	Y	-	Y	Y	-	Y
ENSG00000183873	SCN5A	-0.13345171	0.290854	-0.328767097	0.014074	-0.195795921	0.181208	-0.189292299	0.166963	-0.04791897	0.780853	0.136633326	0.572744	C4	-	-	Y	-	-	-	-
ENSG00000183891	TTC32	-0.414353765	0.002379	-0.061149213	0.751381	-0.292588159	0.027634	0.360631313	0.013823	0.136135434	0.425769	-0.22902796	0.223372	C2	-	Y	-	-	Y	-	-
ENSG00000183935	HTR7P1	-0.806671798	0.009213	-0.172379838	0.703909	-0.092348487	0.826933	0.642106371	0.06137	0.729289069	0.019435	0.083902894	NA	C3	-	Y	-	-	-	Y	-
ENSG00000183971	NPW	-0.71005062	0.026513	-0.608856532	0.072325	-0.570786786	0.089532	0.108042077	0.823847	0.154677587	0.743575	0.04136173	NA	C3	-	Y	-	-	-	-	-
ENSG00000184005	ST6GALNAC3	-0.489324243	3.98E-09	-0.086107579	0.520585	0.001933501	0.991165	0.409883393	1.18E-05	0.505916515	3.41E-08	0.090412871	0.638347	C4	-	Y	-	-	Y	Y	-
ENSG00000184009	ACTG1	0.600930316	5.24E-61	0.300355895	4.20E-12	0.24432156	2.12E-08	-0.293935231	3.21E-31	-0.3417369	5.96E-45	-0.053123577	0.272207	C5	-	Y	Y	-	-	Y	-
ENSG00000184058	TBX1	2.244027466	5.67E-07	1.371700023	0.037102	1.270112804	0.037491	-0.863994783	0.050755	-0.958344916	0.019294	-0.099302998	NA	C1	-	Y	Y	Y	-	Y	-
ENSG00000184083	FAM120C	0.204705915	0.088929	0.046780628	0.806965	0.32068514	0.004963	-0.151510413	0.218866	0.130398109	0.258647	0.276583497	0.035084	C4	-	-	-	Y	-	-	-
ENSG00000184117	NIPSNAP1	-0.361391371	2.03E-14	-0.195150934	0.001178	-0.113162673	0.048048	0.17259415	0.000283	0.263048241	2.03E-11	0.085166648	0.221658	C4	-	Y	-	-	-	-	-
ENSG00000184144	CNTN2	-0.446872315	2.51E-08	-0.23438024	0.010521	-0.108159715	0.282914	0.218904231	0.014296	0.353347192	1.75E-05	0.129138727	0.301721	C4	-	Y	-	-	-	Y	-
ENSG00000184160	ADRA2C	-0.418927706	0.001396	-0.213166013	0.301969	-0.088622942	0.659088	0.211999354	0.256578	0.345351066	0.021768	0.127534775	0.697915	C4	-	Y	-	-	-	Y	-
ENSG00000184178	SCFD2	-0.275571945	0.029227	-0.130878478	0.442739	0.020181942	0.921877	0.150912522	0.282773	0.310240468	0.010912	0.154001602	0.432967	C4	-	-	-	-	-	Y	-
ENSG00000184185	KCNJ12	-0.945554051	0.017058	-0.678869607	0.153242	-0.360472141	0.440575	0.267163205	0.675956	0.600643048	0.231534	0.325326993	NA	C3	-	Y	-	-	-	-	-
ENSG00000184208	C22orf46	-0.536082842	1.90E-09	-0.299577143	0.003669	-0.205069457	0.07672	0.242835494	0.008516	0.345759973	0.000283	0.097489609	0.575574	C4	-	Y	-	-	-	Y	-
ENSG00000184209	SNRNP35	-0.305187923	0.002657	-0.332223782	0.004184	-0.21898798	0.064633	-0.020100771	0.890303	0.101129628	0.377372	0.115954461	0.50052	C4	-	Y	Y	-	-	-	-
ENSG00000184226	PCDH9	0.382127124	9.66E-06	0.267025966	0.01082	0.112015464	0.500159	-0.108550516	0.317548	-0.255468124	0.208686	-0.153362989	0.64532	C5	-	Y	-	-	-	-	-
ENSG00000184232	OAF	-0.55957888	0.031639	-0.397112589	0.183392	-0.152127331	0.62861	0.17031257	0.630552	0.422482525	0.125509	0.248052356	0.556648	C4	-	Y	-	-	-	-	-
ENSG00000184254	ALDH1A3	-1.363503802	2.65E-11	-0.315830633	0.200452	-0.208703924	0.445428	1.053166798	6.06E-06	1.169500292	9.34E-07	0.110412612	0.828373	C3	-	Y	-	-	Y	Y	-
ENSG00000184261	KCNK12	0.658451277	0.088409	-0.214241196	0.76307	0.082421299	0.900873	-0.865579355	0.042978	-0.560952579	0.174631	0.299755113	NA	C5	-	-	-	-	Y	-	-
ENSG00000184305	CCSER1	0.310291761	0.031759	-0.191973377	0.332656	-0.248438591	0.160033	-0.496419062	0.000195	-0.544550985	3.78E-05	-0.053508184	0.879868	C5	-	Y	-	-	Y	Y	-
ENSG00000184343	SRPK3	-0.723326287	0.00217	0.127331902	0.684954	0.220990635	0.404353	0.856245354	0.000334	0.959061958	5.07E-05	0.096652394	0.835119	C4	-	Y	-	-	Y	Y	-
ENSG00000184349	EFNA5	-0.346123972	0.002754	-0.213769517	0.187203	-0.346721965	0.008285	0.13867525	0.381159	0.014056702	0.940401	-0.130042593	0.607587	C4	-	Y	-	Y	-	-	-
ENSG00000184368	MAP7D2	-0.404116022	0.000104	-0.29465481	0.026231	-0.129246723	0.374827	0.115371119	0.44906	0.289489864	0.019347	0.169239266	0.432716	C4	-	Y	-	-	-	-	-
ENSG00000184371	CSF1	-0.881087312	0.006925	-0.436143751	0.26856	-0.588386366	0.135543	0.453581789	0.283869	0.307977637	0.532319	-0.151091416	NA	C3	-	Y	-	-	-	-	-
ENSG00000184381	PLA2G6	-0.81368788	1.63E-06	-0.202360891	0.429273	-0.072827422	0.788842	0.618475257	0.0037	0.755643863	0.000138	0.131046641	0.759727	C4	-	Y	-	-	Y	Y	-
ENSG00000184402	SS18L1	0.479197129	4.14E-18	0.20999755	0.004534	0.155677392	0.023539	-0.262445378	1.63E-06	-0.308905697	5.29E-10	-0.051745328	0.653173	C5	-	Y	-	-	-	Y	-
ENSG00000184470	TXNRD2	-0.177714469	0.210821	0.247209143	0.059647	0.11811375	0.477378	0.431590957	0.000154	0.310625521	0.016834	-0.126386376	0.515426	C4	-	-	-	-	Y	Y	-
ENSG00000184489	PTP4A3	-0.971391714	9.64E-14	-0.104886596	0.545592	-0.346810435	0.011984	0.873369513	1.99E-12	0.639583556	2.88E-06	-0.2389413	0.151423	C2	-	Y	-	Y	Y	Y	-
ENSG00000184508	HDDC3	-0.630399542	0.001494	-0.062090086	0.832122	-0.350943812	0.118882	0.575508304	0.003772	0.294323175	0.231532	-0.286573111	0.332752	C3	-	Y	-	-	Y	-	-
ENSG00000184517	ZFP1	0.145708618	0.160701	-0.193510231	0.088938	-0.052114819	0.726047	-0.332806154	0.000274	-0.1830984	0.072882	0.144275465	0.352857	C5	-	-	-	-	Y	-	-
ENSG00000184524	CEND1	-0.560146586	8.30E-07	-0.020321653	0.926556	0.030367317	0.867896	0.5461109	5.59E-07	0.605328746	3.46E-10	0.054574062	0.818037	C4	-	Y	-	-	Y	Y	-
ENSG00000184564	SLITRK6	0.808372864	1.21E-10	0.051566609	0.833724	-0.009249681	0.972309	-0.751077133	1.15E-13	-0.802795595	3.33E-14	-0.057437638	0.841508	C5	-	Y	-	-	Y	Y	-
ENSG00000184588	PDE4B	0.302356136	0.000123	0.058083865	0.637076	0.025832861	0.83882	-0.238215953	0.000514	-0.261893121	8.26E-05	-0.029140722	0.857353	C5	-	Y	-	-	-	-	-
ENSG00000184601	C14orf180	-0.68209307	0.007282	0.568464158	0.032801	0.500733965	0.060294	1.2571663	5.39E-08	1.198115055	3.60E-07	-0.0649816	0.911454	C2	-	Y	Y	-	Y	Y	-
ENSG00000184602	SNN	-0.338726822	7.76E-07	-0.169013176	0.040622	-0.20431986	0.005747	0.176064252	0.025933	0.149123452	0.056714	-0.03244244	0.845052	C4	-	Y	-	-	-	-	-
ENSG00000184608	FAM167A-AS1	-1.302128747	0.003722	0.371552641	0.39732	0.090416818	0.877682	1.682490317	1.20E-05	1.408279391	0.001179	-0.278272481	NA	C3	-	Y	-	-	Y	Y	-
ENSG00000184613	NELL2	-0.167833902	0.296751	-0.409345493	0.006028	-0.370137953	0.010064	-0.235014136	0.08415	-0.187528005	0.176005	0.042434851	0.883781	C4	-	-	Y	Y	-	-	-
ENSG00000184616	SPDYE12P	-1.128557551	0.000781	-0.618110602	0.056032	-0.501009416	0.117839	0.514950427	0.248278	0.641905363	0.125816	0.120374379	NA	C3	-	Y	-	-	-	-	-
ENSG00000184619	KRBA2	0.311437504	0.478109	0.28491574	0.621893	0.767802569	0.041835	-0.023231067	0.959645	0.47035217	0.090587	0.489269081	0.231211	C2	-	-	-	Y	-	-	-
ENSG00000184634	MED12	0.301191507	2.33E-06	0.165304468	0.048277	0.262899058	8.27E-05	-0.129406751	0.061188	-0.023810612	0.762398	0.100511636	0.316749	C1	-	Y	-	-	-	-	-
ENSG00000184635	ZNF93	0.16336259	0.325038	-0.153060395	0.449101	-0.102432108	0.567694	-0.309971833	0.040134	-0.251407122	0.065212	0.053534268	0.862177	C5	-	-	-	-	Y	-	-
ENSG00000184675	AMER1	0.483417962	2.40E-06	0.132715316	0.326901	0.225482667	0.048225	-0.344488986	0.000346	-0.243550294	0.012472	0.095896346	0.565651	C5	-	Y	-	-	Y	-	-
ENSG00000184697	CLDN6	-3.241628301	0.032707	-2.029124645	NA	-2.901383704	NA	1.23319986	NA	0.357438887	NA	-0.877210222	NA	C3	-	Y	-	-	-	-	-
ENSG00000184708	EIF4ENIF1	0.300127682	1.19E-06	0.102296551	0.258365	0.071185625	0.451653	-0.191217477	0.002927	-0.214248245	0.000778	-0.028233841	0.862177	C5	Y	Y	-	-	-	-	-
ENSG00000184719	RNLS	-1.700568775	0.038782	-0.756141196	NA	-0.351473986	0.72225	0.954812497	0.393449	1.364140746	0.166827	0.406361852	NA	C3	-	Y	-	-	-	-	-

ENSG00000184743	ATL3	0.09902209	0.295522	-0.287116787	0.003535	0.001785488	0.992803	-0.380137994	3.17E-07	-0.082580281	0.491963	0.291167234	0.037476	C5	-	-	-	-	Y	-	-
ENSG00000184785	SMIM10	-0.961127252	0.004544	-0.934455355	0.018557	-0.687103522	0.052051	0.029491457	0.963157	0.288551528	0.551543	0.251235949	NA	C3	-	Y	Y	-	-	-	-
ENSG00000184792	OSBP2	-0.671099235	2.32E-05	-0.176516898	0.419726	-0.018162244	0.946887	0.501060787	0.00639	0.667637697	0.000136	0.16092749	0.605037	C4	-	Y	-	-	Y	Y	-
ENSG00000184809	B3GALT5-AS1	-1.906851596	0.044223	-0.65137989	NA	-1.471891654	NA	1.268938878	0.263949	0.451148457	0.781811	-0.82334575	NA	C3	-	Y	-	-	-	-	-
ENSG00000184828	ZBTB7C	0.246828143	0.524831	-0.57709433	0.181228	-0.15372339	0.751045	-0.816217878	0.015841	-0.3855504	0.272745	0.425414842	NA	C5	-	-	-	-	Y	-	-
ENSG00000184838	PRR16	-0.419281565	2.90E-07	-0.199751746	0.065845	-0.278555197	0.00737	0.225954149	0.011479	0.155797636	0.113727	-0.075927164	0.686328	C4	-	Y	-	-	-	-	-
ENSG00000184857	TMEM186	-0.139883968	0.216229	-0.306566328	0.033384	-0.189665113	0.13275	-0.16021267	0.265626	-0.034905136	0.821477	0.120039423	0.619005	C5	-	-	Y	-	-	-	-
ENSG00000184863	RBM33	0.569251038	1.09E-17	0.340424749	3.68E-07	0.132982833	0.085964	-0.222490887	0.000134	-0.421919344	4.72E-15	-0.204602398	0.001663	C5	-	Y	Y	-	-	Y	-
ENSG00000184887	BTBD6	0.116479625	0.347846	0.123821397	0.357132	-0.20464575	0.081585	0.014035251	0.93168	-0.306243384	0.005665	-0.325549221	0.008562	C4	-	-	-	-	-	Y	Y
ENSG00000184897	H1-10	-0.728217751	3.57E-21	-0.26944267	0.008956	-0.278543297	0.005293	0.46534884	5.90E-19	0.4647309	6.44E-19	-0.006010315	0.976804	C4	-	Y	-	-	Y	Y	-
ENSG00000184898	RBM43	-1.003148299	4.01E-06	0.018449915	0.961535	-0.881062639	0.000153	1.027897442	5.53E-06	0.136726583	0.71236	-0.896334347	0.000741	C2	-	Y	-	Y	Y	-	Y
ENSG00000184905	TCEAL2	-0.514607802	0.002271	-0.479236694	0.017652	-0.35726951	0.071057	0.04250746	0.860443	0.172062514	0.370135	0.124277713	0.710842	C4	-	Y	Y	-	-	-	-
ENSG00000184916	JAG2	-0.395876065	0.015759	-0.161578778	0.442101	-0.283257179	0.188354	0.241713059	0.210371	0.127339407	0.611671	-0.120338043	0.755306	C4	-	Y	-	-	-	-	-
ENSG00000184925	LCN12	-2.379094428	0.001525	0.053073502	0.955022	-0.005307087	0.995008	2.441537659	0.000693	2.390081233	0.001032	-0.054180735	NA	C3	-	Y	-	-	Y	Y	-
ENSG00000184983	NDUFA6	-0.383147338	0.000294	-0.193347816	0.126304	-0.374326195	0.000292	0.196596628	0.119828	0.023742375	0.886796	-0.177959161	0.270419	C4	-	Y	-	Y	-	-	-
ENSG00000184985	SORCS2	0.75408679	0.003403	0.355333947	0.335984	0.443461826	0.204854	-0.392089084	0.157883	-0.295354289	0.329708	0.091194209	NA	C5	-	Y	-	-	-	-	-
ENSG00000184986	TMEM121	0.263940319	0.339689	0.285257956	0.390202	0.602986527	0.026467	0.027686691	0.939777	0.354103539	0.185069	0.320779912	0.421518	C4	-	-	-	Y	-	-	-
ENSG00000184992	BRI3BP	0.319027069	0.000206	0.108381819	0.358872	0.108989216	0.307324	-0.204216463	0.011055	-0.195506505	0.011215	0.003141445	0.988432	C5	-	Y	-	-	-	-	-
ENSG00000185008	ROBO2	0.078750157	0.566008	-0.482196797	0.00011	-0.29115764	0.034378	-0.554666291	4.83E-08	-0.355593048	0.001148	0.19376166	0.288265	C5	-	-	Y	-	Y	Y	-
ENSG00000185009	AP3M1	0.320901133	3.03E-08	0.014160102	0.90054	0.060105223	0.465534	-0.300549863	7.61E-09	-0.246327604	5.17E-07	0.049039305	0.638566	C5	-	Y	-	-	Y	-	-
ENSG00000185010	F8	-0.940194787	0.025898	-0.201049724	0.77021	-0.146106026	0.794852	0.748366436	0.177271	0.808542396	0.092093	0.057576037	NA	C3	-	Y	-	-	-	-	-
ENSG00000185022	MAFF	0.469494131	0.041561	0.656996351	0.006736	0.252683087	0.384234	0.191421407	0.3429	-0.203285327	0.318704	-0.399821795	0.086779	C1	-	Y	Y	-	-	-	-
ENSG00000185049	NELFA	0.327987083	5.17E-06	0.104384789	0.326985	0.105999012	0.313492	-0.217265293	0.005121	-0.207442215	0.008577	0.004495359	0.984988	C5	-	Y	-	-	-	-	-
ENSG00000185100	ADSS1	-0.495285182	0.000683	-0.321696518	0.05418	-0.10115093	0.614392	0.180281578	0.315645	0.408702851	0.007053	0.223417273	0.319638	C4	-	Y	-	-	-	Y	-
ENSG00000185122	HSF1	0.365697082	3.87E-07	0.146635576	0.176641	0.069156394	0.532489	-0.212019029	0.002555	-0.281791365	4.29E-06	-0.074973829	0.58436	C5	Y	Y	-	-	-	-	-
ENSG00000185129	PURA	0.437600994	1.49E-05	-0.146104398	0.270659	0.061365088	0.690252	-0.577937959	7.06E-12	-0.361996304	4.26E-05	0.21062222	0.089669	C5	-	Y	-	-	Y	Y	-
ENSG00000185133	INPP5J	-0.373957786	0.041825	-0.222734348	0.295119	-0.344499167	0.057891	0.157511978	0.49342	0.044295737	0.868349	-0.118783872	0.730772	C4	-	Y	-	-	-	-	-
ENSG00000185158	LRRC37B	0.449536772	0.001059	0.153034415	0.552005	0.263869461	0.077365	-0.289999613	0.123452	-0.171076	0.260969	0.1141247	0.738896	C5	-	Y	-	-	-	-	-
ENSG00000185164	NOMO2	0.548412149	1.55E-09	0.417652301	2.27E-05	0.360614112	2.27E-05	-0.124258915	0.224059	-0.173018113	0.037957	-0.053255969	0.741063	C5	-	Y	Y	Y	-	-	-
ENSG00000185168	LINC00482	-1.533628703	0.043317	0.931660926	0.119182	0.029970781	0.975315	2.474062385	0.000195	1.578495154	0.048107	-0.898974876	NA	C2	-	Y	-	-	Y	Y	-
ENSG00000185215	TNFAIP2	-1.092414955	1.14E-05	-0.844364262	0.013644	-0.527216287	0.046398	0.256119382	0.562194	0.580430127	0.049666	0.32031072	NA	C3	-	Y	Y	Y	-	Y	-
ENSG00000185220	PGBD2	-0.438496222	0.001551	-0.804113619	1.87E-08	-0.299581843	0.084985	-0.359441302	0.031897	0.153884299	0.428617	0.508486369	0.016082	C3	-	Y	Y	-	Y	-	Y
ENSG00000185238	PRMT3	-0.721085551	2.05E-10	-0.055532921	0.76564	-0.121429493	0.404353	0.671774061	6.45E-09	0.614086137	7.69E-08	-0.063232536	0.796794	C2	-	Y	-	-	Y	Y	-
ENSG00000185246	PRPF39	0.767344699	8.71E-30	0.137890656	0.21148	0.296423143	0.000811	-0.62255834	7.94E-21	-0.456105095	6.44E-12	0.161238448	0.177551	C5	Y	Y	-	-	Y	Y	-
ENSG00000185250	PPIL6	0.275913106	0.318641	0.636612044	0.012655	0.345503847	0.238788	0.366714892	0.146961	0.084185411	0.80254	-0.288309295	0.442693	C4	-	-	Y	-	-	-	-
ENSG00000185252	ZNF74	0.523538938	1.20E-15	0.089180325	0.40994	0.237297314	0.001549	-0.427824377	1.73E-10	-0.271461269	1.43E-05	0.150877632	0.13614	C5	-	Y	-	-	Y	-	-
ENSG00000185267	CDNF	-1.028250205	0.012703	-0.412883023	0.39547	-0.479491634	0.314848	0.622379801	0.192927	0.564055646	0.270379	-0.063405308	NA	C3	-	Y	-	-	-	-	-
ENSG00000185269	NOTUM	-1.072478144	0.001636	-0.458130154	0.294925	-0.655450584	0.088608	0.618676905	0.149143	0.431746529	0.353	-0.193446556	NA	C3	-	Y	-	-	-	-	-
ENSG00000185291	IL3RA	1.346938228	0.00366	0.373918151	0.658467	0.305574321	0.715877	-0.96949961	0.053327	-1.025036731	0.044556	-0.067875095	NA	C1	-	Y	-	-	-	Y	-
ENSG00000185304	RGPD2	0.58177701	0.016607	0.080624793	0.857843	0.150592009	0.665549	-0.495023446	0.062263	-0.416922076	0.095066	0.072892594	0.910446	C5	-	Y	-	-	-	-	-
ENSG00000185340	GAS2L1	-0.585943567	1.24E-06	-0.011179911	0.961535	-0.118983215	0.477378	0.581520007	8.24E-07	0.482048502	0.000129	-0.104788264	0.637005	C4	-	Y	-	-	Y	Y	-
ENSG00000185344	ATP6V0A2	0.386127322	1.92E-09	0.028759807	0.822756	0.012987186	0.920006	-0.350515002	3.34E-12	-0.358700232	1.17E-12	-0.013240871	0.938937	C5	-	Y	-	-	Y	Y	-
ENSG00000185347	TEDC1	-0.454624298	0.000254	-0.124570629	0.470209	-0.311430092	0.023016	0.336701938	0.010905	0.158218453	0.299814	-0.183907011	0.353469	C4	-	Y	-	Y	Y	-	-
ENSG00000185352	HS6ST3	-0.118360465	0.771932	0.795565378	0.009705	0.654346762	0.030709	0.91974888	0.000987	0.786968186	0.005495	-0.138403253	0.79673	C2	-	-	Y	Y	Y	Y	-
ENSG00000185361	TNFAIP8L1	0.158375063	0.237465	0.231886243	0.167021	0.313990775	0.026963	0.07992575	0.648144	0.170368036	0.238336	0.084694949	0.778535	C4	-	-	-	Y	-	-	-
ENSG00000185379	RAD51D	-0.320833272	0.004779	-0.263761245	0.055097	-0.231601574	0.084675	0.063500479	0.691168	0.104060316	0.472943	0.035089249	0.910998	C4	-	Y	-	-	-	-	-
ENSG00000185404	SP140L	-0.931514985	0.015513	-0.385324951	0.355233	-0.076121071	0.877623	0.553646576	0.244449	0.869041493	0.037104	0.311415211	NA	C2	-	Y	-	-	-	Y	-
ENSG00000185420	SMYD3	-0.436628214	0.000428	0.138549713	0.330311	0.028269693	0.877682	0.581261146	8.98E-07	0.479731212	0.000113	-0.10734138	0.587028	C4	-	Y	-	-	Y	Y	-
ENSG00000185432	METTL7A	-0.267148037	0.000519	-0.094504332	0.351454	0.02897363	0.801606	0.178925785	0.02408	0.310751958	1.63E-05	0.126251899	0.246682	C4	-	-	-	-	-	Y	-
ENSG00000185437	SH3BGR	-1.65501654	0.028134	-0.474951871	0.616354	0.16560189	0.83963	1.195603331	0.206349	1.833606387	0.008816	0.645714407	NA	C3	-	Y	-	-	-	Y	-
ENSG00000185442	FAM174B	-0.341621558	0.062071	-0.27564121	0.213331	-0.46692113	0.020022	0.072314077	0.761098	-0.110150047	0.641725	-0.188098813	0.554373	C4	-	-	-	Y	-	-	-
ENSG00000185482	STAC3	-0.879029636	0.012479	-0.133845648	0.781406	0.03397479	0.948056	0.748923683	0.047882	0.927498081	0.011792	0.171159758	NA	C2	-	Y	-	-	Y	Y	-
ENSG00000185483	ROR1	-0.281477914	0.000963	0.176660101	0.067188	0.098734071	0.356627	0.464419991	3.48E-11	0.394880606	4.70E-08	-0.074878334	0.554373	C4	-	-	-	-	Y	Y	-
ENSG00000185485	SDHAP1	0.311456765	0.013849	-0.087045308	0.658356	0.132758359	0.438687	-0.39187611	0.002578	-0.163873829	0.266994	0.222682645	0.274702	C5	-	Y	-	-	Y	-	-
ENSG00000185519	FAM131C	-0.482893061	0.069487	-0.669902171	0.035371	-0.12069333	0.752237	-0.178824696	0.662629	0.377031689	0.24399	0.551507661	NA	C4	-	-	Y	-	-	-	-
ENSG00000185559	DLK1	0.31047619	0.001183	-0.277937223	0.442159	-0.225533099	0.47239	-0.582828828	0.026177	-0.521861821	0.025847	0.056375594	0.949752	C5	-	Y	-	-	Y	Y	-
ENSG00000185567	AHNAK2	-0.851429838	7.65E-06	-0.011338961	0.977723	0.240502287	0.238304	0.846022146	1.99E-05	1.106146474	4.86E-14	0.256748336	0.357029	C4	-	Y	-	-	Y	Y	-
ENSG00000185585	OLFML2A	-1.147573328	0.040986	-0.95382497	0.153235	-0.967565303	0.104455	0.208498527	0.826227	0.192844834	0.834491	-0.018597476	NA	C3	-	Y	-	-	-	-	-
ENSG00000185608	MRPL40	-0.591988905	9.51E-07	-0.193819241	0.280758	-0.14878244	0.331747	0.405287482	0.005988	0.458228617	0.000261	0.048174677	0.888988	C2	-	Y	-	-	Y	Y	-
ENSG00000185621	LMLN	0.415581134	0.001151	0.326907145	0.049968	0.379680278	0.012401	-0.082467877	0.608709	-0.021368094	0.906446	0.055670588	0.860105	C1	-	Y	Y	Y	-	-	-
ENSG00000185630	PBX1	-0.540015101	1.67E-10	-0.125968988	0.180261	0.025668508	0.827004	0.421144187	2.01E-06	0.580186295	2.49E-12	0.154445208	0.157472	C4	-	Y	-	-	Y	Y	-
ENSG00000185634	SHC4	-1.11353454	0.002803	-0.758243569	0.095578	-0.300693356	0.502639	0.361168983	0.49581	0.827864489	0.034859	0.459758555	NA	C3	-	Y	-	-	-	Y	-
ENSG00000185658	BRWD1	0.592843055	5.30E-16	0.209465545	0.040622	0.335871396	1.65E-05	-0.377270217	9.89E-08	-0.242568644	5.77E-05	0.129514251	0.253798	C5	-	Y	-	Y	Y	-	-
ENSG00000185684	EP400P1	0.584077464	5.35E-09	0.139191626	0.391755	0.103124137	0.523104	-0.437490094	2.40E-07	-0.466088765	2.58E-08	-0.033728939	0.894788	C1	-	Y	-	-	Y	Y	-
ENSG00000185697	MYBL1	0.531061383	4.96E-07	0.311645615	0.015628	0.258513155	0.067132	-0.213378438	0.018903	-0.258670603	0.008242	-0.050521921	0.824504	C5	-	Y	Y	-	-	-	-
ENSG00000185716	MOSMO	0.386613502	6.70E-07	0.019584937	0.903543	0.101598849	0.350172	-0.361123352	1.57E-07	-0.270694277	1.66E-05	0.085248477	0.518074	C5	-	Y	-	-	Y	-	-
ENSG00000185722	ANKFY1	0.415402758	1.28E-12	0.264581162	0.000443	0.229239371	0.000612	-0.144515821	0.007377	-0.17178448	9.60E-05	-0.032426221	0.783466	C1	-	Y	-	-	-	-	-

ENSG00000185730	ZNF696	0.166160105	0.062021	-0.16546962	0.161359	0.070926235	0.533493	-0.325246557	0.000293	-0.080716279	0.393404	0.239206048	0.040729	C5	-	-	-	-	Y	-	-
ENSG00000185736	ADARB2	0.907952995	0.206673	-1.782436151	NA	-1.146910802	NA	-2.679941952	0.001221	-2.042917071	0.008918	0.643761518	NA	C5	-	-	-	-	Y	Y	-
ENSG00000185737	NRG3	-0.347440471	0.53008	0.202032746	0.765083	0.571068537	0.224085	0.554767225	0.282049	0.932662948	0.03157	0.37107402	NA	C2	-	-	-	-	-	Y	-
ENSG00000185760	KCNQ5	-0.706407028	9.37E-05	-0.328206051	0.144632	-0.144761446	0.532132	0.384164344	0.086051	0.5762558	0.00296	0.186300113	0.580345	C2	-	Y	-	-	-	Y	-
ENSG00000185800	DMWD	0.45517718	1.88E-08	0.221799251	0.008318	0.20800081	0.004231	-0.226936837	0.006702	-0.232575459	0.002094	-0.011005745	0.952524	C5	-	Y	-	-	-	-	-
ENSG00000185803	SLC52A2	0.338154106	0.000235	0.101673611	0.446733	0.224202498	0.061044	-0.229643505	0.005631	-0.099077222	0.34628	0.125481828	0.410403	C5	-	Y	-	-	-	-	-
ENSG00000185808	PIGP	-0.602049898	3.57E-05	-0.157191046	0.479176	-0.120883669	0.556073	0.450805146	0.001608	0.496826889	0.000151	0.040642324	0.908742	C4	-	Y	-	-	Y	Y	-
ENSG00000185813	PCYT2	0.093344252	0.402143	0.343589244	0.000252	0.394894959	1.81E-05	0.256815156	0.007707	0.316247491	0.000767	0.054165709	0.780374	C4	-	-	Y	Y	-	Y	-
ENSG00000185838	GNB1L	1.049033746	5.61E-12	0.39055301	0.090924	0.588055715	0.002311	-0.650725833	2.13E-05	-0.446773891	0.00302	0.199641511	0.51883	C5	-	Y	-	Y	Y	Y	-
ENSG00000185875	THNSL1	-0.183206307	0.260219	-0.383065286	0.010621	-0.097000481	0.589258	-0.193542408	0.218312	0.100402581	0.53817	0.289485996	0.07753	C4	-	-	Y	-	-	-	-
ENSG00000185880	TRIM69	0.197567219	0.041564	0.246133012	0.032356	0.441422288	2.62E-06	0.055003516	0.662629	0.258640365	0.003922	0.198366117	0.142583	C4	-	-	-	Y	-	-	-
ENSG00000185900	POMK	-0.122107433	0.103769	-0.35081031	1.75E-08	-0.138655666	0.051305	-0.222223125	0.000908	-0.001854757	0.982909	0.215038782	0.002304	C4	-	-	Y	-	-	-	-
ENSG00000185904	LINC00839	0.374446217	5.60E-11	0.121567351	0.135352	0.185388124	0.005315	-0.2466443	2.12E-05	-0.174361937	0.001637	0.066920617	0.527723	C5	-	Y	-	-	-	-	-
ENSG00000185947	ZNF267	0.281252318	0.128513	-0.068388037	0.829056	-0.202421538	0.420939	-0.344431246	0.046159	-0.469605451	0.005207	-0.130987467	0.723061	C5	-	-	-	-	Y	Y	-
ENSG00000185950	IRS2	0.187991788	1.37E-05	0.362238089	2.22E-11	0.061887091	0.443286	0.180796353	0.000171	-0.11113517	0.045419	-0.297478347	6.78E-06	C4	-	-	Y	-	-	-	-
ENSG00000185955	C7orf61	2.183806536	0.007352	0.64251891	NA	1.047350826	NA	-1.531878274	0.042632	-1.116861385	0.137866	0.408308409	NA	C5	-	Y	-	-	Y	-	-
ENSG00000185963	BICD2	0.388953496	1.57E-09	0.238840403	0.002448	0.244431553	0.002548	-0.144208382	0.013659	-0.130218605	0.037317	0.008540094	0.961589	C5	Y	Y	-	-	-	-	-
ENSG00000185986	SDHAP3	0.355695841	0.001763	0.039901831	0.852933	0.168535375	0.274975	-0.308919029	0.003819	-0.172378493	0.136947	0.131313998	0.511403	C5	-	Y	-	-	Y	-	-
ENSG00000186001	LRCH3	0.473333953	9.18E-12	0.223465056	0.012354	0.28761255	0.000328	-0.243787985	4.06E-05	-0.171269475	0.003764	0.067160444	0.562859	C5	-	Y	-	-	-	-	-
ENSG00000186017	ZNF566	0.318558899	0.005191	-0.415420974	0.000782	-0.139493096	0.383377	-0.727613324	1.16E-13	-0.443867006	3.45E-05	0.278704608	0.082351	C5	-	Y	Y	-	Y	Y	-
ENSG00000186026	ZNF284	0.485578772	0.007785	0.088642458	0.730882	0.391073432	0.030997	-0.390061278	0.043526	-0.079929992	0.746625	0.305105344	0.215786	C5	-	Y	-	Y	Y	-	-
ENSG00000186063	AIDA	0.438325907	6.50E-10	0.247258812	0.004454	0.247518674	0.004063	-0.184493469	0.005138	-0.176018014	0.009376	0.003080615	0.988432	C1	-	Y	-	-	-	-	-
ENSG00000186073	C15orf41	-0.317997642	0.021808	-0.0535879	0.79461	0.028926956	0.88615	0.271599213	0.070301	0.36148594	0.009368	0.085295447	0.739574	C4	-	Y	-	-	-	Y	-
ENSG00000186088	GSAP	-1.05113822	7.97E-13	-0.520568211	0.002086	-0.445010241	0.003663	0.539307102	0.00526	0.620701925	0.000403	0.077036909	0.848238	C3	-	Y	Y	Y	Y	Y	-
ENSG00000186106	ANKRD46	0.498400578	6.85E-10	0.229871481	0.017648	0.318976961	0.000326	-0.262167153	0.00128	-0.164728087	0.05733	0.091906405	0.522204	C5	-	Y	-	Y	-	-	-
ENSG00000186111	PIP5K1C	0.404323616	3.84E-09	0.325255499	5.77E-05	0.380217323	1.61E-07	-0.072779593	0.363349	-0.009468088	0.919461	0.057983595	0.649547	C1	-	Y	Y	Y	-	-	-
ENSG00000186130	ZBTB6	0.231288255	0.000958	-0.187769154	0.055097	0.033143983	0.79572	-0.412446402	2.77E-08	-0.183525328	0.018394	0.223869184	0.059027	C5	-	-	-	-	Y	-	-
ENSG00000186132	C2orf76	-0.429539445	0.019429	-0.114436583	0.67618	-0.163071303	0.465285	0.321683044	0.146016	0.281155542	0.19221	-0.045642281	0.927911	C4	-	Y	-	-	-	-	-
ENSG00000186162	CIDECP1	0.382462864	0.032559	0.203320086	0.421481	0.03489225	0.910443	-0.172739843	0.444204	-0.332835906	0.098027	-0.165342108	0.656395	C5	-	Y	-	-	-	-	-
ENSG00000186174	BCL9L	0.613923491	3.04E-07	0.497626174	0.000181	0.704467285	1.70E-07	-0.109886821	0.450528	0.105174845	0.497897	0.209867115	0.294251	C1	-	Y	Y	Y	-	-	-
ENSG00000186205	MTARC1	-0.371043301	0.000307	-0.322652789	0.002097	-0.208350629	0.06548	0.05431271	0.715302	0.177587785	0.144372	0.117504108	0.51965	C4	-	Y	Y	-	-	-	-
ENSG00000186230	ZNF749	0.337762553	0.012831	-0.022063358	0.935333	0.17174897	0.292264	-0.352733291	0.007256	-0.151984015	0.22524	0.195381455	0.328007	C4	-	Y	-	-	Y	-	-
ENSG00000186231	KLHL32	-1.410686847	0.08422	0.219020985	0.813518	-0.310588877	0.718727	1.637096547	0.029773	1.114063817	0.202959	-0.525960155	NA	C3	-	-	-	-	Y	-	-
ENSG00000186280	KDM4D	-0.610016596	0.002798	-0.271787723	0.306135	-0.439181301	0.057735	0.345655647	0.166423	0.185474961	0.510189	-0.165129072	0.706114	C4	-	Y	-	-	-	-	-
ENSG00000186281	GPAT2	-2.0030401	0.031309	-0.634873031	NA	-0.852305392	NA	1.364102287	0.276939	1.16378625	0.362123	-0.211573847	NA	C3	-	Y	-	-	-	-	-
ENSG00000186298	PPP1CC	0.333528705	3.75E-15	0.236935311	1.17E-07	0.110484174	0.051143	-0.090097324	0.022951	-0.208310289	1.67E-07	-0.123526829	0.022852	C5	-	Y	-	-	-	-	-
ENSG00000186300	ZNF555	0.471626681	0.000122	0.053788377	0.823625	0.336517171	0.032906	-0.411388033	0.000578	-0.120278249	0.398223	0.285559203	0.137224	C5	-	Y	-	Y	Y	-	-
ENSG00000186301	MST1P2	-1.150229351	0.027906	-0.2401055	0.735453	-0.249384365	0.640784	0.917633539	0.118176	0.916242156	0.066922	-0.005514141	NA	C3	-	Y	-	-	-	-	-
ENSG00000186310	NAP1L3	-0.813819763	6.39E-07	-0.219747557	0.271422	-0.519723432	0.001507	0.599448663	0.001193	0.308609937	0.143305	-0.296755337	0.240869	C3	-	Y	-	Y	Y	-	-
ENSG00000186314	PRELID2	-1.110562964	2.43E-06	-0.157359022	0.620112	-0.16254125	0.603322	0.96033558	0.000105	0.962481759	0.000183	-0.002336709	0.997282	C3	-	Y	-	-	Y	Y	-
ENSG00000186318	BACE1	0.318836471	8.79E-11	0.237480133	3.97E-05	0.184523747	0.00999	-0.074838227	0.174023	-0.119527071	0.050139	-0.049944452	0.67488	C5	Y	Y	-	-	-	-	-
ENSG00000186350	RXRA	-0.371001412	1.25E-06	-0.13649521	0.232296	-0.038841172	0.726832	0.240737924	0.010317	0.346896162	6.03E-06	0.100363453	0.507382	C4	-	Y	-	-	-	Y	-
ENSG00000186376	ZNF75D	0.491566348	2.20E-05	0.146593926	0.479378	0.256569614	0.0691	-0.338727413	0.009961	-0.220538116	0.056755	0.113036326	0.652484	C5	-	Y	-	-	Y	-	-
ENSG00000186395	KRT10	-0.784940916	4.03E-07	-0.364550121	0.070965	-0.556059972	0.001465	0.426847488	0.009514	0.244076971	0.165134	-0.188274886	0.512021	C4	-	Y	-	Y	Y	-	-
ENSG00000186416	NKRF	-0.002580566	0.985592	-0.329941422	0.003193	-0.214815563	0.073243	-0.320716906	0.000301	-0.198233289	0.040935	0.117514491	0.488578	C4	-	-	Y	-	Y	-	-
ENSG00000186432	KPNA4	0.31456485	1.21E-07	0.045414876	0.634388	0.058652475	0.508111	-0.262703787	3.55E-06	-0.241112387	3.23E-05	0.016185409	0.920276	C5	-	Y	-	-	-	-	-
ENSG00000186448	ZNF197	0.24575633	0.001099	-0.089685608	0.412825	0.049209803	0.670704	-0.329082861	2.18E-07	-0.181908333	0.006122	0.141741872	0.157294	C5	-	-	-	-	Y	-	-
ENSG00000186469	GNG2	-0.523672798	1.52E-17	-0.272968354	1.00E-05	-0.180237958	0.01852	0.257262637	5.56E-05	0.358228781	1.38E-07	0.09558875	0.39019	C4	-	Y	-	-	-	Y	-
ENSG00000186470	BTN3A2	-0.534391645	3.25E-07	-0.238264468	0.035962	-0.092850935	0.476113	0.303013982	0.008191	0.456439939	1.59E-05	0.14841839	0.332868	C4	-	Y	-	-	Y	Y	-
ENSG00000186472	PCLO	-0.298393096	0.151068	0.05488722	0.799772	0.251932274	0.063474	0.359133112	0.000737	0.564306384	1.60E-10	0.200446384	0.126415	C4	-	-	-	-	Y	Y	-
ENSG00000186480	INSIG1	-0.8231709	1.65E-24	-0.217684556	0.055697	-0.230186505	0.005612	0.611378117	1.61E-09	0.607792911	7.23E-13	-0.009430036	0.97312	C3	-	Y	-	-	Y	Y	-
ENSG00000186487	MYT1L	-0.203434541	0.003547	0.024593201	0.843247	0.17747871	0.015686	0.234295739	7.23E-05	0.395516368	2.59E-15	0.156045529	0.033276	C4	-	-	-	-	-	Y	-
ENSG00000186496	ZNF396	-0.884105952	0.004103	-0.854236718	0.010534	-0.237459569	0.579013	0.035296278	0.943622	0.661202638	0.050709	0.619984207	NA	C4	-	Y	Y	-	-	-	-
ENSG00000186510	CLCNKA	-0.905130189	0.00113	-0.419187355	0.306362	-0.025315523	0.953102	0.495853479	0.232199	0.894290819	0.002861	0.395635593	NA	C4	-	Y	-	-	-	Y	-
ENSG00000186517	ARHGAP30	-2.14257415	9.80E-10	-0.687980448	0.054405	-0.85205354	0.014443	1.468268757	0.001403	1.304040039	0.00733	-0.16685327	NA	C3	-	Y	-	Y	Y	Y	-
ENSG00000186523	FAM86B1	-0.970083165	0.004949	-0.130372244	0.77208	-0.02621922	0.956889	0.848401195	0.025698	0.95870018	0.009578	0.106401541	NA	C2	-	Y	-	-	Y	Y	-
ENSG00000186575	NF2	0.473975114	6.01E-07	0.172016603	0.145739	0.077367993	0.576052	-0.295649667	0.000789	-0.382154115	1.24E-05	-0.091914731	0.557664	C5	-	Y	-	-	-	Y	-
ENSG00000186591	UBE2H	0.347627632	9.54E-11	0.224603934	0.000313	0.258627516	1.26E-06	-0.116655803	0.03309	-0.074301773	0.14499	0.037159869	0.701775	C5	Y	Y	-	-	-	-	-
ENSG00000186603	HPDL	-1.062861741	0.017158	-0.747576602	0.11653	-1.061676876	0.043342	0.322132075	0.569061	0.016308675	0.981325	-0.311043121	NA	C3	-	Y	-	Y	-	-	-
ENSG00000186615	KTN1-AS1	-0.177338356	0.455751	0.13976172	0.676376	0.233468936	0.34872	0.323959019	0.188632	0.425706216	0.043813	0.096263746	0.843922	C4	-	-	-	-	-	Y	-
ENSG00000186635	ARAP1	-0.398081064	1.53E-09	-0.108749633	0.20466	0.037158794	0.716415	0.295709337	2.20E-05	0.449895189	2.10E-11	0.148770739	0.132933	C4	-	Y	-	-	-	Y	-
ENSG00000186642	PDE2A	-0.43069554	0.002807	-0.047772453	0.805639	0.04738712	0.801677	0.38899826	0.009777	0.492767957	0.000916	0.098169431	0.682921	C2	-	Y	-	-	Y	Y	-
ENSG00000186654	PRR5	-0.523934442	0.003596	-0.638207959	0.001425	-0.466034072	0.028285	-0.108022252	0.669297	0.072261373	0.791801	0.174764151	0.644363	C4	-	Y	Y	Y	-	-	-
ENSG00000186665	C17orf58	-0.436768678	3.97E-05	-0.134304936	0.342438	-0.267641872	0.027891	0.308999854	0.011415	0.183807574	0.180376	-0.130476671	0.52512	C4	-	Y	-	-	Y	-	-
ENSG00000186732	MPPED1	-0.702670055	0.026758	-0.707812939	0.071303	-0.098706685	0.835849	0.003241621	0.994598	0.618675093	0.080017	0.61114093	NA	C4	-	Y	-	-	-	-	-
ENSG00000186767	SPIN4	0.173808804	0.094298	-0.135350152	0.28585	-0.046852863	0.73819	-0.302693308	0.000356	-0.206170372	0.015575	0.090807533	0.527723	C5	-	-	-	-	Y	-	-
ENSG00000186787	SPIN2B	0.408530356	0.036411	-0.067596341	0.83899	0.09306509	0.71424	-0.468632273	0.028592	-0.300658674	0.13677	0.163744075	0.652484	C5	-	Y	-	-	Y	-	-

ENSG00000186806	VSIG10L	-0.422461407	0.087277	0.064942675	0.835311	-0.096394491	0.771006	0.493944543	0.041088	0.340414162	0.241884	-0.158156923	0.711528	C2	-	-	-	-	Y	-	-
ENSG00000186814	ZSCAN30	-0.175945705	0.087005	0.001567345	0.993784	0.208210409	0.057506	0.183513434	0.030214	0.398825335	1.99E-07	0.209819362	0.051179	C4	-	-	-	-	-	Y	-
ENSG00000186815	TPCN1	-0.336125544	0.000347	-0.010006894	0.952806	0.096544325	0.399651	0.33239304	0.000212	0.447160521	1.52E-07	0.109145428	0.431094	C4	-	Y	-	-	Y	Y	-
ENSG00000186862	PDZD7	-0.712737382	0.000431	0.046079628	0.887253	-0.022820508	0.943516	0.76625675	0.000223	0.704643889	0.000896	-0.066203965	0.887679	C2	-	Y	-	-	Y	Y	-
ENSG00000186868	MAPT	-0.128252951	0.100397	-0.052258992	0.663449	0.183822567	0.023305	0.082538596	0.366724	0.326915401	1.70E-06	0.239141528	0.009564	C4	-	-	-	-	-	Y	-
ENSG00000186889	TMEM17	-1.021931882	1.26E-07	-0.203769023	0.389136	-0.315908987	0.144762	0.824143738	4.50E-05	0.72092846	0.000838	-0.109130647	0.778793	C2	-	Y	-	-	Y	Y	-
ENSG00000186897	C1QL4	-1.312649836	4.29E-07	-1.057983768	9.28E-05	-0.834815615	0.001398	0.261984915	0.539019	0.492679389	0.172371	0.225614911	NA	C3	-	Y	Y	Y	-	-	-
ENSG00000186907	RTN4RL2	-0.756874889	0.046955	-0.360877225	0.49071	-0.377725226	0.362146	0.407190319	0.44519	0.394189514	0.399223	-0.014195289	NA	C3	-	Y	-	-	-	-	-
ENSG00000186951	PPARA	0.465262284	1.57E-06	0.205686331	0.096746	0.406709041	0.000125	-0.254022429	0.000687	-0.044702867	0.654952	0.203954762	0.048933	C5	-	Y	-	Y	-	-	-
ENSG00000186952	TMEM232	-1.270501212	0.000523	0.001733966	0.997458	0.102845452	0.833666	1.278895109	0.001074	1.388082086	0.000412	0.10381588	NA	C3	-	Y	-	-	Y	Y	-
ENSG00000187013	LINC02875	-1.206976305	0.007283	-0.348964437	0.511043	-0.358699519	0.519302	0.865044661	0.076529	0.862613288	0.10599	-0.007356573	NA	C3	-	Y	-	-	-	-	-
ENSG00000187045	TMPRSS6	0.997139923	0.002564	0.238668364	0.691668	0.124877985	0.83678	-0.751915665	0.033459	-0.857370796	0.013524	-0.111043183	NA	C5	-	Y	-	-	Y	Y	-
ENSG00000187091	PLCD1	-0.580594302	0.000113	-0.057391977	0.806965	-0.060147111	0.79804	0.529631996	0.000215	0.535249216	0.000497	0.000183693	0.99962	C4	-	Y	-	-	Y	Y	-
ENSG00000187097	ENTPD5	-0.304642239	0.008406	0.164659441	0.269141	0.06780814	0.7004	0.476301493	8.94E-05	0.387106684	0.002554	-0.094653058	0.70884	C4	-	Y	-	-	Y	Y	-
ENSG00000187118	CMC1	-0.448091026	0.000645	-0.109388159	0.516834	0.036610516	0.83963	0.345078146	0.006107	0.499578318	1.71E-05	0.148587827	0.36932	C4	-	Y	-	-	Y	Y	-
ENSG00000187122	SLIT1	-0.33984011	0.000774	-0.507813057	8.28E-06	-0.313755584	0.004003	-0.161579443	0.171913	0.040784259	0.758914	0.197041452	0.1995	C4	-	Y	Y	Y	-	-	-
ENSG00000187123	LYPD6	-0.064911338	0.676223	0.01490632	0.950782	0.249438503	0.054311	0.086500871	0.569061	0.328848827	0.00262	0.236658242	0.133549	C4	-	-	-	-	-	Y	-
ENSG00000187187	ZNF546	0.353822335	0.068061	0.284706442	0.172541	0.441659006	0.020721	-0.06282424	0.803304	0.102394223	0.66997	0.159698	0.617822	C4	-	-	-	Y	-	-	-
ENSG00000187193	MT1X	-0.816342165	0.001154	-0.475166198	0.104219	-0.396733861	0.157943	0.347885188	0.26221	0.435117181	0.132414	0.081432863	0.895032	C4	-	Y	-	-	-	-	-
ENSG00000187243	MAGED4B	0.297181067	0.709752	1.592873224	0.038628	0.798957706	0.254749	1.303852793	0.061478	0.517116322	0.452398	-0.791447453	NA	C4	-	-	Y	-	-	-	-
ENSG00000187257	RSBN1L	0.260709118	1.97E-05	-0.102627438	0.297303	0.02455836	0.813051	-0.356793518	1.77E-10	-0.221584427	4.46E-06	0.129973324	0.139879	C5	-	-	-	-	Y	-	-
ENSG00000187260	WDR86	-0.161076692	0.125445	0.137522409	0.241262	0.148186165	0.198943	0.304992099	0.000366	0.324016121	0.000283	0.013567615	0.952689	C4	-	-	-	-	Y	Y	-
ENSG00000187391	MAGI2	-0.489677906	4.40E-07	-0.077661721	0.624786	-0.298398785	0.005581	0.418556761	3.06E-05	0.20625843	0.050137	-0.217336241	0.11381	C4	-	Y	-	-	Y	-	-
ENSG00000187479	C11orf96	-0.259730437	0.006025	0.118268575	0.592508	-0.187924862	0.084899	0.385329331	2.30E-08	0.086951643	0.13066	-0.303811444	0.000399	C4	-	-	-	-	Y	-	Y
ENSG00000187486	KCNJ11	-1.575406917	0.007318	-0.556202117	0.398712	-0.70046977	0.264113	1.025962917	0.127448	0.890142939	0.221129	-0.141006689	NA	C3	-	Y	-	-	-	-	-
ENSG00000187498	COL4A1	-0.191747163	0.01677	0.170642086	0.151865	0.196658243	0.029677	0.368547535	1.62E-05	0.40313592	7.09E-09	0.029004673	0.899704	C4	-	-	-	-	Y	Y	-
ENSG00000187514	PTMA	-0.180001627	3.13E-06	-0.250309447	6.87E-07	-0.321623453	1.99E-11	-0.063679199	0.059575	-0.126808863	2.99E-05	-0.068372307	0.296844	C4	Y	-	-	Y	-	-	-
ENSG00000187522	HSPA14	0.434628587	2.94E-07	-0.188674175	0.152184	-0.197146965	0.07838	-0.616577103	1.98E-11	-0.617147415	2.34E-13	-0.005680403	0.985228	C5	-	Y	-	-	Y	Y	-
ENSG00000187583	PLEKHN1	-0.814379592	0.124849	0.549857708	0.343679	0.472023238	0.371694	1.37364401	0.007642	1.301118002	0.008242	-0.074965575	NA	C2	-	-	-	-	Y	Y	-
ENSG00000187601	MAGEH1	-0.180207396	0.174456	-0.368662203	0.011259	-0.180484034	0.197378	-0.182194201	0.230173	0.014630177	0.934252	0.191129446	0.354246	C4	-	-	Y	-	-	-	-
ENSG00000187607	ZNF286A	0.074455645	0.403839	-0.263852504	0.003087	-0.0780005	0.457484	-0.331872359	3.49E-06	-0.137940185	0.070085	0.188578305	0.068615	C5	-	-	-	-	Y	-	-
ENSG00000187616	MYMK	-2.714576358	0.00623	-0.429510432	NA	-0.874612771	0.342374	2.285444728	0.042704	1.850915421	0.155348	-0.441333476	NA	C3	-	Y	-	-	Y	-	-
ENSG00000187626	ZKSCAN4	0.229530298	0.068587	-0.32982807	0.031137	0.038786086	0.856978	-0.553213297	8.17E-06	-0.176579985	0.218474	0.371506928	0.04521	C5	-	-	Y	-	Y	-	Y
ENSG00000187630	DHRS4L2	-0.531370189	0.035536	-0.257388279	0.41618	-0.134881215	0.694008	0.280416181	0.351051	0.411739898	0.143057	0.125640728	0.812315	C4	-	Y	-	-	-	-	-
ENSG00000187634	SAMD11	0.290396748	0.00098	0.238006959	0.009377	-0.087579959	0.408524	-0.045647465	0.636515	-0.363004544	8.47E-08	-0.322489419	2.41E-06	C4	-	-	-	-	-	Y	Y
ENSG00000187672	ERC2	-0.427953967	0.04988	-0.092129456	0.770887	-0.150178119	0.578004	0.341834542	0.163105	0.292254416	0.246263	-0.055003017	0.915603	C2	-	Y	-	-	-	-	-
ENSG00000187676	B3GLCT	-0.339760385	0.001299	0.021493527	0.904799	-0.191686526	0.116444	0.367431113	0.000185	0.162388501	0.156674	-0.210252035	0.119129	C4	-	Y	-	-	Y	-	-
ENSG00000187714	SLC18A3	-0.753744929	3.27E-13	-0.512576684	2.42E-05	-0.378425595	0.000698	0.247760358	0.058042	0.39011774	0.00046	0.137044286	0.499035	C4	-	Y	Y	Y	-	Y	-
ENSG00000187720	THSD4	-0.09511717	0.872149	1.855964524	1.22E-07	1.878873957	5.50E-09	1.956679347	5.25E-10	1.988941619	2.07E-11	0.027252401	0.973837	C2	-	-	Y	Y	Y	Y	-
ENSG00000187730	GABRD	-0.519510492	0.172451	-1.394500118	0.001829	-0.92712654	0.023015	-0.866652141	0.077173	-0.393097388	0.423968	0.467088494	NA	C3	-	-	Y	Y	-	-	-
ENSG00000187736	NHEJ1	0.360458219	0.003265	0.003074757	0.991538	0.195096055	0.183906	-0.351437367	0.004815	-0.151205529	0.200172	0.195383034	0.306197	C5	-	Y	-	-	Y	-	-
ENSG00000187790	FANCM	0.758282727	9.02E-08	0.216275858	0.312013	0.43440217	0.00929	-0.536419387	8.05E-05	-0.309892428	0.024777	0.221465362	0.32098	C1	-	Y	-	Y	Y	Y	-
ENSG00000187792	ZNF70	0.455720639	1.57E-05	0.097016126	0.558172	0.425965824	0.00048	-0.352558505	0.000744	-0.014955738	0.926262	0.331830884	0.018074	C5	-	Y	-	Y	Y	-	Y
ENSG00000187796	CARD9	-0.43827575	1.90E-07	-0.140791851	0.285412	0.000443805	0.997172	0.303796792	0.002544	0.453425892	2.23E-08	0.144004443	0.343347	C4	-	Y	-	-	Y	Y	-
ENSG00000187800	PEAR1	-0.87840051	1.80E-14	-0.065832658	0.71909	-0.265165085	0.048201	0.81914899	5.02E-15	0.628109334	1.85E-08	-0.196562646	0.209913	C2	-	Y	-	-	Y	Y	-
ENSG00000187824	TMEM220	-1.088841814	4.89E-07	-0.553730933	0.026062	-0.571527498	0.008469	0.540942489	0.062719	0.532783364	0.049635	-0.013696835	0.983166	C4	-	Y	Y	Y	-	Y	-
ENSG00000187837	H1-2	-0.31510553	0.050545	0.239269161	0.168434	-0.13722083	0.627016	0.561334938	0.00011	0.193389964	0.415969	-0.37521982	0.168713	C2	-	-	-	-	Y	-	-
ENSG00000187840	EIF4EBP1	-0.267967002	0.001874	-0.311374056	0.009019	-0.179263903	0.089924	-0.036250921	0.756007	0.103448347	0.162305	0.134677555	0.361022	C4	-	-	Y	-	-	-	-
ENSG00000187866	FAM122A	0.559663459	9.55E-08	-0.063630499	0.73591	0.240618008	0.060581	-0.616921558	9.81E-09	-0.304246614	0.005592	0.307082494	0.03989	C5	-	Y	-	-	Y	Y	Y
ENSG00000187902	SHISA7	-0.630681112	0.015957	-0.599010528	0.037947	-0.066310772	0.851431	0.039709125	0.931468	0.578950661	0.040483	0.535367317	NA	C4	-	Y	Y	-	-	Y	-
ENSG00000187942	LDLRAD2	-1.763359024	0.03246	-1.233505803	NA	-0.710495587	0.425752	0.545585489	0.697541	1.066841153	0.287162	0.522773457	NA	C3	-	Y	-	-	-	-	-
ENSG00000188010	MORN2	-0.297612642	0.024821	-0.27313972	0.081882	-0.339454619	0.019063	0.03122504	0.838538	-0.026569474	0.867421	-0.063353929	0.78571	C4	-	-	-	Y	-	-	-
ENSG00000188026	RILPL1	0.544988686	7.04E-06	0.385848104	0.012151	0.389387283	0.00334	-0.15231896	0.345404	-0.140844569	0.335204	0.006350197	0.986686	C5	-	Y	Y	Y	-	-	-
ENSG00000188033	ZNF490	0.698997925	0.000548	0.128872989	0.738568	0.254682456	0.423003	-0.563439554	0.00931	-0.429462259	0.057404	0.128722053	0.811549	C5	-	Y	-	-	Y	-	-
ENSG00000188060	RAB42	-1.16702042	0.000596	-0.700667031	0.087206	-0.36242355	0.374048	0.475910337	0.320071	0.819407816	0.037279	0.339974896	NA	C4	-	Y	-	-	-	Y	-
ENSG00000188092	GPR89B	1.056171335	2.08E-08	0.541725763	0.011111	0.547289998	0.008679	-0.509313375	0.123148	-0.494509328	0.153336	0.0081294	0.989727	C5	-	Y	Y	Y	-	-	-
ENSG00000188133	TMEM215	-1.52596651	6.74E-06	-0.208812198	0.626419	-0.70475198	0.033127	1.321379852	0.000367	0.836147158	0.0481	-0.492591563	NA	C3	-	Y	-	Y	Y	Y	-
ENSG00000188167	TMPPE	1.057154655	0.002513	0.839425386	0.049809	0.950445911	0.016588	-0.210456554	0.597006	-0.091885372	0.834208	0.1134642	NA	C1	-	Y	Y	Y	-	-	-
ENSG00000188177	ZC3H6	-0.296992347	0.003763	-0.385508421	0.000326	0.073628816	0.589782	-0.081850556	0.549934	0.385206559	0.000108	0.462005251	2.71E-05	C4	-	-	Y	-	-	Y	Y
ENSG00000188186	LAMTOR4	-0.306730069	0.000364	-0.116198931	0.372452	-0.153334202	0.18728	0.196864926	0.0105	0.168534551	0.030882	-0.033895646	0.863083	C4	-	Y	-	-	-	-	-
ENSG00000188199	NUTM2B	-1.627555823	0.001984	-0.905508226	0.138486	-0.925329202	0.162251	0.73030585	0.350878	0.717673423	0.402833	-0.01699944	NA	C3	-	Y	-	-	-	-	-
ENSG00000188211	NCR3LG1	-0.251481436	0.08338	0.114977562	0.585186	0.10592094	0.579387	0.372185531	0.00179	0.371694237	0.000992	-0.005762114	0.986496	C4	-	-	-	-	Y	Y	-
ENSG00000188215	DCUN1D3	0.331037529	0.018739	-0.405733395	0.008645	-0.015478069	0.950834	-0.730529616	5.95E-08	-0.331790619	0.026155	0.393148399	0.042023	C5	-	Y	Y	-	Y	Y	Y
ENSG00000188243	COMMD6	-0.352222247	0.004554	-0.289621111	0.074324	-0.100511374	0.574917	0.068963506	0.712529	0.266366748	0.050729	0.192097577	0.414349	C4	-	Y	-	-	-	-	-
ENSG00000188277	C15orf62	0.848679394	0.008165	0.133364366	0.806241	0.210527833	0.682623	-0.710534048	0.008512	-0.623750003	0.039798	0.08061012	NA	C5	-	Y	-	-	Y	Y	-
ENSG00000188283	ZNF383	0.354669674	0.007259	-0.124300177	0.558494	0.063619626	0.762108	-0.472447695	0.000812	-0.276542199	0.053735	0.190839319	0.45687	C4	-	Y	-	-	Y	-	-
ENSG00000188290	HES4	-0.265333142	0.164851	0.090918528	0.763207	0.130986417	0.587026	0.362444247	0.017436	0.410926102	0.002455	0.043294185	0.905466	C4	-	-	-	-	Y	Y	-

ENSG00000188315	C3orf62	0.118734491	0.475804	-0.056199315	0.806916	-0.227887296	0.138738	-0.168579096	0.312745	-0.331625179	0.019435	-0.168805334	0.46238	C5	-	-	-	-	-	Y	-
ENSG00000188322	SBK1	0.382280332	5.88E-12	0.122966273	0.113958	0.065490055	0.372631	-0.252967719	2.42E-05	-0.302189295	6.95E-09	-0.054641306	0.643062	C5	-	Y	-	-	-	Y	-
ENSG00000188338	SLC38A3	-1.152147403	0.000128	-0.475189078	0.157791	-0.003013495	0.994131	0.681146388	0.073157	1.163495046	0.00019	0.474899679	NA	C2	-	Y	-	-	-	Y	-
ENSG00000188352	FOCAD	-0.239529171	0.001904	0.0872194	0.446666	0.116116554	0.174595	0.333117657	1.01E-05	0.3702708	1.48E-08	0.031551022	0.852721	C4	-	-	-	-	Y	Y	-
ENSG00000188385	JAKMIP3	-0.330124286	0.086484	0.133526365	0.61478	0.52199038	0.000942	0.470230606	0.013205	0.866681172	1.02E-08	0.392558435	0.053244	C4	-	-	-	Y	Y	Y	-
ENSG00000188428	BLOC1S5	0.514858474	1.10E-05	0.067948261	0.740277	0.110238693	0.531402	-0.441004396	2.56E-06	-0.390269181	4.28E-05	0.045281991	0.849659	C5	-	Y	-	-	Y	Y	-
ENSG00000188483	IER5L	0.329753411	0.095482	-0.133549746	0.689634	0.240048791	0.322082	-0.45672515	0.011688	-0.074480195	0.727435	0.376582245	0.13614	C5	-	-	-	-	Y	-	-
ENSG00000188486	H2AX	-0.311867395	1.79E-06	-0.179806075	0.073134	-0.203887189	0.050074	0.138721792	0.088854	0.122632804	0.169426	-0.021166045	0.935209	C4	-	Y	-	-	-	-	-
ENSG00000188488	SERPINA5	-0.705826258	4.66E-10	-0.133722636	0.394003	-0.067086592	0.681009	0.579399721	6.29E-06	0.653375539	7.22E-08	0.069263889	0.794384	C4	Y	Y	-	-	Y	Y	-
ENSG00000188493	C19orf54	-0.440023911	0.000275	-0.258924413	0.060663	-0.337561391	0.011786	0.187885493	0.174022	0.117442922	0.450342	-0.075524037	0.766211	C4	-	Y	-	Y	-	-	-
ENSG00000188542	DUSP28	-0.308428018	0.143736	0.098118664	0.723781	-1.97E-05	0.999916	0.412976192	0.049215	0.323698395	0.222229	-0.094880268	0.851731	C2	-	-	-	-	Y	-	-
ENSG00000188549	CCDC9B	-0.761072908	9.25E-12	-0.203766289	0.135352	-0.030195278	0.891808	0.564099781	2.72E-06	0.745465146	2.59E-08	0.175562018	0.445857	C4	-	Y	-	-	Y	Y	-
ENSG00000188566	NDOR1	0.366453951	2.88E-05	0.120047401	0.34472	0.018201167	0.901354	-0.239786722	0.00981	-0.333440845	4.39E-05	-0.099146124	0.524775	C5	-	Y	-	-	-	Y	-
ENSG00000188573	FBLL1	-0.232723793	0.040645	-0.359278661	0.020165	-0.28527193	0.021897	-0.120136326	0.480318	-0.037739789	0.818968	0.077452493	0.80241	C5	-	-	Y	-	-	-	-
ENSG00000188599	NPIPP1	-0.01749199	0.95499	-0.540737731	0.023418	-0.150695391	0.589194	-0.516731055	0.013429	-0.118199004	0.636276	0.393340217	0.18621	C5	-	-	Y	-	Y	-	-
ENSG00000188613	NANOS1	-0.343849787	0.008155	-0.024884438	0.909425	-0.196854506	0.233382	0.32535254	0.014623	0.161633108	0.3201	-0.168991892	0.454595	C4	-	Y	-	-	Y	-	-
ENSG00000188643	S100A16	1.323753112	0.054573	1.907157366	0.000553	1.949200835	0.000229	0.59032315	0.307556	0.640080769	0.246761	0.046742689	NA	C2	-	-	Y	Y	-	-	-
ENSG00000188647	PTAR1	0.51578812	1.76E-11	0.054072375	0.718023	0.270101642	0.006874	-0.455363661	1.39E-13	-0.231016134	0.000247	0.218838092	0.036158	C5	-	Y	-	-	Y	-	-
ENSG00000188659	SAXO2	-0.530897379	0.220144	0.134694616	0.798593	0.262963053	0.523104	0.672239657	0.113493	0.808309767	0.037959	0.131352036	NA	C3	-	-	-	-	-	Y	-
ENSG00000188677	PARVB	-0.530297738	1.30E-07	-0.189046452	0.141257	-0.136259112	0.281872	0.347774183	0.001052	0.409121467	5.35E-05	0.055614809	0.796431	C4	-	Y	-	-	Y	Y	-
ENSG00000188681	TEKT4P2	0.860822819	1.69E-05	0.424210088	0.107329	0.384427843	0.152687	-0.430405581	0.035589	-0.461627988	0.028669	-0.036788035	0.948525	C5	-	Y	-	-	Y	Y	-
ENSG00000188687	SLC4A5	-0.888229768	0.008972	0.324635582	0.441183	-0.004133251	0.993811	1.220949005	0.000345	0.898681012	0.0144	-0.32688967	NA	C4	-	Y	-	-	Y	Y	-
ENSG00000188690	UROS	-0.301718814	0.003365	-0.006863628	0.971342	-0.05781336	0.693179	0.301693206	0.000409	0.25906748	0.002648	-0.048144321	0.778408	C4	-	Y	-	-	Y	-	-
ENSG00000188707	ZBED6CL	-0.696123205	3.11E-05	-0.038515396	0.8922	-0.316866592	0.075883	0.663407152	6.85E-05	0.393465094	0.024487	-0.275868912	0.225609	C3	-	Y	-	-	Y	Y	-
ENSG00000188710	QRFP	-1.398201996	0.019118	-0.266709344	0.749687	-0.296994668	0.690869	1.144686791	0.145909	1.118510581	0.143169	-0.027804352	NA	C3	-	Y	-	-	-	-	-
ENSG00000188732	FAM221A	0.251222786	0.000911	-0.065997985	0.574502	0.061474749	0.574529	-0.310778668	1.53E-05	-0.175150027	0.019617	0.130292463	0.241546	C5	-	-	-	-	Y	-	-
ENSG00000188735	TMEM120B	0.556998316	5.44E-11	0.373515464	0.000104	0.43476862	2.13E-07	-0.177128671	0.064627	-0.10752694	0.260701	0.063749444	0.701775	C5	-	Y	Y	Y	-	-	-
ENSG00000188747	NOXA1	-1.344660362	0.001856	-0.251293285	0.660755	-0.408202501	0.423289	1.095723995	0.03024	0.950582505	0.077048	-0.152290191	NA	C3	-	Y	-	-	Y	-	-
ENSG00000188766	SPRED3	0.718420655	1.28E-08	0.414497882	0.012636	0.424775351	0.001059	-0.297923607	0.038791	-0.279013369	0.020192	0.013546802	0.97109	C1	-	Y	Y	Y	-	-	-
ENSG00000188786	MTF1	0.128912141	0.173003	-0.347989052	0.011013	0.052848916	0.682382	-0.470263608	8.45E-06	-0.061634849	0.517058	0.403394038	0.002869	C5	-	-	Y	-	Y	-	Y
ENSG00000188825	LINC00910	-1.205626953	3.91E-08	-0.099998973	0.806329	-0.155104228	0.580146	1.114277188	4.23E-05	1.065167986	5.71E-06	-0.053101937	0.936869	C2	-	Y	-	-	Y	Y	-
ENSG00000188827	SLX4	0.655166482	6.91E-12	0.390252489	0.00029	0.495365784	8.03E-08	-0.258697357	0.010179	-0.145228949	0.151596	0.107478087	0.503233	C5	-	Y	Y	Y	-	-	-
ENSG00000188833	ENTPD8	-1.341789727	0.010844	-0.996290765	0.092334	-0.505055052	0.388357	0.358819487	0.672102	0.85206797	0.196269	0.492382295	NA	C3	-	Y	-	-	-	-	-
ENSG00000188848	BEND4	0.43462295	4.22E-08	0.245480003	0.013271	0.329089272	9.00E-05	-0.183275835	0.001276	-0.091150338	0.076673	0.086878604	0.327637	C1	-	Y	-	Y	-	-	-
ENSG00000188856	RPSAP47	0.079791001	0.637327	-0.111154436	0.60877	0.27104013	0.088015	-0.184802494	0.134255	0.206443394	0.062051	0.38590553	0.006834	C4	-	-	-	-	-	-	Y
ENSG00000188859	FAM78B	-0.325052429	0.220142	-0.042411041	0.910528	0.135655008	0.660742	0.288202383	0.277506	0.474835981	0.046219	0.180598793	0.636064	C4	-	-	-	-	-	Y	-
ENSG00000188868	ZNF563	-0.570854123	0.098907	-0.446148361	0.284716	0.208248311	0.604549	0.132325411	0.793693	0.794092773	0.017631	0.657693501	NA	C4	-	-	-	-	-	Y	-
ENSG00000188878	FBF1	-0.240070296	0.219735	0.224028234	0.304836	0.212135589	0.375645	0.471104208	0.002373	0.466951189	0.007381	-0.009134941	0.983166	C4	-	-	-	-	Y	Y	-
ENSG00000188909	BSX	-4.762887593	0.000469	-1.24088791	NA	-1.793473173	NA	3.506667983	NA	2.996697107	NA	-0.523239008	NA	C3	-	Y	-	-	-	-	-
ENSG00000188917	TRMT2B	-0.465768652	0.000918	-0.106030691	0.600707	-0.345184819	0.014223	0.368054589	0.028304	0.135102174	0.476508	-0.237380004	0.303262	C2	-	Y	-	Y	Y	-	-
ENSG00000188921	HACD4	-0.44176995	0.421894	0.531633957	0.313135	0.140166035	0.836477	0.981110964	0.036241	0.597136146	0.280415	-0.388382308	NA	C2	-	-	-	-	Y	-	-
ENSG00000189042	ZNF567	-0.019346649	0.902999	-0.393596548	0.01528	0.012377924	0.948703	-0.367774052	0.0106	0.046525702	0.757617	0.408614371	0.024557	C5	-	-	Y	-	Y	-	Y
ENSG00000189056	RELN	0.408977395	0.039058	0.845873853	2.25E-06	0.837581255	1.06E-06	0.442904427	0.006397	0.442936325	0.00525	-0.004780399	0.991385	C1	-	Y	Y	Y	Y	Y	-
ENSG00000189057	FAM111B	-0.091298082	0.329311	-0.320629914	0.009383	-0.137044175	0.273214	-0.222528723	0.038979	-0.031534295	0.808048	0.187171488	0.321747	C5	-	-	Y	-	-	-	-
ENSG00000189060	H1-0	-1.004997033	8.47E-76	-0.210548914	0.006736	-0.216438112	0.001392	0.800942622	2.32E-75	0.803704746	5.61E-114	-0.002756963	0.985334	C2	-	Y	-	-	Y	Y	-
ENSG00000189079	ARID2	0.233392457	0.013962	-0.094617134	0.52493	-0.097267124	0.462929	-0.321696662	3.06E-07	-0.316468088	3.00E-08	3.80E-05	0.999689	C5	-	-	-	-	Y	Y	-
ENSG00000189114	BLOC1S3	0.168985532	0.328509	-0.330813198	0.13329	-0.207883519	0.348759	-0.493220482	0.006916	-0.362249974	0.045988	0.126241232	0.765371	C5	-	-	-	-	Y	Y	-
ENSG00000189143	CLDN4	-0.710650315	0.003295	-0.176769169	0.606043	0.181313318	0.523001	0.540348926	0.065149	0.906476954	0.000226	0.360873853	0.323182	C4	-	Y	-	-	-	Y	-
ENSG00000189144	ZNF573	0.921800296	3.83E-07	0.667345868	0.00331	0.602097115	0.005507	-0.247081533	0.180041	-0.304987184	0.077387	-0.063377179	0.882986	C1	-	Y	Y	Y	-	-	-
ENSG00000189149	CRYM-AS1	-1.355464717	0.030216	-0.775913852	0.235225	-1.02296189	0.09809	0.594467581	0.513094	0.350500516	0.734522	-0.247376376	NA	C3	-	Y	-	-	-	-	-
ENSG00000189157	FAM47E	-1.016292701	0.000186	-0.430337757	0.187203	-0.302903053	0.36308	0.593595204	0.074246	0.728291864	0.021783	0.129688671	NA	C2	-	Y	-	-	-	Y	-
ENSG00000189164	ZNF527	0.269879295	0.070111	-0.201120086	0.345379	-0.016491449	0.954255	-0.464908835	0.004601	-0.271262082	0.129461	0.188374264	0.570078	C5	-	-	-	-	Y	-	-
ENSG00000189184	PCDH18	-0.224859807	0.145143	0.16320892	0.415145	0.164954272	0.464586	0.393679507	0.004601	0.404903806	0.012745	0.003629763	0.994294	C4	-	-	-	-	Y	Y	-
ENSG00000189195	BTBD8	0.263282612	0.138638	0.440386275	0.013939	0.425446364	0.015969	0.183324083	0.312745	0.176468912	0.334	-0.012030225	0.97796	C4	-	-	Y	Y	-	-	-
ENSG00000189212	DPY19L2P1	-0.180698122	0.457769	0.122841433	0.692621	0.250384061	0.275999	0.310271191	0.195531	0.445749601	0.033293	0.130655476	0.73559	C4	-	-	-	-	-	Y	-
ENSG00000189221	MAOA	-0.412441759	5.35E-06	-0.249632856	0.030109	-0.200341487	0.114194	0.169307753	0.127754	0.227027324	0.047115	0.0525214	0.841093	C4	-	Y	-	-	-	-	-
ENSG00000189283	FHIT	-0.270046718	0.457408	-0.813007685	0.045684	-0.69050156	0.038874	-0.535514946	0.169459	-0.404636408	0.236819	0.126902701	NA	C3	-	-	Y	Y	-	-	-
ENSG00000189298	ZKSCAN3	0.185242384	0.13088	-0.210525983	0.153826	0.188671581	0.180629	-0.389357202	0.000607	0.017781909	0.91502	0.401924435	0.004114	C5	-	-	-	-	Y	-	Y
ENSG00000189308	LIN54	0.347735373	2.59E-06	0.153353858	0.136997	0.084773248	0.442834	-0.188064811	0.022927	-0.248388642	0.001509	-0.065673898	0.693852	C5	-	Y	-	-	-	-	-
ENSG00000189337	KAZN	-0.400341072	0.001093	-0.140522223	0.397843	-0.090506199	0.584945	0.26652555	0.051077	0.324287599	0.011097	0.052977798	0.851487	C4	-	Y	-	-	-	Y	-
ENSG00000189350	TOGARAM2	-1.626056753	1.99E-05	-1.130620436	0.00451	-0.630134379	0.093423	0.507908996	0.391881	1.010233626	0.030576	0.501293316	NA	C3	-	Y	Y	-	-	Y	-
ENSG00000189362	NEMP2	-0.374297179	0.008371	0.030231806	0.885134	-0.119267939	0.559018	0.41049818	0.002181	0.269694358	0.116671	-0.146302477	0.559361	C4	-	Y	-	-	Y	-	-
ENSG00000189366	ALG1L	-0.793645078	0.15501	0.355456349	0.562049	-0.439901195	0.532132	1.154526689	0.02156	0.368215845	0.630705	-0.792917366	NA	C2	-	-	-	-	Y	-	-
ENSG00000189367	KIAA0408	-0.644198263	0.030217	-0.319778648	0.271517	-0.193543424	0.513851	0.329862405	0.317548	0.464805635	0.117108	0.12877475	0.769798	C4	-	Y	-	-	-	-	-
ENSG00000189376	C8orf76	0.173430993	0.350978	-0.278199057	0.174597	-0.130451244	0.568438	-0.445042474	0.009325	-0.289021841	0.109399	0.150581417	0.638566	C4	-	-	-	-	Y	-	-
ENSG00000189403	HMGB1	-0.183574102	1.15E-07	-0.158986488	0.0009	-0.321983788	2.66E-13	0.031206163	0.465482	-0.123764162	0.000348	-0.160084348	0.001909	C4	-	-	-	Y	-	-	-
ENSG00000189419	SPATA41	-1.694403907	3.08E-05	-0.374655795	0.406821	-0.820228138	0.059573	1.326807085	0.003605	0.888998396	0.11556	-0.443245724	NA	C3	-	Y	-	-	Y	-	-

ENSG00000189423	USP32P3	0.624040223	0.008794	0.488507346	0.046686	0.528450127	0.074324	-0.129797545	0.654491	-0.080517591	0.825209	0.043888828	0.947133	C1	-	Y	Y	-	-	-	-
ENSG00000196074	SYCP2	-0.340334205	0.013619	0.01485593	0.953741	0.180420565	0.230704	0.361180933	0.016578	0.535411468	4.39E-05	0.168308714	0.454595	C4	-	Y	-	-	Y	Y	-
ENSG00000196092	PAX5	0.440419719	2.46E-05	0.287615675	0.020014	0.321787947	0.004837	-0.146939359	0.093259	-0.104545272	0.241505	0.036989197	0.843713	C1	-	Y	-	Y	-	-	-
ENSG00000196110	ZNF699	0.154494822	0.430065	-0.252971985	0.245011	0.002937936	0.992533	-0.401267515	0.008783	-0.137374482	0.457175	0.258599001	0.282846	C5	-	-	-	-	Y	-	-
ENSG00000196116	TDRD7	-0.189662326	0.303277	-0.017078771	0.957038	0.246750668	0.170478	0.179003417	0.339247	0.451434267	0.00171	0.268005961	0.213226	C4	-	-	-	-	-	Y	-
ENSG00000196139	AKR1C3	-1.099552447	0.001497	-0.815804104	0.033245	-0.928680515	0.015975	0.2912681	0.551383	0.186497177	0.736232	-0.110044145	NA	C4	-	Y	Y	Y	-	-	-
ENSG00000196143	OR11H13P	1.159313165	0.330831	-0.133673886	NA	-2.167767701	NA	-1.288895884	0.333502	-3.317345001	0.042	-2.033203752	NA	C5	-	-	-	-	-	Y	-
ENSG00000196150	ZNF250	0.150940561	0.149913	-0.2654657	0.022279	-0.067549602	0.67237	-0.410075444	2.12E-05	-0.204301998	0.064479	0.200917492	0.230243	C5	-	-	-	-	Y	-	-
ENSG00000196151	WDSUB1	0.288075449	0.056587	0.35153554	0.029003	0.363093612	0.016272	0.069886139	0.691479	0.089437635	0.590594	0.014095447	0.967582	C4	-	-	Y	Y	-	-	-
ENSG00000196152	ZNF79	0.128058493	0.48257	-0.290113055	0.124296	-0.031615097	0.907685	-0.411976721	0.018273	-0.144734573	0.494036	0.261830223	0.364794	C5	-	-	-	-	Y	-	-
ENSG00000196154	S100A4	-0.561939198	0.021439	-0.400700341	0.110995	-0.226722279	0.407091	0.168603522	0.60325	0.350122741	0.21534	0.176740022	0.67662	C4	-	Y	-	-	-	-	-
ENSG00000196155	PLEKHG4	-0.50350391	1.46E-13	-0.09946135	0.333252	-0.02189864	0.827838	0.4106812	6.52E-08	0.49623221	7.90E-16	0.080654332	0.522361	C4	-	Y	-	-	Y	Y	-
ENSG00000196177	ACADSB	-0.261349333	0.010777	0.058402567	0.638789	-0.044710189	0.70825	0.326366839	0.000362	0.231112978	0.013347	-0.099975694	0.401822	C4	-	-	-	-	Y	-	-
ENSG00000196182	STK40	0.523662839	2.99E-10	0.11183258	0.50719	0.23977781	0.03957	-0.405596902	7.39E-06	-0.269707947	0.000511	0.130898649	0.492723	C5	-	Y	-	-	Y	-	-
ENSG00000196187	TMEM63A	-0.752889334	0.006335	-0.2558062	0.417509	-0.194964185	0.541494	0.504101018	0.103588	0.572931781	0.05869	0.063757795	0.917776	C2	-	Y	-	-	-	-	-
ENSG00000196189	SEMA4A	-0.896188718	0.004373	-0.430004653	0.268407	-0.26060179	0.537998	0.471973981	0.245694	0.650172659	0.093944	0.172936199	NA	C3	-	Y	-	-	-	-	-
ENSG00000196214	ZNF766	-0.355676534	5.24E-05	-0.275202805	0.004575	-0.2918016	0.007677	0.086761852	0.454367	0.078563698	0.554962	-0.013692803	0.961034	C4	-	Y	-	-	-	-	-
ENSG00000196220	SRGAP3	-1.186767962	1.33E-13	-0.15102372	0.511709	0.019163679	0.943897	1.043101811	6.44E-09	1.220455529	1.39E-12	0.172568392	0.58134	C3	-	Y	-	-	Y	Y	-
ENSG00000196236	XPNPEP3	0.329845047	0.002543	0.109139082	0.513674	0.098706675	0.559968	-0.214351674	0.074708	-0.216341062	0.08559	-0.007683896	0.982152	C5	-	Y	-	-	-	-	-
ENSG00000196247	ZNF107	-0.255997624	0.005212	-0.426463062	3.75E-05	-0.335426608	0.000529	-0.16442638	0.06307	-0.065005515	0.48462	0.094110579	0.536705	C4	-	-	Y	Y	-	-	-
ENSG00000196263	ZNF471	-0.021195598	0.89574	0.015496793	0.947174	0.321783995	0.004163	0.043304094	0.790107	0.357523912	0.00028	0.308778184	0.02897	C4	-	-	-	Y	-	Y	Y
ENSG00000196267	ZNF836	-0.003995177	0.986546	-0.402943622	0.047204	-0.061037559	0.811268	-0.392343445	0.030983	-0.042086094	0.858391	0.34520283	0.157664	C5	-	-	Y	-	Y	-	-
ENSG00000196268	ZNF493	-0.250130533	0.046289	-0.133136044	0.512616	0.104197712	0.549054	0.123503603	0.456939	0.369435948	0.001872	0.240217269	0.248512	C4	-	-	-	-	-	Y	-
ENSG00000196275	GTF2IRD2	-0.413943023	0.000118	-0.134599836	0.378646	-0.417094364	0.000574	0.285859014	0.011202	0.011904467	0.944337	-0.279589307	0.072294	C4	-	Y	-	Y	-	-	-
ENSG00000196277	GRM7	-0.516381894	0.00683	0.244585178	0.273934	0.004896255	0.988069	0.766411056	1.04E-06	0.535653591	0.002733	-0.236338609	0.353885	C2	-	Y	-	-	Y	Y	-
ENSG00000196284	SUPT3H	-0.549998735	0.001004	-0.16786395	0.488358	0.164062034	0.40811	0.38839346	0.041024	0.728619338	3.23E-06	0.334761878	0.136143	C4	-	Y	-	-	Y	Y	-
ENSG00000196313	POM121	0.405575874	1.03E-10	0.269478982	6.72E-05	0.222582975	0.001091	-0.129549061	0.011118	-0.168619526	0.000636	-0.044448855	0.634454	C1	-	Y	-	-	-	-	-
ENSG00000196338	NLGN3	-0.557659182	1.17E-05	-0.282893897	0.030756	-0.01875083	0.922903	0.281906488	0.058968	0.553516021	3.18E-05	0.266776496	0.112319	C4	-	Y	-	-	-	Y	-
ENSG00000196345	ZKSCAN7	-0.207548726	0.028278	-0.335754194	0.003474	-0.078734498	0.519846	-0.121599044	0.347424	0.143572418	0.170104	0.259967977	0.069496	C4	-	-	Y	-	-	-	-
ENSG00000196358	NTNG2	-0.017753175	0.894643	0.53333303	1.62E-06	0.513987737	5.02E-05	0.558062519	5.64E-10	0.546276684	1.75E-07	-0.014677946	0.97796	C4	-	-	Y	Y	Y	Y	-
ENSG00000196366	C9orf163	-1.244876205	0.006323	-0.106146232	0.87795	-0.739550586	0.133302	1.145230408	0.022208	0.519297537	0.408477	-0.63133134	NA	C3	-	Y	-	-	Y	-	-
ENSG00000196367	TRRAP	0.459485695	1.52E-11	0.281522667	0.000428	0.267109339	0.000632	-0.171782546	9.48E-05	-0.178236826	3.36E-05	-0.011625401	0.93194	C5	-	Y	-	-	-	-	-
ENSG00000196369	SRGAP2B	0.541193133	0.000885	0.371673944	0.039507	0.113578932	0.641457	-0.162963479	0.344189	-0.41265051	0.009072	-0.254690326	0.250087	C1	-	Y	Y	-	-	Y	-
ENSG00000196381	ZNF781	-0.489976258	0.003169	0.017896466	0.948627	0.015337887	0.953074	0.513091382	0.003165	0.51995784	0.002724	0.000666526	0.998367	C2	-	Y	-	-	Y	Y	-
ENSG00000196391	ZNF774	0.850606132	8.22E-11	0.205353438	0.316103	0.654204351	5.79E-06	-0.639280238	1.38E-07	-0.182012439	0.167664	0.451777607	0.003511	C1	-	Y	-	Y	Y	-	Y
ENSG00000196417	ZNF765	0.199985637	0.108735	-0.177607535	0.313135	0.10735974	0.502147	-0.371057501	0.004808	-0.077974416	0.601686	0.287664029	0.108493	C4	-	-	-	-	Y	-	-
ENSG00000196418	ZNF124	-0.327430231	6.87E-05	-0.410085231	9.05E-05	-0.527277971	3.05E-08	-0.076189646	0.56372	-0.185212315	0.088315	-0.113453849	0.591359	C4	-	Y	Y	Y	-	-	-
ENSG00000196421	C20orf204	-1.346461279	1.09E-09	0.160983474	0.565978	-0.15269056	0.56896	1.513702763	5.55E-12	1.208423389	1.35E-07	-0.310930744	0.287144	C3	-	Y	-	-	Y	Y	-
ENSG00000196422	PPP1R26	0.32106613	1.50E-09	0.139342243	0.037468	0.205530806	0.001397	-0.175279145	0.001038	-0.100962343	0.091601	0.069047553	0.492604	C5	-	Y	-	-	-	-	-
ENSG00000196428	TSC22D2	0.465949412	9.19E-07	-0.146423809	0.309682	0.14178342	0.28497	-0.605687341	9.18E-12	-0.309482885	0.000673	0.290758389	0.022166	C5	-	Y	-	-	Y	Y	-
ENSG00000196437	ZNF569	0.432540724	1.49E-07	-0.006540861	0.971342	0.143631081	0.211233	-0.432571238	5.57E-07	-0.274413305	0.00188	0.15312703	0.309024	C5	-	Y	-	-	Y	-	-
ENSG00000196455	PIK3R4	0.43214921	5.20E-06	-0.002754439	0.990205	0.127902276	0.330518	-0.428769874	2.72E-09	-0.28994735	1.45E-05	0.133503308	0.28742	C5	-	Y	-	-	Y	-	-
ENSG00000196470	SIAH1	-0.294667289	0.000649	-0.271437145	0.01611	-0.446645812	2.35E-06	0.029850938	0.833805	-0.137117298	0.19954	-0.172372242	0.285182	C4	-	-	-	Y	-	-	-
ENSG00000196505	GDAP2	0.563208415	8.50E-08	0.088843233	0.612146	0.129930129	0.370442	-0.46806179	1.64E-06	-0.418750703	7.77E-06	0.043991521	0.855372	C5	-	Y	-	-	Y	Y	-
ENSG00000196517	SLC6A9	-1.228336854	3.58E-07	-1.055268074	0.000213	-0.516399481	0.061244	0.178552125	0.655487	0.726786161	0.008721	0.542785252	NA	C3	-	Y	Y	-	-	Y	-
ENSG00000196535	MYO18A	-0.313258761	8.15E-05	0.004293508	0.979284	0.062090438	0.587286	0.323937515	3.28E-05	0.390187267	3.96E-08	0.061053666	0.689354	C4	-	Y	-	-	Y	Y	-
ENSG00000196542	SPTSSB	-1.055305044	5.46E-05	-0.492201923	0.09278	-0.235650999	0.549725	0.569267005	0.088654	0.83447601	0.016591	0.259696141	NA	C4	-	Y	-	-	-	Y	-
ENSG00000196544	BORCS6	0.479131639	0.063844	0.295521789	0.3177	0.544726499	0.020907	-0.176775433	0.529734	0.080917677	0.784642	0.251894335	0.432208	C1	-	-	-	Y	-	-	-
ENSG00000196547	MAN2A2	0.460065608	2.10E-11	0.311508044	0.000436	0.235980107	0.012619	-0.141654935	0.021459	-0.209564712	0.001041	-0.072915271	0.60095	C1	-	Y	Y	-	-	-	-
ENSG00000196549	MME	-0.545520391	0.157496	0.213308206	0.663588	0.291518101	0.476113	0.765951492	0.048164	0.851956426	0.019899	0.081224911	NA	C4	-	-	-	-	Y	Y	-
ENSG00000196562	SULF2	-0.238170956	0.060996	-0.534913093	0.000174	-0.659527533	1.84E-07	-0.290843622	0.05535	-0.406754641	0.003183	-0.12129104	0.676618	C4	-	-	Y	Y	-	Y	-
ENSG00000196584	XRCC2	0.509938518	1.15E-12	0.236785323	0.006057	0.239322263	0.005453	-0.266448064	1.86E-07	-0.256621674	1.78E-06	0.0047864	0.97796	C5	Y	Y	-	-	-	-	-
ENSG00000196588	MRTFA	0.428906319	2.14E-05	0.211478356	0.132815	0.102502322	0.500508	-0.210776209	0.061202	-0.311626595	0.002687	-0.106150048	0.602159	C5	-	Y	-	-	-	Y	-
ENSG00000196593	ANKRD20A19P	-4.283567527	0.003484	-5.062516383	NA	-1.837455264	NA	-0.772849495	NA	2.465493852	NA	3.235111965	NA	C3	-	Y	-	-	-	-	-
ENSG00000196597	ZNF782	0.397141216	0.001971	0.137644614	0.476799	0.100784822	0.613549	-0.253158429	0.061808	-0.281689294	0.038482	-0.033980707	0.926517	C5	-	Y	-	-	-	-	-
ENSG00000196605	ZNF846	-0.186447251	0.344633	0.219223195	0.31251	0.191294755	0.398901	0.412195703	0.022477	0.392368337	0.040725	-0.025074294	0.958405	C4	-	-	-	-	Y	Y	-
ENSG00000196646	ZNF136	0.003369854	0.97954	-0.417817813	0.000516	-0.286005233	0.034779	-0.414784017	0.000201	-0.27445993	0.027711	0.135544458	0.601463	C5	-	-	Y	-	Y	-	-
ENSG00000196652	ZKSCAN5	-0.126263973	0.065998	-0.308311998	3.14E-05	-0.107246783	0.198071	-0.175673257	0.015706	0.033799597	0.713436	0.203947175	0.027575	C4	-	-	Y	-	-	-	-
ENSG00000196653	ZNF502	-0.313049371	0.013978	-0.124119953	0.517059	-0.371320634	0.007124	0.196002601	0.156223	-0.043364535	0.799327	-0.244595297	0.176021	C4	-	Y	-	Y	-	-	-
ENSG00000196659	TTC30B	-0.423129777	0.001172	-0.100327751	0.608807	-0.0634143	0.740568	0.329626705	0.026722	0.374473059	0.008546	0.039755949	0.908824	C4	-	Y	-	-	Y	Y	-
ENSG00000196660	SLC30A10	-1.292395776	0.00035	-0.646815761	0.119409	-0.89544487	0.021761	0.649854177	0.156712	0.412945277	0.423364	-0.243631345	NA	C4	-	Y	-	Y	-	-	-
ENSG00000196668	LINC00173	-3.307948715	0.035954	0.737746798	NA	-0.33545014	NA	4.060015913	0.007363	2.981871571	NA	-1.076010078	NA	C2	-	Y	-	-	Y	-	-
ENSG00000196712	NF1	0.51870007	7.93E-09	0.190755805	0.137187	0.202093029	0.06013	-0.321445204	0.009157	-0.302103644	1.22E-05	0.014112534	0.952439	C5	-	Y	-	-	Y	Y	-
ENSG00000196741	LINC01560	-0.485329348	0.050168	0.034980991	0.921957	-0.060809835	0.837931	0.528366381	0.042476	0.439063129	0.085383	-0.092770172	0.840067	C4	-	-	-	-	Y	-	-
ENSG00000196743	GM2A	-0.420816037	7.25E-05	-0.101816168	0.506513	-0.024806375	0.883585	0.326503621	0.011599	0.410619686	0.00051	0.079136511	0.748723	C4	-	Y	-	-	Y	Y	-
ENSG00000196747	H2AC13	-1.5433746	0.123115	-0.257275437	NA	0.361754238	0.718232	1.292224214	0.196311	1.919817485	0.020598	0.623583438	NA	C3	-	-	-	-	-	Y	-
ENSG00000196756	SNHG17	-0.113925911	0.202644	-0.016070629	0.933448	-0.353019934	0.000118	0.104268998	0.390141	-0.224258489	0.013758	-0.33386405	0.015467	C4	-	-	-	Y	-	-	Y

ENSG00000196757	ZNF700	-0.114502294	0.364076	-0.328679613	0.014778	-0.161474494	0.249429	-0.208146244	0.063017	-0.031792338	0.819543	0.170920129	0.303368	C4	-	-	Y	-	-	-	-
ENSG00000196781	TLE1	-0.359268896	1.73E-10	-0.149561941	0.085506	-0.14596978	0.026309	0.216157382	0.001975	0.228065479	5.55E-05	0.006448947	0.974616	C4	-	Y	-	-	-	-	-
ENSG00000196782	MAML3	-0.586625522	1.02E-26	-0.105329408	0.156444	-0.148323459	0.029754	0.487763639	6.80E-19	0.453084392	2.27E-16	-0.040189928	0.752303	C4	-	Y	-	-	Y	Y	-
ENSG00000196787	H2AC11	-0.359994691	0.225658	-0.304984591	0.350293	0.257178691	0.380717	0.059681439	0.88915	0.631984312	0.021647	0.565103547	0.095679	C4	-	-	-	-	-	Y	-
ENSG00000196793	ZNF239	-0.45991975	8.08E-06	-0.270286922	0.022446	-0.193537676	0.123856	0.196227379	0.12305	0.281162344	0.020384	0.079726338	0.728916	C4	-	Y	-	-	-	-	-
ENSG00000196810	CTBP1-DT	-0.34042744	0.001064	-0.059009607	0.680662	-0.075262931	0.549054	0.287666043	0.00724	0.280047687	0.008006	-0.01341356	0.957248	C4	-	Y	-	-	-	-	-
ENSG00000196812	ZSCAN16	0.091869224	0.571622	-0.237566981	0.105259	-0.122610427	0.440867	-0.323076071	0.01943	-0.199573446	0.170003	0.117912597	0.597621	C5	-	-	-	-	Y	-	-
ENSG00000196821	ILRUN	0.561384157	1.59E-23	0.334109264	4.73E-07	0.233873314	0.000821	-0.220915691	1.30E-05	-0.313158319	3.65E-10	-0.097488846	0.285162	C5	-	Y	Y	-	-	Y	-
ENSG00000196839	ADA	-0.839486257	6.31E-06	-0.133019867	0.615652	-0.209763062	0.3848	0.713357849	0.000127	0.644662053	0.001133	-0.073972214	0.859676	C2	-	Y	-	-	Y	Y	-
ENSG00000196843	ARID5A	0.83744717	0.000753	0.02585776	0.962896	0.432340491	0.187572	-0.804873039	0.001944	-0.390575007	0.146062	0.409149282	NA	C5	-	Y	-	-	Y	-	-
ENSG00000196862	RGPD4	1.299139542	0.009827	0.53088598	0.499366	0.873105915	0.177319	-0.761128445	0.141179	-0.411183602	0.461633	0.344816829	NA	C1	-	Y	-	-	-	-	-
ENSG00000196865	NHLRC2	0.178097836	0.105594	-0.179533957	0.199477	-0.033399532	0.835107	-0.351137855	0.000324	-0.197147845	0.031096	0.148930266	0.354512	C5	-	-	-	-	Y	-	-
ENSG00000196876	SCN8A	0.305085507	0.001185	0.176116458	0.165292	0.223379714	0.053524	-0.122820849	0.139979	-0.067368866	0.464336	0.050092278	0.778535	C1	-	Y	-	-	-	-	-
ENSG00000196911	KPNA5	0.316282784	0.000129	0.132153728	0.299642	0.206378402	0.033762	-0.177816443	0.065986	-0.09518275	0.317294	0.077346382	0.658238	C5	-	Y	-	-	-	-	-
ENSG00000196912	ANKRD36B	0.411855034	0.058643	0.627941144	0.001751	0.545582979	0.009709	0.222076172	0.28252	0.148170335	0.521091	-0.079291336	0.833312	C1	-	-	Y	Y	-	-	-
ENSG00000196924	FLNA	0.503777337	1.90E-22	0.360281322	1.53E-10	0.286197697	5.30E-08	-0.137167232	0.000368	-0.203087054	7.47E-11	-0.071115201	0.172644	C1	-	Y	Y	-	-	-	-
ENSG00000196935	SRGAP1	0.064316147	0.692073	-0.289630954	0.13037	-0.099927379	0.597184	-0.347521587	0.026357	-0.149696022	0.321494	0.192384294	0.481401	C5	-	-	-	-	Y	-	-
ENSG00000196937	FAM3C	0.45203569	8.60E-11	0.082957301	0.476179	-0.109210138	0.273214	-0.36251282	1.23E-06	-0.546632233	4.49E-15	-0.189226796	0.093981	C5	-	Y	-	-	Y	Y	-
ENSG00000196943	NOP9	0.344741265	6.38E-10	0.089962796	0.404484	0.121467936	0.146588	-0.247891119	0.000222	-0.208792971	0.000492	0.034441312	0.853204	C5	-	Y	-	-	-	-	-
ENSG00000196951	SCOC-AS1	-0.074101905	0.796221	0.018894679	0.962318	0.406090309	0.058289	0.099742579	0.74374	0.494933851	0.017467	0.390559519	0.165108	C4	-	-	-	-	-	Y	-
ENSG00000196967	ZNF585A	0.36711493	0.00022	-0.300251724	0.021985	0.309065669	0.008497	-0.660721008	3.91E-12	-0.043501446	0.736778	0.611911272	4.06E-08	C5	-	Y	Y	Y	Y	-	Y
ENSG00000196968	FUT11	0.344112595	0.000146	0.362042325	0.000129	0.200064887	0.071975	0.024520003	0.843734	-0.1290282	0.221129	-0.158967118	0.231807	C4	-	Y	Y	-	-	-	-
ENSG00000196975	ANXA4	-0.617851021	2.44E-08	-0.085452149	0.59047	-0.004372466	0.982114	0.538999884	6.43E-06	0.628160251	2.74E-07	0.083796809	0.721501	C4	-	Y	-	-	Y	Y	-
ENSG00000196981	WDR5B	0.340584902	0.000659	-0.156840721	0.333745	0.176026998	0.164201	-0.49105694	4.96E-06	-0.15007839	0.167664	0.335548578	0.023349	C5	-	Y	-	-	Y	-	Y
ENSG00000196990	FAM163B	-0.544018289	4.08E-11	-0.229740734	0.019018	-0.187514602	0.042076	0.320886034	7.92E-05	0.37135363	5.98E-07	0.044977228	0.787224	C4	-	Y	-	-	Y	Y	-
ENSG00000197008	ZNF138	0.377882043	4.80E-06	-0.07393787	0.606555	0.064744205	0.621166	-0.445436301	7.93E-10	-0.298688089	2.47E-05	0.141456139	0.253798	C5	-	Y	-	-	Y	-	-
ENSG00000197013	ZNF429	-0.28471086	0.029356	-0.490821096	0.000518	-0.142261984	0.360919	-0.199846394	0.193325	0.156879884	0.272929	0.351163494	0.033873	C4	-	-	Y	-	-	-	Y
ENSG00000197020	ZNF100	-0.080778306	0.477525	-0.552088301	1.40E-05	-0.292657349	0.015464	-0.464404221	2.63E-05	-0.19741639	0.071908	0.262589696	0.161374	C5	-	-	Y	-	Y	-	-
ENSG00000197024	ZNF398	0.482050976	1.02E-09	0.042647393	0.760042	0.187296241	0.050418	-0.433017142	4.10E-09	-0.280356718	0.000177	0.147341795	0.1995	C5	-	Y	-	-	Y	-	-
ENSG00000197043	ANXA6	-0.611222483	2.74E-23	-0.427341083	1.34E-08	-0.271543543	0.000104	0.190248677	0.010015	0.354509541	7.06E-09	0.158805659	0.116803	C4	-	Y	Y	-	-	Y	-
ENSG00000197044	ZNF441	-0.201386598	0.080579	0.170970737	0.166285	-0.192090991	0.09232	0.378355667	0.000471	0.023914907	0.883259	-0.360201729	0.002326	C4	-	-	-	-	Y	-	Y
ENSG00000197050	ZNF420	0.534904736	5.61E-08	-0.121484183	0.437927	0.219116397	0.059413	-0.649606094	3.62E-12	-0.301396115	0.000807	0.342625336	0.003748	C5	-	Y	-	-	Y	Y	Y
ENSG00000197062	ZSCAN26	0.39906529	0.001444	-0.017393707	0.944237	0.199612645	0.20549	-0.409008226	1.10E-05	-0.185489437	0.064382	0.21830927	0.115306	C5	-	Y	-	-	Y	-	-
ENSG00000197063	MAFG	0.517248279	8.68E-23	0.039126904	0.664689	0.200589752	0.004357	-0.47164698	1.57E-20	-0.302165927	6.41E-08	0.164310123	0.047395	C5	-	Y	-	-	Y	Y	-
ENSG00000197077	KIAA1671	0.500287219	2.07E-05	0.342507403	0.009186	0.327362827	0.018131	-0.151778951	0.150871	-0.158229842	0.170886	-0.012045523	0.966961	C5	-	Y	Y	Y	-	-	-
ENSG00000197093	GAL3ST4	0.617350961	8.87E-07	0.228130785	0.250281	-0.05146073	0.810107	-0.381710978	0.002755	-0.654213829	5.40E-10	-0.278075582	0.139879	C5	-	Y	-	-	Y	Y	-
ENSG00000197102	DYNC1H1	0.40870835	0.000733	0.222138056	0.025373	0.248807174	0.003379	-0.180075162	0.051035	-0.14568841	0.003296	0.029460713	0.806577	C5	-	Y	-	-	-	-	-
ENSG00000197124	ZNF682	-0.212957944	0.090052	-0.419823703	0.000515	-0.296827893	0.015936	-0.200415909	0.165808	-0.069185015	0.684995	0.125850048	0.570078	C4	-	-	Y	-	-	-	-
ENSG00000197134	ZNF257	0.011716854	0.967672	-0.496209715	0.015819	-0.084911326	0.781606	-0.501656541	0.006597	-0.082104832	0.755362	0.414214546	0.139381	C5	-	-	Y	-	Y	-	-
ENSG00000197136	PCNX3	0.306091216	9.42E-08	0.184518087	0.024049	0.11926461	0.0938	-0.114876707	0.082709	-0.172215869	0.000754	-0.06261379	0.591991	C5	-	Y	-	-	-	-	-
ENSG00000197142	ACSL5	1.935081366	0.024272	1.204416463	NA	1.437779332	NA	-0.720436619	0.372417	-0.485895169	0.566079	0.231307259	NA	C1	-	Y	-	-	-	-	-
ENSG00000197162	ZNF785	0.416207976	3.30E-05	0.163755846	0.303155	0.23995111	0.094231	-0.24681665	0.024985	-0.161866963	0.177368	0.078770579	0.762735	C5	-	Y	-	-	-	-	-
ENSG00000197182	MIRLET7BHG	-0.443709332	0.021488	0.336060501	0.07385	0.261596314	0.131905	0.787931047	1.40E-05	0.720560031	2.60E-05	-0.070917241	0.835119	C4	-	Y	-	-	Y	Y	-
ENSG00000197191	CYSRT1	-0.818624877	0.002293	0.297804341	0.390578	0.226811219	0.47111	1.121832287	0.000121	1.05989825	0.000117	-0.067775492	0.924107	C2	-	Y	-	-	Y	Y	-
ENSG00000197256	KANK2	-0.304565482	2.02E-06	-0.062115818	0.592148	-0.098754989	0.252917	0.248768502	0.000673	0.220572774	0.000713	-0.033621948	0.848056	C4	-	Y	-	-	-	-	-
ENSG00000197265	GTF2E2	0.568984893	9.09E-13	0.230829943	0.079356	0.321388981	0.000763	-0.332150621	0.00084	-0.232786355	0.005935	0.093518539	0.642574	C5	Y	Y	-	Y	Y	-	-
ENSG00000197275	RAD54B	0.50088181	0.000142	0.407858742	0.006519	0.211731452	0.179341	-0.086383188	0.611922	-0.274319516	0.041186	-0.19301405	0.341006	C5	-	Y	Y	-	-	-	-
ENSG00000197283	SYNGAP1	0.298132708	3.94E-05	0.149700434	0.097582	0.352181815	4.10E-06	-0.142318254	0.037658	0.068529566	0.378568	0.205428591	0.013285	C1	-	-	-	Y	-	-	-
ENSG00000197296	FITM2	0.329720485	0.031697	0.01639618	0.953449	0.249953218	0.135478	-0.306990337	0.046046	-0.065251121	0.729483	0.236261264	0.263634	C5	-	Y	-	-	Y	-	-
ENSG00000197308	GATA3-AS1	-0.749879689	2.28E-15	-0.162640302	0.17172	-0.261920011	0.010697	0.59414817	4.83E-13	0.503114951	1.04E-09	-0.096644793	0.479268	C2	-	Y	-	-	Y	Y	-
ENSG00000197312	DDI2	0.500045443	2.83E-08	0.080736319	0.552006	0.236335772	0.013585	-0.413382282	1.30E-08	-0.249580712	0.00035	0.158853798	0.090467	C5	-	Y	-	-	Y	-	-
ENSG00000197324	LRP10	-0.112348318	0.239723	0.12066277	0.288463	0.177601097	0.056004	0.23969457	0.009241	0.304592749	0.000266	0.059896556	0.729387	C4	-	-	-	-	-	Y	-
ENSG00000197343	ZNF655	0.382311639	1.69E-11	0.104614258	0.239817	0.256989625	1.08E-05	-0.271145384	1.47E-06	-0.110777238	0.023169	0.155081769	0.03989	C5	-	Y	-	-	-	-	-
ENSG00000197362	ZNF786	0.202738669	0.100026	-0.223646065	0.212375	-0.111485842	0.471993	-0.419380029	0.000828	-0.299754685	0.003235	0.114601046	0.639479	C5	-	-	-	-	Y	-	-
ENSG00000197363	ZNF517	-0.460340189	2.11E-05	-0.261810918	0.037431	-0.040672913	0.799196	0.205412501	0.121698	0.434426787	0.000103	0.224118416	0.148255	C4	-	Y	-	-	-	Y	-
ENSG00000197375	SLC22A5	0.445763454	0.002354	0.214353451	0.300847	0.277096137	0.083704	-0.225189887	0.20503	-0.154187631	0.35576	0.065250632	0.852434	C5	-	Y	-	-	-	-	-
ENSG00000197380	DACT3	0.411825944	1.25E-08	0.226960339	0.018784	0.185278226	0.0719	-0.178832034	0.019651	-0.212071311	0.006306	-0.038801498	0.843922	C5	-	Y	-	-	-	-	-
ENSG00000197406	DIO3	0.656353575	0.080608	-0.48050222	0.474362	0.154997169	0.810812	-1.12903291	0.010075	-0.486956528	0.258793	0.639036347	NA	C5	-	-	-	-	Y	-	-
ENSG00000197442	MAP3K5	-0.53912094	0.000361	-0.351368992	0.072519	-0.366914674	0.034234	0.193680187	0.371941	0.186869692	0.363432	-0.012539691	0.980847	C4	-	Y	-	Y	-	-	-
ENSG00000197444	OGDHL	-0.487363198	9.50E-16	-0.31057997	3.98E-06	-0.23138347	0.000228	0.183307282	0.006415	0.27068924	4.47E-06	0.082246632	0.423104	C4	-	Y	Y	-	-	-	-
ENSG00000197451	HNRNPAB	-0.035663419	0.529627	-0.154855601	0.040973	-0.304114551	1.38E-06	-0.112438458	0.028182	-0.253868866	4.20E-09	-0.146289895	0.099727	C4	Y	-	-	Y	-	-	-
ENSG00000197483	ZNF628	-0.797195486	6.28E-05	-0.008280825	0.9828	-0.480130083	0.028833	0.795041842	0.000241	0.332201101	0.185741	-0.4688261	0.107669	C3	-	Y	-	Y	Y	-	-
ENSG00000197496	SLC2A10	-0.757560466	0.009482	-0.490532208	0.141979	-0.343078468	0.321081	0.274041675	0.431149	0.429886515	0.17893	0.150443389	NA	C4	-	Y	-	-	-	-	-
ENSG00000197497	ZNF665	-0.651757532	0.00187	-0.157962306	0.590051	-0.457077442	0.043917	0.499697679	0.013716	0.209057133	0.391651	-0.295692897	0.306263	C4	-	Y	-	Y	Y	-	-
ENSG00000197498	RPF2	0.29496315	1.69E-05	-0.213341102	0.017429	-0.037827437	0.770015	-0.501901474	1.57E-13	-0.318297048	1.29E-05	0.178352036	0.140467	C5	-	-	-	-	Y	Y	-
ENSG00000197536	IRF1-AS1	-1.392054605	0.01219	-0.487008647	0.469703	-1.054345699	0.102286	0.915797618	0.177158	0.350611498	0.714375	-0.570867691	NA	C2	-	Y	-	-	-	-	-
ENSG00000197548	ATG7	-0.196364369	0.210485	0.183134893	0.23845	0.156034627	0.29796	0.386362922	0.003091	0.367274253	0.003993	-0.023534304	0.934559	C4	-	-	-	-	Y	Y	-

ENSG00000197555	SIPA1L1	-0.348731465	2.22E-06	-0.141432113	0.166448	-0.095720893	0.321729	0.213589897	0.012926	0.267653079	0.000577	0.048541414	0.78048	C4	-	Y	-	-	-	-	-
ENSG00000197558	SSPOP	-0.880710795	0.062298	-0.31855761	0.691023	0.431502092	0.399182	0.573751935	0.409083	1.326445432	0.003914	0.751356518	NA	C4	-	-	-	-	-	Y	-
ENSG00000197562	RAB40C	0.397253305	0.00046	0.329037076	0.013437	0.360547854	0.003846	-0.061661775	0.64134	-0.02192223	0.882009	0.034274602	0.891851	C5	-	Y	Y	Y	-	-	-
ENSG00000197566	ZNF624	0.141900489	0.385136	-0.179194903	0.362227	-0.050935102	0.831744	-0.314691407	0.02065	-0.178658886	0.246356	0.130898664	0.615445	C5	-	-	-	-	Y	-	-
ENSG00000197580	BCO2	1.72351357	8.87E-08	1.099757257	0.004082	1.183558082	0.001043	-0.617756465	0.041442	-0.525460966	0.084604	0.087025753	NA	C1	-	Y	Y	Y	Y	-	-
ENSG00000197601	FAR1	0.445591878	1.46E-16	0.054923815	0.571718	0.160751149	0.011277	-0.384068672	5.97E-11	-0.270101262	2.69E-07	0.108673335	0.262262	C5	-	Y	-	-	Y	-	-
ENSG00000197619	ZNF615	0.364325658	0.008686	-0.192173635	0.335477	0.458787133	0.001281	-0.550250778	0.000103	0.108998301	0.499432	0.653682878	2.72E-05	C5	-	Y	-	Y	Y	-	Y
ENSG00000197622	CDC42SE1	0.415507746	2.51E-20	0.251725713	1.20E-05	0.212398081	3.08E-05	-0.157634207	0.000456	-0.188509147	9.13E-07	-0.036335083	0.701152	C5	-	Y	-	-	-	-	-
ENSG00000197647	ZNF433	-0.26182419	0.352185	0.349995854	0.263569	-0.140386866	0.686838	0.619291342	0.01827	0.136845329	0.685592	-0.48727572	0.168713	C4	-	-	-	-	Y	-	-
ENSG00000197712	FAM114A1	-0.446709991	0.000551	-0.170153555	0.34647	0.111073191	0.513517	0.282549073	0.047744	0.572158086	1.64E-06	0.284597476	0.099019	C4	-	Y	-	-	-	Y	-
ENSG00000197713	RPE	0.548398159	1.36E-14	0.231787081	0.008198	0.358928574	4.10E-06	-0.310317376	6.75E-07	-0.175138092	0.006712	0.129822344	0.182394	C5	Y	Y	-	Y	Y	-	-
ENSG00000197724	PHF2	0.315140666	3.88E-07	0.151888432	0.051285	0.119651936	0.075131	-0.156856038	0.027183	-0.180710673	0.003371	-0.029400198	0.843071	C5	-	Y	-	-	-	-	-
ENSG00000197747	S100A10	0.091916517	0.916798	1.234374755	0.022709	1.271709312	0.009175	1.148397091	0.029259	1.19519251	0.015649	0.039778981	NA	C2	-	-	Y	Y	Y	Y	-
ENSG00000197748	CFAP43	-0.618455762	0.076036	-0.015220769	0.978244	0.075531952	0.869648	0.611210783	0.106826	0.709260772	0.047563	0.093579687	NA	C3	-	-	-	-	-	Y	-
ENSG00000197753	LHFPL5	-0.486833438	0.069009	0.046617071	0.905766	-0.277431103	0.375645	0.539986619	0.027111	0.223995991	0.467552	-0.321500187	0.361691	C4	-	-	-	-	Y	-	-
ENSG00000197757	HOXC6	0.394841271	0.005333	0.054961889	0.830121	0.332064466	0.047874	-0.333352346	0.032354	-0.048173766	0.815096	0.279812254	0.220878	C5	-	Y	-	Y	Y	-	-
ENSG00000197763	TXNRD3	-0.72032676	0.00122	-0.174510383	0.546021	-0.410966811	0.130275	0.551431322	0.008909	0.323981436	0.235686	-0.233135262	0.535931	C4	-	Y	-	-	Y	-	-
ENSG00000197774	EME2	-0.103635307	0.500879	0.282087535	0.024891	-0.063670201	0.664128	0.392079178	0.002785	0.054741069	0.745509	-0.342787711	0.011471	C4	-	-	-	-	Y	-	Y
ENSG00000197776	KLHDC1	-1.191756565	8.16E-08	-0.560738346	0.018461	-0.250891017	0.346718	0.637569005	0.017706	0.955136393	0.000127	0.312362021	0.397037	C4	-	Y	Y	-	Y	Y	-
ENSG00000197779	ZNF81	0.568849151	2.43E-06	0.139593298	0.41716	0.230945296	0.103117	-0.423409315	5.35E-07	-0.324146729	9.23E-05	0.094048665	0.514573	C5	-	Y	-	-	Y	Y	-
ENSG00000197782	ZNF780A	0.326355047	0.00085	-0.168096523	0.240998	0.11013842	0.389531	-0.487831359	2.10E-07	-0.202024958	0.020689	0.280749681	0.027176	C5	-	Y	-	-	Y	-	-
ENSG00000197798	FAM118B	0.34674136	0.009048	0.1903692	0.238666	0.158068773	0.356249	-0.150360747	0.230649	-0.174688904	0.176005	-0.029833078	0.919306	C5	-	Y	-	-	-	-	-
ENSG00000197818	SLC9A8	-0.679608286	1.82E-11	-0.364984445	0.002182	-0.463580332	6.35E-06	0.321570937	0.00914	0.230770198	0.057597	-0.095613135	0.655216	C4	-	Y	Y	Y	Y	-	-
ENSG00000197841	ZNF181	0.450196802	1.36E-06	0.033459448	0.863947	0.221016421	0.05536	-0.410692698	8.04E-06	-0.214680896	0.016626	0.190556607	0.184455	C5	-	Y	-	-	Y	-	-
ENSG00000197857	ZNF44	0.487525769	0.000863	0.20997125	0.30732	0.329407424	0.058715	-0.27115652	0.09252	-0.143637323	0.407032	0.122351241	0.679242	C5	-	Y	-	-	-	-	-
ENSG00000197859	ADAMTSL2	-0.555336813	1.66E-06	-0.459700295	0.00039	-0.355522021	0.0102	0.102428985	0.54145	0.214426506	0.155833	0.106890452	0.68708	C4	-	Y	Y	Y	-	-	-
ENSG00000197860	SGTB	0.585995101	4.57E-09	0.221844612	0.08859	0.30145909	0.01224	-0.357716277	3.57E-05	-0.269671594	0.003132	0.082548275	0.63838	C5	-	Y	-	Y	Y	-	-
ENSG00000197872	CYRIA	-1.781441198	4.02E-09	-1.114381367	0.000232	-0.536710643	0.068353	0.670059882	0.130767	1.258864912	0.000429	0.581153991	NA	C3	-	Y	Y	-	-	Y	-
ENSG00000197885	NKIRAS1	0.358718475	7.21E-07	0.066165113	0.575725	0.115804546	0.237378	-0.286255663	7.82E-05	-0.228330273	0.00199	0.052529886	0.736482	C5	-	Y	-	-	-	-	-
ENSG00000197889	MEIG1	-1.245101224	0.093098	-0.148711454	0.894329	0.286625496	0.746498	1.099041819	0.236227	1.545917463	0.047602	0.438306684	NA	C2	-	-	-	-	-	Y	-
ENSG00000197928	ZNF677	-0.352396878	8.88E-05	0.00431595	0.979553	0.005273709	0.97199	0.363390438	4.80E-05	0.372402822	1.22E-05	0.004168802	0.985912	C4	-	Y	-	-	Y	Y	-
ENSG00000197930	ERO1A	0.363058532	2.60E-12	0.227759785	0.001803	0.160718637	0.024836	-0.128838403	0.039245	-0.187460095	0.000743	-0.064139651	0.601463	C5	-	Y	-	-	-	-	-
ENSG00000197933	ZNF823	0.521791094	0.000233	-0.328627041	0.12173	0.063984467	0.797598	-0.843870174	8.42E-08	-0.443286774	0.003936	0.395787006	0.121334	C5	-	Y	-	-	Y	Y	-
ENSG00000197956	S100A6	-0.484713279	4.42E-05	-0.35008268	0.023296	-0.284663559	0.041206	0.140978049	0.340424	0.215146583	0.075907	0.068584539	0.804185	C4	-	Y	Y	-	-	-	-
ENSG00000197961	ZNF121	0.375453941	1.87E-06	0.123511098	0.224198	0.061683931	0.454259	-0.245300049	0.006095	-0.299126149	3.31E-05	-0.058797589	0.687297	C5	-	Y	-	-	-	-	-
ENSG00000197969	VPS13A	0.539494221	8.47E-10	0.239654405	0.025587	0.364118854	7.16E-05	-0.293519372	1.12E-06	-0.161157509	0.005592	0.126998972	0.128656	C1	-	Y	-	Y	-	-	-
ENSG00000197989	SNHG12	0.17967213	0.164724	0.028114286	0.886132	-0.201498845	0.134274	-0.145724768	0.297385	-0.366431575	0.002476	-0.226213937	0.196223	C5	-	-	-	-	-	Y	-
ENSG00000198010	DLGAP2	0.615204315	0.511218	-1.568862632	NA	-0.70896637	NA	-2.177194999	0.049646	-1.309411378	0.199298	0.865803697	NA	C5	-	-	-	-	Y	-	-
ENSG00000198018	ENTPD7	0.436316244	5.97E-07	0.191839756	0.093095	0.103452832	0.470901	-0.23807389	0.002091	-0.318349815	0.000204	-0.08556099	0.613509	C5	-	Y	-	-	-	Y	-
ENSG00000198028	ZNF560	-0.525389146	0.005223	-0.302620154	0.231861	-0.277896929	0.224404	0.228905633	0.358156	0.261990558	0.255269	0.027814729	0.960328	C4	-	Y	-	-	-	-	-
ENSG00000198039	ZNF273	0.088864991	0.493805	-0.381177535	0.001564	-0.149923958	0.272284	-0.464477566	5.91E-08	-0.224399548	0.012396	0.234611433	0.053674	C4	-	-	Y	-	Y	-	-
ENSG00000198042	MAK16	0.056604769	0.520549	-0.187800246	0.01858	-0.277543168	0.000676	-0.238088369	0.000412	-0.319632886	7.35E-06	-0.086810072	0.509778	C4	-	-	-	-	-	Y	-
ENSG00000198046	ZNF667	-0.334982597	0.000176	-0.235871056	0.027883	-0.079841286	0.605141	0.105660326	0.323756	0.270114478	0.011197	0.159340431	0.349912	C4	-	Y	-	-	-	-	-
ENSG00000198075	SULT1C4	0.004153091	0.978634	-0.015977448	0.942992	0.304301003	0.012047	-0.013554168	0.936173	0.314466029	0.002107	0.322809519	0.024557	C4	-	-	-	Y	-	Y	Y
ENSG00000198093	ZNF649	0.162189092	0.214725	-0.246972134	0.122493	0.143253575	0.357305	-0.402927951	0.001073	-0.004454822	0.977756	0.392866399	0.011856	C5	-	-	-	-	Y	-	Y
ENSG00000198105	ZNF248	0.651790095	2.34E-21	0.15688229	0.080272	0.397743061	6.26E-07	-0.488485036	5.65E-15	-0.239317114	0.000627	0.243758927	0.00601	C5	-	Y	-	Y	Y	-	-
ENSG00000198121	LPAR1	-0.568486803	3.54E-07	-0.117839475	0.455469	-0.206014468	0.113798	0.456828268	8.78E-05	0.377178183	0.001532	-0.085380916	0.697636	C4	-	Y	-	-	Y	Y	-
ENSG00000198125	MB	-2.655504678	0.042738	-1.603986633	NA	-2.419728911	NA	1.025955212	NA	0.255316151	NA	-0.775656816	NA	C3	-	Y	-	-	-	-	-
ENSG00000198130	HIBCH	-0.304996062	0.000146	-0.130696847	0.267972	0.043913569	0.725734	0.180683809	0.072536	0.36362041	1.83E-05	0.177643847	0.197685	C4	-	Y	-	-	-	Y	-
ENSG00000198146	ZNF770	0.48415724	6.99E-09	0.057786642	0.695334	0.176111295	0.073947	-0.420166356	6.08E-10	-0.293503074	1.09E-06	0.121345025	0.254177	C5	-	Y	-	-	Y	-	-
ENSG00000198153	ZNF849P	1.401086736	0.000313	-0.323986193	0.707732	0.371924833	0.612596	-1.725468371	1.54E-05	-1.016692031	0.01029	0.699198755	NA	C5	-	Y	-	-	Y	Y	-
ENSG00000198155	ZNF876P	-0.207446483	0.461839	0.267842864	0.33553	0.170713628	0.542133	0.482446732	0.01162	0.392533298	0.039604	-0.097202188	0.75917	C2	-	-	-	-	Y	Y	-
ENSG00000198157	HMGN5	-0.715759753	0.045386	-0.12538407	0.820521	-0.500030663	0.257521	0.599038213	0.158656	0.230035665	0.678942	-0.374700847	NA	C2	-	Y	-	-	-	-	-
ENSG00000198182	ZNF607	0.329075801	0.001912	-0.218922287	0.139007	0.133957621	0.342374	-0.542160861	6.16E-08	-0.180339879	0.077317	0.356516642	0.005376	C5	-	Y	-	-	Y	-	Y
ENSG00000198185	ZNF334	-0.317256308	4.64E-07	-0.079702448	0.417815	0.059473628	0.49068	0.244038049	0.000548	0.391356773	7.84E-11	0.142029794	0.137701	C4	-	Y	-	-	-	Y	-
ENSG00000198208	RPS6KL1	0.055744238	0.682891	0.35562215	0.00228	0.353651762	0.000849	0.306217261	0.004231	0.312407622	0.001821	0.000776398	0.997573	C4	-	-	Y	Y	Y	Y	-
ENSG00000198231	DDX42	-0.342002532	3.26E-20	-0.150361469	0.001662	-0.249028463	2.73E-10	0.198245484	5.75E-08	0.107644101	0.002359	-0.095932849	0.071212	C4	-	Y	-	-	-	-	-
ENSG00000198246	SLC29A3	-0.803414315	6.46E-11	-0.228052753	0.194517	-0.307047107	0.031919	0.582120663	8.22E-05	0.510978637	0.000273	-0.076706086	0.814924	C4	-	Y	-	Y	Y	Y	-
ENSG00000198252	STYX	0.289452748	0.06174	0.021560913	0.945042	0.433626381	0.004615	-0.263385401	0.059951	0.158547553	0.18774	0.416796037	0.007115	C4	-	-	-	Y	-	-	Y
ENSG00000198265	HELZ	0.556614763	7.06E-10	0.230359129	0.063312	0.33674037	0.000691	-0.319974108	5.36E-05	-0.205510989	0.002666	0.109281601	0.437738	C5	-	Y	-	Y	Y	-	-
ENSG00000198270	TMEM116	-0.42679926	0.003824	0.315618933	0.098926	0.062792052	0.769995	0.749005599	6.55E-07	0.504813786	0.000283	-0.249061564	0.271549	C4	-	Y	-	-	Y	Y	-
ENSG00000198298	ZNF485	-0.00248501	0.989548	-0.330611199	0.046686	-0.087908279	0.7004	-0.321874374	0.026359	-0.071184385	0.723423	0.245345871	0.326298	C5	-	-	Y	-	Y	-	-
ENSG00000198300	PEG3	0.351204364	0.007968	0.249949042	0.135079	0.112960034	0.531402	-0.09437073	0.479371	-0.224489632	0.03775	-0.135396618	0.469108	C1	-	Y	-	-	-	-	-
ENSG00000198315	ZKSCAN8	0.381496871	2.84E-09	0.044273406	0.708035	0.169648374	0.038829	-0.331252738	1.38E-11	-0.197425352	5.16E-05	0.128338507	0.128594	C5	-	Y	-	-	Y	-	-
ENSG00000198331	HYLS1	-0.450323875	0.024894	-0.399391563	0.066373	-0.201150147	0.399331	0.057483919	0.842075	0.263909656	0.243362	0.201123615	0.543441	C4	-	Y	-	-	-	-	-
ENSG00000198380	GFPT1	0.408584108	1.38E-07	0.204812962	0.038288	0.198909301	0.039436	-0.197085583	0.003321	-0.195365164	0.003877	-0.003426305	0.98674	C5	-	Y	-	-	-	-	-
ENSG00000198382	UVRAG	0.379547416	0.000176	0.250063115	0.065864	0.07470379	0.686622	-0.122400423	0.294646	-0.290703817	0.009526	-0.172226277	0.392863	C1	-	Y	-	-	-	-	-

ENSG00000198393	ZNF26	-0.263056307	0.000431	-0.233152497	0.00586	-0.366014648	2.70E-06	0.036478335	0.744797	-0.088159154	0.367984	-0.130055393	0.309109	C4	-	-	-	Y	-	-	-
ENSG00000198399	ITSN2	0.459216992	5.73E-07	0.420642777	9.84E-06	0.313904492	0.001277	-0.032017323	0.788841	-0.130559908	0.173938	-0.10390595	0.460756	C1	-	Y	Y	Y	-	-	-
ENSG00000198400	NTRK1	-1.091230172	1.69E-37	-0.209372475	0.205429	-0.071001122	0.538424	0.890906354	7.16E-06	1.034841823	1.76E-30	0.13908421	0.709413	C3	-	Y	-	-	Y	Y	-
ENSG00000198408	OGA	-1.226770349	1.92E-168	-0.520440257	2.70E-22	-0.639101551	4.84E-43	0.712898071	1.46E-51	0.60247629	2.95E-48	-0.115692785	0.108919	C3	-	Y	Y	Y	Y	Y	-
ENSG00000198416	ZNF658B	-1.123183795	0.000465	-0.359347395	0.387673	-0.317405359	0.378958	0.774389865	0.054818	0.819973982	0.025387	0.043566486	NA	C3	-	Y	-	-	-	Y	-
ENSG00000198417	MT1F	0.486622301	0.498458	-1.673063176	NA	0.029080687	0.980158	-2.146926451	0.004676	-0.441741989	0.557804	1.706073076	NA	C5	-	-	-	-	Y	-	-
ENSG00000198429	ZNF69	0.507038514	0.024193	0.236176493	0.457768	0.396444346	0.140142	-0.264116384	0.142975	-0.097052528	0.657382	0.161963132	0.61105	C1	-	Y	-	-	-	-	-
ENSG00000198431	TXNRD1	-0.164732213	0.000511	-0.375164893	1.25E-12	-0.400521052	2.80E-11	-0.203683457	5.42E-07	-0.22153136	3.22E-06	-0.022438069	0.876139	C4	-	-	Y	Y	-	-	-
ENSG00000198440	ZNF583	0.341101693	0.017829	-0.107311961	0.656918	-0.014860235	0.954659	-0.441624188	0.0028	-0.34138721	0.018133	0.095001478	0.767316	C5	-	Y	-	-	Y	Y	-
ENSG00000198468	FLVCR1-DT	-0.663353726	0.000107	-0.183881888	0.396283	-0.370922144	0.053865	0.486258617	0.010571	0.307140924	0.162401	-0.184187374	0.557664	C3	-	Y	-	-	Y	-	-
ENSG00000198478	SH3BGRL2	0.381967574	7.37E-08	0.112942784	0.253664	0.196138667	0.044239	-0.262576404	0.000146	-0.171147192	0.037547	0.086293167	0.558716	C5	-	Y	-	-	-	-	-
ENSG00000198538	ZNF28	0.073651164	0.548818	-0.295324543	0.019176	0.169974953	0.189218	-0.362588805	0.000903	0.11115124	0.369391	0.467995506	0.000337	C4	-	-	-	-	Y	-	Y
ENSG00000198553	KCNRG	-2.052618016	0.000961	-0.304489044	0.712364	-0.34814802	0.597184	1.750539055	0.015984	1.719920489	0.010617	-0.04079606	NA	C3	-	Y	-	-	Y	Y	-
ENSG00000198556	ZNF789	-0.163959432	0.154617	0.187793543	0.180027	0.114181718	0.429879	0.357743753	0.000169	0.292688539	0.001719	-0.070348176	0.730027	C4	-	-	-	-	Y	-	-
ENSG00000198569	SLC34A3	-0.791960465	0.013622	0.661550662	0.012826	0.563289032	0.039862	1.459078327	5.71E-09	1.369501655	1.14E-07	-0.095303284	0.840846	C2	-	Y	Y	Y	Y	Y	-
ENSG00000198570	RD3	-0.826637795	1.48E-41	-0.210201591	0.004129	-0.254421961	5.92E-06	0.623213431	7.93E-19	0.586844706	7.55E-22	-0.041352692	0.766122	C4	-	Y	-	-	Y	Y	-
ENSG00000198598	MMP17	-0.237583551	0.350729	-0.057931996	0.868649	0.259909022	0.280881	0.185970636	0.507204	0.512530733	0.021228	0.320727906	0.278505	C4	-	-	-	-	-	Y	-
ENSG00000198646	NCOA6	0.539263527	3.56E-18	0.35076542	1.57E-06	0.329527858	2.41E-06	-0.181543765	0.000114	-0.195153109	1.81E-05	-0.018868229	0.887295	C1	-	Y	Y	Y	-	-	-
ENSG00000198682	PAPSS2	-0.440650104	0.013933	-0.313755029	0.122376	-0.19574932	0.367871	0.133235734	0.557997	0.259635535	0.199392	0.120839658	0.719904	C4	-	Y	-	-	-	-	-
ENSG00000198695	MT-ND6	-0.734859244	6.46E-24	-0.738467891	5.98E-23	-0.478502586	6.51E-10	0.00316189	0.96457	0.271531983	3.54E-10	0.263223197	1.46E-08	C4	Y	Y	Y	Y	-	-	-
ENSG00000198707	CEP290	0.299323788	0.000431	0.218105505	0.036571	0.331177839	0.000278	-0.075131399	0.356979	0.046521636	0.584435	0.116216728	0.290571	C4	-	-	-	Y	-	-	-
ENSG00000198712	MT-CO2	-0.368085435	1.72E-09	-0.28379544	0.000215	-0.303675549	5.94E-05	0.091160703	0.012134	0.079502237	0.045002	-0.016925849	0.893044	C4	-	Y	-	Y	-	-	-
ENSG00000198718	TOGARAM1	0.509939986	0.000256	0.14643488	0.544338	0.230745146	0.217536	-0.357286688	0.010331	-0.265226956	0.044655	0.086981197	0.78425	C1	-	Y	-	-	Y	-	-
ENSG00000198727	MT-CYB	-1.328823645	4.06E-237	-1.237825338	6.09E-169	-1.27945091	1.37E-137	0.097590229	0.015615	0.064213512	0.209309	-0.038646532	0.713445	C3	-	Y	Y	Y	-	-	-
ENSG00000198730	CTR9	0.329335445	3.79E-08	0.06431762	0.451649	0.096757851	0.17	-0.258495259	2.34E-07	-0.217828669	6.23E-06	0.03518763	0.712971	C5	-	Y	-	-	-	-	-
ENSG00000198740	ZNF652	0.112734404	0.308627	-0.198954021	0.056734	-0.105970046	0.358538	-0.305528994	0.001975	-0.203852857	0.045434	0.096007396	0.56882	C5	-	-	-	-	Y	-	-
ENSG00000198743	SLC5A3	0.6191172	1.54E-10	0.226683847	0.084243	0.459928969	1.15E-05	-0.385637276	8.98E-07	-0.144660665	0.077317	0.235542025	0.029841	C1	-	Y	-	Y	Y	-	-
ENSG00000198753	PLXNB3	-0.303121549	0.000479	-0.031589786	0.831808	0.158447337	0.11455	0.277949567	0.002649	0.47633366	2.54E-08	0.192966179	0.107928	C4	-	Y	-	-	-	Y	-
ENSG00000198756	COLGALT2	-0.54408319	0.000332	-0.182307538	0.398612	-0.148034988	0.506965	0.368896849	0.04083	0.410790647	0.024098	0.036405405	0.939631	C4	-	Y	-	-	Y	Y	-
ENSG00000198763	MT-ND2	-0.679367584	1.06E-32	-0.331377473	5.77E-08	-0.418994384	1.31E-11	0.354803388	4.82E-19	0.275248258	3.79E-11	-0.084656228	0.19895	C4	-	Y	Y	Y	Y	-	-
ENSG00000198780	FAM169A	0.343503891	0.000546	0.179557439	0.191837	0.330581907	0.005868	-0.157632552	0.151648	0.001240548	0.992341	0.153784985	0.391666	C1	-	Y	-	Y	-	-	-
ENSG00000198783	ZNF830	0.341109675	6.43E-05	-0.171386346	0.16732	-0.136399964	0.240372	-0.50632185	2.54E-09	-0.462992508	9.26E-09	0.037920508	0.869474	C5	-	Y	-	-	Y	Y	-
ENSG00000198785	GRIN3A	-1.094008068	0.067027	0.413607749	0.495471	-0.315930567	0.662092	1.513856137	0.007001	0.793401034	0.282419	-0.725994564	NA	C2	-	-	-	-	Y	-	-
ENSG00000198786	MT-ND5	-0.52783655	3.71E-56	-0.438548106	1.74E-32	-0.257247415	6.18E-17	0.095990059	0.012474	0.28534631	5.67E-26	0.184196094	2.34E-07	C4	Y	Y	Y	-	-	-	-
ENSG00000198792	TMEM184B	0.616651653	5.50E-11	0.282792138	0.011472	0.306435232	0.004625	-0.327482318	0.000707	-0.295538763	0.00241	0.026518089	0.917283	C5	-	Y	-	Y	Y	-	-
ENSG00000198795	ZNF521	-0.918150467	7.81E-05	-0.417289948	0.076482	-0.381213167	0.077584	0.510082006	0.064181	0.552410148	0.031716	0.039294024	0.943467	C2	-	Y	-	-	-	Y	-
ENSG00000198797	BRINP2	-3.631436671	0.046665	-0.736090551	NA	-1.400104428	NA	2.888723466	NA	2.249229709	NA	-0.649044221	NA	C4	-	Y	-	-	-	-	-
ENSG00000198804	MT-CO1	-0.297444105	3.74E-12	-0.312021289	1.30E-10	-0.378560685	3.67E-15	-0.007975565	0.877588	-0.066403849	0.081082	-0.063688098	0.341131	C4	Y	-	Y	Y	-	-	-
ENSG00000198818	SFT2D1	0.387251596	6.00E-05	0.07235546	0.691497	0.076298988	0.684007	-0.30913838	0.002504	-0.296141577	0.00925	0.006215018	0.986496	C5	-	Y	-	-	Y	-	-
ENSG00000198822	GRM3	0.353657045	0.628307	1.312827785	0.015819	0.490057263	0.549054	0.964790363	0.045505	0.15039576	0.851481	-0.820107643	NA	C2	-	-	Y	-	Y	-	-
ENSG00000198824	CHAMP1	-0.098593089	0.11045	-0.402790004	6.37E-09	-0.290088663	3.02E-05	-0.29749301	1.11E-06	-0.176976405	0.004646	0.115818497	0.325251	C4	-	-	Y	-	-	-	-
ENSG00000198830	HMGN2	-0.233251423	2.81E-09	-0.208659859	0.00042	-0.351817591	1.97E-16	0.031334865	0.552339	-0.103629912	0.000488	-0.140385277	0.012564	C4	-	-	-	Y	-	-	-
ENSG00000198832	SELENOM	-0.507546094	0.004014	-0.339291896	0.098671	-0.315146007	0.159406	0.175058144	0.382688	0.207222576	0.333958	0.027118423	0.955888	C4	-	Y	-	-	-	-	-
ENSG00000198835	GJC2	0.88855072	0.000259	0.096421736	0.859913	0.163743977	0.693638	-0.785047391	0.007156	-0.709476995	0.007092	0.069656928	NA	C5	-	Y	-	-	Y	Y	-
ENSG00000198837	DENND4B	0.303787516	9.18E-09	0.173206074	0.00085	0.200338336	1.78E-05	-0.124148904	0.021082	-0.088793682	0.095479	0.029969231	0.736151	C5	-	Y	-	-	-	-	-
ENSG00000198838	RYR3	-0.34493021	0.169758	0.134787217	0.658356	-0.203290132	0.477332	0.486405464	0.024135	0.156434995	0.579661	-0.334912471	0.240869	C2	-	-	-	-	Y	-	-
ENSG00000198840	MT-ND3	-0.729373518	1.60E-11	-0.643471808	2.31E-05	-0.536581586	9.81E-05	0.093215216	0.413394	0.208107255	3.99E-06	0.109809721	0.59664	C4	-	Y	Y	Y	-	-	-
ENSG00000198841	KTI12	-0.095692366	0.666686	-0.059084059	0.865012	-0.480527422	0.022487	0.042904944	0.882566	-0.369660889	0.077811	-0.418034034	0.18424	C5	-	-	-	Y	-	-	-
ENSG00000198846	TOX	-0.486931661	1.08E-06	-0.121972787	0.386824	-0.130422409	0.305488	0.371665506	7.61E-05	0.370993287	5.07E-05	-0.006243153	0.9809	C4	-	Y	-	-	Y	Y	-
ENSG00000198857	HSD3BP5	1.950153705	0.004057	0.330714573	NA	1.34042107	0.097363	-1.606607141	0.035928	-0.595102229	0.393906	1.007248146	NA	C1	-	Y	-	-	Y	-	-
ENSG00000198862	LTN1	0.484198798	1.77E-15	0.178199244	0.03599	0.252889253	0.001486	-0.299163339	4.59E-09	-0.216447597	9.70E-05	0.07746692	0.506753	C5	-	Y	-	-	-	-	-
ENSG00000198863	RUNDC1	0.423963505	7.77E-07	-0.052941769	0.742741	0.121495414	0.300081	-0.470257742	4.21E-08	-0.28785339	0.000474	0.177118466	0.183573	C5	-	Y	-	-	Y	-	-
ENSG00000198865	CCDC152	-0.348937906	0.008407	-0.381338768	0.010537	-0.404708615	0.002178	-0.026170669	0.899466	-0.041277927	0.829014	-0.020567943	0.955948	C4	-	Y	Y	Y	-	-	-
ENSG00000198870	STKLD1	-0.642958127	0.021974	-0.092587361	0.818748	-0.114105414	0.781353	0.556608547	0.044471	0.542607208	0.079416	-0.018773484	0.979338	C4	-	Y	-	-	Y	-	-
ENSG00000198876	DCAF12	0.311342466	6.34E-09	0.114811971	0.129676	0.123704822	0.10901	-0.189860674	0.00014	-0.17295283	0.001594	0.01176304	0.943467	C5	Y	Y	-	-	-	-	-
ENSG00000198879	SFMBT2	0.300283187	1.85E-07	0.306915954	4.79E-06	0.241078297	0.0013	0.012789172	0.869696	-0.044643668	0.543478	-0.063031073	0.600876	C4	-	Y	Y	-	-	-	-
ENSG00000198885	ITPRIPL1	0.341840578	3.38E-05	-0.009465409	0.954346	0.173128739	0.103516	-0.344920018	3.81E-05	-0.154237587	0.108938	0.185515939	0.15234	C5	-	Y	-	-	Y	-	-
ENSG00000198886	MT-ND4	-0.58472745	9.54E-37	-0.807200148	1.62E-78	-0.793847229	5.05E-53	-0.215803784	3.31E-11	-0.194275663	2.39E-06	0.01628274	0.879022	C4	-	Y	Y	Y	-	-	-
ENSG00000198887	SMC5	0.305393189	0.000319	0.055124325	0.720969	0.059895167	0.647091	-0.243962039	0.000139	-0.230931634	2.88E-05	0.007611061	0.969121	C5	-	Y	-	-	-	-	-
ENSG00000198888	MT-ND1	-0.463399746	9.57E-19	-0.217892691	0.001163	-0.320256301	2.46E-06	0.252115251	2.16E-09	0.158023646	0.050198	-0.099347724	0.264463	C4	-	Y	-	Y	-	-	-
ENSG00000198890	PRMT6	-0.336039497	3.86E-09	-0.210656442	0.002089	-0.369620278	3.05E-09	0.131965369	0.046093	-0.01877614	0.834367	-0.156011737	0.073764	C4	-	Y	-	Y	-	-	-
ENSG00000198894	CIPC	0.558949838	3.35E-09	0.111280187	0.457771	0.29680713	0.005509	-0.441661909	5.18E-08	-0.247967141	0.001649	0.188316595	0.10912	C5	-	Y	-	-	Y	-	-
ENSG00000198899	MT-ATP6	-0.48558702	2.73E-20	-0.570764123	5.41E-22	-0.497641948	8.03E-16	-0.078299534	0.078611	0.002940407	0.960892	0.076052496	0.35326	C4	-	Y	Y	Y	-	-	-
ENSG00000198900	TOP1	0.434689661	7.07E-16	0.23321664	5.42E-05	0.175516901	0.005068	-0.195150521	1.06E-05	-0.246832832	2.02E-08	-0.056973104	0.479368	C5	-	Y	-	-	-	-	-
ENSG00000198909	MAP3K3	0.482429597	2.72E-16	0.259015169	0.000156	0.187848432	0.007392	-0.216848497	0.000634	-0.279910198	2.77E-06	-0.068234192	0.557808	C5	-	Y	-	-	-	-	-
ENSG00000198910	L1CAM	-0.214952268	1.21E-07	0.048726755	0.476799	0.122385066	0.010132	0.27013942	1.04E-11	0.351995355	6.57E-26	0.076559584	0.246845	C4	-	-	-	-	-	Y	-
ENSG00000198915	RASGEF1A	3.993081702	0.028473	3.633092246	NA	2.053415068	NA	-0.352762761	NA	-1.941709683	NA	-1.590139305	NA	C1	-	Y	-	-	-	-	-

ENSG00000198919	DZIP3	-0.461669148	5.18E-08	-0.00076948	0.996844	0.161333455	0.162367	0.466789934	2.73E-09	0.637703552	4.56E-17	0.165036465	0.226149	C4	-	Y	-	-	Y	Y	-
ENSG00000198920	KIAA0753	0.375684568	6.45E-07	0.025080393	0.87519	0.098519041	0.339161	-0.344085118	4.65E-05	-0.262513567	0.000611	0.0762294	0.653289	C5	-	Y	-	-	Y	-	-
ENSG00000198925	ATG9A	0.428442609	8.09E-05	0.221154714	0.07385	0.302672593	0.00566	-0.200705694	0.082431	-0.111170765	0.362696	0.084506634	0.644631	C5	-	Y	-	Y	-	-	-
ENSG00000198931	APRT	-0.424959295	1.02E-07	-0.232495848	0.01935	-0.187665837	0.047072	0.19874636	0.016534	0.252093844	0.000925	0.047982811	0.771055	C4	-	Y	-	-	-	-	-
ENSG00000198932	GPRASP1	-0.520652586	2.55E-11	-0.202969831	0.048524	-0.062777987	0.578891	0.324131891	0.000117	0.472597733	1.67E-10	0.143082425	0.240869	C4	-	Y	-	-	Y	Y	-
ENSG00000198937	CCDC167	-0.644753407	1.66E-11	-0.264139582	0.014382	-0.366296294	0.000302	0.387215135	7.02E-05	0.293492377	0.004444	-0.099227943	0.556648	C2	-	Y	-	Y	Y	-	-
ENSG00000198938	MT-CO3	-1.085861875	4.38E-130	-0.953153418	1.15E-67	-0.879873546	5.08E-52	0.139521926	0.000213	0.220955959	1.01E-08	0.076181275	0.395152	C3	Y	Y	Y	Y	-	-	-
ENSG00000198952	SMG5	0.408985525	1.05E-26	0.093404351	0.097711	0.033953645	0.633997	-0.308944317	1.05E-17	-0.360492966	1.39E-22	-0.056614469	0.476373	C5	Y	Y	-	-	Y	Y	-
ENSG00000198959	TGM2	1.266677358	0.000315	1.638324535	0.00021	1.591430541	1.40E-06	0.377984254	0.383883	0.339505308	0.300822	-0.045676959	NA	C1	-	Y	Y	Y	-	-	-
ENSG00000198963	RORB	0.812436166	2.41E-27	0.425691688	2.73E-05	0.462381409	1.49E-07	-0.380031664	3.26E-09	-0.335796814	3.03E-10	0.039185347	0.828373	C5	Y	Y	Y	Y	Y	Y	-
ENSG00000198964	SGMS1	0.317269618	0.028238	0.234319478	0.177362	0.059042849	0.784304	-0.076385121	0.656703	-0.243492227	0.087368	-0.17275191	0.423104	C1	-	Y	-	-	-	-	-
ENSG00000199038	MIR210	-1.60904143	0.294353	0.790529624	NA	0.953770048	NA	2.408747403	0.096	2.578423339	0.045641	0.167675273	NA	C2	-	-	-	-	-	Y	-
ENSG00000200534	SNORA33	-0.713895984	0.343394	0.872900734	0.167042	-0.197720766	0.820555	1.59011909	0.012127	0.532597729	0.537617	-1.067400741	NA	C4	-	-	-	-	Y	-	-
ENSG00000200966	RN7SKP87	-0.646494439	0.438448	1.036430712	0.117254	0.693000243	0.347367	1.689637995	0.006615	1.352394208	0.047697	-0.339027291	NA	C2	-	-	-	-	Y	Y	-
ENSG00000201201	RN7SKP118	-2.690912094	NA	0.055690137	NA	2.050787561	NA	2.761888608	NA	4.750673299	0.002627	2.016437143	NA	C3	-	-	-	-	-	Y	-
ENSG00000202399	Y_RNA	-3.719639712	0.009997	-0.652701672	NA	-0.72163776	NA	3.091334626	NA	3.014472016	NA	-0.070435355	NA	C3	-	Y	-	-	-	-	-
ENSG00000203307	FAM78B-AS1	2.447479732	0.018155	1.116187033	NA	1.141969481	NA	-1.322307255	0.178859	-1.299249612	0.232621	0.027853796	NA	C5	-	Y	-	-	-	-	-
ENSG00000203497	PDCD4-AS1	-0.532087023	0.016115	-0.121055195	0.704854	-0.023216787	0.949421	0.418175427	0.087951	0.523744569	0.032132	0.100409591	0.840067	C4	-	Y	-	-	-	Y	-
ENSG00000203506	RBMS3-AS2	-0.79150427	0.000104	-0.503738866	0.022308	-0.672926218	0.001762	0.294221877	0.266893	0.133234927	0.678113	-0.166484933	0.701817	C3	-	Y	Y	Y	-	-	-
ENSG00000203663	OR2L2	-3.502833895	0.019319	-1.185458091	NA	-1.278631958	NA	2.351962863	NA	2.234910979	NA	-0.109107953	NA	C3	-	Y	-	-	-	-	-
ENSG00000203667	COX20	-0.375845374	0.000295	-0.037866381	0.831467	-0.194515988	0.105043	0.344832185	0.00266	0.195989477	0.113141	-0.153894654	0.39019	C4	-	Y	-	-	Y	-	-
ENSG00000203709	MIR29B2CHG	0.692891228	0.012166	0.601533729	0.074629	0.923565956	0.001231	-0.084403495	0.823317	0.246031691	0.417974	0.32501308	0.481267	C4	-	Y	-	Y	-	-	-
ENSG00000203761	MSTO2P	0.185446591	0.195691	-0.188765351	0.309692	-0.161554765	0.33507	-0.36847844	0.003693	-0.332704553	0.004971	0.030522693	0.928882	C5	-	-	-	-	Y	Y	-
ENSG00000203778	FAM229B	-0.578908882	3.73E-05	-0.135595911	0.512727	-0.294509736	0.075648	0.449893015	0.005721	0.298955529	0.088036	-0.156114017	0.58593	C4	-	Y	-	-	Y	-	-
ENSG00000203808	BVES-AS1	-0.152351703	0.520594	-0.244163927	0.364884	0.19693298	0.399627	-0.08478074	0.713074	0.363106138	0.01915	0.441686855	0.022715	C4	-	-	-	-	-	Y	Y
ENSG00000203865	ATP1A1-AS1	-0.465276886	0.011715	-0.111106487	0.677058	0.076245183	0.772723	0.361020251	0.073889	0.555965458	0.003218	0.190075541	0.540963	C4	-	Y	-	-	-	Y	-
ENSG00000203965	EFCAB7	0.445266318	0.000345	0.080402201	0.714328	0.406435311	0.008513	-0.358878026	0.003135	-0.02370565	0.898596	0.329098244	0.070541	C5	-	Y	-	Y	Y	-	-
ENSG00000203999	LINC01270	-0.758085208	9.85E-08	-0.314673562	0.047233	-0.341458512	0.037809	0.450461942	0.004708	0.43149067	0.011596	-0.02420679	0.952689	C2	-	Y	Y	Y	Y	Y	-
ENSG00000204054	LINC00963	-0.810891227	1.12E-08	-0.201860877	0.313135	-0.200732461	0.254576	0.615099671	1.76E-05	0.625120211	3.47E-06	0.004989789	0.990434	C4	-	Y	-	-	Y	Y	-
ENSG00000204060	FOXO6	-0.36429689	0.245394	-0.112855228	0.812869	0.186301217	0.610737	0.258008485	0.433123	0.566085235	0.028565	0.302640461	0.467109	C4	-	-	-	-	-	Y	-
ENSG00000204084	INPP5B	-0.344742561	0.00117	-0.22903412	0.050713	-0.180187071	0.130242	0.122131109	0.352363	0.179202719	0.143487	0.051740829	0.8242	C4	-	Y	-	-	-	-	-
ENSG00000204091	TDRG1	-0.448772436	0.189636	-0.693987409	0.061167	0.201830084	0.617481	-0.239261015	0.5688	0.664598463	0.029381	0.898201629	NA	C4	-	-	-	-	-	Y	-
ENSG00000204103	MAFB	-0.490949422	6.45E-05	-0.312738047	0.053491	-0.434047772	0.002377	0.184683416	0.266384	0.071718733	0.706077	-0.118102907	0.688161	C4	-	Y	-	Y	-	-	-
ENSG00000204104	TRAF3IP1	0.660029412	4.43E-12	0.290799716	0.018026	0.252473362	0.040889	-0.362653416	2.16E-05	-0.392879793	5.50E-06	-0.035598197	0.877754	C5	-	Y	-	-	Y	Y	-
ENSG00000204116	CHIC1	0.436654675	0.004518	0.115659008	0.612146	0.113652717	0.632404	-0.315363926	0.012419	-0.308093926	0.029546	0.001475682	0.997245	C5	-	Y	-	-	Y	Y	-
ENSG00000204120	GIGYF2	0.516749034	6.28E-18	0.237232482	7.73E-05	0.217640579	0.001142	-0.236969684	2.86E-06	-0.284704073	1.73E-09	-0.053107523	0.57455	C5	Y	Y	-	-	-	-	-
ENSG00000204128	C2orf72	-0.23254776	0.093925	-0.4036716	0.005651	-0.049812872	0.785135	-0.165222046	0.319855	0.197091915	0.164356	0.356478616	0.033226	C4	-	-	Y	-	-	-	Y
ENSG00000204131	NHSL2	-0.04747889	0.82851	0.164918038	0.448455	0.385421879	0.139933	0.217773016	0.038533	0.447442343	6.43E-06	0.224244694	0.106986	C4	-	-	-	-	-	Y	-
ENSG00000204136	GGTA1P	-1.240005264	0.036479	-0.407040696	0.580094	-0.200992499	0.778858	0.841391366	0.256462	1.055070668	0.111217	0.209697686	NA	C3	-	Y	-	-	-	-	-
ENSG00000204152	TIMM23B	-0.004163508	0.982639	-0.004399585	0.987462	-0.335654822	0.016314	0.005740621	0.978531	-0.316687731	0.025197	-0.328243194	0.089669	C4	-	-	-	Y	-	Y	-
ENSG00000204160	ZDHHC18	0.492653809	4.75E-11	0.190197986	0.080435	0.241801411	0.006661	-0.295467824	6.34E-05	-0.235888295	0.000491	0.054020831	0.732446	C5	-	Y	-	-	-	-	-
ENSG00000204186	ZDBF2	0.440626104	3.09E-06	0.223603325	0.092652	0.375760737	0.000366	-0.21131112	0.0178	-0.0503054	0.606076	0.15550667	0.264463	C5	-	Y	-	Y	-	-	-
ENSG00000204217	BMPR2	0.472091227	1.76E-10	0.208572308	0.030203	0.277400488	0.002357	-0.257053799	0.000166	-0.179916605	0.013105	0.071670501	0.627756	C5	-	Y	-	-	-	-	-
ENSG00000204228	HSD17B8	-0.610075069	0.010586	-0.417675125	0.244195	-0.068935888	0.853069	0.19818728	0.557674	0.556998219	0.013283	0.352742892	0.415965	C3	-	Y	-	-	-	Y	-
ENSG00000204237	OXLD1	-0.448502069	4.17E-06	-0.212044953	0.079835	-0.138273685	0.254636	0.243000859	0.019919	0.325226721	0.000685	0.076894582	0.67409	C4	-	Y	-	-	-	Y	-
ENSG00000204241	LINC02731	-2.157626473	2.43E-06	-0.309443922	0.516146	-0.170927	0.723596	1.855188987	0.000339	2.00095914	6.22E-05	0.141351687	NA	C3	-	Y	-	-	Y	Y	-
ENSG00000204253	HNRNPCP2	-0.075774641	0.691449	-0.377143985	0.029912	-0.27386519	0.1152	-0.295181596	0.085301	-0.183058566	0.313751	0.10644284	0.746966	C4	-	-	Y	-	-	-	-
ENSG00000204267	TAP2	-0.861030267	0.010634	-0.433183439	0.318155	-0.197192142	0.698518	0.434930874	0.288667	0.678286277	0.084283	0.238276257	NA	C4	-	Y	-	-	-	-	-
ENSG00000204283	LINC01973	-2.462086573	2.71E-07	-0.015467358	0.981231	-0.395359612	0.366731	2.454429563	2.26E-06	2.081743534	0.000103	-0.377370918	NA	C3	-	Y	-	-	Y	Y	-
ENSG00000204301	NOTCH4	-0.329320823	0.023745	-0.099935387	0.635944	-0.321544083	0.027356	0.235247543	0.156712	0.02265659	0.91563	-0.218833884	0.316368	C4	-	Y	-	Y	-	-	-
ENSG00000204311	PJVK	-0.841613388	0.00339	-0.375580374	0.2637	-0.625708557	0.03258	0.471238223	0.191251	0.231168364	0.579386	-0.246652843	NA	C3	-	Y	-	Y	-	-	-
ENSG00000204314	PRRT1	-0.56721253	0.016607	-0.24009155	0.425267	-0.329460743	0.20618	0.334843868	0.229987	0.252490655	0.379199	-0.086421017	0.87281	C2	-	Y	-	-	-	-	-
ENSG00000204315	FKBPL	0.091821219	0.741736	-0.390234283	0.154029	0.169560212	0.553283	-0.47554039	0.028434	0.092718901	0.720589	0.562672179	0.029061	C5	-	-	-	-	Y	-	Y
ENSG00000204344	STK19	0.058420026	0.734426	-0.263201133	0.069877	-0.313800972	0.023116	-0.315600292	0.022178	-0.357297207	0.008707	-0.04751078	0.88366	C5	-	-	-	Y	Y	Y	-
ENSG00000204381	LAYN	-0.712646133	0.002955	0.182747169	0.563778	0.009277818	0.982114	0.90457403	0.000437	0.736763758	0.009912	-0.173471878	0.737367	C2	-	Y	-	-	Y	Y	-
ENSG00000204382	XAGE1B	-2.438027426	0.031849	-0.307675205	NA	-1.274997813	NA	2.139086745	0.081665	1.176093334	NA	-0.965243727	NA	C3	-	Y	-	-	-	-	-
ENSG00000204386	NEU1	0.364978342	0.000311	0.197546849	0.210973	0.307521277	0.011229	-0.160706565	0.202975	-0.042684263	0.758769	0.112624828	0.612037	C5	-	Y	-	Y	-	-	-
ENSG00000204388	HSPA1B	1.523764368	1.00E-31	0.943268799	7.35E-12	1.499305879	4.14E-39	-0.574144197	1.40E-06	-0.009767226	0.95182	0.559660989	9.85E-07	C1	-	Y	Y	Y	Y	-	Y
ENSG00000204389	HSPA1A	1.053519814	2.45E-19	0.557826522	1.90E-05	1.115297188	6.20E-32	-0.489857945	8.17E-05	0.076218594	0.57889	0.559501144	9.85E-07	C1	-	Y	Y	Y	Y	-	Y
ENSG00000204390	HSPA1L	0.515943623	0.128662	0.115440585	0.831336	0.817416524	0.002006	-0.395666961	0.324928	0.316153746	0.331752	0.703206719	NA	C1	-	-	-	Y	-	-	-
ENSG00000204406	MBD5	-0.061472892	0.632522	-0.429504804	0.000477	-0.133051288	0.315405	-0.362887018	7.99E-05	-0.056939541	0.589861	0.300271471	0.013081	C4	-	-	Y	-	Y	-	Y
ENSG00000204410	MSH5	-0.196421924	0.037195	0.104484822	0.43008	0.15652963	0.167581	0.307078822	0.000367	0.367827058	1.36E-05	0.055035448	0.775214	C4	-	-	-	-	Y	Y	-
ENSG00000204427	ABHD16A	0.325332463	0.039203	-0.290105971	0.19464	-0.055392092	0.839727	-0.608718716	0.000241	-0.365872896	0.03264	0.237774492	0.456043	C5	-	Y	-	-	Y	Y	-
ENSG00000204439	C6orf47	0.398314019	6.65E-05	-0.006149533	0.975269	0.142690162	0.268444	-0.398093986	6.51E-05	-0.240916021	0.02003	0.151749654	0.344923	C5	-	Y	-	-	Y	-	-
ENSG00000204514	ZNF814	0.458850869	0.000192	0.249008435	0.126824	0.483202743	0.000314	-0.203420425	0.151717	0.039196639	0.824955	0.237059073	0.215908	C4	-	Y	-	Y	-	-	-
ENSG00000204519	ZNF551	-0.405980571	2.17E-05	-0.163010722	0.168438	-0.217540133	0.111931	0.249349496	0.006479	0.202448892	0.08803	-0.051825508	0.836208	C4	-	Y	-	-	-	-	-
ENSG00000204524	ZNF805	0.375942624	0.007458	-0.15995554	0.479463	-0.030379979	0.90413	-0.528875983	0.00012	-0.391529323	0.003358	0.132066483	0.643966	C5	-	Y	-	-	Y	Y	-

ENSG00000204525	HLA-C	-0.327606135	0.012948	-0.279876705	0.108574	-0.252693678	0.104548	0.05422115	0.780713	0.089733796	0.586818	0.030405645	0.937494	C4	-	Y	-	-	-	-	-
ENSG00000204560	DHX16	0.305971092	5.69E-06	0.067446356	0.461554	0.079074289	0.364113	-0.232088064	0.000445	-0.212154315	0.00159	0.014527419	0.933407	C5	-	Y	-	-	-	-	-
ENSG00000204569	PPP1R10	-0.722782956	3.53E-35	-0.406520088	4.06E-08	-0.469701248	1.54E-16	0.322664593	5.98E-06	0.267645906	8.00E-06	-0.060403056	0.662988	C4	-	Y	Y	Y	Y	-	-
ENSG00000204577	LILRB3	1.235592943	0.013102	0.245983268	NA	0.152568743	0.877765	-0.989413235	0.098838	-1.070857256	0.0446	-0.090393637	NA	C5	-	Y	-	-	-	Y	-
ENSG00000204590	GNL1	0.296757987	3.07E-13	0.029073619	0.754677	-0.0509263	0.404987	-0.261114105	1.62E-08	-0.332986	2.25E-19	-0.077293888	0.37294	C5	Y	-	-	-	-	Y	-
ENSG00000204599	TRIM39	-0.039549819	0.809636	-0.265230035	0.057355	-0.325660319	0.016694	-0.219722783	0.119239	-0.271436562	0.053494	-0.056966945	0.853543	C5	-	-	-	Y	-	-	-
ENSG00000204604	ZNF468	0.219758522	0.057475	-0.23863839	0.103146	0.020974089	0.913217	-0.451818839	3.77E-05	-0.184501507	0.1093	0.262215027	0.10078	C5	-	-	-	-	Y	-	-
ENSG00000204611	ZNF616	0.140915775	0.194143	-0.166093674	0.208306	0.100748812	0.424049	-0.300528673	0.003973	-0.025539621	0.851974	0.269717891	0.037402	C5	-	-	-	-	Y	-	-
ENSG00000204623	ZNRD1ASP	0.182337812	0.254555	0.379702013	0.03023	0.565213351	5.11E-05	0.203481824	0.196846	0.397607984	0.001449	0.188776699	0.397037	C4	-	-	Y	Y	-	Y	-
ENSG00000204624	DISP3	-0.25105636	0.136661	-0.407979208	0.036768	-0.386969629	0.030532	-0.150524779	0.425911	-0.120883478	0.510189	0.024365876	0.955785	C4	-	-	Y	Y	-	-	-
ENSG00000204653	ASPDH	-0.847093496	3.24E-12	-0.710473427	9.69E-08	-0.741606954	8.71E-08	0.143255216	0.440546	0.120302169	0.549113	-0.028477728	0.947861	C4	-	Y	Y	Y	-	-	-
ENSG00000204681	GABBR1	0.282732447	0.000552	0.199945872	0.036039	0.404502952	7.72E-07	-0.076285847	0.350263	0.136442232	0.061759	0.207246453	0.013285	C4	-	-	-	Y	-	-	-
ENSG00000204682	MIR1915HG	-0.485670636	0.01592	-0.356626583	0.126124	-0.109261101	0.677521	0.135360081	0.623198	0.390797553	0.068285	0.250155176	0.433494	C4	-	Y	-	-	-	-	-
ENSG00000204713	TRIM27	0.346454695	1.64E-12	0.005953029	0.962058	0.041993426	0.603322	-0.333748376	7.83E-11	-0.29002131	1.05E-11	0.038748823	0.755359	C5	-	Y	-	-	Y	-	-
ENSG00000204767	INSYN2B	1.490490883	6.70E-08	1.080595189	0.002899	1.294689182	4.11E-05	-0.407389022	0.10135	-0.181035678	0.48531	0.219535351	0.63838	C1	-	Y	Y	Y	-	-	-
ENSG00000204789	ZNF204P	-0.787379475	9.40E-05	-0.440054156	0.057445	-0.069900893	0.820922	0.353383549	0.162866	0.732165451	0.000897	0.373343689	0.229251	C4	-	Y	-	-	-	Y	-
ENSG00000204792	LINC01291	-0.919970737	0.000202	-0.606663181	0.041649	-0.547018391	0.042913	0.317042782	0.371821	0.387305277	0.225004	0.06448764	0.933535	C3	-	Y	Y	Y	-	-	-
ENSG00000204860	FAM201A	-1.494136902	0.016958	-1.09098975	0.075018	-0.537988391	0.379026	0.410802328	0.669049	0.971060818	0.186636	0.555620578	NA	C3	-	Y	-	-	-	-	-
ENSG00000204904	LINC01545	-1.602096486	0.301526	0.779417322	NA	-0.195543978	NA	2.383833329	0.036126	1.418120979	0.347744	-0.973022765	NA	C3	-	-	-	-	Y	-	-
ENSG00000204922	UQCC3	-0.414533954	0.013738	-0.25578994	0.23712	-0.251390276	0.166187	0.165526569	0.409008	0.178228218	0.30551	0.008040385	0.985228	C4	-	Y	-	-	-	-	-
ENSG00000204923	FBXO48	0.92692397	1.16E-09	-0.13456411	0.660103	0.514329059	0.006457	-1.05477349	1.62E-10	-0.398448723	0.012611	0.651601426	0.00412	C5	-	Y	-	Y	Y	Y	Y
ENSG00000204934	ATP6V0E2-AS1	0.290375223	0.037795	0.177312212	0.342167	0.370057434	0.018714	-0.106607381	0.532538	0.094352992	0.590594	0.195633789	0.398245	C4	-	-	-	Y	-	-	-
ENSG00000204946	ZNF783	-0.718531008	3.72E-22	-0.450821687	8.08E-07	-0.511106834	7.20E-11	0.274567665	0.0054	0.222130222	0.015354	-0.057587853	0.7794	C4	-	Y	Y	Y	-	-	-
ENSG00000204950	LRRC10B	-1.898748808	0.000223	-0.954272383	0.076599	-0.757439001	0.166776	0.954637357	0.184404	1.156330107	0.096768	0.197426807	NA	C3	-	Y	-	-	-	-	-
ENSG00000204954	C12orf73	0.348165367	0.003509	0.294518888	0.038314	0.233677697	0.088343	-0.0473364	0.754096	-0.099409618	0.437777	-0.0574568	0.808271	C5	-	Y	-	-	-	-	-
ENSG00000204962	PCDHA8	0.919681939	0.000173	0.561879887	0.112755	0.427070943	0.222245	-0.350037664	0.223355	-0.4772452	0.072334	-0.132085323	0.840232	C1	-	Y	-	-	-	-	-
ENSG00000205054	LINC01121	-3.731787125	0.036485	-0.39627959	NA	0.222529721	NA	3.358068636	NA	3.966867823	0.012506	0.636528562	NA	C3	-	Y	-	-	-	Y	-
ENSG00000205084	TMEM231	0.406648376	0.005699	0.305509189	0.169787	0.358253075	0.027192	-0.094273497	0.673747	-0.033519685	0.869531	0.055999415	0.896771	C1	-	Y	-	Y	-	-	-
ENSG00000205085	FAM71F2	-1.236360532	0.005381	-0.073607725	0.905766	-0.231231599	0.676857	1.16954476	0.015507	1.02050485	0.052634	-0.154770297	NA	C3	-	Y	-	-	Y	-	-
ENSG00000205086	C2orf91	-0.681716346	0.003996	0.129199601	0.708556	-0.054769558	0.883564	0.818313947	0.000633	0.641855021	0.012206	-0.181259647	0.678846	C4	-	Y	-	-	Y	Y	-
ENSG00000205116	TMEM88B	-1.231531484	0.193969	-0.048527684	NA	0.4758112	0.620225	1.191454642	0.18329	1.724087599	0.021662	0.521807081	NA	C2	-	-	-	-	-	Y	-
ENSG00000205126	ACCSL	-2.312086802	0.000139	-0.485735665	0.430744	-0.43939077	0.462412	1.834014194	0.008836	1.888265069	0.00668	0.049011783	NA	C3	-	Y	-	-	Y	Y	-
ENSG00000205189	ZBTB10	0.141790324	0.053607	-0.295776405	0.001062	-0.076100756	0.379301	-0.430929243	4.21E-08	-0.203295414	0.002316	0.222416821	0.033267	C5	-	-	-	-	Y	-	-
ENSG00000205212	CCDC144NL	-0.730526098	0.019251	-0.005066042	0.992467	0.041240612	0.929166	0.732230632	0.02317	0.785916067	0.012332	0.049251562	NA	C4	-	Y	-	-	Y	Y	-
ENSG00000205213	LGR4	-0.658203536	0.000263	-0.534269541	0.009763	-0.298312867	0.165428	0.130461534	0.633261	0.374590198	0.087763	0.238956961	0.497614	C4	-	Y	Y	-	-	-	-
ENSG00000205238	SPDYE2	-0.006689834	0.983334	0.471054397	0.058208	0.46451499	0.061666	0.483917795	0.034693	0.486130954	0.039925	-0.003518132	0.995923	C4	-	-	-	-	Y	Y	-
ENSG00000205269	TMEM170B	0.37208917	2.96E-06	0.277775907	0.001964	0.369814357	2.59E-05	-0.08798237	0.285784	0.012395525	0.909841	0.094936395	0.463858	C5	-	Y	-	Y	-	-	-
ENSG00000205309	NT5M	-0.439507346	0.010867	-0.220343385	0.366419	-0.167835194	0.450053	0.225397666	0.25692	0.287107751	0.098152	0.05612252	0.892655	C4	-	Y	-	-	-	-	-
ENSG00000205352	PRR13	-0.373508425	0.000963	-0.256918566	0.048287	-0.213915428	0.08684	0.123215624	0.323075	0.174762761	0.11904	0.046167623	0.838445	C4	-	Y	-	-	-	-	-
ENSG00000205423	CNEP1R1	0.298756742	0.010181	-0.131206649	0.434755	0.109603498	0.502213	-0.423506665	9.16E-05	-0.174237495	0.152405	0.243690367	0.132933	C5	-	-	-	-	Y	-	-
ENSG00000205464	ATP6AP1L	-0.453439159	0.002169	0.260780202	0.103056	0.138006699	0.417323	0.721335602	4.12E-07	0.606118352	1.83E-05	-0.119536065	0.611359	C2	-	Y	-	-	Y	Y	-
ENSG00000205517	RGL3	-0.148196122	0.219488	0.052764586	0.758965	0.164460113	0.188595	0.20740074	0.06957	0.327408253	0.00182	0.114443768	0.512021	C4	-	-	-	-	-	Y	-
ENSG00000205571	SMN2	0.173217602	0.114285	-0.043195361	0.810332	-0.210100515	0.116489	-0.21002765	0.040504	-0.369064145	0.000329	-0.164228207	0.336188	C5	-	-	-	-	-	Y	-
ENSG00000205583	STAG3L1	0.330312269	0.069358	0.457791514	0.012597	0.380181233	0.039116	0.133872806	0.526717	0.064280085	0.782664	-0.074740126	0.840067	C1	-	-	Y	Y	-	-	-
ENSG00000205593	DENND6B	-0.572693302	1.07E-05	-0.156318417	0.336401	-0.180123325	0.224782	0.422383091	0.002581	0.407493911	0.003502	-0.020928599	0.949752	C4	-	Y	-	-	Y	Y	-
ENSG00000205643	CDPF1	-0.231925571	0.218553	0.05971175	0.825483	0.111911216	0.614393	0.29854884	0.111081	0.358705197	0.040643	0.055288507	0.887679	C4	-	-	-	-	-	Y	-
ENSG00000205670	SMIM11A	-2.895870222	0.000403	-0.72585511	0.677618	-2.143708133	0.008031	2.185377155	0.006092	0.769443625	0.443124	-1.422336746	NA	C4	-	Y	-	Y	Y	-	-
ENSG00000205683	DPF3	-0.749146492	0.004612	0.203460155	0.520117	0.511291126	0.043136	0.958400633	0.000198	1.27490361	3.76E-07	0.31090731	0.392653	C4	-	Y	-	Y	Y	Y	-
ENSG00000205704	LINC00634	-1.134069538	0.001298	-0.00408532	0.994753	-0.228306098	0.568338	1.137834283	0.002826	0.920728637	0.016964	-0.221304912	NA	C3	-	Y	-	-	Y	Y	-
ENSG00000205707	ETFRF1	-0.238518568	0.170738	0.164489771	0.38913	-0.003032475	0.990999	0.409905618	0.011807	0.250447172	0.167664	-0.164646262	0.514285	C4	-	-	-	-	Y	-	-
ENSG00000205765	C5orf51	0.298558253	1.77E-07	-0.073853415	0.453868	-0.008445343	0.943368	-0.365555058	1.23E-11	-0.292319012	4.49E-08	0.068120648	0.576142	C5	-	-	-	-	Y	-	-
ENSG00000205791	LOH12CR2	-1.256558467	0.004598	-0.721110306	0.089526	-0.305116237	0.523106	0.539357221	0.369828	0.965240531	0.062249	0.419198313	NA	C3	-	Y	-	-	-	-	-
ENSG00000205913	SRRM2-AS1	-0.841100608	0.012826	-0.132433833	0.789116	-0.405485502	0.312246	0.714005692	0.057026	0.449844979	0.302961	-0.269841347	NA	C3	-	Y	-	-	-	-	-
ENSG00000205918	PDPK2P	0.734199056	1.76E-05	0.330708222	0.174791	0.49921998	0.023777	-0.396520742	0.009514	-0.220942342	0.207421	0.170836395	0.587615	C1	-	Y	-	Y	Y	-	-
ENSG00000205930	C21orf62-AS1	-0.131028719	0.743378	0.30580051	0.356641	0.479644214	0.086767	0.444635333	0.170584	0.625119667	0.035967	0.177034404	0.699353	C4	-	-	-	-	-	Y	-
ENSG00000205978	NYNRIN	-0.422725572	2.40E-05	-0.091803943	0.452399	0.117803162	0.345042	0.337618081	0.000579	0.555047855	2.24E-08	0.212379238	0.092592	C4	-	Y	-	-	Y	Y	-
ENSG00000206052	DOK6	0.472790591	1.85E-09	0.435654733	1.99E-06	0.186714538	0.083619	-0.031088962	0.762775	-0.271160825	0.000406	-0.24592591	0.022715	C1	-	Y	Y	-	-	-	-
ENSG00000206077	ZDHHC11B	-0.342044367	0.044238	-0.058095527	0.865848	0.021939917	0.939707	0.290907994	0.189937	0.378616036	0.033654	0.083261732	0.860933	C4	-	Y	-	-	-	Y	-
ENSG00000206127	GOLGA8O	-1.759319999	0.011327	0.133658167	0.875571	-0.212353427	0.795159	1.901911951	0.006604	1.560066335	0.047859	-0.343856785	NA	C3	-	Y	-	-	Y	Y	-
ENSG00000206417	H1-10-AS1	-0.616842826	0.004263	-0.210822413	0.468515	-0.316298235	0.203609	0.412292052	0.098889	0.315410438	0.229929	-0.10265368	0.834557	C4	-	Y	-	-	-	-	-
ENSG00000206418	RAB12	-0.036459825	0.776377	-0.175481602	0.170026	-0.433244904	0.000748	-0.132764202	0.16542	-0.38244891	0.000213	-0.255116819	0.106116	C5	-	-	-	Y	-	Y	-
ENSG00000206530	CFAP44	-0.446111395	3.59E-05	-0.068041639	0.685843	0.112509217	0.371226	0.385002611	0.001005	0.573130432	1.37E-08	0.183488684	0.231807	C4	-	Y	-	-	Y	Y	-
ENSG00000206535	LNP1	-1.266842766	9.62E-09	-0.394112224	0.092152	-0.600160087	0.005777	0.876337095	0.000965	0.681230975	0.016486	-0.20199044	0.645068	C3	-	Y	-	Y	Y	Y	-
ENSG00000206561	COLQ	0.504814536	0.041298	-0.019621337	0.971119	-0.025053428	0.957918	-0.517451725	0.089271	-0.515318791	0.070405	-0.004297391	0.996649	C5	-	Y	-	-	-	-	-
ENSG00000206573	THUMPD3-AS1	-0.343883194	0.000175	0.385115319	7.43E-05	-0.055368597	0.693824	0.735924092	1.58E-15	0.303161475	0.00289	-0.438077678	9.06E-05	C4	-	Y	Y	-	Y	Y	Y
ENSG00000206634	SNORA22	-1.4154245	0.009247	-0.765887783	0.212375	-0.651269425	0.312283	0.651166087	0.438529	0.77770875	0.339583	0.11874847	NA	C4	-	Y	-	-	-	-	-
ENSG00000206712	RNU6-26P	3.19560779	0.001272	1.799395001	NA	1.464166525	NA	-1.414948613	0.111147	-1.712131271	0.037131	-0.321732617	NA	C5	-	Y	-	-	-	Y	-

ENSG00000207399	RNU6-1011P	3.502589949	0.043154	2.5285275	NA	2.871337837	NA	-0.962927275	NA	-0.61281182	0.696131	0.343718538	NA	C1	-	Y	-	-	-	-	-
ENSG00000207554	MIR647	0.884754578	0.02075	0.727644956	0.172682	0.700558769	0.102931	-0.150148631	0.78531	-0.16925054	0.718034	-0.025515621	NA	C1	-	Y	-	-	-	-	-
ENSG00000210082	MT-RNR2	0.44657602	4.23E-42	0.70480931	7.39E-54	0.646354342	7.38E-56	0.265001762	3.75E-10	0.21459168	3.35E-09	-0.055625182	0.547978	C4	-	Y	Y	Y	-	-	-
ENSG00000211445	GPX3	-0.563509139	0.000339	-0.272884853	0.331751	-0.589825599	0.006114	0.299125378	0.252899	-0.011407067	0.970109	-0.317740802	0.478011	C4	-	Y	-	Y	-	-	-
ENSG00000211455	STK38L	0.302153476	9.37E-06	0.315796856	2.94E-05	0.313488866	0.000111	0.019952579	0.835411	0.026222081	0.795054	0.000617709	0.997573	C4	-	Y	Y	Y	-	-	-
ENSG00000211456	SACM1L	0.339821156	5.22E-07	0.051608284	0.633024	0.159655263	0.04709	-0.281883247	8.48E-06	-0.165560098	0.01279	0.110936961	0.269356	C5	-	Y	-	-	-	-	-
ENSG00000211459	MT-RNR1	0.99612573	4.73E-85	1.542909524	2.23E-212	1.424123229	7.80E-143	0.553444017	1.30E-51	0.443056266	1.10E-11	-0.115801567	0.082795	C1	-	Y	Y	Y	Y	Y	-
ENSG00000211584	SLC48A1	-0.233216573	0.06433	0.017614072	0.934379	0.224148359	0.081105	0.257716436	0.056374	0.472150524	5.66E-05	0.20952619	0.223156	C4	-	-	-	-	-	Y	-
ENSG00000211956	IGHV4-34	0.859939606	0.011894	0.198081575	0.736128	0.850920708	0.029519	-0.656000573	0.054835	0.004565392	0.991171	0.655330186	NA	C1	-	Y	-	Y	-	-	-
ENSG00000212123	PRR22	-0.966445578	0.001035	-0.413386975	0.223268	-0.653767747	0.036735	0.564572282	0.129886	0.327828306	0.440412	-0.240497583	NA	C3	-	Y	-	Y	-	-	-
ENSG00000212127	TAS2R14	-1.143243012	0.000586	-0.563220192	0.143187	-0.506978496	0.143721	0.590771627	0.208772	0.652337806	0.130364	0.058743772	NA	C3	-	Y	-	-	-	-	-
ENSG00000212694	LINC01089	-0.672885835	1.29E-08	0.209351278	0.076106	-0.057153215	0.708349	0.888927533	4.58E-16	0.630623509	5.53E-08	-0.263587071	0.031239	C2	-	Y	-	-	Y	Y	-
ENSG00000212719	LINC02693	0.266551384	0.045133	-0.153280294	0.437366	0.229515456	0.137173	-0.413450645	0.003271	-0.022477191	0.906639	0.385691607	0.032865	C5	-	-	-	-	Y	-	Y
ENSG00000212747	RTL8B	-0.578893484	6.30E-11	-0.306826412	0.002442	-0.499890985	2.41E-07	0.278684578	0.003089	0.093959183	0.426198	-0.190076944	0.159066	C4	-	Y	Y	Y	-	-	-
ENSG00000212766	EWSAT1	-4.12104879	0.002916	-0.371316168	NA	-1.413955192	NA	3.761185889	0.011608	2.721743719	NA	-1.041786891	NA	C3	-	Y	-	-	Y	-	-
ENSG00000212907	MT-ND4L	-0.416365607	3.01E-17	-0.641056974	1.75E-21	-0.538156193	2.93E-23	-0.217872302	2.95E-05	-0.106939202	0.000699	0.105873304	0.218545	C4	-	Y	Y	Y	-	-	-
ENSG00000212916	MAP10	-1.113839976	0.002076	0.10403427	0.824069	-0.175672304	0.674183	1.224055336	0.000529	0.953496953	0.011601	-0.276542255	NA	C3	-	Y	-	-	Y	Y	-
ENSG00000213015	ZNF580	-0.143035649	0.27723	0.172243229	0.22376	0.147079599	0.297097	0.321873221	0.001	0.305144682	0.001997	-0.022025525	0.929329	C4	-	-	-	-	Y	Y	-
ENSG00000213057	C1orf220	0.250172199	0.484422	0.681190125	0.036373	0.504187063	0.123804	0.437271658	0.156652	0.268210411	0.417974	-0.173252945	NA	C1	-	-	Y	-	-	-	-
ENSG00000213088	ACKR1	-1.054945802	0.020782	-0.197381631	0.757177	-0.013501967	0.984963	0.862653757	0.1063	1.055419214	0.041932	0.186984715	NA	C3	-	Y	-	-	-	Y	-
ENSG00000213096	ZNF254	0.048450577	0.616818	-0.321306232	0.000544	-0.135356255	0.166329	-0.363367279	1.23E-05	-0.16921694	0.047586	0.189046678	0.144337	C5	-	-	Y	-	Y	-	-
ENSG00000213123	TCTEX1D2	-0.350194555	0.046013	-0.045888779	0.884697	-0.121857246	0.607238	0.311557816	0.089881	0.24335663	0.168066	-0.07323935	0.849099	C4	-	Y	-	-	-	-	-
ENSG00000213145	CRIP1	-1.445066671	0.011652	-0.360356036	0.60504	-0.466737421	0.500604	1.095065547	0.094323	0.99271757	0.170933	-0.105736481	NA	C3	-	Y	-	-	-	-	-
ENSG00000213171	LINGO4	-1.200303721	1.80E-26	-0.059810409	0.72203	-0.280751172	0.008945	1.147767342	1.09E-22	0.934146725	1.83E-16	-0.217840318	0.13614	C3	-	Y	-	-	Y	Y	-
ENSG00000213185	FAM24B	0.543777476	0.036838	0.11367361	0.809619	-0.015529844	0.975362	-0.424483473	0.120183	-0.544489129	0.039798	-0.125939478	NA	C5	-	Y	-	-	-	Y	-
ENSG00000213199	ASIC3	-0.2666314	0.021324	0.154500633	0.268449	-0.036526712	0.849802	0.427641156	2.89E-05	0.245108489	0.049749	-0.187948093	0.264463	C2	-	-	-	-	Y	-	-
ENSG00000213221	DNLZ	-1.195805573	0.004028	-0.356547363	0.559714	-0.514513056	0.263042	0.842986313	0.101796	0.695796048	0.149433	-0.155339039	NA	C3	-	Y	-	-	-	-	-
ENSG00000213341	CHUK	0.524919487	6.61E-17	0.121022833	0.194308	0.106019363	0.250577	-0.397178343	2.92E-12	-0.404187262	1.69E-12	-0.01230195	0.947028	C5	-	Y	-	-	Y	Y	-
ENSG00000213366	GSTM2	-0.302543775	0.010172	-0.098026287	0.527249	-0.121055366	0.397525	0.210697299	0.079009	0.196111345	0.113097	-0.019916526	0.943834	C4	-	Y	-	-	-	-	-
ENSG00000213397	HAUS7	-0.6069053	0.0058	-0.218606117	0.461402	-0.511310219	0.020721	0.392244495	0.160181	0.109986463	0.741189	-0.28892161	0.470464	C4	-	Y	-	Y	-	-	-
ENSG00000213411	RBM22P2	-2.596336685	0.043684	-0.693120167	NA	-0.889485147	NA	1.897497293	NA	1.720491364	NA	-0.190329227	NA	C3	-	Y	-	-	-	-	-
ENSG00000213413	PVRIG	1.071089583	5.22E-05	0.618503629	0.092378	0.853795992	0.005315	-0.449042734	0.067782	-0.20344833	0.426819	0.239956214	0.581966	C1	-	Y	-	Y	-	-	-
ENSG00000213445	SIPA1	-0.260450909	0.005949	-0.001618657	0.994099	0.147913064	0.232515	0.265503936	0.01713	0.423010279	1.82E-05	0.152072923	0.413415	C4	-	-	-	-	-	Y	-
ENSG00000213462	ERV3-1	0.309884866	0.005285	0.252703396	0.072637	0.320442893	0.017985	-0.051708282	0.598583	0.024965313	0.834491	0.070771526	0.711161	C4	-	Y	-	Y	-	-	-
ENSG00000213465	ARL2	-0.328721718	0.001857	-0.059602242	0.739578	-0.085875421	0.566145	0.276060857	0.008267	0.257727831	0.0113	-0.02381374	0.931126	C4	-	Y	-	-	-	-	-
ENSG00000213599	SLX1A-SULT1A3	0.432656351	0.076744	-0.219051714	0.592838	-0.027157014	0.949421	-0.645565171	0.01943	-0.445223062	0.089124	0.194998652	NA	C5	-	-	-	-	Y	-	-
ENSG00000213625	LEPROT	-0.331355827	1.54E-06	-0.011429343	0.930488	-0.104572898	0.264735	0.326368256	2.74E-06	0.241511411	0.001198	-0.09030154	0.482463	C4	-	Y	-	-	Y	-	-
ENSG00000213672	NCKIPSD	0.263528113	0.046578	0.376427019	0.006095	0.361010622	0.005061	0.119577544	0.269594	0.11276658	0.270532	-0.011945083	0.958884	C1	-	-	Y	Y	-	-	-
ENSG00000213693	SEC14L1P1	-0.75279785	0.025659	-0.358378453	0.341097	-0.607447786	0.097631	0.401382135	0.326518	0.159859866	0.759737	-0.246751773	NA	C3	-	Y	-	-	-	-	-
ENSG00000213694	S1PR3	-1.261591271	1.08E-30	-0.174899249	0.172613	-0.408648218	0.000129	1.093025158	2.01E-20	0.867542002	1.81E-12	-0.230927474	0.137108	C3	-	Y	-	Y	Y	Y	-
ENSG00000213699	SLC35F6	0.308538993	6.27E-06	-0.015219977	0.906151	0.117667607	0.288741	-0.317335448	4.40E-06	-0.176392548	0.041186	0.135938001	0.317364	C5	-	Y	-	-	Y	-	-
ENSG00000213780	GTF2H4	0.447870229	0.003195	0.07411504	0.776034	0.123677057	0.573772	-0.367244482	0.02234	-0.30915425	0.057573	0.052513109	0.894358	C5	-	Y	-	-	Y	-	-
ENSG00000213782	DDX47	0.225907301	0.180143	0.372372812	0.035167	-0.102034722	0.698563	0.152863164	0.386015	-0.313053194	0.088631	-0.471489306	0.028724	C2	-	-	Y	-	-	-	Y
ENSG00000213799	ZNF845	-0.031229492	0.844527	-0.388534018	0.013622	-0.112705547	0.497349	-0.35062311	0.013285	-0.066796116	0.680198	0.278884695	0.188711	C5	-	-	Y	-	Y	-	-
ENSG00000213830	CFL1P5	1.272219041	0.047783	-0.259222989	NA	0.13964172	0.9062	-1.523508327	0.037487	-1.119050341	0.092752	0.402158985	NA	C5	-	Y	-	-	Y	-	-
ENSG00000213839	TMX2P1	0.520074846	0.015075	0.175431079	0.609705	0.131260881	0.658342	-0.338277411	0.159921	-0.373706061	0.074444	-0.041466028	0.943467	C5	-	Y	-	-	-	-	-
ENSG00000213888	LINC01521	-0.943245876	9.36E-10	-0.294746355	0.061856	-0.374947699	0.008841	0.655611118	2.19E-05	0.582475577	0.000152	-0.076873148	0.777401	C4	-	Y	-	Y	Y	Y	-
ENSG00000213903	LTB4R	-0.615986157	1.98E-07	-0.142840976	0.368165	-0.248823746	0.065181	0.479885443	0.000224	0.381815819	0.004724	-0.103186814	0.65992	C2	-	Y	-	-	Y	Y	-
ENSG00000213918	DNASE1	0.251658642	0.000883	0.304645128	0.000717	0.390523914	2.13E-06	0.058864671	0.519367	0.153690134	0.038227	0.088984571	0.514931	C4	-	-	Y	Y	-	-	-
ENSG00000213928	IRF9	0.342655522	0.160948	-0.149770351	0.684954	0.247700893	0.400181	-0.485326416	0.030682	-0.079557768	0.780192	0.40014735	0.19895	C5	-	-	-	-	Y	-	-
ENSG00000213977	TAX1BP3	-0.532027202	0.002132	-0.007717042	0.98135	-0.188283538	0.408355	0.531141103	0.001581	0.35870157	0.042617	-0.177391118	0.543441	C4	-	Y	-	-	Y	Y	-
ENSG00000213988	ZNF90	-0.29756377	0.012944	-0.2995688	0.044607	-0.321036336	0.014787	0.004222785	0.98186	-0.008859575	0.960892	-0.018645225	0.959272	C4	-	-	-	Y	-	-	-
ENSG00000213996	TM6SF2	-0.415231262	0.091006	0.177553654	0.565203	-0.128520308	0.712908	0.599384946	0.003175	0.301910598	0.246183	-0.303219595	0.348389	C4	-	-	-	-	Y	-	-
ENSG00000214021	TTLL3	0.421654342	1.39E-06	0.19072208	0.099486	0.097771193	0.453829	-0.224479306	0.019106	-0.309207775	0.000771	-0.090155308	0.619333	C5	-	Y	-	-	-	Y	-
ENSG00000214026	MRPL23	-0.44535554	0.022965	-0.364907126	0.050714	-0.247928082	0.312136	0.087188579	0.703857	0.212968876	0.35787	0.120190299	0.736482	C4	-	Y	-	-	-	-	-
ENSG00000214029	ZNF891	0.49323146	0.000106	0.549890606	2.28E-05	0.683631606	2.29E-07	0.06327683	0.578364	0.205371599	0.051782	0.136713501	0.378696	C1	-	Y	Y	Y	-	-	-
ENSG00000214043	LINC02347	-1.799641324	0.015782	-0.178024118	0.863828	-0.58119778	0.441446	1.62329776	0.064443	1.231976597	0.179882	-0.399123242	NA	C3	-	Y	-	-	-	-	-
ENSG00000214046	SMIM7	-0.203994669	0.034319	-0.048291451	0.720969	-0.315175659	0.001334	0.163168339	0.144891	-0.096546995	0.450266	-0.264826398	0.051813	C4	-	-	-	Y	-	-	-
ENSG00000214063	TSPAN4	-0.296016407	0.016739	0.081727755	0.70052	0.099728802	0.549054	0.38447237	0.001653	0.410719546	8.95E-05	0.021192539	0.948558	C4	-	-	-	-	Y	Y	-
ENSG00000214100	PLAC9P1	-1.118867576	0.008153	-0.822613665	0.112752	-0.414696153	0.432025	0.299243435	0.651297	0.719230739	0.168851	0.412015879	NA	C3	-	Y	-	-	-	-	-
ENSG00000214140	PRCD	-1.045725388	2.31E-44	-0.026555325	0.834963	-0.218565869	0.009808	1.025733088	1.73E-42	0.842045538	2.84E-26	-0.18903552	0.063593	C2	-	Y	-	-	Y	Y	-
ENSG00000214174	AMZ2P1	-0.615790473	7.05E-14	-0.228096953	0.033391	-0.2909834	0.001756	0.394406464	3.96E-05	0.33955431	0.000244	-0.060072795	0.766716	C4	-	Y	-	-	Y	Y	-
ENSG00000214176	PLEKHM1P1	0.156881091	0.214786	0.309418704	0.027627	0.113462918	0.456775	0.157615012	0.141224	-0.028863406	0.818325	-0.191974107	0.176802	C4	-	-	Y	-	-	-	-
ENSG00000214182	PTMAP5	-0.091884216	0.633732	-0.424453816	0.014842	-0.254731229	0.173509	-0.326369764	0.021487	-0.148346941	0.359813	0.172659652	0.473243	C5	-	-	Y	-	Y	-	-
ENSG00000214184	GCC2-AS1	-1.915429807	0.003435	-1.046026	0.123043	-1.020064312	0.147796	0.884189781	0.339565	0.908478495	0.348265	0.021603574	NA	C3	-	Y	-	-	-	-	-
ENSG00000214193	SH3D21	-0.37819815	0.002149	0.186888949	0.308914	0.392418941	0.003958	0.57152795	1.78E-05	0.785302956	2.39E-11	0.208165768	0.349512	C4	-	Y	-	Y	Y	Y	-
ENSG00000214194	SMIM30	-0.384347907	2.86E-05	-0.057530732	0.654586	-0.144492183	0.133209	0.333425264	0.0003	0.254568996	0.00595	-0.083949424	0.561947	C4	-	Y	-	-	Y	-	-

ENSG00000214226	C17orf67	0.535072878	0.021492	0.252139424	0.33553	0.469137431	0.018131	-0.274548195	0.299527	-0.05162727	0.866223	0.220365627	0.491439	C1	-	Y	-	Y	-	-	-
ENSG00000214357	NEURL1B	-0.426768551	1.09E-07	-0.286165186	0.001759	-0.18094602	0.035328	0.147426198	0.142505	0.26061039	0.001598	0.108368256	0.432722	C4	-	Y	-	-	-	-	-
ENSG00000214367	HAUS3	0.273998084	0.000417	-0.125468144	0.320949	0.076405748	0.493675	-0.392585808	2.00E-06	-0.183033535	0.017649	0.204355976	0.097949	C5	-	-	-	-	Y	-	-
ENSG00000214376	VSTM5	-2.677669869	3.59E-06	-1.329028667	0.010274	-1.622417055	0.001329	1.363328338	0.105166	1.070266915	0.246044	-0.295369374	NA	C3	-	Y	Y	Y	-	-	-
ENSG00000214439	FAM185BP	0.870026792	0.003131	0.460982568	0.294925	0.840669633	0.011374	-0.404843024	0.19427	-0.014682175	0.966844	0.384123192	NA	C1	-	Y	-	Y	-	-	-
ENSG00000214455	RCN1P2	2.404053186	0.003672	0.704665527	NA	0.847094927	NA	-1.695584774	0.029867	-1.543626588	0.036361	0.14635651	NA	C5	-	Y	-	-	Y	Y	-
ENSG00000214456	PLIN5	-0.510374778	0.076575	0.136177285	0.723794	0.225025317	0.488821	0.654186089	0.017666	0.750792926	0.0048	0.092101418	0.869474	C4	-	-	-	-	Y	Y	-
ENSG00000214517	PPME1	0.332391541	5.58E-09	0.182678835	0.003563	0.133333251	0.036735	-0.143095717	0.014933	-0.184278384	0.000841	-0.04637317	0.64974	C5	-	Y	-	-	-	-	-
ENSG00000214530	STARD10	-0.257629887	0.154137	0.114989164	0.63096	0.116978913	0.601809	0.379199628	0.028523	0.389405229	0.023944	0.00499768	0.991437	C4	-	-	-	-	Y	Y	-
ENSG00000214562	NUTM2D	-0.875312034	4.41E-05	-0.411422593	0.06657	-0.445756877	0.048048	0.470076587	0.069186	0.444330098	0.10277	-0.031340942	0.958405	C3	-	Y	-	Y	-	-	-
ENSG00000214575	CPEB1	-0.596172304	0.048855	-0.231633479	0.575102	-0.088587376	0.841632	0.371470358	0.29437	0.522790317	0.113812	0.145794199	NA	C4	-	Y	-	-	-	-	-
ENSG00000214595	EML6	0.417589456	3.04E-05	0.397671282	0.000995	0.304554352	0.019695	-0.012859114	0.930508	-0.098945973	0.415464	-0.0903372	0.674578	C5	-	Y	Y	Y	-	-	-
ENSG00000214652	ZNF727	0.130494823	0.172729	-0.18537946	0.103056	0.088942304	0.54274	-0.309314443	0.001188	-0.026770456	0.858161	0.277146926	0.066193	C5	-	-	-	-	Y	-	-
ENSG00000214783	POLR2J4	-0.33474087	0.040648	0.06673309	0.785715	-0.023568056	0.923979	0.407387965	0.013917	0.325806053	0.054978	-0.087547133	0.781334	C4	-	Y	-	-	Y	-	-
ENSG00000214826	DDX12P	0.495981226	7.47E-14	0.245426868	0.021149	0.161668867	0.078393	-0.243801163	0.003669	-0.319885279	3.78E-06	-0.080769106	0.648243	C5	-	Y	-	-	-	Y	-
ENSG00000214975	PPIAP29	-1.3148141	0.008641	-0.454178882	0.471998	-1.049118462	0.029717	0.871823527	0.192085	0.28114669	0.730218	-0.594717693	NA	C3	-	Y	-	Y	-	-	-
ENSG00000215041	NEURL4	0.448688371	3.22E-10	0.250570034	0.004164	0.244245171	0.002104	-0.191546804	0.003013	-0.190205272	0.001607	-0.004052953	0.982152	C1	-	Y	-	-	-	-	-
ENSG00000215045	GRID2IP	-0.731440403	0.00627	-0.438095087	0.175557	-0.086311988	0.819199	0.300568515	0.40882	0.66001633	0.023486	0.354669193	NA	C4	-	Y	-	-	-	Y	-
ENSG00000215067	ALOX12-AS1	-0.047988045	0.899952	0.601142787	0.057569	0.318296022	0.327779	0.656342951	0.027353	0.380911525	0.215638	-0.280910849	0.59011	C2	-	-	-	-	Y	-	-
ENSG00000215218	UBE2QL1	-0.578656701	7.55E-36	-0.246252608	0.000526	-0.272366022	3.29E-06	0.339220051	1.33E-09	0.320989419	3.66E-11	-0.023194371	0.887679	C4	-	Y	-	-	Y	Y	-
ENSG00000215251	FASTKD5	-0.04818604	0.58908	-0.268184639	0.003276	-0.315312104	4.54E-05	-0.213533765	0.009776	-0.252582771	0.000675	-0.044129638	0.818037	C4	-	-	-	Y	-	-	-
ENSG00000215256	DHRS4-AS1	-0.536319729	7.65E-06	-0.147354897	0.385738	-0.054134079	0.751709	0.394960776	0.000606	0.496945425	7.68E-07	0.097349654	0.609142	C4	-	Y	-	-	Y	Y	-
ENSG00000215270	TOMM40P2	1.740057094	1.34E-11	0.655196549	0.048842	0.812801927	0.008802	-1.078711303	2.02E-05	-0.912225327	0.000353	0.160501829	NA	C5	-	Y	Y	Y	Y	Y	-
ENSG00000215271	HOMEZ	0.326396259	0.008516	0.210893122	0.22376	0.570438815	1.75E-06	-0.109561807	0.463605	0.258649644	0.019572	0.363104928	0.011514	C4	-	Y	-	Y	-	-	Y
ENSG00000215301	DDX3X	-0.079882344	0.102829	-0.103097341	0.068554	-0.302237805	2.22E-12	-0.016730726	0.715703	-0.207805125	9.89E-14	-0.196377125	2.16E-08	C4	-	-	-	Y	-	-	-
ENSG00000215375	MYL5	-0.982785185	1.71E-11	0.000702327	0.997464	-0.183394954	0.373371	0.990452603	3.88E-11	0.814232638	7.42E-06	-0.181921462	0.519301	C3	-	Y	-	-	Y	Y	-
ENSG00000215386	MIR99AHG	-0.843632158	4.14E-07	-0.096525029	0.713114	0.016302792	0.950834	0.755307649	0.000106	0.874966839	7.15E-07	0.115579271	0.746187	C3	-	Y	-	-	Y	Y	-
ENSG00000215397	SCRT2	0.037517962	0.819346	-0.292470839	0.056834	-0.132291084	0.442181	-0.323587223	0.012791	-0.155224613	0.281053	0.163126579	0.482772	C4	-	-	-	-	Y	-	-
ENSG00000215417	MIR17HG	-0.765675503	2.33E-08	0.174375309	0.273993	-0.56843741	0.000354	0.946278082	1.30E-13	0.212196056	0.279857	-0.739964691	1.22E-06	C2	-	Y	-	Y	Y	-	Y
ENSG00000215424	MCM3AP-AS1	-0.165791746	0.194884	0.069603405	0.659354	0.233526296	0.053364	0.241698243	0.026917	0.413885289	7.81E-05	0.16695312	0.250087	C4	-	-	-	-	-	Y	-
ENSG00000215440	NPEPL1	0.092884143	0.661729	-0.045227288	0.886728	-0.556020064	0.003611	-0.130352822	0.507777	-0.633678732	4.47E-05	-0.508592166	0.017184	C5	-	-	-	Y	-	Y	Y
ENSG00000215478	CES5AP1	-0.789196759	0.068748	0.194265308	0.743135	0.195830913	0.705378	0.989743907	0.01895	1.000794613	0.01328	0.003705762	NA	C3	-	-	-	-	Y	Y	-
ENSG00000215788	TNFRSF25	-0.378039133	0.000416	-0.401700877	0.001601	-0.129700546	0.307844	-0.017159509	0.930601	0.263122294	0.024007	0.274783165	0.09071	C4	-	Y	Y	-	-	-	-
ENSG00000215883	CYB5RL	-0.231458185	0.07042	-0.029139577	0.891637	0.217143927	0.103461	0.208547789	0.122907	0.463136845	4.54E-05	0.248931458	0.127801	C4	-	-	-	-	-	Y	-
ENSG00000215912	TTC34	0.1669547	0.866041	1.98341606	0.000142	1.337615109	0.026374	1.821428278	0.000198	1.183341582	0.039775	-0.640750189	NA	C2	-	-	Y	Y	Y	Y	-
ENSG00000216937	CCDC7	-0.643103606	0.02692	-0.331373066	0.326555	0.130200303	0.709445	0.319475865	0.387017	0.787592841	0.00634	0.464264482	NA	C4	-	Y	-	-	-	Y	-
ENSG00000217128	FNIP1	0.277437616	0.003863	0.191721629	0.113958	-0.039244576	0.77627	-0.079245024	0.514887	-0.302165987	0.00057	-0.228415828	0.066219	C5	-	-	-	-	-	Y	-
ENSG00000218336	TENM3	-0.438151006	1.60E-08	0.132838456	0.29165	0.017181554	0.896648	0.577546727	9.22E-13	0.470043962	1.85E-13	-0.112793238	0.43015	C4	-	Y	-	-	Y	Y	-
ENSG00000218510	LINC00339	-0.262405018	0.206032	0.087045071	0.713114	0.247653795	0.154797	0.355069675	0.076732	0.524719037	0.003994	0.163482731	0.528427	C4	-	-	-	-	-	Y	-
ENSG00000218739	CEBPZOS	-0.365527829	2.32E-08	-0.045267924	0.66931	-0.106530719	0.258174	0.326642492	6.93E-09	0.273847056	2.53E-05	-0.058272902	0.641821	C4	-	Y	-	-	Y	-	-
ENSG00000218891	ZNF579	0.405040188	0.000141	0.00483435	0.98251	0.091430341	0.559599	-0.393421498	0.00011	-0.298963859	0.003186	0.089321034	0.661027	C5	-	Y	-	-	Y	-	-
ENSG00000219102	HNRNPA3P12	3.515228132	0.045099	3.170879152	NA	3.807512398	NA	-0.331479226	NA	0.307254375	0.831337	0.630980482	NA	C5	-	Y	-	-	-	-	-
ENSG00000219481	NBPF1	0.529474173	4.20E-13	0.301231891	0.000329	0.345796479	5.40E-10	-0.221738195	0.001212	-0.061111793	0.435326	0.155096533	0.092206	C5	-	Y	Y	Y	-	-	-
ENSG00000219626	FAM228B	-0.188820801	0.213798	0.173661527	0.301196	0.116095026	0.4628	0.368845097	0.004967	0.319334137	0.009764	-0.054448254	0.832353	C4	-	-	-	-	Y	Y	-
ENSG00000219891	ZSCAN12P1	-0.405610853	0.090374	0.068287704	0.824778	0.220118059	0.360294	0.480738138	0.024956	0.640123462	0.001938	0.155303566	0.619596	C2	-	-	-	-	Y	Y	-
ENSG00000220205	VAMP2	-0.307451974	1.50E-05	0.029912498	0.829709	-0.117088437	0.15123	0.343814757	6.73E-06	0.205213763	0.002438	-0.144074475	0.191653	C4	-	Y	-	-	Y	-	-
ENSG00000220744	RPL5P18	2.962419582	0.024664	2.707636633	NA	2.499687703	NA	-0.26663776	0.831394	-0.447464935	0.689915	-0.190304815	NA	C1	-	Y	-	-	-	-	-
ENSG00000220785	MTMR9LP	-0.705011831	0.01079	-0.079331906	0.815068	-0.158164974	0.588927	0.630808568	0.035062	0.562386608	0.068332	-0.075215171	0.894304	C3	-	Y	-	-	Y	-	-
ENSG00000220804	LINC01881	0.67388186	0.006867	0.379960269	0.272152	0.473250591	0.078939	-0.287633543	0.335853	-0.186045814	0.505943	0.09614079	0.868641	C1	-	Y	-	-	-	-	-
ENSG00000221817	PPP3CB-AS1	-0.546855592	0.030763	-0.204466689	0.588681	-0.058204814	0.881222	0.347533046	0.294032	0.503189448	0.07843	0.149469629	0.79892	C4	-	Y	-	-	-	-	-
ENSG00000221819	GAS8-AS1	-1.188054659	0.0176	0.271476961	0.64429	0.070061799	0.922342	1.468540429	0.002282	1.272020631	0.017248	-0.200102495	NA	C3	-	Y	-	-	Y	Y	-
ENSG00000221821	C6orf226	-0.677407598	0.011258	0.005374144	0.991997	-0.340603908	0.24456	0.690189008	0.016887	0.351984213	0.245862	-0.342422664	0.416552	C2	-	Y	-	-	Y	-	-
ENSG00000221843	C2orf16	-0.490953416	0.018369	0.067477463	0.832941	0.37212577	0.071861	0.564938592	0.004133	0.877635764	9.34E-07	0.30813853	0.224782	C4	-	Y	-	-	Y	Y	-
ENSG00000221866	PLXNA4	0.18725901	0.016962	0.566198918	2.37E-05	0.395859013	0.004189	0.38550374	1.85E-05	0.223029421	0.019334	-0.167748163	0.393747	C4	-	-	Y	Y	Y	-	-
ENSG00000221886	ZBED8	0.372279426	0.009326	-0.080757088	0.752289	0.377756707	0.038946	-0.446331225	0.000774	0.019455244	0.924199	0.461314437	0.01688	C5	-	Y	-	Y	Y	-	Y
ENSG00000221890	NPTXR	-0.479796786	1.05E-05	-0.177011393	0.166692	-0.181004	0.180327	0.309328491	0.009945	0.31354727	0.01475	-0.001096846	0.997282	C4	-	Y	-	-	Y	Y	-
ENSG00000221909	FAM200A	0.176141975	0.038588	-0.348340794	0.000755	-0.059564989	0.619366	-0.518002036	5.73E-09	-0.221145632	0.007742	0.291614844	0.016243	C5	-	-	Y	-	Y	-	-
ENSG00000221914	PPP2R2A	0.32393529	1.08E-08	0.048234008	0.685638	0.064237253	0.434344	-0.269024622	5.13E-05	-0.24496101	5.79E-06	0.018744628	0.91973	C5	-	Y	-	-	-	-	-
ENSG00000221923	ZNF880	-0.419683607	0.000879	0.067390339	0.720479	-0.026980874	0.884094	0.492812152	0.000111	0.407320302	0.001011	-0.091911606	0.676845	C4	-	Y	-	-	Y	Y	-
ENSG00000221990	EXOC3-AS1	-1.180222261	1.36E-10	-0.348292131	0.092971	-0.347698486	0.063445	0.83939173	7.56E-05	0.846852526	3.22E-05	0.003188103	0.995777	C2	-	Y	-	-	Y	Y	-
ENSG00000222009	BTBD19	-0.694584088	0.012945	-0.386123872	0.16678	-0.335798046	0.215902	0.3136871	0.339565	0.372367053	0.233545	0.053024372	0.930139	C2	-	Y	-	-	-	-	-
ENSG00000222267	RNU6-892P	-4.259956472	0.023374	-1.057516563	NA	-2.329698548	NA	3.221616757	NA	1.944282946	NA	-1.273664093	NA	C4	-	Y	-	-	-	-	-
ENSG00000223442	TH2LCRR	-0.914980235	0.211113	0.503692799	0.486511	-0.439861111	0.625095	1.42927103	0.038146	0.486901294	0.644342	-0.945481639	NA	C2	-	-	-	-	Y	-	-
ENSG00000223459	TCAF1P1	0.244534934	0.047365	0.247795473	0.065915	0.308480321	0.012786	0.009193942	0.951638	0.07838126	0.511913	0.064002174	0.734205	C4	-	-	-	Y	-	-	-
ENSG00000223501	VPS52	-0.233737225	0.000962	0.106493149	0.203255	0.005596864	0.964182	0.346667521	2.69E-08	0.253883559	0.000151	-0.098102049	0.326733	C4	-	-	-	-	Y	-	-
ENSG00000223508	RPL23AP53	0.504001816	0.161572	0.481105252	0.22649	0.655134925	0.046864	-0.015707141	0.972717	0.166229684	0.667549	0.178288514	NA	C1	-	-	-	Y	-	-	-
ENSG00000223546	LINC00630	-0.344024021	0.123591	-0.187367086	0.523667	0.111501888	0.693638	0.162563113	0.517337	0.469702404	0.015446	0.302844973	0.28183	C4	-	-	-	-	-	Y	-

ENSG00000223547	ZNF844	-0.206837779	0.309258	0.179919129	0.514739	0.172884036	0.45592	0.394410482	0.066345	0.394549623	0.041684	-0.004333324	0.994294	C4	-	-	-	-	-	Y	-
ENSG00000223573	TINCR	1.611911688	0.015431	0.788951795	NA	1.033770607	0.20159	-0.814702522	0.21738	-0.563813942	0.395873	0.244590713	NA	C5	-	Y	-	-	-	-	-
ENSG00000223611	SUPT20HL2	-1.319419198	0.085816	-0.0386547	0.972405	0.211167502	0.787413	1.294883438	0.144269	1.545070447	0.037334	0.252992387	NA	C2	-	-	-	-	-	Y	-
ENSG00000223658	C1GALT1C1L	-1.089565045	3.82E-07	-0.36709657	0.127792	-0.599941341	0.003913	0.729952838	0.00357	0.504294002	0.05646	-0.230091095	0.544646	C3	-	Y	-	Y	Y	-	-
ENSG00000223705	NSUN5P1	0.132947078	0.027453	0.388882247	1.27E-11	0.325351479	9.91E-09	0.26237568	1.17E-06	0.20715821	0.000125	-0.060638773	0.536385	C4	-	-	Y	Y	-	-	-
ENSG00000223745	CCDC18-AS1	-0.532312476	9.68E-09	0.132693247	0.276955	0.077743284	0.560149	0.671795535	4.72E-13	0.624748457	3.65E-11	-0.052289299	0.804185	C2	-	Y	-	-	Y	Y	-
ENSG00000223764	LINC02593	-0.711889082	3.19E-20	0.025049304	0.851663	-0.165346269	0.077608	0.743637984	7.93E-29	0.561513874	1.11E-14	-0.187564391	0.040262	C2	-	Y	-	-	Y	Y	-
ENSG00000223768	LINC00205	-0.130183392	0.061545	0.17511003	0.018373	0.343706569	6.22E-08	0.311463952	3.48E-08	0.48851756	8.37E-21	0.171747852	0.016999	C4	-	-	-	Y	Y	Y	-
ENSG00000223791	UBE2E1-AS1	-0.708326881	0.188797	-0.22180169	0.766872	0.382844691	0.476489	0.495472274	0.459522	1.105354673	0.028042	0.607297269	NA	C2	-	-	-	-	-	Y	-
ENSG00000223797	ENTPD3-AS1	-1.058647274	0.003027	-0.203802679	0.700143	-0.312316675	0.460236	0.863423212	0.026618	0.761897559	0.044436	-0.104043978	NA	C3	-	Y	-	-	Y	Y	-
ENSG00000223802	CERS1	0.479000004	0.015147	0.113799733	0.729423	0.297899837	0.25157	-0.359294772	0.027775	-0.166681618	0.381594	0.187294819	0.541126	C1	-	Y	-	-	Y	-	-
ENSG00000223891	OSER1-DT	-0.434418728	0.004994	-0.133345943	0.549224	-0.034594224	0.901695	0.308123843	0.045009	0.415564543	0.010787	0.102131307	0.753054	C4	-	Y	-	-	Y	Y	-
ENSG00000223960	CHROMR	0.215327534	0.00255	0.269730487	0.003684	0.355029388	1.94E-07	0.060717106	0.526757	0.154183981	0.013755	0.088536127	0.491137	C4	-	-	-	Y	-	-	-
ENSG00000223979	SMCR2	-1.668378906	0.038033	-0.53773988	NA	-1.140347704	0.181726	1.137443023	0.216971	0.544582469	0.64424	-0.598956997	NA	C4	-	Y	-	-	-	-	-
ENSG00000224020	MIR181A2HG	-0.903446274	0.012703	-0.430695923	0.293364	-0.43866481	0.374827	0.480511625	0.179308	0.480130425	0.281225	-0.004891021	NA	C3	-	Y	-	-	-	-	-
ENSG00000224023	EDRF1-DT	-1.447430829	0.013098	-0.573707871	0.487356	-0.376835336	0.631596	0.88422968	0.283207	1.084094871	0.138774	0.194135301	NA	C3	-	Y	-	-	-	-	-
ENSG00000224043	CCNT2-AS1	-0.518181596	0.020331	-0.141037956	0.655058	-0.279405687	0.327437	0.382421936	0.124796	0.253191278	0.381952	-0.134643768	0.785782	C4	-	Y	-	-	-	-	-
ENSG00000224078	SNHG14	-0.237871599	0.001613	-0.085205348	0.456815	0.113643947	0.191782	0.159131492	0.042476	0.366099204	1.04E-09	0.201751024	0.029061	C4	-	-	-	-	-	Y	-
ENSG00000224165	DNAJC27-AS1	-0.698758597	0.085983	-0.011950329	0.98614	0.153413141	0.762258	0.694688357	0.101083	0.867113904	0.027203	0.169023712	NA	C3	-	-	-	-	-	Y	-
ENSG00000224183	SDHDP6	0.361899481	0.211434	-0.29316473	0.453868	-0.126177908	0.779536	-0.649086559	0.029699	-0.473127602	0.146923	0.170271915	NA	C5	-	-	-	-	Y	-	-
ENSG00000224189	HAGLR	-0.613182103	9.01E-12	-0.191789845	0.083905	0.019913752	0.900258	0.427993318	1.04E-05	0.647675573	2.42E-12	0.214575235	0.102519	C4	-	Y	-	-	Y	Y	-
ENSG00000224222	LINC02636	-4.150950901	0.037904	-1.49196853	NA	-1.003715512	NA	2.647742142	NA	3.16350031	NA	0.491025977	NA	C4	-	Y	-	-	-	-	-
ENSG00000224281	SLC25A5-AS1	-0.502225046	0.197422	0.192040856	0.701441	-0.039850836	0.945618	0.700688063	0.0468	0.478181657	0.237383	-0.228495244	NA	C2	-	-	-	-	Y	-	-
ENSG00000224294	PINCR	-2.230857529	0.054124	0.325577987	NA	0.294425143	NA	2.562483427	0.020912	2.538611497	0.030308	-0.027472788	NA	C2	-	-	-	-	Y	Y	-
ENSG00000224383	PRR29	-1.180966716	0.001129	-0.745365599	0.052135	-0.271020303	0.53117	0.439921083	0.376949	0.923364007	0.024355	0.477141563	NA	C4	-	Y	-	-	-	Y	-
ENSG00000224424	PRKAR2A-AS1	-1.422680109	0.031388	-0.10552253	0.92055	0.018639077	0.98584	1.326487998	0.082847	1.455856641	0.052411	0.124066776	NA	C3	-	Y	-	-	-	-	-
ENSG00000224577	LINC01117	-0.758860513	0.037397	0.050250144	0.930817	0.146247896	0.768497	0.815283777	0.026083	0.920659598	0.011649	0.099118926	NA	C2	-	Y	-	-	Y	Y	-
ENSG00000224596	ZMIZ1-AS1	-0.979374847	0.01255	-0.181308304	0.758224	-0.445311794	0.329859	0.804098779	0.08518	0.548577324	0.274513	-0.260906863	NA	C2	-	Y	-	-	-	-	-
ENSG00000224597	SVIL-AS1	0.624412084	2.10E-09	0.29583771	0.038822	0.436808763	0.001295	-0.321780615	0.003989	-0.173347371	0.177568	0.144250211	0.524775	C5	-	Y	-	Y	Y	-	-
ENSG00000224609	HSD52	-1.642377168	0.003103	-0.381818865	0.573795	-0.84815267	0.121147	1.263228263	0.059246	0.809937066	0.283456	-0.461109458	NA	C3	-	Y	-	-	-	-	-
ENSG00000224660	SH3BP5-AS1	-0.539640412	7.78E-06	-0.03246289	0.876915	-0.225846116	0.092134	0.513685114	1.94E-05	0.32817817	0.008088	-0.190534912	0.274702	C4	-	Y	-	-	Y	Y	-
ENSG00000224729	PCOLCE-AS1	-1.103753137	0.000785	-0.429434992	0.254813	-0.529081565	0.070814	0.683232932	0.094323	0.589247001	0.118292	-0.097074301	NA	C3	-	Y	-	-	-	-	-
ENSG00000224825	RORB-AS1	0.484219055	0.020304	0.374288536	0.147252	0.147433	0.714444	-0.103562274	0.696104	-0.322855493	0.261451	-0.225079312	0.651132	C1	-	Y	-	-	-	-	-
ENSG00000224831	TMEM183B	0.460123346	0.019001	0.148577484	0.632235	0.184425908	0.484342	-0.304543577	0.119225	-0.26091227	0.172748	0.038010016	0.93752	C5	-	Y	-	-	-	-	-
ENSG00000224837	GCSHP5	-0.322416719	0.095488	0.122726061	0.639449	0.012426566	0.968288	0.45132715	0.017015	0.349706849	0.085556	-0.107264105	0.771622	C4	-	-	-	-	Y	-	-
ENSG00000224885	EIPR1-IT1	-1.614721622	1.79E-06	-0.670473716	0.081392	-0.918468377	0.007332	0.945130539	0.034647	0.711177522	0.129832	-0.241382335	NA	C3	-	Y	-	Y	Y	-	-
ENSG00000224903	RNF32-AS1	-0.525016118	0.05654	0.098572297	0.807193	-0.011428681	0.979525	0.630923136	0.026272	0.528740534	0.085054	-0.107485671	0.860105	C2	-	-	-	-	Y	-	-
ENSG00000224928	KRT8P30	-0.59175347	0.30344	0.442016289	0.395671	0.452497303	0.368706	1.040045466	0.033003	1.058199534	0.031562	0.013137418	NA	C3	-	-	-	-	Y	Y	-
ENSG00000224986	PPP1R8P1	-3.639094343	0.038764	-1.973849628	NA	-0.196700841	NA	1.68161315	NA	3.458620156	NA	1.782250272	NA	C3	-	Y	-	-	-	-	-
ENSG00000225125	RANP4	2.204121759	0.04552	2.138091436	NA	1.303803814	NA	-0.057404478	0.963157	-0.883480927	0.439503	-0.828329616	NA	C1	-	Y	-	-	-	-	-
ENSG00000225138	SLC9A3-AS1	-0.306354179	0.106152	0.530446497	0.015819	0.656984074	0.000125	0.843050881	1.16E-06	0.979029709	1.04E-11	0.130027688	0.703647	C2	-	-	Y	Y	Y	Y	-
ENSG00000225190	PLEKHM1	0.245950064	0.037595	0.310110537	0.032206	0.231524081	0.104284	0.070657277	0.641871	1.04E-05	0.999916	-0.075798055	0.77505	C5	-	-	Y	-	-	-	-
ENSG00000225234	TRAPPC12-AS1	0.858501573	4.32E-06	0.416174392	0.114468	0.490185776	0.025146	-0.435628915	0.039862	-0.353652249	0.074033	0.076758528	0.877863	C1	-	Y	-	Y	Y	-	-
ENSG00000225246	RPS2P1	1.12284975	0.138181	-0.660564206	NA	-0.023065612	NA	-1.773657474	0.040829	-1.128427303	0.192859	0.640905948	NA	C5	-	-	-	-	Y	-	-
ENSG00000225265	TAF1A-AS1	-0.576386442	0.038357	-0.075136382	0.878418	-0.076958266	0.833177	0.509651544	0.139598	0.514533393	0.072753	0.002083758	0.997573	C3	-	Y	-	-	-	-	-
ENSG00000225285	LINC01770	-3.498512542	0.004387	-0.065012852	NA	0.378737843	0.665549	3.447799379	0.007423	3.89123614	0.000762	0.450934188	NA	C3	-	Y	-	-	Y	Y	-
ENSG00000225377	NRSN2-AS1	-0.388470924	0.006521	0.268600087	0.082563	0.317642798	0.092176	0.663701319	1.53E-06	0.721043309	1.39E-05	0.051165473	0.90074	C4	-	Y	-	-	Y	Y	-
ENSG00000225439	BOLA3-AS1	0.434661861	5.99E-05	0.278454167	0.037852	0.272949295	0.032976	-0.149871273	0.16764	-0.146634496	0.174457	-0.002214444	0.994002	C5	-	Y	-	-	-	-	-
ENSG00000225449	RAB6C-AS1	-1.319173494	0.002711	-0.070771677	0.913461	-0.425149501	0.404775	1.257375366	0.007504	0.909042291	0.08721	-0.352369126	NA	C3	-	Y	-	-	Y	-	-
ENSG00000225492	GBP1P1	-3.23989161	0.037491	-2.325867192	NA	-3.342763596	NA	0.904687482	NA	-0.097070097	NA	-1.003941568	NA	C3	-	Y	-	-	-	-	-
ENSG00000225518	LINC01703	-0.807302245	0.048832	0.148111895	0.805922	-0.30989292	0.520778	0.963697131	0.023027	0.512560796	0.281446	-0.455352307	NA	C2	-	Y	-	-	Y	-	-
ENSG00000225526	MKRN2OS	1.615716728	0.017505	1.559640296	NA	1.863055415	0.00892	-0.047851621	0.955704	0.263308883	0.692077	0.303671819	NA	C1	-	Y	-	Y	-	-	-
ENSG00000225614	ZNF469	-0.718683123	0.00036	-0.50607371	0.022447	-0.455932022	0.03062	0.218853682	0.415707	0.276977165	0.268118	0.052899688	0.9222	C4	-	Y	Y	Y	-	-	-
ENSG00000225619	MYT1L-AS1	0.993149415	0.372641	2.138728653	0.003791	1.420622008	0.119457	1.15429993	0.102006	0.444074144	0.644142	-0.714053152	NA	C2	-	-	Y	-	-	-	-
ENSG00000225630	MTND2P28	-0.562972061	6.57E-41	-0.258301788	1.96E-05	-0.423611516	4.83E-19	0.311123293	1.49E-11	0.154135112	0.000112	-0.162443199	0.015038	C4	-	Y	-	Y	Y	-	-
ENSG00000225648	SBDSP1	0.491457688	3.20E-09	0.082108298	0.548356	0.185890602	0.218707	-0.402980419	1.65E-06	-0.291331359	0.011154	0.106930168	0.643048	C5	-	Y	-	-	Y	-	-
ENSG00000225675	LINC01771	-0.830001288	0.207006	0.136042148	0.881981	0.41783361	0.553604	0.974081384	0.144933	1.263145977	0.040364	0.285042037	NA	C4	-	-	-	-	-	Y	-
ENSG00000225697	SLC26A6	0.528644946	3.62E-08	0.265626774	0.014261	0.376952024	0.000182	-0.256320239	0.011113	-0.136951635	0.218597	0.114248393	0.48568	C5	-	Y	-	Y	-	-	-
ENSG00000225756	DBH-AS1	-0.186372198	0.164724	-0.070539142	0.735571	-0.395661451	0.020329	0.1218871	0.462818	-0.19442573	0.285164	-0.32107457	0.182857	C2	-	-	-	Y	-	-	-
ENSG00000225791	TRAM2-AS1	-0.643441585	4.68E-07	-0.013681949	0.957855	-0.228846908	0.130589	0.636858405	9.15E-06	0.42922652	0.002956	-0.212483706	0.344923	C4	-	Y	-	-	Y	Y	-
ENSG00000225793	TMEM30A-DT	-0.609332747	0.167377	0.481534614	0.324428	0.106942855	0.876328	1.096705523	0.008112	0.73049137	0.146076	-0.369686732	NA	C2	-	-	-	-	Y	-	-
ENSG00000225891	DHDDS-AS1	-1.993473408	0.000338	-0.324203463	0.588681	-0.049152317	0.940467	1.671482596	0.008503	1.959408283	0.000735	0.278722965	NA	C3	-	Y	-	-	Y	Y	-
ENSG00000225920	RIMKLBP2	-0.835196589	0.19275	0.282943554	0.717292	0.337503474	0.631312	1.125778538	0.057795	1.187043115	0.042646	0.058912207	NA	C4	-	-	-	-	-	Y	-
ENSG00000225988	LAMP5-AS1	-1.726523324	0.008569	0.016852538	0.987462	-0.812869545	0.235019	1.756957118	0.017571	0.929227559	0.298452	-0.829690132	NA	C2	-	Y	-	-	Y	-	-
ENSG00000226053	LINC01776	-0.95840167	0.002429	-0.195519187	0.667128	-0.411621282	0.253438	0.76979299	0.020673	0.561353846	0.110868	-0.213107143	NA	C2	-	Y	-	-	Y	-	-
ENSG00000226121	AHCTF1P1	0.636419381	5.92E-07	0.306434181	0.073444	0.345131086	0.020464	-0.324906047	0.007507	-0.277100809	0.014812	0.042736708	0.887043	C1	-	Y	-	Y	Y	-	-
ENSG00000226137	BAIAP2-DT	-0.075898728	0.497545	0.023227528	0.879702	0.212356744	0.050594	0.105671008	0.270468	0.303065557	0.000653	0.192004723	0.108067	C4	-	-	-	-	-	Y	-

ENSG00000226167	AP4B1-AS1	0.653471038	0.088289	-0.253784365	0.688269	0.315804559	0.524105	-0.900927472	0.025014	-0.323114938	0.440852	0.573374015	NA	C5	-	-	-	-	Y	-	-
ENSG00000226174	TEX22	-0.741590723	0.062644	0.165428345	0.727225	0.067068111	0.896886	0.913659659	0.01622	0.824202088	0.039098	-0.095514377	NA	C3	-	-	-	-	Y	Y	-
ENSG00000226200	SGMS1-AS1	-1.976727234	9.26E-26	-0.540525378	0.002321	-0.849450051	1.39E-05	1.442672403	5.05E-13	1.142717523	1.58E-06	-0.305755267	0.320894	C3	-	Y	Y	Y	Y	Y	-
ENSG00000226220	CICP22	0.634835073	0.260519	-0.327773589	0.71323	-0.114386523	0.910789	-0.960987936	0.042081	-0.733804628	0.174893	0.21620004	NA	C5	-	-	-	-	Y	-	-
ENSG00000226259	GTF2H2B	0.678665715	5.42E-05	0.379682777	0.08	0.330778312	0.117099	-0.293042199	0.051617	-0.333421063	0.02232	-0.045649801	0.903722	C1	-	Y	-	-	-	Y	-
ENSG00000226261	PIMREGP1	-3.726332406	0.024007	-1.033773021	NA	-2.085353561	NA	2.716733285	NA	1.66359484	NA	-1.05851306	NA	C3	-	Y	-	-	-	-	-
ENSG00000226318	RPS3AP38	1.223377713	0.014983	0.578149259	0.365534	0.761142727	0.214497	-0.638175324	0.226719	-0.447994338	0.440119	0.185304546	NA	C5	-	Y	-	-	-	-	-
ENSG00000226328	NUP50-DT	-0.353938542	0.039836	0.188959704	0.328413	0.251909559	0.144018	0.549369	0.000456	0.62068707	5.65E-05	0.065872657	0.832773	C4	-	Y	-	-	Y	Y	-
ENSG00000226342	NMD3P1	0.256136389	0.758389	1.083690984	0.115421	1.439857549	0.008711	0.83396138	0.181472	1.198612629	0.021577	0.355250327	NA	C2	-	-	-	Y	-	Y	-
ENSG00000226450	CYP2D8P	1.017883345	0.046121	0.185334729	0.857678	0.722593304	0.233382	-0.828466711	0.162336	-0.280327873	0.631773	0.542525427	NA	C1	-	Y	-	-	-	-	-
ENSG00000226478	UPF3AP1	-1.911895968	0.042462	-0.425487687	NA	-1.532828264	NA	1.48417511	0.224241	0.399470513	0.822353	-1.094208626	NA	C4	-	Y	-	-	-	-	-
ENSG00000226479	TMEM185B	-0.171705442	0.027502	0.146058398	0.101179	0.058959441	0.562902	0.324115647	8.86E-06	0.245369082	0.000978	-0.084243969	0.503233	C4	-	-	-	-	Y	-	-
ENSG00000226508	LINC01918	-0.786420704	0.010968	-0.235078929	0.552006	-0.67909091	0.036168	0.559697871	0.118693	0.122683024	0.804857	-0.44202708	NA	C3	-	Y	-	Y	-	-	-
ENSG00000226562	CYP4F26P	-0.1202416	0.754452	0.118004936	0.804219	0.413563338	0.193055	0.244125411	0.416279	0.548680564	0.022058	0.29996594	0.412815	C3	-	-	-	-	-	Y	-
ENSG00000226581	LINC02848	0.568328263	0.027005	0.737441896	0.003267	0.914529515	0.000153	0.175596341	0.531064	0.360869401	0.151424	0.180307385	0.647102	C1	-	Y	Y	Y	-	-	-
ENSG00000226648	PLCG1-AS1	-0.316009324	0.255406	0.268561929	0.391959	-0.32415985	0.356249	0.59146092	0.025262	0.006915266	0.985859	-0.590637962	0.12527	C2	-	-	-	-	Y	-	-
ENSG00000226686	LINC01535	-0.196609634	0.641225	0.646881717	0.038822	0.489933383	0.15319	0.849193217	0.006857	0.701358557	0.041104	-0.154096756	NA	C2	-	-	Y	-	Y	Y	-
ENSG00000226688	ENTPD1-AS1	0.305253904	0.129232	0.452815447	0.04235	0.019745549	0.953315	0.153380507	0.489483	-0.271205299	0.176085	-0.429564686	0.084221	C1	-	-	Y	-	-	-	-
ENSG00000226696	LENG8-AS1	0.28528976	0.22237	0.571737726	0.017008	0.358382974	0.142418	0.293284309	0.225807	0.087764688	0.757534	-0.210462793	0.576607	C1	-	-	Y	-	-	-	-
ENSG00000226711	FAM66C	-0.572980279	0.008631	-0.00227041	0.995465	-0.249237573	0.32717	0.577182927	0.007029	0.338137474	0.151424	-0.243909406	0.457706	C2	-	Y	-	-	Y	-	-
ENSG00000226746	SMCR5	-1.231965109	0.300093	0.909823647	NA	-1.512867686	NA	2.149093952	0.024639	-0.263682448	NA	-2.421552129	NA	C2	-	-	-	-	Y	-	-
ENSG00000226777	FAM30A	0.708980853	1.73E-10	0.539213934	3.38E-05	0.309419494	0.033936	-0.16307138	0.205824	-0.384911172	0.001123	-0.226885961	0.215908	C1	-	Y	Y	Y	-	Y	-
ENSG00000226803	ZNF451-AS1	1.302787878	0.021383	0.55563529	NA	0.447589491	0.620862	-0.7396591	0.208086	-0.842111768	0.13963	-0.106717826	NA	C5	-	Y	-	-	-	-	-
ENSG00000226819	MEIS1-AS3	-0.250984022	0.813946	0.808420609	NA	1.132816701	0.10491	1.063439246	0.187064	1.39800588	0.049459	0.324891553	NA	C2	-	-	-	-	-	Y	-
ENSG00000226823	SUGT1P1	-1.415054432	0.003865	-0.3903288	0.573037	-0.718631252	0.190574	1.027639397	0.101496	0.712256634	0.285488	-0.320954631	NA	C3	-	Y	-	-	-	-	-
ENSG00000226856	THORLNC	-2.02473939	0.010445	-1.861374529	NA	-0.563247787	0.500666	0.175970476	0.916983	1.478350959	0.150306	1.300367114	NA	C3	-	Y	-	-	-	-	-
ENSG00000226864	ATE1-AS1	0.707956937	0.042083	0.810382962	0.038849	0.828538086	0.028236	0.10691884	0.786252	0.134342671	0.728975	0.022183203	NA	C1	-	Y	Y	Y	-	-	-
ENSG00000226891	LINC01359	-1.513408246	0.003475	0.195993631	0.781866	-0.170457197	0.827838	1.717195501	0.001743	1.357843588	0.037061	-0.364934308	NA	C3	-	Y	-	-	Y	Y	-
ENSG00000226913	BSN-DT	-1.105346472	0.03873	0.173703735	0.812864	-0.257925755	0.697878	1.285395766	0.013396	0.863282284	0.141464	-0.428217455	NA	C4	-	Y	-	-	Y	-	-
ENSG00000226953	NCKAP5-AS2	-1.637981899	0.033898	-0.354495254	NA	0.61376608	0.337644	1.292734135	0.167069	2.267307895	0.001253	0.972146651	NA	C2	-	Y	-	-	-	Y	-
ENSG00000227036	LINC00511	-1.228760515	0.015355	-0.389300345	0.507256	-0.167477187	0.788438	0.845886242	0.093497	1.076320887	0.022574	0.222719654	NA	C3	-	Y	-	-	-	Y	-
ENSG00000227199	ST7-AS1	-1.263092875	6.13E-09	-0.111958667	0.723352	-0.657581932	0.001413	1.158226072	2.69E-07	0.620907346	0.011585	-0.542598595	0.034505	C3	-	Y	-	Y	Y	Y	Y
ENSG00000227212	PFN1P6	0.422051399	0.05111	0.488056375	0.143139	0.486339984	0.034013	0.072585688	0.840237	0.078936609	0.768803	0.001448092	0.997918	C1	-	-	-	Y	-	-	-
ENSG00000227258	SMIM2-AS1	0.833554513	0.170931	0.402983148	NA	1.146742886	0.04777	-0.424265391	0.544472	0.328313978	0.600238	0.744109139	NA	C1	-	-	-	Y	-	-	-
ENSG00000227268	KLLN	-1.069720418	2.51E-11	-0.412922585	0.012677	-0.153574522	0.414082	0.663909658	0.000253	0.930807514	2.93E-08	0.26225791	0.247997	C2	-	Y	Y	-	Y	Y	-
ENSG00000227345	PARG	0.289168519	1.38E-05	-0.01873036	0.892009	-0.016261693	0.896648	-0.30103376	2.11E-06	-0.290730615	1.32E-06	0.005093264	0.979827	C5	-	-	-	-	Y	-	-
ENSG00000227354	RBM26-AS1	-1.00201753	8.07E-09	-0.231922975	0.234191	-0.20768508	0.314848	0.778242527	7.32E-05	0.808732046	7.26E-05	0.025948275	0.95765	C3	-	Y	-	-	Y	Y	-
ENSG00000227394	RPL11P1	-1.488621665	0.353448	1.362590711	NA	0.593099115	NA	2.858954276	0.017715	2.098434777	0.138942	-0.769902077	NA	C2	-	-	-	-	Y	-	-
ENSG00000227398	KIF9-AS1	0.427116013	0.040148	0.200515753	0.543148	0.157743629	0.672056	-0.220469688	0.39194	-0.255569974	0.365058	-0.039158061	0.957684	C1	-	Y	-	-	-	-	-
ENSG00000227487	NCAM1-AS1	-0.114954699	0.638946	0.768062843	0.000455	0.18994418	0.368893	0.890775799	2.08E-05	0.319114828	0.104592	-0.574842217	0.020599	C4	-	-	Y	-	Y	-	Y
ENSG00000227620	ALG1L8P	-1.897438662	0.000473	-0.223636075	0.737037	-0.683133531	0.204565	1.679694437	0.004692	1.229689867	0.069633	-0.456190917	NA	C3	-	Y	-	-	Y	-	-
ENSG00000227630	LINC01132	-2.079940874	0.011243	-0.934432853	NA	-1.492281886	0.07838	1.156142037	0.287026	0.602461955	0.66916	-0.557868649	NA	C3	-	Y	-	-	-	-	-
ENSG00000227676	LINC01068	2.760357627	5.36E-08	1.010914185	NA	0.823028482	NA	-1.740065789	9.47E-05	-1.922366978	2.97E-05	-0.185806135	NA	C5	-	Y	-	-	Y	Y	-
ENSG00000227888	FAM66A	-1.471244636	0.040496	-1.525687596	NA	-1.647509853	0.040889	-0.043491795	0.974945	-0.158828505	0.911472	-0.120498613	NA	C3	-	Y	-	Y	-	-	-
ENSG00000227953	LINC01341	-1.149092846	1.27E-06	-0.793172605	0.001417	-0.698634013	0.00654	0.362166837	0.278221	0.465294553	0.155019	0.097492854	NA	C3	-	Y	Y	Y	-	-	-
ENSG00000228038	VN1R51P	-1.457165723	0.01501	-0.336151386	0.704399	0.014918028	0.986747	1.127601771	0.14543	1.486167919	0.026438	0.352998061	NA	C3	-	Y	-	-	-	Y	-
ENSG00000228065	LINC01515	-0.140371815	0.49304	0.166562598	0.396229	0.256841944	0.172572	0.313627736	0.046035	0.41228467	0.011074	0.093438611	0.736645	C4	-	-	-	-	Y	Y	-
ENSG00000228109	MELTF-AS1	-0.445977702	0.112041	0.1018186	0.824069	0.082649675	0.811737	0.556833944	0.090211	0.543940028	0.046871	-0.015894198	0.982455	C2	-	-	-	-	-	Y	-
ENSG00000228203	RNF144A-AS1	-0.581702179	0.000242	-0.231305664	0.2441	-0.032917647	0.901924	0.356962372	0.048915	0.563316486	0.001085	0.201235208	0.474909	C4	-	Y	-	-	Y	Y	-
ENSG00000228253	MT-ATP8	-0.435718815	0.010194	-0.576030671	0.013861	-0.242067697	0.259095	-0.133091918	0.42819	0.208732114	6.17E-05	0.33697561	0.082351	C3	-	Y	Y	-	-	-	-
ENSG00000228314	CYP4F29P	-0.1766218	0.513308	0.208321946	0.460167	-0.010200161	0.976532	0.392099333	0.045038	0.181419073	0.405899	-0.217203718	0.40972	C2	-	-	-	-	Y	-	-
ENSG00000228315	GUSBP11	0.378997305	0.001168	0.365886641	0.002815	0.192002882	0.165026	-0.006405779	0.969161	-0.172276076	0.171503	-0.17091812	0.305844	C1	-	Y	Y	-	-	-	-
ENSG00000228409	CCT6P1	-0.204178551	0.037336	-0.097354435	0.438232	-0.304867456	0.003111	0.113093867	0.326709	-0.085948033	0.50294	-0.204512807	0.148255	C4	-	-	-	Y	-	-	-
ENSG00000228451	SDAD1P1	-0.543375068	0.00782	-0.006499789	0.986091	0.042621879	0.889552	0.543238117	0.008958	0.600490445	0.003139	0.05221655	0.911454	C2	-	Y	-	-	Y	Y	-
ENSG00000228486	C2orf92	-0.159670476	0.461217	0.379056882	0.054934	0.005834865	0.986747	0.545848971	0.002727	0.180561893	0.483672	-0.371606219	0.19895	C4	-	-	-	-	Y	-	-
ENSG00000228526	MIR34AHG	-0.001716244	0.992011	0.333228144	0.012278	0.15377386	0.311814	0.341784592	0.006028	0.170308485	0.218323	-0.176568736	0.355374	C4	-	-	Y	-	Y	-	-
ENSG00000228544	CCDC183-AS1	0.068065804	0.870175	0.526564153	0.061652	0.664862218	0.010478	0.465254892	0.113307	0.611216137	0.027132	0.14158874	0.758618	C1	-	-	-	Y	-	Y	-
ENSG00000228594	FNDC10	-0.364120425	0.000522	-0.100048623	0.52609	0.132478299	0.356443	0.270613425	0.014689	0.511220928	8.74E-07	0.235400266	0.128137	C4	-	Y	-	-	-	Y	-
ENSG00000228709	LINC02575	-0.782823497	8.31E-12	0.29635842	0.307689	0.052101172	0.775458	1.085927239	2.30E-08	0.849709617	3.65E-18	-0.242148485	0.503233	C4	-	Y	-	-	Y	Y	-
ENSG00000228727	SAPCD1	-0.709962148	0.000411	-0.473173493	0.047274	-0.60589081	0.005356	0.243166428	0.379207	0.119047526	0.702412	-0.129696377	0.788353	C4	-	Y	Y	Y	-	-	-
ENSG00000228775	WEE2-AS1	-1.242947997	2.33E-11	-0.29237761	0.208628	-0.328179746	0.081925	0.958207156	3.32E-05	0.929213781	1.29E-05	-0.033518795	0.95032	C2	-	Y	-	-	Y	Y	-
ENSG00000228782	MRPL45P2	-0.05824441	0.892491	0.634151383	0.034779	-0.10923303	0.817951	0.69966901	0.006528	-0.036430639	0.93342	-0.741201132	0.023062	C2	-	-	Y	-	Y	-	Y
ENSG00000228794	LINC01128	0.37119677	0.000209	0.125346679	0.369617	0.062117938	0.691527	-0.239240397	0.011101	-0.294565111	0.001583	-0.060578659	0.766671	C5	-	Y	-	-	-	-	-
ENSG00000228812	LAMA5-AS1	-1.844263933	0.036575	0.077751621	0.941382	-1.352554811	0.098772	1.928801536	0.023364	0.507146086	0.720075	-1.427572308	NA	C3	-	Y	-	-	Y	-	-
ENSG00000228878	SEPTIN7-DT	-0.926334386	0.001044	0.300074565	0.360551	0.29158789	0.443076	1.233329572	9.23E-06	1.232383795	9.33E-05	-0.006916898	0.99358	C4	-	Y	-	-	Y	Y	-
ENSG00000228882	CICP9	4.441083029	0.008356	3.266394507	NA	2.034623271	NA	-1.163969322	NA	-2.398254832	NA	-1.233170111	NA	C5	-	Y	-	-	-	-	-
ENSG00000228889	UBAC2-AS1	-1.310208948	0.000107	-0.439619628	0.280887	-0.007269799	0.988193	0.87472403	0.027815	1.317986243	7.54E-05	0.434178798	NA	C3	-	Y	-	-	Y	Y	-

ENSG00000228903	RASA4CP	-0.625807227	0.030644	-0.078033159	0.879081	-0.150991873	0.687385	0.554835685	0.129446	0.489324688	0.14195	-0.070257784	NA	C2	-	Y	-	-	-	-	-
ENSG00000228915	OR7E128P	-0.980918301	0.231276	-0.239898564	0.815068	0.463150769	0.56418	0.748883875	0.382791	1.45735989	0.036904	0.706140761	NA	C3	-	-	-	-	-	Y	-
ENSG00000228971	LINC02607	-1.03911098	0.013605	0.234048855	0.638329	0.406405102	0.384257	1.278941386	0.001651	1.460775759	0.00057	0.175363171	NA	C3	-	Y	-	-	Y	Y	-
ENSG00000229081	LINC01165	-4.097736848	0.007065	-2.437412746	NA	-1.298144052	NA	1.674176654	NA	2.814106624	NA	1.140789541	NA	C3	-	Y	-	-	-	-	-
ENSG00000229127	KANSL1L-AS1	-0.848296727	0.090014	0.123923769	0.859167	0.310267857	0.574529	0.977179436	0.044139	1.172820526	0.01142	0.188844458	NA	C2	-	-	-	-	Y	Y	-
ENSG00000229205	LINC00200	-1.20641617	4.10E-07	-0.909501956	0.000108	-0.677885816	0.002666	0.298823056	0.434171	0.542950457	0.091155	0.236785413	0.643729	C3	-	Y	Y	Y	-	-	-
ENSG00000229267	SNHG31	-1.362643612	0.014835	-0.487791727	0.481288	-0.422303165	0.590658	0.880644845	0.151393	0.953150458	0.16613	0.066930966	NA	C3	-	Y	-	-	-	-	-
ENSG00000229325	ACAP2-IT1	0.507594509	0.208942	1.215133217	0.000689	0.746947704	0.095009	0.714524621	0.027553	0.254975495	0.572809	-0.464833543	NA	C2	-	-	Y	-	Y	-	-
ENSG00000229358	DPY19L1P1	0.314429372	0.219112	0.60905741	0.013261	0.523575148	0.046805	0.301853963	0.239208	0.224528395	0.426111	-0.082762185	0.882986	C4	-	-	Y	Y	-	-	-
ENSG00000229419	RALGAPA1P1	0.176578108	0.442863	0.450895791	0.082631	0.482402842	0.012484	0.281160416	0.260326	0.320672487	0.100408	0.035438866	0.948722	C4	-	-	-	Y	-	-	-
ENSG00000229431	MED8-AS1	-1.117780113	0.011497	-1.102226497	0.029994	-0.740988528	0.169518	0.026686409	0.97381	0.390168889	0.58111	0.356594219	NA	C4	-	Y	Y	-	-	-	-
ENSG00000229589	ACVR2B-AS1	-0.925751789	0.00066	-0.565101512	0.051428	-0.475471847	0.072058	0.368854718	0.325287	0.464673502	0.162841	0.092025525	0.891855	C4	-	Y	-	-	-	-	-
ENSG00000229644	NAMPTP1	0.643360591	0.025157	0.364925838	0.363563	0.291327578	0.401216	-0.272330497	0.412529	-0.337829639	0.223264	-0.069525407	0.921908	C5	-	Y	-	-	-	-	-
ENSG00000229666	MAST4-AS1	-1.308880647	0.000437	-0.372576756	0.362429	-0.372194992	0.359042	0.943207069	0.019876	0.951070659	0.021805	0.003132805	NA	C3	-	Y	-	-	Y	Y	-
ENSG00000229692	SOS1-IT1	0.954914175	4.85E-05	0.098094963	0.838536	0.569221733	0.045525	-0.849562914	8.14E-06	-0.371357959	0.04368	0.472814531	0.092833	C1	-	Y	-	Y	Y	Y	-
ENSG00000229809	ZNF688	-0.675917459	1.14E-05	-0.229966465	0.186338	-0.560947806	0.000647	0.452607792	0.004271	0.130000727	0.557766	-0.328092825	0.124344	C3	-	Y	-	Y	Y	-	-
ENSG00000229816	DDX50P1	0.643122477	0.091241	-0.140253494	0.833724	-0.108937202	0.876431	-0.777560525	0.04486	-0.736082165	0.083574	0.03253777	NA	C5	-	-	-	-	Y	-	-
ENSG00000229891	LINC01315	-0.818984219	0.011308	-0.502654302	0.183643	-0.25848479	0.475253	0.323947552	0.434609	0.575479979	0.080897	0.248432313	NA	C3	-	Y	-	-	-	-	-
ENSG00000229921	KIF25-AS1	-1.397568766	0.00037	-0.395477397	0.363563	-0.436037677	0.228008	1.010106651	0.032005	0.975950421	0.026236	-0.037747403	NA	C3	-	Y	-	-	Y	Y	-
ENSG00000229956	ZRANB2-AS2	-0.87725532	0.01996	-0.161072257	0.72915	-0.20933068	0.666441	0.723249684	0.064275	0.682233921	0.120992	-0.045670866	NA	C3	-	Y	-	-	-	-	-
ENSG00000230091	TMEM254-AS1	0.330961125	0.604654	1.28008736	0.010745	0.637333611	0.313492	0.956827736	0.042476	0.321210239	0.617116	-0.63952477	NA	C2	-	-	Y	-	Y	-	-
ENSG00000230314	ELOVL2-AS1	-1.232194114	0.038107	-0.169474333	0.829485	-0.298461316	0.698518	1.06925162	0.106069	0.948567039	0.205189	-0.126047771	NA	C3	-	Y	-	-	-	-	-
ENSG00000230330	HMGN2P3	-0.139610251	0.442964	-0.24698862	0.230077	-0.351815301	0.042076	-0.101004142	0.629143	-0.19780037	0.270369	-0.102007605	0.761898	C4	-	-	-	Y	-	-	-
ENSG00000230359	TPI1P2	-0.79937496	0.001637	-0.475868099	0.118712	-0.665373553	0.032882	0.330538641	0.279861	0.149115155	0.706389	-0.187091979	NA	C2	-	Y	-	Y	-	-	-
ENSG00000230371	KARS1P2	-2.403867101	0.00405	-1.901596362	NA	-0.959831434	0.255607	0.499495272	NA	1.462627937	0.20695	0.954902312	NA	C3	-	Y	-	-	-	-	-
ENSG00000230417	LINC00595	-1.584093173	0.032683	-0.705161285	0.364449	0.008329499	0.992783	0.881013439	0.347424	1.607131666	0.025799	0.71525882	NA	C2	-	Y	-	-	-	Y	-
ENSG00000230449	RPL7P4	-3.944774695	0.031791	-2.015715246	NA	-4.036058528	NA	1.887732762	NA	NA	NA	-1.974077298	NA	C3	-	Y	-	-	-	-	-
ENSG00000230461	PROX1-AS1	-0.351218169	0.001875	-0.045777292	0.836899	-0.058987646	0.767993	0.312028298	0.026464	0.306993694	0.029005	-0.012034405	0.97796	C4	-	Y	-	-	Y	Y	-
ENSG00000230487	PSMG3-AS1	-0.996050463	6.17E-18	-0.065189811	0.700789	0.154925874	0.188679	0.937981854	1.99E-14	1.165611241	4.15E-26	0.223185215	0.123495	C3	-	Y	-	-	Y	Y	-
ENSG00000230590	FTX	0.037874897	0.77619	0.055447303	0.692837	0.300717371	0.000269	0.025318071	0.906036	0.277752505	0.046399	0.248291786	0.018555	C4	-	-	-	Y	-	-	-
ENSG00000230630	DNM3OS	-0.129165404	0.331652	0.189541693	0.097711	-0.001617141	0.9925	0.32551142	0.002109	0.142492221	0.22524	-0.187861058	0.104701	C4	-	-	-	-	Y	-	-
ENSG00000230658	KLHL7-DT	-1.30741603	0.000835	0.111829963	0.816502	-0.233231385	0.559018	1.428527673	0.000126	1.089145944	0.005566	-0.341305486	NA	C3	-	Y	-	-	Y	Y	-
ENSG00000230982	DSTNP1	0.406914536	0.308614	-0.037806431	0.957928	0.737043054	0.031449	-0.438510097	0.339565	0.345291502	0.368098	0.777213781	NA	C1	-	-	-	Y	-	-	-
ENSG00000231074	HCG18	-0.113850045	0.180504	0.262407312	0.003206	0.182771006	0.043214	0.382362045	2.05E-10	0.311308956	1.63E-07	-0.076552594	0.526777	C4	-	-	-	-	Y	Y	-
ENSG00000231105	ECE1-AS1	-2.034911221	0.014805	-0.160322053	0.877978	0.108577532	0.905233	1.875185395	0.047571	2.158612586	0.010556	0.272327391	NA	C2	-	Y	-	-	Y	Y	-
ENSG00000231154	MORF4L2-AS1	-0.514341383	0.223404	0.754008849	0.037759	0.204732617	0.674503	1.274827045	4.63E-05	0.733644646	0.043383	-0.544832314	NA	C2	-	-	Y	-	Y	Y	-
ENSG00000231160	KLF3-AS1	-0.603020889	0.004737	0.04520848	0.878383	-0.084734021	0.732303	0.655244648	0.002478	0.533043786	0.015293	-0.12708516	0.703663	C3	-	Y	-	-	Y	Y	-
ENSG00000231185	SPRY4-AS1	-0.982939718	0.000643	0.143836392	0.706992	-0.267089913	0.456692	1.134714079	8.89E-05	0.729691791	0.033498	-0.40916252	NA	C2	-	Y	-	-	Y	Y	-
ENSG00000231205	ZNF826P	-0.761312498	8.96E-09	-0.511555338	0.000427	-0.402569801	0.007903	0.255628329	0.144088	0.373693439	0.025729	0.112578602	0.716025	C4	-	Y	Y	Y	-	Y	-
ENSG00000231233	CFAP58-DT	-1.270435315	0.027005	-0.523868579	0.482083	0.075949393	0.922939	0.752165815	0.341918	1.360448636	0.028231	0.602034966	NA	C4	-	Y	-	-	-	Y	-
ENSG00000231258	ZSWIM5P2	-4.056542371	0.040235	-0.635493276	NA	-1.12757865	NA	3.445729956	NA	2.94462154	NA	-0.49388874	NA	C3	-	Y	-	-	-	-	-
ENSG00000231312	MAP4K3-DT	-0.658060675	0.010769	0.077406731	0.851346	0.135755262	0.682112	0.742604226	0.004695	0.808760685	0.001085	0.061640561	0.916226	C4	-	Y	-	-	Y	Y	-
ENSG00000231327	LINC01816	-1.196292339	0.009686	-0.554305311	0.321794	-0.646014375	0.190897	0.648455108	0.315715	0.565055621	0.376801	-0.088693826	NA	C3	-	Y	-	-	-	-	-
ENSG00000231340	ACTG1P10	2.621176243	0.022496	2.409562418	NA	1.766518672	NA	-0.211742906	0.862909	-0.841600369	0.396366	-0.63387396	NA	C1	-	Y	-	-	-	-	-
ENSG00000231345	BEND3P1	0.323436471	0.07585	-0.027501697	0.93442	0.097381428	0.715046	-0.345402756	0.034792	-0.211543473	0.222196	0.128102218	0.69007	C5	-	-	-	-	Y	-	-
ENSG00000231365	WARS2-AS1	-0.356596353	0.062922	0.36091252	0.061981	-0.069529674	0.801823	0.724342717	1.70E-06	0.302149636	0.10812	-0.427864394	0.025025	C2	-	-	-	-	Y	-	Y
ENSG00000231439	WASIR2	-0.68926277	0.0257	-0.380872445	0.313711	-0.176106104	0.671201	0.316025136	0.410315	0.528439404	0.12426	0.207489309	NA	C4	-	Y	-	-	-	-	-
ENSG00000231527	FAM27C	0.620306013	0.040807	0.395215565	0.321901	0.230238363	0.586378	-0.218387209	0.52149	-0.375813816	0.22521	-0.163251948	NA	C1	-	Y	-	-	-	-	-
ENSG00000231544	RSL24D1P11	2.952639905	0.036938	1.54437391	NA	2.583147876	NA	-1.384012779	0.280588	-0.356816167	0.769119	1.021723285	NA	C5	-	Y	-	-	-	-	-
ENSG00000231584	FAHD2CP	-0.880491229	5.98E-07	0.128674392	0.600209	-0.060985323	0.810546	1.016750323	3.42E-08	0.834211916	7.43E-06	-0.186802874	0.526499	C3	-	Y	-	-	Y	Y	-
ENSG00000231607	DLEU2	-0.436997434	6.62E-05	-0.064209556	0.71323	-0.210115212	0.119067	0.379358137	0.00085	0.241636851	0.055995	-0.143030239	0.465158	C4	-	Y	-	-	Y	-	-
ENSG00000231638	LUARIS	-2.106755077	0.014868	-0.367195616	0.704399	-0.273457458	0.775106	1.745529823	0.031953	1.845995845	0.02383	0.095324827	NA	C3	-	Y	-	-	Y	Y	-
ENSG00000231663	COA6-AS1	-0.752004427	0.078712	0.263609211	0.612885	-0.344440018	0.453291	1.02532086	0.012567	0.422847477	0.381496	-0.603851773	NA	C2	-	-	-	-	Y	-	-
ENSG00000231764	DLX6-AS1	-0.425863924	1.52E-05	0.025731829	0.869892	0.030726531	0.859431	0.458176921	1.06E-06	0.471279167	1.44E-05	0.007795388	0.978268	C2	-	Y	-	-	Y	Y	-
ENSG00000231770	TMEM44-AS1	-0.991306923	2.84E-08	-0.28701706	0.203799	-0.526827345	0.006933	0.712240854	0.000802	0.47910575	0.037644	-0.238317778	0.498978	C3	-	Y	-	Y	Y	Y	-
ENSG00000231890	DARS-AS1	-1.284564842	0.006456	0.631746084	0.143187	0.652534934	0.143434	1.923994208	5.66E-06	1.951734977	1.23E-05	0.023811425	NA	C2	-	Y	-	-	Y	Y	-
ENSG00000232000	CLCN3P1	-0.440611881	1.89E-07	-0.116124866	0.316274	-0.166848704	0.100251	0.330938921	9.04E-05	0.288440665	0.000804	-0.047893067	0.793512	C4	-	Y	-	-	Y	-	-
ENSG00000232024	LSM12P1	0.402319084	0.004516	-0.075536684	0.774441	0.127208664	0.538378	-0.471710692	0.001846	-0.2601891	0.090499	0.205882925	0.435983	C5	-	Y	-	-	Y	-	-
ENSG00000232040	ZBED9	0.477470354	0.000537	0.086483095	0.713114	0.275596141	0.113978	-0.384135639	0.00161	-0.187961189	0.171503	0.191095772	0.365693	C1	-	Y	-	-	Y	-	-
ENSG00000232044	SILC1	-1.517007186	8.23E-11	-0.231038679	0.443695	-1.033944436	5.77E-06	1.291181978	1.69E-07	0.497136923	0.106913	-0.800263488	0.004543	C3	-	Y	-	Y	Y	-	Y
ENSG00000232075	MRPL35P2	-1.620804611	0.019756	-0.515961905	0.499366	-0.700519036	0.373966	1.119299552	0.196007	0.936122786	0.352953	-0.187672827	NA	C3	-	Y	-	-	-	-	-
ENSG00000232093	DCST1-AS1	-1.005978862	0.008552	0.136867002	0.806916	-0.360131055	0.523089	1.149931161	0.002591	0.661091431	0.200976	-0.495437516	NA	C3	-	Y	-	-	Y	-	-
ENSG00000232104	RFX3-AS1	-0.65784493	0.124598	0.282367304	0.590375	-0.113200988	0.841632	0.946929436	0.014996	0.559549385	0.202553	-0.39147183	NA	C2	-	-	-	-	Y	-	-
ENSG00000232112	TMA7	-0.293213069	2.87E-06	-0.306008288	2.06E-05	-0.335726405	4.33E-07	-0.006400498	0.942025	-0.027826192	0.69013	-0.026551043	0.839789	C4	-	-	Y	Y	-	-	-
ENSG00000232160	RAP2C-AS1	-0.566610353	0.039203	-0.090529947	0.824312	0.136575776	0.711022	0.481748105	0.064699	0.717491067	0.004304	0.230662205	0.576984	C4	-	Y	-	-	-	Y	-
ENSG00000232186	TERF1P7	0.547961101	0.459023	-1.377195261	NA	-0.219092914	0.84434	-1.919192451	0.022672	-0.751804575	0.367266	1.166135242	NA	C5	-	-	-	-	Y	-	-
ENSG00000232434	AJM1	-0.503061724	0.011582	-0.049451766	0.872137	0.160846615	0.557935	0.459314341	0.028671	0.678532695	0.001864	0.213945423	0.548406	C4	-	Y	-	-	Y	Y	-

ENSG00000232445	EMSLR	-1.532620136	0.045077	-1.021834158	NA	-0.181308969	0.850641	0.514572358	0.669133	1.365199019	0.110262	0.843780109	NA	C3	-	Y	-	-	-	-	-
ENSG00000232457	SLC16A6P1	-2.055713797	0.001606	0.509842737	0.442851	-0.617646032	0.394187	2.571545335	3.74E-05	1.453127557	0.072621	-1.124204012	NA	C2	-	Y	-	-	Y	-	-
ENSG00000232485	RPL37A-DT	-1.433689356	0.002757	-0.416867597	0.442039	-0.047729867	0.939262	1.024272719	0.064004	1.400050568	0.004069	0.371976257	NA	C2	-	Y	-	-	-	Y	-
ENSG00000232653	GOLGA8N	-0.577673978	0.151349	0.183574384	0.720678	0.314567491	0.46342	0.768684882	0.034374	0.907995932	0.009463	0.133778748	NA	C4	-	-	-	-	Y	Y	-
ENSG00000232677	LINC00665	-0.455926669	3.97E-08	-0.14944683	0.182315	-0.177955484	0.119947	0.312821754	0.000762	0.292668987	0.003832	-0.025184233	0.927911	C4	-	Y	-	-	Y	-	-
ENSG00000232812	LINC01717	-1.019439117	7.42E-07	0.383806712	0.101228	0.204006686	0.331172	1.412105639	2.24E-10	1.23777775	4.52E-10	-0.176935352	0.61329	C3	-	Y	-	-	Y	Y	-
ENSG00000232818	RPS2P32	-0.494288921	0.003295	-0.068523694	0.782716	-0.40364854	0.018115	0.432975666	0.018974	0.105430507	0.667033	-0.332353705	0.152979	C2	-	Y	-	Y	Y	-	-
ENSG00000232859	LYRM9	-0.944788815	0.018654	0.212985645	0.710089	-0.126055044	0.81425	1.166084004	0.008622	0.833058059	0.065705	-0.337292076	NA	C2	-	Y	-	-	Y	-	-
ENSG00000232888	RPS11P5	1.367592588	0.004486	0.689687764	0.348446	1.257695113	0.017213	-0.668691302	0.14196	-0.094016341	0.858428	0.568818773	NA	C1	-	Y	-	Y	-	-	-
ENSG00000232892	RGS5-AS1	-0.707360429	0.003641	-0.01371105	0.972251	-0.338407488	0.229878	0.701478077	0.003733	0.384214212	0.190128	-0.322046558	0.37282	C2	-	Y	-	-	Y	-	-
ENSG00000232956	SNHG15	0.071381486	0.462015	-0.126927813	0.244304	-0.274344903	0.007123	-0.192127128	0.03682	-0.330971045	0.000405	-0.143952724	0.387119	C5	-	-	-	-	-	Y	-
ENSG00000232995	RGS5	-0.680116536	0.011183	0.200670221	0.556685	0.146014321	0.725246	0.886308842	0.001438	0.841490457	0.00931	-0.05027667	0.948931	C2	-	Y	-	-	Y	Y	-
ENSG00000233016	SNHG7	0.327184203	0.000447	0.291349404	0.010935	0.068759878	0.640368	-0.029332852	0.812718	-0.243730853	0.005719	-0.219732567	0.082976	C5	-	Y	-	-	-	-	-
ENSG00000233024	NPIPA9	0.462549375	0.004268	0.027327456	0.948627	-0.227189831	0.421412	-0.427383295	0.031263	-0.676415527	9.02E-05	-0.25059218	0.562428	C5	-	Y	-	-	Y	Y	-
ENSG00000233038	PTPRN2-AS1	-1.877899552	0.037749	-0.89307235	NA	-1.326663087	NA	0.978819954	0.458263	0.568301002	0.711864	-0.420852181	NA	C3	-	Y	-	-	-	-	-
ENSG00000233058	LINC00884	-1.260144194	0.000994	-0.317861011	0.521351	-0.700964129	0.072953	0.946856074	0.041339	0.572621885	0.268002	-0.379750265	NA	C3	-	Y	-	-	Y	-	-
ENSG00000233073	GRM3-AS1	0.436373355	0.173238	0.257438584	0.623567	0.663496311	0.048609	-0.171194717	0.716132	0.242117117	0.517235	0.407227416	NA	C1	-	-	-	Y	-	-	-
ENSG00000233077	LINC01271	-0.99518887	0.037615	0.032018339	0.965676	0.19831899	0.717354	1.032779127	0.036088	1.207473586	0.009257	0.16884993	NA	C2	-	Y	-	-	Y	Y	-
ENSG00000233098	CCDC144NL-AS1	-0.364007786	7.83E-09	-0.146957066	0.081213	-0.074715571	0.391715	0.223398607	0.001085	0.303778756	9.23E-07	0.075120827	0.535179	C4	-	Y	-	-	-	Y	-
ENSG00000233198	RNF224	-1.474311318	6.84E-07	0.06349641	0.890639	-0.441350353	0.196633	1.544304723	1.85E-07	1.048086442	0.001947	-0.501389644	0.219596	C3	-	Y	-	-	Y	Y	-
ENSG00000233225	SSBP3P1	-0.101496602	0.828112	0.252816412	0.540238	0.747396384	0.007184	0.361713529	0.365853	0.864051804	0.00524	0.497885713	NA	C4	-	-	-	Y	-	Y	-
ENSG00000233296	TMEM18-DT	-0.183735501	0.777498	-0.047982218	0.959039	-1.616319739	0.013522	0.141415364	0.8551	-1.418669462	0.043902	-1.566370342	NA	C5	-	-	-	Y	-	Y	-
ENSG00000233382	NKAPP1	-0.96293267	0.017665	0.22130254	0.66559	-0.381003323	0.425543	1.192463954	0.00297	0.596730316	0.231416	-0.599917	NA	C2	-	Y	-	-	Y	-	-
ENSG00000233396	LINC01719	-0.205491263	0.213935	-0.132860918	0.56151	0.090500919	0.671313	0.078829382	0.694225	0.310555909	0.034642	0.226634433	0.322314	C4	-	-	-	-	-	Y	-
ENSG00000233452	STXBP5-AS1	1.344655001	0.004292	0.870898952	0.190164	0.978628086	0.080275	-0.468050002	0.382468	-0.352226229	0.487277	0.111777855	NA	C1	-	Y	-	-	-	-	-
ENSG00000233515	LINC01518	-0.038129782	0.882667	0.34589241	0.082952	0.184809266	0.410082	0.390791745	0.032012	0.237663961	0.240579	-0.158134047	0.603589	C4	-	-	-	-	Y	-	-
ENSG00000233527	ZNF529-AS1	-0.397786922	0.150344	0.144820453	0.64388	-0.197035428	0.508111	0.548702674	0.033178	0.215500093	0.501781	-0.338922845	0.297861	C3	-	-	-	-	Y	-	-
ENSG00000233593	LINC02609	-0.711438386	0.010428	-0.013744483	0.977466	0.087755671	0.824554	0.704543052	0.015754	0.813458835	0.003932	0.10426619	0.861463	C4	-	Y	-	-	Y	Y	-
ENSG00000233695	GAS6-AS1	-1.197387504	4.95E-06	-0.450435352	0.106417	-0.082282929	0.813846	0.753059958	0.014995	1.129740273	5.26E-05	0.37112826	0.322231	C2	-	Y	-	-	Y	Y	-
ENSG00000233806	LINC01237	0.128991189	0.73979	0.672904237	0.034462	0.481782148	0.124851	0.550240169	0.061898	0.367401767	0.231887	-0.18900815	0.70249	C1	-	-	Y	-	-	-	-
ENSG00000233858	LINC02599	-1.580515245	0.068384	-0.777184876	NA	0.331828649	0.70825	0.812175122	0.491193	1.928174039	0.017463	1.113669044	NA	C3	-	-	-	-	-	Y	-
ENSG00000233901	LINC01503	-3.432354643	0.027208	-1.096131936	NA	-0.727171659	NA	2.323299745	NA	2.718497751	NA	0.372856693	NA	C4	-	Y	-	-	-	-	-
ENSG00000233922	LINC01694	-1.414361546	3.20E-19	-0.585319779	0.000447	-0.419243794	0.010867	0.835769692	2.38E-06	1.009653229	1.98E-09	0.168936513	0.554373	C3	-	Y	Y	Y	Y	Y	-
ENSG00000233930	KRTAP5-AS1	0.630109422	0.004986	0.545960795	0.083944	0.532614216	0.05626	-0.076806767	0.809399	-0.082164312	0.776282	-0.010685656	0.988432	C1	-	Y	-	-	-	-	-
ENSG00000234006	DDX39B-AS1	-2.7813717	0.033179	-1.26477551	NA	-0.46319038	NA	1.520037363	NA	2.331231407	0.081068	0.802809725	NA	C3	-	Y	-	-	-	-	-
ENSG00000234036	TXNP6	-4.356776779	0.007653	-2.415033388	NA	-1.67367977	NA	1.911314832	NA	2.707931247	NA	0.767720418	NA	C3	-	Y	-	-	-	-	-
ENSG00000234215	CT66	-0.496864126	0.169032	0.264418087	0.52944	0.265617254	0.46837	0.767716644	0.030236	0.777569372	0.020189	0.003994554	NA	C2	-	-	-	-	Y	Y	-
ENSG00000234224	TMEM229A	0.484970983	2.69E-10	0.428416355	1.99E-06	0.316157729	0.000214	-0.050204008	0.611595	-0.154070871	0.040356	-0.109222822	0.392863	C1	-	Y	Y	Y	-	-	-
ENSG00000234284	ZNF879	0.353405877	0.049378	0.019993891	0.953543	0.138042306	0.54873	-0.326979606	0.092106	-0.200906532	0.290103	0.120808037	0.734205	C5	-	Y	-	-	-	-	-
ENSG00000234362	LINC01914	-1.443949427	0.024168	-0.688277039	0.337673	-0.3089678	0.655489	0.764971286	0.368225	1.148648268	0.097056	0.382095429	NA	C2	-	Y	-	-	-	-	-
ENSG00000234409	CCDC188	-0.542389422	1.15E-06	0.069895671	0.676	-0.035858021	0.830558	0.618790012	2.73E-08	0.521330413	3.34E-06	-0.102829993	0.59011	C4	-	Y	-	-	Y	Y	-
ENSG00000234420	ZNF37BP	0.324974136	1.06E-05	0.206538232	0.013545	0.084405766	0.389295	-0.111910339	0.073063	-0.226291463	7.81E-05	-0.119795956	0.136143	C1	-	Y	-	-	-	-	-
ENSG00000234444	ZNF736	0.124768394	0.238801	-0.210434111	0.043691	-0.151855668	0.18717	-0.329128136	2.56E-06	-0.262393689	0.000656	0.061344329	0.669046	C5	-	-	-	-	Y	-	-
ENSG00000234465	PINLYP	-0.901612078	0.008906	-0.655367928	0.111904	-0.086167187	0.853646	0.252618145	0.622529	0.831103346	0.018627	0.572458388	NA	C4	-	Y	-	-	-	Y	-
ENSG00000234492	RPL34-DT	1.331444726	0.036414	1.275594713	0.107973	1.494324518	0.038943	-0.053588398	0.947147	0.1794348	0.802094	0.225104841	NA	C1	-	Y	-	Y	-	-	-
ENSG00000234494	SP2-AS1	-0.700498585	2.47E-05	-0.23566468	0.291066	-0.137711949	0.540853	0.472068189	0.016852	0.577419084	0.001692	0.100477842	0.794766	C4	-	Y	-	-	Y	Y	-
ENSG00000234678	ELF3-AS1	-0.097461108	0.759583	0.500877549	0.035021	0.177792717	0.559599	0.605081631	0.005282	0.290230096	0.266613	-0.319932986	0.277798	C2	-	-	Y	-	Y	-	-
ENSG00000234684	SDCBP2-AS1	-0.795393665	1.26E-05	-0.098808803	0.694652	-0.266089345	0.21826	0.70261194	0.000163	0.544212543	0.00957	-0.163960604	0.614025	C2	-	Y	-	-	Y	Y	-
ENSG00000234690	EPCAM-DT	-0.310243575	0.117081	0.129244256	0.638289	0.237621211	0.313816	0.4461026	0.010318	0.563018054	0.001155	0.111610253	0.752014	C4	-	-	-	-	Y	Y	-
ENSG00000234771	SLC25A25-AS1	0.461580476	7.26E-07	0.386483752	0.00056	0.349383461	0.000901	-0.068745619	0.563392	-0.09758066	0.361796	-0.034376343	0.88523	C1	-	Y	Y	Y	-	-	-
ENSG00000234776	C11orf94	1.604969166	0.034058	0.476278669	NA	0.935079725	NA	-1.124341595	0.145953	-0.653261687	0.408826	0.463757432	NA	C1	-	Y	-	-	-	-	-
ENSG00000234912	SNHG20	-0.015017645	0.92239	0.377172801	0.001256	0.112691967	0.435258	0.398498665	0.000105	0.142259297	0.236819	-0.261561315	0.087174	C4	-	-	Y	-	Y	-	-
ENSG00000234944	LINC02623	-2.270079835	1.33E-09	-0.477460401	0.215006	-0.235366094	0.613825	1.800407021	1.97E-05	2.048463619	1.86E-06	0.243816062	NA	C3	-	Y	-	-	Y	Y	-
ENSG00000235016	SEMA3F-AS1	-0.471239613	0.349595	0.402578576	0.407946	0.269916616	0.633238	0.880743506	0.025839	0.756706153	0.09235	-0.129297229	NA	C3	-	-	-	-	Y	-	-
ENSG00000235092	ID2-AS1	-0.721177296	3.47E-08	0.128381978	0.482318	-0.114510932	0.506702	0.856513615	1.75E-10	0.621147239	5.91E-06	-0.24010204	0.210811	C2	-	Y	-	-	Y	Y	-
ENSG00000235098	ANKRD65	-1.358476088	0.006812	-0.362618601	0.577537	-0.784921499	0.216075	1.002008251	0.063671	0.587825472	0.407325	-0.418163161	NA	C4	-	Y	-	-	-	-	-
ENSG00000235162	C12orf75	-0.007628265	0.959165	0.430521909	9.64E-06	0.428750562	8.22E-05	0.444587136	3.82E-07	0.451503677	3.56E-06	0.000731777	0.997573	C4	-	-	Y	Y	Y	Y	-
ENSG00000235169	SMIM1	-1.086756332	0.004727	-1.109579387	0.008618	-0.609695031	0.159339	-0.016337104	0.979533	0.491752945	0.328798	0.502791326	NA	C3	-	Y	Y	-	-	-	-
ENSG00000235217	TSPY26P	-0.482185138	0.006849	-0.12073146	0.626049	-0.070816073	0.779664	0.368549155	0.076383	0.426194601	0.035024	0.052400712	0.909803	C2	-	Y	-	-	-	Y	-
ENSG00000235257	ITGA9-AS1	-0.216315865	0.402143	0.470048285	0.034503	0.34768709	0.140396	0.692736249	0.000774	0.578631998	0.008078	-0.119532588	0.755306	C2	-	-	Y	-	Y	Y	-
ENSG00000235316	DUSP8P5	0.414799876	0.230328	0.957844517	0.001276	0.36688493	0.369247	0.550243248	0.034569	-0.032092069	0.939319	-0.587532092	0.08446	C2	-	-	Y	-	Y	-	-
ENSG00000235374	SSR4P1	-0.88548012	0.020172	-0.245086843	0.630758	0.005662981	0.992725	0.644433168	0.148334	0.905367669	0.033263	0.255518117	NA	C4	-	Y	-	-	-	Y	-
ENSG00000235453	SMIM27	-0.481553664	0.006711	-0.05266511	0.859906	-0.302689071	0.139658	0.435008813	0.022589	0.193653501	0.387123	-0.247015522	0.399036	C4	-	Y	-	-	Y	-	-
ENSG00000235481	UBE2R2-AS1	-0.690523409	0.297615	0.482158905	0.475403	0.15771045	0.862822	1.176297927	0.043197	0.862872787	0.232951	-0.31842938	NA	C2	-	-	-	-	Y	-	-
ENSG00000235597	LINC01102	-1.060186005	3.35E-06	-0.238501132	0.489291	-0.267160796	0.273119	0.831191801	0.005806	0.808248163	0.001449	-0.026020869	0.97084	C3	-	Y	-	-	Y	Y	-
ENSG00000235652	FBXO30-DT	-0.475202365	0.082988	0.053232195	0.900098	0.163549457	0.645081	0.534997287	0.05465	0.653474903	0.017145	0.113267142	0.840416	C2	-	-	-	-	-	Y	-
ENSG00000235703	LINC00894	0.13344548	0.661394	0.131409874	0.716545	0.502292213	0.027326	0.005807106	0.986664	0.383740957	0.115303	0.374396141	0.239425	C4	-	-	-	Y	-	-	-

ENSG00000235706	DICER1-AS1	-0.675854865	0.000384	-0.078745992	0.794065	-0.102512305	0.684161	0.603739437	0.003391	0.588391517	0.002871	-0.020677099	0.96749	C2	-	Y	-	-	Y	Y	-
ENSG00000235750	KIAA0040	-1.071994981	6.75E-06	-0.743972053	0.012591	-0.331646457	0.217507	0.337223546	0.368301	0.754778964	0.005322	0.414571466	0.354552	C4	-	Y	Y	-	-	Y	-
ENSG00000235823	OLMALINC	-0.713079016	4.49E-08	-0.33213926	0.040002	0.044232193	0.826933	0.387635896	0.014601	0.771690662	5.59E-09	0.379213654	0.037528	C4	-	Y	Y	-	Y	Y	Y
ENSG00000235890	TSPEAR-AS1	-0.588353103	5.58E-15	0.212358636	0.151865	0.461025814	1.26E-05	0.807453628	5.14E-14	1.064272649	7.20E-33	0.249077905	0.479466	C4	-	Y	-	Y	Y	Y	-
ENSG00000235897	TM4SF19-AS1	-1.567418747	0.174021	0.763589933	NA	0.151469186	NA	2.336546179	0.023867	1.730999168	0.198553	-0.608104347	NA	C2	-	-	-	-	Y	-	-
ENSG00000235903	CPB2-AS1	-0.067218076	0.871893	0.541058628	0.09278	0.435281452	0.208047	0.614427558	0.027845	0.517043539	0.090926	-0.102754167	0.871807	C1	-	-	-	-	Y	-	-
ENSG00000235904	RBMS3-AS3	-0.432496168	0.002883	-0.370275328	0.028633	-0.154834642	0.409076	0.068429628	0.756235	0.292381402	0.078584	0.218475362	0.384989	C4	-	Y	Y	-	-	-	-
ENSG00000235954	TTC28-AS1	-0.284464616	0.002175	0.12171455	0.343679	-0.032576898	0.825179	0.412971837	2.14E-05	0.266824242	0.006556	-0.151571452	0.328007	C2	-	-	-	-	Y	-	-
ENSG00000236017	ASMTL-AS1	-0.43180042	0.069828	0.114070965	0.717894	0.048938508	0.887801	0.553480958	0.021023	0.495709494	0.049932	-0.062707425	0.906938	C2	-	-	-	-	Y	Y	-
ENSG00000236088	COX10-AS1	0.544387138	8.60E-11	0.260367777	0.012029	0.342488502	0.001304	-0.277721544	1.88E-05	-0.187430958	0.017851	0.084891582	0.559116	C5	-	Y	-	Y	-	-	-
ENSG00000236144	TMEM147-AS1	-0.472222202	2.69E-06	0.116522761	0.3891	-0.057027919	0.677344	0.595930265	2.25E-08	0.429797596	3.46E-05	-0.170708701	0.263595	C4	-	Y	-	-	Y	Y	-
ENSG00000236296	GUSBP5	-1.143479242	0.022168	-0.042115572	0.950272	-1.208462265	0.015995	1.112374307	0.033535	-0.05034524	0.956628	-1.166750835	NA	C2	-	Y	-	Y	Y	-	-
ENSG00000236397	DDX11L2	-0.468376526	0.105263	0.250574761	0.473795	0.135728536	0.699967	0.727321047	0.009932	0.618968433	0.027595	-0.11166749	0.841508	C2	-	-	-	-	Y	Y	-
ENSG00000236404	VLDLR-AS1	-0.805417106	1.01E-08	-0.53295558	0.00051	-0.343378804	0.024702	0.277901835	0.12918	0.477026344	0.002857	0.193040314	0.457415	C3	-	Y	Y	Y	-	Y	-
ENSG00000236437	LINC02151	-1.898009565	0.000225	-0.703887793	0.176283	-0.967908304	0.058201	1.19920419	0.058016	0.942093753	0.190128	-0.261495735	NA	C3	-	Y	-	-	-	-	-
ENSG00000236502	SIX3-AS1	-0.62134995	0.000206	-0.482037764	0.019847	-0.173704559	0.386321	0.146175888	0.551843	0.462507145	0.005739	0.311696293	0.227704	C4	-	Y	Y	-	-	Y	-
ENSG00000236562	IFITM3P1	-3.795916258	0.034139	0.13391885	NA	-1.567311207	NA	3.93316516	NA	2.248079902	NA	-1.700207005	NA	C4	-	Y	-	-	-	-	-
ENSG00000236565	HNRNPA3P5	0.107566755	0.451916	-0.178279854	0.277767	-0.385035102	0.006387	-0.279610512	0.046982	-0.477910134	0.000421	-0.203242228	0.392235	C5	-	-	-	Y	-	Y	-
ENSG00000236570	RAD23BP1	1.666668068	0.043885	1.318091467	NA	1.17622001	NA	-0.343154972	0.688249	-0.472534133	0.604972	-0.135875751	NA	C1	-	Y	-	-	-	-	-
ENSG00000236618	PITPNA-AS1	-0.813340788	3.89E-08	-0.431371429	0.007198	-0.507816596	0.000551	0.388673881	0.028053	0.320403332	0.074153	-0.073509519	0.832105	C4	-	Y	Y	Y	Y	-	-
ENSG00000236714	LINC01844	0.499472197	0.443626	0.526426207	0.456308	1.05884619	0.047659	0.032590258	0.966234	0.574211807	0.308585	0.534710962	NA	C1	-	-	-	Y	-	-	-
ENSG00000236753	MKLN1-AS	-0.66986516	0.000474	-0.045795189	0.885016	-0.229073991	0.370952	0.630899841	0.001442	0.455547521	0.04608	-0.180799503	0.617535	C2	-	Y	-	-	Y	Y	-
ENSG00000236778	INTS6-AS1	-0.529059248	0.045261	0.044401766	0.92365	0.045597939	0.904966	0.579071966	0.060812	0.589454144	0.034773	0.004534612	0.995809	C4	-	Y	-	-	-	Y	-
ENSG00000236859	NIFK-AS1	-0.587202389	7.40E-05	-0.092881462	0.644573	-0.130955101	0.461723	0.500802093	0.001048	0.471122328	0.002235	-0.03521012	0.91982	C4	-	Y	-	-	Y	Y	-
ENSG00000236914	LINC01852	-2.11517736	0.002095	-0.72138033	0.320949	-1.208214816	0.056885	1.397769123	0.090295	0.920444062	0.32455	-0.483318205	NA	C3	-	Y	-	-	-	-	-
ENSG00000237036	ZEB1-AS1	0.278541151	0.022829	0.263655186	0.074491	0.44758685	0.000288	-0.008344446	0.962532	0.183586121	0.141465	0.186803184	0.304742	C4	-	-	-	Y	-	-	-
ENSG00000237149	ZNF503-AS2	-0.67555536	0.012385	-0.278020212	0.496231	-0.008706797	0.984744	0.405362271	0.245691	0.681604236	0.014373	0.272594185	0.605953	C4	-	Y	-	-	-	Y	-
ENSG00000237159	CNTFR-AS1	-0.123800977	0.598165	0.147823727	0.601867	0.45977711	0.021172	0.277584737	0.180131	0.598244123	0.000627	0.31545298	0.222964	C4	-	-	-	Y	-	Y	-
ENSG00000237187	NR2F1-AS1	-0.45186766	0.035322	-0.553799715	0.017008	-0.485805049	0.061044	-0.094774144	0.771794	-0.019405287	0.958371	0.069957869	0.914971	C5	-	Y	Y	-	-	-	-
ENSG00000237190	CDKN2AIPNL	-0.358538314	1.39E-05	-0.126116382	0.232585	-0.215997129	0.012049	0.238870038	0.003059	0.15733998	0.053638	-0.087049175	0.491512	C4	-	Y	-	-	-	-	-
ENSG00000237232	ZNF295-AS1	-1.59918931	0.040986	-0.35446697	0.705345	-1.59449628	0.044082	1.266441256	0.194893	0.02115763	0.988019	-1.247569794	NA	C2	-	Y	-	Y	-	-	-
ENSG00000237289	CKMT1B	-0.388275352	0.006519	0.091633764	0.643043	-0.087291318	0.678761	0.486596127	0.000474	0.315728996	0.052946	-0.176305812	0.463182	C2	-	Y	-	-	Y	-	-
ENSG00000237361	TUSC8	-1.753212053	1.34E-05	0.033381937	0.950782	-0.547632595	0.124132	1.797456659	1.48E-05	1.220749961	0.010136	-0.578941105	NA	C3	-	Y	-	-	Y	Y	-
ENSG00000237380	HOXD-AS2	-0.790748289	0.000168	-0.294326132	0.251035	-0.214766404	0.376451	0.503717782	0.025075	0.591228488	0.003864	0.083497636	0.844803	C4	-	Y	-	-	Y	Y	-
ENSG00000237399	PITRM1-AS1	-0.227007622	0.489757	0.387376041	0.200615	-0.052683756	0.910456	0.620281088	0.022198	0.188789476	0.627702	-0.436595166	0.282002	C2	-	-	-	-	Y	-	-
ENSG00000237435	LINC02790	-2.668867116	0.026636	-0.287020468	NA	-1.17287782	NA	2.404609958	0.116323	1.510960845	NA	-0.892123529	NA	C3	-	Y	-	-	-	-	-
ENSG00000237436	CAMTA1-DT	-0.961840899	0.066963	0.107531908	0.892343	-0.728288291	0.207925	1.076176502	0.041088	0.249346649	0.735602	-0.833017294	NA	C4	-	-	-	-	Y	-	-
ENSG00000237489	C10orf143	-1.615701782	0.015637	-0.521809304	0.509978	-0.534569212	0.471979	1.103388411	0.156551	1.094935486	0.163713	-0.010246008	NA	C3	-	Y	-	-	-	-	-
ENSG00000237491	LINC01409	0.632586987	0.035597	0.318508762	0.444143	0.177205079	0.719261	-0.30740506	0.344642	-0.440655896	0.196306	-0.138378108	NA	C1	-	Y	-	-	-	-	-
ENSG00000237575	PYY2	-1.381372045	0.22709	0.697515043	NA	-1.002344778	NA	2.081192882	0.034284	0.394801621	0.817873	-1.696644895	NA	C4	-	-	-	-	Y	-	-
ENSG00000237732	CT75	0.263559204	0.279294	0.462448043	0.031536	0.317104433	0.187275	0.204713268	0.363024	0.067688266	0.810447	-0.142460039	0.672717	C1	-	-	Y	-	-	-	-
ENSG00000237943	PRKCQ-AS1	0.224118677	0.381378	0.210105871	0.47833	0.452682291	0.04714	-0.007993002	0.97944	0.243455701	0.328078	0.24544292	0.478223	C4	-	-	-	Y	-	-	-
ENSG00000237973	MTCO1P12	-0.432021556	1.14E-05	-0.285915251	0.026402	-0.155068395	0.282747	0.152124179	0.188614	0.291878439	0.005523	0.133783934	0.501998	C4	-	Y	-	-	-	-	-
ENSG00000237977	EIF4HP2	-2.013934023	0.039812	0.031948111	NA	-0.261384194	NA	2.045297607	0.079158	1.76772656	0.114227	-0.287954521	NA	C2	-	Y	-	-	-	-	-
ENSG00000237978	KCNMB2-AS1	0.784030518	0.353448	1.337928863	NA	1.714754879	0.011602	0.559476645	0.404683	0.945311536	0.096813	0.381070067	NA	C2	-	-	-	Y	-	-	-
ENSG00000237984	PTENP1	-0.671822626	0.006988	0.001601739	0.997102	-0.441884953	0.115507	0.680511496	0.002738	0.244579528	0.3768	-0.440558663	0.158813	C2	-	Y	-	-	Y	-	-
ENSG00000238057	ZEB2-AS1	-1.722505723	0.01024	-0.812557682	0.316156	-1.192700361	0.106712	0.919765837	0.344071	0.54061625	0.630705	-0.384008727	NA	C3	-	Y	-	-	-	-	-
ENSG00000238083	LRRC37A2	0.375781788	0.025214	0.245968792	0.311249	0.446068211	0.010225	-0.123060899	0.596611	0.08485746	0.691375	0.203103747	0.512115	C4	-	Y	-	Y	-	-	-
ENSG00000238097	LINC02037	-2.477316281	1.21E-14	-1.213552195	1.03E-05	-0.568048147	0.060784	1.26866856	0.002743	1.922967034	5.66E-07	0.648447068	NA	C3	-	Y	Y	-	Y	Y	-
ENSG00000238098	ABCA17P	-0.903737681	0.088533	0.273510924	0.657264	0.442721588	0.340809	1.186363145	0.028434	1.360268731	0.004594	0.172499912	NA	C3	-	-	-	-	Y	Y	-
ENSG00000238113	LINC01410	-0.130059857	0.405241	0.135525989	0.466606	0.273951521	0.039483	0.271937573	0.050894	0.418323114	0.000359	0.142112472	0.481401	C4	-	-	-	-	-	Y	-
ENSG00000238120	LINC01589	2.032770448	0.045099	0.643794689	NA	0.76633465	NA	-1.373698078	0.162825	-1.250089295	0.201481	0.122674305	NA	C5	-	Y	-	-	-	-	-
ENSG00000238197	PAXBP1-AS1	-0.364287375	0.00024	-0.060340998	0.718023	-0.0416547	0.772723	0.310543085	0.005152	0.337155899	0.000606	0.021539781	0.937494	C4	-	Y	-	-	Y	Y	-
ENSG00000238278	ALG1L6P	-0.000857786	0.998397	0.542026268	0.024394	0.181310318	0.560563	0.549731023	0.025892	0.196843504	0.526458	-0.357753058	0.258449	C1	-	-	Y	-	Y	-	-
ENSG00000239152	RNA5SP310	3.850043471	0.04501	NA	NA	0.716345821	NA	-3.814915771	NA	-3.119275639	NA	0.690627359	NA	C5	-	Y	-	-	-	-	-
ENSG00000239306	RBM14	-0.420420304	1.83E-11	-0.045504035	0.672798	0.050418704	0.510174	0.381205166	3.50E-08	0.485540287	1.01E-17	0.098715604	0.334999	C4	-	Y	-	-	Y	Y	-
ENSG00000239569	KMT2E-AS1	-0.69770538	0.036444	0.061936523	0.906521	0.043836657	0.9267	0.766602914	0.013334	0.757151292	0.00939	-0.016232393	0.982152	C2	-	Y	-	-	Y	Y	-
ENSG00000239779	WBP1	-0.320009627	0.007352	-0.164767659	0.271776	-0.21973782	0.055998	0.16173293	0.249102	0.115004905	0.376319	-0.051946468	0.832494	C4	-	Y	-	-	-	-	-
ENSG00000239883	PARGP1	0.315503838	0.021894	0.303915684	0.048786	0.335868609	0.024618	-0.004971004	0.974031	0.034279687	0.822349	0.033937441	0.899704	C1	-	Y	Y	Y	-	-	-
ENSG00000240053	LY6G5B	-0.311424932	0.006346	-0.139383716	0.394679	-0.405818604	0.000421	0.178592849	0.158796	-0.079991048	0.554487	-0.263990633	0.089669	C4	-	Y	-	Y	-	-	-
ENSG00000240204	SMKR1	-0.750591258	0.000755	-0.27071419	0.329402	-0.206013083	0.482352	0.486192278	0.041452	0.559269883	0.020663	0.067638124	0.899832	C3	-	Y	-	-	Y	Y	-
ENSG00000240230	COX19	-0.069098062	0.568632	-0.33362242	0.007949	-0.016212652	0.923979	-0.258010889	0.026709	0.067737458	0.593595	0.320224028	0.024805	C4	-	-	Y	-	-	-	Y
ENSG00000240370	RPL13P5	-0.440048099	0.195426	0.00576971	0.992183	0.302902906	0.387675	0.453009362	0.236132	0.75775417	0.01552	0.300110849	NA	C4	-	-	-	-	-	Y	-
ENSG00000240445	FOXO3B	0.285915437	0.003359	-0.049734684	0.780239	0.140719187	0.270587	-0.329002714	0.000126	-0.130780261	0.151013	0.193028904	0.148376	C5	-	-	-	-	Y	-	-
ENSG00000240583	AQP1	-0.944614947	5.17E-07	-0.585589985	0.001897	-0.462799474	0.013733	0.363546519	0.144576	0.496731979	0.031077	0.12649346	0.757257	C3	-	Y	Y	Y	-	Y	-
ENSG00000240616	RPS6P25	0.829273451	0.087433	0.234036947	0.788981	-0.281192976	0.73136	-0.587354569	0.285316	-1.095320716	0.035804	-0.511430458	NA	C5	-	-	-	-	-	Y	-
ENSG00000240849	TMEM189	0.508830747	2.55E-08	0.20016309	0.155527	0.314647345	0.005401	-0.302079974	0.002657	-0.179582659	0.071438	0.117322238	0.540963	C5	-	Y	-	Y	Y	-	-

ENSG00000240857	RDH14	0.340908212	0.000538	-0.028367803	0.877978	0.056468448	0.734248	-0.362648859	0.000146	-0.269956204	0.009597	0.087831792	0.672369	C5	-	Y	-	-	Y	-	-
ENSG00000240875	LINC00886	-2.124054749	0.000477	-0.519277845	0.505338	-0.685105958	0.290973	1.616321953	0.049074	1.454944105	0.075911	-0.165938244	NA	C3	-	Y	-	-	Y	-	-
ENSG00000240891	PLCXD2	0.483935038	0.001211	0.352925836	0.050596	0.402251123	0.020684	-0.125467907	0.412275	-0.067653909	0.697518	0.052463658	0.868324	C1	-	Y	-	Y	-	-	-
ENSG00000241158	ADAMTS9-AS1	-1.311599878	5.43E-08	-0.054795064	0.88773	-0.912062369	0.002399	1.265304182	4.34E-06	0.412976398	0.322224	-0.858734523	0.031239	C3	-	Y	-	Y	Y	-	Y
ENSG00000241213	LINC02024	-1.661566597	0.416605	1.279375472	NA	-0.164279015	NA	2.941123775	0.047997	1.511485401	NA	-1.440956644	NA	C4	-	-	-	-	Y	-	-
ENSG00000241218	CSP2	2.818289627	0.037665	2.481998553	NA	1.878080776	NA	-0.337596099	0.786006	-0.928226468	0.426198	-0.598106006	NA	C1	-	Y	-	-	-	-	-
ENSG00000241316	SUCLG2-AS1	-1.209081018	0.000485	-0.623101476	0.103056	-0.194464306	0.66083	0.591586343	0.11582	1.027994217	0.001637	0.431057547	NA	C4	-	Y	-	-	-	Y	-
ENSG00000241343	RPL36A	-0.063240219	0.595571	0.219286075	0.059465	-0.224877397	0.023016	0.288785497	0.002373	-0.147176919	0.128797	-0.441648522	6.41E-06	C4	-	-	-	-	-	-	Y
ENSG00000241370	RPP21	-0.047855395	0.761557	-0.196854289	0.19544	-0.329814307	0.019632	-0.142472694	0.31197	-0.26717309	0.046696	-0.130019727	0.572524	C4	-	-	-	Y	-	-	-
ENSG00000241399	CD302	-0.468631135	0.197538	-0.011764216	0.98614	0.114713654	0.815583	0.460342216	0.146394	0.597771424	0.027148	0.132498777	0.830388	C4	-	-	-	-	-	Y	-
ENSG00000241404	EGFL8	0.490800822	0.003352	0.186200196	0.457296	0.124411167	0.654857	-0.298037422	0.095209	-0.351641805	0.059794	-0.058798529	0.899704	C5	-	Y	-	-	-	-	-
ENSG00000241468	ATP5MF	-0.311842899	1.05E-07	-0.248452698	0.000526	-0.269343931	2.53E-05	0.070079538	0.209704	0.057318528	0.252898	-0.018138062	0.884025	C4	-	Y	-	-	-	-	-
ENSG00000241472	PTPRG-AS1	-0.086755117	0.73594	0.041511099	0.899319	0.577698419	0.002923	0.135187011	0.592022	0.679324554	0.000286	0.539115958	0.018074	C4	-	-	-	Y	-	Y	Y
ENSG00000241684	ADAMTS9-AS2	0.482270745	0.006392	0.548987239	0.001715	0.562232271	0.002445	0.072839406	0.722142	0.094381891	0.658458	0.016172785	0.969121	C1	-	Y	Y	Y	-	-	-
ENSG00000241697	TMEFF1	0.229399771	0.055201	0.215395629	0.121829	-0.173862635	0.274829	-0.007713025	0.963679	-0.388626688	0.00183	-0.386350749	0.012854	C4	-	-	-	-	-	Y	Y
ENSG00000241839	PLEKHO2	-0.098766635	0.585248	-0.064412325	0.794343	0.203762237	0.247173	0.040918503	0.830535	0.317383625	0.017282	0.271098504	0.136078	C4	-	-	-	-	-	Y	-
ENSG00000242049	DNAJB8-AS1	-3.305664547	0.028665	-1.532704865	NA	-0.935712782	NA	1.80391773	NA	2.384658829	NA	0.59127665	NA	C3	-	Y	-	-	-	-	-
ENSG00000242125	SNHG3	-0.066998115	0.495871	0.39952816	7.45E-06	-0.012113252	0.937186	0.473043755	8.84E-12	0.069989543	0.449305	-0.408609595	3.18E-06	C4	-	-	Y	-	Y	-	Y
ENSG00000242173	ARHGDIG	-0.374694078	0.05206	-0.287859245	0.15156	-0.570026091	0.001789	0.093246137	0.707153	-0.180022804	0.441467	-0.279181257	0.294907	C4	-	-	-	Y	-	-	-
ENSG00000242247	ARFGAP3	0.632957745	7.75E-10	0.420314184	0.000525	0.486278576	5.10E-05	-0.206196999	0.06882	-0.131957637	0.293986	0.068932518	0.771568	C1	-	Y	Y	Y	-	-	-
ENSG00000242265	PEG10	0.819730727	2.46E-65	0.577946722	1.36E-21	0.321743317	1.18E-08	-0.235274155	3.99E-09	-0.483380826	1.47E-59	-0.253479878	1.57E-06	C1	Y	Y	Y	Y	-	Y	-
ENSG00000242294	STAG3L5P	0.399404176	0.000154	0.004409095	0.985258	0.158574778	0.252905	-0.389337509	0.00013	-0.226321245	0.022677	0.157600433	0.359205	C5	-	Y	-	-	Y	-	-
ENSG00000242338	BMS1P4	-0.798133295	0.029429	-0.292152079	0.548345	-0.350164814	0.418476	0.515413485	0.265396	0.461952387	0.319002	-0.057025792	NA	C3	-	Y	-	-	-	-	-
ENSG00000242498	ARPIN	-0.720731728	4.60E-21	-0.209394784	0.038147	-0.303769846	0.00014	0.518072863	5.84E-09	0.431631258	1.10E-07	-0.091337377	0.558058	C2	-	Y	-	Y	Y	Y	-
ENSG00000242686	PDE6B-AS1	-0.45140956	0.071157	-0.096319225	0.792841	-0.623114753	0.017802	0.361969482	0.088764	-0.156391741	0.57638	-0.523484784	0.04026	C2	-	-	-	Y	-	-	Y
ENSG00000242715	CCDC169	-0.940584462	0.00097	-0.213103312	0.593896	0.01623245	0.97107	0.735404768	0.023279	0.971061252	0.000864	0.232551975	NA	C4	-	Y	-	-	Y	Y	-
ENSG00000242756	RHOT1P3	2.156078488	0.007847	1.964163407	NA	1.543155699	NA	-0.192877265	0.820721	-0.598413919	0.400675	-0.411607066	NA	C1	-	Y	-	-	-	-	-
ENSG00000242779	ZNF702P	-0.86760898	0.003816	-0.259706343	0.475428	0.00296352	0.994011	0.616322375	0.06208	0.884891055	0.001674	0.266923098	0.525014	C4	-	Y	-	-	-	Y	-
ENSG00000243069	ARHGEF26-AS1	-1.048026934	0.326041	0.518590681	NA	0.753349581	0.396626	1.572836165	0.117399	1.81435446	0.044112	0.236809004	NA	C2	-	-	-	-	-	Y	-
ENSG00000243137	PSG4	-0.613814157	0.553994	0.96501543	NA	1.221456114	0.080665	1.585680083	0.04453	1.849357655	0.009811	0.259483306	NA	C2	-	-	-	-	Y	Y	-
ENSG00000243147	MRPL33	-0.375747831	2.17E-06	-0.162038424	0.138601	-0.141833038	0.166616	0.220257281	0.000529	0.249005326	1.82E-05	0.023328905	0.887679	C4	-	Y	-	-	-	-	-
ENSG00000243244	STON1	-0.905336793	2.07E-07	-0.197689807	0.424645	0.123987126	0.645457	0.714366396	0.000126	1.04412903	1.22E-08	0.32403806	0.261182	C4	-	Y	-	-	Y	Y	-
ENSG00000243279	PRAF2	-0.385327918	2.83E-05	-0.2099472	0.057542	-0.168968456	0.119364	0.182036148	0.036839	0.231535196	0.005017	0.043967628	0.796431	C4	-	Y	-	-	-	-	-
ENSG00000243317	STMP1	-0.645097706	3.19E-20	-0.239367292	0.002555	-0.246927394	0.00107	0.412179169	1.55E-12	0.413260386	2.29E-13	-0.004159457	0.979084	C4	-	Y	-	-	Y	Y	-
ENSG00000243323	PTPRVP	-2.342563597	0.0001	-0.465123762	0.456448	-0.220143374	0.724611	1.887554643	0.005988	2.13797807	0.000798	0.248001526	NA	C2	-	Y	-	-	Y	Y	-
ENSG00000243335	KCTD7	0.449066849	1.90E-08	0.177641788	0.049986	0.248452501	0.005321	-0.26511918	0.000877	-0.186018912	0.030946	0.073637041	0.615724	C5	-	Y	-	-	-	-	-
ENSG00000243547	HNRNPKP4	-0.305775956	0.283746	-0.007583169	0.988132	0.237484975	0.434385	0.30434958	0.27733	0.558736437	0.011239	0.250337273	0.50724	C4	-	-	-	-	-	Y	-
ENSG00000243701	DUBR	0.217513516	0.127309	0.353573909	0.024553	0.428535111	0.001326	0.142777712	0.342874	0.225595958	0.075792	0.077408213	0.758087	C4	-	-	Y	Y	-	-	-
ENSG00000243708	PLA2G4B	0.741421017	0.090135	-0.030791609	0.970759	-0.173993151	0.802385	-0.765140691	0.067705	-0.899624878	0.029562	-0.139802296	NA	C5	-	-	-	-	-	Y	-
ENSG00000243753	HLA-L	-0.451610457	0.18111	0.118928553	0.801585	0.368298775	0.30528	0.578545989	0.08688	0.834821485	0.008005	0.251801722	0.64324	C4	-	-	-	-	-	Y	-
ENSG00000243819	RN7SL832P	-1.656700023	2.60E-06	-0.440756862	0.175406	-1.034927532	0.000789	1.225031276	0.002579	0.63668692	0.21296	-0.592341808	NA	C3	-	Y	-	Y	Y	-	-
ENSG00000243943	ZNF512	0.324179057	1.62E-13	0.145345715	0.00766	0.219765193	2.57E-06	-0.172307134	4.96E-05	-0.089719355	0.036797	0.077148968	0.220878	C5	-	Y	-	-	-	-	-
ENSG00000243970	PPIEL	-0.24590473	0.044953	-0.010948666	0.963184	0.144179823	0.311814	0.241209972	0.060036	0.404978717	0.000139	0.158534334	0.395182	C4	-	-	-	-	-	Y	-
ENSG00000243988	RPS24P17	-3.822481398	0.048241	-1.038039113	NA	-1.901745366	NA	2.837759753	NA	1.916780225	NA	-0.898188008	NA	C3	-	Y	-	-	-	-	-
ENSG00000243989	ACY1	-0.665207517	0.001872	-0.292402717	0.268185	-0.064833446	0.842558	0.379129784	0.150084	0.615015277	0.011845	0.23026339	0.565557	C4	-	Y	-	-	-	Y	-
ENSG00000244026	FAM86DP	-0.220781211	0.28393	0.185522111	0.44167	0.147172673	0.576101	0.412030327	0.012345	0.382546337	0.037021	-0.035169515	0.937494	C4	-	-	-	-	Y	Y	-
ENSG00000244045	TMEM199	0.083717814	0.518922	-0.146178997	0.189694	-0.277918716	0.002733	-0.223442342	0.045728	-0.346667141	0.000748	-0.128785251	0.368432	C5	-	-	-	-	-	Y	-
ENSG00000244050	DEFB109F	-1.400683681	0.155408	0.598105441	NA	0.083961545	NA	2.008051383	0.022036	1.498240829	0.128316	-0.509500314	NA	C2	-	-	-	-	Y	-	-
ENSG00000244124	ATP1B3-AS1	0.460700717	0.200909	0.80881022	0.014669	0.803634702	0.00708	0.355033148	0.319563	0.357249204	0.278556	-0.001817517	0.997573	C1	-	-	Y	Y	-	-	-
ENSG00000244165	P2RY11	-0.697244638	6.18E-06	-0.296434256	0.166724	-0.726246654	7.56E-06	0.407630623	0.028816	-0.014129377	0.955539	-0.426946298	0.076282	C4	-	Y	-	Y	Y	-	-
ENSG00000244187	TMEM141	-0.406684369	0.001013	-0.161691807	0.400243	-0.014639298	0.943897	0.251854822	0.083209	0.407144684	0.000354	0.150325788	0.497614	C4	-	Y	-	-	-	Y	-
ENSG00000244300	GATA2-AS1	0.130969018	0.345281	0.471766057	7.22E-05	0.178112687	0.203277	0.347565759	0.000257	0.061750513	0.630108	-0.291290486	0.009797	C4	-	-	Y	-	Y	-	-
ENSG00000244306	DUXAP10	-0.401429964	9.90E-11	0.112282984	0.222587	0.102296578	0.346072	0.519934101	1.04E-16	0.518499683	9.47E-13	-0.007368492	0.975811	C4	-	Y	-	-	Y	Y	-
ENSG00000244462	RBM12	-0.422046099	3.18E-27	-0.192794392	4.73E-05	-0.325656517	1.46E-13	0.23566013	2.16E-09	0.11105491	0.007699	-0.12994557	0.018507	C4	-	Y	-	Y	-	-	-
ENSG00000244486	SCARF2	0.308125556	8.90E-06	0.201248519	0.018126	0.012308641	0.923027	-0.100010889	0.169259	-0.2808054	7.81E-06	-0.186248212	0.032039	C5	-	Y	-	-	-	-	-
ENSG00000244509	APOBEC3C	-0.82082696	2.55E-07	-0.425355063	0.021685	-0.432081841	0.017609	0.402137368	0.037063	0.403336865	0.039765	-0.004221352	0.993712	C4	-	Y	Y	Y	Y	Y	-
ENSG00000244625	MIATNB	-0.268784313	0.152258	0.145904772	0.549314	-0.138684046	0.556073	0.421247705	0.018804	0.144789241	0.511161	-0.281664963	0.269356	C4	-	-	-	-	Y	-	-
ENSG00000244879	GABPB1-AS1	-0.177696304	0.017081	0.394590403	1.52E-07	0.351493688	7.88E-06	0.578722401	3.76E-26	0.544108644	1.58E-21	-0.040153184	0.778535	C4	-	-	Y	Y	Y	Y	-
ENSG00000244968	LIFR-AS1	-0.988071944	0.015333	0.185275358	0.738658	-0.125808942	0.829065	1.181004114	0.000378	0.877757383	0.019352	-0.30832282	NA	C4	-	Y	-	-	Y	Y	-
ENSG00000245060	LINC00847	0.393835397	0.003791	0.178509177	0.535625	0.219998196	0.387737	-0.209275617	0.342874	-0.159197562	0.479309	0.044303461	0.933843	C5	-	Y	-	-	-	-	-
ENSG00000245248	USP2-AS1	-1.468020049	0.006737	-0.106189805	0.885571	0.047344544	0.947403	1.368060371	0.016261	1.530436841	0.005052	0.156677139	NA	C3	-	Y	-	-	Y	Y	-
ENSG00000245532	NEAT1	-0.791145196	4.73E-69	0.296646087	5.76E-06	0.107041283	0.117656	1.094545415	5.14E-113	0.91324391	5.03E-106	-0.18675385	0.014002	C2	-	Y	-	-	Y	Y	-
ENSG00000245556	SCAMP1-AS1	-0.327656759	0.020902	0.099028059	0.635638	-0.193626721	0.235969	0.433464728	0.00228	0.149002841	0.361829	-0.289421471	0.126619	C4	-	Y	-	-	Y	-	-
ENSG00000245573	BDNF-AS	-1.912411711	3.33E-10	-0.684400648	0.072531	-0.818458518	0.009081	1.240941603	0.003798	1.108470175	0.00642	-0.135714172	NA	C3	-	Y	-	Y	Y	Y	-
ENSG00000245711	NADK2-AS1	-0.883817975	0.05831	0.094487783	0.879702	0.148973185	0.788438	0.985459601	0.024329	1.047624143	0.016373	0.057242974	NA	C2	-	-	-	-	Y	Y	-
ENSG00000245848	CEBPA	-1.006619631	0.124399	0.299488112	0.707648	0.105928667	0.912574	1.313636725	0.01932	1.129102942	0.086236	-0.190143961	NA	C2	-	-	-	-	Y	-	-
ENSG00000245849	RAD51-AS1	-0.361499058	0.00072	0.063393155	0.674961	-0.016580615	0.922081	0.431363272	4.79E-05	0.359586314	0.001127	-0.077153004	0.690413	C4	-	Y	-	-	Y	Y	-

ENSG00000245870	LINC00682	-0.538986817	3.10E-13	-0.526135402	1.65E-17	-0.450748367	4.40E-12	0.019502186	0.862019	0.103137991	0.242952	0.07822362	0.498731	C4	-	Y	Y	Y	-	-	-
ENSG00000245937	LINC01184	-0.463065172	4.61E-05	-0.120209761	0.491847	-0.087797138	0.587202	0.349091368	0.004025	0.38983329	0.000474	0.035448484	0.904875	C4	-	Y	-	-	Y	Y	-
ENSG00000246067	RAB30-DT	-0.54567184	0.000246	0.099238462	0.688778	0.247305426	0.147796	0.651165154	1.18E-05	0.808214681	8.71E-10	0.151855184	0.556648	C2	-	Y	-	-	Y	Y	-
ENSG00000246263	UBR5-AS1	-0.710374868	0.328514	0.357226467	0.627178	0.893705173	0.068826	1.077297616	0.108451	1.618274963	0.003134	0.541294396	NA	C2	-	-	-	-	-	Y	-
ENSG00000246273	SBF2-AS1	-0.535142923	0.011422	-0.072018458	0.831756	-0.107482327	0.715619	0.470718581	0.010138	0.442984413	0.017364	-0.03360028	0.94118	C4	-	Y	-	-	Y	Y	-
ENSG00000246334	PRR7-AS1	0.609449972	0.028966	0.622765738	0.029145	-0.155093297	0.712962	0.019895649	0.957671	-0.749792498	0.00764	-0.774938533	0.01558	C1	-	Y	Y	-	-	Y	Y
ENSG00000246339	EXTL3-AS1	-0.559324479	0.133312	0.243229293	0.620112	0.010725601	0.986169	0.809435588	0.005536	0.585392451	0.081292	-0.22942014	NA	C4	-	-	-	-	Y	-	-
ENSG00000246695	RASSF8-AS1	-0.252046826	0.063373	0.330016851	0.021037	0.18317062	0.234145	0.58848355	1.25E-06	0.450117397	0.00029	-0.143686945	0.498637	C4	-	-	Y	-	Y	Y	-
ENSG00000246859	STARD4-AS1	-0.489633346	3.94E-08	-0.016227215	0.92817	-0.163271086	0.159539	0.479351453	3.71E-09	0.340273418	2.69E-05	-0.144124277	0.304072	C4	-	Y	-	-	Y	Y	-
ENSG00000246898	LINC00920	-1.884131176	1.38E-06	-0.250414708	0.612745	-0.895198058	0.013288	1.642382649	0.000462	1.003085158	0.057781	-0.642466181	NA	C3	-	Y	-	Y	Y	-	-
ENSG00000246922	UBAP1L	0.571435322	0.00339	0.230120202	0.383616	0.30596052	0.23932	-0.335658887	0.064764	-0.25098303	0.230774	0.078773568	0.855994	C1	-	Y	-	-	-	-	-
ENSG00000247081	BAALC-AS1	1.798122006	2.32E-12	-0.157121406	0.815832	0.216874562	0.689149	-1.954190737	1.85E-13	-1.568912622	2.27E-10	0.377539823	NA	C5	-	Y	-	-	Y	Y	-
ENSG00000247095	MIR210HG	-0.363723583	0.111963	0.504376049	0.023887	0.13708427	0.679419	0.875218882	1.00E-05	0.51606167	0.033936	-0.365413603	0.270818	C2	-	-	Y	-	Y	Y	-
ENSG00000247271	ZBED5-AS1	-1.323321694	2.25E-08	-0.326436036	0.291629	-0.563948285	0.028943	1.004103852	4.81E-05	0.77477758	0.002326	-0.233892993	0.580345	C3	-	Y	-	Y	Y	Y	-
ENSG00000247315	ZCCHC3	0.072894114	0.396239	-0.296699885	0.000534	-0.112131165	0.278116	-0.362903479	5.60E-08	-0.170644705	0.019536	0.187397984	0.087997	C4	-	-	-	-	Y	-	-
ENSG00000247516	MIR4458HG	-0.232718499	0.050608	0.071452549	0.679083	-0.319111161	0.012332	0.3103623	0.010678	-0.0716846	0.66828	-0.387506696	0.008404	C4	-	-	-	Y	Y	-	Y
ENSG00000247572	CKMT2-AS1	-0.357605608	0.013302	0.190087795	0.322556	0.118483109	0.530512	0.554629513	6.55E-05	0.491153222	0.000181	-0.068240278	0.826688	C4	-	Y	-	-	Y	Y	-
ENSG00000247596	TWF2	-0.406494947	2.82E-05	-0.235586186	0.045696	-0.210241284	0.084683	0.17740425	0.078651	0.210796742	0.039288	0.028219668	0.910675	C4	-	Y	-	-	-	-	-
ENSG00000247626	MARS2	0.530378012	2.04E-06	-0.226025068	0.19249	0.18222117	0.244804	-0.749925055	3.34E-11	-0.333524884	0.002858	0.41087288	0.0098	C5	-	Y	-	-	Y	Y	Y
ENSG00000247627	MTND4P12	-0.995460145	0.038917	-0.580113287	0.315689	-0.821416108	0.114546	0.421971169	0.477354	0.189828887	0.783236	-0.238619522	NA	C4	-	Y	-	-	-	-	-
ENSG00000247708	STX18-AS1	-0.607711825	0.000361	-0.019059586	0.949349	-0.052779161	0.828016	0.594803109	0.000163	0.569343397	0.000239	-0.031174314	0.933843	C2	-	Y	-	-	Y	Y	-
ENSG00000247828	TMEM161B-AS1	-0.138390801	0.268777	0.274262054	0.008761	0.347857111	0.001473	0.419433573	6.46E-05	0.500982803	4.03E-06	0.07624097	0.709413	C4	-	-	-	Y	Y	Y	-
ENSG00000248008	NRAV	0.379740297	0.002313	0.375601524	0.008178	0.605707162	9.20E-07	0.00218723	0.988994	0.240498088	0.039095	0.232820097	0.137224	C4	-	Y	Y	Y	-	-	-
ENSG00000248079	DPH6-DT	-2.027369191	9.47E-06	-0.596445202	0.221594	-0.539878398	0.264113	1.443126798	0.01152	1.501725375	0.008735	0.055434664	NA	C3	-	Y	-	-	Y	Y	-
ENSG00000248174	LINC02268	-0.749907617	6.93E-17	-0.577812061	5.04E-10	-0.32187827	0.000408	0.178106085	0.126344	0.442740056	3.91E-06	0.258811256	0.035627	C4	-	Y	Y	Y	-	Y	-
ENSG00000248275	TRIM52-AS1	-0.399321962	0.033506	0.116585736	0.660755	-0.227213676	0.412607	0.522981446	0.004942	0.186621161	0.48458	-0.342541986	0.287789	C4	-	Y	-	-	Y	-	-
ENSG00000248323	LUCAT1	-1.367773096	0.021355	-0.832494642	0.205429	-1.519716699	0.013217	0.543716468	0.513094	-0.138542326	0.89915	-0.685617039	NA	C3	-	Y	-	Y	-	-	-
ENSG00000248487	ABHD14A	-0.756867479	1.06E-05	-0.283031798	0.172292	-0.376228319	0.051774	0.480847824	0.011667	0.39559211	0.050872	-0.090281219	0.817441	C2	-	Y	-	-	Y	-	-
ENSG00000248498	ASNSP1	-2.957158076	8.41E-17	-1.471369214	2.08E-07	-1.040102499	0.000244	1.494294581	0.002499	1.934820233	1.80E-05	0.435178106	NA	C3	-	Y	Y	Y	Y	Y	-
ENSG00000248508	SRP14-AS1	-0.707871013	4.88E-06	0.057748223	0.770085	0.058828037	0.752373	0.773359828	2.81E-07	0.781014341	2.26E-07	0.003954951	0.991437	C3	-	Y	-	-	Y	Y	-
ENSG00000248527	MTATP6P1	-0.552358197	3.87E-05	-0.597364598	0.00295	-0.332878287	0.04746	-0.037565044	0.848097	0.234889584	4.21E-06	0.267472755	0.19888	C3	-	Y	Y	Y	-	-	-
ENSG00000248668	OXCT1-AS1	-0.255473327	0.625757	0.765893123	0.072481	0.260099302	0.653432	1.028498699	0.007543	0.530348497	0.26958	-0.50310985	NA	C2	-	-	-	-	Y	-	-
ENSG00000248727	LINC01948	-1.170751252	1.96E-13	-0.39226257	0.020103	-0.687577042	2.32E-05	0.786283006	2.13E-05	0.497851203	0.018339	-0.293321685	0.274702	C3	-	Y	Y	Y	Y	Y	-
ENSG00000248866	USP46-DT	-1.229293034	2.34E-05	-0.078123792	0.856621	-0.368058475	0.230215	1.158739578	0.000202	0.87554701	0.008005	-0.287173165	NA	C3	-	Y	-	-	Y	Y	-
ENSG00000249129	SUDS3P1	0.906078911	0.00023	0.666697905	0.036165	0.426452891	0.18217	-0.233128783	0.466766	-0.464720814	0.086358	-0.236833796	0.649942	C5	-	Y	Y	-	-	-	-
ENSG00000249131	PSD2-AS1	-2.300532138	0.063809	0.303089664	NA	-0.586330219	NA	2.616630889	0.045817	1.728653875	NA	-0.889284164	NA	C2	-	-	-	-	Y	-	-
ENSG00000249306	LINC01411	-0.961624215	0.121242	0.279901605	0.716237	-0.650355455	0.343372	1.253114055	0.048292	0.325936092	0.720075	-0.929621935	NA	C2	-	-	-	-	Y	-	-
ENSG00000249307	LINC01088	-1.368054985	0.008989	-0.415292369	0.502516	-0.327406346	0.578004	0.957939271	0.127754	1.055414436	0.079001	0.090952308	NA	C3	-	Y	-	-	-	-	-
ENSG00000249378	LINC01060	-1.441986625	0.04754	-0.803967723	NA	-0.750582904	0.38775	0.644864404	0.591922	0.707546745	0.482785	0.061117225	NA	C4	-	Y	-	-	-	-	-
ENSG00000249395	CASC9	-0.929215314	0.00033	-0.526456835	0.026535	-0.609128269	0.011575	0.410983462	0.18973	0.33585172	0.320007	-0.080089351	0.890821	C3	-	Y	Y	Y	-	-	-
ENSG00000249421	ADAMTS19-AS1	-0.7380827	0.007141	-0.040215953	0.93176	0.016612317	0.97107	0.705195213	0.01627	0.76896945	0.00702	0.059099804	0.934528	C2	-	Y	-	-	Y	Y	-
ENSG00000249471	ZNF324B	0.58262252	5.52E-06	0.159427274	0.442617	0.304845998	0.061384	-0.416652723	0.002278	-0.263192321	0.060038	0.14830048	0.564182	C5	-	Y	-	-	Y	-	-
ENSG00000249550	LINC01234	-0.416112052	0.028267	-0.165822795	0.56241	-0.185746791	0.491071	0.257012165	0.268853	0.245320163	0.303777	-0.017930799	0.9772	C4	-	Y	-	-	-	-	-
ENSG00000249846	LINC02021	-0.775532863	0.12695	0.495151515	0.346721	-0.125995221	0.857792	1.277418565	0.003306	0.664197136	0.225111	-0.618015698	NA	C2	-	-	-	-	Y	-	-
ENSG00000249853	HS3ST5	-0.610470665	0.004968	-0.406674371	0.149929	-0.521307905	0.0216	0.209764032	0.530377	0.103613789	0.75948	-0.112500653	0.85031	C4	-	Y	-	Y	-	-	-
ENSG00000250067	YJEFN3	-0.305272167	0.002918	-0.168170322	0.18962	-0.086944041	0.511217	0.143735027	0.212013	0.233381835	0.019967	0.083803759	0.641552	C4	-	Y	-	-	-	-	-
ENSG00000250091	DNAH10OS	0.664588859	0.000455	0.25187022	0.343678	0.412302783	0.049667	-0.40603925	0.026881	-0.237879362	0.198458	0.162440451	0.602159	C1	-	Y	-	Y	Y	-	-
ENSG00000250151	ARPC4-TTLL3	1.303907525	2.32E-05	0.64236287	0.132551	0.859086805	0.036519	-0.654432493	0.039974	-0.430293054	0.231782	0.219954711	NA	C5	-	Y	-	Y	Y	-	-
ENSG00000250251	PKD1P6	0.47642566	6.44E-05	0.212776846	0.141642	0.212100958	0.141316	-0.257264425	0.011449	-0.249717578	0.019295	0.002285937	0.993919	C1	-	Y	-	-	-	-	-
ENSG00000250303	LINC02762	-0.860667208	0.03269	0.379078879	0.32234	0.04973425	0.925178	1.247182878	0.000761	0.924663205	0.026112	-0.326522317	NA	C2	-	Y	-	-	Y	Y	-
ENSG00000250317	SMIM20	-0.323645595	0.007663	-0.225702399	0.099649	-0.266824501	0.044229	0.104379536	0.421244	0.072049697	0.615007	-0.037704335	0.881861	C4	-	Y	-	-	-	-	-
ENSG00000250337	PURPL	-0.340515012	0.257854	0.52683049	0.061607	0.463952124	0.081612	0.873676749	0.000959	0.819251713	0.001553	-0.060054013	0.921602	C4	-	-	-	-	Y	Y	-
ENSG00000250366	TUNAR	-0.445207073	0.0213	-0.523412761	0.021962	-0.019343399	0.956889	-0.072390626	0.824976	0.440569529	0.066603	0.508900503	0.133153	C4	-	Y	Y	-	-	-	-
ENSG00000250432	FAM242C	-1.452318084	0.002441	-0.190968441	0.770038	-0.497317653	0.356627	1.26680776	0.019209	0.969998299	0.106928	-0.302865322	NA	C3	-	Y	-	-	Y	-	-
ENSG00000250462	LRRC37BP1	0.254735023	0.009646	-0.060731128	0.713698	-0.042108076	0.802553	-0.309108535	0.002373	-0.282034075	0.007718	0.021532662	0.943467	C5	-	-	-	-	Y	-	-
ENSG00000250479	CHCHD10	-0.563421469	0.00442	-0.234788824	0.3239	-0.288429432	0.17843	0.335001092	0.12256	0.289679913	0.19061	-0.050689457	0.912195	C3	-	Y	-	-	-	-	-
ENSG00000250483	PPM1AP1	4.23909083	0.015238	3.200061651	NA	3.006222445	NA	-1.009179054	NA	-1.211054883	0.427505	-0.202598098	NA	C5	-	Y	-	-	-	-	-
ENSG00000250486	FAM218A	-1.70684052	0.033327	-0.042942267	0.970129	-0.033089738	0.975315	1.670171152	0.040832	1.687860321	0.03643	0.012165414	NA	C3	-	Y	-	-	Y	Y	-
ENSG00000250565	ATP6V1E2	-0.946001397	1.32E-23	-0.19567111	0.068694	-0.149386173	0.172586	0.756974724	7.31E-14	0.811226085	6.51E-16	0.049123576	0.815747	C2	-	Y	-	-	Y	Y	-
ENSG00000250571	GLI4	-0.306835524	0.014646	0.219628333	0.127443	0.002058219	0.992725	0.533082779	3.47E-06	0.323751991	0.012887	-0.214625377	0.227894	C4	-	Y	-	-	Y	Y	-
ENSG00000250802	ZBED3-AS1	-1.242766016	4.79E-16	-0.554000453	0.000655	-0.350533794	0.049093	0.695645	6.74E-05	0.907225877	1.70E-07	0.206263167	0.46654	C3	-	Y	Y	Y	Y	Y	-
ENSG00000250903	GMDS-DT	-0.866256019	0.003107	-0.162510752	0.697879	0.184395033	0.594362	0.712458191	0.030121	1.064929661	0.000181	0.34971039	0.423104	C4	-	Y	-	-	Y	Y	-
ENSG00000250959	GLUD1P3	0.515861241	0.008285	0.637049296	0.001229	0.242217118	0.356499	0.127242432	0.570704	-0.25932725	0.242473	-0.391995703	0.128177	C1	-	Y	Y	-	-	-	-
ENSG00000250986	LINC02600	-0.858021089	0.000268	-0.764747504	0.009388	-0.320872559	0.224998	0.098369198	0.823377	0.551758082	0.046935	0.447038761	NA	C4	-	Y	Y	-	-	Y	-
ENSG00000251192	ZNF674	0.305280376	0.0766	-0.093397053	0.758346	0.065207644	0.813206	-0.392684888	0.035092	-0.225830097	0.235013	0.161503098	0.654522	C5	-	-	-	-	Y	-	-
ENSG00000251247	ZNF345	-0.207846689	0.153175	-0.323581516	0.049912	-0.236755814	0.153412	-0.10944984	0.566311	-0.014471948	0.947483	0.090133306	0.78739	C4	-	-	Y	-	-	-	-
ENSG00000251287	ALG1L2	-1.885946451	0.00858	-0.480043576	0.558263	-0.629422834	0.403906	1.412973178	0.109979	1.272581428	0.172247	-0.146409657	NA	C3	-	Y	-	-	-	-	-

ENSG00000251322	SHANK3	0.302612969	0.002612	0.130691343	0.360551	0.285131923	0.009843	-0.165653013	0.1295	-0.002690646	0.982914	0.157347792	0.310184	C5	-	Y	-	-	-	-	-
ENSG00000251323	LINC02728	-1.419767277	0.000329	0.255979555	0.549404	-0.898819433	0.024847	1.682205692	8.17E-06	0.53654031	0.34721	-1.151543122	NA	C3	-	Y	-	Y	Y	-	-
ENSG00000251365	LINC02236	-1.140746544	0.000989	-0.699658726	0.076281	-0.37461132	0.371598	0.442376457	0.380447	0.779907247	0.070066	0.329856012	NA	C4	-	Y	-	-	-	-	-
ENSG00000251396	LINC01301	-1.52221836	0.013673	-0.203385189	0.806019	-0.26744845	0.709445	1.322008867	0.061048	1.269223747	0.07172	-0.060609237	NA	C3	-	Y	-	-	-	-	-
ENSG00000251432	LINC02615	-0.693775014	0.081311	0.263541579	0.557171	0.286477029	0.542139	0.965263317	0.010635	0.994932426	0.015394	0.025203061	NA	C2	-	-	-	-	Y	Y	-
ENSG00000251474	RPL32P3	0.284493436	0.014219	0.419742272	0.000596	0.330531449	0.011093	0.141629521	0.278314	0.060529103	0.697099	-0.086692342	0.713445	C4	-	-	Y	Y	-	-	-
ENSG00000251521	IMPA1P1	-2.925060668	0.040148	-1.934801274	NA	-2.263675696	NA	1.023464172	NA	0.682460339	NA	-0.340768049	NA	C3	-	Y	-	-	-	-	-
ENSG00000251573	LINC02106	-0.685269442	0.015821	-0.206041671	0.611384	-0.520052281	0.105839	0.486173083	0.139548	0.179540369	0.660863	-0.311634446	NA	C4	-	Y	-	-	-	-	-
ENSG00000251595	ABCA11P	-0.609907948	0.016575	-0.259144484	0.430349	-0.500652541	0.07857	0.356999486	0.213831	0.123832673	0.732797	-0.238471054	0.601273	C4	-	Y	-	-	-	-	-
ENSG00000251821	RNU6-583P	1.494119922	0.010878	1.290709823	0.054494	1.423312907	0.020802	-0.197094498	0.771701	-0.056602234	0.936346	0.134684047	NA	C1	-	Y	-	Y	-	-	-
ENSG00000251880	RNU7-75P	-1.466092092	0.05201	0.620912696	0.411318	-1.106683599	0.167766	2.094171756	0.001794	0.372003776	0.74919	-1.724072286	NA	C2	-	-	-	-	Y	-	-
ENSG00000251939	RNU6-1278P	3.714668791	0.035067	2.00402014	NA	1.709366475	NA	-1.683936248	NA	-1.987520289	0.259636	-0.293806271	NA	C5	-	Y	-	-	-	-	-
ENSG00000252947	SCARNA1	4.778854714	0.002387	2.184918407	NA	4.277659578	NA	-2.671052204	NA	-0.495648946	0.720075	2.157073819	NA	C5	-	Y	-	-	-	-	-
ENSG00000253230	MIR124-1HG	0.117084478	0.382137	0.26138912	0.010067	0.367668932	0.000557	0.151112986	0.239337	0.265448185	0.028131	0.109118423	0.551714	C4	-	-	-	Y	-	-	-
ENSG00000253250	C8orf88	-0.669674568	0.003532	-0.294944362	0.294158	-0.197681304	0.464077	0.379958961	0.162866	0.486830123	0.047862	0.099931102	0.838445	C3	-	Y	-	-	-	Y	-
ENSG00000253276	CCDC71L	-0.550953292	1.40E-12	-0.178220827	0.070952	-0.335267134	0.000824	0.379348895	1.62E-06	0.230336987	0.016564	-0.153831734	0.286084	C4	-	Y	-	Y	Y	-	-
ENSG00000253320	AZIN1-AS1	-0.71985153	0.000682	0.051711313	0.88354	0.150064643	0.571368	0.778812053	0.000712	0.884252291	3.95E-05	0.10068356	0.827579	C4	-	Y	-	-	Y	Y	-
ENSG00000253457	SMIM18	-0.764236848	4.54E-05	-0.150449317	0.551743	-0.142968588	0.551839	0.620609114	0.00113	0.636271387	0.000849	0.010278412	0.982359	C4	-	Y	-	-	Y	Y	-
ENSG00000253540	FAM86HP	-1.037772464	0.006415	0.016512555	0.977723	-0.266192049	0.563685	1.061431816	0.003112	0.787025753	0.053244	-0.279215903	NA	C3	-	Y	-	-	Y	-	-
ENSG00000253716	MINCR	-0.767487603	0.000319	-0.077446178	0.817488	-0.161769309	0.558975	0.696908906	0.000436	0.621106019	0.002131	-0.080985285	0.843648	C2	-	Y	-	-	Y	Y	-
ENSG00000253948	VPS13B-DT	-0.268209985	0.563174	0.469703496	0.343678	0.433568367	0.296336	0.744691336	0.064105	0.716475689	0.046517	-0.030887506	NA	C4	-	-	-	-	-	Y	-
ENSG00000254004	ZNF260	0.249495881	0.001611	-0.071926471	0.565978	0.069900255	0.576101	-0.314427725	5.50E-09	-0.165518197	0.009567	0.144163511	0.1507	C5	-	-	-	-	Y	-	-
ENSG00000254294	IMPDH1P6	-3.233332115	0.042112	-0.647232028	NA	-1.024821939	NA	2.578174876	NA	2.219874142	NA	-0.367769475	NA	C4	-	Y	-	-	-	-	-
ENSG00000254338	MAFA-AS1	-2.277089755	0.027022	-1.611097717	NA	-1.644432206	NA	0.671486287	NA	0.642400409	NA	-0.032559184	NA	C3	-	Y	-	-	-	-	-
ENSG00000254427	LINC02696	-0.53173546	0.192637	0.381052251	0.456828	-0.157751947	0.75227	0.920295475	0.029698	0.387884413	0.395907	-0.536590692	NA	C4	-	-	-	-	Y	-	-
ENSG00000254518	LINC02750	-0.355058775	0.657245	0.169592675	NA	0.854275146	0.15696	0.528662292	0.513371	1.223180568	0.035638	0.683337875	NA	C2	-	-	-	-	-	Y	-
ENSG00000254535	PABPC4L	-2.042309504	0.021317	0.225570605	NA	-0.277733103	0.772191	2.277040523	0.01082	1.77855069	0.07663	-0.500980809	NA	C3	-	Y	-	-	Y	-	-
ENSG00000254634	SMG1P6	1.19180167	0.032711	0.591624876	0.427814	0.483400047	0.519846	-0.593743426	0.268373	-0.69182486	0.185225	-0.104113101	NA	C5	-	Y	-	-	-	-	-
ENSG00000254635	WAC-AS1	-0.512973456	2.26E-12	-0.182921117	0.051322	-0.165669723	0.05448	0.3366312	3.93E-05	0.362068896	2.34E-06	0.020320379	0.924406	C4	-	Y	-	-	Y	Y	-
ENSG00000254656	RTL1	0.202284309	0.002382	-0.128634341	0.273843	0.053069802	0.682112	-0.324506223	8.96E-05	-0.134823338	0.330142	0.186022839	0.461613	C5	-	-	-	-	Y	-	-
ENSG00000254726	MEX3A	0.321604575	1.64E-16	0.093968002	0.061652	0.121452738	0.011907	-0.221237323	3.56E-12	-0.185493103	1.51E-08	0.030337521	0.677722	C5	-	Y	-	-	-	-	-
ENSG00000254788	CKLF-CMTM1	1.662503309	0.000474	1.100078226	0.058134	0.950992933	0.118323	-0.556282147	0.228201	-0.696438263	0.135515	-0.145596799	NA	C1	-	Y	-	-	-	-	-
ENSG00000254815	LMNTD2-AS1	-0.679080873	0.025018	-0.216933446	0.60877	0.20146154	0.606832	0.46819301	0.165582	0.895270091	0.002271	0.421164049	0.341115	C4	-	Y	-	-	-	Y	-
ENSG00000254860	TMEM9B-AS1	-0.264907751	0.236482	0.599797068	0.003494	0.459499112	0.022623	0.871219356	6.57E-06	0.738997176	0.000126	-0.137367483	0.703138	C2	-	-	Y	Y	Y	Y	-
ENSG00000254995	STX16-NPEPL1	0.475018357	0.021533	0.310940535	0.229187	-0.188066655	0.519529	-0.158613041	0.547028	-0.648388571	0.003139	-0.495381437	0.101407	C5	-	Y	-	-	-	Y	-
ENSG00000254996	ANKHD1-EIF4EBP3	0.422646291	0.011072	0.232420815	0.300845	0.289743493	0.137901	-0.183597593	0.315098	-0.117755184	0.535535	0.06013122	0.871807	C5	-	Y	-	-	-	-	-
ENSG00000255008	LINC02739	-4.443865647	0.005779	-0.455050706	NA	-0.750617319	NA	4.006748935	NA	3.715372661	NA	-0.295195191	NA	C3	-	Y	-	-	-	-	-
ENSG00000255052	FAM66D	-1.339373623	2.01E-06	-0.001760509	0.997102	-0.188603883	0.613549	1.341914114	8.76E-06	1.165012396	0.000388	-0.182870848	NA	C3	-	Y	-	-	Y	Y	-
ENSG00000255079	LINC02704	-3.144977847	7.13E-05	-0.923518948	0.192199	-0.921698597	0.146019	2.212888398	0.033403	2.236624788	0.024476	0.009578498	NA	C3	-	Y	-	-	Y	Y	-
ENSG00000255121	CCDC84-DT	-0.819748079	5.14E-05	-0.183031278	0.491974	-0.15724414	0.560509	0.642635223	0.004136	0.677233884	0.003165	0.028881803	0.958884	C4	-	Y	-	-	Y	Y	-
ENSG00000255152	MSH5-SAPCD1	-0.597092133	0.000162	-0.247193911	0.184136	-0.111018195	0.585852	0.35618878	0.04781	0.501112266	0.002502	0.139232755	0.614845	C4	-	Y	-	-	Y	Y	-
ENSG00000255248	MIR100HG	1.252030245	1.15E-08	0.355553479	0.380188	0.094410385	0.840345	-0.890829099	9.07E-05	-1.142134513	1.78E-07	-0.258225618	NA	C5	-	Y	-	-	Y	Y	-
ENSG00000255433	LINC02735	-0.541013521	5.53E-07	0.376419672	0.000465	0.691680971	3.40E-14	0.924238919	5.90E-19	1.247382737	3.18E-39	0.318387314	0.004545	C4	-	Y	Y	Y	Y	Y	Y
ENSG00000255441	SIGLEC10-AS1	-1.833850411	0.191703	0.567120327	NA	0.08525236	NA	2.408609552	0.035822	1.936470552	0.141785	-0.482680368	NA	C2	-	-	-	-	Y	-	-
ENSG00000255571	MIR9-3HG	-0.202044726	0.118505	-0.304959343	0.035155	-0.157585841	0.283654	-0.096702981	0.497484	0.058652329	0.674917	0.150386056	0.422915	C4	-	-	Y	-	-	-	-
ENSG00000255618	LINC02440	-1.09003482	0.02576	-0.24112861	0.705764	-0.30048125	0.613825	0.853994584	0.118241	0.804310466	0.159579	-0.055897963	NA	C3	-	Y	-	-	-	-	-
ENSG00000255624	C10orf88B	-0.827179727	0.011124	-0.283782043	0.419009	-0.274547735	0.454812	0.549826509	0.142179	0.567439659	0.148749	0.012169322	NA	C3	-	Y	-	-	-	-	-
ENSG00000255650	FAM222A-AS1	0.047048541	0.698962	0.390481637	1.11E-05	0.157242063	0.134211	0.350058602	1.17E-07	0.124846227	0.120277	-0.230648482	0.003008	C4	-	-	Y	-	Y	-	-
ENSG00000255690	TRIL	-0.90202878	6.50E-10	-0.21652054	0.278274	-0.803906235	1.29E-06	0.692077939	9.23E-06	0.113332788	0.625477	-0.584778878	0.004545	C4	-	Y	-	Y	Y	-	Y
ENSG00000255737	AGAP2-AS1	-0.164217877	0.272823	-0.207700743	0.210973	-0.383852555	0.017631	-0.036744507	0.850267	-0.204705536	0.231363	-0.173610737	0.50724	C5	-	-	-	Y	-	-	-
ENSG00000255837	TAS2R20	-0.932284409	0.064744	-0.968974533	0.077312	-1.057016555	0.042055	-0.031473626	0.971147	-0.109539531	0.900438	-0.084088078	NA	C3	-	-	-	Y	-	-	-
ENSG00000255857	PXN-AS1	-0.97719719	2.28E-05	-0.326504903	0.323928	-0.389558104	0.131261	0.655979723	0.022675	0.60245009	0.020719	-0.060231661	0.931045	C3	-	Y	-	-	Y	Y	-
ENSG00000256043	CTSO	-0.599870215	0.014162	-0.45723224	0.085277	-0.463322907	0.06055	0.151221942	0.666342	0.150794455	0.660707	-0.004113609	0.995809	C4	-	Y	-	-	-	-	-
ENSG00000256053	COA8	0.203022744	0.163243	-0.104853179	0.580533	0.024007916	0.908699	-0.301487277	0.029435	-0.164346859	0.267495	0.131601162	0.553021	C5	-	-	-	-	Y	-	-
ENSG00000256060	TRAPPC2B	-0.065263487	0.746571	-0.434955831	0.024049	-0.254613056	0.173116	-0.363693722	0.050788	-0.174609916	0.366657	0.183602985	0.56882	C5	-	-	Y	-	-	-	-
ENSG00000256073	URB1-AS1	-0.523474733	0.004155	-0.217429203	0.397875	-0.317449207	0.177129	0.312671178	0.148988	0.220706271	0.357315	-0.096629195	0.83987	C4	-	Y	-	-	-	-	-
ENSG00000256092	SBNO1-AS1	-0.431141464	0.284617	0.342689086	0.460836	-0.407759466	0.377841	0.779330575	0.019986	0.038608823	0.941127	-0.745967143	NA	C2	-	-	-	-	Y	-	-
ENSG00000256294	ZNF225	0.337274264	0.011326	-0.365044986	0.029869	0.105693405	0.56179	-0.695756299	4.75E-07	-0.216907523	0.124445	0.473520815	0.006925	C5	-	Y	Y	-	Y	-	Y
ENSG00000256612	CYP2B7P	4.636163247	0.0082	2.394703317	NA	3.607846985	NA	-2.203906623	NA	-1.012818972	0.492226	1.190205473	NA	C5	-	Y	-	-	-	-	-
ENSG00000256683	ZNF350	-0.345023209	0.027022	-0.118245727	0.677037	-0.341486771	0.05699	0.233146061	0.271979	0.018263101	0.940084	-0.22076829	0.506753	C4	-	Y	-	-	-	-	-
ENSG00000256746	MADD-AS1	2.004058845	0.00124	1.024311746	NA	1.360759738	0.106185	-0.974178668	0.112641	-0.6268048	0.316703	0.339615769	NA	C5	-	Y	-	-	-	-	-
ENSG00000257108	NHLRC4	-1.395326027	0.001321	-0.673060987	0.158725	-0.24807221	0.640055	0.730510339	0.190818	1.16287665	0.015364	0.427696676	NA	C3	-	Y	-	-	-	Y	-
ENSG00000257135	ODC1-DT	-2.301693923	0.006489	-1.052180253	NA	-0.915037187	0.257911	1.268679364	0.305789	1.403114393	0.228275	0.135428517	NA	C3	-	Y	-	-	-	-	-
ENSG00000257167	TMPO-AS1	-1.117436665	2.49E-11	-0.488922545	0.013249	-0.725005047	0.000136	0.634731871	0.000762	0.407105294	0.058761	-0.233791233	0.490789	C3	-	Y	Y	Y	Y	-	-
ENSG00000257185	LINC02293	-1.982881919	0.004866	-0.616288798	0.428121	-0.432452052	0.578486	1.377174223	0.094903	1.565961874	0.048091	0.186228741	NA	C3	-	Y	-	-	-	Y	-
ENSG00000257218	GATC	0.47883055	2.51E-08	0.218568205	0.034308	0.188571308	0.068326	-0.253888876	0.000624	-0.275851452	0.000187	-0.027395584	0.880846	C5	-	Y	-	-	-	-	-
ENSG00000257242	LINC01619	-1.167253376	0.000507	-0.863887791	0.020342	-1.243236336	0.000182	0.308194083	0.545114	-0.061273245	0.918451	-0.375750056	NA	C3	-	Y	Y	Y	-	-	-

ENSG00000257315	ZBED6	1.058875107	0.000598	0.28707282	0.570027	0.502653225	0.222122	-0.765640185	0.01728	-0.541815865	0.10064	0.218182378	NA	C5	-	Y	-	-	Y	-	-
ENSG00000257446	ZNF878	1.002445017	0.037606	0.482980631	0.531233	0.544393595	0.392251	-0.51346527	0.363736	-0.443055663	0.396699	0.064427342	NA	C1	-	Y	-	-	-	-	-
ENSG00000257556	LINC02298	-1.235457347	0.003003	-0.445168686	0.352833	-0.285019758	0.521417	0.792583269	0.14576	0.966637886	0.044228	0.165295851	NA	C3	-	Y	-	-	-	Y	-
ENSG00000257698	GIHCG	-0.485433095	0.00226	-0.105611033	0.647793	-0.079185509	0.692625	0.387393098	0.029127	0.421172359	0.007837	0.029810379	0.93984	C4	-	Y	-	-	Y	Y	-
ENSG00000257704	INAFM1	-0.181033462	0.404146	-0.156802012	0.591301	-0.43860826	0.044909	0.030997307	0.920169	-0.242854513	0.311878	-0.278815495	0.444313	C5	-	-	-	Y	-	-	-
ENSG00000257815	PRANCR	-0.430366381	0.141903	0.298350477	0.394887	0.266773685	0.427836	0.736774905	0.013473	0.712290725	0.015846	-0.030045958	0.96897	C2	-	-	-	-	Y	Y	-
ENSG00000257842	LINC02588	-2.370602898	0.001643	-1.162453772	NA	-0.501046191	0.514586	1.189734475	0.362166	1.882691654	0.047585	0.674815188	NA	C3	-	Y	-	-	-	Y	-
ENSG00000257913	DDN-AS1	-0.715716354	0.000397	-0.284851926	0.300845	-0.182650439	0.488757	0.436448486	0.089333	0.547817541	0.018416	0.105241156	0.835336	C4	-	Y	-	-	-	Y	-
ENSG00000258057	BCDIN3D-AS1	-0.694434414	0.023102	-0.230601897	0.575725	0.260308524	0.433461	0.470114395	0.130596	0.969831478	9.38E-05	0.493572971	0.128838	C4	-	Y	-	-	-	Y	-
ENSG00000258289	CHURC1	-0.661595049	4.19E-21	-0.141422606	0.126671	-0.242617247	0.002013	0.52668416	4.64E-12	0.43369414	1.08E-08	-0.098244873	0.45719	C2	-	Y	-	-	Y	Y	-
ENSG00000258388	PPT2-EGFL8	0.244106145	0.257181	-0.361089234	0.176641	-0.151484039	0.589194	-0.598518732	0.007535	-0.380595451	0.086498	0.212266364	0.614845	C5	-	-	-	-	Y	-	-
ENSG00000258405	ZNF578	-0.523756977	0.001672	-0.72554204	0.000102	-0.549130971	0.001409	-0.195337694	0.405618	-0.01071469	0.965562	0.179672618	0.609536	C3	-	Y	Y	Y	-	-	-
ENSG00000258498	DIO3OS	0.937084539	0.031889	-0.107344334	0.905519	-0.034116865	0.96874	-1.036254245	0.026457	-0.956545622	0.035751	0.07559405	NA	C5	-	Y	-	-	Y	Y	-
ENSG00000258499	LINC02287	-3.672468659	0.012799	-2.176054436	NA	-2.435081469	NA	1.476268864	NA	1.259308862	NA	-0.235227373	NA	C3	-	Y	-	-	-	-	-
ENSG00000258643	BCL2L2-PABPN1	1.143190525	0.030803	0.265373742	NA	0.408906196	0.609794	-0.868363089	0.159522	-0.719958121	0.197779	0.144747648	NA	C1	-	Y	-	-	-	-	-
ENSG00000258742	LINC02833	-4.353118089	0.008154	-2.410250329	NA	-1.663832831	NA	1.920784345	NA	2.714885977	NA	0.7786776	NA	C3	-	Y	-	-	-	-	-
ENSG00000258839	MC1R	-0.669710411	0.019806	-0.483934533	0.115421	-0.410781592	0.162046	0.19220002	0.606382	0.273673512	0.417974	0.07595605	NA	C3	-	Y	-	-	-	-	-
ENSG00000258913	LINC02691	-0.367732632	0.00107	0.067404735	0.713599	0.01761325	0.921934	0.442059583	0.000347	0.400041737	0.00046	-0.04695801	0.863083	C4	-	Y	-	-	Y	Y	-
ENSG00000258986	TMEM179	-0.382295857	1.31E-05	-0.047740892	0.745665	-0.114939263	0.286824	0.341189743	4.93E-05	0.282249029	0.000481	-0.064662237	0.673664	C4	-	Y	-	-	Y	-	-
ENSG00000259218	LINC00928	0.902306322	0.001965	0.625301439	0.09613	1.152826818	0.000117	-0.271250042	0.395329	0.264521679	0.368295	0.530838813	0.155779	C1	-	Y	-	Y	-	-	-
ENSG00000259291	ZNF710-AS1	-0.478090786	0.000411	-0.070490785	0.775102	-0.253325001	0.080656	0.413935339	0.011101	0.239742512	0.116166	-0.180111674	0.475258	C4	-	Y	-	-	Y	-	-
ENSG00000259343	TMC3-AS1	-0.628593354	0.114143	-0.056640268	0.918758	0.224504403	0.613549	0.579573597	0.171836	0.868453023	0.025894	0.283702128	NA	C4	-	-	-	-	-	Y	-
ENSG00000259399	TGIF2-RAB5IF	0.837227468	0.385969	-1.939581491	NA	0.382750454	NA	-2.781515851	0.021496	-0.44104082	0.667388	2.331865679	NA	C5	-	-	-	-	Y	-	-
ENSG00000259429	UBE2Q2P2	0.346518494	0.033615	0.356083639	0.078472	0.454974877	0.006198	0.014216366	0.9498	0.122797955	0.427885	0.103392366	0.714139	C1	-	Y	-	Y	-	-	-
ENSG00000259439	LINC01833	-0.443754415	1.21E-09	0.067566169	0.57733	-0.03478053	0.751391	0.517854208	3.87E-12	0.423935721	1.96E-10	-0.098977313	0.417908	C4	-	Y	-	-	Y	Y	-
ENSG00000259448	LINC02352	-1.751935115	0.002587	-0.10751174	0.88773	0.012407605	0.986747	1.6488255	0.008086	1.779221282	0.003072	0.122884414	NA	C3	-	Y	-	-	Y	Y	-
ENSG00000259836	AGGF1P6	0.014936349	0.97908	-1.044603153	0.08571	0.169709073	0.785381	-1.053868673	0.029038	0.168197762	0.731457	1.217287421	NA	C5	-	-	-	-	Y	-	-
ENSG00000259954	IL21R-AS1	-4.152025048	0.037828	-1.462092598	NA	-0.607802643	NA	2.69407484	NA	3.565320841	NA	0.859103104	NA	C4	-	Y	-	-	-	-	-
ENSG00000260097	SPDYE6	-0.212493339	0.561467	0.621700106	0.054302	0.420575809	0.16711	0.841762878	0.004052	0.647858059	0.022044	-0.196822435	0.686381	C2	-	-	-	-	Y	Y	-
ENSG00000260219	CD2BP2-DT	-0.553177358	0.026508	-0.370751649	0.236732	-0.25534867	0.419142	0.189191876	0.559801	0.312977131	0.280665	0.118605202	0.833878	C4	-	Y	-	-	-	-	-
ENSG00000260230	FRRS1L	-0.527858243	4.11E-06	-0.538813602	9.18E-06	-0.347688371	0.002977	-0.004616977	0.97944	0.194720021	0.13677	0.193824591	0.280134	C4	-	Y	Y	Y	-	-	-
ENSG00000260287	TBC1D3G	2.201362529	0.007787	1.647133696	NA	1.95853668	0.023386	-0.548622972	0.562232	-0.228027653	0.804857	0.314481342	NA	C1	-	Y	-	Y	-	-	-
ENSG00000260291	FRG2GP	-2.372133791	0.02415	-1.695567284	NA	-0.134097668	0.911222	0.687230336	NA	2.252847161	0.06095	1.565288052	NA	C3	-	Y	-	-	-	-	-
ENSG00000260428	SCX	-1.123452567	0.00262	-0.376773459	0.433794	-0.470004635	0.280582	0.751975434	0.097715	0.6680362	0.154268	-0.090209938	NA	C3	-	Y	-	-	-	-	-
ENSG00000260442	ATP2A1-AS1	-0.523573218	0.05545	-0.327127068	0.3783	-0.017887224	0.966843	0.203511414	0.53583	0.520486106	0.030173	0.312015449	0.430361	C4	-	-	-	-	-	Y	-
ENSG00000260455	NBAT1	-0.836389907	0.000492	-0.14561436	0.687775	-0.435975738	0.163664	0.69702666	0.003714	0.415367714	0.170104	-0.288459178	0.519048	C2	-	Y	-	-	Y	-	-
ENSG00000260518	BMS1P8	0.507959712	2.53E-05	-0.220947918	0.251001	0.120858855	0.494324	-0.722160448	4.50E-08	-0.37266501	0.002519	0.344323603	0.079744	C5	-	Y	-	-	Y	Y	-
ENSG00000260565	ERVK13-1	0.233718773	0.030213	0.363752659	0.002092	0.338454837	0.001807	0.135270755	0.162466	0.119279603	0.196147	-0.021402752	0.927111	C4	-	-	Y	Y	-	-	-
ENSG00000260589	STAM-AS1	-0.310639337	0.468308	0.064254567	0.919114	-1.225341293	0.007931	0.383294827	0.409014	-0.899030932	0.074733	-1.290195664	NA	C5	-	-	-	Y	-	-	-
ENSG00000260644	HERC2P5	1.044859281	0.043869	0.948265747	0.114654	0.970365389	0.069074	-0.0907093	0.886631	-0.061817216	0.917894	0.022932962	NA	C1	-	Y	-	-	-	-	-
ENSG00000260765	CES1P2	2.338997552	1.71E-07	0.880244437	0.299033	0.159461039	0.885991	-1.450601338	0.002002	-2.16989295	1.23E-06	-0.717891078	NA	C5	-	Y	-	-	Y	Y	-
ENSG00000260802	SERTM2	-1.080443231	0.010946	-0.476797652	0.408571	-0.396000152	0.480753	0.614627946	0.278794	0.699221766	0.198589	0.07872821	NA	C3	-	Y	-	-	-	-	-
ENSG00000260804	LINC01963	-0.705408656	3.05E-09	-0.151196267	0.313072	-0.253791198	0.068248	0.560979152	3.93E-09	0.466714624	1.18E-05	-0.09967016	0.564944	C2	-	Y	-	-	Y	Y	-
ENSG00000260852	FBXL19-AS1	0.320650226	0.022165	0.234627015	0.133587	-0.05283005	0.8004	-0.079524397	0.628744	-0.358948082	0.007693	-0.284867223	0.092592	C5	-	Y	-	-	-	Y	-
ENSG00000260903	XKR7	0.347198773	1.66E-09	0.122705857	0.166548	0.161442834	0.021565	-0.218051635	0.000334	-0.17122826	0.001434	0.041688425	0.752303	C5	-	Y	-	-	-	-	-
ENSG00000260916	CCPG1	0.687441272	9.66E-06	0.109471669	0.692114	0.27671421	0.178805	-0.571694369	3.29E-05	-0.396356064	0.004351	0.169964012	0.520704	C5	-	Y	-	-	Y	Y	-
ENSG00000261087	ZNNT1	1.506406283	0.001359	-0.093640791	0.933641	1.068996538	0.091764	-1.597938922	0.000126	-0.421666183	0.352542	1.167523427	NA	C1	-	Y	-	-	Y	-	-
ENSG00000261126	RBFADN	1.334749975	0.004855	1.015464502	0.140777	1.189769809	0.02546	-0.308566156	0.5548	-0.128740314	0.792035	0.174787936	NA	C1	-	Y	-	Y	-	-	-
ENSG00000261150	EPPK1	2.608205522	0.002278	1.727527648	NA	1.168268822	NA	-0.875797987	0.259812	-1.42642044	0.075848	-0.556512403	NA	C1	-	Y	-	-	-	-	-
ENSG00000261221	ZNF865	0.316418564	0.00464	0.210688375	0.145836	0.248167	0.057034	-0.099272692	0.409699	-0.053490083	0.678781	0.0405649	0.867365	C5	-	Y	-	-	-	-	-
ENSG00000261408	TEN1-CDK3	0.231696283	0.193237	-0.096908885	0.731587	-0.571670219	0.009004	-0.320863025	0.046577	-0.788072985	4.05E-06	-0.471381019	0.070162	C5	-	-	-	Y	Y	Y	-
ENSG00000261453	LINC01735	-0.947343895	0.004292	0.037675927	0.941382	-0.065312704	0.882257	0.993373032	0.003315	0.897538481	0.006705	-0.09948618	NA	C3	-	Y	-	-	Y	Y	-
ENSG00000261455	LINC01003	-0.324606084	0.03157	0.147679868	0.4219	-0.012728938	0.959696	0.478773413	0.001105	0.326613771	0.048199	-0.157625106	0.537085	C4	-	Y	-	-	Y	Y	-
ENSG00000261456	TUBB8	-3.720036759	0.008896	-1.036327943	NA	-0.714974181	NA	2.71433102	NA	3.024373507	0.08069	0.31671731	NA	C3	-	Y	-	-	-	-	-
ENSG00000261504	LINC01686	-2.369018358	0.009269	-0.590311883	NA	-0.9370165	0.317182	1.783724931	0.102464	1.448931981	0.206461	-0.342701584	NA	C4	-	Y	-	-	-	-	-
ENSG00000261556	SMG1P7	-0.079267042	0.585283	0.245491011	0.059516	0.208494138	0.123607	0.331027071	0.003586	0.3024802	0.011611	-0.034127526	0.902224	C4	-	-	-	-	Y	Y	-
ENSG00000261609	GAN	0.318060286	0.020475	0.039782634	0.863885	-0.0292219	0.901341	-0.271682081	0.043561	-0.332437701	0.015701	-0.066157049	0.82887	C5	-	Y	-	-	-	Y	-
ENSG00000261618	LINC02605	-0.396007038	0.055201	0.158575541	0.56679	-0.421255837	0.072008	0.560344343	0.009105	-0.01043123	0.974071	-0.576246987	0.03989	C2	-	-	-	-	Y	-	Y
ENSG00000261678	SCRT1	0.23002022	0.020183	0.303068373	0.02923	0.433356108	8.79E-06	0.079565958	0.589812	0.218329502	0.02505	0.132865078	0.512021	C4	-	-	Y	Y	-	-	-
ENSG00000261794	GOLGA8H	-1.569355014	0.000815	-0.124703417	0.846586	-0.233881163	0.664878	1.446757048	0.006209	1.349124489	0.011344	-0.105506831	NA	C3	-	Y	-	-	Y	Y	-
ENSG00000261796	ISY1-RAB43	4.227291166	0.013785	3.887521347	NA	3.194314972	NA	-0.323733145	NA	-1.000970212	0.547131	-0.679137265	NA	C1	-	Y	-	-	-	-	-
ENSG00000261934	PCDHGA9	-0.444671558	0.406088	0.511400894	0.333829	0.050444305	0.942501	0.962707324	0.039307	0.509482846	0.345871	-0.458019008	NA	C2	-	-	-	-	Y	-	-
ENSG00000262001	DLGAP1-AS2	-1.192585392	0.013239	0.073089538	0.914492	0.063574009	0.933328	1.274369723	0.007413	1.27173008	0.018249	-0.0076301	NA	C2	-	Y	-	-	Y	Y	-
ENSG00000262155	LINC02175	-0.223552193	0.539083	0.352886042	0.292967	0.633547106	0.032499	0.582339043	0.045521	0.871596174	0.002039	0.283605685	0.507587	C4	-	-	-	Y	Y	Y	-
ENSG00000262188	LINC01978	0.498956233	0.493697	-0.561607866	NA	-1.8497885	NA	-1.0533767	0.174276	-2.334067282	0.007742	-1.28541173	NA	C5	-	-	-	-	-	Y	-
ENSG00000262468	LINC01569	-0.908669665	0.045525	-0.256239517	0.665994	-0.075771905	0.913085	0.661450033	0.231793	0.846605537	0.125697	0.180245046	NA	C4	-	Y	-	-	-	-	-
ENSG00000262484	CCER2	-2.522139192	0.00088	-1.468148686	0.041767	-1.845852252	0.015251	1.065272347	0.323512	0.695785704	0.596499	-0.375631315	NA	C4	-	Y	Y	Y	-	-	-

ENSG00000262585	LINC01979	0.609176689	0.234041	0.368882809	0.583268	-1.202117205	0.126428	-0.233788876	0.690499	-1.795683163	0.004472	-1.570914239	NA	C5	-	-	-	-	-	Y	-
ENSG00000262655	SPON1	1.521304325	0.045814	0.270694649	NA	0.77934416	0.438396	-1.244393141	0.129446	-0.72852906	0.375183	0.511292587	NA	C5	-	Y	-	-	-	-	-
ENSG00000262919	CCNQ	0.354037236	0.042337	0.116771293	0.66559	0.039731931	0.890404	-0.231740901	0.161849	-0.299307244	0.055632	-0.072803469	0.833312	C5	-	Y	-	-	-	-	-
ENSG00000263001	GTF2I	0.3312659	1.98E-07	0.18110403	0.020739	0.10181026	0.197621	-0.143648761	0.000931	-0.214956697	3.86E-09	-0.07670551	0.220878	C5	-	Y	-	-	-	-	-
ENSG00000263002	ZNF234	0.414424209	1.66E-05	-0.101512675	0.502458	0.224483597	0.077584	-0.509547457	1.26E-09	-0.174769768	0.081651	0.329171593	0.005789	C5	-	Y	-	-	Y	-	Y
ENSG00000263069	RNF213-AS1	0.590050841	0.000197	0.441268534	0.014669	0.361868841	0.095097	-0.142575585	0.402457	-0.213052536	0.259033	-0.076272407	0.841371	C1	-	Y	Y	-	-	-	-
ENSG00000263072	ZNF213-AS1	-0.487967943	3.85E-06	0.027857955	0.878389	-0.121282444	0.410392	0.522352758	2.33E-07	0.38151973	0.000664	-0.146270061	0.396423	C4	-	Y	-	-	Y	Y	-
ENSG00000263327	TAPT1-AS1	-0.418783367	0.301501	0.457999426	0.254732	0.625543444	0.103244	0.884257783	0.012283	1.059747263	0.00327	0.169808402	NA	C4	-	-	-	-	Y	Y	-
ENSG00000263400	TMEM220-AS1	-1.535812452	0.089358	0.315137449	NA	0.35382173	0.745033	1.860392911	0.023159	1.904948012	0.044422	0.041152048	NA	C2	-	-	-	-	Y	Y	-
ENSG00000263412	NFE2L1-DT	-0.541442138	0.080994	0.380507883	0.196882	-0.062610705	0.876811	0.930407423	0.001106	0.493725019	0.136934	-0.440452912	0.213832	C2	-	-	-	-	Y	-	-
ENSG00000263513	FAM72C	0.42863798	0.059038	-0.021769287	0.961535	0.373914166	0.191372	-0.444769174	0.047384	-0.039826883	0.902328	0.398593507	0.254841	C4	-	-	-	-	Y	-	-
ENSG00000263818	RDM1P5	-0.980865733	0.082988	-0.694667525	0.329402	0.204483075	0.760007	0.296898128	0.735666	1.200119058	0.03157	0.903469723	NA	C4	-	-	-	-	-	Y	-
ENSG00000263874	LINC00672	-0.577775123	4.71E-05	-0.218445504	0.258941	-0.241818282	0.186894	0.365415601	0.02987	0.350867511	0.042699	-0.019812053	0.966518	C4	-	Y	-	-	Y	Y	-
ENSG00000264247	LINC00909	-0.468795722	6.97E-06	-0.219359196	0.086481	-0.397624063	0.000833	0.256159856	0.014869	0.086359714	0.526005	-0.175323732	0.28183	C4	-	Y	-	Y	-	-	-
ENSG00000264278	ZNF236-DT	-0.214690775	0.766689	0.804018873	0.113888	-0.092026884	0.91492	1.028096812	0.048557	0.137940399	0.871365	-0.894686133	NA	C2	-	-	-	-	Y	-	-
ENSG00000264522	OTUD7B	0.459956355	7.59E-10	0.123310844	0.33553	0.179064872	0.054679	-0.330218421	1.89E-05	-0.266302199	3.69E-05	0.058548731	0.728646	C5	-	Y	-	-	Y	-	-
ENSG00000264655	PPIAP54	-1.840469527	0.190912	0.794224188	NA	0.746362018	NA	2.642048343	0.024491	2.601356098	0.019222	-0.042252032	NA	C2	-	-	-	-	Y	Y	-
ENSG00000264720	MIR3117	-3.937385069	0.038291	0.197932697	NA	-2.592755353	NA	4.159266234	NA	1.353198695	NA	-2.797075333	NA	C2	-	Y	-	-	-	-	-
ENSG00000264745	TTC39C-AS1	-1.259357773	0.029803	-0.143619069	0.879551	0.238506986	0.722567	1.12736287	0.098677	1.512658536	0.005869	0.38802656	NA	C3	-	Y	-	-	-	Y	-
ENSG00000265096	C1QTNF1-AS1	-1.847852282	5.31E-14	-0.191460138	0.480774	-0.184542376	0.465241	1.664674996	3.50E-10	1.677921835	1.34E-10	0.009667293	0.986496	C3	-	Y	-	-	Y	Y	-
ENSG00000265293	ARGFXP2	-1.323030205	0.585482	1.542093148	NA	0.5697848	NA	2.873357347	0.043648	1.910865478	NA	-0.966981136	NA	C2	-	-	-	-	Y	-	-
ENSG00000265366	GLUD1P2	-0.653223	0.164487	0.288288388	0.617188	0.062482634	0.925854	0.950887375	0.043421	0.72983616	0.152921	-0.224779296	NA	C2	-	-	-	-	Y	-	-
ENSG00000265590	CFAP298-TCP10L	0.809187061	0.075198	0.269466749	0.709221	1.133843412	0.007262	-0.533351928	0.266974	0.339190814	0.431051	0.86723799	NA	C1	-	-	-	Y	-	-	-
ENSG00000265646	TUFMP1	-0.312649899	0.07877	0.297005425	0.15518	0.380358759	0.036766	0.616376571	0.000366	0.707721973	1.58E-05	0.085832793	0.821705	C4	-	-	-	Y	Y	Y	-
ENSG00000265688	MAFG-DT	-0.214154036	0.369101	0.230371044	0.376047	0.227211562	0.357738	0.451812592	0.024729	0.456532131	0.021196	0.000101935	0.999689	C4	-	-	-	-	Y	Y	-
ENSG00000265763	ZNF488	-0.554598545	0.330938	0.067877716	0.934027	0.603039941	0.229911	0.629444622	0.269368	1.172262206	0.010976	0.538792182	NA	C4	-	-	-	-	-	Y	-
ENSG00000265790	RNASEH1P1	-1.37430512	3.19E-05	-0.276565078	0.483963	-0.432633485	0.188638	1.100447147	0.003197	0.957117171	0.01093	-0.152245745	NA	C3	-	Y	-	-	Y	Y	-
ENSG00000265802	RN7SL49P	3.909856535	0.02726	2.3588261	NA	3.037393439	NA	-1.552042249	NA	-0.855619977	0.54488	0.692149486	NA	C5	-	Y	-	-	-	-	-
ENSG00000265806	MIR4292	0.634864481	0.024664	0.178837649	0.71323	0.140621963	0.754269	-0.450269919	0.151282	-0.479631277	0.111234	-0.035630315	NA	C5	-	Y	-	-	-	-	-
ENSG00000265817	FSBP	-0.950692473	3.99E-13	-0.1310839	0.514639	-0.663369256	7.79E-07	0.825723779	6.66E-08	0.30199052	0.083926	-0.529571697	0.00412	C3	-	Y	-	Y	Y	-	Y
ENSG00000265972	TXNIP	-0.968092619	7.85E-93	-0.1949444	0.020809	-0.032912735	0.66593	0.779789211	3.06E-34	0.950021657	7.90E-111	0.165126299	0.076138	C2	-	Y	-	-	Y	Y	-
ENSG00000266074	BAHCC1	-0.526647727	9.57E-14	-0.010694385	0.928309	-0.192083343	0.009661	0.522361188	6.15E-19	0.349080527	3.46E-08	-0.178794919	0.00987	C4	-	Y	-	-	Y	Y	-
ENSG00000266075	RN7SL574P	-2.343382267	0.043039	-1.16853119	NA	-1.764076653	NA	1.169602299	NA	0.592942702	NA	-0.584950082	NA	C4	-	Y	-	-	-	-	-
ENSG00000266236	NARF-IT1	-0.575252104	0.182954	0.323350324	0.510228	-0.096578513	0.870247	0.905695542	0.005207	0.493570063	0.207212	-0.417352608	NA	C2	-	-	-	-	Y	-	-
ENSG00000266392	MIR4740	-2.957600903	0.028515	-1.994933051	NA	-1.486843569	NA	1.001313738	NA	1.469560307	NA	0.480800519	NA	C3	-	Y	-	-	-	-	-
ENSG00000266405	CBX3P2	-1.354977587	0.000298	0.180115042	0.732041	-0.149437361	0.768708	1.542916079	1.92E-05	1.220440187	0.001318	-0.326201891	NA	C3	-	Y	-	-	Y	Y	-
ENSG00000266714	MYO15B	-0.676600516	1.86E-08	-0.3062679	0.099246	-0.085754469	0.688781	0.377389558	0.025003	0.605592678	3.72E-05	0.221446576	0.45687	C4	-	Y	-	-	Y	Y	-
ENSG00000266736	GTF2IP6	-1.083682443	0.000836	-0.266016229	0.517758	-0.168476351	0.677562	0.826679243	0.033174	0.930327369	0.011175	0.100147104	NA	C2	-	Y	-	-	Y	Y	-
ENSG00000266777	SH3GL1P1	0.782455796	0.033581	-0.134971247	0.857678	0.109677574	0.877682	-0.91161925	0.026012	-0.658767015	0.126778	0.247393721	NA	C5	-	Y	-	-	Y	-	-
ENSG00000266904	LINC00663	0.02723907	0.951338	0.627658502	0.01991	0.337288352	0.292926	0.60643205	0.01492	0.324408289	0.27564	-0.286694039	0.437474	C1	-	-	Y	-	Y	-	-
ENSG00000266916	ZNF793-AS1	-0.891919542	3.48E-05	-0.388261564	0.117254	-0.53413173	0.023174	0.509346075	0.045084	0.372554383	0.186147	-0.142462295	0.761933	C3	-	Y	-	Y	Y	-	-
ENSG00000267080	ASB16-AS1	-0.600712398	5.56E-05	-0.052598318	0.849901	-0.123029531	0.557332	0.554453949	0.000185	0.492579265	0.000412	-0.066744002	0.842935	C4	-	Y	-	-	Y	Y	-
ENSG00000267100	ILF3-DT	-0.615023817	7.86E-13	-0.067032401	0.602394	-0.39922364	5.94E-05	0.554683203	4.22E-10	0.230555454	0.037228	-0.329520763	0.0049	C4	-	Y	-	Y	Y	-	Y
ENSG00000267106	ZNF561-AS1	-0.6015029	0.000229	-0.225458656	0.269875	-0.268152591	0.175291	0.383399183	0.049328	0.348116715	0.091595	-0.040284738	0.933843	C4	-	Y	-	-	Y	-	-
ENSG00000267221	C17orf113	-0.574454486	0.033819	0.241065021	0.454883	0.058789999	0.887844	0.822987915	0.001667	0.648148158	0.026458	-0.179491982	0.71566	C2	-	Y	-	-	Y	Y	-
ENSG00000267254	ZNF790-AS1	-0.26530634	0.155393	0.185300627	0.455182	-0.062059868	0.809543	0.456480986	0.006816	0.217949455	0.210644	-0.243111712	0.32601	C4	-	-	-	-	Y	-	-
ENSG00000267278	MAP3K14-AS1	-0.689232921	0.036774	-0.162494216	0.686582	-0.003706077	0.993706	0.535049944	0.139043	0.69982382	0.048363	0.160444171	NA	C3	-	Y	-	-	-	Y	-
ENSG00000267280	TBX2-AS1	-0.064365039	0.551397	0.337054365	0.010538	0.255585354	0.005909	0.407576797	7.14E-05	0.334862299	2.34E-06	-0.078123162	0.70884	C4	-	-	Y	-	Y	Y	-
ENSG00000267296	CEBPA-DT	-1.426318034	0.019391	-0.471189604	0.557171	0.069408369	0.933328	0.959767854	0.228342	1.510783597	0.019636	0.544624051	NA	C4	-	Y	-	-	-	Y	-
ENSG00000267325	LINC01415	-0.946632535	0.000276	-0.465832207	0.12993	-0.297304263	0.364748	0.487362542	0.074718	0.664042367	0.011553	0.171012801	0.721353	C4	-	Y	-	-	-	Y	-
ENSG00000267454	ZNF582-AS1	-0.515062775	0.039013	-0.12586793	0.720939	-0.155518327	0.625147	0.395487437	0.164609	0.374636298	0.189808	-0.026331886	0.967582	C2	-	Y	-	-	-	-	-
ENSG00000267680	ZNF224	0.434484156	2.05E-05	0.193704458	0.119306	0.18118306	0.116498	-0.234418559	0.031646	-0.238594228	0.022103	-0.009795918	0.971963	C5	-	Y	-	-	-	-	-
ENSG00000267796	LIN37	0.455457334	0.002974	-0.02046667	0.950782	0.32270945	0.100251	-0.468537332	0.002675	-0.117907697	0.529469	0.345929913	0.148255	C5	-	Y	-	-	Y	-	-
ENSG00000267858	MZF1-AS1	0.244385428	0.180894	0.242945605	0.195016	0.373193499	0.041231	0.005511877	0.981252	0.143624206	0.50294	0.132513152	0.674063	C4	-	-	-	Y	-	-	-
ENSG00000267909	CCDC177	-1.784069329	0.00421	-0.490578191	0.473795	-0.017694046	0.982982	1.300223203	0.059211	1.782047143	0.004448	0.475386164	NA	C2	-	Y	-	-	-	Y	-
ENSG00000268061	NAPA-AS1	-0.82602668	0.032164	-0.132573191	0.829476	-0.287853738	0.556073	0.699435458	0.081845	0.551800327	0.178408	-0.152179517	NA	C2	-	Y	-	-	-	-	-
ENSG00000268089	GABRQ	-0.623805882	0.044575	-0.625494923	0.099714	-0.425318195	0.256406	0.004397977	0.992382	0.213806965	0.611459	0.204484305	NA	C3	-	Y	-	-	-	-	-
ENSG00000268471	MIR4453HG	-0.212115752	0.257954	0.187879628	0.217968	0.040375586	0.841146	0.405471375	0.016537	0.26735379	0.146936	-0.144383935	0.523946	C1	-	-	-	-	Y	-	-
ENSG00000268521	VN1R83P	1.215331532	0.014847	0.067956923	NA	0.027198551	0.979435	-1.142469603	0.028229	-1.172922165	0.029071	-0.038645058	NA	C5	-	Y	-	-	Y	Y	-
ENSG00000269113	TRABD2B	-0.084450545	0.938371	0.680422887	0.434149	1.172035061	0.072824	0.772220112	0.235289	1.273119863	0.01829	0.495937255	NA	C2	-	-	-	-	-	Y	-
ENSG00000269235	ZNF350-AS1	-1.8995781	0.000972	-0.519529245	0.431712	-0.924746143	0.07672	1.397555087	0.056786	0.991553358	0.199356	-0.40474306	NA	C3	-	Y	-	-	-	-	-
ENSG00000269343	ZNF587B	-0.862808453	4.28E-16	-0.195512509	0.23845	-0.646522024	3.95E-10	0.673878137	3.38E-07	0.231119698	0.074811	-0.448007154	0.004543	C3	-	Y	-	Y	Y	-	Y
ENSG00000269386	RAB11B-AS1	-1.109342523	0.002166	-0.33648228	0.508758	-0.36052388	0.391064	0.78077752	0.053667	0.764608944	0.039753	-0.020174145	NA	C4	-	Y	-	-	-	Y	-
ENSG00000269416	LINC01224	-0.39375396	8.53E-06	-0.019669114	0.903389	-0.14871228	0.193055	0.380598627	7.34E-06	0.259882494	0.004756	-0.12610499	0.379545	C4	-	Y	-	-	Y	-	-
ENSG00000269609	RPARP-AS1	-0.53567057	0.002504	0.101194024	0.710865	0.132795441	0.578625	0.642598712	2.19E-05	0.684036378	4.61E-06	0.036326005	0.927111	C4	-	Y	-	-	Y	Y	-
ENSG00000269713	NBPF9	0.332178847	2.35E-06	0.146921192	0.127714	0.13218437	0.131006	-0.178602026	0.014022	-0.185587231	0.005443	-0.012134563	0.951255	C5	-	Y	-	-	-	-	-
ENSG00000269821	KCNQ1OT1	-0.40537592	0.107195	-0.063664083	0.879527	0.391824567	0.086878	0.3504562	0.264165	0.811570029	0.000364	0.45806784	0.173277	C4	-	-	-	-	-	Y	-

ENSG00000269834	ZNF528-AS1	-0.656112616	9.42E-08	-0.182710129	0.291246	-0.19834039	0.176886	0.48047293	0.00091	0.472795925	0.000442	-0.012669027	0.974597	C2	-	Y	-	-	Y	Y	-
ENSG00000269929	MIRLET7A1HG	-0.489997002	0.049071	0.352144599	0.168148	-0.281363945	0.355573	0.848566919	4.10E-05	0.223217721	0.449075	-0.630788416	0.015734	C2	-	Y	-	-	Y	-	Y
ENSG00000270170	NCBP2AS2	-0.248191173	0.015125	-0.128150873	0.326985	-0.301145909	0.006877	0.126687734	0.2477	-0.03804282	0.783987	-0.170131925	0.244612	C4	-	-	-	Y	-	-	-
ENSG00000270580	PKD1P6-NPIPP1	0.429923759	0.028092	-0.021923391	0.95256	0.052230055	0.865322	-0.445400718	0.026797	-0.363211605	0.072742	0.077079506	0.866789	C5	-	Y	-	-	Y	-	-
ENSG00000270647	TAF15	-0.491319005	4.07E-16	-0.367604284	3.27E-09	-0.465724515	1.64E-14	0.130396863	0.022422	0.040544867	0.560085	-0.095351396	0.235768	C4	-	Y	Y	Y	-	-	-
ENSG00000270673	YTHDF3-AS1	-1.646328359	0.043376	-0.840956178	NA	-0.279805646	0.758271	0.810919357	0.489665	1.380916322	0.141637	0.564704648	NA	C3	-	Y	-	-	-	-	-
ENSG00000270816	LINC00221	0.188332474	0.007834	-0.030051046	0.815911	-0.148465038	0.108232	-0.211830143	0.005377	-0.322039231	1.43E-05	-0.11546771	0.397037	C5	-	-	-	-	-	Y	-
ENSG00000270872	SRGAP2D	0.634052111	0.004499	0.303783202	0.318657	0.276756003	0.320057	-0.324849894	0.159522	-0.343175381	0.111611	-0.023226693	0.967582	C5	-	Y	-	-	-	-	-
ENSG00000271086	NAMA	-0.863336861	0.080357	0.176423646	0.770457	0.425508232	0.419017	1.048920437	0.035034	1.302887081	0.009654	0.250823585	NA	C2	-	-	-	-	Y	Y	-
ENSG00000271147	ARMCX5-GPRASP2	-0.783077217	6.84E-07	-0.255934586	0.158707	-0.092594129	0.643597	0.533201494	0.002029	0.704876531	8.31E-06	0.166025361	0.503876	C2	-	Y	-	-	Y	Y	-
ENSG00000271161	BOLA2P2	-0.75789855	0.087999	-0.426278683	0.486759	-1.082363912	0.016272	0.335360913	0.580809	-0.310301341	0.60704	-0.65177953	NA	C3	-	-	-	Y	-	-	-
ENSG00000271270	TMCC1-AS1	0.246401282	0.063204	0.325367572	0.063085	0.700432325	4.51E-07	0.08534699	0.638123	0.469276579	0.000246	0.377632976	0.060047	C4	-	-	-	Y	-	Y	-
ENSG00000271524	BNIP3P17	-0.263000622	0.353202	-0.958222654	0.001776	-0.825488574	0.004982	-0.690692966	0.031318	-0.548057376	0.088256	0.139162997	NA	C5	-	-	Y	Y	Y	-	-
ENSG00000271550	BNIP3P11	-1.042400735	0.014182	-0.280633903	0.606912	-0.747435178	0.095754	0.766692159	0.121698	0.309944579	0.622782	-0.462912742	NA	C3	-	Y	-	-	-	-	-
ENSG00000271614	ATP2B1-AS1	-1.090867068	0.001164	-0.275346682	0.47833	-0.288710215	0.406946	0.825836802	0.032154	0.816649713	0.029502	-0.010887777	NA	C3	-	Y	-	-	Y	Y	-
ENSG00000271824	SMIM32	2.351108359	0.000904	1.351121479	NA	1.435821156	NA	-0.993504446	0.132885	-0.900183141	0.165674	0.088870481	NA	C5	-	Y	-	-	-	-	-
ENSG00000272031	ANKRD34A	0.226947958	0.395232	0.57167435	0.010025	0.782591704	3.90E-05	0.351357011	0.125961	0.570071671	0.003943	0.214117144	0.470096	C4	-	-	Y	Y	-	Y	-
ENSG00000272047	GTF2H5	-0.362813907	0.002259	-0.021983651	0.899319	-0.044859992	0.761272	0.348279295	0.003444	0.332744423	0.004345	-0.019679889	0.939474	C4	-	Y	-	-	Y	Y	-
ENSG00000272142	LYRM4-AS1	-0.420771427	0.066763	-0.088776503	0.796389	0.132281213	0.646646	0.338053941	0.188989	0.56761089	0.012632	0.223904163	0.541178	C4	-	-	-	-	-	Y	-
ENSG00000272143	FGF14-AS2	-1.512378603	8.05E-14	-0.511488011	0.036588	-0.92537838	7.02E-06	1.010449499	0.000135	0.601654943	0.034152	-0.413603157	0.293243	C3	-	Y	Y	Y	Y	Y	-
ENSG00000272325	NUDT3	0.239984568	0.075869	0.414381418	0.001893	0.290934499	0.035056	0.180876111	0.189283	0.065544548	0.689343	-0.120314961	0.570442	C4	-	-	Y	-	-	-	-
ENSG00000272333	KMT2B	0.486526994	1.63E-15	0.180059801	0.01858	0.200856111	0.005155	-0.299932968	3.66E-08	-0.271034095	6.95E-07	0.023411848	0.870214	C5	-	Y	-	-	-	-	-
ENSG00000272391	POM121C	0.310849632	3.34E-10	0.208339291	0.00016	0.122303358	0.031853	-0.09623343	0.021501	-0.17401215	2.79E-06	-0.083070811	0.134182	C5	-	Y	-	-	-	-	-
ENSG00000272455	MRPL20-DT	0.077393216	0.788254	0.577431478	0.025135	0.159511268	0.639345	0.507047998	0.029857	0.097051526	0.758877	-0.416096951	0.25635	C4	-	-	Y	-	Y	-	-
ENSG00000272556	GTF2IP13	-0.689818587	2.03E-05	-0.317536314	0.135251	-0.224085646	0.253932	0.377825731	0.020935	0.479873414	0.000711	0.097268305	0.749431	C4	-	Y	-	-	Y	Y	-
ENSG00000272645	GTF2IP20	-0.222402814	0.096169	-0.383267164	0.037082	0.120020189	0.44039	-0.154708552	0.403091	0.356858082	0.001381	0.506892309	0.004047	C4	-	-	Y	-	-	Y	Y
ENSG00000272686	WASL-DT	-0.350222693	0.001043	0.062021561	0.726244	-0.235790969	0.083619	0.419131344	0.000338	0.129237906	0.385206	-0.295689555	0.086625	C4	-	Y	-	-	Y	-	-
ENSG00000272690	LINC02018	-1.585663826	0.002333	-0.200416325	0.768372	0.026458811	0.969873	1.396150488	0.018882	1.626691931	0.003289	0.229036398	NA	C3	-	Y	-	-	Y	Y	-
ENSG00000272695	GAS6-DT	-0.630012957	0.14384	0.359686641	0.562049	0.309051953	0.548993	0.99809328	0.046638	0.954196072	0.034812	-0.048541417	NA	C2	-	-	-	-	Y	Y	-
ENSG00000272870	SAP30-DT	-0.272711893	0.040794	0.069495213	0.741084	0.183863183	0.290565	0.349050832	0.01484	0.47149037	0.001029	0.116099096	0.682272	C4	-	-	-	-	Y	Y	-
ENSG00000272886	DCP1A	0.49771304	2.27E-09	0.064588257	0.637662	0.17617193	0.082035	-0.42659943	3.96E-12	-0.307034854	5.19E-07	0.114098877	0.248773	C5	-	Y	-	-	Y	Y	-
ENSG00000272888	LINC01578	-0.431335086	1.47E-13	-0.155742603	0.031694	-0.429654686	1.04E-11	0.28202173	2.15E-06	0.016505376	0.861864	-0.270991892	0.000127	C4	-	Y	-	Y	-	-	-
ENSG00000272899	ATP6V1FNB	1.332622864	3.74E-07	0.650929043	0.091231	0.624358903	0.089378	-0.675210681	0.009644	-0.692772263	0.006926	-0.023664439	NA	C1	-	Y	-	-	Y	Y	-
ENSG00000272902	TBC1D8-AS1	-0.872283997	0.009113	-0.316544537	0.446333	-0.43134351	0.233382	0.564190933	0.143353	0.455357191	0.252073	-0.11229664	NA	C2	-	Y	-	-	-	-	-
ENSG00000273024	INTS4P2	-0.658257189	0.197737	0.000368157	0.999443	0.45604717	0.336486	0.661580382	0.213217	1.128331718	0.009698	0.456964572	NA	C2	-	-	-	-	-	Y	-
ENSG00000273032	DGCR5	-0.460894062	0.000641	-0.073670039	0.712952	0.017894723	0.931707	0.393990833	0.002657	0.493838449	5.76E-05	0.09499336	0.658238	C4	-	Y	-	-	Y	Y	-
ENSG00000273142	LINC02604	-0.643158846	0.000502	-0.265453304	0.343219	-0.044106516	0.879742	0.384588807	0.111337	0.61427006	0.001117	0.223798224	0.541648	C4	-	Y	-	-	-	Y	-
ENSG00000273173	SNURF	0.848091175	0.030834	0.465557708	0.369816	0.046292162	0.947416	-0.375987855	0.388532	-0.78643687	0.051017	-0.416154059	NA	C5	-	Y	-	-	-	-	-
ENSG00000273213	H3-2	0.404439257	0.005985	0.212377996	0.256011	0.365759377	0.020247	-0.185166631	0.179847	-0.02421059	0.894661	0.155393335	0.446648	C4	-	Y	-	Y	-	-	-
ENSG00000273274	ZBTB8B	0.833528645	1.93E-10	0.400346835	0.022203	0.565576238	0.000244	-0.427261516	0.001778	-0.252693982	0.072597	0.1685038	0.511548	C1	-	Y	Y	Y	Y	-	-
ENSG00000273344	PAXIP1-AS1	-0.414823838	0.013076	0.189539333	0.367178	-0.17155354	0.429879	0.610958206	7.11E-05	0.258081923	0.164239	-0.358220068	0.092384	C4	-	Y	-	-	Y	-	-
ENSG00000273356	LINC02019	1.740240245	0.00561	0.885301524	NA	-0.19165327	NA	-0.857618969	0.113592	-1.91134533	0.004264	-1.073795194	NA	C5	-	Y	-	-	-	Y	-
ENSG00000273559	CWC25	0.283639548	0.000461	-0.090177359	0.464002	-0.073353617	0.536828	-0.367500505	4.94E-06	-0.342285215	1.35E-05	0.019909364	0.931318	C5	-	-	-	-	Y	Y	-
ENSG00000273802	H2BC8	-0.383606988	0.343568	-0.850699693	0.010353	-0.376161035	0.281734	-0.459509573	0.296614	0.02240043	0.963651	0.477363051	NA	C5	-	-	Y	-	-	-	-
ENSG00000273820	USP27X	0.693107579	6.21E-08	-0.126508096	0.61198	0.312020745	0.054855	-0.813984229	7.72E-09	-0.366363189	0.005002	0.442208494	0.025209	C5	-	Y	-	-	Y	Y	Y
ENSG00000273888	FRMD6-AS1	-1.37034793	0.002442	-0.284113259	0.624536	-0.115276513	0.857403	1.091209834	0.021176	1.269005356	0.007442	0.1720375	NA	C3	-	Y	-	-	Y	Y	-
ENSG00000274070	CASTOR2	0.599043261	5.52E-12	0.13362686	0.280887	0.32414687	0.000945	-0.459278045	1.18E-09	-0.260171813	0.000915	0.193587859	0.076902	C5	-	Y	-	Y	Y	-	-
ENSG00000274211	SOCS7	0.607549531	3.97E-24	0.327097257	7.25E-06	0.314505556	1.71E-06	-0.273951236	1.37E-07	-0.278379073	2.18E-09	-0.009792802	0.948042	C1	-	Y	Y	Y	-	-	-
ENSG00000274349	ZNF658	0.17855698	0.340285	-0.241541234	0.287586	0.189626389	0.364748	-0.413910085	0.023732	0.025795175	0.91563	0.434062025	0.057727	C5	-	-	-	-	Y	-	-
ENSG00000274419	TBC1D3D	0.681771574	0.00892	0.437276247	0.183604	0.408304648	0.313853	-0.239201229	0.361924	-0.258549691	0.426619	-0.02653645	0.974427	C1	-	Y	-	-	-	-	-
ENSG00000274487	NPEPPSP1	0.433131552	0.033599	0.100548064	0.769297	0.185144306	0.505663	-0.325468182	0.171045	-0.233383243	0.337751	0.086828433	0.870116	C5	-	Y	-	-	-	-	-
ENSG00000274588	DGKK	1.201270749	2.68E-07	0.14031361	0.780069	0.505159598	0.143119	-1.051268546	4.35E-07	-0.682050867	0.001151	0.36595784	0.391494	C5	-	Y	-	-	Y	Y	-
ENSG00000274750	H3C6	-0.683848652	0.022777	-0.65938922	0.049701	-0.518831388	0.113001	0.032163051	0.949511	0.180398672	0.664862	0.143644485	NA	C3	-	Y	Y	-	-	-	-
ENSG00000275004	ZNF280B	0.238249353	0.146137	-0.134590362	0.57972	-0.040945559	0.883564	-0.366393373	0.014395	-0.264924496	0.103987	0.096282574	0.779741	C5	-	-	-	-	Y	-	-
ENSG00000275052	PPP4R3B	0.279786385	1.31E-07	-0.028197619	0.79392	0.036321983	0.664167	-0.30130754	1.71E-09	-0.228963791	1.29E-07	0.067106705	0.484596	C5	-	-	-	-	Y	-	-
ENSG00000275074	NUDT18	-0.510464412	0.148706	0.152069323	0.736531	0.354460946	0.402991	0.669367526	0.028549	0.881207007	0.008322	0.206076156	0.724779	C4	-	-	-	-	Y	Y	-
ENSG00000275111	ZNF2	0.612020514	4.11E-06	-0.436476563	0.017628	0.122309056	0.566017	-1.042200786	1.73E-13	-0.474977595	0.000695	0.561633264	0.004675	C5	-	Y	Y	-	Y	Y	Y
ENSG00000275328	SPDYE19P	-0.391632769	0.771677	1.321137355	NA	1.102439076	NA	1.714564007	0.028516	1.506254772	0.085922	-0.213207396	NA	C2	-	-	-	-	Y	-	-
ENSG00000275395	FCGBP	-2.020814945	0.017209	-1.030961882	NA	-1.051126748	0.227246	0.988343143	0.425033	0.980964464	0.439159	-0.014839516	NA	C3	-	Y	-	-	-	-	-
ENSG00000275591	XKR5	-0.141548616	0.330902	0.164279828	0.304836	0.025246279	0.902366	0.312260363	0.014786	0.181372967	0.202415	-0.136164894	0.515302	C4	-	-	-	-	Y	-	-
ENSG00000275763	ZNF516-DT	-1.522484113	0.016502	-0.33763269	0.688269	0.279967108	0.682382	1.186466396	0.118983	1.817786413	0.002825	0.619516381	NA	C2	-	Y	-	-	-	Y	-
ENSG00000275835	TUBGCP5	-0.415818064	8.54E-05	-0.10437018	0.383294	-0.306644506	0.002272	0.317416549	0.002652	0.123698038	0.338427	-0.199362886	0.123495	C4	-	Y	-	Y	Y	-	-
ENSG00000275895	U2AF1L5	0.097656918	0.667377	0.228388477	0.333654	-0.292866855	0.241019	0.137664801	0.483046	-0.375156613	0.054779	-0.51809096	0.02515	C2	-	-	-	-	-	-	Y
ENSG00000276043	UHRF1	-0.343398633	2.06E-11	-0.36280431	0.000313	-0.493334566	2.55E-17	-0.012905522	0.918082	-0.135513539	0.013762	-0.127509127	0.405479	C4	-	Y	Y	Y	-	-	-
ENSG00000276141	WHAMMP3	0.882550557	0.272646	-0.63384774	NA	-1.439203038	NA	-1.512800683	0.095333	-2.309800281	0.019075	-0.804168086	NA	C5	-	-	-	-	-	Y	-
ENSG00000276234	TADA2A	-0.457413595	3.55E-08	-0.392201647	2.94E-05	-0.265052355	0.005314	0.071909647	0.561466	0.207106126	0.038151	0.12991537	0.413014	C4	-	Y	Y	-	-	-	-
ENSG00000276550	HERC2P2	0.449724521	1.02E-11	0.142600742	0.196882	0.494098675	1.26E-09	-0.300215623	8.19E-07	0.058624343	0.416199	0.354081916	6.79E-05	C1	-	Y	-	Y	Y	-	Y

ENSG00000276644	DACH1	-1.008137702	0.021427	0.022665325	0.970129	0.01853122	0.976852	1.039973132	0.017058	1.039846987	0.029774	-0.002846482	NA	C2	-	Y	-	-	Y	Y	-
ENSG00000276710	CSPG4P10	0.369967996	0.00055	0.200818158	0.098282	0.295871512	0.007009	-0.162310502	0.127147	-0.059179439	0.638575	0.097678604	0.534979	C5	-	Y	-	-	-	-	-
ENSG00000277072	STAG3L2	0.09386638	0.51306	0.506431538	1.28E-05	0.31633606	0.011698	0.41871226	3.50E-05	0.23712064	0.034692	-0.18714748	0.217098	C1	-	-	Y	Y	Y	-	-
ENSG00000277149	TYW1B	0.649440528	0.009304	0.26007225	0.460458	0.270092553	0.448148	-0.383055467	0.147331	-0.364456746	0.198553	0.012972678	0.985912	C5	-	Y	-	-	-	-	-
ENSG00000277156	TOMM40P1	1.451595165	4.94E-11	0.616022877	0.046468	0.158623591	0.705073	-0.828341173	9.37E-05	-1.278106263	4.59E-09	-0.454779946	NA	C5	-	Y	Y	-	Y	Y	-
ENSG00000277161	PIGW	-0.430864098	1.31E-06	-0.171211211	0.095497	-0.416595898	2.67E-06	0.266282976	0.003419	0.028929511	0.827863	-0.242456453	0.024557	C4	-	Y	-	Y	-	-	-
ENSG00000277363	SRCIN1	-0.473522041	4.83E-07	-0.080352054	0.592763	0.138939487	0.249641	0.399444902	7.70E-05	0.626973532	1.82E-11	0.222101288	0.112713	C4	-	Y	-	-	Y	Y	-
ENSG00000277443	MARCKS	-0.32924699	4.77E-17	-0.174122625	0.001485	-0.222473999	1.79E-06	0.16183338	7.48E-06	0.121648972	0.000132	-0.045523737	0.528999	C4	-	Y	-	-	-	-	-
ENSG00000277449	CEBPB-AS1	-2.006856679	0.000857	0.092285176	0.899171	-0.169211551	0.808284	2.106332161	0.00041	1.854096255	0.004215	-0.258611018	NA	C3	-	Y	-	-	Y	Y	-
ENSG00000277462	ZNF670	0.170817115	0.115486	-0.299040535	0.007786	-0.050491215	0.749341	-0.463478283	5.31E-07	-0.206549022	0.0456	0.251508038	0.055235	C4	-	-	-	-	Y	-	-
ENSG00000277531	PNMA8C	0.729398154	4.78E-07	0.254560781	0.219954	0.580284506	0.000227	-0.46807256	4.48E-05	-0.135403957	0.284774	0.327387714	0.031665	C1	-	Y	-	Y	Y	-	Y
ENSG00000277586	NEFL	-0.643821864	6.93E-55	-0.263821984	1.89E-08	-0.100375512	0.045714	0.386540984	2.01E-20	0.558124545	1.41E-48	0.166208549	0.001012	C4	-	Y	-	-	Y	Y	-
ENSG00000277893	SRD5A2	4.23354199	0.028216	0.749891191	NA	1.424557642	NA	-3.47685395	NA	-2.786525606	NA	0.678696655	NA	C5	-	Y	-	-	-	-	-
ENSG00000278023	RDM1	-0.692434175	0.039133	-0.417927551	0.374427	-0.197494401	0.637751	0.280611612	0.582746	0.509522849	0.18741	0.223086672	NA	C4	-	Y	-	-	-	-	-
ENSG00000278175	GLIDR	-0.749883861	3.83E-09	-0.211994471	0.118712	-0.254057731	0.050301	0.544874443	7.33E-05	0.510346848	0.000274	-0.039322749	0.896892	C4	-	Y	-	-	Y	Y	-
ENSG00000278224	PRICKLE4	0.130018597	0.892286	1.327135493	0.032623	1.11301717	0.089924	1.202551266	0.02534	0.997319223	0.090614	-0.209824121	NA	C2	-	-	Y	-	Y	-	-
ENSG00000278338	VWA8-AS1	-2.399412538	0.035148	-0.923747202	NA	-0.741156892	NA	1.509048472	NA	1.669635693	0.314097	0.163852692	NA	C3	-	Y	-	-	-	-	-
ENSG00000278535	DHRS11	-0.352017515	0.009514	0.117667211	0.499263	0.062320505	0.717032	0.476914041	0.000701	0.429004406	0.001555	-0.052480533	0.854444	C2	-	Y	-	-	Y	Y	-
ENSG00000278540	ACACA	-0.089859864	0.279359	0.174151119	0.046686	0.293941291	5.17E-05	0.270717441	2.00E-07	0.398137392	7.65E-18	0.122096825	0.094565	C4	-	-	-	-	-	Y	-
ENSG00000278616	BEND3P3	0.910854719	0.003926	0.619119457	0.139075	0.563035007	0.145224	-0.284857913	0.462024	-0.332978717	0.349336	-0.053641814	NA	C1	-	Y	-	-	-	-	-
ENSG00000278709	NKILA	-0.374072246	0.164199	0.127990763	0.727231	0.341885647	0.233697	0.508612986	0.05465	0.730549377	0.00368	0.216525649	0.619543	C4	-	-	-	-	-	Y	-
ENSG00000278768	BACE1-AS	-0.192049489	0.204043	-0.322219891	0.029794	-0.200336174	0.230775	-0.123911886	0.453266	0.006600198	0.972988	0.124827492	0.613838	C4	-	-	Y	-	-	-	-
ENSG00000279730	KMT5AP1	0.767496705	0.193493	-0.411551028	NA	-0.61989064	0.513384	-1.173497388	0.049646	-1.369318035	0.027742	-0.205484797	NA	C5	-	-	-	-	Y	Y	-
ENSG00000280213	UCKL1-AS1	0.741457897	0.057518	0.47203762	0.369761	0.976746416	0.010865	-0.263616965	0.552469	0.250566168	0.527325	0.509947173	NA	C1	-	-	-	Y	-	-	-
ENSG00000280303	ERICD	-0.622004282	0.076098	-0.133654509	0.797931	0.229961239	0.580032	0.495335138	0.190984	0.867352855	0.007813	0.366908728	NA	C4	-	-	-	-	-	Y	-
ENSG00000280594	BTG3-AS1	0.640130903	0.023868	-0.175750473	0.727395	0.021696359	0.967124	-0.80950158	0.011229	-0.603943188	0.051571	0.200715524	NA	C5	-	Y	-	-	Y	-	-
ENSG00000280670	CCDC163	0.669931985	0.028735	0.16002414	0.753436	0.308254456	0.437438	-0.503730145	0.125943	-0.347524073	0.296606	0.151107988	NA	C5	-	Y	-	-	-	-	-
ENSG00000280693	SH3PXD2A-AS1	3.244414505	0.04603	3.256754164	NA	2.58763318	NA	0.017801902	0.988864	-0.645806424	0.618932	-0.67047816	NA	C1	-	Y	-	-	-	-	-
ENSG00000280734	LINC01232	-0.740938978	0.118863	-0.0127049	0.987931	0.323652361	0.525157	0.736651485	0.171873	1.079506377	0.015509	0.340475055	NA	C4	-	-	-	-	-	Y	-
ENSG00000280832	GSEC	0.205148466	0.40303	0.510302685	0.030523	0.231682901	0.455412	0.311676397	0.163861	0.040711195	0.903419	-0.276340716	0.46204	C1	-	-	Y	-	-	-	-
ENSG00000280916	FOXCUT	-1.493322359	2.25E-05	-0.136713847	0.774441	-0.192553588	0.674147	1.363332368	0.000266	1.315820379	0.000991	-0.05287867	NA	C2	-	Y	-	-	Y	Y	-
ENSG00000280987	MATR3	0.874564072	0.024664	0.729610899	0.097758	0.648794857	0.181672	-0.139112453	0.744746	-0.212093924	0.634678	-0.077871677	NA	C1	-	Y	-	-	-	-	-
ENSG00000281183	NPTN-IT1	0.31910853	0.217056	0.623503452	0.017733	0.52011922	0.041231	0.310354874	0.217025	0.215779487	0.406336	-0.100582732	0.839995	C1	-	-	Y	Y	-	-	-
ENSG00000281207	SLFNL1-AS1	-0.509818588	0.138882	0.281305746	0.473191	-0.029573789	0.949319	0.796056606	0.019508	0.494793785	0.172227	-0.308010239	NA	C2	-	-	-	-	Y	-	-
ENSG00000281358	RASSF1-AS1	-2.914450626	0.012795	-0.859452372	NA	-2.192541234	NA	2.072106402	NA	0.745418132	NA	-1.338505502	NA	C3	-	Y	-	-	-	-	-
ENSG00000281398	SNHG4	-0.012435227	0.939139	0.485497937	2.89E-05	0.074011071	0.592108	0.504305592	1.58E-06	0.10124456	0.385813	-0.408561221	0.000842	C4	-	-	Y	-	Y	-	Y
ENSG00000281490	CICP14	0.30802627	0.001835	0.186862665	0.109208	0.242782705	0.014426	-0.114630087	0.314194	-0.050684336	0.675007	0.059003288	0.746983	C5	-	Y	-	-	-	-	-
ENSG00000281649	EBLN3P	0.399320468	4.37E-11	0.145800037	0.074864	0.246260381	0.001393	-0.24710251	9.38E-06	-0.138156884	0.028727	0.103410111	0.326733	C5	-	Y	-	-	-	-	-
ENSG00000282815	TEX13C	0.058613937	0.899295	-0.165007736	0.74858	-0.722805218	0.065268	-0.216607195	0.615447	-0.766217168	0.043071	-0.554437288	NA	C5	-	-	-	-	-	Y	-
ENSG00000282995	FRG1EP	0.335637569	0.040123	0.18337485	0.40513	0.273576473	0.165085	-0.145979893	0.437575	-0.047137227	0.835248	0.093195934	0.784312	C5	-	Y	-	-	-	-	-
ENSG00000283023	FRG1GP	0.732390105	0.000301	0.398731039	0.103763	0.495755563	0.01056	-0.327263341	0.124813	-0.22231835	0.261681	0.102089607	0.776003	C1	-	Y	-	Y	-	-	-
ENSG00000283503	LINC01902	-1.585815767	0.035213	0.209390986	0.814161	0.669393776	0.284282	1.801780082	0.006442	2.270718711	0.00013	0.463462576	NA	C2	-	Y	-	-	Y	Y	-
ENSG00000283929	MIR5010	1.300945933	0.08927	-0.738374069	NA	-0.201322427	NA	-2.05171624	0.024457	-1.486644146	0.08087	0.532740218	NA	C5	-	-	-	-	Y	-	-
ENSG00000283971	MIR4442	0.408044937	0.010087	0.156424987	0.496231	0.147605405	0.51425	-0.245671268	0.11582	-0.24581054	0.127156	-0.005642892	0.990051	C5	-	Y	-	-	-	-	-
ENSG00000284024	HSPA14	0.211699909	0.034806	-0.256686233	0.020165	-0.038522327	0.815718	-0.462224018	3.80E-09	-0.236244609	0.008746	0.220806183	0.085494	C5	-	-	-	-	Y	-	-
ENSG00000284237	LINC02767	-2.383937243	0.000898	-0.934714381	0.177362	-1.598742755	0.012681	1.441099515	0.160507	0.796916509	0.518843	-0.654567951	NA	C3	-	Y	-	Y	-	-	-
ENSG00000284308	C2orf81	-0.848273636	3.08E-05	-0.29945918	0.257611	-0.42911142	0.06465	0.555411138	0.009065	0.43390902	0.050731	-0.126500211	0.757257	C4	-	Y	-	-	Y	-	-
ENSG00000285219	HULC	-1.100288218	0.017254	-0.655994613	0.192199	-0.165809888	0.761143	0.446632908	0.484495	0.949274923	0.054145	0.493150503	NA	C2	-	Y	-	-	-	-	-
ENSG00000285230	RALY-AS1	-0.991373555	1.89E-07	-0.246740384	0.312628	-0.292270185	0.215176	0.751179699	0.000804	0.714101297	0.002288	-0.04217231	0.943025	C3	-	Y	-	-	Y	Y	-
ENSG00000285410	GABPB1-IT1	-0.317071994	4.12E-06	0.144847228	0.187834	-0.007475295	0.953102	0.468200917	1.05E-08	0.324331287	6.07E-06	-0.149612133	0.259765	C4	-	Y	-	-	Y	Y	-
ENSG00000285437	POLR2J3	-0.40459724	0.222647	0.371111294	0.280905	0.34002552	0.354548	0.781204772	0.009671	0.75921252	0.01952	-0.027316029	NA	C4	-	-	-	-	Y	Y	-
ENSG00000285533	RELA-DT	-0.159876879	0.525525	0.288980328	0.256011	-0.093770097	0.788438	0.455632329	0.030945	0.081127626	0.801044	-0.380219793	0.239664	C2	-	-	-	-	Y	-	-
ENSG00000285825	CCDC15-DT	-0.809246125	0.03776	-0.54890617	0.184889	-0.637859114	0.088824	0.26870595	0.608196	0.186294258	0.733849	-0.086079866	NA	C3	-	Y	-	-	-	-	-
ENSG00000286532	PARTICL	-0.938848046	0.003013	-0.297826223	0.430047	-0.502008067	0.114165	0.648599153	0.085114	0.451070041	0.264401	-0.201646263	NA	C3	-	Y	-	-	-	-	-
ENSG00000286733	LINC02348	-2.312338193	0.001643	-1.258062447	NA	-1.221343952	0.073955	1.057389638	0.331217	1.1049688	0.299547	0.040063592	NA	C3	-	Y	-	-	-	-	-
ENSG00000287151	C2orf27A	0.382601741	0.023162	0.274247453	0.208838	0.398399832	0.034873	-0.102574763	0.59493	0.030479659	0.890282	0.12723721	0.673664	C1	-	Y	-	Y	-	-	-
ENSG00000288596	C8orf44	-0.139412133	0.509143	0.375837459	0.106994	0.456543861	0.008841	0.521954764	0.005351	0.6105171	6.12E-05	0.083795762	0.832266	C4	-	-	-	Y	Y	Y	-

Supplementary Table 2. The list of DNB genes and their neighboring genes.

DNBs	neighboring genes
ABCA3	TTF1
ABCA3	ESR1
ABCC1	LMBRD1
ABCC1	UBC
ABCC1	TCN2
ABCC1	TMOD2
ABCC1	LTC4S
ABCD4	ATP1A1
ABCD4	ATP1A3
ABCD4	ABCB6
ABCD4	ABCA2
ABCF2	ABCB8
ABCF2	UBC
ABCF2	ABCB6
ABHD5	PLIN5
ABHD5	PLIN1
ABHD5	PRKACA
ABHD5	PRKACB
ABHD5	PNPLA2
ABI2	ABL1
ABI2	EPS8
ABI2	ARPC2
ABI2	ARPC3
ABI2	TRIM32
ABI2	ACTR2
ABI2	ACTR3
ABI2	ARPC1B
ABI2	RAC1
ABI2	NCKAP1
ABI2	RAC2
ABI2	BRK1
ABI2	ARPC5
ABI2	CYFIP2
ABI2	UBC
ABI2	WASF2
ABI2	WASF1
ABI2	SRA1
ABI2	RAC3
ACTR6	ZNHIT1
ACTR6	INO80
ACTR6	RUVBL1
ACTR6	RUVBL2
ACTR6	VPS72

ACTR6	YEATS4
ADAMTS14	PCOLCE
ADAMTS14	BMP1
ADAMTS14	PCOLCE2
AGFG1	EPS15
AGFG1	VAMP7
AGFG2	EPS15L1
AGFG2	EPS15
AGPAT5	GPAT4
AGPAT5	LPIN1
AGPAT5	GPAM
AGPAT5	SLC26A1
AGPAT5	PLA2G4B
AGPAT5	LPIN2
AHCYL2	DNMT1
AHCYL2	MAT2A
AHCYL2	CBS
AHCYL2	DNMT3B
AHCYL2	DNMT3A
AHCYL2	BHMT
AHCYL2	MTR
AHI1	NPHP1
AHI1	LCA10
AHI1	TMEM216
AHI1	HAP1
AHI1	TMEM67
AHI1	CC2D2A
AIMP2	TP53
AIMP2	KARS1
AIMP2	FUBP1
AIMP2	EEF1E1
AIMP2	UBA1
AIMP2	LARS1
AIMP2	PRKN
AIMP2	UBC
AIMP2	AIMP1
AIMP2	IARS1
AKAP8L	TMPO
AKAP8L	DHX9
ANKRD27	RAB38
ANKRD27	RAB21
ANKRD27	RAB32
ANKRD42	PSMD4
ANKRD42	RPN2
ANKRD42	PSMC3
ANKRD42	PSMC6

ANKRD42	PAAF1
ANKRD42	PSMD3
ANKRD42	PSMC4
ANP32A	HAP1
ANP32A	SET
ANP32A	TNFSF13
ANP32A	NUP214
ANP32A	PPP2CB
ANP32A	ATXN1
ANP32A	HMGB2
ANP32A	ESR1
ANP32A	ELAVL1
ANP32A	EMB
ANP32A	NME1
APH1A	PSEN1
APH1A	NCSTN
APH1A	PSENEN
APIP	ENOPH1
APIP	MRI1
AQP10	AQP6
AQP10	MIP
ARCN1	COPZ2
ARCN1	COPB1
ARCN1	COPB2
ARCN1	COPA
ARCN1	COPE
ARCN1	UBC
ARCN1	COPZ1
ARCN1	COPG1
ATF7IP	PCM1
ATF7IP	SP1
ATF7IP	SETDB1
ATF7IP	GTF2E2
ATG14	BECN1
ATG14	PIK3C3
ATG14	PIK3R4
ATG14	NRBF2
ATP1B3	ATP1A3
ATP1B3	ATP1B2
ATP1B3	UBC
ATP1B3	FXYD6
ATP1B3	ATP1B1
ATP1B3	ATP1A1
ATP1B3	FXYD1
ATP1B3	FXYD7
ATP1B3	FXYD2

ATP8B2	TMEM30A
ATP8B2	TMEM30B
AUH	HMGCL
AUH	MCCC1
AUH	HMGCS1
BACE1	PSEN1
BACE1	ITM2B
BACE1	GGA1
BACE1	APP
BACE1	RTN3
BACE1	NCSTN
BACE1	RANBP9
BACE1	LRP1
BACE1	PLSCR1
BACE1	SORL1
BACE1	FURIN
BANF1	ACTL6A
BANF1	SMARCA4
BANF1	VRK3
BANF1	PPP2CB
BANF1	ANKLE2
BANF1	PPP2R2D
BANF1	PPP2R1A
BANF1	VRK1
BANF1	VRK2
BANF1	EMD
BCAT1	MYC
BCAT1	BCKDHB
BCAT1	TMEM91
BCAT1	MAX
BCL2L2	BIK
BCL2L2	AKT1
BCL2L2	BAX
BCL2L2	BID
BCL2L2	BAD
BCL2L2	MCL1
BCL2L2	BCL2L11
BEX2	NGF
BEX2	NGFR
BEX2	RIPK2
BEX2	TRAF6
BEX3	NGFR
BEX3	CASP2
BEX3	YWHAE
BEX3	NGF
BEX3	TSC1

BEX3	SORT1
BEX3	CASP3
BEX3	DIABLO
BICD2	DYNC1H1
BICD2	RAB6A
BICD2	NEK8
BICD2	EXD1
BNIP3L	TP53
BNIP3L	STEAP3
BNIP3L	TMEM11
BNIP3L	RHEB
BNIP3L	GABARAP
BNIP3L	BNIP3
BNIP3L	EP300
BNIP3L	CREBBP
BNIP3L	BCL2
BRK1	WASF2
BRK1	ACTG1
BRK1	NCKAP1
BRK1	ELMO2
BRK1	ACTR2
BRK1	ABL1
BRK1	BAIAP2
BRK1	WASF1
BRK1	MAPK3
BRK1	ARPC3
BRK1	ABI1
BRK1	SRA1
BRK1	MAPK1
BRK1	ELMO1
BRK1	ARPC5
BRK1	ARPC1B
BRK1	CRK
BRK1	SYK
BRK1	ARPC2
BRK1	RAC1
BRK1	ABI2
BRK1	VAV3
BRK1	VAV2
BRK1	ACTR3
BRK1	CYFIP2
BRK1	DOCK1
BRK1	ARPC1A
BTAF1	DHODH
BTAF1	TBP
BTAF1	DRAP1

BTAF1	DR1
BTAF1	ISYNA1
BTF3	NACA
BTF3	UBC
BTF3	NACAD
BTF3	ESR1
C16orf70	KIF17
C16orf70	CASK
C2orf49	RTCB
C2orf49	FAM98B
C2orf49	DDX1
CACNG4	CAMK2G
CACNG4	CAMK2D
CACNG4	CAMK2A
CACNG4	EPB41L1
CACNG4	MDM2
CACNG4	CACNG2
CACNG4	DLG4
CACNG4	AKAP5
CACNG4	MYO6
CACNG4	DLG1
CACNG4	CACNG8
CACUL1	SKP1
CACUL1	CDK2
CALU	SARS2
CALU	AURKA
CAP1	PFN1
CAP1	CFL1
CAP1	ROBO1
CAP1	PPIA
CAP1	ABL1
CAP1	DSTN
CAP1	ABL2
CAP1	CAP2
CAP1	GPC1
CAP1	ADCY1
CAP1	SLIT2
CAP1	CFL2
CAPZA1	UBC
CAPZA1	WASH6P
CAPZA1	CARMIL1
CAPZA1	CD2AP
CAPZA1	APP
CAPZA1	CAPZA2
CAPZA1	CAPZB
CAPZA1	S100B

CAPZA1	MAGOH
CAPZA1	AGER
CAPZA1	HMGB1P1
CASC3	RPL37A
CASC3	RPS3A
CASC3	RPL30
CASC3	RPS15A
CASC3	RPL18A
CASC3	RPL8
CASC3	RPS16
CASC3	RPL10A
CASC3	RBM8A
CASC3	SMG5
CASC3	RPL9P7
CASC3	RPL12
CASC3	RPL28
CASC3	MAGOH
CASC3	RPL37
CASC3	RPS6
CASC3	RPL27A
CASC3	RPS15
CASC3	RPL14
CASC3	RPL23A
CASC3	RPS4X
CASC3	RPL3L
CASC3	RPL13A
CASC3	EIF4G1
CASC3	RPL26
CASC3	SMG6
CASC3	UPF1
CASC3	RPL21
CASC3	ETF1
CASC3	RPS24
CASC3	RPL34
CASC3	PPP2CB
CASC3	UPF3B
CASC3	RPS26
CASC3	RPS19
CASC3	RPL41
CASC3	SMG7
CASC3	RPL4
CASC3	RPL17
CASC3	RPL23
CASC3	UPF2
CASC3	NCBP2
CASC3	RPL39

CASC3	RPS13
CASC3	RPL38
CASC3	RPS14
CASC3	RPL11
CASC3	PPP2R2D
CASC3	RPS27
CASC3	SMG9
CASC3	RPS12
CASC3	PPP2R1A
CASC3	RPS10-NUDT3
CASC3	RPL7
CASC3	RLIM
CASC3	RPL15
CASC3	RPS3
CASC3	STAU1
CASC3	RPS2
CASC3	UBC
CASC3	RPL13
CASC3	RPS8
CASC3	RPL5
CASC3	RPL3
CASC3	RPS29
CASC3	RPSA
CASC3	RPL26L1
CASC3	RPL18
CASC3	RPLP0P6
CASC3	RRP1
CASC3	SMG1
CASC3	FAU
CASC3	RPS21
CASC3	RPL6
CASC3	RPL35
CASC3	RPL36
CASC3	EIF4A3
CASC3	RPS23
CASC3	RPL29
CASC3	RPL24
CASC3	RPL19
CASC3	SMG8
CASC3	RPLP2
CASC3	RPL22
CASC3	NCBP1
CASC3	RPS11
CASC3	RPL35A
CASC3	RPL32
CASC3	UPF3A

CASC3	RPL31
CASC3	RPS5
CASC3	RPS7
CASC3	RPL27
CASC3	RNPS1
CASC3	RPS28
CASC3	RPS20
CCDC6	RET
CCDC6	PPP4C
CCDC6	CREB1
CCND1	TLE5
CCND1	PSMD12
CCND1	TRAM1
CCND1	ILK
CCND1	CTBP1
CCND1	TAF1
CCND1	TSC2
CCND1	EIF2AK3
CCND1	AGAP2
CCND1	PIK3R1
CCND1	HDAC3
CCND1	SRC
CCND1	HDAC9
CCND1	PSME3
CCND1	CDKN2B
CCND1	RAF1
CCND1	MAPK8
CCND1	HK3
CCND1	FOS
CCND1	TP53
CCND1	CDH2
CCND1	RB1
CCND1	STAT1
CCND1	ZNF225
CCND1	PSMA5
CCND1	PSMD5
CCND1	RPN1
CCND1	PIK3CA
CCND1	MYC
CCND1	MAPK14
CCND1	PSMB2
CCND1	BIRC5
CCND1	CCNH
CCND1	FBXO4
CCND1	TLE1
CCND1	RAC1

CCND1	EP300
CCND1	CREBBP
CCND1	GADD45A
CCND1	CDKN2C
CCND1	RBPJ
CCND1	E2F4
CCND1	HDAC1
CCND1	PSMC3
CCND1	SKP1
CCND1	ERBB3
CCND1	HNF1A
CCND1	ATM
CCND1	PSMD13
CCND1	STAT3
CCND1	MYB
CCND1	PSMB3
CCND1	PSMB7
CCND1	CDKN1A
CCND1	FGFR1
CCND1	PSMA4
CCND1	PSMA6
CCND1	ELAVL1
CCND1	SMAD4
CCND1	PSMB1
CCND1	RBL1
CCND1	E2F2
CCND1	STAT5B
CCND1	KAT2B
CCND1	CDKN2D
CCND1	RPRD1B
CCND1	MAPK12
CCND1	PSMD10
CCND1	EIF4E
CCND1	CDC25A
CCND1	PSMF1
CCND1	STAT5A
CCND1	CREB1
CCND1	CDC42
CCND1	HSPA8
CCND1	LEF1
CCND1	TERT
CCND1	CUL1
CCND1	CTNNB1
CCND1	CDK14
CCND1	ZBTB17
CCND1	NME2

CCND1	RELA
CCND1	PSME1
CCND1	E2F5
CCND1	PSMD7
CCND1	CDK2
CCND1	FOXO1
CCND1	PSMC2
CCND1	NOTCH1
CCND1	PSMC5
CCND1	MNAT1
CCND1	E2F1
CCND1	MTOR
CCND1	WNT5A
CCND1	MAPK1
CCND1	MAML1
CCND1	HBP1
CCND1	CDK4
CCND1	TCF7L2
CCND1	BCL2
CCND1	PSMA1
CCND1	PSMB4
CCND1	JUN
CCND1	EMB
CCND1	PSMC4
CCND1	PSMB5
CCND1	ATF2
CCND1	BRCA1
CCND1	CDKN2A
CCND1	CCNE1
CCND1	HGF
CCND1	RBL2
CCND1	CHUK
CCND1	PSMD11
CCND1	NRAS
CCND1	PSMB10
CCND1	MYBL2
CCND1	TFDP2
CCND1	CDK6
CCND1	PSMA7
CCND1	PSMA3
CCND1	CCNA1
CCND1	MYCN
CCND1	CCND3
CCND1	KRAS
CCND1	PSMD8
CCND1	MAML3

CCND1	PSMD6
CCND1	NFKB1
CCND1	SKP2
CCND1	EGFR
CCND1	PSME4
CCND1	MCM7
CCND1	MAML2
CCND1	ABL1
CCND1	CDK1
CCND1	CDC27
CCND1	CDK9
CCND1	TCF7L1
CCND1	CDKN1B
CCND1	TFDP1
CCND1	CDKN1C
CCND1	PSMD9
CCND1	EIF4EBP1
CCND1	CCNDBP1
CCND1	ESR1
CCND1	UBC
CCND1	CCNB1
CCND1	NMI
CCND1	BTG2
CCND1	PCNA
CCND1	FOSL1
CCND1	PSMD3
CCND1	NEUROD1
CCND1	TEP1
CCND1	ERBB2
CCND1	GSK3B
CCND1	PSMB6
CCND1	PSME2
CCND1	E2F3
CCND1	BCL6
CCND1	FGF2
CCND1	CDH1
CCND1	ETS1
CCND1	RAC3
CCND1	STK35
CCND1	PSMC1
CCND1	PSMC6
CCND1	AKT1
CCND1	PSMD14
CCND1	RPN2
CCND1	SNW1
CCND1	CDK5

CCND1	FBXO31
CCND1	PSMD4
CCNL2	CDK11B
CCNL2	DYRK1A
CCT5	TRIM28
CCT5	TUBA1C
CCT5	STRN
CCT5	TUBB6
CCT5	TCP1
CCT5	TUBB4A
CCT5	PPP2CB
CCT5	TUBB1
CCT5	PPP4C
CCT5	MKKS
CCT5	BBS10
CCT5	STRN3
CCT5	CCT6A
CCT5	CCT2
CCT5	CCT8
CCT5	PFDN5
CCT5	CCT6B
CCT5	SPHK1
CCT5	CCT4
CCT5	STK3
CCT5	TUBA1B
CCT5	CCT3
CCT5	ACTG1
CCT5	PPP2R2C
CCT5	TUBB2A
CCT5	PFDN4
CCT5	TUBA4A
CCT5	TRAF3IP3
CCT5	TUBB4B
CCT5	UBC
CCT5	TUBB2B
CCT5	MOB4
CCT5	PFDN1
CCT5	STRN4
CCT5	HSPE1
CCT5	CCT7
CCT5	PFDN2
CD151	ITGB4
CD151	LAMB3
CD151	ITGA3
CD151	LAMA3
CD151	LAMC2

CD151	IGSF8
CD151	PLEC
CD151	UBC
CD151	ITGA6
CD151	GP5
CD320	UBC
CD320	TCN2
CD46	UBC
CD46	C4A
CD63	TIMP1
CD63	SRC
CD63	UBC
CDCA7	MAX
CDCA7	MYC
CDK13	CCNL1
CDK13	POLR2A
CDK13	CDK4
CDK13	CCNK
CDK13	CDK12
CDK13	SUPT5H
CDK20	POLR2A
CDK20	POLR3A
CDK20	CCNH
CDK20	UBXN7
CDK20	MNAT1
CDK5RAP1	EXOSC3
CDK5RAP1	CDK5R1
CDK5RAP1	EXOSC5
CDV3	TSFM
CDV3	QKI
CDV3	PPP1R12A
CHCHD3	SAMM50
CHCHD3	IMMT
CHRNA3	CHRNA7
CHRNA3	CHRNB4
CHRNA3	CHRNA2
CHRNA3	CHRNB2
CIP2A	UBC
CIP2A	MYC
CIR1	HDAC1
CIR1	RBPJ
CIR1	NCOR2
CIR1	HDAC2
CKS2	CCNA2
CKS2	UBC
CKS2	SKP2

CKS2	CDKN3
CKS2	CCNB1
CKS2	CCNB2
CKS2	CDK2
CKS2	CCNA1
CKS2	CDK1
CLDN12	CLDN20
CLDN12	CLDN2
CLDN12	CLDN18
CLDN12	CLDN15
CLDN12	CLDN23
CLDN12	CLDN9
CLDN15	CLDN9
CLDN15	CLDN20
CLDN15	CLDN23
CLDN15	TJP1
CLDN15	CLDN12
CLDN15	CLDN18
CNN2	FSCN1
CNN2	TPM1
CNN2	ACTA2
CNN2	UBC
CNN2	ZYX
CNTNAP2	EPB41
CNTNAP2	KCNA2
CNTNAP2	CNTN2
COA6	COX5B
COA6	COX6A1
COA6	COX6A1P2
COA6	COX7C
COA6	COX5A
COA6	GATC
COMMD10	COMMD8
COMMD10	COMMD7
COMMD8	COMMD10
COMMD8	COMMD7
COPB1	UBC
COPB1	COPG1
COPB1	COPA
COPB1	GBF1
COPB1	ARCN1
COPB1	COPB2
COPB1	ARFGAP3
COPB1	COPG2
COPB1	POLR3B
COPB1	COPZ2

COPB1	KDELR1
COPB1	ARFGAP1
COPB1	COPZ1
COPB1	ARF1
COPB1	COPE
CRK	TLN1
CRK	DOCK2
CRK	VWF
CRK	PAK2
CRK	ITGA2B
CRK	PTK2B
CRK	PIK3CB
CRK	RAP1B
CRK	CBL
CRK	SRC
CRK	DBNL
CRK	PTK2
CRK	MAP4K5
CRK	RAC3
CRK	ABI1
CRK	ANGPT1
CRK	EPHA2
CRK	ELMO3
CRK	WASF2
CRK	PDGFRA
CRK	VAV3
CRK	FRS3
CRK	ELMO2
CRK	IRS2
CRK	GRAP2
CRK	PTPN1
CRK	SYN1
CRK	BRAF
CRK	YES1
CRK	MET
CRK	STAT5A
CRK	SOS1
CRK	PRKCH
CRK	MAP3K7
CRK	HGF
CRK	PTPN11
CRK	SLK
CRK	MAP3K1
CRK	GFRA1
CRK	MAP2K4
CRK	RAPGEF1

CRK	PRKCQ
CRK	PRKCE
CRK	KHDRBS1
CRK	PRKCD
CRK	MAP4K1
CRK	ITGAV
CRK	BCR
CRK	GAB2
CRK	CRKL
CRK	DOCK3
CRK	PRKCA
CRK	BRK1
CRK	FLT1
CRK	ITGB3
CRK	INSR
CRK	VAV2
CRK	PEAK1
CRK	RET
CRK	PIK3R1
CRK	KIT
CRK	PIK3CD
CRK	RHOA
CRK	ABL1
CRK	RAC1
CRK	NCK1
CRK	TRIP6
CRK	PIK3CA
CRK	DOCK5
CRK	NEDD9
CRK	PDGFRB
CRK	BCAR1
CRK	SYK
CRK	FGFR1
CRK	SHC1
CRK	DOCK1
CRK	ATF2
CRK	LCP2
CRK	BAIAP2
CRK	FRS2
CRK	KIDINS220
CRK	MAPK8
CRK	PIK3R2
CRK	CBLB
CRK	PXN
CRK	RPS6KB1
CRK	MAPK3

CRK	YWHAB
CRK	ZAP70
CRK	PRKCG
CRK	NTRK1
CRK	NGF
CRK	EFNA5
CRK	IGF1R
CRK	EPHB3
CRK	ELMO1
CRK	GAB1
CRK	IRS1
CRK	ARHGAP32
CRK	NCKAP1
CRK	FN1
CRK	ABL2
CRK	FLT4
CRK	GRB2
CRK	VEGFD
CRK	CYFIP2
CRK	EGFR
CRTC2	SIK3
CRTC2	PRKAA2
CRTC2	YWHAZ
CRTC2	SIK1
CRTC2	YWHAE
CRTC2	YWHAG
CRTC2	YWHAQ
CRTC2	YWHAB
CRTC2	SIK2
CRTC2	CREB1
CRTC2	YWHAH
CRTC2	PRKAA1
CRTC2	SFN
CSDE1	IGF2BP1
CSDE1	STRAP
CSDE1	YBX1
CSDE1	DHX9
CSDE1	SYNCRIP
CSDE1	HNRNPU
CSDE1	CDK11B
CSDE1	MAX
CSDE1	MYC
CSDE1	RLIM
CSDE1	UBC
CTC1	TEN1
CTC1	STN1

CTC1	POT1
CTDNEP1	PPP2R2C
CTDNEP1	PPP3CB
CTDNEP1	PPP2R5C
CTDNEP1	CDK1
CTDNEP1	PPP1CC
CTDNEP1	PPP4R4
CTDNEP1	CCNB1
CTDNEP1	PPP1CA
CTDNEP1	BMPR2
CTDNEP1	CYCS
CTDNEP1	PPP2R5A
CTDNEP1	PPP2R2D
CTDNEP1	CNEP1R1
CTDNEP1	PPP1R12A
CTDNEP1	PPP3CA
CTDNEP1	PPP4C
CTDNEP1	PPP3R1
CTDNEP1	PPP2R1A
CTDNEP1	TOX4
CTDNEP1	PPP1CB
CTDNEP1	PPP2R3A
CTDNEP1	PPP4R2
CTDNEP1	WDR82
CTDNEP1	LPIN1
CTDNEP1	SHOC2
CTDNEP1	PPP2CB
CTSV	CD74
CTSV	CTSS
CTSV	IFI30
CTSV	COL18A1
CTU2	MOCS3
CTU2	URM1
CTU2	CTU1
CUEDC2	SOCS3
CUEDC2	UBC
CUEDC2	CHUK
DAPK3	MYL12B
DAPK3	ATF4
DAPK3	DAXX
DCAF1	SAMHD1
DCAF1	IL17RB
DCAF1	DDB1
DCAF12	ERCC8
DCAF12	DCAF6
DCAF12	DCAF11

DCAF12	DCAF7
DCAF12	RBX1
DCAF12	CRBN
DCAF12	DCAF4
DCAF12	PEX19
DCAF12	DCAF8
DCAF12	DCAF13
DCAF12	TRPC4AP
DCAF12	DCAF10
DCAF12	CUL4A
DCAF12	DTL
DCAF12	DCAF16
DCAF12	DDB1
DCAF12	DCAF17
DCAF12	DCAF5
DCAF12	CUL4B
DCAF12	FBXW5
DCAF7	CUL4B
DCAF7	DYRK1A
DCAF7	DDB1
DCAF7	DCAF10
DCAF7	TRPC4AP
DCAF7	DCAF6
DCAF7	CRBN
DCAF7	DCAF8
DCAF7	DCAF17
DCAF7	DCAF12
DCAF7	PEX19
DCAF7	DYRK2
DCAF7	DCAF5
DCAF7	DCAF4
DCAF7	UBC
DCAF7	CUL4A
DCAF7	DCAF11
DCAF7	HIPK2
DCAF7	DCAF16
DCAF7	DCAF13
DCAF7	FBXW5
DCAF7	DTL
DCAF7	ERCC8
DCAF7	DYRK1B
DCAF7	RBX1
DDRGK1	UFL1
DDRGK1	CDK5RAP3
DDRGK1	UFM1
DDX1	EIF2S1

DDX1	RELA
DDX1	CSTF2
DDX1	ATM
DDX1	UBC
DDX1	RTCB
DDX1	FAM98B
DDX1	C2orf49
DEK	KAT2B
DEK	UBC
DENR	MCTS1
DENR	UBC
DGUOK	PNP
DGUOK	RRM2B
DGUOK	GUK1
DGUOK	ITPA
DHFRP1	TYMS
DHFRP1	MTFMT
DHFRP1	ATIC
DHFRP1	FPGS
DHFRP1	CCNA1
DHFRP1	AMT
DHFRP1	TFDP1
DHFRP1	SPR
DHFRP1	ALDH1L1
DHFRP1	FTCD
DHFRP1	MTR
DHFRP1	SP1
DHFRP1	CDC6
DHFRP1	CCNE1
DHFRP1	RRM2
DHFRP1	ORC1
DHFRP1	MTHFD1L
DHFRP1	CDK1
DHFRP1	PCNA
DHFRP1	E2F4
DHFRP1	RBL1
DHFRP1	ALDH1L2
DHFRP1	RBL2
DHFRP1	SHMT1
DHFRP1	FBXO31
DHFRP1	POLA1
DHFRP1	SLC25A32
DHFRP1	MTHFD1
DHFRP1	UBC
DHFRP1	CDT1
DHFRP1	TFDP2

DHFRP1	CDC45
DHFRP1	SHMT2
DHFRP1	HSPD1
DHFRP1	E2F1
DHFRP1	DECR1
DHFRP1	GGH
DHX30	RAB31
DHX30	UBC
DMTF1	FOSL1
DMTF1	JUNB
DMTF1	JUN
DMTF1	FOS
DNAJC2	HSPA14
DNAJC2	RPL31
DNAJC2	HDLBP
DNAL1	DYNLL2
DNAL1	FOXJ1
DNAL1	DYNLL1
DNAL1	DNAH5
DPCD	RUVBL1
DPCD	RUVBL2
DPF2	SMARCA4
DPF2	SMARCC1
DPF2	ITGB1
DPF2	ITGA1
DR1	DRAP1
DR1	NR2F2
DR1	SGF29
DR1	KAT14
DR1	POLE3
DR1	TADA2A
DR1	KAT2B
DR1	KAT2A
DR1	MBIP
DR1	BTAF1
DR1	TBP
DR1	YEATS2
DRAP1	DR1
DRAP1	TBP
DRAP1	SMAD4
DRAP1	SMAD2
DRAP1	FOXH1
DRAP1	SMAD3
DRAP1	BTAF1
DRAP1	NFYB
DROSHA	POLR2D

DROSHA	POLR2J
DROSHA	POLR2K
DROSHA	RANP1
DROSHA	POLR2B
DROSHA	DGCR8
DROSHA	KHDRBS1
DROSHA	POLR2L
DROSHA	POLR2H
DROSHA	AGO1
DROSHA	POLR2C
DROSHA	POLR2A
DROSHA	AGO4
DROSHA	XPO5
DROSHA	POLR2G
DROSHA	DDX17
DROSHA	POLR2I
DROSHA	TP53
DROSHA	POLR2E
DROSHA	AGO2
DROSHA	POLR2F
DTL	DCAF5
DTL	TP53
DTL	ERCC8
DTL	CUL4A
DTL	DCAF13
DTL	DCAF17
DTL	UBC
DTL	CUL4B
DTL	RBX1
DTL	DCAF8
DTL	PCNA
DTL	CRBN
DTL	DDB1
DTL	KMT5A
DTL	DCAF12
DTL	DCAF10
DTL	CDT1
DTL	FBXW5
DTL	DCAF11
DTL	TRPC4AP
DTL	DCAF4
DTL	CDKN1A
DTL	DCAF7
DTL	DCAF16
DTL	PEX19
DTL	DCAF6

DTX2	NOTCH2
DTX2	NOTCH1
DTX2	NOTCH3
E2F4	HDAC1
E2F4	CDC6
E2F4	CDKN1B
E2F4	CDC25A
E2F4	CDK1
E2F4	ETV3
E2F4	CDK4
E2F4	CBX4
E2F4	SMARCA2
E2F4	CCNB1
E2F4	TFDP2
E2F4	PCNA
E2F4	MYC
E2F4	TRRAP
E2F4	CCND1
E2F4	CCNE1
E2F4	ID2
E2F4	SMAD3
E2F4	TFDP1
E2F4	DYRK1A
E2F4	RBL1
E2F4	SKP2
E2F4	LIN54
E2F4	SKP1
E2F4	SMAD4
E2F4	RBBP4
E2F4	BRCA1
E2F4	E2F5
E2F4	CEBPA
E2F4	DHFRP1
E2F4	RBL2
E2F4	LIN52
E2F4	NCOR2
E2F4	CUL1
E2F4	SIN3A
E2F4	CDKN1A
E2F4	TOP2A
E2F4	SMAD2
E2F4	MYBL2
E2F4	TP53
E2F4	RB1
E2F4	HDAC2
E2F4	EMB

E2F4	E2F1
E2F4	DDX20
E2F4	CTBP1
E2F4	LIN37
E2F4	SIN3B
E2F4	HDAC5
E2F4	TGS1
E2F4	CDK2
EAF1	ELL
EAF1	ELL3
EAF1	ELL2
ECD	RPN1
ECD	RPN2
ECD	PSMC5
ECD	PSMC3
ECSIT	TRAF6
ECSIT	MYD88
ECSIT	IRAK4
ECSIT	TRIM59
ECSIT	NDUFAF1
ECSIT	MAP3K1
ECSIT	IRAK2
ECSIT	TIRAP
EDEM3	MAN1B1
EDEM3	SEL1L
EEFSEC	PSTK
EEFSEC	SECISBP2
EEFSEC	SEPHS1
EEFSEC	TRNAU1AP
EIF2S1	POP1
EIF2S1	RPS8
EIF2S1	P4HB
EIF2S1	EIF2B4
EIF2S1	RPL31
EIF2S1	RPL3L
EIF2S1	DDX1
EIF2S1	EIF3K
EIF2S1	RPL17
EIF2S1	RPL37A
EIF2S1	RPL18
EIF2S1	EIF3B
EIF2S1	EIF3E
EIF2S1	RPL23
EIF2S1	EIF2S3
EIF2S1	RPL26
EIF2S1	EIF2AK4

EIF2S1	RPL27A
EIF2S1	RPL11
EIF2S1	EIF3F
EIF2S1	RPS10-NUDT3
EIF2S1	RLIM
EIF2S1	RPL12
EIF2S1	EIF2AK2
EIF2S1	RPL26L1
EIF2S1	RPL35A
EIF2S1	RPS29
EIF2S1	RPL15
EIF2S1	RPS19
EIF2S1	EIF4A1
EIF2S1	EIF3J
EIF2S1	RPS27
EIF2S1	EIF2S2
EIF2S1	RPL8
EIF2S1	RPS20
EIF2S1	MYC
EIF2S1	RPS5
EIF2S1	RPS7
EIF2S1	RPL22
EIF2S1	RPL23A
EIF2S1	RPL30
EIF2S1	RPL39
EIF2S1	EIF2B5
EIF2S1	EIF3D
EIF2S1	RPL14
EIF2S1	EIF3A
EIF2S1	FAU
EIF2S1	UBC
EIF2S1	RPL24
EIF2S1	RPS3
EIF2S1	RPS4X
EIF2S1	RPS12
EIF2S1	EIF2AK1
EIF2S1	RPL13
EIF2S1	RPS14
EIF2S1	EIF1AX
EIF2S1	EIF5B
EIF2S1	RPL28
EIF2S1	RPLP0P6
EIF2S1	RPS11
EIF2S1	RPS2
EIF2S1	RPL19
EIF2S1	EIF2B2

EIF2S1	RPL38
EIF2S1	EIF2AK3
EIF2S1	RPS16
EIF2S1	RPL32
EIF2S1	RPS13
EIF2S1	PPP1R15A
EIF2S1	EIF2B1
EIF2S1	EIF3H
EIF2S1	RPLP2
EIF2S1	ATF4
EIF2S1	RPS15A
EIF2S1	EIF3C
EIF2S1	RPL5
EIF2S1	RPL18A
EIF2S1	EIF4G3
EIF2S1	RPL41
EIF2S1	EIF4G1
EIF2S1	RPS21
EIF2S1	EIF4B
EIF2S1	RPL9P7
EIF2S1	RPSA
EIF2S1	MAX
EIF2S1	RPS28
EIF2S1	EIF3G
EIF2S1	EP300
EIF2S1	RPL36
EIF2S1	EIF1
EIF2S1	EIF5
EIF2S1	EIF3I
EIF2S1	CREBBP
EIF2S1	NAT1
EIF2S1	EIF4A2
EIF2S1	RPS23
EIF2S1	EIF4E
EIF2S1	RPL10A
EIF2S1	RPS24
EIF2S1	RPL21
EIF2S1	RPS3A
EIF2S1	RPS6
EIF2S1	RPL29
EIF2S1	RPL6
EIF2S1	RPL27
EIF2S1	EIF2B3
EIF2S1	RPL7
EIF2S1	RPS26
EIF2S1	RPL13A

EIF2S1	RPL37
EIF2S1	RPL3
EIF2S1	RPL34
EIF2S1	RRP1
EIF2S1	RPL4
EIF2S1	STRN
EIF2S1	RPS15
EIF2S1	EIF3CL
EIF2S1	RPL35
EIF3A	RPL37A
EIF3A	EIF2S3
EIF3A	EIF4A1
EIF3A	EIF3M
EIF3A	EIF3H
EIF3A	RPL8
EIF3A	RPL27
EIF3A	NAT1
EIF3A	RPS3
EIF3A	RPS21
EIF3A	RPL5
EIF3A	UBC
EIF3A	EIF1B
EIF3A	RPL12
EIF3A	RPL18A
EIF3A	RPSA
EIF3A	EIF3F
EIF3A	EIF3CL
EIF3A	RPS11
EIF3A	RPL41
EIF3A	RPS10-NUDT3
EIF3A	RPL30
EIF3A	RPLP2
EIF3A	RPL3L
EIF3A	RPL32
EIF3A	RPS5
EIF3A	RPL35
EIF3A	EIF2S2
EIF3A	RPL36
EIF3A	EIF2S1
EIF3A	EIF5B
EIF3A	RPL35A
EIF3A	CASP4
EIF3A	RPL37
EIF3A	RPL4
EIF3A	RPL26L1
EIF3A	EIF3B

EIF3A	RPL17
EIF3A	EIF4A2
EIF3A	PTBP1
EIF3A	RPL9P7
EIF3A	RPL13A
EIF3A	FAU
EIF3A	RPS6KB1
EIF3A	EIF3G
EIF3A	RPS23
EIF3A	RPLP0P6
EIF3A	EIF1
EIF3A	RPL21
EIF3A	EIF4G1
EIF3A	RPS4X
EIF3A	RPL22
EIF3A	RPL26
EIF3A	RPL24
EIF3A	RPS15
EIF3A	RPL27A
EIF3A	EIF3L
EIF3A	EIF3I
EIF3A	RPS3A
EIF3A	RPL38
EIF3A	EIF4B
EIF3A	CASP2
EIF3A	RPS13
EIF3A	EIF4G3
EIF3A	CASP7
EIF3A	RPL18
EIF3A	EIF3J
EIF3A	RPS12
EIF3A	EIF3K
EIF3A	RPL10A
EIF3A	RPS7
EIF3A	RPS28
EIF3A	ABCE1
EIF3A	RPL31
EIF3A	EIF3E
EIF3A	RPL23
EIF3A	CASP9
EIF3A	PTBP2
EIF3A	RPL34
EIF3A	RPS15A
EIF3A	RPS20
EIF3A	RRP1
EIF3A	EIF3D

EIF3A	RPS2
EIF3A	CASP3
EIF3A	RPS27
EIF3A	RPL3
EIF3A	RPL7
EIF3A	RLIM
EIF3A	RPL11
EIF3A	EIF3C
EIF3A	USP3
EIF3A	RPL15
EIF3A	RPL39
EIF3A	NAP1L4
EIF3A	RPS6
EIF3A	EIF5
EIF3A	RPL29
EIF3A	RPS19
EIF3A	RPS14
EIF3A	CASP8
EIF3A	RPL14
EIF3A	RPL6
EIF3A	EIF4E
EIF3A	CASP6
EIF3A	RPS26
EIF3A	WDR48
EIF3A	RPL19
EIF3A	RPL13
EIF3A	RPS8
EIF3A	RPL28
EIF3A	RPS16
EIF3A	RPS29
EIF3A	EIF1AX
EIF3A	RPS24
EIF3A	RPL23A
EIF3E	RPL9P7
EIF3E	RPS26
EIF3E	RPS15
EIF3E	NAP1L4
EIF3E	RPS29
EIF3E	RPL37A
EIF3E	RPL36
EIF3E	RPL15
EIF3E	EIF2S1
EIF3E	EIF3K
EIF3E	RPL26L1
EIF3E	RPL29
EIF3E	RPS28

EIF3E	UBC
EIF3E	RPS4X
EIF3E	RPL3
EIF3E	EIF2S2
EIF3E	EIF4A2
EIF3E	RPL24
EIF3E	RPL35
EIF3E	RPS8
EIF3E	EIF3H
EIF3E	RPL22
EIF3E	RPL6
EIF3E	RLIM
EIF3E	RPL35A
EIF3E	RPL5
EIF3E	EIF3G
EIF3E	EIF3J
EIF3E	EIF3I
EIF3E	RPL28
EIF3E	RPS6
EIF3E	EIF4A1
EIF3E	RPL23A
EIF3E	EIF3M
EIF3E	EIF5B
EIF3E	RPL32
EIF3E	RPL18A
EIF3E	RPS19
EIF3E	RPS14
EIF3E	RPL30
EIF3E	RPS3
EIF3E	IFIT1
EIF3E	EIF3F
EIF3E	EIF2S3
EIF3E	RPS11
EIF3E	RPS15A
EIF3E	EIF1AX
EIF3E	RPL19
EIF3E	RPL10A
EIF3E	RPL7
EIF3E	RPL3L
EIF3E	RPL23
EIF3E	PSMD12
EIF3E	RPL8
EIF3E	EIF4G1
EIF3E	EIF3C
EIF3E	RPS2
EIF3E	RPS12

EIF3E	EIF3A
EIF3E	RPL41
EIF3E	RRP1
EIF3E	RPS16
EIF3E	RPL4
EIF3E	EIF4E
EIF3E	RPL21
EIF3E	RPS13
EIF3E	RPL38
EIF3E	EIF5
EIF3E	EIF3D
EIF3E	RPL18
EIF3E	RPL13
EIF3E	RPL13A
EIF3E	RPS24
EIF3E	FAU
EIF3E	EIF3CL
EIF3E	RPL11
EIF3E	RPL39
EIF3E	RPL27A
EIF3E	RPS7
EIF3E	RPL12
EIF3E	RPL27
EIF3E	EIF3L
EIF3E	RPS21
EIF3E	RPS27
EIF3E	RPLP0P6
EIF3E	RPS5
EIF3E	EIF4B
EIF3E	RPL31
EIF3E	RPL37
EIF3E	RPL14
EIF3E	RPS20
EIF3E	RPSA
EIF3E	RPL34
EIF3E	RPLP2
EIF3E	RPS10-NUDT3
EIF3E	RPS23
EIF3E	RPL26
EIF3E	RPS3A
EIF3E	EIF3B
EIF3E	RPL17
EIF3I	RPS7
EIF3I	RPS19
EIF3I	RPL24
EIF3I	RPL21

EIF3I	RPL23
EIF3I	RPS21
EIF3I	RPL19
EIF3I	EIF3M
EIF3I	RPS3A
EIF3I	EIF1AX
EIF3I	RPL9P7
EIF3I	EIF1
EIF3I	RPL14
EIF3I	USP3
EIF3I	EIF4G1
EIF3I	RPL35
EIF3I	RPL35A
EIF3I	RPS14
EIF3I	RPS20
EIF3I	EIF3CL
EIF3I	RPS10-NUDT3
EIF3I	RPL13
EIF3I	EIF4A1
EIF3I	RPL15
EIF3I	EIF3E
EIF3I	RPL8
EIF3I	EIF4B
EIF3I	EIF5B
EIF3I	EIF3G
EIF3I	RPL10A
EIF3I	RPS27
EIF3I	RPS12
EIF3I	EIF1B
EIF3I	EIF3H
EIF3I	FAU
EIF3I	RPS24
EIF3I	RPL41
EIF3I	EIF3C
EIF3I	RPL17
EIF3I	RPS29
EIF3I	EIF5
EIF3I	RPS3
EIF3I	RPL32
EIF3I	EIF3B
EIF3I	RPLP2
EIF3I	RPL39
EIF3I	RPL5
EIF3I	RPS8
EIF3I	EIF3A
EIF3I	RPL31

EIF3I	RPS28
EIF3I	RPL11
EIF3I	RPL36
EIF3I	RPL30
EIF3I	EIF2S2
EIF3I	EIF3L
EIF3I	RPL27A
EIF3I	RPS6
EIF3I	EIF3F
EIF3I	RPL3L
EIF3I	RPS15
EIF3I	RPL23A
EIF3I	EIF3D
EIF3I	ABCE1
EIF3I	EIF4A2
EIF3I	RPL13A
EIF3I	EIF2S3
EIF3I	RRP1
EIF3I	RPL18A
EIF3I	RPS11
EIF3I	RPL26L1
EIF3I	RPL7
EIF3I	RLIM
EIF3I	EIF3K
EIF3I	EIF3J
EIF3I	RPL12
EIF3I	RPL6
EIF3I	RPL37
EIF3I	EIF2S1
EIF3I	RPLP0P6
EIF3I	RPS4X
EIF3I	RPS13
EIF3I	RPS16
EIF3I	RPL22
EIF3I	EIF4E
EIF3I	RPS5
EIF3I	RPS23
EIF3I	RPS15A
EIF3I	RPL3
EIF3I	RPL29
EIF3I	RPL4
EIF3I	RPL18
EIF3I	RPL38
EIF3I	RPL28
EIF3I	RPL34
EIF3I	RPS26

EIF3I	RPS2
EIF3I	UBC
EIF3I	RPL26
EIF3I	RPL37A
EIF3I	RPSA
EIF3I	RPL27
EIF3J	EIF2S2
EIF3J	RPL5
EIF3J	EIF3C
EIF3J	RPS2
EIF3J	RPL35
EIF3J	RPSA
EIF3J	RPL6
EIF3J	EIF4G1
EIF3J	EIF5B
EIF3J	RPL22
EIF3J	RPL37A
EIF3J	RPS4X
EIF3J	RPL18
EIF3J	RPL37
EIF3J	RPS26
EIF3J	RPL3
EIF3J	EIF4A1
EIF3J	RPS28
EIF3J	EIF2S3
EIF3J	RPS13
EIF3J	EIF2S1
EIF3J	RPS6
EIF3J	RPS29
EIF3J	RPL15
EIF3J	RPS8
EIF3J	EIF3M
EIF3J	RPS16
EIF3J	RPL19
EIF3J	RPS21
EIF3J	RPS10-NUDT3
EIF3J	EIF3E
EIF3J	RPL13A
EIF3J	EIF3L
EIF3J	RPS20
EIF3J	RPL26
EIF3J	RPL18A
EIF3J	RPL24
EIF3J	EIF4B
EIF3J	RPL28
EIF3J	RLIM

EIF3J	EIF1B
EIF3J	ABCE1
EIF3J	SIAH1
EIF3J	EIF1
EIF3J	RPL39
EIF3J	RPL3L
EIF3J	RPS3A
EIF3J	RPS12
EIF3J	RPS3
EIF3J	RPL41
EIF3J	RPL31
EIF3J	EIF3D
EIF3J	RPL9P7
EIF3J	EIF4E
EIF3J	RPS11
EIF3J	RPL30
EIF3J	RPL38
EIF3J	RPLP2
EIF3J	RPS23
EIF3J	RRP1
EIF3J	RPL14
EIF3J	RPL4
EIF3J	RPS19
EIF3J	EIF3B
EIF3J	FAU
EIF3J	RPL32
EIF3J	EIF3A
EIF3J	EIF1AX
EIF3J	EIF4A2
EIF3J	RPS15A
EIF3J	RPL10A
EIF3J	EIF3CL
EIF3J	EIF3K
EIF3J	RPS27
EIF3J	RPL23
EIF3J	RPL27
EIF3J	UBC
EIF3J	RPL29
EIF3J	RPL11
EIF3J	EIF3F
EIF3J	RPS14
EIF3J	RPL17
EIF3J	RPS24
EIF3J	RPL23A
EIF3J	RPL26L1
EIF3J	EIF5

EIF3J	EIF3I
EIF3J	RPL8
EIF3J	RPL21
EIF3J	RPL34
EIF3J	EIF3H
EIF3J	RPL36
EIF3J	RPS15
EIF3J	RPL13
EIF3J	RPS7
EIF3J	RPL7
EIF3J	EIF3G
EIF3J	RPS5
EIF3J	RPL35A
EIF3J	RPL12
EIF3J	RPL27A
EIF3J	RPLP0P6
EIF3L	EIF3J
EIF3L	EIF3M
EIF3L	EIF3F
EIF3L	EIF3D
EIF3L	EIF3G
EIF3L	EIF3K
EIF3L	EIF3C
EIF3L	EIF3A
EIF3L	EIF3H
EIF3L	EIF3I
EIF3L	EIF3B
EIF3L	EIF3E
EIF3L	UBC
EIF4A2	RPS14
EIF4A2	NAT1
EIF4A2	EIF3G
EIF4A2	CNOT11
EIF4A2	EIF4E2
EIF4A2	EIF3B
EIF4A2	RPL27A
EIF4A2	RPS7
EIF4A2	RPS5
EIF4A2	RPS8
EIF4A2	RPL13A
EIF4A2	EIF4G3
EIF4A2	RPL21
EIF4A2	RPL37A
EIF4A2	RPL31
EIF4A2	RPS19
EIF4A2	EIF4H

EIF4A2	RPL35A
EIF4A2	RPL6
EIF4A2	CNOT6L
EIF4A2	CNOT1
EIF4A2	RLIM
EIF4A2	RPL11
EIF4A2	RPL38
EIF4A2	EIF3E
EIF4A2	RPS26
EIF4A2	RPL9P7
EIF4A2	RPS2
EIF4A2	RPL36
EIF4A2	EIF5B
EIF4A2	RPL35
EIF4A2	CNOT10
EIF4A2	RPS20
EIF4A2	RPL4
EIF4A2	ARIH1
EIF4A2	CNOT9
EIF4A2	EIF4EBP3
EIF4A2	CNOT4
EIF4A2	EIF5
EIF4A2	RPS16
EIF4A2	RRP1
EIF4A2	RPL39
EIF4A2	RPS4X
EIF4A2	RPL23
EIF4A2	NOM1
EIF4A2	RPS28
EIF4A2	RPL17
EIF4A2	RPS3A
EIF4A2	RPL22
EIF4A2	RPL26
EIF4A2	RPS11
EIF4A2	RPS15
EIF4A2	RPS27
EIF4A2	RPL26L1
EIF4A2	PARN
EIF4A2	RPSA
EIF4A2	RPS24
EIF4A2	EIF3A
EIF4A2	RPL34
EIF4A2	RPL41
EIF4A2	RPS23
EIF4A2	RPS3
EIF4A2	CNOT2

EIF4A2	HERC5
EIF4A2	RPL28
EIF4A2	RPL8
EIF4A2	RPL5
EIF4A2	RPL23A
EIF4A2	RPL3L
EIF4A2	RPL37
EIF4A2	PAN3
EIF4A2	RPLP2
EIF4A2	PAIP1
EIF4A2	RPL7
EIF4A2	EIF2S3
EIF4A2	HRH1
EIF4A2	EIF3J
EIF4A2	RPS10-NUDT3
EIF4A2	EIF3H
EIF4A2	RPL30
EIF4A2	EIF4EBP1
EIF4A2	EIF3I
EIF4A2	RPL32
EIF4A2	RPS12
EIF4A2	RPLP0P6
EIF4A2	RPL13
EIF4A2	RPL15
EIF4A2	RPS6
EIF4A2	RPL19
EIF4A2	RPL10A
EIF4A2	RPS29
EIF4A2	UBC
EIF4A2	UBE2L6
EIF4A2	EIF1
EIF4A2	EIF4E
EIF4A2	CNOT6
EIF4A2	EIF3K
EIF4A2	EIF2S1
EIF4A2	CNOT7
EIF4A2	PDCD4
EIF4A2	ISG15
EIF4A2	EIF1AX
EIF4A2	EIF3C
EIF4A2	EIF4A3
EIF4A2	RPL27
EIF4A2	RPL14
EIF4A2	RPS15A
EIF4A2	FAU
EIF4A2	CNOT8

EIF4A2	RPS21
EIF4A2	EIF4A1
EIF4A2	EIF4E3
EIF4A2	RPL12
EIF4A2	TNKS1BP1
EIF4A2	RPS13
EIF4A2	EIF4G1
EIF4A2	EIF4B
EIF4A2	EIF3F
EIF4A2	PAN2
EIF4A2	RPL24
EIF4A2	EIF2S2
EIF4A2	RPL18A
EIF4A2	EIF3D
EIF4A2	RPL29
EIF4A2	RPL18
EIF4A2	RPL3
EIF4B	RPL13
EIF4B	RPL4
EIF4B	RPL35
EIF4B	RPL7
EIF4B	EIF3K
EIF4B	RPL17
EIF4B	USP11
EIF4B	RPS11
EIF4B	EIF3F
EIF4B	EIF3D
EIF4B	RPL37
EIF4B	RPL12
EIF4B	RPL6
EIF4B	RPS19
EIF4B	CNOT8
EIF4B	RPL31
EIF4B	CNOT4
EIF4B	CNOT9
EIF4B	RPS7
EIF4B	RPS23
EIF4B	RPL9P7
EIF4B	RPS6
EIF4B	EIF3C
EIF4B	RPS15
EIF4B	RPS6KB2
EIF4B	EIF4G1
EIF4B	PARN
EIF4B	RPS14
EIF4B	RPS10-NUDT3

EIF4B	RPS24
EIF4B	RPS27
EIF4B	RPL10A
EIF4B	EIF3I
EIF4B	RPL18A
EIF4B	FAU
EIF4B	RPL13A
EIF4B	RPS12
EIF4B	EIF3J
EIF4B	EIF3G
EIF4B	RPS26
EIF4B	EIF2S2
EIF4B	EIF3B
EIF4B	RPLP2
EIF4B	RPL23
EIF4B	RPL24
EIF4B	RPL8
EIF4B	RPL26
EIF4B	RPL22
EIF4B	RPS21
EIF4B	EIF5B
EIF4B	RPS15A
EIF4B	PAN3
EIF4B	CNOT11
EIF4B	EIF4A3
EIF4B	EIF1AX
EIF4B	RPS13
EIF4B	RPL27A
EIF4B	CNOT7
EIF4B	CNOT1
EIF4B	RPL23A
EIF4B	RPL29
EIF4B	EIF3A
EIF4B	RPL28
EIF4B	RPL35A
EIF4B	EIF5
EIF4B	PAK2
EIF4B	RPL38
EIF4B	RPS3
EIF4B	CSTF2
EIF4B	CNOT2
EIF4B	NUP153
EIF4B	RPL26L1
EIF4B	RPL39
EIF4B	RPL15
EIF4B	RPL27

EIF4B	PAIP1
EIF4B	RPSA
EIF4B	EIF4G3
EIF4B	RPL30
EIF4B	RPS28
EIF4B	RPL37A
EIF4B	RPS8
EIF4B	NAT1
EIF4B	RPLP0P6
EIF4B	EIF2S1
EIF4B	RPS16
EIF4B	NUP107
EIF4B	RPS4X
EIF4B	TNKS1BP1
EIF4B	RPL14
EIF4B	CNOT6L
EIF4B	RPL18
EIF4B	PAN2
EIF4B	EIF4A1
EIF4B	RPS20
EIF4B	RPS2
EIF4B	RPL41
EIF4B	RPL34
EIF4B	RPS6KB1
EIF4B	EIF3H
EIF4B	EIF3E
EIF4B	RPL32
EIF4B	RPL11
EIF4B	RLIM
EIF4B	RPS29
EIF4B	RPL36
EIF4B	RPL5
EIF4B	MAGOH
EIF4B	UBC
EIF4B	EIF4E
EIF4B	EIF4A2
EIF4B	RPS6KA1
EIF4B	RPS5
EIF4B	RPL19
EIF4B	CNOT6
EIF4B	RPL21
EIF4B	EIF2S3
EIF4B	RPL3
EIF4B	CNOT10
EIF4B	RPL3L
EIF4B	RRP1

EIF4B	RPS3A
EIF4ENIF1	EIF4E2
EIF4ENIF1	EIF4E
EIF4H	EIF4A2
EIF4H	EIF4A1
EIF4H	EIF4G1
EIF5	RPL10A
EIF5	EIF2S3
EIF5	RPL18
EIF5	RPS26
EIF5	UBC
EIF5	EIF3C
EIF5	RPL26
EIF5	RPS8
EIF5	EIF3K
EIF5	RPL38
EIF5	EIF5B
EIF5	EIF4G1
EIF5	RPS5
EIF5	RPL8
EIF5	RPS7
EIF5	RPS28
EIF5	RPL23A
EIF5	RPS21
EIF5	RPL5
EIF5	RPL27
EIF5	RPL19
EIF5	RPS10-NUDT3
EIF5	RPS15
EIF5	RPS15A
EIF5	EIF3F
EIF5	RPL24
EIF5	RPL35A
EIF5	RPS27
EIF5	RPL3L
EIF5	EIF3G
EIF5	EIF3D
EIF5	RPL14
EIF5	EIF3CL
EIF5	EIF4A2
EIF5	RPL34
EIF5	EIF3B
EIF5	RPS24
EIF5	EIF1AX
EIF5	RPS19
EIF5	RPS3A

EIF5	RPLP0P6
EIF5	RPL15
EIF5	RPS3
EIF5	RPL12
EIF5	RPS2
EIF5	RPL3
EIF5	EIF3I
EIF5	FAU
EIF5	EIF1
EIF5	RPS6
EIF5	RPS29
EIF5	RPL6
EIF5	RPLP2
EIF5	RPL31
EIF5	EIF3H
EIF5	EIF4B
EIF5	RPL21
EIF5	RPL4
EIF5	RPL30
EIF5	RPS12
EIF5	RPS16
EIF5	RRP1
EIF5	RPL35
EIF5	RPL39
EIF5	RPL37A
EIF5	RPL36
EIF5	RPL26L1
EIF5	EIF3E
EIF5	RPL18A
EIF5	EIF2S2
EIF5	ABCE1
EIF5	RPL37
EIF5	RPL17
EIF5	RPS13
EIF5	RPL22
EIF5	EIF3J
EIF5	RPL32
EIF5	EIF4A1
EIF5	EIF4E
EIF5	RPL13A
EIF5	EIF2S1
EIF5	RPL11
EIF5	RPS23
EIF5	EIF2B5
EIF5	RPL13
EIF5	RPS20

EIF5	EIF3A
EIF5	RPL9P7
EIF5	RPL29
EIF5	RPL41
EIF5	RPS4X
EIF5	RPS14
EIF5	RPS11
EIF5	RPL28
EIF5	RPL7
EIF5	RPSA
EIF5	RPL27A
EIF5	RPL23
EIF5B	RPL26L1
EIF5B	RPL13
EIF5B	UBC
EIF5B	RPL19
EIF5B	RPS3
EIF5B	RPS5
EIF5B	RPL28
EIF5B	EIF3J
EIF5B	RPL37A
EIF5B	EIF5
EIF5B	RPL27
EIF5B	RPL32
EIF5B	EIF1
EIF5B	RPS24
EIF5B	RPL9P7
EIF5B	RPL24
EIF5B	RPS15
EIF5B	RPS3A
EIF5B	EIF3G
EIF5B	RPL22
EIF5B	RPL30
EIF5B	RPS15A
EIF5B	EIF4A2
EIF5B	RPS4X
EIF5B	EIF3CL
EIF5B	RPS8
EIF5B	EIF4A1
EIF5B	RPS6
EIF5B	RPL35A
EIF5B	RRP1
EIF5B	RPL15
EIF5B	EIF3I
EIF5B	RPL29
EIF5B	RPS2

EIF5B	EIF3E
EIF5B	RPL36
EIF5B	EIF1AX
EIF5B	EIF3A
EIF5B	RPL34
EIF5B	EIF3F
EIF5B	RPS10-NUDT3
EIF5B	RPL23A
EIF5B	RPS14
EIF5B	EIF4B
EIF5B	RPS16
EIF5B	EIF2S1
EIF5B	RPLP0P6
EIF5B	RPS11
EIF5B	RPS20
EIF5B	EIF2S3
EIF5B	RPL13A
EIF5B	EIF3D
EIF5B	RPS13
EIF5B	RPS26
EIF5B	RPL11
EIF5B	EIF4G1
EIF5B	RPL17
EIF5B	RPL27A
EIF5B	RPL35
EIF5B	EIF4E
EIF5B	RPS27
EIF5B	RPS29
EIF5B	EIF3B
EIF5B	RPS28
EIF5B	FAU
EIF5B	EIF3C
EIF5B	RPL7
EIF5B	EIF2S2
EIF5B	RPS19
EIF5B	RPL41
EIF5B	RPS21
EIF5B	RPL14
EIF5B	RPS7
EIF5B	RPL38
EIF5B	RPSA
EIF5B	RPS12
EIF5B	RPL12
EIF5B	RPL3L
EIF5B	RPL37
EIF5B	EIF3K

EIF5B	RPL23
EIF5B	RPL3
EIF5B	RPLP2
EIF5B	RPL8
EIF5B	EIF3H
EIF5B	RPL21
EIF5B	RPL39
EIF5B	RPS23
EIF5B	RPL18A
EIF5B	RPL5
EIF5B	RPL26
EIF5B	RPL31
EIF5B	RPL6
EIF5B	RPL18
EIF5B	RPL10A
EIF5B	RPL4
ELF1	E2F2
ELF1	E2F3
ELF1	E2F1
ELF1	RB1
ELF1	TFDP1
ELP2	WDR61
ELP2	UBC
ELP2	KTI12
ELP2	ELP6
ELP2	ELP1
ELP2	JAK1
ELP2	ELP5
ELP2	ELP4
ELP2	NFYA
ELP2	DPH3
ELP2	STAT3
ELP2	ELP3
EMC7	EMC3
EMC7	EMC1
EMC7	MMGT1
EMC7	EMC4
EMC7	EMC8
EMC7	EMC6
EMC7	EMC9
EMC7	EMC10
EMC7	EMC2
ENDOG	UBC
ENDOG	AIFM1
ENSA	ARPP19
ENSA	MASTL

ENSA	CCNB1
ENSA	CDK1
ERF	KEAP1
ERF	ETS2
ERH	CAD
ERH	POLDIP3
ERH	MAGOH
FBXL19	PSD
FBXL19	USP47
FBXL19	FBXW5
FBXL19	FBXO4
FBXL19	RBX1
FBXL19	FBXO17
FBXL19	CCNF
FBXL19	FBXL3
FBXL19	FBXO9
FBXL19	SKP2
FBXL19	SKP1
FBXL19	FBXW11
FBXL19	FBXO27
FBXL19	FBXO31
FBXL19	CUL1
FBXL19	BTRC
FBXL19	FBXW7
FBXL19	FBXO6
FBXL19	FBXL5
FBXL19	FBXO44
FBXL6	SKP1
FBXL6	CUL1
FBXL7	SKP1
FBXL7	CCNF
FBXO22	SKP1
FBXO22	UBC
FBXO45	PAM
FBXO45	SKP1
FBXO45	UNC13A
FBXO7	CUL1
FBXO7	PSMF1
FBXO7	SKP1
FBXW2	UBE2D3
FBXW2	GCM1
FBXW2	CUL1
FBXW2	SKP1
FHOD1	RAC3
FHOD1	RAC1
FNTA	PGGT1B

FNTA	CASP3
FNTA	CHURC1-FNTB
FNTA	GNGT1
FNTA	FNTB
FPGS	DHFRP1
FPGS	GGH
FPGS	UBC
FPGS	TYMS
FTL	FECH
FTL	AP1S1
FTL	UBC
FTL	AP1S3
FTL	PUM1
FTL	DNAJC6
FTL	AP1G1
FTL	AP1S2
FTL	ARRB1
FTL	HSPA8
FTL	FTH1
FTL	CLINT1
FTL	SH3D19
FTL	AP1M1
FTL	PSENEN
FTL	AP1B1
FUCA1	USP16
FUCA1	MARK2
FXR1	CYFIP2
FXR1	SRA1
FXR1	DICER1
FXR1	AGO2
FXR1	UBC
FXR2	CYFIP2
FXR2	TDRD3
FXR2	AGO1
FXR2	SRA1
GAK	CLTB
GAK	CLHC1
GAK	CLTC
GAK	UBC
GAK	CLTA
GAK	CLTCL1
GALK1	IL11RA
GALK1	GALM
GALK1	GALE
GALK1	GALT
GAPDH	PCNA

GAPDH	SYNCRIP
GAPDH	MYC
GAPDH	HSPD1
GAPDH	FOS
GAPDH	ESR1
GAPDH	ATG12
GAPDH	PGAM1
GAPDH	ENO1
GAPDH	EGFR
GAPDH	SIAH1
GAPDH	PKM
GAPDH	UBC
GAPDH	RAB2A
GAPDH	BPGM
GAPDH	MAX
GAPDH	TF
GAPDH	EEF1A1P5
GAPDH	PGK1
GAPDH	HDAC9
GAPDH	ALDOC
GAPDH	ENO2
GAPDH	ALDOA
GAPDH	ALDOB
GAPDH	TPI1
GAPDH	PRDM10
GAPDH	ACTG1
GAPDH	NLK
GAPDH	EDN1
GAPDH	SLC2A4
GAPDH	TSPO
GAPDH	RPL13A
GAPDH	AKT1
GAPDH	PLD2
GAPDH	APP
GAPDH	AKT2
GAPDH	TKT
GAPDH	SRC
GAPDH	ENO3
GAPDH	HTT
GAPDH	TP53
GGCT	GGT5
GGCT	GGT1
GGCT	ANPEP
GGCT	LAP3
GGCT	GCLC
GGCT	GCLM

GGCT	GSS
GGCT	GGT7
GGH	FPGS
GGH	DHFRP1
GIGYF2	CNOT9
GIGYF2	SF1
GIGYF2	UBC
GIGYF2	GRB10
GLMN	FKBP4
GLMN	RBX1
GLMN	CUL1
GLMN	FKBP1A
GNG8	GNG13
GNG8	GNG3
GNG8	ADCY1
GNG8	GNG4
GNG8	GCGR
GNG8	ADCY9
GNG8	GNB2
GNG8	GNAS
GNG8	ADCY8
GNG8	GNGT1
GNG8	GNG7
GNG8	GNB4
GNG8	GNG12
GNG8	ADCY6
GNG8	ADCY7
GNG8	GNG10
GNG8	GNG2
GNG8	GNB1
GNG8	GNAT2
GNG8	GNG5
GNG8	ADCY5
GNG8	GNG11
GNG8	ADCY3
GNL1	SDAD1
GNL1	PA2G4
GNL1	PES1
GNL1	NGB
GNL1	NMD3
GNL2	MRTO4
GNL2	NOP2
GNL2	MDN1
GNL2	EIF6
GNL2	PA2G4
GNL2	NLE1

GNL2	GNL3L
GNL2	RRS1
GNL2	NGB
GNL2	TSR1
GNL2	RSL24D1
GNL2	FTSJ3
GNL2	NSUN6
GNL2	NIFK
GNL2	NSA2
GNL2	TEX10
GNL2	GNL3
GNL2	SDAD1
GNL2	RPF2
GNL2	PES1
GNPAT	FAR2
GNPAT	AGPS
GNPAT	GPD2
GNPAT	GPD1
GNPAT	GPD1L
GNPAT	FAR1
GOPC	GOLGA3
GOPC	STX6
GOPC	IRF2BP2
GOPC	NDUFB10
GOT2	TAT
GOT2	MPST
GOT2	ADSS2
GOT2	DDC
GOT2	MDH2
GOT2	MIF
GOT2	CTH
GOT2	PRX
GOT2	MDH1
GOT2	CS
GOT2	IDH2
GOT2	UBC
GOT2	TST
GOT2	NAT8L
GOT2	TPO
GOT2	ALDH4A1
GOT2	IDH1
GOT2	NIT2
GOT2	ASNS
GOT2	SLC25A13
GOT2	LDHB
GOT2	CAD

GOT2	PC
GOT2	HOGA1
GOT2	CDO1
GOT2	ASS1
GOT2	GLUD1
GOT2	GPT2
GOT2	ADSS1
GOT2	PAH
GOT2	GPT
GOT2	HPD
GOT2	LDHAL6A
GOT2	IDH3B
GOT2	SLC25A12
GOT2	TH
GPS2	TP53
GPS2	HDAC3
GPS2	TBL1X
GPS2	NCOR2
GPS2	NCOR1
GPX1	GSTP1
GPX1	CYP2C8
GPX1	GGT1
GPX1	ABL1
GPX1	GGT7
GPX1	TP53
GPX1	SOD1
GPX1	GSR
GPX1	GSTT2B
GPX1	GGT5
GPX1	MGST2
GPX1	GSTM4
GPX1	CAT
GPX1	CYP2J2
GPX1	GSTO1
GPX1	GSTM2
GPX1	GSTA4
GPX1	SOD2
GPX1	MIEN1
GPX1	GSTK1
GPX1	GSTO2
GPX1	GSTM3
GPX1	MGST3
GPX1	MGST1
GPX1	GSS
GPX1	TXN
GREM2	BMP2

GREM2	CHRDL1
GREM2	FSTL1
GSPT1	UPF3A
GSPT1	PABPC3
GSPT1	PABPC4
GSPT1	RLIM
GSPT1	UPF2
GSPT1	UBC
GSPT1	ETF1
GSPT1	UPF1
GSPT1	PABPC1L
GSPT1	UPF3B
GTF2E2	GTF2B
GTF2E2	GTF2F1
GTF2E2	CCNH
GTF2E2	POLR2A
GTF2E2	TAF1
GTF2E2	TAF13
GTF2E2	SND1
GTF2E2	TBP
GTF2E2	GTF2H3
GTF2E2	POLR2J
GTF2E2	POLR2E
GTF2E2	POLR2D
GTF2E2	POLR2F
GTF2E2	POLR2G
GTF2E2	POLR2L
GTF2E2	ERCC3
GTF2E2	TAF6
GTF2E2	GTF2A2
GTF2E2	GTF2A1
GTF2E2	GTF2F2
GTF2E2	ERCC2
GTF2E2	POLR2K
GTF2E2	GTF2H5
GTF2E2	POLR2C
GTF2E2	ATF7IP
GTF2E2	GTF2H1
GTF2E2	TAF15
GTF2E2	POLR2I
GTF2E2	POLR2B
GTF2E2	TAF11
GTF2E2	GTF2E1
GTF2E2	TAF4B
GTF2E2	POLR2H
GTF2E2	TAF4

GTF2E2	TAF5
GTF2E2	MNAT1
GTF2E2	TAF10
HACD2	HSD17B12
HACD2	TECR
HAND1	ADSS2
HAND1	MEF2C
HAND1	HAND2
HAND1	GATA4
HIRA	ASF1B
HIRA	ASF1A
HM13	RNF139
HM13	DFFB
HM13	UBC
HMGA1	CDK1
HMGA1	HIPK2
HMGA1	ASF1A
HMGA1	EP400
HMGA1	CABIN1
HMGA1	UBN1
HMGA1	LMNB1
HMGA1	CREBBP
HMGA1	RPS6KB1
HMGA1	RB1
HMGA1	PRMT6
HMGA1	UBE2I
HMGA1	CDKN1A
HMGA1	MAX
HMGA1	MYC
HMGA1	PPARG
HMGA1	TP53
HMGA1	KAT2B
HMGN1	RPS6KA5
HMGN1	HMGN2
HNRNPAB	MRPS25
HNRNPAB	PTBP1
HNRNPAB	MRPS7
HNRNPAB	UBC
HNRNPAB	SF3A2
HNRNPH3	UBC
HNRNPH3	HNRNPA1
HNRNPH3	HNRNPA2B1
HNRNPH3	HNRNPH1
HNRNPH3	HNRNPUL1
HNRNPH3	HNRNPA1L2
HNRNPH3	HNRNPF

HS2ST1	GLCE
HS2ST1	HS6ST1
HSF1	HSPA8
HSF1	SMARCA4
HSF1	HSP90AB2P
HSF1	PRKACA
HSF1	HSPA1A
HSF1	DAXX
HSF1	MTOR
HSF1	XRCC6
HSF1	HMOX1
HSF1	BAG3
HSF1	DNAJB1
HSF1	MAPK9
HSF1	PRKDC
HSF1	HSBP1
HSF1	MAPK8
HSF1	HSPB1
HSF1	SUMO1
HSF1	TRPV1
HSF1	HSPA4
HSF1	JUN
HSF1	VAMP2
HSF1	ARNTL
HSF1	ABCB1
HSF1	HSP90AA1
HSF1	STAT1
HSF1	SERPINE1
ID1	ETS2
ID1	SMAD1
ID1	CEBPB
ID1	SMAD4
ID1	THBS1
ID1	CDC20
ID1	TCF3
ID1	SMAD5
ID1	HDAC1
ID1	SMAD9
ID2	TCF4
ID2	ARNT
ID2	HDAC3
ID2	HIF1A
ID2	CTNNB1
ID2	TCF7L2
ID2	E2F4
ID2	TCF3

ID2	MAX
ID2	RB1
ID2	PIM1
ID2	MYC
ID2	TCF12
IMPACT	COPS5
IMPACT	GCN1
IPO5	RAG2
IPO5	UBC
IPO5	NUP98
IPO5	KPNB1
IPO5	RANBP1
IPO5	IMP4
IPO5	SENP2
IPO5	SENP1
IPO5	RANP1
IPO5	NUP62
IPO5	NUP153
IPO5	IPO7
IPO5	RPL23A
IPO7	KPNB1
IPO7	SMAD2
IPO7	RANP1
IPO7	RPL23A
IPO7	UBC
IPO7	IPO5
JAM3	TJP1
JAM3	ITGB2
JAM3	PARD3
JAM3	ITGA4
JAM3	ITGB1
JAM3	JAM2
KANSL3	PHF20
KANSL3	KAT8
KANSL3	NSL1
KANSL3	WDR5
KANSL3	KANSL2
KANSL3	OGT
KANSL3	MCRS1
KARS1	NARS1
KARS1	TRMT1
KARS1	LARS1
KARS1	TXLNA
KARS1	RPSA
KARS1	AIMP2
KARS1	HMGCS1

KARS1	EEF1E1
KARS1	IARS1
KARS1	AIMP1
KARS1	UBC
KARS1	UBA1
KCTD3	MRI1
KCTD3	LRRC40
KIF14	CIT
KIF14	PRC1
KIF15	KIF26A
KIF15	TPX2
KIF15	NUMA1
KIF15	AURKA
KIF5B	KLC2
KIF5B	KLC4
KIF5B	CDH2
KIF5B	CTNNB1
KIF5B	CTNND1
KIF5B	VAMP2
KIF5B	CPE
KIF5B	SYBU
KIF5B	MYO5A
KIF5B	RAB27A
KIF5B	CTNNA1
KIF5B	KLC1
KLF11	EP300
KLF11	SIN3A
KREMEN1	DKK1
KREMEN1	KREMEN2
KREMEN1	LRP6
KRIT1	RAP1B
KRIT1	ITGB1BP1
KRIT1	PDCD10
KRIT1	CCM2
LAMP1	EGFR
LAMP1	CD8A
LAMP1	NCAM1
LCOR	ESR1
LCOR	CTBP1
LGALS1	MYC
LGALS1	MAX
LGALS1	GEMIN4
LGALS1	KRAS
LGALS1	TNC
LGALS1	UBC
LGALS1	SUSD2

LGALS1	ZBTB17
LIMA1	CTNND1
LIMA1	CTNNB1
LIMA1	CTNNA1
LIMA1	CDH1
LRRFIP1	FLII
LRRFIP1	MYD88
LRRFIP1	CTNNB1
LSM14A	DDX6
LSM14A	DCP2
LSM14A	DDX58
LSM7	SNRPF
LSM7	PRPF6
LSM7	LSM5
LSM7	SNRPD2
LSM7	DCP2
LSM7	ZMAT2
LSM7	DCP1B
LSM7	LSM3
LSM7	LSM1
LSM7	DDX6
LSM7	PATL1
LSM7	NAA38
LSM7	WDR44
LSM7	CRNKL1
LSM7	SART1
LSM7	LSM6
LSM7	LSM4
LSM7	SNRPB
LSM7	LSM2
LSM7	USP4
LSM7	EDC4
LSM7	XRN1
LSM7	EDC3
LSM7	SNRPD3
LSM7	TXNL4A
LSM7	SNRPN
LSM7	SART3
LSM7	SNRPD1
LSM7	PRPF4
LSM7	LSM10
LUC7L	PRPF40A
LUC7L	RBM25
LUC7L	NCBP2
LUC7L	PRPF39
LUC7L	SNRNP70

LUC7L	SNRPC
LUC7L3	SART1
LUC7L3	SRPK2
LUC7L3	RBM25
LYAR	SSB
LYAR	LARP7
MAP1A	DLG2
MAP1A	DLG4
MAPKAPK2	MAPK14
MAPKAPK2	CREB1
MAPKAPK2	ARPC5
MAPKAPK2	HSPB1
MAPKAPK2	TCF3
MAPKAPK2	ALOX5
MAPKAPK2	HNRNPA0
MARK1	MARK2
MARK1	USP21
MARK1	MAPT
MARK1	USP16
MARK4	MAPT
MARK4	STRADA
MARK4	STRADB
MARK4	USP21
MARK4	USP16
MARK4	RPTOR
MARK4	CAB39
MARK4	STK11
MARK4	MARK2
MAT2A	UBC
MAT2A	AHCY
MAT2A	DNMT1
MAT2A	DNMT3A
MAT2A	AMD1
MAT2A	SP1
MAT2A	MAT2B
MAT2A	AHCYL2
MAT2A	MYB
MAT2A	AHCYL1
MAT2A	MTR
MAT2A	DNMT3B
MAT2A	BHMT
MAT2A	TAT
MAT2A	IL4I1
MATN3	COMP
MATN3	MATN1
MDK	ITGA6

MDK	GPC2
MDK	SDC4
MDK	ITGA4
MDK	ITGB1
MERTK	PIK3R1
MERTK	GAS6
MERTK	PROS1
METTL1	NSUN2
METTL1	TRMT10A
METTL1	WDR4
METTL14	WTAP
METTL14	METTL4
METTL14	METTL3
MGAT5	MGAT4B
MGAT5	MGAT4A
MGAT5	MGAT3
MIF	IL4I1
MIF	GATA4
MIF	SRA1
MIF	GOT2
MIF	SAP18
MIF	SMAD9
MIF	TIRAP
MIF	UBC
MIF	KITLG
MIF	ACVR1
MIF	SF1
MIF	AMHR2
MIF	COPS5
MIF	BMPR1B
MIF	GOT1
MIF	BMPR1A
MIF	SMAD5
MIF	HPD
MIF	CD74
MIF	TAT
MIF	PTPN11
MIF	MYD88
MIF	FKBP1A
MIF	BMPR2
MIF	FGF2
MIF	SMAD1
MIF	TLR4
MIF4GD	LSM5
MIF4GD	SLBP
MKI67	NIFK

MKI67	UBC
MLLT1	AFF1
MLLT1	CDK9
MLLT1	MLLT10
MLLT1	DOT1L
MLLT1	MLLT3
MLLT1	AFF4
MLLT1	MLLT6
MLX	FASN
MLX	MNT
MLX	MXD4
MLX	ACACB
MLX	ACACA
MLX	MLXIP
MMADHC	MMACHC
MMADHC	MTRR
MMADHC	MTR
MOCS2	MOCS3
MOCS2	GPHN
MOCS2	MOCS1
MOGS	TIMM44
MOGS	RPN1
MOGS	STT3A
MOGS	RPN2
MOGS	WBP1
MOGS	CANX
MOGS	DAD1
MOGS	TUSC3
MOGS	STT3B
MOGS	MLEC
MOGS	GANC
MOGS	GANAB
MON2	ARL1
MON2	ARL16
MPRIP	PPP1R12A
MPRIP	RHOA
MPRIP	PRKG1
MPRIP	PPP1R12C
MRPL24	RPS29
MRPL24	MRPS5
MRPL24	SEC61A1
MRPL24	RPL11
MRPL24	RPS18
MRPL24	MRPL21
MRPL24	RPL4
MRPL24	MRPL51

MRPL24	RPL15
MRPL24	MRPS14
MRPL24	EFL1
MRPL24	MRTO4
MRPL24	RPS9
MRPL24	RPL13A
MRPL24	RPL9P7
MRPL24	RPL10
MRPL24	RPS15
MRPL24	GFM1
MRPL24	IMP3
MRPL24	RPL17-C18orf32
MRPL24	RPS12
MRPL24	RPL17
MRPL24	MRPL36
MRPL24	RPL5
MRPL24	MRPL40
MRPL24	RPL10A
MRPL24	MRPL17
MRPL24	MRPL3
MRPL24	MRPL2
MRPL24	MRPS2
MRPL24	RPS14
MRPL24	MRPL11
MRPL24	RPS15A
MRPL24	GFM2
MRPL24	RPL8
MRPL24	RPS23
MRPL24	RPL27A
MRPL24	RPSAP58
MRPL24	RPL7
MRPL24	RPL35
MRPL24	MRPL44
MRPL24	SLC25A10
MRPL24	RPS2
MRPL24	SEC61A2
MRPL24	RPL29
MRPL24	RPL23
MRPL24	MRPL15
MRPL24	TSR1
MRPL24	EFTUD2
MRPL24	MRPL39
MRPL24	MRPL19
MRPL24	RPL12
MRPL24	RPS5
MRPL24	RPL3L

MRPL24	RPLP0P6
MRPL24	RPL37A
MRPL24	RPS11
MRPL24	UBC
MRPL24	MRPL37
MRPL24	MRPL16
MRPL24	MRPL33
MRPL24	MRPL27
MRPL24	EEF2
MRPL24	MRPL22
MRPL24	RPS3
MRPL24	RPL23A
MRPL24	MRPL4
MRPL24	MRPS11
MRPL24	RPL3
MRPL24	RPL32
MRPL24	RPS20
MRPL24	RPL34
MRPL24	RPSA
MRPL24	RPL21
MRPL24	RPL31
MRPL24	RPL7L1
MRPL24	MRPL1
MRPL24	RPL36
MRPL24	MRPS7
MRPL24	MRPS10
MRPL24	RPL13
MRPL24	RPL19
MRPL24	MRPL12
MRPL44	MRPL47
MRPL44	MRPL51
MRPL44	MRPL39
MRPL44	MRPL38
MRPL44	MRPL19
MRPL44	TMEM177
MRPL44	RPL13
MRPL44	TSR1
MRPL44	MRPL37
MRPL44	MRPL24
MRPL44	MRPL32
MRPL44	MRPL50
MRPL44	MRPL9
MRPL44	MRPL42
MRPL50	TSR1
MRPL50	MRPL19
MRPL50	MRPL32

MRPL50	MRPL15
MRPL50	MRPL37
MRPL50	ATP6V0A1
MRPL50	MRPL47
MRPL50	MRPL38
MRPL50	MRPL40
MRPL50	MRPL44
MRPL50	TMEM177
MRPS22	MRPS25
MRPS22	MRPS35
MRPS22	MRPS16
MRPS22	MRPS26
MRPS31	SARNP
MRPS31	SRPRB
MSL1	MSL3
MSL1	KAT8
MSL1	MSL2
MT-CO1	MT-ND4
MT-CO1	COX4I1
MT-CO1	COX7A2L
MT-CO1	MT-CO2
MT-CO1	MT-CO3
MT-CO1	UQCRFS1P1
MT-CO1	ROPN1B
MT-CO1	CYC1
MT-CO1	COX5A
MT-CO1	MT-ATP6
MT-CO1	COX4I2
MT-CO1	UQCRB
MT-CO1	COX8A
MT-CO1	COX10
MT-CO1	MT-ND5
MT-CO1	UQCRQ
MT-CO1	MT-ND2
MT-CO1	UQCRC1
MT-CO1	COX6C
MT-CO1	COX6A1
MT-CO1	UQCR10
MT-CO1	COX6B1
MT-CO1	CYCS
MT-CO1	UQCRHL
MT-CO1	COX7C
MT-CO1	NDUFA4
MT-CO1	COX5B
MT-CO1	UQCRC2
MT-CO1	MT-CYB

MT-CO3	COX7A2L
MT-CO3	CYC1
MT-CO3	UQCRC2
MT-CO3	MT-ATP6
MT-CO3	ROPN1B
MT-CO3	COX6C
MT-CO3	COX5A
MT-CO3	COX6A1
MT-CO3	MT-ND5
MT-CO3	MT-CO1
MT-CO3	COX7C
MT-CO3	COX5B
MT-CO3	UQCRHL
MT-CO3	UQCRC1
MT-CO3	MT-ND3
MT-CO3	MT-CYB
MT-CO3	UQCRFS1P1
MT-CO3	COX4I1
MT-CO3	COX8A
MT-CO3	COX10
MT-CO3	UQCR10
MT-CO3	CYCS
MT-CO3	UQCRQ
MT-CO3	MT-ND2
MT-CO3	MT-ND4
MT-CO3	COX6B1
MT-CO3	NDUFA4
MT-CO3	MT-CO2
MT-CO3	UQCRB
MTF2	EZH2
MTF2	SUZ12
MTF2	EZH1
MTMR6	PI4K2B
MTMR6	PI4K2A
MTMR6	INPP4B
MTMR6	RAB1B
MTMR6	MTMR9
MTMR6	PIPSL
MTMR6	SYNJ2
MTMR6	SYNJ1
MTMR6	INPP4A
MTMR9	EMILIN1
MTMR9	MTMR8
MTMR9	MTMR6
MT-ND5	MT-CYB
MT-ND5	NDUFA4

MT-ND5	NDUFB7
MT-ND5	NDUFS8
MT-ND5	NDUFB3
MT-ND5	NDUFS5
MT-ND5	NDUFS6
MT-ND5	NDUFB6
MT-ND5	NDUFB1
MT-ND5	NDUFV3
MT-ND5	NDUFB5
MT-ND5	NDUFA12
MT-ND5	NDUFB11
MT-ND5	NDUFA9
MT-ND5	MT-CO3
MT-ND5	NDUFA5
MT-ND5	NDUFA6
MT-ND5	NDUFA1
MT-ND5	ROPN1B
MT-ND5	NDUFS3
MT-ND5	NDUFB8
MT-ND5	UQCRQ
MT-ND5	MT-ND4L
MT-ND5	UQCRFS1P1
MT-ND5	NDUFAB1
MT-ND5	NDUFB4
MT-ND5	LRPPRC
MT-ND5	MT-ND6
MT-ND5	NDUFA2
MT-ND5	NDUFAF1
MT-ND5	MT-CO1
MT-ND5	NDUFB10
MT-ND5	MT-ND2
MT-ND5	NDUFA8
MT-ND5	NDUFC2-KCTD14
MT-ND5	MT-ND4
MT-ND5	MT-CO2
MT-ND5	MT-ND3
MT-ND5	NDUFV1
MT-ND5	CYC1
MT-ND5	NDUFA10
MT-ND5	NDUFA7
MT-ND5	NDUFS1
MT-ND5	NDUFA13
MT-ND5	UQCRHL
MT-ND5	MT-ATP6
MT-ND5	UQCR10
MT-ND5	UQCRB

MT-ND5	NDUFV2
MT-ND5	NDUFS2
MT-ND5	NDUFB9
MT-ND5	NDUFB2
MT-ND5	CYCS
MT-ND5	NDUFC1
MT-ND5	NDUFA11
MT-ND5	UQCRC2
MT-ND5	NDUFS4
MT-ND5	UQCRC1
MT-ND5	NDUFS7
MT-ND6	NDUFC1
MT-ND6	NDUFV3
MT-ND6	NDUFAB1
MT-ND6	NDUFS2
MT-ND6	NDUFV1
MT-ND6	MT-CYB
MT-ND6	NDUFA11
MT-ND6	NDUFS1
MT-ND6	NDUFB9
MT-ND6	MT-ND2
MT-ND6	NDUFS3
MT-ND6	MT-ND4
MT-ND6	NDUFS5
MT-ND6	NDUFB6
MT-ND6	NDUFS4
MT-ND6	NDUFB11
MT-ND6	NDUFA1
MT-ND6	NDUFA12
MT-ND6	ROPN1B
MT-ND6	NDUFV2
MT-ND6	MT-ND4L
MT-ND6	NDUFC2-KCTD14
MT-ND6	NDUFB1
MT-ND6	NDUFB3
MT-ND6	MT-ND3
MT-ND6	UQCRFS1P1
MT-ND6	NDUFA9
MT-ND6	CYC1
MT-ND6	NDUFB2
MT-ND6	UQCRB
MT-ND6	NDUFB7
MT-ND6	UQCRHL
MT-ND6	MT-ND5
MT-ND6	NDUFA5
MT-ND6	UQCRC2

MT-ND6	NDUFB5
MT-ND6	CYCS
MT-ND6	NDUFA2
MT-ND6	NDUFB4
MT-ND6	UQCR10
MT-ND6	NDUFB8
MT-ND6	NDUFA4
MT-ND6	NDUFA13
MT-ND6	NDUFS8
MT-ND6	NDUFS6
MT-ND6	NDUFA8
MT-ND6	NDUFB10
MT-ND6	NDUFA10
MT-ND6	UQCRC1
MT-ND6	UQCRQ
MT-ND6	NDUFS7
MTX2	TOMM20
MTX2	SAMM50
MTX2	TOMM5
MTX2	TOMM40
MTX2	TOMM22
MTX2	TOMM7
MTX2	IMMT
MTX2	MTX1
NAA16	NAA10
NAA16	NAA30
NAA16	NAA11
NAA16	HYPK
NACA	BTF3
NACA	RPL32
NACA	BTF3L4
NACA	UBC
NACAD	BTF3
NACAD	BTF3L4
NAGK	PGM3
NAGK	HEXB
NAGK	HEXA
NAGK	GNPNAT1
NAPG	STX8
NAPG	NSF
NCS1	PI4KB
NCS1	ARF1
NEU3	NPL
NEU3	GALC
NEU3	GAL3ST1
NEU3	GRB2

NEU3	UGT8
NEU3	ARSA
NIF3L1	TACC1
NIF3L1	PLXNB2
NIF3L1	THOC7
NMT1	CASP8
NMT1	BID
NOLC1	SNU13
NOLC1	CSNK2A3
NOLC1	GTF2B
NOLC1	RRS1
NOLC1	FBL
NOLC1	NOP56
NOLC1	NOP58
NOLC1	RPA1
NOLC1	COIL
NPDC1	UBC
NPDC1	MADD
NQO1	VKORC1
NQO1	ODC1
NQO1	GGCX
NUB1	UBE2K
NUB1	PSMD4
NUB1	NEDD8-MDP1
OAT	ARG1
OAT	UBC
OAT	ALDH4A1
OAT	ODC1
OAT	OTC
OAT	ARG2
OAT	ALDH18A1
OAT	ABHD14A-ACY1
ODC1	PSME4
ODC1	PSMD4
ODC1	PSMB6
ODC1	ARG1
ODC1	OAZ2
ODC1	PSMD10
ODC1	PSMD6
ODC1	DLD
ODC1	PSMD13
ODC1	OTC
ODC1	PSMA6
ODC1	PSMA1
ODC1	PSMC3
ODC1	SRM

ODC1	PSMB4
ODC1	CREBBP
ODC1	PSMD11
ODC1	OAT
ODC1	MAX
ODC1	ARG2
ODC1	PSMA4
ODC1	PSMB3
ODC1	PSMD5
ODC1	PSMC6
ODC1	PSMD8
ODC1	PSMD9
ODC1	EP300
ODC1	OAZ1
ODC1	PSMC1
ODC1	PSMA5
ODC1	AMD1
ODC1	NQO1
ODC1	PSME1
ODC1	SAT2
ODC1	RPN1
ODC1	DAO
ODC1	PSMB1
ODC1	PSMC5
ODC1	PSMF1
ODC1	PSMB2
ODC1	AGMAT
ODC1	AADAT
ODC1	PSMC2
ODC1	PSMB10
ODC1	PSME2
ODC1	PSMB7
ODC1	PSMA7
ODC1	PSME3
ODC1	OAZ3
ODC1	PSMD7
ODC1	PSMD3
ODC1	PSMD14
ODC1	PSMD12
ODC1	PSMC4
ODC1	OGDH
ODC1	MYC
ODC1	DLST
ODC1	PSMB5
ODC1	ABHD14A-ACY1
ODC1	RPN2

ODC1	AZIN1
ODC1	SAT1
ODC1	PSMA3
OSTM1	RGS19
OSTM1	CLCN7
OSTM1	RGS20
PANK3	PPAT
PANK3	PPCDC
PANK3	PPCS
PANK3	ENPP3
PANK4	ENPP3
PANK4	PPCDC
PANK4	PPCS
PANK4	PPAT
PBX3	HOXC6
PBX3	MEIS3
PCK2	FOS
PCK2	PC
PCK2	FOXO1
PCK2	CREB1
PCK2	NR2F2
PCK2	AKT1
PCK2	NCOA1
PCOLCE	ADAMTS3
PCOLCE	ADAMTS14
PCOLCE	B2M
PCOLCE	ADAMTS2
PCOLCE	BMP1
PDIA4	HYOU1
PDIA4	APOB
PDIA4	PPIB
PDIA4	UBC
PDIA4	ERO1A
PDIA4	HSP90B1
PDIA4	HSPA5
PDIA4	CALR
PDLIM5	MYOZ2
PDLIM5	ACTN1
PDPR	GLDC
PDPR	GCSH
PDRG1	PFDN2
PDRG1	PFDN4
PEBP1	UBC
PEBP1	CHUK
PEBP1	PRKCZ
PEBP1	RAF1

PEBP1	BIRC5
PEBP1	MAP2K3
PEBP1	PRKCA
PEBP1	MAP2K6
PEBP1	INCENP
PEBP1	AURKB
PEBP1	CDCA8
PEBP1	IKBKB
PEG10	MYC
PEG10	UBC
PEG10	MAX
PGLYRP1	SPG7
PGLYRP1	HSPBP1
PGRMC1	SERBP1
PGRMC1	PLIN3
PGRMC1	SPCS2
PHB2	UBC
PHB2	UQCRC1
PHB2	HDAC1
PHB2	PECR
PHB2	ESR1
PHB2	PHB
PHF1	MAPT
PHF1	EZH2
PHF10	SMARCA4
PHF10	ACTL6A
PHF10	ARID1A
PHF10	SMARCC2
PHF10	SMARCE1
PHF10	SMARCA2
PHF10	SMARCB1
PHF10	SMARCC1
PHF10	SMARCD1
PHF10	DPF3
PHF10	ACTL6B
PHOX2B	ASCL1
PHOX2B	HAND2
PHOX2B	DBH
PIGS	PIGT
PIGS	GPAA1
PIGS	PIGO
PIGS	PLAUR
PIGS	PIGK
PIGS	PGAP1
PIGS	PIGU
PIGS	PIGF

PIGU	GPAA1
PIGU	PIGF
PIGU	PLAUR
PIGU	PIGK
PIGU	PIGO
PIGU	PIGT
PIGU	PGAP1
PIGU	PIGS
PIR	NCKAP1
PIR	PSMA7
PIR	RAC1
PIR	NCK1
PIR	ABL2
PIR	WASF2
PITPNA	PLCG1
PITPNA	DCC
PITPNA	NTN1
PMAIP1	POU4F1
PMAIP1	BAD
PMAIP1	E2F1
PMAIP1	BCL2L11
PMAIP1	MCL1
PMAIP1	BBC3
PMAIP1	BCL2
PMAIP1	MAX
PMAIP1	BCL2L1
PMAIP1	TP53
PMAIP1	BAK1
PMAIP1	MYC
PMAIP1	TFDP1
PMAIP1	UBC
PMAIP1	BID
PMVK	MVK
PMVK	NFYC
PMVK	SP1
PMVK	MVD
PMVK	NFYA
PMVK	SREBF2
PMVK	NFYB
POLG2	POLG
POLG2	GABPAP
POMGNT1	FKTN
POMGNT1	POMT1
POMT1	POMGNT1
POMT1	DAG1
PPIA	PPP3R1

PPIA	UBC
PPIA	BSG
PPIA	CAP1
PPIA	TAGLN2
PPIA	PPP3CB
PPIA	PFN1
PPIA	UBL5
PPIA	NFATC3
PPIA	MAPK1
PPIA	CFL1
PPIA	PPP3CA
PPP1R14B	NUBP2
PPP1R14B	ILK
PPP4R1	PPP4C
PPP4R1	PPP4R2
PPP4R3A	PPP4R2
PPP4R3A	PPP4C
PRDM10	TSPO
PRDM10	GAPDH
PRDM4	HDAC1
PRDM4	NGF
PRDM4	HDAC3
PRDM4	NGFR
PRDM4	SORT1
PRDM4	HDAC2
PRDX6	PARK7
PRDX6	PRDX2
PRDX6	PRDX1
PRDX6	PRDX3
PREP	SUCLG2
PREP	PDHB
PREP	APP
PRKAB2	PRKAG1
PRKAB2	PRKAG2
PRKAB2	PRKAA1
PRPF39	SNRNP70
PRPF39	SNRPD1
PRPF39	LUC7L2
PRPF39	TRNAU1AP
PRPF39	SNRPF
PRPF39	SF3A3
PRPF39	NCBP1
PRPF39	LUC7L
PRPF39	PRPF40A
PRPF39	RBM25
PRPF39	SNRPD3

PRPF39	SART1
PRPF39	SNRNP35
PRPF39	PRPF40B
PRPF39	ZMAT5
PRPF39	SNRPA1
PRPF39	SNRPC
PRPSAP1	PRPS1
PRPSAP1	PRPS2
PSAT1	PHGDH
PSAT1	PSPHP1
PSAT1	UBC
PSIP1	SCG2
PSIP1	SP1
PSIP1	EXOSC5
PSIP1	HSPA2
PSIP1	RBBP8
PSIP1	SRSF1
PTDSS1	PLA2G6
PTDSS1	PLA2G10
PTDSS1	CHPT1
PTDSS1	LCAT
PTDSS1	PLD1
PTDSS1	CEPT1
PTDSS1	PLA2G12A
PTDSS1	LPCAT2
PTDSS1	LPCAT1
PTDSS1	PLB1
PTDSS1	LPCAT4
PTDSS1	JMJD7-PLA2G4B
PTDSS1	PSD
PTDSS1	PNMT
PTDSS1	PLD3
PTDSS1	PLD4
PTDSS1	PLA2G4C
PTDSS1	PTDSS2
PTDSS1	PLD2
PTDSS1	PLA2G4B
PTMA	CREBBP
PTMA	SRSF2
PTMA	EP300
PTMA	ACTL6A
PTMA	MYC
PTMA	UBC
PTMA	RUVBL1
PTMA	PTMS
PTMA	KAT5

PTMA	RUVBL2
PTMA	MAX
PTPN23	PTK2
PTPN23	GRAP2
PTPN23	TSG101
PTPN23	CHMP4B
PTPN23	SRC
PTPN23	GRB2
PTPN23	UBC
PTPRS	NCAM1
PTPRS	PPFIA1
PTPRS	PPFIA3
PTPRS	AGRN
PTS	GCHFR
PTS	SPR
PTS	GCH1
PTS	ALPL
PURB	PURA
PURB	RFC3
PYCR1	P4HA1
PYCR1	ALDH18A1
PYCR1	ALDH4A1
PYCR1	P4HA3
PYCR1	P4HA2
PYCR1	LAP3
QPRT	NT5C1A
QPRT	ENPP3
QPRT	NT5E
QPRT	NMNAT3
QPRT	NT5C
QPRT	NMRK1
QPRT	NT5C2
QPRT	NMNAT2
QPRT	NT5M
QPRT	NUDT12
QPRT	POMP
QPRT	NMNAT1
RAB11FIP3	RAB11FIP5
RAB11FIP3	RAB25
RAB11FIP3	RAB11B
RAB11FIP3	PI4KB
RAB11FIP3	ARF5
RAB11FIP3	RAB11A
RAB11FIP3	ARF6
RAB21	RABGEF1
RAB21	ANKRD27

RAB23	SMO
RAB23	GLI2
RAB33A	STX5
RAB33A	ATG16L1
RAB35	UBC
RAB35	DENND1B
RAB3GAP2	UBC
RAB3GAP2	UNC13B
RAB3GAP2	VAMP2
RAB3GAP2	RIMS1
RAB3GAP2	RAB3GAP1
RAB3GAP2	SNAP25
RABEP1	GGA2
RABEP1	RABGEF1
RABEP1	RAB5B
RABEP1	GGA1
RABEP1	EEA1
RABEP1	AP1G1
RABEP1	RAB4A
RABEP1	GGA3
RABEP1	HUNK
RABEP1	PRKD1
RABEP1	RAB5A
RABEP2	RABGEF1
RABEP2	RAB5A
RACK1	PTPN11
RACK1	ELOB
RACK1	RPL19
RACK1	HABP4
RACK1	RPS27
RACK1	DNM1
RACK1	TYK2
RACK1	SDC2
RACK1	IFNAR1
RACK1	KRAS
RACK1	RPL24
RACK1	AGTRAP
RACK1	HK2
RACK1	ABL1
RACK1	RPL12
RACK1	RLIM
RACK1	RPS10-NUDT3
RACK1	MRPS5
RACK1	RPL3L
RACK1	RASA1
RACK1	HDLBP

RACK1	RPL38
RACK1	RPL13A
RACK1	RPL3
RACK1	RPL39L
RACK1	RPS11
RACK1	RPL18A
RACK1	RPL7
RACK1	RPS28
RACK1	RB1
RACK1	SLC9A3R1
RACK1	RPL15
RACK1	RPS5
RACK1	IGF1R
RACK1	RPS2
RACK1	RPLP0P6
RACK1	RPS15A
RACK1	RPL29
RACK1	RPS29
RACK1	PRKCE
RACK1	SLK
RACK1	EEF1G
RACK1	RSRC1
RACK1	RPL4
RACK1	KRBA2
RACK1	RPL14
RACK1	RPL35A
RACK1	RPL23A
RACK1	FAU
RACK1	RPL30
RACK1	RPS13
RACK1	RRP1
RACK1	RPL36A
RACK1	RPL5
RACK1	RPL36AL
RACK1	RPS17
RACK1	RPL18
RACK1	RPS25
RACK1	EIF6
RACK1	RPL21
RACK1	RPL11
RACK1	JAK1
RACK1	UBC
RACK1	SMPD2
RACK1	RPSA
RACK1	RPL8
RACK1	RPS3A

RACK1	RPS20
RACK1	RPS12
RACK1	EFTUD2
RACK1	RPS26
RACK1	GNB1
RACK1	RPL39
RACK1	SHC1
RACK1	RPS14
RACK1	ELOC
RACK1	RPS24
RACK1	RPL28
RACK1	TNFRSF1A
RACK1	HK3
RACK1	HIF1A
RACK1	RPL7L1
RACK1	RPL7A
RACK1	RPLP2
RACK1	RPS9
RACK1	RPL26L1
RACK1	RPL17
RACK1	NSMAF
RACK1	RPL9P7
RACK1	RPL23
RACK1	RPL27
RACK1	RPL35
RACK1	PDE4D
RACK1	RPS3
RACK1	RPS21
RACK1	RPL10
RACK1	MRPS7
RACK1	RPL6
RACK1	RPSAP58
RACK1	RPS15
RACK1	RPL37A
RACK1	STAT1
RACK1	RPS4X
RACK1	RPS27L
RACK1	PRKD1
RACK1	RPL17-C18orf32
RACK1	PLEC
RACK1	RPL13
RACK1	RPS8
RACK1	SRC
RACK1	RPS6
RACK1	RPS16
RACK1	RPL27A

RACK1	RPS18
RACK1	RPS7
RACK1	HSP90AA1
RACK1	SMPD3
RACK1	PRKCD
RACK1	SOS1
RACK1	GNG2
RACK1	GRB2
RACK1	RPL10A
RACK1	EEF2
RACK1	RPS23
RACK1	RPS19
RACK1	IFNAR2
RACK1	IRS1
RACK1	RPL31
RACK1	RPL36
RACK1	SERBP1
RACK1	MRPS2
RACK1	RPL32
RACK1	RPL26
RAP2A	TNIK
RAP2A	RAPGEF3
RAP2A	RAPGEF6
RAP2A	UBC
RAPGEF4	KCNJ11
RAPGEF4	GNAI3
RAPGEF4	PCLO
RAPGEF4	ABCC8
RAPGEF4	RAP1B
RAPGEF4	RAF1
RAPGEF4	RAPGEF3
RAPGEF4	RAP1GAP2
RAPGEF4	GNAS
RAPGEF4	SIPA1
RAPGEF4	RIMS2
RAPGEF4	UNC13A
RAPGEF4	RAP1GAP
RB1CC1	SMARCB1
RB1CC1	MTOR
RB1CC1	ULK2
RB1CC1	ATG5
RB1CC1	MLST8
RB1CC1	ULK1
RB1CC1	RPTOR
RB1CC1	ATG13
RB1CC1	ATG16L1

RB1CC1	ATG101
RB1CC1	PTK2B
RB1CC1	TP53
RBM10	GRIA1
RBM10	MAGOH
RBM8A	CASC3
RBM8A	EIF4A3
RBM8A	MAGOH
RBM8A	UPF3B
RCC2	RPS27
RCC2	KNL1
RCC2	KNTC1
RCC2	NDEL1
RCC2	PMF1-BGLAP
RCC2	EMB
RCC2	CENPC
RCC2	NUP37
RCC2	RANGAP1
RCC2	AURKC
RCC2	NDC80
RCC2	TAOK1
RCC2	CENPP
RCC2	CENPS
RCC2	KIF2C
RCC2	SEC13
RCC2	ZWILCH
RCC2	CENPN
RCC2	BUB1
RCC2	CENPK
RCC2	NUP107
RCC2	MAD2L1
RCC2	NUP43
RCC2	PAFAH1B1
RCC2	RAD21
RCC2	STAG2
RCC2	PLK1
RCC2	AURKB
RCC2	SKA2
RCC2	CLIP1
RCC2	CKAP5
RCC2	MAD1L1
RCC2	CENPM
RCC2	BUB1B
RCC2	CENPO
RCC2	NUDC
RCC2	KIF2A

RCC2	AHCTF1
RCC2	BUB3
RCC2	CDCA8
RCC2	SMC3
RCC2	NUF2
RCC2	MIS12
RCC2	B9D2
RCC2	NDE1
RCC2	CENPQ
RCC2	CENPI
RCC2	INCENP
RCC2	ITGB3BP
RCC2	ERCC6L
RCC2	CENPE
RCC2	STAG1
RCC2	NUP160
RCC2	MAPRE1
RCC2	PMF1
RCC2	ESPL1
RCC2	CENPF
RCC2	NSL1
RCC2	ZW10
RCC2	WAPL
RCC2	PPP1CC
RCC2	KIF18A
RCC2	CENPU
RCC2	DSN1
RCC2	SGO2
RCC2	MAST1
RCC2	CENPT
RCC2	CDC20
RCC2	ZWINT
RCC2	SPDL1
RCC2	UBC
RCC2	CENPA
RCC2	CENPL
RCC2	SGO1
RCC2	CLASP2
RCC2	SPC24
RCC2	NUP85
RCC2	CDCA5
RCC2	PDS5A
RCC2	BIRC5
RCC2	RGPD8
RCC2	PDS5B
RCC2	NUP133

RCN2	SFXN1
RCN2	PPP3CA
RCOR2	HDAC2
RCOR2	KDM1A
RDH11	RBP2
RDH11	UBC
RET	CREB1
RET	SHC1
RET	PTK2
RET	GRB10
RET	GRB2
RET	PTPN11
RET	CCDC6
RET	PIK3R1
RET	IRS1
RET	BCAR1
RET	UBC
RET	FRS2
RET	PLCG1
RET	CTNNA1
RET	MAPK1
RET	KRAS
RET	SOS1
RET	GFRA2
RET	STAT3
RET	ATF2
RET	CBLB
RET	DOK6
RET	CTNND1
RET	MAPK3
RET	GAB1
RET	CRK
RET	SRC
RET	PXN
RET	NCK1
RET	PSPN
RET	CDH1
RET	RASA1
RET	DOK4
RET	AKAP5
RET	PIK3CA
RET	SHANK3
RET	MAPK8
RET	GFRA1
RET	RAP1B
RET	CBL

RET	PDLIM7
RET	PRKCA
RET	PRKACA
RET	CTNNB1
RET	DOK1
RFNG	LHX2
RFNG	NOTCH3
RFNG	NOTCH1
RFNG	NOTCH2
RFNG	LHX9
RIMKLB	RIMKLA
RIMKLB	NAT8L
RING1	RYBP
RING1	E2F6
RNF14	TAGLN
RNF14	HNRNPA1
RNF216	UBC
RNF216	TLR4
RNF41	ERBB4
RNF41	BIRC6
RNF41	UBC
RNF41	UBE2D1
RNF41	MARK2
RNF41	ERBB3
RNF41	AKT1
RNF41	USP8
RNF41	PRKN
RNF41	ERBB2
RORB	NR2C2AP
RORB	NR2F2
RORB	NR2F6
RORB	NR1D1
RORB	VDR
RORB	RARB
RORB	NRBP1
RORB	ESRRG
RORB	RARA
RORB	ESR1
RORB	MED1
RORB	PPARD
RORB	NR1H2
RORB	THRA
RORB	NR2C2
RORB	RORC
RORB	HNF4G
RORB	ESR2

RORB	NR4A1
RORB	RORA
RORB	ESRRA
RORB	PPARA
RORB	NR2C1
RORB	ESRRB
RORB	THRB
RORB	RARG
RORB	NR1D2
RORB	PPARG
RORB	SF1
RPE	PGD
RPE	RPIA
RPE	TKT
RPE	XYLB
RPL10A	EIF3G
RPL10A	SRP19
RPL10A	SMG9
RPL10A	RPL32
RPL10A	RPS8
RPL10A	RPL9P7
RPL10A	RPL7A
RPL10A	SMG6
RPL10A	EIF5
RPL10A	RPL3L
RPL10A	RPL4
RPL10A	RPL26L1
RPL10A	MRTO4
RPL10A	CASC3
RPL10A	FAU
RPL10A	RPL18
RPL10A	RPLP2
RPL10A	RPS3
RPL10A	RPS2
RPL10A	UPF2
RPL10A	RPL13
RPL10A	EFL1
RPL10A	RPL27
RPL10A	RPL3
RPL10A	SMG1
RPL10A	UPF3A
RPL10A	SRP72
RPL10A	GFM2
RPL10A	RPL6
RPL10A	GFM1
RPL10A	EIF3D

RPL10A	MRPL15
RPL10A	RPL15
RPL10A	EEF1A1P5
RPL10A	RPN2
RPL10A	RPL22
RPL10A	RPLP0P6
RPL10A	EIF4B
RPL10A	RPS6
RPL10A	RPS27
RPL10A	RPS11
RPL10A	RPL39L
RPL10A	SEC11C
RPL10A	PPP2R2D
RPL10A	RPS10-NUDT3
RPL10A	RPL23A
RPL10A	RPL12
RPL10A	SEC11B
RPL10A	RPL7
RPL10A	MRPL33
RPL10A	RPL31
RPL10A	MRPL24
RPL10A	RPL14
RPL10A	RLIM
RPL10A	MRPL22
RPL10A	EIF3H
RPL10A	RPS26
RPL10A	EIF3I
RPL10A	ETF1
RPL10A	SEC61B
RPL10A	RPS5
RPL10A	RPL27A
RPL10A	WBP1
RPL10A	MRPL17
RPL10A	MRPS11
RPL10A	MRPS2
RPL10A	RPSA
RPL10A	SLC25A5
RPL10A	EIF3F
RPL10A	EIF3E
RPL10A	RPS19
RPL10A	RPL37A
RPL10A	RPS23
RPL10A	RRP1
RPL10A	SRP14
RPL10A	RPS16
RPL10A	EIF4A1

RPL10A	RPN1
RPL10A	RPL36A
RPL10A	RPS28
RPL10A	RPSAP58
RPL10A	RPL41
RPL10A	RPS20
RPL10A	RPL17
RPL10A	PPP2R1A
RPL10A	EEF2
RPL10A	RPL34
RPL10A	EIF2S3
RPL10A	RPL30
RPL10A	RPL7L1
RPL10A	MRPL12
RPL10A	MAGOH
RPL10A	NCBP1
RPL10A	RPL35A
RPL10A	MRPL11
RPL10A	EIF3A
RPL10A	MRPS7
RPL10A	SSR1
RPL10A	RPS15A
RPL10A	RPS29
RPL10A	EIF3J
RPL10A	SEC61A1
RPL10A	RPL13A
RPL10A	SSR2
RPL10A	RPL10
RPL10A	MRPL3
RPL10A	RPL24
RPL10A	TRAM1
RPL10A	RPL36
RPL10A	RNPS1
RPL10A	RPL11
RPL10A	RPS9
RPL10A	MRPL4
RPL10A	RPS15
RPL10A	RPL37
RPL10A	MRPS10
RPL10A	RPL5
RPL10A	RPL26
RPL10A	RPL39
RPL10A	RPS7
RPL10A	RPL28
RPL10A	RPL29
RPL10A	NCBP2

RPL10A	EIF4A2
RPL10A	PPP2CB
RPL10A	RPL35
RPL10A	RPL17-C18orf32
RPL10A	KRBA2
RPL10A	EIF3C
RPL10A	SSR3
RPL10A	EIF4G1
RPL10A	RPS12
RPL10A	EIF2S2
RPL10A	EIF3K
RPL10A	RPS3A
RPL10A	EIF1AX
RPL10A	EIF4A3
RPL10A	SPCS2
RPL10A	EIF2S1
RPL10A	IMP3
RPL10A	RPL38
RPL10A	RPL18A
RPL10A	SEC61G
RPL10A	UPF3B
RPL10A	EIF3B
RPL10A	RPS18
RPL10A	EIF4E
RPL10A	EFTUD2
RPL10A	MRPS9
RPL10A	SPCS3
RPL10A	SRP68
RPL10A	MRPS5
RPL10A	RPS27L
RPL10A	RPS24
RPL10A	RPS13
RPL10A	MRPL16
RPL10A	RPL8
RPL10A	SRP9
RPL10A	RPS21
RPL10A	RPL36AL
RPL10A	SRPRB
RPL10A	SRP54
RPL10A	RACK1
RPL10A	UBC
RPL10A	RPL22L1
RPL10A	RPS14
RPL10A	SMG7
RPL10A	RPS4X
RPL10A	RPL23

RPL10A	EIF5B
RPL10A	UPF1
RPL10A	SPCS1
RPL10A	RPL21
RPL10A	MRPL2
RPL10A	RPL19
RPL10A	SMG5
RPL10A	RPS17
RPL10A	SMG8
RPL13	RPL18A
RPL13	RPL36
RPL13	MRPL34
RPL13	ETF1
RPL13	EIF4B
RPL13	RPS20
RPL13	EIF5B
RPL13	SPCS1
RPL13	MRPS11
RPL13	RPL31
RPL13	RPS29
RPL13	RPL34
RPL13	SPCS3
RPL13	SRP54
RPL13	RPL14
RPL13	RPN1
RPL13	RPL3L
RPL13	RPS27
RPL13	EIF4G1
RPL13	EIF1AX
RPL13	RPS25
RPL13	MRPL49
RPL13	TP53I3
RPL13	PPP2R2D
RPL13	SSR3
RPL13	MRPS10
RPL13	UPF3B
RPL13	MRPL9
RPL13	RPL7A
RPL13	RPL10A
RPL13	MRPL55
RPL13	EIF3D
RPL13	MRPL22
RPL13	EIF3I
RPL13	RPS3A
RPL13	PPP2CB
RPL13	EIF3F

RPL13	RPS21
RPL13	MRPL15
RPL13	EIF6
RPL13	RPS6
RPL13	NCBP2
RPL13	WBP1
RPL13	MRPL16
RPL13	RPL37A
RPL13	RPS27L
RPL13	EIF4A1
RPL13	SMG5
RPL13	RPLP0P6
RPL13	RPS19
RPL13	EIF3B
RPL13	NCBP1
RPL13	RSRC1
RPL13	RPL15
RPL13	MRPS9
RPL13	RPL8
RPL13	SLC25A10
RPL13	MRPL33
RPL13	RPS24
RPL13	MRPL39
RPL13	EIF3K
RPL13	RPS12
RPL13	SRPRB
RPL13	MRPL42
RPL13	RPS9
RPL13	MRPL19
RPL13	TRAM1
RPL13	RPL30
RPL13	RPL4
RPL13	RPL24
RPL13	SRP19
RPL13	RPL21
RPL13	SEC11B
RPL13	WDR31
RPL13	RPS15A
RPL13	PPP2R1A
RPL13	RPL22L1
RPL13	RPLP2
RPL13	MRPL27
RPL13	RPL11
RPL13	MRPL44
RPL13	MRPS5
RPL13	MRTO4

RPL13	SEC11C
RPL13	MRPS7
RPL13	MRPS2
RPL13	EIF2S1
RPL13	MRPL32
RPL13	RPL27A
RPL13	RPS2
RPL13	RPL12
RPL13	RSL24D1
RPL13	MRPL4
RPL13	RPL35A
RPL13	RPS15
RPL13	SRP72
RPL13	RPL28
RPL13	RPS7
RPL13	RPS10-NUDT3
RPL13	EIF4E
RPL13	SSR1
RPL13	RNPS1
RPL13	RPL36AL
RPL13	MRPL12
RPL13	MRPL1
RPL13	SRP68
RPL13	RPL5
RPL13	RPL17-C18orf32
RPL13	SMG1
RPL13	KRBA2
RPL13	RPL9P7
RPL13	UPF2
RPL13	EIF2S2
RPL13	FAU
RPL13	RPN2
RPL13	EIF3G
RPL13	MRPL52
RPL13	RPL38
RPL13	CASC3
RPL13	RPL39L
RPL13	UBC
RPL13	IMP3
RPL13	MRPL3
RPL13	MRPL38
RPL13	RPS26
RPL13	RPL18
RPL13	MRPL11
RPL13	PLEC
RPL13	RPL19

RPL13	EIF4A2
RPL13	SSR2
RPL13	EIF4A3
RPL13	SMG7
RPL13	RPS13
RPL13	RPSA
RPL13	RPL23A
RPL13	RPL29
RPL13	EEF1G
RPL13	RPS28
RPL13	RPL41
RPL13	EIF3E
RPL13	RPL27
RPL13	SRP9
RPL13	RPS5
RPL13	EIF3H
RPL13	RPL7
RPL13	SRP14
RPL13	SPCS2
RPL13	MRPL20
RPL13	RPS8
RPL13	RPSAP58
RPL13	SMG9
RPL13	RPL35
RPL13	RPL26
RPL13	RPS11
RPL13	EEF1A1P5
RPL13	MAGOH
RPL13	MRPL47
RPL13	CUL7
RPL13	SEC61B
RPL13	EIF5
RPL13	EIF3C
RPL13	EIF3J
RPL13	RACK1
RPL13	UPF1
RPL13	RPL6
RPL13	RPL37
RPL13	SEC61G
RPL13	EEF2
RPL13	MRPL46
RPL13	GFM1
RPL13	RPL10
RPL13	RPL22
RPL13	RPL23
RPL13	RPL26L1

RPL13	RPL13A
RPL13	UPF3A
RPL13	RLIM
RPL13	RPS23
RPL13	SMG6
RPL13	SMG8
RPL13	EIF2S3
RPL13	MRPL2
RPL13	EIF3A
RPL13	RPS14
RPL13	RPS18
RPL13	RPL17
RPL13	RPS17
RPL13	MRPL40
RPL13	MRPL17
RPL13	RPL3
RPL13	RPL32
RPL13	RPL36A
RPL13	MRPL24
RPL13	GFM2
RPL13	RPS4X
RPL13	RPS3
RPL13	RPL39
RPL13	RRP1
RPL13	MRPL10
RPL13	RPS16
RPL13	RPL7L1
RPL13	MRPS15
RPL19	RPLP0P6
RPL19	RPL39
RPL19	EIF2S2
RPL19	RACK1
RPL19	RPS26
RPL19	SMG8
RPL19	EIF5B
RPL19	SMG9
RPL19	PPP2R1A
RPL19	RPSAP58
RPL19	FAU
RPL19	UBC
RPL19	RPL39L
RPL19	RPL18
RPL19	EIF3I
RPL19	RPL38
RPL19	RPS7
RPL19	NCBP1

RPL19	RPL31
RPL19	NIFK
RPL19	RPL22
RPL19	MRPS5
RPL19	RPL21
RPL19	SEC61G
RPL19	RPL4
RPL19	SSR1
RPL19	RPL23A
RPL19	RPL5
RPL19	RPL8
RPL19	MRPL3
RPL19	RPL14
RPL19	EEF2
RPL19	RPL37
RPL19	RPS16
RPL19	RPN1
RPL19	RPL27
RPL19	PPP2R2D
RPL19	SPCS3
RPL19	EIF5
RPL19	KRBA2
RPL19	RPS27L
RPL19	RPL37A
RPL19	EIF4G1
RPL19	WDR31
RPL19	RPL10
RPL19	EIF3J
RPL19	TRAM1
RPL19	EIF3F
RPL19	MAGOH
RPL19	RPS27
RPL19	RPL3L
RPL19	RPL26L1
RPL19	RPL11
RPL19	RPL12
RPL19	RPS13
RPL19	RPL7A
RPL19	RPL24
RPL19	PPP2CB
RPL19	EIF3C
RPL19	WBP1
RPL19	RPS24
RPL19	RPS23
RPL19	RPS14
RPL19	SPCS2

RPL19	RPS3A
RPL19	RPL7
RPL19	EIF4A1
RPL19	RPS19
RPL19	RPL23
RPL19	RPSA
RPL19	RPS20
RPL19	SEC11C
RPL19	UPF1
RPL19	RPS6
RPL19	SRP9
RPL19	RPS3
RPL19	RPL15
RPL19	RPN2
RPL19	EIF2S1
RPL19	RPL41
RPL19	RSRC1
RPL19	SRP72
RPL19	EIF3E
RPL19	SEC61B
RPL19	RPL3
RPL19	RPS4X
RPL19	RPS17
RPL19	SMG5
RPL19	MRPL22
RPL19	RPS11
RPL19	RPS29
RPL19	RPLP2
RPL19	SRP68
RPL19	SRP19
RPL19	RLIM
RPL19	SMG6
RPL19	RPS12
RPL19	RPL27A
RPL19	SEC11B
RPL19	RPS28
RPL19	RPL17-C18orf32
RPL19	RPL36A
RPL19	EIF2S3
RPL19	SSR3
RPL19	SMG1
RPL19	RPL35
RPL19	RPS21
RPL19	SRPRB
RPL19	RPL7L1
RPL19	RRP1

RPL19	RPL22L1
RPL19	RPS10-NUDT3
RPL19	RPL13
RPL19	RPS15A
RPL19	RPS9
RPL19	RSL24D1
RPL19	RPL32
RPL19	RPL26
RPL19	MRPL4
RPL19	RPS8
RPL19	RNPS1
RPL19	RPL36
RPL19	EIF3B
RPL19	NCBP2
RPL19	RPS5
RPL19	MRPL15
RPL19	EIF4A2
RPL19	SRP54
RPL19	RPL13A
RPL19	SMG7
RPL19	UPF3A
RPL19	MRPL11
RPL19	EIF1AX
RPL19	EIF4A3
RPL19	CASC3
RPL19	RPS18
RPL19	EIF3K
RPL19	RPL36AL
RPL19	RPS15
RPL19	RPL35A
RPL19	EIF3D
RPL19	RPL29
RPL19	EIF4E
RPL19	MRPL16
RPL19	EIF3H
RPL19	RPS25
RPL19	SSR2
RPL19	RPS2
RPL19	NOP2
RPL19	RPL17
RPL19	RPL9P7
RPL19	RPL34
RPL19	SNU13
RPL19	ETF1
RPL19	EIF4B
RPL19	EIF3A

RPL19	MRPL2
RPL19	EEF1A1P5
RPL19	SPCS1
RPL19	EIF3G
RPL19	UPF3B
RPL19	RPL6
RPL19	RPL18A
RPL19	RPL28
RPL19	SRP14
RPL19	RPL30
RPL19	MRPL24
RPL19	RPL10A
RPL19	UPF2
RPL27	RPL37
RPL27	RPL13A
RPL27	RPL22L1
RPL27	EIF4A3
RPL27	EIF3A
RPL27	RPL23
RPL27	RPL32
RPL27	RNPS1
RPL27	RPL18
RPL27	EEF1A1P5
RPL27	RPL8
RPL27	EIF5B
RPL27	RPS20
RPL27	SMG1
RPL27	RPN2
RPL27	SRP9
RPL27	EIF4G1
RPL27	RPL39L
RPL27	RPL34
RPL27	RPS6
RPL27	RPL41
RPL27	EIF5
RPL27	RPL19
RPL27	RPS28
RPL27	RPL5
RPL27	RPL24
RPL27	RPL10A
RPL27	SRP54
RPL27	RPL37A
RPL27	RSRC1
RPL27	MAGOH
RPL27	RPS11
RPL27	RPS5

RPL27	SMG6
RPL27	SRPRB
RPL27	RPL3L
RPL27	UBC
RPL27	RPL30
RPL27	EIF3B
RPL27	ETF1
RPL27	RPS21
RPL27	EIF3F
RPL27	SEC11C
RPL27	EIF4E
RPL27	RPS27
RPL27	WDR31
RPL27	RPL3
RPL27	RPL35
RPL27	RPL6
RPL27	RPS18
RPL27	EIF4A1
RPL27	RPS7
RPL27	RPL36A
RPL27	RPSA
RPL27	SMG8
RPL27	EEF2
RPL27	RPS3A
RPL27	SRP19
RPL27	SMG9
RPL27	TRAM1
RPL27	RPL7
RPL27	PPP2CB
RPL27	RPL14
RPL27	RPS23
RPL27	RPL23A
RPL27	PPP2R1A
RPL27	RPL15
RPL27	RPL31
RPL27	RPS3
RPL27	RPL36
RPL27	NCBP2
RPL27	EIF1AX
RPL27	SSR1
RPL27	RPS14
RPL27	SMG7
RPL27	RPL17-C18orf32
RPL27	RRP1
RPL27	RPS16
RPL27	RPL38

RPL27	EIF3H
RPL27	RPS2
RPL27	RPL11
RPL27	EIF3D
RPL27	RPS10-NUDT3
RPL27	RPS26
RPL27	KRBA2
RPL27	RPL10
RPL27	UPF1
RPL27	RPS25
RPL27	RPL22
RPL27	EIF4B
RPL27	RPS13
RPL27	RPL7L1
RPL27	RPS24
RPL27	RPL39
RPL27	EIF3G
RPL27	SPCS1
RPL27	RPL12
RPL27	EIF3C
RPL27	SEC61G
RPL27	EIF2S3
RPL27	RPS29
RPL27	EIF3J
RPL27	RPL21
RPL27	RACK1
RPL27	RPL17
RPL27	RPL7A
RPL27	RPLP0P6
RPL27	RPL26L1
RPL27	NCBP1
RPL27	EIF3K
RPL27	RPL13
RPL27	UPF3B
RPL27	RPS12
RPL27	RPS27L
RPL27	RPL35A
RPL27	RPS9
RPL27	RPL29
RPL27	RPL4
RPL27	SMG5
RPL27	EIF3E
RPL27	RPLP2
RPL27	RPS15A
RPL27	SSR2
RPL27	RPS19

RPL27	RPL28
RPL27	SEC61B
RPL27	WBP1
RPL27	RPS17
RPL27	RPS8
RPL27	RPL18A
RPL27	SEC11B
RPL27	RPL26
RPL27	EIF4A2
RPL27	SPCS2
RPL27	RPL9P7
RPL27	SRP14
RPL27	EIF2S1
RPL27	RPL36AL
RPL27	RPSAP58
RPL27	SPCS3
RPL27	RPN1
RPL27	UPF3A
RPL27	SRP72
RPL27	PPP2R2D
RPL27	EIF2S2
RPL27	CASC3
RPL27	RPS15
RPL27	SRP68
RPL27	RLIM
RPL27	RPL27A
RPL27	FAU
RPL27	UPF2
RPL27	SSR3
RPL27	EIF3I
RPL27	RPS4X
RPL3	RPS3A
RPL3	RPS28
RPL3	EEF2
RPL3	SMG6
RPL3	SRP68
RPL3	RPS15A
RPL3	RPL11
RPL3	POLR1E
RPL3	SRP9
RPL3	RPS13
RPL3	POLR2C
RPL3	RPSA
RPL3	LSG1
RPL3	RPN1
RPL3	RNPS1

RPL3	MRPL4
RPL3	SRPRB
RPL3	GFM2
RPL3	RPL7
RPL3	EFL1
RPL3	RPL22L1
RPL3	RPS27L
RPL3	RPS12
RPL3	RPL23
RPL3	RPL10
RPL3	RPS27
RPL3	EIF3J
RPL3	RRP1
RPL3	PPP2R1A
RPL3	EIF3E
RPL3	RPL3L
RPL3	RPL38
RPL3	RPL13A
RPL3	UBC
RPL3	EIF2S3
RPL3	RPL32
RPL3	RPL18A
RPL3	RPL10A
RPL3	MRPS11
RPL3	RPS29
RPL3	RACK1
RPL3	RPL23A
RPL3	RPL17-C18orf32
RPL3	PPP2R2D
RPL3	ETF1
RPL3	RPL24
RPL3	RPS23
RPL3	RPS25
RPL3	RPL5
RPL3	EIF3B
RPL3	RPSAP58
RPL3	EIF1AX
RPL3	SRP19
RPL3	RPL22
RPL3	RPS16
RPL3	RPL27
RPL3	EIF3G
RPL3	RPS21
RPL3	RPL4
RPL3	PPP2CB
RPL3	UPF1

RPL3	NCL
RPL3	EIF3D
RPL3	RPL6
RPL3	SMG5
RPL3	IMP3
RPL3	RPL8
RPL3	SRP54
RPL3	MRPL3
RPL3	SEC61G
RPL3	MRPS9
RPL3	RPL29
RPL3	EIF3F
RPL3	RPL37A
RPL3	RPL41
RPL3	RPS15
RPL3	RPL37
RPL3	EIF5
RPL3	RPLP2
RPL3	RPS7
RPL3	RPL19
RPL3	MRPS10
RPL3	EIF3K
RPL3	RPL12
RPL3	SMG8
RPL3	EIF4A1
RPL3	HSP90AA1
RPL3	TRAM1
RPL3	SMG9
RPL3	SEC61A1
RPL3	SPCS3
RPL3	RPL35A
RPL3	RPS10-NUDT3
RPL3	RPS8
RPL3	RPL36A
RPL3	MRTO4
RPL3	EIF3C
RPL3	MRPL15
RPL3	SEC11C
RPL3	FBL
RPL3	EIF4A3
RPL3	CASC3
RPL3	WDR31
RPL3	RPS3
RPL3	MAGOH
RPL3	NGB
RPL3	EEF1A1P5

RPL3	SPCS2
RPL3	RPS5
RPL3	MRPL22
RPL3	RPA2
RPL3	MRPL11
RPL3	RPN2
RPL3	MRPS2
RPL3	RPS6
RPL3	UPF3B
RPL3	RPS26
RPL3	RPS20
RPL3	RPL31
RPL3	RPL34
RPL3	RPL39L
RPL3	SSR3
RPL3	RPL26L1
RPL3	RPS14
RPL3	SRP72
RPL3	RPL27A
RPL3	RPL7A
RPL3	EIF3A
RPL3	SEC11B
RPL3	RPL36
RPL3	SMG7
RPL3	EFTUD2
RPL3	MRPL2
RPL3	RPL30
RPL3	RPL21
RPL3	MRPS14
RPL3	RPS18
RPL3	RPS17
RPL3	SRP14
RPL3	MRPL12
RPL3	SPCS1
RPL3	EIF3H
RPL3	UPF2
RPL3	RPLP0P6
RPL3	RSRC1
RPL3	RPL18
RPL3	RPS2
RPL3	RPL9P7
RPL3	RPL39
RPL3	NCBP2
RPL3	MRPL24
RPL3	SEC61B
RPL3	RPL17

RPL3	RPS4X
RPL3	MRPS5
RPL3	WBP1
RPL3	RPL7L1
RPL3	EIF3I
RPL3	SSR1
RPL3	EIF5B
RPL3	RSL24D1
RPL3	EIF4G1
RPL3	SSR2
RPL3	RPL36AL
RPL3	EIF4E
RPL3	MRPL16
RPL3	SMG1
RPL3	RPS9
RPL3	UPF3A
RPL3	MRPS7
RPL3	RPL28
RPL3	RPL26
RPL3	FAU
RPL3	EIF6
RPL3	RPL14
RPL3	RPS11
RPL3	EIF2S1
RPL3	EIF2S2
RPL3	RPL15
RPL3	RPL35
RPL3	EIF4B
RPL3	GRWD1
RPL3	RPL13
RPL3	KRBA2
RPL3	GFM1
RPL3	TUFM
RPL3	NCBP1
RPL3	MRPL1
RPL3	RLIM
RPL3	EIF4A2
RPL3	RPS19
RPL3	RPS24
RPL3	MRPL17
RPL35	RPL35A
RPL35	RPS12
RPL35	RPL31
RPL35	SEC61B
RPL35	RPL17-C18orf32
RPL35	MRPL33

RPL35	EIF3J
RPL35	EIF4B
RPL35	SEC61A2
RPL35	MRPS2
RPL35	MRPL32
RPL35	RRP1
RPL35	SEC11C
RPL35	RPL12
RPL35	SEC61A1
RPL35	UPF3A
RPL35	RPL5
RPL35	SMG9
RPL35	NCBP2
RPL35	SRP68
RPL35	MRPL2
RPL35	RPL30
RPL35	EIF3D
RPL35	MRPS14
RPL35	RPL41
RPL35	MRPL1
RPL35	RPN1
RPL35	RPS14
RPL35	RPL29
RPL35	WDR31
RPL35	SRP72
RPL35	EIF3F
RPL35	EEF1A1P5
RPL35	EIF3I
RPL35	RPS21
RPL35	MRPS10
RPL35	MRPS15
RPL35	MRPL36
RPL35	MRPL17
RPL35	MRPS5
RPL35	EIF3E
RPL35	IMP3
RPL35	RPL36
RPL35	PPP2CB
RPL35	RPL22L1
RPL35	MRPL3
RPL35	RPS16
RPL35	EIF4A2
RPL35	PPP2R2D
RPL35	RPS4X
RPL35	EIF1AX
RPL35	RLIM

RPL35	MRPL22
RPL35	SSR2
RPL35	RPL39L
RPL35	FAU
RPL35	RPS18
RPL35	RPL10
RPL35	RPL15
RPL35	MRTO4
RPL35	EIF3A
RPL35	MRPL11
RPL35	RPL27
RPL35	SPCS2
RPL35	RPL7
RPL35	SRP14
RPL35	EIF4A1
RPL35	RPS19
RPL35	RPL37A
RPL35	RPLP2
RPL35	EIF3H
RPL35	RPS6
RPL35	SPCS3
RPL35	EIF2S2
RPL35	EIF3K
RPL35	RPS9
RPL35	MRPS9
RPL35	RPL28
RPL35	RPS17
RPL35	UBC
RPL35	RPL8
RPL35	RSL24D1
RPL35	RPLP0P6
RPL35	KRBA2
RPL35	SRPRB
RPL35	RPS27L
RPL35	WBP1
RPL35	MRPL16
RPL35	RPL36A
RPL35	RPS27
RPL35	RPL17
RPL35	SRP9
RPL35	RPL3L
RPL35	RPL27A
RPL35	SMG5
RPL35	RPS28
RPL35	EIF3B
RPL35	RPL24

RPL35	RPL18
RPL35	RPL11
RPL35	RNPS1
RPL35	RPS3A
RPL35	PPP2R1A
RPL35	EIF5B
RPL35	RPS20
RPL35	MRPL24
RPL35	RPS3
RPL35	RPL19
RPL35	SMG7
RPL35	MRPS7
RPL35	RPL6
RPL35	EIF5
RPL35	SPCS1
RPL35	RPS13
RPL35	EIF3G
RPL35	RPL14
RPL35	RPS29
RPL35	SEC11B
RPL35	EIF3C
RPL35	SRP54
RPL35	SMG8
RPL35	RPL23
RPL35	MRPS11
RPL35	CASC3
RPL35	RPL39
RPL35	RPL7A
RPL35	UPF3B
RPL35	RPL26
RPL35	RACK1
RPL35	SMG1
RPL35	EFL1
RPL35	SSR3
RPL35	MRPL27
RPL35	GFM1
RPL35	RPL36AL
RPL35	RPS25
RPL35	ETF1
RPL35	RPL26L1
RPL35	GFM2
RPL35	EIF2S3
RPL35	EIF4E
RPL35	RPS10-NUDT3
RPL35	RPL4
RPL35	RPL38

RPL35	EIF4G1
RPL35	MRPL19
RPL35	RPL10A
RPL35	RPL13
RPL35	SMG6
RPL35	RPS24
RPL35	RSRC1
RPL35	RPN2
RPL35	RPL21
RPL35	RPS11
RPL35	MRPL20
RPL35	UPF2
RPL35	EFTUD2
RPL35	MRPL4
RPL35	RPSAP58
RPL35	RPS26
RPL35	MRPL15
RPL35	RPL7L1
RPL35	UPF1
RPL35	RPL13A
RPL35	MAGOH
RPL35	SSR1
RPL35	RPL34
RPL35	SRP19
RPL35	MRPL12
RPL35	RPS7
RPL35	RPSA
RPL35	NCBP1
RPL35	RPS23
RPL35	RPL9P7
RPL35	RPL23A
RPL35	RPS5
RPL35	RPL32
RPL35	RPS2
RPL35	RPL3
RPL35	RPL22
RPL35	EIF4A3
RPL35	RPS15A
RPL35	RPS8
RPL35	TRAM1
RPL35	RPL18A
RPL35	RPL37
RPL35	SEC61G
RPL35	RPS15
RPL35	EEF2
RPL35	EIF2S1

RPL36	UBC
RPL36	RPL13
RPL36	RPL13A
RPL36	RPL31
RPL36	RPL9P7
RPL36	RPL26L1
RPL36	SRP9
RPL36	RPS25
RPL36	RPL37A
RPL36	MRPL15
RPL36	EIF4G1
RPL36	RPL22
RPL36	EIF3E
RPL36	RPS18
RPL36	MRPL4
RPL36	EIF3G
RPL36	UPF2
RPL36	RPSAP58
RPL36	SRP14
RPL36	EIF2S3
RPL36	EIF3D
RPL36	SPCS1
RPL36	SMG8
RPL36	RPS20
RPL36	SMG6
RPL36	SMG9
RPL36	SEC61B
RPL36	EIF3H
RPL36	RPL35
RPL36	EEF2
RPL36	EIF4A2
RPL36	RPS26
RPL36	RPL11
RPL36	RPS10-NUDT3
RPL36	EIF2S2
RPL36	RPL17-C18orf32
RPL36	TRAM1
RPL36	RPS27L
RPL36	RSRC1
RPL36	UPF3B
RPL36	RNPS1
RPL36	SRP68
RPL36	RPLP2
RPL36	RPS27
RPL36	EIF3A
RPL36	RPL18

RPL36	UPF3A
RPL36	EIF5B
RPL36	RPL28
RPL36	RPL17
RPL36	RPS6
RPL36	RPS14
RPL36	RPL36AL
RPL36	RPL29
RPL36	RPS24
RPL36	RPL39
RPL36	RPS4X
RPL36	RPS9
RPL36	EEF1A1P5
RPL36	RPL7L1
RPL36	RPL35A
RPL36	EIF3B
RPL36	SRPRB
RPL36	ETF1
RPL36	RPS8
RPL36	RPL5
RPL36	RPL27
RPL36	RPL23A
RPL36	RPS16
RPL36	RRP1
RPL36	RPL24
RPL36	RPL41
RPL36	SEC11C
RPL36	PPP2R1A
RPL36	RPS15
RPL36	EIF3I
RPL36	RSL24D1
RPL36	RPS2
RPL36	RPL8
RPL36	FAU
RPL36	RPL21
RPL36	UPF1
RPL36	RPS28
RPL36	RPS23
RPL36	RPS12
RPL36	SEC11B
RPL36	RPL18A
RPL36	EIF3K
RPL36	RPL3L
RPL36	RPL7
RPL36	KRBA2
RPL36	SPCS2

RPL36	EIF5
RPL36	SSR3
RPL36	RPS17
RPL36	PPP2CB
RPL36	RPL10A
RPL36	SMG7
RPL36	RPL36A
RPL36	RPS29
RPL36	CASC3
RPL36	RPS11
RPL36	RPL32
RPL36	SRP54
RPL36	RPL19
RPL36	RPL22L1
RPL36	WDR31
RPL36	EIF4A3
RPL36	RPS15A
RPL36	RPL3
RPL36	SRP72
RPL36	SEC61G
RPL36	RPS3
RPL36	EIF3F
RPL36	EIF2S1
RPL36	MAGOH
RPL36	SPCS3
RPL36	SRP19
RPL36	RPS3A
RPL36	RPSA
RPL36	RPL14
RPL36	NCBP2
RPL36	RPL37
RPL36	RPS5
RPL36	EIF4E
RPL36	RPL26
RPL36	RPLP0P6
RPL36	WBP1
RPL36	RPL27A
RPL36	EIF3J
RPL36	RPL23
RPL36	RPS19
RPL36	NCBP1
RPL36	PPP2R2D
RPL36	RPS21
RPL36	RLIM
RPL36	RPL39L
RPL36	RPS13

RPL36	EIF4B
RPL36	RPL10
RPL36	RPL12
RPL36	RPL30
RPL36	EIF4A1
RPL36	MRPL24
RPL36	RPS7
RPL36	RPL15
RPL36	EIF3C
RPL36	RPL6
RPL36	SMG5
RPL36	RPL7A
RPL36	RPL4
RPL36	SSR2
RPL36	RPN2
RPL36	RPL34
RPL36	SSR1
RPL36	SMG1
RPL36	RPL38
RPL36	RACK1
RPL36	RPN1
RPL36	EIF1AX
RPL6	RPS11
RPL6	RPL29
RPL6	EIF3J
RPL6	SMG1
RPL6	RPL12
RPL6	SMG5
RPL6	RPL17-C18orf32
RPL6	SEC61B
RPL6	EIF2S3
RPL6	EIF4A2
RPL6	RPS3
RPL6	NCBP2
RPL6	RRP1
RPL6	RPS16
RPL6	RPS15
RPL6	SRPRB
RPL6	RPL39
RPL6	EIF4B
RPL6	RPLP2
RPL6	SSR3
RPL6	PPP2R2D
RPL6	RPS17
RPL6	EIF4E
RPL6	MRPS7

RPL6	RPL31
RPL6	RPL14
RPL6	SMG8
RPL6	EIF3E
RPL6	RPS6
RPL6	EIF4A1
RPL6	EIF4G1
RPL6	RPL7L1
RPL6	RPL10A
RPL6	RPS23
RPL6	SEC11C
RPL6	RPS18
RPL6	ETF1
RPL6	RPS19
RPL6	EIF3D
RPL6	UPF1
RPL6	UBC
RPL6	RPL39L
RPL6	RPL35A
RPL6	EIF1AX
RPL6	RPL5
RPL6	SRP19
RPL6	EIF2S2
RPL6	RPS14
RPL6	RPL27
RPL6	SRP9
RPL6	EIF3H
RPL6	FAU
RPL6	SSR1
RPL6	SPCS1
RPL6	SMG7
RPL6	RPL10
RPL6	WDR31
RPL6	RPL15
RPL6	EEF2
RPL6	KRBA2
RPL6	RPSAP58
RPL6	RPL17
RPL6	RPL3
RPL6	EIF3K
RPL6	UPF3B
RPL6	RNPS1
RPL6	RPL22
RPL6	RPS7
RPL6	RPS5
RPL6	RPL38

RPL6	NCBP1
RPL6	RPL21
RPL6	SEC11B
RPL6	SPCS3
RPL6	RPL4
RPL6	RPS21
RPL6	RPLP0P6
RPL6	RPL28
RPL6	RPL7A
RPL6	RPS15A
RPL6	SRP68
RPL6	EIF3B
RPL6	RPS29
RPL6	RPS20
RPL6	EIF5
RPL6	RPL8
RPL6	EEF1A1P5
RPL6	RPL9P7
RPL6	SMG9
RPL6	RPL37A
RPL6	EIF3G
RPL6	RPL27A
RPL6	RPL18A
RPL6	PPP2R1A
RPL6	RPL22L1
RPL6	RPS10-NUDT3
RPL6	RPN1
RPL6	RPS3A
RPL6	RPL18
RPL6	RPL36A
RPL6	RPS8
RPL6	RPL35
RPL6	RPL3L
RPL6	RPS24
RPL6	SRP54
RPL6	RPL37
RPL6	EIF3F
RPL6	RPS25
RPL6	RLIM
RPL6	RPL30
RPL6	RPL7
RPL6	RPL11
RPL6	RPL41
RPL6	RPS27L
RPL6	CASC3
RPL6	SPCS2

RPL6	RPL36AL
RPL6	TRAM1
RPL6	UPF2
RPL6	RACK1
RPL6	EEF1G
RPL6	EIF6
RPL6	EIF3I
RPL6	PPP2CB
RPL6	SSR2
RPL6	RPL26
RPL6	RPL23
RPL6	SMG6
RPL6	SRP72
RPL6	SRP14
RPL6	RPL26L1
RPL6	RPS9
RPL6	RPN2
RPL6	WBP1
RPL6	RPS26
RPL6	RPS12
RPL6	RPL13A
RPL6	RPL13
RPL6	RPL23A
RPL6	RPS13
RPL6	RPL32
RPL6	MAGOH
RPL6	SEC61G
RPL6	EIF3A
RPL6	EIF2S1
RPL6	EIF4A3
RPL6	RPL24
RPL6	RPS4X
RPL6	UPF3A
RPL6	RPS28
RPL6	RPL36
RPL6	EIF5B
RPL6	RPS2
RPL6	RPL19
RPL6	RPL34
RPL6	EIF3C
RPL6	RPS27
RPL6	RPSA
RPL8	SRP72
RPL8	EIF2S2
RPL8	EIF3H
RPL8	SSR3

RPL8	EIF3A
RPL8	RPS20
RPL8	CASC3
RPL8	RPL36AL
RPL8	RPL27
RPL8	RSL24D1
RPL8	RPS15
RPL8	UBC
RPL8	MRTO4
RPL8	EIF5
RPL8	RPL37A
RPL8	RPL19
RPL8	SEC11C
RPL8	RPL9P7
RPL8	SRPRB
RPL8	PLEC
RPL8	MRPS5
RPL8	UPF2
RPL8	RPL38
RPL8	MRPL27
RPL8	EIF2S1
RPL8	RPS10-NUDT3
RPL8	SRP9
RPL8	RPS17
RPL8	RPS18
RPL8	MRPS10
RPL8	IMP3
RPL8	NCBP2
RPL8	GFM1
RPL8	RPL7L1
RPL8	RPL22L1
RPL8	RPS16
RPL8	RPS7
RPL8	RPS28
RPL8	RPSAP58
RPL8	RPL13
RPL8	EIF3I
RPL8	RPL29
RPL8	RPL24
RPL8	RPS6
RPL8	RPS23
RPL8	RPL17
RPL8	SRP54
RPL8	RPN2
RPL8	RPS5
RPL8	MRPL36

RPL8	MRPL17
RPL8	RPL13A
RPL8	RPL18
RPL8	EIF3G
RPL8	MRPS9
RPL8	UPF1
RPL8	EFL1
RPL8	RPL3
RPL8	EIF4B
RPL8	WDR31
RPL8	RPL35
RPL8	RRP1
RPL8	EIF3D
RPL8	MRPL22
RPL8	GFM2
RPL8	SSR2
RPL8	RPN1
RPL8	RPL23A
RPL8	RPL26L1
RPL8	MRPS2
RPL8	MRPL24
RPL8	PPP2R1A
RPL8	RPSA
RPL8	RPS25
RPL8	EIF4A2
RPL8	SMG9
RPL8	TRAM1
RPL8	EIF4A1
RPL8	SMG8
RPL8	POLR1E
RPL8	RPL7A
RPL8	SPCS1
RPL8	RPL36A
RPL8	RPL12
RPL8	RPS29
RPL8	RPLP2
RPL8	RACK1
RPL8	FAU
RPL8	RPL21
RPL8	RPL6
RPL8	RPS3A
RPL8	RPL39
RPL8	MRPL15
RPL8	NCBP1
RPL8	MRPL4
RPL8	RPL36

RPL8	RLIM
RPL8	MRPL12
RPL8	MRPS7
RPL8	RPL30
RPL8	EIF4A3
RPL8	SMG7
RPL8	EIF3E
RPL8	RPL41
RPL8	SEC61G
RPL8	TUFM
RPL8	RPS12
RPL8	RPS14
RPL8	EIF4G1
RPL8	RPL23
RPL8	RPS11
RPL8	RPL22
RPL8	ETF1
RPL8	RPL37
RPL8	RPS26
RPL8	EFTUD2
RPL8	RPL39L
RPL8	PPP2CB
RPL8	RPL15
RPL8	SMG1
RPL8	RPS21
RPL8	RPL4
RPL8	SRP68
RPL8	RNPS1
RPL8	RPL32
RPL8	RPL35A
RPL8	MRPS14
RPL8	RPL27A
RPL8	RPL5
RPL8	SRP19
RPL8	SSR1
RPL8	WBP1
RPL8	EIF3C
RPL8	RPS3
RPL8	EEF1A1P5
RPL8	RPS4X
RPL8	KRBA2
RPL8	SEC61B
RPL8	RPL17-C18orf32
RPL8	RPS2
RPL8	PPP2R2D
RPL8	RSRC1

RPL8	EEF2
RPL8	RPLP0P6
RPL8	RPL31
RPL8	EIF3J
RPL8	RPS8
RPL8	EIF3F
RPL8	SPCS3
RPL8	SRP14
RPL8	UPF3A
RPL8	RPS9
RPL8	RPL28
RPL8	MRPL11
RPL8	RPS27
RPL8	EIF4E
RPL8	SEC11B
RPL8	EEF1G
RPL8	RPS27L
RPL8	RPL18A
RPL8	EIF5B
RPL8	RPL26
RPL8	UPF3B
RPL8	PA2G4
RPL8	MRPL3
RPL8	RPS13
RPL8	MRPL1
RPL8	RPS15A
RPL8	EIF3B
RPL8	SMG5
RPL8	POLR2C
RPL8	RPL10A
RPL8	EIF2S3
RPL8	RPL14
RPL8	RPL3L
RPL8	SPCS2
RPL8	SMG6
RPL8	RPL34
RPL8	RPL7
RPL8	MRPL16
RPL8	EIF1AX
RPL8	RPS24
RPL8	RPS19
RPL8	EIF3K
RPL8	RPL10
RPL8	RPL11
RPL8	MRPS11
RPL8	MAGOH

RPLP2	RPN1
RPLP2	RPL39
RPLP2	EEF1A1P5
RPLP2	SMG1
RPLP2	SSR1
RPLP2	RPSAP58
RPLP2	RPS14
RPLP2	SRP68
RPLP2	RPL17
RPLP2	RPL5
RPLP2	RPS29
RPLP2	EIF3C
RPLP2	EIF3K
RPLP2	RPL14
RPLP2	RPL41
RPLP2	RPL15
RPLP2	PPP2R2D
RPLP2	RPS13
RPLP2	RPL10A
RPLP2	RPN2
RPLP2	SEC61G
RPLP2	SPCS2
RPLP2	RPS15
RPLP2	RPL6
RPLP2	RPS25
RPLP2	RPL18A
RPLP2	SSR2
RPLP2	RPLP0P6
RPLP2	RPS6
RPLP2	SEC11C
RPLP2	EIF3A
RPLP2	RPS3A
RPLP2	RPS15A
RPLP2	RPL23
RPLP2	RPS19
RPLP2	RRP1
RPLP2	SRP14
RPLP2	RPS16
RPLP2	RPL36
RPLP2	RPL37
RPLP2	SMG9
RPLP2	SMG5
RPLP2	FAU
RPLP2	EIF3B
RPLP2	SMG6
RPLP2	RPL35

RPLP2	RPS8
RPLP2	RPL13
RPLP2	RPS24
RPLP2	RPL36AL
RPLP2	SRPRB
RPLP2	EIF4B
RPLP2	UPF3B
RPLP2	RPL27A
RPLP2	RPL26L1
RPLP2	SEC11B
RPLP2	RPL23A
RPLP2	RPL18
RPLP2	RPL12
RPLP2	RPS17
RPLP2	SMG8
RPLP2	RPL3
RPLP2	ETF1
RPLP2	RPS5
RPLP2	RPS11
RPLP2	RPL3L
RPLP2	RPL19
RPLP2	TRAM1
RPLP2	EIF4A3
RPLP2	EIF3I
RPLP2	RLIM
RPLP2	RPL24
RPLP2	UBC
RPLP2	RPL32
RPLP2	UPF1
RPLP2	RPL8
RPLP2	EIF3J
RPLP2	RPL31
RPLP2	EIF5
RPLP2	RPS3
RPLP2	RPL13A
RPLP2	EIF4A2
RPLP2	EIF2S1
RPLP2	RPS26
RPLP2	EEF2
RPLP2	PPP2R1A
RPLP2	SEC61B
RPLP2	EIF2S3
RPLP2	RPL35A
RPLP2	RPL39L
RPLP2	MAGOH
RPLP2	EIF4A1

RPLP2	SRP72
RPLP2	EIF2S2
RPLP2	RPS12
RPLP2	RACK1
RPLP2	EIF4G1
RPLP2	WDR31
RPLP2	KRBA2
RPLP2	RPL10
RPLP2	EIF3F
RPLP2	RPL29
RPLP2	SPCS3
RPLP2	SRP54
RPLP2	RPS23
RPLP2	RPL4
RPLP2	RPS28
RPLP2	RPL11
RPLP2	RPL28
RPLP2	RNPS1
RPLP2	RPS27L
RPLP2	SRP9
RPLP2	EIF4E
RPLP2	RPL9P7
RPLP2	EIF3H
RPLP2	SMG7
RPLP2	RPS18
RPLP2	UPF3A
RPLP2	RPS21
RPLP2	RPS27
RPLP2	EIF1AX
RPLP2	RPL37A
RPLP2	SPCS1
RPLP2	RPL30
RPLP2	CASC3
RPLP2	RPL27
RPLP2	NCBP1
RPLP2	RPL7A
RPLP2	RPL21
RPLP2	SRP19
RPLP2	RPS20
RPLP2	RPS9
RPLP2	NCBP2
RPLP2	RPL26
RPLP2	EIF5B
RPLP2	RPS4X
RPLP2	RPS10-NUDT3
RPLP2	RSRC1

RPLP2	RPL38
RPLP2	RPL36A
RPLP2	RPL22
RPLP2	RPS7
RPLP2	PPP2CB
RPLP2	RPL34
RPLP2	RPL7
RPLP2	EIF3D
RPLP2	EIF3E
RPLP2	EIF3G
RPLP2	SSR3
RPLP2	RPL17-C18orf32
RPLP2	RPS2
RPLP2	WBP1
RPLP2	UPF2
RPLP2	RPSA
RPP25	RPP14
RPP25	POP1
RPP25	POP4
RPP25	POP5
RPP25	RPP40
RPP25	RPP38
RPP25	POP7
RPS11	RPL6
RPS11	MRTO4
RPS11	RPL23
RPS11	RPS4X
RPS11	RPL41
RPS11	EIF4A3
RPS11	RPS26
RPS11	RPS27L
RPS11	EIF4B
RPS11	RPS29
RPS11	EIF3F
RPS11	RSRC1
RPS11	RPL23A
RPS11	RPL3L
RPS11	RPL36A
RPS11	RPL37
RPS11	MRPS2
RPS11	MRPS18A
RPS11	RPL17
RPS11	RPS28
RPS11	PLEC
RPS11	RPL30
RPS11	RPS12

RPS11	PPP2R1A
RPS11	SMG7
RPS11	GFM1
RPS11	EIF3A
RPS11	RPL18
RPS11	RPS10-NUDT3
RPS11	RRP1
RPS11	ETF1
RPS11	MRPS9
RPS11	RPL37A
RPS11	EIF3B
RPS11	RACK1
RPS11	EIF4A1
RPS11	RPL26L1
RPS11	RPL27
RPS11	GFM2
RPS11	RPL22
RPS11	RPL38
RPS11	RPL28
RPS11	RPS19
RPS11	RPL35A
RPS11	SEC11C
RPS11	WBP1
RPS11	MRPL3
RPS11	RPS6
RPS11	RPL15
RPS11	MRPL15
RPS11	RPL21
RPS11	EIF2S3
RPS11	SMG1
RPS11	EIF3D
RPS11	RPL10A
RPS11	FAU
RPS11	RPS16
RPS11	RPS5
RPS11	MRPS14
RPS11	MRPS16
RPS11	EIF1AX
RPS11	RPL39L
RPS11	RPL39
RPS11	RPS20
RPS11	SMG8
RPS11	SPCS1
RPS11	SRP9
RPS11	EIF4A2
RPS11	RPL7L1

RPS11	MRPL22
RPS11	UPF2
RPS11	RPS17
RPS11	MRPS5
RPS11	EIF4G1
RPS11	EIF3E
RPS11	SRP72
RPS11	RPS23
RPS11	EIF2S1
RPS11	EIF5B
RPS11	RPN2
RPS11	SRP54
RPS11	MRPS18C
RPS11	RPL7
RPS11	RPS21
RPS11	SRP14
RPS11	EFL1
RPS11	MRPS7
RPS11	EIF3K
RPS11	RPLP2
RPS11	SERBP1
RPS11	RPL19
RPS11	RPL27A
RPS11	EIF3J
RPS11	RPS24
RPS11	EIF2S2
RPS11	MRPS11
RPS11	RPL5
RPS11	SEC11B
RPS11	RPSA
RPS11	NCBP1
RPS11	EIF3H
RPS11	RPL17-C18orf32
RPS11	SMG9
RPS11	RPS25
RPS11	RNPS1
RPS11	SEC61B
RPS11	RPS13
RPS11	RPL11
RPS11	RPL14
RPS11	PPP2R2D
RPS11	MRPL16
RPS11	RPL10
RPS11	RPL8
RPS11	MRPS10
RPS11	RPN1

RPS11	SRP68
RPS11	WDR31
RPS11	RPL24
RPS11	MRPS6
RPS11	EIF3C
RPS11	UPF3A
RPS11	RPL7A
RPS11	FBL
RPS11	SMG5
RPS11	RPL26
RPS11	RPL36
RPS11	SRP19
RPS11	EIF3I
RPS11	MRPL4
RPS11	RPL29
RPS11	UPF1
RPS11	SPCS3
RPS11	SEC61G
RPS11	RPL36AL
RPS11	RPS9
RPS11	RPS7
RPS11	MRPL1
RPS11	MRPL24
RPS11	RPL12
RPS11	MRPS15
RPS11	RPS2
RPS11	MRPL11
RPS11	RPS3A
RPS11	RPL34
RPS11	UPF3B
RPS11	RPS14
RPS11	RPL13
RPS11	RPSAP58
RPS11	MRPL2
RPS11	TRAM1
RPS11	RPL13A
RPS11	RPL9P7
RPS11	TUFM
RPS11	RPL35
RPS11	SRPRB
RPS11	IMP3
RPS11	SPCS2
RPS11	PPP2CB
RPS11	RPL18A
RPS11	RPL4
RPS11	EIF3G

RPS11	RPS8
RPS11	CASC3
RPS11	RPL31
RPS11	NSA2
RPS11	SSR3
RPS11	EIF4E
RPS11	MAGOH
RPS11	UBC
RPS11	NCBP2
RPS11	EIF5
RPS11	SSR2
RPS11	RPLP0P6
RPS11	RPL3
RPS11	RPS27
RPS11	EFTUD2
RPS11	RPL32
RPS11	KRBA2
RPS11	EEF2
RPS11	SMG6
RPS11	RPS18
RPS11	RLIM
RPS11	NOP56
RPS11	RPS15
RPS11	RPS15A
RPS11	EEF1A1P5
RPS11	SSR1
RPS11	RPS3
RPS12	RPL35
RPS12	RPS18
RPS12	RPL26
RPS12	EIF3G
RPS12	MRPL1
RPS12	RPS27
RPS12	SSR1
RPS12	RPL38
RPS12	EIF3H
RPS12	MRPS2
RPS12	RPN2
RPS12	RPS27L
RPS12	RPS17
RPS12	EIF3D
RPS12	EIF1AX
RPS12	SRP72
RPS12	UPF1
RPS12	RPS3A
RPS12	SPCS1

RPS12	EIF3C
RPS12	RPL3
RPS12	EIF3K
RPS12	RPL14
RPS12	MRPL2
RPS12	RPS11
RPS12	RPL17
RPS12	MRPL22
RPS12	RPL15
RPS12	RPL3L
RPS12	SMG6
RPS12	SSR3
RPS12	RPL24
RPS12	EEF1A1P5
RPS12	RPS23
RPS12	RPL36AL
RPS12	RPL7L1
RPS12	IMP3
RPS12	SMG7
RPS12	POLR1E
RPS12	RPL7
RPS12	MRPS9
RPS12	SSR2
RPS12	SRP54
RPS12	RPL12
RPS12	PPP2R2D
RPS12	MRPS14
RPS12	MRPS10
RPS12	ETF1
RPS12	RPL13
RPS12	MRPL24
RPS12	EIF4A1
RPS12	RPS3
RPS12	RPLP0P6
RPS12	RPS2
RPS12	SEC11C
RPS12	EIF2S2
RPS12	TUFM
RPS12	SMG9
RPS12	WBP1
RPS12	EIF4B
RPS12	RPS29
RPS12	RPS5
RPS12	MRPS16
RPS12	RPL32
RPS12	MRPL4

RPS12	GFM2
RPS12	RPL37
RPS12	FAU
RPS12	RPL23
RPS12	EIF3I
RPS12	EIF2S1
RPS12	MRPS15
RPS12	MRTO4
RPS12	SRPRB
RPS12	EIF3B
RPS12	RPL35A
RPS12	EIF3J
RPS12	UPF3A
RPS12	SEC61G
RPS12	RPL27A
RPS12	RPS13
RPS12	PLEC
RPS12	RPS7
RPS12	SPCS2
RPS12	EIF4G1
RPS12	RPS8
RPS12	MRPL16
RPS12	CASC3
RPS12	RPS26
RPS12	RPL4
RPS12	PPP2R1A
RPS12	RPS6
RPS12	EIF2S3
RPS12	RPL39L
RPS12	RACK1
RPS12	RPL19
RPS12	EIF4A3
RPS12	RRP1
RPS12	RPSAP58
RPS12	RPL28
RPS12	SMG1
RPS12	RPS25
RPS12	EIF3F
RPS12	RPL29
RPS12	RPL18
RPS12	EIF3A
RPS12	MRPL3
RPS12	RPL8
RPS12	RLIM
RPS12	RPL22
RPS12	KRBA2

RPS12	RPL31
RPS12	EIF5
RPS12	EIF3E
RPS12	RPL36
RPS12	SMG8
RPS12	RPLP2
RPS12	RPS24
RPS12	RPS14
RPS12	EIF4A2
RPS12	RPL11
RPS12	MRPS7
RPS12	EEF2
RPS12	MRPS11
RPS12	RPL9P7
RPS12	GFM1
RPS12	RPL37A
RPS12	SRP9
RPS12	RPS20
RPS12	MRPL11
RPS12	RPS16
RPS12	WDR31
RPS12	RPS15A
RPS12	RPN1
RPS12	RPL26L1
RPS12	EFL1
RPS12	RPL13A
RPS12	RPS21
RPS12	RPL23A
RPS12	NCBP1
RPS12	RPL27
RPS12	SRP14
RPS12	RPS9
RPS12	RPL21
RPS12	SRP19
RPS12	SPCS3
RPS12	MRPS6
RPS12	RPL41
RPS12	NCBP2
RPS12	UBC
RPS12	RPSA
RPS12	RPL10A
RPS12	SEC61B
RPS12	EIF5B
RPS12	RPL10
RPS12	RPL6
RPS12	RPL34

RPS12	RPL36A
RPS12	RPS10-NUDT3
RPS12	PPP2CB
RPS12	RPL17-C18orf32
RPS12	RPL18A
RPS12	RPS28
RPS12	RPL30
RPS12	SEC11B
RPS12	RNPS1
RPS12	RPS15
RPS12	UPF2
RPS12	MRPL12
RPS12	EIF4E
RPS12	MRPS18C
RPS12	EFTUD2
RPS12	MRPS18A
RPS12	MAGOH
RPS12	SRP68
RPS12	RPL39
RPS12	SMG5
RPS12	UPF3B
RPS12	SERBP1
RPS12	MRPL15
RPS12	RPS4X
RPS12	RPS19
RPS12	MRPS5
RPS12	TRAM1
RPS12	RPL5
RPS15	EIF4G1
RPS15	PLEC
RPS15	RPS9
RPS15	MAGOH
RPS15	EIF3E
RPS15	SMG1
RPS15	RPS5
RPS15	RPL14
RPS15	MRPL17
RPS15	MRPS18A
RPS15	RPL7
RPS15	SPCS1
RPS15	WDR31
RPS15	RPL38
RPS15	RPN2
RPS15	RPL18
RPS15	RPL32
RPS15	PPP2CB

RPS15	NCBP1
RPS15	POLR1E
RPS15	RPL8
RPS15	RPS14
RPS15	MRPL16
RPS15	MRPL4
RPS15	EIF5B
RPS15	RPL6
RPS15	RPLP2
RPS15	MRPS18C
RPS15	RPL37
RPS15	RPL36AL
RPS15	FAU
RPS15	SMG8
RPS15	EFTUD2
RPS15	MRPL3
RPS15	RPS8
RPS15	TRAM1
RPS15	CASC3
RPS15	RPL10
RPS15	RPS25
RPS15	RPL31
RPS15	RPL7L1
RPS15	MRPL24
RPS15	EIF5
RPS15	UPF3A
RPS15	RPS7
RPS15	RPS28
RPS15	SEC61G
RPS15	EIF4B
RPS15	EIF3F
RPS15	ETF1
RPS15	RPL37A
RPS15	RLIM
RPS15	RPS2
RPS15	EIF4A1
RPS15	RPS27L
RPS15	RPL41
RPS15	MRPL1
RPS15	PPP2R2D
RPS15	RPL12
RPS15	MRPS16
RPS15	RPL30
RPS15	EEF1A1P5
RPS15	KRBA2
RPS15	SRP68

RPS15	RPS26
RPS15	RPL28
RPS15	SPCS2
RPS15	EIF4A2
RPS15	SRP19
RPS15	SPCS3
RPS15	GFM1
RPS15	MRPS2
RPS15	NCBP2
RPS15	WBP1
RPS15	RPL3
RPS15	POLR2C
RPS15	RPL13
RPS15	RPL18A
RPS15	RPS23
RPS15	EIF2S3
RPS15	MRPL15
RPS15	RPS19
RPS15	SSR2
RPS15	RPS27
RPS15	SRP72
RPS15	RPLP0P6
RPS15	RPL22
RPS15	SMG6
RPS15	RPL11
RPS15	EIF3A
RPS15	RPS3
RPS15	RPL15
RPS15	SMG7
RPS15	RPL36
RPS15	RPL24
RPS15	SSR1
RPS15	RPL21
RPS15	SMG5
RPS15	SEC61B
RPS15	RPS6
RPS15	PPP2R1A
RPS15	RPS10-NUDT3
RPS15	UBC
RPS15	RPL17
RPS15	MRPS10
RPS15	EIF3C
RPS15	RPL5
RPS15	MRPS15
RPS15	EIF1AX
RPS15	SRP9

RPS15	RNPS1
RPS15	RPN1
RPS15	EIF3I
RPS15	RPL13A
RPS15	ATP6V0A2
RPS15	SEC11B
RPS15	EIF3D
RPS15	RPS3A
RPS15	RPS24
RPS15	MRPL22
RPS15	IMP3
RPS15	SSR3
RPS15	EIF2S2
RPS15	RPS16
RPS15	RRP1
RPS15	MRPS14
RPS15	SRPRB
RPS15	RPL23
RPS15	SMG9
RPS15	MRPS11
RPS15	RPL10A
RPS15	MRPS6
RPS15	RSRC1
RPS15	RPSAP58
RPS15	RPL23A
RPS15	MRPL2
RPS15	RACK1
RPS15	MRTO4
RPS15	MRPS7
RPS15	SERBP1
RPS15	RPL26L1
RPS15	RPS20
RPS15	RPL7A
RPS15	SRP54
RPS15	RPL9P7
RPS15	MRPS5
RPS15	TUFM
RPS15	UPF1
RPS15	RPL17-C18orf32
RPS15	EIF3K
RPS15	RPSA
RPS15	RPS18
RPS15	RPL4
RPS15	RPL19
RPS15	EIF3J
RPS15	RPS29

RPS15	UPF3B
RPS15	SRP14
RPS15	EIF3H
RPS15	RPS17
RPS15	RPL29
RPS15	TPT1
RPS15	RPL3L
RPS15	RPL36A
RPS15	RPL39
RPS15	UPF2
RPS15	RPS12
RPS15	RPL35A
RPS15	EIF3B
RPS15	RPL26
RPS15	MRPS9
RPS15	NSA2
RPS15	RPS15A
RPS15	EIF3G
RPS15	EEF2
RPS15	SEC11C
RPS15	RPL39L
RPS15	EIF4E
RPS15	RPS13
RPS15	RPS4X
RPS15	RPL27
RPS15	GFM2
RPS15	EIF4A3
RPS15	RPL27A
RPS15	RPL35
RPS15	RPS21
RPS15	EIF2S1
RPS15	RPS11
RPS15	EFL1
RPS15	RPL34
RPS15	MRPL11
RPS16	RPS20
RPS16	MRPS18A
RPS16	RPS29
RPS16	EIF2S3
RPS16	RPS4X
RPS16	WDR31
RPS16	RLIM
RPS16	EIF3D
RPS16	RPL9P7
RPS16	RPS23
RPS16	RPL23

RPS16	SRP14
RPS16	KRBA2
RPS16	CASC3
RPS16	RPL41
RPS16	MRPL3
RPS16	RPL37
RPS16	RPSA
RPS16	RPL4
RPS16	RPL6
RPS16	RPL37A
RPS16	RPS7
RPS16	RPS9
RPS16	RPL35A
RPS16	RPSAP58
RPS16	RPL19
RPS16	SMG7
RPS16	RPL17-C18orf32
RPS16	RPS14
RPS16	EIF3F
RPS16	SRP54
RPS16	RPL35
RPS16	RPS27L
RPS16	MRPS14
RPS16	MRPL19
RPS16	RPS27
RPS16	EIF3J
RPS16	SMG6
RPS16	RPL36A
RPS16	RPS3
RPS16	RPLP0P6
RPS16	RPL8
RPS16	FAU
RPS16	SEC11C
RPS16	RPL18
RPS16	SPCS1
RPS16	PLEC
RPS16	SRPRB
RPS16	RPL38
RPS16	PPP2R2D
RPS16	SRP9
RPS16	EIF4E
RPS16	RPL5
RPS16	RPLP2
RPS16	RPL3
RPS16	RNPS1
RPS16	EIF4A2

RPS16	RPL26L1
RPS16	UPF1
RPS16	UPF2
RPS16	EEF2
RPS16	RPL27A
RPS16	RPS21
RPS16	RPS11
RPS16	TRAM1
RPS16	ETF1
RPS16	PPP2CB
RPS16	EIF3K
RPS16	UPF3B
RPS16	RPL3L
RPS16	RPS6
RPS16	RPL21
RPS16	MRPS16
RPS16	RPL14
RPS16	RPS18
RPS16	RRP1
RPS16	EIF5B
RPS16	NCBP1
RPS16	EIF2S2
RPS16	EIF3C
RPS16	RPL36AL
RPS16	RPL39L
RPS16	NCBP2
RPS16	SEC11B
RPS16	RPL36
RPS16	RPS5
RPS16	EIF4A1
RPS16	RPL10A
RPS16	RPS24
RPS16	EIF2S1
RPS16	RPL7L1
RPS16	EIF4G1
RPS16	RPL27
RPS16	MRPS2
RPS16	RPL10
RPS16	RPS3A
RPS16	RPL13A
RPS16	RPL7
RPS16	MRPL16
RPS16	MRPL22
RPS16	PPP2R1A
RPS16	RPL22
RPS16	MAGOH

RPS16	EIF5
RPS16	SEC61G
RPS16	SMG5
RPS16	MRPL4
RPS16	RPL29
RPS16	EIF3E
RPS16	RPL28
RPS16	UPF3A
RPS16	MRPL1
RPS16	RPS15
RPS16	MRPL15
RPS16	WBP1
RPS16	EIF3H
RPS16	SEC61B
RPS16	EIF4B
RPS16	MRPS11
RPS16	RPN2
RPS16	RPS26
RPS16	RPS12
RPS16	RPS28
RPS16	RPL18A
RPS16	MRPS7
RPS16	IMP3
RPS16	RPL17
RPS16	RPL12
RPS16	RPS10-NUDT3
RPS16	RPS17
RPS16	RPN1
RPS16	SMG8
RPS16	RPL26
RPS16	SPCS2
RPS16	MRPS18C
RPS16	SRP19
RPS16	EIF3I
RPS16	RSRC1
RPS16	MRPS5
RPS16	RPS2
RPS16	MRPL17
RPS16	SSR3
RPS16	RPL7A
RPS16	EIF3G
RPS16	SERBP1
RPS16	RPL24
RPS16	EIF4A3
RPS16	RPL23A
RPS16	RPL34

RPS16	SSR2
RPS16	RACK1
RPS16	RPS19
RPS16	RPS8
RPS16	MRTO4
RPS16	SSR1
RPS16	RPL11
RPS16	MRPS15
RPS16	RPL32
RPS16	SRP72
RPS16	SMG1
RPS16	EEF1A1P5
RPS16	MRPS10
RPS16	UBC
RPS16	TUFM
RPS16	RPL15
RPS16	SMG9
RPS16	RPL30
RPS16	EIF3B
RPS16	MRPL2
RPS16	MRPS6
RPS16	RPL31
RPS16	SPCS3
RPS16	EIF3A
RPS16	RPS25
RPS16	EIF1AX
RPS16	SRP68
RPS16	RPL13
RPS16	RPS13
RPS16	RPL39
RPS16	RPS15A
RPS19	RPL3L
RPS19	RPL37A
RPS19	EIF3I
RPS19	SPCS2
RPS19	SRPRB
RPS19	RPL39
RPS19	KRBA2
RPS19	RPL26L1
RPS19	EIF3B
RPS19	RPS29
RPS19	RPS8
RPS19	SRP68
RPS19	RPL35A
RPS19	SMG5
RPS19	RPS27

RPS19	RPS21
RPS19	RPL24
RPS19	EIF4A2
RPS19	EIF3F
RPS19	ETF1
RPS19	RPL31
RPS19	SMG1
RPS19	RPL39L
RPS19	SERBP1
RPS19	RPL17-C18orf32
RPS19	RPS13
RPS19	EIF4B
RPS19	RPSA
RPS19	RPL38
RPS19	EIF4G1
RPS19	RPL30
RPS19	EIF4A3
RPS19	MAGOH
RPS19	RPS15A
RPS19	RPS2
RPS19	EIF2S1
RPS19	NSA2
RPS19	RPL9P7
RPS19	RPS7
RPS19	EIF3G
RPS19	WDR31
RPS19	RPN2
RPS19	RPL6
RPS19	RPL15
RPS19	RPS11
RPS19	RPS17
RPS19	RPL4
RPS19	RSRC1
RPS19	RPL13
RPS19	PPP2R1A
RPS19	RPS9
RPS19	MRPS2
RPS19	RPS4X
RPS19	RPL32
RPS19	NCBP2
RPS19	RPS14
RPS19	RPL28
RPS19	RPLP2
RPS19	SRP19
RPS19	RPL18
RPS19	RPL35

RPS19	CASC3
RPS19	MRPS15
RPS19	SPCS1
RPS19	RPL21
RPS19	RPL7
RPS19	EIF3E
RPS19	SMG7
RPS19	RPL13A
RPS19	TRAM1
RPS19	RPL41
RPS19	SRP9
RPS19	RPL19
RPS19	RPL5
RPS19	EIF5
RPS19	SMG8
RPS19	RPS27L
RPS19	RPL29
RPS19	EEF1A1P5
RPS19	RPL7L1
RPS19	SRP54
RPS19	SSR3
RPS19	MRPS10
RPS19	RPL10A
RPS19	EIF4A1
RPS19	RPS15
RPS19	RPS23
RPS19	RPS19BP1
RPS19	RPL23
RPS19	RPS24
RPS19	EIF3H
RPS19	RPL10
RPS19	EIF4E
RPS19	EIF3J
RPS19	SMG9
RPS19	MRPS5
RPS19	SEC61B
RPS19	RPS20
RPS19	RRP1
RPS19	EIF3K
RPS19	RPS3
RPS19	PLEC
RPS19	RPL12
RPS19	RPL36A
RPS19	RPS3A
RPS19	PPP2R2D
RPS19	EEF2

RPS19	UPF3A
RPS19	RPL17
RPS19	EIF3C
RPS19	EIF2S3
RPS19	RNPS1
RPS19	RPS5
RPS19	RPS10-NUDT3
RPS19	EIF5B
RPS19	MRPS9
RPS19	RPS28
RPS19	SPCS3
RPS19	SEC61G
RPS19	SEC11B
RPS19	RPL14
RPS19	UPF3B
RPS19	FGF2
RPS19	RPS18
RPS19	SRP72
RPS19	MRPS11
RPS19	RPL22
RPS19	RPLP0P6
RPS19	RPL36
RPS19	RPL27A
RPS19	RPL26
RPS19	RPSAP58
RPS19	RPL27
RPS19	SMG6
RPS19	SSR1
RPS19	EIF3A
RPS19	RPS26
RPS19	RLIM
RPS19	SRP14
RPS19	MRPS14
RPS19	RPS16
RPS19	RPL36AL
RPS19	SEC11C
RPS19	UPF2
RPS19	RPN1
RPS19	WBP1
RPS19	RPL18A
RPS19	SLC25A5
RPS19	UPF1
RPS19	UBC
RPS19	PPP2CB
RPS19	RPL23A
RPS19	EIF3D

RPS19	EIF1AX
RPS19	RPL34
RPS19	RPL37
RPS19	SSR2
RPS19	RPS6
RPS19	RPL11
RPS19	RPS25
RPS19	EIF2S2
RPS19	NCBP1
RPS19	FAU
RPS19	RPL8
RPS19	RACK1
RPS19	MRPS7
RPS19	RPS12
RPS19	RPL7A
RPS19	RPL3
RPS2	EIF4A3
RPS2	RPL23
RPS2	EIF3K
RPS2	EIF3J
RPS2	SPCS1
RPS2	RPS17
RPS2	CDK9
RPS2	MRPL2
RPS2	RPL3L
RPS2	PPP2CB
RPS2	RPL15
RPS2	RPL39
RPS2	EIF3B
RPS2	POLR1E
RPS2	RPL21
RPS2	MRPS6
RPS2	MRPS18A
RPS2	MRPL16
RPS2	RPS20
RPS2	RPS4X
RPS2	MRPS18C
RPS2	MRPL1
RPS2	UPF3B
RPS2	RPL12
RPS2	SMG1
RPS2	RPL30
RPS2	RPL10A
RPS2	TRAM1
RPS2	SPCS2
RPS2	RPS28

RPS2	RPS9
RPS2	GFM2
RPS2	FAU
RPS2	SEC11C
RPS2	RPS19
RPS2	EIF3G
RPS2	RPS23
RPS2	RPS14
RPS2	SNU13
RPS2	EIF4A2
RPS2	KRBA2
RPS2	EFL1
RPS2	NIFK
RPS2	MRPS15
RPS2	MRPS9
RPS2	POLR2C
RPS2	RPS27L
RPS2	TPT1
RPS2	SMG6
RPS2	MRPS7
RPS2	POLR2A
RPS2	SRPRB
RPS2	RPS15
RPS2	RPL29
RPS2	RACK1
RPS2	RPS12
RPS2	SRP68
RPS2	RPS8
RPS2	RPL7A
RPS2	RPS26
RPS2	RPSA
RPS2	EIF5B
RPS2	PPP2R1A
RPS2	FBL
RPS2	RSRC1
RPS2	RPL24
RPS2	RPL9P7
RPS2	EIF3D
RPS2	RPL10
RPS2	MRPS2
RPS2	EIF4E
RPS2	MRPS10
RPS2	RPL37
RPS2	WDR31
RPS2	RPL31
RPS2	EEF2

RPS2	RPS18
RPS2	NSA2
RPS2	RPL4
RPS2	RPL36AL
RPS2	RPL7L1
RPS2	RPL13
RPS2	EIF3C
RPS2	RPS7
RPS2	EIF5
RPS2	EIF2S1
RPS2	EIF3H
RPS2	RNPS1
RPS2	SEC61A1
RPS2	SRP19
RPS2	MRPL15
RPS2	RPL37A
RPS2	SEC11B
RPS2	RPL38
RPS2	MRTO4
RPS2	RPS25
RPS2	SRP72
RPS2	RPL5
RPS2	RPS5
RPS2	MRPS11
RPS2	MRPL11
RPS2	EIF4A1
RPS2	SPCS3
RPS2	SEC61G
RPS2	SMG9
RPS2	RPL36
RPS2	RPL27
RPS2	CASC3
RPS2	RPN1
RPS2	MRPL24
RPS2	NCBP2
RPS2	UPF2
RPS2	RPS10-NUDT3
RPS2	SSR3
RPS2	EIF3E
RPS2	PRMT3
RPS2	MRPL3
RPS2	MAGOH
RPS2	RRP1
RPS2	RPL13A
RPS2	MRPL22
RPS2	RPL34

RPS2	RPL11
RPS2	RPS3
RPS2	TSFM
RPS2	RLIM
RPS2	ETF1
RPS2	RPL36A
RPS2	RPS24
RPS2	EIF3A
RPS2	RPN2
RPS2	RPS15A
RPS2	MRPL4
RPS2	IMP3
RPS2	RPS6
RPS2	SMG8
RPS2	RPL26
RPS2	EIF2S3
RPS2	NCBP1
RPS2	RPL17
RPS2	SRP54
RPS2	SSR2
RPS2	UBC
RPS2	RPS11
RPS2	RPL8
RPS2	RPL23A
RPS2	EIF1AX
RPS2	RPSAP58
RPS2	RPL26L1
RPS2	SEC61B
RPS2	SRP14
RPS2	RPL14
RPS2	RPL28
RPS2	MRPL12
RPS2	RPL7
RPS2	RPL3
RPS2	PPP2R2D
RPS2	EFTUD2
RPS2	SMG5
RPS2	RPS16
RPS2	RPLP0P6
RPS2	EIF4B
RPS2	RPL18
RPS2	SEC61A2
RPS2	RPS21
RPS2	RPS27
RPS2	EIF4G1
RPS2	PLEC

RPS2	MRPL17
RPS2	RPL18A
RPS2	SERBP1
RPS2	TUFM
RPS2	RPS3A
RPS2	RPL19
RPS2	EIF3F
RPS2	MRPS14
RPS2	RPL35A
RPS2	RPL32
RPS2	RPS29
RPS2	GFM1
RPS2	EIF2S2
RPS2	WBP1
RPS2	SMG7
RPS2	RPL41
RPS2	MRPS16
RPS2	RPL35
RPS2	RPS13
RPS2	RPL22
RPS2	SRP9
RPS2	UPF1
RPS2	EIF3I
RPS2	EEF1A1P5
RPS2	RPL6
RPS2	RPLP2
RPS2	RPL17-C18orf32
RPS2	UPF3A
RPS2	SSR1
RPS2	RPL27A
RPS20	RPS16
RPS20	RPL5
RPS20	MRPL22
RPS20	SSR3
RPS20	RSRC1
RPS20	RPL13
RPS20	RPL8
RPS20	RPL12
RPS20	RPL7A
RPS20	RPL39L
RPS20	MRPS11
RPS20	MRPL17
RPS20	EEF1A1P5
RPS20	RPL18A
RPS20	RPL27
RPS20	RPS2

RPS20	RPL3L
RPS20	RPL38
RPS20	RPS25
RPS20	RPL14
RPS20	RPL28
RPS20	RNPS1
RPS20	RPL36
RPS20	EIF3I
RPS20	UBC
RPS20	PPP2CB
RPS20	EFTUD2
RPS20	RPS5
RPS20	RPL17-C18orf32
RPS20	EIF1AX
RPS20	RPL23
RPS20	SERBP1
RPS20	RPL11
RPS20	NCBP2
RPS20	EIF4A2
RPS20	RPL10
RPS20	MRPS16
RPS20	EIF2S1
RPS20	MRPL11
RPS20	RPS18
RPS20	MRPS6
RPS20	RPL30
RPS20	MRPS7
RPS20	RPL32
RPS20	RPL24
RPS20	RPL29
RPS20	MRPL4
RPS20	EIF2S3
RPS20	RPL4
RPS20	SRP68
RPS20	EIF3J
RPS20	WBP1
RPS20	RPL37A
RPS20	RPN1
RPS20	MRPL3
RPS20	SMG1
RPS20	MAGOH
RPS20	RPL37
RPS20	SSR1
RPS20	SPCS3
RPS20	RPL26
RPS20	EIF3G

RPS20	RPL22
RPS20	IMP3
RPS20	RPL13A
RPS20	EIF3C
RPS20	RPL36AL
RPS20	RPL26L1
RPS20	RPL9P7
RPS20	RPS7
RPS20	RPL27A
RPS20	RPS11
RPS20	RPS26
RPS20	EIF4A3
RPS20	EIF3B
RPS20	RPL19
RPS20	MRPL2
RPS20	SMG7
RPS20	EIF2S2
RPS20	MRPS9
RPS20	EIF5B
RPS20	EIF4G1
RPS20	SEC61A1
RPS20	SRP72
RPS20	EIF3K
RPS20	SSR2
RPS20	RPS21
RPS20	EIF4E
RPS20	RPL41
RPS20	MRPS18A
RPS20	SRP54
RPS20	SMG9
RPS20	RPL10A
RPS20	TUFM
RPS20	MRPS5
RPS20	RPL6
RPS20	RPL21
RPS20	FAU
RPS20	RACK1
RPS20	RPSA
RPS20	RPL35
RPS20	SRP19
RPS20	POLR1E
RPS20	RPS19
RPS20	RPS4X
RPS20	RPSAP58
RPS20	RPL7L1
RPS20	RPS10-NUDT3

RPS20	KRBA2
RPS20	MRPL16
RPS20	WDR31
RPS20	GFM2
RPS20	MRPS18C
RPS20	EIF3A
RPS20	MRPL15
RPS20	RPL3
RPS20	EIF3F
RPS20	UPF3B
RPS20	RPS14
RPS20	RPL31
RPS20	MRTO4
RPS20	RPS12
RPS20	UPF2
RPS20	RPS28
RPS20	RPS6
RPS20	GFM1
RPS20	SMG5
RPS20	MRPS2
RPS20	RPS15
RPS20	RPS8
RPS20	RPL23A
RPS20	SRP9
RPS20	MRPS14
RPS20	RPL35A
RPS20	RLIM
RPS20	SRPRB
RPS20	RPL39
RPS20	POLR2C
RPS20	RPS9
RPS20	EIF3H
RPS20	SPCS2
RPS20	RPL36A
RPS20	SEC11C
RPS20	EIF5
RPS20	EIF4B
RPS20	SPCS1
RPS20	ETF1
RPS20	TRAM1
RPS20	SEC11B
RPS20	RPS15A
RPS20	SRP14
RPS20	RPLP2
RPS20	UPF3A
RPS20	PPP2R1A

RPS20	SEC61G
RPS20	RPL7
RPS20	MRPL24
RPS20	RPS27
RPS20	RPN2
RPS20	PLEC
RPS20	EIF3E
RPS20	NCBP1
RPS20	SEC61B
RPS20	RPS17
RPS20	EIF3D
RPS20	RPL15
RPS20	RPS27L
RPS20	RPS24
RPS20	SMG6
RPS20	RPS3A
RPS20	MRPL1
RPS20	EFL1
RPS20	SMG8
RPS20	RPS3
RPS20	EEF2
RPS20	RPL18
RPS20	RPS13
RPS20	EIF4A1
RPS20	PPP2R2D
RPS20	RPS23
RPS20	RRP1
RPS20	RPL34
RPS20	CASC3
RPS20	UPF1
RPS20	MRPS15
RPS20	RPS29
RPS20	RPLP0P6
RPS20	RPL17
RPS21	SSR3
RPS21	PPP2R1A
RPS21	EIF3H
RPS21	EIF3I
RPS21	RPL12
RPS21	RPL18
RPS21	RPL39
RPS21	RPL36A
RPS21	EIF3B
RPS21	PPP2R2D
RPS21	RPL32
RPS21	EIF3A

RPS21	RPS19
RPS21	RPLP0P6
RPS21	RPL7
RPS21	EIF3K
RPS21	RPL35A
RPS21	SMG8
RPS21	RPSA
RPS21	RPS9
RPS21	RPS5
RPS21	EIF3C
RPS21	RPS27
RPS21	SEC11B
RPS21	NCBP1
RPS21	RPL35
RPS21	RPL17
RPS21	EIF3D
RPS21	EIF5
RPS21	EIF3G
RPS21	SPCS1
RPS21	RPL38
RPS21	SMG1
RPS21	EIF2S2
RPS21	SEC61G
RPS21	UPF2
RPS21	EIF4A3
RPS21	WDR31
RPS21	RPL13
RPS21	RPS8
RPS21	EIF4A1
RPS21	EIF3J
RPS21	SSR1
RPS21	SRP19
RPS21	RPN2
RPS21	RPL37A
RPS21	RPL27
RPS21	MRPS7
RPS21	RPL27A
RPS21	KRBA2
RPS21	RPL26
RPS21	UPF1
RPS21	RPS26
RPS21	RPL3
RPS21	RRP1
RPS21	RPS16
RPS21	RPL3L
RPS21	RPL18A

RPS21	ETF1
RPS21	PLEC
RPS21	RNPS1
RPS21	RPL24
RPS21	RPL21
RPS21	RPSAP58
RPS21	MRPS14
RPS21	RPS10-NUDT3
RPS21	RPL34
RPS21	RPS15A
RPS21	EIF4B
RPS21	MAGOH
RPS21	SMG6
RPS21	RPL9P7
RPS21	FAU
RPS21	RPS25
RPS21	SRP68
RPS21	WBP1
RPS21	RPS11
RPS21	SMG5
RPS21	RPS4X
RPS21	RPL29
RPS21	RPL6
RPS21	RPS20
RPS21	UBC
RPS21	RPS14
RPS21	RPL23
RPS21	RPS6
RPS21	RPS28
RPS21	RPL7A
RPS21	RPS7
RPS21	RPL22
RPS21	RPL26L1
RPS21	RPS23
RPS21	RPL28
RPS21	RPL10
RPS21	SEC11C
RPS21	SRP54
RPS21	EIF3F
RPS21	SSR2
RPS21	RPS18
RPS21	RPL19
RPS21	RPS24
RPS21	RPS27L
RPS21	RPL13A
RPS21	RPL30

RPS21	EIF2S3
RPS21	SMG9
RPS21	RPL41
RPS21	CASC3
RPS21	PPP2CB
RPS21	RPL31
RPS21	RPL8
RPS21	RPL23A
RPS21	EIF1AX
RPS21	RPS13
RPS21	EIF2S1
RPS21	UPF3B
RPS21	SRP14
RPS21	SERBP1
RPS21	RACK1
RPS21	RPS12
RPS21	MRPS9
RPS21	SRPRB
RPS21	RPS29
RPS21	EIF5B
RPS21	MRPS11
RPS21	UPF3A
RPS21	RPS17
RPS21	RPLP2
RPS21	EIF4E
RPS21	RPL36AL
RPS21	RPL36
RPS21	EIF3E
RPS21	SRP9
RPS21	RPS2
RPS21	RLIM
RPS21	SMG7
RPS21	RPN1
RPS21	EEF2
RPS21	RPL17-C18orf32
RPS21	RPS3
RPS21	EIF4A2
RPS21	RPL39L
RPS21	RPL5
RPS21	TRAM1
RPS21	SEC61B
RPS21	EIF4G1
RPS21	RPL11
RPS21	RPL10A
RPS21	SPCS2
RPS21	SPCS3

RPS21	MRPS10
RPS21	RPL37
RPS21	SRP72
RPS21	RPS3A
RPS21	EEF1A1P5
RPS21	RPL15
RPS21	RPL4
RPS21	RPS15
RPS21	RPL14
RPS21	NCBP2
RPS21	RSRC1
RPS27	CENPO
RPS27	RPL18
RPS27	B9D2
RPS27	NCBP1
RPS27	SERBP1
RPS27	RPS15A
RPS27	PPP2CB
RPS27	RCC2
RPS27	RPS3A
RPS27	NSL1
RPS27	RACK1
RPS27	STAG1
RPS27	RPL37
RPS27	RPS12
RPS27	RGPD8
RPS27	RPL13A
RPS27	RPL36A
RPS27	EIF4G1
RPS27	RPS19
RPS27	RPL15
RPS27	WBP1
RPS27	EIF3F
RPS27	RPL37A
RPS27	RPL13
RPS27	INCENP
RPS27	SMG7
RPS27	PPP1CC
RPS27	MRPS7
RPS27	SRP54
RPS27	RPL23
RPS27	NUP107
RPS27	RPS21
RPS27	RPL23A
RPS27	RPL3
RPS27	KNL1

RPS27	MAPRE1
RPS27	RPL41
RPS27	RPL4
RPS27	CDCA5
RPS27	SMC3
RPS27	TRAM1
RPS27	EIF3C
RPS27	RPL30
RPS27	NUP160
RPS27	WDR31
RPS27	RPL32
RPS27	EIF2S1
RPS27	RPL28
RPS27	EIF3G
RPS27	RPL12
RPS27	SPCS3
RPS27	RPS16
RPS27	SPDL1
RPS27	ERCC6L
RPS27	EIF4A1
RPS27	RRP1
RPS27	RPL24
RPS27	SKA2
RPS27	CENPN
RPS27	RPS17
RPS27	MRPS5
RPS27	SRPRB
RPS27	RPL19
RPS27	RPL31
RPS27	SGO2
RPS27	EIF5
RPS27	SMG1
RPS27	RPL17
RPS27	SSR3
RPS27	SEC61G
RPS27	RPS28
RPS27	SEC11B
RPS27	BUB1B
RPS27	EIF4B
RPS27	ITGB3BP
RPS27	RPL27
RPS27	SEC13
RPS27	CENPL
RPS27	CENPE
RPS27	EIF4A3
RPS27	RPL10A

RPS27	RPL36
RPS27	RPS7
RPS27	RPL27A
RPS27	RPL21
RPS27	RPL9P7
RPS27	UBC
RPS27	STAG2
RPS27	RPS24
RPS27	RPN1
RPS27	NCBP2
RPS27	PLEC
RPS27	SMG8
RPS27	SRP68
RPS27	RPL7A
RPS27	TAOK1
RPS27	AHCTF1
RPS27	BUB3
RPS27	EIF3I
RPS27	SMG5
RPS27	EIF4A2
RPS27	ETF1
RPS27	UPF2
RPS27	PPP2R1A
RPS27	MAGOH
RPS27	RPL11
RPS27	SRP72
RPS27	RSRC1
RPS27	ZWINT
RPS27	RPS3
RPS27	CKAP5
RPS27	NSA2
RPS27	RPL29
RPS27	SEC11C
RPS27	PAFAH1B1
RPS27	RPSA
RPS27	MIS12
RPS27	CLASP2
RPS27	PDS5A
RPS27	RPL35
RPS27	CASC3
RPS27	NDE1
RPS27	RPS15
RPS27	NUP43
RPS27	MRPS15
RPS27	RANGAP1
RPS27	FAU

RPS27	RPL36AL
RPS27	PMF1
RPS27	SPCS2
RPS27	EIF2S3
RPS27	CDC20
RPS27	RPS14
RPS27	RPL38
RPS27	ESPL1
RPS27	SSR2
RPS27	RPS25
RPS27	NDC80
RPS27	RPL26L1
RPS27	EIF5B
RPS27	DSN1
RPS27	CENPQ
RPS27	RPL7
RPS27	BUB1
RPS27	CENPC
RPS27	RPN2
RPS27	EIF4E
RPS27	MRPS10
RPS27	RPL14
RPS27	EIF3J
RPS27	EMB
RPS27	NUDC
RPS27	KNTC1
RPS27	CENPM
RPS27	EIF3H
RPS27	KIF2C
RPS27	UPF3A
RPS27	EIF3B
RPS27	MAD2L1
RPS27	BIRC5
RPS27	RPS5
RPS27	NDEL1
RPS27	EIF3A
RPS27	EIF2S2
RPS27	RPLP0P6
RPS27	EIF3K
RPS27	RPL35A
RPS27	KIF2A
RPS27	RPS8
RPS27	SSR1
RPS27	RPS9
RPS27	RPL18A
RPS27	CENPF

RPS27	CLIP1
RPS27	RNPS1
RPS27	CDCA8
RPS27	MAD1L1
RPS27	EIF1AX
RPS27	RPLP2
RPS27	NUP133
RPS27	CENPI
RPS27	RPL3L
RPS27	RPL26
RPS27	RPSAP58
RPS27	SMG9
RPS27	KRBA2
RPS27	CENPP
RPS27	EEF1A1P5
RPS27	PMF1-BGLAP
RPS27	MAST1
RPS27	EIF3E
RPS27	RPL8
RPS27	RAD21
RPS27	RPL17-C18orf32
RPS27	RPS2
RPS27	RLIM
RPS27	MRPS14
RPS27	CENPK
RPS27	PDS5B
RPS27	RPS20
RPS27	RPS26
RPS27	PLK1
RPS27	ZWILCH
RPS27	SMG6
RPS27	SRP14
RPS27	RPL5
RPS27	RPL10
RPS27	AURKB
RPS27	UPF3B
RPS27	CENPT
RPS27	SRP19
RPS27	RPL39
RPS27	RPS23
RPS27	NUP85
RPS27	RPL39L
RPS27	SPCS1
RPS27	NUF2
RPS27	RPL34
RPS27	SPC24

RPS27	RPS18
RPS27	NUP37
RPS27	RPL22
RPS27	WAPL
RPS27	RPS29
RPS27	EEF2
RPS27	RPS10-NUDT3
RPS27	CENPA
RPS27	CENPU
RPS27	RPS11
RPS27	RPS4X
RPS27	SEC61B
RPS27	SGO1
RPS27	ZW10
RPS27	SRP9
RPS27	PPP2R2D
RPS27	KIF18A
RPS27	RPS6
RPS27	UPF1
RPS27	EIF3D
RPS27	RPS13
RPS27	RPL6
RPS27	CENPS
RPS4X	SMG9
RPS4X	RPL7A
RPS4X	SEC11C
RPS4X	RPS16
RPS4X	RPS11
RPS4X	EIF3G
RPS4X	RPS6
RPS4X	SMG7
RPS4X	RPL9P7
RPS4X	SMG6
RPS4X	TRAM1
RPS4X	MAGOH
RPS4X	SMG5
RPS4X	RPS3
RPS4X	SRP9
RPS4X	RPL27A
RPS4X	RPL26L1
RPS4X	RPS17
RPS4X	MRPL4
RPS4X	EIF3J
RPS4X	RPS2
RPS4X	RPS24
RPS4X	RPL17

RPS4X	KRBA2
RPS4X	RPL15
RPS4X	EIF3E
RPS4X	RPL18
RPS4X	UPF1
RPS4X	RPL10
RPS4X	FAU
RPS4X	RPL31
RPS4X	UPF3B
RPS4X	RPL38
RPS4X	EIF3F
RPS4X	CASC3
RPS4X	EIF2S2
RPS4X	EIF1AX
RPS4X	EIF5B
RPS4X	RPL35
RPS4X	RPS13
RPS4X	ETF1
RPS4X	RPS19
RPS4X	RPL28
RPS4X	PPP2R2D
RPS4X	RPL5
RPS4X	UBC
RPS4X	EIF4A2
RPS4X	NCBP1
RPS4X	RPS28
RPS4X	EEF2
RPS4X	EIF2S3
RPS4X	MRPS5
RPS4X	RPL12
RPS4X	RPL13A
RPS4X	RPS29
RPS4X	SRPRB
RPS4X	EIF3B
RPS4X	SMG8
RPS4X	MRPL2
RPS4X	SRP14
RPS4X	RPS3A
RPS4X	RPN2
RPS4X	RPL36
RPS4X	SPCS1
RPS4X	EIF2S1
RPS4X	RPL17-C18orf32
RPS4X	SPCS3
RPS4X	EIF3C
RPS4X	RPL30

RPS4X	RPL18A
RPS4X	MRPS14
RPS4X	RPL19
RPS4X	RPL7
RPS4X	EEF1A1P5
RPS4X	RPLP0P6
RPS4X	EIF4A3
RPS4X	RPL21
RPS4X	RPS21
RPS4X	EIF3A
RPS4X	RPN1
RPS4X	RPS15A
RPS4X	RPS14
RPS4X	RPSAP58
RPS4X	RPS9
RPS4X	RSRC1
RPS4X	UPF2
RPS4X	RPS18
RPS4X	NCBP2
RPS4X	RPS23
RPS4X	SSR1
RPS4X	SRP72
RPS4X	RPL24
RPS4X	RPL37A
RPS4X	RPS20
RPS4X	RPL35A
RPS4X	RPL3L
RPS4X	RPL11
RPS4X	EIF3K
RPS4X	RPS5
RPS4X	RPL23
RPS4X	PPP2CB
RPS4X	RLIM
RPS4X	UPF3A
RPS4X	RPL36AL
RPS4X	RPS10-NUDT3
RPS4X	RPL29
RPS4X	RPL32
RPS4X	RPL39L
RPS4X	EIF4B
RPS4X	SEC61G
RPS4X	RPS25
RPS4X	RPL4
RPS4X	RNPS1
RPS4X	RPS27L
RPS4X	SMG1

RPS4X	RPL8
RPS4X	RPL36A
RPS4X	RPL26
RPS4X	SRP68
RPS4X	SPCS2
RPS4X	SEC11B
RPS4X	WDR31
RPS4X	EIF4G1
RPS4X	RACK1
RPS4X	RPL41
RPS4X	SEC61B
RPS4X	PPP2R1A
RPS4X	PLEC
RPS4X	SRP19
RPS4X	EIF3I
RPS4X	RPL37
RPS4X	RPL39
RPS4X	RPL14
RPS4X	RPL3
RPS4X	RPL23A
RPS4X	RPS7
RPS4X	RPL22
RPS4X	EIF3D
RPS4X	EIF3H
RPS4X	SSR3
RPS4X	EIF4E
RPS4X	RPS8
RPS4X	RPS26
RPS4X	RPLP2
RPS4X	RRP1
RPS4X	EIF5
RPS4X	RPL6
RPS4X	SRP54
RPS4X	SSR2
RPS4X	RPSA
RPS4X	RPS27
RPS4X	SERBP1
RPS4X	RPL34
RPS4X	RPS15
RPS4X	WBP1
RPS4X	RPL10A
RPS4X	RPS12
RPS4X	EIF4A1
RPS4X	RPL13
RPS4X	MRPS7
RPS4X	RPL27

RPS4X	RPL7L1
RPS6KC1	SPHK1
RPS6KC1	PRDX3
RPS8	EIF2S1
RPS8	RPSA
RPS8	RPL18A
RPS8	UPF3A
RPS8	RPL27A
RPS8	RPL10A
RPS8	RPS6
RPS8	RPL10
RPS8	RPS3
RPS8	EIF4G1
RPS8	RPL36A
RPS8	EIF4A3
RPS8	SMG1
RPS8	EIF3K
RPS8	RPL39
RPS8	RPS19
RPS8	SEC11B
RPS8	EIF4A2
RPS8	RPL7
RPS8	PPP2R1A
RPS8	RPS26
RPS8	RPN1
RPS8	EIF5
RPS8	SRP19
RPS8	MRPS5
RPS8	UPF3B
RPS8	SRP68
RPS8	RPL4
RPS8	SPCS3
RPS8	EIF3C
RPS8	RPL11
RPS8	SMG5
RPS8	SSR1
RPS8	RPS15
RPS8	WDR31
RPS8	RPS9
RPS8	EIF3E
RPS8	NCBP1
RPS8	RPL36AL
RPS8	SSR3
RPS8	SRP54
RPS8	RPSAP58
RPS8	EIF3J

RPS8	SMG6
RPS8	RPS5
RPS8	RPS23
RPS8	RPL39L
RPS8	EIF5B
RPS8	SERBP1
RPS8	PPP2R2D
RPS8	RPS21
RPS8	FBL
RPS8	RPL26
RPS8	RPL18
RPS8	RRP1
RPS8	UBC
RPS8	RPS13
RPS8	RPS25
RPS8	RPS2
RPS8	RPL5
RPS8	GNL3
RPS8	RPL37
RPS8	PPP2CB
RPS8	EIF3D
RPS8	RPL22
RPS8	SPCS1
RPS8	RPL23A
RPS8	RPLP2
RPS8	RPL28
RPS8	RPL23
RPS8	EEF1A1P5
RPS8	SEC61B
RPS8	RPS17
RPS8	RPL31
RPS8	RPL15
RPS8	RPL36
RPS8	RPL14
RPS8	NCBP2
RPS8	RPS14
RPS8	RPS12
RPS8	RPL30
RPS8	SNU13
RPS8	RPS15A
RPS8	RPS7
RPS8	EIF3I
RPS8	SMG7
RPS8	RPL26L1
RPS8	RPL3
RPS8	RPL13A

RPS8	RPS3A
RPS8	UPF1
RPS8	EIF2S3
RPS8	EIF4E
RPS8	CASC3
RPS8	RPL41
RPS8	SRP9
RPS8	UPF2
RPS8	RPL6
RPS8	EIF3G
RPS8	RPL29
RPS8	SEC61G
RPS8	SRP72
RPS8	RPL3L
RPS8	NIFK
RPS8	RPS24
RPS8	SEC11C
RPS8	TRAM1
RPS8	EIF4B
RPS8	EIF3F
RPS8	RPS18
RPS8	RPS28
RPS8	SSR2
RPS8	RPL19
RPS8	RPL17-C18orf32
RPS8	RPS27
RPS8	RPS27L
RPS8	RPLP0P6
RPS8	PLEC
RPS8	EIF4A1
RPS8	EIF1AX
RPS8	RPS20
RPS8	RPL13
RPS8	RPL8
RPS8	RPL12
RPS8	RPL17
RPS8	RPL9P7
RPS8	RPL7A
RPS8	RPS10-NUDT3
RPS8	KRBA2
RPS8	RNPS1
RPS8	EEF2
RPS8	ETF1
RPS8	EEF1G
RPS8	MAGOH
RPS8	RPL34

RPS8	RPL27
RPS8	RSRC1
RPS8	RACK1
RPS8	RPL32
RPS8	RPL24
RPS8	RLIM
RPS8	RPS16
RPS8	RPS29
RPS8	RPS11
RPS8	EIF3H
RPS8	WBP1
RPS8	RPL21
RPS8	RPS4X
RPS8	RPL37A
RPS8	RPN2
RPS8	SMG9
RPS8	SPCS2
RPS8	RPL35A
RPS8	EIF2S2
RPS8	SRP14
RPS8	SMG8
RPS8	FAU
RPS8	EIF3A
RPS8	RPL35
RPS8	RPL38
RPS8	EIF3B
RPS8	MRPS7
RPS8	RPL7L1
RPS8	SRPRB
RPSA	SSR3
RPSA	RPS8
RPSA	MRTO4
RPSA	MRPS15
RPSA	PPP2R2D
RPSA	SRP19
RPSA	EIF3K
RPSA	EIF3J
RPSA	RPS7
RPSA	MRPS6
RPSA	SEC11B
RPSA	RPL10
RPSA	LAMC2
RPSA	RPL3
RPSA	SRP72
RPSA	RPL13A
RPSA	RPS16

RPSA	RPL12
RPSA	RPS18
RPSA	RPL18
RPSA	RPS21
RPSA	SMG8
RPSA	RPL36A
RPSA	RPS23
RPSA	EIF2S2
RPSA	MRPS18A
RPSA	KARS1
RPSA	EIF3A
RPSA	SRP54
RPSA	MRPL3
RPSA	RPS19
RPSA	UBC
RPSA	MRPS10
RPSA	MRPS18C
RPSA	RPL41
RPSA	RPS29
RPSA	SEC61B
RPSA	SRP14
RPSA	UPF2
RPSA	NCBP1
RPSA	RPS5
RPSA	LAMA5
RPSA	RPS6
RPSA	RRP1
RPSA	SMG5
RPSA	RPL27A
RPSA	EIF3H
RPSA	KRBA2
RPSA	MRPL15
RPSA	RPL26
RPSA	RPL29
RPSA	RPS28
RPSA	SERBP1
RPSA	RPL7A
RPSA	RPLP0P6
RPSA	RPL39
RPSA	EIF3B
RPSA	RPS2
RPSA	RPL27
RPSA	SSR2
RPSA	SMG7
RPSA	EIF3F
RPSA	UPF1

RPSA	RPN1
RPSA	RPL9P7
RPSA	FAU
RPSA	EIF3D
RPSA	UPF3A
RPSA	RPL19
RPSA	MRPL4
RPSA	EIF4A2
RPSA	RPL11
RPSA	RNPS1
RPSA	RPL10A
RPSA	EIF2S3
RPSA	EEF1G
RPSA	RPS27
RPSA	RPN2
RPSA	RPL8
RPSA	RPS15A
RPSA	EIF1AX
RPSA	MRPS16
RPSA	MRPS5
RPSA	IMP3
RPSA	EEF1A1P5
RPSA	RACK1
RPSA	WBP1
RPSA	LAMB1
RPSA	RPL3L
RPSA	RPS11
RPSA	SRP9
RPSA	UPF3B
RPSA	RPS20
RPSA	SMG1
RPSA	PRNP
RPSA	RPL21
RPSA	MRPL11
RPSA	SPCS2
RPSA	RPL18A
RPSA	RPS17
RPSA	SEC11C
RPSA	RPL7
RPSA	EIF4E
RPSA	CASC3
RPSA	SSR1
RPSA	RPL34
RPSA	RPL32
RPSA	EIF4B
RPSA	RPL36AL

RPSA	SPCS1
RPSA	RPS10-NUDT3
RPSA	MRPL16
RPSA	EIF3C
RPSA	LAMB2
RPSA	EIF3G
RPSA	MAGOH
RPSA	EEF2
RPSA	LAMB3
RPSA	LAMC1
RPSA	MRPL1
RPSA	PPP1R16B
RPSA	RPL17-C18orf32
RPSA	RPL13
RPSA	RPL23A
RPSA	RPS13
RPSA	RPL22
RPSA	EIF2S1
RPSA	RPL5
RPSA	EIF4A3
RPSA	RPL26L1
RPSA	LAMC3
RPSA	LAMA3
RPSA	TRAM1
RPSA	RPL28
RPSA	RPL37A
RPSA	RSRC1
RPSA	RPL36
RPSA	RPS9
RPSA	RPS26
RPSA	MRPL2
RPSA	EFTUD2
RPSA	RPS12
RPSA	RPL15
RPSA	MRPS7
RPSA	EIF5B
RPSA	PPP2R1A
RPSA	MRPS11
RPSA	RPS3A
RPSA	RPS15
RPSA	RPL14
RPSA	NCBP2
RPSA	PPP2CB
RPSA	RPL31
RPSA	RPS3
RPSA	SMG6

RPSA	RPL30
RPSA	SRPRB
RPSA	ETF1
RPSA	MRPL24
RPSA	EIF4G1
RPSA	PLEC
RPSA	RPL24
RPSA	EIF3E
RPSA	RPL37
RPSA	SRP68
RPSA	RPL35
RPSA	RPS27L
RPSA	MRPL22
RPSA	MRPS14
RPSA	RPL35A
RPSA	RLIM
RPSA	RPL4
RPSA	RPS25
RPSA	RPS4X
RPSA	SPCS3
RPSA	RPL7L1
RPSA	SEC61G
RPSA	RPL38
RPSA	SMG9
RPSA	RPL17
RPSA	MRPS9
RPSA	WDR31
RPSA	EIF5
RPSA	RPS14
RPSA	RPL23
RPSA	EIF3I
RPSA	RPS24
RPSA	RPL6
RPSA	EIF4A1
RPSA	RPLP2
RRP15	NIFK
RRP15	PPAN-P2RY11
RRP15	SURF6
RTCB	C2orf49
RTCB	UBC
RTCB	DDX1
RTCB	FAM98B
RTN3	BACE1
RTN3	RTN4
SART3	NAA38
SART3	USP4

SART3	LSM3
SART3	LSM5
SART3	PRPF6
SART3	LSM2
SART3	TUT1
SART3	LSM4
SART3	MEPCE
SART3	PRPF4
SART3	USP15
SART3	PPIH
SART3	USP39
SART3	LSM7
SART3	LSM6
SART3	COIL
SAT2	SRM
SAT2	AGMAT
SAT2	MAOB
SAT2	DAO
SAT2	ODC1
SAT2	HIF1A
SAT2	MAOA
SCG2	PSIP1
SCG2	LEF1
SCG2	RBBP5
SCG2	BCL9L
SCG2	APC
SCG2	CHGB
SCG2	PYGO1
SCG2	NFKB1
SCG2	SMAD4
SCG2	KMT2D
SCG2	CTBP1
SCG2	JUND
SCG2	KMT2B
SCG2	SCG3
SCG2	SMAD2
SCG2	PYGO2
SCG2	MYC
SCG2	SMAD3
SCG2	CDKN2B
SCG2	CTNNB1
SCG2	BTRC
SCG2	ASH2L
SCG2	FANCD2
SCG2	TCF7L2
SCG2	WDR5

SEC23IP	PCNA
SEC23IP	PTK2
SEC23IP	UBE2H
SEC23IP	URM1
SEC23IP	SSU72
SEC24A	CANX
SEC24A	CD74
SEC24A	SEC24B
SEC24A	SEC24D
SEC24A	STMN2
SEC24A	HLA-C
SEC24A	STAT6
SEC24A	SEC23A
SEC24A	SEC24C
SEC24A	SREBF2
SEC24A	SEC31A
SEC24A	MBTPS1
SEC24A	SEC13
SEC24A	HLA-B
SEC24A	THADA
SEC24A	B2M
SEC24A	SAR1B
SEC24A	SAR1A
SEC24A	PREB
SEC24A	SCAP
SEC62	SEC61B
SEC62	SEC61A1
SEC62	SEC63
SEC62	SEC61G
SEC62	SPCS1
SEC63	SEC62
SEC63	UBC
SEC63	CSNK2A2
SEC63	CSNK2A3
SEC63	SEC61A2
SEC63	SEC61B
SEC63	SEC61A1
SEMA6D	PIGB
SEMA6D	PLXNA1
SENP1	SUMO1
SENP1	RANGAP1
SENP1	RANBP6
SENP1	SUMO3
SENP1	IPO5
SENP1	HDAC1
SERBP1	RPS27

SERBP1	PGRMC1
SERBP1	RPS9
SERBP1	RPS23
SERBP1	RPS19
SERBP1	RPS10-NUDT3
SERBP1	RPS6
SERBP1	SERPINE1
SERBP1	RPS20
SERBP1	RPS8
SERBP1	RPS5
SERBP1	PRMT1
SERBP1	RPSA
SERBP1	RPS3A
SERBP1	RPS7
SERBP1	CHD3
SERBP1	RPS13
SERBP1	RPS3
SERBP1	RPS15A
SERBP1	RPS11
SERBP1	RPS17
SERBP1	EEF2
SERBP1	UBC
SERBP1	RPS21
SERBP1	RPS14
SERBP1	FAU
SERBP1	RPS28
SERBP1	RPS15
SERBP1	RPS25
SERBP1	RPS16
SERBP1	RPS2
SERBP1	RPS4X
SERBP1	RPS24
SERBP1	RPS12
SERBP1	RPS26
SERBP1	RPS29
SERBP1	RACK1
SERPINA5	PROC
SERPINA5	PLAU
SERPINA5	F2
SERPINA5	HK3
SERPINF1	PNPLA2
SERPINF1	LRP6
SERPINF1	VEGFA
SETX	UBC
SETX	POLR2A
SETX	UBXN7

SETX	POLR3A
SFPQ	HDAC1
SFPQ	UBC
SFPQ	SNRPA
SFPQ	PTBP1
SFPQ	SIN3A
SFPQ	NONO
SFPQ	SLTM
SFPQ	SNHG4
SFRP1	WNT10A
SFRP1	MYC
SFRP1	FZD6
SFRP1	WNT5B
SFRP1	WNT6
SFRP1	WNT16
SFRP1	WNT2B
SFRP1	MAX
SFRP1	WNT4
SFRP1	WNT9A
SFRP1	WNT5A
SFRP1	WNT8B
SFRP1	WNT3
SFXN1	RCN2
SFXN1	TOMM7
SFXN1	TOMM20
SFXN2	SPG7
SFXN2	TOMM7
SGCB	CAV3
SGCB	SGCD
SGCB	SGCG
SGCB	DMD
SGCB	SGCE
SGCB	DAG1
SHPRH	UBE2V2
SHPRH	RAD18
SHPRH	PCNA
SHQ1	DKC1
SHQ1	NOP10
SHQ1	GAR1
SHQ1	NHP2
SIGMAR1	MSMO1
SIGMAR1	CYP51A1
SIGMAR1	UBC
SIGMAR1	SQLE
SIGMAR1	ERG28
SLC25A37	FXN

SLC25A37	FECH
SLC25A37	ABCB10
SLC25A37	LYRM4
SLC25A37	ABCB7
SLC25A6	TOMM40
SLC25A6	TIMM50
SLC25A6	TIMM10B
SLC25A6	TOMM5
SLC25A6	VDAC3
SLC25A6	TIMM22
SLC25A6	TOMM20
SLC25A6	VDAC1
SLC25A6	VDAC2
SLC25A6	PPIF
SLC25A6	TIMM9
SLC25A6	UBC
SLC25A6	TOMM22
SLC25A6	TOMM7
SLC25A6	TIMM10
SLC25A6	TIMM21
SLC30A6	SLC30A4
SLC30A6	SLC30A1
SLC33A1	B4GALNT1
SLC33A1	ST8SIA1
SLC33A1	ST8SIA5
SLC35B2	SULT4A1
SLC35B2	PAPSS1
SLC35B2	PAPSS2
SLC35D1	UGDH
SLC35D1	SLC35A3
SLC44A2	CHAT
SLC44A2	ACHE
SLC5A6	HLCS
SLC5A6	PCCB
SLC5A6	PCCA
SLC5A6	ACACB
SLC5A6	ACACA
SLC5A6	MCCC1
SLC5A6	PC
SMAP2	ING3
SMAP2	UCHL5
SMAP2	MEAF6
SMAP2	KAT5
SMAP2	INO80
SMAP2	VPS72
SMAP2	ACTR5

SMAP2	ACTL6A
SMAP2	EAF2
SMAP2	TRRAP
SMAP2	INO80E
SMAP2	RUVBL1
SMAP2	MORF4L1
SMAP2	EPC1
SMAP2	YY1
SMAP2	ACTG1
SMAP2	EP400
SMAP2	ACTR8
SMAP2	YEATS4
SMAP2	MORF4L2
SMAP2	ANP32E
SMAP2	INO80C
SMAP2	MCRS1
SMAP2	RUVBL2
SMAP2	INO80B
SMAP2	MRGBP
SMAP2	NFRKB
SMG1	RPL15
SMG1	RPS15
SMG1	RPL29
SMG1	RPLP2
SMG1	RPL30
SMG1	RUVBL1
SMG1	RPTOR
SMG1	RPS8
SMG1	EIF4G1
SMG1	RPL6
SMG1	RPS3
SMG1	RPL12
SMG1	RPL37
SMG1	TELO2
SMG1	PPP2R2D
SMG1	ETF1
SMG1	RPL27
SMG1	RPS19
SMG1	RPL4
SMG1	SMG7
SMG1	RPL35A
SMG1	RPS2
SMG1	RPS6
SMG1	RPL17
SMG1	RPS21
SMG1	RPL41

SMG1	RPL10A
SMG1	RPS28
SMG1	POLR2E
SMG1	RPL11
SMG1	RNPS1
SMG1	UBC
SMG1	RPLP0P6
SMG1	RPS5
SMG1	RPS27
SMG1	SMG8
SMG1	RPL3L
SMG1	RPS11
SMG1	RPS23
SMG1	RPL26
SMG1	TTI1
SMG1	RPS20
SMG1	RPL39
SMG1	RPL21
SMG1	HSPA4
SMG1	RPL34
SMG1	RPS15A
SMG1	RPL27A
SMG1	SMG9
SMG1	RPL9P7
SMG1	UPF1
SMG1	RPS13
SMG1	RPL22
SMG1	RPL13
SMG1	RPL7
SMG1	TUBA1B
SMG1	RPSA
SMG1	RPS12
SMG1	UPF3A
SMG1	RPL37A
SMG1	PPP2R1A
SMG1	RPL19
SMG1	UPF3B
SMG1	RPL18A
SMG1	FAU
SMG1	CASC3
SMG1	RPL8
SMG1	PPP2CB
SMG1	MLST8
SMG1	RPL23A
SMG1	RPL35
SMG1	UPF2

SMG1	RPL26L1
SMG1	RPL28
SMG1	RPS4X
SMG1	SMG6
SMG1	SMG5
SMG1	RPL38
SMG1	RPL5
SMG1	RICTOR
SMG1	RPL31
SMG1	RPS10-NUDT3
SMG1	RPL32
SMG1	RRP1
SMG1	RLIM
SMG1	MAGOH
SMG1	NCBP2
SMG1	RPS16
SMG1	RPL3
SMG1	RPS14
SMG1	RPL13A
SMG1	RPL23
SMG1	RPL24
SMG1	RPL18
SMG1	RPS24
SMG1	RUVBL2
SMG1	EIF4A3
SMG1	RPS26
SMG1	NCBP1
SMG1	RPS29
SMG1	RPS3A
SMG1	RPL36
SMG1	RPL14
SMG1	RPS7
SMG5	RRP1
SMG5	RPS15A
SMG5	RPL23
SMG5	RPL28
SMG5	RPL26
SMG5	RPL39
SMG5	RPS10-NUDT3
SMG5	RPS4X
SMG5	RPS19
SMG5	RPL32
SMG5	CASC3
SMG5	RPL6
SMG5	RPL7
SMG5	SMG7

SMG5	RPL29
SMG5	RPS24
SMG5	RPL31
SMG5	RPS8
SMG5	RPS28
SMG5	UPF1
SMG5	RPS23
SMG5	RPL26L1
SMG5	PPP2R2D
SMG5	RPL41
SMG5	RPSA
SMG5	SMG6
SMG5	RPS14
SMG5	PPP2R1A
SMG5	RPL13
SMG5	EIF4A3
SMG5	RPS6
SMG5	RPL18
SMG5	RPL22
SMG5	RPL17
SMG5	RPL24
SMG5	PPP2CB
SMG5	RPLP2
SMG5	UPF3A
SMG5	RPL3L
SMG5	RPL37
SMG5	PRKACA
SMG5	RLIM
SMG5	RPL21
SMG5	RPL3
SMG5	RPL4
SMG5	MAGOH
SMG5	RPS3
SMG5	RPS27
SMG5	UPF3B
SMG5	RPL37A
SMG5	RPL18A
SMG5	RPL19
SMG5	RPLP0P6
SMG5	RPL35A
SMG5	RPS21
SMG5	HSP90AA1
SMG5	RPL35
SMG5	RPL38
SMG5	RPS3A
SMG5	RPS15

SMG5	RPL5
SMG5	UPF2
SMG5	RPL9P7
SMG5	RPL23A
SMG5	RPL13A
SMG5	RPS16
SMG5	RPL30
SMG5	RPS13
SMG5	RPS7
SMG5	UBC
SMG5	RPS11
SMG5	NCBP2
SMG5	TERT
SMG5	RPL27A
SMG5	RPS20
SMG5	SMG1
SMG5	RPL27
SMG5	RPL34
SMG5	ETF1
SMG5	SMG9
SMG5	RPS2
SMG5	NCBP1
SMG5	RPS29
SMG5	RPS26
SMG5	RPL8
SMG5	PTGES3
SMG5	FAU
SMG5	RPS5
SMG5	RPL14
SMG5	RPL12
SMG5	RPL36
SMG5	RPL15
SMG5	SMG8
SMG5	RPS12
SMG5	RNPS1
SMG5	RPL11
SMG5	RPL10A
SMG5	EIF4G1
SMG7	RPL23
SMG7	PPP2CB
SMG7	RPL38
SMG7	RPS4X
SMG7	RPS10-NUDT3
SMG7	RPL14
SMG7	RPL15
SMG7	SMG5

SMG7	RPS27
SMG7	SMG1
SMG7	RPL39
SMG7	UPF3A
SMG7	RPL34
SMG7	RPS11
SMG7	RPL22
SMG7	RPS16
SMG7	RPL37A
SMG7	RPLP0P6
SMG7	RPL30
SMG7	RPS14
SMG7	RPS12
SMG7	RNPS1
SMG7	RPL21
SMG7	MAGOH
SMG7	RPL9P7
SMG7	RPL26
SMG7	RPSA
SMG7	RPL6
SMG7	RPL26L1
SMG7	RPL27A
SMG7	CASC3
SMG7	RPL5
SMG7	PPP2R1A
SMG7	RPS19
SMG7	UPF2
SMG7	RPL41
SMG7	RPS24
SMG7	UBC
SMG7	RPS26
SMG7	RPS20
SMG7	RPL17
SMG7	RRP1
SMG7	UPF3B
SMG7	RPL23A
SMG7	FAU
SMG7	RPL27
SMG7	RPS8
SMG7	RPS15
SMG7	SMG6
SMG7	SMG9
SMG7	RPL8
SMG7	RPS28
SMG7	RPL24
SMG7	RLIM

SMG7	ETF1
SMG7	RPS13
SMG7	RPL35
SMG7	RPL29
SMG7	RPL4
SMG7	RPL31
SMG7	RPL36
SMG7	RPL13
SMG7	RPL28
SMG7	RPL11
SMG7	RPL13A
SMG7	RPS29
SMG7	RPL3
SMG7	RPL19
SMG7	RPLP2
SMG7	RPL18
SMG7	RPS23
SMG7	RPS5
SMG7	RPL18A
SMG7	EIF4A3
SMG7	RPL7
SMG7	PPP2R2D
SMG7	RPS21
SMG7	NCBP2
SMG7	EIF4G1
SMG7	RPS3
SMG7	RPL37
SMG7	RPS15A
SMG7	RPL12
SMG7	RPS6
SMG7	RPS2
SMG7	SMG8
SMG7	UPF1
SMG7	RPS3A
SMG7	NCBP1
SMG7	RPL10A
SMG7	RPL32
SMG7	RPL35A
SMG7	RPL3L
SMG7	RPS7
SNRPB	DDX20
SNRPB	SNRPB2
SNRPB	HNRNPA1
SNRPB	MAGOH
SNRPB	SNRNP40
SNRPB	LSM6

SNRPB	RBMX
SNRPB	HNRNPK
SNRPB	LSM2
SNRPB	RAE1
SNRPB	SRRM1
SNRPB	POLR2K
SNRPB	NUP133
SNRPB	SF3A1
SNRPB	SRSF6
SNRPB	PRPF6
SNRPB	U2AF2
SNRPB	SNRPF
SNRPB	CCAR1
SNRPB	PTBP1
SNRPB	SNRPC
SNRPB	HNRNPF
SNRPB	SF3B1
SNRPB	SNRPD3
SNRPB	PABPN1
SNRPB	SMN2
SNRPB	POLR2J
SNRPB	TGS1
SNRPB	EFTUD2
SNRPB	SRSF4
SNRPB	SRSF9
SNRPB	CDC40
SNRPB	WBP4
SNRPB	SNRPD1
SNRPB	PCBP1
SNRPB	SF3B5
SNRPB	SUGP1
SNRPB	NUDT21
SNRPB	DNAJC8
SNRPB	HNRNPD
SNRPB	NUP54
SNRPB	NUP88
SNRPB	STRAP
SNRPB	SNRPD2
SNRPB	SRSF1
SNRPB	SLBP
SNRPB	HNRNPC
SNRPB	GEMIN4
SNRPB	LSM10
SNRPB	AAAS
SNRPB	SNUPN
SNRPB	YBX1

SNRPB	CPSF7
SNRPB	TPR
SNRPB	NUP35
SNRPB	PHF5A
SNRPB	POLR2G
SNRPB	GEMIN2
SNRPB	HNRNPA0
SNRPB	RBM5
SNRPB	NCBP2
SNRPB	CPSF2
SNRPB	SNRNP70
SNRPB	LSM11
SNRPB	SF3A2
SNRPB	UPF3B
SNRPB	SRSF7
SNRPB	CSTF3
SNRPB	U2AF1L4
SNRPB	CSTF1
SNRPB	SNRPG
SNRPB	NUP188
SNRPB	PAPOLA
SNRPB	HNRNPUL1
SNRPB	CPSF3
SNRPB	RGPD8
SNRPB	POLR2I
SNRPB	SNRPA
SNRPB	POLR2L
SNRPB	PRMT5
SNRPB	HNRNPR
SNRPB	NCBP1
SNRPB	LSM4
SNRPB	ALYREF
SNRPB	GEMIN8
SNRPB	SNRPE
SNRPB	HNRNPH1
SNRPB	POLR2F
SNRPB	POLR2A
SNRPB	CLP1
SNRPB	LSM7
SNRPB	DDX23
SNRPB	NUP153
SNRPB	SRSF2
SNRPB	POLR2H
SNRPB	PHAX
SNRPB	NUP62
SNRPB	NUP107

SNRPB	SRSF3
SNRPB	POLR2D
SNRPB	CSTF2
SNRPB	SNRNP200
SNRPB	PCBP2
SNRPB	HNRNPA3
SNRPB	RNPS1
SNRPB	HNRNPM
SNRPB	POLR2B
SNRPB	CLNS1A
SNRPB	HNRNPA2B1
SNRPB	SNRPA1
SNRPB	SNU13
SNRPB	PCF11
SNRPB	NUP93
SNRPB	SF3A3
SNRPB	WDR77
SNRPB	HNRNPU
SNRPB	POLR2E
SNRPB	GTF2F1
SNRPB	DHX9
SNRPB	SF3B3
SNRPB	GTF2F2
SNRPB	CD2BP2
SNRPB	NFX1
SNRPB	SRSF5
SNRPB	LSM3
SNRPB	SRSF11
SNRPB	GEMIN5
SNRPB	FUS
SNRPB	NUP50
SNRPB	PRPF8
SNRPB	NUP160
SNRPB	POLR2C
SNRPB	PRPF4
SNRPB	NAA38
SNRPB	TXNL4A
SNRPB	ZNF473
SNRPB	NUP210
SNRPB	NUP214
SNRPB	GEMIN7
SNRPB	NUP155
SNRPB	NUP37
SNRPB	GEMIN6
SNRPB	NUP205
SNRPB	DHX38

SNRPB	NUP85
SNRPB	HNRNPL
SNRPB	NUP43
SPCS3	RPS26
SPCS3	RPS28
SPCS3	RPL3L
SPCS3	RPN2
SPCS3	RPL7
SPCS3	GATA4
SPCS3	MBOAT4
SPCS3	RPL13
SPCS3	SPCS2
SPCS3	SEC11C
SPCS3	SRP68
SPCS3	RPL9P7
SPCS3	SSR2
SPCS3	ISL1
SPCS3	RPLP0P6
SPCS3	RPS8
SPCS3	FAU
SPCS3	SRP14
SPCS3	RPL12
SPCS3	RPL28
SPCS3	RPL35A
SPCS3	RPS23
SPCS3	RPL39
SPCS3	RPL19
SPCS3	RPL5
SPCS3	RPL24
SPCS3	SRP72
SPCS3	RPS27
SPCS3	RPL11
SPCS3	RPS7
SPCS3	SEC61G
SPCS3	RPL22
SPCS3	RPL4
SPCS3	RPL14
SPCS3	RPN1
SPCS3	RPL27A
SPCS3	RPL38
SPCS3	SPCS1
SPCS3	SSR1
SPCS3	RPS20
SPCS3	SRP19
SPCS3	RPL35
SPCS3	RPL29

SPCS3	RPS3A
SPCS3	RPS15
SPCS3	RPS4X
SPCS3	RPL6
SPCS3	RPL30
SPCS3	RPL34
SPCS3	RPL41
SPCS3	RPS24
SPCS3	RPL21
SPCS3	RPL3
SPCS3	RPS3
SPCS3	RPS10-NUDT3
SPCS3	RPS2
SPCS3	RPL37A
SPCS3	WBP1
SPCS3	UBC
SPCS3	RPL31
SPCS3	SEC61B
SPCS3	SRP9
SPCS3	SSR3
SPCS3	RPS15A
SPCS3	RPL18
SPCS3	SRP54
SPCS3	RPL26
SPCS3	RPS5
SPCS3	RPS13
SPCS3	RPLP2
SPCS3	RPS14
SPCS3	SEC11B
SPCS3	RPL23A
SPCS3	RPS11
SPCS3	RPL23
SPCS3	RPS19
SPCS3	RPL36
SPCS3	RPL13A
SPCS3	RPS12
SPCS3	TCF7L2
SPCS3	SRPRB
SPCS3	RPL8
SPCS3	RPL17
SPCS3	RPL26L1
SPCS3	TRAM1
SPCS3	RPS29
SPCS3	RPL37
SPCS3	RRP1
SPCS3	RPS6

SPCS3	RPL10A
SPCS3	RPL32
SPCS3	RPL18A
SPCS3	RPL27
SPCS3	RPSA
SPCS3	RPS21
SPCS3	RPS16
SPCS3	RPL15
SPIRE1	FMN1
SPIRE1	FMN2
SREK1	SRSF3
SREK1	SRSF2
SREK1	SRSF6
SRP14	SRP9
SRP14	RPS3
SRP14	RPL27A
SRP14	RPL23A
SRP14	RPL24
SRP14	RPL26
SRP14	RPS23
SRP14	RPN1
SRP14	RPS16
SRP14	RPS10-NUDT3
SRP14	TRAM1
SRP14	RPL36
SRP14	RPL23
SRP14	SPCS3
SRP14	RPL31
SRP14	RPS6
SRP14	RPSA
SRP14	FAU
SRP14	SEC11C
SRP14	RPL15
SRP14	SEC11B
SRP14	SEC61G
SRP14	UBC
SRP14	RPS29
SRP14	RPL12
SRP14	RPS13
SRP14	RPLP2
SRP14	SRPRB
SRP14	RPL35
SRP14	RPS4X
SRP14	RPL7
SRP14	RPL30
SRP14	RPN2

SRP14	RPL34
SRP14	SEC61B
SRP14	RPLP0P6
SRP14	RRP1
SRP14	RPS11
SRP14	RPL10A
SRP14	RPL4
SRP14	RPL18A
SRP14	RPL26L1
SRP14	RPL18
SRP14	RPL13A
SRP14	RPL37A
SRP14	RPL35A
SRP14	SRP72
SRP14	RPS24
SRP14	SRP54
SRP14	RPS21
SRP14	RPS28
SRP14	RPS26
SRP14	RPL14
SRP14	RPS12
SRP14	SPCS2
SRP14	RPL13
SRP14	SSR1
SRP14	RPL3
SRP14	RPL17
SRP14	RPS7
SRP14	RPS2
SRP14	RPL5
SRP14	RPL32
SRP14	RPL28
SRP14	RPL8
SRP14	RPL22
SRP14	RPL6
SRP14	RPL29
SRP14	RPL9P7
SRP14	RPL3L
SRP14	RPS15
SRP14	RPS3A
SRP14	RPS20
SRP14	RPS19
SRP14	RPL37
SRP14	RPS27
SRP14	WBP1
SRP14	RPL27
SRP14	RPS5

SRP14	SPCS1
SRP14	RPL38
SRP14	SRP19
SRP14	RPS14
SRP14	RPS15A
SRP14	RPS8
SRP14	SRP68
SRP14	RPL39
SRP14	RPL41
SRP14	RPL11
SRP14	RPL19
SRP14	SSR3
SRP14	RPL21
SRP14	SSR2
SRP68	RPL5
SRP68	RPS24
SRP68	RPL18
SRP68	TRAM1
SRP68	RPL3
SRP68	RPL35A
SRP68	RPS13
SRP68	RPL22
SRP68	RPLP2
SRP68	RPS19
SRP68	RPL35
SRP68	RPL3L
SRP68	RPL13A
SRP68	SPCS3
SRP68	RPL23
SRP68	RPS8
SRP68	RPS3
SRP68	WBP1
SRP68	SPCS1
SRP68	RPL37
SRP68	RPS23
SRP68	RPLP0P6
SRP68	SSB
SRP68	RPL4
SRP68	RPL34
SRP68	RRP1
SRP68	RPL30
SRP68	UBC
SRP68	RPL31
SRP68	SRP9
SRP68	RPN1
SRP68	RPL18A

SRP68	RPL29
SRP68	RPS20
SRP68	RPL36
SRP68	RPS2
SRP68	SEC61G
SRP68	RPS6
SRP68	RPL11
SRP68	RPS27
SRP68	RPS15
SRP68	RPL41
SRP68	RPL12
SRP68	SSR1
SRP68	RPL32
SRP68	RPL9P7
SRP68	SRP72
SRP68	RPS21
SRP68	RPL26
SRP68	RPS5
SRP68	RPS14
SRP68	RPL19
SRP68	RPS29
SRP68	RPL13
SRP68	RPL6
SRP68	RPL7
SRP68	RPS11
SRP68	SRPRB
SRP68	RPL38
SRP68	SPCS2
SRP68	FAU
SRP68	SRP19
SRP68	SEC11B
SRP68	RPL21
SRP68	RPL8
SRP68	RPL15
SRP68	SSR2
SRP68	RPS7
SRP68	RPS28
SRP68	RPL14
SRP68	RPN2
SRP68	RPS4X
SRP68	RPS26
SRP68	SSR3
SRP68	RPL24
SRP68	RPL28
SRP68	SEC11C
SRP68	RPL39

SRP68	RPS3A
SRP68	RPL10A
SRP68	RPL27A
SRP68	RPS10-NUDT3
SRP68	RPSA
SRP68	RPL26L1
SRP68	RPS12
SRP68	SEC61B
SRP68	RPL23A
SRP68	RPL17
SRP68	SRP14
SRP68	RPL27
SRP68	RPS15A
SRP68	RPL37A
SRP68	RPS16
SRP68	SRP54
SRP72	RPL8
SRP72	RPL4
SRP72	SPCS1
SRP72	SRPRB
SRP72	RPSA
SRP72	RPL22
SRP72	RPS12
SRP72	RPL34
SRP72	SEC11C
SRP72	RPL12
SRP72	UBC
SRP72	RPL35
SRP72	WBP1
SRP72	RPL35A
SRP72	RPL21
SRP72	SEC61G
SRP72	RPL10A
SRP72	SPCS3
SRP72	RPL37
SRP72	RPS10-NUDT3
SRP72	RPN1
SRP72	RPL32
SRP72	RPS6
SRP72	RPL13A
SRP72	SEC61B
SRP72	RPL11
SRP72	SSR3
SRP72	RPS15A
SRP72	RPL31
SRP72	RPL30

SRP72	RPL26
SRP72	RPL3L
SRP72	RPS28
SRP72	RPS13
SRP72	RPS27
SRP72	RPS26
SRP72	RPS7
SRP72	RPS5
SRP72	RPS11
SRP72	SRP68
SRP72	RPL13
SRP72	RPL19
SRP72	RPS24
SRP72	RPL24
SRP72	RPS20
SRP72	RPL9P7
SRP72	RPS15
SRP72	RPS2
SRP72	RPS4X
SRP72	RPL38
SRP72	RPL37A
SRP72	RPS29
SRP72	TRAM1
SRP72	SPCS2
SRP72	RPL18A
SRP72	RPN2
SRP72	SRP14
SRP72	RPS8
SRP72	RPL28
SRP72	RPLP2
SRP72	RPLP0P6
SRP72	RPL15
SRP72	RPL23A
SRP72	RRP1
SRP72	RPL14
SRP72	RPL17
SRP72	RPL41
SRP72	RPS23
SRP72	RPL3
SRP72	FAU
SRP72	SSB
SRP72	SEC11B
SRP72	RPL36
SRP72	RPS19
SRP72	SSR2
SRP72	RPL6

SRP72	RPL29
SRP72	SRP54
SRP72	RPL39
SRP72	RPS14
SRP72	RPL18
SRP72	RPL23
SRP72	SRP9
SRP72	RPS16
SRP72	RPL7
SRP72	RPL27
SRP72	RPL27A
SRP72	RPS3
SRP72	RPL26L1
SRP72	SSR1
SRP72	RPS21
SRP72	RPL5
SRP72	RPS3A
SRP72	SRP19
SRPK1	SRSF2
SRPK1	SRSF4
SRPK1	HNRNPC
SRPK1	SNRNP70
SRPK1	SRSF1
SRPK1	SRSF7
SRPK1	LBR
SRPK1	SF3A3
SRPK1	U2AF2
SRPK1	SRSF3
SRPK1	U2AF1L4
SRPK1	SRSF9
SRPK1	SRSF5
SRPK1	SRSF6
SRSF12	MAGOH
SRSF12	SRSF2
SSH2	CFL2
SSH2	CFL1
SSH3	CFL1
SSH3	CFL2
SSX2IP	ACTN3
SSX2IP	ACTN1
STAU1	RPS6
STAU1	RLIM
STAU1	UPF1
STAU1	CASC3
STRAP	SNRPF
STRAP	MAP3K7

STRAP	SRF
STRAP	SNRPD3
STRAP	SMAD3
STRAP	SNRPD2
STRAP	SNRPG
STRAP	GEMIN8
STRAP	CSDE1
STRAP	TAB2
STRAP	GEMIN5
STRAP	SMAD2
STRAP	TGFB1
STRAP	SNRPD1
STRAP	GEMIN7
STRAP	SNRPB
STRAP	TAB1
STRAP	PDPK1
STRAP	ZFYVE9
STRAP	XIAP
STRAP	GEMIN4
STRAP	TGFBR1
STRAP	DDX20
STRAP	NEDD4L
STRAP	NME1
STRAP	GEMIN6
STRAP	SNRPE
STRAP	GEMIN2
STRAP	CTNNB1
STRAP	SMAD7
STRAP	PBLD
STRAP	TGFBR2
STRAP	PML
STRAP	ZPR1
STRAP	SMN2
STT3B	RPN1
STT3B	MAGT1
STT3B	SEC61A2
STT3B	SEC61A1
STT3B	RPN2
STT3B	WBP1
STT3B	TUSC3
STT3B	MOGS
STT3B	DOLPP1
STT3B	DAD1
STT3B	ALG10
STT3B	ALG10B
SUV39H2	PLOD3

SUV39H2	PLOD1
SUV39H2	PLOD2
SYT7	SYNCRIP
SYT7	STX4
TAGLN2	SNRPF
TAGLN2	UBC
TAGLN2	PPIA
TARDBP	SOD1
TARDBP	UBQLN1
TARDBP	UBQLN2
TARDBP	UBC
TARDBP	ATXN2
TARDBP	FUS
TARDBP	SQSTM1
TARDBP	C9orf72
TARDBP	HSP90AA1
TARDBP	G3BP1
TARDBP	VAPB
TARDBP	VCP
TBX2	BMP2
TBX2	ZNF225
TBX3	LEF1
TBX3	HDAC1
TCF12	TCF3
TCF12	TWIST1
TCF12	ASCL1
TCF12	ID2
TCF12	TWIST2
TDP2	ETS1
TDP2	HSP90AB2P
TDP2	UBC
TDP2	NFKB1
TDP2	HSP90AA1
TDP2	PARK7
TDP2	HSP90B1
TDP2	NFKBIA
TDP2	RELA
TFE3	E2F3
TFE3	SMAD2
TFE3	SMAD3
TFE3	COL1A2
TIMM8B	TIMM10
TIMM8B	TIMM13
TIMM8B	TIMM10B
TIMM8B	TIMM9
TIMM8B	UBC

TIMM8B	TIMM23B
TLX2	SMAD4
TLX2	SMAD9
TLX2	SMAD1
TLX2	SMAD5
TMED2	SEC13
TMED2	FURIN
TMED2	TMED10
TMED2	TMED9
TMED2	TMED4
TMED2	NOTCH1
TMEM30A	ATP8A1
TMEM30A	ATP8B1
TMEM30A	UBC
TMEM30A	ATP8B2
TMEM9	OXCT1
TMEM9	VMA21
TMPO	LMNB1
TMPO	UBC
TMPO	AKAP8L
TMPO	LMNA
TNFRSF12A	TRAF2
TNFRSF12A	TNFSF13
TOMM20	BCL2
TOMM20	GRPEL2
TOMM20	MTX2
TOMM20	TIMM44
TOMM20	TIMM17A
TOMM20	TOMM5
TOMM20	TIMM22
TOMM20	GRPEL1
TOMM20	TIMM50
TOMM20	PINK1
TOMM20	TOMM40L
TOMM20	TIMM10
TOMM20	TIMM21
TOMM20	TOMM22
TOMM20	PAM16
TOMM20	SAMM50
TOMM20	TIMM23B
TOMM20	DNAJC19
TOMM20	TOMM40
TOMM20	VDAC1
TOMM20	TOMM7
TOMM20	SLC25A6
TOMM20	TIMM9

TOMM20	SFXN1
TOMM20	GFER
TOMM20	HSPA9
TOMM20	MTX1
TOMM20	CHCHD4
TP53I3	MRPL52
TP53I3	RPL13
TP53I3	CSE1L
TP53I3	PPP1R13B
TP53I3	RPL23
TP53I3	TP53BP2
TP53I3	TP53
TP53I3	MRPL42
TP53I3	MRPL51
TP53I3	MRPL10
TP53I3	MRPL3
TP53I3	MRPL55
TPM4	DMD
TPM4	TNNC2
TPM4	ACTN3
TPM4	MYLK
TPM4	MYH11
TPM4	NAA25
TPM4	TLN1
TPM4	ACTG2
TPM4	ACTA2
TPM4	UBC
TPM4	NEB
TPM4	VIM
TPM4	TPM1
TPM4	CALD1
TPM4	TTN
TPM4	MYL6
TPM4	TNNI2
TPM4	MYL12B
TPM4	MYBPC3
TPM4	PXN
TPM4	TMOD1
TPM4	LMOD1
TPM4	TNNC1
TPM4	SORBS1
TPM4	TNNT1
TPM4	MYL6B
TPM4	TNNI3
TPM4	TCAP
TPM4	ITGA1

TPM4	ITGB5
TPM4	VCL
TPM4	SORBS3
TPM4	TPM3
TPM4	TRIP6
TPM4	MYH3
TPM4	TNNT2
TPM4	MYL3
TRA2A	SAP18
TRA2A	MAGOH
TRA2A	PRPF40A
TRA2A	SRSF10
TRAF7	SKP1
TRAF7	CDC6
TRIM23	IKBKG
TRIM23	UBE2E1
TRMT10C	WDR4
TRMT10C	PRORP
TRMT5	TRMT1
TRMT5	TRMT12
TSEN54	TSEN2
TSEN54	TSEN15
TSEN54	TSEN34
TUBB2B	TUBA1C
TUBB2B	UBC
TUBB2B	TUBB2A
TUBB2B	TBCA
TUBB2B	PFDN5
TUBB2B	DYNC2H1
TUBB2B	ACTG1
TUBB2B	CCT7
TUBB2B	TBCB
TUBB2B	TCP1
TUBB2B	TBCE
TUBB2B	PFDN2
TUBB2B	PFDN1
TUBB2B	TUBB4B
TUBB2B	CCT2
TUBB2B	TUBB1
TUBB2B	TUBB6
TUBB2B	DYNC1H1
TUBB2B	CCT6A
TUBB2B	TBCD
TUBB2B	PFDN4
TUBB2B	TUBA3D
TUBB2B	CCT3

TUBB2B	TUBA4A
TUBB2B	TUBA3E
TUBB2B	CCT4
TUBB2B	CCT8
TUBB2B	CCT5
TUBB2B	TUBB4A
TUBB2B	ARL2
TUBB2B	TBCC
TUBB2B	TUBA1B
TXN2	PTBP3
TXN2	TOMM22
TXN2	MAP3K5
TXN2	TXNRD2
TXN2	SOD2
U2SURP	SF3A2
U2SURP	DDX42
UBE2G1	RBX1
UBE2G1	UBC
UBE2G1	PRKN
UBE2G1	UBE2G2
UBE2G1	SUMO1
UBE2G1	GPR37
UBE2G1	AMFR
UBE2H	UBA3
UBE2H	PRDX1
UBE2H	LACTB2
UBE2H	SEC23IP
UBE2H	TXNDC17
UBE2H	PRDX2
UBE2H	SSU72
UBE2H	TXNL1
UBE2J1	UBC
UBE2J1	SEL1L
UBE2J1	PRKN
UBE2J1	FAF2
UBE2J1	AUP1
UBE2J1	OS9
UBE2R2	RBX1
UBE2R2	UBC
UBE2R2	FBXW7
UBE2R2	SKP1
UBE2R2	CUL1
UBXN6	NSFL1C
UBXN6	VPS39
UBXN6	VCP
UBXN6	XPO6

UCP2	GHRL
UCP2	PPARA
UCP2	SIRT1
UCP2	PPARG
UCP2	UCP3
UCP2	NRF1
UCP2	UCP1
UNC5B	NEO1
UNC5B	DCC
UNC5B	CASP3
UNC5B	UNC5D
UNC5B	PIK3CA
UNC5B	NTN1
UNC5B	UNC5C
UNC5B	UNC5A
UNC5B	SLK
UNC5B	AGAP2
UNC5B	DAPK1
UNC5B	PIK3R1
USP28	GMPS
USP28	MYC
USP28	TP53BP1
USP28	USP21
USP28	UBC
USP28	SUMO3
USP28	RCHY1
USP30	ATXN7L3
USP30	USP1
VBP1	PFDN5
VBP1	PFDN6
VBP1	PFDN4
VBP1	PFDN2
VBP1	MSH4
VPS26B	SNX1
VPS26B	SNX2
VPS26B	MGAT3
VPS26B	VPS29
VPS26B	VPS35
VPS54	VPS52
VPS54	VPS53
VPS54	VPS51
VRK1	TP53BP1
VRK1	ATF2
VRK1	TP53
VRK1	PLK3
VRK1	BANF1

VRK1	ANKLE2
VRK1	JUN
WBP2	WBP1
WBP2	YAP1
WBP2	UBC
WDR1	TXNDC17
WDR1	TYMS
WDR1	TTN
WDR1	UBE2K
WDR1	UBC
WDR1	CFL2
WDR1	SH3GL1
WDR1	HABP4
WDR1	LPXN
WDR1	ACTA1
WDR1	VCL
WDR1	RHOU
WDR1	CAPZB
WDR1	BRPF3
WDR1	SVIL
WDR1	TUBA4A
WDR1	SH3PXD2A
WDR1	ACTA2
WDR1	SOD1
WDR1	AFAP1L1
WDR1	DSTN
WDR1	ACTR3
WDR1	TLN1
WDR1	PLEK
WDR1	ACTG2
WDR1	ACTG1
WDR1	HNRNPK
WDR1	CFL1
WDR1	TXN
WDR44	LSM7
WDR44	LSM4
WDR6	UBC
WDR6	STK11
XPO5	MPP4
XPO5	RANP1
XPO5	NUP214
XPO5	NUP153
XPO5	UBC
XPO5	TARBP2
XPO5	PRKRA
XPO5	DROSHA

XPO5	ZNF346
XPO5	DGCR8
XPO5	DICER1
XPO5	ZNF705E
XPO6	UBXN6
XPO6	RANP1
XPO6	PFN1
XPO6	VPS39
XPO7	VPS37B
XPO7	TLK2
XPO7	USP14
XPO7	XRN2
XPO7	EMB
XRCC2	RAD51B
XRCC2	SFR1
XRCC2	RAD51D
XRCC2	XRCC3
XRCC2	FANCB
XRCC2	RFFL
XRCC2	BRCA1
XRCC2	SWI5
XRCC2	RAD51C
YEATS2	POLE4
YEATS2	ZZZ3
YEATS2	KAT14
YEATS2	KAT2B
YEATS2	TADA2A
YEATS2	TADA3
YEATS2	TADA2B
YEATS2	WDR5
YEATS2	SGF29
YEATS2	POLE3
YEATS2	DR1
YEATS2	MBIP
YEATS2	KAT2A
YIF1A	YIPF7
YIF1A	YIPF5
YIF1A	VAPB
ZBTB18	TSN
ZBTB18	DNMT3A
ZC3H18	MRPL53
ZC3H18	TAGLN
ZC3H18	TIMM44
ZC3H18	NDUFA7
ZC3H18	MPLKIP
ZC3H18	USP10

ZC3H18	USP39
ZC3H18	ZC3H11A
ZC3H18	UBL4A
ZCCHC8	THOC6
ZCCHC8	ZC3H15
ZER1	COMMD1
ZER1	RBX1
ZER1	CUL2
ZER1	ELOB
ZFYVE26	KIF13A
ZFYVE26	AP5Z1
ZFYVE27	VAPA
ZFYVE27	SPAST
ZFYVE27	VAPB
ZFYVE27	FKBP8
ZMYND8	INTS1
ZMYND8	INTS6
ZMYND8	INTS5
ZNF385A	CDKN1A
ZNF385A	SFN
ZZZ3	TADA2A
ZZZ3	YEATS2
ZZZ3	KAT14
ZZZ3	SGF29

Supplementary Table 3. The score of DNB genes based on five priority criteria.

gene_id	gene_symbol	TF	PPI top100	KEGG	DEG	DEGs in neighboring genes
ENSG00000167972	ABCA3	0	0	9	1	1
ENSG00000103222	ABCC1	0	0	9	0	2
ENSG00000119688	ABCD4	0	0	18	1	1
ENSG00000033050	ABCF2	0	0	7	0	2
ENSG00000011198	ABHD5	0	0	120	1	2
ENSG00000138443	ABI2	0	1	191	0	2
ENSG00000075089	ACTR6	1	0	1	0	2
ENSG00000138316	ADAMTS14	0	0	0	1	0
ENSG00000173744	AGFG1	0	0	1	0	0
ENSG00000106351	AGFG2	0	0	2	1	1
ENSG00000155189	AGPAT5	0	0	21	0	4
ENSG00000158467	AHCYL2	0	0	17	1	3
ENSG00000135541	AHI1	0	0	4	1	1
ENSG00000106305	AIMP2	0	0	63	0	3
ENSG00000011243	AKAP8L	0	0	0	1	0
ENSG00000105186	ANKRD27	0	0	0	1	2
ENSG00000137494	ANKRD42	0	0	46	1	0
ENSG00000140350	ANP32A	1	1	33	0	0
ENSG00000117362	APH1A	0	0	12	0	1
ENSG00000149089	APIP	0	0	3	1	1
ENSG00000143595	AQP10	0	0	0	1	0
ENSG00000095139	ARCN1	0	0	6	0	1
ENSG00000171681	ATF7IP	1	0	15	1	1
ENSG00000126775	ATG14	0	0	47	1	2
ENSG00000069849	ATP1B3	0	0	61	0	2
ENSG00000143515	ATP8B2	0	0	0	1	0
ENSG00000148090	AUH	0	0	0	1	2
ENSG00000186318	BACE1	0	0	15	1	3
ENSG00000175334	BANF1	0	0	46	1	2
ENSG00000060982	BCAT1	0	0	36	1	3
ENSG00000129473	BCL2L2	0	0	213	1	2
ENSG00000133134	BEX2	0	0	41	0	3
ENSG00000166681	BEX3	0	0	76	1	2
ENSG00000185963	BICD2	0	0	3	1	2
ENSG00000104765	BNIP3L	0	0	160	1	3
ENSG00000254999	BRK1	0	0	462	0	5
ENSG00000095564	BTAF1	1	0	6	1	1
ENSG00000145741	BTF3	1	0	15	0	1
ENSG00000125149	C16orf70	0	0	0	1	1
ENSG00000135974	C2orf49	0	0	0	1	0
ENSG00000075461	CACNG4	0	1	138	0	4
ENSG00000151893	CACUL1	0	0	28	0	0
ENSG00000128595	CALU	0	0	2	0	1
ENSG00000131236	CAP1	0	0	72	0	2
ENSG00000116489	CAPZA1	0	1	21	0	2
ENSG00000108349	CASC3	0	0	227	0	8
ENSG00000108091	CCDC6	0	0	41	1	1
ENSG00000110092	CCND1	1	1	2734	0	59
ENSG00000221978	CCNL2	0	0	0	0	0
ENSG00000150753	CCT5	0	1	155	0	9
ENSG00000177697	CD151	0	1	56	0	3
ENSG00000167775	CD320	0	0	6	0	1

ENSG00000117335	CD46	0	0	9	0	1
ENSG00000135404	CD63	0	0	48	0	1
ENSG00000144354	CDCA7	0	0	33	1	2
ENSG00000065883	CDK13	1	0	30	1	1
ENSG00000156345	CDK20	0	0	8	1	1
ENSG00000101391	CDK5RAP1	0	0	5	0	0
ENSG00000091527	CDV3	0	0	7	0	2
ENSG00000106554	CHCHD3	1	0	0	0	0
ENSG00000080644	CHRNA3	0	0	10	1	3
ENSG00000163507	CIP2A	0	0	37	1	2
ENSG00000138433	CIR1	1	0	32	0	0
ENSG00000123975	CKS2	0	0	75	0	3
ENSG00000157224	CLDN12	0	0	24	1	1
ENSG00000106404	CLDN15	0	0	26	0	2
ENSG00000064666	CNN2	0	0	16	1	4
ENSG00000174469	CNTNAP2	0	0	0	1	1
ENSG00000168275	COA6	0	0	49	1	1
ENSG00000145781	COMMD10	0	0	0	0	1
ENSG00000169019	COMMD8	0	0	0	1	0
ENSG00000129083	COPB1	0	1	18	0	2
ENSG00000167193	CRK	1	1	1834	0	47
ENSG00000160741	CRTC2	1	1	126	1	2
ENSG00000009307	CSDE1	1	1	41	0	3
ENSG00000178971	CTC1	0	0	0	1	1
ENSG00000175826	CTDNEP1	0	1	279	0	8
ENSG00000136943	CTSV	0	0	4	1	0
ENSG00000174177	CTU2	0	0	0	1	1
ENSG00000107874	CUEDC2	0	0	64	0	2
ENSG00000167657	DAPK3	1	0	51	1	1
ENSG00000145041	DCAF1	1	0	8	1	1
ENSG00000198876	DCAF12	0	0	25	1	7
ENSG00000136485	DCAF7	0	1	32	0	10
ENSG00000198171	DDRGK1	0	0	0	0	0
ENSG00000079785	DDX1	1	0	95	0	4
ENSG00000124795	DEK	1	0	11	0	1
ENSG00000139726	DENR	0	0	6	0	1
ENSG00000114956	DGUOK	0	0	3	0	0
ENSG00000188985	DHFRP1	0	1	142	0	18
ENSG00000132153	DHX30	0	0	7	0	2
ENSG00000135164	DMTF1	1	0	95	0	3
ENSG00000105821	DNAJC2	1	0	2	1	1
ENSG00000119661	DNAL1	0	0	10	1	0
ENSG00000166171	DPCD	0	0	1	0	1
ENSG00000133884	DPF2	1	0	32	1	0
ENSG00000117505	DR1	1	0	17	1	5
ENSG00000175550	DRAP1	1	0	63	0	2
ENSG00000113360	DROSHA	0	1	74	0	6
ENSG00000143476	DTL	0	1	121	1	11
ENSG00000091073	DTX2	0	0	25	1	3
ENSG00000205250	E2F4	1	1	468	0	18
ENSG00000144597	EAF1	1	0	3	0	0
ENSG00000122882	ECD	1	0	20	1	0
ENSG00000130159	ECSIT	0	0	80	0	2
ENSG00000116406	EDEM3	0	0	3	1	0
ENSG00000132394	EEFSEC	0	0	0	1	0
ENSG00000134001	EIF2S1	0	1	331	0	19

ENSG00000107581	EIF3A	0	1	328	1	18
ENSG00000104408	EIF3E	0	0	189	0	9
ENSG00000084623	EIF3I	0	0	179	0	12
ENSG00000104131	EIF3J	0	0	182	1	11
ENSG00000100129	EIF3L	0	0	8	0	3
ENSG00000156976	EIF4A2	0	1	224	1	20
ENSG00000063046	EIF4B	0	1	284	0	16
ENSG00000184708	EIF4ENIF1	0	0	12	1	0
ENSG00000106682	EIF4H	0	0	1	0	2
ENSG00000100664	EIF5	0	0	179	1	10
ENSG00000158417	EIF5B	0	0	179	1	9
ENSG00000120690	ELF1	1	0	95	1	2
ENSG00000134759	ELP2	1	0	58	0	7
ENSG00000134153	EMC7	0	0	0	1	2
ENSG00000167136	ENDOG	0	0	8	0	1
ENSG00000143420	ENSA	0	0	15	0	0
ENSG00000105722	ERF	1	0	7	1	1
ENSG00000100632	ERH	0	0	4	1	1
ENSG00000099364	FBXL19	0	1	59	0	6
ENSG00000182325	FBXL6	0	0	19	0	0
ENSG00000183580	FBXL7	0	0	10	0	0
ENSG00000167196	FBXO22	0	0	17	1	1
ENSG00000174013	FBXO45	0	0	10	1	1
ENSG00000100225	FBXO7	0	0	20	0	0
ENSG00000119402	FBXW2	0	0	23	1	0
ENSG00000135723	FHOD1	1	0	70	1	0
ENSG00000168522	FNTA	0	0	55	1	0
ENSG00000136877	FPGS	0	0	12	0	1
ENSG00000087086	FTL	0	1	37	0	4
ENSG00000179163	FUCA1	0	0	0	1	2
ENSG00000114416	FXR1	0	0	10	0	2
ENSG00000129245	FXR2	0	0	3	1	1
ENSG00000178950	GAK	0	0	22	0	2
ENSG00000108479	GALK1	0	0	1	1	1
ENSG00000111640	GAPDH	0	1	541	0	12
ENSG00000006625	GGCT	0	0	16	0	3
ENSG00000137563	GGH	0	0	4	0	0
ENSG00000204120	GIGYF2	0	0	8	1	2
ENSG00000174842	GLMN	0	0	23	1	0
ENSG00000167414	GNG8	0	1	541	1	10
ENSG00000204590	GNL1	0	0	1	1	0
ENSG00000134697	GNL2	0	0	2	1	6
ENSG00000116906	GNPAT	0	0	1	0	1
ENSG00000047932	GOPC	0	0	12	1	1
ENSG00000125166	GOT2	0	1	109	0	14
ENSG00000132522	GPS2	1	0	56	0	1
ENSG00000233276	GPX1	0	1	167	0	8
ENSG00000180875	GREM2	0	0	4	1	1
ENSG00000103342	GSPT1	0	0	22	0	3
ENSG00000197265	GTF2E2	0	1	72	1	9
ENSG00000206527	HACD2	0	0	0	0	1
ENSG00000113196	HAND1	1	0	10	1	3
ENSG00000100084	HIRA	1	0	0	1	1
ENSG00000101294	HM13	0	0	7	0	2
ENSG00000137309	HMGA1	1	1	224	0	7
ENSG00000205581	HMGN1	0	0	8	0	1

ENSG00000197451	HNRNPAB	0	0	8	1	1
ENSG00000096746	HNRNPH3	0	0	12	1	3
ENSG00000153936	HS2ST1	0	0	0	0	2
ENSG00000185122	HSF1	1	1	379	1	11
ENSG00000125968	ID1	1	0	60	1	5
ENSG00000115738	ID2	1	0	136	1	6
ENSG00000154059	IMPACT	0	0	0	1	0
ENSG00000065150	IPO5	0	0	25	0	2
ENSG00000205339	IPO7	0	0	30	0	1
ENSG00000166086	JAM3	0	1	59	1	1
ENSG00000114982	KANSL3	0	0	2	0	1
ENSG00000065427	KARS1	0	1	11	0	3
ENSG00000136636	KCTD3	0	0	1	0	1
ENSG00000118193	KIF14	0	0	0	0	0
ENSG00000163808	KIF15	0	0	2	0	1
ENSG00000170759	KIF5B	0	0	75	1	5
ENSG00000172059	KLF11	1	0	28	1	0
ENSG00000183762	KREMEN1	0	0	11	1	1
ENSG00000001631	KRIT1	0	0	14	1	0
ENSG00000185896	LAMP1	0	0	50	0	1
ENSG00000196233	LCOR	1	0	11	0	0
ENSG00000100097	LGALS1	0	0	124	1	4
ENSG00000050405	LIMA1	0	0	50	1	1
ENSG00000124831	LRRFIP1	1	0	49	0	0
ENSG00000257103	LSM14A	0	0	8	0	1
ENSG00000130332	LSM7	0	1	31	0	9
ENSG00000007392	LUC7L	0	0	7	1	1
ENSG00000108848	LUC7L3	0	0	2	0	1
ENSG00000145220	LYAR	1	0	0	1	0
ENSG00000166963	MAP1A	0	0	9	1	2
ENSG00000162889	MAPKAPK2	0	0	116	1	2
ENSG00000116141	MARK1	0	0	4	0	3
ENSG00000007047	MARK4	0	0	29	1	5
ENSG00000168906	MAT2A	0	1	44	1	4
ENSG00000132031	MATN3	0	0	4	1	1
ENSG00000110492	MDK	0	0	43	1	2
ENSG00000153208	MERTK	0	0	96	0	1
ENSG00000037897	METTL1	0	0	0	0	2
ENSG00000145388	METTL14	0	0	0	1	0
ENSG00000152127	MGAT5	0	0	0	0	1
ENSG00000240972	MIF	0	1	143	0	10
ENSG00000125457	MIF4GD	0	0	2	1	0
ENSG00000148773	MKI67	0	0	6	0	1
ENSG00000130382	MLLT1	1	0	4	0	3
ENSG00000108788	MLX	1	0	17	1	4
ENSG00000168288	MMADHC	0	0	3	0	2
ENSG00000164172	MOCS2	0	0	4	0	0
ENSG00000115275	MOGS	0	0	11	1	1
ENSG00000061987	MON2	0	0	0	1	1
ENSG00000133030	MPRIP	0	0	54	0	1
ENSG00000143314	MRPL24	0	0	122	1	8
ENSG00000135900	MRPL44	0	0	6	1	2
ENSG00000136897	MRPL50	0	0	8	0	4
ENSG00000175110	MRPS22	0	0	1	0	1
ENSG00000102738	MRPS31	0	0	1	1	1
ENSG00000188895	MSL1	0	0	0	0	1

ENSG00000198804	MT-CO1	0	1	248	1	10
ENSG00000198938	MT-CO3	0	0	236	1	10
ENSG00000143033	MTF2	1	0	1	1	1
ENSG00000139505	MTMR6	0	0	1	1	3
ENSG00000104643	MTMR9	0	0	0	0	1
ENSG00000198786	MT-ND5	0	1	558	1	14
ENSG00000198695	MT-ND6	0	1	533	1	9
ENSG00000128654	MTX2	0	0	3	1	1
ENSG00000172766	NAA16	0	0	0	0	1
ENSG00000196531	NACA	1	0	9	0	1
ENSG00000136274	NACAD	0	0	0	0	0
ENSG00000124357	NAGK	0	0	0	1	1
ENSG00000134265	NAPG	0	0	2	0	0
ENSG00000107130	NCS1	0	0	7	0	0
ENSG00000162139	NEU3	0	0	48	0	5
ENSG00000196290	NIF3L1	1	0	2	0	0
ENSG00000136448	NMT1	0	0	50	0	0
ENSG00000166197	NOLC1	0	0	14	0	1
ENSG00000107281	NPDC1	0	0	6	0	1
ENSG00000181019	NQO1	0	0	5	1	1
ENSG00000013374	NUB1	0	0	10	0	0
ENSG00000065154	OAT	0	1	26	0	4
ENSG00000115758	ODC1	0	1	381	1	8
ENSG00000081087	OSTM1	0	0	0	1	0
ENSG00000120137	PANK3	0	0	2	0	2
ENSG00000157881	PANK4	0	0	2	1	2
ENSG00000167081	PBX3	1	0	0	0	1
ENSG00000100889	PCK2	0	0	198	1	2
ENSG00000106333	PCOLCE	0	0	4	0	3
ENSG00000155660	PDIA4	0	0	37	0	2
ENSG00000163110	PDLIM5	0	0	8	1	0
ENSG00000090857	PDPR	0	0	6	0	1
ENSG00000088356	PDRG1	0	0	0	1	0
ENSG00000089220	PEBP1	0	0	308	0	6
ENSG00000242265	PEG10	0	0	39	1	3
ENSG00000008438	PGLYRP1	0	0	1	1	0
ENSG00000101856	PGRMC1	0	0	1	0	1
ENSG00000215021	PHB2	0	1	37	0	2
ENSG00000112511	PHF1	1	0	5	0	1
ENSG00000130024	PHF10	1	0	24	0	1
ENSG00000109132	PHOX2B	1	0	0	1	2
ENSG00000087111	PIGS	0	0	1	0	5
ENSG00000101464	PIGU	0	0	1	1	4
ENSG00000087842	PIR	1	0	74	1	1
ENSG00000174238	PITPNA	0	0	40	0	0
ENSG00000141682	PMAIP1	0	1	288	1	8
ENSG00000163344	PMVK	0	0	17	1	3
ENSG00000256525	POLG2	0	0	0	0	0
ENSG00000085998	POMGNT1	0	0	0	0	1
ENSG00000130714	POMT1	0	0	5	1	0
ENSG00000196262	PPIA	0	1	225	0	2
ENSG00000173457	PPP1R14B	0	0	5	1	0
ENSG00000154845	PPP4R1	0	0	1	0	0
ENSG00000100796	PPP4R3A	0	0	2	0	0
ENSG00000170325	PRDM10	1	0	8	1	0
ENSG00000110851	PRDM4	1	0	39	0	1

ENSG00000117592	PRDX6	0	0	4	0	0
ENSG00000085377	PREP	0	0	11	0	1
ENSG00000131791	PRKAB2	0	0	68	1	0
ENSG00000185246	PRPF39	0	1	15	1	6
ENSG00000161542	PRPSAP1	0	0	4	0	0
ENSG00000135069	PSAT1	0	0	14	1	2
ENSG00000164985	PSIP1	1	0	26	0	3
ENSG00000156471	PTDSS1	0	1	92	1	9
ENSG00000187514	PTMA	0	1	95	1	4
ENSG00000076201	PTPN23	0	0	119	1	1
ENSG00000105426	PTPRS	0	0	2	0	1
ENSG00000150787	PTS	0	0	4	0	2
ENSG00000146676	PURB	1	0	1	1	2
ENSG00000183010	PYCR1	0	0	11	1	3
ENSG00000103485	QPRT	0	0	13	1	6
ENSG00000090565	RAB11FIP3	0	0	19	0	1
ENSG00000080371	RAB21	0	0	0	1	1
ENSG00000112210	RAB23	0	0	5	1	1
ENSG00000134594	RAB33A	0	0	3	0	1
ENSG00000111737	RAB35	0	0	7	0	1
ENSG00000118873	RAB3GAP2	0	0	10	1	4
ENSG00000029725	RABEP1	0	1	21	1	2
ENSG00000177548	RABEP2	0	0	8	1	0
ENSG00000204628	RACK1	0	1	723	0	18
ENSG00000125249	RAP2A	0	0	15	1	3
ENSG00000091428	RAPGEF4	0	0	183	1	8
ENSG00000023287	RB1CC1	0	0	173	1	3
ENSG00000182872	RBM10	0	0	14	0	0
ENSG00000265241	RBM8A	0	0	13	0	1
ENSG00000179051	RCC2	0	1	90	1	25
ENSG00000117906	RCN2	0	0	29	0	0
ENSG00000167771	RCOR2	1	0	12	1	0
ENSG00000072042	RDH11	0	0	6	0	1
ENSG00000165731	RET	0	1	1146	1	18
ENSG00000169733	RFNG	0	0	26	1	3
ENSG00000166532	RIMKLB	0	0	3	1	0
ENSG00000204227	RING1	1	0	0	0	0
ENSG00000013561	RNF14	1	0	2	1	2
ENSG00000011275	RNF216	0	0	27	0	1
ENSG00000181852	RNF41	0	0	145	1	6
ENSG00000198963	RORB	1	1	58	1	13
ENSG00000197713	RPE	0	0	8	1	2
ENSG00000198755	RPL10A	0	0	326	0	26
ENSG00000167526	RPL13	0	1	328	0	32
ENSG00000108298	RPL19	0	0	308	0	26
ENSG00000131469	RPL27	0	0	296	0	23
ENSG00000100316	RPL3	0	1	335	0	27
ENSG00000136942	RPL35	0	1	330	0	29
ENSG00000130255	RPL36	0	0	301	0	24
ENSG00000089009	RPL6	0	0	298	0	23
ENSG00000161016	RPL8	0	1	322	0	26
ENSG00000177600	RPLP2	0	0	294	0	23
ENSG00000178718	RPP25	0	0	0	1	2
ENSG00000142534	RPS11	0	1	317	0	26
ENSG00000112306	RPS12	0	0	316	0	26
ENSG00000115268	RPS15	0	1	325	0	27

ENSG00000105193	RPS16	0	0	314	0	24
ENSG00000105372	RPS19	0	0	326	0	25
ENSG00000140988	RPS2	0	1	330	0	26
ENSG00000008988	RPS20	0	1	322	0	26
ENSG00000171858	RPS21	0	0	299	0	23
ENSG00000177954	RPS27	0	1	379	0	49
ENSG00000198034	RPS4X	0	0	299	0	24
ENSG00000136643	RPS6KC1	0	0	6	0	1
ENSG00000142937	RPS8	0	0	298	0	24
ENSG00000168028	RPSA	0	1	376	0	27
ENSG00000067533	RRP15	0	0	0	0	0
ENSG00000100220	RTCB	0	0	6	0	2
ENSG00000133318	RTN3	0	0	3	0	1
ENSG00000075856	SART3	0	0	17	1	6
ENSG00000141504	SAT2	0	0	38	1	3
ENSG00000171951	SCG2	0	1	260	1	11
ENSG00000107651	SEC23IP	0	0	28	0	2
ENSG00000113615	SEC24A	0	1	51	1	6
ENSG00000008952	SEC62	0	0	14	0	1
ENSG00000025796	SEC63	0	0	35	0	3
ENSG00000137872	SEMA6D	0	0	2	1	1
ENSG00000079387	SENP1	1	0	17	0	0
ENSG00000142864	SERBP1	0	0	88	0	2
ENSG00000188488	SERPINA5	0	0	14	1	1
ENSG00000132386	SERPINF1	0	0	32	1	1
ENSG00000107290	SETX	0	0	10	1	2
ENSG00000116560	SFPQ	1	0	21	1	2
ENSG00000104332	SFRP1	0	0	193	1	6
ENSG00000164466	SFXN1	0	0	1	0	0
ENSG00000156398	SFXN2	0	0	1	1	0
ENSG00000163069	SGCB	0	0	27	0	1
ENSG00000146414	SHPRH	0	0	5	1	1
ENSG00000144736	SHQ1	0	0	0	1	1
ENSG00000147955	SIGMAR1	0	0	8	1	1
ENSG00000147454	SLC25A37	0	0	0	1	1
ENSG00000169100	SLC25A6	0	1	65	0	3
ENSG00000152683	SLC30A6	0	0	0	0	1
ENSG00000169359	SLC33A1	0	0	0	1	0
ENSG00000157593	SLC35B2	0	0	0	0	1
ENSG00000116704	SLC35D1	0	0	0	1	2
ENSG00000129353	SLC44A2	0	0	3	1	0
ENSG00000138074	SLC5A6	0	0	17	0	3
ENSG00000084070	SMAP2	0	1	34	1	9
ENSG00000157106	SMG1	0	1	259	1	9
ENSG00000198952	SMG5	0	1	296	1	6
ENSG00000116698	SMG7	0	0	224	1	6
ENSG00000125835	SNRPB	0	1	179	0	32
ENSG00000129128	SPCS3	0	0	200	0	9
ENSG00000134278	SPIRE1	0	0	0	1	0
ENSG00000153914	SREK1	0	0	4	0	1
ENSG00000140319	SRP14	0	0	177	0	7
ENSG00000167881	SRP68	0	0	177	1	6
ENSG00000174780	SRP72	0	0	177	0	7
ENSG00000096063	SRPK1	0	0	16	1	5
ENSG00000154548	SRSF12	0	0	4	1	0
ENSG00000141298	SSH2	0	0	10	1	0

ENSG00000172830	SSH3	0	0	10	0	0
ENSG00000117155	SSX2IP	0	0	10	1	0
ENSG00000124214	STAU1	0	0	14	1	0
ENSG00000023734	STRAP	1	1	244	0	12
ENSG00000163527	STT3B	0	0	12	0	5
ENSG00000152455	SUV39H2	1	0	0	0	2
ENSG00000011347	SYT7	0	0	1	1	1
ENSG00000158710	TAGLN2	0	0	8	0	1
ENSG00000120948	TARDBP	0	1	54	1	3
ENSG00000121068	TBX2	1	0	3	0	1
ENSG00000135111	TBX3	1	0	30	0	1
ENSG00000140262	TCF12	1	0	7	1	3
ENSG00000111802	TDP2	1	0	200	1	4
ENSG00000068323	TFE3	1	0	72	0	0
ENSG00000150779	TIMM8B	0	0	6	0	2
ENSG00000115297	TLX2	1	0	24	0	2
ENSG00000086598	TMED2	0	0	14	0	2
ENSG00000112697	TMEM30A	0	0	6	0	3
ENSG00000116857	TMEM9	0	0	0	1	1
ENSG00000120802	TMPO	0	0	11	0	3
ENSG00000006327	TNFRSF12A	0	0	25	1	0
ENSG00000173726	TOMM20	0	1	70	0	6
ENSG00000115129	TP53I3	0	0	58	1	0
ENSG00000167460	TPM4	0	1	123	1	17
ENSG00000164548	TRA2A	0	0	9	1	0
ENSG00000131653	TRAF7	0	0	12	0	1
ENSG00000113595	TRIM23	0	0	46	1	0
ENSG00000174173	TRMT10C	0	0	0	0	1
ENSG00000126814	TRMT5	0	0	0	0	1
ENSG00000182173	TSEN54	0	0	0	1	1
ENSG00000137285	TUBB2B	0	1	146	0	7
ENSG00000100348	TXN2	0	0	30	0	2
ENSG00000163714	U2SURP	0	0	3	0	1
ENSG00000132388	UBE2G1	0	0	37	0	2
ENSG00000186591	UBE2H	0	0	4	1	0
ENSG00000198833	UBE2J1	0	0	18	0	2
ENSG00000107341	UBE2R2	0	0	39	0	1
ENSG00000167671	UBXN6	0	0	8	1	0
ENSG00000175567	UCP2	0	0	34	1	2
ENSG00000107731	UNC5B	0	0	244	1	4
ENSG00000048028	USP28	0	0	42	1	3
ENSG00000135093	USP30	0	0	1	1	1
ENSG00000155959	VBP1	0	0	0	0	0
ENSG00000151502	VPS26B	0	0	5	0	0
ENSG00000143952	VPS54	0	0	0	1	2
ENSG00000100749	VRK1	0	0	123	1	5
ENSG00000132471	WBP2	1	0	7	0	2
ENSG00000071127	WDR1	0	1	97	1	10
ENSG00000131725	WDR44	0	0	4	1	1
ENSG00000178252	WDR6	0	0	14	0	1
ENSG00000124571	XPO5	0	1	15	1	4
ENSG00000169180	XPO6	0	0	8	0	1
ENSG00000130227	XPO7	0	0	3	0	0
ENSG00000196584	XRCC2	0	0	5	1	3
ENSG00000163872	YEATS2	1	0	14	1	6
ENSG00000174851	YIF1A	0	0	2	0	0

ENSG00000179456	ZBTB18	1	0	2	0	0
ENSG00000158545	ZC3H18	0	0	13	0	3
ENSG00000033030	ZCCHC8	0	0	1	1	0
ENSG00000160445	ZER1	0	0	19	0	0
ENSG00000072121	ZFYVE26	0	0	0	0	0
ENSG00000155256	ZFYVE27	0	0	3	1	1
ENSG00000101040	ZMYND8	1	0	0	1	0
ENSG00000161642	ZNF385A	1	0	41	1	1
ENSG00000036549	ZZZ3	1	0	0	1	3

Supplementary Table 4. Transcription factor binding sites for HSF1 in the 2000 bp sequence upstream of the *SERPINE1* gene.

Matrix ID	Name	Score	Relative score	Start	End	Strand	Predicted sequence
MA0486.2	MA0486.2.HSF1	9.819874	0.846839638	893	905	-	ttcgggatgattc
MA0486.1	MA0486.1.HSF1	10.21667	0.82964658	1787	1801	+	gttcctgaatgctct
MA0486.2	MA0486.2.HSF1	7.573777	0.818483174	663	675	+	ttctaaactgttc

Supplementary Table 5. The primer pairs of *β-actin* , *HSF1* , *MAPKAPK2* and *SERPINE1* .

Gene	Primer	Primer sequence
β-actin	Forward Primer	5'-CATGTACGTTGCTATCCAGGC-3'
	Reverse Primer	5'-CTCCTTAATGTCACGCACGAT-3'
HSF1	Forward Primer	5'-CCATGAAGCATGAGAATGAGGC-3'
	Reverse Primer	5'-CTTGTTGACGACTTTCTGTTGC-3'
MAPKAPK2	Forward Primer	5'-CCCACCCAGAGAATGACCATC-3'
	Reverse Primer	5'-ACAGAATCCTCTGCTCACAACC-3'
SERPINE1	Forward Primer	5'-CAGACCAAGAGCCTCTCCAC-3'
	Reverse Primer	5'-ATCACTTGGCCCATGAAAAG-3'

Supplementary Table 6. Overview of the GEO datasets used.

Dataset	GEO accession	Disease	Tissue	Platform	data processing	Sample composition
GSE20292	GSE20292	PD	substantia nigra	GPL96	Affymetrix Human Genome U133A Array	11PD and 18HC
GSE68719	GSE68719	PD	prefrontal cortex	GPL11154	Illumina HiSeq 2000 (Homo sapiens)	29PD and 44HC
PD-peripheral blood dataset	GSE6613	PD	peripheral blood	GPL96	Affymetrix Human Genome U133A Array	50PD and 22HC
PD-peripheral blood dataset	GSE72267	PD	peripheral blood	GPL571	Affymetrix Human Genome U133A 2.0 Array	40PD and 19HC
PD-peripheral blood dataset	GSE99039	PD	peripheral blood	GPL570	Affymetrix Human Genome U133 Plus 2.0 Array	205PD and 233HC
PD-peripheral blood dataset	GSE100054	PD	peripheral blood	GPL23126	Affymetrix Human Clariom D Assay	10PD and 9HC
GSE150696	GSE150696	DLB	prefrontal cortex	GPL17585	Affymetrix Human Transcriptome Array 2.0	12DLB and 9HC
GSE199258	GSE199258	MSA	cerebellar white matter	GPL16791	Illumina HiSeq 2500 (Homo sapiens)	19MSA and 19HC

Supplementary Table 7. Substantial nigra sample's informations from GSE20292.

Sample accession	disease state	Tissue	Age	Gender	Platform	GEO accession	data_processing
GSM508708	control	substantia nigra	57	male	GPL96	GSE20292	Affymetrix Human Genome U133A Array
GSM508710	PD	substantia nigra	73	male	GPL96	GSE20292	Affymetrix Human Genome U133A Array
GSM508711	PD	substantia nigra	80	female	GPL96	GSE20292	Affymetrix Human Genome U133A Array
GSM508712	PD	substantia nigra	84	female	GPL96	GSE20292	Affymetrix Human Genome U133A Array
GSM508713	PD	substantia nigra	70	female	GPL96	GSE20292	Affymetrix Human Genome U133A Array
GSM508714	PD	substantia nigra	82	female	GPL96	GSE20292	Affymetrix Human Genome U133A Array
GSM508715	PD	substantia nigra	70	male	GPL96	GSE20292	Affymetrix Human Genome U133A Array
GSM508716	PD	substantia nigra	80	male	GPL96	GSE20292	Affymetrix Human Genome U133A Array
GSM508717	control	substantia nigra	94	female	GPL96	GSE20292	Affymetrix Human Genome U133A Array
GSM508718	PD	substantia nigra	70	male	GPL96	GSE20292	Affymetrix Human Genome U133A Array
GSM508720	control	substantia nigra	79	male	GPL96	GSE20292	Affymetrix Human Genome U133A Array
GSM508721	control	substantia nigra	67	male	GPL96	GSE20292	Affymetrix Human Genome U133A Array
GSM508722	control	substantia nigra	54	male	GPL96	GSE20292	Affymetrix Human Genome U133A Array
GSM508723	control	substantia nigra	73	female	GPL96	GSE20292	Affymetrix Human Genome U133A Array
GSM508724	control	substantia nigra	82	male	GPL96	GSE20292	Affymetrix Human Genome U133A Array
GSM508725	control	substantia nigra	72	female	GPL96	GSE20292	Affymetrix Human Genome U133A Array
GSM508726	control	substantia nigra	73	female	GPL96	GSE20292	Affymetrix Human Genome U133A Array
GSM508728	PD	substantia nigra	75	male	GPL96	GSE20292	Affymetrix Human Genome U133A Array
GSM508729	control	substantia nigra	74	male	GPL96	GSE20292	Affymetrix Human Genome U133A Array
GSM508730	control	substantia nigra	72	female	GPL96	GSE20292	Affymetrix Human Genome U133A Array
GSM508731	PD	substantia nigra	79	female	GPL96	GSE20292	Affymetrix Human Genome U133A Array
GSM508732	PD	substantia nigra	67	male	GPL96	GSE20292	Affymetrix Human Genome U133A Array
GSM508733	control	substantia nigra	75	male	GPL96	GSE20292	Affymetrix Human Genome U133A Array
GSM508734	control	substantia nigra	81	male	GPL96	GSE20292	Affymetrix Human Genome U133A Array
GSM508735	control	substantia nigra	55	male	GPL96	GSE20292	Affymetrix Human Genome U133A Array
GSM521253	control	substantia nigra	59	male	GPL96	GSE20292	Affymetrix Human Genome U133A Array
GSM606624	control	substantia nigra	41	male	GPL96	GSE20292	Affymetrix Human Genome U133A Array
GSM606625	control	substantia nigra	42	male	GPL96	GSE20292	Affymetrix Human Genome U133A Array
GSM606626	control	substantia nigra	53	male	GPL96	GSE20292	Affymetrix Human Genome U133A Array

Supplementary Table 8. Prefrontal cortex sample's informations from GSE68719.

Sample accession	disease state	Tissue	Age	Gender	Platform	GEO accession	data_processing
GSM1679648	control	prefrontal cortex	73	male	GPL11154	GSE68719	Illumina HiSeq 2000 (Homo sapiens)
GSM1679649	control	prefrontal cortex	91	male	GPL11154	GSE68719	Illumina HiSeq 2000 (Homo sapiens)
GSM1679650	control	prefrontal cortex	82	male	GPL11154	GSE68719	Illumina HiSeq 2000 (Homo sapiens)
GSM1679651	control	prefrontal cortex	97	male	GPL11154	GSE68719	Illumina HiSeq 2000 (Homo sapiens)
GSM1679652	control	prefrontal cortex	86	male	GPL11154	GSE68719	Illumina HiSeq 2000 (Homo sapiens)
GSM1679653	control	prefrontal cortex	91	male	GPL11154	GSE68719	Illumina HiSeq 2000 (Homo sapiens)
GSM1679654	control	prefrontal cortex	81	male	GPL11154	GSE68719	Illumina HiSeq 2000 (Homo sapiens)
GSM1679655	control	prefrontal cortex	79	male	GPL11154	GSE68719	Illumina HiSeq 2000 (Homo sapiens)
GSM1679656	control	prefrontal cortex	63	male	GPL11154	GSE68719	Illumina HiSeq 2000 (Homo sapiens)
GSM1679657	control	prefrontal cortex	66	male	GPL11154	GSE68719	Illumina HiSeq 2000 (Homo sapiens)
GSM1679658	control	prefrontal cortex	69	male	GPL11154	GSE68719	Illumina HiSeq 2000 (Homo sapiens)
GSM1679659	control	prefrontal cortex	79	male	GPL11154	GSE68719	Illumina HiSeq 2000 (Homo sapiens)
GSM1679660	control	prefrontal cortex	61	male	GPL11154	GSE68719	Illumina HiSeq 2000 (Homo sapiens)
GSM1679661	control	prefrontal cortex	58	male	GPL11154	GSE68719	Illumina HiSeq 2000 (Homo sapiens)
GSM1679662	control	prefrontal cortex	70	male	GPL11154	GSE68719	Illumina HiSeq 2000 (Homo sapiens)
GSM1679663	control	prefrontal cortex	66	male	GPL11154	GSE68719	Illumina HiSeq 2000 (Homo sapiens)
GSM1679664	control	prefrontal cortex	73	male	GPL11154	GSE68719	Illumina HiSeq 2000 (Homo sapiens)
GSM1679665	control	prefrontal cortex	60	male	GPL11154	GSE68719	Illumina HiSeq 2000 (Homo sapiens)
GSM1679666	control	prefrontal cortex	76	male	GPL11154	GSE68719	Illumina HiSeq 2000 (Homo sapiens)
GSM1679667	control	prefrontal cortex	61	male	GPL11154	GSE68719	Illumina HiSeq 2000 (Homo sapiens)
GSM1679668	control	prefrontal cortex	62	male	GPL11154	GSE68719	Illumina HiSeq 2000 (Homo sapiens)
GSM1679669	control	prefrontal cortex	69	male	GPL11154	GSE68719	Illumina HiSeq 2000 (Homo sapiens)
GSM1679670	control	prefrontal cortex	61	male	GPL11154	GSE68719	Illumina HiSeq 2000 (Homo sapiens)
GSM1679671	control	prefrontal cortex	88	male	GPL11154	GSE68719	Illumina HiSeq 2000 (Homo sapiens)
GSM1679672	control	prefrontal cortex	93	male	GPL11154	GSE68719	Illumina HiSeq 2000 (Homo sapiens)
GSM1679673	control	prefrontal cortex	53	male	GPL11154	GSE68719	Illumina HiSeq 2000 (Homo sapiens)
GSM1679674	control	prefrontal cortex	57	male	GPL11154	GSE68719	Illumina HiSeq 2000 (Homo sapiens)
GSM1679675	control	prefrontal cortex	46	male	GPL11154	GSE68719	Illumina HiSeq 2000 (Homo sapiens)
GSM1679676	control	prefrontal cortex	57	male	GPL11154	GSE68719	Illumina HiSeq 2000 (Homo sapiens)
GSM1679677	control	prefrontal cortex	80	male	GPL11154	GSE68719	Illumina HiSeq 2000 (Homo sapiens)
GSM1679678	control	prefrontal cortex	74	male	GPL11154	GSE68719	Illumina HiSeq 2000 (Homo sapiens)
GSM1679679	control	prefrontal cortex	69	male	GPL11154	GSE68719	Illumina HiSeq 2000 (Homo sapiens)
GSM1679680	control	prefrontal cortex	76	male	GPL11154	GSE68719	Illumina HiSeq 2000 (Homo sapiens)
GSM1679681	control	prefrontal cortex	78	male	GPL11154	GSE68719	Illumina HiSeq 2000 (Homo sapiens)
GSM1679682	control	prefrontal cortex	87	male	GPL11154	GSE68719	Illumina HiSeq 2000 (Homo sapiens)
GSM1679683	control	prefrontal cortex	86	male	GPL11154	GSE68719	Illumina HiSeq 2000 (Homo sapiens)
GSM1679684	control	prefrontal cortex	54	male	GPL11154	GSE68719	Illumina HiSeq 2000 (Homo sapiens)
GSM1679685	control	prefrontal cortex	68	male	GPL11154	GSE68719	Illumina HiSeq 2000 (Homo sapiens)
GSM1679686	control	prefrontal cortex	52	male	GPL11154	GSE68719	Illumina HiSeq 2000 (Homo sapiens)
GSM1679687	control	prefrontal cortex	46	male	GPL11154	GSE68719	Illumina HiSeq 2000 (Homo sapiens)
GSM1679688	control	prefrontal cortex	55	male	GPL11154	GSE68719	Illumina HiSeq 2000 (Homo sapiens)
GSM1679689	control	prefrontal cortex	57	male	GPL11154	GSE68719	Illumina HiSeq 2000 (Homo sapiens)
GSM1679690	control	prefrontal cortex	66	male	GPL11154	GSE68719	Illumina HiSeq 2000 (Homo sapiens)
GSM1679691	control	prefrontal cortex	64	male	GPL11154	GSE68719	Illumina HiSeq 2000 (Homo sapiens)
GSM1679692	PD	prefrontal cortex	74	male	GPL11154	GSE68719	Illumina HiSeq 2000 (Homo sapiens)
GSM1679693	PD	prefrontal cortex	83	male	GPL11154	GSE68719	Illumina HiSeq 2000 (Homo sapiens)
GSM1679694	PD	prefrontal cortex	80	male	GPL11154	GSE68719	Illumina HiSeq 2000 (Homo sapiens)
GSM1679695	PD	prefrontal cortex	83	male	GPL11154	GSE68719	Illumina HiSeq 2000 (Homo sapiens)
GSM1679696	PD	prefrontal cortex	80	male	GPL11154	GSE68719	Illumina HiSeq 2000 (Homo sapiens)
GSM1679697	PD	prefrontal cortex	84	male	GPL11154	GSE68719	Illumina HiSeq 2000 (Homo sapiens)
GSM1679698	PD	prefrontal cortex	88	male	GPL11154	GSE68719	Illumina HiSeq 2000 (Homo sapiens)
GSM1679699	PD	prefrontal cortex	81	male	GPL11154	GSE68719	Illumina HiSeq 2000 (Homo sapiens)
GSM1679700	PD	prefrontal cortex	77	male	GPL11154	GSE68719	Illumina HiSeq 2000 (Homo sapiens)
GSM1679701	PD	prefrontal cortex	64	male	GPL11154	GSE68719	Illumina HiSeq 2000 (Homo sapiens)
GSM1679702	PD	prefrontal cortex	85	male	GPL11154	GSE68719	Illumina HiSeq 2000 (Homo sapiens)
GSM1679703	PD	prefrontal cortex	94	male	GPL11154	GSE68719	Illumina HiSeq 2000 (Homo sapiens)
GSM1679704	PD	prefrontal cortex	80	male	GPL11154	GSE68719	Illumina HiSeq 2000 (Homo sapiens)
GSM1679705	PD	prefrontal cortex	85	male	GPL11154	GSE68719	Illumina HiSeq 2000 (Homo sapiens)
GSM1679706	PD	prefrontal cortex	75	male	GPL11154	GSE68719	Illumina HiSeq 2000 (Homo sapiens)
GSM1679707	PD	prefrontal cortex	74	male	GPL11154	GSE68719	Illumina HiSeq 2000 (Homo sapiens)
GSM1679708	PD	prefrontal cortex	89	male	GPL11154	GSE68719	Illumina HiSeq 2000 (Homo sapiens)
GSM1679709	PD	prefrontal cortex	66	male	GPL11154	GSE68719	Illumina HiSeq 2000 (Homo sapiens)
GSM1679710	PD	prefrontal cortex	65	male	GPL11154	GSE68719	Illumina HiSeq 2000 (Homo sapiens)
GSM1679711	PD	prefrontal cortex	85	male	GPL11154	GSE68719	Illumina HiSeq 2000 (Homo sapiens)
GSM1679712	PD	prefrontal cortex	64	male	GPL11154	GSE68719	Illumina HiSeq 2000 (Homo sapiens)
GSM1679713	PD	prefrontal cortex	64	male	GPL11154	GSE68719	Illumina HiSeq 2000 (Homo sapiens)
GSM1679714	PD	prefrontal cortex	75	male	GPL11154	GSE68719	Illumina HiSeq 2000 (Homo sapiens)
GSM1679715	PD	prefrontal cortex	68	male	GPL11154	GSE68719	Illumina HiSeq 2000 (Homo sapiens)
GSM1679716	PD	prefrontal cortex	95	male	GPL11154	GSE68719	Illumina HiSeq 2000 (Homo sapiens)
GSM1679717	PD	prefrontal cortex	74	male	GPL11154	GSE68719	Illumina HiSeq 2000 (Homo sapiens)
GSM1679718	PD	prefrontal cortex	68	male	GPL11154	GSE68719	Illumina HiSeq 2000 (Homo sapiens)
GSM1679719	PD	prefrontal cortex	79	male	GPL11154	GSE68719	Illumina HiSeq 2000 (Homo sapiens)
GSM1679720	PD	prefrontal cortex	70	male	GPL11154	GSE68719	Illumina HiSeq 2000 (Homo sapiens)

Supplementary Table 9. Peripheral blood sample's informations from PD-peripheral blood dataset.

Sample accession	disease state	Tissue	Age	Gender	Sample accession	Platform	data_processing	Integrated as data set
GSM153404	PD	peripheral blood	NA	NA	GSE6613	GPL96	Affymetrix Human Genome U133A Array	PD-peripheral blood dataset
GSM153405	control	peripheral blood	NA	NA	GSE6613	GPL96	Affymetrix Human Genome U133A Array	PD-peripheral blood dataset
GSM153406	control	peripheral blood	NA	NA	GSE6613	GPL96	Affymetrix Human Genome U133A Array	PD-peripheral blood dataset
GSM153407	PD	peripheral blood	NA	NA	GSE6613	GPL96	Affymetrix Human Genome U133A Array	PD-peripheral blood dataset
GSM153408	PD	peripheral blood	NA	NA	GSE6613	GPL96	Affymetrix Human Genome U133A Array	PD-peripheral blood dataset
GSM153410	PD	peripheral blood	NA	NA	GSE6613	GPL96	Affymetrix Human Genome U133A Array	PD-peripheral blood dataset
GSM153411	PD	peripheral blood	NA	NA	GSE6613	GPL96	Affymetrix Human Genome U133A Array	PD-peripheral blood dataset
GSM153412	PD	peripheral blood	NA	NA	GSE6613	GPL96	Affymetrix Human Genome U133A Array	PD-peripheral blood dataset
GSM153413	PD	peripheral blood	NA	NA	GSE6613	GPL96	Affymetrix Human Genome U133A Array	PD-peripheral blood dataset
GSM153414	PD	peripheral blood	NA	NA	GSE6613	GPL96	Affymetrix Human Genome U133A Array	PD-peripheral blood dataset
GSM153415	PD	peripheral blood	NA	NA	GSE6613	GPL96	Affymetrix Human Genome U133A Array	PD-peripheral blood dataset
GSM153416	PD	peripheral blood	NA	NA	GSE6613	GPL96	Affymetrix Human Genome U133A Array	PD-peripheral blood dataset
GSM153417	PD	peripheral blood	NA	NA	GSE6613	GPL96	Affymetrix Human Genome U133A Array	PD-peripheral blood dataset
GSM153418	PD	peripheral blood	NA	NA	GSE6613	GPL96	Affymetrix Human Genome U133A Array	PD-peripheral blood dataset
GSM153419	control	peripheral blood	NA	NA	GSE6613	GPL96	Affymetrix Human Genome U133A Array	PD-peripheral blood dataset
GSM153420	PD	peripheral blood	NA	NA	GSE6613	GPL96	Affymetrix Human Genome U133A Array	PD-peripheral blood dataset
GSM153421	PD	peripheral blood	NA	NA	GSE6613	GPL96	Affymetrix Human Genome U133A Array	PD-peripheral blood dataset
GSM153422	PD	peripheral blood	NA	NA	GSE6613	GPL96	Affymetrix Human Genome U133A Array	PD-peripheral blood dataset
GSM153423	control	peripheral blood	NA	NA	GSE6613	GPL96	Affymetrix Human Genome U133A Array	PD-peripheral blood dataset
GSM153424	PD	peripheral blood	NA	NA	GSE6613	GPL96	Affymetrix Human Genome U133A Array	PD-peripheral blood dataset
GSM153425	control	peripheral blood	NA	NA	GSE6613	GPL96	Affymetrix Human Genome U133A Array	PD-peripheral blood dataset
GSM153427	control	peripheral blood	NA	NA	GSE6613	GPL96	Affymetrix Human Genome U133A Array	PD-peripheral blood dataset
GSM153428	control	peripheral blood	NA	NA	GSE6613	GPL96	Affymetrix Human Genome U133A Array	PD-peripheral blood dataset
GSM153429	control	peripheral blood	NA	NA	GSE6613	GPL96	Affymetrix Human Genome U133A Array	PD-peripheral blood dataset
GSM153430	PD	peripheral blood	NA	NA	GSE6613	GPL96	Affymetrix Human Genome U133A Array	PD-peripheral blood dataset
GSM153432	PD	peripheral blood	NA	NA	GSE6613	GPL96	Affymetrix Human Genome U133A Array	PD-peripheral blood dataset
GSM153433	control	peripheral blood	NA	NA	GSE6613	GPL96	Affymetrix Human Genome U133A Array	PD-peripheral blood dataset
GSM153434	PD	peripheral blood	NA	NA	GSE6613	GPL96	Affymetrix Human Genome U133A Array	PD-peripheral blood dataset
GSM153435	PD	peripheral blood	NA	NA	GSE6613	GPL96	Affymetrix Human Genome U133A Array	PD-peripheral blood dataset
GSM153436	PD	peripheral blood	NA	NA	GSE6613	GPL96	Affymetrix Human Genome U133A Array	PD-peripheral blood dataset
GSM153437	PD	peripheral blood	NA	NA	GSE6613	GPL96	Affymetrix Human Genome U133A Array	PD-peripheral blood dataset
GSM153439	PD	peripheral blood	NA	NA	GSE6613	GPL96	Affymetrix Human Genome U133A Array	PD-peripheral blood dataset
GSM153441	PD	peripheral blood	NA	NA	GSE6613	GPL96	Affymetrix Human Genome U133A Array	PD-peripheral blood dataset
GSM153442	PD	peripheral blood	NA	NA	GSE6613	GPL96	Affymetrix Human Genome U133A Array	PD-peripheral blood dataset
GSM153443	PD	peripheral blood	NA	NA	GSE6613	GPL96	Affymetrix Human Genome U133A Array	PD-peripheral blood dataset
GSM153444	control	peripheral blood	NA	NA	GSE6613	GPL96	Affymetrix Human Genome U133A Array	PD-peripheral blood dataset
GSM153445	PD	peripheral blood	NA	NA	GSE6613	GPL96	Affymetrix Human Genome U133A Array	PD-peripheral blood dataset
GSM153446	PD	peripheral blood	NA	NA	GSE6613	GPL96	Affymetrix Human Genome U133A Array	PD-peripheral blood dataset
GSM153448	control	peripheral blood	NA	NA	GSE6613	GPL96	Affymetrix Human Genome U133A Array	PD-peripheral blood dataset
GSM153449	PD	peripheral blood	NA	NA	GSE6613	GPL96	Affymetrix Human Genome U133A Array	PD-peripheral blood dataset
GSM153451	control	peripheral blood	NA	NA	GSE6613	GPL96	Affymetrix Human Genome U133A Array	PD-peripheral blood dataset
GSM153452	control	peripheral blood	NA	NA	GSE6613	GPL96	Affymetrix Human Genome U133A Array	PD-peripheral blood dataset
GSM153453	PD	peripheral blood	NA	NA	GSE6613	GPL96	Affymetrix Human Genome U133A Array	PD-peripheral blood dataset
GSM153454	PD	peripheral blood	NA	NA	GSE6613	GPL96	Affymetrix Human Genome U133A Array	PD-peripheral blood dataset
GSM153455	PD	peripheral blood	NA	NA	GSE6613	GPL96	Affymetrix Human Genome U133A Array	PD-peripheral blood dataset
GSM153462	PD	peripheral blood	NA	NA	GSE6613	GPL96	Affymetrix Human Genome U133A Array	PD-peripheral blood dataset
GSM153465	PD	peripheral blood	NA	NA	GSE6613	GPL96	Affymetrix Human Genome U133A Array	PD-peripheral blood dataset
GSM153477	control	peripheral blood	NA	NA	GSE6613	GPL96	Affymetrix Human Genome U133A Array	PD-peripheral blood dataset
GSM153479	control	peripheral blood	NA	NA	GSE6613	GPL96	Affymetrix Human Genome U133A Array	PD-peripheral blood dataset
GSM153481	PD	peripheral blood	NA	NA	GSE6613	GPL96	Affymetrix Human Genome U133A Array	PD-peripheral blood dataset
GSM153482	PD	peripheral blood	NA	NA	GSE6613	GPL96	Affymetrix Human Genome U133A Array	PD-peripheral blood dataset
GSM153483	PD	peripheral blood	NA	NA	GSE6613	GPL96	Affymetrix Human Genome U133A Array	PD-peripheral blood dataset
GSM153484	control	peripheral blood	NA	NA	GSE6613	GPL96	Affymetrix Human Genome U133A Array	PD-peripheral blood dataset
GSM153485	PD	peripheral blood	NA	NA	GSE6613	GPL96	Affymetrix Human Genome U133A Array	PD-peripheral blood dataset
GSM153488	control	peripheral blood	NA	NA	GSE6613	GPL96	Affymetrix Human Genome U133A Array	PD-peripheral blood dataset
GSM153489	PD	peripheral blood	NA	NA	GSE6613	GPL96	Affymetrix Human Genome U133A Array	PD-peripheral blood dataset
GSM153490	PD	peripheral blood	NA	NA	GSE6613	GPL96	Affymetrix Human Genome U133A Array	PD-peripheral blood dataset
GSM153491	PD	peripheral blood	NA	NA	GSE6613	GPL96	Affymetrix Human Genome U133A Array	PD-peripheral blood dataset
GSM153492	PD	peripheral blood	NA	NA	GSE6613	GPL96	Affymetrix Human Genome U133A Array	PD-peripheral blood dataset
GSM153493	PD	peripheral blood	NA	NA	GSE6613	GPL96	Affymetrix Human Genome U133A Array	PD-peripheral blood dataset
GSM153494	PD	peripheral blood	NA	NA	GSE6613	GPL96	Affymetrix Human Genome U133A Array	PD-peripheral blood dataset
GSM153495	PD	peripheral blood	NA	NA	GSE6613	GPL96	Affymetrix Human Genome U133A Array	PD-peripheral blood dataset
GSM153496	control	peripheral blood	NA	NA	GSE6613	GPL96	Affymetrix Human Genome U133A Array	PD-peripheral blood dataset
GSM153497	control	peripheral blood	NA	NA	GSE6613	GPL96	Affymetrix Human Genome U133A Array	PD-peripheral blood dataset
GSM153498	PD	peripheral blood	NA	NA	GSE6613	GPL96	Affymetrix Human Genome U133A Array	PD-peripheral blood dataset
GSM153500	control	peripheral blood	NA	NA	GSE6613	GPL96	Affymetrix Human Genome U133A Array	PD-peripheral blood dataset
GSM153501	PD	peripheral blood	NA	NA	GSE6613	GPL96	Affymetrix Human Genome U133A Array	PD-peripheral blood dataset
GSM153502	PD	peripheral blood	NA	NA	GSE6613	GPL96	Affymetrix Human Genome U133A Array	PD-peripheral blood dataset
GSM153503	control	peripheral blood	NA	NA	GSE6613	GPL96	Affymetrix Human Genome U133A Array	PD-peripheral blood dataset
GSM153505	PD	peripheral blood	NA	NA	GSE6613	GPL96	Affymetrix Human Genome U133A Array	PD-peripheral blood dataset
GSM153506	PD	peripheral blood	NA	NA	GSE6613	GPL96	Affymetrix Human Genome U133A Array	PD-peripheral blood dataset
GSM153508	control	peripheral blood	NA	NA	GSE6613	GPL96	Affymetrix Human Genome U133A Array	PD-peripheral blood dataset
GSM1859079	control	peripheral blood	NA	NA	GSE72267	GPL571	Affymetrix Human Genome U133A 2.0 Array	PD-peripheral blood dataset
GSM1859080	PD	peripheral blood	NA	NA	GSE72267	GPL571	Affymetrix Human Genome U133A 2.0 Array	PD-peripheral blood dataset
GSM1859081	PD	peripheral blood	NA	NA	GSE72267	GPL571	Affymetrix Human Genome U133A 2.0 Array	PD-peripheral blood dataset
GSM1859082	control	peripheral blood	NA	NA	GSE72267	GPL571	Affymetrix Human Genome U133A 2.0 Array	PD-peripheral blood dataset
GSM1859083	control	peripheral blood	NA	NA	GSE72267	GPL571	Affymetrix Human Genome U133A 2.0 Array	PD-peripheral blood dataset
GSM1859084	PD	peripheral blood	NA	NA	GSE72267	GPL571	Affymetrix Human Genome U133A 2.0 Array	PD-peripheral blood dataset
GSM1859085	control	peripheral blood	NA	NA	GSE72267	GPL571	Affymetrix Human Genome U133A 2.0 Array	PD-peripheral blood dataset
GSM1859086	PD	peripheral blood	NA	NA	GSE72267	GPL571	Affymetrix Human Genome U133A 2.0 Array	PD-peripheral blood dataset
GSM1859087	control	peripheral blood	NA	NA	GSE72267	GPL571	Affymetrix Human Genome U133A 2.0 Array	PD-peripheral blood dataset
GSM1859088	PD	peripheral blood	NA	NA	GSE72267	GPL571	Affymetrix Human Genome U133A 2.0 Array	PD-peripheral blood dataset
GSM1859089	PD	peripheral blood	NA	NA	GSE72267	GPL571	Affymetrix Human Genome U133A 2.0 Array	PD-peripheral blood dataset
GSM1859090	control	peripheral blood	NA	NA	GSE72267	GPL571	Affymetrix Human Genome U133A 2.0 Array	PD-peripheral blood dataset
GSM1859091	control	peripheral blood	NA	NA	GSE72267	GPL571	Affymetrix Human Genome U133A 2.0 Array	PD-peripheral blood dataset
GSM1859092	PD	peripheral blood	NA	NA	GSE72267	GPL571	Affymetrix Human Genome U133A 2.0 Array	PD-peripheral blood dataset
GSM1859093	control	peripheral blood	NA	NA	GSE72267	GPL571	Affymetrix Human Genome U133A 2.0 Array	PD-peripheral blood dataset
GSM1859094	control	peripheral blood	NA	NA	GSE72267	GPL571	Affymetrix Human Genome U133A 2.0 Array	PD-peripheral blood dataset
GSM1859095	PD	peripheral blood	NA	NA	GSE72267	GPL571	Affymetrix Human Genome U133A 2.0 Array	PD-peripheral blood dataset

GSM1859096	PD	peripheral blood	NA	NA	GSE72267	GPL571	Affymetrix Human Genome U133A 2.0 Array	PD-peripheral blood dataset
GSM1859097	control	peripheral blood	NA	NA	GSE72267	GPL571	Affymetrix Human Genome U133A 2.0 Array	PD-peripheral blood dataset
GSM1859098	PD	peripheral blood	NA	NA	GSE72267	GPL571	Affymetrix Human Genome U133A 2.0 Array	PD-peripheral blood dataset
GSM1859099	control	peripheral blood	NA	NA	GSE72267	GPL571	Affymetrix Human Genome U133A 2.0 Array	PD-peripheral blood dataset
GSM1859100	PD	peripheral blood	NA	NA	GSE72267	GPL571	Affymetrix Human Genome U133A 2.0 Array	PD-peripheral blood dataset
GSM1859101	control	peripheral blood	NA	NA	GSE72267	GPL571	Affymetrix Human Genome U133A 2.0 Array	PD-peripheral blood dataset
GSM1859102	PD	peripheral blood	NA	NA	GSE72267	GPL571	Affymetrix Human Genome U133A 2.0 Array	PD-peripheral blood dataset
GSM1859103	PD	peripheral blood	NA	NA	GSE72267	GPL571	Affymetrix Human Genome U133A 2.0 Array	PD-peripheral blood dataset
GSM1859104	control	peripheral blood	NA	NA	GSE72267	GPL571	Affymetrix Human Genome U133A 2.0 Array	PD-peripheral blood dataset
GSM1859105	PD	peripheral blood	NA	NA	GSE72267	GPL571	Affymetrix Human Genome U133A 2.0 Array	PD-peripheral blood dataset
GSM1859106	control	peripheral blood	NA	NA	GSE72267	GPL571	Affymetrix Human Genome U133A 2.0 Array	PD-peripheral blood dataset
GSM1859107	control	peripheral blood	NA	NA	GSE72267	GPL571	Affymetrix Human Genome U133A 2.0 Array	PD-peripheral blood dataset
GSM1859108	PD	peripheral blood	NA	NA	GSE72267	GPL571	Affymetrix Human Genome U133A 2.0 Array	PD-peripheral blood dataset
GSM1859109	control	peripheral blood	NA	NA	GSE72267	GPL571	Affymetrix Human Genome U133A 2.0 Array	PD-peripheral blood dataset
GSM1859110	PD	peripheral blood	NA	NA	GSE72267	GPL571	Affymetrix Human Genome U133A 2.0 Array	PD-peripheral blood dataset
GSM1859111	control	peripheral blood	NA	NA	GSE72267	GPL571	Affymetrix Human Genome U133A 2.0 Array	PD-peripheral blood dataset
GSM1859112	PD	peripheral blood	NA	NA	GSE72267	GPL571	Affymetrix Human Genome U133A 2.0 Array	PD-peripheral blood dataset
GSM1859113	control	peripheral blood	NA	NA	GSE72267	GPL571	Affymetrix Human Genome U133A 2.0 Array	PD-peripheral blood dataset
GSM1859114	PD	peripheral blood	NA	NA	GSE72267	GPL571	Affymetrix Human Genome U133A 2.0 Array	PD-peripheral blood dataset
GSM1859115	PD	peripheral blood	NA	NA	GSE72267	GPL571	Affymetrix Human Genome U133A 2.0 Array	PD-peripheral blood dataset
GSM1859116	control	peripheral blood	NA	NA	GSE72267	GPL571	Affymetrix Human Genome U133A 2.0 Array	PD-peripheral blood dataset
GSM1859117	PD	peripheral blood	NA	NA	GSE72267	GPL571	Affymetrix Human Genome U133A 2.0 Array	PD-peripheral blood dataset
GSM1859118	PD	peripheral blood	NA	NA	GSE72267	GPL571	Affymetrix Human Genome U133A 2.0 Array	PD-peripheral blood dataset
GSM1859119	PD	peripheral blood	NA	NA	GSE72267	GPL571	Affymetrix Human Genome U133A 2.0 Array	PD-peripheral blood dataset
GSM1859120	PD	peripheral blood	NA	NA	GSE72267	GPL571	Affymetrix Human Genome U133A 2.0 Array	PD-peripheral blood dataset
GSM1859121	PD	peripheral blood	NA	NA	GSE72267	GPL571	Affymetrix Human Genome U133A 2.0 Array	PD-peripheral blood dataset
GSM1859122	PD	peripheral blood	NA	NA	GSE72267	GPL571	Affymetrix Human Genome U133A 2.0 Array	PD-peripheral blood dataset
GSM1859123	PD	peripheral blood	NA	NA	GSE72267	GPL571	Affymetrix Human Genome U133A 2.0 Array	PD-peripheral blood dataset
GSM1859124	PD	peripheral blood	NA	NA	GSE72267	GPL571	Affymetrix Human Genome U133A 2.0 Array	PD-peripheral blood dataset
GSM1859125	PD	peripheral blood	NA	NA	GSE72267	GPL571	Affymetrix Human Genome U133A 2.0 Array	PD-peripheral blood dataset
GSM1859126	PD	peripheral blood	NA	NA	GSE72267	GPL571	Affymetrix Human Genome U133A 2.0 Array	PD-peripheral blood dataset
GSM1859127	PD	peripheral blood	NA	NA	GSE72267	GPL571	Affymetrix Human Genome U133A 2.0 Array	PD-peripheral blood dataset
GSM1859128	PD	peripheral blood	NA	NA	GSE72267	GPL571	Affymetrix Human Genome U133A 2.0 Array	PD-peripheral blood dataset
GSM1859129	PD	peripheral blood	NA	NA	GSE72267	GPL571	Affymetrix Human Genome U133A 2.0 Array	PD-peripheral blood dataset
GSM1859130	PD	peripheral blood	NA	NA	GSE72267	GPL571	Affymetrix Human Genome U133A 2.0 Array	PD-peripheral blood dataset
GSM1859131	PD	peripheral blood	NA	NA	GSE72267	GPL571	Affymetrix Human Genome U133A 2.0 Array	PD-peripheral blood dataset
GSM1859132	PD	peripheral blood	NA	NA	GSE72267	GPL571	Affymetrix Human Genome U133A 2.0 Array	PD-peripheral blood dataset
GSM1859133	PD	peripheral blood	NA	NA	GSE72267	GPL571	Affymetrix Human Genome U133A 2.0 Array	PD-peripheral blood dataset
GSM1859134	PD	peripheral blood	NA	NA	GSE72267	GPL571	Affymetrix Human Genome U133A 2.0 Array	PD-peripheral blood dataset
GSM1859135	PD	peripheral blood	NA	NA	GSE72267	GPL571	Affymetrix Human Genome U133A 2.0 Array	PD-peripheral blood dataset
GSM1859136	PD	peripheral blood	NA	NA	GSE72267	GPL571	Affymetrix Human Genome U133A 2.0 Array	PD-peripheral blood dataset
GSM1859137	PD	peripheral blood	NA	NA	GSE72267	GPL571	Affymetrix Human Genome U133A 2.0 Array	PD-peripheral blood dataset
GSM2630758	control	peripheral blood	NA	female	GSE99039	GPL570	Affymetrix Human Genome U133 Plus 2.0 Array	PD-peripheral blood dataset
GSM2630759	PD	peripheral blood	NA	male	GSE99039	GPL570	Affymetrix Human Genome U133 Plus 2.0 Array	PD-peripheral blood dataset
GSM2630760	PD	peripheral blood	NA	female	GSE99039	GPL570	Affymetrix Human Genome U133 Plus 2.0 Array	PD-peripheral blood dataset
GSM2630761	control	peripheral blood	NA	female	GSE99039	GPL570	Affymetrix Human Genome U133 Plus 2.0 Array	PD-peripheral blood dataset
GSM2630762	PD	peripheral blood	NA	female	GSE99039	GPL570	Affymetrix Human Genome U133 Plus 2.0 Array	PD-peripheral blood dataset
GSM2630763	PD	peripheral blood	NA	female	GSE99039	GPL570	Affymetrix Human Genome U133 Plus 2.0 Array	PD-peripheral blood dataset
GSM2630764	PD	peripheral blood	NA	male	GSE99039	GPL570	Affymetrix Human Genome U133 Plus 2.0 Array	PD-peripheral blood dataset
GSM2630765	PD	peripheral blood	NA	male	GSE99039	GPL570	Affymetrix Human Genome U133 Plus 2.0 Array	PD-peripheral blood dataset
GSM2630766	PD	peripheral blood	NA	male	GSE99039	GPL570	Affymetrix Human Genome U133 Plus 2.0 Array	PD-peripheral blood dataset
GSM2630768	PD	peripheral blood	NA	female	GSE99039	GPL570	Affymetrix Human Genome U133 Plus 2.0 Array	PD-peripheral blood dataset
GSM2630769	control	peripheral blood	NA	female	GSE99039	GPL570	Affymetrix Human Genome U133 Plus 2.0 Array	PD-peripheral blood dataset
GSM2630770	PD	peripheral blood	NA	male	GSE99039	GPL570	Affymetrix Human Genome U133 Plus 2.0 Array	PD-peripheral blood dataset
GSM2630771	control	peripheral blood	NA	female	GSE99039	GPL570	Affymetrix Human Genome U133 Plus 2.0 Array	PD-peripheral blood dataset
GSM2630772	control	peripheral blood	NA	male	GSE99039	GPL570	Affymetrix Human Genome U133 Plus 2.0 Array	PD-peripheral blood dataset
GSM2630773	PD	peripheral blood	NA	male	GSE99039	GPL570	Affymetrix Human Genome U133 Plus 2.0 Array	PD-peripheral blood dataset
GSM2630774	control	peripheral blood	NA	female	GSE99039	GPL570	Affymetrix Human Genome U133 Plus 2.0 Array	PD-peripheral blood dataset
GSM2630777	control	peripheral blood	NA	female	GSE99039	GPL570	Affymetrix Human Genome U133 Plus 2.0 Array	PD-peripheral blood dataset
GSM2630778	control	peripheral blood	NA	male	GSE99039	GPL570	Affymetrix Human Genome U133 Plus 2.0 Array	PD-peripheral blood dataset
GSM2630779	control	peripheral blood	NA	female	GSE99039	GPL570	Affymetrix Human Genome U133 Plus 2.0 Array	PD-peripheral blood dataset
GSM2630780	control	peripheral blood	NA	female	GSE99039	GPL570	Affymetrix Human Genome U133 Plus 2.0 Array	PD-peripheral blood dataset
GSM2630781	PD	peripheral blood	NA	male	GSE99039	GPL570	Affymetrix Human Genome U133 Plus 2.0 Array	PD-peripheral blood dataset
GSM2630783	control	peripheral blood	NA	female	GSE99039	GPL570	Affymetrix Human Genome U133 Plus 2.0 Array	PD-peripheral blood dataset
GSM2630784	control	peripheral blood	NA	male	GSE99039	GPL570	Affymetrix Human Genome U133 Plus 2.0 Array	PD-peripheral blood dataset
GSM2630785	control	peripheral blood	NA	male	GSE99039	GPL570	Affymetrix Human Genome U133 Plus 2.0 Array	PD-peripheral blood dataset
GSM2630787	control	peripheral blood	NA	female	GSE99039	GPL570	Affymetrix Human Genome U133 Plus 2.0 Array	PD-peripheral blood dataset
GSM2630788	control	peripheral blood	NA	female	GSE99039	GPL570	Affymetrix Human Genome U133 Plus 2.0 Array	PD-peripheral blood dataset
GSM2630789	control	peripheral blood	NA	male	GSE99039	GPL570	Affymetrix Human Genome U133 Plus 2.0 Array	PD-peripheral blood dataset
GSM2630790	control	peripheral blood	NA	female	GSE99039	GPL570	Affymetrix Human Genome U133 Plus 2.0 Array	PD-peripheral blood dataset
GSM2630791	PD	peripheral blood	NA	male	GSE99039	GPL570	Affymetrix Human Genome U133 Plus 2.0 Array	PD-peripheral blood dataset
GSM2630793	PD	peripheral blood	NA	male	GSE99039	GPL570	Affymetrix Human Genome U133 Plus 2.0 Array	PD-peripheral blood dataset
GSM2630794	PD	peripheral blood	NA	male	GSE99039	GPL570	Affymetrix Human Genome U133 Plus 2.0 Array	PD-peripheral blood dataset
GSM2630795	PD	peripheral blood	NA	female	GSE99039	GPL570	Affymetrix Human Genome U133 Plus 2.0 Array	PD-peripheral blood dataset
GSM2630799	control	peripheral blood	NA	female	GSE99039	GPL570	Affymetrix Human Genome U133 Plus 2.0 Array	PD-peripheral blood dataset
GSM2630800	control	peripheral blood	NA	female	GSE99039	GPL570	Affymetrix Human Genome U133 Plus 2.0 Array	PD-peripheral blood dataset
GSM2630801	PD	peripheral blood	NA	female	GSE99039	GPL570	Affymetrix Human Genome U133 Plus 2.0 Array	PD-peripheral blood dataset
GSM2630802	PD	peripheral blood	NA	female	GSE99039	GPL570	Affymetrix Human Genome U133 Plus 2.0 Array	PD-peripheral blood dataset
GSM2630803	control	peripheral blood	NA	female	GSE99039	GPL570	Affymetrix Human Genome U133 Plus 2.0 Array	PD-peripheral blood dataset
GSM2630804	PD	peripheral blood	NA	female	GSE99039	GPL570	Affymetrix Human Genome U133 Plus 2.0 Array	PD-peripheral blood dataset
GSM2630805	PD	peripheral blood	NA	male	GSE99039	GPL570	Affymetrix Human Genome U133 Plus 2.0 Array	PD-peripheral blood dataset
GSM2630806	PD	peripheral blood	NA	male	GSE99039	GPL570	Affymetrix Human Genome U133 Plus 2.0 Array	PD-peripheral blood dataset
GSM2630807	PD	peripheral blood	NA	female	GSE99039	GPL570	Affymetrix Human Genome U133 Plus 2.0 Array	PD-peripheral blood dataset
GSM2630810	control	peripheral blood	NA	female	GSE99039	GPL570	Affymetrix Human Genome U133 Plus 2.0 Array	PD-peripheral blood dataset
GSM2630812	control	peripheral blood	NA	male	GSE99039	GPL570	Affymetrix Human Genome U133 Plus 2.0 Array	PD-peripheral blood dataset
GSM2630813	PD	peripheral blood	NA	male	GSE99039	GPL570	Affymetrix Human Genome U133 Plus 2.0 Array	PD-peripheral blood dataset
GSM2630815	PD	peripheral blood	NA	male	GSE99039	GPL570	Affymetrix Human Genome U133 Plus 2.0 Array	PD-peripheral blood dataset
GSM2630816	control	peripheral blood	NA	female	GSE99039	GPL570	Affymetrix Human Genome U133 Plus 2.0 Array	PD-peripheral blood dataset
GSM2630817	PD	peripheral blood	NA	female	GSE99039	GPL570	Affymetrix Human Genome U133 Plus 2.0 Array	PD-peripheral blood dataset
GSM2630818	control	peripheral blood	NA	female	GSE99039	GPL570	Affymetrix Human Genome U133 Plus 2.0 Array	PD-peripheral blood dataset
GSM2630821	PD	peripheral blood	NA	female	GSE99039	GPL570	Affymetrix Human Genome U133 Plus 2.0 Array	PD-peripheral blood dataset
GSM2630822	PD	peripheral blood	NA	male	GSE99039	GPL570	Affymetrix Human Genome U133 Plus 2.0 Array	PD-peripheral blood dataset

GSM2630823	PD	peripheral blood	NA	female	GSE99039	GPL570	Affymetrix Human Genome U133 Plus 2.0 Array	PD-peripheral blood dataset
GSM2630824	PD	peripheral blood	NA	female	GSE99039	GPL570	Affymetrix Human Genome U133 Plus 2.0 Array	PD-peripheral blood dataset
GSM2630825	PD	peripheral blood	NA	female	GSE99039	GPL570	Affymetrix Human Genome U133 Plus 2.0 Array	PD-peripheral blood dataset
GSM2630826	PD	peripheral blood	NA	female	GSE99039	GPL570	Affymetrix Human Genome U133 Plus 2.0 Array	PD-peripheral blood dataset
GSM2630827	PD	peripheral blood	NA	female	GSE99039	GPL570	Affymetrix Human Genome U133 Plus 2.0 Array	PD-peripheral blood dataset
GSM2630828	PD	peripheral blood	NA	male	GSE99039	GPL570	Affymetrix Human Genome U133 Plus 2.0 Array	PD-peripheral blood dataset
GSM2630829	PD	peripheral blood	NA	male	GSE99039	GPL570	Affymetrix Human Genome U133 Plus 2.0 Array	PD-peripheral blood dataset
GSM2630830	control	peripheral blood	NA	female	GSE99039	GPL570	Affymetrix Human Genome U133 Plus 2.0 Array	PD-peripheral blood dataset
GSM2630835	PD	peripheral blood	NA	female	GSE99039	GPL570	Affymetrix Human Genome U133 Plus 2.0 Array	PD-peripheral blood dataset
GSM2630837	PD	peripheral blood	NA	female	GSE99039	GPL570	Affymetrix Human Genome U133 Plus 2.0 Array	PD-peripheral blood dataset
GSM2630838	PD	peripheral blood	NA	female	GSE99039	GPL570	Affymetrix Human Genome U133 Plus 2.0 Array	PD-peripheral blood dataset
GSM2630839	PD	peripheral blood	NA	male	GSE99039	GPL570	Affymetrix Human Genome U133 Plus 2.0 Array	PD-peripheral blood dataset
GSM2630840	control	peripheral blood	NA	female	GSE99039	GPL570	Affymetrix Human Genome U133 Plus 2.0 Array	PD-peripheral blood dataset
GSM2630841	PD	peripheral blood	NA	male	GSE99039	GPL570	Affymetrix Human Genome U133 Plus 2.0 Array	PD-peripheral blood dataset
GSM2630842	PD	peripheral blood	NA	female	GSE99039	GPL570	Affymetrix Human Genome U133 Plus 2.0 Array	PD-peripheral blood dataset
GSM2630843	control	peripheral blood	NA	male	GSE99039	GPL570	Affymetrix Human Genome U133 Plus 2.0 Array	PD-peripheral blood dataset
GSM2630853	control	peripheral blood	NA	male	GSE99039	GPL570	Affymetrix Human Genome U133 Plus 2.0 Array	PD-peripheral blood dataset
GSM2630854	PD	peripheral blood	NA	female	GSE99039	GPL570	Affymetrix Human Genome U133 Plus 2.0 Array	PD-peripheral blood dataset
GSM2630855	control	peripheral blood	NA	male	GSE99039	GPL570	Affymetrix Human Genome U133 Plus 2.0 Array	PD-peripheral blood dataset
GSM2630856	PD	peripheral blood	NA	male	GSE99039	GPL570	Affymetrix Human Genome U133 Plus 2.0 Array	PD-peripheral blood dataset
GSM2630857	control	peripheral blood	NA	female	GSE99039	GPL570	Affymetrix Human Genome U133 Plus 2.0 Array	PD-peripheral blood dataset
GSM2630861	control	peripheral blood	NA	female	GSE99039	GPL570	Affymetrix Human Genome U133 Plus 2.0 Array	PD-peripheral blood dataset
GSM2630862	PD	peripheral blood	NA	male	GSE99039	GPL570	Affymetrix Human Genome U133 Plus 2.0 Array	PD-peripheral blood dataset
GSM2630863	control	peripheral blood	NA	male	GSE99039	GPL570	Affymetrix Human Genome U133 Plus 2.0 Array	PD-peripheral blood dataset
GSM2630864	control	peripheral blood	NA	female	GSE99039	GPL570	Affymetrix Human Genome U133 Plus 2.0 Array	PD-peripheral blood dataset
GSM2630865	control	peripheral blood	NA	male	GSE99039	GPL570	Affymetrix Human Genome U133 Plus 2.0 Array	PD-peripheral blood dataset
GSM2630866	control	peripheral blood	NA	male	GSE99039	GPL570	Affymetrix Human Genome U133 Plus 2.0 Array	PD-peripheral blood dataset
GSM2630867	PD	peripheral blood	NA	male	GSE99039	GPL570	Affymetrix Human Genome U133 Plus 2.0 Array	PD-peripheral blood dataset
GSM2630868	control	peripheral blood	NA	female	GSE99039	GPL570	Affymetrix Human Genome U133 Plus 2.0 Array	PD-peripheral blood dataset
GSM2630869	control	peripheral blood	NA	male	GSE99039	GPL570	Affymetrix Human Genome U133 Plus 2.0 Array	PD-peripheral blood dataset
GSM2630870	PD	peripheral blood	NA	male	GSE99039	GPL570	Affymetrix Human Genome U133 Plus 2.0 Array	PD-peripheral blood dataset
GSM2630871	control	peripheral blood	NA	female	GSE99039	GPL570	Affymetrix Human Genome U133 Plus 2.0 Array	PD-peripheral blood dataset
GSM2630872	control	peripheral blood	NA	female	GSE99039	GPL570	Affymetrix Human Genome U133 Plus 2.0 Array	PD-peripheral blood dataset
GSM2630873	control	peripheral blood	NA	female	GSE99039	GPL570	Affymetrix Human Genome U133 Plus 2.0 Array	PD-peripheral blood dataset
GSM2630874	control	peripheral blood	NA	female	GSE99039	GPL570	Affymetrix Human Genome U133 Plus 2.0 Array	PD-peripheral blood dataset
GSM2630876	control	peripheral blood	NA	female	GSE99039	GPL570	Affymetrix Human Genome U133 Plus 2.0 Array	PD-peripheral blood dataset
GSM2630877	control	peripheral blood	NA	female	GSE99039	GPL570	Affymetrix Human Genome U133 Plus 2.0 Array	PD-peripheral blood dataset
GSM2630878	control	peripheral blood	NA	male	GSE99039	GPL570	Affymetrix Human Genome U133 Plus 2.0 Array	PD-peripheral blood dataset
GSM2630879	control	peripheral blood	NA	male	GSE99039	GPL570	Affymetrix Human Genome U133 Plus 2.0 Array	PD-peripheral blood dataset
GSM2630880	control	peripheral blood	NA	female	GSE99039	GPL570	Affymetrix Human Genome U133 Plus 2.0 Array	PD-peripheral blood dataset
GSM2630881	control	peripheral blood	NA	female	GSE99039	GPL570	Affymetrix Human Genome U133 Plus 2.0 Array	PD-peripheral blood dataset
GSM2630882	control	peripheral blood	NA	female	GSE99039	GPL570	Affymetrix Human Genome U133 Plus 2.0 Array	PD-peripheral blood dataset
GSM2630883	control	peripheral blood	NA	male	GSE99039	GPL570	Affymetrix Human Genome U133 Plus 2.0 Array	PD-peripheral blood dataset
GSM2630884	control	peripheral blood	NA	male	GSE99039	GPL570	Affymetrix Human Genome U133 Plus 2.0 Array	PD-peripheral blood dataset
GSM2630885	control	peripheral blood	NA	male	GSE99039	GPL570	Affymetrix Human Genome U133 Plus 2.0 Array	PD-peripheral blood dataset
GSM2630886	PD	peripheral blood	NA	female	GSE99039	GPL570	Affymetrix Human Genome U133 Plus 2.0 Array	PD-peripheral blood dataset
GSM2630888	control	peripheral blood	NA	male	GSE99039	GPL570	Affymetrix Human Genome U133 Plus 2.0 Array	PD-peripheral blood dataset
GSM2630890	PD	peripheral blood	NA	male	GSE99039	GPL570	Affymetrix Human Genome U133 Plus 2.0 Array	PD-peripheral blood dataset
GSM2630891	PD	peripheral blood	NA	male	GSE99039	GPL570	Affymetrix Human Genome U133 Plus 2.0 Array	PD-peripheral blood dataset
GSM2630892	PD	peripheral blood	NA	male	GSE99039	GPL570	Affymetrix Human Genome U133 Plus 2.0 Array	PD-peripheral blood dataset
GSM2630893	PD	peripheral blood	NA	male	GSE99039	GPL570	Affymetrix Human Genome U133 Plus 2.0 Array	PD-peripheral blood dataset
GSM2630895	PD	peripheral blood	NA	female	GSE99039	GPL570	Affymetrix Human Genome U133 Plus 2.0 Array	PD-peripheral blood dataset
GSM2630896	control	peripheral blood	NA	female	GSE99039	GPL570	Affymetrix Human Genome U133 Plus 2.0 Array	PD-peripheral blood dataset
GSM2630897	control	peripheral blood	NA	female	GSE99039	GPL570	Affymetrix Human Genome U133 Plus 2.0 Array	PD-peripheral blood dataset
GSM2630898	PD	peripheral blood	NA	male	GSE99039	GPL570	Affymetrix Human Genome U133 Plus 2.0 Array	PD-peripheral blood dataset
GSM2630899	control	peripheral blood	NA	male	GSE99039	GPL570	Affymetrix Human Genome U133 Plus 2.0 Array	PD-peripheral blood dataset
GSM2630900	PD	peripheral blood	NA	female	GSE99039	GPL570	Affymetrix Human Genome U133 Plus 2.0 Array	PD-peripheral blood dataset
GSM2630901	PD	peripheral blood	NA	male	GSE99039	GPL570	Affymetrix Human Genome U133 Plus 2.0 Array	PD-peripheral blood dataset
GSM2630902	PD	peripheral blood	NA	male	GSE99039	GPL570	Affymetrix Human Genome U133 Plus 2.0 Array	PD-peripheral blood dataset
GSM2630903	PD	peripheral blood	NA	male	GSE99039	GPL570	Affymetrix Human Genome U133 Plus 2.0 Array	PD-peripheral blood dataset
GSM2630904	PD	peripheral blood	NA	male	GSE99039	GPL570	Affymetrix Human Genome U133 Plus 2.0 Array	PD-peripheral blood dataset
GSM2630905	control	peripheral blood	NA	male	GSE99039	GPL570	Affymetrix Human Genome U133 Plus 2.0 Array	PD-peripheral blood dataset
GSM2630906	control	peripheral blood	NA	male	GSE99039	GPL570	Affymetrix Human Genome U133 Plus 2.0 Array	PD-peripheral blood dataset
GSM2630907	control	peripheral blood	NA	female	GSE99039	GPL570	Affymetrix Human Genome U133 Plus 2.0 Array	PD-peripheral blood dataset
GSM2630908	control	peripheral blood	NA	female	GSE99039	GPL570	Affymetrix Human Genome U133 Plus 2.0 Array	PD-peripheral blood dataset
GSM2630909	control	peripheral blood	NA	female	GSE99039	GPL570	Affymetrix Human Genome U133 Plus 2.0 Array	PD-peripheral blood dataset
GSM2630910	control	peripheral blood	NA	male	GSE99039	GPL570	Affymetrix Human Genome U133 Plus 2.0 Array	PD-peripheral blood dataset
GSM2630911	PD	peripheral blood	NA	female	GSE99039	GPL570	Affymetrix Human Genome U133 Plus 2.0 Array	PD-peripheral blood dataset
GSM2630912	control	peripheral blood	NA	male	GSE99039	GPL570	Affymetrix Human Genome U133 Plus 2.0 Array	PD-peripheral blood dataset
GSM2630913	control	peripheral blood	NA	female	GSE99039	GPL570	Affymetrix Human Genome U133 Plus 2.0 Array	PD-peripheral blood dataset
GSM2630914	PD	peripheral blood	NA	male	GSE99039	GPL570	Affymetrix Human Genome U133 Plus 2.0 Array	PD-peripheral blood dataset
GSM2630915	PD	peripheral blood	NA	female	GSE99039	GPL570	Affymetrix Human Genome U133 Plus 2.0 Array	PD-peripheral blood dataset
GSM2630916	control	peripheral blood	NA	female	GSE99039	GPL570	Affymetrix Human Genome U133 Plus 2.0 Array	PD-peripheral blood dataset
GSM2630917	control	peripheral blood	NA	male	GSE99039	GPL570	Affymetrix Human Genome U133 Plus 2.0 Array	PD-peripheral blood dataset
GSM2630918	PD	peripheral blood	NA	NA	GSE99039	GPL570	Affymetrix Human Genome U133 Plus 2.0 Array	PD-peripheral blood dataset
GSM2630921	PD	peripheral blood	NA	male	GSE99039	GPL570	Affymetrix Human Genome U133 Plus 2.0 Array	PD-peripheral blood dataset
GSM2630922	control	peripheral blood	NA	male	GSE99039	GPL570	Affymetrix Human Genome U133 Plus 2.0 Array	PD-peripheral blood dataset
GSM2630923	control	peripheral blood	NA	female	GSE99039	GPL570	Affymetrix Human Genome U133 Plus 2.0 Array	PD-peripheral blood dataset
GSM2630924	control	peripheral blood	NA	male	GSE99039	GPL570	Affymetrix Human Genome U133 Plus 2.0 Array	PD-peripheral blood dataset
GSM2630925	control	peripheral blood	NA	female	GSE99039	GPL570	Affymetrix Human Genome U133 Plus 2.0 Array	PD-peripheral blood dataset
GSM2630927	control	peripheral blood	NA	female	GSE99039	GPL570	Affymetrix Human Genome U133 Plus 2.0 Array	PD-peripheral blood dataset
GSM2630928	control	peripheral blood	NA	female	GSE99039	GPL570	Affymetrix Human Genome U133 Plus 2.0 Array	PD-peripheral blood dataset
GSM2630929	control	peripheral blood	NA	female	GSE99039	GPL570	Affymetrix Human Genome U133 Plus 2.0 Array	PD-peripheral blood dataset
GSM2630930	control	peripheral blood	NA	female	GSE99039	GPL570	Affymetrix Human Genome U133 Plus 2.0 Array	PD-peripheral blood dataset
GSM2630931	PD	peripheral blood	NA	male	GSE99039	GPL570	Affymetrix Human Genome U133 Plus 2.0 Array	PD-peripheral blood dataset
GSM2630932	control	peripheral blood	NA	female	GSE99039	GPL570	Affymetrix Human Genome U133 Plus 2.0 Array	PD-peripheral blood dataset
GSM2630938	PD	peripheral blood	NA	male	GSE99039	GPL570	Affymetrix Human Genome U133 Plus 2.0 Array	PD-peripheral blood dataset
GSM2630939	PD	peripheral blood	NA	female	GSE99039	GPL570	Affymetrix Human Genome U133 Plus 2.0 Array	PD-peripheral blood dataset
GSM2630940	PD	peripheral blood	NA	female	GSE99039	GPL570	Affymetrix Human Genome U133 Plus 2.0 Array	PD-peripheral blood dataset
GSM2630941	PD	peripheral blood	NA	male	GSE99039	GPL570	Affymetrix Human Genome U133 Plus 2.0 Array	PD-peripheral blood dataset
GSM2630942	PD	peripheral blood	NA	female	GSE99039	GPL570	Affymetrix Human Genome U133 Plus 2.0 Array	PD-peripheral blood dataset
GSM2630944	PD	peripheral blood	NA	female	GSE99039	GPL570	Affymetrix Human Genome U133 Plus 2.0 Array	PD-peripheral blood dataset

GSM2630945	PD	peripheral blood	NA	female	GSE99039	GPL570	Affymetrix Human Genome U133 Plus 2.0 Array	PD-peripheral blood dataset
GSM2630947	PD	peripheral blood	NA	male	GSE99039	GPL570	Affymetrix Human Genome U133 Plus 2.0 Array	PD-peripheral blood dataset
GSM2630948	PD	peripheral blood	NA	female	GSE99039	GPL570	Affymetrix Human Genome U133 Plus 2.0 Array	PD-peripheral blood dataset
GSM2630949	PD	peripheral blood	NA	female	GSE99039	GPL570	Affymetrix Human Genome U133 Plus 2.0 Array	PD-peripheral blood dataset
GSM2630950	PD	peripheral blood	NA	male	GSE99039	GPL570	Affymetrix Human Genome U133 Plus 2.0 Array	PD-peripheral blood dataset
GSM2630951	PD	peripheral blood	NA	male	GSE99039	GPL570	Affymetrix Human Genome U133 Plus 2.0 Array	PD-peripheral blood dataset
GSM2630956	PD	peripheral blood	NA	male	GSE99039	GPL570	Affymetrix Human Genome U133 Plus 2.0 Array	PD-peripheral blood dataset
GSM2630957	PD	peripheral blood	NA	female	GSE99039	GPL570	Affymetrix Human Genome U133 Plus 2.0 Array	PD-peripheral blood dataset
GSM2630958	control	peripheral blood	NA	female	GSE99039	GPL570	Affymetrix Human Genome U133 Plus 2.0 Array	PD-peripheral blood dataset
GSM2630959	PD	peripheral blood	NA	female	GSE99039	GPL570	Affymetrix Human Genome U133 Plus 2.0 Array	PD-peripheral blood dataset
GSM2630960	PD	peripheral blood	NA	female	GSE99039	GPL570	Affymetrix Human Genome U133 Plus 2.0 Array	PD-peripheral blood dataset
GSM2630961	control	peripheral blood	NA	male	GSE99039	GPL570	Affymetrix Human Genome U133 Plus 2.0 Array	PD-peripheral blood dataset
GSM2630964	PD	peripheral blood	NA	male	GSE99039	GPL570	Affymetrix Human Genome U133 Plus 2.0 Array	PD-peripheral blood dataset
GSM2630965	control	peripheral blood	NA	female	GSE99039	GPL570	Affymetrix Human Genome U133 Plus 2.0 Array	PD-peripheral blood dataset
GSM2630966	PD	peripheral blood	NA	female	GSE99039	GPL570	Affymetrix Human Genome U133 Plus 2.0 Array	PD-peripheral blood dataset
GSM2630967	PD	peripheral blood	NA	female	GSE99039	GPL570	Affymetrix Human Genome U133 Plus 2.0 Array	PD-peripheral blood dataset
GSM2630968	PD	peripheral blood	NA	male	GSE99039	GPL570	Affymetrix Human Genome U133 Plus 2.0 Array	PD-peripheral blood dataset
GSM2630969	PD	peripheral blood	NA	male	GSE99039	GPL570	Affymetrix Human Genome U133 Plus 2.0 Array	PD-peripheral blood dataset
GSM2630970	PD	peripheral blood	NA	male	GSE99039	GPL570	Affymetrix Human Genome U133 Plus 2.0 Array	PD-peripheral blood dataset
GSM2630971	control	peripheral blood	NA	female	GSE99039	GPL570	Affymetrix Human Genome U133 Plus 2.0 Array	PD-peripheral blood dataset
GSM2630972	PD	peripheral blood	NA	male	GSE99039	GPL570	Affymetrix Human Genome U133 Plus 2.0 Array	PD-peripheral blood dataset
GSM2630973	PD	peripheral blood	NA	male	GSE99039	GPL570	Affymetrix Human Genome U133 Plus 2.0 Array	PD-peripheral blood dataset
GSM2630974	control	peripheral blood	NA	male	GSE99039	GPL570	Affymetrix Human Genome U133 Plus 2.0 Array	PD-peripheral blood dataset
GSM2630975	PD	peripheral blood	NA	female	GSE99039	GPL570	Affymetrix Human Genome U133 Plus 2.0 Array	PD-peripheral blood dataset
GSM2630976	PD	peripheral blood	NA	female	GSE99039	GPL570	Affymetrix Human Genome U133 Plus 2.0 Array	PD-peripheral blood dataset
GSM2630977	PD	peripheral blood	NA	female	GSE99039	GPL570	Affymetrix Human Genome U133 Plus 2.0 Array	PD-peripheral blood dataset
GSM2630978	PD	peripheral blood	NA	male	GSE99039	GPL570	Affymetrix Human Genome U133 Plus 2.0 Array	PD-peripheral blood dataset
GSM2630979	PD	peripheral blood	NA	female	GSE99039	GPL570	Affymetrix Human Genome U133 Plus 2.0 Array	PD-peripheral blood dataset
GSM2630980	control	peripheral blood	NA	female	GSE99039	GPL570	Affymetrix Human Genome U133 Plus 2.0 Array	PD-peripheral blood dataset
GSM2630981	PD	peripheral blood	NA	female	GSE99039	GPL570	Affymetrix Human Genome U133 Plus 2.0 Array	PD-peripheral blood dataset
GSM2630982	PD	peripheral blood	NA	male	GSE99039	GPL570	Affymetrix Human Genome U133 Plus 2.0 Array	PD-peripheral blood dataset
GSM2630983	PD	peripheral blood	NA	female	GSE99039	GPL570	Affymetrix Human Genome U133 Plus 2.0 Array	PD-peripheral blood dataset
GSM2630984	control	peripheral blood	NA	female	GSE99039	GPL570	Affymetrix Human Genome U133 Plus 2.0 Array	PD-peripheral blood dataset
GSM2630985	PD	peripheral blood	NA	female	GSE99039	GPL570	Affymetrix Human Genome U133 Plus 2.0 Array	PD-peripheral blood dataset
GSM2630986	PD	peripheral blood	NA	male	GSE99039	GPL570	Affymetrix Human Genome U133 Plus 2.0 Array	PD-peripheral blood dataset
GSM2630988	PD	peripheral blood	NA	male	GSE99039	GPL570	Affymetrix Human Genome U133 Plus 2.0 Array	PD-peripheral blood dataset
GSM2630989	PD	peripheral blood	NA	male	GSE99039	GPL570	Affymetrix Human Genome U133 Plus 2.0 Array	PD-peripheral blood dataset
GSM2630990	control	peripheral blood	NA	female	GSE99039	GPL570	Affymetrix Human Genome U133 Plus 2.0 Array	PD-peripheral blood dataset
GSM2630991	PD	peripheral blood	NA	female	GSE99039	GPL570	Affymetrix Human Genome U133 Plus 2.0 Array	PD-peripheral blood dataset
GSM2630992	PD	peripheral blood	NA	female	GSE99039	GPL570	Affymetrix Human Genome U133 Plus 2.0 Array	PD-peripheral blood dataset
GSM2630993	control	peripheral blood	NA	male	GSE99039	GPL570	Affymetrix Human Genome U133 Plus 2.0 Array	PD-peripheral blood dataset
GSM2630994	PD	peripheral blood	NA	female	GSE99039	GPL570	Affymetrix Human Genome U133 Plus 2.0 Array	PD-peripheral blood dataset
GSM2630995	PD	peripheral blood	NA	male	GSE99039	GPL570	Affymetrix Human Genome U133 Plus 2.0 Array	PD-peripheral blood dataset
GSM2630997	PD	peripheral blood	NA	male	GSE99039	GPL570	Affymetrix Human Genome U133 Plus 2.0 Array	PD-peripheral blood dataset
GSM2630998	PD	peripheral blood	NA	female	GSE99039	GPL570	Affymetrix Human Genome U133 Plus 2.0 Array	PD-peripheral blood dataset
GSM2630999	control	peripheral blood	NA	female	GSE99039	GPL570	Affymetrix Human Genome U133 Plus 2.0 Array	PD-peripheral blood dataset
GSM2631001	control	peripheral blood	NA	male	GSE99039	GPL570	Affymetrix Human Genome U133 Plus 2.0 Array	PD-peripheral blood dataset
GSM2631002	PD	peripheral blood	NA	male	GSE99039	GPL570	Affymetrix Human Genome U133 Plus 2.0 Array	PD-peripheral blood dataset
GSM2631003	PD	peripheral blood	NA	female	GSE99039	GPL570	Affymetrix Human Genome U133 Plus 2.0 Array	PD-peripheral blood dataset
GSM2631004	PD	peripheral blood	NA	female	GSE99039	GPL570	Affymetrix Human Genome U133 Plus 2.0 Array	PD-peripheral blood dataset
GSM2631005	control	peripheral blood	NA	female	GSE99039	GPL570	Affymetrix Human Genome U133 Plus 2.0 Array	PD-peripheral blood dataset
GSM2631006	PD	peripheral blood	NA	male	GSE99039	GPL570	Affymetrix Human Genome U133 Plus 2.0 Array	PD-peripheral blood dataset
GSM2631007	PD	peripheral blood	NA	male	GSE99039	GPL570	Affymetrix Human Genome U133 Plus 2.0 Array	PD-peripheral blood dataset
GSM2631008	PD	peripheral blood	NA	male	GSE99039	GPL570	Affymetrix Human Genome U133 Plus 2.0 Array	PD-peripheral blood dataset
GSM2631009	PD	peripheral blood	NA	male	GSE99039	GPL570	Affymetrix Human Genome U133 Plus 2.0 Array	PD-peripheral blood dataset
GSM2631010	control	peripheral blood	NA	female	GSE99039	GPL570	Affymetrix Human Genome U133 Plus 2.0 Array	PD-peripheral blood dataset
GSM2631011	control	peripheral blood	NA	male	GSE99039	GPL570	Affymetrix Human Genome U133 Plus 2.0 Array	PD-peripheral blood dataset
GSM2631012	control	peripheral blood	NA	male	GSE99039	GPL570	Affymetrix Human Genome U133 Plus 2.0 Array	PD-peripheral blood dataset
GSM2631013	PD	peripheral blood	NA	male	GSE99039	GPL570	Affymetrix Human Genome U133 Plus 2.0 Array	PD-peripheral blood dataset
GSM2631014	PD	peripheral blood	NA	male	GSE99039	GPL570	Affymetrix Human Genome U133 Plus 2.0 Array	PD-peripheral blood dataset
GSM2631015	control	peripheral blood	NA	female	GSE99039	GPL570	Affymetrix Human Genome U133 Plus 2.0 Array	PD-peripheral blood dataset
GSM2631016	control	peripheral blood	NA	female	GSE99039	GPL570	Affymetrix Human Genome U133 Plus 2.0 Array	PD-peripheral blood dataset
GSM2631017	control	peripheral blood	NA	female	GSE99039	GPL570	Affymetrix Human Genome U133 Plus 2.0 Array	PD-peripheral blood dataset
GSM2631019	PD	peripheral blood	NA	female	GSE99039	GPL570	Affymetrix Human Genome U133 Plus 2.0 Array	PD-peripheral blood dataset
GSM2631020	control	peripheral blood	NA	female	GSE99039	GPL570	Affymetrix Human Genome U133 Plus 2.0 Array	PD-peripheral blood dataset
GSM2631021	PD	peripheral blood	NA	male	GSE99039	GPL570	Affymetrix Human Genome U133 Plus 2.0 Array	PD-peripheral blood dataset
GSM2631022	control	peripheral blood	NA	male	GSE99039	GPL570	Affymetrix Human Genome U133 Plus 2.0 Array	PD-peripheral blood dataset
GSM2631023	control	peripheral blood	NA	female	GSE99039	GPL570	Affymetrix Human Genome U133 Plus 2.0 Array	PD-peripheral blood dataset
GSM2631024	control	peripheral blood	NA	female	GSE99039	GPL570	Affymetrix Human Genome U133 Plus 2.0 Array	PD-peripheral blood dataset
GSM2631025	control	peripheral blood	NA	female	GSE99039	GPL570	Affymetrix Human Genome U133 Plus 2.0 Array	PD-peripheral blood dataset
GSM2631026	control	peripheral blood	NA	female	GSE99039	GPL570	Affymetrix Human Genome U133 Plus 2.0 Array	PD-peripheral blood dataset
GSM2631027	control	peripheral blood	NA	male	GSE99039	GPL570	Affymetrix Human Genome U133 Plus 2.0 Array	PD-peripheral blood dataset
GSM2631028	control	peripheral blood	NA	female	GSE99039	GPL570	Affymetrix Human Genome U133 Plus 2.0 Array	PD-peripheral blood dataset
GSM2631029	control	peripheral blood	NA	female	GSE99039	GPL570	Affymetrix Human Genome U133 Plus 2.0 Array	PD-peripheral blood dataset
GSM2631030	PD	peripheral blood	NA	female	GSE99039	GPL570	Affymetrix Human Genome U133 Plus 2.0 Array	PD-peripheral blood dataset
GSM2631031	control	peripheral blood	NA	female	GSE99039	GPL570	Affymetrix Human Genome U133 Plus 2.0 Array	PD-peripheral blood dataset
GSM2631032	control	peripheral blood	NA	female	GSE99039	GPL570	Affymetrix Human Genome U133 Plus 2.0 Array	PD-peripheral blood dataset
GSM2631033	control	peripheral blood	NA	female	GSE99039	GPL570	Affymetrix Human Genome U133 Plus 2.0 Array	PD-peripheral blood dataset
GSM2631035	PD	peripheral blood	NA	female	GSE99039	GPL570	Affymetrix Human Genome U133 Plus 2.0 Array	PD-peripheral blood dataset
GSM2631036	PD	peripheral blood	NA	female	GSE99039	GPL570	Affymetrix Human Genome U133 Plus 2.0 Array	PD-peripheral blood dataset
GSM2631037	control	peripheral blood	NA	female	GSE99039	GPL570	Affymetrix Human Genome U133 Plus 2.0 Array	PD-peripheral blood dataset
GSM2631038	control	peripheral blood	NA	female	GSE99039	GPL570	Affymetrix Human Genome U133 Plus 2.0 Array	PD-peripheral blood dataset
GSM2631039	control	peripheral blood	NA	female	GSE99039	GPL570	Affymetrix Human Genome U133 Plus 2.0 Array	PD-peripheral blood dataset
GSM2631040	control	peripheral blood	NA	female	GSE99039	GPL570	Affymetrix Human Genome U133 Plus 2.0 Array	PD-peripheral blood dataset
GSM2631041	control	peripheral blood	NA	female	GSE99039	GPL570	Affymetrix Human Genome U133 Plus 2.0 Array	PD-peripheral blood dataset
GSM2631042	control	peripheral blood	NA	female	GSE99039	GPL570	Affymetrix Human Genome U133 Plus 2.0 Array	PD-peripheral blood dataset
GSM2631043	control	peripheral blood	NA	male	GSE99039	GPL570	Affymetrix Human Genome U133 Plus 2.0 Array	PD-peripheral blood dataset
GSM2631044	control	peripheral blood	NA	female	GSE99039	GPL570	Affymetrix Human Genome U133 Plus 2.0 Array	PD-peripheral blood dataset
GSM2631045	control	peripheral blood	NA	female	GSE99039	GPL570	Affymetrix Human Genome U133 Plus 2.0 Array	PD-peripheral blood dataset
GSM2631046	control	peripheral blood	NA	female	GSE99039	GPL570	Affymetrix Human Genome U133 Plus 2.0 Array	PD-peripheral blood dataset
GSM2631047	control	peripheral blood	NA	female	GSE99039	GPL570	Affymetrix Human Genome U133 Plus 2.0 Array	PD-peripheral blood dataset
GSM2631048	PD	peripheral blood	NA	female	GSE99039	GPL570	Affymetrix Human Genome U133 Plus 2.0 Array	PD-peripheral blood dataset

GSM2631049	control	peripheral blood	NA	female	GSE99039	GPL570	Affymetrix Human Genome U133 Plus 2.0 Array	PD-peripheral blood dataset
GSM2631050	PD	peripheral blood	NA	female	GSE99039	GPL570	Affymetrix Human Genome U133 Plus 2.0 Array	PD-peripheral blood dataset
GSM2631051	PD	peripheral blood	NA	male	GSE99039	GPL570	Affymetrix Human Genome U133 Plus 2.0 Array	PD-peripheral blood dataset
GSM2631052	PD	peripheral blood	NA	female	GSE99039	GPL570	Affymetrix Human Genome U133 Plus 2.0 Array	PD-peripheral blood dataset
GSM2631053	PD	peripheral blood	NA	male	GSE99039	GPL570	Affymetrix Human Genome U133 Plus 2.0 Array	PD-peripheral blood dataset
GSM2631054	control	peripheral blood	NA	female	GSE99039	GPL570	Affymetrix Human Genome U133 Plus 2.0 Array	PD-peripheral blood dataset
GSM2631055	control	peripheral blood	NA	male	GSE99039	GPL570	Affymetrix Human Genome U133 Plus 2.0 Array	PD-peripheral blood dataset
GSM2631056	control	peripheral blood	NA	female	GSE99039	GPL570	Affymetrix Human Genome U133 Plus 2.0 Array	PD-peripheral blood dataset
GSM2631057	control	peripheral blood	NA	female	GSE99039	GPL570	Affymetrix Human Genome U133 Plus 2.0 Array	PD-peripheral blood dataset
GSM2631058	control	peripheral blood	NA	female	GSE99039	GPL570	Affymetrix Human Genome U133 Plus 2.0 Array	PD-peripheral blood dataset
GSM2631059	control	peripheral blood	NA	female	GSE99039	GPL570	Affymetrix Human Genome U133 Plus 2.0 Array	PD-peripheral blood dataset
GSM2631060	PD	peripheral blood	NA	male	GSE99039	GPL570	Affymetrix Human Genome U133 Plus 2.0 Array	PD-peripheral blood dataset
GSM2631061	control	peripheral blood	NA	male	GSE99039	GPL570	Affymetrix Human Genome U133 Plus 2.0 Array	PD-peripheral blood dataset
GSM2631064	control	peripheral blood	NA	female	GSE99039	GPL570	Affymetrix Human Genome U133 Plus 2.0 Array	PD-peripheral blood dataset
GSM2631065	control	peripheral blood	NA	female	GSE99039	GPL570	Affymetrix Human Genome U133 Plus 2.0 Array	PD-peripheral blood dataset
GSM2631066	PD	peripheral blood	NA	male	GSE99039	GPL570	Affymetrix Human Genome U133 Plus 2.0 Array	PD-peripheral blood dataset
GSM2631067	PD	peripheral blood	NA	female	GSE99039	GPL570	Affymetrix Human Genome U133 Plus 2.0 Array	PD-peripheral blood dataset
GSM2631068	control	peripheral blood	NA	female	GSE99039	GPL570	Affymetrix Human Genome U133 Plus 2.0 Array	PD-peripheral blood dataset
GSM2631070	control	peripheral blood	NA	female	GSE99039	GPL570	Affymetrix Human Genome U133 Plus 2.0 Array	PD-peripheral blood dataset
GSM2631071	PD	peripheral blood	NA	male	GSE99039	GPL570	Affymetrix Human Genome U133 Plus 2.0 Array	PD-peripheral blood dataset
GSM2631072	control	peripheral blood	NA	female	GSE99039	GPL570	Affymetrix Human Genome U133 Plus 2.0 Array	PD-peripheral blood dataset
GSM2631073	control	peripheral blood	NA	male	GSE99039	GPL570	Affymetrix Human Genome U133 Plus 2.0 Array	PD-peripheral blood dataset
GSM2631074	control	peripheral blood	NA	male	GSE99039	GPL570	Affymetrix Human Genome U133 Plus 2.0 Array	PD-peripheral blood dataset
GSM2631075	control	peripheral blood	NA	female	GSE99039	GPL570	Affymetrix Human Genome U133 Plus 2.0 Array	PD-peripheral blood dataset
GSM2631076	control	peripheral blood	NA	male	GSE99039	GPL570	Affymetrix Human Genome U133 Plus 2.0 Array	PD-peripheral blood dataset
GSM2631077	control	peripheral blood	NA	female	GSE99039	GPL570	Affymetrix Human Genome U133 Plus 2.0 Array	PD-peripheral blood dataset
GSM2631078	PD	peripheral blood	NA	male	GSE99039	GPL570	Affymetrix Human Genome U133 Plus 2.0 Array	PD-peripheral blood dataset
GSM2631079	control	peripheral blood	NA	male	GSE99039	GPL570	Affymetrix Human Genome U133 Plus 2.0 Array	PD-peripheral blood dataset
GSM2631080	control	peripheral blood	NA	female	GSE99039	GPL570	Affymetrix Human Genome U133 Plus 2.0 Array	PD-peripheral blood dataset
GSM2631081	control	peripheral blood	NA	female	GSE99039	GPL570	Affymetrix Human Genome U133 Plus 2.0 Array	PD-peripheral blood dataset
GSM2631082	control	peripheral blood	NA	female	GSE99039	GPL570	Affymetrix Human Genome U133 Plus 2.0 Array	PD-peripheral blood dataset
GSM2631083	control	peripheral blood	NA	male	GSE99039	GPL570	Affymetrix Human Genome U133 Plus 2.0 Array	PD-peripheral blood dataset
GSM2631084	control	peripheral blood	NA	female	GSE99039	GPL570	Affymetrix Human Genome U133 Plus 2.0 Array	PD-peripheral blood dataset
GSM2631085	control	peripheral blood	NA	female	GSE99039	GPL570	Affymetrix Human Genome U133 Plus 2.0 Array	PD-peripheral blood dataset
GSM2631089	PD	peripheral blood	NA	female	GSE99039	GPL570	Affymetrix Human Genome U133 Plus 2.0 Array	PD-peripheral blood dataset
GSM2631090	PD	peripheral blood	NA	female	GSE99039	GPL570	Affymetrix Human Genome U133 Plus 2.0 Array	PD-peripheral blood dataset
GSM2631091	control	peripheral blood	NA	female	GSE99039	GPL570	Affymetrix Human Genome U133 Plus 2.0 Array	PD-peripheral blood dataset
GSM2631094	PD	peripheral blood	NA	male	GSE99039	GPL570	Affymetrix Human Genome U133 Plus 2.0 Array	PD-peripheral blood dataset
GSM2631095	PD	peripheral blood	NA	female	GSE99039	GPL570	Affymetrix Human Genome U133 Plus 2.0 Array	PD-peripheral blood dataset
GSM2631096	PD	peripheral blood	NA	male	GSE99039	GPL570	Affymetrix Human Genome U133 Plus 2.0 Array	PD-peripheral blood dataset
GSM2631097	PD	peripheral blood	NA	female	GSE99039	GPL570	Affymetrix Human Genome U133 Plus 2.0 Array	PD-peripheral blood dataset
GSM2631099	PD	peripheral blood	NA	female	GSE99039	GPL570	Affymetrix Human Genome U133 Plus 2.0 Array	PD-peripheral blood dataset
GSM2631100	control	peripheral blood	NA	male	GSE99039	GPL570	Affymetrix Human Genome U133 Plus 2.0 Array	PD-peripheral blood dataset
GSM2631101	control	peripheral blood	NA	female	GSE99039	GPL570	Affymetrix Human Genome U133 Plus 2.0 Array	PD-peripheral blood dataset
GSM2631102	control	peripheral blood	NA	female	GSE99039	GPL570	Affymetrix Human Genome U133 Plus 2.0 Array	PD-peripheral blood dataset
GSM2631103	control	peripheral blood	NA	female	GSE99039	GPL570	Affymetrix Human Genome U133 Plus 2.0 Array	PD-peripheral blood dataset
GSM2631104	PD	peripheral blood	NA	male	GSE99039	GPL570	Affymetrix Human Genome U133 Plus 2.0 Array	PD-peripheral blood dataset
GSM2631105	control	peripheral blood	NA	male	GSE99039	GPL570	Affymetrix Human Genome U133 Plus 2.0 Array	PD-peripheral blood dataset
GSM2631106	PD	peripheral blood	NA	female	GSE99039	GPL570	Affymetrix Human Genome U133 Plus 2.0 Array	PD-peripheral blood dataset
GSM2631107	PD	peripheral blood	NA	male	GSE99039	GPL570	Affymetrix Human Genome U133 Plus 2.0 Array	PD-peripheral blood dataset
GSM2631108	PD	peripheral blood	NA	male	GSE99039	GPL570	Affymetrix Human Genome U133 Plus 2.0 Array	PD-peripheral blood dataset
GSM2631109	control	peripheral blood	NA	female	GSE99039	GPL570	Affymetrix Human Genome U133 Plus 2.0 Array	PD-peripheral blood dataset
GSM2631110	control	peripheral blood	NA	female	GSE99039	GPL570	Affymetrix Human Genome U133 Plus 2.0 Array	PD-peripheral blood dataset
GSM2631111	control	peripheral blood	NA	female	GSE99039	GPL570	Affymetrix Human Genome U133 Plus 2.0 Array	PD-peripheral blood dataset
GSM2631112	control	peripheral blood	NA	female	GSE99039	GPL570	Affymetrix Human Genome U133 Plus 2.0 Array	PD-peripheral blood dataset
GSM2631114	PD	peripheral blood	NA	female	GSE99039	GPL570	Affymetrix Human Genome U133 Plus 2.0 Array	PD-peripheral blood dataset
GSM2631115	PD	peripheral blood	NA	male	GSE99039	GPL570	Affymetrix Human Genome U133 Plus 2.0 Array	PD-peripheral blood dataset
GSM2631116	control	peripheral blood	NA	female	GSE99039	GPL570	Affymetrix Human Genome U133 Plus 2.0 Array	PD-peripheral blood dataset
GSM2631117	PD	peripheral blood	NA	male	GSE99039	GPL570	Affymetrix Human Genome U133 Plus 2.0 Array	PD-peripheral blood dataset
GSM2631118	control	peripheral blood	NA	male	GSE99039	GPL570	Affymetrix Human Genome U133 Plus 2.0 Array	PD-peripheral blood dataset
GSM2631119	PD	peripheral blood	NA	female	GSE99039	GPL570	Affymetrix Human Genome U133 Plus 2.0 Array	PD-peripheral blood dataset
GSM2631120	PD	peripheral blood	NA	female	GSE99039	GPL570	Affymetrix Human Genome U133 Plus 2.0 Array	PD-peripheral blood dataset
GSM2631121	control	peripheral blood	NA	male	GSE99039	GPL570	Affymetrix Human Genome U133 Plus 2.0 Array	PD-peripheral blood dataset
GSM2631122	control	peripheral blood	NA	male	GSE99039	GPL570	Affymetrix Human Genome U133 Plus 2.0 Array	PD-peripheral blood dataset
GSM2631123	control	peripheral blood	NA	female	GSE99039	GPL570	Affymetrix Human Genome U133 Plus 2.0 Array	PD-peripheral blood dataset
GSM2631124	PD	peripheral blood	NA	female	GSE99039	GPL570	Affymetrix Human Genome U133 Plus 2.0 Array	PD-peripheral blood dataset
GSM2631125	control	peripheral blood	NA	female	GSE99039	GPL570	Affymetrix Human Genome U133 Plus 2.0 Array	PD-peripheral blood dataset
GSM2631126	control	peripheral blood	NA	female	GSE99039	GPL570	Affymetrix Human Genome U133 Plus 2.0 Array	PD-peripheral blood dataset
GSM2631127	PD	peripheral blood	NA	male	GSE99039	GPL570	Affymetrix Human Genome U133 Plus 2.0 Array	PD-peripheral blood dataset
GSM2631128	PD	peripheral blood	NA	male	GSE99039	GPL570	Affymetrix Human Genome U133 Plus 2.0 Array	PD-peripheral blood dataset
GSM2631129	PD	peripheral blood	NA	male	GSE99039	GPL570	Affymetrix Human Genome U133 Plus 2.0 Array	PD-peripheral blood dataset
GSM2631130	control	peripheral blood	NA	female	GSE99039	GPL570	Affymetrix Human Genome U133 Plus 2.0 Array	PD-peripheral blood dataset
GSM2631131	control	peripheral blood	NA	female	GSE99039	GPL570	Affymetrix Human Genome U133 Plus 2.0 Array	PD-peripheral blood dataset
GSM2631132	control	peripheral blood	NA	female	GSE99039	GPL570	Affymetrix Human Genome U133 Plus 2.0 Array	PD-peripheral blood dataset
GSM2631133	control	peripheral blood	NA	female	GSE99039	GPL570	Affymetrix Human Genome U133 Plus 2.0 Array	PD-peripheral blood dataset
GSM2631134	PD	peripheral blood	NA	male	GSE99039	GPL570	Affymetrix Human Genome U133 Plus 2.0 Array	PD-peripheral blood dataset
GSM2631135	control	peripheral blood	NA	male	GSE99039	GPL570	Affymetrix Human Genome U133 Plus 2.0 Array	PD-peripheral blood dataset
GSM2631136	control	peripheral blood	NA	female	GSE99039	GPL570	Affymetrix Human Genome U133 Plus 2.0 Array	PD-peripheral blood dataset
GSM2631137	control	peripheral blood	NA	female	GSE99039	GPL570	Affymetrix Human Genome U133 Plus 2.0 Array	PD-peripheral blood dataset
GSM2631138	control	peripheral blood	NA	female	GSE99039	GPL570	Affymetrix Human Genome U133 Plus 2.0 Array	PD-peripheral blood dataset
GSM2631140	control	peripheral blood	NA	female	GSE99039	GPL570	Affymetrix Human Genome U133 Plus 2.0 Array	PD-peripheral blood dataset
GSM2631142	control	peripheral blood	NA	female	GSE99039	GPL570	Affymetrix Human Genome U133 Plus 2.0 Array	PD-peripheral blood dataset
GSM2631143	control	peripheral blood	NA	female	GSE99039	GPL570	Affymetrix Human Genome U133 Plus 2.0 Array	PD-peripheral blood dataset
GSM2631144	PD	peripheral blood	NA	male	GSE99039	GPL570	Affymetrix Human Genome U133 Plus 2.0 Array	PD-peripheral blood dataset
GSM2631146	PD	peripheral blood	NA	male	GSE99039	GPL570	Affymetrix Human Genome U133 Plus 2.0 Array	PD-peripheral blood dataset
GSM2631148	PD	peripheral blood	NA	female	GSE99039	GPL570	Affymetrix Human Genome U133 Plus 2.0 Array	PD-peripheral blood dataset
GSM2631149	control	peripheral blood	NA	male	GSE99039	GPL570	Affymetrix Human Genome U133 Plus 2.0 Array	PD-peripheral blood dataset
GSM2631150	PD	peripheral blood	NA	NA	GSE99039	GPL570	Affymetrix Human Genome U133 Plus 2.0 Array	PD-peripheral blood dataset
GSM2631151	PD	peripheral blood	NA	NA	GSE99039	GPL570	Affymetrix Human Genome U133 Plus 2.0 Array	PD-peripheral blood dataset
GSM2631152	control	peripheral blood	NA	NA	GSE99039	GPL570	Affymetrix Human Genome U133 Plus 2.0 Array	PD-peripheral blood dataset
GSM2631153	control	peripheral blood	NA	NA	GSE99039	GPL570	Affymetrix Human Genome U133 Plus 2.0 Array	PD-peripheral blood dataset
GSM2631154	PD	peripheral blood	NA	NA	GSE99039	GPL570	Affymetrix Human Genome U133 Plus 2.0 Array	PD-peripheral blood dataset

GSM2631155	PD	peripheral blood	NA	NA	GSE99039	GPL570	Affymetrix Human Genome U133 Plus 2.0 Array	PD-peripheral blood dataset
GSM2631156	PD	peripheral blood	NA	NA	GSE99039	GPL570	Affymetrix Human Genome U133 Plus 2.0 Array	PD-peripheral blood dataset
GSM2631157	PD	peripheral blood	NA	NA	GSE99039	GPL570	Affymetrix Human Genome U133 Plus 2.0 Array	PD-peripheral blood dataset
GSM2631158	control	peripheral blood	NA	NA	GSE99039	GPL570	Affymetrix Human Genome U133 Plus 2.0 Array	PD-peripheral blood dataset
GSM2631159	control	peripheral blood	NA	NA	GSE99039	GPL570	Affymetrix Human Genome U133 Plus 2.0 Array	PD-peripheral blood dataset
GSM2631160	PD	peripheral blood	NA	NA	GSE99039	GPL570	Affymetrix Human Genome U133 Plus 2.0 Array	PD-peripheral blood dataset
GSM2631161	control	peripheral blood	NA	NA	GSE99039	GPL570	Affymetrix Human Genome U133 Plus 2.0 Array	PD-peripheral blood dataset
GSM2631162	control	peripheral blood	NA	NA	GSE99039	GPL570	Affymetrix Human Genome U133 Plus 2.0 Array	PD-peripheral blood dataset
GSM2631163	control	peripheral blood	NA	NA	GSE99039	GPL570	Affymetrix Human Genome U133 Plus 2.0 Array	PD-peripheral blood dataset
GSM2631164	PD	peripheral blood	NA	NA	GSE99039	GPL570	Affymetrix Human Genome U133 Plus 2.0 Array	PD-peripheral blood dataset
GSM2631165	control	peripheral blood	NA	NA	GSE99039	GPL570	Affymetrix Human Genome U133 Plus 2.0 Array	PD-peripheral blood dataset
GSM2631166	PD	peripheral blood	NA	NA	GSE99039	GPL570	Affymetrix Human Genome U133 Plus 2.0 Array	PD-peripheral blood dataset
GSM2631167	control	peripheral blood	NA	NA	GSE99039	GPL570	Affymetrix Human Genome U133 Plus 2.0 Array	PD-peripheral blood dataset
GSM2631168	PD	peripheral blood	NA	NA	GSE99039	GPL570	Affymetrix Human Genome U133 Plus 2.0 Array	PD-peripheral blood dataset
GSM2631169	PD	peripheral blood	NA	NA	GSE99039	GPL570	Affymetrix Human Genome U133 Plus 2.0 Array	PD-peripheral blood dataset
GSM2631170	PD	peripheral blood	NA	NA	GSE99039	GPL570	Affymetrix Human Genome U133 Plus 2.0 Array	PD-peripheral blood dataset
GSM2631172	control	peripheral blood	NA	NA	GSE99039	GPL570	Affymetrix Human Genome U133 Plus 2.0 Array	PD-peripheral blood dataset
GSM2631173	control	peripheral blood	NA	female	GSE99039	GPL570	Affymetrix Human Genome U133 Plus 2.0 Array	PD-peripheral blood dataset
GSM2631175	control	peripheral blood	NA	NA	GSE99039	GPL570	Affymetrix Human Genome U133 Plus 2.0 Array	PD-peripheral blood dataset
GSM2631176	control	peripheral blood	NA	NA	GSE99039	GPL570	Affymetrix Human Genome U133 Plus 2.0 Array	PD-peripheral blood dataset
GSM2631177	control	peripheral blood	NA	NA	GSE99039	GPL570	Affymetrix Human Genome U133 Plus 2.0 Array	PD-peripheral blood dataset
GSM2631178	PD	peripheral blood	NA	NA	GSE99039	GPL570	Affymetrix Human Genome U133 Plus 2.0 Array	PD-peripheral blood dataset
GSM2631179	control	peripheral blood	NA	male	GSE99039	GPL570	Affymetrix Human Genome U133 Plus 2.0 Array	PD-peripheral blood dataset
GSM2631180	control	peripheral blood	NA	female	GSE99039	GPL570	Affymetrix Human Genome U133 Plus 2.0 Array	PD-peripheral blood dataset
GSM2631181	control	peripheral blood	NA	male	GSE99039	GPL570	Affymetrix Human Genome U133 Plus 2.0 Array	PD-peripheral blood dataset
GSM2631182	PD	peripheral blood	NA	male	GSE99039	GPL570	Affymetrix Human Genome U133 Plus 2.0 Array	PD-peripheral blood dataset
GSM2631183	PD	peripheral blood	NA	male	GSE99039	GPL570	Affymetrix Human Genome U133 Plus 2.0 Array	PD-peripheral blood dataset
GSM2631184	PD	peripheral blood	NA	male	GSE99039	GPL570	Affymetrix Human Genome U133 Plus 2.0 Array	PD-peripheral blood dataset
GSM2631185	control	peripheral blood	NA	female	GSE99039	GPL570	Affymetrix Human Genome U133 Plus 2.0 Array	PD-peripheral blood dataset
GSM2631186	PD	peripheral blood	NA	female	GSE99039	GPL570	Affymetrix Human Genome U133 Plus 2.0 Array	PD-peripheral blood dataset
GSM2631187	PD	peripheral blood	NA	female	GSE99039	GPL570	Affymetrix Human Genome U133 Plus 2.0 Array	PD-peripheral blood dataset
GSM2631188	PD	peripheral blood	NA	female	GSE99039	GPL570	Affymetrix Human Genome U133 Plus 2.0 Array	PD-peripheral blood dataset
GSM2631189	PD	peripheral blood	NA	female	GSE99039	GPL570	Affymetrix Human Genome U133 Plus 2.0 Array	PD-peripheral blood dataset
GSM2631190	PD	peripheral blood	NA	female	GSE99039	GPL570	Affymetrix Human Genome U133 Plus 2.0 Array	PD-peripheral blood dataset
GSM2631191	control	peripheral blood	NA	female	GSE99039	GPL570	Affymetrix Human Genome U133 Plus 2.0 Array	PD-peripheral blood dataset
GSM2631192	PD	peripheral blood	NA	female	GSE99039	GPL570	Affymetrix Human Genome U133 Plus 2.0 Array	PD-peripheral blood dataset
GSM2631194	control	peripheral blood	NA	male	GSE99039	GPL570	Affymetrix Human Genome U133 Plus 2.0 Array	PD-peripheral blood dataset
GSM2631195	control	peripheral blood	NA	male	GSE99039	GPL570	Affymetrix Human Genome U133 Plus 2.0 Array	PD-peripheral blood dataset
GSM2631196	control	peripheral blood	NA	male	GSE99039	GPL570	Affymetrix Human Genome U133 Plus 2.0 Array	PD-peripheral blood dataset
GSM2631197	control	peripheral blood	NA	male	GSE99039	GPL570	Affymetrix Human Genome U133 Plus 2.0 Array	PD-peripheral blood dataset
GSM2631198	control	peripheral blood	NA	male	GSE99039	GPL570	Affymetrix Human Genome U133 Plus 2.0 Array	PD-peripheral blood dataset
GSM2631200	PD	peripheral blood	NA	female	GSE99039	GPL570	Affymetrix Human Genome U133 Plus 2.0 Array	PD-peripheral blood dataset
GSM2631220	PD	peripheral blood	NA	male	GSE99039	GPL570	Affymetrix Human Genome U133 Plus 2.0 Array	PD-peripheral blood dataset
GSM2631221	control	peripheral blood	NA	male	GSE99039	GPL570	Affymetrix Human Genome U133 Plus 2.0 Array	PD-peripheral blood dataset
GSM2631224	PD	peripheral blood	NA	male	GSE99039	GPL570	Affymetrix Human Genome U133 Plus 2.0 Array	PD-peripheral blood dataset
GSM2631225	PD	peripheral blood	NA	male	GSE99039	GPL570	Affymetrix Human Genome U133 Plus 2.0 Array	PD-peripheral blood dataset
GSM2631227	control	peripheral blood	NA	female	GSE99039	GPL570	Affymetrix Human Genome U133 Plus 2.0 Array	PD-peripheral blood dataset
GSM2631230	control	peripheral blood	NA	male	GSE99039	GPL570	Affymetrix Human Genome U133 Plus 2.0 Array	PD-peripheral blood dataset
GSM2631231	control	peripheral blood	NA	female	GSE99039	GPL570	Affymetrix Human Genome U133 Plus 2.0 Array	PD-peripheral blood dataset
GSM2631232	control	peripheral blood	NA	male	GSE99039	GPL570	Affymetrix Human Genome U133 Plus 2.0 Array	PD-peripheral blood dataset
GSM2631234	control	peripheral blood	NA	male	GSE99039	GPL570	Affymetrix Human Genome U133 Plus 2.0 Array	PD-peripheral blood dataset
GSM2631235	control	peripheral blood	NA	female	GSE99039	GPL570	Affymetrix Human Genome U133 Plus 2.0 Array	PD-peripheral blood dataset
GSM2631236	control	peripheral blood	NA	female	GSE99039	GPL570	Affymetrix Human Genome U133 Plus 2.0 Array	PD-peripheral blood dataset
GSM2631237	control	peripheral blood	NA	male	GSE99039	GPL570	Affymetrix Human Genome U133 Plus 2.0 Array	PD-peripheral blood dataset
GSM2631238	control	peripheral blood	NA	female	GSE99039	GPL570	Affymetrix Human Genome U133 Plus 2.0 Array	PD-peripheral blood dataset
GSM2631239	control	peripheral blood	NA	female	GSE99039	GPL570	Affymetrix Human Genome U133 Plus 2.0 Array	PD-peripheral blood dataset
GSM2631240	control	peripheral blood	NA	male	GSE99039	GPL570	Affymetrix Human Genome U133 Plus 2.0 Array	PD-peripheral blood dataset
GSM2631241	control	peripheral blood	NA	female	GSE99039	GPL570	Affymetrix Human Genome U133 Plus 2.0 Array	PD-peripheral blood dataset
GSM2631242	control	peripheral blood	NA	male	GSE99039	GPL570	Affymetrix Human Genome U133 Plus 2.0 Array	PD-peripheral blood dataset
GSM2631243	control	peripheral blood	NA	female	GSE99039	GPL570	Affymetrix Human Genome U133 Plus 2.0 Array	PD-peripheral blood dataset
GSM2631244	PD	peripheral blood	NA	male	GSE99039	GPL570	Affymetrix Human Genome U133 Plus 2.0 Array	PD-peripheral blood dataset
GSM2631245	PD	peripheral blood	NA	male	GSE99039	GPL570	Affymetrix Human Genome U133 Plus 2.0 Array	PD-peripheral blood dataset
GSM2631246	PD	peripheral blood	NA	male	GSE99039	GPL570	Affymetrix Human Genome U133 Plus 2.0 Array	PD-peripheral blood dataset
GSM2631247	PD	peripheral blood	NA	male	GSE99039	GPL570	Affymetrix Human Genome U133 Plus 2.0 Array	PD-peripheral blood dataset
GSM2631248	control	peripheral blood	NA	male	GSE99039	GPL570	Affymetrix Human Genome U133 Plus 2.0 Array	PD-peripheral blood dataset
GSM2631249	PD	peripheral blood	NA	female	GSE99039	GPL570	Affymetrix Human Genome U133 Plus 2.0 Array	PD-peripheral blood dataset
GSM2631252	control	peripheral blood	NA	female	GSE99039	GPL570	Affymetrix Human Genome U133 Plus 2.0 Array	PD-peripheral blood dataset
GSM2631253	PD	peripheral blood	NA	male	GSE99039	GPL570	Affymetrix Human Genome U133 Plus 2.0 Array	PD-peripheral blood dataset
GSM2631254	PD	peripheral blood	NA	male	GSE99039	GPL570	Affymetrix Human Genome U133 Plus 2.0 Array	PD-peripheral blood dataset
GSM2631256	PD	peripheral blood	NA	female	GSE99039	GPL570	Affymetrix Human Genome U133 Plus 2.0 Array	PD-peripheral blood dataset
GSM2631257	PD	peripheral blood	NA	female	GSE99039	GPL570	Affymetrix Human Genome U133 Plus 2.0 Array	PD-peripheral blood dataset
GSM2631258	PD	peripheral blood	NA	female	GSE99039	GPL570	Affymetrix Human Genome U133 Plus 2.0 Array	PD-peripheral blood dataset
GSM2631259	PD	peripheral blood	NA	female	GSE99039	GPL570	Affymetrix Human Genome U133 Plus 2.0 Array	PD-peripheral blood dataset
GSM2631260	PD	peripheral blood	NA	female	GSE99039	GPL570	Affymetrix Human Genome U133 Plus 2.0 Array	PD-peripheral blood dataset
GSM2631261	PD	peripheral blood	NA	female	GSE99039	GPL570	Affymetrix Human Genome U133 Plus 2.0 Array	PD-peripheral blood dataset
GSM2631262	PD	peripheral blood	NA	female	GSE99039	GPL570	Affymetrix Human Genome U133 Plus 2.0 Array	PD-peripheral blood dataset
GSM2631263	PD	peripheral blood	NA	female	GSE99039	GPL570	Affymetrix Human Genome U133 Plus 2.0 Array	PD-peripheral blood dataset
GSM2631264	PD	peripheral blood	NA	female	GSE99039	GPL570	Affymetrix Human Genome U133 Plus 2.0 Array	PD-peripheral blood dataset
GSM2631265	PD	peripheral blood	NA	female	GSE99039	GPL570	Affymetrix Human Genome U133 Plus 2.0 Array	PD-peripheral blood dataset
GSM2631266	PD	peripheral blood	NA	male	GSE99039	GPL570	Affymetrix Human Genome U133 Plus 2.0 Array	PD-peripheral blood dataset
GSM2631267	PD	peripheral blood	NA	female	GSE99039	GPL570	Affymetrix Human Genome U133 Plus 2.0 Array	PD-peripheral blood dataset
GSM2631268	PD	peripheral blood	NA	male	GSE99039	GPL570	Affymetrix Human Genome U133 Plus 2.0 Array	PD-peripheral blood dataset
GSM2631269	PD	peripheral blood	NA	male	GSE99039	GPL570	Affymetrix Human Genome U133 Plus 2.0 Array	PD-peripheral blood dataset
GSM2631271	PD	peripheral blood	NA	female	GSE99039	GPL570	Affymetrix Human Genome U133 Plus 2.0 Array	PD-peripheral blood dataset
GSM2631272	control	peripheral blood	NA	male	GSE99039	GPL570	Affymetrix Human Genome U133 Plus 2.0 Array	PD-peripheral blood dataset
GSM2631273	control	peripheral blood	NA	male	GSE99039	GPL570	Affymetrix Human Genome U133 Plus 2.0 Array	PD-peripheral blood dataset
GSM2631274	control	peripheral blood	NA	female	GSE99039	GPL570	Affymetrix Human Genome U133 Plus 2.0 Array	PD-peripheral blood dataset
GSM2631275	control	peripheral blood	NA	male	GSE99039	GPL570	Affymetrix Human Genome U133 Plus 2.0 Array	PD-peripheral blood dataset
GSM2631276	control	peripheral blood	NA	female	GSE99039	GPL570	Affymetrix Human Genome U133 Plus 2.0 Array	PD-peripheral blood dataset
GSM2631277	control	peripheral blood	NA	female	GSE99039	GPL570	Affymetrix Human Genome U133 Plus 2.0 Array	PD-peripheral blood dataset
GSM2631278	control	peripheral blood	NA	male	GSE99039	GPL570	Affymetrix Human Genome U133 Plus 2.0 Array	PD-peripheral blood dataset
GSM2631279	control	peripheral blood	NA	female	GSE99039	GPL570	Affymetrix Human Genome U133 Plus 2.0 Array	PD-peripheral blood dataset

GSM2631280	control	peripheral blood	NA	female	GSE99039	GPL570	Affymetrix Human Genome U133 Plus 2.0 Array	PD-peripheral blood dataset
GSM2631281	control	peripheral blood	NA	male	GSE99039	GPL570	Affymetrix Human Genome U133 Plus 2.0 Array	PD-peripheral blood dataset
GSM2631283	control	peripheral blood	NA	female	GSE99039	GPL570	Affymetrix Human Genome U133 Plus 2.0 Array	PD-peripheral blood dataset
GSM2631284	control	peripheral blood	NA	female	GSE99039	GPL570	Affymetrix Human Genome U133 Plus 2.0 Array	PD-peripheral blood dataset
GSM2631287	control	peripheral blood	NA	female	GSE99039	GPL570	Affymetrix Human Genome U133 Plus 2.0 Array	PD-peripheral blood dataset
GSM2631289	control	peripheral blood	NA	female	GSE99039	GPL570	Affymetrix Human Genome U133 Plus 2.0 Array	PD-peripheral blood dataset
GSM2631290	PD	peripheral blood	NA	male	GSE99039	GPL570	Affymetrix Human Genome U133 Plus 2.0 Array	PD-peripheral blood dataset
GSM2631291	PD	peripheral blood	NA	male	GSE99039	GPL570	Affymetrix Human Genome U133 Plus 2.0 Array	PD-peripheral blood dataset
GSM2631293	PD	peripheral blood	NA	male	GSE99039	GPL570	Affymetrix Human Genome U133 Plus 2.0 Array	PD-peripheral blood dataset
GSM2631295	control	peripheral blood	NA	female	GSE99039	GPL570	Affymetrix Human Genome U133 Plus 2.0 Array	PD-peripheral blood dataset
GSM2631296	PD	peripheral blood	NA	male	GSE99039	GPL570	Affymetrix Human Genome U133 Plus 2.0 Array	PD-peripheral blood dataset
GSM2631297	PD	peripheral blood	NA	male	GSE99039	GPL570	Affymetrix Human Genome U133 Plus 2.0 Array	PD-peripheral blood dataset
GSM2631298	control	peripheral blood	NA	NA	GSE99039	GPL570	Affymetrix Human Genome U133 Plus 2.0 Array	PD-peripheral blood dataset
GSM2631300	control	peripheral blood	NA	NA	GSE99039	GPL570	Affymetrix Human Genome U133 Plus 2.0 Array	PD-peripheral blood dataset
GSM2631301	control	peripheral blood	NA	NA	GSE99039	GPL570	Affymetrix Human Genome U133 Plus 2.0 Array	PD-peripheral blood dataset
GSM2631304	control	peripheral blood	NA	NA	GSE99039	GPL570	Affymetrix Human Genome U133 Plus 2.0 Array	PD-peripheral blood dataset
GSM2631305	control	peripheral blood	NA	NA	GSE99039	GPL570	Affymetrix Human Genome U133 Plus 2.0 Array	PD-peripheral blood dataset
GSM2631306	control	peripheral blood	NA	NA	GSE99039	GPL570	Affymetrix Human Genome U133 Plus 2.0 Array	PD-peripheral blood dataset
GSM2631310	control	peripheral blood	NA	NA	GSE99039	GPL570	Affymetrix Human Genome U133 Plus 2.0 Array	PD-peripheral blood dataset
GSM2631312	control	peripheral blood	NA	NA	GSE99039	GPL570	Affymetrix Human Genome U133 Plus 2.0 Array	PD-peripheral blood dataset
GSM2668809	control	peripheral blood	67	male	GSE100054	GPL23126	Affymetrix Human Clariom D Assay	PD-peripheral blood dataset
GSM2668810	control	peripheral blood	54	male	GSE100054	GPL23126	Affymetrix Human Clariom D Assay	PD-peripheral blood dataset
GSM2668811	control	peripheral blood	53	female	GSE100054	GPL23126	Affymetrix Human Clariom D Assay	PD-peripheral blood dataset
GSM2668812	control	peripheral blood	65	female	GSE100054	GPL23126	Affymetrix Human Clariom D Assay	PD-peripheral blood dataset
GSM2668813	control	peripheral blood	60	female	GSE100054	GPL23126	Affymetrix Human Clariom D Assay	PD-peripheral blood dataset
GSM2668814	control	peripheral blood	60	male	GSE100054	GPL23126	Affymetrix Human Clariom D Assay	PD-peripheral blood dataset
GSM2668815	control	peripheral blood	60	male	GSE100054	GPL23126	Affymetrix Human Clariom D Assay	PD-peripheral blood dataset
GSM2668816	control	peripheral blood	64	female	GSE100054	GPL23126	Affymetrix Human Clariom D Assay	PD-peripheral blood dataset
GSM2668817	control	peripheral blood	78	male	GSE100054	GPL23126	Affymetrix Human Clariom D Assay	PD-peripheral blood dataset
GSM2668818	PD	peripheral blood	67	male	GSE100054	GPL23126	Affymetrix Human Clariom D Assay	PD-peripheral blood dataset
GSM2668819	PD	peripheral blood	75	male	GSE100054	GPL23126	Affymetrix Human Clariom D Assay	PD-peripheral blood dataset
GSM2668820	PD	peripheral blood	55	female	GSE100054	GPL23126	Affymetrix Human Clariom D Assay	PD-peripheral blood dataset
GSM2668821	PD	peripheral blood	70	female	GSE100054	GPL23126	Affymetrix Human Clariom D Assay	PD-peripheral blood dataset
GSM2668822	PD	peripheral blood	58	male	GSE100054	GPL23126	Affymetrix Human Clariom D Assay	PD-peripheral blood dataset
GSM2668823	PD	peripheral blood	80	male	GSE100054	GPL23126	Affymetrix Human Clariom D Assay	PD-peripheral blood dataset
GSM2668824	PD	peripheral blood	70	female	GSE100054	GPL23126	Affymetrix Human Clariom D Assay	PD-peripheral blood dataset
GSM2668825	PD	peripheral blood	53	male	GSE100054	GPL23126	Affymetrix Human Clariom D Assay	PD-peripheral blood dataset
GSM2668826	PD	peripheral blood	69	female	GSE100054	GPL23126	Affymetrix Human Clariom D Assay	PD-peripheral blood dataset
GSM2668827	PD	peripheral blood	73	female	GSE100054	GPL23126	Affymetrix Human Clariom D Assay	PD-peripheral blood dataset

Supplementary Table 10. Prefrontal cortex sample's informations from GSE150696.

Sample accession	disease state	Tissue	Age	Gender	GEO accession	Platform	data_processing
GSM4556859	control	prefrontal cortex	79	female	GSE150696	GPL17585	Affymetrix Human Transcriptome Array 2.0
GSM4556860	control	prefrontal cortex	96	female	GSE150696	GPL17585	Affymetrix Human Transcriptome Array 2.0
GSM4556861	control	prefrontal cortex	85	male	GSE150696	GPL17585	Affymetrix Human Transcriptome Array 2.0
GSM4556862	control	prefrontal cortex	90	female	GSE150696	GPL17585	Affymetrix Human Transcriptome Array 2.0
GSM4556863	control	prefrontal cortex	89	female	GSE150696	GPL17585	Affymetrix Human Transcriptome Array 2.0
GSM4556864	control	prefrontal cortex	86	male	GSE150696	GPL17585	Affymetrix Human Transcriptome Array 2.0
GSM4556865	control	prefrontal cortex	80	male	GSE150696	GPL17585	Affymetrix Human Transcriptome Array 2.0
GSM4556866	control	prefrontal cortex	77	male	GSE150696	GPL17585	Affymetrix Human Transcriptome Array 2.0
GSM4556867	control	prefrontal cortex	80	female	GSE150696	GPL17585	Affymetrix Human Transcriptome Array 2.0
GSM4556868	DLB	prefrontal cortex	92	female	GSE150696	GPL17585	Affymetrix Human Transcriptome Array 2.0
GSM4556869	DLB	prefrontal cortex	80	female	GSE150696	GPL17585	Affymetrix Human Transcriptome Array 2.0
GSM4556870	DLB	prefrontal cortex	70	female	GSE150696	GPL17585	Affymetrix Human Transcriptome Array 2.0
GSM4556872	DLB	prefrontal cortex	88	female	GSE150696	GPL17585	Affymetrix Human Transcriptome Array 2.0
GSM4556875	DLB	prefrontal cortex	91	female	GSE150696	GPL17585	Affymetrix Human Transcriptome Array 2.0
GSM4556876	DLB	prefrontal cortex	75	female	GSE150696	GPL17585	Affymetrix Human Transcriptome Array 2.0
GSM4556877	DLB	prefrontal cortex	82	male	GSE150696	GPL17585	Affymetrix Human Transcriptome Array 2.0
GSM4556878	DLB	prefrontal cortex	75	male	GSE150696	GPL17585	Affymetrix Human Transcriptome Array 2.0
GSM4556879	DLB	prefrontal cortex	84	male	GSE150696	GPL17585	Affymetrix Human Transcriptome Array 2.0
GSM4556880	DLB	prefrontal cortex	77	male	GSE150696	GPL17585	Affymetrix Human Transcriptome Array 2.0
GSM4556881	DLB	prefrontal cortex	71	male	GSE150696	GPL17585	Affymetrix Human Transcriptome Array 2.0
GSM4556882	DLB	prefrontal cortex	77	male	GSE150696	GPL17585	Affymetrix Human Transcriptome Array 2.0

Supplementary Table 11. Cerebellar white matter sample's informations from GSE199258.

Sample accession	disease state	Tissue	Age	Gender	GEO accession	Platform	data_processing
GSM5968282	MSA	cerebellar white matter	67	female	GSE199258	GPL16791	Illumina HiSeq 2500 (Homo sapiens)
GSM5968283	MSA	cerebellar white matter	82	female	GSE199258	GPL16791	Illumina HiSeq 2500 (Homo sapiens)
GSM5968284	control	cerebellar white matter	83	female	GSE199258	GPL16791	Illumina HiSeq 2500 (Homo sapiens)
GSM5968285	MSA	cerebellar white matter	64	female	GSE199258	GPL16791	Illumina HiSeq 2500 (Homo sapiens)
GSM5968286	MSA	cerebellar white matter	74	male	GSE199258	GPL16791	Illumina HiSeq 2500 (Homo sapiens)
GSM5968287	control	cerebellar white matter	73	male	GSE199258	GPL16791	Illumina HiSeq 2500 (Homo sapiens)
GSM5968288	MSA	cerebellar white matter	75	male	GSE199258	GPL16791	Illumina HiSeq 2500 (Homo sapiens)
GSM5968289	control	cerebellar white matter	71	male	GSE199258	GPL16791	Illumina HiSeq 2500 (Homo sapiens)
GSM5968290	control	cerebellar white matter	68	female	GSE199258	GPL16791	Illumina HiSeq 2500 (Homo sapiens)
GSM5968291	control	cerebellar white matter	59	female	GSE199258	GPL16791	Illumina HiSeq 2500 (Homo sapiens)
GSM5968292	control	cerebellar white matter	74	male	GSE199258	GPL16791	Illumina HiSeq 2500 (Homo sapiens)
GSM5968293	MSA	cerebellar white matter	84	female	GSE199258	GPL16791	Illumina HiSeq 2500 (Homo sapiens)
GSM5968294	MSA	cerebellar white matter	60	female	GSE199258	GPL16791	Illumina HiSeq 2500 (Homo sapiens)
GSM5968295	MSA	cerebellar white matter	62	female	GSE199258	GPL16791	Illumina HiSeq 2500 (Homo sapiens)
GSM5968296	MSA	cerebellar white matter	61	male	GSE199258	GPL16791	Illumina HiSeq 2500 (Homo sapiens)
GSM5968297	control	cerebellar white matter	79	male	GSE199258	GPL16791	Illumina HiSeq 2500 (Homo sapiens)
GSM5968298	MSA	cerebellar white matter	82	male	GSE199258	GPL16791	Illumina HiSeq 2500 (Homo sapiens)
GSM5968299	MSA	cerebellar white matter	64	male	GSE199258	GPL16791	Illumina HiSeq 2500 (Homo sapiens)
GSM5968300	MSA	cerebellar white matter	61	male	GSE199258	GPL16791	Illumina HiSeq 2500 (Homo sapiens)
GSM5968301	MSA	cerebellar white matter	71	female	GSE199258	GPL16791	Illumina HiSeq 2500 (Homo sapiens)
GSM5968302	MSA	cerebellar white matter	74	male	GSE199258	GPL16791	Illumina HiSeq 2500 (Homo sapiens)
GSM5968303	MSA	cerebellar white matter	67	male	GSE199258	GPL16791	Illumina HiSeq 2500 (Homo sapiens)
GSM5968304	MSA	cerebellar white matter	72	female	GSE199258	GPL16791	Illumina HiSeq 2500 (Homo sapiens)
GSM5968305	MSA	cerebellar white matter	74	female	GSE199258	GPL16791	Illumina HiSeq 2500 (Homo sapiens)
GSM5968306	MSA	cerebellar white matter	69	male	GSE199258	GPL16791	Illumina HiSeq 2500 (Homo sapiens)
GSM5968307	control	cerebellar white matter	84	male	GSE199258	GPL16791	Illumina HiSeq 2500 (Homo sapiens)
GSM5968308	control	cerebellar white matter	81	female	GSE199258	GPL16791	Illumina HiSeq 2500 (Homo sapiens)
GSM5968309	control	cerebellar white matter	73	female	GSE199258	GPL16791	Illumina HiSeq 2500 (Homo sapiens)
GSM5968310	control	cerebellar white matter	70	female	GSE199258	GPL16791	Illumina HiSeq 2500 (Homo sapiens)
GSM5968311	MSA	cerebellar white matter	70	male	GSE199258	GPL16791	Illumina HiSeq 2500 (Homo sapiens)
GSM5968312	control	cerebellar white matter	71	female	GSE199258	GPL16791	Illumina HiSeq 2500 (Homo sapiens)
GSM5968313	control	cerebellar white matter	69	male	GSE199258	GPL16791	Illumina HiSeq 2500 (Homo sapiens)
GSM5968314	control	cerebellar white matter	72	female	GSE199258	GPL16791	Illumina HiSeq 2500 (Homo sapiens)
GSM5968315	control	cerebellar white matter	61	male	GSE199258	GPL16791	Illumina HiSeq 2500 (Homo sapiens)
GSM5968316	control	cerebellar white matter	61	male	GSE199258	GPL16791	Illumina HiSeq 2500 (Homo sapiens)
GSM5968317	control	cerebellar white matter	67	male	GSE199258	GPL16791	Illumina HiSeq 2500 (Homo sapiens)
GSM5968318	control	cerebellar white matter	64	male	GSE199258	GPL16791	Illumina HiSeq 2500 (Homo sapiens)
GSM5968319	control	cerebellar white matter	67	female	GSE199258	GPL16791	Illumina HiSeq 2500 (Homo sapiens)
