# Supplementary material for: MAPKAPK2, a potential dynamic network biomarker of α-synuclein prior to its aggregation in PD patients
Source: NPJ Parkinsons Dis. 2023 Mar 16;9:41. doi: 10.1038/s41531-023-00479-z (PMC10020541; doi:10.1038/s41531-023-00479-z)
Supplement: Supplementary file 2 — Reporting Summary [file 41531_2023_479_MOESM2_ESM.pdf]

nature portfolio

Corresponding author(s): Fei Ling, Yuhu Zhang, Rui Liu, Wei Luo

Last updated by author(s): Feb 19, 2023

Reporting Summary

Nature Portfolio wishes to improve the reproducibility of the work that we publish. This form provides structure for consistency and transparency in reporting. For further information on Nature Portfolio policies, see our Editorial Policies and the Editorial Policy Checklist.

Statistics

For all statistical analyses, confirm that the following items are present in the figure legend, table legend, main text, or Methods section.

n/a	Confirmed	
☐	☒	The exact sample size (*n*) for each experimental group/condition, given as a discrete number and unit of measurement
☐	☒	A statement on whether measurements were taken from distinct samples or whether the same sample was measured repeatedly
☐	☒	The statistical test(s) used AND whether they are one- or two-sided<br>*Only common tests should be described solely by name; describe more complex techniques in the Methods section.*
☐	☒	A description of all covariates tested
☐	☒	A description of any assumptions or corrections, such as tests of normality and adjustment for multiple comparisons
☐	☒	A full description of the statistical parameters including central tendency (e.g. means) or other basic estimates (e.g. regression coefficient) AND variation (e.g. standard deviation) or associated estimates of uncertainty (e.g. confidence intervals)
☐	☒	For null hypothesis testing, the test statistic (e.g. *F*, *t*, *r*) with confidence intervals, effect sizes, degrees of freedom and *P* value noted<br>*Give P values as exact values whenever suitable.*
☒	☐	For Bayesian analysis, information on the choice of priors and Markov chain Monte Carlo settings
☒	☐	For hierarchical and complex designs, identification of the appropriate level for tests and full reporting of outcomes
☐	☒	Estimates of effect sizes (e.g. Cohen's *d*, Pearson's *r*), indicating how they were calculated

Our web collection on statistics for biologists contains articles on many of the points above.

Software and code

Policy information about availability of computer code

Data collection	Data is publicly available. No specific software is required.
Data analysis	All analysis were conducted using the open source statistical software R (version: 4.0.4) and GraphPad Prism (version 8.3.0).

For manuscripts utilizing custom algorithms or software that are central to the research but not yet described in published literature, software must be made available to editors and reviewers. We strongly encourage code deposition in a community repository (e.g. GitHub). See the Nature Portfolio guidelines for submitting code & software for further information.

Data

Policy information about availability of data

All manuscripts must include a data availability statement. This statement should provide the following information, where applicable:

- Accession codes, unique identifiers, or web links for publicly available datasets
- A description of any restrictions on data availability
- For clinical datasets or third party data, please ensure that the statement adheres to our policy

The RNA-seq data were deposited in the NCBI Sequence Read Archive (SRA) database (https://www.ncbi.nlm.nih.gov/sra/) under the BioProject accession PRJNA859664. Publicly available datasets were analysed in this study. These data can be found here: https://www.ncbi.nlm.nih.gov/geo/query/acc.cgi?acc=GSE20292; https://www.ncbi.nlm.nih.gov/geo/query/acc.cgi?acc=GSE68719; https://www.ncbi.nlm.nih.gov/geo/query/acc.cgi?acc=GSE6613; https://www.ncbi.nlm.nih.gov/geo/query/acc.cgi?acc=GSE72267; https://www.ncbi.nlm.nih.gov/geo/query/acc.cgi?acc=GSE99039; https://www.ncbi.nlm.nih.gov/geo/

query/acc.cgi?acc=GSE100054; https://www.ncbi.nlm.nih.gov/geo/query/acc.cgi?acc=GSE150696; and https://www.ncbi.nlm.nih.gov/geo/query/acccgi?acc=GSE199258.

Human research participants

Policy information about studies involving human research participants and Sex and Gender in Research.

Reporting on sex and gender	No sex- and gender-based analyses have been performed. Because this study does not consider the influence of sex or gender on the research content.
Population characteristics	The clinical data for analysis was obtained from the GEO database, and participant type is outlined in the supplements.
Recruitment	The clinical data for analysis was obtained from the GEO database, and recruitment for these cohorts are outlined in the relevant GSE datasets.
Ethics oversight	This was a shared agreement between the NIH and GEO, with the proper protocols in place.

Note that full information on the approval of the study protocol must also be provided in the manuscript.

Field-specific reporting

Please select the one below that is the best fit for your research. If you are not sure, read the appropriate sections before making your selection.

☒ Life sciences ☐ Behavioural & social sciences ☐ Ecological, evolutionary & environmental sciences

For a reference copy of the document with all sections, see nature.com/documents/nr-reporting-summary-flat.pdf

Life sciences study design

All studies must disclose on these points even when the disclosure is negative.

Sample size	In this study, four time points were set, and four parallels were set at each time point.Individual experiments were performed with replicate measures of at least n=4.
Data exclusions	No data was excluded from the study.
Replication	Experiments were performed in quadruplicate to demonstrate reproducibility.
Randomization	No randomization was performed in this study.
Blinding	In the process of data collection and distribution, researchers knew nothing about each group, and only disclosed it when analysis was needed.

Reporting for specific materials, systems and methods

We require information from authors about some types of materials, experimental systems and methods used in many studies. Here, indicate whether each material, system or method listed is relevant to your study. If you are not sure if a list item applies to your research, read the appropriate section before selecting a response.

Materials & experimental systems

n/a	Involved in the study	
☐	☒	Antibodies
☐	☒	Eukaryotic cell lines
☒	☐	Palaeontology and archaeology
☒	☐	Animals and other organisms
☒	☐	Clinical data
☒	☐	Dual use research of concern

Methods

n/a	Involved in the study	
☒	☐	ChIP-seq
☒	☐	Flow cytometry
☒	☐	MRI-based neuroimaging

Antibodies

Antibodies used	5G4 antibody, Cat#MABN389, purchased from Merck, is used for immunostaining analysis. Anti-p-α-syn antibody[EP1536Y], Cat# ab51253, purchased from abcam, is used for immunostaining analysis.

Validation	*Describe the validation of each primary antibody for the species and application, noting any validation statements on the manufacturer's website, relevant citations, antibody profiles in online databases, or data provided in the manuscript.*

Eukaryotic cell lines

Policy information about cell lines and Sex and Gender in Research

Cell line source(s)	Human neuroblastoma cells (SH-SY5Y cells) were purchased from Procell Life Science & Technology Co., Ltd.
Authentication	Cell lines were not authenticated after initial receipt from Procell Life Science & Technology Co., Ltd.
Mycoplasma contamination	Cell lines were not tested for mycoplasma contamination.
Commonly misidentified lines (See ICLAC register)	*Name any commonly misidentified cell lines used in the study and provide a rationale for their use.*
